# Genome-Wide Analysis of Binding Sites and Direct Target Genes of the Orphan Nuclear Receptor NR2F1/COUP-TFI

**DOI:** 10.1371/journal.pone.0008910

**Published:** 2010-01-27

**Authors:** Celina Montemayor, Oscar A. Montemayor, Alex Ridgeway, Feng Lin, David A. Wheeler, Scott D. Pletcher, Fred A. Pereira

**Affiliations:** 1 Department of Molecular and Cellular Biology, Baylor College of Medicine, Houston, Texas, United States of America; 2 Hufftington Center on Aging, Baylor College of Medicine, Houston, Texas, United States of America; 3 Bobby R. Alford Department of Otolaringology – Head and Neck Surgery, Baylor College of Medicine, Houston, Texas, United States of America; 4 Program in Cell and Molecular Biology, Baylor College of Medicine, Houston, Texas, United States of America; 5 Department of Human and Molecular Genetics, Baylor College of Medicine, Houston, Texas, United States of America; 6 Human Genome Center, Baylor College of Medicine, Houston, Texas, United States of America; 7 Department of Bioengineering, Rice University, Houston, Texas, United States of America; Institute of Genetics and Molecular and Cellular Biology, France

## Abstract

**Background:**

Identification of *bona fide* direct nuclear receptor gene targets has been challenging but essential for understanding regulation of organismal physiological processes.

**Results:**

We describe a methodology to identify transcription factor binding sites and target genes *in vivo* by intersecting microarray data, computational binding site queries, and evolutionary conservation. We provide detailed experimental validation of each step and, as a proof of principle, utilize the methodology to identify novel direct targets of the orphan nuclear receptor NR2F1 (COUP-TFI). The first step involved validation of microarray gene expression profiles obtained from wild-type and COUP-TFI^−/−^ inner ear tissues. Secondly, we developed a bioinformatic tool to search for COUP-TFI DNA binding sites in genomes, using a classification-type Hidden Markov Model trained with 49 published COUP-TF response elements. We next obtained a ranked list of candidate *in vivo* direct COUP-TFI targets by integrating the microarray and bioinformatics analyses according to the degree of binding site evolutionary conservation and microarray statistical significance. Lastly, as proof-of-concept, 5 specific genes were validated for direct regulation. For example, the fatty acid binding protein 7 (Fabp7) gene is a direct COUP-TFI target *in vivo* because: i) we identified 2 conserved COUP-TFI binding sites in the Fabp7 promoter; ii) Fapb7 transcript and protein levels are significantly reduced in COUP-TFI^−/−^ tissues and in MEFs; iii) chromatin immunoprecipitation demonstrates that COUP-TFI is recruited to the Fabp7 promoter *in vitro* and *in vivo* and iv) it is associated with active chromatin having increased H3K9 acetylation and enrichment for CBP and SRC-1 binding in the newborn brain.

**Conclusion:**

We have developed and validated a methodology to identify *in vivo* direct nuclear receptor target genes. This bioinformatics tool can be modified to scan for response elements of transcription factors, cis-regulatory modules, or any flexible DNA pattern.

## Introduction

The nuclear receptor family encompasses a set of ligand-regulated transcription factors that bridge a variety of systemic endocrine signals with a tissue-specific gene regulation response [1]. Although it is known that these hormone ligands play crucial roles in numerous homeostatic and pathologic processes–such as metabolism, development, cell division and cancer, and reproduction–the list of specific genes targeted by each nuclear receptor is far from exhaustive. Thus, a more complete catalogue of all nuclear receptor DNA binding sites and gene targets is an attractive goal: to have a deeper mechanistic understanding of a hormone's actions in health and disease, and also allow more precise pharmacologic manipulations to modulate its therapeutic activities and/or unwanted secondary effects.

Numerous efforts have recently been directed towards establishing comprehensive nuclear receptor gene regulatory networks. High-throughput methods that aim at defining the precise genomic sites where a nuclear receptor is physically associated (a process termed “location analysis”) are derived from the chromatin immunoprecipitation (ChIP) technique [2]. Variants of this approach have been used to search for the genomic binding sites of 7 nuclear receptors, each technique having different degrees of bias in terms of the genomic regions probed, resolution, amplification of DNA fragments, analysis algorithms, and other factors [2]. These studies have yielded invaluable information regarding tens to thousands of DNA sites bound and regulated by each specific transcription factor. However, the sensitivity of these assays is limited, they depend on the quality of the antibody, require a specific minimum number of cells to be technically feasible, and tend to be costly due to the need of extensive sequencing or hybridization arrays. Furthermore, the list of binding sites generated is considered to be incomplete, since the procedure is highly susceptible to experimental manipulations, may not detect transient interactions, and is limited to the specific tissue and developmental time assayed. This process can be more difficult when studying orphan receptors, since a ligand is not available to probe for hormone-dependent gene regulation.

COUP-TFs (Chicken Ovalbumin Upstream Promoter Transcription Factors) are members of the orphan subfamily of nuclear receptors [3], [4]. The two homologues in mice, COUP-TFI/NR2F1 (Entrez Gene ID 13865) and COUP-TFII/NR2F2 (Entrez Gene ID 11819), have overlapping expression patterns but independent, essential functions [3], [5], [6]. COUP-TFI is required for central and peripheral neurogenesis and cortical patterning [7], [8]. The COUP-TFI^−/−^ mouse has a high incidence of perinatal mortality, malformations in the glossopharyngeal ganglion, defects in axonal arborization, and loss of cortical layer IV due to the absence of thalamocortical connections [7], [8]. On the other hand, a transgenic mouse that overexpresses COUP-TFI in the developing telencephalon correlates with ventral cortical cell fating and increases the rate of cell-cycle exit and differentiation of the cortical ventricular zone and subventricular zone progenitors, thereby depleting the progenitor pool prematurely and unbalancing the normal proportion of early and late-born neurons [9]. Conversely, knockdown of both COUP-TFI and COUP-TFII in primary neurospheres *in vitro* and at e10.5 *in vivo* results in prolonged generation of early-born neurons at the expense of gliogenesis, suggesting that the precise temporal expression of both COUP-TF homologues is required by neural precursor stem cells (NPSC) to acquire gliogenic competency [10]. Furthermore, COUP-TFI^−/−^ mice display an intriguing inner ear phenotype consisting of a shorter cochlear duct, supernumerary outer hair cells and occasional inner hair cell duplications, decreased innervation, and postnatal degeneration of the basal turn of the organ of Corti [6], [11]. These malformations are incompatible with hearing. Indeed, a deaf child who has many of the abnormalities identified in the COUP-TFI^−/−^ mice was recently discovered to have a chromosomal microdeletion of the entire COUP-TFI locus [12].

We have pursued the identification of COUP-TFI *in vivo* direct targets to reveal genes and pathways that are important for inner ear development and functional maturation. Within the nuclear receptor superfamily, COUP-TFs have the highest degree of evolutionary conservation [3], [4]. Although these are highly studied receptors, the search for their targets has previously been done only on a gene-by-gene basis [5]. There are over 75 known *in vitro* COUP-TF targets, most of them related to lipid/steroid metabolism. However, validated *in vivo* data is limited, and information on inner ear targets is nonexistent. *In vitro* analyses have characterized COUP-TFs to mainly be transcriptional repressors but they can also activate some target genes [3], [5]. COUP-TFs bind with the highest affinity to DNA response elements of the AGGTCAnAGGTCA configuration, or DR1s (direct repeats with 1 spacer) [13]. However, these orphan receptors are notorious for the diversity of their response elements, as they can also bind promiscuously to sites ranging from the DR0 to the DR13 configuration, as well as to everted and inverted repeats, making the bioinformatic search for DNA binding sites particularly challenging. In order to contend with these issues and to identify candidate COUP-TFI inner ear and brain target genes, we have employed a methodology that intersects microarray data, *in silico* genomic analysis with a Hidden Markov Model, and evolutionary conservation filtering.

## Results

### Identification of Candidate COUP-TFI Gene Targets by Microarray Analysis

In order to establish a list of candidate COUP-TFI gene targets, we analyzed the differential gene expression profiles of the wild-type and the COUP-TFI^−/−^ P0 inner ears. For this purpose, we performed a total of 8 microarray experiments using Affymetrix MG-U74Av2 chips, including 2 biological and 2 experimental replicates per genotype sample. Due to limiting RNA yields from the newborn inner ear, each microarray chip was hybridized with an RNA pool from multiple tissue samples, a method that has the additional advantage of eliminating some of the normal biological variability across individuals [14].

Given the lack of consensus within the bioinformatics community regarding the different microarray normalization methods [15], we analyzed our data using two different algorithms: GC-Robust Multi-Array (GCRMA) [16] and dChip [17]. Each normalized expression dataset was subsequently analyzed by 2-way ANOVA, evaluating both genotype and experimental effects. This statistical approach allowed us to 1) account for an experimental effect observed in the expression value of many genes, therefore increasing the power of the analysis, and 2) filter out potential expression differences due to contamination during dissections (contaminating genes would present as probes with a significant interaction p-value). Using this methodology, the gene hits from the GCRMA-normalized expression dataset consisted of 256 genes with a significant genotype effect (p<0.01) and no interaction (p>0.01). Similar cutoffs applied on the dChip-normalized dataset resulted in 250 significant gene hits. Within both groups, *COUP-TFI* has the lowest genotype p-value, validating our statistical approach (**Table S1**).

**Figure 1:**
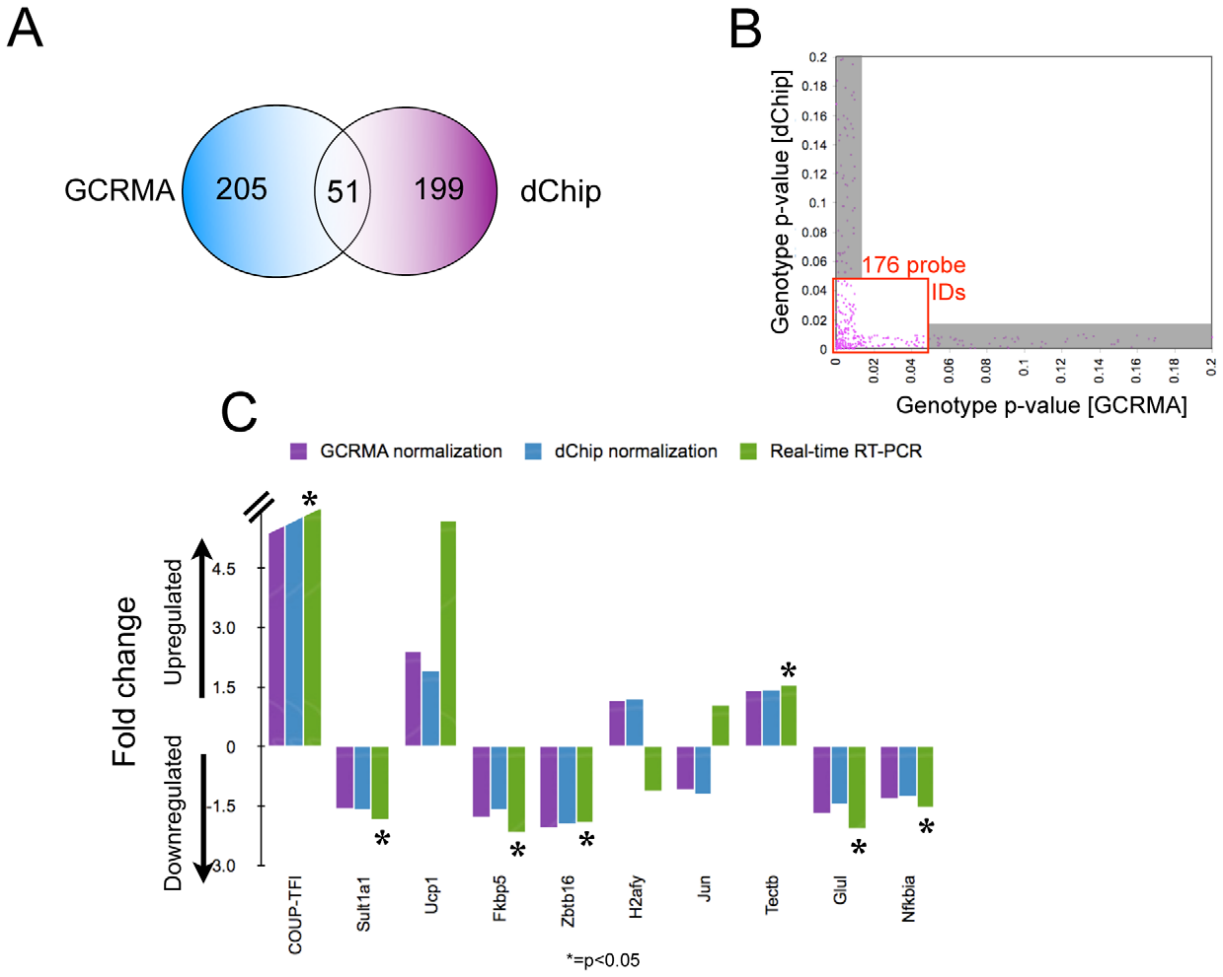
COUP-TFI inner ear microarray analysis and validation. **A.** Normalization of the COUP-TFI microarray data by GCRMA yielded 256 significant gene hits (genotype p<0.01, interaction p>0.01), while normalization with dChip resulted in 250 significant expression changes. Only 51 probes overlap in both data sets. **B.** Correlation of genotype p-values for probe sets that do not interact (p>0.01) and have a genotype p<0.01 in the GCRMA or dChip-normalized data sets. Probes with genotype p<0.01 by one normalization method but genotype p>0.05 by the other method (shaded area) were eliminated from the final hit list. **C.** Real-time RT-PCR validation of the top 10 microarray hits. The graph compares the wild-type/COUP-TFI^−/−^ fold change indicated by each set of normalized microarray data and by real-time RT-PCR performed with individual pairs of inner ears (n = 4, * = p<0.05).

Surprisingly, only 51 genes were present in both hit lists (**Figure 1A**). In fact, the correlation between the outputs of the two normalization methods was quite limited, as evidenced by plotting the two corresponding genotype p-values for each probe (r^2^ = 0.07) - although we did observe a stronger correlation among the lower p-values (data not shown). The same trend was observed after plotting the interaction p-values, the genotype rank number for each probe, and the genotype p-values of those probes that did not have a significant interaction (p>0.01) in both methods (data not shown). In view of these facts and in order to obtain a hit list with the maximum confidence, we narrowed down our final gene list to 176 probe IDs (171 unique genes) that have the best genotype p-value correlation in the two normalization methods (no interaction, a genotype p<0.01 by one normalization method, and a genotype p<0.05 in the other) (**Figure 1B**) (See **Table S1** and **Table S2** for complete expression and statistical data as well as BED format files). Within the 176 candidate COUP-TFI microarray targets, five gene ontology categories are significantly enriched: cell adhesion, cartilage development, regulation of progression through cell cycle, myeloid cell differentiation and cellular lipid metabolism. Most of the COUP-TF targets known today are associated to lipid/steroid metabolism, and this pathway, specifically the modulation of cholesterol levels, plays an important role in hair cell mechanotransduction and outer hair cell function [18], [19].

We next examined the expression level of the top 10 genes on the list by real-time RT-PCR in individual pairs of wild-type and COUP-TFI^−/−^ P0 mouse inner ears (n = 4). With this approach, we verified 7 out of the 10 gene expression changes with statistical significance (p<0.05) (**Figure 1C**). These results are noteworthy when comparing whole inner ears because COUP-TFI is only expressed in a restricted domain of the inner ear (mostly the sensory epithelium) at this stage [6]. From the genes that were not validated with statistical significance, *jun* is an *in vitro* indirect COUP-TF target [20], and *ucp1* expression is deregulated upon COUP-TF knockdown in a skeletal muscle cell culture model [21], suggesting that our microarray hit list likely contains real COUP-TFI targets, some of which likely cannot be validated by the sensitivity of our assay. Additional real-time validation of the microarray results is displayed in **Table S3**.

Of the 176 probe IDs in the hit list, 59% (104 probes) have a lower expression level in the wild-type samples, a result consistent with the proposed mechanism of COUP-TF action as predominantly a transcriptional repressor [5]. In addition, 7 gene hits have been directly implicated in human and/or mouse hearing loss (**Table 1**), and 7 others are related to gene families associated with inner ear abnormalities (**Table 2**) [22]–[24]. We also determined that 43% (75) of our gene hits are documented inner ear transcripts curated in inner ear expression databases [24]–[26].

**Table 1:** Microarray hits associated with human/mouse hearing loss.

Gene	Description	Wild-type/COUP-TFI−/−	Species	References
Agc1	Aggrecan 1	0.86	Mouse	Rittenhouse et al 1978
Col7a1	Procollagen type 1 alpha 1	0.89	Mouse	Bohne and Harding 1997; Sokolov et al 1995
Crym	Crystallin, mu	1.22	Human	Abe et al 2003
Gjb2	Gap junction membrane channel protein beta 2	1.2	Human/Mouse	Gilford et al 1994; Kelsell et al 1997; Brown et al 1996; Cohen-Salmon et al 2002
Mgp	Matrix gla protein	0.88	Human Keutel Syndrome	Munroe et al 1999
Pdgfra	Platelet derived growth factor receptor, alpha polypeptide	0.89	Mouse	Deol et al 1970; Duttlinger et al 1995; Stephenson et al 1991
Sod1	Superoxide dismutase 1, soluble	0.74	Mouse	Seidman et al 1991; Seidman et al 1993; McFadden et al 1999; Ohlemiller et al 1999

**Table 2:** Microarray hits related to gene families implicated in human/mouse hearing loss.

Gene	Description	Wild-type/COUP-TFI−/−	Human family	Mouse family
Aldh1a1	Aldehyde dehydrogenase family 1, subfamily A1	0.75		Raldh2
Cldn1	Claudin 1	0.83	Claudin14	Claudin11
Col6a1	Procollagen type 6 alpha 1	0.92	Col2a1, Col4a3, Col4a4, Col4a5, Col11a1, Col11a2, Col9a1	Col11a2, Col4a3, Col4a4, Col4a5, Col2a1
Kcna5	Potassium voltage-gated channel, shaker-related subfamily, member 5	0.79	Kcne1, Kcnq1, Kcnq4	Kcne1
Slc25a1	Solute carrier family 25 (mitochondrial carrier, citrate transporter), member 1	1.15		Slc12a2, Slc12a6, Slc12a7, Slc17a5, Slc19a2, Slc1a3, Slc26a4, Slc26a5, Slc4a2, Slc4a7, Slc30a4, Slc9a1
Tectb	Tectorin beta	1.4	Tecta	Tecta
Timm23	Translocase of inner mitochondrial membrane 23 homolog (yeast)	1.17	Timm8a	Timm8a

### *In Silico* Genomic Prediction of COUP-TF Binding Sites Using a Hidden Markov Model

In an effort to validate further the microarray gene hits and differentiate between direct and indirect COUP-TFI targets, we set out to identify if COUP-TF response elements were present within the nearby regulatory sequences of the microarray gene hits. This was problematic because the available public transcription factor search sites were inadequate for this purpose, since their position frequency matrices were derived from less than 20 known binding sites and they only described one type of response element, namely, the DR1 configuration. Since COUP-TFs can bind a diversity of DNA response elements [13], we decided to construct a Hidden Markov Model (HMM) that would allow flexibility in the direction of the half-sites and in the number (if any) of nucleotide spacer sites [27]. This strategy would allow us to take advantage of the firm probabilistic HMM model and to extend our search beyond individual promoters into the genomic scale.

**Figure 2:**
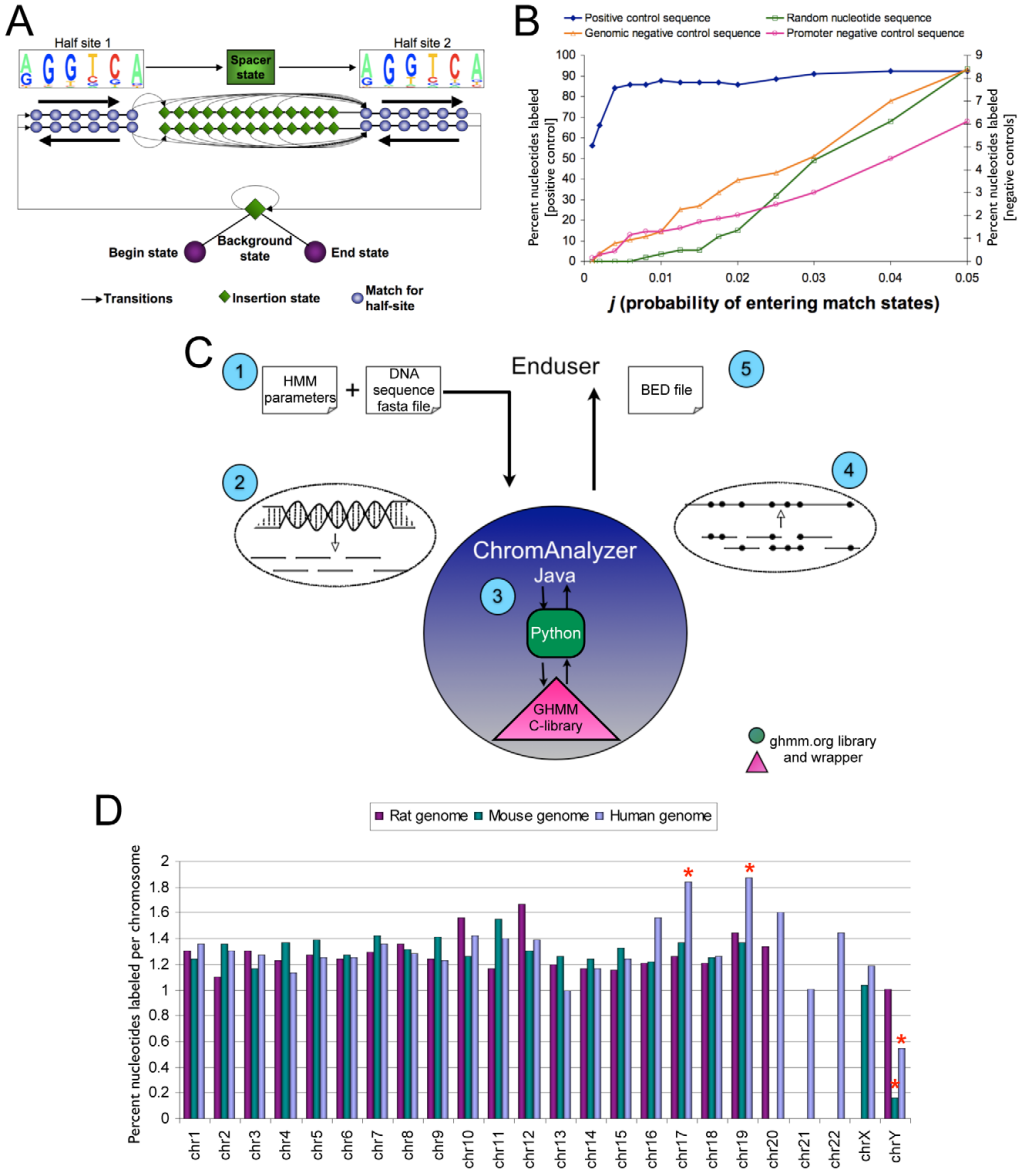
COUP-TF Hidden Markov Model (HMM) framework, validation and genomic scans. **A.** Graphical representation of the COUP-TF HMM framework. Each arrow represents a transition state, diamonds and circles denote emission states. The system starts from a background state (ie, a non-binding site nucleotide) and searches for matches to direct repeats ranging from the DR0 to the DR13 configuration. **B.** Sensitivity and selectivity of the COUP-TF HMM. To evaluate sensitivity of the COUP-TF HMM, a positive control sequence containing all 49 training sites was analyzed iteratively with a Viterbi algorithm and increasing values of *j*. Results are plotted as the percentage of training-site nucleotides that were correctly labeled (left y-axis). To evaluate selectivity (right y-axis), three negative control sequences of the same size were evaluated in a similar manner. **C.** The ChromAnalyzer Java library performs custom HMM searches at a genomic level. (1) The end user inputs 2 text files: one with the corresponding HMM parameters, and another file with a chromosome fasta file. (2) ChromAnalyzer translates the chromosome to numerical format and fragments the sequence into smaller, overlapping fragments. (3) G-HMM software [29] searches for HMM matches in each fragment using the Viterbi algorithm. (4) The location of each HMM match is translated into chromosomal coordinates and (5) delivered to the enduser as a standard BED file. **D.** Distribution of COUP-TF HMM matches in the entire mouse, human and rat genomes. * = percentage of labeled nucleotides differs from the species' mean by more than 2 standard deviations.

To obtain the specific COUP-TF HMM parameters, we first created a detailed database of all COUP-TF targets reported in the literature, which we annotated and verified as described in the Materials and Methods section. This *direct* COUP-TF target gene database consists of 55 genes and 61 binding sites from 7 different species. The specific binding regions were classified as half-sites, direct (DR), inverted (IR) and everted (ER) repeats of various spacings. Half-sites were discarded from the list, since they can be described with simple position weight matrices, and biochemical data suggests that COUP-TFs bind as dimers [13]. Everted and inverted repeats were also discarded since they were too few to create statistically reliable position frequency matrixes for each half-site, and the literature suggests that the nucleotide distribution is not equivalent in each orientation [27]. The final list of HMM training sequences consists of 49 direct repeats that range from the DR0 to the DR13 spacer configuration (**File S1**). HMM emission and transition probabilities were then determined as described by Sandelin et al [27], using Laplace's rule as a pseudocount strategy [28]. A classification-type HMM with circular local alignment and a reverse complement version was generated with this data (**Figure 2A** and **File S1**).

We used the GHMM C++ library [29] (python bindings) to examine target sequences with our COUP-TF HMM parameters and the Viterbi algorithm [28]. To evaluate the sensitivity of our model and optimize the *j* probability (probability of entering the match state), we used the Viterbi algorithm to analyze a 9000bp sequence containing all 49 training sites and ∼100bp of their surrounding native genomic environment. We were able to detect a high proportion of the training set (87% correctly labeled training site nucleotides) at a stringent *j* value of 0.01, and up to 92% correctly-labeled nucleotides with *j*> = 0.04 (**Figure 2B**). These results are consistent with previous reports, which indicate a sensitivity plateau when *j*>0.04 [27], but our HMM has an improved level of maximum sensitivity (92% vs 85%). On the other hand, a negative control random nucleotide sequence of the same size yielded no false positives up to a *j* value of 0.006, with only 8.4% false detection at a *j* of 0.05 **(** **Figure 2B** **)**. We also tested two other negative controls: 1) a sequence of the same length that reflects nucleotide distribution at the genomic level and 2) a sequence of the same size that reflects nucleotide distribution within 5000bp of established transcription start sites (UCSC, mm8 assembly, 46% GC content). Both experiments yielded low false positive detection rates even at high values of *j* (8.3% and 6.1%, respectively, **Figure 2B**). Based on these results, we selected a *j* value of 0.006 to run complete genome scans, a probability that allows for a high sensitivity with very low false-positive costs.

Short nucleotide sequences can be processed directly using the GHMM python bindings [29]. However, since there is no publicly available software to process entire chromosomes, we created the ChromAnalyzer Java library. This tool was designed with the flexibility required to identify binding sites of any transcription factor (**Figure 2C**, described in detail in the Materials and Methods section). Using this software and the HMM parameters described above, we searched the entire mouse, human, and rat genomes for candidate COUP-TF DNA binding sites. We found a total of 2,436,761 unique COUP-TF HMM matches in the mouse genome; as expected, the distribution of spacer states was similar to that of the training set (data not shown). On average, we found 1 site every 1085bp, and the number of sites was roughly proportional to the size of each chromosome. In fact, the percentage of nucleotides labeled as a COUP-TF HMM match was fairly constant throughout the genome **(** **Figure 2D** **)**. The human genome yielded a similar density of COUP-TF HMM matches (1 site every 1086bp), with a constant percentage of labeled nucleotides throughout the genome, except for human chromosomes 17 and 19, which had a significantly higher number of sites when correlated to the chromosome size. The human and mouse chromosome Y, on the other hand, resulted in significantly fewer HMM matches than expected from the number of sequenced base pairs in these chromosomes. The rat genome had a slightly lower COUP-TF match frequency, resulting in 1 site every 1124 genomic base pairs. The complete list of candidate COUP-TF binding sites (ie, COUP-TF HMM matches) in the mouse genome is available in BED format directly from the authors. This is essentially a database of the DR0-DR13 sites in the entire mouse genome that can be uploaded into the UCSC Genome Browser [30]. Similar files corresponding to the COUP-TF HMM matches in the rat and human genomes are also available.

The number of genomic COUP-TF HMM matches is high when compared to the number of nuclear receptor binding sites found by ChIP-based location analyses [2]. However, this number of HMM matches corresponds to the sum of 9 different spacer configurations. As further validation, we observed that most of these sites are present within non-coding genomic sequences; only 2.7% of all HMM matches had any overlap with coding regions (defined here as both UTRs and coding exons). On average, we found 1 site every 933 *non*-coding bps, and only 1 site per 13,073 coding bps. This represents a 36.8-fold enrichment of candidate COUP-TF binding sites within the non-coding genomic regions. After correcting for the size of both coding and non-coding genomic compartments, we still observed an 11.63-fold enrichment in the non-coding section (roughly 1 in every 79.3 non-coding bps was labeled as a COUP-TF site, compared to 1 in every 1110 non-coding bps). At any rate, the high density of COUP-TF HMM matches resulted in at least one site within the promoters of all but 2 of our microarray hits (intersected via the UCSC Table Browser [30]). Thus, an additional filtering step was required to identify functional regulatory sites.

### Correlation of Microarray and Bioinformatics Data with Evolutionary Conservation

At present, the most popular way of increasing the specificity of computational transcription factor binding site searches is a technique known as phylogenetic footprinting [31]. This concept is based on the assumption that evolution selects against mutations within DNA regions that have an important regulatory function; thus creating evolutionary “cold spots” within the critical genomic segments that regulate gene expression. To perform this analysis, we used UCSC's [30] “Most Conserved” track and phastCons conservation score to filter our candidate COUP-TF binding site list. For each microarray hit, we extracted and classified COUP-TF HMM matches within any of 3 proximal regulatory regions: 1) promoter; defined here as 5000bp upstream of each transcription start site associated with the gene, 2) 5′ UTR, and 3) first intron of all transcripts associated with the gene. The classification included one of 3 mutually exclusive degrees of conservation, in order of decreasing importance: 1) overlap with a UCSC Most Conserved segment, 2) conservation score >0.7, and 3) conservation score >0.3. We found a total of 258 individual HMM matches that fulfilled one of the conservation conditions, corresponding to 93 unique genes within our microarray hit list. An interesting finding was that the highest number of COUP-TF conserved sites was found within the first introns of those transcripts (149 intronic DNA elements in total, compared to 65 in promoters and 24 within the 5′UTRs). This information, along with our COUP-TF microarray results, was used to create a ranked list of candidate COUP-TFI targets in the inner ear. Ranking was weighed with the following criteria, in decreasing order: 1) lower microarray genotype p-value or higher fold change 2) presence and number of “Most Conserved” COUP-TF HMM matches (in any location) 3) presence and number of COUP-TF HMM matches with a conservation score above 0.7 and 4) presence and number of COUP-TF HMM matches with a conservation score above 0.3. The top 25 hits are displayed in **Table 3**, and the full database is available as **Table S3**. Our analysis is validated by the fact that *Acox1*, a known COUP-TFI target [32], is among the highest ranked candidate target genes in our database.

**Table 3:** Top 25 candidate inner ear COUP-TFI targets.

Rank	Probe ID	Gene Symbol	Microarray genotype p-value Rank*	Microarray fold-change Rank*	P MC	P >0.7	P >0.3	5U MC	5U >0.7	5U >0.3	FI MC	FI >0.7	FI >0.3
1	161750_F_AT	NCAM1	119	88							10	9	8
2	103212_AT	CDCA7L	34	31							9	5	8
3	101469_AT	NEDD9	98	94	3						5***	1	2
4	92958_AT	FOXO3A	124	159	1	1					6		
5	101515_AT	ACOX1	29	30	9	1							
6	100952_AT	STIM1	103	24				1			4	2	8
7	103575_AT	CHD4	33	34				7**	1			1	
8	161461_AT	BMP7	15	115	1				1		2	2	1
9	94297_AT	FKBP5	173	171	1						1		3
10	95731_AT	SESN1	162	167							2		3
11	104671_AT	AMPD3	142	96	1		1	2					1
12	100354_AT	TBX15	9	133							2	3	1
13	101542_F_AT	DDX3X	7	126	3**	1							
14	102414_I_AT	DNAJC3	23	142							3		1
15	98967_AT	FABP7	148	165	1	1							
16	92202_G_AT	ZBTB16	172	174	1								1
17	104728_AT	PROS1	70	62				1	1		1	1	
18	160139_AT	HSPB8	125	146	1		1		1				
19	93527_AT	KLF9	151	155							1		2
20	96825_AT	TENC1	72	73	3								
21	102426_AT	CASQ1	113	132		1				1	1		
22	93619_AT	PER1	158	154	1		1						
23	103460_AT	DDIT4	164	170	1								
24	103040_AT	CD83	117	145	1	1							
25	98108_AT	CRABP1	100	122	2								

Ranking criteria (leftmost column) integrate microarray data and number of conserved COUP-TF HMM hits, as well as the degree of evoluationary conservation. P = promoter (5000bp); 5U = 5′UTR; FI = first intron of all transcripts associated with the gene.

MC = Most conserved site; >0.7 = site with a conservation score greater than 0.7; >0.3 = site with a conservation score greater than 0.3.

*Microarray ranking range from 1–175, higher rank numerical value with lower genotype p-value or greater fold-change.

**Corresponds to a late transcription start site.

***Sites distributed in the first intron of different transcripts, or in the first intron of a particular splice variant.

### Validation of a Candidate COUP-TFI Target *In Vitro* and *In Vivo*

**Figure 3:**
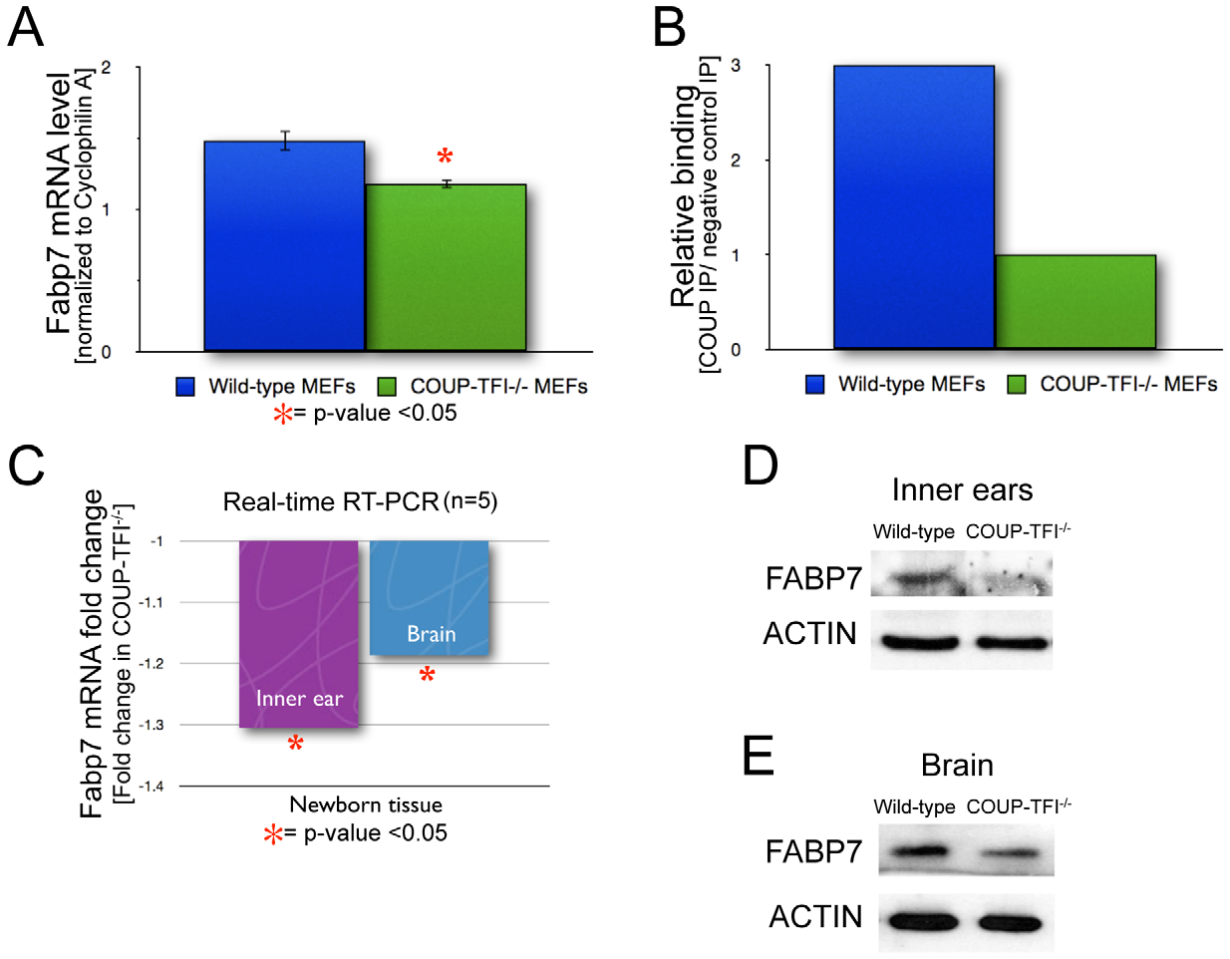
Fabp7 is a COUP-TFI target. **A.** Real-time RT-PCR shows lower *Fabp7* transcript levels in COUP-TFI^−/−^ MEFs (n = 5; * = p<0.01). **B.** Chromatin immunoprecipitation assay demonstrates binding of COUP-TFI to the wild-type *Fabp7* promoter in cell culture. **C.** Real-time RT-PCR for *Fabp7* transcript levels using P0 inner ears and newborn brain cortex (n = 5, * = p<0.05). **D.** Western Blot analysis for FABP7 in P0 inner ear whole cell protein extracts, analysis revealed a 34% reduction in FABP7 protein levels in the COUP-TFI^−/−^ tissue. **E.** Western Blot analysis for FABP7 in P0 brain whole cell protein extracts, analysis revealed a 27% reduction in FABP7 protein levels in the COUP-TFI^−/−^ brains.

The *Fabp7* gene (fatty acid binding protein 7, brain, Entrez Gene ID 12140) was ranked 15^th^ within the candidate target gene list (**Table 3**) and selected for *in vitro* validation experiments using wild-type and COUP-TFI^−/−^ mouse embryonic fibroblasts (MEFs). *Fabp7* is the brain-type member (BLBP) of the intracellular lipid binding protein family – although it is also expressed in other tissues such as the retina and mammary gland [33]. In particular, Fabp7 and COUP-TFI have similar temporal and spatial expression patterns in the ventricular zone of the developing brain [10], [34], [35]. Of note, the majority of known COUP-TF targets belong to the glucose and lipid metabolism pathways **(Table S2)**, and COUP-TFII is described to induce Fabp2 (intestinal fatty acid-binding protein) expression *in vitro* [36]. *Fabp7* is down-regulated in the COUP-TFI^−/−^ inner ear as determined by our microarrays and contains 2 closely-spaced conserved COUP-TF HMM matches (DR0 and DR2) within its upstream promoter. As a preliminary step, we verified significant changes in the expression level of *Fabp7* in the COUP-TFI^−/−^ MEF cell line by real-time RT-PCR (**Figure 3A**). Next, we performed chromatin immunoprecipitation (ChIP) to determine if COUP-TFI binds directly to the predicted response elements. In wild-type MEFs, there is a 3-fold enrichment of COUP-TFI physical association to the binding site region of the *Fabp7* promoter as compared to binding in COUP-TFI^−/−^ MEFs (**Figure 3B**).

To validate *Fabp7* as an *in vivo* target gene, we analyzed the *Fabp7* transcript levels in wild-type and COUP-TFI^−/−^ tissues. Using real-time RT-PCR we measured that there was a lower *Fabp7* mRNA level in COUP-TFI^−/−^ P0 inner ears as compared to wild-type controls (**Figure 3C**). Similar changes in *Fabp7* transcript levels were observed in the COUP-TFI^−/−^ P0 brain cortex (**Figure 3C**). These differences in *Fabp7* expression were also evident at the protein level: FABP7 protein was 34% lower in inner ears and 27% lower in the brain of newborn COUP-TFI^−/−^ mice, when compared to the corresponding wild-type tissue controls (**Figures 3D and E**). The moderate decrease of Fabp7 transcript and protein levels can be explained by a compensatory upregulation of COUP-TFII levels in both COUP-TFI^−/−^ brain and inner ear tissues (**Figure S1**).

**Figure 4:**
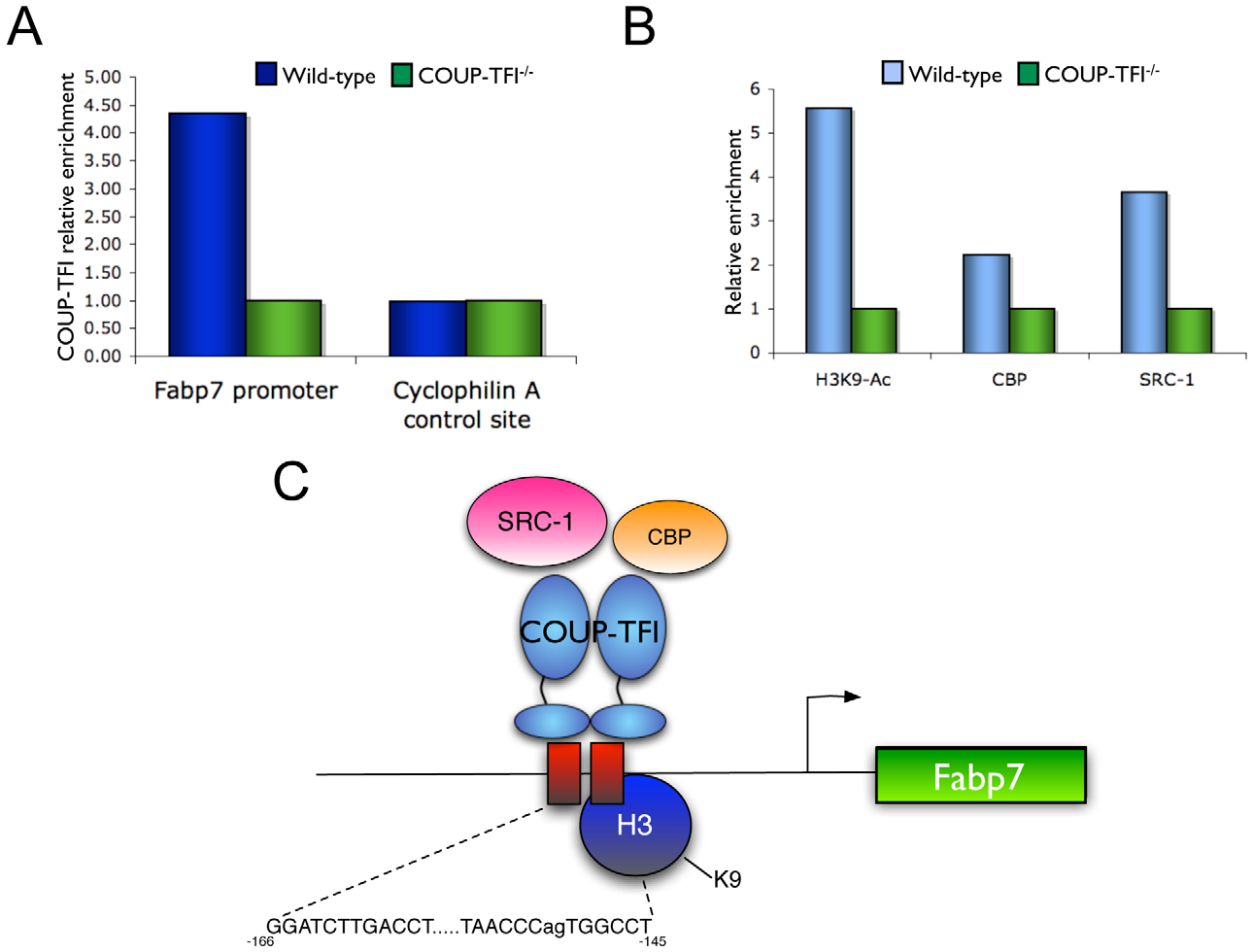
Validation of Fabp7 as a COUP-TFI target *in vivo*. **A.** *In vivo* chromatin immunoprecipitation assay demonstrates physical binding of COUP-TFI to the *Fabp7* promoter in the wild-type newborn brain, COUP-TFI^−/−^ brains were used as a negative control. No COUP-TFI binding enrichment is observed at a negative control genomic region (Cyclophilin A). **B.** *In vivo* chromatin immunoprecipitation reveals higher levels of H3K9 acetylation in the *Fabp7* promoter of the wild-type newborn brain tissue, as well as increased physical association of CBP and SRC-1. **C.** Schematic diagram of the proposed mechanism of *Fabp7* regulation by COUP-TFI.

We next used the newborn brain to determine if COUP-TFI was directly binding to the *Fabp7* promoter *in vivo*. ChIP experiments were used and confirmed that COUP-TFI was enriched and physically associated to the *Fabp7* promoter binding sites in the wild-type compared to COUP-TFI^−/−^ P0 brain (**Figure 4A**). No COUP-TFI binding was observed at the negative control genomic region (the housekeeping gene Cyclophilin A) (**Figure 4A**). To elucidate the state of the chromatin at the *Fabp7* promoter by which COUP-TFI regulates *Fabp7* expression, we compared the levels of H3K9 acetylation, a marker of active chromatin, in the wild-type and COUP-TFI^−/−^ brain tissue. *In vivo* ChIP revealed that H3K9 acetylation is more enriched at the *Fabp7* promoter in the wild-type than in COUP-TFI^−/−^ newborn brains (**Figure 4B**), consistent with a mechanism that COUP-TFI regulation of *Fabp7* is associated with higher chromatin accessibility and transcriptional activity.

To identify potential candidate histone acetyltransferases that might be recruited to the *Fabp7* promoter to enhance COUP-TFI regulation, a series of *in vivo* ChIPs were performed to test if CBP, p300 and SRC-1 cofactors were present. CBP/p300 are general coactivators recruited by most nuclear receptors, and SRC-1 is a known COUP-TFI coactivator at the PEPCK and NGFI-A promoters [37], [38]. *In vivo* ChIP analyses revealed an enrichment of CBP and SRC-1 recruitment to the wild-type brain *Fabp7* promoter compared to the COUP-TFI^−/−^ samples (**Figure 4B**). There was no *in vivo* p300 recruitment observed for either genotype (data not shown). These results lead to a gene regulation model (**Figure 4C** **)**, where COUP-TFI binds the conserved DR0/DR2 sites in the proximal upstream *Fabp7* promoter, recruiting CBP, SRC-1 and possibly other coactivators that promote local H3K9 acetylation, creating an active chromatin environment and increasing the transcription rate of the *Fabp7* gene.

### Validation of Other Predicted COUP-TFI Targets

**Figure 5:**
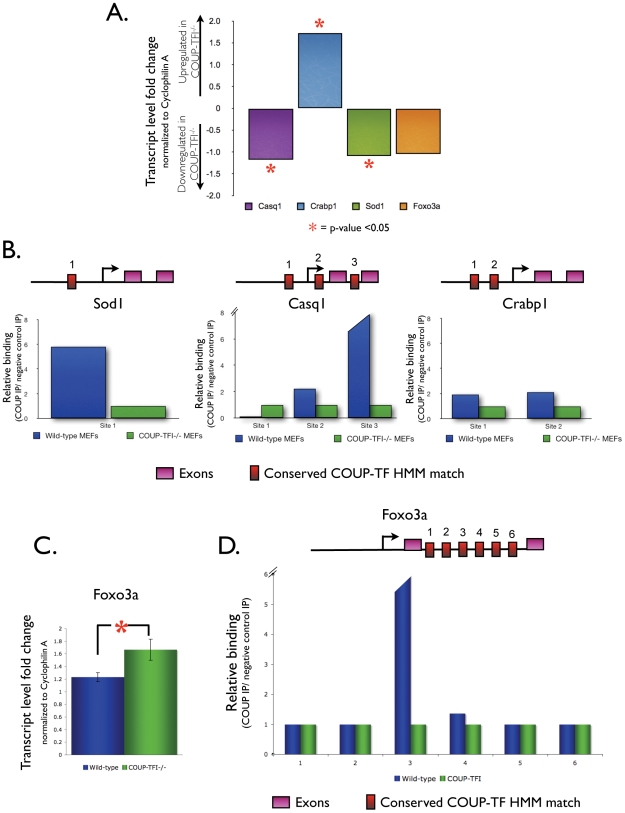
Validation of other COUP-TFI targets. **A.** Real-time RT-PCR shows significantly deregulated *Casq1*, *Crabp1*, and *Sod1* transcript levels in COUP-TFI^−/−^ MEFs (n = 5; * = p<0.05). **B.** Chromatin immunoprecipitation assay in cell culture demonstrates binding of COUP-TFI to at least one conserved COUP-TF HMM match near the *Casq1*, *Crabp1*, and *Sod1* genes. **C.** Real-time RT-PCR for *Foxo3a* transcript levels using newborn brain cortex (n = 5, * = p<0.05). **D.** *In vivo* chromatin immunoprecipitation assay demonstrates physical binding of COUP-TFI to the third conserved COUP-TF HMM match in the *Foxo3a* first intron.

In order to expand the experimental validation of candidate COUP-TFI target genes–and to strengthen the ‘proof-of-concept’ of our methodology–we performed additional *in vitro* validation experiments using wild-type and COUP-TFI^−/−^ MEFs. We selected 4 additional candidate target genes that contain at least one conserved COUP-TF HMM match within their nearby regulatory regions, and first determined if they are also modulated in a COUP-TFI-dependent manner in these cell lines by performing real-time RT-PCR. Three genes (*Crapb1*: cellular retinoic acid binding protein 1, Entrez Gene ID 12903; *Sod1*: superoxide dismutase 1, soluble, Entrez Gene ID 20655; and *Casq1*: calsequestrin1, Entrez Gene ID 12372) were significantly deregulated at the transcript level in COUP-TFI^−/−^ MEFs when compared to wild-type cells, while one gene (*Foxo3a*: forkhead box O3a, Entrez Gene ID 56484) did not show statistically significant changes in this context (**Figure 5A**). We then investigated physical recruitment of COUP-TFI to the predicted response elements for each of the 3 deregulated genes. ChIP assays using primer sets flanking each of the candidate binding sites confirmed COUP-TFI recruitment to at least one response element in each candidate gene in the wild-type cells (**Figure 5B**), but not in the COUP-TFI^−/−^ negative control reactions. Specifically, ChIP assays revealed 2-fold enrichment for each of the *Crabp1* promoter COUP-TF response elements, 2-fold enrichment for the binding site in the *Casq1* 5′UTR as well as over 8-fold enrichment for the response element in the first intron of this gene, and 6-fold enrichment for the conserved COUP-TF HMM match in the *Sod1* promoter (**Figure 5B**).

We did not evaluate the physical association of COUP-TFI to the *Foxo3a* predicted binding sites *in vitro*, since this gene's expression is not deregulated in the absence of COUP-TFI in our cell culture model (**Figure 5A**). However, the microarray data indicated that *Foxo3a* is deregulated in the COUP-TFI^−/−^ newborn inner ear, and therefore is a potential direct COUP-TFI target *in vivo*. Thus, we explored *Foxo3a* expression regulation and recruitment of COUP-TFI, as described for the *Fabp7* gene. Indeed, real-time RT-PCR for the upstream *Foxo3a* transcript revealed a significantly higher *Foxo3a mRNA* levels in the COUP-TFI^−/−^ newborn cortex when compared to wild-type controls (**Figure 5C**). *In vivo* ChIP assays also verified physical association and enrichment of COUP-TFI to one of the six conserved COUP-TF HMM sites located within the first intron of this gene (**Figure 5D**).

## Discussion

We have described a methodology to search for transcription factor binding sites that is particularly suited for factors that have a flexible DNA binding profile: the intersection of microarray data, HMM genome scan, and UCSC conservation tracks. Hidden Markov Models are widely used in bioinformatics for applications that range from exon boundary prediction to conservation analyses [39]. The use of HMMs to search for transcription factor binding sites has been reported by several groups [27], [40]–[42]; however, there is no study that integrates genomic HMM scans with conservation and expression data, or that corroborates predicted novel targets with experimental validation. Moreover, no publicly available software allows the search of entire genomes for matches to a custom HMM. Thus, this study constitutes the first large-scale expression analysis for COUP-TFI/NR2F1 with validation of direct COUP-TFI regulation *in vitro* and *in vivo* of novel target genes predicted by a bioinformatic approach.

We address the uncertainty regarding the performance of different microarray normalization methods [15] by comparing the results of two algorithms -GCRMA and dChIP. Although the correlation between these two methods was limited, we reconciled both sets of results by extracting gene probes with a significant genotype difference and the best p-value correlation and validated the results by quantitative real-time RT-PCR using inner ear tissue.

The number of total COUP-TF HMM hits in the entire mouse genome is several orders of magnitude higher than the number of binding sites reported by ChIP-based location analyses [2]. However, the number of COUP-TF HMM matches found within a random nucleotide sequence is negligible **(** **Figure 2B** **)**, and most of the sites are enriched within the non-coding genomic regions. These facts support the idea that these DNA-binding compatible sequences are not found in error, but rather that this is their true distribution and density within the genome. Other researches have reached similar conclusions [31]. The prevailing dogma posits that most transcription factor binding sites found *in silico* – although they would readily bind the protein *in vitro*- will not have a functional role *in vivo* (a concept defined as the “futility theorem” [31]). Indeed, most ChIP-based assays have only a partial overlap with binding sites found by computational searches, and most sites predicted *in silico* are not occupied by the cognate transcription factor when pursued experimentally. However, three factors account for at least part of this disparity in the number of transcription factor binding sites. First, most ChIP-based datasets are considered to be incomplete, since the information is restricted to a particular tissue or cell line and developmental time point. For example, some nuclear receptor binding sites may be functional only in a tissue-specific or developmental-time specific manner, and subordinate to chromatin modifications that occur at a certain stage of cell fating, during development, aging or disease state. Second, ChIP assays have specific technical limitations, such as the sensitivity of the antibody and the particular experimental conditions, and they may not detect transient protein-DNA interactions. In fact, high-throughput ChIP assays performed for the same nuclear receptor under similar experimental conditions have only partial overlap in their results [2], [43], [44]. Third, it is likely that the functional role of a given DNA-binding site is also determined by the specific content of the surrounding DNA, and that unique combinatorial interactions between several transcription factors determine the expression profile of each cell. This concept is also supported by available nuclear receptor location data [43]. However, we envision that it is feasible to increase the selectivity of bioinformatics searches by modeling higher order cis-regulatory modules (CRMs) [31], consisting of several transcription factor binding sites, using the same HMM statistical principles and software described herein.

Our bioinformatic search for transcription factor binding sites is limited by the fact that it will not detect regulatory sites where recruitment to the DNA is dependent on tethering to other transcriptional cofactor(s), a mechanism that is estimated to account for 30% of nuclear receptor-dependent gene regulation [2]. Physical association assays based on the chromatin immunoprecipitation technique are required to detect this type of downstream targets. Another constraint of our approach is that we limited our scan for COUP-TF HMM matches within the proximal regulatory regions, and therefore our analysis excludes regulatory sites located far from the gene's transcription start site, and it is susceptible to any mis-annotations in the corresponding genome assembly. Location assays have indeed identified that a significant proportion of nuclear receptor binding sites are distant from the regulated genes [43]. Thus, chromatin immunoprecipitations remain the gold standard to detect DNA-protein interactions. We present the ChromAnalyzer software tool not as a substitute for ChIP-based assays, but as a reasonable alternative to perform quick and inexpensive genome-wide nuclear receptor location analyses, particularly for those laboratories with a limited budget or who are technically limited by the size of their cell population, such as stem cell studies or our own work on the developing and newborn cochlear sensory epithelia, which is only composed of 16,000 mechanosensory hair cells. Following ChromAnalyzer bioinformatic examination, the tissue and time-specific binding sites can be subsequently filtered with the aid of expression and physical association assays, as we have demonstrated. The definition of proximal regulatory elements can be revised and the microarray correlation analysis repeated iteratively with new parameters as necessary, although ChIP-based approaches are still necessary to identify very distant or non-canonical binding sites.

As the third layer of correlation, we used phylogenetic footprinting to filter the HMM hits, extracting the location of binding sites near microarray gene hits that are also conserved across evolution. Although phylogenetic footprinting aids in prioritizing target lists and increases specificity by ∼90% [31], it is important to bear in mind that it will miss conserved sites in regions that are not easily aligned. Therefore, absence of a conserved site – or absence of a site altogether – does not fully discard the possibility of regulation by COUP-TFI. Furthermore, the site might be missed by a particularly low *j* value or consist of an atypical nucleotide distribution. At present, our genomic HMM scans are not intended to be used in isolation, but rather as one layer of evidence to guide the design of experiments, accelerating the otherwise arduous process of gene-wise transcription factor binding site validation. Furthermore, ChromAnalyzer HMM scans are meant to be an iterative process: each newfound, validated COUP-TFI DNA binding site will be incorporated into the HMM training list, and thus the COUP-TF HMM parameters will be fine-tuned to reflect the exact profile of the known COUP-TF binding sites.

We first describe the detailed and mechanistic experimental validation *in vitro* and *in vivo* of one of the highest-ranked candidate COUP-TFI targets identified by our analysis: the fatty-acid binding protein 7 (Fabp7) gene. Fabp7 is one of 9 intracellular fatty acid binding proteins, and it is expressed in the embryonic and adult nervous system, retina and mammary gland [33]. Fabps act as lipid chaperones, promoting cellular uptake and transport of fatty acids and targeting them to specific organelles and metabolic pathways, including the nucleus for transcriptional regulation [45]. Fabp7 is a marker for radial glial cells –the neuronal and glial precursor stem cells- during embryonic neurogenesis [33], [46], [47], where COUP-TFI and COUP-TFII are also expressed and required for the timely neurogenesis to gliogenesis switch [10]. Although Fabp7^−/−^ mice have no anatomical neurologic abnormalities –possibly due to compensatory overexpression of other Fabp homologues-, they do exhibit increased anxiety and fear memory [48], decreased prepulse inhibition (a schizophrenia endophenotype), and decreased neurogenesis in the adult hippocampus [49].

Although the role and expression pattern of Fabp7 is not known in all tissues, our results suggest that COUP-TFI lies upstream of Fapb7 and mediates the regulation of proliferation and differentiation of stem cells during neurogenesis and inner ear development. Several FABP proteins interact with orphan receptors to deliver their ligands in the nucleus, and their transcription rate is in turn regulated in a positive feedback loop by the activated nuclear receptor. For example, the *Fabp1* gene is a PPARα transcriptional target, and FABP1 transports PPARα ligands and can physically interact with this receptor in the nucleus [50]. FABP7 binds long-chain fatty acids (LCFAs), with highest affinity (10^−9^) for docosahexanoic acid (DHA) [51], an essential fatty acid and an abundant lipid component in the nervous system. It is possible that Fabp7 transports a lipid ligand that activates COUP-TFI. A recent study demonstrated that supraphysiologic levels of retinoic acid activate COUP-TFII and used X-ray crystallographic data to propose that ligand binding releases COUP-TFII from a repressed state to allow cofactor binding and regulation of its target genes [52]. However, endogenous ligands for these orphan receptors remain elusive.

As further ‘proof-of–concept’ of our methodology, 3 novel direct COUP-TFI targets *in vitro* - *Crabp1*, *Sod1* and *Casq1*-, as well as one novel target *in vivo* –*Foxo3a-* were validated. These genes are known to be expressed in the cochlea and are relevant for inner ear physiology: *Crabp1* is expressed during cochlear development and controls the intracellular concentration of free retinoids [53], [54] (key hormone regulators of inner ear embryogenesis [55]); *Casq1* participates in the calcium buffering system that is upregulated to protect hair cells from noise trauma and lies within DFNA49, an autosomal dominant non-syndromic hearing loss locus [56]–[58]; and mutations in *Sod1* are associated with early-onset hearing loss in mice [59]. Interestingly, *Foxo3a* regulates metabolic processes and is linked to longevity in species ranging from *C. Elegans* to humans [60], [61], and it is deregulated in the inner ears of calorie-restricted mice [62]. As stated, we will continue the experimental validation of candidate target genes, and will iteratively complement our approach with ChIP-seq analyses for COUP-TFI target genes throughout cochlear and brain development to refine the identification of COUP-TFI targets.

The bioinformatics tool we describe can be modified to scan for response elements of any transcription factor, combinations of several types of response elements, or any kind of flexible DNA pattern. A primary requirement is the availability of a pool of annotated binding sites or SELEX dataset in order to train a Hidden Markov Model. We propose that given the vast number of expression datasets and experimental data already available, this approach can be readily applied to expand the pool of candidate target genes for other nuclear receptors as well.

## Materials and Methods

### Ethics Statement

All studies involving the use of animals were approved by the Baylor College of Medicine Institutional Animal Care and Use Committee according to policies, procedures, and regulations related to the protection of animals mandated by the federal government of the United States.

### Microarray Hybridizations and Data Analysis

Microarray hybridizations were performed on Affymetrix MG-U74Av2 chips through the BCM Microarray Core Facility. Two sets of hybridizations (experimental replicates) were performed at a different point in time, each set consisting of one RNA pool per genotype hybridized in duplicate. Our microarray data is MIAME compliant and has been deposited into the Gene Expression Omnibus (GEO) database under the series accession number GSE16744.

Microarray raw data was normalized using GCRMA [16] and dChip [17] software. Careful inspection revealed a considerable experimental (or time-point) effect in the expression value for many genes. Therefore, the data was analyzed using a 2-way analysis of variance, evaluating for genotype and experimental (time-point) effects. This approach increased the power of the analysis, since some of the genotype random variability was explained by the experimental factor, making it easier to find significant differences. Additionally, genes with a significant interaction (p<0.01) were eliminated from the list, since these theoretically encompass expression differences resulting from tissue contamination during inner ear dissection. Indeed, a gene ontology analysis performed on the interacting genes revealed a significant overrepresentation of multiple striated-muscle related classes (EASE score as low as 1.67E^−18^ [63]), validating our statistical approach. The final microarray hit list was assembled as described in the Results section of this manuscript. Gene ontology analysis was performed using DAVID-EASE software [64].

### RNA Isolation and Real-Time RT-PCR

Whole P0 inner ears were placed in RNA Later (Ambion) after dissection, stored at 4C overnight and then at −20C until genotype was determined [7]; cell culture samples were lysed with Buffer RLT (RNeasy Mini Kit; Qiagen), centrifuged for 2 minutes at 14,000rpm on QIAshredder columns (Qiagen) and stored at −80C. Total RNA was isolated using the RNeasy Mini Kit (Qiagen), following the manufacturer's instructions, and quantified by NanoDrop spectrophotometry (Wilmington, DE). 1.5µg of RNA were reverse-transcribed in a final volume of 20µl using SuperScript II RT (Invitrogen) and oligo-dT primers (Invitrogen). Real-time PCR reactions were performed in an ABI Prism 7000 Sequence Detection System using 0.25µl of cDNA and SYBR Green JumpStart Taq ReadyMix (Sigma). Amplification efficiency was determined via a standard curve, and relative sample quantities were normalized to Cyclophilin A levels as a loading control (See **File S2** for a list of primers). Real-time RT-PCR for COUP-TFII (**Figure S1**) was performed with Taqman probes (Applied Biosystems Mm00772789_m1). Statistical analysis was performed using a one-tailed Student's *t*-test and p = 0.05 as a cutoff.

### Creation of the COUP-TF Target Gene Database and HMM Training Set

In order to collect a reliable list of HMM training sequences, we created a database of all the known COUP-TF targets. The initial list included a total of 69 candidate direct targets (79 binding sites) and included the gene name, species, type of site (as described in the literature), binding site sequence (as described in the literature), position within the gene, activation/repression, competing elements or cofactors, tissues/cells, experiments performed, locus link number, OMIM link and Jackson lab ID. Entries judged to have insufficient experimental evidence for direct binding were eliminated. The nucleotide sequence of each binding site was verified by crosschecking with the UCSC database, and the specific genomic coordinates for each sequence were recorded. To verify the binding site classification (ie, half-site, direct, inverted or everted repeats), we used Consite [65] to scan each sequence with a classical half-site position frequency matrix, and correlated the scores obtained with the experimental evidence. The final candidate HMM training sequences (**Table S2**) correspond to the nucleotides in the plus strand of each site, including one or both half-sites (in capitals), the spacer region (lower case), and 3 nucleotides upstream and downstream of the half-sites (lower case).

### *In Silico* Genomic Prediction of COUP-TF Binding Sites and Conservation Analysis

We created the ChromAnalyzer Java library **(** **Figure 3** **)** to analyze entire chromosomes for matches to any custom Hidden Markov Model. ChromAnalyzer was written as a Java wrapper that communicates with the python bindings of the GHMM C++ library [29]. The input consists of a text file describing the HMM parameters and a text file containing a sequence of any length in fasta format. The output delivered to the enduser consists of BED coordinates for all the HMM matches found in the sequence along with the final count of non-overlapping HMM matches and their classification by size. This output format allows visualization of the results in the UCSC browser, as well as their intersection with any microarray data and other UCSC databases. Entire genomes can be analyzed in this manner in less than 48 hours (measured on a PC with 2.4 GHz dual-core Athlon 64 CPU and 2GB RAM, running Linux Fedora Core 5). We've also created a ChromAnalyzer variant that searches multiple fasta fragments of less than 20,000 bp. Both ChromAnalyzer programs can be obtained directly from the authors on the open source basis. Phylogenetic footprinting was performed by intersecting our microarray and HMM results with the UCSC conservation tracks via the UCSC Table Browser functions [30].

### Isolation of Mouse Embryonic Fibroblasts

Mouse embryos (e12–e14) were removed from the uterus and placed in labeled 6-well plates with PBS. The corresponding yolk sac was placed in a separate tube for genotyping [7]. In the tissue culture hood, embryos were transferred to 3ml of DMEM with 15% FBS, suspended in this media 3 times with an 18G needle, and subsequently added to a 10cm tissue culture plate with 7ml of DMEM and 15% FBS. Cells were left undisturbed for 2 days, and split twice in a 1∶4 ratio before freezing down stocks.

### *In Vitro* and *In Vivo* Chromatin Immunoprecipitation (ChIP)

Wild-type and COUP-TFI^−/−^ MEFs were grown to 80% confluency in 15cm tissue culture plates. Chromatin crosslinking and immunoprecipitation were performed using the Active Motif ChIP-IT™ Express Kit, following the manufacturer's instructions except for the number of washes, which was increased to 6, and the final PCR amplification, which was quantified with ABI Prism 7000 Sequence Detection System using Power SYBR Green PCR Master Mix (Applied Biosystems), 5ul of immunoprecipitated chromatin or input, and the corresponding primer pair (refer to **File S2** for a complete list of primer sets). For *in vivo* ChIPs, P0 brains were placed in 1% formaldehyde immediately after dissection. Tissues were fixed for 15min, and the reaction was then quenched in 0.125M glycine for 5 min. Nuclear extraction, lysis, immunoprecipitation and real-time PCR were performed as described above. Immunoprecipitations were performed overnight at 4C with 3µg of anti-COUP-TFI (Perseus Proteomics Inc. 2ZH8132H), anti-H3K9 (kind gift from Dr. Nikolai A. Timchenko), anti-SCR-1 (Abcam ab84) or anti-CBP (Abcam ab2832) antibodies. Negative IP reactions were performed with mouse IgG (Sigma), or no antibody. Results are plotted as relative binding (percent of input of the corresponding IP/percent of input of negative control IP). COUP-TFI^−/−^ relative binding values were set to 1.

### Western Blot and Protein Quantification

Brain and whole inner ears were flash frozen in liquid nitrogen and stored at −80C immediately after dissection. To obtain whole-cell protein extracts, tissues were lysed in 50mM HEPES (pH 7.5), 150mM NaCl, 0.5% DOC, 1% NP40, 0.05% SDS and protease inhibitor cocktail (Roche) for 30 min on ice and subsequently centrifuged at 14,000rpm for 10min. The supernatant was used for protein quantification with the Bradford assay method (Bio-Rad) and absorbance at 595nm was measured using PowerWave XS spectrophotometer (Witec AG). Seventy-five µg of inner ear total protein and 12µg of brain total protein were analyzed by SDS-PAGE using a 12.5% acrylamide gel. Proteins were transferred to a nitrocellulose membrane (Bio-Rad), and Western Blot was performed by with anti-Fabp7 antibody (Santa Cruz Biotechnology FL-132) in a 1∶200 dilution, and anti-actin antibody (Santa Cruz Biotechnology, sc-47778) at a 1∶1000 dilution, incubated overnight at 4C. Protein quantification was performed with ImageJ software [66] with the Gel Analysis method.

## Supporting Information

Figure S1 COUP-TFII transcript levels are upregulated in the COUP-TFI^−/−^ tissue. A. COUP-TFII transcript levels in wild-type and COUP-TFI^−/−^P0 inner ears (n = 5; * = p<0.05). B. COUP-TFII transcript levels in wild-type and COUP-TFI^−/−^ P0 brain cortex (n = 5; * = p<0.05).
(0.30 MB TIF)

Table S1 COUP-TFI microarray database. Complete gene expression and statistical data for all probes in the Affymetrix MG-U74Av2 microarray chip. The database is indexed by probe ID and includes the following fields from left to right: gene name, description, expression/statistics from the RMA-normalized set (5 columns), expression/statistics from the dChip-normalized set (5 columns), and a final column indicating which probes fulfill the criteria for the final candidate COUP-TFI target gene list (interaction p>0.01; genotype p<0.01 by one normalization method and genotype p<0.05 in the other method: 176 probes total). Individual columns for each normalized set includes the following fields from left to right: expression fold change (wild-type average/mutant average), genotype p-value, time-point (or experimental) p-value, interaction p-value, and a column denoting the initial hit list, i.e., probe sets with genotype p<0.01 and interaction p>0.01 (denoted as “G no I”) in that particular normalization method.
(2.90 MB PDF)

Table S2 HMM training sites. Complete description of the 49 COUP-TF response elements used to calculate the COUP-TF HMM parameters. The included fields are, from left to right: name of the sequence, Locus Link number, gene name, brief gene description, species, competing elements or cofactors (as described in the literature), final HMM training sequence (formatted as described in the Methods section), type of binding site (determined as described in the Methods section), orientation of the response element (plus or minus strand), position within the gene/promoter (as described in the literature), activation or repression by COUP-TF, specific tissues/cells that the experiments were performed in, list of experimental assays performed, absolute genomic coordinates (4 columns), OMIM link and Jackson lab ID number. Genomic coordinates correspond to the galGal3, hg18, mm9, rn4, and StrPur2 assemblies.
(0.04 MB XLS)

Table S3 Complete candidate inner ear COUP-TFI target database. Genes are listed in decreasing rank order. MC = HMM match within a most conserved UCSC site, >0.3 = HMM match with a conservation score greater than 0.3, >0.7 = HMM match with a conservation score greater than 0.7.
(0.06 MB XLS)

File S1 COUP-TF HMM in XML format. XML file denoting the precise transition and emission parameters used for the COUP-TF HMM genomic search.
(0.02 MB TXT)

File S2 List of primers. This file includes two tables: 1) the list of primers used for real-time RT-PCR microarray validation; and 2) the list of primers used in chromatin immunoprecipitations coupled to real-time PCR.
(0.02 MB XLS)
