# Supplementary figures and images for: Genome-Wide Analysis of Binding Sites and Direct Target Genes of the Orphan Nuclear Receptor NR2F1/COUP-TFI
Source: PLoS One. 2010 Jan 27;5(1):e8910. doi: 10.1371/journal.pone.0008910 (PMC2811727; doi:10.1371/journal.pone.0008910)

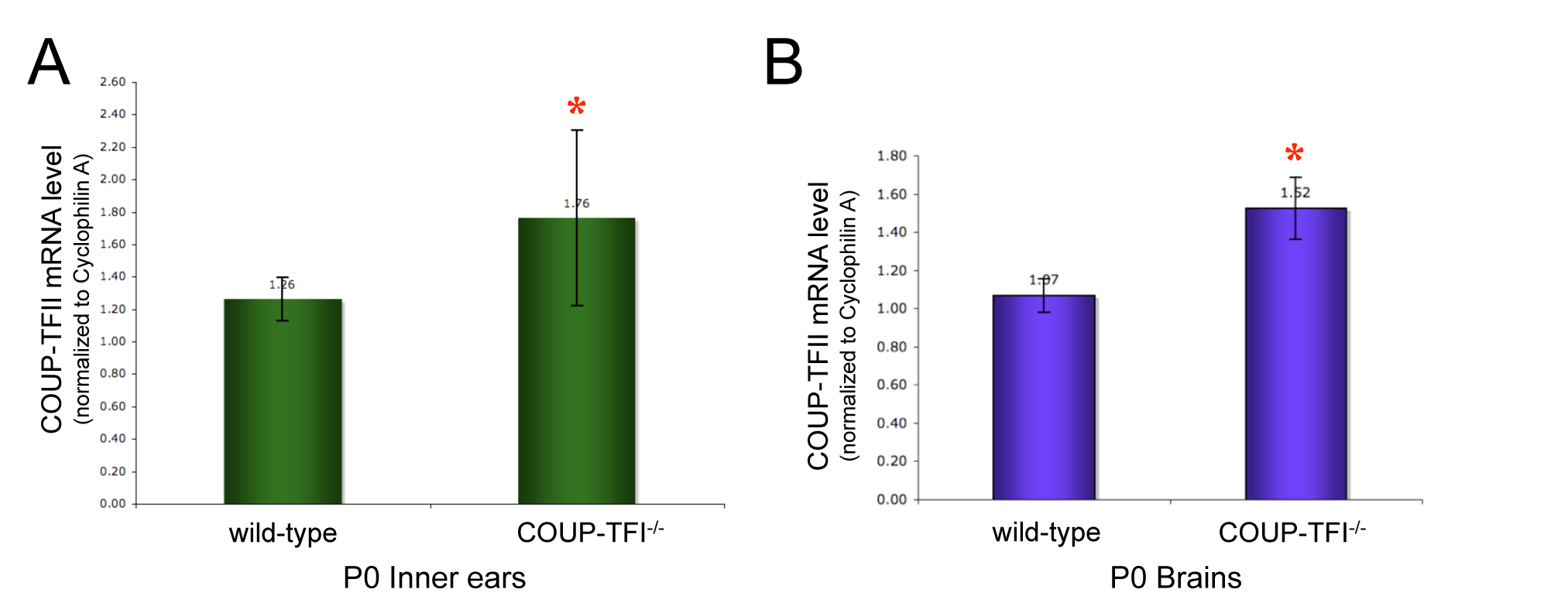

Supplement: Figure S1 — COUP-TFII transcript levels are upregulated in the COUP-TFI−/− tissue. A. COUP-TFII transcript levels in wild-type and COUP-TFI−/−P0 inner ears (n = 5; * = p<0.05). B. COUP-TFII transcript levels in wild-type and COUP-TFI−/− P0 brain cortex (n = 5; * = p<0.05). (0.30 MB TIF) [file pone.0008910.s001.tif]
