# Supplementary material for: Genome-Wide Analysis of Binding Sites and Direct Target Genes of the Orphan Nuclear Receptor NR2F1/COUP-TFI
Source: PLoS One. 2010 Jan 27;5(1):e8910. doi: 10.1371/journal.pone.0008910 (PMC2811727; doi:10.1371/journal.pone.0008910)
Supplement: Table S1 — COUP-TFI microarray database. Complete gene expression and statistical data for all probes in the Affymetrix MG-U74Av2 microarray chip. The database is indexed by probe ID and includes the following fields from left to right: gene name, description, expression/statistics from the RMA-normalized set (5 columns), expression/statistics from the dChip-normalized set (5 columns), and a final column indicating which probes fulfill the criteria for the final candidate COUP-TFI target gene list (interaction p>0.01; genotype p<0.01 by one normalization method and genotype p<0.05 in the other method: 176 probes total). Individual columns for each normalized set includes the following fields from left to right: expression fold change (wild-type average/mutant average), genotype p-value, time-point (or experimental) p-value, interaction p-value, and a column denoting the initial hit list, i.e., probe sets with genotype p<0.01 and interaction p>0.01 (denoted as “G no I”) in that particular normalization method. (2.90 MB PDF) [file pone.0008910.s002.pdf]

			RMA					d Chip					
Probe ID	Gene symbol	Description	Fold change	Genotype p-value	Time point p-value	Interaction p-value	RMA G no I	Fold change	Genotype p-value	Time point p-value	Interaction p-value	dChip G no I	176 final set
100001_at	Cd3g	CD3 antigen, gamma polypeptide	0.8999	0.0998	0.0095	0.1901	No	0.9293	0.224	0.0776	0.033	No	No
100002_at	Itih3	inter-alpha trypsin inhibitor, heavy chain 3	0.9553	0.3479	0.3857	0.1293	No	1.1441	0.0142	0.001	0.0238	No	No
100003_at	Ryr1	ryanodine receptor 1, skeletal muscle	0.6962	0.0034	0.0546	0.0077	No	0.7542	0.008	0.0298	0.0141	Yes	No
100004_at	Ints7	integrator complex subunit 7	1.0393	0.1846	0	0.944	No	1.0004	0.743	0.0046	0.0324	No	No
100005_at	Traf4	Tnf receptor associated factor 4	0.9877	0.2206	0	0.2117	No	1.0128	0.475	0.0008	0.177	No	No
100006_at	Cdh11	cadherin 11	0.9881	0.7832	0.0011	0.0057	No	0.9754	0.76	0.0004	0.002	No	No
100007_at	Irf2bp1	interferon regulatory factor 2 binding protein 1	0.9917	0.8265	0.0849	0.7848	No	1.0387	0.459	0.204	0.573	No	No
100009_r_at	Sox2	SRY-box containing gene 2	1.2806	0.0532	0.0009	0.8138	No	1.2749	0.0624	0.0017	0.588	No	No
100010_at	Klf3	Kruppel-like factor 3 (basic)	0.9898	0.6823	0.1312	0.8543	No	1.0514	0.0296	0.0005	0.0695	No	No
100011_at	Klf3	Kruppel-like factor 3 (basic)	0.9386	0.135	0.0002	0.071	No	1.2162	0.0553	0.705	0.675	No	No
100012_at	Laptm5	lysosomal-associated protein transmembrane 5	1.0145	0.5069	0.0893	0.0111	No	1.0607	0.199	0.125	0.332	No	No
100013_at	Ifi35	interferon-induced protein 35	0.986	0.4421	0.0197	0.8523	No	0.9723	0.375	0.0092	0.0576	No	No
100014_at	Tlk2	tousled-like kinase 2 (Arabidopsis)	1.0179	0.6762	0.6323	0.5609	No	0.902	0.0307	0.0056	0.0642	No	No
100015_at	Yes1	Yamaguchi sarcoma viral (v-yes) oncogene homolog 1	0.9666	0.3645	0.0011	0.2786	No	1.0566	0.582	0.213	0.474	No	No
100016_at	Mmp11	matrix metallopeptidase 11	1.0108	0.5631	0.0019	0.6533	No	1.0787	0.112	0.445	0.435	No	No
100017_at	Mybph	myosin binding protein H	0.7663	0.0016	0.0168	0.1961	Yes	0.8927	0.0263	0.0004	0.487	No	Yes
100018_at	Mtf1	metal response element binding transcription factor 1	0.9976	0.963	0.8062	0.8675	No	1.0317	0.474	0.251	0.303	No	No
100019_at	Cspg2	chondroitin sulfate proteoglycan 2	1.0479	0.4889	0.0063	0.4334	No	0.9564	0.593	0.0377	0.403	No	No
100020_at	Slc4a2	solute carrier family 4 (anion exchanger), member 2	0.9883	0.4997	0.0044	0.2528	No	0.9692	0.119	0.0004	0.934	No	No
100021_at	Chrna1	cholinergic receptor, nicotinic, alpha polypeptide 1 (muscle)	0.9556	0.2207	0.0393	0.7827	No	1.0246	0.703	0.137	0.397	No	No
100022_at	Cish	cytokine inducible SH2-containing protein	0.908	0.0981	0.8057	0.128	No	0.9862	0.787	0.0077	0.664	No	No
100023_at	Mybl2	myeloblastosis oncogene-like 2	1.062	0.1998	0.0196	0.2023	No	1.1926	0.0083	0.0139	0.0103	Yes	No
100024_at	Shrm	shroom	1.1864	0.0931	0.0003	0.1959	No	1.1269	0.138	0.105	0.738	No	No
100026_at	Bcat1	branched chain aminotransferase 1, cytosolic	0.9618	0.2486	0.0019	0.9007	No	0.958	0.0286	0.0002	0.498	No	No
100027_s_at	Pex14	peroxisomal biogenesis factor 14	1.0806	0.0041	0.0206	0.8563	Yes	1.01	0.951	0.0001	0.0029	No	No
100028_r_at	Pex14	peroxisomal biogenesis factor 14	1.0443	0.2281	0.6663	0.3049	No	1.1105	0.229	0.141	0.809	No	No
100029_at	Pex14	peroxisomal biogenesis factor 14	1.0527	0.069	0.0034	0.6923	No	1.0283	0.128	0.0007	0.433	No	No
100030_at	Upp1	uridine phosphorylase 1	0.9925	0.8721	0.0291	0.2135	No	1.0277	0.503	0.0075	0.506	No	No
100032_at	Sp1	trans-acting transcription factor 1	0.9679	0.5994	0.0001	0.485	No	0.9415	0.195	0.0004	0.118	No	No
100033_at	Msh2	mutS homolog 2 (E. coli)	0.9235	0.2712	0.0786	0.2038	No	0.9177	0.0112	0.537	0.0018	No	No
100034_at	Serpinb5	serine (or cysteine) peptidase inhibitor, clade B, member 5	0.972	0.5082	0.0185	0.1223	No	0.9093	0.398	0.0263	0.847	No	No
100035_at	Npr1	natriuretic peptide receptor 1	1.0022	0.9796	0.6177	0.6288	No	1.153	0.209	0.735	0.0393	No	No
100037_at	Ddx18	DEAD (Asp-Glu-Ala-Asp) box polypeptide 18	0.8787	0.0053	0.0107	0.0296	Yes	0.8859	0.0293	0.134	0.071	No	Yes
100039_at	Tmem4	transmembrane protein 4	1.0597	0.445	0.8915	0.741	No	0.9726	0.652	0.0022	0.019	No	No
100040_at	Mrpl17	mitochondrial ribosomal protein L17	1.0697	0.1602	0.0205	0.0558	No	1.0325	0.667	0.232	0.607	No	No
100041_at	D11Ertd333e	DNA segment, Chr 11, ERATO Doi 333, expressed	0.9763	0.525	0.0026	0.1022	No	0.9811	0.794	0.0211	0.28	No	No
100042_at	Hagh	hydroxyacyl glutathione hydrolase	1.0285	0.3614	0.0742	0.2937	No	1.0108	0.72	0.246	0.0942	No	No
100043_f_at	2310020A21 Rik	RIKEN cDNA 2310020A21 gene	0.9557	0.3158	0.7637	0.6684	No	0.896	0.0263	0.0502	0.343	No	No
100044_at	Cldn11	claudin 11	1.0457	0.67	0.0544	0.1912	No	0.988	0.913	0.0074	0.463	No	No
100046_at	Mthfd2	methylenetetrahydrofolate dehydrogenase (NAD+ dependent), methenyltetrahydrofolate cyclohydrolase	1.1186	0.0023	0.0003	0.0262	Yes	1.1468	0.0001	0.0001	0.0007	No	No
100047_at	Snap25	synaptosomal-associated protein 25	1.0601	0.5853	0.0231	0.219	No	1.0506	0.251	0.028	0.0135	No	No
100048_at	Rap1a /// LOC630619 /// LOC640407 /// LOC668838 /// LOC672896 /// LOC677336	RAS-related protein-1a /// similar to Ras-related protein Rap-1A (Ras-related protein Krev-1) /// similar to Ras-related protein Rap-1A (Ras-related protein Krev-1) /// similar to Ras-related protein Rap-1A precursor (Ras-related protein Krev-1) /// similar to Ras-related protein Rap-1A precursor (Ras-related protein Krev-1) /// similar to Ras-related protein Rap-1A precursor (Ras-related protein Krev-1)	1.0267	0.4492	0.0061	0.2568	No	0.9706	0.653	0.105	0.833	No	No
100049_at	Hmga1	high mobility group AT-hook 1	1.0546	0.2221	0.0036	0.8177	No	0.8837	0.269	0.0123	0.161	No	No
100050_at	Id1	inhibitor of DNA binding 1	0.9719	0.4259	0.0108	0.0135	No	0.9175	0.134	0.0078	0.0162	No	No
100051_at	Stom	stomatin	0.9457	0.0827	0.3625	0.9145	No	0.9961	0.82	0.174	0.0563	No	No
100052_at	Stom	stomatin	1.0731	0.3771	0.0085	0.3914	No	1.0471	0.569	0.0053	0.309	No	No
100054_s_at	D2Wsu81e	DNA segment, Chr 2, Wayne State University 81, expressed	0.9304	0.285	0.7706	0.5845	No	0.9194	0.22	0.873	0.497	No	No
100056_at	Fbxw2	F-box and WD-40 domain protein 2	0.9488	0.0962	0.003	0.4949	No	0.9155	0.0345	0.0065	0.0597	No	No
100057_at	Nsmce1	non-SMC element 1 homolog (S. cerevisiae)	1.0288	0.5408	0.01	0.535	No	1.0624	0.151	0.0066	0.436	No	No
100058_at	2810453I06 Rik	RIKEN cDNA 2810453I06 gene	1.0086	0.7095	0.2305	0.5124	No	1.0371	0.168	0.0151	0.217	No	No
100059_at	Cyba	cytochrome b-245, alpha polypeptide	1.0088	0.6745	0.0008	0.895	No	0.9891	0.755	0.0089	0.23	No	No
100060_i_at	Klk1 /// Klk1b5	kallikrein 1 /// kallikrein 1-related peptidase b5	0.8749	0.1032	0.0049	0.0727	No	0.7895	0.239	0.005	0.331	No	No
100061_f_at	Klk1	kallikrein 1	0.9453	0.53	0.0379	0.319	No	0.9102	0.196	0.0025	0.599	No	No
100062_at	LOC671904	similar to DNA replication licensing factor MCM3 (DNA polymerase alpha holoenzyme-associated protein P1) (P1-MCM3)	1.1481	0.0278	0.0053	0.8684	No	1.2459	0.0658	0.0088	0.734	No	No
100064_f_at	Gja1	gap junction membrane channel protein alpha 1	1.0075	0.9214	0.0059	0.2353	No	0.9739	0.657	0.0148	0.333	No	No
100065_r_at	Gja1	gap junction membrane channel protein alpha 1	1	0.9998	0.0037	0.5694	No	1.0065	0.952	0.0119	0.732	No	No
100066_at	Gart	phosphoribosylglycinamide formyltransferase	0.9716	0.3526	0.4329	0.2679	No	1.0098	0.728	0.123	0.462	No	No
100067_at	Akr1b3 /// LOC235852 /// LOC382486 /// LOC385448 /// LOC385495 /// LOC672268	aldo-keto reductase family 1, member B3 (aldose reductase) /// similar to Aldose reductase (AR) (Aldehyde reductase) /// similar to Aldose reductase (AR) (Aldehyde reductase) /// similar to Aldose reductase (AR) (Aldehyde reductase) /// similar to Aldose reductase (AR) (Aldehyde reductase) /// similar to Aldose reductase (AR) (Aldehyde reductase)	1.0489	0.4129	0.008	0.9233	No	0.936	0.21	0.0008	0.887	No	No
100068_at	Aldh1a1	aldehyde dehydrogenase family 1, subfamily A1	0.7479	0.0016	0.0013	0.9424	Yes	0.7527	0.0024	0.0016	0.633	Yes	Yes
100069_at	Cyp2f2	cytochrome P450, family 2, subfamily f, polypeptide 2	1.0361	0.3325	0.1693	0.0137	No	1.1216	0.064	0.0555	0.0762	No	No
100071_at	Mup1 /// Mup2 /// LOC670615	major urinary protein 1 /// major urinary protein 2 /// similar to alpha-2u globulin PGCL3	0.9945	0.8981	0.1555	0.6236	No	0.9986	0.995	0.101	0.824	No	No
100072_at	Wdr39	WD repeat domain 39	0.8377	0.001	0.0316	0.0022	No	0.8689	0.0004	0.0068	0.0009	No	No
100073_at	2510005D08 Rik	RIKEN cDNA 2510005D08 gene	0.9415	0.2095	0.4261	0.1156	No	1.0139	0.732	0.104	0.511	No	No
100074_at	Tmed9	transmembrane emp24 protein transport domain containing 9	1.0424	0.3386	0.1346	0.3457	No	1.0404	0.391	0.378	0.573	No	No
100078_at	Apoa4	apolipoprotein A-IV	1.0343	0.1049	0.0013	0.0314	No	0.9693	0.27	0.0004	0.389	No	No
100079_at	Ndufb9	NADH dehydrogenase (ubiquinone) 1 beta subcomplex, 9	1.0894	0.167	0.9076	0.4457	No	1.0253	0.577	0.0164	0.208	No	No
100080_at	Psp	parotid secretory protein	1.1325	0.1308	0.6609	0.7247	No	1.0163	0.682	0.0155	0.277	No	No
100081_at	Stip1	stress-induced phosphoprotein 1	0.9938	0.9139	0.3879	0.4119	No	1.0616	0.364	0.265	0.511	No	No
100082_at	Tcf1	transcription factor 1	1.034	0.6095	0.725	0.7923	No	1.0059	0.98	0.0685	0.496	No	No
100084_at	Vil2	villin 2	1.1227	0.1361	0.0748	0.3759	No	1.1391	0.0954	0.163	0.511	No	No
100085_at	Ggt1	gamma-glutamyltransferase 1	1.084	0.1545	0.1917	0.2222	No	0.9886	0.81	0.179	0.4	No	No
100086_at	Lrpap1	low density lipoprotein receptor-related protein associated protein 1	0.934	0.0601	0.0006	0.0425	No	0.9514	0.404	0.0028	0.348	No	No
100088_at	Ppp1cb	protein phosphatase 1, catalytic subunit, beta isoform	1.1218	0.4048	0.0198	0.3985	No	1.1553	0.182	0.0193	0.422	No	No

100089_at	Ppic	peptidylprolyl isomerase C	1.1109	0.0668	0.0217	0.9536	No	1.0075	0.921	0.79	0.425	No	No
100091_at	Slc35b1	solute carrier family 35, member B1	1.1373	0.0086	0.004	0.2917	Yes	1.1515	0.0002	0.0001	0.0021	No	No
100093_at	Pctk1	PCTAIRE-motif protein kinase 1	1.0555	0.2928	0.0467	0.4441	No	1.0404	0.219	0.0608	0.735	No	No
100094_at	Supt5h	suppressor of Ty 5 homolog (S. cerevisiae)	0.9506	0.4224	0.0631	0.5462	No	0.9634	0.295	0.0156	0.664	No	No
100095_at	Scarb1	scavenger receptor class B, member 1	1.0718	0.2467	0.0073	0.6968	No	1.1166	0.0299	0.0057	0.537	No	No
100099_at	Smpd1	sphingomyelin phosphodiesterase 1, acid lysosomal	0.9894	0.66	0.8081	0.8791	No	0.9972	0.929	0.105	0.374	No	No
100100_at	Serpinb1a	serine (or cysteine) peptidase inhibitor, clade B, member 1a	1.0366	0.5768	0.1861	0.5266	No	1.0633	0.241	0.0045	0.0076	No	No
100101_at	Snrpa	small nuclear ribonucleoprotein polypeptide A	1.0169	0.801	0.1442	0.7581	No	1.0306	0.546	0.0021	0.364	No	No
100103_f_at	2210010C04 Rik	RIKEN cDNA 2210010C04 gene	1.0918	0.1224	0.0453	0.0399	No	1.0008	0.967	0.0709	0.472	No	No
100104_r_at	2210010C04 Rik	RIKEN cDNA 2210010C04 gene	0.9145	0.0955	0.9789	0.6187	No	0.7623	0.0728	0.334	0.378	No	No
100106_at	Tff3	trefoil factor 3, intestinal	1.0197	0.4787	0.9753	0.2303	No	1.1037	0.0639	0.293	0.122	No	No
100109_s_at	Krt1-13	keratin complex 1, acidic, gene 13	0.9602	0.2022	0.1002	0.4672	No	0.9871	0.382	0.0006	0.0548	No	No
100112_at	Cxcl12	chemokine (C-X-C motif) ligand 12	1.1063	0.0916	0.4625	0.1533	No	1.1103	0.0004	0.0004	0.0679	Yes	No
100113_s_at	Kifap3	kinesin-associated protein 3	0.9753	0.4801	0.1945	0.4937	No	0.9383	0.362	0.295	0.838	No	No
100115_at	2610028H07 Rik	RIKEN cDNA 2610028H07 gene	1.0475	0.2779	0.013	0.0631	No	0.9843	0.809	0.0217	0.578	No	No
100116_at	2810417H13 Rik	RIKEN cDNA 2810417H13 gene	1.1261	0.0036	0.0001	0.3042	Yes	1.3096	0.0242	0.0582	0.21	No	Yes
100117_at	Diablo	diablo homolog (Drosophila)	1.0038	0.9334	0.0078	0.648	No	0.9972	0.963	0.992	0.456	No	No
100120_at	Nid1	nidogen 1	0.9383	0.3552	0.1293	0.3183	No	0.9377	0.417	0.377	0.0518	No	No
100122_at	Gnb5	guanine nucleotide binding protein, beta 5	1.0545	0.1153	0.0004	0.0686	No	0.9747	0.322	0.0006	0.0829	No	No
100123_f_at	Itgb1	integrin beta 1 (fibronectin receptor beta)	1.0012	0.9881	0.0117	0.2555	No	1.0166	0.878	0.142	0.122	No	No
100124_r_at	Itgb1	integrin beta 1 (fibronectin receptor beta)	0.9191	0.1296	0.0767	0.7286	No	0.94	0.338	0.514	0.692	No	No
100125_at	Pa2g4	proliferation-associated 2G4	1.0351	0.3994	0.0297	0.0812	No	1.079	0.146	0.0017	0.018	No	No
100126_at	Chrac1	chromatin accessibility complex 1	1.0086	0.8685	0.4392	0.1061	No	1.0501	0.475	0.467	0.0977	No	No
100127_at	Crabp2	cellular retinoic acid binding protein II	1.2029	0.1044	0.1132	0.8688	No	1.1799	0.0151	0.899	0.657	No	No
100128_at	Cdc2a	cell division cycle 2 homolog A (S. pombe)	1.0437	0.1308	0.0004	0.2458	No	1.0201	0.449	0.0019	0.239	No	No
100130_at	Jun	Jun oncogene	0.9235	0.0002	0.0004	0.2725	Yes	0.8349	0.0006	0.015	0.11	Yes	Yes
100131_at	Scg5	secretogranin V	0.9852	0.773	0.0106	0.1016	No	0.9841	0.579	0.0151	0.163	No	No
100133_at	Fyn	Fyn proto-oncogene	1.0274	0.7572	0.0085	0.3809	No	1.0317	0.771	0.0011	0.227	No	No
100134_at	Eng	endoglin	1.0194	0.4998	0.0138	0.2131	No	0.9975	0.77	0.0102	0.0623	No	No
100136_at	Lamp2	lysosomal membrane glycoprotein 2	0.9944	0.9393	0.6367	0.8359	No	0.954	0.734	0.405	0.729	No	No
100138_f_at	Rbm14	RNA binding motif protein 14	1.024	0.4499	0.0307	0.3617	No	0.8962	0.106	0.381	0.13	No	No
100139_at	Pcsk1n	proprotein convertase subtilisin/kexin type 1 inhibitor	1.0461	0.4321	0.5107	0.8097	No	1.025	0.537	0.376	0.246	No	No
100141_at	Fau	Finkel-Biskis-Reilly murine sarcoma virus (FBR-MuSV) ubiquitously expressed (fox derived)	1.0996	0.2918	0.0382	0.1494	No	1.1581	0.0406	0.0018	0.0073	No	No
100142_at	Dgcr2	DiGeorge syndrome critical region gene 2	0.9918	0.7947	0.0026	0.8568	No	0.9373	0.0033	0	0.835	Yes	No
100143_at	Zyx	zyxin	1.063	0.3799	0.0021	0.8647	No	0.9937	0.471	0.0003	0.003	No	No
100144_at	Ncl	nucleolin	0.9771	0.4889	0.5782	0.473	No	0.9995	0.996	0.213	0.669	No	No
100147_at	Aco1	aconitase 1	1.0331	0.4564	0.0568	0.3431	No	1.1253	0.199	0.468	0.974	No	No
100148_at	Ctcf	CCCTC-binding factor	1.0698	0.6076	0.0246	0.8323	No	1.0625	0.609	0.011	0.936	No	No
100149_at	Mcm6	minichromosome maintenance deficient 6 (MIS5 homolog, S. pombe) (S. cerevisiae)	1.0358	0.6414	0.9218	0.5956	No	1.0526	0.444	0.788	0.5	No	No
100150_f_at	Ins2	insulin II	0.9747	0.6299	0.0344	0.3946	No	1.0712	0.087	0.0012	0.331	No	No
100151_at	Serinc3	serine incorporator 3	0.943	0.3211	0.0017	0.826	No	0.9636	0.592	0.0124	0.897	No	No
100152_at	Ncam1	neural cell adhesion molecule 1	1.0555	0.5074	0.5378	0.3681	No	0.9209	0.391	0.157	0.61	No	No
100153_at	Ncam1	neural cell adhesion molecule 1	0.8696	0.0001	0	0	No	0.9098	0.248	0.0021	0.0329	No	No
100154_at	Tapbp	TAP binding protein	1.0219	0.7193	0.7931	0.9997	No	0.9934	0.954	0.0015	0.107	No	No
100155_at	Ddr1	discoidin domain receptor family, member 1	0.914	0.068	0.2693	0.0581	No	0.9085	0.148	0.311	0.0534	No	No
100156_at	Mcm5	minichromosome maintenance deficient 5, cell division cycle 46 (S. cerevisiae)	1.2	0.01	0.0019	0.2991	Yes	1.2013	0.0117	0.002	0.391	No	Yes
100213_f_at	Rpl41	ribosomal protein L41	0.9948	0.528	0.0004	0.0419	No	0.9905	0.391	0.0206	0.114	No	No
100225_f_at	Psmc3	proteasome (prosome, macropain) 26S subunit, ATPase 3	1.0109	0.6962	0.1451	0.0537	No	1.0827	0.0377	0.308	0.252	No	No
100272_at	Jarid1d	jumonji, AT rich interactive domain 1D (Rbp2 like)	0.9786	0.7798	0.1505	0.4655	No	0.9477	0.841	0.038	0.339	No	No
100277_at	Inhba	inhibin beta-A	1.0451	0.5283	0.4507	0.7694	No	1.0126	0.835	0.255	0.957	No	No
100278_at	Cdkn1b	cyclin-dependent kinase inhibitor 1B (P27)	0.8694	0.0937	0.032	0.0978	No	0.8585	0.0864	0.638	0.0411	No	No
100279_at	St3gal2	ST3 beta-galactoside alpha-2,3-sialyltransferase 2	0.9982	0.9683	0.0738	0.6547	No	0.9683	0.226	0.0026	0.0645	No	No
100280_at	Hist1h1t	histone 1, H1t	0.8919	0.0694	0.0245	0.1569	No	0.9937	0.817	0.117	0.094	No	No
100281_at	Gmcl1	Germ cell-less homolog 1 (Drosophila)	1.034	0.4455	0.1248	0.1619	No	1.0396	0.431	0.605	0.385	No	No
100282_at	Htr7	5-hydroxytryptamine (serotonin) receptor 7	0.9807	0.5374	0.2527	0.423	No	1.035	0.553	0.166	0.965	No	No
100283_at	Sox10	SRY-box containing gene 10	1.0037	0.8978	0.0364	0.4646	No	0.9448	0.48	0.655	0.383	No	No
100284_at	Zic4	zinc finger protein of the cerebellum 4	1.0222	0.6808	0.067	0.8063	No	0.9211	0.286	0.0792	0.426	No	No
100285_at	Col4a3	procollagen, type IV, alpha 3	1.0539	0.4949	0.054	0.4549	No	0.9833	0.879	0.0262	0.127	No	No
100286_at	Mst1	macrophage stimulating 1 (hepatocyte growth factor-like)	0.968	0.5482	0.0024	0.9838	No	0.9975	0.893	0.0013	0.747	No	No
100287_at	Igbp1b	immunoglobulin (CD79A) binding protein 1b	1.0206	0.7057	0.2526	0.5802	No	1.0536	0.501	0.848	0.859	No	No
100289_at	Efna3	ephrin A3	1.0091	0.8608	0.0446	0.2895	No	0.9102	0.0493	0.0655	0.26	No	No
100290_f_at	6230416J20 Rik	RIKEN cDNA 6230416J20 gene	1.0022	0.9737	0.0334	0.1652	No	0.9503	0.686	0.014	0.337	No	No
100291_at	Cbl	Casitas B-lineage lymphoma	0.9542	0.5526	0.9467	0.6039	No	0.9151	0.073	0.0075	0.0159	No	No
100292_at	Sh2d2a	SH2 domain protein 2A	0.9405	0.0887	0.2104	0.5063	No	0.9247	0.0781	0.837	0.0474	No	No
100293_at	Cd59b	CD59b antigen	1.163	0.0804	0.4401	0.0555	No	1.1502	0.047	0.0865	0.0535	No	No
100294_at	Dffb	DNA fragmentation factor, beta subunit	0.9679	0.5631	0.1799	0.9415	No	1.0276	0.501	0.0936	0.0744	No	No
100295_at	LOC207685	hypothetical protein LOC207685	0.994	0.9095	0.0711	0.8219	No	0.9908	0.537	0.0017	0.0453	No	No
100296_at	Tex2	testis expressed gene 2	1.0357	0.2763	0.9642	0.0382	No	1.0409	0.286	0.0081	0.0359	No	No
100297_at	Wdr26	WD repeat domain 26	1.0134	0.8608	0.0002	0.6624	No	1.0209	0.768	0.0001	0.912	No	No
100298_at	Pspn	persephin	0.9911	0.8482	0.0373	0.8142	No	1.0487	0.105	0.0039	0.063	No	No
100299_f_at	LOC636944 /// LOC672386 /// LOC672390	similar to Ig kappa chain V-V region K2 precursor /// similar to Ig kappa chain V-V region K2 precursor /// similar to Ig kappa chain V-V region L7 precursor	0.9744	0.5211	0.3	0.8887	No	1.0094	0.98	0.658	0.974	No	No
100300_at	Cybb	cytochrome b-245, beta polypeptide	0.9555	0.4609	0.0635	0.1416	No	0.8981	0.0829	0.0071	0.24	No	No
100301_at	Esrrb	estrogen related receptor, beta	0.9543	0.4551	0.064	0.292	No	0.9965	0.838	0.034	0.0722	No	No
100302_at	Maff	v-maf musculoaponeurotic fibrosarcoma oncogene family, protein F (avian)	0.8563	0.0558	0.0064	0.2543	No	0.8503	0.062	0.0099	0.0566	No	No
100303_at	H2-M10.1	histocompatibility 2, M region locus 10.1	1.0262	0.418	0.0283	0.205	No	0.8795	0.31	0.0183	0.113	No	No
100304_at	H2-M10.1	histocompatibility 2, M region locus 10.1	1.0407	0.5496	0.7836	0.6832	No	1.0691	0.347	0.848	0.336	No	No
100305_g_at	H2-M10.1	histocompatibility 2, M region locus 10.1	0.9771	0.6301	0.5714	0.3429	No	0.9251	0.0538	0.0009	0.12	No	No
100306_at	2700007P21 Rik	RIKEN cDNA 2700007P21 gene	0.9694	0.1679	0.2866	0.4806	No	0.969	0.738	0.765	0.685	No	No
100307_at	Nfix	nuclear factor I/X	0.9756	0.4891	0.005	0.3784	No	0.8179	0.0733	0.0008	0.859	No	No
100308_at	Col8a1	procollagen, type VIII, alpha 1	1.3108	0.0002	0	0.0025	No	1.1955	0.0212	0.0002	0.266	No	No
100309_at	Met	met proto-oncogene	0.8875	0.0761	0.3703	0.1913	No	0.9158	0.223	0.201	0.205	No	No
100311_f_at	Ear1	eosinophil-associated, ribonuclease A family, member 1	0.9626	0.351	0.0106	0.7935	No	1.1733	0.0385	0.108	0.406	No	No
100313_at	Itga8	integrin alpha 8	0.9779	0.7219	0.2254	0.7761	No	1.0638	0.405	0.423	0.133	No	No
100314_f_at	Itga8	integrin alpha 8	1.0235	0.7607	0.2377	0.5614	No	1.0859	0.236	0.0332	0.848	No	No
100315_at	Atp8a1	ATPase, aminophospholipid transporter (APLT), class I, type 8A, member 1	1.01	0.9098	0.4804	0.9958	No	1.0387	0.52	0.21	0.0694	No	No
100317_at	Ppef2	protein phosphatase, EF hand calcium-binding domain 2	0.9351	0.4228	0.4651	0.9607	No	0.9639	0.512	0.189	0.131	No	No
100318_at	Dnajb13	DnaJ (Hsp40) related, subfamily B, member 13	0.9403	0.1598	0.1098	0.6367	No	0.9897	0.444	0.0003	0.106	No	No
100319_at	Il10	interleukin 10	0.996	0.9347	0.2182	0.1553	No	1.048	0.297	0.213	0.452	No	No
100320_at	Kpna4	karyopherin (importin) alpha 4	0.9612	0.5462	0.006	0.2287	No	1.0107	0.897	0.0477	0.931	No	No
100321_f_at	2210418O10 Rik	RIKEN cDNA 2210418O10 gene	1.069	0.4554	0.4776	0.3595	No	0.9079	0.242	0.0318	0.166	No	No

100322_at	---	---	1.0786	0.1478	0.0327	0.3022	No	1.1351	0.0416	0.0265	0.943	No	No
100323_at	Amd1 /// Amd2	S-adenosylmethionine decarboxylase 1 /// S-adenosylmethionine decarboxylase 2	1.0311	0.4413	0.0003	0.3923	No	1.0455	0.26	0.004	0.599	No	No
100324_g_at	Amd1 /// Amd2	S-adenosylmethionine decarboxylase 1 /// S-adenosylmethionine decarboxylase 2	1.0559	0.4015	0.0787	0.3436	No	1.1142	0.1	0.164	0.331	No	No
100325_at	Gp49a	glycoprotein 49 A	1.2888	0.0222	0.4301	0.604	No	1.0971	0.292	0.03	0.56	No	No
100326_f_at	Klra10 /// Klra8 /// Klra21 /// LOC640965 /// Klra29 /// LOC667769 /// LOC673223	killer cell lectin-like receptor subfamily A, member 10 /// killer cell lectin-like receptor, subfamily A, member 8 /// killer cell lectin-like receptor subfamily A, member 21 /// similar to killer cell lectin-like receptor subfamily A, member 21 /// killer cell lectin-like receptor subfamily A member 29 /// similar to killer cell lectin-like receptor subfamily A, member 21 /// similar to Killer cell lectin-like receptor 8 (T-cell surface glycoprotein Ly-49H) (Ly49-H antigen) (Lymphocyte antigen 49H)	0.9832	0.4543	0.106	0.6462	No	1.0581	0.104	0.0052	0.0647	No	No
100327_at	Lta	lymphotoxin A	1.1475	0.0769	0.001	0.132	No	1.1142	0.0664	0.0011	0.0914	No	No
100328_s_at	Pira1 /// Pira11 /// Pira2 /// Pira3 /// Pira4 /// Pira6 /// Lilrb3 /// LOC619608 /// LOC664887 /// LOC669449 /// LOC669458 /// LOC669506 /// LOC675749	paired-Ig-like receptor A1 /// paired-Ig-like receptor A11 /// paired-Ig-like receptor A2 /// paired-Ig-like receptor A3 /// paired-Ig-like receptor A4 /// paired-Ig-like receptor A6 /// leukocyte immunoglobulin-like receptor, subfamily B (with TM and ITIM domains), member 3 /// hypothetical LOC619608 /// similar to paired-Ig-like receptor A6 isoform a /// similar to paired Ig-like receptor A11 /// similar to paired-Ig-like receptor A11 /// similar to paired-Ig-like receptor A6 isoform a /// similar to paired-Ig-like receptor A1	0.9685	0.4032	0.041	0.3442	No	0.9334	0.367	0.0128	0.913	No	No
100329_at	Serpina1a /// Serpina1b /// Serpina1c	serine (or cysteine) peptidase inhibitor, clade A, member 1a /// serine (or cysteine) preptidase inhibitor, clade A, member 1b /// serine (or cysteine) peptidase inhibitor, clade A, member 1c	1.0473	0.6396	0.0562	0.5338	No	1.0896	0.246	0.362	0.837	No	No
100330_at	Prdx2 /// LOC633295	peroxiredoxin 2 /// similar to Peroxiredoxin-2 (Thioredoxin peroxidase 1) (Thioredoxin-dependent peroxide reductase 1) (Thiol-specific antioxidant protein) (TSA)	0.9592	0.5151	0.884	0.6855	No	1.1316	0.129	0.0421	0.168	No	No
100331_g_at	Prdx2	peroxiredoxin 2	1.0828	0.1012	0.5386	0.1982	No	1.067	0.119	0.219	0.135	No	No
100332_s_at	Prdx6 /// Prdx6-rs1	peroxiredoxin 6 /// peroxiredoxin 6, related sequence 1	1.0405	0.5676	0.2398	0.7148	No	1.0265	0.449	0.0216	0.822	No	No
100333_at	Saa2	serum amyloid A 2	1.0118	0.9043	0.0556	0.4092	No	1.002	0.942	0.151	0.581	No	No
100334_f_at	Egfbp2	epidermal growth factor binding protein type B	0.8321	0.0057	0.0255	0.069	Yes	0.7639	0.0005	0.001	0.0045	No	No
100335_at	Atp7b	ATPase, Cu++ transporting, beta polypeptide	0.9541	0.4763	0.979	0.561	No	0.9756	0.43	0.0047	0.521	No	No
100336_s_at	Bglap-rs1 /// Bglap1 /// Bglap2	bone gamma-carboxyglutamate protein, related sequence 1 /// bone gamma carboxyglutamate protein 1 /// bone gamma-carboxyglutamate protein 2	0.9576	0.2195	0.0001	0.0249	No	0.984	0.649	0	0.0353	No	No
100337_at	Cacna1e	calcium channel, voltage-dependent, R type, alpha 1E subunit	0.9827	0.7303	0.0498	0.5975	No	1.074	0.0494	0.642	0.0044	No	No
100338_s_at	Cacna1e	calcium channel, voltage-dependent, R type, alpha 1E subunit	1.0147	0.8928	0.8366	0.7133	No	1.0569	0.158	0.0035	0.22	No	No
100339_at	Slc10a1	solute carrier family 10 (sodium/bile acid cotransporter family), member 1	0.9919	0.9251	0.4231	0.9358	No	1.0112	0.895	0.989	0.853	No	No
100340_at	Slc10a1	solute carrier family 10 (sodium/bile acid cotransporter family), member 1	0.9682	0.3136	0.7694	0.3728	No	0.892	0.317	0.573	0.141	No	No
100341_g_at	Slc10a1	solute carrier family 10 (sodium/bile acid cotransporter family), member 1	0.9832	0.8157	0.122	0.9054	No	1.0383	0.607	0.11	0.956	No	No
100342_i_at	Tuba1	tubulin, alpha 1	1.0368	0.2234	0.0001	0.1537	No	1	0.85	0	0.282	No	No
100343_f_at	Tuba1	tubulin, alpha 1	1.1022	0.0509	0.1772	0.0233	No	1.0583	0.0125	0.347	0.0301	No	No
100344_at	---	---	1.0517	0.4129	0.0598	0.0524	No	0.979	0.905	0.0005	0.0378	No	No
100345_f_at	Vamp8	vesicle-associated membrane protein 8	0.9673	0.3697	0.0011	0.0408	No	0.9616	0.551	0.0026	0.0676	No	No
100346_at	Fgf9	fibroblast growth factor 9	0.9985	0.9755	0.2531	0.8587	No	1.0041	0.919	0.295	0.793	No	No
100347_at	D17892	expressed sequence D17892	1.0283	0.3513	0.0152	0.029	No	0.9261	0.0066	0.0001	0.14	Yes	No
100348_at	LOC665081	hypothetical protein LOC665081	0.9824	0.8136	0.3496	0.543	No	1.0028	0.916	0.239	0.39	No	No
100349_at	---	---	0.9905	0.875	0.0656	0.7585	No	0.9735	0.644	0.152	0.77	No	No
100350_at	---	---	1.0522	0.4121	0.0646	0.0525	No	1.1269	0.235	0.0544	0.0499	No	No
100351_f_at	Defcr2 /// Defcr3 /// Defcr17 /// Defcr23 /// LOC626708	defensin related cryptdin 2 /// defensin related cryptdin 3 /// defensin related cryptdin 17 /// defensin-related cryptdin 23 /// defensin related cryptdin 26	1.0345	0.2644	0.6551	0.0372	No	0.9706	0.406	0.331	0.0384	No	No
100352_at	Hspa4	heat shock protein 4	0.9328	0.1292	0.0207	0.0362	No	0.9571	0.351	0.0628	0.0896	No	No
100353_g_at	Hspa4	heat shock protein 4	1.0037	0.9032	0.3534	0.4306	No	0.9088	0.0345	0.631	0.0355	No	No
100354_at	Tbx15	T-box 15	0.812	0.0054	0.0828	0.1715	Yes	0.8199	0.0434	0.0781	0.748	No	Yes
100355_g_at	Tbx15	T-box 15	0.9277	0.0933	0.0236	0.1252	No	0.8886	0.0069	0.0355	0.0583	Yes	No
100356_at	Gprasp1	G protein-coupled receptor associated sorting protein 1	1.011	0.7848	0.7021	0.935	No	0.9383	0.304	0.251	0.296	No	No
100357_g_at	Gprasp1	G protein-coupled receptor associated sorting protein 1	1.0399	0.5529	0.886	0.821	No	0.994	0.781	0.0561	0.867	No	No
100358_s_at	Tcp10a /// Tcp10b /// Tcp10c	t-complex protein 10a /// t-complex protein 10b /// t-complex protein 10c	0.9844	0.8201	0.0419	0.433	No	1.0378	0.466	0.0458	0.847	No	No
100359_at	Tcp10b	t-complex protein 10b	1.1045	0.278	0.132	0.646	No	1.0108	0.447	0.0001	0.105	No	No

100360_f_at	Igh-4 /// Igh-6 /// Igh-VJ558 /// LOC238447 /// Igh-1a /// LOC435333 /// LOC619994 /// LOC629882 /// LOC636017 /// LOC636441 /// LOC641157 /// LOC668577 /// LOC668589 /// LOC668591 /// LOC671088 /// LOC671729 ///	immunoglobulin heavy chain 4 (serum IgG1) /// immunoglobulin heavy chain 6 (heavy chain of IgM) /// immunoglobulin heavy chain (J558 family) /// similar to immunoglobulin heavy chain variable region /// immunoglobulin heavy chain 1a (serum IgG2a) /// similar to monoclonal antibody heavy chain /// similar to Ig heavy chain V region VH558 A1/A4 precursor /// similar to Ig heavy chain V region 108A precursor /// similar to Ig heavy chain V region 93G7 precursor /// similar to Ig heavy chain V region VH558 A1/A4 precursor /// similar to Ig heavy chain V region VH558 A1/A4 precursor /// similar to Ig heavy chain V region 93G7 precursor /// similar to Ig heavy chain V region VH558 A1/A4 precursor /// similar to Ig heavy chain V region VH558 A1/A4 precursor /// similar to Ig heavy chain V region 36-65 /// similar to Ig heavy chain V region VH558 A1/A4 precursor /// similar to Ig heavy chain V-I region V35 precursor /// similar to Ig heavy chain V-I region V35 precursor /// similar to Ig heavy chain V region 93G7 precursor /// similar to Ig heavy chain V region 93G7 precursor /// similar to Ig heavy ch	0.9854	0.7999	0.1087	0.57	No	0.9942	0.871	0.951	0.337	No	No
100361_f_at	Igh-1a	Immunoglobulin heavy chain 1a (serum IgG2a)	1.0119	0.7294	0.2833	0.4821	No	1.0218	0.592	0.418	0.1	No	No
100362_f_at	Igh-4 /// Igh-6 /// Igh-VJ558 /// Igh-1a /// LOC434609 /// LOC435333 /// LOC619994 /// LOC630549 /// LOC641157 /// LOC668589 /// LOC671729 /// LOC675659 /// LOC677445	immunoglobulin heavy chain 4 (serum IgG1) /// immunoglobulin heavy chain 6 (heavy chain of IgM) /// immunoglobulin heavy chain (J558 family) /// immunoglobulin heavy chain 1a (serum IgG2a) /// similar to Ig heavy chain V region VH558 A1/A4 precursor /// similar to monoclonal antibody heavy chain /// similar to Ig heavy chain V region VH558 A1/A4 precursor /// similar to Ig heavy chain V region B1-8/186-2 precursor /// similar to Ig heavy chain V region VH558 A1/A4 precursor /// similar to Ig heavy chain V region VH558 A1/A4 precursor /// similar to Ig heavy chain V region VH558 A1/A4 precursor /// similar to Ig heavy chain V region 102 precursor /// similar to Ig heavy chain V region VH558 A1/A4 precursor	0.9276	0.2783	0.5433	0.3335	No	0.9708	0.411	0.0159	0.512	No	No
100363_at	Syt9	synaptotagmin IX	0.9808	0.7774	0.2199	0.4172	No	0.9949	1	0.0081	0.538	No	No
100364_at	Syt6	synaptotagmin VI	0.8983	0.0039	0.006	0.1122	Yes	0.9621	0.359	0.276	0.984	No	No
100365_at	Syt7	synaptotagmin VII	0.9801	0.8496	0.373	0.514	No	1.0342	0.537	0.155	0.312	No	No
100366_at	Casp9	caspase 9	1.0026	0.9455	0.9369	0.2101	No	1.0245	0.633	0.0856	0.998	No	No
100367_g_at	Casp9	caspase 9	1.057	0.042	0.0032	0.7986	No	0.9464	0.492	0.338	0.41	No	No
100368_at	Casp9	caspase 9	0.925	0.0286	0.0009	0.0628	No	0.9972	0.985	0.0121	0.0839	No	No
100369_at	St6galnac6	ST6 (alpha-N-acetyl-neuraminyl-2,3-beta-galactosyl-1,3)-N-acetylgalactosaminide alpha-2,6-sialyltransferase 6	0.9639	0.3085	0.0148	0.4235	No	1.0038	0.946	0.135	0.431	No	No
100370_at	Slc12a5	solute carrier family 12, member 5	0.9555	0.5047	0.0407	0.9962	No	1.0225	0.625	0.236	0.877	No	No
100371_at	Hnrpa1 /// LOC225307 /// LOC665646	heterogeneous nuclear ribonucleoprotein A1 /// heterogeneous nuclear ribonucleoprotein A1 pseudogene /// similar to Heterogeneous nuclear ribonucleoprotein A1 (Helix-destabilizing protein) (Single-strand binding protein) (hnRNP core protein A1) (HDP-1) (Topoisomerase-inhibitor suppressed)	0.9716	0.7374	0.2118	0.2036	No	1.0071	0.85	0.0721	0.156	No	No
100372_at	Gabrg1	gamma-aminobutyric acid (GABA-A) receptor, subunit gamma 1	1.0494	0.2819	0.2246	0.0059	No	1.1321	0.328	0.0001	0.11	No	No
100373_at	Slc39a1	solute carrier family 39 (zinc transporter), member 1	0.9972	0.9377	0.0189	0.7858	No	1.0542	0.487	0.0115	0.397	No	No
100374_at	Dmrt1	doublesex and mab-3 related transcription factor 1	0.9897	0.8312	0.7873	0.9132	No	0.971	0.594	0.169	0.335	No	No
100375_i_at	Igh-4 /// Igh-VJ558 /// LOC380823 /// LOC382696 /// VH186.2-D-J-C mu /// LOC544903 /// LOC619833 /// LOC629941 /// LOC631531 /// LOC636134 /// LOC636244 /// LOC637785 /// LOC637794 /// LOC640207 /// LOC640979 ///	immunoglobulin heavy chain 4 (serum IgG1) /// immunoglobulin heavy chain (J558 family) /// similar to Ig heavy chain V region B1-8/186-2 precursor /// similar to Ig heavy chain V region 3 precursor /// similar to monoclonal antibody heavy chain /// similar to immunoglobulin mu-chain /// similar to Ig heavy chain V region 102 precursor /// similar to Ig heavy chain V region 3 precursor /// similar to Ig heavy chain V region VH558 B4 precursor /// similar to Ig heavy chain V region 3 precursor /// similar to Ig heavy chain V region 102 precursor /// similar to Ig heavy chain V region 3 precursor /// similar to Ig heavy chain V region 3 precursor /// similar to Ig heavy chain V region 102 precursor /// similar to Ig heavy chain V region 23 precursor /// similar to Ig heavy chain V region 3 precursor /// similar to Ig heavy chain V region 3 precursor /// similar to Ig heavy chain V region 145 precursor /// similar to Ig heavy chain V region 102 precursor /// similar to Ig heavy chain V region 3 precursor	0.9658	0.6174	0.0671	0.3055	No	1.0813	0.522	0.116	0.615	No	No
100376_f_at	Igh-VJ558	Anti-human CD20 antibody 1F5 gamma heavy chain variable region /// Immunoglobulin heavy chain (J558 family) /// Immunoglobulin heavy chain (J558 family)	0.9334	0.3045	0.7278	0.5094	No	0.9439	0.428	0.744	0.671	No	No
100377_f_at	LOC634206 /// LOC634222 /// LOC634284 /// LOC637000 /// LOC640522	similar to Ig heavy chain V-I region V35 precursor /// similar to Ig heavy chain V-I region V35 precursor /// similar to Ig heavy chain V-I region V35 precursor /// similar to Ig heavy chain V-I region V35 precursor /// similar to Ig heavy chain V region 108A precursor	0.9449	0.3769	0.044	0.7202	No	1.0435	0.644	0.612	0.114	No	No
100378_at	---	---	1.0598	0.563	0.7057	0.756	No	0.9659	0.598	0.124	0.216	No	No
100379_f_at	---	---	0.9748	0.6301	0.0077	0.0588	No	0.9832	0.767	0.133	0.511	No	No
100380_at	H3f3a	H3 histone, family 3A	1.1847	0.106	0.0094	0.0886	No	1.0668	0.306	0.004	0.781	No	No
100381_at	Acta1	actin, alpha 1, skeletal muscle	0.7345	0.0013	0.0009	0.001	No	0.8421	0.0023	0.0019	0.0005	No	No
100382_at	St6galnac1	ST6 (alpha-N-acetyl-neuraminyl-2,3-beta-galactosyl-1,3)-N-acetylgalactosaminide alpha-2,6-sialyltransferase 1	0.9614	0.7146	0.0576	0.4584	No	1.1593	0.0344	0.001	0.0225	No	No
100383_at	Zfp179	zinc finger protein 179	1.0355	0.6532	0.2908	0.8963	No	1.0095	0.865	0.832	0.564	No	No

100384_at	Hsf4	heat shock transcription factor 4	0.959	0.5065	0.0275	0.731	No	0.9295	0.37	0.183	0.345	No	No
100385_at	Glra1	glycine receptor, alpha 1 subunit	0.9185	0.1339	0.2753	0.3323	No	1.0287	0.782	0.839	0.287	No	No
100386_at	Gnaz	guanine nucleotide binding protein, alpha z subunit	1.0214	0.5758	0.0098	0.5564	No	1.0267	0.671	0.913	0.176	No	No
100387_f_at	Klra12 /// Klra13 /// Klra4 /// Klra23 /// Klra18 /// LOC634585 /// Klra33 /// LOC673407	killer cell lectin-like receptor subfamily A, member 12 /// killer cell lectin-like receptor subfamily A, member 13 /// killer cell lectin-like receptor, subfamily A, member 4 /// killer cell lectin-like receptor subfamily A, member 23 /// killer cell lectin-like receptor, subfamily A, member 18 /// similar to Killer cell lectin-like receptor 4 (T-cell surface glycoprotein Ly-49D) (Ly49-D antigen) (Lymphocyte antigen 49D) /// killer cell lectin-like receptor subfamily A member 33 /// similar to killer cell lectin-like receptor subfamily A, member 13	1.0084	0.7956	0.5857	0.787	No	1.1399	0.313	0.184	0.772	No	No
100388_at	Gnao1	guanine nucleotide binding protein, alpha o	1.0328	0.5277	0.99	0.4872	No	1.0013	0.94	0.0668	0.294	No	No
100389_at	Colq	collagen-like tail subunit (single strand of homotrimer) of asymmetric acetylcholinesterase	0.9924	0.9213	0.0418	0.5343	No	1.0151	0.895	0.0072	0.0538	No	No
100390_s_at	Bfsp1	beaded filament structural protein in lens-CP94	0.955	0.4078	0.0131	0.1018	No	0.9724	0.818	0.152	0.553	No	No
100391_at	Mapk8	mitogen activated protein kinase 8	0.9428	0.0493	0.0462	0.0575	No	1.0003	0.95	0.105	0.977	No	No
100392_at	Apln	apelin	0.9481	0.1785	0.0086	0.5763	No	0.9204	0.0191	0.0013	0.511	No	No
100393_at	Vip	vasoactive intestinal polypeptide	0.9215	0.5901	0.6485	0.7622	No	0.9102	0.238	0.675	0.181	No	No
100394_at	Podxl	podocalyxin-like	1.0658	0.0902	0.022	0.1277	No	1.0365	0.905	0.126	0.0739	No	No
100395_at	Gli2	GLI-Kruppel family member GLI2	0.9109	0.0025	0.0823	0.3581	Yes	0.9917	0.959	0.936	0.806	No	No
100396_i_at	Tyms-ps	thymidylate synthase, pseudogene	1.1019	0.1188	0.0748	0.4532	No	0.9121	0.303	0.0002	0.0132	No	No
100397_at	Tyrobp	TYRO protein tyrosine kinase binding protein	0.8719	0.0192	0.3724	0.0097	No	1.0384	0.642	0.554	0.248	No	No
100398_at	Kif3a	kinesin family member 3A	0.9508	0.3211	0.459	0.5483	No	1.0036	0.959	0.0531	0.718	No	No
100400_at	4921531G14Rik	RIKEN cDNA 4921531G14 gene	1	0.9999	0.0045	0.2782	No	0.984	0.837	0.003	0.908	No	No
100401_at	Son	Son cell proliferation protein	1.038	0.1977	0.0123	0.3611	No	0.9675	0.67	0.0056	0.678	No	No
100402_f_at	Zp3r	zona pellucida 3 receptor	1.0829	0.0289	0.0066	0.6768	No	1.0234	0.584	0.0055	0.589	No	No
100403_at	Myl7	myosin, light polypeptide 7, regulatory	1.0468	0.22	0.0008	0.863	No	1.0838	0.172	0.0038	0.634	No	No
100404_at	Ift88	intraflagellar transport 88 homolog (Chlamydomonas)	1.0995	0.1594	0.8223	0.0715	No	1.0168	0.392	0.0023	0.094	No	No
100405_at	Cbx3 /// LOC629578 /// LOC632383 /// LOC633016 /// LOC672750 /// LOC675988	chromobox homolog 3 (Drosophila HP1 gamma) /// similar to chromobox homolog 3 /// similar to chromobox homolog 3 /// similar to chromobox homolog 3 /// similar to chromobox homolog 3 /// similar to chromobox homolog 3	0.9419	0.2041	0.0005	0.9648	No	1.0742	0.201	0.0002	0.829	No	No
100406_at	Ptpn5	protein tyrosine phosphatase, non-receptor type 5	0.9277	0.236	0.6505	0.4956	No	0.9125	0.162	0.81	0.319	No	No
100407_at	Gal	galanin	1.0418	0.3434	0.0961	0.5727	No	1.1186	0.0523	0.255	0.938	No	No
100408_at	Pole3	polymerase (DNA directed), epsilon 3 (p17 subunit)	1.0703	0.1884	0.026	0.3433	No	1.0414	0.495	0.0181	0.95	No	No
100409_at	Cdh3	cadherin 3	0.982	0.7271	0.5975	0.3911	No	0.9258	0.249	0.986	0.393	No	No
100410_at	C330027G06Rik	RIKEN cDNA C330027G06 gene	0.9395	0.2234	0.1605	0.2269	No	0.9542	0.358	0.438	0.638	No	No
100411_at	---	---	0.9292	0.1319	0.514	0.151	No	0.8893	0.47	0.784	0.228	No	No
100412_g_at	Aebp1	AE binding protein 1	0.9675	0.389	0.0018	0.1122	No	0.9539	0.256	0.0019	0.271	No	No
100413_at	Ylpm1	YLP motif containing 1	0.9135	0.0903	0.1487	0.0936	No	0.9688	0.309	0.169	0.0128	No	No
100414_s_at	Mpo	myeloperoxidase	0.9697	0.5722	0.4202	0.301	No	0.883	0.334	0.0286	0.0861	No	No
100415_at	Mpo	myeloperoxidase	0.9847	0.8663	0.6407	0.8665	No	0.934	0.285	0.0676	0.177	No	No
100416_at	Melk	maternal embryonic leucine zipper kinase	0.9716	0.5882	0.791	0.4122	No	1.095	0.0897	0.0004	0.0536	No	No
100417_at	Slc7a6	solute carrier family 7 (cationic amino acid transporter, y+ system), member 6	1.0018	0.9602	0.003	0.0241	No	1.0457	0.283	0.0244	0.0106	No	No
100418_at	Gng2	guanine nucleotide binding protein (G protein), gamma 2 subunit	0.9464	0.402	0.0746	0.669	No	0.9817	0.493	0.0648	0.139	No	No
100420_at	Flg /// LOC620355 /// LOC675741	filaggrin /// similar to Filaggrin /// similar to Filaggrin	0.9578	0.1276	0.0027	0.9384	No	0.9337	0.16	0.0603	0.845	No	No
100421_at	Krt2-4	keratin complex 2, basic, gene 4	1.0945	0.1883	0.8898	0.5586	No	1.1527	0.0465	0.583	0.464	No	No
100422_i_at	Stat5a	signal transducer and activator of transcription 5A	0.8579	0.0026	0.0487	0.0029	No	0.9618	0.286	0.981	0.016	No	No
100423_f_at	Stat5a	signal transducer and activator of transcription 5A	0.9387	0.2918	0.3348	0.7643	No	0.9829	0.672	0.263	0.851	No	No
100424_at	Ercc1	excision repair cross-complementing rodent repair deficiency, complementation group 1	1.0492	0.2809	0.1296	0.0471	No	1.0091	0.897	0.657	0.86	No	No
100425_at	Syk	spleen tyrosine kinase	0.9837	0.5333	0.438	0.1449	No	1.0166	0.68	0.963	0.36	No	No
100426_s_at	Syk	spleen tyrosine kinase	0.8839	0.0953	0.5488	0.4962	No	0.892	0.186	0.945	0.289	No	No
100427_at	Ptpro	protein tyrosine phosphatase, receptor type, O	1.0491	0.3159	0.0007	0.3433	No	0.9285	0.101	0.121	0.736	No	No
100428_at	Lamc2	laminin, gamma 2	1.0147	0.8218	0.522	0.4915	No	1.0094	0.819	0.0612	0.736	No	No
100429_at	Ppox	protoporphyrinogen oxidase	1.1025	0.066	0.2243	0.1755	No	1.1187	0.0553	0.804	0.549	No	No
100430_at	Lepr	leptin receptor	0.9909	0.8442	0.724	0.2495	No	1.0962	0.027	0.479	0.0053	No	No
100431_at	Lepr	leptin receptor	0.9128	0.2549	0.0196	0.0342	No	0.8901	0.0637	0.05	0.0058	No	No
100432_f_at	Mdfi	MyoD family inhibitor	0.8351	0.0305	0.0002	0.1581	No	0.9262	0.222	0.0039	0.539	No	No
100433_r_at	Mdfi	MyoD family inhibitor	0.8186	0.0026	0	0.004	No	0.9376	0.366	0.0016	0.144	No	No
100434_s_at	Mdfi	MyoD family inhibitor	0.8296	0.0149	0.0001	0.0261	No	0.93	0.208	0.0014	0.503	No	No
100435_at	Edg2	endothelial differentiation, lysophosphatidic acid G-protein-coupled receptor, 2	1.0633	0.0973	0.0051	0.8002	No	1.07	0.15	0.0085	0.825	No	No
100436_at	Orm1	orosomucoid 1	0.9248	0.1569	0.0119	0.0527	No	0.8917	0.444	0.126	0.452	No	No
100437_g_at	Orm1 /// Orm2	orosomucoid 1 /// orosomucoid 2	0.9561	0.3794	0.0757	0.5939	No	1.0348	0.299	0.0038	0.182	No	No
100438_at	Gpr19	G protein-coupled receptor 19	0.985	0.6228	0.0285	0.8807	No	1.0124	0.67	0.196	0.541	No	No
100439_i_at	Ank1	ankyrin 1, erythroid	0.8914	0.0563	0.0008	0.0312	No	1.0029	0.774	0.0001	0.414	No	No
100440_f_at	Ank1	ankyrin 1, erythroid	0.9959	0.9096	0.0012	0.5681	No	0.9839	0.373	0.0001	0.282	No	No
100441_s_at	Ank1	ankyrin 1, erythroid	1.0234	0.575	0.0114	0.1024	No	1.0601	0.308	0.0996	0.45	No	No
100442_at	Tbrg4	transforming growth factor beta regulated gene 4	1.0431	0.2367	0.6497	0.0515	No	1.0429	0.817	0.859	0.518	No	No
100443_at	Bcat2	branched chain aminotransferase 2, mitochondrial	0.9988	0.9771	0.0013	0.0195	No	1.0675	0.458	0.0052	0.0143	No	No
100444_at	Cdk5	cyclin-dependent kinase 5	0.9882	0.7836	0.0042	0.9522	No	1.0117	0.816	0.0058	0.721	No	No
100445_f_at	Sprr1b	small proline-rich protein 1B	0.9516	0.4222	0.0836	0.5372	No	0.9404	0.283	0.0257	0.106	No	No
100446_r_at	Sprr1b	small proline-rich protein 1B	1.0356	0.3621	0.0793	0.667	No	1.0164	0.557	0.0082	0.76	No	No
100447_at	Hyal2	hyaluronoglucosaminidase 2	0.9932	0.7563	0.0015	0.1867	No	0.9019	0.0244	0.0007	0.0176	No	No
100448_at	Acvrl1	activin A receptor, type II-like 1	1.0121	0.7716	0.4874	0.1833	No	1.043	0.408	0.114	0.595	No	No
100449_g_at	Acvrl1	activin A receptor, type II-like 1	0.9	0.2511	0.0289	0.6682	No	0.9951	0.877	0.183	0.24	No	No
100450_r_at	Acvrl1	activin A receptor, type II-like 1	0.9884	0.7923	0.0449	0.8113	No	0.978	0.274	0.0005	0.109	No	No
100451_at	Hsf1	heat shock factor 1	1.0463	0.3367	0.0065	0.2074	No	1.082	0.0934	0.0141	0.0824	No	No
100452_at	Klf1	Kruppel-like factor 1 (erythroid)	0.9811	0.5136	0.0142	0.6192	No	1.0139	0.711	0.151	0.297	No	No
100453_at	Camk2b	calcium/calmodulin-dependent protein kinase II, beta	1.015	0.6381	0.1332	0.8096	No	1.0365	0.444	0.569	0.455	No	No
100454_at	Ptpru	protein tyrosine phosphatase, receptor type, U	1.0148	0.5903	0.0142	0.0165	No	1.0166	0.619	0.0096	0.0141	No	No
100455_at	Snap91	synaptosomal-associated protein 91	1.1245	0.2771	0.0041	0.4759	No	1.1785	0.154	0.0141	0.458	No	No

100457_at	Glg1	golgi apparatus protein 1	1.1832	0.0043	0.0001	0.0035	No	1.1625	0.0012	0	0.009	No	No
100458_at	Napb	N-ethylmaleimide sensitive fusion protein attachment protein beta	1.0906	0.146	0.0006	0.0594	No	0.9412	0.533	0.0063	0.685	No	No
100459_at	Rad50	RAD50 homolog (S. cerevisiae)	0.9995	0.9914	0.0211	0.8032	No	1.016	0.583	0.0223	0.0284	No	No
100460_at	Sh2bp1	SH2 domain binding protein 1 (tetratricopeptide repeat containing)	0.9449	0.4315	0.3485	0.2467	No	0.8993	0.0107	0.0026	0.0078	No	No
100461_at	Polr2j	polymerase (RNA) II (DNA directed) polypeptide J	0.9422	0.1639	0.0123	0.0168	No	0.9655	0.388	0.0054	0.0141	No	No
100462_at	Arf6	ADP-ribosylation factor 6	0.9831	0.7281	0.2064	0.2794	No	0.9289	0.0968	0.109	0.243	No	No
100463_at	Csng	casein gamma	1.0231	0.328	0.0382	0.0847	No	1.0967	0.29	0.0627	0.108	No	No
100464_at	3110043O21 Rik	RIKEN cDNA 3110043O21 gene	1.0228	0.7024	0.0122	0.5361	No	0.9633	0.417	0.0199	0.247	No	No
100465_i_at	Gm1673	gene model 1673, (NCBI)	1.0602	0.1534	0.0135	0.3531	No	1.042	0.322	0.139	0.993	No	No
100466_f_at	Gm1673	gene model 1673, (NCBI)	1.0613	0.1265	0.2546	0.0756	No	1.0313	0.124	0.177	0.0395	No	No
100467_at	Lyl1	lymphoblastomic leukemia	0.9811	0.6704	0.0182	0.7095	No	0.9852	0.608	0.338	0.426	No	No
100468_g_at	Lyl1	lymphoblastomic leukemia	0.9294	0.068	0.0406	0.016	No	0.9177	0.0425	0.026	0.0104	No	No
100469_at	Nfya	nuclear transcription factor-Y alpha	0.9138	0.1519	0.7211	0.1533	No	0.9275	0.062	0.178	0.0436	No	No
100470_at	Mapk10	mitogen activated protein kinase 10	1.0286	0.5286	0.0202	0.5376	No	1.049	0.208	0.0118	0.227	No	No
100471_at	1110036O03 Rik	RIKEN cDNA 1110036O03 gene	1.056	0.0453	0.0001	0.123	No	1.0101	0.259	0	0.014	No	No
100472_at	Enah	enabled homolog (Drosophila)	1.0044	0.9356	0.257	0.2873	No	0.9958	0.946	0.223	0.857	No	No
100473_at	Arrb1	arrestin, beta 1	0.9598	0.228	0.0029	0.7464	No	0.9359	0.0099	0	0.0073	No	No
100474_at	St8sia2	ST8 alpha-N-acetyl-neuraminide alpha-2,8-sialyltransferase 2	1.0393	0.5569	0.7651	0.6714	No	0.9159	0.0552	0.0017	0.005	No	No
100475_at	Trim25	tripartite motif protein 25	1.016	0.7681	0.0327	0.1472	No	1.0507	0.416	0.0451	0.881	No	No
100476_at	Ibsp	integrin binding sialoprotein	0.8774	0.0069	0.0014	0.0955	Yes	0.858	0.0188	0.0206	0.186	No	Yes
100477_at	Tmem45a	transmembrane protein 45a	1.0389	0.5094	0.0333	0.1831	No	1.0042	0.929	0.0072	0.695	No	No
100479_at	Dnmt3a	DNA methyltransferase 3A	1.0621	0.2112	0.9925	0.1789	No	0.9953	0.963	0.0428	0.659	No	No
100481_at	Col11a1	procollagen, type XI, alpha 1	0.9461	0.4693	0.0582	0.1418	No	0.8928	0.0374	0.165	0.461	No	No
100482_at	Zfp598	zinc finger protein 598	0.9707	0.3438	0.0056	0.1255	No	0.9095	0.0051	0.314	0.014	Yes	No
100483_at	Ifnar1	interferon (alpha and beta) receptor 1	1.0068	0.8549	0.3547	0.0383	No	1.0367	0.398	0.166	0.435	No	No
100484_at	Mmp13	matrix metallopeptidase 13	0.8971	0.1938	0.0752	0.2024	No	0.8654	0.118	0.0856	0.606	No	No
100486_at	Ezh1	enhancer of zeste homolog 1 (Drosophila)	0.932	0.0797	0.002	0.0892	No	0.9803	0.985	0.0098	0.182	No	No
100488_at	Gba	glucosidase, beta, acid	0.9316	0.0871	0.0124	0.7031	No	0.9503	0.12	0.0007	0.125	No	No
100489_at	Pde7a	phosphodiesterase 7A	1.0408	0.4555	0.002	0.5524	No	0.9878	0.952	0.0007	0.347	No	No
100491_at	Slc16a2	solute carrier family 16 (monocarboxylic acid transporters), member 2	0.979	0.7477	0.1347	0.8853	No	0.9387	0.142	0.101	0.0702	No	No
100492_at	Ap2a2	adaptor protein complex AP-2, alpha 2 subunit	0.8748	0.0247	0.7711	0.1539	No	0.98	0.633	0.0998	0.138	No	No
100493_at	Hsd11b2	hydroxysteroid 11-beta dehydrogenase 2	1.0532	0.2186	0.1185	0.3255	No	0.9455	0.5	0.957	0.946	No	No
100494_at	Fgf1	fibroblast growth factor 1	0.9111	0.0719	0.0162	0.1748	No	0.9556	0.172	0.0002	0.0398	No	No
100495_at	Dvl2	dishevelled 2, dsh homolog (Drosophila)	0.9555	0.1349	0.0057	0.1572	No	0.9472	0.319	0.16	0.273	No	No
100496_at	Pam	peptidylglycine alpha-amidating monooxygenase	0.8865	0.1369	0.7306	0.5323	No	0.837	0.0086	0.0009	0.785	Yes	No
100497_at	Stx3	syntaxin 3	1.0171	0.8272	0.0278	0.1924	No	0.9517	0.473	0.0003	0.742	No	No
100498_g_at	Stx3	syntaxin 3	0.9626	0.572	0.1462	0.217	No	1.0398	0.183	0.0028	0.728	No	No
100499_at	Stx3	syntaxin 3	0.9832	0.6674	0.0045	0.8225	No	1.1407	0.0441	0.0004	0.3	No	No
100500_at	Fadd	Fas (TNFRSF6)-associated via death domain	1.0094	0.8726	0.4159	0.7359	No	0.9969	0.997	0.193	0.415	No	No
100501_at	Atp4b	ATPase, H+/K+ exchanging, beta polypeptide	0.9303	0.4753	0.1757	0.7831	No	0.9228	0.278	0.816	0.779	No	No
100502_at	Pde1c	phosphodiesterase 1C	1.0173	0.6916	0.073	0.4521	No	1.0095	0.885	0.377	0.329	No	No
100505_at	Rit2	Ras-like without CAAX 2	0.9583	0.1009	0.0012	0.057	No	0.8957	0.105	0.002	0.804	No	No
100507_at	Nov	nephroblastoma overexpressed gene	0.9416	0.2108	0.8598	0.1189	No	0.9778	0.595	0.0348	0.0112	No	No
100508_at	Mfng	manic fringe homolog (Drosophila)	1.0168	0.8287	0.8694	0.4285	No	1.024	0.652	0.786	0.24	No	No
100509_at	Rnf19	ring finger protein (C3HC4 type) 19	0.9909	0.8486	0.0181	0.3599	No	0.9688	0.49	0.283	0.927	No	No
100510_at	Sncb	synuclein, beta	1.0401	0.4339	0.8264	0.8987	No	1.0326	0.488	0.624	0.476	No	No
100511_at	Hmha1	histocompatibility (minor) HA-1	0.9261	0.232	0.1256	0.3298	No	0.995	0.94	0.898	0.446	No	No
100512_at	Uchl5	ubiquitin carboxyl-terminal esterase L5	1.063	0.1404	0.0416	0.9969	No	1.135	0.168	0.147	0.39	No	No
100513_at	Ddef1	development and differentiation enhancing	1.0003	0.994	0.1885	0.6688	No	0.9819	0.624	0.0841	0.616	No	No
100514_at	Gna13	guanine nucleotide binding protein, alpha 13	1.1826	0.0046	0	0.0012	No	1.1012	0.0011	0	0.0013	No	No
100515_at	Furin	furin (paired basic amino acid cleaving enzyme)	0.9458	0.0503	0.0008	0.1384	No	0.8937	0.106	0.0298	0.608	No	No
100516_at	Chka	choline kinase alpha	0.9227	0.0656	0.3725	0.6604	No	0.8959	0.0058	0.764	0.0907	Yes	No
100518_at	Arf2	ADP-ribosylation factor 2	1.1262	0.2915	0.3841	0.1598	No	1.0227	0.0955	0.0006	0.0006	No	No
100521_at	Ovgp1	oviductal glycoprotein 1	0.955	0.7056	0.8689	0.8814	No	1.0695	0.399	0.0483	0.0322	No	No
100522_s_at	Wbp5	WW domain binding protein 5	1.0807	0.1758	0.0376	0.1123	No	1.0997	0.295	0.954	0.711	No	No
100523_r_at	Wbp5	WW domain binding protein 5	0.976	0.6828	0.002	0.5857	No	1.0941	0.28	0.0044	0.525	No	No
100525_at	Htf9c	HpaII tiny fragments locus 9c	0.9735	0.0035	0	0.0012	No	1.0237	0.217	0.0026	0.578	No	No
100526_f_at	Adam3	a disintegrin and metallopeptidase domain 3 (cyritestin)	0.8959	0.5731	0.4784	0.5768	No	1.1575	0.497	0.165	0.531	No	No
100527_at	Ccdc56	coiled-coil domain containing 56	1.0013	0.9642	0.0012	0.0196	No	0.9452	0.356	0.01	0.0198	No	No
100528_at	Ube2h	ubiquitin-conjugating enzyme E2H	0.921	0.0431	0.1932	0.8595	No	1.0231	0.0658	0.0254	0.0004	No	No
100529_at	Ube2h	ubiquitin-conjugating enzyme E2H	0.9357	0.0189	0.0043	0.1918	No	0.963	0.289	0.0133	0.842	No	No
100530_at	Ralgds	ral guanine nucleotide dissociation stimulator	0.9839	0.4324	0.0002	0.1847	No	0.9855	0.153	0	0.0287	No	No
100533_s_at	Crem	cAMP responsive element modulator	0.9883	0.8019	0.0423	0.7707	No	1.0137	0.674	0.0078	0.0023	No	No
100534_at	Tsnax	translin-associated factor X	1.0267	0.6163	0.2225	0.6847	No	1.0909	0.0785	0.0274	0.362	No	No
100535_at	E130105L11 Rik	RIKEN cDNA E130105L11 gene	1.1643	0.1397	0.0039	0.5354	No	1.0551	0.36	0.0229	0.159	No	No
100536_at	Mobp	myelin-associated oligodendrocytic basic protein	0.8835	0.448	0.1536	0.3827	No	1.0647	0.334	0.0672	0.723	No	No
100537_at	2310016M24 Rik	RIKEN cDNA 2310016M24 gene	0.9976	0.9759	0.2167	0.7387	No	0.9776	0.719	0.888	0.85	No	No
100538_at	Sod1	superoxide dismutase 1, soluble	0.7327	0.0097	0.0202	0.0862	Yes	0.7537	0.0077	0.0032	0.0495	Yes	Yes
100539_at	Acot7	acyl-CoA thioesterase 7	1.0583	0.152	0.1886	0.4228	No	1.0664	0.0356	0.2	0.165	No	No
100540_at	Lta4h	leukotriene A4 hydrolase	1.0397	0.45	0.0067	0.454	No	0.9883	0.832	0.0006	0.0225	No	No
100542_at	Mep1a	meprin 1 alpha	1.1693	0.0515	0.1062	0.0886	No	1.1478	0.252	0.179	0.295	No	No
100543_s_at	Brd7 /// LOC634327	bromodomain containing 7 /// similar to bromodomain containing 7	1.0139	0.7447	0.0184	0.8561	No	1.0381	0.206	0.0046	0.0499	No	No
100544_at	Brd7 /// LOC634327	bromodomain containing 7 /// similar to bromodomain containing 7	0.9163	0.1141	0.1953	0.4165	No	0.9747	0.759	0.894	0.572	No	No
100546_at	Rbm4	RNA binding motif protein 4	1.0245	0.6755	0.0245	0.6351	No	1.0039	0.675	0.0014	0.0095	No	No
100547_at	4921506I22 Rik	RIKEN cDNA 4921506I22 gene	1.0105	0.7548	0.0122	0.4348	No	0.9995	0.967	0.025	0.956	No	No
100548_at	Pea15	phosphoprotein enriched in astrocytes 15	1.0256	0.4784	0.5691	0.2134	No	1.0076	0.795	0.409	0.645	No	No
100549_at	Hba-x	hemoglobin X, alpha-like embryonic chain in Hba complex	1.0601	0.5335	0.6341	0.3515	No	1.0068	0.967	0.0083	0.258	No	No
100550_f_at	Cox6c	cytochrome c oxidase, subunit VIc	1.1895	0.0332	0.5496	0.3888	No	1.052	0.0327	0.0016	0.964	No	No
100551_r_at	Cox6c	Cytochrome c oxidase, subunit VIc	0.9569	0.4603	0.0033	0.6579	No	1.0523	0.683	0.0139	0.34	No	No
100552_at	Ifngr1	interferon gamma receptor 1	0.8265	0.0006	0.172	0.0177	Yes	0.8227	0.0045	0.275	0.134	Yes	Yes
100553_at	Trim27	tripartite motif protein 27	1.0032	0.9233	0.006	0.3028	No	0.9868	0.835	0.344	0.571	No	No
100554_at	Pdlim1	PDZ and LIM domain 1 (elfin)	0.9367	0.1653	0.0306	0.1029	No	0.9647	0.12	0.0004	0.285	No	No
100555_at	Dscr1	Down syndrome critical region homolog 1 (human)	1.0698	0.2107	0.3809	0.8472	No	1.088	0.195	0.123	0.74	No	No
100556_at	Eif4b	Eukaryotic translation initiation factor 4B	1.0056	0.9435	0.1174	0.6706	No	1.1958	0.0259	0.404	0.136	No	No
100557_g_at	Eif4b	eukaryotic translation initiation factor 4B	0.8984	0.0062	0.0201	0.367	Yes	0.8791	0.0066	0.0137	0.662	Yes	Yes
100558_at	Eif4b	eukaryotic translation initiation factor 4B	0.9889	0.797	0.1629	0.9541	No	0.9981	0.964	0.502	0.859	No	No
100559_at	Dhx16	DEAH (Asp-Glu-Ala-His) box polypeptide 16	1.0483	0.1634	0.2502	0.0103	No	1.032	0.416	0.229	0.416	No	No
100560_at	Pafah1b1	platelet-activating factor acetylhydrolase, isoform 1b, beta1 subunit	0.9245	0.2262	0.0083	0.2428	No	0.9847	0.858	0.0035	0.23	No	No
100561_at	Iqgap1	IQ motif containing GTPase activating protein 1	0.9614	0.5119	0.0012	0.5037	No	0.9372	0.282	0.0196	0.46	No	No
100562_at	2400006H24 Rik	RIKEN cDNA 2400006H24 gene	1.0284	0.2576	0.0028	0.9867	No	1.0134	0.666	0.0179	0.915	No	No
100564_at	Ddt	D-dopachrome tautomerase	0.9665	0.5118	0.6995	0.1276	No	0.9592	0.395	0.121	0.214	No	No

100565_at	Gnpda1 /// LOC231914 /// LOC667410	glucosamine-6-phosphate deaminase 1 /// similar to Glucosamine-6-phosphate isomerase (Glucosamine-6-phosphate deaminase) (GNPDA) (GlcN6P deaminase) (Oscillin) /// similar to Glucosamine-6-phosphate isomerase (Glucosamine-6-phosphate deaminase) (GNPDA) (GlcN6P deaminase) (Oscillin)	0.9712	0.539	0.8435	0.548	No	0.9567	0.437	0.164	0.582	No	No
100566_at	Igfbp5	insulin-like growth factor binding protein 5	1.0132	0.7392	0.2023	0.1258	No	1.0311	0.37	0.0556	0.145	No	No
100567_at	Fabp4	fatty acid binding protein 4, adipocyte	1.2049	0.0435	0.2814	0.265	No	1.144	0.0719	0.149	0.0565	No	No
100568_at	Abce1	ATP-binding cassette, sub-family E (OABP), member 1	1.0185	0.5204	0.613	0.0104	No	0.9453	0.277	0.168	0.318	No	No
100569_at	Anxa2	annexin A2	0.9968	0.9286	0.0005	0.7688	No	0.96	0.229	0.0002	0.185	No	No
100570_at	6330412F12 Rik	RIKEN cDNA 6330412F12 gene	0.9596	0.1998	0.1074	0.9798	No	0.9607	0.266	0.231	0.634	No	No
100571_at	Laptm4b	lysosomal-associated protein transmembrane 4B	0.9451	0.1408	0.0085	0.4817	No	0.9828	0.848	0.0857	0.743	No	No
100572_at	Tm9sf2	transmembrane 9 superfamily member 2	1.1613	0.2215	0.8961	0.3001	No	0.9698	0.392	0.0004	0.977	No	No
100573_f_at	Gpi1 /// LOC676596	glucose phosphate isomerase 1 /// similar to Glucose-6-phosphate isomerase (GPI) (Phosphoglucose isomerase) (PGI) (Phosphohexose isomerase) (PHI) (Neuroleukin) (NLK)	0.8892	0.2146	0.4886	0.1026	No	0.9021	0.299	0.682	0.354	No	No
100574_f_at	Gpi1	glucose phosphate isomerase 1	1.0269	0.5567	0.0473	0.0243	No	1.0002	1	0.435	0.262	No	No
100575_at	Ard1	N-acetyltransferase ARD1 homolog (S. cerevisiae)	1.0623	0.1565	0.0017	0.8004	No	0.9955	0.923	0.0005	0.218	No	No
100576_at	Pafah1b3	platelet-activating factor acetylhydrolase, isoform 1b, alpha1 subunit	1.0297	0.4215	0.0028	0.2251	No	1.0198	0.346	0.0008	0.0371	No	No
100577_at	Snrpd1	small nuclear ribonucleoprotein D1	1.129	0.2148	0.8645	0.8908	No	1.1204	0.447	0.51	0.954	No	No
100578_at	Impdh2	inosine 5'-phosphate dehydrogenase 2	1.0056	0.8054	0.0014	0.0904	No	1.008	0.77	0.0029	0.478	No	No
100579_s_at	Clta	clathrin, light polypeptide (Lca)	1.0259	0.5384	0.5099	0.459	No	1.0244	0.458	0.0066	0.2	No	No
100580_at	Clta	Clathrin, light polypeptide (Lca)	0.9678	0.4559	0.0086	0.4668	No	0.9847	0.84	0.657	0.495	No	No
100581_at	Cstb	cystatin B	1.0177	0.7502	0.0511	0.8636	No	1.0439	0.382	0.0629	0.579	No	No
100582_at	Snx17	sorting nexin 17	1.0122	0.7157	0.0025	0.2705	No	1.0056	0.898	0.0308	0.964	No	No
100583_at	Igh-VJ558 /// LOC238447 /// LOC544903 /// LOC544907	immunoglobulin heavy chain (J558 family) /// similar to immunoglobulin heavy chain variable region /// similar to immunoglobulin mu-chain /// similar to anti-poly(dC) monoclonal antibody heavy chain	1.0032	0.961	0.1362	0.138	No	0.976	0.71	0.375	0.218	No	No
100584_at	Anxa4	annexin A4	0.97	0.4962	0.0832	0.7168	No	0.9566	0.0724	0.0015	0.889	No	No
100585_at	Supt4h1 /// Supt4h2	suppressor of Ty 4 homolog 1 (S. cerevisiae) /// suppressor of Ty 4 homolog 2 (S. cerevisiae)	1.0771	0.0329	0.0022	0.0594	No	1.0727	0.256	0.117	0.1	No	No
100586_i_at	5730403B10 Rik	RIKEN cDNA 5730403B10 gene	0.9087	0.0148	0.0186	0.0157	No	0.9614	0.117	0.0258	0.0564	No	No
100587_f_at	5730403B10 Rik	RIKEN cDNA 5730403B10 gene	0.9564	0.5657	0.8416	0.051	No	0.9227	0.395	0.537	0.0721	No	No
100588_at	Psme2	proteasome (prosome, macropain) 28 subunit, beta	1.0392	0.2971	0.0048	0.3007	No	1.0953	0.0962	0.0022	0.948	No	No
100589_at	Immt	Inner membrane protein, mitochondrial	1.0016	0.9794	0.8485	0.3946	No	1.0582	0.396	0.351	0.623	No	No
100592_at	Ghitm	growth hormone inducible transmembrane protein	0.94	0.2568	0.0539	0.57	No	0.9857	0.661	0.0603	0.96	No	No
100593_at	Tnnt2	troponin T2, cardiac	0.8051	0.01	0.0233	0.0399	No	0.8343	0.0003	0.0115	0.0031	No	No
100594_at	Pigq	phosphatidylinositol glycan, class Q	0.9746	0.5353	0.5915	0.202	No	0.9809	0.64	0.988	0.112	No	No
100595_at	Ptp4a2	protein tyrosine phosphatase 4a2	1.0327	0.6192	0.1851	0.5993	No	1.0057	0.906	0.0329	0.648	No	No
100596_at	Selenbp1	selenium binding protein 1	0.9007	0.0164	0.0011	0.4778	No	0.9147	0.0149	0.0078	0.0091	No	No
100597_at	Gyg	glycogenin	0.9933	0.9042	0.0103	0.1137	No	0.8794	0.293	0.101	0.266	No	No
100599_at	Atf4	activating transcription factor 4	1.0731	0.0007	0	0.0006	No	1.07	0.0276	0.0001	0.0316	No	No
100600_at	Cd24a	CD24a antigen	1.0694	0.1502	0.0549	0.3948	No	1.0377	0.44	0.0177	0.381	No	No
100601_at	Itgb5	integrin beta 5	0.9255	0.0062	0.0001	0.0025	No	1.0074	0.748	0.0097	0.827	No	No
100602_at	Rps15	ribosomal protein S15	1.1521	0.0314	0.8421	0.2616	No	1.0288	0.563	0.0172	0.69	No	No
100603_at	Dync1i2	dynein cytoplasmic 1 intermediate chain 2	1.0019	0.9821	0.0193	0.9044	No	1.0293	0.672	0.0472	0.768	No	No
100605_at	Tpm2	tropomyosin 2, beta	0.8417	0.0137	0.0451	0.0477	No	0.8327	0.018	0.055	0.0116	No	No
100606_at	Prnp	prion protein	0.9815	0.5965	0.6913	0.387	No	1.0234	0.448	0.606	0.182	No	No
100607_at	Pld3	phospholipase D family, member 3	0.9774	0.5936	0.0041	0.0406	No	0.9939	0.959	0.0149	0.0096	No	No
100608_at	Sptlc1	serine palmitoyltransferase, long chain base subunit 1	0.9657	0.3066	0.0592	0.067	No	0.9444	0.148	0.0207	0.17	No	No
100609_at	Cfb	complement factor B	0.9521	0.3917	0.254	0.1394	No	1.0274	0.58	0.297	0.327	No	No
100610_at	Capns1	calpain, small subunit 1	1.0249	0.5026	0.0014	0.4876	No	1.0159	0.422	0.0001	0.644	No	No
100611_at	Lyzs	lysozyme	1.0377	0.769	0.3812	0.1032	No	0.992	0.984	0.386	0.0277	No	No
100612_at	Rrm1	ribonucleotide reductase M1	0.971	0.3583	0.4334	0.4999	No	1.0339	0.216	0.137	0.119	No	No
100613_at	Fkbp8	FK506 binding protein 8	0.9865	0.6382	0.0012	0.6991	No	0.9592	0.165	0.0012	0.462	No	No
100614_at	Mb	myoglobin	0.8944	0.194	0.0515	0.0465	No	0.958	0.542	0.0004	0.0688	No	No
100615_at	---	---	1.111	0.3363	0.4749	0.5788	No	0.988	0.911	0.559	0.62	No	No
100616_at	Cenpa	centromere autoantigen A	1.0935	0.2399	0.3302	0.0436	No	0.9966	0.893	0.279	0.633	No	No
100617_at	Slc25a5 /// LOC433326 /// LOC433923 /// LOC630624 /// LOC669381 /// LOC670371	solute carrier family 25 (mitochondrial carrier, adenine nucleotide translocator), member 5 /// similar to ADP/ATP translocase 2 (Adenine nucleotide translocator 2) (ANT 2) (ADP,ATP carrier protein 2) (Solute carrier family 25 member 5) /// similar to ADP/ATP translocase 2 (Adenine nucleotide translocator 2) (ANT 2) (ADP,ATP carrier protein 2) (Solute carrier family 25 member 5) /// similar to ADP/ATP translocase 2 (Adenine nucleotide translocator 2) (ANT 2) (ADP,ATP carrier protein 2) (Solute carrier family 25 member 5) /// similar to ADP/ATP translocase 2 (Adenine nucleotide translocator 2) (ANT 2) (ADP,ATP carrier protein 2) (Solute carrier family 25 member 5) /// similar to solute carrier family 25, member 5	1.038	0.5566	0.8458	0.3146	No	0.8619	0.238	0.0018	0.138	No	No
100618_f_at	Slc25a5 /// LOC433326 /// LOC433923 /// LOC630624 /// LOC669381 /// LOC670371 /// LOC673916	solute carrier family 25 (mitochondrial carrier, adenine nucleotide translocator), member 5 /// similar to ADP/ATP translocase 2 (Adenine nucleotide translocator 2) (ANT 2) (ADP,ATP carrier protein 2) (Solute carrier family 25 member 5) /// similar to ADP/ATP translocase 2 (Adenine nucleotide translocator 2) (ANT 2) (ADP,ATP carrier protein 2) (Solute carrier family 25 member 5) /// similar to ADP/ATP translocase 2 (Adenine nucleotide translocator 2) (ANT 2) (ADP,ATP carrier protein 2) (Solute carrier family 25 member 5) /// similar to ADP/ATP translocase 2 (Adenine nucleotide translocator 2) (ANT 2) (ADP,ATP carrier protein 2) (Solute carrier family 25 member 5) /// similar to solute carrier family 25, member 5 /// similar to solute carrier family 25, member 5	1.0399	0.2229	0.2469	0.1392	No	1.0149	0.47	0.0023	0.0486	No	No
100619_r_at	Slc25a5 /// LOC433326 /// LOC433923 /// LOC630624 /// LOC669381 /// LOC670371 /// LOC673916	solute carrier family 25 (mitochondrial carrier, adenine nucleotide translocator), member 5 /// similar to ADP/ATP translocase 2 (Adenine nucleotide translocator 2) (ANT 2) (ADP,ATP carrier protein 2) (Solute carrier family 25 member 5) /// similar to ADP/ATP translocase 2 (Adenine nucleotide translocator 2) (ANT 2) (ADP,ATP carrier protein 2) (Solute carrier family 25 member 5) /// similar to ADP/ATP translocase 2 (Adenine nucleotide translocator 2) (ANT 2) (ADP,ATP carrier protein 2) (Solute carrier family 25 member 5) /// similar to ADP/ATP translocase 2 (Adenine nucleotide translocator 2) (ANT 2) (ADP,ATP carrier protein 2) (Solute carrier family 25 member 5) /// similar to solute carrier family 25, member 5 /// similar to solute carrier family 25, member 5	1.0688	0.076	0.1081	0.0292	No	0.9773	0.76	0.0251	0.159	No	No
100620_at	Hpd	4-hydroxyphenylpyruvic acid dioxygenase	1.0384	0.544	0.8094	0.2833	No	0.9215	0.145	0.0066	0.191	No	No

100621_at	Rnh1	ribonuclease/angiogenin inhibitor 1	0.9537	0.0552	0.0006	0.0058	No	0.9727	0.366	0.924	0.138	No	No
100622_at	Prdx6	peroxiredoxin 6	1.0099	0.782	0.0031	0.0173	No	1.0026	0.74	0.0044	0.0269	No	No
100623_at	Clca3	chloride channel calcium activated 3	1.0117	0.6942	0.0038	0.3096	No	0.9912	0.879	0.792	0.169	No	No
100626_at	Odf2	outer dense fiber of sperm tails 2	1.0265	0.5514	0.079	0.9855	No	1.1601	0.0049	0.004	0.319	Yes	No
100628_at	Ndufc1	NADH dehydrogenase (ubiquinone) 1, subcomplex unknown, 1	1.0105	0.8389	0.0273	0.4562	No	0.9919	0.924	0.024	0.599	No	No
100629_at	Gstm5	glutathione S-transferase, mu 5	1.0605	0.3137	0.066	0.6815	No	0.9677	0.47	0.0054	0.012	No	No
100630_f_at	Gstm5	glutathione S-transferase, mu 5	1.0112	0.4697	0.006	0.0027	No	1.0142	0.298	0.0016	0.0052	No	No
100631_r_at	Gstm5	Glutathione S-transferase, mu 5	1.0278	0.5596	0.9084	0.0918	No	0.9859	0.799	0.813	0.541	No	No
100632_at	Prkag1	protein kinase, AMP-activated, gamma 1 non-catalytic subunit	0.9944	0.6769	0.0001	0.038	No	1.0168	0.401	0.0002	0.235	No	No
100633_at	Mosc2	MOCO sulphurase C-terminal domain containing 2	1.0056	0.8236	0.045	0.0476	No	1.0162	0.577	0.135	0.2	No	No
100634_at	Rdh7	retinol dehydrogenase 7	0.9516	0.403	0.1846	0.6543	No	0.9691	0.492	0.256	0.0644	No	No
100635_at	Sar1a	SAR1 gene homolog A (S. cerevisiae)	1.0001	0.9964	0.019	0.0096	No	1.0319	0.12	0.0006	0.028	No	No
100636_at	Eif4ebp1	eukaryotic translation initiation factor 4E binding protein 1	0.9945	0.9255	0.0179	0.0757	No	0.8938	0.0193	0.0002	0.0119	No	No
100669_at	---	---	1.0162	0.6286	0.9999	0.9975	No	0.8331	0.654	0.195	0.429	No	No
100670_at	LOC671835	similar to sodium channel 25	0.9121	0.1301	0.0747	0.142	No	0.9805	0.859	0.262	0.85	No	No
100671_at	Ifna11	interferon alpha family, gene 11	1.0978	0.1393	0.8184	0.2781	No	1.0099	0.929	0.639	0.859	No	No
100672_at	Myo5a	myosin Va	1.0113	0.719	0.9301	0.5561	No	0.9687	0.737	0.293	0.865	No	No
100673_f_at	Klra5	killer cell lectin-like receptor, subfamily A, member 5	1.0335	0.537	0.521	0.0423	No	1.0552	0.279	0.0001	0.336	No	No
100674_f_at	Klra6	killer cell lectin-like receptor, subfamily A, member 6	1.0977	0.3196	0.1723	0.9507	No	1.1614	0.0223	0.0146	0.857	No	No
100675_at	Gabrr1	gamma-aminobutyric acid (GABA-C) receptor, subunit rho 1	0.9688	0.5104	0.0736	0.4922	No	0.9901	0.796	0.18	0.443	No	No
100676_at	Synj2	synaptojanin 2	1.0396	0.45	0.1618	0.0662	No	1.0836	0.478	0.219	0.213	No	No
100677_f_at	V2r1 /// V2r12 /// V2r2 /// V2r8 /// V2r9	vomeronasal 2, receptor, 1 /// vomeronasal 2, receptor, 12 /// vomeronasal 2, receptor, 2 /// vomeronasal 2, receptor, 8 /// vomeronasal 2, receptor, 9	0.9632	0.5814	0.8938	0.7174	No	1.0081	0.878	0.492	0.996	No	No
100678_s_at	V2r13 /// LOC632660	vomeronasal 2, receptor, 13 /// similar to vomeronasal 2, receptor, 8	0.9629	0.6368	0.1656	0.57	No	1.1006	0.431	0.0506	0.848	No	No
100679_at	V2r16	vomeronasal 2, receptor, 16	1.0426	0.1091	0.0018	0.1055	No	0.9321	0.0587	0.992	0.0026	No	No
100680_at	Dub1	deubiquitinating enzyme 1	0.9671	0.3398	0.1921	0.1414	No	0.9847	0.92	0.257	0.646	No	No
100681_f_at	LOC668642	similar to Glandular kallikrein K3 precursor (Tissue kallikrein-3) (mGK-3) (7S nerve growth factor gamma chain) (Gamma-NGF)	0.9529	0.5373	0.289	0.4834	No	0.9436	0.422	0.103	0.63	No	No
100682_f_at	Igh-VJ558 /// Igh-1a /// LOC625794	Immunoglobulin heavy chain (J558 family) /// Immunoglobulin heavy chain 1a (serum IgG2a) /// Hypothetical protein LOC625794	1.0153	0.717	0.9166	0.2244	No	1.0576	0.256	0.414	0.245	No	No
100683_r_at	LOC625794	Hypothetical protein LOC625794	1.07	0.2159	0.0951	0.2067	No	1.0839	0.102	0.618	0.682	No	No
100684_at	Prkcsh	protein kinase C substrate 80K-H	0.9729	0.401	0.0007	0.0648	No	0.9458	0.224	0.0578	0.393	No	No
100685_at	Krt2-1	keratin complex 2, basic, gene 1	1.0037	0.9002	0.1341	0.9195	No	0.9881	0.735	0.721	0.46	No	No
100686_at	Rps2 /// LOC544872 /// LOC545808 /// LOC546164 /// LOC546298 /// LOC620248 /// LOC620559 /// LOC623242 /// LOC625055 /// LOC628962 /// LOC629408 /// LOC634678 /// LOC635931 /// LOC637606 ///	ribosomal protein S2 /// similar to 40S ribosomal protein S2 /// similar to 40S ribosomal protein S2 /// similar to 40S ribosomal protein S2 /// similar to 40S ribosomal protein S2 /// similar to 40S ribosomal protein S2 /// similar to 40S ribosomal protein S2 /// similar to 40S ribosomal protein S2 /// hypothetical LOC634678 /// similar to 40S ribosomal protein S2 /// similar to 40S ribosomal protein S2 /// similar to 40S ribosomal protein S2 /// similar to 40S ribosomal protein S2 /// similar to 40S ribosomal protein S2 /// similar to 40S ribosomal protein S2 /// similar to 40S ribosomal protein S2 /// similar to ribosomal protein S2 /// hypothetical protein LOC669768 /// hypothetical protein LOC670226 /// similar to 40S ribosomal protein S2 /// similar to 40S ribosomal protein S2 /// similar to ribosomal protein S2	1.06	0.0047	0.8438	0.1763	Yes	1.0149	0.3	0.0821	0.302	No	No
100687_f_at	V2r14	vomeronasal 2, receptor, 14	1.0457	0.5713	0.7652	0.0904	No	1.0818	0.494	0.869	0.0407	No	No
100688_at	Crybb2	crystallin, beta B2	0.9532	0.217	0.0019	0.0173	No	0.9157	0.41	0.594	0.347	No	No
100689_at	Svp2	seminal vesicle protein 2	1.0979	0.4167	0.3482	0.9649	No	1.0865	0.586	0.868	0.585	No	No
100690_at	Th	tyrosine hydroxylase	0.9965	0.9321	0.6093	0.6965	No	1.025	0.658	0.292	0.518	No	No
100691_at	Scn3a	sodium channel, voltage-gated, type III, alpha	0.9779	0.7291	0.1824	0.4939	No	1.1001	0.449	0.32	0.0707	No	No
100692_at	---	---	1.0532	0.0376	0.0009	0.1187	No	1.0739	0.0298	0.0475	0.11	No	No
100693_at	Gabrg3	gamma-aminobutyric acid (GABA-A) receptor, subunit gamma 3	1.056	0.4238	0.6254	0.0604	No	1.1776	0.206	0.523	0.543	No	No
100694_at	Rplp1	ribosomal protein, large, P1	1.011	0.7577	0.0032	0.2581	No	1.0079	0.816	0.0025	0.625	No	No
100695_at	Edn2	endothelin 2	0.9563	0.5449	0.18	0.6283	No	0.9631	0.234	0.005	0.58	No	No
100696_at	Pde6a	phosphodiesterase 6A, cGMP-specific, rod, alpha	1.0764	0.2969	0.1732	0.1021	No	0.9811	0.751	0.0167	0.96	No	No
100697_at	Pax3	paired box gene 3	1.0338	0.4326	0.199	0.2317	No	1.1092	0.0501	0.223	0.338	No	No
100698_at	Prop1	paired like homeodomain factor 1	0.9119	0.2291	0.1867	0.5536	No	1.0951	0.339	0.516	0.594	No	No
100699_at	Nfatc2ip	nuclear factor of activated T-cells, cytoplasmic, calcineurin-dependent 2 interacting protein	0.9359	0.2356	0.9444	0.4123	No	1.029	0.331	0.0029	0.0393	No	No
100700_s_at	Nr5a1	nuclear receptor subfamily 5, group A, member 1	0.9691	0.636	0.564	0.7797	No	1.0402	0.328	0.0073	0.443	No	No
100701_r_at	Nr5a1	nuclear receptor subfamily 5, group A, member 1	1.0789	0.1888	0.0557	0.0517	No	0.9395	0.318	0.228	0.204	No	No
100702_at	Shbg	sex hormone binding globulin	0.9992	0.9783	0.8454	0.1135	No	1.0063	0.924	0.353	0.719	No	No
100703_at	Npy2r	neuropeptide Y receptor Y2	0.9677	0.279	0.1585	0.0336	No	0.9597	0.202	0.985	0.18	No	No
100704_at	Cmtm4	CKLF-like MARVEL transmembrane domain containing 4	0.9638	0.5285	0.0087	0.631	No	1.0184	0.645	0.0015	0.974	No	No
100705_at	Lhcgr	luteinizing hormone/choriogonadotropin receptor	0.9658	0.6272	0.8089	0.6374	No	1.0644	0.106	0.517	0.0164	No	No
100706_f_at	Sfmbt2	Scm-like with four mbt domains 2	0.9609	0.3988	0.0017	0.4138	No	1.06	0.286	0.0041	0.0793	No	No
100707_at	Sh3rf1	SH3 domain containing ring finger 1	0.928	0.1681	0.6975	0.8964	No	0.9113	0.291	0.0971	0.496	No	No
100708_at	H3f3b	H3 histone, family 3B	1.0783	0.2819	0.0317	0.6466	No	1.0307	0.619	0.0533	0.8	No	No
100709_at	Tpbpa	trophoblast specific protein alpha	0.9828	0.7116	0.4839	0.4137	No	0.9655	0.0725	0.0697	0.0537	No	No
100710_at	LOC675857	similar to Transitional endoplasmic reticulum ATPase (TER ATPase) (15S Mg(2+)-ATPase p97 subunit) (Valosin-containing protein) (VCP)	1.0313	0.1871	0.6315	0.0124	No	0.9938	0.898	0.898	0.048	No	No
100711_at	Rpl10a	ribosomal protein L10A	0.9936	0.8791	0.0438	0.7519	No	0.9579	0.0609	0.0007	0.0019	No	No
100712_at	Npas1	neuronal PAS domain protein 1	0.9918	0.912	0.0368	0.6679	No	1.0588	0.251	0.0215	0.89	No	No
100713_at	Zfp617	zinc finger protein 617	1.0016	0.9726	0.9775	0.3446	No	0.8908	0.0645	0.968	0.0294	No	No
100714_at	Bapx1	bagpipe homeobox gene 1 homolog (Drosophila)	1.0567	0.3152	0.1056	0.3105	No	0.9962	0.963	0.177	0.647	No	No
100715_at	Msmb	beta-microseminoprotein	1.0389	0.103	0.0034	0.0332	No	0.9935	0.858	0.576	0.219	No	No
100716_at	Has1	hyaluronan synthase1	1.0102	0.8186	0.0054	0.7175	No	0.9902	0.941	0.0386	0.375	No	No
100717_at	U90926	cDNA sequence U90926	1.0343	0.4909	0.1262	0.1767	No	1.0984	0.171	0.721	0.065	No	No
100718_at	Ptma	prothymosin alpha	0.9821	0.84	0.2137	0.123	No	0.981	0.757	0.341	0.362	No	No
100719_f_at	Klk1b16	kallikrein 1-related peptidase b16	0.9879	0.7109	0.6405	0.4705	No	0.8763	0.207	0.86	0.689	No	No
100720_at	Pabpc1	poly A binding protein, cytoplasmic 1	0.9252	0.0093	0.0228	0.3868	Yes	0.9234	0.0066	0.0025	0.0063	No	No

100721_f_at	Igh-VJ558 /// V165-D-J-C mu /// LOC382693 /// LOC382696 /// VH186.2-D-J-C mu /// LOC619833 /// LOC629941 /// LOC635899 /// LOC636044 /// LOC637794 /// LOC668549 /// LOC673286 /// LOC674018	immunoglobulin heavy chain (J558 family) /// IgM variable region /// similar to immunoglobulin heavy chain /// similar to Ig heavy chain V region 3 precursor /// similar to monoclonal antibody heavy chain /// similar to Ig heavy chain V region 102 precursor /// similar to Ig heavy chain V region 3 precursor /// similar to Ig heavy chain V region VH558 A1/A4 precursor /// similar to Ig heavy chain V region 23 precursor /// similar to Ig heavy chain V region 3 precursor /// similar to Ig heavy chain V region 145 precursor /// similar to Ig heavy chain V region 102 precursor /// similar to Ig heavy chain V region 3 precursor	0.945	0.3276	0.4699	0.6909	No	0.9514	0.331	0.129	0.482	No	No
100722_r_at	---	---	1.0484	0.5279	0.0794	0.0584	No	1.0182	0.314	0.0024	0.0106	No	No
100723_f_at	Sprr2e	small proline-rich protein 2E	1.0181	0.5456	0.8284	0.5786	No	1.0034	0.802	0.365	0.971	No	No
100724_at	Zfp28	zinc finger protein 28	1.1199	0.1115	0.2181	0.9745	No	1.2028	0.0038	0.0023	0.251	Yes	No
100725_at	Nqo1	NAD(P)H dehydrogenase, quinone 1	0.9753	0.4047	0.736	0.1417	No	1.1284	0.107	0.221	0.154	No	No
100726_at	Grin2a	glutamate receptor, ionotropic, NMDA2A (epsilon 1)	1.0354	0.3725	0.0072	0.8984	No	0.987	0.653	0.0065	0.192	No	No
100727_at	Rpl28	ribosomal protein L28	0.9764	0.2734	0.0002	0.0265	No	0.9512	0.0734	0.0002	0.0098	No	No
100728_at	Mpdz	multiple PDZ domain protein	0.9738	0.6404	0.0323	0.1838	No	1.0427	0.387	0.145	0.478	No	No
100729_at	Rpl26	ribosomal protein L26	1.0304	0.5557	0.0953	0.3182	No	0.9732	0.573	0.534	0.355	No	No
100730_at	Cd180	CD180 antigen	0.9835	0.5496	0.0017	0.0199	No	1.1055	0.36	0.392	0.0573	No	No
100731_at	Prp15	proline-rich protein 15	1.0108	0.8462	0.127	0.7183	No	0.9987	0.994	0.12	0.0856	No	No
100732_at	Rps8	ribosomal protein S8	1.0484	0.0898	0.0001	0.1843	No	0.9845	0.518	0.0005	0.12	No	No
100733_at	Psma2	proteasome (prosome, macropain) subunit, alpha type 2	1.0648	0.1687	0.0154	0.2077	No	1.1006	0.0661	0.0009	0.729	No	No
100734_at	Rpl3	ribosomal protein L3	1.0553	0.2576	0.9648	0.9076	No	1.0185	0.464	0.0228	0.185	No	No
100735_at	Krtap6-3	keratin associated protein 6-3	0.9897	0.819	0.0345	0.8287	No	1.1592	0.259	0.0998	0.291	No	No
100736_at	Barx2	BarH-like homeobox 2	0.9592	0.4738	0.2068	0.5872	No	1.0146	0.801	0.312	0.623	No	No
100737_at	Onecut1	one cut domain, family member 1	0.8838	0.0986	0.5113	0.4949	No	0.8984	0.214	0.65	0.378	No	No
100738_at	Spi15	serine protease inhibitor 15	1.0693	0.2382	0.0157	0.0287	No	1.0362	0.292	0.0046	0.0318	No	No
100739_at	Spi16	serine protease inhibitor 16	0.9783	0.4252	0.0758	0.7398	No	1.0745	0.311	0.0519	0.0836	No	No
100740_at	Gp5	glycoprotein 5 (platelet)	1.0306	0.4353	0.0226	0.1165	No	1.2007	0.005	0.0296	0.0131	Yes	No
100741_at	Fprl1	formyl peptide receptor-like 1	1.0809	0.199	0.4138	0.0746	No	1.0992	0.132	0.0211	0.0205	No	No
100742_at	AA409749	expressed sequence AA409749	1.0214	0.6663	0.0005	0.178	No	1.025	0.179	0.0004	0.0306	No	No
100743_at	D030070L09 Rik	RIKEN cDNA D030070L09 gene	1.0791	0.2508	0.921	0.3608	No	1.0909	0.233	0.499	0.322	No	No
100744_at	6820424L24 Rik	RIKEN cDNA 6820424L24 gene	0.9993	0.9915	0.4628	0.7125	No	1.0057	0.92	0.841	0.185	No	No
100745_r_at	AA408156	expressed sequence AA408156	0.9948	0.5752	0.0208	0.001	No	1.4622	0.0342	0.0178	0.0216	No	No
100746_at	---	Transcribed locus	0.9619	0.578	0.1408	0.7099	No	0.9577	0.485	0.0237	0.924	No	No
100747_at	AA408954	expressed sequence AA408954	0.9757	0.5416	0.0144	0.4336	No	1.0361	0.175	0.0028	0.0236	No	No
100748_at	D10Wsu159 e	DNA segment, Chr 10, Wayne State University 159, expressed	0.9857	0.678	0.4602	0.3407	No	1.0183	0.719	0.0561	0.479	No	No
100749_at	Zbtb37	zinc finger and BTB domain containing 37	1.0293	0.5879	0.0556	0.2135	No	0.9432	0.245	0.009	0.391	No	No
100750_at	AA414903	expressed sequence AA414903	0.9688	0.3694	0.7408	0.6843	No	1.0827	0.2	0.832	0.931	No	No
100751_at	Adam10	a disintegrin and metallopeptidase domain 10	0.9566	0.5108	0.0084	0.6875	No	0.9356	0.168	0.0029	0.616	No	No
100752_at	Rrh	retinal pigment epithelium derived rhodopsin homolog	1.0356	0.5574	0.7187	0.8278	No	1.0166	0.641	0.733	0.0737	No	No
100753_at	Atp5a1	ATP synthase, H+ transporting, mitochondrial F1 complex, alpha subunit, isoform 1	1.0637	0.3868	0.2793	0.7308	No	0.9974	0.957	0.361	0.645	No	No
100754_at	Evi1	ecotropic viral integration site 1	1.0698	0.511	0.0583	0.9008	No	1.035	0.751	0.274	0.545	No	No
100755_at	Ela2	elastase 2, neutrophil	0.962	0.4282	0.0024	0.5646	No	0.9722	0.378	0.0017	0.767	No	No
100756_r_at	Tyms-ps	thymidylate synthase, pseudogene	1.0267	0.4942	0.0415	0.2509	No	0.9912	0.793	0.13	0.372	No	No
100757_at	Cacnb2	calcium channel, voltage-dependent, beta 2 subunit	0.9797	0.6603	0.166	0.2531	No	1.0483	0.478	0.42	0.0177	No	No
100758_at	Rps28	ribosomal protein S28	0.9587	0.1623	0.0496	0.1757	No	0.9674	0.182	0.0409	0.294	No	No
100759_at	Mrc2	mannose receptor, C type 2	0.9844	0.5421	0.2131	0.9573	No	0.9864	0.381	0.0248	0.0045	No	No
100760_at	Dsc1	desmocollin 1	0.9841	0.751	0.6066	0.7542	No	0.9755	0.633	0.12	0.403	No	No
100761_at	Strn	striatin, calmodulin binding protein	0.9712	0.5084	0.1637	0.8229	No	0.942	0.0641	0.953	0.024	No	No
100762_at	Sema6a	sema domain, transmembrane domain (TM), and cytoplasmic domain, (semaphorin) 6A	0.9185	0.0507	0.0071	0.0349	No	0.8628	0.0786	0.0137	0.0449	No	No
100763_at	D2Ertd127e	DNA segment, Chr 2, ERATO Doi 127, expressed	1.0098	0.7917	0.011	0.584	No	1.0204	0.871	0.979	0.94	No	No
100764_at	Il2rb	interleukin 2 receptor, beta chain	0.9561	0.5361	0.0436	0.4609	No	0.8938	0.104	0.0083	0.418	No	No
100765_at	Fndc7	fibronectin type III domain containing 7	1.0113	0.8195	0.0547	0.2477	No	1.0038	0.918	0.572	0.588	No	No
100766_at	Edn3	endothelin 3	0.9645	0.663	0.2781	0.9812	No	1.0004	0.987	0.269	0.701	No	No
100767_at	Gnpnat1	Glucosamine-phosphate N-acetyltransferase 1	1.0381	0.7146	0.048	0.6646	No	0.9913	0.637	0.0001	0.498	No	No
100768_at	---	---	0.9454	0.2615	0.1054	0.8375	No	0.9388	0.556	0.363	0.948	No	No
100769_at	---	---	0.9298	0.1583	0.0678	0.788	No	0.9691	0.733	0.0366	0.434	No	No
100771_at	Blnk	B-cell linker	0.9523	0.1885	0.055	0.0906	No	1.0011	0.925	0.32	0.0737	No	No
100772_g_at	Blnk	B-cell linker	1.0148	0.7231	0.529	0.5056	No	0.9945	0.825	0.114	0.254	No	No
100773_at	Il12a	interleukin 12a	0.9139	0.1359	0.0389	0.3938	No	0.9918	0.901	0.723	0.843	No	No
100774_at	D12Wsu118 e	DNA segment, Chr 12, Wayne State University 118, expressed	0.9753	0.4904	0.0047	0.8318	No	0.9345	0.185	0.0018	0.146	No	No
100775_at	---	---	1.0218	0.3612	0.1821	0.0306	No	0.9949	0.88	0.0371	0.581	No	No
100776_at	6030490I01 Rik	RIKEN cDNA 6030490I01 gene	0.9106	0.1708	0.5749	0.769	No	0.9664	0.521	0.0092	0.398	No	No
100778_at	Cd38	CD38 antigen	0.9998	0.9976	0.3976	0.7131	No	1.031	0.183	0.0247	0.0042	No	No
100779_at	Il12b	interleukin 12b	1.1132	0.1234	0.2588	0.0996	No	1.0946	0.159	0.0623	0.0938	No	No
100780_at	Rps4x	ribosomal protein S4, X-linked	1.0421	0.3241	0.2345	0.2937	No	1.0341	0.211	0.0191	0.79	No	No
100828_at	Myl4	myosin, light polypeptide 4	0.9323	0.0428	0.0089	0.0657	No	0.8599	0.006	0.0653	0.0129	Yes	Yes
100839_at	---	---	1.0459	0.1979	0.0086	0.1597	No	1.0579	0.0918	0.0158	0.122	No	No
100876_at	Fez1	fasciculation and elongation protein zeta 1 (zygin I)	0.9864	0.7155	0.9987	0.3046	No	1.0909	0.0451	0.281	0.212	No	No
100877_at	1810058I24 Rik	RIKEN cDNA 1810058I24 gene	1.0629	0.0136	0	0.0018	No	1.0025	0.433	0.0001	0.013	No	No
100878_at	Strn3	striatin, calmodulin binding protein 3	1.0066	0.9332	0.0057	0.2321	No	0.9858	0.714	0.0148	0.217	No	No
100879_at	Actn3	actinin alpha 3	0.7728	0.0057	0.9508	0.0036	No	0.8208	0.0002	0.0002	0.0013	No	No
100880_at	9830147J24 Rik	RIKEN cDNA 9830147J24 gene	0.9273	0.0245	0	0.0012	No	0.9827	0.556	0.0002	0.0124	No	No
100882_at	Defb1	defensin beta 1	0.8947	0.2069	0.3413	0.5899	No	0.9603	0.52	0.764	0.668	No	No
100883_at	Arfl4	ADP-ribosylation factor 4-like	1.01	0.73	0.0111	0.1488	No	1.0239	0.324	0.0049	0.15	No	No
100884_at	Akr1b8	aldo-keto reductase family 1, member B8	0.998	0.9737	0.22	0.1832	No	0.992	0.825	0.347	0.018	No	No
100885_at	Nek2	NIMA (never in mitosis gene a)-related expressed kinase 2	1.0871	0.1203	0.0139	0.0936	No	1.0271	0.502	0.0229	0.338	No	No
100886_f_at	Mrpl45	mitochondrial ribosomal protein L45	0.97	0.6837	0.1398	0.4727	No	0.9647	0.347	0.0056	0.076	No	No
100887_at	Smc6l1	SMC6 structural maintenance of chromosomes 6-like 1 (yeast)	0.9622	0.501	0.0378	0.1477	No	1.0718	0.0396	0.0025	0.0229	No	No
100888_at	Sorl1	sortilin-related receptor, LDLR class A repeats-containing	1.0258	0.5774	0.0548	0.2806	No	1.0168	0.786	0.249	0.154	No	No
100889_at	Samd4b	sterile alpha motif domain containing 4B	0.9844	0.5031	0.0398	0.7141	No	0.8943	0.397	0.0846	0.0607	No	No
100890_at	Chaf1b	chromatin assembly factor 1, subunit B (p60)	0.8957	0.117	0.0841	0.4956	No	0.8709	0.021	0.0084	0.0329	No	No
100891_at	Smcp	sperm mitochondria-associated cysteine-rich protein	0.9701	0.4958	0.8896	0.109	No	0.9237	0.315	0.0585	0.607	No	No
100892_at	Ndufaf1	NADH dehydrogenase (ubiquinone) 1 alpha subcomplex, assembly factor 1	0.9924	0.9041	0.1989	0.5967	No	0.9881	0.524	0.952	0.0222	No	No

100893_at	Sptlc2	serine palmitoyltransferase, long chain base subunit 2	1.0584	0.1644	0.1625	0.7488	No	1.0074	0.873	0.606	0.389	No	No
100894_at	Orc5l	origin recognition complex, subunit 5-like (S. cerevisiae)	1.003	0.9701	0.0699	0.5967	No	0.9542	0.512	0.0199	0.396	No	No
100895_at	Lsg1	large subunit GTPase 1 homolog (S. cerevisiae)	0.998	0.9635	0.0232	0.1515	No	1.0305	0.167	0.0018	0.0312	No	No
100896_at	Cacng1	calcium channel, voltage-dependent, gamma subunit 1	0.7093	0.0065	0.4505	0.014	Yes	1.0345	0.532	0.0064	0.662	No	No
100897_f_at	Tapbp	TAP binding protein	1.1471	0.0249	0.1418	0.1077	No	1.0425	0.561	0.0203	0.226	No	No
100898_r_at	Tapbp	TAP binding protein	0.9152	0.1878	0.002	0.5582	No	1.0156	0.956	0.0002	0.338	No	No
100899_s_at	Zbtb22	zinc finger and BTB domain containing 22	0.9896	0.7836	0.2559	0.2045	No	1.0341	0.357	0.184	0.514	No	No
100900_at	Hcfc1	host cell factor C1	0.9716	0.4371	0.0031	0.8488	No	0.9973	0.907	0.0059	0.722	No	No
100901_at	Hcfc1	host cell factor C1	1.0306	0.4771	0.1013	0.7762	No	1.0134	0.775	0.0196	0.198	No	No
100902_at	2610019F03 Rik	RIKEN cDNA 2610019F03 gene	0.9405	0.0423	0.6995	0.0112	No	0.9573	0.128	0.0055	0.779	No	No
100903_at	Parp2	poly (ADP-ribose) polymerase family, member 2	1.079	0.0199	0	0.2176	No	1.034	0.717	0.0121	0.331	No	No
100904_at	D14Ertd209 e	DNA segment, Chr 14, ERATO Doi 209, expressed	1.0403	0.1925	0.4561	0.1513	No	0.989	0.745	0.0762	0.348	No	No
100905_at	Herc4	hect domain and RLD 4	0.9627	0.7029	0.0097	0.6048	No	0.9949	0.87	0.0054	0.263	No	No
100906_at	Itgb7	integrin beta 7	1.0405	0.4759	0.1665	0.7448	No	1.0475	0.282	0.178	0.781	No	No
100907_at	Kcne1l	potassium voltage-gated channel, Isk-related family, member 1-like	0.9086	0.0164	0.0061	0.0145	No	1.0257	0.681	0.0575	0.33	No	No
100908_at	Ptpra	protein tyrosine phosphatase, receptor type, A	0.9862	0.8568	0.6552	0.397	No	0.9553	0.569	0.921	0.597	No	No
100909_at	Prss8	protease, serine, 8 (prostasin)	0.9552	0.2154	0.3032	0.3594	No	0.9442	0.0358	0.0679	0.0399	No	No
100910_at	Surf2	surfeit gene 2	0.8752	0.0189	0.0047	0.0286	No	0.8979	0.0073	0.0168	0.004	No	No
100911_at	Gpaa1	GPI anchor attachment protein 1	1.0537	0.2147	0.1612	0.8128	No	1.003	0.781	0.0031	0.166	No	No
100912_at	Dph5	DPH5 homolog (S. cerevisiae)	0.9545	0.2266	0.1013	0.6145	No	0.9707	0.605	0.237	0.358	No	No
100913_at	BC026682	cDNA sequence BC026682	0.9226	0.2288	0.0738	0.8497	No	0.8919	0.204	0.519	0.906	No	No
100914_at	---	PREDICTED: Mus musculus similar to Rap guanine nucleotide exchange factor 2 (PDZ domain containing guanine nucleotide exchange factor 1) (PDZ-GEF1) (LOC620996), mRNA	0.9828	0.5275	0.0413	0.893	No	1.0385	0.29	0.0788	0.226	No	No
100915_at	Myh9	myosin, heavy polypeptide 9, non-muscle	0.9922	0.8779	0.0049	0.039	No	1.0163	0.834	0.0021	0.0259	No	No
100916_at	Slc22a1	solute carrier family 22 (organic cation transporter), member 1	1.0012	0.9837	0.0714	0.9506	No	0.9827	0.912	0.132	0.735	No	No
100917_at	Wdr55	WD repeat domain 55	0.9748	0.6211	0.9854	0.0429	No	1.0246	0.613	0.02	0.54	No	No
100920_at	---	---	1.0157	0.8753	0.2591	0.1604	No	1.0195	0.725	0.0708	0.131	No	No
100921_at	Tnni3	troponin I, cardiac	0.9803	0.7423	0.129	0.3813	No	0.9885	0.926	0.064	0.169	No	No
100923_at	Myo10	myosin X	1.0587	0.1641	0.0222	0.2395	No	1.0672	0.119	0.0261	0.388	No	No
100924_at	Gata3	GATA binding protein 3	1.0323	0.672	0.0039	0.7948	No	1.1459	0.0508	0.0007	0.235	No	No
100925_at	2700089E24 Rik	RIKEN cDNA 2700089E24 gene	1.047	0.3835	0.4036	0.3211	No	0.9991	0.943	0.0792	0.422	No	No
100926_at	---	---	1.0331	0	0.0001	0	No	1.1317	0.114	0.0685	0.217	No	No
100927_at	Pltp	phospholipid transfer protein	1.0251	0.5361	0.0018	0.4278	No	1.0199	0.674	0.0007	0.355	No	No
100928_at	Fbln2	fibulin 2	1.0643	0.3239	0.0004	0.4589	No	0.9841	0.858	0	0.254	No	No
100929_at	Mtmr9	myotubularin related protein 9	1.1219	0.2701	0.01	0.6227	No	1.0783	0.0916	0.0001	0.614	No	No
100931_at	Arsa	arylsulfatase A	0.9296	0.131	0.1618	0.5556	No	0.9172	0.0228	0.658	0.194	No	No
100932_at	Capn1	calpain 1	0.9552	0.3496	0.0165	0.7128	No	1.0802	0.162	0.0054	0.203	No	No
100933_at	Stx1a	syntaxin 1A (brain)	1.0324	0.2795	0.9102	0.0665	No	0.9826	0.595	0.334	0.162	No	No
100935_at	Mlx	MAX-like protein X	0.9384	0.096	0.412	0.2572	No	1.0034	0.898	0.0061	0.895	No	No
100938_at	Ghrh	growth hormone releasing hormone	0.9841	0.7753	0.1386	0.4891	No	1.1006	0.265	0.15	0.443	No	No
100939_at	Zfp282	zinc finger protein 282	1.0284	0.555	0.0792	0.445	No	1.0489	0.136	0.0154	0.0268	No	No
100941_at	Pias2	protein inhibitor of activated STAT 2	0.9834	0.7522	0.3562	0.8552	No	1.0326	0.513	0.123	0.443	No	No
100943_at	Slc1a4	solute carrier family 1 (glutamate/neutral amino acid transporter), member 4	0.9813	0.5424	0.8032	0.7314	No	0.9862	0.751	0.236	0.114	No	No
100944_at	AW112010	expressed sequence AW112010	1.078	0.4076	0.5586	0.6614	No	1.0024	0.962	0.368	0.942	No	No
100946_at	Hspa1b	heat shock protein 1B	0.9574	0.1677	0.0007	0.7226	No	0.8785	0.0102	0.0042	0.0645	No	No
100947_at	---	CDNA clone IMAGE:1265020	1.0539	0.3282	0.0022	0.036	No	0.9806	0.843	0.0014	0.0194	No	No
100948_at	Ank	progressive ankylosis	1.0309	0.2713	0.0049	0.7424	No	0.977	0.578	0.166	0.234	No	No
100949_at	BC004044	cDNA sequence BC004044	0.9735	0.2781	0.0006	0.2868	No	0.9573	0.398	0.008	0.346	No	No
100951_at	Pkd2	polycystic kidney disease 2	0.9208	0.1849	0.1121	0.4876	No	0.9258	0.256	0.119	0.663	No	No
100952_at	Stim1	stromal interaction molecule 1	0.9028	0.0125	0.0891	0.0818	No	0.9038	0.0044	0.184	0.351	Yes	Yes
100953_at	Timeless	timeless homolog (Drosophila)	0.9678	0.4884	0.1171	0.9843	No	0.9925	0.766	0.0013	0.299	No	No
100954_at	Hrb	HIV-1 Rev binding protein	1.0014	0.9855	0.0457	0.7979	No	1.0064	0.684	0.0033	0.104	No	No
100955_at	Ube2t	ubiquitin-conjugating enzyme E2T (putative)	1.0114	0.8693	0.0472	0.5053	No	1.0352	0.627	0.0077	0.99	No	No
100956_at	Kl	klotho	0.9781	0.7785	0.2486	0.3658	No	1.1359	0.0037	0.0026	0.0027	No	No
100957_at	Ssbp1	single-stranded DNA binding protein 1	1.0259	0.7272	0.0105	0.6343	No	1.0111	0.888	0.0208	0.13	No	No
100958_at	Spata13	spermatogenesis associated 13	0.9485	0.1079	0.0045	0.7041	No	0.9492	0.26	0.126	0.15	No	No
100959_at	S100a13	S100 calcium binding protein A13	1.0048	0.9259	0.0028	0.1233	No	1.0072	0.392	0.0001	0.0281	No	No
100960_g_at	S100a13	S100 calcium binding protein A13	0.9927	0.8844	0.053	0.0501	No	0.9513	0.231	0.0274	0.213	No	No
100961_at	Kcnh2	potassium voltage-gated channel, subfamily H (eag-related), member 2	1.0359	0.4363	0.0338	0.5317	No	0.9921	0.951	0.0083	0.271	No	No
100962_at	Nab2	Ngfi-A binding protein 2	1.0229	0.6553	0.0045	0.4537	No	0.9998	0.746	0.0001	0.0794	No	No
100963_at	Pnkd	paroxysmal nonkinesiogenic dyskinesia	0.9013	0.0354	0.0037	0.033	No	0.9432	0.0805	0.0017	0.0712	No	No
100964_at	Vti1b	vesicle transport through interaction with t-SNAREs 1B homolog	1.1713	0.0816	0.0099	0.6141	No	1.0335	0.588	0.0454	0.418	No	No
100965_at	Cox8c	cytochrome c oxidase, subunit VIIIc	1.0517	0.0881	0.0043	0.0087	No	1.0253	0.518	0.0646	0.107	No	No
100966_at	Serpind1	serine (or cysteine) peptidase inhibitor, clade D, member 1	0.9668	0.4128	0.248	0.9397	No	0.9804	0.672	0.201	0.611	No	No
100967_at	Slc27a2	solute carrier family 27 (fatty acid transporter), member 2	1.0643	0.4504	0.3522	0.8189	No	1.0986	0.229	0.0919	0.52	No	No
100968_at	Cstf3	cleavage stimulation factor, 3' pre-RNA, subunit 3	0.908	0.1056	0.0148	0.1322	No	1.0185	0.668	0.0069	0.29	No	No
100970_at	Akt1	thymoma viral proto-oncogene 1	1.0962	0.1317	0.0061	0.8043	No	1.097	0.104	0.0142	0.542	No	No
100971_at	Tead3	TEA domain family member 3	0.958	0.2121	0.0019	0.8056	No	1.0242	0.644	0.0121	0.857	No	No
100972_s_at	Ccl27	chemokine (C-C motif) ligand 27	0.9143	0.0103	0.0049	0.0028	No	0.9715	0.625	0.0162	0.117	No	No
100973_i_at	Ccl27	chemokine (C-C motif) ligand 27	0.9255	0.4478	0.3555	0.6116	No	1.0524	0.416	0.443	0.498	No	No
100974_at	Ssbp4	single stranded DNA binding protein 4	1.0062	0.9111	0.0054	0.1401	No	1.0899	0.132	0.0012	0.644	No	No
100975_f_at	Ssbp4	Single stranded DNA binding protein 4	0.955	0.279	0.108	0.9725	No	0.9894	0.878	0.451	0.413	No	No
100976_at	Ptpn9	protein tyrosine phosphatase, non-receptor type 9	0.9556	0.3008	0.1949	0.6999	No	0.9931	0.688	0.0034	0.0981	No	No
100977_at	Pdk1	pyruvate dehydrogenase kinase, isoenzyme 1	1.0258	0.5588	0.0727	0.5429	No	1.0406	0.614	0.0464	0.437	No	No
100978_at	Frg1	FSHD region gene 1	0.9656	0.1742	0.001	0.0059	No	1.0183	0.391	0.0025	0.0403	No	No
100979_at	Rnf138	ring finger protein 138	1.0327	0.715	0.0163	0.5078	No	0.9597	0.727	0.0099	0.439	No	No
100980_at	Rock1	Rho-associated coiled-coil forming kinase 1	0.9252	0.2308	0.3765	0.1151	No	1.0278	0.722	0.927	0.0198	No	No
100981_at	Ifit1	interferon-induced protein with tetratricopeptide repeats 1	1.1734	0.0375	0.7902	0.0735	No	1.1723	0.0295	0.692	0.0076	No	No
100982_at	Kctd9	potassium channel tetramerisation domain containing 9	0.982	0.6721	0.7491	0.748	No	0.9165	0.0387	0.246	0.966	No	No
100983_at	Prep	prolyl endopeptidase	0.9434	0.2721	0.0525	0.2896	No	0.9696	0.363	0.914	0.555	No	No
100984_at	Atf1	activating transcription factor 1	1.0307	0.501	0.0025	0.6673	No	1.0091	0.877	0.0069	0.862	No	No
100985_at	Siah1a	seven in absentia 1A	0.9968	0.9422	0.0017	0.2333	No	0.9679	0.523	0.0008	0.397	No	No
100986_at	Fhl2	four and a half LIM domains 2	1.0681	0.1864	0.2814	0.2872	No	1.0182	0.561	0.127	0.357	No	No
100987_f_at	1500019G21 Rik	RIKEN cDNA 1500019G21 gene	1.0858	0.1033	0.0395	0.1062	No	1.0446	0.205	0.0089	0.107	No	No
100988_at	Bcl2l11	BCL2-like 11 (apoptosis facilitator)	1.0487	0.3389	0.0044	0.0321	No	0.9841	0.417	0.0021	0.0182	No	No
100989_at	Itgb1bp1	integrin beta 1 binding protein 1	1.0122	0.7966	0.0142	0.7799	No	1.0964	0.146	0.0085	0.174	No	No
100990_g_at	Itgb1bp1	integrin beta 1 binding protein 1	1.037	0.2887	0.2636	0.0381	No	1.0693	0.059	0.199	0.0615	No	No
100991_at	Itgb1bp1	integrin beta 1 binding protein 1	1.0218	0.4319	0.1275	0.8506	No	1.0455	0.129	0.0837	0.573	No	No
100992_at	Phc1	polyhomeotic-like 1 (Drosophila)	0.9928	0.8326	0.8157	0.0663	No	1.0205	0.539	0.122	0.301	No	No
100994_at	Aatk	apoptosis-associated tyrosine kinase	1.0232	0.68	0.0858	0.3231	No	1.0471	0.105	0.001	0.056	No	No
100995_at	Ciz1	CDKN1A interacting zinc finger protein 1	1.0512	0.2138	0.0142	0.0503	No	1.206	0.0382	0.737	0.0513	No	No
100996_at	Agpat6	1-acylglycerol-3-phosphate O-acyltransferase 6 (lysophosphatidic acid acyltransferase, zeta)	0.9632	0.4678	0.0647	0.1923	No	1.0019	0.92	0.0326	0.345	No	No
100997_at	Des	desmin	0.8397	0.0192	0.2771	0.053	No	0.7468	0.0028	0.893	0.0071	No	No
100998_at	H2-Ab1	histocompatibility 2, class II antigen A, beta 1	1.0237	0.521	0.0014	0.3747	No	1.0015	0.947	0.0002	0.233	No	No

101000_at	Oaz2	ornithine decarboxylase antizyme 2	1.0776	0.1975	0.2331	0.5577	No	1.0891	0.112	0.188	0.357	No	No
101001_at	Gpr177	G protein-coupled receptor 177	1.0462	0.5741	0.4302	0.4049	No	0.9915	0.906	0.0989	0.418	No	No
101002_at	Azin1	antizyme inhibitor 1	0.9965	0.9582	0.2508	0.9837	No	1.0462	0.468	0.651	0.585	No	No
101003_at	Sfrs3	splicing factor, arginine/serine-rich 3 (SRp20)	0.9562	0.5074	0.0472	0.4965	No	1.095	0.359	0.0254	0.803	No	No
101004_f_at	Sfrs3	splicing factor, arginine/serine-rich 3 (SRp20)	1.1578	0.2551	0.0102	0.9984	No	0.9399	0.147	0.0259	0.533	No	No
101006_at	Acat2	acetyl-Coenzyme A acetyltransferase 2	0.9872	0.8687	0.0115	0.6508	No	1.0463	0.611	0.581	0.559	No	No
101007_at	Mknk2	MAP kinase-interacting serine/threonine kinase 2	0.8489	0.0006	0.0003	0.0057	No	0.851	0.0035	0.0774	0.0039	No	No
101008_at	Tcerg1	transcription elongation regulator 1 (CA150)	0.9262	0.2323	0.0039	0.8752	No	1.0992	0.0381	0.111	0.0476	No	No
101009_at	Krt2-8	keratin complex 2, basic, gene 8	1.0312	0.3374	0.0015	0.0362	No	1.0431	0.0381	0.0001	0.0881	No	No
101010_at	Mybbp1a	MYB binding protein (P160) 1a	0.9748	0.5746	0.0129	0.9847	No	1.0242	0.669	0.733	0.3	No	No
101011_at	Cct4	chaperonin subunit 4 (delta)	1.0001	0.9982	0.0155	0.3182	No	1.0433	0.36	0.0041	0.438	No	No
101013_at	Oaz1	ornithine decarboxylase antizyme	1.0914	0.0866	0.1833	0.3608	No	1.0641	0.0335	0.512	0.0145	No	No
101014_at	Ifnar2	interferon (alpha and beta) receptor 2	0.9787	0.6782	0.0613	0.611	No	1.0816	0.411	0.0529	0.725	No	No
101015_s_at	Ifnar2	interferon (alpha and beta) receptor 2	1.0119	0.8369	0.9853	0.0309	No	1.0482	0.247	0.0266	0.0313	No	No
101016_at	Arf1	ADP-ribosylation factor 1	1.0266	0.3814	0.009	0.3774	No	0.9998	0.956	0.0648	0.539	No	No
101017_at	Cdk4	cyclin-dependent kinase 4	1.0538	0.133	0.002	0.1252	No	1.0411	0.165	0.0002	0.782	No	No
101019_at	Ctsc	cathepsin C	1.1444	0.2168	0.0069	0.3077	No	1.0639	0.392	0.0035	0.641	No	No
101020_at	Ctsc	cathepsin C	1.1371	0.0327	0.4002	0.3197	No	1.056	0.328	0.905	0.545	No	No
101022_at	Sfrs1	splicing factor, arginine/serine-rich 1 (ASF/SF2)	0.8807	0.0793	0.0125	0.9703	No	1.0706	0.341	0.0291	0.179	No	No
101023_f_at	0610010E21 Rik	RIKEN cDNA 0610010E21 gene	0.9866	0.7707	0.033	0.0544	No	1.0406	0.547	0.839	0.248	No	No
101024_i_at	Sprr2a	small proline-rich protein 2A	0.9464	0.3541	0.0017	0.5283	No	0.8259	0.106	0.0046	0.909	No	No
101025_f_at	Sprr2a	small proline-rich protein 2A	0.9102	0.0816	0.0021	0.8882	No	1.0577	0.582	0.188	0.787	No	No
101026_at	Pttg1	pituitary tumor-transforming 1	0.9413	0.1875	0.7756	0.3749	No	1.0293	0.753	0.298	0.503	No	No
101027_s_at	Pttg1	pituitary tumor-transforming 1	0.9305	0.1803	0.0059	0.8886	No	1.0332	0.385	0.0007	0.484	No	No
101028_i_at	Actc1	actin, alpha, cardiac	0.8416	0.0063	0.0033	0.0014	No	0.8132	0.0024	0.0025	0.0022	No	No
101029_f_at	Actc1	actin, alpha, cardiac	0.8767	0.0159	0.003	0.0052	No	0.859	0.0001	0.0003	0	No	No
101030_at	Rhob	ras homolog gene family, member B	0.9227	0.2468	0.178	0.9892	No	0.9239	0.376	0.0952	0.739	No	No
101031_at	Surf1	surfeit gene 1	0.941	0.1902	0.1905	0.0712	No	0.9576	0.385	0.033	0.0755	No	No
101033_at	Nudc	nuclear distribution gene C homolog (Aspergillus)	0.9838	0.8292	0.0019	0.5741	No	1.0716	0.224	0.257	0.107	No	No
101034_at	Grb2	growth factor receptor bound protein 2	0.9679	0.5489	0.4891	0.345	No	0.9519	0.16	0.499	0.361	No	No
101035_at	Api5	apoptosis inhibitor 5	1.0613	0.2103	0.096	0.2228	No	1.0423	0.273	0.0085	0.508	No	No
101036_at	Tomm20 /// LOC546321 /// LOC673774	translocase of outer mitochondrial membrane 20 homolog (yeast) /// similar to translocase of outer mitochondrial membrane 20 homolog /// similar to translocase of outer mitochondrial membrane 20 homolog	1.1701	0.0242	0.2534	0.473	No	1.2202	0.0183	0.408	0.0085	No	No
101037_at	Abi1	abl-interactor 1	0.9986	0.9746	0.0108	0.2713	No	0.9763	0.451	0.59	0.298	No	No
101039_at	Col4a2	procollagen, type IV, alpha 2	1.0062	0.7902	0.0072	0.0106	No	0.9816	0.753	0.0878	0.334	No	No
101040_at	Capn2	calpain 2	0.9283	0.08	0.0006	0.24	No	0.9298	0.103	0.0178	0.804	No	No
101041_at	Krt1-14	keratin complex 1, acidic, gene 14	0.9364	0.5701	0.0436	0.2224	No	1.0973	0.344	0.976	0.0839	No	No
101042_f_at	Pepd	peptidase D	1.1262	0.2811	0.0098	0.42	No	1.0969	0.117	0.006	0.0323	No	No
101043_f_at	Try4 /// 1810049H19 Rik	trypsin 4 /// RIKEN cDNA 1810049H19 gene	0.897	0.1946	0.1254	0.7476	No	1.1111	0.278	0.7	0.0978	No	No
101044_at	Alad	aminolevulinate, delta-, dehydratase	1.1228	0.0677	0.3452	0.9746	No	0.9094	0.0292	0.143	0.149	No	No
101045_at	Hadh2	hydroxyacyl-Coenzyme A dehydrogenase type II	1.0332	0.3438	0.0017	0.0805	No	1.0289	0.522	0.0292	0.232	No	No
101046_at	Vim	vimentin	1.001	0.9791	0.9761	0.7895	No	1.0703	0.0545	0.233	0.0196	No	No
101047_at	Cd99	CD99 antigen	0.9877	0.8772	0.0014	0.2857	No	0.9713	0.886	0.0012	0.403	No	No
101048_at	Ptprc	protein tyrosine phosphatase, receptor type, C	0.9664	0.3554	0.0302	0.5896	No	0.9627	0.695	0.011	0.281	No	No
101049_at	Snora65 /// Rpl12 /// LOC546695 /// LOC622589 /// LOC630855 /// LOC633363 /// LOC633570 /// LOC639477 /// LOC668706 /// LOC668782 /// LOC668844 /// LOC669503 /// LOC671640 /// LOC672158 /// LOC672173	small nucleolar RNA, H/ACA box 65 /// ribosomal protein L12 /// similar to 60S ribosomal protein L12 /// similar to 60S ribosomal protein L12 /// similar to 60S ribosomal protein L12 /// similar to 60S ribosomal protein L12 /// similar to 60S ribosomal protein L12 /// similar to 60S ribosomal protein L12 /// similar to 60S ribosomal protein L12 /// similar to 60S ribosomal protein L12 /// similar to 60S ribosomal protein L12 /// similar to 60S ribosomal protein L12 /// similar to 60S ribosomal protein L12 /// similar to 60S ribosomal protein L12	1.0237	0.6315	0.5755	0.3215	No	1.1078	0.128	0.614	0.405	No	No
101050_at	Rfk	riboflavin kinase	1.0423	0.4638	0.0043	0.0866	No	0.9914	0.949	0.0241	0.534	No	No
101051_at	S100a3	S100 calcium binding protein A3	1.0165	0.6521	0.0274	0.0551	No	1.0103	0.885	0.1	0.877	No	No
101052_g_at	S100a3	S100 calcium binding protein A3	0.9651	0.2534	0.255	0.2177	No	0.9656	0.568	0.21	0.178	No	No
101053_at	Prpf6	PRP6 pre-mRNA splicing factor 6 homolog (yeast)	0.929	0.003	0.0342	0.0018	No	0.9027	0.0366	0.574	0.0987	No	No
101054_at	Cd74	CD74 antigen (invariant polypeptide of major histocompatibility complex, class II antigen-associated)	0.9749	0.5298	0.4714	0.0991	No	1.0052	0.851	0.391	0.225	No	No
101055_at	Ppgb	protective protein for beta-galactosidase	0.8701	0.0264	0.002	0.0268	No	0.9089	0.154	0.0015	0.0119	No	No
101056_at	Rdx	radixin	0.9855	0.7193	0.0091	0.0715	No	0.9401	0.245	0.332	0.291	No	No
101057_at	A430005L14 Rik	RIKEN cDNA A430005L14 gene	1.065	0.0585	0.287	0.0837	No	1.0594	0.215	0.0668	0.0378	No	No
101058_at	Amy1	amylase 1, salivary	0.8137	0.0276	0.2206	0.8099	No	0.9328	0.0074	0.0002	0.344	Yes	Yes
101059_at	Ndn	necdin	1.0626	0.3412	0.4738	0.4401	No	1.0426	0.77	0.946	0.714	No	No
101060_at	Pdia3	protein disulfide isomerase associated 3	1.0967	0.0664	0.8089	0.4349	No	1.0809	0.299	0.279	0.435	No	No
101061_at	Ssr2	signal sequence receptor, beta	1.1869	0.0323	0.9229	0.4026	No	1.0746	0.0433	0.0017	0.0238	No	No
101062_at	Hmox2	heme oxygenase (decycling) 2	1.0155	0.7485	0.8004	0.7523	No	1.0226	0.832	0.0862	0.984	No	No
101063_at	Tnnc1	troponin C, cardiac/slow skeletal	0.7929	0.0078	0.0308	0.01	No	0.8623	0.0119	0.0202	0.0084	No	No
101064_at	Plrg1	pleiotropic regulator 1, PRL1 homolog (Arabidopsis)	0.9309	0.2609	0.0128	0.3416	No	0.982	0.598	0.0973	0.198	No	No
101065_at	Pcna	proliferating cell nuclear antigen	1.1973	0.0597	0.0465	0.163	No	1.1419	0.123	0.898	0.529	No	No
101067_at	Mrpl24	mitochondrial ribosomal protein L24	0.9603	0.6461	0.1502	0.2275	No	0.9719	0.917	0.0292	0.0838	No	No
101068_at	Mkrn1	makorin, ring finger protein, 1	0.9573	0.4108	0.1605	0.7614	No	0.9888	0.682	0.0074	0.131	No	No
101069_g_at	Mkrn1	makorin, ring finger protein, 1	1.0136	0.4756	0.0002	0.004	No	1.035	0.301	0.0031	0.0596	No	No
101070_at	Mkrn1	makorin, ring finger protein, 1	0.9794	0.7642	0.0333	0.4526	No	0.9651	0.664	0.304	0.968	No	No
101071_at	Myh6	myosin, heavy polypeptide 6, cardiac muscle, alpha	0.8343	0.1128	0.0949	0.1326	No	0.9003	0.0868	0.595	0.455	No	No
101072_at	Eif4ebp2	eukaryotic translation initiation factor 4E binding protein 2	0.9122	0.1124	0.0273	0.9887	No	0.911	0.284	0.0839	0.66	No	No
101073_at	Lrp1	low density lipoprotein receptor-related protein 1	1.0032	0.911	0.0634	0.1294	No	1.0461	0.0097	0.0062	0.0013	No	No
101074_at	Ddost	dolichyl-di-phosphooligosaccharide-protein glycotransferase	1.0619	0.2618	0.4412	0.2218	No	1.0766	0.186	0.101	0.441	No	No
101075_f_at	Agr2	anterior gradient 2 (Xenopus laevis)	1.1349	0.0549	0.0076	0.004	No	1.1078	0.165	0.346	0.0536	No	No
101076_r_at	Agr2	anterior gradient 2 (Xenopus laevis)	0.8925	0.3683	0.0049	0.2513	No	0.9254	0.213	0.0001	0.955	No	No

101077_at	Slc4a1	solute carrier family 4 (anion exchanger), member 1	1.0102	0.8367	0.9142	0.7801	No	1.0484	0.305	0.306	0.0789	No	No
101078_at	Bsg	basigin	1.0014	0.9546	0.0039	0.1804	No	0.9459	0.337	0.016	0.296	No	No
101079_at	Nxf1	nuclear RNA export factor 1 homolog (S. cerevisiae)	1.0601	0.3618	0.109	0.7392	No	1.0314	0.216	0.135	0.827	No	No
101080_at	Col5a1	Procollagen, type V, alpha 1	0.9081	0.0407	0.0066	0.0594	No	0.9067	0.0031	0.0024	0.0014	No	No
101081_at	Ctbp1	C-terminal binding protein 1	1.1232	0.0046	0.7027	0.2796	Yes	1.0747	0.0338	0.638	0.0825	No	Yes
101082_at	LOC677317	similar to NADP-dependent malic enzyme (NADP-ME) (Malic enzyme 1)	1.098	0.3145	0.6686	0.2919	No	1.0818	0.155	0.204	0.878	No	No
101083_s_at	Lsm2	LSM2 homolog, U6 small nuclear RNA associated (S. cerevisiae)	1.0315	0.4653	0.0422	0.1393	No	1.0525	0.18	0.0062	0.124	No	No
101084_f_at	Dpm3	dolichyl-phosphate mannosyltransferase polypeptide 3	0.9568	0.4211	0.0032	0.085	No	0.9466	0.57	0.0026	0.0885	No	No
101085_at	Mrps24	mitochondrial ribosomal protein S24	1.0155	0.498	0.0035	0.0217	No	1.0119	0.73	0.007	0.542	No	No
101086_f_at	Cnbp1	cellular nucleic acid binding protein 1	1.0374	0.4649	0.9464	0.3805	No	1.0235	0.479	0.219	0.11	No	No
101087_r_at	Cnbp1	cellular nucleic acid binding protein 1	0.9342	0.3184	0.0003	0.2838	No	1.015	0.73	0.0014	0.637	No	No
101088_f_at	Cnbp1	cellular nucleic acid binding protein 1	0.9266	0.1664	0.0474	0.218	No	0.9339	0.0652	0.0003	0.0053	No	No
101089_at	Pdlim3	PDZ and LIM domain 3	0.7641	0.0666	0.7231	0.0299	No	0.8747	0.193	0.0244	0.0679	No	No
101090_at	Fbn1	fibrillin 1	0.9301	0.0284	0	0.0689	No	0.9294	0.0585	0.0005	0.599	No	No
101091_at	Sumo1	SMT3 suppressor of mif two 3 homolog 1 (yeast)	1.024	0.5467	0.0025	0.1208	No	1.006	0.691	0.0021	0.135	No	No
101093_at	Col4a1	procollagen, type IV, alpha 1	0.9259	0.0924	0.0012	0.1412	No	0.9453	0.375	0.0289	0.23	No	No
101094_at	Higd1a	HIG1 domain family, member 1A	1.0194	0.8493	0.017	0.9833	No	0.9826	0.795	0.0027	0.954	No	No
101095_at	Mfap2	microfibrillar-associated protein 2	1.0747	0.063	0.0004	0.7911	No	1.0928	0.048	0.0004	0.69	No	No
101096_s_at	Hax1	HCLS1 associated X-1	0.9337	0.0971	0.1303	0.048	No	0.928	0.108	0.026	0.112	No	No
101097_at	Mrpl21	mitochondrial ribosomal protein L21	1.0515	0.1595	0.1963	0.1769	No	0.964	0.271	0.685	0.0156	No	No
101099_at	Nsg1	neuron specific gene family member 1	0.9183	0.248	0.0041	0.2734	No	1.0707	0.0451	0.0004	0.0089	No	No
101101_at	Ppp2cb	protein phosphatase 2 (formerly 2A), catalytic subunit, beta isoform	1.0107	0.882	0.0883	0.4016	No	1.0057	0.969	0.001	0.369	No	No
101102_at	Igbp1	immunoglobulin (CD79A) binding protein 1	0.9678	0.4538	0.1012	0.6331	No	0.9009	0.114	0.0613	0.823	No	No
101103_at	Cyp4a14	cytochrome P450, family 4, subfamily a, polypeptide 14	1.0462	0.4236	0.4205	0.1981	No	1.0281	0.358	0.0015	0.104	No	No
101104_at	Dscr3	Down syndrome critical region gene 3	1.0007	0.9826	0.1362	0.0664	No	0.9188	0.123	0.514	0.509	No	No
101105_at	Banf1	barrier to autointegration factor 1	1.0591	0.1526	0.2631	0.4419	No	1.0801	0.0137	0.0647	0.612	No	No
101106_at	E430034L04Rik	RIKEN cDNA E430034L04 gene	0.9363	0.4721	0.1739	0.5112	No	0.9406	0.211	0.138	0.566	No	No
101107_at	Calu	calumenin	1.0031	0.9392	0.0052	0.3612	No	0.9571	0.42	0.0008	0.535	No	No
101108_at	Nasp	nuclear autoantigenic sperm protein (histone-binding)	1.093	0.0239	0.0024	0.0932	No	1.1255	0.0615	0.0037	0.215	No	No
101109_at	Dag1	dystroglycan 1	0.9541	0.1944	0.0099	0.097	No	0.9452	0.268	0.0263	0.381	No	No
101110_at	Col6a3 /// LOC674521	procollagen, type VI, alpha 3 /// similar to alpha 3 type VI collagen isoform 1 precursor	0.94	0.2409	0.6049	0.0388	No	0.9566	0.232	0.23	0.0219	No	No
101111_at	Rhoa	ras homolog gene family, member A	0.9725	0.4837	0.8415	0.1824	No	0.9939	0.962	0.324	0.771	No	No
101112_g_at	Rhoa /// LOC433749 /// LOC631743	ras homolog gene family, member A /// similar to aplysia ras-related homolog A2 /// similar to aplysia ras-related homolog A2	0.9999	0.9972	0.0011	0.3238	No	0.9692	0.581	0.0004	0.509	No	No
101113_at	Rhoa	ras homolog gene family, member A	0.9166	0.3371	0.0021	0.1203	No	0.9698	0.391	0.0008	0.206	No	No
101114_at	Srprb	signal recognition particle receptor, B subunit	1.0993	0.0351	0.2328	0.0305	No	1.0139	0.476	0.0124	0.0781	No	No
101115_at	Ltf	lactotransferrin	0.8686	0.0086	0.0079	0.0029	No	0.9314	0.152	0.207	0.0075	No	No
101116_at	Pip5k2a	Phosphatidylinositol-4-phosphate 5-kinase, type II, alpha	0.9882	0.7282	0.3236	0.1684	No	0.882	0.0925	0.977	0.282	No	No
101117_at	AA511261	expressed sequence AA511261	0.9565	0.5689	0.9847	0.2957	No	1.0526	0.277	0.005	0.925	No	No
101118_at	D12Ertd551e	DNA segment, Chr 12, ERATO Doi 551, expressed	1.0185	0.3722	0	0.141	No	0.9265	0.379	0.0728	0.593	No	No
101119_at	---	---	1.0007	0.9836	0.0324	0.467	No	1.0965	0.415	0.253	0.896	No	No
101120_at	Capn2	Calpain 2	0.9436	0.1203	0.1243	0.4318	No	1.0494	0.367	0.228	0.192	No	No
101121_at	---	Transcribed locus	0.9772	0.7298	0.2772	0.0958	No	0.9848	0.917	0.0982	0.173	No	No
101122_at	Epha6	Eph receptor A6	1.1151	0.1119	0.0226	0.1184	No	1.1485	0.0307	0.0145	0.0075	No	No
101123_at	Itm2b	integral membrane protein 2B	1.0543	0.2158	0.0006	0.1081	No	0.968	0.0812	0.0004	0.0033	No	No
101124_at	---	Transcribed locus	1.0953	0.5728	0.891	0.3115	No	1.058	0.5	0.511	0.0795	No	No
101125_at	Hyou1	Hypoxia up-regulated 1	1.018	0.5373	0.6355	0.5651	No	1.1547	0.247	0.0544	0.702	No	No
101126_r_at	---	PREDICTED: Mus musculus hypothetical protein LOC628408 (LOC628408), mRNA	0.9065	0.1059	0.1725	0.6937	No	0.6844	0.147	0.448	0.403	No	No
101127_at	Chx10	C. elegans ceh-10 homeo domain containing homolog	1.0601	0.4019	0.0054	0.4386	No	1.0929	0.367	0.0067	0.0324	No	No
101128_at	Cacna1s	calcium channel, voltage-dependent, L type, alpha 1S subunit	0.7648	0.0016	0.9925	0.5244	Yes	0.9701	0.248	0.0022	0.103	No	No
101129_at	Rpl5	ribosomal protein L5	1.0788	0.1118	0.3178	0.4357	No	1.054	0.123	0.342	0.143	No	No
101130_at	Col1a2	procollagen, type I, alpha 2	0.9273	0.0427	0.0101	0.0367	No	0.9244	0.0463	0.389	0.224	No	No
101131_at	Chrna7	cholinergic receptor, nicotinic, alpha polypeptide 7	0.9567	0.2921	0.4333	0.1306	No	1.0061	0.882	0.49	0.214	No	No
101132_at	Scn7a	sodium channel, voltage-gated, type VII, alpha	0.9608	0.6126	0.9671	0.8173	No	1.0224	0.74	0.0308	0.334	No	No
101133_at	Lcn3	lipocalin 3	0.9253	0.2708	0.0743	0.938	No	1.0392	0.507	0.91	0.181	No	No
101134_at	Gcap8	granule cell antiserum positive 8	0.9348	0.2253	0.2866	0.987	No	0.8155	0.0949	0.0509	0.5	No	No
101135_at	Calcr	calcitonin receptor	0.9657	0.534	0.8577	0.588	No	1.1298	0.0917	0.118	0.0049	No	No
101136_at	Tnfsf8	tumor necrosis factor (ligand) superfamily, member 8	0.96	0.6347	0.5246	0.4222	No	1.032	0.577	0.0368	0.45	No	No
101137_at	Rps3	ribosomal protein S3	1.0206	0.6094	0.056	0.4996	No	0.9944	0.722	0.0018	0.142	No	No
101138_at	Gabrb2	gamma-aminobutyric acid (GABA-A) receptor, subunit beta 2	0.9596	0.5592	0.1642	0.9099	No	1.0322	0.286	0.0008	0.196	No	No
101139_r_at	Muc10	mucin 10, submandibular gland salivary mucin	0.924	0.2936	0.2331	0.5381	No	0.9231	0.334	0.114	0.625	No	No
101140_at	Htr1a	5-hydroxytryptamine (serotonin) receptor 1A	0.9983	0.9688	0.3676	0.3546	No	1.1715	0.0266	0.683	0.0044	No	No
101141_at	---	---	1.0463	0.2174	0.0087	0.2066	No	0.962	0.594	0.104	0.502	No	No
101142_at	Fzd6	frizzled homolog 6 (Drosophila)	0.9715	0.5397	0.0538	0.7832	No	0.9097	0.349	0.296	0.178	No	No
101143_at	Fzd7	frizzled homolog 7 (Drosophila)	1.0474	0.2892	0.009	0.0977	No	1.0394	0.151	0.0046	0.102	No	No
101144_at	Il18r1	interleukin 18 receptor 1	0.984	0.4814	0.0729	0.0311	No	1.0806	0.325	0.104	0.2	No	No
101145_at	Cep250	centrosomal protein 250	1.0183	0.6281	0.0046	0.3372	No	1.028	0.628	0.0324	0.162	No	No
101146_at	Gsh1	genomic screened homeo box 1	1.0639	0.3342	0.1072	0.1605	No	0.9944	0.819	0.0001	0.0561	No	No
101147_at	Gcet2	germinal center expressed transcript 2	0.9995	0.9888	0.6699	0.9633	No	1.0469	0.661	0.45	0.986	No	No
101148_at	Prkci	protein kinase C, iota	0.914	0.1397	0.1414	0.305	No	0.9256	0.236	0.072	0.474	No	No
101149_at	Shc3	src homology 2 domain-containing transforming protein C3	0.8722	0.2684	0.0863	0.3569	No	0.9743	0.673	0.915	0.483	No	No
101150_at	Etv2	ets variant gene 2	0.9352	0.2117	0.0015	0.4028	No	0.9372	0.272	0.0253	0.652	No	No
101151_at	Rcvrn	recoverin	0.9642	0.1376	0.0012	0.1206	No	0.9954	0.84	0.0112	0.397	No	No
101152_at	Htr5a	5-hydroxytryptamine (serotonin) receptor 5A	0.9647	0.5949	0.3653	0.6069	No	0.9349	0.195	0.161	0.353	No	No
101153_at	Ofa	oncofetal antigen	0.9923	0.8866	0.6899	0.4011	No	1.1556	0.138	0.0346	0.163	No	No
101154_at	AA517023	expressed sequence AA517023	0.9136	0.3003	0.4872	0.2895	No	0.9433	0.894	0.0033	0.0227	No	No
101155_at	Hsf2	heat shock factor 2	0.9731	0.583	0.0077	0.623	No	0.965	0.52	0.0179	0.32	No	No
101156_at	Npn2	neoplastic progression 2	0.9526	0.4856	0.045	0.5978	No	0.8733	0.0504	0.0129	0.0091	No	No
101157_at	Igh-1a	Immunoglobulin heavy chain 1a (serum IgG2a)	1.1207	0.012	0.0225	0.0842	No	0.9818	0.38	0.0021	0.0346	No	No
101158_at	Zfp2	zinc finger protein 2	1.0065	0.9457	0.6875	0.1863	No	1.0198	0.746	0.412	0.236	No	No
101159_at	Mitf	microphthalmia-associated transcription factor	1.0149	0.6117	0.0027	0.0075	No	1.0217	0.54	0.0311	0.0423	No	No
101160_at	Cxcl2	chemokine (C-X-C motif) ligand 2	1.0029	0.9609	0.3274	0.0771	No	1.0059	0.884	0.435	0.405	No	No
101161_at	Mc1r	melanocortin 1 receptor	1.0077	0.8579	0.1455	0.4258	No	1.106	0.0033	0.151	0.0219	Yes	No
101162_at	Myf5	myogenic factor 5	1.0039	0.9602	0.01	0.5015	No	0.8266	0.0061	0.0001	0.0293	Yes	No
101163_at	Rag2	recombination activating gene 2	0.9271	0.0101	0.1238	0.6736	No	0.9914	0.723	0.024	0.446	No	No
101164_at	Tnfsf4	tumor necrosis factor (ligand) superfamily, member 4	1.0293	0.2985	0.2575	0.2599	No	0.9545	0.613	0.105	0.51	No	No
101165_at	---	---	0.9681	0.4533	0.4052	0.4318	No	0.9428	0.37	0.159	0.67	No	No
101166_at	Zscan2	zinc finger and SCAN domain containing 2	0.9822	0.6871	0.0052	0.0728	No	0.969	0.593	0.0128	0.921	No	No
101167_at	---	---	1.0659	0.2529	0.9271	0.463	No	1.1029	0.0996	0.0056	0.046	No	No
101168_at	Dnm3os	dynamin 3, opposite strand	1.0124	0.778	0.0027	0.034	No	1.0507	0.248	0.0145	0.034	No	No
101169_at	Rds	retinal degeneration, slow (retinitis pigmentosa 7)	0.9555	0.4475	0.0331	0.2582	No	0.9405	0.228	0.0112	0.269	No	No
101170_at	Htr1f	5-hydroxytryptamine (serotonin) receptor 1F	1.013	0.8558	0.8504	0.5276	No	1.3114	0.0718	0.203	0.144	No	No
101171_at	Dvl3	dishevelled 3, dsh homolog (Drosophila)	0.9536	0.4863	0.044	0.9147	No	0.9447	0.116	0.0071	0.112	No	No
101172_at	Zfp92	zinc finger protein 92	1.0157	0.6885	0.0629	0.1335	No	1.0159	0.723	0.0052	0.735	No	No
101173_at	Pctp	phosphatidylcholine transfer protein	1.0154	0.6776	0.026	0.4676	No	1.0881	0.0176	0.619	0.039	No	No
101174_at	Ocm	oncomodulin	1.0385	0.3399	0.1445	0.1065	No	0.844	0.0072	0.0282	0.0017	No	No
101175_at	Map3k2	mitogen activated protein kinase kinase kinase 2	0.9393	0.6215	0.1242	0.4419	No	1.0954	0.391	0.153	0.553	No	No

101176_at	Lcn4	lipocalin 4	1.0003	0.9939	0.0891	0.4695	No	1.0201	0.408	0.0324	0.0746	No	No
101177_at	Acvr1b	activin A receptor, type 1B	0.9251	0.107	0.0403	0.7924	No	1.0104	0.79	0.0145	0.259	No	No
101178_at	Sema3c	sema domain, immunoglobulin domain (Ig), short basic domain, secreted, (semaphorin) 3C	0.9608	0.5445	0.9968	0.5944	No	0.9769	0.624	0.108	0.38	No	No
101179_at	---	---	1.1667	0.0838	0	0.0196	No	0.9739	0.162	0	0.0207	No	No
101180_at	Atm	ataxia telangiectasia mutated homolog (human)	1.0396	0.6714	0.0157	0.2374	No	1.1149	0.36	0.434	0.432	No	No
101181_at	Cnga2	cyclic nucleotide gated channel alpha 2	1.0655	0.244	0.0649	0.602	No	1.1584	0.019	0.636	0.256	No	No
101182_at	Gli3	GLI-Kruppel family member GLI3	1.0224	0.4438	0.0283	0.0424	No	0.997	0.991	0.427	0.462	No	No
101183_at	Syt2	synaptotagmin II	1.0322	0.6047	0.0581	0.2191	No	1.111	0.287	0.0669	0.437	No	No
101184_at	Hsd17b3	hydroxysteroid (17-beta) dehydrogenase 3	1.1249	0.0073	0.9406	0.007	No	1.0445	0.578	0.0545	0.0452	No	No
101185_at	Foxl1	forkhead box L1	1.0126	0.7745	0.8215	0.1233	No	1.0848	0.198	0.953	0.0438	No	No
101186_at	Ppnr	per-pentamer repeat gene	0.9154	0.119	0.0278	0.1802	No	0.9927	0.877	0.0049	0.896	No	No
101187_at	Serpina5	serine (or cysteine) peptidase inhibitor, clade A, member 5	1.0286	0.6338	0.226	0.8537	No	1.0956	0.202	0.636	0.321	No	No
101188_at	Kcnj3	potassium inwardly-rectifying channel, subfamily J, member 3	1.0112	0.8	0.1136	0.9122	No	1.0155	0.741	0.513	0.248	No	No
101189_at	Hrk	harakiri, BCL2 interacting protein (contains only BH3 domain)	1.0053	0.9197	0.0168	0.3947	No	0.9171	0.215	0.3	0.205	No	No
101190_at	Mtnr1a	melatonin receptor 1A	1.0184	0.7692	0.2524	0.4518	No	0.9763	0.982	0.919	0.888	No	No
101191_at	Doc2b	double C2, beta	1.0451	0.2169	0.0099	0.0021	No	1.0259	0.515	0.108	0.0545	No	No
101192_at	Lhx5	LIM homeobox protein 5	0.96	0.3016	0.1046	0.4034	No	0.9379	0.399	0.0689	0.358	No	No
101193_at	Zik1	zinc finger protein interacting with K protein 1	1.0368	0.1078	0.0139	0.6121	No	0.9124	0.382	0.742	0.651	No	No
101194_at	Stra8	stimulated by retinoic acid gene 8	0.9638	0.235	0.4009	0.1267	No	0.986	0.82	0.885	0.219	No	No
101195_at	Mylc2pl	myosin light chain 2, precursor lymphocyte-specific	0.9717	0.4587	0.5562	0.1314	No	1.0556	0.517	0.285	0.863	No	No
101196_at	Pcsk6	proprotein convertase subtilisin/kexin type 6	0.9466	0.0426	0.0003	0.0164	No	0.8924	0.0124	0.0013	0.0082	No	No
101197_at	Glrp1	glutamine repeat protein 1	1.0731	0.0342	0.0132	0.1221	No	1.1642	0.0126	0.0026	0.0846	No	No
101198_at	Cplx1	complexin 1	1.0489	0.3336	0.9052	0.8949	No	1.0312	0.335	0.154	0.706	No	No
101199_at	Cmklr1	chemokine-like receptor 1	0.9802	0.6915	0.0118	0.1471	No	0.9463	0.201	0.0063	0.0051	No	No
101200_at	Cyp19a1	cytochrome P450, family 19, subfamily a, polypeptide 1	0.9609	0.4081	0.0074	0.6032	No	1.0229	0.891	0.0083	0.301	No	No
101201_r_at	---	---	0.9928	0.9171	0.8601	0.4498	No	1.1304	0.508	0.7	0.535	No	No
101202_at	---	---	0.9657	0.5938	0.2672	0.419	No	1.0031	0.951	0.383	0.944	No	No
101203_r_at	Srrm2	serine/arginine repetitive matrix 2	1.0529	0.0734	0.0005	0.0812	No	0.9483	0.413	0.0035	0.247	No	No
101204_at	---	---	1.0016	0.9307	0.9101	0.0638	No	1.0436	0.143	0.245	0.056	No	No
101205_at	---	Transcribed locus	0.973	0.4322	0.0143	0.5978	No	0.9373	0.776	0.094	0.291	No	No
101206_at	T25656	expressed sequence T25656	1.1044	0.1746	0.5511	0.9168	No	1.1529	0.132	0.11	0.0577	No	No
101207_at	Ppia	peptidylprolyl isomerase A	1.0809	0.0046	0.4578	0.8778	Yes	1.0395	0.0111	0.482	0.221	No	Yes
101208_at	Slc7a1	solute carrier family 7 (cationic amino acid transporter, y+ system), member 1	0.9628	0.6609	0.5709	0.3249	No	0.9824	0.775	0.0422	0.352	No	No
101209_at	Fcer1a	Fc receptor, IgE, high affinity I, alpha polypeptide	0.8972	0.1007	0.2045	0.4993	No	1.3634	0.0153	0.0478	0.0203	No	No
101210_at	Zfp651	zinc finger protein 651	1.006	0.8803	0.3417	0.4738	No	0.9333	0.152	0.603	0.181	No	No
101211_at	D7Bwg0826e	DNA segment, Chr 7, Brigham & Women's Genetics 0826 expressed	1.0143	0.6352	0.0202	0.487	No	0.9998	0.999	0.416	0.644	No	No
101212_at	Rps7	ribosomal protein S7	1.0463	0.0874	0.0024	0.0179	No	1.0299	0.179	0.0005	0.125	No	No
101213_at	Arbp	acidic ribosomal phosphoprotein P0	1.0454	0.1356	0.0813	0.7555	No	1.0352	0.195	0.0275	0.905	No	No
101214_f_at	LOC14433 /// LOC380687 /// AK190093 /// Gapdh /// RP23-227C11.3	similar to glyceraldehyde-3-phosphate dehydrogenase /// similar to glyceraldehyde-3-phosphate dehydrogenase /// cDNA sequence AK190093 /// glyceraldehyde-3-phosphate dehydrogenase /// glyceraldehyde-3-phosphate dehydrogenase pseudogene	0.9821	0.7034	0.0167	0.0705	No	0.97	0.566	0.0014	0.0144	No	No
101215_at	Gabra2	gamma-aminobutyric acid (GABA-A) receptor, subunit alpha 2	1.0057	0.8475	0.2589	0.012	No	1.0914	0.575	0.467	0.253	No	No
101216_at	---	Transcribed locus	0.9812	0.6492	0.0105	0.957	No	0.9912	0.828	0.0076	0.582	No	No
101217_at	---	---	1.0521	0.3946	0.0001	0.1924	No	1.0566	0.223	0.0001	0.478	No	No
101218_at	D7Ertd1e	DNA segment, Chr 7, ERATO Doi 1, expressed	1.0367	0.1169	0.0566	0.0358	No	1.0863	0.0251	0.0034	0.019	No	No
101219_at	---	Transcribed locus	0.957	0.4364	0.5968	0.9294	No	0.9511	0.207	0.002	0.732	No	No
101220_at	C76628	expressed sequence C76628	0.9161	0.0859	0.0282	0.0075	No	0.8838	0.198	0.0158	0.386	No	No
101221_at	Rab3il1	RAB3A interacting protein (rabin3)-like 1	0.9904	0.8276	0.001	0.309	No	0.9723	0.452	0.0008	0.941	No	No
101222_f_at	1810054D07Rik	RIKEN cDNA 1810054D07 gene	0.9902	0.7685	0.1011	0.343	No	1.043	0.37	0.0524	0.399	No	No
101223_r_at	---	---	0.9695	0.2363	0.6219	0.0042	No	0.9974	0.899	0.195	0.16	No	No
101224_at	D8Ertd107e	DNA segment, Chr 8, ERATO Doi 107, expressed	1.0576	0.2788	0.1138	0.3332	No	1.0485	0.337	0.0039	0.851	No	No
101225_at	D5Ertd102e	DNA segment, Chr 5, ERATO Doi 102, expressed	0.9303	0.3357	0.5963	0.3004	No	0.8958	0.0617	0.0289	0.047	No	No
101226_at	Sh3rf1	SH3 domain containing ring finger 1	0.9727	0.694	0.0185	0.7418	No	1.0718	0.0948	0.02	0.372	No	No
101227_at	---	Transcribed locus	1.0506	0.2698	0.7622	0.1066	No	1.1351	0.0171	0.0034	0.0095	No	No
101228_at	Bub3	budding uninhibited by benzimidazoles 3 homolog (S. cerevisiae)	1.0154	0.6406	0.0007	0.319	No	1.0498	0.0621	0.0002	0.0128	No	No
101229_at	C78228	expressed sequence C78228	1.0077	0.6019	0.0066	0.0063	No	1.0127	0.727	0.0044	0.519	No	No
101230_at	Eif4enif1	eukaryotic translation initiation factor 4E nuclear import factor 1	1.0092	0.8967	0.9024	0.3333	No	1.0302	0.661	0.205	0.0831	No	No
101231_at	Xrn1	5'-3' exoribonuclease 1	1.0716	0.2026	0.1022	0.136	No	1.0927	0.0679	0.0035	0.009	No	No
101232_at	C78532	expressed sequence C78532	0.9889	0.7439	0.9872	0.9299	No	0.8684	0.449	0.689	0.409	No	No
101233_at	---	---	0.9953	0.93	0.0198	0.5666	No	0.831	0.163	0.0307	0.123	No	No
101234_at	C78704	expressed sequence C78704	1.0132	0.8147	0.0841	0.8829	No	0.9399	0.381	0.0725	0.531	No	No
101235_f_at	AI450241	Expressed sequence AI450241	1.2084	0.0288	0.3649	0.0929	No	1.1108	0.0259	0.495	0.0137	No	No
101254_at	Ran /// LOC625094 /// LOC671251	RAN, member RAS oncogene family /// similar to RAN, member RAS oncogene family /// similar to RAN, member RAS oncogene family	1.1757	0.0013	0.0132	0.1573	Yes	1.1309	0.014	0.0064	0.224	No	Yes
101255_at	Ubb	ubiquitin B	1.0303	0.1042	0.1083	0.1619	No	1.0283	0.0728	0.0287	0.0239	No	No
101276_at	LOC630270 /// LOC631064 /// LOC668423 /// LOC671238 /// LOC673482 /// LOC674061 /// LOC674072 /// LOC674139 /// LOC674148	similar to Ig heavy chain V region 441 precursor /// similar to Ig heavy chain V region 441 precursor /// similar to Ig heavy chain V region 441 precursor /// similar to Ig heavy chain V region 441 precursor /// similar to Ig heavy chain V region 441 precursor /// similar to Ig heavy chain V region 441 precursor /// similar to Ig heavy chain V region 441 precursor /// similar to Ig heavy chain V region 441 precursor /// similar to Ig heavy chain V region 441 precursor	0.9762	0.5177	0.0798	0.2468	No	1.2613	0.139	0.0012	0.14	No	No
101277_at	D17H6S56E-3	DNA segment, Chr 17, human D6S56E 3	0.9906	0.8466	0.2012	0.196	No	0.9283	0.211	0.689	0.196	No	No
101278_at	Artn	artemin	1.0126	0.4032	0.0015	0.0233	No	1.0031	0.969	0.111	0.349	No	No
101279_f_at	Gm1066	gene model 1066, (NCBI)	0.9738	0.4229	0.0019	0.8403	No	1.0001	0.846	0.0056	0.228	No	No
101280_at	Olfr2	olfactory receptor 2	1.0564	0.2387	0.7092	0.1076	No	1.0347	0.193	0.0069	0.0723	No	No
101281_at	Olfr49	olfactory receptor 49	1.0322	0.7264	0.6918	0.4609	No	1.0303	0.789	0.743	0.109	No	No
101282_at	Gpr44	G protein-coupled receptor 44	0.9864	0.777	0.0208	0.676	No	1.0639	0.531	0.167	0.186	No	No
101286_at	Gja8	gap junction membrane channel protein alpha 8	1.0035	0.9692	0.6997	0.7402	No	0.9771	0.766	0.49	0.481	No	No
101287_s_at	Cyp2d10 /// LOC545123	cytochrome P450, family 2, subfamily d, polypeptide 10 /// similar to Cytochrome P450 2D11 (CYPIID11) (P450-16-alpha) (CC) (Testosterone 16-alpha hydroxylase)	1.0685	0.1381	0.1316	0.1162	No	1.0611	0.206	0.0959	0.2	No	No
101288_at	Fv4	Friend virus susceptibility 4	1.0776	0.3502	0.4321	0.1987	No	1.1329	0.089	0.135	0.0222	No	No
101289_f_at	Klk1b22	kallikrein 1-related peptidase b22	0.929	0.2348	0.0245	0.1057	No	1.0865	0.212	0.25	0.95	No	No
101290_at	Gnrh1	gonadotropin releasing hormone 1	0.959	0.5172	0.014	0.246	No	1.0856	0.545	0.0149	0.0728	No	No

101291_at	LOC384931	similar to Spermidine synthase (Putrescine aminopropyltransferase) (SPDSY)	1.0037	0.9428	0.0738	0.5757	No	1.131	0.0761	0.7	0.769	No	No
101292_f_at	---	---	1.0573	0.0844	0.0354	0.9489	No	0.9228	0.0263	0.141	0.0361	No	No
101293_at	G6pd2	glucose-6-phosphate dehydrogenase 2	0.9059	0.2206	0.0395	0.1354	No	0.9506	0.134	0.0327	0.122	No	No
101294_g_at	G6pd2 /// G6pdx	glucose-6-phosphate dehydrogenase 2 /// glucose-6-phosphate dehydrogenase X-linked	0.9875	0.7805	0.8981	0.3455	No	1.1002	0.0515	0.276	0.904	No	No
101295_s_at	Clns1a /// LOC241715	chloride channel, nucleotide-sensitive, 1A /// hypothetical LOC241715	1.0476	0.0061	0.0009	0.1995	Yes	1.1121	0.0037	0.0005	0.0086	No	No
101296_at	LOC236874 /// LOC628991 /// LOC635720	similar to odorant binding protein Ia /// similar to odorant binding protein Ia /// similar to odorant binding protein Ia	0.9656	0.6601	0.4415	0.6042	No	0.891	0.511	0.7	0.696	No	No
101297_at	Ptprc	protein tyrosine phosphatase, receptor type, C	0.9219	0.448	0.1029	0.3242	No	1.0564	0.935	0.255	0.976	No	No
101298_g_at	Ptprc	protein tyrosine phosphatase, receptor type, C	0.8589	0.1352	0.3656	0.248	No	1.0131	0.741	0.0001	0.0815	No	No
101299_at	H2-M1	histocompatibility 2, M region locus 1	0.9841	0.8177	0.0852	0.4585	No	0.8625	0.237	0.0046	0.321	No	No
101300_at	H2-M2	histocompatibility 2, M region locus 2	1.0586	0.269	0.2934	0.0269	No	1.0465	0.229	0.0049	0.0826	No	No
101301_at	LOC384845	similar to Uridine-cytidine kinase 1 (UCK 1) (Uridine monophosphokinase 1) (Cytidine monophosphokinase 1)	0.9787	0.4453	0.0468	0.141	No	1.0048	0.998	0.0041	0.746	No	No
101302_at	Kcnd1	potassium voltage-gated channel, Shal-related family, member 1	0.9371	0.2353	0.338	0.418	No	0.9666	0.0933	0.0002	0.342	No	No
101303_at	Olfr15	olfactory receptor 15	0.9811	0.7151	0.0317	0.5682	No	1.0854	0.116	0.0068	0.26	No	No
101305_at	Pou3f3	POU domain, class 3, transcription factor 3	0.8589	0.0373	0.0024	0.0742	No	0.9012	0.129	0.0038	0.141	No	No
101306_f_at	---	---	1.062	0.4096	0.0054	0.5822	No	1.0413	0.544	0.0024	0.875	No	No
101307_at	BC060945	cDNA sequence BC060945	1.018	0.4571	0.0488	0.1761	No	0.9972	0.861	0.205	0.0843	No	No
101308_at	Kcnb1	potassium voltage gated channel, Shab-related subfamily, member 1	0.9204	0.1617	0.0861	0.4021	No	0.9551	0.476	0.166	0.929	No	No
101309_at	Sstr3	somatostatin receptor 3	1.0119	0.6942	0.014	0.112	No	1.0643	0.274	0.424	0.43	No	No
101310_at	Tal2	T-cell acute lymphocytic leukemia 2	0.987	0.8818	0.1147	0.7496	No	1.0121	0.833	0.24	0.791	No	No
101311_at	Tcrb-V13	T-cell receptor beta, variable 13	1.1075	0.1176	0.0106	0.8655	No	1.0925	0.232	0.0213	0.547	No	No
101312_at	Grin2b	glutamate receptor, ionotropic, NMDA2B (epsilon 2)	0.9701	0.5578	0.2228	0.155	No	1.0195	0.566	0.227	0.0527	No	No
101313_r_at	---	---	1.0591	0.434	0.0187	0.2772	No	1.0532	0.571	0.0411	0.481	No	No
101314_at	---	---	1.0093	0.8756	0.0266	0.9724	No	1.0092	0.919	0.0267	0.0916	No	No
101315_at	Tuba-rs1	tubulin alpha, related sequence 1	0.9353	0.191	0.8164	0.7459	No	0.9475	0.0423	0.0314	0.432	No	No
101316_at	Wnt7a	wingless-related MMTV integration site 7A	1.0735	0.4288	0.0352	0.4933	No	1.2261	0.0296	0.0053	0.224	No	No
101317_f_at	Ifna1 /// Ifna11 /// Ifna2 /// Ifnab	interferon alpha family, gene 1 /// interferon alpha family, gene 11 /// interferon alpha family, gene 2 /// interferon alpha family, gene B	1.1107	0.0669	0.0093	0.1565	No	1.0503	0.396	0.0013	0.0166	No	No
101318_at	Sstr1	somatostatin receptor 1	1.0293	0.3104	0.2555	0.8675	No	1.0556	0.43	0.428	0.971	No	No
101319_f_at	Igk-V8	Immunoglobulin kappa chain complex /// Immunoglobulin kappa chain variable 8 (V8)	0.9909	0.9097	0.6508	0.3575	No	1.0831	0.274	0.162	0.187	No	No
101320_f_at	LOC380823 /// LOC382696 /// VH186.2-D-J-C mu /// LOC619833 /// LOC629941 /// LOC631531 /// LOC633774 /// LOC636044 /// LOC636134 /// LOC636244 /// LOC637785 /// LOC637794 /// LOC640207 /// LOC640979 ///	similar to Ig heavy chain V region B1-8/186-2 precursor /// similar to Ig heavy chain V region 3 precursor /// similar to monoclonal antibody heavy chain /// similar to Ig heavy chain V region 102 precursor /// similar to Ig heavy chain V region 3 precursor /// similar to Ig heavy chain V region VH558 B4 precursor /// similar to Ig heavy chain V region 23 precursor /// similar to Ig heavy chain V region 23 precursor /// similar to Ig heavy chain V region 3 precursor /// similar to Ig heavy chain V region 102 precursor /// similar to Ig heavy chain V region 3 precursor /// similar to Ig heavy chain V region 3 precursor /// similar to Ig heavy chain V region 102 precursor /// similar to Ig heavy chain V region 23 precursor /// similar to Ig heavy chain V region 3 precursor /// similar to Ig heavy chain V region 3 precursor /// similar to Ig heavy chain V region 145 precursor /// similar to Ig heavy chain V region 102 precursor /// similar to Ig heavy chain V region 3 precursor	0.9841	0.8385	0.1568	0.5203	No	0.9462	0.153	0.0009	0.805	No	No
101321_r_at	LOC380823 /// LOC382696 /// VH186.2-D-J-C mu /// LOC619833 /// LOC629941 /// LOC631531 /// LOC633774 /// LOC636134 /// LOC636244 /// LOC637785 /// LOC637794 /// LOC640207 /// LOC640979 /// LOC641003 ///	similar to Ig heavy chain V region B1-8/186-2 precursor /// similar to Ig heavy chain V region 3 precursor /// similar to monoclonal antibody heavy chain /// similar to Ig heavy chain V region 102 precursor /// similar to Ig heavy chain V region 3 precursor /// similar to Ig heavy chain V region VH558 B4 precursor /// similar to Ig heavy chain V region 23 precursor /// similar to Ig heavy chain V region 3 precursor /// similar to Ig heavy chain V region 102 precursor /// similar to Ig heavy chain V region 3 precursor /// similar to Ig heavy chain V region 3 precursor /// similar to Ig heavy chain V region 102 precursor /// similar to Ig heavy chain V region 23 precursor /// similar to Ig heavy chain V region 3 precursor /// similar to Ig heavy chain V region 3 precursor /// similar to Ig heavy chain V region 102 precursor /// similar to Ig heavy chain V region 3 precursor	1.0073	0.9403	0.0166	0.8037	No	0.9015	0.31	0.0011	0.485	No	No
101324_r_at	---	---	1.118	0.29	0.6342	0.4595	No	1.2423	0.0094	0.258	0.0024	No	No
101325_r_at	Fbs1	fibrosin 1	0.9333	0.4185	0.0716	0.6661	No	0.9202	0.184	0.932	0.128	No	No
101326_at	LOC432709	similar to Ig heavy chain V-II region SESS precursor	0.9552	0.6038	0.4709	0.9393	No	0.9625	0.611	0.0508	0.567	No	No
101327_at	Lcp1	lymphocyte cytosolic protein 1	1.0628	0.1057	0.1797	0.1046	No	1.0558	0.238	0.0016	0.097	No	No
101328_at	Pla2g5	phospholipase A2, group V	0.8694	0.0008	0.002	0.0041	No	0.9614	0.526	0.0305	0.767	No	No
101329_f_at	Igk-V32	Immunoglobulin kappa chain variable 32 (V32)	0.9611	0.4276	0.2412	0.2145	No	1.0127	0.803	0.383	0.129	No	No
101330_f_at	Igk-V32	immunoglobulin kappa chain variable 32 (V32)	0.9892	0.9174	0.344	0.8558	No	1.0777	0.272	0.256	0.723	No	No

101331_f_at	LOC637260 /// Igk-V19-13 /// LOC669070 /// LOC673777 /// LOC676175	similar to Ig kappa chain V-IV region B17 precursor /// immunoglobulin kappa chain variable 19 (V19)-13 /// similar to Ig kappa chain V-V region MPC11 precursor /// similar to Ig kappa chain V-IV region precursor /// similar to Ig kappa chain V-V region MPC11 precursor	0.9784	0.6974	0.3452	0.9423	No	0.8963	0.102	0.132	0.0548	No	No
101332_at	---	Immunoglobulin kappa chain complex	1.0882	0.0807	0.0293	0.661	No	1.1336	0.0744	0.0007	0.073	No	No
101333_at	Galnt4	UDP-N-acetyl-alpha-D-galactosamine:polypeptide N-acetylgalactosaminyltransferase 4	1.0178	0.8074	0.4694	0.8335	No	0.9694	0.673	0.967	0.429	No	No
101334_at	Nkx2-3	NK2 transcription factor related, locus 3 (Drosophila)	1.0428	0.35	0.0079	0.1218	No	1.0278	0.711	0.0029	0.142	No	No
101335_at	Cdk5r2	cyclin-dependent kinase 5, regulatory subunit 2 (p39)	1.0013	0.9808	0.008	0.377	No	1.0355	0.447	0.006	0.178	No	No
101336_at	LOC194360	hypothetical LOC194360	1.0374	0.5007	0.1657	0.6722	No	1.0161	0.687	0.713	0.574	No	No
101337_at	Moxd2	monooxygenase, DBH-like 2	1.0387	0.1348	0.0007	0.1276	No	1.0124	0.899	0.461	0.745	No	No
101338_f_at	LOC436522	trypsinogen 10	0.9498	0.2217	0.3524	0.0648	No	1.0066	0.898	0.6	0.556	No	No
101339_at	Prss3 /// Try4 /// Prss1 /// 1810049H19 Rik /// LOC436522 /// LOC436523 /// LOC673571	protease, serine, 3 /// trypsin 4 /// protease, serine, 1 (trypsin 1) /// RIKEN cDNA 1810049H19 gene /// trypsinogen 10 /// trypsinogen 12 /// similar to Anionic trypsin II precursor (Pretrypsinogen II)	1.0373	0.0799	0.0071	0.5854	No	1.0072	0.916	0.0034	0.167	No	No
101340_at	---	---	0.9831	0.6401	0.7751	0.433	No	0.9552	0.426	0.0355	0.13	No	No
101341_at	H2-M9	histocompatibility 2, M region locus 9	1.0383	0.3348	0.5688	0.0119	No	1.0466	0.214	0.326	0.0228	No	No
101342_at	Notch2	Notch gene homolog 2 (Drosophila)	0.9497	0.4386	0.0591	0.7442	No	0.9368	0.257	0.897	0.177	No	No
101343_at	Sh2d1b1	SH2 domain protein 1B1	1.0008	0.977	0.0271	0.103	No	1.0231	0.904	0.258	0.0231	No	No
101344_at	Cckbr	cholecystokinin B receptor	1.0177	0.6888	0.0641	0.1469	No	0.98	0.62	0.0653	0.531	No	No
101345_at	Egfl8	EGF-like domain 8	1.0267	0.3099	0.0956	0.2256	No	1.007	0.354	0.0228	0.179	No	No
101346_at	Igh-1a	Immunoglobulin heavy chain 1a (serum IgG2a)	1.0308	0.6615	0.0582	0.3454	No	1.013	0.594	0.0054	0.207	No	No
101347_at	Igk-V1	Immunoglobulin kappa chain variable 1 (V1)	1.0367	0.0165	0.0005	0.0028	No	1.0034	0.967	0.711	0.868	No	No
101348_at	Igkv1-135	Immunoglobulin light chain variable region	1.0272	0.5882	0.3665	0.1489	No	1.0705	0.176	0.225	0.0893	No	No
101349_at	Plk1	polo-like kinase 1 (Drosophila)	0.9417	0.4224	0.0763	0.1624	No	0.9468	0.64	0.916	0.224	No	No
101350_g_at	Plk1	polo-like kinase 1 (Drosophila)	0.9863	0.7813	0.0097	0.5861	No	1.0579	0.131	0.01	0.0305	No	No
101351_at	LOC637873 /// LOC672790	similar to putative pheromone receptor (Go-VN5) /// similar to putative pheromone receptor (Go-VN5)	1.1432	0.0092	0.0814	0.4031	Yes	1.2704	0.0242	0.199	0.562	No	Yes
101352_g_at	Gprc2a-rs1 /// LOC333201 /// LOC628403 /// LOC628444 /// LOC628490 /// LOC637852 /// LOC637873 /// LOC637898 /// LOC637992 /// LOC672780 /// LOC672784 /// LOC672790	G protein-coupled receptor, family C, group 2, member A, related sequence 1 /// similar to putative pheromone receptor (Go-VN5) /// similar to putative pheromone receptor (Go-VN5) /// similar to putative pheromone receptor (Go-VN5) /// similar to putative pheromone receptor (Go-VN5) /// similar to putative pheromone receptor (Go-VN5) /// similar to putative pheromone receptor (Go-VN5) /// similar to putative pheromone receptor (Go-VN5) /// similar to putative pheromone receptor (Go-VN5) /// similar to putative pheromone receptor (Go-VN5) /// similar to putative pheromone receptor (Go-VN5)	1.0428	0.3081	0.125	0.0297	No	1.0656	0.0549	0.0385	0.346	No	No
101353_at	LOC333201 /// LOC637898	similar to putative pheromone receptor (Go-VN5) /// similar to putative pheromone receptor (Go-VN5)	1.0127	0.7833	0.0132	0.236	No	1.1255	0.408	0.544	0.0783	No	No
101354_at	Slc18a3	solute carrier family 18 (vesicular monoamine), member 3	0.9651	0.6564	0.7918	0.4207	No	0.9877	0.721	0.231	0.367	No	No
101355_at	Ascl2	achaete-scute complex homolog-like 2 (Drosophila)	0.9705	0.6041	0.319	0.9692	No	1.0326	0.789	0.0035	0.14	No	No
101356_at	Tk2	thymidine kinase 2, mitochondrial	1.0398	0.2403	0.0009	0.2637	No	1.0356	0.146	0.0002	0.0465	No	No
101357_at	Ap2a1	adaptor protein complex AP-2, alpha 1 subunit	1.007	0.8602	0.0171	0.4781	No	1.0495	0.187	0.0055	0.0374	No	No
101358_at	Plcb3	phospholipase C, beta 3	0.9662	0.1753	0.8787	0.052	No	0.9377	0.231	0.0732	0.173	No	No
101359_at	Lamb2	laminin, beta 2	0.8922	0.0141	0.132	0.7302	No	0.9159	0.204	0.435	0.986	No	No
101362_at	Mapk9	mitogen activated protein kinase 9	0.9857	0.7703	0.0371	0.7319	No	0.9804	0.959	0.0403	0.321	No	No
101363_at	Adora2a	adenosine A2a receptor	0.966	0.3724	0.3248	0.3506	No	0.9864	0.737	0.641	0.717	No	No
101364_at	Adora2a	adenosine A2a receptor	1.0044	0.7617	0.4061	0.0767	No	1.0843	0.07	0.0289	0.403	No	No
101366_f_at	Nvl	nuclear VCP-like	0.9399	0.2303	0.0177	0.0514	No	1.0361	0.783	0.0741	0.344	No	No
101367_at	Dctn1	dynactin 1	1.0059	0.9083	0.0193	0.4222	No	1.0491	0.172	0.0063	0.797	No	No
101368_at	Rhox5	reproductive homeobox 5	1.0146	0.6352	0.0011	0.0536	No	1.1435	0.0531	0.0578	0.0375	No	No
101369_at	Zfp213	zinc finger protein 213	0.9803	0.0655	0.0001	0.0018	No	1.0288	0.415	0.062	0.46	No	No
101370_at	Kpna1	karyopherin (importin) alpha 1	1.0668	0.3124	0.1903	0.2513	No	1.0296	0.714	0.154	0.432	No	No
101371_at	Cpsf4	cleavage and polyadenylation specific factor 4	1.0191	0.575	0.0461	0.0695	No	1.0378	0.293	0.006	0.149	No	No
101372_at	Trip13	thyroid hormone receptor interactor 13	1.1218	0.0066	0.2318	0.0227	Yes	1.1333	0.0008	0.0018	0.0035	No	No
101374_at	Krtap6-1	keratin associated protein 6-1	1.0351	0.5822	0.0797	0.0409	No	1.0977	0.241	0.0177	0.213	No	No
101375_at	Krtap6-1	keratin associated protein 6-1	0.9725	0.4438	0.1034	0.0388	No	0.968	0.56	0.742	0.375	No	No
101376_at	Krtap6-1	keratin associated protein 6-1	1.0572	0.1704	0.8392	0.5145	No	1.0335	0.542	0.557	0.433	No	No
101377_at	---	---	0.9801	0.4163	0.0065	0.047	No	0.9892	0.909	0.0441	0.237	No	No
101380_at	Nudt14	nudix (nucleoside diphosphate linked moiety X)-type motif 14	0.9889	0.762	0.0664	0.5773	No	0.9962	0.809	0.0402	0.105	No	No
101381_at	Rabep1	rabaptin, RAB GTPase binding effector protein 1	1.0206	0.7248	0.0518	0.1335	No	0.9569	0.217	0.0077	0.233	No	No
101382_at	Pbx2	pre B-cell leukemia transcription factor 2	1.001	0.9859	0.7057	0.9152	No	1.0842	0.0905	0.899	0.106	No	No
101383_at	Tnnt1	troponin T1, skeletal, slow	0.9569	0.4967	0.8628	0.1111	No	0.9035	0.0892	0.0005	0.941	No	No
101384_at	Degs2	degenerative spermatocyte homolog 2 (Drosophila), lipid desaturase	0.9553	0.3563	0.0199	0.5592	No	0.9856	0.777	0.137	0.411	No	No
101385_at	Mbd3	methyl-CpG binding domain protein 3	1.025	0.1951	0.0191	0.0248	No	1.0186	0.0389	0.0004	0.0004	No	No
101386_at	1810030J14 Rik /// LOC630754	RIKEN cDNA 1810030J14 gene /// similar to Serum amyloid P-component precursor (SAP)	1.0672	0.1983	0.663	0.6358	No	1.0416	0.436	0.0419	0.138	No	No
101387_at	Acy1 /// LOC634623	aminoacylase 1 /// similar to aminoacylase 1	0.9958	0.9243	0.1969	0.7378	No	1.031	0.359	0.0925	0.305	No	No
101388_at	Pgk2	phosphoglycerate kinase 2	1.0785	0.1592	0.0766	0.2567	No	1.0784	0.482	0.922	0.814	No	No
101389_at	Scarb2	scavenger receptor class B, member 2	0.952	0.313	0.173	0.0951	No	0.9156	0.0169	0.0098	0.0146	No	No
101390_at	Muc3 /// LOC666339 /// LOC677034	mucin 3, intestinal /// similar to mucin 17 /// similar to mucin 17	1.0205	0.5356	0.736	0.4911	No	1.0943	0.183	0.0344	0.702	No	No
101392_at	Chmp7	CHMP family, member 7	0.9931	0.7438	0.0024	0.0023	No	1.008	0.739	0.148	0.0013	No	No
101393_at	Anxa3	annexin A3	1.0791	0.3331	0.1776	0.9511	No	0.9685	0.753	0.204	0.206	No	No
101394_at	Sgcg	sarcoglycan, gamma (dystrophin-associated glycoprotein)	0.8788	0.1303	0.3124	0.1227	No	0.9134	0.305	0.277	0.206	No	No
101395_at	Purb	Purine rich element binding protein B	1.0317	0.3947	0.0062	0.95	No	1.1412	0.0188	0.1	0.0754	No	No
101396_at	Tcf2	transcription factor 2	0.943	0.3283	0.0933	0.8202	No	1.1108	0.279	0.0169	0.584	No	No
101397_at	Slc2a8	solute carrier family 2, (facilitated glucose transporter), member 8	1.0117	0.8067	0.1221	0.9916	No	1.021	0.732	0.274	0.72	No	No
101398_at	Stxbp2	syntaxin binding protein 2	0.9673	0.1577	0.0024	0.104	No	0.9706	0.224	0.0425	0.339	No	No
101399_at	Hspg2	perlecan (heparan sulfate proteoglycan 2)	0.8967	0.0238	0.8207	0.3827	No	0.9957	0.863	0.818	0.0169	No	No
101401_at	Bet1	blocked early in transport 1 homolog (S. cerevisiae)	0.9944	0.9306	0.8937	0.6631	No	0.9566	0.32	0.0482	0.46	No	No

101402_at	Thrap4	thyroid hormone receptor associated protein 4	0.9899	0.4503	0.0035	0.0413	No	0.9919	0.856	0.0003	0.0211	No	No
101403_at	Ccl25	chemokine (C-C motif) ligand 25	0.9989	0.9856	0.0044	0.3877	No	0.9236	0.527	0.0111	0.119	No	No
101404_at	2310061I09 Rik	RIKEN cDNA 2310061I09 gene	0.9993	0.9906	0.0662	0.2469	No	1.0079	0.899	0.129	0.0102	No	No
101405_at	Smarcd1	SWI/SNF related, matrix associated, actin dependent regulator of chromatin, subfamily d, member 1	1.0096	0.814	0.0319	0.0669	No	1.0406	0.273	0.0865	0.112	No	No
101406_at	Sult2b1	sulfotransferase family, cytosolic, 2B, member 1	0.9138	0.0873	0.1738	0.2087	No	0.9316	0.36	0.368	0.915	No	No
101407_at	Fxn	frataxin	1.0941	0.3227	0.423	0.2665	No	1.1271	0.103	0.273	0.381	No	No
101408_at	Gamt	guanidinoacetate methyltransferase	1.0234	0.4243	0.0622	0.2283	No	0.98	0.581	0.0067	0.592	No	No
101409_at	Lgtn	ligatin	0.9004	0.002	0.0002	0.0006	No	0.8409	0.0028	0.0634	0.803	Yes	No
101410_at	Cldn4	claudin 4	1.0038	0.9177	0.4654	0.0685	No	0.9649	0.285	0.344	0.087	No	No
101412_at	Sh3gl3	SH3-domain GRB2-like 3	1.0226	0.4871	0.0219	0.2044	No	1.0139	0.81	0.0256	0.917	No	No
101413_at	Edd1	E3 ubiquitin protein ligase, HECT domain containing, 1	0.944	0.5735	0.0138	0.212	No	1.0681	0.568	0.0197	0.549	No	No
101414_at	Lmnb2	lamin B2	1.0947	0.0957	0.3029	0.0411	No	1.1648	0.0332	0.111	0.0073	No	No
101415_i_at	Bat4	HLA-B associated transcript 4	0.9583	0.3144	0.2652	0.5166	No	0.9157	0.478	0.135	0.112	No	No
101416_f_at	Bat4	HLA-B associated transcript 4	0.9822	0.4961	0.119	0.8465	No	0.9376	0.107	0.0648	0.419	No	No
101417_at	Dmrtb1	DMRT-like family B with proline-rich C-terminal, 1	0.7779	0.0537	0.1552	0.0956	No	1.0179	0.67	0.0017	0.97	No	No
101419_at	Tubb4	tubulin, beta 4	1.0937	0.0319	0.2884	0.6421	No	0.9307	0.034	0.001	0.513	No	No
101420_at	Slc32a1	solute carrier family 32 (GABA vesicular transporter), member 1	0.9783	0.5749	0.2766	0.8948	No	0.9426	0.377	0.0492	0.367	No	No
101421_at	Rnf5	ring finger protein 5	0.9823	0.4704	0.062	0.0353	No	1.0266	0.613	0.67	0.615	No	No
101422_at	Fnbp4	formin binding protein 4	1.009	0.8544	0.0169	0.9455	No	1.0227	0.704	0.0134	0.687	No	No
101423_at	Ddx46	DEAD (Asp-Glu-Ala-Asp) box polypeptide 46	1.0184	0.5833	0.0005	0.0479	No	0.9978	0.914	0.0043	0.461	No	No
101424_at	Nmi	N-myc (and STAT) interactor	1.0052	0.9429	0.5731	0.2525	No	0.9928	0.959	0.99	0.935	No	No
101425_at	Gss	glutathione synthetase	0.9779	0.1088	0.0012	0.0524	No	0.9875	0.639	0.0107	0.414	No	No
101426_at	Cerk	ceramide kinase	1.0263	0.625	0.3032	0.2086	No	1.0161	0.519	0.349	0.0502	No	No
101428_at	Ace	angiotensin I converting enzyme (peptidyl-dipeptidase A) 1	1.0681	0.034	0.1465	0.1112	No	1.1191	0.0315	0.561	0.0099	No	No
101429_at	Ddit3	DNA-damage inducible transcript 3	0.9204	0.2864	0.2839	0.3485	No	0.9576	0.19	0.0026	0.123	No	No
101430_at	LOC672274	similar to Transcription factor SOX-4	0.9646	0.2386	0.0198	0.2826	No	0.9784	0.748	0.0001	0.685	No	No
101431_at	Rdh5	retinol dehydrogenase 5	1.0262	0.475	0.0227	0.3446	No	1.0125	0.543	0.0135	0.0957	No	No
101432_at	Trp53i13	tumor protein p53 inducible protein 13	0.9826	0.2424	0	0.0062	No	1.0482	0.0837	0.0002	0.435	No	No
101433_at	Mr1	major histocompatibility complex, class I-related	0.939	0.3071	0.103	0.6828	No	0.9235	0.0358	0.0105	0.862	No	No
101435_at	Akap2	A kinase (PRKA) anchor protein 2	0.9907	0.8852	0.0614	0.5306	No	0.9197	0.0511	0.0018	0.132	No	No
101436_at	Cxcl9	chemokine (C-X-C motif) ligand 9	1.0727	0.033	0.9913	0.2714	No	0.9235	0.488	0.126	0.944	No	No
101437_at	Slk	STE20-like kinase (yeast)	0.9502	0.3195	0.2028	0.6687	No	0.9384	0.219	0.0009	0.572	No	No
101439_at	Arl6	ADP-ribosylation factor-like 6	0.9985	0.9855	0.6268	0.9736	No	1.0089	0.794	0.724	0.56	No	No
101440_at	2900006F19 Rik	RIKEN cDNA 2900006F19 gene	0.9854	0.5646	0.0046	0.3801	No	1.0025	0.946	0.368	0.384	No	No
101441_i_at	Itpr2	inositol 1,4,5-triphosphate receptor 2	0.9604	0.5013	0.1212	0.1687	No	0.9328	0.368	0.354	0.13	No	No
101442_f_at	Itpr2	inositol 1,4,5-triphosphate receptor 2	0.9087	0.081	0.0017	0.3147	No	0.9623	0.125	0.0001	0.132	No	No
101443_at	Plekhh1	pleckstrin homology domain containing, family H (with MyTH4 domain) member 1	0.9953	0.9425	0.4385	0.765	No	1.0404	0.637	0.27	0.84	No	No
101444_at	Thumpd3	THUMP domain containing 3	1.015	0.6842	0.2593	0.904	No	1.0218	0.662	0.256	0.8	No	No
101445_at	Dnmt1	DNA methyltransferase (cytosine-5) 1	1.0037	0.8497	0.4306	0.0152	No	1.0286	0.455	0.198	0.123	No	No
101446_at	Tpd52l1	tumor protein D52-like 1	1.0073	0.8939	0.0091	0.0275	No	0.9361	0.021	0.0001	0.427	No	No
101447_at	Apc	adenomatosis polyposis coli	0.8863	0.0648	0.707	0.7736	No	0.9078	0.0869	0.33	0.178	No	No
101448_at	Hgs	HGF-regulated tyrosine kinase substrate	1.0019	0.748	0.0007	0.0658	No	1.0138	0.393	0.0007	0.705	No	No
101449_at	Trim41	tripartite motif-containing 41	0.935	0.0584	0.0019	0.44	No	1.0137	0.65	0.629	0.851	No	No
101450_at	Csf1	colony stimulating factor 1 (macrophage)	1.0078	0.7157	0.1472	0.0174	No	1	0.996	0.173	0.644	No	No
101451_at	Peg3	paternally expressed 3	0.9718	0.5865	0	0.7014	No	0.9795	0.348	0	0.393	No	No
101453_at	Mia1	melanoma inhibitory activity 1	1.0322	0.4211	0.0032	0.5699	No	0.9917	0.862	0.0074	0.665	No	No
101455_at	Umps	uridine monophosphate synthetase	0.9636	0.5789	0.4519	0.821	No	0.9006	0.0538	0.195	0.123	No	No
101456_at	Zfp106	zinc finger protein 106	0.9822	0.658	0.6457	0.8733	No	1.0405	0.559	0.264	0.755	No	No
101457_at	Jak2	Janus kinase 2	0.9009	0.0252	0.0174	0.7969	No	0.9127	0.189	0.648	0.503	No	No
101458_at	Wee1	wee 1 homolog (S. pombe)	1.1221	0.0372	0.0002	0.0696	No	1.0767	0.3	0.0041	0.52	No	No
101459_at	Chd1	chromodomain helicase DNA binding protein 1	1.0736	0.0173	0	0.4352	No	1.0631	0.0679	0.0001	0.188	No	No
101461_f_at	Pja1	praja1, RING-H2 motif containing	1.0563	0.2411	0.0027	0.4598	No	1.0767	0.278	0.0071	0.271	No	No
101462_r_at	Pja1	praja1, RING-H2 motif containing	1.0954	0.0139	0.0002	0.9417	No	1.1804	0.0376	0.0441	0.789	No	No
101463_at	Apoc4	apolipoprotein C-IV	1.056	0.0082	0.0017	0.0039	No	0.9791	0.501	0.53	1	No	No
101464_at	Timp1	tissue inhibitor of metalloproteinase 1	1.0152	0.7218	0.0348	0.2639	No	1.0443	0.255	0.0092	0.128	No	No
101465_at	Stat1	signal transducer and activator of transcription 1	0.8923	0.1506	0.5589	0.1836	No	1.0298	0.649	0.328	0.0404	No	No
101466_at	Pigf	phosphatidylinositol glycan, class F	1.0134	0.4355	0.0638	0.5383	No	0.9499	0.0397	0.0013	0.0232	No	No
101467_at	S100b	S100 protein, beta polypeptide, neural	1.0061	0.9	0.0011	0.9149	No	1.0094	0.723	0.0011	0.362	No	No
101468_at	Cfp	complement factor properdin	0.8696	0.0194	0.0093	0.4177	No	0.8113	0.0639	0.522	0.0948	No	No
101469_at	Nedd9	neural precursor cell expressed, developmentally down-regulated gene 9	0.8367	0.0045	0.1363	0.3517	Yes	0.8705	0.0128	0.212	0.0706	No	Yes
101470_at	Wdr78	WD repeat domain 78	1.0287	0.1723	0.0034	0.0035	No	1.0036	0.88	0.319	0.0146	No	No
101471_at	Pklr	pyruvate kinase liver and red blood cell	0.9268	0.3226	0.3315	0.7788	No	1.1083	0.551	0.123	0.412	No	No
101472_s_at	Pklr	pyruvate kinase liver and red blood cell	0.953	0.3695	0.3231	0.5689	No	1.079	0.316	0.723	0.178	No	No
101473_at	Nnmt	nicotinamide N-methyltransferase	0.9692	0.306	0.0192	0.7218	No	1.0361	0.323	0.0054	0.296	No	No
101474_at	Mbl1	mannose binding lectin (A)	0.9926	0.8697	0.0074	0.9361	No	1.0416	0.174	0.942	0.527	No	No
101475_at	Bmi1	B lymphoma Mo-MLV insertion region 1	1.028	0.7059	0.0037	0.4258	No	1.0401	0.537	0.0033	0.588	No	No
101476_at	Pabpn1	poly(A) binding protein, nuclear 1	1.0264	0.6959	0.2609	0.2704	No	1.0272	0.712	0.473	0.137	No	No
101480_at	Dnalc4	dynein, axonemal, light chain 4	1.0103	0.7813	0.0017	0.3692	No	1.0739	0.39	0.0119	0.292	No	No
101481_at	Rnf34	ring finger protein 34	1.1127	0.0304	0.3959	0.3981	No	1.0896	0.21	0.0289	0.279	No	No
101482_at	Ppp1cc	protein phosphatase 1, catalytic subunit, gamma isoform	1.0503	0.3371	0.1426	0.7825	No	1.0418	0.501	0.513	0.404	No	No
101483_at	Ccndbp1	cyclin D-type binding-protein 1	0.9192	0.0622	0.1879	0.1499	No	0.9222	0.0167	0.146	0.544	No	No
101484_at	Nbr1	neighbor of Brca1 gene 1	0.8323	0.0117	0.1432	0.0785	No	0.8397	0.0085	0.0289	0.0438	Yes	Yes
101485_at	Ddx48	DEAD (Asp-Glu-Ala-Asp) box polypeptide 48	1.0573	0.2022	0.1361	0.8473	No	1.0484	0.0748	0.0411	0.47	No	No
101486_at	Psmb10	proteasome (prosome, macropain) subunit, beta type 10	1.0404	0.0867	0.0047	0.0241	No	1.0353	0.019	0.0001	0.0022	No	No
101487_f_at	Ly6e	lymphocyte antigen 6 complex, locus E	0.9372	0.0307	0	0.0025	No	0.8992	0.0302	0.0001	0.0026	No	No
101488_r_at	Ly6e	lymphocyte antigen 6 complex, locus E	1.0216	0.7418	0.0009	0.426	No	0.9918	0.597	0	0.053	No	No
101489_at	Amd1	S-adenosylmethionine decarboxylase 1	1.0346	0.6902	0.0237	0.702	No	1.0638	0.222	0.0007	0.728	No	No
101490_at	Hbld2	HESB like domain containing 2	0.973	0.7483	0.0846	0.9253	No	0.9726	0.681	0.945	0.275	No	No
101492_at	Pin1	protein (peptidyl-prolyl cis/trans isomerase) NIMA-interacting 1	1.0319	0.4745	0.0004	0.4189	No	1.0947	0.0356	0.0005	0.215	No	No
101493_at	Afp	Alpha fetoprotein	0.9933	0.8559	0.2787	0.8245	No	1.0984	0.144	0.159	0.487	No	No
101494_at	Afp	alpha fetoprotein	1.0645	0.2881	0.6088	0.0889	No	1.0167	0.833	0.277	0.781	No	No
101495_at	Cd81	CD 81 antigen	0.9811	0.626	0.013	0.4307	No	0.9062	0.111	0.598	0.176	No	No
101498_at	Impa1	inositol (myo)-1(or 4)-monophosphatase 1	1.0416	0.5682	0.0152	0.5171	No	1.0002	0.98	0.0066	0.845	No	No
101499_at	Ilk	integrin linked kinase	0.9426	0.2535	0.0149	0.4782	No	0.9569	0.359	0.0139	0.161	No	No
101500_at	Epb4.1l2	Erythrocyte protein band 4.1-like 2	1.0776	0.2731	0.8264	0.894	No	1.0634	0.475	0.891	0.72	No	No
101501_r_at	Impact	imprinted and ancient	1.0328	0.6581	0.0412	0.3417	No	1.0089	0.882	0.0041	0.146	No	No
101502_at	Tgif	TG interacting factor	0.9656	0.6087	0.8613	0.7835	No	0.9727	0.687	0.27	0.747	No	No
101503_at	Dpysl3	dihydropyrimidinase-like 3	0.9806	0.747	0.0048	0.1368	No	0.9534	0.292	0.0007	0.847	No	No
101505_at	Tbc1d20	TBC1 domain family, member 20	1.0393	0.2097	0.3782	0.181	No	0.9474	0.008	0.0007	0.043	Yes	No
101506_at	Snrpa1	small nuclear ribonucleoprotein polypeptide A'	1.2255	0.0773	0.4795	0.8444	No	1.1625	0.0238	0.053	0.123	No	No
101507_at	Scnm1	sodium channel modifier 1	1.022	0.7395	0.7432	0.8164	No	1.1048	0.107	0.0132	0.308	No	No
101508_at	Khdrbs1	KH domain containing, RNA binding, signal transduction associated 1	0.8443	0.0525	0.4192	0.4918	No	0.9231	0.314	0.428	0.456	No	No
101509_at	Vbp1	von Hippel-Lindau binding protein 1	1.0496	0.3064	0.0082	0.7672	No	1.0029	0.961	0.0152	0.798	No	No
101510_at	Psme1	proteasome (prosome, macropain) 28 subunit, alpha	1.0103	0.8874	0.0602	0.3595	No	1.0743	0.367	0.816	0.994	No	No
101511_at	Ssr4	signal sequence receptor, delta	0.9768	0.862	0.0123	0.7429	No	1.296	0.145	0.0002	0.169	No	No
101513_at	Pfdn5	prefoldin 5	1.0204	0.7205	0.2021	0.929	No	1.037	0.385	0.194	0.0433	No	No
101514_at	Trappc3	trafficking protein particle complex 3	1.0823	0.116	0.5018	0.4403	No	1.1054	0.0114	0.216	0.165	No	No
101515_at	Acox1	acyl-Coenzyme A oxidase 1, palmitoyl	0.8955	0.0312	0.9388	0.8104	No	0.9065	0.007	0.266	0.116	Yes	Yes

101516_at	Cd59a	CD59a antigen	0.8941	0.1121	0.6909	0.1624	No	0.9002	0.0011	0.0569	0.0003	No	No
101517_at	Tex261	testis expressed gene 261	1.0211	0.2741	0.2212	0.0074	No	1.0324	0.585	0.0637	0.0852	No	No
101518_at	Ift20	intraflagellar transport 20 homolog (Chlamydomonas)	0.9658	0.6277	0.2161	0.4559	No	1.005	0.911	0.143	0.269	No	No
101519_at	Srp14	signal recognition particle 14	0.9978	0.9729	0.0393	0.1198	No	1.0651	0.37	0.0866	0.261	No	No
101520_at	Spata6	spermatogenesis associated 6	0.9849	0.7324	0.0006	0.2788	No	1.0045	0.862	0.0009	0.683	No	No
101521_at	Birc5	baculoviral IAP repeat-containing 5	0.9878	0.8958	0.7304	0.7687	No	1.0011	0.938	0.0262	0.448	No	No
101522_at	Tm4sf1	transmembrane 4 superfamily member 1	0.9597	0.5018	0.047	0.1363	No	0.99	0.717	0.0045	0.0172	No	No
101523_at	Hnrpa3 /// LOC545592 /// LOC620454 /// LOC627455 /// LOC627828 /// LOC629025 /// LOC630401 /// LOC630677 /// LOC668131 /// LOC668559 /// LOC670103	heterogeneous nuclear ribonucleoprotein A3 /// similar to heterogeneous nuclear ribonucleoprotein A3 /// similar to heterogeneous nuclear ribonucleoprotein A3 /// similar to heterogeneous nuclear ribonucleoprotein A3 /// similar to heterogeneous nuclear ribonucleoprotein A3 /// similar to heterogeneous nuclear ribonucleoprotein A3 /// similar to heterogeneous nuclear ribonucleoprotein A3 /// hypothetical protein LOC630677 /// similar to heterogeneous nuclear ribonucleoprotein A3 /// similar to heterogeneous nuclear ribonucleoprotein A3 /// similar to heterogeneous nuclear ribonucleoprotein A3	1.1652	0.0924	0.0745	0.4728	No	1.1274	0.191	0.133	0.509	No	No
101524_at	Hnrpa3	heterogeneous nuclear ribonucleoprotein A3	1.1083	0.2784	0.0217	0.0523	No	1.0677	0.522	0.0594	0.157	No	No
101525_at	Ndufb10	NADH dehydrogenase (ubiquinone) 1 beta subcomplex, 10	0.9783	0.4827	0.0017	0.008	No	1.0082	0.637	0.0013	0.158	No	No
101526_at	Msx1	homeo box, msh-like 1	0.9258	0.0504	0.053	0.0117	No	0.9567	0.512	0.0064	0.599	No	No
101527_at	Tcea1	transcription elongation factor A (SII) 1	1.0069	0.9228	0.4892	0.3848	No	1.0448	0.647	0.0566	0.391	No	No
101528_at	Tcea1	transcription elongation factor A (SII) 1	0.9433	0.341	0.0017	0.1833	No	1.0193	0.947	0.0022	0.147	No	No
101529_g_at	Tcea1	transcription elongation factor A (SII) 1	0.9796	0.6577	0.0095	0.1782	No	1.0202	0.817	0.0131	0.748	No	No
101530_at	Eftud2	elongation factor Tu GTP binding domain containing 2	1.0317	0.1195	0.0031	0.637	No	1.0859	0.0708	0.043	0.223	No	No
101531_at	Aldob	aldolase 2, B isoform	0.8364	0.0329	0.846	0.8522	No	1.1521	0.0729	0	0.275	No	No
101532_g_at	Aldob	aldolase 2, B isoform	1.0307	0.4211	0.0644	0.5652	No	0.9973	0.967	0.316	0.727	No	No
101534_at	Tnp2	transition protein 2	0.7964	0.0431	0.823	0.0861	No	0.8259	0.0022	0.0025	0.465	Yes	Yes
101536_at	Ncor1	nuclear receptor co-repressor 1	1.0048	0.8892	0.1419	0.0776	No	0.9683	0.372	0.271	0.211	No	No
101537_at	Es1	esterase 1	0.9404	0.3417	0.0075	0.2316	No	0.9758	0.718	0.0036	0.765	No	No
101538_i_at	Ces3	carboxylesterase 3	0.96	0.5485	0.8984	0.3499	No	0.9085	0.0398	0.0035	0.0955	No	No
101539_f_at	Ces3	carboxylesterase 3	0.9954	0.898	0.2771	0.4746	No	1.0011	0.939	0.0658	0.506	No	No
101540_at	Tdg /// LOC545124 /// LOC624784 /// LOC670176 /// LOC672659	thymine DNA glycosylase /// similar to thymine DNA glycosylase isoform 2 /// similar to G/T mismatch-specific thymine DNA glycosylase (C-JUN leucine zipper interactive protein JZA-3) /// similar to thymine DNA glycosylase isoform 2 /// similar to G/T mismatch-specific thymine DNA glycosylase (C-JUN leucine zipper interactive protein JZA-3)	1.118	0.0238	0.0071	0.0928	No	1.0946	0.0063	0.0085	0.0033	No	No
101541_at	---	---	0.9614	0.4692	0.0026	0.9677	No	1.0356	0.565	0.0004	0.0193	No	No
101542_f_at	Ddx3x	DEAD/H (Asp-Glu-Ala-Asp/His) box polypeptide 3, X-linked	1.3184	0.0076	0.0006	0.0135	Yes	1.1182	0.0431	0.0017	0.0549	No	Yes
101543_f_at	Tuba6	tubulin, alpha 6	1.0819	0.0177	0.0737	0.1006	No	1.0653	0.0177	0.185	0.0537	No	No
101546_at	---	---	0.8817	0.0279	0.4161	0.0173	No	0.9249	0.31	0.0159	0.639	No	No
101548_at	Synj2bp	synaptojanin 2 binding protein	0.8987	0.0569	0.0269	0.1484	No	0.8855	0.0953	0.0104	0.0692	No	No
101550_at	Tes	testis derived transcript	0.9636	0.4343	0.2885	0.4203	No	0.9589	0.696	0.747	0.777	No	No
101551_s_at	Tes	testis derived transcript	1.0996	0.1058	0.9537	0.3274	No	1.0639	0.0471	0.0375	0.0469	No	No
101552_at	Slc34a1	solute carrier family 34 (sodium phosphate), member 1	1.0019	0.9807	0.9081	0.8385	No	1.0418	0.681	0.532	0.741	No	No
101553_at	Fga	fibrinogen, alpha polypeptide	1.0181	0.6375	0.5857	0.0486	No	0.9932	0.993	0.906	0.141	No	No
101554_at	Nfkbia	nuclear factor of kappa light chain gene enhancer in B-cells inhibitor, alpha	0.765	0.0008	0.0008	0.408	Yes	0.7949	0.0006	0.0001	0.0328	Yes	Yes
101555_at	Rac1	RAS-related C3 botulinum substrate 1	1.0293	0.4111	0.3959	0.3354	No	0.9664	0.399	0.217	0.737	No	No
101557_at	Bckdk	branched chain ketoacid dehydrogenase kinase	0.9906	0.7447	0.0005	0.7779	No	0.9455	0.144	0.0008	0.234	No	No
101558_s_at	Psmb5	proteasome (prosome, macropain) subunit, beta type 5	1.222	0.0263	0.8754	0.9704	No	1.226	0.0578	0.628	0.977	No	No
101560_at	Emb	embigin	0.9527	0.0775	0.0523	0.5281	No	0.9993	0.998	0.727	0.638	No	No
101561_at	Mt2	metallothionein 2	0.6565	0.0007	0.1286	0.0419	Yes	0.8348	0.0329	0.62	0.285	No	Yes
101562_at	Hspa14	heat shock protein 14	1.1264	0.0495	0.1702	0.5925	No	1.0302	0.249	0.0017	0.103	No	No
101564_at	Cnot7	CCR4-NOT transcription complex, subunit 7	1.0378	0.6607	0.0262	0.3689	No	1.0441	0.557	0.164	0.331	No	No
101565_f_at	Serpina1a /// Serpina1b /// Serpina1c /// Serpina1d	serine (or cysteine) peptidase inhibitor, clade A, member 1a /// serine (or cysteine) peptidase inhibitor, clade A, member 1b /// serine (or cysteine) peptidase inhibitor, clade A, member 1c /// serine (or cysteine) peptidase inhibitor, clade A, member 1d	0.9993	0.981	0.0062	0.2707	No	0.9978	0.87	0.0299	0.179	No	No
101566_f_at	Mup1 /// Mup2	major urinary protein 1 /// major urinary protein 2	0.948	0.1735	0.0538	0.5253	No	0.9002	0.317	0.0199	0.0664	No	No
101567_at	Prosc	Proline synthetase co-transcribed	1.0611	0.3126	0.1621	0.8385	No	0.9979	0.861	0.0306	0.144	No	No
101568_at	Prosc	proline synthetase co-transcribed	0.9279	0.1364	0.9869	0.1424	No	0.9444	0.0921	0.115	0.294	No	No
101569_at	Krt2-6g	keratin complex 2, basic, gene 6g	1.0646	0.2624	0.2725	0.2265	No	1.1304	0.0236	0.713	0.618	No	No
101570_at	Igfbp4	insulin-like growth factor binding protein 4	1.0385	0.6299	0.2483	0.722	No	1.0719	0.432	0.425	0.644	No	No
101571_g_at	Igfbp4	insulin-like growth factor binding protein 4	0.9625	0.4819	0.0035	0.5033	No	0.995	0.745	0.0006	0.43	No	No
101572_f_at	Serpina1a	serine (or cysteine) peptidase inhibitor, clade A, member 1a	0.9266	0.0061	0.6077	0.6335	Yes	0.923	0.231	0.127	0.304	No	No
101573_f_at	Rpl27a /// LOC432798 /// LOC433476 /// LOC546052 /// LOC640316 /// LOC665189 /// LOC666648 /// LOC668899 /// LOC669549 /// LOC670518 /// LOC671327 /// LOC675878	ribosomal protein L27a /// similar to 60S ribosomal protein L27a (L29) /// similar to ribosomal protein L27a /// similar to ribosomal protein L27a /// similar to ribosomal protein L27a /// similar to ribosomal protein L27a /// similar to ribosomal protein L27a /// similar to ribosomal protein L27a /// similar to ribosomal protein L27a /// similar to ribosomal protein L27a /// similar to 60S ribosomal protein L27a /// hypothetical protein LOC675878	1.0517	0.4022	0.0575	0.6455	No	1.0029	0.582	0.0001	0.0256	No	No
101574_f_at	Serpina1e	serine (or cysteine) peptidase inhibitor, clade A, member 1e	1.0359	0.516	0.0724	0.6008	No	1.0117	0.914	0.737	0.868	No	No

101575_i_at	Serpina1b	serine (or cysteine) preptidase inhibitor, clade A, member 1b	1.1318	0.0156	0.1257	0.017	No	1.079	0.291	0.577	0.0728	No	No
101576_f_at	Serpina1a /// Serpina1b /// Serpina1c /// Serpina1d	serine (or cysteine) peptidase inhibitor, clade A, member 1a /// serine (or cysteine) preptidase inhibitor, clade A, member 1b /// serine (or cysteine) peptidase inhibitor, clade A, member 1c /// serine (or cysteine) peptidase inhibitor, clade A, member 1d	0.9938	0.8489	0.0256	0.6538	No	1.0014	0.943	0.072	0.82	No	No
101577_at	Rps6 /// LOC434404 /// LOC626259 /// LOC629575 /// LOC638497 /// LOC667739 /// LOC667922 /// LOC671432 /// LOC672192 /// LOC672620	ribosomal protein S6 /// similar to 40S ribosomal protein S6 /// similar to 40S ribosomal protein S6 /// similar to 40S ribosomal protein S6 /// similar to 40S ribosomal protein S6 /// similar to 40S ribosomal protein S6 /// similar to 40S ribosomal protein S6 /// similar to 40S ribosomal protein S6 /// similar to 40S ribosomal protein S6 /// similar to 40S ribosomal protein S6	1.0142	0.0712	0.0066	0.0024	No	1.0389	0.0993	0.249	0.14	No	No
101578_f_at	Actb	actin, beta, cytoplasmic	0.5431	0.1256	0.0408	0.2996	No	0.8905	0.522	0.0005	0.57	No	No
101579_at	Srp9	signal recognition particle 9	1.0257	0.3831	0.1719	0.0598	No	1.0352	0.381	0.276	0.204	No	No
101580_at	Cox7b	cytochrome c oxidase subunit VIIb	0.9672	0.6744	0.0371	0.2784	No	1.0017	0.884	0.046	0.585	No	No
101581_at	Ube3a	ubiquitin protein ligase E3A	1.1177	0.1505	0.0058	0.4935	No	1.0186	0.712	0.0546	0.221	No	No
101582_at	Gnl2	guanine nucleotide binding protein-like 2 (nucleolar)	0.9882	0.8267	0.1179	0.5803	No	0.9879	0.772	0.0055	0.997	No	No
101583_at	Btg2	B-cell translocation gene 2, anti-proliferative	0.9425	0.1399	0.4651	0.072	No	1.0032	0.917	0.242	0.606	No	No
101584_at	Rsu1	Ras suppressor protein 1	0.9317	0.0888	0.0112	0.4988	No	0.9721	0.477	0.661	0.394	No	No
101585_at	Pgrmc1	progesterone receptor membrane component 1	1.011	0.6642	0.0005	0.1731	No	0.9698	0.348	0.0474	0.487	No	No
101587_at	Ephx1	epoxide hydrolase 1, microsomal	0.949	0.0794	0.1129	0.0261	No	0.9157	0.0755	0.791	0.247	No	No
101588_at	Slc16a1	solute carrier family 16 (monocarboxylic acid transporters), member 1	0.9751	0.6141	0.5537	0.0946	No	1.0281	0.24	0.981	0.145	No	No
101589_at	Hmgn2	high mobility group nucleosomal binding domain 2	1.1273	0.0851	0.0053	0.6479	No	1.0509	0.468	0.233	0.173	No	No
101590_at	Lamp2	lysosomal membrane glycoprotein 2	0.9855	0.7919	0.021	0.113	No	0.9418	0.218	0.073	0.227	No	No
101591_at	Xpnpep1	X-prolyl aminopeptidase (aminopeptidase P) 1, soluble	1.0209	0.5609	0.9301	0.621	No	1.046	0.116	0.37	0.222	No	No
101593_at	Crip2	cysteine rich protein 2	0.9715	0.5907	0.0186	0.1857	No	0.9819	0.993	0.0066	0.048	No	No
101595_at	Ranbp9	RAN binding protein 9	1.0585	0.4537	0.0243	0.0226	No	1.0651	0.31	0.0439	0.0195	No	No
101596_at	---	---	0.9913	0.7634	0.6399	0.9391	No	1.0366	0.917	0.0091	0.115	No	No
101597_at	D12Ertd216e	DNA segment, Chr 12, ERATO Doi 216, expressed	0.9984	0.9711	0.1741	0.226	No	0.8282	0.0076	0.0003	0.462	Yes	No
101598_at	---	Transcribed locus	1.1442	0.2232	0.2067	0.6244	No	1.1196	0.0975	0.209	0.299	No	No
101599_at	C030018P15Rik	RIKEN cDNA C030018P15 gene	0.9759	0.4063	0.6419	0.9728	No	1.0372	0.268	0.0033	0.075	No	No
101600_at	C79015	expressed sequence C79015	0.9728	0.7997	0.4978	0.938	No	1.0342	0.541	0.0677	0.547	No	No
101601_at	DXErtd223e	DNA segment, Chr X, ERATO Doi 223, expressed	0.9281	0.2907	0.3158	0.9458	No	0.9594	0.717	0.202	0.654	No	No
101602_at	Galnt4	UDP-N-acetyl-alpha-D-galactosamine:polypeptide N-acetylgalactosaminyltransferase 4	1.1051	0.0513	0.2236	0.0069	No	1.1154	0.0195	0.395	0.0049	No	No
101603_g_at	Galnt4	UDP-N-acetyl-alpha-D-galactosamine:polypeptide N-acetylgalactosaminyltransferase 4	1.0237	0.7206	0.1046	0.9783	No	0.9792	0.769	0.0388	0.695	No	No
101604_at	C79296	expressed sequence C79296	0.9718	0.3521	0.3779	0.0476	No	1.1123	0.207	0.244	0.0085	No	No
101605_at	D3Ertd246e	DNA segment, Chr 3, ERATO Doi 246, expressed	1.0841	0.0094	0.7077	0.0203	Yes	1.2832	0.0965	0.0875	0.0173	No	No
101606_at	D1Ertd259e	DNA segment, Chr 1, ERATO Doi 259, expressed	1.017	0.5646	0.2588	0.3572	No	1.0074	0.762	0.012	0.264	No	No
101607_at	C80068	expressed sequence C80068	0.9587	0.3799	0.0079	0.0969	No	0.8051	0.0735	0.155	0.0479	No	No
101608_at	C80360	expressed sequence C80360	0.9636	0.5171	0.4162	0.4378	No	0.9494	0.721	0.5	0.423	No	No
101609_at	Slc5a11	solute carrier family 5 (sodium/glucose cotransporter), member 11	0.9446	0.0704	0.0105	0.2114	No	0.9549	0.225	0.0008	0.0116	No	No
101610_at	Oxsm	3-oxoacyl-ACP synthase, mitochondrial	0.9133	0.2367	0.1034	0.3716	No	0.9452	0.482	0.0845	0.829	No	No
101611_at	D11Ertd326e	DNA segment, Chr 11, ERATO Doi 326, expressed	0.926	0.2323	0.0324	0.1083	No	0.9475	0.216	0.0039	0.301	No	No
101612_at	Lats1	large tumor suppressor	1.0338	0.5579	0.0423	0.3615	No	1.0271	0.572	0.0091	0.816	No	No
101613_at	---	PREDICTED: Mus musculus similar to developmentally regulated repeat element-containing transcript 1 (LOC622619), mRNA	1.2237	0.0541	0.0839	0.067	No	0.981	0.626	0.011	0.19	No	No
101614_at	---	---	0.977	0.4179	0.0404	0.1523	No	1.0251	0.917	0.0146	0.0489	No	No
101615_at	Pcnt	Pericentrin (kendrin)	1.0512	0.3248	0.0466	0.1726	No	0.9647	0.603	0.264	0.57	No	No
101616_at	Gm1067	gene model 1067, (NCBI)	1.0431	0.505	0.0691	0.5884	No	0.9313	0.275	0.0197	0.915	No	No
101617_s_at	---	P53-variant (p53)	0.9791	0.6004	0.0167	0.0454	No	1.0603	0.47	0.502	0.308	No	No
101618_r_at	---	P53-variant (p53)	0.9672	0.3978	0.0056	0.0824	No	0.9731	0.813	0.0115	0.33	No	No
101619_at	Ifna6	interferon alpha family, gene 6	0.91	0.2625	0.2741	0.5331	No	0.9763	0.734	0.107	0.278	No	No
101620_at	Gp1ba	glycoprotein 1b, alpha polypeptide	0.947	0.5068	0.03	0.4439	No	0.9245	0.445	0.0031	0.0234	No	No
101621_at	Abph	androgen-binding protein eta	1.0234	0.6736	0.6052	0.4689	No	1.0744	0.163	0.131	0.22	No	No
101622_at	Alx4	aristaless 4	1.0126	0.8104	0.0293	0.6468	No	1.0437	0.311	0.0149	0.329	No	No
101623_at	Slc16a8	solute carrier family 16 (monocarboxylic acid transporters), member 8	0.9844	0.3398	0.509	0.0318	No	0.9885	0.845	0.116	0.441	No	No
101624_at	Kcns1	K+ voltage-gated channel, subfamily S, 1	0.9937	0.8743	0.0011	0.1814	No	1.0118	0.88	0.0002	0.449	No	No
101625_at	Kcns2	K+ voltage-gated channel, subfamily S, 2	1.0663	0.238	0.0773	0.1007	No	1.0252	0.505	0.0091	0.0667	No	No
101626_at	Adam7	a disintegrin and metallopeptidase domain 7	1.0282	0.7263	0.3878	0.4754	No	0.9313	0.349	0.92	0.662	No	No
101627_at	Pld1	phospholipase D1	1.0063	0.8687	0.079	0.0928	No	0.9744	0.444	0.0746	0.0634	No	No
101628_g_at	Pld1	phospholipase D1	0.9975	0.914	0.0505	0.2061	No	0.9331	0.233	0.0143	0.308	No	No
101629_s_at	Stx1b2 /// Stx1b1	syntaxin 1B2 /// syntaxin 1B1	0.9336	0.0231	0.0757	0.7307	No	0.9106	0.0344	0.668	0.543	No	No
101630_f_at	V2r1 /// V2r12 /// V2r2 /// V2r8 /// V2r9	vomeronasal 2, receptor, 1 /// vomeronasal 2, receptor, 12 /// vomeronasal 2, receptor, 2 /// vomeronasal 2, receptor, 8 /// vomeronasal 2, receptor, 9	1.0666	0.4322	0.5208	0.7669	No	1.0153	0.782	0.855	0.757	No	No
101631_at	Sox11	SRY-box containing gene 11	0.796	0.0306	0.0106	0.1135	No	0.8369	0.0536	0.0196	0.0705	No	No
101632_at	Tnfrsf11a	tumor necrosis factor receptor superfamily, member 11a	0.9301	0.2253	0.6601	0.8272	No	0.9847	0.644	0.0233	0.158	No	No
101633_at	Igkv17-127 /// LOC383196 /// LOC432992 /// LOC630980 /// LOC632171 /// Igkv17-121 /// LOC672212 /// LOC675694	immunoglobulin kappa chain variable 17-127 /// similar to Ig kappa chain V region EV15 precursor /// similar to Ig kappa chain V region EV15 precursor /// similar to Ig kappa chain V region EV15 precursor /// similar to Ig kappa chain V region EV15 precursor /// immunoglobulin kappa chain variable 17-121 /// similar to Ig kappa chain V region EV15 precursor /// similar to Ig kappa chain V region EV15 precursor	1.0597	0.336	0.1895	0.0984	No	1.1724	0.0223	0.0527	0.0784	No	No
101634_at	Npm1	nucleophosmin 1	1.0475	0.2113	0.0169	0.1153	No	0.9884	0.675	0.0195	0.578	No	No
101635_f_at	Mup5	major urinary protein 5	0.9338	0.3315	0.6745	0.5569	No	0.8998	0.0249	0.0758	0.317	No	No
101636_at	Spt2	salivary protein 2	0.9487	0.334	0.0881	0.3525	No	0.9829	0.808	0.0152	0.852	No	No
101637_at	Ceacam10	CEA-related cell adhesion molecule 10	1.0236	0.7766	0.0102	0.7522	No	0.9546	0.455	0.741	0.323	No	No
101638_s_at	Cyp3a11 /// Cyp3a16	cytochrome P450, family 3, subfamily a, polypeptide 11 /// cytochrome P450, family 3, subfamily a, polypeptide 16	1.0418	0.3096	0.0025	0.0546	No	1.0226	0.651	0.0224	0.444	No	No

101639_r_at	Cyp3a16	cytochrome P450, family 3, subfamily a, polypeptide 16	0.9863	0.7768	0.6356	0.617	No	0.917	0.736	0.33	0.948	No	No
101640_f_at	Gm1418 /// Gm1419 /// Gm1499 /// Igkv4-61 /// Igkv4-59 /// LOC672342 /// LOC673628 /// LOC675950	gene model 1418, (NCBI) /// gene model 1419, (NCBI) /// gene model 1499, (NCBI) /// immunoglobulin kappa chain variable 4-61 /// immunoglobulin kappa chain variable 4-59 /// similar to Ig kappa chain V-IV region S107B precursor /// similar to Ig kappa chain V-IV region S107B precursor /// similar to Ig kappa chain V-IV region S107B precursor	0.9763	0.657	0.9468	0.1303	No	0.8874	0.0154	0.474	0.0904	No	No
101641_at	---	---	1.0498	0.1766	0.0107	0.098	No	1.1061	0.113	0.0364	0.214	No	No
101642_at	---	---	1.0085	0.6474	0.6481	0.1184	No	1.0541	0.405	0.206	0.852	No	No
101643_at	Stx1b1	syntaxin 1B1	1.0805	0.2089	0.3226	0.6608	No	1.1144	0.153	0.262	0.135	No	No
101644_f_at	V2r1 /// V2r12 /// V2r2 /// V2r8 /// V2r9	vomeronasal 2, receptor, 1 /// vomeronasal 2, receptor, 12 /// vomeronasal 2, receptor, 2 /// vomeronasal 2, receptor, 8 /// vomeronasal 2, receptor, 9	1.1408	0.0489	0.1459	0.0506	No	1.0658	0.232	0.0542	0.177	No	No
101645_f_at	V2r10 /// V2r11	vomeronasal 2, receptor, 10 /// vomeronasal 2, receptor, 11	0.9837	0.8342	0.3777	0.53	No	0.9413	0.371	0.172	0.309	No	No
101646_at	Pigq	phosphatidylinositol glycan, class Q	0.952	0.3129	0.0284	0.4995	No	0.9611	0.171	0.0015	0.614	No	No
101647_at	Adk	Adenosine kinase	0.8959	0.0859	0.0223	0.6983	No	0.8181	0.0429	0.654	0.266	No	No
101648_at	Foxd4	forkhead box D4	0.9707	0.5867	0.0167	0.5533	No	0.8996	0.193	0.248	0.243	No	No
101649_at	Pip5k1a	phosphatidylinositol-4-phosphate 5-kinase, type 1 alpha	1.0364	0.2864	0.0093	0.0822	No	0.9667	0.296	0.0004	0.0721	No	No
101650_at	Pcdha4 /// Pcdha6 /// Pcdha7 /// Pcdha5 /// Pcdha11 /// Pcdha10 /// Pcdha1 /// Pcdha9 /// Pcdha3 /// Pcdha12 /// Pcdha2 /// Pcdha8 /// Pcdhac1 /// Pcdhac2	protocadherin alpha 4 /// protocadherin alpha 6 /// protocadherin alpha 7 /// protocadherin alpha 5 /// protocadherin alpha 11 /// protocadherin alpha 10 /// protocadherin alpha 1 /// protocadherin alpha 9 /// protocadherin alpha 3 /// protocadherin alpha 12 /// protocadherin alpha 2 /// protocadherin alpha 8 /// protocadherin alpha subfamily C, 1 /// protocadherin alpha subfamily C, 2	1.0013	0.9495	0.7008	0.401	No	1.0139	0.753	0.964	0.192	No	No
101651_at	Cntfr	ciliary neurotrophic factor receptor	1.0226	0.4253	0.0002	0.6638	No	1.0033	0.984	0.0008	0.651	No	No
101652_i_at	H2-D4	histocompatibility 2, D region locus 4	0.9602	0.6646	0.017	0.4769	No	0.9833	0.792	0.0013	0.462	No	No
101653_f_at	H2-D4	histocompatibility 2, D region locus 4	0.9413	0.0917	0.0003	0.0232	No	1.0752	0.113	0.0065	0.533	No	No
101654_at	Hist1h2bb	histone 1, H2bb	0.9568	0.3951	0.0077	0.0981	No	1.0091	0.818	0.358	0.517	No	No
101655_at	Zfp94	zinc finger protein 94	0.9163	0.242	0.2422	0.6146	No	0.8246	0.259	0.632	0.335	No	No
101656_f_at	Igh-V7183 /// Igk-V1 /// Igkv1-117 /// Cr1 /// Igkv1-110	immunoglobulin heavy chain (V7183 family) /// immunoglobulin kappa chain variable 1 (V1) /// immunoglobulin kappa chain variable 1-117 /// Ig kappa chain /// immunoglobulin kappa chain variable 1-110	0.9805	0.6866	0.0041	0.6338	No	0.9744	0.972	0.0882	0.398	No	No
101657_at	Bmp8b	bone morphogenetic protein 8b	1.0618	0.3092	0.0556	0.8158	No	1.1091	0.0282	0.0051	0.74	No	No
101658_f_at	H2-Q8	histocompatibility 2, Q region locus 8	1.0089	0.704	0.001	0.324	No	1.0484	0.261	0.0032	0.93	No	No
101659_at	Hsd3b2	hydroxysteroid dehydrogenase-2, delta<5>-3-beta	1.1462	0.0818	0.2354	0.0065	No	1.2928	0.087	0.385	0.0438	No	No
101660_at	Mtr	5-methyltetrahydrofolate-homocysteine methyltransferase	0.9123	0.3452	0.4658	0.9056	No	0.9484	0.355	0.208	0.797	No	No
101661_r_at	---	Transcribed locus	0.9844	0.7148	0.1035	0.0213	No	1.1465	0.236	0.0214	0.158	No	No
101662_at	Suz12	Suppressor of zeste 12 homolog (Drosophila)	1.0535	0.5263	0.5053	0.8792	No	1.1221	0.0991	0.595	0.263	No	No
101663_s_at	Hhat	Hedgehog acyltransferase	0.9042	0.3002	0.4283	0.3705	No	0.9732	0.802	0.823	0.905	No	No
101664_at	Rps3a	ribosomal protein S3a	1.0445	0.1026	0.0282	0.9719	No	1.0369	0.118	0.0135	0.193	No	No
101665_at	Nr5a1	nuclear receptor subfamily 5, group A, member 1	1.0271	0.3695	0.1099	0.6132	No	0.9981	0.952	0.0014	0.0131	No	No
101666_at	Nr5a1	nuclear receptor subfamily 5, group A, member 1	0.9307	0.4944	0.2798	0.3546	No	0.9848	0.846	0.0137	0.83	No	No
101667_at	Cwf19l1	CWF19-like 1, cell cycle control (S. pombe)	1.1772	0.0737	0.0087	0.0702	No	1.057	0.518	0.0001	0.208	No	No
101668_at	AA589418	expressed sequence AA589418	0.9307	0.2979	0.6717	0.3851	No	0.9503	0.209	0.229	0.0853	No	No
101669_at	---	---	1.0314	0.1417	0.004	0.0775	No	1.0203	0.836	0.132	0.522	No	No
101670_at	Stk4	Serine/threonine kinase 4	0.9359	0.6241	0.3016	0.6335	No	0.8639	0.362	0.251	0.68	No	No
101671_at	---	Transcribed locus	1.0533	0.4671	0.8274	0.5102	No	1.1332	0.308	0.177	0.803	No	No
101672_at	BC021785	CDNA sequence BC021785	0.9846	0.8883	0.9477	0.7936	No	0.9493	0.61	0.0428	0.702	No	No
101673_r_at	AA675035	expressed sequence AA675035	1.0308	0.6055	0.6438	0.2851	No	1.0064	0.834	0.0609	0.255	No	No
101674_at	Stk36	Serine/threonine kinase 36 (fused homolog, Drosophila)	0.9418	0.3694	0.0724	0.4244	No	1.2076	0.105	0.0924	0.0624	No	No
101675_at	---	---	0.9958	0.9424	0.004	0.293	No	0.9457	0.56	0.261	0.89	No	No
101676_at	Gpx3	glutathione peroxidase 3	0.8236	0.0297	0.0405	0.2879	No	0.8047	0.0031	0.0039	0.0619	Yes	Yes
101677_at	1110035H17Rik	RIKEN cDNA 1110035H17 gene	1.0533	0.2921	0.0404	0.0191	No	1.0585	0.346	0.156	0.289	No	No
101678_r_at	---	---	1.087	0.1966	0.2073	0.0804	No	1.1249	0.014	0.15	0.0043	No	No
101679_at	Opn1mw	opsin 1 (cone pigments), medium-wave-sensitive (color blindness, deutan)	1.0914	0.0016	0.0185	0.0011	No	1.0208	0.747	0.0672	0.534	No	No
101680_at	Rpl27a /// LOC432798 /// LOC433476 /// LOC545487 /// LOC665189 /// LOC668899 /// LOC670518 /// LOC671583 /// LOC673608	ribosomal protein L27a /// similar to 60S ribosomal protein L27a (L29) /// similar to ribosomal protein L27a /// similar to ribosomal protein L27a /// similar to ribosomal protein L27a /// similar to ribosomal protein L27a /// similar to ribosomal protein L27a /// similar to ribosomal protein L27a /// similar to 60S ribosomal protein L27a (L29)	0.9874	0.7807	0.0004	0.245	No	1.0045	0.715	0.0001	0.381	No	No
101681_f_at	H2-Bl	histocompatibility 2, blastocyst	0.9929	0.8546	0.101	0.1215	No	0.9474	0.318	0.0216	0.446	No	No
101682_f_at	Mup4	major urinary protein 4	0.9	0.0144	0.1532	0.5166	No	0.8777	0.0962	0.192	0.242	No	No
101683_at	Gipc1	GIPC PDZ domain containing family, member 1	1.0382	0.5754	0.0091	0.157	No	1.0689	0.439	0.0068	0.137	No	No
101684_r_at	Srst	simple repeat sequence-containing transcript	1.1195	0.1259	0.0499	0.0089	No	1.1347	0.0928	0.0616	0.0105	No	No
101685_f_at	---	---	1.0013	0.9709	0.0398	0.5654	No	0.9884	0.178	0.0002	0.0012	No	No
101686_at	Zfp59	Zinc finger protein 59	1.0484	0.429	0.0186	0.3883	No	0.9298	0.273	0.0198	0.687	No	No
101687_r_at	---	---	1.0203	0.7084	0.4556	0.2003	No	1.0758	0.269	0.0017	0.174	No	No
101688_at	D4Ertd58e	DNA segment, Chr 4, ERATO Doi 58, expressed	1.0105	0.8077	0.0043	0.1673	No	1.0269	0.0912	0.0001	0.0059	No	No
101689_at	AA416453	expressed sequence AA416453	0.9924	0.9038	0.2663	0.7068	No	1.0416	0.165	0.0007	0.0215	No	No
101690_r_at	---	---	1.0682	0.1727	0.0713	0.106	No	1.2363	0.0404	0.005	0.0349	No	No
101691_s_at	C77405	expressed sequence C77405	0.9089	0.204	0.65	0.9441	No	0.9077	0.405	0.246	0.769	No	No
101692_r_at	C77405	expressed sequence C77405	0.9376	0.2356	0.0005	0.3246	No	0.9192	0.334	0.0001	0.651	No	No

101693_f_at	Timm8a2	translocase of inner mitochondrial membrane 8 homolog a2 (yeast)	1.0226	0.7407	0.2523	0.4763	No	0.9919	0.993	0.0015	0.167	No	No
101694_f_at	Myst2	MYST histone acetyltransferase 2	1.061	0.1668	0.0223	0.2336	No	1.0581	0.35	0.0843	0.148	No	No
101695_at	Eif3s6	Eukaryotic translation initiation factor 3, subunit 6	1.0029	0.9659	0.8489	0.7776	No	0.9737	0.72	0.179	0.445	No	No
101696_r_at	---	---	0.9177	0.3596	0.0006	0.3862	No	0.9431	0.323	0.0004	0.41	No	No
101697_f_at	---	---	0.995	0.9216	0.0081	0.1052	No	0.918	0.388	0.0181	0.0382	No	No
101698_f_at	Krt2-17	keratin complex 2, basic, gene 17	1.0331	0.5063	0.011	0.0474	No	1.0259	0.41	0.0015	0.103	No	No
101699_at	Phxr2	per-hexamer repeat gene 2	0.9867	0.8808	0.5931	0.2036	No	1.0547	0.652	0.422	0.497	No	No
101700_at	Phxr4	per-hexamer repeat gene 4	1.0409	0.5141	0.0597	0.1216	No	0.9466	0.408	0.997	0.14	No	No
101701_at	Cdh8	cadherin 8	0.9862	0.6879	0.8705	0.9579	No	0.9712	0.49	0.0552	0.328	No	No
101702_at	Rs1h	retinoschisis 1 homolog (human)	0.9595	0.5733	0.0503	0.1971	No	0.9901	0.905	0.802	0.958	No	No
101703_at	---	---	0.9542	0.1575	0.0007	0.2432	No	0.9658	0.586	0.0146	0.887	No	No
101704_at	Hnf4g	hepatocyte nuclear factor 4, gamma	0.9182	0.1871	0.1062	0.2683	No	0.9556	0.592	0.15	0.552	No	No
101705_at	Phxr1	per-hexamer repeat gene 1	0.9762	0.2989	0.0338	0.4854	No	1.1014	0.264	0.0004	0.0134	No	No
101706_at	Cnga3	cyclic nucleotide gated channel alpha 3	0.9717	0.5923	0.0006	0.0743	No	0.9883	0.879	0.166	0.774	No	No
101707_at	Aldh1a2	aldehyde dehydrogenase family 1, subfamily A2	1.0537	0.2248	0.019	0.0787	No	1.0087	0.799	0.0053	0.367	No	No
101708_at	Myo1f	myosin IF	0.9205	0.2471	0.5347	0.1208	No	0.9667	0.512	0.033	0.423	No	No
101709_at	Ahrr	aryl-hydrocarbon receptor repressor	0.9805	0.6113	0.9928	0.8169	No	0.9814	0.515	0.0348	0.334	No	No
101710_at	Gria4	glutamate receptor, ionotropic, AMPA4 (alpha 4)	1.0009	0.9806	0.1408	0.7098	No	1.0262	0.644	0.108	0.289	No	No
101711_at	Mds1	myelodysplasia syndrome 1 homolog (human)	1.0597	0.063	0.4633	0.0122	No	1.1303	0.481	0.758	0.0801	No	No
101712_at	P2rx7	purinergic receptor P2X, ligand-gated ion channel, 7	1.0378	0.6118	0.5962	0.5717	No	1.0076	0.0449	0	0.0002	No	No
101713_at	Slc7a11	solute carrier family 7 (cationic amino acid transporter, y+ system), member 11	1.0433	0.0881	0.3523	0.3013	No	1.0795	0.422	0.272	0.189	No	No
101714_at	Odz1	odd Oz/ten-m homolog 1 (Drosophila)	1.0509	0.4496	0.6547	0.5735	No	1.0213	0.77	0.659	0.786	No	No
101715_at	Ear4	eosinophil-associated, ribonuclease A family, member 4	0.9132	0.121	0.8605	0.0475	No	1.0277	0.577	0.0446	0.0893	No	No
101716_at	Ear5	eosinophil-associated, ribonuclease A family, member 5	1.008	0.9488	0.0433	0.5974	No	0.8687	0.184	0.376	0.55	No	No
101717_at	Sstr4	somatostatin receptor 4	1.0792	0.1731	0.2747	0.1779	No	1.0173	0.466	0.0001	0.535	No	No
101718_f_at	Gm1419 /// Gm1502 /// Igkv4-61	gene model 1419, (NCBI) /// gene model 1502, (NCBI) /// immunoglobulin kappa chain variable 4-61	1.109	0.0249	0.9758	0.0208	No	1.1194	0.0234	0.376	0.0059	No	No
101719_at	Pnmt	phenylethanolamine-N-methyltransferase	0.9835	0.6144	0.0757	0.2552	No	1.0399	0.473	0.0888	0.177	No	No
101720_f_at	Igk-V8	immunoglobulin kappa chain variable 8 (V8)	1.0104	0.8584	0.0184	0.5422	No	1.0321	0.579	0.0169	0.37	No	No
101723_r_at	Adam28	a disintegrin and metallopeptidase domain 28	1.034	0.5749	0.0024	0.1786	No	1.0352	0.624	0.0024	0.225	No	No
101724_at	Hes2	hairy and enhancer of split 2 (Drosophila)	1.0003	0.9951	0.0218	0.6853	No	1.0878	0.094	0.15	0.424	No	No
101725_at	B3galt1	UDP-Gal:betaGlcNAc beta 1,3-galactosyltransferase, polypeptide 1	1.0257	0.2854	0.0162	0.0055	No	1.013	0.704	0.394	0.0862	No	No
101726_at	Nmbr	neuromedin B receptor	1.017	0.7564	0.2352	0.2714	No	1.0133	0.871	0.328	0.117	No	No
101727_at	Nfkbie	nuclear factor of kappa light polypeptide gene enhancer in B-cells inhibitor, epsilon	0.964	0.5453	0.0334	0.5665	No	1.0199	0.709	0.157	0.605	No	No
101728_at	C5ar1	complement component 5a receptor 1	0.9628	0.086	0.0059	0.0556	No	0.9253	0.0347	0.0046	0.0083	No	No
101729_at	Gja9	gap junction membrane channel protein alpha 9	1.0638	0.1483	0.0005	0.1656	No	0.9966	0.773	0.0011	0.271	No	No
101730_at	Cdh6	cadherin 6	1.0013	0.9813	0.2306	0.7699	No	1.0058	0.856	0.224	0.315	No	No
101731_at	---	---	0.9733	0.3119	0.0001	0.5135	No	0.9759	0.235	0.0001	0.27	No	No
101732_at	Phxr5	per-hexamer repeat gene 5	1.2134	0.0157	0.0109	0.025	No	1.1337	0.0617	0.0177	0.0156	No	No
101733_at	Ccr1l1	chemokine (C-C motif) receptor 1-like 1	0.9738	0.5321	0.0768	0.7574	No	1.0616	0.371	0.543	0.917	No	No
101734_at	Grid2	glutamate receptor, ionotropic, delta 2	0.9362	0.1785	0.8375	0.1146	No	0.951	0.393	0.862	0.037	No	No
101735_f_at	Ang2	angiogenin, ribonuclease A family, member 2	1.0084	0.8893	0.6536	0.9768	No	1.1351	0.0146	0.248	0.0286	No	No
101736_at	Ppyr1	pancreatic polypeptide receptor 1	1.0922	0.2842	0.2591	0.6833	No	1.1892	0.0115	0.0852	0.024	No	No
101737_at	Fshb	follicle stimulating hormone beta	0.9996	0.9943	0.9047	0.7882	No	1.0971	0.355	0.845	0.156	No	No
101738_at	Lhb	luteinizing hormone beta	0.9472	0.257	0.3631	0.7195	No	0.9168	0.111	0.823	0.088	No	No
101739_at	Cmar	cell matrix adhesion regulator	0.9767	0.6112	0.0104	0.6413	No	1.0408	0.517	0.0122	0.696	No	No
101740_at	Adra1a	adrenergic receptor, alpha 1a	1.0259	0.6288	0.7247	0.285	No	0.9593	0.188	0.0166	0.988	No	No
101741_at	Psmb5	proteasome (prosome, macropain) subunit, beta type 5	1.0148	0.732	0.0074	0.3741	No	0.9581	0.349	0.0332	0.571	No	No
101742_at	V2R2	tissue-type vomeronasal neurons putative pheromone receptor V2R2	0.9326	0.0115	0.0074	0.1521	No	1.0569	0.549	0.176	0.982	No	No
101743_f_at	Igh-1a	immunoglobulin heavy chain 1a (serum IgG2a)	1.0111	0.862	0.0375	0.7297	No	1.0308	0.247	0.0187	0.0062	No	No
101744_i_at	Igh-VJ558 /// Igh-1a	Immunoglobulin heavy chain (J558 family) /// Immunoglobulin heavy chain 1a (serum IgG2a)	1.0515	0.1609	0.1306	0.9918	No	1.3223	0.0479	0.295	0.0333	No	No
101745_f_at	Igh-6	immunoglobulin heavy chain 6 (heavy chain of IgM)	0.9682	0.6308	0.2226	0.2607	No	0.971	0.491	0.002	0.302	No	No
101746_i_at	Igh-VJ558	Immunoglobulin heavy chain (J558 family)	1.1068	0.0156	0.0021	0.0213	No	1.3563	0.0045	0	0.152	Yes	Yes
101747_f_at	Igh-VJ558	Immunoglobulin heavy chain (J558 family)	1.0631	0.4113	0.0072	0.486	No	1.0857	0.0333	0.0768	0.0264	No	No
101748_at	Bdkrb1	bradykinin receptor, beta 1	1.0237	0.673	0.7809	0.641	No	1.0141	0.734	0.743	0.0843	No	No
101749_at	Igh	Immunoglobulin heavy chain complex	1.0778	0.1543	0.0199	0.3046	No	1.2994	0.0413	0.722	0.0948	No	No
101751_f_at	Gm189 /// Gm1419 /// Igkv4-61 /// Igkv4-56 /// Igkv13-78-1 /// Igkv4-73 /// Igkv4-69 /// Igkv4-63 /// LOC636598 /// LOC636646 /// LOC636677 /// LOC636730 /// Igkv4-62 /// LOC636818 /// Igkv4-77 /// Igkv4-59 /// LOC670901 /// LOC672339 /// LOC672342	gene model 189, (NCBI) /// gene model 1419, (NCBI) /// immunoglobulin kappa chain variable 4-61 /// immunoglobulin kappa chain variable 4-56 /// immunoglobulin kappa chain variable 13-78-1 /// immunoglobulin kappa chain variable 4-73 /// immunoglobulin kappa chain variable 4-69 /// immunoglobulin kappa chain variable 4-63 /// similar to Ig kappa chain V-IV region S107B precursor /// similar to Ig kappa chain V-IV region S107B precursor /// similar to Ig kappa chain V-IV region S107B precursor /// similar to Ig kappa chain V-IV region S107B precursor /// immunoglobulin kappa chain variable 4-62 /// similar to Ig kappa chain V-VI region NQ2-17.4.1 /// immunoglobulin kappa chain variable 4-77 /// immunoglobulin kappa chain variable 4-59 /// hypothetical protein LOC670901 /// similar to Ig kappa chain V-IV region S107B precursor /// similar to Ig kappa chain V-IV region S107B precursor /// similar to Ig kappa chain V-IV region S107B precursor /// similar to Ig kappa chain V-IV region S107B precursor	1.0304	0.2138	0.0645	0.3648	No	1.0348	0.399	0.0567	0.422	No	No
101752_f_at	Igh-1a	Immunoglobulin heavy chain 1a (serum IgG2a)	0.955	0.7776	0.6696	0.8105	No	1.0378	0.354	0.0065	0.157	No	No
101753_s_at	Lyzs /// Lzp-s	lysozyme /// P lysozyme structural	0.8762	0.0221	0.0001	0.0131	No	0.8775	0.0531	0.0002	0.0175	No	No
101754_f_at	Sprr2g	small proline-rich protein 2G	1.0092	0.8354	0.5911	0.935	No	0.952	0.0899	0.0221	0.0195	No	No
101755_f_at	Sprr2j	small proline-rich protein 2J	1.0016	0.9666	0.056	0.0744	No	0.9296	0.0653	0.0309	0.928	No	No
101756_f_at	Sprr2k	small proline-rich protein 2K	1.0931	0.013	0.0592	0.0032	No	1.1309	0.0507	0.45	0.0732	No	No
101757_at	Nfe2l1	nuclear factor, erythroid derived 2,-like 1	1.0455	0.118	0.0077	0.3576	No	1.0423	0.0151	0	0.561	No	No
101758_at	Grem1	gremlin 1	0.9745	0.2	0.0003	0.0636	No	0.9331	0.197	0.0169	0.149	No	No
101759_at	---	---	1.0061	0.8743	0.2215	0.5403	No	0.9356	0.636	0.0009	0.275	No	No
101760_at	Fut2	fucosyltransferase 2	0.8855	0.0167	0.0005	0.7453	No	0.9449	0.157	0.0032	0.158	No	No

101761_f_at	Sprr2d /// Sprr2e /// Sprr2f /// Sprr2h /// Sprr2i /// Sprr2j	small proline-rich protein 2D /// small proline-rich protein 2E /// small proline-rich protein 2F /// small proline-rich protein 2H /// small proline-rich protein 2I /// small proline-rich protein 2J	1.0803	0.2306	0.0284	0.0511	No	1.0443	0.291	0.0294	0.124	No	No
101762_at	Sprr3	small proline-rich protein 3	0.951	0.4603	0.0772	0.236	No	0.8681	0.0391	0.543	0.036	No	No
101763_at	Gpr50	G-protein-coupled receptor 50	0.9764	0.5017	0.057	0.3746	No	0.9861	0.71	0.0761	0.598	No	No
101764_at	Htr1d	5-hydroxytryptamine (serotonin) receptor 1D	0.9854	0.2989	0.0006	0.0032	No	1.0207	0.597	0.0898	0.0625	No	No
101765_at	Amh	anti-Mullerian hormone	1.0339	0.5044	0.0475	0.2857	No	1.0614	0.346	0.674	0.804	No	No
101768_at	---	---	1.0954	0.191	0.7685	0.9595	No	1.1649	0.087	0.202	0.744	No	No
101769_at	Pcdha10	protocadherin alpha 10	0.9162	0.0355	0.0688	0.0672	No	1.0564	0.515	0.0171	0.8	No	No
101770_i_at	Pcdha12	protocadherin alpha 12	1.053	0.4333	0.0304	0.7332	No	0.9754	0.746	0.0004	0.691	No	No
101771_r_at	Pcdha12 /// LOC634282	protocadherin alpha 12 /// similar to protocadherin alpha 12	0.9213	0.2307	0.0054	0.9117	No	0.9361	0.222	0.0034	0.262	No	No
101772_r_at	Pcdha11	protocadherin alpha 11	0.9359	0.1928	0.0055	0.1293	No	0.9422	0.0327	0.0001	0.0058	No	No
101773_r_at	Pcdha9	protocadherin alpha 9	1.0568	0.3613	0.1184	0.322	No	0.9882	0.756	0.0481	0.903	No	No
101774_at	Col4a4	procollagen, type IV, alpha 4	0.9966	0.9606	0.0704	0.8904	No	0.9868	0.606	0.0049	0.106	No	No
101775_at	Lmx1b	LIM homeobox transcription factor 1 beta	0.9152	0.1945	0.0853	0.8526	No	1.0107	0.821	0.72	0.967	No	No
101776_at	Erbb3	v-erb-b2 erythroblastic leukemia viral oncogene homolog 3 (avian)	0.9979	0.9629	0.9956	0.385	No	1.1022	0.0151	0.0376	0.155	No	No
101777_at	Erbb4	v-erb-a erythroblastic leukemia viral oncogene homolog 4 (avian)	1.0059	0.8905	0.001	0.0542	No	0.9841	0.965	0.002	0.202	No	No
101778_at	Gja5	gap junction membrane channel protein alpha 5	0.9904	0.7331	0.9031	0.0686	No	1.064	0.109	0.666	0.169	No	No
101779_at	Drd3	dopamine receptor 3	0.9909	0.8517	0.0455	0.4302	No	1.1009	0.229	0.0977	0.0971	No	No
101780_at	---	---	0.9499	0.3146	0.8831	0.0271	No	0.8816	0.213	0.572	0.0455	No	No
101781_f_at	LOC238836 /// LOC433418 /// LOC623365 /// LOC633468 /// LOC638008 /// LOC673620	similar to H3 histone, family 3B /// similar to H3 histone, family 3B /// similar to H3 histone, family 3B /// similar to H3 histone, family 3B /// similar to Histone H3.3A CG5825-PB, isoform B	1.0892	0.067	0.1351	0.0343	No	1.1216	0.34	0.829	0.573	No	No
101782_at	---	---	0.9757	0.8097	0.0234	0.7174	No	1.2174	0.0503	0.0369	0.606	No	No
101783_i_at	---	---	1.069	0.3281	0.2163	0.8282	No	1.0144	0.841	0.801	0.453	No	No
101784_f_at	---	---	1.0859	0.0367	0.0032	0.9231	No	1.0894	0.272	0.0917	0.873	No	No
101786_at	Kcna4	potassium voltage-gated channel, shaker-related subfamily, member 4	0.9625	0.3823	0.7939	0.0491	No	0.9923	0.937	0.698	0.275	No	No
101787_f_at	Ccrn4l	CCR4 carbon catabolite repression 4-like (S. cerevisiae)	0.9653	0.5121	0.0008	0.0127	No	0.9168	0.275	0.0888	0.271	No	No
101788_f_at	Ifna1	interferon alpha family, gene 1	1.0617	0.2677	0.0759	0.0547	No	1.0197	0.517	0.227	0.0116	No	No
101789_i_at	Ifna4	interferon alpha family, gene 4	1.1642	0.0188	0.1974	0.0472	No	1.1621	0.0276	0.586	0.0725	No	No
101790_f_at	Ifna4	interferon alpha family, gene 4	1.0378	0.2649	0.6027	0.4128	No	1.0517	0.0758	0.47	0.228	No	No
101791_f_at	Ifna5	interferon alpha family, gene 5	0.9813	0.6157	0.7018	0.4444	No	0.9557	0.441	0.0671	0.512	No	No
101792_at	---	---	0.9716	0.6531	0.2977	0.291	No	1.01	0.883	0.264	0.272	No	No
101793_at	Fcgr1	Fc receptor, IgG, high affinity I	1.0182	0.7798	0.032	0.828	No	1.0168	0.718	0.025	0.816	No	No
101794_f_at	Defcr23	defensin-related cryptdin 23	0.9761	0.4438	0.193	0.0623	No	0.9858	0.695	0.346	0.104	No	No
101795_f_at	Defcr-rs12	defensin related cryptdin, related sequence 12	0.9274	0.103	0.2483	0.5206	No	0.8935	0.0283	0.426	0.231	No	No
101796_at	Pcdh7	protocadherin 7	0.9316	0.3067	0.9244	0.6661	No	0.9659	0.593	0.0507	0.562	No	No
101797_at	Six6	sine oculis-related homeobox 6 homolog (Drosophila)	0.9475	0.4696	0.0344	0.6604	No	0.9978	0.94	0.714	0.33	No	No
101798_at	---	---	1.0057	0.9582	0.0919	0.0937	No	1.0666	0.499	0.724	0.3	No	No
101799_at	---	---	1.1317	0.161	0.0015	0.4311	No	0.9945	0.693	0.001	0.315	No	No
101800_at	Fpr-rs2	formyl peptide receptor, related sequence 2	0.9989	0.9861	0.0558	0.2391	No	1.1114	0.111	0.0133	0.0336	No	No
101801_at	Fpr-rs3	formyl peptide receptor, related sequence 3	0.9818	0.6998	0.2946	0.6645	No	0.9463	0.4	0.222	0.303	No	No
101802_at	Fpr-rs4	formyl peptide receptor, related sequence 4	1.0198	0.6247	0.4559	0.3738	No	1.0001	0.925	0.324	0.638	No	No
101803_at	LOC546020	similar to Mucin-5AC (Mucin 5 subtype AC, tracheobronchial) (Tracheobronchial mucin) (TBM) (Major airway glycoprotein)	1.009	0.8829	0.0437	0.6173	No	1.1599	0.1	0.0597	0.0377	No	No
101804_at	---	---	0.9152	0.3518	0.8999	0.0763	No	0.8103	0.0101	0.0409	0.0365	No	No
101805_f_at	Nfil3	nuclear factor, interleukin 3, regulated	0.9632	0.41	0.021	0.88	No	0.9504	0.329	0.0832	0.596	No	No
101806_at	Galr3	galanin receptor 3	1.1029	0.1045	0.0626	0.9136	No	1.2139	0.0693	0.158	0.461	No	No
101807_at	Magea4	melanoma antigen, family A, 4	0.9854	0.843	0.9054	0.5528	No	0.9577	0.261	0.694	0.699	No	No
101808_at	Magea7	melanoma antigen, family A, 7	0.8894	0.0591	0.4298	0.1987	No	0.9964	0.94	0.125	0.933	No	No
101809_at	C1ql1	complement component 1, q subcomponent-like 1	1.0046	0.9597	0.0954	0.9684	No	0.9633	0.472	0.215	0.44	No	No
101810_at	Fshr	follicle stimulating hormone receptor	0.9692	0.3259	0.5517	0.0945	No	1.0013	0.968	0.927	0.681	No	No
101811_at	Atoh7	atonal homolog 7 (Drosophila)	1.1265	0.171	0.1443	0.5693	No	1.0056	0.94	0.152	0.563	No	No
101814_at	Gdf11	growth differentiation factor 11	0.931	0.0399	0.0005	0.792	No	0.9552	0.33	0.039	0.189	No	No
101815_at	Bmp10	bone morphogenetic protein 10	0.9619	0.2009	0.0258	0.2219	No	1.2588	0.03	0.0152	0.339	No	No
101816_at	Serpinb3a	serine (or cysteine) peptidase inhibitor, clade B (ovalbumin), member 3A	1.0193	0.3689	0.0175	0.4625	No	0.9778	0.822	0.0467	0.164	No	No
101820_at	LOC546644	similar to Lymphocyte antigen Ly-6G.1 precursor	1.0362	0.3317	0.0048	0.0704	No	1.3508	0.0021	0.221	0.007	No	No
101821_at	Chrm4	cholinergic receptor, muscarinic 4	0.8921	0.0705	0.4029	0.3066	No	1.0112	0.917	0.0252	0.585	No	No
101822_at	Mc3r	melanocortin 3 receptor	0.975	0.5585	0.0215	0.8789	No	0.9091	0.0185	0.0067	0.0055	No	No
101823_at	LOC547428	similar to TRAV19	0.99	0.9084	0.8123	0.4102	No	0.97	0.491	0.06	0.583	No	No
101824_at	LOC436539	similar to TRAV6-2	0.948	0.512	0.4346	0.6981	No	1.0128	0.761	0.257	0.084	No	No
101825_at	Nat3	N-acetyltransferase 3	0.9307	0.1319	0.01	0.3263	No	0.894	0.328	0.793	0.449	No	No
101826_at	LOC434035	Immunoglobulin kappa-chain VK-1	0.9922	0.8882	0.1321	0.4897	No	1.0106	0.808	0.0695	0.256	No	No
101827_at	Hpvc2	human papillomavirus 18 E5 central sequence motif gene 2	1.1104	0.0931	0.0001	0.1387	No	1.0754	0.18	0.0042	0.0144	No	No
101828_at	Ret	ret proto-oncogene	0.9679	0.6731	0.101	0.38	No	0.9337	0.175	0.0056	0.0916	No	No
101829_at	Chrm1	cholinergic receptor, muscarinic 1, CNS	1.0263	0.4469	0.013	0.4169	No	1.0416	0.176	0.0026	0.727	No	No
101830_at	Ryr3	ryanodine receptor 3	1.008	0.8422	0.0173	0.0261	No	1.0329	0.635	0.799	0.18	No	No
101831_at	Shh	sonic hedgehog	0.9779	0.4628	0.0041	0.016	No	1.0633	0.219	0.0668	0.505	No	No
101834_at	Mapk3	mitogen activated protein kinase 3	1.1097	0.0568	0.0146	0.4738	No	1.1601	0.0989	0.0631	0.165	No	No
101835_at	Lrmp	lymphoid-restricted membrane protein	0.9837	0.7695	0.3203	0.4036	No	0.9868	0.808	0.341	0.597	No	No
101836_at	Ppm1b	protein phosphatase 1B, magnesium dependent, beta isoform	1.004	0.951	0.0052	0.5526	No	0.9795	0.851	0.0104	0.95	No	No
101837_g_at	Ppm1b	protein phosphatase 1B, magnesium dependent, beta isoform	0.9702	0.3832	0.0008	0.2516	No	0.9785	0.513	0.0073	0.0935	No	No
101838_r_at	Ppm1b	protein phosphatase 1B, magnesium dependent, beta isoform	0.9206	0.0647	0.0057	0.748	No	0.9316	0.178	0.0091	0.486	No	No
101839_at	1700074P13Rik	RIKEN cDNA 1700074P13 gene	1.0454	0.5031	0.1399	0.9942	No	1.0255	0.723	0.988	0.679	No	No
101840_at	Egfr	epidermal growth factor receptor	0.9139	0.1611	0.1157	0.7845	No	0.9426	0.255	0.0694	0.789	No	No
101841_at	Egfr	epidermal growth factor receptor	0.9709	0.3332	0.7684	0.2305	No	0.8888	0.0713	0.0564	0.0222	No	No
101842_g_at	Egfr	epidermal growth factor receptor	1.0061	0.8749	0.0302	0.1267	No	0.9648	0.358	0.0211	0.314	No	No
101843_at	Sh2bpsm1	SH2-B PH domain containing signaling mediator 1	0.9627	0.3027	0.0447	0.4728	No	0.9532	0.258	0.623	0.639	No	No
101844_at	Pipox	pipecolic acid oxidase	1.0356	0.6548	0.7045	0.3359	No	1.0265	0.756	0.667	0.894	No	No

101845_s_at	Csprs /// Sp100-rs /// LOC621807 /// LOC624074 /// LOC665317 /// LOC665338 /// LOC665378 /// LOC673083 /// LOC673105 /// LOC673135 /// LOC673206 /// LOC673671 /// LOC673849 /// LOC673911 /// LOC673925	component of Sp100-rs /// similar to component of Sp100-rs /// similar to component of Sp100-rs /// similar to component of Sp100-rs /// similar to component of Sp100-rs /// similar to component of Sp100-rs /// similar to component of Sp100-rs /// similar to component of Sp100-rs /// similar to component of Sp100-rs /// similar to component of Sp100-rs /// similar to component of Sp100-rs /// similar to component of Sp100-rs	1.0651	0.2076	0.061	0.0261	No	1.0686	0.641	0.96	0.256	No	No
101846_r_at	Csprs /// LOC665317 /// LOC665338 /// LOC673925 /// LOC673997	component of Sp100-rs /// similar to component of Sp100-rs /// similar to component of Sp100-rs /// similar to component of Sp100-rs /// similar to component of Sp100-rs	1.0247	0.7534	0.8467	0.3913	No	1.1841	0.254	0.416	0.144	No	No
101847_at	Sp100	nuclear antigen Sp100	0.9542	0.2046	0.0425	0.6628	No	0.8044	0.0155	0.043	0.0753	No	No
101848_g_at	Sp100	nuclear antigen Sp100	1.0025	0.9686	0.643	0.3503	No	1.0826	0.579	0.294	0.921	No	No
101850_at	Spa17	sperm autoantigenic protein 17	0.8869	0.1558	0.0743	0.0518	No	0.9596	0.654	0.0503	0.167	No	No
101851_at	Cd200	Cd200 antigen	0.9146	0.4056	0.3473	0.7783	No	0.9878	0.928	0.648	0.224	No	No
101853_f_at	Cfh	complement component factor h	0.9055	0.1046	0.0058	0.121	No	0.8794	0.0313	0.463	0.161	No	No
101854_r_at	---	---	0.9709	0.4837	0.0209	0.0722	No	1.0166	0.583	0.762	0.0022	No	No
101855_at	Mtap6	microtubule-associated protein 6	1.0047	0.7895	0.2565	0.152	No	1.0325	0.52	0.0096	0.453	No	No
101856_at	Kctd17	potassium channel tetramerisation domain containing 17	1.0065	0.7334	0	0.0035	No	0.9968	0.772	0.0047	0.0635	No	No
101857_at	Srpk2	serine/arginine-rich protein specific kinase 2	0.9807	0.8445	0.1446	0.5822	No	0.9834	0.626	0.0057	0.852	No	No
101858_at	Rfng	radical fringe gene homolog (Drosophila)	0.9187	0.0154	0.3607	0.8042	No	0.9069	0.242	0.533	0.76	No	No
101859_at	Aqp7	aquaporin 7	0.9987	0.9768	0.011	0.4901	No	0.9827	0.617	0.0075	0.405	No	No
101860_at	Gmfb	glia maturation factor, beta	0.9857	0.5781	0.4668	0.0696	No	0.9835	0.678	0.031	0.14	No	No
101861_at	Sgce	sarcoglycan, epsilon	0.9865	0.6131	0.0025	0.092	No	0.9577	0.12	0.0049	0.0263	No	No
101862_at	Cyp2b9	cytochrome P450, family 2, subfamily b, polypeptide 9	0.9878	0.7642	0.2457	0.0156	No	1.0379	0.314	0.27	0.0413	No	No
101863_at	Cdc91l1	CDC91 cell division cycle 91-like 1 (S. cerevisiae)	1.1098	0.1911	0.4861	0.1755	No	1.2179	0.124	0.173	0.0697	No	No
101864_at	Actl7b	actin-like 7b	0.9768	0.6549	0.8467	0.6373	No	1.0274	0.871	0.011	0.62	No	No
101865_at	Pip5k2a	phosphatidylinositol-4-phosphate 5-kinase, type II, alpha	1.0306	0.4343	0.3649	0.4073	No	1.025	0.503	0.0291	0.116	No	No
101866_at	Arfrp1	ADP-ribosylation factor related protein 1	1.0454	0.3326	0.2187	0.1035	No	1.012	0.87	0.307	0.264	No	No
101867_at	Gpam	glycerol-3-phosphate acyltransferase, mitochondrial	0.9298	0.0905	0.0899	0.7458	No	0.9414	0.25	0.489	0.448	No	No
101868_i_at	H2-DMb2	histocompatibility 2, class II, locus Mb2	1.0003	0.9976	0.7791	0.2193	No	1.0203	0.876	0.718	0.13	No	No
101869_s_at	Hbb-b1 /// Hbb-b2	hemoglobin, beta adult major chain /// hemoglobin, beta adult minor chain	1.0248	0.4395	0.0011	0.3972	No	0.9899	0.615	0.0005	0.943	No	No
101870_at	Igh-4	immunoglobulin heavy chain 4 (serum IgG1)	1.0116	0.8279	0.8851	0.8855	No	1.1469	0.0729	0.641	0.225	No	No
101871_f_at	Igh-4	immunoglobulin heavy chain 4 (serum IgG1)	1.0065	0.9173	0.0067	0.165	No	0.9745	0.395	0.0003	0.74	No	No
101872_at	Gsta2 /// LOC625953	glutathione S-transferase, alpha 2 (Yc2) /// similar to Glutathione S-transferase Ya chain (GST class-alpha) (Ya1)	0.9915	0.9011	0.7917	0.46	No	0.9584	0.599	0.0233	0.366	No	No
101873_at	Jak3	Janus kinase 3	0.9463	0.2472	0.2344	0.1137	No	1.1514	0.0103	0.0197	0.0152	No	No
101874_s_at	Prlpe	prolactin-like protein E	0.9154	0.3739	0.2079	0.3032	No	1.0466	0.326	0.012	0.0981	No	No
101875_at	Ppp2r5d	protein phosphatase 2, regulatory subunit B (B56), delta isoform	0.9746	0.6137	0.6707	0.4602	No	0.9886	0.858	0.118	0.724	No	No
101876_s_at	H2-T10 /// H2-T22 /// H2-T9	histocompatibility 2, T region locus 10 /// histocompatibility 2, T region locus 22 /// histocompatibility 2, T region locus 9	0.9792	0.3003	0.0036	0.4839	No	1.0037	0.856	0.234	0.0395	No	No
101877_at	Slc31a1	solute carrier family 31, member 1	1.0815	0.03	0.6272	0.0904	No	1.0728	0.064	0.187	0.932	No	No
101878_at	LOC669118	similar to B-cell differentiation antigen CD72 (Lyb-2) (Antigen Ly-32)	0.9761	0.5112	0.0532	0.3191	No	0.8558	0.0007	0.0001	0.0003	No	No
101879_s_at	Crry	complement receptor related protein	0.9005	0.0386	0.0193	0.0644	No	0.9583	0.424	0.383	0.186	No	No
101880_at	Col18a1	procollagen, type XVIII, alpha 1	1.0334	0.637	0.0095	0.5999	No	1.0364	0.635	0.047	0.466	No	No
101881_g_at	Col18a1	procollagen, type XVIII, alpha 1	1.0038	0.8956	0.0072	0.5747	No	1.0272	0.447	0.11	0.289	No	No
101882_s_at	Col18a1	procollagen, type XVIII, alpha 1	0.9456	0.2125	0.0089	0.8217	No	1.0119	0.766	0.0054	0.709	No	No
101883_s_at	Xlr3a /// Xlr3b /// MGC76689 /// LOC630164	X-linked lymphocyte-regulated 3A /// X-linked lymphocyte-regulated 3B /// hypothetical protein LOC574437 /// similar to X-LINKED LYMPHOCYTE-REGULATED PROTEIN 3A (XLR RELATED PROTEIN A12)	0.7964	0.0192	0.0196	0.0439	No	0.8171	0.0041	0.0041	0.0034	No	No
101884_at	Xlr4a	X-linked lymphocyte-regulated 4A	0.8197	0.1388	0.1353	0.5392	No	0.8045	0.159	0.114	0.386	No	No
101885_at	Gas5	growth arrest specific 5	0.997	0.9732	0.898	0.9239	No	0.8746	0.0498	0.695	0.236	No	No
101886_f_at	H2-D1	histocompatibility 2, D region locus 1	0.9737	0.5333	0.1646	0.0223	No	1.0248	0.554	0.676	0.0463	No	No
101887_at	Agt	angiotensinogen (serpin peptidase inhibitor, clade A, member 8)	0.9208	0.0767	0.0078	0.181	No	1.0385	0.0902	0.0002	0.161	No	No
101888_at	Rora	RAR-related orphan receptor alpha	0.9184	0.5734	0.1718	0.2856	No	0.8864	0.198	0.102	0.342	No	No
101889_s_at	Rora	RAR-related orphan receptor alpha	1.0004	0.9921	0.9796	0.3598	No	1.0362	0.252	0.46	0.0373	No	No
101890_f_at	Dnajc2	DnaJ (Hsp40) homolog, subfamily C, member 2	0.9722	0.4987	0.0011	0.5657	No	0.9489	0.18	0.0039	0.99	No	No
101891_at	Hsd17b2	hydroxysteroid (17-beta) dehydrogenase 2	0.9967	0.9213	0.0023	0.1512	No	1.0468	0.0946	0.0005	0.0187	No	No
101892_f_at	Fts	fused toes	0.9513	0.5584	0.0048	0.9973	No	0.9764	0.77	0.002	0.397	No	No
101893_r_at	Fts	fused toes	0.9695	0.2965	0.0073	0.0829	No	1.1517	0.429	0.72	0.476	No	No
101894_s_at	Fts	fused toes	0.9299	0.3835	0.6521	0.9734	No	0.8985	0.221	0.899	0.756	No	No
101896_at	Cd1d2	CD1d2 antigen	1.0479	0.5794	0.8546	0.4202	No	1.1222	0.091	0.235	0.752	No	No
101897_g_at	Cd1d1 /// Cd1d2	CD1d1 antigen /// CD1d2 antigen	1.0204	0.2501	0.0394	0.0378	No	1.0524	0.0955	0.0177	0.0768	No	No
101898_s_at	H2-Q10	histocompatibility 2, Q region locus 10	1.1001	0.221	0.5672	0.3192	No	0.9909	0.154	0	0.305	No	No
101899_at	F2	coagulation factor II	1.0026	0.9729	0.0135	0.289	No	1.0165	0.749	0.0274	0.712	No	No
101900_at	Cdkn2b	cyclin-dependent kinase inhibitor 2B (p15, inhibits CDK4)	0.9975	0.9666	0.08	0.3527	No	1.2534	0.0334	0.0014	0.0165	No	No
101901_at	Mpdu1	mannose-P-dolichol utilization defect 1	0.9611	0.2048	0.1615	0.0554	No	0.9787	0.399	0.0582	0.0067	No	No

101902_at	Rbpsuh	recombining binding protein suppressor of hairless (Drosophila)	0.8551	0.0949	0.1265	0.3262	No	0.9378	0.318	0.0287	0.237	No	No
101903_at	Smyd1	SET and MYND domain containing 1	0.9192	0.1045	0.1389	0.486	No	0.899	0.125	0.0862	0.696	No	No
101904_at	Smyd1	SET and MYND domain containing 1	0.9832	0.8078	0.5788	0.5459	No	1.0017	0.963	0.895	0.618	No	No
101905_at	Itch	itchy	1.0236	0.2497	0.001	0.0006	No	0.9285	0.357	0.0007	0.328	No	No
101906_at	Smc4l1 /// LOC630556	SMC4 structural maintenance of chromosomes 4-like 1 (yeast) /// similar to SMC4 structural maintenance of chromosomes 4-like 1	1.0065	0.8974	0.0054	0.7813	No	0.9656	0.659	0.0202	0.83	No	No
101907_s_at	Ceacam2	CEA-related cell adhesion molecule 2	1.0114	0.8523	0.0059	0.8738	No	0.9602	0.293	0.147	0.0474	No	No
101908_s_at	Ceacam1 /// Ceacam2	CEA-related cell adhesion molecule 1 /// CEA-related cell adhesion molecule 2	0.8677	0.0311	0.5483	0.5023	No	0.8732	0.0104	0.531	0.0322	No	No
101909_f_at	Mup3	major urinary protein 3	0.9697	0.1078	0.0315	0.7596	No	0.9905	0.8	0.0693	0.468	No	No
101910_f_at	Mup3	major urinary protein 3	0.9569	0.4708	0.2104	0.451	No	0.9312	0.241	0.905	0.111	No	No
101912_at	1100001G20 Rik	RIKEN cDNA 1100001G20 gene	0.9692	0.4317	0.4073	0.0575	No	1.1102	0.0366	0.0085	0.0071	No	No
101913_at	Clcn5	chloride channel 5	0.9354	0.0718	0.0038	0.1333	No	0.9766	0.773	0.0093	0.0568	No	No
101914_at	Angel2	angel homolog 2 (Drosophila)	0.8728	0.0238	0.0177	0.3311	No	0.8982	0.0177	0.0043	0.199	No	No
101916_at	Llgl2	lethal giant larvae homolog 2 (Drosophila)	0.9873	0.7027	0.0007	0.0118	No	1.0065	0.516	0.0016	0.0145	No	No
101917_at	Il2ra	interleukin 2 receptor, alpha chain	1.0344	0.5226	0.3716	0.0552	No	1.1088	0.0586	0.948	0.0028	No	No
101918_at	Tgfb1	transforming growth factor, beta 1	0.9877	0.5749	0	0.2865	No	1.1415	0.154	0.0003	0.0418	No	No
101919_at	Zfx	zinc finger protein X-linked	0.9779	0.4094	0.0005	0.4334	No	0.9914	0.938	0.0007	0.433	No	No
101920_at	Pole2	polymerase (DNA directed), epsilon 2 (p59 subunit)	1.1401	0.0441	0.1998	0.4362	No	1.1609	0.0091	0.0028	0.0047	No	No
101921_at	Rab4a	RAB4A, member RAS oncogene family	0.898	0.0234	0.1133	0.8647	No	0.9627	0.101	0.0064	0.151	No	No
101922_at	Kdelr2	KDEL (Lys-Asp-Glu-Leu) endoplasmic reticulum protein retention receptor 2	0.959	0.5493	0.0016	0.0488	No	0.9999	0.328	0.0003	0.0163	No	No
101923_at	Pla2g7	phospholipase A2, group VII (platelet-activating factor acetylhydrolase, plasma)	0.9687	0.4776	0.0032	0.0713	No	0.9576	0.499	0.553	0.434	No	No
101924_at	Rad51l3	RAD51-like 3 (S. cerevisiae)	0.9773	0.6096	0.7157	0.1761	No	1.1102	0.107	0.0412	0.234	No	No
101926_at	Pim2	proviral integration site 2	1.1072	0.1757	0.4546	0.1994	No	0.9848	0.716	0.0069	0.26	No	No
101927_at	Prkar1b	protein kinase, cAMP dependent regulatory, type I beta	0.9732	0.6054	0.456	0.7291	No	1.0023	0.973	0.283	0.479	No	No
101928_at	Serpinf2	serine (or cysteine) peptidase inhibitor, clade F, member 2	1.0769	0.2009	0.3011	0.0537	No	1.0669	0.122	0.0349	0.0101	No	No
101929_at	Dynll2	dynein light chain LC8-type 2	1.0263	0.7648	0.0229	0.9435	No	1.0351	0.657	0.0051	0.665	No	No
101930_at	Nfix	nuclear factor I/X	1.1489	0.024	0.8422	0.7543	No	1.1355	0.149	0.331	0.737	No	No
101931_at	Ptdss1	phosphatidylserine synthase 1	1.0289	0.636	0.0092	0.66	No	1.0164	0.737	0.0249	0.753	No	No
101932_at	Ptpre	protein tyrosine phosphatase, receptor type, E	0.9682	0.5341	0.0081	0.4002	No	0.9835	0.184	0	0.0086	No	No
101933_at	Rab10	RAB10, member RAS oncogene family	0.9911	0.8877	0.1796	0.4523	No	0.9741	0.362	0.0103	0.971	No	No
101934_at	Fez2	fasciculation and elongation protein zeta 2 (zygin II)	0.996	0.865	0.2894	0.062	No	0.9734	0.428	0.0921	0.0445	No	No
101936_at	Clk4	CDC like kinase 4	0.9424	0.1953	0.0051	0.0467	No	0.8875	0.306	0.374	0.266	No	No
101937_s_at	Clk4	CDC like kinase 4	1.0359	0.5514	0.3935	0.1919	No	1.0264	0.519	0.0372	0.0506	No	No
101938_at	Pabpc2	poly A binding protein, cytoplasmic 2	1.0079	0.9286	0.8409	0.5714	No	0.9965	0.924	0.0632	0.0525	No	No
101939_at	Rnf141	ring finger protein 141	0.9559	0.2128	0.04	0.7203	No	0.9552	0.211	0.0211	0.725	No	No
101940_at	Smpd2	sphingomyelin phosphodiesterase 2, neutral	1.0295	0.452	0.6413	0.569	No	1.0661	0.0499	0.4	0.0122	No	No
101942_at	Sfrs14	splicing factor, arginine/serine-rich 14	0.9685	0.3717	0.4395	0.3075	No	0.9991	0.982	0.0096	0.398	No	No
101943_at	Tceb3	transcription elongation factor B (SIII), polypeptide 3	0.939	0.299	0.4564	0.4722	No	0.9542	0.414	0.0198	0.787	No	No
101944_at	Lypla1	lysophospholipase 1	1.0234	0.5079	0.0014	0.2969	No	1.0028	0.806	0.0001	0.461	No	No
101945_g_at	Lypla1	lysophospholipase 1	1.0293	0.5081	0.8204	0.6848	No	0.9747	0.765	0.734	0.674	No	No
101946_at	Lypla1	lysophospholipase 1	1.0718	0.1013	0.5674	0.0849	No	1.0968	0.0357	0.225	0.154	No	No
101947_at	Akap8l	A kinase (PRKA) anchor protein 8-like	0.9272	0.0295	0.0019	0.1071	No	1.0705	0.434	0.0729	0.492	No	No
101948_at	Lamb1-1	laminin B1 subunit 1	1.0693	0.3442	0.0009	0.7627	No	1.0133	0.902	0.0127	0.682	No	No
101949_at	Pmm2	phosphomannomutase 2	0.9575	0.1792	0.0008	0.2439	No	0.9506	0.528	0.625	0.588	No	No
101950_at	Gipc2	GIPC PDZ domain containing family, member 2	0.9687	0.4175	0.3884	0.2317	No	1.0945	0.212	0.0166	0.986	No	No
101952_at	Setd4	SET domain containing 4	1.043	0.1704	0.0691	0.1821	No	1.0869	0.028	0.665	0.0076	No	No
101953_at	Edg1	endothelial differentiation sphingolipid G-protein-coupled receptor 1	0.9956	0.922	0.4966	0.9179	No	0.9867	0.879	0.806	0.569	No	No
101954_at	H2afz	H2A histone family, member Z	1.1783	0.0217	0.0046	0.8332	No	1.1219	0.132	0.275	0.831	No	No
101955_at	Hspa5	heat shock 70kD protein 5 (glucose-regulated protein)	1.1957	0.0116	0.0042	0.1516	No	1.1193	0.0237	0.004	0.816	No	No
101956_at	Ckap4	cytoskeleton-associated protein 4	1.0359	0.2781	0	0.0076	No	1.0231	0.489	0.0001	0.461	No	No
101957_f_at	Parp1	poly (ADP-ribose) polymerase family, member 1	1.0126	0.7758	0.1116	0.0397	No	0.9893	0.745	0.555	0.0406	No	No
101958_f_at	LOC664889 /// LOC672007 /// LOC674691	similar to Transcription factor Dp-1 (E2F dimerization partner 1) (DRTF1-polypeptide 1) /// similar to Transcription factor Dp-1 (E2F dimerization partner 1) (DRTF1-polypeptide 1) /// similar to Transcription factor Dp-1 (E2F dimerization partner 1) (DRTF1-polypeptide 1)	1.0653	0.3337	0.184	0.1306	No	1.0328	0.466	0.0892	0.181	No	No
101959_r_at	LOC664889 /// LOC672007 /// LOC674691	similar to Transcription factor Dp-1 (E2F dimerization partner 1) (DRTF1-polypeptide 1) /// similar to Transcription factor Dp-1 (E2F dimerization partner 1) (DRTF1-polypeptide 1) /// similar to Transcription factor Dp-1 (E2F dimerization partner 1) (DRTF1-polypeptide 1)	1.0866	0.4334	0.3307	0.4616	No	1.0669	0.529	0.907	0.717	No	No
101960_at	D10Wsu52e	DNA segment, Chr 10, Wayne State University 52, expressed	1.0796	0.1966	0.5106	0.2465	No	0.9902	0.917	0.175	0.0125	No	No
101961_at	Bub3	budding uninhibited by benzimidazoles 3 homolog (S. cerevisiae)	1.1078	0.1076	0.4854	0.5481	No	1.0959	0.147	0.587	0.131	No	No
101962_at	Ddx17	DEAD (Asp-Glu-Ala-Asp) box polypeptide 17	1.0701	0.0343	0.0003	0.0051	No	1.0139	0.592	0.0094	0.0973	No	No
101963_at	Ctsl	cathepsin L	0.95	0.5922	0.5487	0.2283	No	0.9153	0.121	0.434	0.0322	No	No
101964_at	Tkt	transketolase	1.0734	0.1366	0.0069	0.5299	No	1.0761	0.0166	0.0002	0.477	No	No
101965_at	Rnf13	ring finger protein 13	0.8994	0.0007	0.0028	0.8035	Yes	0.8871	0.004	0.031	0.713	Yes	Yes
101966_s_at	Rnf13	ring finger protein 13	0.9546	0.407	0.0501	0.0903	No	0.9516	0.308	0.512	0.211	No	No
101967_at	Sdf2	stromal cell derived factor 2	0.9437	0.3563	0.9893	0.8866	No	0.9243	0.102	0.0856	0.0384	No	No
101968_at	Odf1	outer dense fiber of sperm tails 1	0.954	0.1776	0.41	0.2136	No	0.969	0.665	0.596	0.716	No	No
101969_at	Nbl1	neuroblastoma, suppression of tumorigenicity 1	0.9928	0.8789	0.0016	0.2156	No	0.946	0.154	0.0003	0.0135	No	No
101970_at	Lrig1	leucine-rich repeats and immunoglobulin-like domains 1	0.9545	0.3402	0.6154	0.1266	No	0.9346	0.0218	0.853	0.0008	No	No
101971_at	2500002L14 Rik	RIKEN cDNA 2500002L14 gene	1.0268	0.6765	0.361	0.6508	No	1.0064	0.839	0.175	0.444	No	No
101972_at	Napsa	napsin A aspartic peptidase	0.9934	0.925	0.5369	0.7366	No	1.0212	0.814	0.445	0.807	No	No
101973_at	Cited2 /// LOC671495	Cbp/p300-interacting transactivator, with Glu/Asp-rich carboxy-terminal domain, 2 /// similar to Cbp/p300-interacting transactivator, with Glu/Asp-rich carboxy-terminal domain, 2	0.8835	0.0963	0.0392	0.4915	No	0.895	0.0429	0.0373	0.602	No	No
101975_at	Dlk1	delta-like 1 homolog (Drosophila)	0.9165	0.0016	0	0.0203	Yes	0.8739	0.0103	0.0001	0.875	No	Yes
101976_at	Cops4	COP9 (constitutive photomorphogenic) homolog, subunit 4 (Arabidopsis thaliana)	1.2031	0.0701	0.1668	0.6872	No	1.0621	0.102	0.0049	0.964	No	No
101977_at	Nudt3	nudix (nucleotide diphosphate linked moiety X)-type motif 3	1.0701	0.2433	0.1346	0.4633	No	1.0561	0.264	0.217	0.981	No	No
101978_at	Phospho2	phosphatase, orphan 2	0.983	0.8181	0.8223	0.8677	No	0.9804	0.6	0.0518	0.596	No	No
101979_at	Gadd45g	growth arrest and DNA-damage-inducible 45 gamma	0.9002	0.0415	0.1013	0.057	No	1.0526	0.438	0.0083	0.0847	No	No
101980_at	Sub1	SUB1 homolog (S. cerevisia	1.0505	0.5291	0.0089	0.0672	No	0.9531	0.367	0.0057	0.076	No	No
101981_at	Nr1h2	nuclear receptor subfamily 1, group H, member 2	0.9933	0.8852	0.3734	0.3454	No	1.0401	0.51	0.185	0.392	No	No
101982_at	Vasp	vasodilator-stimulated phosphoprotein	1.0586	0.1565	0.0861	0.1297	No	0.997	0.795	0.018	0.0349	No	No
101984_at	Atox1	ATX1 (antioxidant protein 1) homolog 1 (yeast)	1.0008	0.9788	0.011	0.082	No	0.9944	0.959	0.0216	0.0692	No	No
101985_at	Plg	plasminogen	1.0664	0.3328	0.1565	0.5918	No	1.0634	0.311	0.209	0.0487	No	No
101989_at	Uqcrc1	ubiquinol-cytochrome c reductase core protein 1	0.942	0.0959	0.0389	0.051	No	0.9765	0.259	0.0014	0.0087	No	No
101990_at	Ldhb	lactate dehydrogenase B	0.9175	0.3198	0.1391	0.9719	No	0.9176	0.276	0.102	0.722	No	No
101991_at	Fmo1	flavin containing monooxygenase 1	0.9037	0.1039	0.0025	0.6614	No	0.9567	0.418	0.0029	0.832	No	No
101992_at	Psmb6	proteasome (prosome, macropain) subunit, beta type 6	1.1214	0.0149	0.3678	0.7417	No	1.0748	0.276	0.517	0.637	No	No
101993_at	Tnc	tenascin C	0.9173	0.0179	0.0006	0.0075	No	0.9095	0.152	0.068	0.188	No	No
101995_at	Sqstm1	sequestosome 1	0.8826	0.0087	0.005	0.1629	Yes	0.9003	0.0293	0.0035	0.257	No	Yes

101996_at	Ptpn2	protein tyrosine phosphatase, non-receptor type 2	0.9837	0.656	0.0139	0.3218	No	1.0233	0.757	0.0193	0.0398	No	No
101997_at	Atg12	autophagy-related 12 (yeast)	0.9477	0.2861	0.0052	0.1127	No	1.0314	0.447	0.0094	0.847	No	No
101998_at	4833420G17 Rik	RIKEN cDNA 4833420G17 gene	0.8833	0.0866	0.0044	0.511	No	0.96	0.0772	0.0004	0.0097	No	No
102000_f_at	Uqcrc2	ubiquinol cytochrome c reductase core protein 2	1.0214	0.7782	0.0149	0.2296	No	0.9927	0.803	0.0149	0.272	No	No
102001_at	Rrm2	ribonucleotide reductase M2	1.1019	0.0982	0.0331	0.12	No	1.0291	0.663	0.433	0.273	No	No
102002_at	Ubqln2	ubiquilin 2	1.0701	0.3873	0.0011	0.4728	No	1.0919	0.233	0.0012	0.49	No	No
102003_at	Gbf1	golgi-specific brefeldin A-resistance factor 1	0.9752	0.5819	0.2281	0.8178	No	0.8998	0.0179	0.0071	0.0086	No	No
102004_at	Acaa1b	acetyl-Coenzyme A acyltransferase 1B	1.0386	0.2631	0.0059	0.3708	No	1.0836	0.0953	0.0273	0.907	No	No
102007_at	Hccs	holocytochrome c synthetase	1.0161	0.703	0.0287	0.1001	No	1.0196	0.585	0.0038	0.19	No	No
102009_at	Cyfip2	cytoplasmic FMR1 interacting protein 2	0.9834	0.7387	0.0029	0.5409	No	1.0063	0.948	0.0461	0.19	No	No
102010_at	Abcf2	ATP-binding cassette, sub-family F (GCN20), member 2	0.9421	0.1188	0.0675	0.284	No	1.0356	0.687	0.287	0.153	No	No
102011_at	2610507L03 Rik	RIKEN cDNA 2610507L03 gene	0.9551	0.1572	0.0212	0.0571	No	0.8717	0.0014	0.0392	0.0104	Yes	No
102012_at	Scap2	src family associated phosphoprotein 2	0.9927	0.4908	0.0001	0.037	No	0.9775	0.601	0.0005	0.0252	No	No
102013_at	Rdh16	retinol dehydrogenase 16	0.9715	0.4542	0.0071	0.2545	No	0.9687	0.617	0.165	0.267	No	No
102014_at	Homer3	homer homolog 3 (Drosophila)	0.914	0.1515	0.6286	0.1699	No	1.0212	0.507	0.0677	0.0219	No	No
102015_at	Ddx49	DEAD (Asp-Glu-Ala-Asp) box polypeptide 49	1.0256	0.6021	0.0441	0.1679	No	1.0368	0.455	0.006	0.616	No	No
102016_at	Cidec	cell death-inducing DFFA-like effector c	1.0082	0.9242	0.7058	0.2321	No	1.0737	0.604	0.912	0.419	No	No
102017_at	Prpf4b	PRP4 pre-mRNA processing factor 4 homolog B (yeast)	1.1095	0.4871	0.0122	0.2743	No	0.9797	0.825	0.0092	0.236	No	No
102018_at	Cnot3	CCR4-NOT transcription complex, subunit 3	0.9472	0.0657	0.0001	0.0076	No	1.1354	0.256	0.536	0.102	No	No
102019_at	Mrpl13	mitochondrial ribosomal protein L13	1.0612	0.2108	0.0535	0.3021	No	1.079	0.0418	0.789	0.647	No	No
102020_at	Kcnk3	potassium channel, subfamily K, member 3	0.9947	0.948	0.1353	0.8169	No	0.9417	0.3	0.198	0.346	No	No
102021_at	Il4ra	interleukin 4 receptor, alpha	0.9238	0.0609	0.0194	0.5278	No	0.9523	0.396	0.139	0.326	No	No
102022_at	Mcee	methylmalonyl CoA epimerase	0.9052	0.0694	0.0234	0.2301	No	0.9128	0.0233	0.0282	0.742	No	No
102024_at	Ncoa3	nuclear receptor coactivator 3	0.9239	0.0183	0.0282	0.0607	No	0.8712	0.237	0.125	0.45	No	No
102025_at	Cxcl13	chemokine (C-X-C motif) ligand 13	1.0471	0.6002	0.3037	0.7095	No	0.9861	0.924	0.749	0.717	No	No
102026_s_at	Chkb	choline kinase beta	0.9693	0.5338	0.3027	0.8568	No	0.9594	0.553	0.566	0.368	No	No
102027_s_at	Chkb	choline kinase beta	0.9584	0.4917	0.2512	0.6948	No	0.9438	0.0626	0.0047	0.453	No	No
102028_at	Rassf5	Ras association (RalGDS/AF-6) domain family 5	0.9748	0.1965	0.0009	0.0086	No	0.9542	0.207	0.0008	0.373	No	No
102029_at	Il16	interleukin 16	0.9983	0.9781	0.0908	0.4641	No	0.9493	0.316	0.0541	0.946	No	No
102030_at	4833408C14 Rik	RIKEN cDNA 4833408C14 gene	0.9748	0.7964	0.6224	0.7985	No	0.9137	0.391	0.702	0.922	No	No
102031_at	Rnaseh1	ribonuclease H1	0.9388	0.2884	0.9078	0.4052	No	1.0022	0.965	0.697	0.704	No	No
102032_at	Twsg1	twisted gastrulation homolog 1 (Drosophila)	0.9759	0.4423	0.4184	0.829	No	0.9645	0.218	0.0987	0.468	No	No
102033_at	Tesk1	testis specific protein kinase 1	1.0176	0.3907	0.0011	0.0116	No	0.9733	0.239	0.0022	0.0972	No	No
102035_at	Tpmt	thiopurine methyltransferase	0.9792	0.7666	0.9453	0.9523	No	0.9871	0.578	0.0741	0.0862	No	No
102036_at	Thap7	THAP domain containing 7	0.9699	0.5867	0.0123	0.3744	No	0.9212	0.171	0.0767	0.0548	No	No
102037_at	Mapre2	microtubule-associated protein, RP/EB family, member 2	1.065	0.2784	0.0358	0.5784	No	1.0031	0.949	0.903	0.701	No	No
102038_at	Lnx1	ligand of numb-protein X 1	0.979	0.6329	0.2356	0.9585	No	0.959	0.646	0.425	0.579	No	No
102039_at	Gtf2h4	general transcription factor II H, polypeptide 4	1.0031	0.9514	0.0142	0.8031	No	0.9513	0.283	0.0022	0.763	No	No
102040_at	Gprk6	G protein-coupled receptor kinase 6	1.0034	0.9325	0.8205	0.1472	No	0.8922	0.0022	0.371	0.0022	No	No
102041_at	Myom2	myomesin 2	0.7529	0.0012	0.0055	0.0023	No	0.8355	0.018	0.0048	0.0204	No	No
102042_at	1810037C20 Rik	RIKEN cDNA 1810037C20 gene	0.8888	0.0442	0.0418	0.806	No	0.9133	0.0144	0.0004	0.119	No	No
102043_at	Tuft1	tuftelin 1	1.0466	0.2902	0.0108	0.1038	No	0.949	0.248	0.0977	0.831	No	No
102044_at	Wisp1	WNT1 inducible signaling pathway protein 1	0.9758	0.7683	0.7227	0.8219	No	0.9727	0.536	0.128	0.464	No	No
102046_at	Fusip1	FUS interacting protein (serine-arginine rich) 1	1.101	0.4101	0.0588	0.3169	No	1.0939	0.226	0.0159	0.848	No	No
102047_at	Nmt1	N-myristoyltransferase 1	1.0396	0.3918	0.0096	0.0954	No	1.0867	0.0332	0.001	0.491	No	No
102048_at	Ankrd1	ankyrin repeat domain 1 (cardiac muscle)	0.8272	0.0113	0.0354	0.0452	No	0.8044	0.0085	0.0157	0.021	Yes	Yes
102049_at	Pdk4	pyruvate dehydrogenase kinase, isoenzyme 4	0.7546	0.0041	0.4311	0.043	Yes	0.7913	0.0005	0.026	0.001	No	No
102050_at	Nfatc3	nuclear factor of activated T-cells, cytoplasmic, calcineurin-dependent 3	0.9772	0.5698	0.0011	0.293	No	1.0349	0.45	0.0033	0.826	No	No
102052_at	Abhd5	abhydrolase domain containing 5	0.9373	0.1605	0.1466	0.034	No	0.9221	0.0799	0.0044	0.034	No	No
102053_at	Plscr2	phospholipid scramblase 2	0.8745	0.0056	0.1784	0.5053	Yes	0.9867	0.762	0.0022	0.596	No	No
102054_at	Ankfy1	ankyrin repeat and FYVE domain containing 1	0.9848	0.4097	0.0023	0.0009	No	1.0235	0.169	0.0005	0.0047	No	No
102056_f_at	2610002J02 Rik	RIKEN cDNA 2610002J02 gene	0.9813	0.8304	0.0326	0.0517	No	1.0373	0.876	0.0548	0.0832	No	No
102057_r_at	2610002J02 Rik	RIKEN cDNA 2610002J02 gene	1.0451	0.5894	0.2942	0.6531	No	1.1243	0.166	0.83	0.497	No	No
102058_at	Mrpl9	mitochondrial ribosomal protein L9	1.0153	0.7447	0.9868	0.0278	No	0.9764	0.591	0.351	0.0101	No	No
102059_at	Nicn1	nicolin 1	1.0234	0.3795	0.1251	0.3786	No	0.9946	0.809	0.054	0.0572	No	No
102060_at	Golga4	golgi autoantigen, golgin subfamily a, 4	1.039	0.637	0.5099	0.4793	No	1.132	0.0006	0.0007	0.022	Yes	No
102061_at	Mlf1	myeloid leukemia factor 1	1.0886	0.5614	0.0142	0.1405	No	1.0173	0.995	0.0131	0.158	No	No
102062_at	Smarcc1	SWI/SNF related, matrix associated, actin dependent regulator of chromatin, subfamily c, member 1	0.9717	0.6029	0.0001	0.0095	No	0.9499	0.595	0.169	0.317	No	No
102063_at	Pdpk1	3-phosphoinositide dependent protein kinase-1	1.0117	0.7733	0.3464	0.5082	No	1.0005	0.984	0.458	0.621	No	No
102064_at	Casp1	caspase 1	0.8822	0.0199	0.02	0.9311	No	0.988	0.907	0.0017	0.578	No	No
102065_at	Fcna	ficolin A	0.8667	0.0009	0.0004	0.1444	Yes	0.9696	0.57	0.962	0.866	No	No
102069_at	Mtf2	metal response element binding transcription factor 2	0.9646	0.5327	0.745	0.8256	No	0.9998	0.984	0.461	0.939	No	No
102070_at	Col9a3	procollagen, type IX, alpha 3	0.9472	0.1096	0	0.8741	No	0.9299	0.059	0	0.449	No	No
102071_at	1500034J01 Rik	RIKEN cDNA 1500034J01 gene	0.9997	0.9907	0.0022	0.4321	No	1.0061	0.624	0.001	0.773	No	No
102072_g_at	1500034J01 Rik	RIKEN cDNA 1500034J01 gene	0.9777	0.2735	0.0005	0.0117	No	0.9582	0.213	0.0015	0.111	No	No
102073_at	Park2	parkin	0.9923	0.8508	0.0292	0.4705	No	0.8958	0.255	0.305	0.407	No	No
102074_at	Otp	orthopedia homolog (Drosophila)	1.0203	0.6377	0.12	0.2861	No	1.0018	0.981	0.775	0.836	No	No
102075_at	Mcpt-ps1	mast cell protease, pseudogene 1	0.9738	0.7751	0.4945	0.7286	No	1.1137	0.216	0.112	0.611	No	No
102076_at	Gm1419 /// Igkv4-77 /// LOC672339 /// LOC672342 /// LOC675870	gene model 1419, (NCBI) /// immunoglobulin kappa chain variable 4-77 /// similar to Ig kappa chain V-IV region S107B precursor /// similar to Ig kappa chain V-IV region S107B precursor /// similar to Ig kappa chain V-IV region S107B precursor	1.0144	0.7105	0.0025	0.0713	No	1.0959	0.0603	0.0093	0.0123	No	No
102077_at	Cyp51	Cytochrome P450, family 51	0.9702	0.5533	0.1218	0.6438	No	0.8798	0.211	0.0253	0.846	No	No
102078_at	Pth	parathyroid hormone	0.9614	0.4647	0.8097	0.4083	No	0.8715	0.368	0.218	0.106	No	No
102079_at	Aym1	activator of yeast meiotic promoters 1	1.0185	0.5624	0.133	0.0617	No	1.0998	0.138	0.188	0.322	No	No
102080_at	---	---	1.0132	0.8365	0.607	0.7762	No	1.0352	0.511	0.94	0.214	No	No
102081_at	Robo3	roundabout homolog 3 (Drosophila)	0.9917	0.895	0.4685	0.277	No	0.9216	0.332	0.198	0.983	No	No
102082_at	Gnas	GNAS (guanine nucleotide binding protein, alpha stimulating) complex locus	0.9658	0.5366	0.2186	0.2926	No	0.9779	0.406	0.201	0.344	No	No
102083_at	Rit2	Ras-like without CAAX 2	1.015	0.7209	0.8032	0.3271	No	1.0573	0.433	0.0637	0.115	No	No
102084_f_at	Cyp2c38	cytochrome P450, family 2, subfamily c, polypeptide 38	1.0197	0.6904	0.1634	0.2574	No	1.0379	0.461	0.0208	0.447	No	No
102085_at	Insm1	insulinoma-associated 1	1.0931	0.0155	0.0274	0.0022	No	1.0269	0.626	0.0231	0.342	No	No
102086_r_at	D15Ertd50e	DNA segment, Chr 15, ERATO Doi 50, expressed	0.9067	0.0142	0.14	0.1823	No	0.8098	0.567	0.606	0.493	No	No
102087_at	Hoxa3	homeo box A3	1.0378	0.0682	0.0001	0.0088	No	1.0418	0.463	0.0043	0.497	No	No
102088_at	Klf12	Kruppel-like factor 12	1.0281	0.4212	0.2454	0.3806	No	1.0167	0.381	0.162	0.713	No	No
102089_at	Matn3	matrilin 3	0.8799	0.0675	0.0004	0.1702	No	0.8188	0.0024	0	0.196	Yes	No
102090_f_at	Smyd5	SET and MYND domain containing 5	1.0137	0.7838	0.0723	0.9386	No	1.1785	0.0163	0.0013	0.015	No	No
102091_f_at	---	---	1.0637	0.3689	0.0036	0.8105	No	1.0371	0.264	0.0016	0.283	No	No
102092_s_at	Ovgp1	oviductal glycoprotein 1	0.927	0.5666	0.0006	0.2093	No	0.9429	0.617	0.0001	0.424	No	No
102093_f_at	Sfrs4	splicing factor, arginine/serine-rich 4 (SRp75)	1.0418	0.3684	0.2649	0.5316	No	0.9746	0.645	0.716	0.616	No	No
102094_f_at	---	---	0.8977	0.0451	0.0405	0.0194	No	0.883	0.13	0.0602	0.059	No	No
102095_f_at	Spock2	sparc/osteonectin, cwcv and kazal-like domains proteoglycan 2	0.9352	0.2351	0.0066	0.0287	No	0.9293	0.346	0.0121	0.957	No	No

102096_f_at	Mup1 /// Mup2 /// Mup3 /// Mup4 /// Mup5 /// MGC107671 /// LOC620807 /// LOC634915 /// LOC635101 /// LOC670615	major urinary protein 1 /// major urinary protein 2 /// major urinary protein 3 /// major urinary protein 4 /// major urinary protein 5 /// similar to alpha-2u-globulin V precursor - mouse /// similar to Major urinary protein 4 precursor (MUP 4) /// similar to Major urinary protein 4 precursor (MUP 4) /// similar to major urinary protein 4 /// similar to alpha-2u globulin PGCL3	0.9782	0.289	0.0008	0.0268	No	1.0945	0.272	0.194	0.769	No	No
102097_f_at	Ndufa3	NADH dehydrogenase (ubiquinone) 1 alpha subcomplex, 3	1.0458	0.4679	0.7947	0.1948	No	1.0767	0.161	0.497	0.164	No	No
102098_at	---	---	1.0402	0.3139	0.0035	0.5371	No	0.9963	0.988	0.0114	0.667	No	No
102099_f_at	Kctd17	potassium channel tetramerisation domain containing 17	0.9532	0.4474	0.0568	0.9148	No	0.9095	0.0676	0.784	0.0468	No	No
102100_f_at	---	---	1.0401	0.6332	0.7189	0.7089	No	1.0534	0.515	0.256	0.708	No	No
102101_f_at	---	---	0.9185	0.3443	0.0048	0.8246	No	0.9737	0.554	0.0008	0.423	No	No
102102_at	---	Transcribed locus, weakly similar to XP_576435.1 PREDICTED: similar to ORF4 [Rattus norvegicus]	0.9675	0.1949	0.0015	0.0301	No	0.9659	0.674	0.01	0.108	No	No
102103_f_at	Txnl2	thioredoxin-like 2	1.0359	0.1061	0.1082	0.0137	No	1.0103	0.757	0.097	0.101	No	No
102104_f_at	Ms4a6c	membrane-spanning 4-domains, subfamily A, member 6C	0.8208	0.0426	0.7591	0.9742	No	0.8131	0.0411	0.523	0.989	No	No
102105_f_at	Ptgds	prostaglandin D2 synthase (brain)	0.9731	0.5632	0.0216	0.222	No	0.9748	0.575	0.121	0.0718	No	No
102106_at	C76472	expressed sequence C76472	1.0682	0.1268	0.0943	0.0172	No	0.994	0.751	0.011	0.174	No	No
102107_at	---	---	1.0309	0.4923	0.9226	0.1004	No	1.1726	0.0548	0.402	0.01	No	No
102108_f_at	Myh9	myosin, heavy polypeptide 9, non-muscle	0.9437	0.0974	0.7068	0.6196	No	0.9206	0.481	0.682	0.287	No	No
102109_at	Rpl13	ribosomal protein L13	1.0219	0.5282	0.0015	0.1762	No	1.0066	0.501	0.0001	0.0629	No	No
102110_at	---	Transcribed locus	0.9113	0.0863	0.7407	0.8285	No	0.9382	0.134	0.466	0.478	No	No
102111_f_at	---	---	1.2644	0.0577	0.0128	0.019	No	1.1287	0.0599	0.0077	0.0145	No	No
102112_s_at	---	---	1.0687	0.0077	0.0035	0.006	No	1.1298	0.0002	0.0001	0.0001	No	No
102113_f_at	Tsta3	tissue specific transplantation antigen P35B	0.9493	0.2183	0.0005	0.1175	No	0.9905	0.902	0.0994	0.381	No	No
102114_f_at	Angptl4	angiopoietin-like 4	1.0418	0.5001	0.035	0.0809	No	1.0757	0.159	0.0114	0.114	No	No
102115_r_at	Nusap1	nucleolar and spindle associated protein 1	1.0421	0.5676	0.4384	0.2064	No	1.1042	0.118	0.303	0.0093	No	No
102116_f_at	Syt3	synaptotagmin III	1.0472	0.3006	0.0393	0.1665	No	1.1029	0.172	0.309	0.0173	No	No
102117_at	Rabl4	RAB, member of RAS oncogene family-like 4	0.9406	0.0342	0.1626	0.0128	No	0.9579	0.337	0.0472	0.18	No	No
102118_at	Ankrd17	ankyrin repeat domain 17	0.9548	0.2228	0.0144	0.06	No	0.9841	0.861	0.0019	0.154	No	No
102119_at	---	---	0.9616	0.6215	0.0573	0.3508	No	0.9711	0.738	0.159	0.955	No	No
102120_f_at	LOC675539	similar to Hippocalcin-like protein 1 (Visinin-like protein 3) (VILIP-3) (Neural visinin-like protein 3) (NVL-3) (NVP-3)	1.0368	0.4523	0.0023	0.8324	No	1.1598	0.0462	0.0202	0.22	No	No
102121_f_at	Krt1-19	keratin complex 1, acidic, gene 19	0.957	0.2328	0.0003	0.0018	No	0.9284	0.434	0.0017	0.0044	No	No
102122_f_at	---	---	0.9556	0.3756	0.0016	0.6148	No	0.8942	0.188	0.0266	0.331	No	No
102123_at	Lip1	Lysosomal acid lipase 1	1.0036	0.8646	0.0003	0.3781	No	0.9849	0.747	0.0083	0.659	No	No
102124_f_at	Cox4i1	cytochrome c oxidase subunit IV isoform 1	1.0549	0.3829	0.2903	0.0823	No	1.0951	0.383	0.989	0.0337	No	No
102125_f_at	---	---	1.0708	0.3246	0.0891	0.7545	No	1.0509	0.283	0.183	0.725	No	No
102126_at	Rps12 /// LOC670751 /// LOC670832 /// LOC671641 /// LOC672008 /// LOC672959	ribosomal protein S12 /// similar to ribosomal protein S12 /// similar to ribosomal protein S12 /// similar to 40S ribosomal protein S12 /// similar to ribosomal protein S12 /// similar to ribosomal protein S12	1.0473	0.0124	0	0.0304	No	1.0308	0.0284	0.0004	0.103	No	No
102127_at	Lgtn	Ligatin	0.989	0.9289	0.4473	0.9036	No	0.999	1	0.995	0.683	No	No
102128_f_at	Mrps25	mitochondrial ribosomal protein S25	1.109	0.2588	0.0544	0.2915	No	1.0745	0.363	0.0268	0.499	No	No
102129_at	Rps17 /// LOC268650 /// LOC383032	ribosomal protein S17 /// similar to ribosomal protein S17 /// similar to 40S ribosomal protein S17	1.076	0.2229	0.037	0.362	No	1.0608	0.691	0.676	0.743	No	No
102130_f_at	---	In vitro fertilized eggs cDNA, RIKEN full-length enriched library, clone:7420405G17 product:unclassifiable, full insert sequence	1.0458	0.1661	0.3169	0.2418	No	1.0833	0.138	0.167	0.325	No	No
102131_f_at	Rnf20	ring finger protein 20	1.0032	0.8921	0.0001	0.5914	No	1.0639	0.0529	0.0023	0.027	No	No
102132_i_at	Akp5	alkaline phosphatase 5	0.9796	0.6881	0.4601	0.7527	No	0.9643	0.858	0.0222	0.225	No	No
102133_at	Zfp568	zinc finger protein 568	0.875	0.1323	0.0404	0.417	No	0.9165	0.205	0.369	0.411	No	No
102134_f_at	Atp5g2 /// LOC626403	ATP synthase, H+ transporting, mitochondrial F0 complex, subunit c (subunit 9), isoform 2 /// similar to ATP synthase, H+ transporting, mitochondrial F0 complex, subunit c (subunit 9), isoform 2	1.0132	0.2949	0	0.2601	No	1.1076	0.133	0.0482	0.969	No	No
102135_at	C78893	expressed sequence C78893	1.0563	0.0631	0.1737	0.1558	No	1.0837	0.0489	0.174	0.0144	No	No
102136_r_at	C79246	expressed sequence C79246	1.0462	0.3973	0.0044	0.0585	No	1.2	0.0079	0.112	0.0171	Yes	No
102137_f_at	---	---	1.0204	0.2631	0.0006	0.0209	No	1.0033	0.815	0.159	0.979	No	No
102138_at	D13Ertd37e	DNA segment, Chr 13, ERATO Doi 37, expressed	0.9142	0.2092	0.0082	0.7228	No	0.8784	0.344	0.344	0.747	No	No
102139_at	D13Ertd205e	DNA segment, Chr 13, ERATO Doi 205, expressed	1.0343	0.6092	0.0191	0.2384	No	1.0415	0.619	0.383	0.49	No	No
102140_at	C77137	expressed sequence C77137	1.0327	0.5074	0.1501	0.1744	No	1.0761	0.0433	0.0025	0.0185	No	No
102141_f_at	4933434E20Rik	RIKEN cDNA 4933434E20 gene	1.0225	0.4575	0.0006	0.0114	No	1.0742	0.0321	0.0001	0.0803	No	No
102142_r_at	LOC236874 /// LOC628991 /// LOC635720	similar to odorant binding protein Ia /// similar to odorant binding protein Ia /// similar to odorant binding protein Ia	1.1387	0.0516	0.7641	0.0723	No	1.1126	0.118	0.019	0.0625	No	No
102143_at	Eno3	Enolase 3, beta muscle	0.9398	0.4296	0.7439	0.6467	No	1.0066	0.926	0.557	0.935	No	No
102144_f_at	Sfpq	splicing factor proline/glutamine rich (polypyrimidine tract binding protein associated)	1.1036	0.2801	0.0048	0.1701	No	1.0517	0.874	0.0074	0.277	No	No
102145_f_at	Esrra	estrogen related receptor, alpha	0.9524	0.2464	0.8963	0.0395	No	0.9891	0.812	0.901	0.0971	No	No
102146_at	Insr	insulin receptor	0.9497	0.5024	0.858	0.551	No	1.0069	0.872	0.723	0.567	No	No
102147_at	Akp3	alkaline phosphatase 3, intestine, not Mn requiring	0.9746	0.4937	0.0024	0.0834	No	0.899	0.0516	0.0072	0.0233	No	No
102148_f_at	Ifna1 /// Ifna5 /// Ifna9	interferon alpha family, gene 1 /// interferon alpha family, gene 5 /// interferon alpha family, gene 9	1.0254	0.6668	0.9968	0.9388	No	0.9842	0.767	0.0224	0.655	No	No

102149_f_at	Ifna1 /// Ifna2 /// Ifna5 /// Ifna7 /// Ifna9 /// Ifnab /// Ifna6T /// LOC242517 /// Ifna12 /// LOC384100 /// Ifna14	interferon alpha family, gene 1 /// interferon alpha family, gene 2 /// interferon alpha family, gene 5 /// interferon alpha family, gene 7 /// interferon alpha family, gene 9 /// interferon alpha family, gene B /// interferon alpha 6T /// alpha-interferon /// interferon alpha family, gene 12 /// interferon alpha 8/6 precursor; IFNa8/6 /// interferon alpha 14	0.9845	0.8242	0.9704	0.2912	No	1.065	0.31	0.19	0.281	No	No
102150_f_at	LOC242517	alpha-interferon	1.0653	0.3854	0.915	0.6641	No	1.0332	0.274	0.0043	0.969	No	No
102151_at	Adrb1	adrenergic receptor, beta 1	1.011	0.8503	0.202	0.7663	No	0.9137	0.0134	0.0024	0.0044	No	No
102152_f_at	LOC632502	similar to Ig heavy chain V region M167 precursor	1.0828	0.0797	0.5142	0.0526	No	1.0808	0.202	0.105	0.193	No	No
102153_at	---	---	0.9971	0.9506	0.0942	0.8935	No	1.0031	0.859	0.0068	0.571	No	No
102154_f_at	Igk-V8	immunoglobulin kappa chain variable 8 (V8)	1.0299	0.6768	0.025	0.8146	No	1.2205	0.0643	0.0032	0.0254	No	No
102155_f_at	LOC672291	similar to Ig kappa chain V-V region MOPC 173	1.022	0.7877	0.4278	0.6987	No	0.9051	0.516	0.326	0.584	No	No
102156_f_at	Igk-V28 /// Igk-V8 /// LOC545854 /// Igk-V21	immunoglobulin kappa chain variable 28 (V28) /// immunoglobulin kappa chain variable 8 (V8) /// immunoglobulin kappa chain /// immunoglobulin kappa chain variable 21 (V21)	1.019	0.6163	0.3998	0.5339	No	1.1448	0.0557	0.835	0.0705	No	No
102157_f_at	Igkv10-95	immunoglobulin kappa chain variable 10-95	0.9757	0.6488	0.1077	0.448	No	1.1531	0.101	0.0379	0.288	No	No
102158_at	---	---	1.024	0.5328	0.056	0.0857	No	1.0516	0.0871	0.0045	0.0372	No	No
102159_at	Igh-4 /// Igh-6 /// Igh-V7183 /// Igh-VJ558 /// LOC238447 /// Igh-1a /// Ighg /// LOC380804 /// LOC544896 /// LOC544903 /// LOC630253 /// LOC630296 /// LOC630298 /// LOC630302 /// LOC641178 /// LOC668395 /// LOC674089 ///	immunoglobulin heavy chain 4 (serum IgG1) /// immunoglobulin heavy chain 6 (heavy chain of IgM) /// immunoglobulin heavy chain (V7183 family) /// immunoglobulin heavy chain (J558 family) /// similar to immunoglobulin heavy chain variable region /// immunoglobulin heavy chain 1a (serum IgG2a) /// Immunoglobulin heavy chain (gamma polypeptide) /// Ig heavy chain V region /// similar to Ig heavy chain V region 5-84 precursor /// similar to immunoglobulin mu-chain /// similar to Ig heavy chain V region 5-84 precursor /// similar to Ig heavy chain V region RF precursor /// similar to Ig heavy chain V region 345 precursor /// similar to Ig heavy chain V region 5-84 precursor /// similar to Ig heavy chain V region 345 precursor /// similar to Ig heavy chain V region 5-84 precursor /// similar to Ig heavy chain V region 345 precursor /// similar to Ig heavy chain V region 5-76 precursor /// similar to Ig heavy chain V region 7-39 precursor /// similar to Ig heavy chain V region 5-84 precursor /// similar to Ig heavy chain V region 5-76 precursor /// similar to Ig heavy chain V region 5-76 pre	1.0504	0.222	0.1376	0.2654	No	1.084	0.422	0.0255	0.165	No	No
102160_at	---	---	1.0721	0.0918	0.4913	0.1853	No	1.053	0.368	0.0225	0.947	No	No
102161_f_at	H2-D1 /// H2-L /// H2-T22 /// LOC56628 /// LOC547343 /// LOC547349 /// LOC630499 /// LOC633617 /// LOC676708	histocompatibility 2, D region locus 1 /// histocompatibility 2, D region /// histocompatibility 2, T region locus 22 /// MHC (A.CA/J(H-2K-f) class I antigen /// similar to H-2 class I histocompatibility antigen, L-D alpha chain precursor /// similar to MHC class I antigen precursor /// similar to H-2 class I histocompatibility antigen, D-K alpha chain precursor (H-2D(K)) /// similar to H-2 class I histocompatibility antigen, D-K alpha chain precursor (H-2D(K)) /// similar to H-2 class I histocompatibility antigen, L-D alpha chain precursor	0.9292	0.1008	0.0069	0.0666	No	0.9348	0.0252	0.0004	0.876	No	No
102162_at	---	---	0.961	0.5199	0.3796	0.6805	No	0.9887	0.753	0.0087	0.587	No	No
102163_at	Snrpn /// LOC545062	small nuclear ribonucleoprotein N /// similar to small nuclear ribonucleoparticle-associated protein	0.9783	0.6095	0.0535	0.0821	No	1.005	0.565	0.0001	0.0867	No	No
102164_at	Gja10	gap junction membrane channel protein alpha 10	0.9703	0.5879	0.0396	0.617	No	0.7281	0.497	0.187	0.741	No	No
102165_at	V1rb5	vomeronasal 1 receptor, B5	1.1288	0.0288	0.1922	0.086	No	1.05	0.237	0.153	0.84	No	No
102166_g_at	V1rb6 /// V1rb5	vomeronasal 1 receptor, B6 /// vomeronasal 1 receptor, B5	1.1894	0.0579	0.1911	0.0252	No	1.1773	0.0472	0.102	0.0378	No	No
102167_at	V1rb6	vomeronasal 1 receptor, B6	1.0132	0.5963	0.0212	0.1901	No	1.0334	0.334	0.396	0.334	No	No
102168_at	Olfr92	olfactory receptor 92	1.0668	0.4253	0.1523	0.4206	No	1.009	0.842	0.178	0.884	No	No
102169_at	Olfr93	olfactory receptor 93	1.1119	0.2356	0.9398	0.1252	No	1.1056	0.0635	0.0274	0.248	No	No
102170_at	Olfr94	olfactory receptor 94	1.0293	0.3277	0.0338	0.5485	No	1.0786	0.227	0.662	0.0292	No	No
102171_r_at	Nr1i3	nuclear receptor subfamily 1, group I, member 3	0.9573	0.4859	0.7806	0.9824	No	0.9906	0.926	0.712	0.444	No	No
102192_r_at	Acsm3	acyl-CoA synthetase medium-chain family member 3	0.8986	0.0109	0.0051	0.1135	No	0.5552	0.0762	0.234	0.0421	No	No
102193_at	Exod1	exonuclease domain containing 1	0.9751	0.4711	0.6738	0.1418	No	0.9773	0.655	0.0271	0.496	No	No
102194_at	2810432D09Rik	RIKEN cDNA 2810432D09 gene	0.9808	0.356	0.04	0.0035	No	0.9046	0.0148	0.002	0.0247	No	No
102195_at	Map4k4	mitogen-activated protein kinase kinase kinase kinase 4	0.9553	0.4352	0.0203	0.9406	No	0.9703	0.369	0.0936	0.686	No	No
102196_at	Gna11	guanine nucleotide binding protein, alpha 11	1.033	0.4411	0.1741	0.0903	No	1.0474	0.324	0.554	0.127	No	No
102197_at	Nucb2	nucleobindin 2	1.0153	0.8157	0.5739	0.8687	No	0.9987	0.884	0.0148	0.375	No	No
102198_at	Kcnn4	potassium intermediate/small conductance calcium-activated channel, subfamily N, member 4	0.9543	0.3602	0.0996	0.1689	No	0.8949	0.223	0.292	0.655	No	No
102199_at	Tarsl1	threonyl-tRNA synthetase-like 1	1.0189	0.611	0.1235	0.2725	No	1.0051	0.898	0.593	0.779	No	No
102200_at	Aqp8	aquaporin 8	1.0006	0.9892	0.2194	0.1719	No	1.0066	0.851	0.204	0.175	No	No
102201_s_at	Psen1	presenilin 1	0.9084	0.0158	0.0125	0.1349	No	0.8842	0.0012	0.659	0.0213	Yes	Yes
102202_s_at	Mpv17	Mpv17 transgene, kidney disease mutant	1.0042	0.951	0.0031	0.5842	No	1.0541	0.256	0.0133	0.944	No	No
102203_at	Pp11r	placental protein 11 related	0.9562	0.5779	0.62	0.6784	No	1.0183	0.867	0.839	0.029	No	No
102204_at	Mafb	v-maf musculoaponeurotic fibrosarcoma oncogene family, protein B (avian)	1.0942	0.0325	0.0269	0.113	No	1.0835	0.0271	0.0608	0.162	No	No
102205_at	Gnptab	N-acetylglucosamine-1-phosphate transferase, alpha and beta subunits	0.9967	0.9438	0.6105	0.4952	No	0.9758	0.48	0.583	0.256	No	No
102206_at	Fkbpl	FK506 binding protein-like	1.0301	0.2696	0.0041	0.1698	No	1.0397	0.327	0.002	0.552	No	No
102207_at	Nrbp2	nuclear receptor binding protein 2	0.9524	0.1193	0.0004	0.2805	No	0.9907	0.652	0.0022	0.174	No	No
102208_at	St3gal6	ST3 beta-galactoside alpha-2,3-sialyltransferase 6	0.814	0.0122	0.0038	0.8474	No	0.8404	0.0118	0.001	0.851	No	No
102209_at	Nfatc1	nuclear factor of activated T-cells, cytoplasmic, calcineurin-dependent 1	0.9939	0.9364	0.7646	0.4871	No	0.9905	0.851	0.453	0.695	No	No
102210_at	Dtnb	dystrobrevin, beta	0.9391	0.0528	0.0029	0.4483	No	1.0388	0.461	0.0942	0.708	No	No
102211_r_at	Sec24b	SEC24 related gene family, member B (S. cerevisiae)	1.03	0.4848	0.001	0.0518	No	1.0687	0.198	0.0011	0.114	No	No
102212_at	Rhox6	reproductive homeobox 6	0.9682	0.6305	0.4673	0.8072	No	0.9023	0.164	0.0367	0.42	No	No
102213_at	Cmya1	cardiomyopathy associated 1	0.8761	0.0024	0.0583	0.0789	Yes	0.9511	0.268	0.111	0.586	No	No
102214_at	Prx	periaxin	1.0549	0.3057	0.4838	0.0857	No	0.9466	0.103	0.363	0.475	No	No
102215_at	Ift81	intraflagellar transport 81 homolog (Chlamydomonas)	1.0115	0.8497	0.9535	0.1891	No	0.9658	0.109	0.832	0.0311	No	No
102216_at	Alox12b	arachidonate 12-lipoxygenase, 12R type	1.0165	0.6307	0.0026	0.2426	No	1.0735	0.187	0.0263	0.429	No	No
102217_at	Gprk5	G protein-coupled receptor kinase 5	0.969	0.2886	0.86	0.8062	No	0.9743	0.561	0.376	0.484	No	No

102218_at	Il6	interleukin 6	0.9901	0.8431	0.9774	0.8684	No	1.0849	0.367	0.799	0.969	No	No
102219_at	Rfx2	regulatory factor X, 2 (influences HLA class II expression)	1.028	0.3891	0.0525	0.3578	No	0.9982	0.938	0.953	0.427	No	No
102220_at	Utf1	undifferentiated embryonic cell transcription factor 1	0.9984	0.9451	0.0005	0.9316	No	0.9688	0.17	0.0018	0.0388	No	No
102221_at	Syngr1	synaptogyrin 1	1.0648	0.138	0.4402	0.5089	No	1.0616	0.295	0.602	0.786	No	No
102222_at	Utx	ubiquitously transcribed tetratricopeptide repeat gene, X chromosome	1.0402	0.2996	0.0001	0.0922	No	1.0149	0.487	0.0001	0.318	No	No
102223_at	Ppl	periplakin	1.073	0.0744	0.7848	0.8767	No	1.0967	0.168	0.147	0.369	No	No
102224_at	D930020L01	hypothetical protein D930020L01	0.8424	0.0106	0.0002	0.0313	No	0.9451	0.154	0.0015	0.589	No	No
102225_at	Rabgap1l	RAB GTPase activating protein 1-like	0.99	0.8986	0.1359	0.2262	No	0.9867	0.851	0.475	0.212	No	No
102226_at	Cpxm2	carboxypeptidase X 2 (M14 family)	0.9819	0.2639	0	0.0075	No	1.0103	0.628	0.0004	0.344	No	No
102227_g_at	Cpxm2	carboxypeptidase X 2 (M14 family)	0.98	0.4296	0.0526	0.2103	No	1.0767	0.147	0.285	0.282	No	No
102228_at	Lat	linker for activation of T cells	0.985	0.7271	0.9928	0.1845	No	1.025	0.349	0.0582	0.0175	No	No
102229_at	Nmt2	N-myristoyltransferase 2	1.0194	0.5502	0.0094	0.1579	No	1.0998	0.102	0.26	0.224	No	No
102230_at	Hyal1 /// Nat6	hyaluronoglucosaminidase 1 /// N-acetyltransferase 6	0.9408	0.2109	0.0371	0.3582	No	0.9248	0.0403	0.0196	0.007	No	No
102231_at	Creb3l1	cAMP responsive element binding protein 3-like 1	0.9859	0.2071	0	0.0158	No	1.0215	0.522	0.16	0.466	No	No
102232_at	LOC674308	similar to solute carrier family 39 (zinc transporter), member 9	0.9333	0.1409	0.0713	0.7896	No	0.9218	0.0817	0.0063	0.206	No	No
102233_at	4833442J19 Rik	RIKEN cDNA 4833442J19 gene	0.8359	0.0309	0.0257	0.6367	No	0.9311	0.158	0.0007	0.525	No	No
102234_at	---	---	1.0918	0.0873	0.4053	0.6308	No	1.1089	0.0309	0.119	0.753	No	No
102235_at	Mycl1	v-myc myelocytomatosis viral oncogene homolog 1, lung carcinoma derived (avian)	1.0127	0.588	0.0018	0.368	No	1.0554	0.0245	0.0002	0.421	No	No
102237_at	Cd28	CD28 antigen	1.0438	0.36	0.3192	0.3166	No	1.0628	0.331	0.0379	0.185	No	No
102238_at	Ascl1	achaete-scute complex homolog-like 1 (Drosophila)	1.0059	0.9164	0.3592	0.5491	No	1.0234	0.739	0.0097	0.282	No	No
102239_at	Bcl3	B-cell leukemia/lymphoma 3	0.9745	0.2424	0.0013	0.18	No	0.998	0.903	0.0012	0.699	No	No
102240_at	Ppargc1a	peroxisome proliferative activated receptor, gamma, coactivator 1 alpha	0.9048	0.0059	0.5735	0.0914	Yes	0.8308	0.104	0.0941	0.102	No	No
102241_f_at	Tcf7l2	transcription factor 7-like 2, T-cell specific, HMG-box	1.049	0.4664	0.2477	0.2702	No	1.0093	0.686	0.0025	0.145	No	No
102242_at	Per3	period homolog 3 (Drosophila)	1.0991	0.0057	0.0901	0.0098	No	1.0708	0.0112	0.304	0.351	No	No
102243_at	Ehf	ets homologous factor	0.9817	0.7766	0.0323	0.6793	No	0.9596	0.341	0.0118	0.267	No	No
102244_at	Tesp1	testicular serine protease 1	1.0029	0.9717	0.2688	0.9036	No	0.9763	0.868	0.655	0.977	No	No
102247_at	Ubr1	ubiquitin protein ligase E3 component n-recognin 1	0.9993	0.9896	0.7822	0.2785	No	0.9551	0.53	0.306	0.705	No	No
102248_f_at	Cask	calcium/calmodulin-dependent serine protein kinase (MAGUK family)	1.01	0.9147	0.0113	0.5355	No	1.0514	0.457	0.0116	0.403	No	No
102249_at	Avil	advillin	0.9747	0.3254	0.0006	0.0091	No	1.0276	0.464	0.0281	0.112	No	No
102250_at	Il27ra	interleukin 27 receptor, alpha	1.0339	0.1048	0.0001	0.329	No	1.0225	0.475	0.0006	0.329	No	No
102251_at	Trpm1	transient receptor potential cation channel, subfamily M, member 1	0.9333	0.147	0.0924	0.2582	No	1.022	0.584	0.0037	0.207	No	No
102252_at	Pfdn2	prefoldin 2	1.0278	0.5474	0.4527	0.8563	No	1.0248	0.586	0.004	0.484	No	No
102253_at	AF067063	cDNA sequence AF067063	0.9032	0.1071	0.4568	0.3374	No	0.9976	0.938	0.143	0.996	No	No
102254_f_at	LOC625360	similar to 2-cell-stage, variable group, member 3	1.0568	0.3757	0.9578	0.8731	No	0.9892	0.917	0.0058	0.122	No	No
102255_at	Osmr	oncostatin M receptor	0.9191	0.1762	0.0271	0.1742	No	0.921	0.262	0.0215	0.306	No	No
102256_at	Tbx15	T-box 15	0.9026	0.1441	0.5846	0.2824	No	0.8711	0.0167	0.0005	0.214	No	No
102257_at	Pknox1	Pbx/knotted 1 homeobox	0.9597	0.4839	0.1032	0.754	No	0.9931	0.434	0.0405	0.866	No	No
102258_at	Stra6	stimulated by retinoic acid gene 6	1.0216	0.5741	0.8704	0.0152	No	0.9667	0.667	0.151	0.529	No	No
102259_at	Ywhag	3-monooxygenase/tryptophan 5-monooxygenase activation protein, gamma polypeptide	1.0542	0.3191	0.2553	0.3924	No	1.0461	0.311	0.671	0.962	No	No
102260_at	Gfi1b	growth factor independent 1B	1.0676	0.2843	0.2746	0.752	No	1.1907	0.0159	0.585	0.273	No	No
102261_f_at	Col13a1	procollagen, type XIII, alpha 1	0.8977	0.1231	0.3043	0.081	No	0.9881	0.235	0.0001	0.0056	No	No
102262_r_at	Col13a1	procollagen, type XIII, alpha 1	1.0416	0.393	0.3055	0.8021	No	0.9983	0.993	0.964	0.206	No	No
102263_at	Zfp143	zinc finger protein 143	0.9959	0.8973	0.5826	0.1538	No	1.0163	0.716	0.736	0.599	No	No
102264_at	Slfn1	schlafen 1	1.0423	0.13	0.0162	0.0288	No	0.9609	0.585	0.0321	0.112	No	No
102265_at	Myf6	myogenic factor 6	0.8828	0.0046	0.5647	0.0086	No	0.9755	0.821	0.0045	0.436	No	No
102266_at	Inha	inhibin alpha	1.0163	0.6267	0.3861	0.0994	No	0.9824	0.673	0.0909	0.984	No	No
102267_at	B3galt4	UDP-Gal:betaGalNAc beta 1,3-galactosyltransferase, polypeptide 4	1.0172	0.7834	0.2154	0.1367	No	1.0309	0.385	0.805	0.111	No	No
102268_at	Rexo1	REX1, RNA exonuclease 1 homolog (S. cerevisiae)	0.9989	0.9533	0.0012	0.03	No	1.062	0.161	0.131	0.101	No	No
102269_at	Alox12e	arachidonate lipoxygenase, epidermal	1.05	0.1675	0.0046	0.0174	No	1.0221	0.733	0.603	0.237	No	No
102271_at	D11Bwg0280e	DNA segment, Chr 11, Brigham & Women's Genetics 0280e expressed	1.0274	0.3064	0.944	0.06	No	1.0493	0.296	0.009	0.202	No	No
102272_at	Cd160	CD160 antigen	1.0826	0.1743	0.0158	0.4713	No	0.9772	0.894	0.0339	0.111	No	No
102273_at	Atp6v0a2	ATPase, H+ transporting, lysosomal V0 subunit A2	1.0866	0.1432	0.1295	0.1595	No	1.0325	0.657	0.673	0.918	No	No
102274_at	H2-Oa	histocompatibility 2, O region alpha locus	1.004	0.8804	0.0546	0.5047	No	1.0584	0.277	0.258	0.233	No	No
102275_at	Zfp185	zinc finger protein 185	0.9926	0.9009	0.7323	0.9416	No	0.8793	0.0507	0.0876	0.083	No	No
102277_at	Zfp26	zinc finger protein 26	1.0255	0.7083	0.0091	0.1148	No	0.9957	0.938	0.0342	0.336	No	No
102278_at	Ppie	peptidylprolyl isomerase E (cyclophilin E)	1.0529	0.527	0.3712	0.8445	No	1.0352	0.364	0.0281	0.183	No	No
102279_at	Ube1l	ubiquitin-activating enzyme E1-like	0.9972	0.8471	0.0005	0.7448	No	1.0245	0.377	0.0047	0.426	No	No
102280_at	Pcdh7	protocadherin 7	1.066	0.4358	0.0115	0.525	No	1.0678	0.241	0.0016	0.826	No	No
102281_at	Tnfrsf7	tumor necrosis factor receptor superfamily, member 7	1.0158	0.7905	0.0059	0.6805	No	1.0322	0.373	0.0124	0.301	No	No
102282_g_at	Tnfrsf7	tumor necrosis factor receptor superfamily, member 7	1.0825	0.0698	0.1475	0.6137	No	1.0205	0.527	0.36	0.647	No	No
102283_at	Tiam1	T-cell lymphoma invasion and metastasis 1	1.0336	0.1897	0.0003	0.1894	No	0.9774	0.542	0.0143	0.751	No	No
102284_at	Masp1	mannan-binding lectin serine peptidase 1	0.9937	0.9002	0.0227	0.1306	No	0.9741	0.575	0.0912	0.0768	No	No
102285_at	Mgmt	O-6-methylguanine-DNA methyltransferase	1.0424	0.3461	0.0331	0.2008	No	1.0742	0.19	0.924	0.117	No	No
102286_at	Araf	v-raf murine sarcoma 3611 viral oncogene homolog	0.9659	0.2913	0.0111	0.0326	No	0.9199	0.228	0.68	0.194	No	No
102287_at	Cd247	CD247 antigen	0.9465	0.5149	0.0998	0.1098	No	1.0017	0.849	0.0742	0.295	No	No
102288_at	Cd247	CD247 antigen	1.0084	0.8194	0.1316	0.1116	No	0.9165	0.248	0.0179	0.318	No	No
102289_r_at	Cr2	complement receptor 2	1.0049	0.9076	0.1172	0.1975	No	0.7958	0.556	0.0044	0.0175	No	No
102290_at	Alox12	arachidonate 12-lipoxygenase	0.9973	0.9607	0.0235	0.6646	No	1.0196	0.708	0.712	0.102	No	No
102291_at	F9	coagulation factor IX	1.0419	0.4932	0.9217	0.1977	No	1.096	0.0671	0.206	0.0382	No	No
102292_at	Gadd45a	growth arrest and DNA-damage-inducible 45 alpha	0.8756	0.0032	0.015	0.0602	Yes	0.9186	0.0656	0.206	0.3	No	No
102293_at	Zfpn1a1	zinc finger protein, subfamily 1A, 1 (Ikaros)	1.0671	0.0728	0.0473	0.1334	No	0.9416	0.081	0.0515	0.944	No	No
102294_at	Zfpn1a1	zinc finger protein, subfamily 1A, 1 (Ikaros)	1.0125	0.8543	0.5481	0.5388	No	1.169	0.0955	0.227	0.0459	No	No
102295_at	Kcna5	potassium voltage-gated channel, shaker-related subfamily, member 5	0.7874	0.0036	0.1105	0.7894	Yes	0.8021	0.0065	0.0075	0.371	Yes	Yes
102296_at	Pcsk2	proprotein convertase subtilisin/kexin type 2	1.0147	0.8192	0.0303	0.1953	No	1.026	0.616	0.009	0.964	No	No
102297_at	Zfp535	zinc finger protein 535	1.0046	0.8591	0.0035	0.7998	No	0.9545	0.104	0.0051	0.0916	No	No
102298_at	Ngfb	nerve growth factor, beta	0.9674	0.6949	0.174	0.2607	No	0.9861	0.921	0.555	0.781	No	No
102299_at	Prkca	protein kinase C, alpha	1.0274	0.4399	0.7055	0.5396	No	1.0675	0.204	0.278	0.175	No	No
102300_at	Rapsn	receptor-associated protein of the synapse	1.0278	0.0983	0.1986	0.0343	No	1.077	0.0656	0.216	0.685	No	No
102301_at	Capza3	capping protein (actin filament) muscle Z-line, alpha 3	1.0336	0.3933	0.011	0.3282	No	1.0603	0.181	0.0311	0.474	No	No
102302_at	Bckdhb	branched chain ketoacid dehydrogenase E1, beta polypeptide	1.1308	0.2522	0.0494	0.1357	No	1.1094	0.113	0.121	0.342	No	No
102303_i_at	Zfp61	zinc finger protein 61	1.0674	0.0649	0.0038	0.1308	No	1.0088	0.786	0.0448	0.296	No	No
102304_f_at	Zfp61	zinc finger protein 61	0.8944	0.1836	0.0731	0.2177	No	0.8925	0.0719	0.0273	0.0658	No	No
102305_at	Gpr37l1	G protein-coupled receptor 37-like 1	1.0484	0.3758	0.2234	0.5168	No	1.0056	0.921	0.28	0.676	No	No
102306_at	Hs2st1	heparan sulfate 2-O-sulfotransferase 1	0.941	0.2326	0.0038	0.3874	No	0.9215	0.276	0.0046	0.173	No	No
102307_at	Dcx	doublecortin	1.1204	0.0227	0.0006	0.017	No	1.0514	0.189	0.0007	0.0806	No	No
102308_at	Tulp3	tubby-like protein 3	1.0272	0.3789	0.0415	0.2895	No	1.0752	0.0586	0.662	0.0118	No	No
102309_at	Zfp326	zinc finger protein 326	1.1805	0.0813	0.0116	0.4059	No	1.0998	0.0238	0.0007	0.014	No	No
102310_at	Ccl22	chemokine (C-C motif) ligand 22	1.045	0.3932	0.1604	0.6582	No	1.0712	0.188	0.0319	0.165	No	No
102311_at	Gp9	glycoprotein 9 (platelet)	1.0508	0.4756	0.1456	0.3201	No	1.0775	0.19	0.412	0.355	No	No
102312_r_at	1500034J01 Rik	RIKEN cDNA 1500034J01 gene	0.9636	0.4375	0.0032	0.5852	No	0.9359	0.313	0.0134	0.211	No	No
102313_at	Gch1	GTP cyclohydrolase 1	0.9332	0.4394	0.0926	0.9993	No	0.9687	0.371	0.0104	0.349	No	No
102314_at	Slc2a4	solute carrier family 2 (facilitated glucose transporter), member 4	0.8248	0.0512	0.1832	0.3336	No	1.052	0.322	0.456	0.891	No	No
102315_at	Cirh1a	cirrhosis, autosomal recessive 1A (human)	1.1586	0.0662	0.4022	0.7446	No	1.0812	0.112	0.0898	0.671	No	No

102316_at	Capn5	calpain 5	0.8941	0.0646	0.0442	0.2886	No	0.9098	0.0913	0.0073	0.598	No	No
102317_at	Vamp4	vesicle-associated membrane protein 4	1.0383	0.3785	0.0859	0.2224	No	1.0617	0.0335	0.0041	0.0155	No	No
102318_at	St8sia4	ST8 alpha-N-acetyl-neuraminide alpha-2,8-sialyltransferase 4	1.0363	0.7325	0.0102	0.7329	No	1.0144	0.761	0.0046	0.92	No	No
102319_at	Snx12	sorting nexin 12	1.0739	0.2072	0.0626	0.703	No	1.0604	0.336	0.0047	0.213	No	No
102320_at	Snx12	sorting nexin 12	1.0354	0.3717	0.5835	0.6877	No	1.1204	0.0314	0.0699	0.938	No	No
102321_at	Adcy6	adenylate cyclase 6	0.9511	0.3467	0.0074	0.7548	No	0.9791	0.42	0.0003	0.661	No	No
102322_at	Ugdh	UDP-glucose dehydrogenase	1.0326	0.5834	0.3315	0.0579	No	0.9178	0.126	0.218	0.115	No	No
102323_at	Hap1	huntingtin-associated protein 1	0.9505	0.1129	0.0128	0.6202	No	0.9877	0.765	0.0377	0.366	No	No
102324_at	Dnajc4	DnaJ (Hsp40) homolog, subfamily C, member 4	1.0375	0.2199	0.0295	0.5505	No	1.0452	0.303	0.0081	0.0496	No	No
102326_at	Ncf2	neutrophil cytosolic factor 2	0.9802	0.5514	0.0918	0.049	No	0.9204	0.25	0.197	0.0201	No	No
102327_at	Aoc3	amine oxidase, copper containing 3	0.9905	0.8186	0.0411	0.3184	No	1.0145	0.782	0.927	0.899	No	No
102328_at	Casp8	caspase 8	1.0383	0.482	0.0307	0.2127	No	1.1625	0.0428	0.034	0.89	No	No
102329_at	Cideb	cell death-inducing DNA fragmentation factor, alpha subunit-like effector B	0.9705	0.4381	0.0024	0.6205	No	1.0477	0.246	0.0626	0.17	No	No
102330_at	Aif1	allograft inflammatory factor 1	0.989	0.9001	0.6455	0.4844	No	0.9492	0.346	0.154	0.0712	No	No
102331_at	St5	suppression of tumorigenicity 5	0.9784	0.5117	0.0012	0.1579	No	1.0259	0.202	0.0007	0.0072	No	No
102332_at	Ulk1	Unc-51 like kinase 1 (C. elegans)	0.9888	0.6018	0.7196	0.9863	No	0.8956	0.0912	0.584	0.32	No	No
102333_at	Epn2	epsin 2	0.9987	0.9449	0.0131	0.1431	No	0.9784	0.543	0.0698	0.142	No	No
102334_at	Dok2	docking protein 2	1.0046	0.9515	0.0594	0.4962	No	1.051	0.0936	0.0002	0.0652	No	No
102335_at	Kcnk1	potassium channel, subfamily K, member 1	0.9016	0.1258	0.3488	0.3652	No	0.8687	0.0156	0.547	0.211	No	No
102336_at	Tmem131	transmembrane protein 131	0.9565	0.4235	0.0003	0.282	No	0.9422	0.364	0.001	0.222	No	No
102337_s_at	Fcgr2b	Fc receptor, IgG, low affinity IIb	0.9278	0.3475	0.1322	0.8296	No	0.9154	0.159	0.0134	0.902	No	No
102338_at	Pde9a	phosphodiesterase 9A	0.9449	0.4319	0.1399	0.3026	No	0.9303	0.25	0.0042	0.863	No	No
102340_at	Mina	myc induced nuclear antigen	1.0048	0.9405	0.2401	0.9707	No	0.9535	0.0575	0.0335	0.0549	No	No
102341_at	Gla	galactosidase, alpha	1.0018	0.9809	0.4619	0.5967	No	1.0427	0.455	0.0676	0.389	No	No
102342_at	Nsf	N-ethylmaleimide sensitive fusion protein	0.9556	0.4467	0.917	0.6219	No	1.0602	0.151	0.0035	0.0608	No	No
102343_at	Cd320	CD320 antigen	0.9512	0.1139	0.2975	0.0106	No	1.0158	0.465	0.0086	0.938	No	No
102344_s_at	Tcea3	transcription elongation factor A (SII), 3	0.9305	0.1692	0.0044	0.5851	No	0.8831	0.0437	0.0017	0.321	No	No
102345_at	Lefty1	left right determination factor 1	1.0557	0.0175	0.0014	0.1554	No	1.0825	0.146	0.0418	0.123	No	No
102346_at	Ercc3	excision repair cross-complementing rodent repair deficiency, complementation group 3	1.0579	0.3067	0.0953	0.9764	No	1.0741	0.128	0.0783	0.445	No	No
102348_at	LOC432823	similar to hypothetical protein MGC37588	0.8244	0.2006	0.0011	0.5391	No	0.8513	0.27	0.0009	0.876	No	No
102349_at	Adprhl2	ADP-ribosylhydrolase like 2	1.008	0.8466	0.1321	0.503	No	1.021	0.62	0.0596	0.798	No	No
102350_at	Adprhl2	ADP-ribosylhydrolase like 2	1.0049	0.9075	0.0299	0.1876	No	1.0183	0.56	0.0508	0.401	No	No
102351_at	Cpa3	carboxypeptidase A3, mast cell	0.9317	0.3809	0.4604	0.2868	No	0.9075	0.0639	0.771	0.0862	No	No
102352_at	Galnt10	UDP-N-acetyl-alpha-D-galactosamine:polypeptide N-acetylgalactosaminyltransferase 10	0.9325	0.0279	0.0046	0.2034	No	0.9064	0.0324	0.0086	0.285	No	No
102353_at	Itgb2	integrin beta 2	0.9615	0.3055	0.0675	0.7333	No	1.0142	0.829	0.004	0.141	No	No
102354_at	Tcf19	transcription factor 19	1.0861	0.0219	0.0223	0.2973	No	1.0056	0.917	0.0047	0.417	No	No
102356_at	Wdr23	WD repeat domain 23	0.927	0.095	0.1719	0.2468	No	0.954	0.299	0.0439	0.181	No	No
102357_at	AU020772	expressed sequence AU020772	0.9563	0.2116	0.001	0.8229	No	1.0183	0.564	0.0001	0.0687	No	No
102360_at	Mthfr	5,10-methylenetetrahydrofolate reductase	0.9864	0.5492	0.0002	0.0645	No	1.0291	0.676	0.255	0.891	No	No
102362_i_at	Junb	Jun-B oncogene	0.9911	0.8194	0.2118	0.4909	No	1.0251	0.558	0.442	0.895	No	No
102363_r_at	Junb	Jun-B oncogene	1.0691	0.1743	0.0365	0.6839	No	1.0572	0.291	0.404	0.0889	No	No
102364_at	Jund1	Jun proto-oncogene related gene d1	0.9214	0.1092	0.0018	0.99	No	0.889	0.0262	0.0009	0.187	No	No
102366_at	Retn	resistin	1.0014	0.9828	0.5939	0.5176	No	0.9992	0.965	0.911	0.435	No	No
102368_at	2610208E05 Rik	RIKEN cDNA 2610208E05 gene	1.0242	0.5506	0.0462	0.2236	No	0.9759	0.288	0.0035	0.0196	No	No
102370_at	Dhrs8	dehydrogenase/reductase (SDR family) member 8	0.899	0.0079	0.0052	0.0158	Yes	0.8582	0.0211	0.606	0.0645	No	Yes
102371_at	Nr4a1	nuclear receptor subfamily 4, group A, member 1	1.0029	0.951	0.4407	0.4701	No	0.9287	0.302	0.0038	0.513	No	No
102372_at	Igj	immunoglobulin joining chain	1.1135	0.1756	0.0114	0.1435	No	1.0305	0.309	0.0005	0.0986	No	No
102373_at	Enpep	glutamyl aminopeptidase	1.1003	0.1959	0.0007	0.1082	No	1.0031	0.586	0.0003	0.109	No	No
102374_at	Dscr1l2	Down syndrome critical region gene 1-like 2	0.9739	0.311	0.0914	0.4039	No	1.0276	0.247	0.0276	0.0964	No	No
102375_at	Smyd5	SET and MYND domain containing 5	0.9572	0.3749	0.0738	0.1333	No	1.0193	0.233	0.003	0.0171	No	No
102376_r_at	Pcp2	Purkinje cell protein 2 (L7)	0.9923	0.8425	0.0454	0.6984	No	1.0255	0.628	0.0254	0.701	No	No
102378_at	Sspn	sarcospan	1.0197	0.6653	0.9636	0.7534	No	0.9278	0.284	0.0507	0.575	No	No
102379_at	Rassf1	Ras association (RalGDS/AF-6) domain family 1	0.9757	0.6133	0.8737	0.606	No	1.0209	0.497	0.752	0.301	No	No
102380_s_at	Hoxd4	homeo box D4	1.0163	0.7456	0.3763	0.4863	No	1.0424	0.631	0.134	0.138	No	No
102381_at	Acsl4	acyl-CoA synthetase long-chain family member 4	1.1752	0.201	0.0016	0.681	No	0.9621	0.919	0.0022	0.648	No	No
102382_at	Arntl	aryl hydrocarbon receptor nuclear translocator-like	1.0319	0.372	0.004	0.1158	No	1.0407	0.329	0.0084	0.0686	No	No
102383_at	5730593F17 Rik	RIKEN cDNA 5730593F17 gene	1.0542	0.5049	0.5363	0.1687	No	1.0443	0.766	0.0042	0.36	No	No
102384_at	Smarca2	SWI/SNF related, matrix associated, actin dependent regulator of chromatin, subfamily a, member 2	1.1558	0.1214	0.0106	0.0377	No	1.0916	0.125	0.0069	0.0302	No	No
102385_at	Wdr43	WD repeat domain 43	0.9875	0.7849	0.0738	0.6577	No	1.006	0.801	0.0237	0.14	No	No
102387_at	LOC670726	similar to ribosomal protein L24-like	1.0136	0.3827	0.3114	0.127	No	0.9278	0.123	0.362	0.125	No	No
102389_s_at	Gap43	growth associated protein 43	1.1472	0.1074	0.0021	0.2657	No	1.0683	0.233	0.0021	0.699	No	No
102393_at	Cryaa	crystallin, alpha A	1.0555	0.0378	0.0015	0.0681	No	0.9878	0.699	0.934	0.423	No	No
102395_at	Pmp22	peripheral myelin protein	0.9328	0.0302	0.152	0.5271	No	0.9245	0.121	0.0448	0.183	No	No
102396_at	Ift172	intraflagellar transport 172 homolog (Chlamydomonas)	1.0043	0.8779	0.0016	0.4894	No	1.0022	0.851	0.0085	0.0864	No	No
102397_at	Cbfa2t3h	core-binding factor, runt domain, alpha subunit 2, translocated to, 3 homolog (human)	1.0458	0.3587	0.0159	0.2807	No	1.1564	0.076	0.0119	0.611	No	No
102398_at	Rxrb	retinoid X receptor beta	0.9207	0.0004	0.0001	0.0012	No	0.9159	0.0228	0.0097	0.0281	No	No
102399_at	Rbpms	RNA binding protein gene with multiple splicing	0.9766	0.5577	0.9448	0.228	No	0.9968	0.947	0.013	0.219	No	No
102400_at	AU014645	expressed sequence AU014645	1.0218	0.4415	0.0077	0.7038	No	1.0441	0.311	0.0066	0.58	No	No
102401_at	Irf1	interferon regulatory factor 1	1.0119	0.7558	0.8403	0.9078	No	1.0674	0.0045	0.0027	0.0306	Yes	No
102402_at	Gbas	glioblastoma amplified sequence	0.9588	0.576	0.8388	0.6913	No	0.8966	0.0663	0.0371	0.352	No	No
102403_at	Cdc45l	cell division cycle 45 homolog (S. cerevisiae)-like	1.0402	0.273	0.9333	0.5578	No	1.0457	0.222	0.0392	0.0194	No	No
102404_at	Lamc1	laminin, gamma 1	1.0582	0.2416	0.04	0.1403	No	1.0047	0.959	0.393	0.135	No	No
102405_at	Mag	myelin-associated glycoprotein	0.9175	0.3137	0.2535	0.3889	No	0.9705	0.218	0.0342	0.0484	No	No
102406_at	Cma1	chymase 1, mast cell	0.9861	0.7721	0.0113	0.9428	No	1.0002	0.989	0.107	0.508	No	No
102407_at	Cma1	chymase 1, mast cell	1.0074	0.8123	0	0.0024	No	0.9361	0.907	0.0003	0.0072	No	No
102408_g_at	Cma1	chymase 1, mast cell	0.9229	0.0044	0.0001	0.0247	Yes	0.9431	0.236	0.244	0.643	No	No
102409_at	Lsm8	LSM8 homolog, U6 small nuclear RNA associated (S. cerevisiae)	1.043	0.4046	0.7938	0.2558	No	1.0507	0.144	0.0088	0.0386	No	No
102410_at	Hs3st1	heparan sulfate (glucosamine) 3-O-sulfotransferase 1	0.9438	0.3284	0.0047	0.4246	No	0.9501	0.193	0.0011	0.118	No	No
102411_at	Zfp239	zinc finger protein 239	1.0738	0.3386	0.6003	0.766	No	1.0276	0.611	0.142	0.952	No	No
102412_at	Nup107	nucleoporin 107	1.0021	0.9365	0.0821	0.0506	No	0.9905	0.827	0.328	0.118	No	No
102413_at	Lmo1	LIM domain only 1	0.9735	0.5544	0.769	0.6648	No	0.9949	0.928	0.0765	0.56	No	No
102414_i_at	Dnajc3	DnaJ (Hsp40) homolog, subfamily C, member 3	0.8002	0.0346	0.2291	0.0479	No	0.8147	0.0065	0.0501	0.197	Yes	Yes
102415_r_at	Dnajc3	DnaJ (Hsp40) homolog, subfamily C, member 3	0.8762	0.0273	0.1895	0.0219	No	0.8191	0.012	0.0088	0.0724	No	No
102416_at	Cyp17a1	cytochrome P450, family 17, subfamily a, polypeptide 1	0.9665	0.3023	0.0571	0.0149	No	1.0692	0.257	0.741	0.198	No	No
102418_at	Tex19	testis expressed gene 19	1.0827	0.3609	0.0408	0.3017	No	0.9249	0.239	0.0001	0.0122	No	No
102419_at	Rarg	retinoic acid receptor, gamma	0.9534	0.3033	0.0004	0.4485	No	0.958	0.437	0.0003	0.342	No	No
102421_at	2400010G15 Rik	RIKEN cDNA 2400010G15 gene	1.0381	0.2934	0.0008	0.5203	No	1.0152	0.512	0.0001	0.325	No	No
102423_at	Uxs1	UDP-glucuronate decarboxylase 1	1.0596	0.4273	0.3366	0.5586	No	1.2098	0.0354	0.0823	0.174	No	No
102424_at	Ccl3	chemokine (C-C motif) ligand 3	0.9938	0.8566	0.604	0.1803	No	0.9475	0.403	0.0531	0.0524	No	No
102425_at	Tle1	transducin-like enhancer of split 1, homolog of Drosophila E(spl)	0.9165	0.2812	0.0128	0.262	No	0.8834	0.104	0.0077	0.13	No	No
102426_at	Casq1	calsequestrin 1	0.7668	0.0132	0.0073	0.0261	No	0.8684	0.0027	0.0018	0.0175	Yes	Yes
102427_at	Fyttd1	forty-two-three domain containing 1	1.0215	0.7562	0.0276	0.0862	No	0.9484	0.549	0.0228	0.113	No	No
102429_at	Slc22a12	solute carrier family 22 (organic anion/cation transporter), member 12	0.9791	0.7215	0.2982	0.6128	No	1.0141	0.524	0.0016	0.129	No	No
102430_at	Myd88	myeloid differentiation primary response gene 88	1.0311	0.5097	0.0254	0.8359	No	1.0034	0.888	0.0143	0.883	No	No
102431_at	Mapt	microtubule-associated protein tau	1.1108	0.1623	0.0001	0.2625	No	1.0387	0.455	0.0003	0.49	No	No

102476_f_at	Xpo7	Exportin 7	0.9834	0.8186	0.0011	0.0664	No	1.0258	0.835	0.0012	0.147	No	No
102477_at	---	Transcribed locus	1.0496	0.1078	0.043	0.0603	No	1.0663	0.398	0.902	0.757	No	No
102478_f_at	Fbxw4	F-box and WD-40 domain protein 4	0.9797	0.6601	0.179	0.2209	No	1.0522	0.0749	0.309	0.0122	No	No
102552_at	Gja3	gap junction membrane channel protein alpha 3	0.9275	0.0054	0.2584	0.0049	No	0.942	0.246	0.0339	0.305	No	No
102553_at	---	Immunoglobulin kappa chain complex	1.0047	0.9178	0.0089	0.1981	No	0.9871	0.599	0.0013	0.345	No	No
102554_at	Olfr16	olfactory receptor 16	1.131	0.0087	0.2063	0.0105	Yes	1.0081	0.677	0.0291	0.0173	No	No
102555_at	---	---	1.0425	0.6682	0.4909	0.1494	No	1.1536	0.21	0.0634	0.0214	No	No
102556_at	Adra2a	adrenergic receptor, alpha 2a	0.8313	0.0322	0.0669	0.0951	No	0.9435	0.2	0.097	0.293	No	No
102557_at	LOC621893	similar to histone H2A	1.0499	0.2958	0.7317	0.1579	No	1.0858	0.286	0.391	0.257	No	No
102558_at	Hist2h2be	histone 2, H2be	0.9985	0.9832	0.0326	0.6451	No	0.9406	0.466	0.0056	0.498	No	No
102559_at	Bmp2	bone morphogenetic protein 2	1.013	0.032	0	0.0001	No	0.9141	0.309	0.005	0.0179	No	No
102560_at	Cntn4	contactin 4	1.1008	0.2387	0.1615	0.3402	No	1.0316	0.708	0.552	0.955	No	No
102561_at	Atp5k	ATP synthase, H+ transporting, mitochondrial F1F0 complex, subunit e	1.0772	0.216	0.1296	0.0979	No	0.9886	0.94	0.618	0.0983	No	No
102562_at	---	---	0.9181	0.2074	0.1825	0.3376	No	1.0341	0.281	0.029	0.0217	No	No
102566_at	Dcc	deleted in colorectal carcinoma	1.0155	0.8327	0.0924	0.4536	No	1.1173	0.2	0.329	0.0823	No	No
102567_at	Hoxd13	homeo box D13	0.9896	0.8934	0.0305	0.7201	No	0.9921	0.678	0.0039	0.151	No	No
102568_at	Nobox	NOBOX oogenesis homeobox	0.9727	0.2374	0.0425	0.1042	No	0.9573	0.454	0.0174	0.439	No	No
102569_at	Atoh1	atonal homolog 1 (Drosophila)	0.9815	0.8436	0.0494	0.6971	No	1.0019	0.822	0.0033	0.373	No	No
102570_at	---	---	1.0892	0.1095	0.8172	0.0321	No	1.1319	0.0052	0.0039	0.0271	Yes	No
102571_at	Gjb6	gap junction membrane channel protein beta 6	1.0527	0.0821	0.0015	0.0103	No	1.0731	0.0943	0.0176	0.548	No	No
102572_at	Rasgrf2	RAS protein-specific guanine nucleotide-releasing factor 2	0.9748	0.7837	0.22	0.7451	No	0.9724	0.736	0.315	0.657	No	No
102573_at	Nxph1	neurexophilin 1	1.0438	0.414	0.0203	0.9361	No	0.946	0.38	0.0103	0.607	No	No
102574_at	Fgf11	fibroblast growth factor 11	0.9605	0.4238	0.1466	0.8975	No	0.948	0.246	0.8	0.295	No	No
102575_at	Orm3	orosomucoid 3	0.9527	0.3262	0.3114	0.5063	No	1.0287	0.608	0.578	0.53	No	No
102576_at	Hrh1	histamine receptor H 1	1.0029	0.9648	0.0846	0.8486	No	1.1728	0.21	0.921	0.573	No	No
102577_at	Kcnab3	potassium voltage-gated channel, shaker-related subfamily, beta member 3	1.0904	0.1567	0.0132	0.6895	No	1.07	0.362	0.018	0.493	No	No
102578_at	Pax5	paired box gene 5	0.9296	0.246	0.147	0.8013	No	0.9817	0.719	0.669	0.202	No	No
102579_f_at	Hoxa6	homeo box A6	0.9799	0.5192	0.0747	0.0751	No	1.0291	0.896	0.172	0.561	No	No
102580_r_at	Hoxa6	homeo box A6	1.0069	0.8625	0.0042	0.4233	No	0.9996	0.982	0.0378	0.716	No	No
102581_at	Mycs	myc-like oncogene, s-myc protein	0.9644	0.6055	0.2232	0.756	No	0.9706	0.618	0.702	0.962	No	No
102582_at	Apc2	adenomatosis polyposis coli 2	0.9951	0.9231	0.0041	0.4863	No	1.0468	0.124	0.0002	0.918	No	No
102583_at	Olfr138	olfactory receptor 138	1.0507	0.1179	0.2421	0.0375	No	1.1117	0.0473	0.762	0.012	No	No
102584_at	Dyrk1b	dual-specificity tyrosine-(Y)-phosphorylation regulated kinase 1b	0.9609	0.1333	0.0008	0.0681	No	1.0468	0.25	0.0001	0.0023	No	No
102585_f_at	Gm1419 /// Gm1502 /// Igkv4-61 /// Igkv4-69 /// LOC636677 /// Igkv4-59 /// LOC672342	gene model 1419, (NCBI) /// gene model 1502, (NCBI) /// immunoglobulin kappa chain variable 4-61 /// immunoglobulin kappa chain variable 4-69 /// similar to Ig kappa chain V-IV region S107B precursor /// immunoglobulin kappa chain variable 4-59 /// similar to Ig kappa chain V-IV region S107B precursor	1.0433	0.3034	0.2602	0.1863	No	1.0293	0.508	0.697	0.395	No	No
102586_at	Ror1	receptor tyrosine kinase-like orphan receptor 1	1.0316	0.5674	0.0193	0.6209	No	1.0339	0.53	0.0312	0.163	No	No
102599_at	Tpt1	tumor protein, translationally-controlled 1	1.0369	0.2397	0.4815	0.106	No	1.0066	0.69	0.049	0.0705	No	No
102612_at	Nrl	neural retina leucine zipper gene	1.0921	0.1025	0.4662	0.0427	No	1.0098	0.654	0.0067	0.173	No	No
102613_at	Tiaf2	TGF-beta1-induced anti-apoptotic factor 2	1.0487	0.4716	0.7475	0.5406	No	1.1006	0.233	0.0467	0.644	No	No
102614_at	Prox1	prospero-related homeobox 1	1.0101	0.8578	0.1982	0.6198	No	1.0619	0.331	0.0298	0.128	No	No
102619_at	Fgd3	FYVE, RhoGEF and PH domain containing 3	1.0677	0.4175	0.0954	0.5685	No	1.1665	0.202	0.0904	0.659	No	No
102620_at	Dnajb5	DnaJ (Hsp40) homolog, subfamily B, member 5	0.9203	0.2108	0.0089	0.5697	No	0.9748	0.516	0.0076	0.449	No	No
102621_at	Cdon	cell adhesion molecule-related/down-regulated by oncogenes	0.9762	0.7544	0.3797	0.2605	No	1.008	0.879	0.541	0.0743	No	No
102622_r_at	Clcn1	chloride channel 1	1.0481	0.3865	0.4913	0.8361	No	1.014	0.433	0.0019	0.0243	No	No
102623_at	Sema3f	sema domain, immunoglobulin domain (Ig), short basic domain, secreted, (semaphorin) 3 F	1.0092	0.8634	0.1169	0.3093	No	0.9673	0.596	0.504	0.962	No	No
102624_at	Stc2	stanniocalcin 2	0.8841	0.0386	0.4782	0.7359	No	0.9365	0.0694	0.0275	0.498	No	No
102625_f_at	Zfp54	zinc finger protein 54	1.0101	0.6947	0.1008	0.1504	No	1.2338	0.146	0.632	0.057	No	No
102626_r_at	Zfp54	zinc finger protein 54	1.0262	0.4673	0.0357	0.1001	No	1.0479	0.898	0.0001	0.208	No	No
102627_at	Igf2bp1	insulin-like growth factor 2 mRNA binding protein 1	1.0705	0.0841	0.0568	0.0751	No	1.092	0.0145	0.0038	0.0271	No	No
102628_at	Fhit	fragile histidine triad gene	0.9601	0.5752	0.9602	0.8824	No	0.9892	0.83	0.219	0.679	No	No
102629_at	Tnf	tumor necrosis factor	1.0439	0.2016	0.353	0.0937	No	1.0741	0.176	0.367	0.572	No	No
102630_s_at	Lta	lymphotoxin A	0.9098	0.2598	0.0067	0.3989	No	0.9863	0.491	0.0002	0.225	No	No
102631_at	Blm	Bloom syndrome homolog (human)	0.9834	0.7604	0.0355	0.5125	No	0.8891	0.0695	0.0085	0.769	No	No
102632_at	Aspm	asp (abnormal spindle)-like, microcephaly associated (Drosophila)	0.9883	0.4928	0.0019	0.1078	No	0.9693	0.589	0.178	0.945	No	No
102633_at	Grap2	GRB2-related adaptor protein 2	1.0283	0.393	0.0633	0.7193	No	1.0025	0.969	0.0572	0.486	No	No
102635_at	Vti1a	vesicle transport through interaction with t-SNAREs homolog 1A (yeast)	1.0179	0.7854	0.0452	0.1783	No	1.0015	0.993	0.352	0.323	No	No
102636_at	Klc2	kinesin light chain 2	0.991	0.8361	0.051	0.5813	No	0.9351	0.123	0.386	0.904	No	No
102637_at	Tgfbr3	transforming growth factor, beta receptor III	0.9095	0.0912	0.7928	0.2887	No	0.9679	0.209	0.0104	0.0821	No	No
102638_at	Cst7	cystatin F (leukocystatin)	0.9926	0.9127	0.9294	0.2636	No	0.9131	0.289	0.302	0.705	No	No
102639_at	Chst2	carbohydrate sulfotransferase 2	1.1319	0.0103	0.0155	0.5605	No	1.0433	0.114	0.0292	0.61	No	No
102640_at	ICRFP703B 1614Q5.5	ICRFP703B1614Q5.5 gene	1.0496	0.3824	0.3408	0.885	No	1.1169	0.13	0.0086	0.458	No	No
102641_at	Sfpi1	SFFV proviral integration 1	1.0087	0.6102	0.0008	0.9573	No	0.9823	0.443	0.0007	0.131	No	No
102642_at	Slc11a2	solute carrier family 11 (proton-coupled divalent metal ion transporters), member 2	0.9861	0.7343	0.0092	0.1111	No	1.0534	0.0913	0.0081	0.014	No	No
102643_at	Hoxa2	homeo box A2	0.9832	0.4169	0.0047	0.7988	No	1.0398	0.345	0.489	0.511	No	No
102644_at	Mdfic	MyoD family inhibitor domain containing	0.9533	0.3238	0.0033	0.2221	No	0.925	0.314	0.0395	0.641	No	No
102645_at	Eea1	early endosome antigen 1	1.0243	0.7098	0.0028	0.2611	No	1.0029	0.794	0.0012	0.305	No	No
102646_at	Usp34	ubiquitin specific peptidase 34	0.8818	0.1453	0.0116	0.7947	No	0.9702	0.603	0.0199	0.257	No	No
102647_g_at	Usp34	ubiquitin specific peptidase 34	0.9928	0.8721	0.0005	0.1221	No	1.0061	0.471	0.0004	0.0351	No	No
102648_at	Sftpd	surfactant associated protein D	1.0713	0.2715	0.0226	0.1011	No	1.0189	0.499	0.013	0.0284	No	No
102649_s_at	Raet1a /// Raet1b /// Raet1c /// Raet1d /// Raet1e	retinoic acid early transcript 1, alpha /// retinoic acid early transcript beta /// retinoic acid early transcript gamma /// retinoic acid early transcript delta /// retinoic acid early transcript 1E	0.9441	0.286	0.4599	0.1656	No	0.9878	0.388	0.137	0.0024	No	No
102650_at	Rgs11	regulator of G-protein signaling 11	1.0373	0.2408	0.8668	0.3288	No	1.0087	0.873	0.245	0.469	No	No
102651_at	Gck	glucokinase	1.0033	0.9507	0.4543	0.7329	No	1.0945	0.106	0.823	0.419	No	No
102652_at	Pou3f1	POU domain, class 3, transcription factor 1	0.9591	0.4326	0.6773	0.2856	No	1.0714	0.179	0.104	0.36	No	No
102653_at	Ryr2	ryanodine receptor 2, cardiac	0.9946	0.9201	0.6229	0.6009	No	0.8406	0.613	0.429	0.576	No	No
102654_at	Gata1	GATA binding protein 1	1.0002	0.9942	0.1373	0.2007	No	0.9894	0.789	0.0069	0.97	No	No
102655_at	Itga4	integrin alpha 4	0.9688	0.5967	0.0505	0.8053	No	1.1568	0.0641	0.0024	0.029	No	No
102656_at	Itga4	integrin alpha 4	1.0884	0.3448	0.1598	0.3195	No	1.0382	0.633	0.344	0.538	No	No
102657_at	Hlx1	H2.0-like homeo box 1 (Drosophila)	0.9981	0.9533	0.0942	0.4542	No	1.0186	0.681	0.0629	0.373	No	No
102658_at	Il1r2	interleukin 1 receptor, type II	0.9179	0.3332	0.6674	0.6952	No	0.8619	0.0204	0.0763	0.491	No	No
102659_at	Cln3	ceroid lipofuscinosis, neuronal 3, juvenile (Batten, Spielmeyer-Vogt disease)	1.038	0.0047	0.018	0.0658	Yes	1.06	0.0529	0.0168	0.0484	No	No
102660_at	Hoxc4	homeo box C4	1.0043	0.8992	0.2678	0.4655	No	1.0378	0.636	0.508	0.211	No	No
102661_at	Egr2	early growth response 2	1.0056	0.8622	0.0019	0.1628	No	1.0044	0.977	0.103	0.645	No	No
102662_at	Asgr2	asialoglycoprotein receptor 2	0.9733	0.7243	0.0095	0.6823	No	1.0052	0.923	0.251	0.674	No	No
102663_at	Plaur	plasminogen activator, urokinase receptor	1.0009	0.9859	0.6951	0.6055	No	1.0538	0.361	0.145	0.419	No	No
102664_at	Cdk5r1	cyclin-dependent kinase 5, regulatory subunit (p35) 1	1.0411	0.5474	0.5452	0.3396	No	1.1302	0.249	0.423	0.11	No	No
102665_at	Trh	thyrotropin releasing hormone	0.9989	0.9821	0.0152	0.7089	No	0.9796	0.486	0.0079	0.61	No	No
102666_at	Tyr	tyrosinase	0.9944	0.9079	0.0206	0.0427	No	1.0253	0.4	0.0018	0.121	No	No
102667_at	Wnt3a	wingless-related MMTV integration site 3A	1.0511	0.2846	0.287	0.2506	No	1.0439	0.481	0.877	0.742	No	No
102668_at	Ppara	peroxisome proliferator activated receptor alpha	0.9684	0.4842	0.0731	0.4842	No	0.9253	0.195	0.58	0.298	No	No
102669_at	Siglec1	sialic acid binding Ig-like lectin 1, sialoadhesin	0.9743	0.7482	0.0854	0.677	No	0.8941	0.34	0.0073	0.0567	No	No

102670_at	Vps54	vacuolar protein sorting 54 (yeast)	0.9401	0.3044	0.0502	0.8228	No	0.9909	0.972	0.0034	0.0577	No	No
102671_at	Creb1	cAMP responsive element binding protein 1	0.992	0.9327	0.8053	0.9088	No	1.04	0.348	0.0926	0.34	No	No
102672_g_at	Creb1	cAMP responsive element binding protein 1	0.9705	0.6045	0.0745	0.3559	No	1.0763	0.0536	0.79	0.0153	No	No
102673_at	Creb1	cAMP responsive element binding protein 1	1.1031	0.062	0.0045	0.0124	No	1.1069	0.0773	0.0011	0.0418	No	No
102674_at	Cnr2	cannabinoid receptor 2 (macrophage)	0.8994	0.2877	0.1082	0.4649	No	0.6586	0.387	0.0748	0.555	No	No
102675_at	Zp3	zona pellucida glycoprotein 3	0.9697	0.3994	0.2059	0.196	No	1.0028	0.952	0.0529	0.773	No	No
102676_at	Vhlh	von Hippel-Lindau syndrome homolog	0.9989	0.9529	0.0049	0.8794	No	0.967	0.662	0.0062	0.756	No	No
102677_at	Arhgdig	Rho GDP dissociation inhibitor (GDI) gamma	1.0351	0.2611	0.0149	0.3926	No	1.0934	0.0236	0.0002	0.0052	No	No
102678_at	Trim21	tripartite motif protein 21	1.0344	0.3192	0.9864	0.6581	No	1.02	0.667	0.972	0.706	No	No
102679_at	Madcam1	mucosal vascular addressin cell adhesion molecule 1	0.9452	0.218	0.1266	0.6202	No	0.9994	0.923	0.222	0.0151	No	No
102680_g_at	Madcam1	mucosal vascular addressin cell adhesion molecule 1	0.9842	0.7926	0.3569	0.4189	No	1.0483	0.274	0.0354	0.572	No	No
102681_at	Tnfrsf4	tumor necrosis factor receptor superfamily, member 4	1.0699	0.1013	0.0056	0.0985	No	1.1248	0.11	0.545	0.392	No	No
102682_at	Epha8	Eph receptor A8	1.0181	0.6491	0.6341	0.5499	No	0.914	0.113	0.0013	0.0479	No	No
102683_at	Slc30a3	solute carrier family 30 (zinc transporter), member 3	1.0644	0.092	0.0403	0.0186	No	0.8522	0.0888	0.0001	0.0034	No	No
102684_at	Smr2	submaxillary gland androgen regulated protein 2	1.0052	0.9375	0.0621	0.6188	No	1.1697	0.102	0.0162	0.251	No	No
102685_at	Smr2	submaxillary gland androgen regulated protein 2	0.9566	0.3934	0.0554	0.5801	No	0.875	0.0206	0.777	0.0211	No	No
102686_at	Suv420h2	suppressor of variegation 4-20 homolog 2 (Drosophila)	0.9455	0.1947	0.0227	0.2755	No	0.9567	0.438	0.0105	0.594	No	No
102687_at	Suv39h2	suppressor of variegation 3-9 homolog 2 (Drosophila)	0.9959	0.9257	0.1155	0.9394	No	1.0578	0.183	0.0014	0.579	No	No
102688_f_at	Gzmd	granzyme D	1.0101	0.8324	0.3944	0.4992	No	0.9932	0.991	0.918	0.305	No	No
102689_at	Tapbp	TAP binding protein	0.9762	0.5635	0.8445	0.879	No	1.0408	0.325	0.503	0.198	No	No
102690_at	Cyp2b19	cytochrome P450, family 2, subfamily b, polypeptide 19	1.02	0.7248	0.6614	0.2624	No	1.0075	0.837	0.0587	0.898	No	No
102691_at	Zfp385	zinc finger protein 385	0.9852	0.454	0.0017	0.0522	No	0.9818	0.439	0.0008	0.281	No	No
102692_s_at	H2-T18 /// LOC633417 /// LOC674370	histocompatibility 2, T region locus 18 /// similar to histocompatibility 2, T region locus 3 /// similar to histocompatibility 2, T region locus 3	1.081	0.0088	0.0001	0.0841	Yes	1.1096	0.0949	0.0038	0.627	No	No
102693_f_at	Klk1b26	kallikrein 1-related petidase b26	0.9738	0.6988	0.0355	0.8378	No	0.9102	0.155	0.0029	0.949	No	No
102694_at	Psg16	pregnancy specific glycoprotein 16	0.9853	0.8178	0.2079	0.7833	No	0.8337	0.625	0.236	0.559	No	No
102695_at	Tcrg-C	T-cell receptor gamma, constant region	1.0734	0.1116	0.2093	0.9852	No	0.9551	0.395	0.267	0.515	No	No
102696_s_at	Pitpnb	phosphatidylinositol transfer protein, beta	0.994	0.9568	0.0856	0.9936	No	0.942	0.538	0.0373	0.113	No	No
102697_at	Pitpnb	phosphatidylinositol transfer protein, beta	1.0242	0.2922	0.0238	0.3184	No	1.0057	0.871	0.206	0.727	No	No
102698_at	Epas1	endothelial PAS domain protein 1	1.0073	0.7651	0.082	0.0017	No	0.9558	0.0209	0	0.109	No	No
102699_at	Mx2	myxovirus (influenza virus) resistance 2	0.9517	0.474	0.7517	0.1608	No	1.0527	0.56	0.548	0.0868	No	No
102700_at	Tbr1	T-box brain gene 1	1.0496	0.2378	0.0478	0.4416	No	1.1043	0.265	0.723	0.097	No	No
102701_at	Cyp2b10	cytochrome P450, family 2, subfamily b, polypeptide 10	0.9694	0.4209	0.2018	0.2391	No	0.9409	0.243	0.0817	0.316	No	No
102702_at	Cuedc1	CUE domain containing 1	0.935	0.4038	0.0634	0.5103	No	0.9527	0.512	0.106	0.292	No	No
102703_s_at	Aqp4	aquaporin 4	0.949	0.638	0.1852	0.2901	No	0.9524	0.802	0.109	0.319	No	No
102704_at	Aqp4	aquaporin 4	1.0944	0.2747	0.0182	0.2213	No	1.0215	0.836	0.0025	0.512	No	No
102705_at	Il2	interleukin 2	1.0166	0.7033	0.4943	0.2047	No	1.046	0.497	0.0994	0.156	No	No
102706_i_at	Serpina3c	serine (or cysteine) peptidase inhibitor, clade A, member 3C	1.017	0.6071	0.1313	0.5317	No	1.2486	0.163	0.157	0.136	No	No
102707_f_at	Serpina3c	serine (or cysteine) peptidase inhibitor, clade A, member 3C	0.9329	0.1448	0.007	0.0446	No	0.9964	0.798	0.0164	0.938	No	No
102708_at	Btn1a1	butyrophilin, subfamily 1, member A1	1.0174	0.6977	0.9314	0.1274	No	1.0543	0.279	0.0503	0.0433	No	No
102709_at	Hira	histone cell cycle regulation defective homolog A (S. cerevisiae)	1.0203	0.6622	0.75	0.163	No	0.884	0.0373	0.325	0.725	No	No
102710_at	Apbb1ip	amyloid beta (A4) precursor protein-binding, family B, member 1 interacting protein	1.0684	0.0579	0	0.0381	No	0.9248	0.25	0.0054	0.765	No	No
102711_at	Rgs14	regulator of G-protein signaling 14	1.0422	0.3384	0.0134	0.4668	No	1.037	0.206	0.0047	0.0323	No	No
102712_at	Saa3	serum amyloid A 3	0.7467	0.0028	0.0331	0.6511	Yes	0.8957	0.0117	0.0005	0.596	No	Yes
102713_at	Gata4	GATA binding protein 4	0.9445	0.2031	0.9908	0.789	No	0.9557	0.326	0.955	0.919	No	No
102714_at	Hspa1l	heat shock protein 1-like	0.9291	0.263	0.8157	0.1891	No	0.8652	0.029	0.0039	0.0304	No	No
102715_at	Nr2f1	nuclear receptor subfamily 2, group F, member 1	10.3371	0	0.0004	0.0247	Yes	11.145	0	0.0003	0.679	Yes	Yes
102716_at	Eya3	eyes absent 3 homolog (Drosophila)	1.024	0.6679	0.4982	0.0894	No	1.1259	0.175	0.156	0.0756	No	No
102717_at	Oas1g	2'-5' oligoadenylate synthetase 1G	1.0229	0.3986	0.5807	0.7757	No	0.9543	0.695	0.79	0.823	No	No
102718_at	Ccr5	chemokine (C-C motif) receptor 5	1.006	0.9202	0.6839	0.7243	No	1.2644	0.22	0.853	0.371	No	No
102719_f_at	Ccr5	chemokine (C-C motif) receptor 5	0.8867	0.2188	0.1669	0.2454	No	0.7943	0.0803	0.033	0.0111	No	No
102720_at	Tek	endothelial-specific receptor tyrosine kinase	0.8823	0.0083	0.0002	0.1115	Yes	0.8794	0.0152	0.0004	0.529	No	Yes
102721_at	AI324046	expressed sequence AI324046	0.9663	0.4936	0.1344	0.8597	No	1.0044	0.98	0.311	0.209	No	No
102722_g_at	AI324046	expressed sequence AI324046	1.0531	0.0226	0.2876	0.4203	No	1.1533	0.021	0.0165	0.461	No	No
102723_at	AI324046	expressed sequence AI324046	0.9611	0.4334	0.0321	0.1522	No	1.0402	0.526	0.0095	0.944	No	No
102724_at	Rabep1	rabaptin, RAB GTPase binding effector protein 1	1.07	0.06	0.1836	0.0147	No	1.0464	0.287	0.175	0.0145	No	No
102725_at	Kcnab1	potassium voltage-gated channel, shaker-related subfamily, beta member 1	0.9259	0.1991	0.1158	0.1885	No	0.8816	0.0911	0.277	0.111	No	No
102726_at	Tac1	tachykinin 1	1.0857	0.1993	0	0.6724	No	1.0444	0.147	0	0.182	No	No
102727_at	Bdnf	brain derived neurotrophic factor	0.9173	0.0899	0	0.6232	No	1.0076	0.0209	0	0.0004	No	No
102728_f_at	Gzme	granzyme E	0.9493	0.298	0.0166	0.581	No	1.0506	0.56	0.0578	0.112	No	No
102729_f_at	Hsd3b6	hydroxysteroid dehydrogenase-6, delta<5>-3-beta	0.9837	0.8214	0.7585	0.6931	No	1.0049	0.952	0.585	0.245	No	No
102730_at	H2-M3	histocompatibility 2, M region locus 3	0.9929	0.906	0.9354	0.7843	No	1.0822	0.384	0.0812	0.103	No	No
102731_g_at	H2-M3	histocompatibility 2, M region locus 3	1.0832	0.1601	0.0476	0.3115	No	0.9582	0.184	0.0318	0.112	No	No
102732_at	Tln1	talin 1	1.1315	0.0002	0.0002	0.0713	Yes	1.0185	0.613	0.0228	0.444	No	No
102733_at	Gzmc	granzyme C	1.0471	0.5578	0.0536	0.9346	No	1.0896	0.0914	0.176	0.185	No	No
102734_at	Birc2	baculoviral IAP repeat-containing 2	0.9702	0.3257	0.0216	0.5441	No	0.9409	0.169	0.488	0.298	No	No
102735_at	Tex9	testis expressed gene 9	0.8816	0.0211	0.0089	0.0407	No	0.9688	0.608	0.584	0.827	No	No
102736_at	Ccl2	chemokine (C-C motif) ligand 2	1.2809	0.0192	0.8215	0.1271	No	1.1355	0.045	0.392	0.0268	No	No
102737_at	Edn1	endothelin 1	0.7869	0.0087	0.0056	0.2613	Yes	0.934	0.303	0.0099	0.966	No	No
102738_s_at	Edn1	endothelin 1	1.0524	0.2118	0.0004	0.0139	No	1.042	0.13	0.0002	0.0475	No	No
102739_s_at	Smr1 /// Smr3	submaxillary gland androgen regulated protein 1 /// submaxillary gland androgen regulated protein 3	1.0378	0.5434	0.3745	0.3789	No	1.0354	0.358	0.611	0.0091	No	No
102740_at	Mkrn3	makorin, ring finger protein, 3	1.1585	0.0736	0.0056	0.1289	No	1.1626	0.153	0.0118	0.192	No	No
102741_at	Adar	adenosine deaminase, RNA-specific	1.0488	0.2712	0.5586	0.375	No	1.0182	0.614	0.0928	0.309	No	No
102742_g_at	Mapt	microtubule-associated protein tau	1.0934	0.1298	0.0002	0.0308	No	1.1258	0.002	0	0.0011	No	No
102743_at	Mapt	microtubule-associated protein tau	1.0004	0.9913	0.0011	0.1805	No	0.9821	0.448	0.0001	0.541	No	No
102744_at	Tcrg /// LOC624071 /// LOC631361 /// LOC670271	T-cell receptor gamma chain /// T cell receptor gamma chain /// similar to T-cell receptor gamma chain C region C7.5 /// similar to T-cell receptor gamma chain C region C7.5 /// region containing T-cell receptor gamma chain; T-cell receptor gamma, variable 4	0.9917	0.9057	0.0526	0.8284	No	1.0213	0.97	0.013	0.0979	No	No
102745_at	Tcrg /// LOC670271	T-cell receptor gamma chain /// region containing T-cell receptor gamma chain; T-cell receptor gamma, variable 4	0.9887	0.8637	0.0329	0.2211	No	0.97	0.62	0.0329	0.703	No	No
102746_at	BC089491	cDNA sequence BC089491	0.9549	0.4494	0.0423	0.3798	No	0.9809	0.559	0.0527	0.963	No	No
102747_at	Tcte1	t-complex-associated testis expressed 1	1.0104	0.7645	0.1077	0.9846	No	1.0293	0.602	0.0016	0.0279	No	No
102748_at	---	---	0.9772	0.5545	0.0195	0.4184	No	1.0367	0.562	0.045	0.508	No	No
102749_at	Cox7a1	cytochrome c oxidase, subunit VIIa 1	0.9932	0.9159	0.1185	0.3549	No	0.9155	0.114	0.248	0.0092	No	No
102750_at	Apba3	amyloid beta (A4) precursor protein-binding, family A, member 3	0.9657	0.471	0.0277	0.4396	No	1.0262	0.156	0.0003	0.612	No	No
102751_at	Tgfb3	transforming growth factor, beta 3	1.0325	0.4513	0.136	0.2527	No	1.0158	0.634	0.0065	0.31	No	No
102752_at	Cyfip1	cytoplasmic FMR1 interacting protein 1	0.9898	0.8781	0.0093	0.3171	No	0.9651	0.652	0.0148	0.689	No	No
102753_at	Men1	multiple endocrine neoplasia 1	0.9258	0.0555	0.0047	0.0647	No	1.1862	0.0815	0.835	0.627	No	No
102754_at	Birc6	baculoviral IAP repeat-containing 6	1.0454	0.7002	0.0026	0.7915	No	0.9958	0.751	0.0004	0.341	No	No

102755_at	Itlna	intelectin a	0.971	0.5318	0.1287	0.2085	No	1.0002	0.96	0.0293	0.387	No	No
102758_at	Rbm6	RNA binding motif protein 6	0.8901	0.0528	0.3088	0.966	No	0.887	0.129	0.0101	0.446	No	No
102759_at	Pik3r2	phosphatidylinositol 3-kinase, regulatory subunit, polypeptide 2 (p85 beta)	1.0277	0.4083	0.0139	0.6616	No	1.0579	0.312	0.985	0.0825	No	No
102761_at	Grpel2	GrpE-like 2, mitochondrial	1.0456	0.3287	0.0059	0.0506	No	1.0248	0.579	0.0049	0.0068	No	No
102762_r_at	Rhag	Rhesus blood group-associated A glycoprotein	1.0385	0.2752	0.5004	0.0885	No	0.9687	0.301	0.374	0.611	No	No
102763_at	S3-12	plasma membrane associated protein, S3-12	0.8353	0.0501	0.0174	0.0292	No	0.8576	0.0716	0.234	0.015	No	No
102764_at	Trap1a	tumor rejection antigen P1A	1.0773	0.1937	0.5689	0.1448	No	1.0769	0.129	0.427	0.244	No	No
102765_at	Cops7b	COP9 (constitutive photomorphogenic) homolog, subunit 7b (Arabidopsis thaliana)	1.0011	0.9762	0.9824	0.9618	No	1.0644	0.0636	0.229	0.013	No	No
102766_at	Mycbp	c-myc binding protein	1.0302	0.5458	0.7614	0.3689	No	1.1899	0.0365	0.1	0.686	No	No
102767_at	Gng12	guanine nucleotide binding protein (G protein), gamma 12	0.9759	0.6657	0.0098	0.7552	No	0.9226	0.0311	0.0006	0.324	No	No
102768_i_at	Sc5d	sterol-C5-desaturase (fungal ERG3, delta-5-desaturase) homolog (S. cerevisae)	1.3314	0.1267	0.0222	0.6025	No	1.365	0.0566	0.0101	0.291	No	No
102769_f_at	Sc5d	sterol-C5-desaturase (fungal ERG3, delta-5-desaturase) homolog (S. cerevisae)	1.3145	0.0054	0.004	0.8046	Yes	1.085	0.271	0.583	0.405	No	No
102770_at	Gypa	glycophorin A	1.0309	0.7083	0.5188	0.5787	No	0.9658	0.372	0.0098	0.824	No	No
102771_at	Setdb1	SET domain, bifurcated 1	1.017	0.6985	0.0628	0.096	No	1.0351	0.254	0.0014	0.346	No	No
102772_at	Abl1	v-abl Abelson murine leukemia oncogene 1	1.0035	0.9465	0.1295	0.8634	No	1.0311	0.203	0.0158	0.0626	No	No
102773_at	Car8	carbonic anhydrase 8	1.0672	0.2514	0.0036	0.1926	No	0.8971	0.369	0.0001	0.0233	No	No
102774_at	Egf	epidermal growth factor	1.0714	0.3082	0.0377	0.1383	No	1.0303	0.468	0.139	0.627	No	No
102776_at	Usp38	ubiquitin specific peptidase 38	0.978	0.6055	0.0025	0.4115	No	1.0587	0.082	0.0005	0.773	No	No
102777_at	---	---	0.9741	0.6322	0.8695	0.7486	No	0.949	0.175	0.0049	0.796	No	No
102778_at	Cd79a	CD79A antigen (immunoglobulin-associated alpha)	0.9095	0.2509	0.0093	0.5192	No	0.9974	0.899	0.119	0.0585	No	No
102779_at	Gadd45b	growth arrest and DNA-damage-inducible 45 beta	0.9633	0.2053	0.2353	0.6019	No	1.0267	0.45	0.439	0.342	No	No
102780_at	Srxn1	sulfiredoxin 1 homolog (S. cerevisiae)	1.0012	0.9671	0.0683	0.4195	No	0.9496	0.401	0.0016	0.414	No	No
102781_at	Ccnl2	cyclin L2	1.1	0.0447	0.0005	0.2026	No	0.9919	0.67	0.0071	0.0639	No	No
102782_at	Riok1	RIO kinase 1 (yeast)	0.9218	0.0866	0.1467	0.9049	No	1.0405	0.516	0.0015	0.0057	No	No
102783_at	2310009E04 Rik	RIKEN cDNA 2310009E04 gene	1.034	0.3713	0.1485	0.088	No	1.0041	0.909	0.389	0.138	No	No
102784_at	4632413K17 Rik	RIKEN cDNA 4632413K17 gene	0.9747	0.2965	0.0003	0.1615	No	1.1342	0.0928	0.0049	0.85	No	No
102785_at	Matn1	matrilin 1, cartilage matrix protein 1	1.0578	0.2455	0.0003	0.2451	No	0.975	0.081	0	0.0368	No	No
102786_at	Clcn3	chloride channel 3	0.9597	0.437	0.0002	0.1249	No	0.9576	0.516	0.0001	0.404	No	No
102787_at	Gpr56	G protein-coupled receptor 56	1.0192	0.5352	0.0259	0.1846	No	0.993	0.896	0.89	0.535	No	No
102788_s_at	Pitx2	paired-like homeodomain transcription factor 2	0.9047	0.1159	0.1297	0.1062	No	1.0115	0.751	0.409	0.0379	No	No
102789_at	Gata2	GATA binding protein 2	0.961	0.1427	0.0036	0.0094	No	1.0597	0.121	0.0018	0.189	No	No
102790_at	Jtb	jumping translocation breakpoint	0.986	0.7017	0.0096	0.8115	No	0.9966	0.968	0.0251	0.787	No	No
102791_at	Psmb8	proteosome (prosome, macropain) subunit, beta type 8 (large multifunctional peptidase 7)	0.9478	0.4812	0.2864	0.2572	No	0.8277	0.0566	0.131	0.0584	No	No
102792_at	Ung	uracil DNA glycosylase	1.0941	0.1125	0.0168	0.0573	No	1.0073	0.662	0.0008	0.169	No	No
102793_at	Krtap8-1	keratin associated protein 8-1	1.0356	0.5638	0.2535	0.7747	No	1.1194	0.21	0.0134	0.146	No	No
102794_at	Cxcr4	chemokine (C-X-C motif) receptor 4	0.9561	0.4876	0.0584	0.9002	No	1.0286	0.526	0.134	0.452	No	No
102795_at	Mesp1	mesoderm posterior 1	0.9527	0.1276	0.1199	0.4222	No	0.9695	0.311	0.0566	0.424	No	No
102796_at	Npm3 /// Npm3-ps1 /// LOC639633	nucleoplasmin 3 /// nucleoplasmin 3, pseudogene 1 /// similar to Nucleoplasmin-3	1.0443	0.139	0.0282	0.0468	No	1.0249	0.464	0.915	0.223	No	No
102797_at	Dhrs3	dehydrogenase/reductase (SDR family) member 3	0.8786	0.1183	0.0243	0.6114	No	0.9313	0.183	0.0358	0.371	No	No
102798_at	Adm	adrenomedullin	0.8341	0.0107	0.0296	0.303	No	0.9383	0.186	0.0098	0.194	No	No
102799_at	C4bp	complement component 4 binding protein	1.009	0.9289	0.2503	0.8543	No	0.9837	0.944	0.233	0.781	No	No
102800_at	Foxc2	forkhead box C2	0.9516	0.3234	0.1093	0.3093	No	0.8972	0.0364	0.0191	0.149	No	No
102801_at	Bglap-rs1	bone gamma-carboxyglutamate protein, related sequence 1	0.9807	0.6565	0.0581	0.013	No	1.1023	0.0562	0.0096	0.0055	No	No
102802_at	Il18	interleukin 18	0.9256	0.0551	0.0174	0.414	No	0.8896	0.004	0.113	0.705	Yes	No
102803_at	Lig3	ligase III, DNA, ATP-dependent	1.0641	0.1491	0.177	0.8417	No	1.007	0.842	0.297	0.451	No	No
102804_at	Ceacam1 /// Ceacam2	CEA-related cell adhesion molecule 1 /// CEA-related cell adhesion molecule 2	1.038	0.51	0.0831	0.3575	No	1.0542	0.268	0.0247	0.226	No	No
102805_at	Ceacam1	CEA-related cell adhesion molecule 1	0.9045	0.1733	0.0597	0.6043	No	1.024	0.708	0.0032	0.0347	No	No
102806_g_at	Ceacam1	CEA-related cell adhesion molecule 1	0.8121	0.0484	0.0329	0.1419	No	0.8125	0.0431	0.0221	0.27	No	No
102807_at	Prune	prune homolog (Drosophila)	0.9204	0.0077	0.0015	0.8753	Yes	0.9651	0.133	0.0002	0.689	No	No
102808_at	Scn1b	sodium channel, voltage-gated, type I, beta	0.9837	0.7462	0.1214	0.2679	No	0.9134	0.411	0.524	0.208	No	No
102809_s_at	Lck	lymphocyte protein tyrosine kinase	1.1033	0.0309	0.3775	0.5687	No	1.0988	0.154	0.0863	0.497	No	No
102811_at	Ext1	exostoses (multiple) 1	1.0341	0.6651	0.4458	0.4039	No	1.0449	0.322	0.279	0.707	No	No
102812_i_at	Ube1dc1	ubiquitin-activating enzyme E1-domain containing 1	1.0765	0.4268	0.6102	0.2752	No	1.0134	0.982	0.875	0.315	No	No
102813_f_at	Ube1dc1	ubiquitin-activating enzyme E1-domain containing 1	1.0747	0.0943	0.0889	0.0073	No	1.1122	0.0351	0.0025	0.626	No	No
102814_f_at	Defcr-rs1	defensin related sequence cryptdin peptide (paneth cells)	1.0345	0.2953	0.4222	0.1944	No	1.0202	0.434	0.112	0.487	No	No
102815_at	Anxa11	annexin A11	0.831	0.0714	0.0119	0.4959	No	0.8244	0.0681	0.0111	0.673	No	No
102816_at	Serpina3m	serine (or cysteine) peptidase inhibitor, clade A, member 3M	1.0074	0.8393	0.921	0.7809	No	1.2088	0.178	0.842	0.271	No	No
102817_at	U2af1-rs1	U2 small nuclear ribonucleoprotein auxiliary factor (U2AF) 1, related sequence 1	0.8176	0.0097	0.5472	0.367	Yes	0.862	0.0171	0.0827	0.286	No	Yes
102818_at	Xmr /// LOC546272 /// MGC118208 /// LOC619991 /// LOC640712 /// LOC664829 /// LOC664861 /// LOC664877 /// LOC664890 /// LOC664906 /// LOC664923 /// LOC664944 /// LOC664958 /// LOC664989 ///	Xlr-related, meiosis regulated /// Xmr protein /// similar to Xlr-related, meiosis regulated /// similar to XMR /// similar to Xlr-related, meiosis regulated /// similar to Xlr-related, meiosis regulated /// similar to Xlr-related, meiosis regulated /// similar to Xlr-related, meiosis regulated /// similar to Xlr-related, meiosis regulated /// similar to Xlr-related, meiosis regulated /// similar to Xlr-related, meiosis regulated /// similar to Xlr-related, meiosis regulated /// similar to Xlr-related, meiosis regulated /// similar to Xlr-related, meiosis regulated /// similar to Xlr-related, meiosis regulated /// similar to Xlr-related, meiosis regulated	0.7862	0.006	0.0001	0.0334	Yes	0.8219	0.695	0.0111	0.178	No	No
102819_at	Nap1l2	nucleosome assembly protein 1-like 2	1.1613	0.0087	0.0005	0.0172	Yes	1.0883	0.0256	0.0013	0.0067	No	No
102820_at	Cyp2b13	cytochrome P450, family 2, subfamily b, polypeptide 13	0.9552	0.2944	0.4681	0.2425	No	0.9718	0.818	0.0258	0.497	No	No
102821_s_at	Ran /// Rasl2-9 /// LOC625094 /// LOC671251	RAN, member RAS oncogene family /// RAS-like, family 2, locus 9 /// similar to RAN, member RAS oncogene family /// similar to RAN, member RAS oncogene family	1.1776	0.0214	0.0769	0.6483	No	1.1313	0.0198	0.0095	0.568	No	No
102822_at	Gzmf	granzyme F	0.9024	0.2008	0.2891	0.9792	No	0.8726	0.186	0.0505	0.909	No	No
102823_at	Ighg	Immunoglobulin heavy chain (gamma polypeptide)	1.1025	0.0169	0.0001	0.7435	No	1.0247	0.37	0.0001	0.774	No	No
102824_g_at	Ighg	Immunoglobulin heavy chain (gamma polypeptide)	0.9725	0.7141	0.1005	0.1067	No	0.9561	0.635	0.891	0.602	No	No
102825_at	Ighg	Immunoglobulin heavy chain (gamma polypeptide)	1.0411	0.6676	0.5145	0.2558	No	0.9859	0.699	0.0484	0.789	No	No

102826_at	Akr1b7	aldo-keto reductase family 1, member B7	0.9692	0.5992	0.0124	0.4023	No	0.7164	0.0388	0.0006	0.0366	No	No
102827_at	Nek7	NIMA (never in mitosis gene a)-related expressed kinase 7	0.8596	0.0394	0.9693	0.4336	No	0.8582	0.0296	0.844	0.603	No	No
102828_at	Map2k6	mitogen activated protein kinase kinase 6	0.9814	0.5962	0.0104	0.4246	No	0.954	0.0811	0.164	0.066	No	No
102829_s_at	Map2k6	mitogen activated protein kinase kinase 6	1.1234	0.0946	0.0719	0.0787	No	1.0723	0.099	0.0532	0.0776	No	No
102830_at	Cd86	CD86 antigen	0.9881	0.6969	0.8743	0.7743	No	1.0658	0.118	0.0028	0.02	No	No
102831_s_at	Cd86	CD86 antigen	0.8918	0.1239	0.0553	0.2591	No	0.9788	0.845	0.065	0.778	No	No
102832_at	Mapkapk5 /// Adam1a	MAP kinase-activated protein kinase 5 /// a disintegrin and metallopeptidase domain 1a	1.0247	0.7334	0.4709	0.4764	No	0.9963	0.901	0.0003	0.113	No	No
102833_at	Cbx2	chromobox homolog 2 (Drosophila Pc class)	0.9817	0.6054	0.0996	0.2559	No	0.9688	0.683	0.728	0.478	No	No
102834_at	LOC213320 /// LOC666308 /// LOC666316 /// LOC670749 /// LOC673669	similar to crooked neck protein /// similar to crooked neck protein /// similar to crooked neck protein /// hypothetical protein LOC670749 /// similar to crooked neck protein	0.9203	0.3019	0.1615	0.0638	No	1.0377	0.426	0.307	0.808	No	No
102835_at	Ap2a2	adaptor protein complex AP-2, alpha 2 subunit	0.9343	0.1522	0.7871	0.4314	No	0.969	0.508	0.331	0.128	No	No
102836_at	RP23-136K12.4	putative phosphatase	0.9416	0.2356	0.9318	0.7916	No	0.9841	0.753	0.0002	0.481	No	No
102838_at	Sell	selectin, lymphocyte	1.032	0.6728	0.9285	0.3422	No	1.0075	0.982	0.398	0.244	No	No
102839_at	Plscr1	phospholipid scramblase 1	0.9373	0.0069	0.0031	0.0916	Yes	0.9607	0.466	0.151	0.369	No	No
102840_at	Ptpn12	protein tyrosine phosphatase, non-receptor type 12	1.0209	0.6004	0.0007	0.0177	No	1.0097	0.967	0.0014	0.369	No	No
102841_at	LOC668206	similar to ubiquitin A-52 residue ribosomal protein fusion product 1	1.0228	0.5788	0.0195	0.4573	No	1.0251	0.653	0.787	0.997	No	No
102843_s_at	Igh-1a	immunoglobulin heavy chain 1a (serum IgG2a)	0.9689	0.6622	0.111	0.7705	No	1.0452	0.557	0.134	0.286	No	No
102844_at	Igh-1a	immunoglobulin heavy chain 1a (serum IgG2a)	1.005	0.9023	0.0033	0.8843	No	0.9437	0.609	0.0045	0.6	No	No
102845_at	Pdlim7	PDZ and LIM domain 7	0.9365	0.0885	0.0028	0.029	No	0.9726	0.396	0.03	0.158	No	No
102846_at	Tssk2	testis-specific serine kinase 2	0.9704	0.8083	0.2657	0.4919	No	0.8856	0.134	0.0271	0.364	No	No
102847_s_at	Cyp2a4 /// Cyp2a5 /// LOC330491	cytochrome P450, family 2, subfamily a, polypeptide 4 /// cytochrome P450, family 2, subfamily a, polypeptide 5 /// hypothetical gene supported by X89864; NM_007812	1.0921	0.0684	0.0944	0.1206	No	1.0752	0.226	0.0264	0.485	No	No
102848_f_at	2610524H06 Rik	RIKEN cDNA 2610524H06 gene	1.0169	0.7251	0.0338	0.1757	No	1.0041	0.94	0.116	0.585	No	No
102849_at	Kcnj8	potassium inwardly-rectifying channel, subfamily J, member 8	0.9618	0.1299	0.0661	0.0038	No	1.0011	0.975	0.979	0.29	No	No
102850_at	Tnk2	tyrosine kinase, non-receptor, 2	0.96	0.3827	0.3645	0.5242	No	0.9751	0.446	0.0967	0.464	No	No
102851_s_at	Ptpn6	protein tyrosine phosphatase, non-receptor type 6	0.8886	0.0996	0.1228	0.2287	No	0.9215	0.266	0.309	0.283	No	No
102852_at	Cdh2	cadherin 2	1.1191	0.1404	0.0001	0.018	No	0.965	0.575	0.0003	0.0482	No	No
102853_at	Cspg6	chondroitin sulfate proteoglycan 6	0.9481	0.5831	0.2349	0.2706	No	0.9922	0.903	0.641	0.284	No	No
102854_s_at	Atp7a	ATPase, Cu++ transporting, alpha polypeptide	1.0003	0.9962	0.0009	0.7577	No	0.9983	0.643	0.0001	0.1	No	No
102856_at	Sox10	SRY-box containing gene 10	0.9608	0.3442	0.5092	0.066	No	0.9883	0.752	0.226	0.3	No	No
102857_at	Akap4	A kinase (PRKA) anchor protein 4	1.0541	0.2067	0.1622	0.2854	No	1.0736	0.252	0.897	0.335	No	No
102858_at	Pkib	protein kinase inhibitor beta, cAMP dependent, testis specific	1.0135	0.833	0.5443	0.6641	No	1.0769	0.182	0.0501	0.508	No	No
102859_at	Reep1	receptor accessory protein 1	1.0763	0.0736	0.0002	0.0303	No	1.0282	0.224	0.0002	0.0663	No	No
102860_at	Serpina3g	serine (or cysteine) peptidase inhibitor, clade A, member 3G	0.9325	0.0916	0.1243	0.8398	No	0.9398	0.227	0.26	0.747	No	No
102861_at	Slc22a18	solute carrier family 22 (organic cation transporter), member 18	1.0376	0.1652	0.0634	0.364	No	1.0209	0.763	0.0328	0.722	No	No
102862_at	---	---	0.9775	0.6633	0.1031	0.7159	No	0.9662	0.576	0.0169	0.434	No	No
102863_at	Zkscan1	zinc finger with KRAB and SCAN domains 1	1.0001	0.9989	0.0008	0.9739	No	1.0127	0.519	0.0003	0.238	No	No
102864_at	Hoxa7	homeo box A7	0.9117	0.2927	0.6047	0.9237	No	0.9662	0.492	0.951	0.729	No	No
102865_at	Smad5	MAD homolog 5 (Drosophila)	1.0499	0.415	0.6197	0.9727	No	1.1185	0.112	0.949	0.148	No	No
102866_at	---	---	0.9969	0.9415	0.0105	0.3942	No	0.9962	0.888	0.01	0.309	No	No
102867_at	Tead4	TEA domain family member 4	1.0861	0.0704	0.0335	0.1682	No	1.0451	0.284	0.0727	0.203	No	No
102868_g_at	Tead4	TEA domain family member 4	0.9949	0.9269	0.2113	0.0543	No	0.9379	0.291	0.0306	0.0561	No	No
102869_at	Efna2	ephrin A2	1.0363	0.2933	0.0135	0.0996	No	1.0698	0.184	0.0126	0.478	No	No
102870_at	Gpr178	G protein-coupled receptor 178	1.1103	0.4354	0.0004	0.7298	No	1.144	0.54	0.0006	0.641	No	No
102871_at	Ephb6	Eph receptor B6	1.0039	0.9077	0.1965	0.8153	No	1.0213	0.57	0.763	0.0958	No	No
102872_f_at	Zfp51	zinc finger protein 51	1.0013	0.9864	0.0766	0.4235	No	0.9037	0.434	0.0804	0.829	No	No
102873_at	Tap2	transporter 2, ATP-binding cassette, sub-family B (MDR/TAP)	0.9011	0.1935	0.0658	0.3163	No	0.9538	0.409	0.0065	0.387	No	No
102874_at	Ptpn11	protein tyrosine phosphatase, non-receptor type 11	0.9445	0.0156	0.0025	0.0685	No	1.0512	0.297	0.0021	0.609	No	No
102875_at	Rps6kc1	ribosomal protein S6 kinase polypeptide 1	0.9136	0.0069	0.0808	0.004	No	1.0344	0.422	0.0398	0.121	No	No
102876_at	Gzmg	granzyme G	0.9643	0.6703	0.544	0.6231	No	1.0506	0.637	0.982	0.77	No	No
102877_at	Gzmb	granzyme B	0.9597	0.2837	0.919	0.2128	No	0.9939	0.884	0.0114	0.882	No	No
102878_at	Rad52	RAD52 homolog (S. cerevisiae)	0.9205	0.0314	0.0008	0.2444	No	0.8997	0.117	0.005	0.348	No	No
102879_s_at	Fcgr1	Fc receptor, IgG, high affinity I	1.0078	0.8578	0.2024	0.3296	No	1.0392	0.699	0.427	0.246	No	No
102880_at	E130103I17 Rik	RIKEN cDNA E130103I17 gene	0.8798	0.0115	0.4219	0.0365	No	0.9457	0.193	0.106	0.317	No	No
102881_at	E130103I17 Rik	RIKEN cDNA E130103I17 gene	0.9376	0.1232	0.0112	0.0747	No	0.9746	0.623	0.0123	0.0318	No	No
102882_at	Zfp46	zinc finger protein 46	0.9458	0.4772	0.3602	0.834	No	0.8499	0.676	0.847	0.901	No	No
102883_at	Rpap1	RNA polymerase II associated protein 1	0.9334	0.1455	0.1966	0.9533	No	0.9913	0.909	0.0208	0.872	No	No
102884_at	Inpp5d	inositol polyphosphate-5-phosphatase D	0.941	0.4213	0.1611	0.9916	No	0.9491	0.449	0.11	0.926	No	No
102885_at	Sybl1	synaptobrevin like 1	0.9923	0.9323	0.3133	0.1681	No	1.0487	0.516	0.499	0.0336	No	No
102886_at	Gpc4	glypican 4	0.9559	0.367	0.0804	0.4543	No	0.9387	0.196	0.95	0.123	No	No
102887_at	Tnfrsf11b	tumor necrosis factor receptor superfamily, member 11b (osteoprotegerin)	1.0487	0.0554	0.8248	0.3158	No	1.0687	0.484	0.909	0.35	No	No
102888_s_at	Limk1	LIM-domain containing, protein kinase	0.9511	0.2183	0.0394	0.567	No	0.9687	0.497	0.0088	0.735	No	No
102889_r_at	Limk1	LIM-domain containing, protein kinase	0.9509	0.1929	0.027	0.8097	No	0.9938	0.913	0.046	0.0461	No	No
102890_at	Snta1	syntrophin, acidic 1	0.8299	0.0025	0.5226	0.0019	No	0.8892	0.0198	0.108	0.0053	No	No
102891_at	Wrn	Werner syndrome homolog (human)	0.9773	0.3745	0.8541	0.0692	No	1.0968	0.0891	0.0039	0.234	No	No
102892_at	Kcnab2	potassium voltage-gated channel, shaker-related subfamily, beta member 2	1.0893	0.0349	0.2151	0.05	No	0.9063	0.0144	0.0001	0.164	No	No
102893_at	Pou2f1	POU domain, class 2, transcription factor 1	1.0526	0.3274	0.1199	0.4919	No	1.1372	0.0279	0.0162	0.0884	No	No
102894_g_at	Pou2f1	POU domain, class 2, transcription factor 1	1.0772	0.1056	0.0081	0.2136	No	0.9703	0.458	0.212	0.923	No	No
102895_at	Nipbl	Nipped-B homolog (Drosophila)	0.9011	0.3668	0.0136	0.6312	No	0.9858	0.874	0.0082	0.694	No	No
102896_at	Dok1	docking protein 1	0.7943	0.0011	0.0001	0.0194	Yes	0.8822	0.0006	0	0.0102	Yes	Yes
102898_at	Hgf	hepatocyte growth factor	1.055	0.1705	0.4203	0.7471	No	1.496	0.0296	0.997	0.348	No	No
102899_at	St6galnac3	ST6 (alpha-N-acetyl-neuraminyl-2,3-beta-galactosyl-1,3)-N-acetylgalactosaminide alpha-2,6-sialyltransferase 3	0.9752	0.7755	0.3499	0.7178	No	1.1124	0.338	0.902	0.339	No	No
102900_at	Six3 /// E130112M24Rik	sine oculis-related homeobox 3 homolog (Drosophila) /// RIKEN cDNA E130112M24 gene	0.9418	0.4499	0.5538	0.2084	No	0.9523	0.602	0.0746	0.563	No	No
102901_at	Six3	sine oculis-related homeobox 3 homolog (Drosophila)	1.0536	0.4093	0.0414	0.7342	No	1.0379	0.328	0.0178	0.0874	No	No
102902_at	Lhx3	LIM homeobox protein 3	1.0224	0.6522	0.3247	0.0979	No	1.0039	0.878	0.521	0.308	No	No
102904_at	H2-Ea	histocompatibility 2, class II antigen E alpha	0.9979	0.9693	0.0659	0.0936	No	0.916	0.0804	0.0996	0.135	No	No
102905_at	Casp4	caspase 4, apoptosis-related cysteine peptidase	0.9991	0.989	0.4111	0.7635	No	1.1261	0.318	0.118	0.161	No	No
102906_at	Tgtp	T-cell specific GTPase	0.9167	0.0562	0.1266	0.1099	No	0.9389	0.148	0.0206	0.92	No	No
102907_at	Phldb2	pleckstrin homology-like domain, family B, member 2	1.0036	0.958	0.0068	0.5989	No	1.0334	0.398	0.004	0.168	No	No
102908_at	Epb4.2	erythrocyte protein band 4.2	1.0746	0.0727	0.8815	0.3489	No	1.3533	0.0399	0.179	0.867	No	No

102910_at	Abcb1a	ATP-binding cassette, sub-family B (MDR/TAP), member 1A	0.9655	0.6112	0.0754	0.659	No	0.9553	0.609	0.0072	0.96	No	No
102911_at	Brca2	breast cancer 2	1.0827	0.1726	0.0124	0.119	No	1.0629	0.0904	0.0013	0.0783	No	No
102912_at	Tnks2	tankyrase, TRF1-interacting ankyrin-related ADP-ribose polymerase 2	1.0467	0.5882	0.0012	0.3577	No	1.0094	0.82	0.0077	0.596	No	No
102913_at	Bcl2a1a /// Bcl2a1b /// Bcl2a1d	B-cell leukemia/lymphoma 2 related protein A1a /// B-cell leukemia/lymphoma 2 related protein A1b /// B-cell leukemia/lymphoma 2 related protein A1d	0.9383	0.3694	0.7826	0.2307	No	1.1294	0.0167	0.0001	0.0372	No	No
102914_s_at	Bcl2a1a /// Bcl2a1b /// Bcl2a1c /// Bcl2a1d	B-cell leukemia/lymphoma 2 related protein A1a /// B-cell leukemia/lymphoma 2 related protein A1b /// B-cell leukemia/lymphoma 2 related protein A1c /// B-cell leukemia/lymphoma 2 related protein A1d	1.0351	0.1772	0.0034	0.0128	No	1.0176	0.727	0.0671	0.408	No	No
102915_at	F2rl1	coagulation factor II (thrombin) receptor-like 1	1.0345	0.435	0.5335	0.2443	No	0.9694	0.657	0.341	0.873	No	No
102916_s_at	Tnxb	tenascin XB	0.8629	0.0374	0.0762	0.4963	No	0.9346	0.177	0.0347	0.795	No	No
102917_at	C2ta	class II transactivator	0.9841	0.6166	0.8959	0.3678	No	1.0419	0.408	0.0023	0.418	No	No
102918_at	Muc1	mucin 1, transmembrane	0.9583	0.4852	0.1101	0.29	No	0.8995	0.191	0.216	0.398	No	No
102919_at	Mafk	v-maf musculoaponeurotic fibrosarcoma oncogene family, protein K (avian)	0.9381	0.1974	0.1191	0.2079	No	1.1068	0.0814	0.168	0.0419	No	No
102920_at	9130422G05 Rik	RIKEN cDNA 9130422G05 gene	1.0461	0.2838	0.0001	0.0552	No	1.0642	0.327	0.001	0.901	No	No
102921_s_at	Fas	Fas (TNF receptor superfamily member)	0.9617	0.5173	0.0136	0.2697	No	0.804	0.0422	0.128	0.599	No	No
102922_at	Pitpnc1	phosphatidylinositol transfer protein, cytoplasmic 1	0.9516	0.3953	0.001	0.7917	No	0.9591	0.571	0.922	0.339	No	No
102923_at	Pnoc	prepronociceptin	0.8309	0.0526	0.3463	0.203	No	1.018	0.719	0.0034	0.938	No	No
102924_at	Dtx1	deltex 1 homolog (Drosophila)	1.0897	0.0355	0.0026	0.6865	No	1.0699	0.0122	0.085	0.111	No	No
102925_at	Dusp9	dual specificity phosphatase 9	0.9732	0.7102	0.1477	0.4295	No	0.9529	0.347	0.002	0.7	No	No
102926_at	Gfra3	glial cell line derived neurotrophic factor family receptor alpha 3	0.9605	0.3144	0.0121	0.3694	No	0.9027	0.187	0.636	0.299	No	No
102927_s_at	Hdh	Huntington disease gene homolog	0.9594	0.1089	0.5082	0.8863	No	0.9481	0.175	0.168	0.179	No	No
102928_at	Hdh	Huntington disease gene homolog	1.0129	0.7287	0.2516	0.9356	No	0.9421	0.0291	0.0303	0.918	No	No
102929_s_at	Flt3l	FMS-like tyrosine kinase 3 ligand	0.969	0.4602	0.0288	0.3487	No	0.9973	0.987	0.0046	0.859	No	No
102930_at	Si	silver	0.9742	0.386	0.9038	0.2245	No	1.0417	0.158	0.112	0.0372	No	No
102931_at	Wap	whey acidic protein	0.9424	0.5483	0.3278	0.4439	No	0.9099	0.158	0.542	0.386	No	No
102932_at	Nr5a2	nuclear receptor subfamily 5, group A, member 2	1.0492	0.3744	0.6656	0.0549	No	0.7952	0.0097	0.0012	0.892	Yes	No
102933_at	Plxna3	plexin A3	0.9899	0.7922	0.1227	0.3119	No	0.9592	0.256	0.134	0.49	No	No
102934_s_at	Cdc25c	cell division cycle 25 homolog C (S. cerevisiae)	0.9402	0.2227	0.6108	0.3439	No	1.0413	0.476	0.0256	0.789	No	No
102935_at	Cdc25c	cell division cycle 25 homolog C (S. cerevisiae)	0.8679	0.0351	0.5756	0.0385	No	0.885	0.0873	0.246	0.222	No	No
102936_at	LOC675709	similar to Beta-1,4-galactosyltransferase 6 (Beta-1,4-GalTase 6) (Beta4Gal-T6) (b4Gal-T6) (UDP-galactose:beta-N-acetylglucosamine beta-1,4-galactosyltransferase 6) (UDP-Gal:beta-GlcNAc beta-1,4-galactosyltransferase 6)	1.0926	0.2947	0.177	0.7608	No	1.0984	0.0033	0.0019	0.0008	No	No
102937_at	LOC546257 /// LOC619649	similar to transcription elongation factor B polypeptide 3 binding protein 1 isoform 1 /// similar to transcription elongation factor B polypeptide 3 binding protein 1 isoform 1	1.0273	0.5953	0.7306	0.7819	No	0.9524	0.375	0.0552	0.512	No	No
102938_at	Lect2	leukocyte cell-derived chemotaxin 2	0.971	0.5228	0.0205	0.9206	No	0.9968	0.971	0.0277	0.64	No	No
102939_s_at	Cd22	CD22 antigen	1.0809	0.0831	0.1977	0.0481	No	1.0951	0.105	0.0636	0.278	No	No
102940_at	Ltb	lymphotoxin B	1.0168	0.7687	0.0115	0.5828	No	0.956	0.208	0.0025	0.0304	No	No
102941_at	Ugcgl1	UDP-glucose ceramide glucosyltransferase-like 1	0.9535	0.3771	0.4065	0.9365	No	0.7914	0.0571	0.0677	0.958	No	No
102942_at	Specc1	spectrin domain with coiled-coils 1	1.076	0.2063	0.1104	0.0923	No	0.9934	0.976	0.0471	0.0299	No	No
102943_at	Atg7	autophagy-related 7 (yeast)	1.0665	0.1295	0.0929	0.5198	No	1.0536	0.299	0.185	0.775	No	No
102944_at	RP23-336J1.4	Ras-GTPase-activating protein SH3-domain binding protein	0.952	0.1859	0.0014	0.46	No	0.9667	0.276	0.0005	0.839	No	No
102946_r_at	---	---	1.0102	0.8158	0.8286	0.4137	No	1.0311	0.117	0.0011	0.19	No	No
102947_at	Slc22a2	solute carrier family 22 (organic cation transporter), member 2	0.9978	0.9517	0.0702	0.1285	No	0.9891	0.922	0.0022	0.0761	No	No
102948_at	LOC625599	similar to Glycosyl-phosphatidylinositol-anchored molecule-like protein precursor	0.9618	0.2233	0.003	0.0724	No	0.937	0.382	0.165	0.407	No	No
102949_g_at	Hemt1	hematopoietic cell transcript 1	0.9576	0.5812	0.0137	0.5489	No	0.7896	0.0448	0.0082	0.808	No	No
102950_at	Hemt1	hematopoietic cell transcript 1	1.0337	0.6435	0.0042	0.8245	No	1.1497	0.298	0.0029	0.996	No	No
102951_at	Cradd	CASP2 and RIPK1 domain containing adaptor with death domain	0.9612	0.0056	0.4029	0.0262	Yes	0.9488	0.146	0.215	0.832	No	No
102952_g_at	Cradd	CASP2 and RIPK1 domain containing adaptor with death domain	0.9872	0.56	0.0007	0.2234	No	0.9987	0.953	0.0055	0.825	No	No
102953_at	Msln	mesothelin	1.0397	0.3369	0.0008	0.049	No	1.059	0.0535	0.0126	0.0038	No	No
102954_at	Sox5	SRY-box containing gene 5	1.028	0.169	0.562	0.0207	No	0.9901	0.943	0.0169	0.0853	No	No
102955_at	Nfil3	nuclear factor, interleukin 3, regulated	0.6846	0.0002	0.0116	0.0088	No	0.6751	0.0012	0.0213	0.0287	Yes	No
102956_at	Msx2	homeo box, msh-like 2	0.9062	0.0036	0	0.0024	No	1.0073	0.899	0	0.358	No	No
102957_at	Lcp2	lymphocyte cytosolic protein 2	0.9698	0.7562	0.105	0.5263	No	0.9757	0.523	0.0125	0.473	No	No
102958_at	Ebf2	early B-cell factor 2	1.0277	0.3604	0.0131	0.5961	No	0.9276	0.114	0.947	0.0172	No	No
102959_at	Tle4	transducin-like enhancer of split 4, homolog of Drosophila E(spl)	0.9882	0.8128	0.0215	0.5869	No	1.0503	0.504	0.256	0.993	No	No
102960_at	Rag1ap1	recombination activating gene 1 activating protein 1	0.9949	0.943	0.1852	0.6943	No	0.9968	0.954	0.0692	0.949	No	No
102961_at	Hrg	histidine-rich glycoprotein	0.9372	0.3964	0.001	0.2195	No	0.9998	0.684	0.0002	0.307	No	No
102962_at	38961	septin 1	0.9937	0.8336	0.0008	0.2642	No	1.0643	0.0339	0.0001	0.275	No	No
102963_at	E2f1	E2F transcription factor 1	1.0348	0.3509	0.7617	0.0271	No	1.1775	0.0619	0.514	0.0302	No	No
102964_at	Zfp639	zinc finger protein 639	1.0056	0.891	0.1159	0.3766	No	1.0265	0.666	0.138	0.433	No	No
102965_at	AI481105	expressed sequence AI481105	0.9202	0.1178	0.0245	0.7166	No	0.8642	0.243	0.0804	0.463	No	No
102966_at	Cnot6l	CCR4-NOT transcription complex, subunit 6-like	0.8858	0.2989	0.0259	0.0517	No	1.001	0.825	0.0001	0.418	No	No
102967_at	Gdap1	ganglioside-induced differentiation-associated-protein 1	0.9868	0.8218	0.0005	0.5367	No	1.0036	0.809	0.0002	0.608	No	No
102968_at	Ggtla1	gamma-glutamyltransferase-like activity 1	1.0334	0.5259	0.0504	0.7963	No	0.9842	0.625	0.0563	0.143	No	No
102969_at	0610025P10 Rik	RIKEN cDNA 0610025P10 gene	0.9656	0.3194	0.7149	0.0732	No	1.0932	0.148	0.0874	0.534	No	No
102970_at	Psmc3ip	proteasome (prosome, macropain) 26S subunit, ATPase 3, interacting protein	1.0417	0.4903	0.4098	0.4871	No	1.0395	0.413	0.878	0.436	No	No
102971_at	Cd3e	CD3 antigen, epsilon polypeptide	0.9616	0.5109	0.8995	0.5173	No	0.9039	0.129	0.152	0.794	No	No
102972_s_at	Dab1	disabled homolog 1 (Drosophila)	0.9661	0.5682	0.1193	0.4695	No	0.9744	0.638	0.628	0.398	No	No
102973_f_at	Mug2	murinoglobulin 2	1.0035	0.9573	0.0931	0.8926	No	1.0571	0.65	0.0199	0.175	No	No
102974_at	Marco	macrophage receptor with collagenous structure	0.9698	0.3159	0.0005	0.5619	No	1.0611	0.335	0.0056	0.0546	No	No
102975_at	Cd8a /// LOC636147 /// LOC669166	CD8 antigen, alpha chain /// similar to T-cell surface glycoprotein CD8 alpha chain precursor (T-cell surface glycoprotein Lyt-2) /// similar to T-cell surface glycoprotein CD8 alpha chain precursor (T-cell surface glycoprotein Lyt-2) (CD8a antigen)	0.9569	0.3436	0.329	0.3873	No	1.0291	0.862	0.427	0.482	No	No
102976_at	Brca1	breast cancer 1	0.9633	0.5764	0.0741	0.4025	No	1.022	0.608	0.0056	0.924	No	No
102977_at	a	nonagouti	1.0057	0.8134	0.1004	0.1621	No	0.9831	0.644	0.117	0.0125	No	No
102978_at	A430104N18 Rik	RIKEN cDNA A430104N18 gene	1.0116	0.7946	0.0069	0.383	No	0.9455	0.152	0.0018	0.421	No	No
102979_at	Shf	Src homology 2 domain containing F	1.0158	0.7964	0.0108	0.3622	No	1.264	0.021	0.0104	0.0682	No	No
102980_at	Nmt1	N-myristoyltransferase 1	1.0065	0.7947	0.3589	0.0098	No	1.0842	0.148	0.832	0.0463	No	No
102981_at	Gabpa	GA repeat binding protein, alpha	1.0864	0.0961	0.0874	0.044	No	1.0064	0.84	0.0006	0.942	No	No
102982_at	B230308N11 Rik	RIKEN cDNA B230308N11 gene	1.0426	0.3744	0.0214	0.0397	No	1.0025	0.811	0.0024	0.103	No	No
102983_at	Smad1	MAD homolog 1 (Drosophila)	1.0132	0.8383	0.1328	0.9637	No	1.086	0.201	0.524	0.897	No	No
102984_g_at	Smad1	MAD homolog 1 (Drosophila)	1.0638	0.2834	0.5112	0.0816	No	1.1043	0.16	0.07	0.0276	No	No
102985_at	Srpk1	serine/arginine-rich protein specific kinase 1	1.0565	0.1023	0.0178	0.5231	No	1.0448	0.125	0.0007	0.554	No	No
102986_at	Myod1	myogenic differentiation 1	0.9565	0.4288	0.1265	0.9937	No	0.9363	0.153	0.0268	0.91	No	No

102987_at	2010109K11 Rik	RIKEN cDNA 2010109K11 gene	1.0206	0.6748	0.2068	0.7523	No	0.9114	0.157	0.0007	0.635	No	No
102988_at	Inppl1	inositol polyphosphate phosphatase-like 1	0.9167	0.0672	0.2642	0.0425	No	0.9545	0.463	0.178	0.533	No	No
102989_at	Mt4	metallothionein 4	1.04	0.3955	0.0009	0.1895	No	1.0034	0.69	0.0002	0.151	No	No
102990_at	Col3a1	procollagen, type III, alpha 1	1.0811	0.4186	0.0259	0.5199	No	1.0265	0.658	0.0904	0.94	No	No
102991_s_at	H2-Ke6	H2-K region expressed gene 6	1.061	0.1629	0.1227	0.2047	No	1.094	0.225	0.231	0.0607	No	No
102992_at	H2-Ke6	H2-K region expressed gene 6	0.9706	0.5424	0.864	0.3305	No	0.9073	0.134	0.141	0.389	No	No
102993_at	Ggta1	glycoprotein galactosyltransferase alpha 1, 3	0.93	0.0129	0.1607	0.0068	No	0.9145	0.0683	0.079	0.0713	No	No
102994_at	Stat4	signal transducer and activator of transcription 4	0.9732	0.5146	0.0061	0.102	No	0.9655	0.566	0.0314	0.685	No	No
102995_s_at	Gzma	granzyme A	0.9671	0.6823	0.0252	0.6716	No	0.8274	0.162	0.0146	0.418	No	No
102996_at	Ell	elongation factor RNA polymerase II	0.9715	0.4444	0.3523	0.5707	No	1.0207	0.585	0.0299	0.341	No	No
102998_at	Cyp1a2	cytochrome P450, family 1, subfamily a, polypeptide 2	0.9944	0.9391	0.0665	0.815	No	1.0626	0.121	0.438	0.209	No	No
103001_at	Vegfb	vascular endothelial growth factor B	0.9672	0.425	0.0038	0.1265	No	1.0231	0.479	0.152	0.0584	No	No
103002_at	B4galt1	UDP-Gal:betaGlcNAc beta 1,4- galactosyltransferase, polypeptide 1	1.0637	0.2552	0.83	0.3443	No	1.0023	0.928	0.0712	0.283	No	No
103003_i_at	Cd44	CD44 antigen	1.0385	0.3567	0.9213	0.4036	No	1.0435	0.994	0.865	0.895	No	No
103004_r_at	Cd44	CD44 antigen	1.017	0.7901	0.0519	0.1076	No	1.1586	0.37	0.0496	0.166	No	No
103005_s_at	Cd44	CD44 antigen	0.8741	0.0995	0.0613	0.7414	No	1.0119	0.742	0.505	0.981	No	No
103006_at	Atf5	activating transcription factor 5	0.9674	0.3342	0.005	0.9507	No	0.9524	0.172	0.0025	0.135	No	No
103007_at	Efna1	ephrin A1	0.9441	0.3723	0.0184	0.8176	No	0.9268	0.0134	0.015	0.114	No	No
103009_at	Hira	histone cell cycle regulation defective homolog A (S. cerevisiae)	0.9947	0.846	0.0085	0.4011	No	1.0623	0.306	0.67	0.0653	No	No
103010_at	2610005L07 Rik	RIKEN cDNA 2610005L07 gene	1.0897	0.185	0.3773	0.585	No	1.1305	0.049	0.0183	0.032	No	No
103011_at	Sin3a	transcriptional regulator, SIN3A (yeast)	0.9989	0.9695	0.0048	0.0314	No	1.0086	0.174	0	0.0032	No	No
103012_at	Ccl21b /// Ccl21a /// Ccl21c	chemokine (C-C motif) ligand 21b /// chemokine (C-C motif) ligand 21a /// chemokine (C-C motif) ligand 21c (leucine)	0.9505	0.2017	0.0008	0.0099	No	0.8986	0.0189	0.0009	0.0048	No	No
103013_at	Usf2	upstream transcription factor 2	1.0175	0.7809	0.0595	0.4723	No	1.0654	0.116	0.0203	0.0842	No	No
103015_at	Bcl6	B-cell leukemia/lymphoma 6	1.0267	0.5738	0.0009	0.0358	No	0.8965	0.266	0.0005	0.326	No	No
103016_s_at	Cd68	CD68 antigen	0.9488	0.4269	0.1317	0.3859	No	0.9743	0.554	0.0131	0.896	No	No
103017_at	LOC664862	similar to transmembrane 7 superfamily member 1	1.0136	0.6694	0.0027	0.1794	No	1.0248	0.594	0.0298	0.392	No	No
103018_at	Vdp	vesicle docking protein	1.1213	0.2108	0.0039	0.3704	No	1.0172	0.776	0.005	0.243	No	No
103020_s_at	Map3k1 /// LOC670493 /// LOC674573	mitogen activated protein kinase kinase kinase 1 /// similar to Mitogen-activated protein kinase kinase kinase 1 (MAPK/ERK kinase kinase 1) (MEK kinase 1) (MEKK 1) /// similar to Mitogen-activated protein kinase kinase kinase 1 (MAPK/ERK kinase kinase 1) (MEK kinase 1) (MEKK 1)	1.1099	0.0841	0.0005	0.6055	No	1.1735	0.0077	0.0028	0.0237	Yes	No
103021_r_at	Map3k1 /// LOC670493 /// LOC674573	mitogen activated protein kinase kinase kinase 1 /// similar to Mitogen-activated protein kinase kinase kinase 1 (MAPK/ERK kinase kinase 1) (MEK kinase 1) (MEKK 1) /// similar to Mitogen-activated protein kinase kinase kinase 1 (MAPK/ERK kinase kinase 1) (MEK kinase 1) (MEKK 1)	1.1855	0.1576	0.0055	0.5772	No	1.2027	0.181	0.0016	0.865	No	No
103022_at	Map3k1 /// LOC670493 /// LOC674573	mitogen activated protein kinase kinase kinase 1 /// similar to Mitogen-activated protein kinase kinase kinase 1 (MAPK/ERK kinase kinase 1) (MEK kinase 1) (MEKK 1) /// similar to Mitogen-activated protein kinase kinase kinase 1 (MAPK/ERK kinase kinase 1) (MEK kinase 1) (MEKK 1)	0.7673	0.0998	0.0122	0.0585	No	0.7943	0.121	0.0004	0.019	No	No
103023_at	Lcat	lecithin cholesterol acyltransferase	1.0259	0.6212	0.9152	0.0168	No	1.0459	0.411	0.398	0.0807	No	No
103024_at	Adam8	a disintegrin and metallopeptidase domain 8	0.9622	0.4975	0.9937	0.4152	No	0.9858	0.512	0.0035	0.17	No	No
103025_at	Mov10	Moloney leukemia virus 10	0.9626	0.525	0.4871	0.4515	No	1.0315	0.585	0.346	0.712	No	No
103026_f_at	Crygd /// Crygf	crystallin, gamma D /// crystallin, gamma F	0.9987	0.9564	0.0051	0.5582	No	1.0747	0.0024	0.0005	0.214	Yes	No
103027_at	1810030O07 Rik	RIKEN cDNA 1810030O07 gene	1.0471	0.3469	0.9577	0.3454	No	1.0694	0.239	0.275	0.211	No	No
103028_at	Itk	IL2-inducible T-cell kinase	1.1284	0.087	0.7759	0.6912	No	1.0337	0.713	0.106	0.125	No	No
103029_at	Pdcd4 /// LOC670861	programmed cell death 4 /// similar to programmed cell death 4	0.8743	0.3939	0.5062	0.8638	No	0.9082	0.101	0.0047	0.203	No	No
103030_at	Dnm1	dynamin 1	1.0046	0.9041	0.2483	0.0539	No	0.94	0.238	0.62	0.773	No	No
103031_g_at	Dnm1	dynamin 1	1.0678	0.149	0.0039	0.2244	No	1.0048	0.375	0.0001	0.0097	No	No
103032_at	Tpst1	protein-tyrosine sulfotransferase 1	1.0544	0.1296	0.0106	0.1871	No	1.0133	0.724	0.208	0.206	No	No
103033_at	C4b /// C4a /// LOC672587 /// LOC675521	complement component 4B (Childo blood group) /// complement component 4A (Rodgers blood group) /// similar to sex-limited protein /// similar to Complement C4 precursor	0.871	0.012	0.0292	0.0502	No	0.9173	0.0261	0.0326	0.0393	No	No
103034_at	Ccne1	cyclin E1	1.0169	0.5756	0.0727	0.3359	No	1.03	0.158	0.013	0.0922	No	No
103035_at	Tap1	transporter 1, ATP-binding cassette, sub-family B (MDR/TAP)	0.9785	0.4703	0.9225	0.0744	No	1.0373	0.443	0.502	0.808	No	No
103036_at	Xrcc6	X-ray repair complementing defective repair in Chinese hamster cells 6	0.8959	0.0037	0.2788	0.0515	Yes	0.9651	0.276	0.164	0.0289	No	No
103037_at	Ctf1	cardiotrophin 1	0.9662	0.3499	0.031	0.2413	No	0.9443	0.132	0.0185	0.349	No	No
103038_at	Guca1a	guanylate cyclase activator 1a (retina)	0.9566	0.2749	0.0127	0.2408	No	0.9655	0.519	0.0929	0.123	No	No
103039_at	Itga5	integrin alpha 5 (fibronectin receptor alpha)	0.9163	0.2144	0.0041	0.581	No	0.8344	0.0439	0.0103	0.0238	No	No
103040_at	Cd83	CD83 antigen	1.3013	0.0118	0.0214	0.5179	No	1.2078	0.0035	0.0079	0.398	Yes	Yes
103041_at	Efna4	ephrin A4	0.9961	0.9418	0.2035	0.4684	No	0.978	0.703	0.0513	0.586	No	No
103043_at	Mtcp1	mature T-cell proliferation 1	1.0027	0.9619	0.2777	0.1866	No	0.9358	0.0882	0.0164	0.348	No	No
103044_g_at	Mtcp1	mature T-cell proliferation 1	0.9863	0.6471	0.0481	0.3195	No	0.9667	0.176	0.0696	0.0355	No	No
103045_at	Mtcp1	mature T-cell proliferation 1	1.0489	0.1683	0.6195	0.0407	No	1.0575	0.126	0.267	0.0015	No	No
103046_at	Car4	carbonic anhydrase 4	0.973	0.4493	0.391	0.5306	No	0.9591	0.768	0.105	0.15	No	No
103047_at	Pxmp3	peroxisomal membrane protein 3	1.0378	0.6923	0.959	0.4691	No	1.0323	0.638	0.83	0.157	No	No
103048_at	Mycn	v-myc myelocytomatosis viral related oncogene, neuroblastoma derived (avian)	1.118	0.0644	0.0036	0.5827	No	1.1104	0.0031	0.222	0.0064	No	No
103049_at	---	---	1.0284	0.5768	0.96	0.821	No	0.9972	0.954	0.109	0.48	No	No
103050_at	Tcf21	transcription factor 21	0.982	0.4822	0.8527	0.5667	No	0.9811	0.729	0.832	0.654	No	No
103051_at	Expi	extracellular proteinase inhibitor	0.9523	0.1868	0.0171	0.315	No	0.9007	0.074	0.025	0.553	No	No
103052_r_at	Nr2f2	nuclear receptor subfamily 2, group F, member 2	0.9827	0.8841	0.004	0.7291	No	0.934	0.37	0.0003	0.824	No	No
103053_at	Myog	myogenin	0.8865	0.1239	0.3141	0.5004	No	0.9124	0.0977	0.0354	0.295	No	No
103054_at	Polr2a	polymerase (RNA) II (DNA directed) polypeptide A	0.9454	0.156	0.0008	0.1348	No	1.0247	0.362	0.0107	0.267	No	No
103055_r_at	Polr2a	polymerase (RNA) II (DNA directed) polypeptide A	1.0313	0.4803	0.4983	0.0884	No	1.0218	0.555	0.152	0.114	No	No
103056_at	Utp18	UTP18, small subunit (SSU) processome component, homolog (yeast)	1.0902	0.1088	0.1374	0.2548	No	1.119	0.144	0.242	0.37	No	No
103057_at	Pold1	polymerase (DNA directed), delta 1, catalytic subunit	1.0006	0.9792	0.0003	0.1714	No	1.0614	0.718	0.0041	0.0281	No	No
103058_f_at	Tcp10a	t-complex protein 10a	0.8027	0.0713	0.1256	0.2496	No	0.8304	0.0321	0.0217	0.0465	No	No
103059_at	Fxyd3	FXYD domain-containing ion transport regulator 3	0.9089	0.3296	0.1825	0.1952	No	0.9128	0.125	0.0033	0.0247	No	No
103060_at	Lgi4	leucine-rich repeat LGI family, member 4	1.0303	0.3934	0.0206	0.5777	No	0.994	0.859	0.0732	0.266	No	No
103061_at	Gad1	glutamic acid decarboxylase 1	1.3452	0.0006	0.001	0.0531	Yes	1.251	0.0002	0.0003	0.0013	No	No
103062_at	Rab33b	RAB33B, member of RAS oncogene family	1.2034	0.0193	0.0013	0.0293	No	1.097	0.0602	0.0012	0.0199	No	No
103063_at	Zfp62	zinc finger protein 62	1.0269	0.7482	0.0069	0.8206	No	0.9571	0.806	0.0537	0.732	No	No
103064_at	Chek1	checkpoint kinase 1 homolog (S. pombe)	1.0011	0.9892	0.0576	0.9221	No	1.0254	0.298	0.0332	0.0129	No	No
103065_at	Slc20a1	solute carrier family 20, member 1	1.162	0.0044	0.0017	0.0232	Yes	0.9393	0.152	0.0032	0.104	No	No
103066_at	Tyki	thymidylate kinase family LPS-inducible member	1.0374	0.4414	0.0426	0.3732	No	0.994	0.978	0.437	0.282	No	No
103067_at	Rala	v-ral simian leukemia viral oncogene homolog A (ras related)	1.1389	0.0217	0.1361	0.3304	No	1.1285	0.0414	0.489	0.865	No	No
103068_at	Akr1e1	aldo-keto reductase family 1, member E1	0.9362	0.3946	0.0133	0.9527	No	0.9242	0.138	0.0093	0.581	No	No
103069_at	Lin9	lin-9 homolog (C. elegans)	0.8902	0.1171	0.1877	0.2551	No	0.9605	0.61	0.528	0.769	No	No

103070_at	Sirpa	signal-regulatory protein alpha	1.0636	0.1678	0.7171	0.0434	No	0.9907	0.909	0.0394	0.109	No	No
103071_at	Topbp1	topoisomerase (DNA) II beta binding protein	1.0977	0.1982	0.0028	0.467	No	1.1152	0.14	0.0328	0.414	No	No
103072_at	Hsd3b1	hydroxysteroid dehydrogenase-1, delta<5>-3-beta	1.0038	0.9669	0.0889	0.8799	No	0.9897	1	0.192	0.437	No	No
103073_i_at	Taf9 /// LOC545600 /// LOC671012	TAF9 RNA polymerase II, TATA box binding protein (TBP)-associated factor /// similar to TAF9 RNA polymerase II, TATA box binding protein (TBP)-associated factor isoform 1 /// similar to TAF9 RNA polymerase II, TATA box binding protein (TBP)-associated factor isoform 1	1.0255	0.2788	0.0003	0.1277	No	1.002	0.886	0.0019	0.0453	No	No
103074_f_at	Taf9 /// LOC545600 /// LOC671012	TAF9 RNA polymerase II, TATA box binding protein (TBP)-associated factor /// similar to TAF9 RNA polymerase II, TATA box binding protein (TBP)-associated factor isoform 1 /// similar to TAF9 RNA polymerase II, TATA box binding protein (TBP)-associated factor isoform 1	1.2241	0.1261	0.0503	0.7126	No	1.1744	0.014	0.0009	0.134	No	No
103075_at	Pou5f1	POU domain, class 5, transcription factor 1	0.98	0.6309	0.0359	0.9609	No	0.943	0.476	0.103	0.919	No	No
103076_at	Chmp5	chromatin modifying protein 5	0.9461	0.5473	0.0711	0.5832	No	1.0127	0.894	0.342	0.904	No	No
103078_at	Tmem150	transmembrane protein 150	1.0535	0.4382	0.5304	0.3595	No	1.005	0.756	0.0321	0.0593	No	No
103079_at	Arid2	AT rich interactive domain 2 (Arid-rfx like)	1.062	0.278	0.0072	0.0284	No	0.9701	0.326	0.14	0.349	No	No
103080_at	Samhd1	SAM domain and HD domain, 1	0.8593	0.1005	0.5651	0.1601	No	0.8837	0.117	0.675	0.168	No	No
103081_at	Baz1b	bromodomain adjacent to zinc finger domain, 1B	0.9248	0.1174	0.0211	0.2573	No	0.9344	0.216	0.0475	0.228	No	No
103082_at	E230022H04 Rik	RIKEN cDNA E230022H04 gene	0.8934	0.0436	0.0001	0.8069	No	0.9137	0.201	0.0012	0.799	No	No
103083_at	Lipe	lipase, hormone sensitive	1.025	0.6036	0.3439	0.8606	No	1.0561	0.375	0.225	0.241	No	No
103084_at	Csrp3	cysteine and glycine-rich protein 3	0.7932	0.0074	0.0207	0.0092	No	0.8305	0	0.0003	0	No	No
103085_at	Hebp1	heme binding protein 1	0.9776	0.4619	0.0009	0.1523	No	0.9834	0.955	0.0014	0.0039	No	No
103086_at	Hoxa5	homeo box A5	1.0049	0.9327	0.0135	0.0961	No	1.0611	0.378	0.0152	0.302	No	No
103087_at	Sult1a1	sulfotransferase family 1A, phenol-preferring, member 1	0.6426	0	0.8694	0.0446	Yes	0.6334	0.0001	0.0724	0.0473	Yes	Yes
103088_at	Chl1	cell adhesion molecule with homology to L1CAM	0.9586	0.7164	0.3953	0.4443	No	0.9306	0.47	0.108	0.366	No	No
103089_at	Cd48	CD48 antigen	1.007	0.9001	0.2614	0.9613	No	0.9936	0.952	0.869	0.283	No	No
103090_at	2410003P15 Rik	RIKEN cDNA 2410003P15 gene	0.997	0.8972	0.0027	0.4467	No	1.0158	0.703	0.0305	0.161	No	No
103091_at	Relb	avian reticuloendotheliosis viral (v-rel) oncogene related B	1.0844	0.0034	0	0.0661	Yes	1.0944	0.0043	0.0001	0.964	Yes	Yes
103092_at	Trim37	tripartite motif protein 37	1.0802	0.2333	0.0011	0.1838	No	1.006	0.951	0.0019	0.234	No	No
103094_at	Serf1	small EDRK-rich factor 1	1.0404	0.3346	0.1504	0.8641	No	1.0362	0.0394	0.0187	0.992	No	No
103095_at	Grb7	growth factor receptor bound protein 7	0.9498	0.4194	0.1674	0.4492	No	0.9258	0.112	0.0185	0.953	No	No
103096_at	Zfp259	zinc finger protein 259	0.9525	0.3507	0.8201	0.45	No	1.0572	0.267	0.169	0.202	No	No
103097_at	0610038F07 Rik	RIKEN cDNA 0610038F07 gene	0.9916	0.8089	0.0118	0.6334	No	1.0423	0.774	0.0132	0.343	No	No
103098_at	Baiap2	brain-specific angiogenesis inhibitor 1-associated protein 2	0.9389	0.3495	0.1374	0.5343	No	0.9463	0.155	0.0006	0.636	No	No
103099_f_at	Cxxc1	CXXC finger 1 (PHD domain)	0.983	0.7641	0.0208	0.3086	No	1.0293	0.465	0.0267	0.227	No	No
103100_at	Wwtr1	WW domain containing transcription regulator 1	0.9862	0.7706	0.1086	0.237	No	0.9079	0.0452	0.0269	0.352	No	No
103101_at	Tarbp2	TAR (HIV) RNA binding protein 2	0.9802	0.3213	0.0007	0.6377	No	0.9829	0.618	0.0005	0.126	No	No
103132_at	C78948	expressed sequence C78948	0.9878	0.8514	0.9247	0.3674	No	1.1142	0.0735	0.771	0.673	No	No
103198_at	6330512M04 Rik	RIKEN cDNA 6330512M04 gene	0.9415	0.098	0.0035	0.0844	No	1.0457	0.195	0.481	0.813	No	No
103199_at	Xcl1	chemokine (C motif) ligand 1	1.0242	0.6453	0.068	0.7834	No	0.882	0.0795	0.0087	0.725	No	No
103200_at	6330500D04 Rik	RIKEN cDNA 6330500D04 gene	1.0362	0.3194	0.0125	0.2178	No	0.9264	0.316	0.0129	0.935	No	No
103201_at	Ttk	Ttk protein kinase	1.032	0.617	0.1071	0.7083	No	1.0279	0.551	0.0063	0.84	No	No
103202_at	Gbp4	guanylate nucleotide binding protein 4	1.1829	0.0792	0.1632	0.1071	No	1.158	0.137	0.428	0.0565	No	No
103203_f_at	E2f8	E2F transcription factor 8	1.0826	0.367	0.0077	0.1278	No	1.0957	0.0894	0.0004	0.149	No	No
103204_r_at	E2f8	E2F transcription factor 8	1.0117	0.7366	0.1306	0.0039	No	1.1545	0.054	0.988	0.0091	No	No
103205_at	Tcirg1	T-cell, immune regulator 1, ATPase, H+ transporting, lysosomal V0 protein A3	0.9698	0.1353	0.027	0.1463	No	1.0019	0.994	0.0249	0.426	No	No
103206_at	Cog5	component of oligomeric golgi complex 5	0.9409	0.26	0.0062	0.3193	No	1.0667	0.271	0.0054	0.0595	No	No
103207_at	Pola1	polymerase (DNA directed), alpha 1	1.0606	0.5201	0.0391	0.3848	No	1.0488	0.179	0.0015	0.059	No	No
103208_at	Kcnc1	potassium voltage gated channel, Shaw-related subfamily, member 1	0.9878	0.9169	0.8674	0.5206	No	1.0106	0.915	0.379	0.399	No	No
103209_at	Ltbp1	latent transforming growth factor beta binding protein 1	0.886	0.0603	0.0428	0.2142	No	0.8599	0.0638	0.0701	0.963	No	No
103210_at	Csf2rb2	colony stimulating factor 2 receptor, beta 2, low-affinity (granulocyte-macrophage)	1.1019	0.0649	0.0672	0.0079	No	1.1455	0.0044	0.0007	0.0023	No	No
103211_at	Wdfy2	WD repeat and FYVE domain containing 2	0.9038	0.1303	0.6489	0.2727	No	0.9916	0.731	0.0043	0.396	No	No
103212_at	Cdca7l	cell division cycle associated 7 like	1.1234	0.01	0.0025	0.0136	Yes	1.1022	0.0274	0.003	0.0296	No	Yes
103213_at	Cdh15 /// LOC672914	cadherin 15 /// similar to Muscle-cadherin precursor (M-cadherin) (Cadherin-15) (Cadherin-14)	0.9563	0.2332	0.003	0.0417	No	0.9299	0.252	0.552	0.862	No	No
103214_at	Hspb2	heat shock protein 2	0.8867	0.0001	0	0	No	0.9371	0.0659	0.02	0.117	No	No
103215_g_at	Hspb2	heat shock protein 2	0.8555	0.0056	0.0528	0.0064	No	0.9497	0.144	0.0737	0.0413	No	No
103216_f_at	Ikbkg	inhibitor of kappaB kinase gamma	0.918	0.0732	0.0719	0.457	No	0.9994	0.996	0.346	0.0282	No	No
103217_at	Cflar	CASP8 and FADD-like apoptosis regulator	0.91	0.0021	0.0026	0.0077	No	0.9333	0.0936	0.0071	0.275	No	No
103218_at	Slc10a3	solute carrier family 10 (sodium/bile acid cotransporter family), member 3	1.0448	0.2677	0.1795	0.846	No	1.0416	0.26	0.0448	0.292	No	No
103219_at	Acpp	acid phosphatase, prostate	0.9264	0.3783	0.1525	0.5918	No	0.9755	0.553	0.153	0.779	No	No
103220_at	Ndnl2	necdin-like 2	0.9785	0.7487	0.7463	0.3762	No	1.0642	0.264	0.159	0.233	No	No
103221_at	Saal1	serum amyloid A-like 1	0.9549	0.0212	0.0012	0.8738	No	0.9914	0.812	0.122	0.796	No	No
103222_at	Eps8	epidermal growth factor receptor pathway substrate 8	1.0127	0.7451	0.0003	0.4876	No	0.991	0.607	0.0003	0.205	No	No
103223_at	Prkce	protein kinase C, epsilon	1.164	0.0919	0.0003	0.9787	No	1.0995	0.0365	0.0003	0.046	No	No
103224_at	Rab33a	RAB33A, member of RAS oncogene family	1.1141	0.0128	0.0066	0.0399	No	1.092	0.0743	0.631	0.019	No	No
103225_at	Dnase1l1	deoxyribonuclease 1-like 1	1.0014	0.9792	0.955	0.0465	No	0.9805	0.697	0.753	0.0206	No	No
103226_at	Mrc1	mannose receptor, C type 1	0.9002	0.219	0.0031	0.5304	No	0.8966	0.0368	0.0009	0.123	No	No
103227_at	Ugt2b34	UDP glucuronosyltransferase 2 family, polypeptide B34	0.9728	0.4137	0.3749	0.1453	No	0.9472	0.299	0.0242	0.148	No	No
103228_at	Mtmr7	myotubularin related protein 7	1.0177	0.5288	0.0007	0.4299	No	1.0156	0.402	0.0011	0.0416	No	No
103229_at	Ncoa1	nuclear receptor coactivator 1	1.0036	0.9491	0.0068	0.8731	No	1.1383	0.0676	0.033	0.272	No	No
103231_at	Rhoh	ras homolog gene family, member H	0.9705	0.562	0.023	0.2606	No	1.0223	0.475	0.196	0.076	No	No
103232_at	Bpy2ip1	BPY2 interacting protein 1	0.9571	0.1045	0.0044	0.0843	No	0.9659	0.401	0.413	0.818	No	No
103233_at	Hipk3	homeodomain interacting protein kinase 3	0.9467	0.3101	0.1132	0.5364	No	0.9933	0.806	0.0635	0.158	No	No
103234_at	Nefh	neurofilament, heavy polypeptide	1.1111	0.0326	0.0032	0.2628	No	1.1619	0.262	0.88	0.184	No	No
103235_at	Npy	neuropeptide Y	1.0369	0.3198	0.6963	0.9632	No	1.0097	0.826	0.827	0.219	No	No
103236_at	Ring1	ring finger protein 1	1.0494	0.2234	0.0021	0.5135	No	1.0115	0.895	0.0011	0.117	No	No
103237_at	Mxd3	Max dimerization protein 3	1.0919	0.0765	0.009	0.8726	No	1.0028	0.812	0.0194	0.142	No	No
103238_at	Wnt4	wingless-related MMTV integration site 4	0.9933	0.91	0.0163	0.0785	No	0.9057	0.0364	0.547	0.0147	No	No
103239_at	Cdx2	caudal type homeo box 2	0.857	0.0726	0.0055	0.837	No	1.102	0.0986	0.0005	0.0029	No	No
103240_f_at	Ear2	eosinophil-associated, ribonuclease A family, member 2	1.0106	0.6818	0.73	0.395	No	0.916	0.018	0.196	0.0175	No	No
103241_at	Adcy2	adenylate cyclase 2	0.9414	0.0177	0.0003	0.6398	No	1.0314	0.143	0.001	0.017	No	No
103242_at	Ap1g1	adaptor protein complex AP-1, gamma 1 subunit	1.0238	0.4189	0.0155	0.1005	No	0.9725	0.204	0.0095	0.4	No	No
103243_at	Emp2	epithelial membrane protein 2	0.9591	0.4317	0.4113	0.9496	No	1.0166	0.64	0.0133	0.0066	No	No
103244_at	Clec11a	C-type lectin domain family 11, member a	1.0351	0.292	0.0016	0.4296	No	1.0551	0.183	0.0154	0.486	No	No
103245_at	Cst8	cystatin 8 (cystatin-related epididymal spermatogenic)	0.9921	0.8875	0.4916	0.7256	No	0.968	0.783	0.0404	0.313	No	No
103247_at	Mpp3	membrane protein, palmitoylated 3 (MAGUK p55 subfamily member 3)	1.0723	0.142	0.0993	0.0257	No	0.9853	0.593	0.004	0.17	No	No
103248_at	Fkbp1b	FK506 binding protein 1b	0.9747	0.2419	0.0002	0.0405	No	0.9575	0.0183	0.16	0.0171	No	No
103249_at	Chrd	chordin	0.8921	0.0882	0.0033	0.8421	No	1.0145	0.738	0.0222	0.89	No	No
103250_at	Dfna5h	deafness, autosomal dominant 5 homolog (human)	0.9475	0.3084	0.0511	0.8343	No	0.9686	0.403	0.0013	0.355	No	No
103251_at	Cdadc1	cytidine and dCMP deaminase domain containing 1	0.9187	0.2244	0.0214	0.9208	No	0.9145	0.157	0.0235	0.979	No	No
103252_at	Gpr37	G protein-coupled receptor 37	1.0102	0.8314	0.736	0.7324	No	1.0737	0.232	0.755	0.387	No	No
103253_at	Lin7b	lin-7 homolog B (C. elegans)	1.006	0.8772	0.0034	0.3205	No	1.0439	0.332	0.0022	0.206	No	No
103254_at	Trafd1	TRAF type zinc finger domain containing 1	0.9127	0.1093	0.0415	0.2861	No	0.9156	0.144	0.0192	0.154	No	No

103255_at	Traf5 /// LOC622602	Tnf receptor-associated factor 5 /// similar to TNF receptor-associated factor 5	1.0531	0.1148	0.4017	0.6182	No	0.9947	0.797	0.0297	0.512	No	No
103256_at	Ms4a4d	membrane-spanning 4-domains, subfamily A, member 4D	1.0449	0.3188	0.6878	0.247	No	1.0525	0.549	0.0591	0.724	No	No
103257_at	Tmem56	transmembrane protein 56	0.998	0.9483	0.0166	0.778	No	1.0336	0.352	0.0018	0.762	No	No
103258_at	Ly75	lymphocyte antigen 75	1.0561	0.3939	0.0202	0.0431	No	1.0985	0.0058	0.0001	0.0008	No	No
103259_at	Gfi1	growth factor independent 1	0.956	0.4426	0.1359	0.4522	No	1.0852	0.404	0.398	0.308	No	No
103260_at	1110060D06 Rik /// Dock5	RIKEN cDNA 1110060D06 gene /// dedicator of cytokinesis 5	0.9663	0.6075	0.0482	0.1272	No	1.0614	0.338	0.35	0.0483	No	No
103261_at	Gspt2	G1 to S phase transition 2	1.065	0.1534	0.035	0.0453	No	0.9623	0.335	0.0024	0.502	No	No
103262_at	Hipk1	homeodomain interacting protein kinase 1	0.9684	0.5551	0.0963	0.7172	No	1.0772	0.329	0.0743	0.474	No	No
103263_at	Epc1	enhancer of polycomb homolog 1 (Drosophila)	0.9692	0.3184	0.0021	0.2855	No	0.9891	0.657	0.0047	0.173	No	No
103264_at	Mtmr1	myotubularin related protein 1	0.9531	0.1773	0.8699	0.6573	No	1.0305	0.555	0.972	0.543	No	No
103265_r_at	Epb4.1l1	erythrocyte protein band 4.1-like 1	0.954	0.457	0.0897	0.3497	No	1.121	0.135	0.0118	0.0948	No	No
103266_at	Ltb4r1	leukotriene B4 receptor 1	0.9887	0.6953	0.1076	0.2239	No	0.9178	0.0114	0.102	0.0222	No	No
103267_i_at	---	Adult male olfactory brain cDNA, RIKEN full-length enriched library, clone:6430562O12 product:hypothetical protein, full insert sequence	0.9829	0.7165	0.0173	0.0258	No	0.941	0.47	0.178	0.204	No	No
103268_r_at	---	Adult male olfactory brain cDNA, RIKEN full-length enriched library, clone:6430562O12 product:hypothetical protein, full insert sequence	0.992	0.876	0.8043	0.1242	No	1.0401	0.716	0.81	0.177	No	No
103269_f_at	Zfp125	zinc finger protein 125	0.965	0.6131	0.0007	0.2308	No	0.9802	0.775	0.0003	0.102	No	No
103270_at	Gtse1	G two S phase expressed protein 1	0.9878	0.7675	0.0099	0.3059	No	0.99	0.863	0.0881	0.311	No	No
103271_at	Lrp6	low density lipoprotein receptor-related protein 6	0.8828	0.0675	0.004	0.727	No	0.8566	0.171	0.0137	0.861	No	No
103272_at	Capn3	calpain 3	0.9443	0.187	0.4441	0.6624	No	1.0893	0.0075	0.0006	0.0089	No	No
103273_s_at	Abcc8	ATP-binding cassette, sub-family C (CFTR/MRP), member 8	0.9555	0.4073	0.5288	0.0816	No	1.0551	0.672	0.642	0.147	No	No
103274_at	Abcc8	ATP-binding cassette, sub-family C (CFTR/MRP), member 8	1.0764	0.0623	0.7299	0.056	No	1.0712	0.392	0.165	0.111	No	No
103275_at	Atp6v0a1	ATPase, H+ transporting, lysosomal V0 subunit A1	1.0245	0.4176	0.5442	0.0356	No	1.0215	0.167	0.236	0.0011	No	No
103276_at	Large	like-glycosyltransferase	0.9857	0.7057	0.0564	0.2501	No	1.0257	0.594	0.82	0.44	No	No
103277_s_at	Med12	mediator of RNA polymerase II transcription, subunit 12 homolog (yeast)	0.8317	0.0007	0.0003	0.0075	No	0.8741	0.0094	0.0025	0.0589	Yes	No
103278_at	Padi4	peptidyl arginine deiminase, type IV	0.9777	0.5481	0.9401	0.2429	No	1.0606	0.203	0.304	0.173	No	No
103279_at	Sh2d1a	SH2 domain protein 1A	1.0394	0.569	0.0531	0.7733	No	1.0922	0.414	0.887	0.274	No	No
103281_at	Cd2ap	CD2-associated protein	1.0302	0.6372	0.0141	0.3076	No	0.9483	0.434	0.0446	0.884	No	No
103282_at	Rasgrp2	RAS, guanyl releasing protein 2	0.9731	0.3634	0.1457	0.4252	No	0.8354	0.0023	0.0016	0.0428	Yes	No
103283_at	Elf5	E74-like factor 5	1.0595	0.3095	0.6016	0.2458	No	1.2139	0.0642	0.14	0.0204	No	No
103284_at	Cyp8b1	cytochrome P450, family 8, subfamily b, polypeptide 1	0.9917	0.917	0.2611	0.7513	No	0.9517	0.14	0.0065	0.151	No	No
103285_at	Mbd4	methyl-CpG binding domain protein 4	1.0186	0.7321	0.0092	0.113	No	0.9334	0.333	0.0337	0.986	No	No
103286_at	Stam2	signal transducing adaptor molecule (SH3 domain and ITAM motif) 2	0.941	0.2649	0.4541	0.4094	No	0.9131	0.164	0.0107	0.175	No	No
103288_at	Nrip1	nuclear receptor interacting protein 1	0.6931	0.0086	0.0001	0.0336	Yes	0.8606	0.0006	0	0.0005	No	No
103289_at	Corin	corin	0.9569	0.0232	0.0508	0.0005	No	0.963	0.56	0.146	0.115	No	No
103291_at	Magea2	melanoma antigen, family A, 2	1.0271	0.5411	0.27	0.921	No	1.0733	0.168	0.0558	0.671	No	No
103292_at	Stc1	stanniocalcin 1	1.0576	0.4177	0.4062	0.8827	No	0.9895	0.808	0.0294	0.284	No	No
103293_at	Gosr1	golgi SNAP receptor complex member 1	0.9833	0.7017	0.0068	0.8556	No	1.0366	0.397	0.0432	0.32	No	No
103294_at	Rgs5	regulator of G-protein signaling 5	1.1589	0.0005	0.0084	0.0005	No	1.1483	0.001	0.0007	0.0005	No	No
103295_at	9130229H14 Rik	RIKEN cDNA 9130229H14 gene	1.0324	0.6249	0.0153	0.6148	No	1.0414	0.222	0.0007	0.153	No	No
103296_at	Cst9	cystatin 9	0.9253	0.2758	0.4989	0.3776	No	1.0436	0.68	0.0444	0.252	No	No
103297_at	Pfkfb1	6-phosphofructo-2-kinase/fructose-2,6-biphosphatase 1	1.0289	0.7077	0.1521	0.4004	No	1.0164	0.762	0.725	0.148	No	No
103298_at	Speg	SPEG complex locus	0.9416	0.636	0.0169	0.6156	No	1.0971	0.131	0.0443	0.0907	No	No
103299_at	Pld4	phospholipase D family, member 4	0.9281	0.2468	0.125	0.6487	No	0.9332	0.297	0.239	0.91	No	No
103300_at	Abcb7	ATP-binding cassette, sub-family B (MDR/TAP), member 7	1.0624	0.385	0.0096	0.3992	No	1.1027	0.416	0.324	0.587	No	No
103301_i_at	Sox3	SRY-box containing gene 3	1.0659	0.2462	0.1054	0.289	No	1.0112	0.946	0.517	0.471	No	No
103302_r_at	Sox3	SRY-box containing gene 3	1.1057	0.234	0.0387	0.2766	No	0.9892	0.907	0.58	0.368	No	No
103303_at	Chd7	Chromodomain helicase DNA binding protein 7	1.0776	0.5587	0.0044	0.286	No	0.923	0.357	0.0031	0.563	No	No
103305_at	Itgb4	integrin beta 4	0.9642	0.2347	0.9678	0.0184	No	0.9493	0.15	0.645	0.0086	No	No
103306_at	Serpinb6c	serine (or cysteine) peptidase inhibitor, clade B, member 6c	0.9568	0.1538	0.0544	0.0138	No	0.9446	0.439	0.397	0.598	No	No
103308_at	C79407	expressed sequence C79407	0.9904	0.7837	0.0081	0.446	No	1.0532	0.163	0.0288	0.0116	No	No
103309_at	Frap1	FK506 binding protein 12-rapamycin associated protein 1	0.8536	0.0108	0.0087	0.1167	No	0.9385	0.0421	0.005	0.142	No	No
103310_at	Jarid1b	jumonji, AT rich interactive domain 1B (Rbp2 like)	0.9947	0.9363	0.0102	0.1792	No	0.9961	0.971	0.0676	0.489	No	No
103311_at	Osbpl5	oxysterol binding protein-like 5	1.0035	0.9321	0.0058	0.0629	No	1.0059	0.798	0.0046	0.028	No	No
103312_f_at	2610101J03 Rik	RIKEN cDNA 2610101J03 gene	0.9698	0.5536	0.0005	0.3409	No	0.9924	0.749	0.0248	0.0568	No	No
103313_r_at	2610101J03 Rik	RIKEN cDNA 2610101J03 gene	0.9754	0.2434	0.0002	0.0113	No	0.6631	0.531	0.276	0.0984	No	No
103314_at	Parp8	poly (ADP-ribose) polymerase family, member 8	1.0429	0.6165	0.0206	0.1077	No	0.956	0.631	0.674	0.705	No	No
103315_at	Tnrc6a	trinucleotide repeat containing 6a	0.9146	0.1032	0.0008	0.1073	No	0.9815	0.724	0.0952	0.746	No	No
103316_at	Camsap1	calmodulin regulated spectrin-associated protein 1	0.912	0.142	0.2324	0.7197	No	0.9201	0.0658	0.867	0.0453	No	No
103317_at	Coch	coagulation factor C homolog (Limulus polyphemus)	1.1253	0.0821	0.0003	0.3141	No	1.0863	0.456	0.003	0.639	No	No
103318_at	Gabpb1	GA repeat binding protein, beta 1	1.0631	0.0597	0.165	0.0046	No	1.044	0.362	0.764	0.0876	No	No
103319_at	Psmd10	proteosome (prosome, macropain) 26S subunit, non-ATPase, 10	1.0307	0.7473	0.349	0.3062	No	0.9483	0.341	0.0009	0.239	No	No
103321_at	Mobkl1a	MOB1, Mps One Binder kinase activator-like 1A (yeast)	1.0389	0.5992	0.0016	0.2357	No	0.9855	0.328	0.0001	0.0877	No	No
103322_at	Rpia	ribose 5-phosphate isomerase A	0.9865	0.7595	0.0154	0.797	No	0.9482	0.487	0.0527	0.795	No	No
103326_at	Lrrc35	leucine rich repeat containing 35	0.9178	0.0252	0.1793	0.0447	No	1.0128	0.556	0.0253	0.0321	No	No
103327_at	Prrx2	paired related homeobox 2	1.1519	0.0077	0.0013	0.0443	Yes	1.0645	0.0259	0.0013	0.779	No	Yes
103328_at	Tank	TRAF family member-associated Nf-kappa B activator	1.212	0.1302	0.0049	0.7043	No	1.0635	0.333	0.0081	0.508	No	No
103329_at	Strbp	spermatid perinuclear RNA binding protein	1.0588	0.2207	0.0244	0.1016	No	0.9861	0.86	0.124	0.856	No	No
103330_at	Strbp	spermatid perinuclear RNA binding protein	0.9088	0.3953	0.0613	0.8594	No	0.9837	0.563	0.111	0.945	No	No
103331_at	C030006K11 Rik	RIKEN cDNA C030006K11 gene	0.9594	0.3636	0.1988	0.2057	No	0.9175	0.151	0.0452	0.059	No	No
103332_at	Gpr125	G protein-coupled receptor 125	1.0081	0.9237	0.4048	0.8503	No	0.9242	0.308	0.3	0.992	No	No
103333_at	G6pc	glucose-6-phosphatase, catalytic	0.995	0.9155	0.3579	0.8147	No	0.9891	0.812	0.0771	0.473	No	No
103334_at	Crcp	calcitonin gene-related peptide-receptor component protein	1.0653	0.0791	0.0199	0.3768	No	1.0449	0.133	0.108	0.672	No	No
103335_at	Lgals9	lectin, galactose binding, soluble 9	1.0379	0.2124	0.1104	0.899	No	1.07	0.136	0.142	0.512	No	No
103336_r_at	Bpnt1	bisphosphate 3'-nucleotidase 1	1.0026	0.9614	0.283	0.1179	No	1.0642	0.568	0	0.675	No	No
103338_at	Parp6	poly (ADP-ribose) polymerase family, member 6	0.9254	0.0925	0.0465	0.5518	No	0.9182	0.0343	0.0175	0.565	No	No
103340_at	Rhd	Rh blood group, D antigen	0.9794	0.5629	0.0804	0.0851	No	1.0234	0.594	0.331	0.0504	No	No
103341_at	Ctps	cytidine 5'-triphosphate synthase	1.0409	0.138	0.1148	0.1669	No	1.0719	0.1	0.015	0.348	No	No
103342_at	Eed	embryonic ectoderm development	0.979	0.8485	0.2346	0.2889	No	0.9236	0.39	0.561	0.173	No	No
103343_at	5430432M24 Rik	RIKEN cDNA 5430432M24 gene	0.9443	0.1579	0.0989	0.9198	No	0.9204	0.0282	0.004	0.637	No	No
103344_at	Dnajc1	DnaJ (Hsp40) homolog, subfamily C, member 1	0.9769	0.6646	0.1081	0.5767	No	1.0393	0.408	0.371	0.347	No	No
103345_at	Spna2 /// LOC381358	spectrin alpha 2 /// similar to Spectrin alpha chain, brain (Spectrin, non-erythroid alpha chain) (Alpha-II spectrin) (Fodrin alpha chain)	0.9588	0.4019	0.0106	0.9305	No	1.0487	0.155	0.0082	0.164	No	No
103346_at	Clk2	CDC-like kinase 2	0.9855	0.63	0.3204	0.5271	No	1.0539	0.143	0.0094	0.128	No	No
103347_at	Cramp1l	Crm, cramped-like (Drosophila)	0.9451	0.3518	0.7645	0.096	No	1.0013	0.951	0.356	0.0464	No	No
103348_at	Osbp	oxysterol binding protein	0.948	0.1356	0.1518	0.2339	No	1.07	0.245	0.591	0.167	No	No
103349_at	Lyn /// LOC676654	Yamaguchi sarcoma viral (v-yes-1) oncogene homolog /// similar to Yamaguchi sarcoma viral (v-yes-1) oncogene homolog	1.0761	0.4192	0.1648	0.6867	No	0.9518	0.319	0.53	0.137	No	No
103350_at	Psmd7	proteasome (prosome, macropain) 26S subunit, non-ATPase, 7	0.9697	0.4602	0.2483	0.1692	No	0.9859	0.72	0.647	0.0979	No	No

103352_at	Dpagt1	dolichyl-phosphate (UDP-N-acetylglucosamine) acetylglucosaminephosphotransferase 1 (GlcNAc-1-P transferase)	1.0085	0.827	0.1341	0.374	No	1.0012	0.956	0.048	0.516	No	No
103353_f_at	Cyp4b1	cytochrome P450, family 4, subfamily b, polypeptide 1	1.1396	0.0217	0.0152	0.0149	No	1.1435	0.0059	0.0234	0.0045	No	No
103354_at	Mrps31	mitochondrial ribosomal protein S31	0.9252	0.0253	0.8223	0.0116	No	1.0059	0.717	0.0957	0.0158	No	No
103355_at	2010001M06 Rik	RIKEN cDNA 2010001M06 gene	0.8986	0.4373	0.0535	0.516	No	1.006	0.855	0.0269	0.221	No	No
103356_at	Dock7	dedicator of cytokinesis 7	1.0031	0.9586	0.0258	0.2399	No	1.0724	0.315	0.0158	0.103	No	No
103357_at	Slc2a2	solute carrier family 2 (facilitated glucose transporter), member 2	1.0405	0.3398	0.9838	0.2539	No	1.0211	0.574	0.0186	0.118	No	No
103359_at	Mettl8	methyltransferase like 8	1.037	0.4101	0.0514	0.0468	No	1.1297	0.0048	0.0001	0.0017	No	No
103360_at	Tssk1	testis-specific serine kinase 1	0.9429	0.3813	0.1266	0.4801	No	0.9715	0.437	0.0094	0.0824	No	No
103361_at	6230416A05 Rik	RIKEN cDNA 6230416A05 gene	1.081	0.1259	0.0222	0.2882	No	1.0762	0.163	0.0088	0.0997	No	No
103362_at	Ptger4	prostaglandin E receptor 4 (subtype EP4)	0.8725	0.0369	0.3252	0.8729	No	0.7997	0.117	0.698	0.91	No	No
103363_at	Tbn	taube nuss	0.9793	0.4646	0.2336	0.0275	No	0.9711	0.275	0.0111	0.296	No	No
103364_f_at	5730494M16 Rik	RIKEN cDNA 5730494M16 gene	1.16	0.0175	0.0035	0.9687	No	1.1143	0.0139	0.0003	0.199	No	No
103365_s_at	Arhgap9	Rho GTPase activating protein 9	0.9149	0.0353	0.0017	0.0214	No	0.9289	0.163	0.993	0.0512	No	No
103366_at	Arhgap9	Rho GTPase activating protein 9	1.0171	0.794	0.7507	0.7217	No	1.0158	0.0086	0	0.0001	No	No
103367_at	B4galnt1	beta-1,4-N-acetyl-galactosaminyl transferase 1	0.9516	0.187	0.0151	0.0226	No	0.9362	0.251	0.0459	0.101	No	No
103369_at	9430029L20 Rik	RIKEN cDNA 9430029L20 gene	0.8886	0.0441	0.1025	0.3539	No	0.967	0.344	0.87	0.256	No	No
103370_at	Lin7c	lin-7 homolog C (C. elegans)	1.0232	0.547	0.0004	0.2629	No	1.0026	0.988	0.0011	0.812	No	No
103371_at	Slc39a7	solute carrier family 39 (zinc transporter), member 7	1.0563	0.3568	0.769	0.4509	No	1.0704	0.331	0.515	0.457	No	No
103372_at	0610007P22 Rik	RIKEN cDNA 0610007P22 gene	0.9779	0.7878	0.0149	0.3972	No	1.02	0.734	0.0565	0.466	No	No
103374_at	Zcchc3	zinc finger, CCHC domain containing 3	1.0735	0.3011	0.2579	0.6904	No	1.0101	0.768	0.495	0.825	No	No
103375_at	Zcchc3	zinc finger, CCHC domain containing 3	1.0155	0.6842	0.1508	0.1711	No	1.0171	0.481	0.0022	0.389	No	No
103376_s_at	Pitpnm1	phosphatidylinositol membrane-associated 1	1.0066	0.8623	0.0058	0.296	No	1.0737	0.0302	0.0004	0.0523	No	No
103377_at	Lrp2	low density lipoprotein receptor-related protein 2	1.0364	0.3997	0.0004	0.0859	No	1.0293	0.0117	0	0.0001	No	No
103378_at	Prlpa	prolactin-like protein A	0.9442	0.2992	0.25	0.7825	No	0.996	0.949	0.7	0.342	No	No
103379_at	Fbxo3	F-box only protein 3	1.1355	0.2247	0.0056	0.2893	No	1.0307	0.671	0.0011	0.996	No	No
103381_at	Sec31l1	SEC31-like 1 (S. cerevisiae)	0.9909	0.8213	0.0119	0.468	No	0.9893	0.737	0.0049	0.904	No	No
103384_at	Rgs10	Regulator of G-protein signalling 10	1.1038	0.0802	0.8635	0.3553	No	1.1404	0.011	0.928	0.0056	No	No
103385_at	Tera	teratocarcinoma expressed, serine rich	0.9539	0.3531	0.0158	0.5979	No	0.9893	0.906	0.0272	0.24	No	No
103386_at	Acot8	acyl-CoA thioesterase 8	1.0511	0.2794	0.3937	0.9954	No	1.0201	0.595	0.0123	0.139	No	No
103387_at	Phf1	PHD finger protein 1	1.0442	0.298	0.0116	0.3466	No	1.0778	0.111	0.0064	0.0406	No	No
103388_at	P42pop	Myb protein P42POP	0.9837	0.3971	0.579	0.1377	No	0.9907	0.881	0.402	0.674	No	No
103389_at	Aass	aminoadipate-semialdehyde synthase	1.0233	0.5966	0.328	0.1352	No	0.9217	0.232	0.227	0.658	No	No
103391_at	Ccbl2	cysteine conjugate-beta lyase 2	0.8793	0.0204	0.8742	0.0229	No	0.8888	0.0661	0.594	0.0081	No	No
103392_at	Adcy7	adenylate cyclase 7	1.003	0.9594	0.0432	0.9504	No	1.0044	0.827	0.0265	0.143	No	No
103393_at	Pspc1	paraspeckle protein 1	1.0891	0.2841	0.002	0.5045	No	1.0376	0.73	0.0006	0.355	No	No
103394_at	Fxyd5	FXYD domain-containing ion transport regulator 5	1.0789	0.0585	0.0009	0.1444	No	1.0483	0.0053	0.0001	0.0014	No	No
103395_at	Sgca	sarcoglycan, alpha (dystrophin-associated glycoprotein)	0.8588	0.0182	0.0318	0.0116	No	0.8837	0.066	0.0471	0.0466	No	No
103397_at	Hrb	HIV-1 Rev binding protein	1.0192	0.5948	0.0054	0.34	No	1.0412	0.122	0.0555	0.663	No	No
103398_at	Pxn	paxillin	1.0213	0.5342	0.0003	0.2293	No	1.0182	0.788	0.008	0.808	No	No
103399_at	Scmh1	sex comb on midleg homolog 1	0.977	0.6308	0.3826	0.4992	No	1.0069	0.86	0.227	0.85	No	No
103400_at	Tug1	taurine upregulated gene 1	0.9827	0.8701	0.3314	0.4484	No	0.9428	0.54	0.31	0.778	No	No
103401_at	Acads	acyl-Coenzyme A dehydrogenase, short chain	0.9569	0.211	0.0091	0.0938	No	0.9401	0.192	0.099	0.332	No	No
103402_at	Tm7sf3	transmembrane 7 superfamily member 3	1.0725	0.2669	0.9387	0.5214	No	1.0461	0.431	0.51	0.911	No	No
103403_at	2310007H09 Rik	RIKEN cDNA 2310007H09 gene	1.004	0.9495	0.8233	0.7869	No	0.997	0.986	0.467	0.0647	No	No
103404_at	Rere	arginine glutamic acid dipeptide (RE) repeats	0.9872	0.7685	0.997	0.5134	No	0.9062	0.0162	0.0009	0.706	No	No
103405_at	2610019A05 Rik	RIKEN cDNA 2610019A05 gene	1.0395	0.2905	0.0152	0.6668	No	1.043	0.386	0.0084	0.9	No	No
103406_at	Xab1	XPA binding protein 1	1.0631	0.2046	0.1918	0.0353	No	1.047	0.342	0.411	0.0244	No	No
103407_at	1300017J02 Rik	RIKEN cDNA 1300017J02 gene	1.0068	0.9236	0.6703	0.1852	No	1.1221	0.0278	0.0926	0.0093	No	No
103408_at	Prkd2	protein kinase D2	0.923	0.09	0.2622	0.2237	No	0.917	0.0242	0.007	0.395	No	No
103409_at	Baiap2l1	BAI1-associated protein 2-like 1	0.9256	0.17	0.8898	0.7472	No	1.0159	0.602	0.0434	0.306	No	No
103411_at	Gna11	guanine nucleotide binding protein, alpha 11	1.0163	0.5138	0.0002	0.3029	No	1.0368	0.0008	0	0.0741	Yes	No
103412_at	3732412D22 Rik	RIKEN cDNA 3732412D22 gene	0.9414	0.3541	0.0002	0.3053	No	0.9633	0.515	0	0.845	No	No
103413_at	Pwp1	PWP1 homolog (S. cerevisiae)	1.044	0.3984	0.2031	0.7388	No	0.9507	0.271	0.278	0.95	No	No
103414_at	Skiv2l	superkiller viralicidic activity 2-like (S. cerevisiae)	1.1163	0.0275	0.071	0.1318	No	1.1824	0.0261	0.408	0.0282	No	No
103415_at	Wrnip1	Werner helicase interacting protein 1	1.01	0.8591	0.0242	0.5518	No	1.0456	0.534	0.0316	0.909	No	No
103416_at	Mapk6	mitogen-activated protein kinase 6	1.0579	0.1888	0.0055	0.0125	No	1.0012	0.668	0.003	0.0079	No	No
103418_at	Rfc4	replication factor C (activator 1) 4	1.0144	0.6719	0.9652	0.3373	No	1.0598	0.209	0.117	0.731	No	No
103420_at	Emd	emerin	0.9575	0.5751	0.6357	0.5326	No	1.0034	0.918	0.928	0.191	No	No
103421_at	Frrs1 /// LOC635993	ferric-chelate reductase 1 /// similar to stromal cell derived factor receptor 2	0.9281	0.0659	0.5065	0.0395	No	0.8861	0.0654	0.0121	0.0369	No	No
103422_at	Cd1d1	CD1d1 antigen	1.0099	0.8726	0.0012	0.0369	No	0.9966	0.535	0.0003	0.0266	No	No
103423_at	Cyb561	cytochrome b-561	0.9663	0.4309	0.0062	0.7019	No	1.0531	0.0618	0.0021	0.0168	No	No
103424_at	6330442E10 Rik	RIKEN cDNA 6330442E10 gene	0.941	0.2725	0.0912	0.954	No	0.9346	0.235	0.0123	0.718	No	No
103427_at	Fbxl3	F-box and leucine-rich repeat protein 3	0.854	0.1842	0.77	0.7863	No	0.9554	0.536	0.143	0.995	No	No
103428_at	Pold3	polymerase (DNA-directed), delta 3, accessory subunit	0.9747	0.4245	0.6724	0.966	No	1.0159	0.606	0.241	0.144	No	No
103429_i_at	Adi1	acireductone dioxygenase 1	1.1077	0.4954	0.2945	0.6084	No	1.0643	0.501	0.784	0.407	No	No
103430_at	Dbn1	drebrin 1	1.1057	0.0321	0.0205	0.2902	No	1.0758	0.257	0.45	0.89	No	No
103432_at	Isg20	interferon-stimulated protein	0.9904	0.6849	0.2484	0.0142	No	0.9281	0.172	0.958	0.039	No	No
103433_at	Pscd3	pleckstrin homology, Sec7 and coiled-coil domains 3	0.837	0.0515	0.4356	0.7042	No	0.9548	0.547	0.0479	0.906	No	No
103434_at	Pscd3	pleckstrin homology, Sec7 and coiled-coil domains 3	0.896	0.2183	0.1687	0.7856	No	0.9312	0.0584	0.0089	0.195	No	No
103435_at	9430080K19 Rik	RIKEN cDNA 9430080K19 gene	0.9795	0.7953	0.0227	0.4464	No	0.8978	0.131	0.861	0.334	No	No
103436_at	Gtpbp1	GTP binding protein 1	1.021	0.4706	0.0135	0.6423	No	1.0344	0.631	0.362	0.887	No	No
103437_at	Zfp57	zinc finger protein 57	1.0789	0.1141	0.0358	0.1764	No	0.9649	0.466	0.0765	0.233	No	No
103438_at	Dio2	deiodinase, iodothyronine, type II	0.9166	0.021	0.2323	0.0554	No	0.9482	0.268	0.0476	0.438	No	No
103439_at	Psd	pleckstrin and Sec7 domain containing	1.0098	0.8323	0.0775	0.1742	No	0.9563	0.0349	0.0007	0.161	No	No
103440_at	Gabpa	GA repeat binding protein, alpha	1.0427	0.7433	0.0336	0.6401	No	1.0072	0.976	0.255	0.918	No	No
103441_at	Csnk2a1	casein kinase II, alpha 1 polypeptide	0.9171	0.3205	0.7082	0.1913	No	0.936	0.35	0.101	0.249	No	No
103442_at	Dhrs7b	dehydrogenase/reductase (SDR family) member 7B	1.0341	0.6346	0.2631	0.3892	No	1.0493	0.464	0.329	0.612	No	No
103443_at	Aim1	absent in melanoma 1	1.0467	0.2535	0.0169	0.4167	No	1.0553	0.134	0.0043	0.189	No	No
103444_at	Dna2l	DNA2 DNA replication helicase 2-like (yeast)	1.0584	0.494	0.7484	0.7063	No	1.0848	0.114	0.021	0.0414	No	No
103445_at	Hoxb6	homeo box B6	1.0007	0.9825	0.005	0.2087	No	0.9175	0.176	0.0027	0.135	No	No
103446_at	Ifih1	interferon induced with helicase C domain 1	0.9669	0.2523	0.0103	0.0121	No	0.9399	0.102	0.003	0.326	No	No
103447_at	D15Wsu169 e	DNA segment, Chr 15, Wayne State University 169, expressed	1.002	0.9375	0.6564	0.2474	No	1.0172	0.768	0.798	0.827	No	No
103448_at	S100a8	S100 calcium binding protein A8 (calgranulin A)	0.8602	0.0715	0.0817	0.2181	No	0.9323	0.122	0.195	0.0736	No	No
103449_at	Lsm14b	LSM14 homolog B (SCD6, S. cerevisiae)	0.9639	0.6188	0.0734	0.3688	No	0.9111	0.18	0.112	0.39	No	No
103450_at	Bcas3	breast carcinoma amplified sequence 3	0.8841	0.0023	0.0003	0.1041	Yes	0.9162	0.126	0.0011	0.52	No	No
103451_at	Ptk2b	PTK2 protein tyrosine kinase 2 beta	1.0512	0.244	0.0005	0.9	No	1.2066	0.0011	0.0003	0.0009	No	No
103452_at	Prodh2	proline dehydrogenase (oxidase) 2	0.8704	0.0578	0.0505	0.7612	No	0.9537	0.323	0.345	0.803	No	No
103454_at	Spic	Spi-C transcription factor (Spi-1/PU.1 related)	0.9937	0.8922	0.8623	0.6225	No	1.0376	0.592	0.144	0.557	No	No
103455_at	Exoc8	exocyst complex component 8	0.9854	0.7156	0.3693	0.2785	No	0.9544	0.488	0.0078	0.278	No	No
103456_at	Ece1	endothelin converting enzyme 1	0.9167	0.1618	0.0329	0.4613	No	0.966	0.0967	0.0029	0.005	No	No
103457_at	Rev3l	REV3-like, catalytic subunit of DNA polymerase zeta RAD54 like (S. cerevisiae)	1.0031	0.9517	0.0001	0.152	No	1.0179	0.876	0.001	0.674	No	No
103458_at	Hc	hemolytic complement	0.8675	0.013	0.2908	0.3992	No	0.9723	0.624	0.696	0.391	No	No
103459_at	Slc39a6	solute carrier family 39 (metal ion transporter), member 6	1.1074	0.1172	0.0023	0.2917	No	1.1329	0.119	0.0385	0.46	No	No
103460_at	Ddit4	DNA-damage-inducible transcript 4	0.624	0.0013	0.0088	0.0432	Yes	0.6177	0.001	0.0006	0.0291	Yes	Yes
103462_at	Dock2	dedicator of cyto-kinesis 2	1.043	0.6358	0.5804	0.6939	No	1.2235	0.0606	0.0394	0.0647	No	No

103463_at	Pramel4 /// Pramel5	preferentially expressed antigen in melanoma like 4 /// preferentially expressed antigen in melanoma like 5	0.968	0.5777	0.0658	0.9631	No	1.0988	0.0713	0.0307	0.0199	No	No
103465_f_at	Saa2	serum amyloid A 2	0.9611	0.2755	0.0036	0.6259	No	1.0029	0.952	0.63	0.422	No	No
103466_at	Cyhr1	cysteine and histidine rich 1	0.972	0.5265	0.0625	0.6419	No	1.0269	0.625	0.0479	0.199	No	No
103467_g_at	Cyhr1	cysteine and histidine rich 1	0.9096	0.0697	0.1927	0.0075	No	0.9268	0.171	0.0486	0.012	No	No
103468_at	Mns1	meiosis-specific nuclear structural protein 1	1.004	0.9045	0.1468	0.9739	No	0.9889	0.844	0.119	0.538	No	No
103469_at	Elovl3	elongation of very long chain fatty acids (FEN1/Elo2, SUR4/Elo3, yeast)-like 3	0.9492	0.1663	0.3557	0.4139	No	0.9606	0.536	0.696	0.365	No	No
103470_at	Pramel6	preferentially expressed antigen in melanoma like 6	1.0282	0.696	0.9749	0.3402	No	1.0195	0.689	0.907	0.76	No	No
103471_at	Tbc1d15	TBC1 domain family, member 15	1.1067	0.3454	0.0196	0.0624	No	1.0371	0.545	0.0182	0.222	No	No
103472_at	BC053393	cDNA sequence BC053393	0.9606	0.2997	0.0046	0.1137	No	0.8592	0.115	0.111	0.811	No	No
103473_at	Pak4	p21 (CDKN1A)-activated kinase 4	1.0171	0.7413	0.1419	0.2989	No	1.022	0.769	0.651	0.888	No	No
103475_s_at	Ate1	arginine-tRNA-protein transferase 1	0.938	0.3284	0.3157	0.331	No	0.9565	0.418	0.628	0.749	No	No
103476_at	D5Ertd40e	DNA segment, Chr 5, ERATO Doi 40, expressed	1.0874	0.116	0.76	0.5975	No	1.0395	0.664	0.284	0.365	No	No
103477_at	Cdx1	caudal type homeo box 1	0.9854	0.6676	0.0066	0.2038	No	0.9063	0.0661	0.33	0.0789	No	No
103478_at	2210018M03 Rik	RIKEN cDNA 2210018M03 gene	1.0001	0.9985	0.0172	0.4491	No	0.9774	0.837	0.0495	0.419	No	No
103479_at	Samd10	sterile alpha motif domain containing 10	0.9886	0.7636	0.8193	0.1628	No	1.0214	0.487	0.0572	0.601	No	No
103480_at	Cd4	CD4 antigen	1.0035	0.8532	0.3715	0.5848	No	1.0892	0.0803	0.56	0.276	No	No
103481_at	AU016916	expressed sequence AU016916	0.9219	0.2292	0.0034	0.5458	No	0.8536	0.0255	0.0002	0.125	No	No
103483_at	Ercc5	excision repair cross-complementing rodent repair deficiency, complementation group 5	0.9873	0.2832	0.0001	0.0135	No	0.9094	0.195	0.0408	0.933	No	No
103484_at	Popdc3	popeye domain containing 3	0.938	0.098	0.1437	0.4075	No	0.8881	0.0754	0.0416	0.915	No	No
103485_at	BC030046	cDNA sequence BC030046	1.0279	0.4276	0.0006	0.0085	No	1.0264	0.585	0.206	0.756	No	No
103486_at	Il1b	interleukin 1 beta	0.9103	0.0403	0.5612	0.4299	No	0.9929	0.887	0.0061	0.368	No	No
103487_at	Ly6h	lymphocyte antigen 6 complex, locus H	1.0743	0.0635	0.21	0.2018	No	1.1035	0.007	0.69	0.413	Yes	No
103488_at	Selpl	selectin, platelet (p-selectin) ligand	0.9531	0.3615	0.7701	0.2708	No	1.0019	0.967	0.474	0.163	No	No
103489_at	Ubxd5	UBX domain containing 5	0.9889	0.7467	0.0149	0.2038	No	0.9143	0.0178	0.189	0.086	No	No
103490_at	Wnt11	wingless-related MMTV integration site 11	0.9216	0.0787	0.0021	0.012	No	0.8624	0.0374	0.0012	0.0026	No	No
103491_at	Nav1	neuron navigator 1	0.9699	0.5737	0.7765	0.3515	No	0.9789	0.532	0.213	0.418	No	No
103492_at	Cpxm1	carboxypeptidase X 1 (M14 family)	1.1051	0.0662	0.0475	0.855	No	1.0814	0.0643	0.0015	0.75	No	No
103493_at	Nmb	neuromedin B	0.9456	0.3623	0.0785	0.246	No	0.9541	0.258	0.363	0.54	No	No
103494_at	Tspan8	tetraspanin 8	1.0514	0.2138	0.0051	0.3889	No	1.0987	0.245	0.0747	0.991	No	No
103495_at	Etnk1	Ethanolamine kinase 1	0.9463	0.3454	0.1133	0.5705	No	0.8941	0.506	0.728	0.586	No	No
103496_at	Arid3a	AT rich interactive domain 3A (Bright like)	1.1018	0.0742	0.0076	0.563	No	0.9591	0.628	0.9	0.56	No	No
103497_at	BC025546	cDNA sequence BC025546	1.0725	0.213	0.0007	0.068	No	1.095	0.159	0.0022	0.446	No	No
103498_at	Gcgr	glucagon receptor	1.0037	0.94	0.2099	0.7972	No	1.1295	0.0341	0.897	0.0139	No	No
103499_at	Vwf	Von Willebrand factor homolog	0.8598	0.0018	0.0319	0.0064	No	0.859	0.0223	0.702	0.036	No	No
103500_at	Acvr2a	Activin receptor IIA	1.031	0.6317	0.0006	0.4735	No	1.0376	0.775	0.0014	0.857	No	No
103501_at	Pura	purine rich element binding protein A	0.973	0.543	0.0062	0.0315	No	0.9678	0.564	0.0095	0.0295	No	No
103502_at	Nrk	Nik related kinase	0.9054	0.0351	0.0002	0.0055	No	0.954	0.359	0.0011	0.344	No	No
103503_at	Plcg2	phospholipase C, gamma 2	1.0313	0.6352	0.0497	0.3525	No	1.0823	0.279	0.621	0.386	No	No
103504_at	Ssbp2	single-stranded DNA binding protein 2	0.9439	0.4398	0.0821	0.1554	No	0.9434	0.448	0.0918	0.229	No	No
103506_f_at	Dsc2	desmocollin 2	0.9758	0.7608	0.553	0.4083	No	0.9721	0.703	0.502	0.388	No	No
103507_at	Emr1	EGF-like module containing, mucin-like, hormone receptor-like sequence 1	0.9264	0.0146	0.0555	0.3429	No	0.9479	0.352	0.548	0.768	No	No
103508_at	Pcgf5	polycomb group ring finger 5	0.9339	0.2799	0.0062	0.0636	No	1.0225	0.689	0.0001	0.464	No	No
103509_at	Tnfrsf9	tumor necrosis factor receptor superfamily, member 9	1.0273	0.7054	0.056	0.6836	No	0.9812	0.72	0.0939	0.247	No	No
103510_at	Slc6a12	solute carrier family 6 (neurotransmitter transporter, betaine/GABA), member 12	0.9675	0.3107	0.7654	0.8547	No	0.9229	0.0748	0.0042	0.0991	No	No
103511_at	Arhgap8	Rho GTPase activating protein 8	1.0918	0.0066	0.0001	0.9402	Yes	1.0125	0.705	0.0042	0.678	No	No
103512_at	Bzrpl1	benzodiazapine receptor, peripheral-like 1	1.0034	0.9451	0.8414	0.9579	No	1.0117	0.672	0.595	0.134	No	No
103513_at	Wnt5b	wingless-related MMTV integration site 5B	1.0302	0.2202	0.0003	0.0253	No	1.01	0.497	0.0003	0.0608	No	No
103514_at	Tnfrsf21	tumor necrosis factor receptor superfamily, member 21	1.0582	0.3162	0.0632	0.5661	No	1.0873	0.0022	0.0005	0.0108	Yes	No
103515_at	Cant1	calcium activated nucleotidase 1	0.9569	0.0474	0.0012	0.0785	No	1.0342	0.322	0.0041	0.0428	No	No
103516_at	Celsr1	cadherin EGF LAG seven-pass G-type receptor 1	1.0256	0.5733	0.0899	0.7697	No	1.0562	0.667	0.0062	0.0614	No	No
103517_at	A530040E14 Rik	RIKEN cDNA A530040E14 gene	1.0369	0.335	0.0073	0.0885	No	1.1367	0.0058	0.0306	0.137	Yes	No
103518_at	Ctla2b	cytotoxic T lymphocyte-associated protein 2 beta	1.0376	0.5678	0.0359	0.7506	No	0.9535	0.578	0.584	0.207	No	No
103519_at	Ces1	carboxylesterase 1	0.9948	0.9323	0.2241	0.1043	No	1.0273	0.7	0.249	0.948	No	No
103520_at	Vegfa	vascular endothelial growth factor A	1.0183	0.5326	0.9262	0.262	No	1.1346	0.0853	0.579	0.601	No	No
103521_r_at	Mpp7	membrane protein, palmitoylated 7 (MAGUK p55 subfamily member 7)	0.9563	0.4751	0.0338	0.1691	No	1.1369	0.0556	0.462	0.065	No	No
103522_at	Sgcd	sarcoglycan, delta (dystrophin-associated glycoprotein)	0.9198	0.2008	0.1211	0.7094	No	0.8675	0.101	0.412	0.859	No	No
103523_at	Lenq8	leukocyte receptor cluster (LRC) member 8	1.0215	0.6096	0.0232	0.0415	No	1.0529	0.0999	0.0006	0.131	No	No
103524_at	Cdan1	congenital dyserythropoietic anemia, type I (human)	1.015	0.2456	0.0006	0.5953	No	0.9236	0.17	0.0145	0.29	No	No
103525_at	Hnrpll	heterogeneous nuclear ribonucleoprotein L-like	0.9554	0.0247	0.0001	0.001	No	0.9205	0.238	0.0248	0.549	No	No
103526_at	Padi2 /// LOC638935	peptidyl arginine deiminase, type II /// similar to peptidyl arginine deiminase, type II	1.0191	0.4327	0.0004	0.3277	No	1.041	0.389	0.005	0.884	No	No
103527_at	Slc35e4	solute carrier family 35, member E4	0.9435	0.0905	0.5802	0.0244	No	0.9418	0.21	0.0722	0.363	No	No
103529_at	Tnfaip8l1	tumor necrosis factor, alpha-induced protein 8-like 1	1.0294	0.5869	0.5116	0.6506	No	1.1128	0.247	0.963	0.0681	No	No
103530_at	Fancg	Fanconi anemia, complementation group G	0.9906	0.6322	0.002	0.146	No	0.978	0.513	0.0023	0.716	No	No
103531_f_at	Ero1lb	ERO1-like beta (S. cerevisiae)	1.0301	0.55	0.2896	0.4353	No	1.0416	0.431	0.136	0.469	No	No
103532_at	Eomes	eomesodermin homolog (Xenopus laevis)	1.0436	0.0486	0.2939	0.0185	No	1.2642	0.0503	0.0248	0.932	No	No
103533_at	Hbb-bh1	hemoglobin Z, beta-like embryonic chain	1.0511	0.095	0.0007	0.1196	No	0.977	0.694	0.0189	0.968	No	No
103534_at	Hbb-b2	hemoglobin, beta adult minor chain	0.9479	0.2112	0.0003	0.4988	No	0.9637	0.321	0.0009	0.979	No	No
103535_at	Hbb-y	hemoglobin Y, beta-like embryonic chain	1.0522	0.2755	0.1408	0.9159	No	1.0394	0.362	0.0469	0.291	No	No
103536_at	Tmeff2	transmembrane protein with EGF-like and two follistatin-like domains 2	0.974	0.7546	0.032	0.4157	No	0.943	0.284	0.0027	0.124	No	No
103537_at	Eif2ak3	eukaryotic translation initiation factor 2 alpha kinase 3	1.0192	0.74	0.5851	0.5784	No	1.0562	0.43	0.706	0.504	No	No
103538_at	Tbx3	T-box 3	0.9551	0.5439	0.0213	0.4308	No	0.9588	0.559	0.0015	0.0875	No	No
103539_at	Tec	cytoplasmic tyrosine kinase, Dscr28C related (Drosophila)	1.0469	0.1611	0.0015	0.0453	No	0.9805	0.485	0.568	0.634	No	No
103540_at	---	---	1.0094	0.8615	0.2894	0.3396	No	1.0863	0.211	0.195	0.346	No	No
103541_at	Tcte2	t-complex-associated testis expressed 2	0.9583	0.4204	0.2148	0.6107	No	0.9687	0.388	0.0623	0.0238	No	No
103542_at	Tnks1bp1	tankyrase 1 binding protein 1	0.8851	0.0637	0.5249	0.7834	No	0.9569	0.0546	0.01	0.0565	No	No
103543_at	Sez6l	seizure related 6 homolog like	0.9833	0.4373	0.0273	0.2309	No	0.9	0.317	0.459	0.456	No	No
103544_at	Pus1	pseudouridine synthase 1	1.0378	0.4838	0.0391	0.9756	No	1.0015	0.97	0.118	0.746	No	No
103545_at	2610019E17 Rik	RIKEN cDNA 2610019E17 gene	1.0293	0.733	0.0621	0.9691	No	1.0291	0.639	0.034	0.704	No	No
103546_at	Fosl2 /// LOC634417	fos-like antigen 2 /// similar to fos-like antigen 2	1.03	0.7386	0.0722	0.4466	No	1.6637	0.0037	0.0002	0.258	Yes	No
103547_at	Slc41a1	solute carrier family 41, member 1	0.9073	0.199	0.0938	0.2078	No	1.0365	0.527	0.248	0.246	No	No
103548_at	Tac2	tachykinin 2	1.0291	0.6513	0.3241	0.1778	No	0.9867	0.907	0.418	0.541	No	No
103549_at	Nes	nestin	0.9958	0.9603	0.0006	0.0457	No	0.9563	0.197	0.0072	0.729	No	No
103550_at	Ednrb	endothelin receptor type B	1.0322	0.1563	0	0.0399	No	1.0574	0.229	0	0.0281	No	No
103551_at	Plekha5	pleckstrin homology domain containing, family A member 5	1.063	0.3677	0.0429	0.3431	No	1.0254	0.643	0.0452	0.414	No	No
103552_at	Rabep1	rabaptin, RAB GTPase binding effector protein 1	0.9161	0.2964	0.06	0.7875	No	1.1298	0.0578	0.301	0.0257	No	No
103553_at	Mcm10	minichromosome maintenance deficient 10 (S. cerevisiae)	1.0563	0.1331	0.2186	0.3708	No	1.059	0.51	0.0744	0.642	No	No
103554_at	Adam19	a disintegrin and metallopeptidase domain 19 (meltrin beta)	0.9404	0.4969	0.0204	0.4506	No	0.8611	0.0024	0.025	0.0082	No	No
103555_at	Chmp4b	chromatin modifying protein 4B	1.1359	0.0117	0.0017	0.2165	No	1.1217	0.0257	0.0246	0.271	No	No
103556_at	---	---	1.01	0.5858	0.0005	0.5776	No	1.0177	0.549	0.0131	0.179	No	No
103557_at	Vps37c	vacuolar protein sorting 37C (yeast)	1.0265	0.4416	0.0033	0.9089	No	0.9993	0.971	0.016	0.828	No	No
103558_at	Krt1-1	keratin complex 1, acidic, gene 1	1.0348	0.6735	0.4541	0.7351	No	0.9896	0.825	0.807	0.919	No	No
103559_at	Prkaca	protein kinase, cAMP dependent, catalytic, alpha	0.8862	0.1793	0.3742	0.8941	No	0.9208	0.237	0.0857	0.522	No	No
103560_at	Lysmd2	LysM, putative peptidoglycan-binding, domain containing 2	1.0303	0.3614	0.0027	0.9086	No	1.0374	0.178	0.0006	0.242	No	No

103562_f_at	1300007C21 Rik /// LOC433762 /// LOC666026 /// LOC668280 /// LOC668462 /// LOC669505 /// LOC672861	RIKEN cDNA 1300007C21 gene /// similar to Retrovirus-related POL polyprotein (Endonuclease) /// hypothetical protein LOC666026 /// hypothetical protein LOC668280 /// similar to RIKEN cDNA 1300007C21 gene /// hypothetical protein LOC669505 /// hypothetical protein LOC672861	0.9659	0.4796	0.0132	0.3032	No	0.9803	0.31	0.0014	0.145	No	No
103563_at	Ibrdc3	IBR domain containing 3	1.0273	0.3531	0.0055	0.2875	No	0.9848	0.563	0.0133	0.13	No	No
103564_at	Uvrag	UV radiation resistance associated gene	0.9568	0.2084	0.0067	0.7961	No	0.9503	0.387	0.538	0.816	No	No
103565_at	1810009A15 Rik	RIKEN cDNA 1810009A15 gene	1.0161	0.3983	0.0002	0.0093	No	0.9846	0.483	0.0001	0.0897	No	No
103567_at	Frvl	furry homolog-like (Drosophila)	0.9969	0.9191	0.0003	0.3486	No	0.9444	0.216	0.0017	0.485	No	No
103568_at	Srpx	sushi-repeat-containing protein	0.9167	0.1537	0.0182	0.6201	No	0.973	0.506	0.0005	0.886	No	No
103569_at	Sh3glb1	SH3-domain GRB2-like B1 (endophilin)	0.9705	0.5272	0.6759	0.2358	No	0.988	0.761	0.037	0.74	No	No
103570_at	C1qtnf3	C1q and tumor necrosis factor related protein 3	1.052	0.5424	0.0754	0.6123	No	1.0615	0.567	0.0941	0.604	No	No
103571_at	Lst1	leukocyte specific transcript 1	0.983	0.327	0.0045	0.0092	No	0.8978	0.0669	0.653	0.0085	No	No
103573_at	Pip5k1a	phosphatidylinositol-4-phosphate 5-kinase, type 1 alpha	1.1122	0.0016	0.5091	0.0024	No	1.0675	0.0607	0.214	0.02	No	No
103574_at	Ablim1	actin-binding LIM protein 1	0.9182	0.3559	0.0324	0.5271	No	0.9908	0.667	0.0091	0.407	No	No
103575_at	---	---	1.124	0.0067	0.0002	0.1465	Yes	1.1083	0.0307	0.0024	0.0704	No	Yes
103577_at	Pfkfb3	6-phosphofructo-2-kinase/fructose-2,6-biphosphatase 3	1.1092	0.078	0.0143	0.256	No	0.9252	0.174	0.0109	0.417	No	No
103578_at	Tom1 /// LOC545878	target of myb1 homolog (chicken) /// similar to TOM1	0.9921	0.8406	0.2244	0.8354	No	1.0601	0.0236	0.0172	0.031	No	No
103579_at	Rac2	RAS-related C3 botulinum substrate 2	0.942	0.0909	0.2399	0.6983	No	1.0214	0.337	0.118	0.0573	No	No
103580_at	BC013529	cDNA sequence BC013529	0.9447	0.1114	0.0937	0.3716	No	0.9417	0.0177	0.0007	0.496	No	No
103581_at	Acot1	acyl-CoA thioesterase 1	0.8622	0.0177	0.0892	0.0882	No	0.8629	0.0117	0.318	0.116	No	No
103582_r_at	Ube2w	ubiquitin-conjugating enzyme E2W (putative)	1.0266	0.5253	0.0331	0.5271	No	1.3477	0.0991	0.0074	0.348	No	No
103584_at	5830471E12 Rik	RIKEN cDNA 5830471E12 gene	0.951	0.3108	0.0028	0.5852	No	0.9665	0.451	0.0048	0.787	No	No
103585_at	Spast	spastin	0.9666	0.6495	0.0047	0.5868	No	1.0597	0.722	0.0067	0.821	No	No
103588_at	Adam15	a disintegrin and metallopeptidase domain 15 (metargidin)	0.9105	0.0471	0.1836	0.2442	No	0.9473	0.0929	0.092	0.265	No	No
103589_at	Krt1-16	keratin complex 1, acidic, gene 16	0.9346	0.3042	0.1796	0.0593	No	0.9824	0.887	0.0558	0.889	No	No
103590_at	Ggcx	gamma-glutamyl carboxylase	1.0262	0.254	0.0023	0.2888	No	0.9611	0.263	0.0073	0.766	No	No
103591_at	Taf6	TAF6 RNA polymerase II, TATA box binding protein (TBP)-associated factor	0.9318	0.2642	0.0542	0.5547	No	0.989	0.83	0.0014	0.768	No	No
103592_at	Map2k5	mitogen activated protein kinase kinase 5	0.9251	0.1059	0.3389	0.2399	No	0.9329	0.17	0.0199	0.837	No	No
103593_at	Nppa	natriuretic peptide precursor type A	0.9873	0.778	0.1934	0.3654	No	1.01	0.848	0.27	0.769	No	No
103594_at	Fbxl15	F-box and leucine-rich repeat protein 15	1.0064	0.9325	0.1551	0.3678	No	1.0524	0.396	0.0318	0.889	No	No
103595_at	Zfp524	zinc finger protein 524	0.9057	0.2378	0.01	0.6057	No	1.007	0.825	0.0019	0.218	No	No
103596_at	Dgka	diacylglycerol kinase, alpha	0.9522	0.1605	0.052	0.0236	No	0.9133	0.0401	0.119	0.0017	No	No
103597_at	Dct	dopachrome tautomerase	0.9818	0.2573	0.2001	0.0144	No	1.0596	0.154	0.0077	0.0525	No	No
103598_at	Dhx9	DEAH (Asp-Glu-Ala-His) box polypeptide 9	1.0179	0.7829	0.0203	0.9246	No	1.1488	0.202	0.23	0.0588	No	No
103599_at	Mdm1	transformed mouse 3T3 cell double minute 1	1.1414	0.0129	0.1847	0.4679	No	1.2317	0.0512	0.219	0.0838	No	No
103600_at	Epb4.9	erythrocyte protein band 4.9	0.9662	0.0996	0.8742	0.4141	No	0.9446	0.0836	0.0061	0.574	No	No
103601_at	Ptrh1	peptidyl-tRNA hydrolase 1 homolog (S. cerevisiae)	1.0419	0.2321	0.07	0.0434	No	1.0918	0.245	0.602	0.0105	No	No
103602_at	Dao1	D-amino acid oxidase 1	0.9565	0.3368	0.0515	0.3854	No	0.8696	0.0031	0.0024	0.0044	No	No
103603_at	Eftud1	elongation factor Tu GTP binding domain containing 1	0.9003	0.0733	0.0963	0.4601	No	0.9393	0.255	0.144	0.523	No	No
103604_at	Rgs19	regulator of G-protein signaling 19	0.9995	0.9926	0.063	0.2338	No	0.9414	0.339	0.0828	0.687	No	No
103605_g_at	Rgs19	regulator of G-protein signaling 19	0.9708	0.6158	0.0763	0.108	No	1.1902	0.105	0.0534	0.695	No	No
103606_r_at	Rgs19	regulator of G-protein signaling 19	1.079	0.0169	0.0317	0.3081	No	1.071	0.077	0.107	0.113	No	No
103607_at	Gpx7	glutathione peroxidase 7	0.9396	0.2016	0.0081	0.3626	No	0.9772	0.606	0.0012	0.557	No	No
103608_at	Fars2	phenylalanine-tRNA synthetase 2 (mitochondrial)	0.9637	0.1759	0.3138	0.0475	No	0.9653	0.314	0.793	0.325	No	No
103609_at	Cryga	crystallin, gamma A	1.0713	0.276	0.5256	0.0548	No	1.0573	0.134	0.0095	0.0197	No	No
103610_at	Pde4b	phosphodiesterase 4B, cAMP specific	1.0401	0.4888	0.0975	0.5977	No	1.0672	0.22	0.0152	0.304	No	No
103611_at	Cd47	CD47 antigen (Rh-related antigen, integrin-associated signal transducer)	1.0782	0.3439	0.0612	0.8167	No	1.1818	0.103	0.584	0.956	No	No
103612_at	Aqp2	aquaporin 2	1.0313	0.2227	0.0007	0.5201	No	0.9117	0.154	0.285	0.0623	No	No
103613_at	Aldoa-ps1	aldolase 1, A isoform, pseudogene 1	0.9338	0.1681	0.0019	0.1015	No	0.9703	0.39	0.0014	0.489	No	No
103614_at	Nfkb2	nuclear factor of kappa light polypeptide gene enhancer in B-cells 2, p49/p100	0.9828	0.6518	0.0024	0.6139	No	1.0536	0.522	0.0052	0.332	No	No
103615_at	AI447904 /// BC094916	expressed sequence AI447904 /// cDNA sequence BC094916	1.0022	0.9733	0.2662	0.9474	No	0.9762	0.589	0.0233	0.421	No	No
103616_at	Col11a2	procollagen, type XI, alpha 2	0.9072	0.0217	0	0.8167	No	0.9166	0.0874	0.0001	0.932	No	No
103617_at	Cd55	CD55 antigen	1.0146	0.6028	0.2306	0.2909	No	1.0061	0.911	0.8	0.297	No	No
103618_at	Ckmt2	creatine kinase, mitochondrial 2	0.7175	0	0	0	No	0.902	0.0095	0.988	0.004	No	No
103619_at	Cyb5b	cytochrome b5 type B	1.0663	0.353	0.0445	0.1818	No	1.0359	0.278	0.0038	0.392	No	No
103620_s_at	Smn1	survival motor neuron 1	0.9998	0.996	0.5369	0.5284	No	1.0352	0.463	0.51	0.646	No	No
103622_at	Mlycd	malonyl-CoA decarboxylase	1.013	0.7349	0.2734	0.7474	No	0.9855	0.638	0.618	0.439	No	No
103623_at	Fbn2	fibrillin 2	0.8561	0.0035	0.0001	0.0484	Yes	0.908	0.0981	0	0.192	No	No
103624_at	2900073H19 Rik	RIKEN cDNA 2900073H19 gene	0.9155	0.0772	0.7155	0.0392	No	0.9733	0.268	0.482	0.033	No	No
103625_at	Afg3l1	AFG3(ATPase family gene 3)-like 1 (yeast)	0.9294	0.0552	0.6864	0.5685	No	0.9609	0.0418	0.928	0.11	No	No
103628_at	---	---	1.0549	0.0988	0.0069	0.0135	No	1.0917	0.0196	0.0577	0.0183	No	No
103629_g_at	Lef1	lymphoid enhancer binding factor 1	0.967	0.5069	0.0729	0.1812	No	1.0417	0.726	0.248	0.353	No	No
103630_at	Lars	leucyl-tRNA synthetase	0.9286	0.0637	0.2866	0.6784	No	0.9207	0.0143	0.0778	0.5	No	No
103631_at	Zfp688	zinc finger protein 688	1.0385	0.3057	0.0036	0.3592	No	0.9758	0.526	0.0016	0.303	No	No
103632_at	Hmgb4	high-mobility group box 4	0.88	0.1389	0.1023	0.6984	No	0.9156	0.397	0.0265	0.476	No	No
103634_at	Isgf3g	interferon dependent positive acting transcription factor 3 gamma	1.0734	0.1559	0.0061	0.9651	No	0.9496	0.559	0.312	0.575	No	No
103635_at	Tgds	TDP-glucose 4,6-dehydratase	1.0333	0.1071	0.0108	0.0016	No	1.0704	0.158	0.918	0.765	No	No
103636_at	Pnkd	paroxysmal nonkinesiogenic dyskinesia	1.022	0.5565	0.0593	0.2599	No	1.0282	0.328	0.012	0.368	No	No
103637_at	Naga	N-acetyl galactosaminidase, alpha	0.989	0.3076	0.0021	0.0412	No	0.8461	0.0032	0.0314	0.0012	No	No
103638_at	Fnbp1	formin binding protein 1	1.0488	0.406	0.0034	0.5445	No	1.2243	0.0139	0.167	0.0225	No	No
103639_at	Ifit2	interferon-induced protein with tetratricopeptide repeats 2	0.9416	0.3226	0.2277	0.0493	No	0.9533	0.531	0.654	0.177	No	No
103641_at	BC030183	cDNA sequence BC030183	0.9433	0.376	0.2606	0.841	No	0.9713	0.585	0.135	0.429	No	No
103642_at	RP23-336J1.4	Ras-GTPase-activating protein SH3-domain binding protein	1.0721	0.1521	0.0181	0.2096	No	1.0557	0.175	0.0079	0.0426	No	No
103643_at	Zdhhc16	zinc finger, DHHC domain containing 16	0.9828	0.5407	0.0126	0.0184	No	1.0248	0.297	0.0266	0.168	No	No
103644_at	Dpep1	dipeptidase 1 (renal)	0.7967	0.0025	0.0391	0.4816	Yes	0.781	0.0004	0.0268	0.968	Yes	Yes
103645_at	Dnajb8	DnaJ (Hsp40) homolog, subfamily B, member 8	1.0869	0.0423	0.4957	0.0134	No	1.0856	0.163	0.0722	0.504	No	No
103646_at	Crat	carnitine acetyltransferase	0.9503	0.3516	0.9164	0.2092	No	1.1142	0.0458	0.0207	0.6	No	No
103647_at	Glb1	galactosidase, beta 1	0.9719	0.526	0.0301	0.1448	No	0.9332	0.179	0.0653	0.054	No	No
103648_at	Tacstd2	tumor-associated calcium signal transducer 2	0.9519	0.0951	0.002	0.0033	No	1.1335	0.0052	0.0052	0.31	Yes	No
103649_at	Hao3	hydroxyacid oxidase (glycolate oxidase) 3	1.0158	0.5898	0.003	0.7908	No	0.9679	0.729	0.0035	0.742	No	No
103650_at	Vps53	vacuolar protein sorting 53 (yeast)	0.9784	0.6138	0.467	0.5258	No	0.9781	0.465	0.57	0.886	No	No
103651_r_at	Gtf2f2	general transcription factor IIF, polypeptide 2	1.0004	0.9904	0.944	0.8032	No	1.0368	0.459	0.214	0.852	No	No
103653_at	Mras	muscle and microspikes RAS	0.914	0.0084	0.0024	0.4273	Yes	1.1296	0.174	0.191	0.0245	No	No
103654_at	Nsbp1	nucleosome binding protein 1	1.0068	0.8307	0.0159	0.9933	No	0.9695	0.453	0.155	0.275	No	No
103655_at	Hdac3	histone deacetylase 3	1.0929	0.094	0.018	0.1195	No	1.0168	0.582	0.167	0.101	No	No
103656_at	Lancl1	LanC (bacterial lantibiotic synthetase component C)-like 1	1.0365	0.0869	0.0609	0.3024	No	1.0282	0.54	0.23	0.124	No	No
103657_i_at	Mtf2	metal response element binding transcription factor 2	0.9997	0.9858	0	0.0027	No	1.0664	0.097	0.0015	0.0332	No	No
103658_r_at	Mtf2	metal response element binding transcription factor 2	1.0328	0.6993	0.1226	0.2242	No	0.9879	0.906	0.0176	0.445	No	No

103660_at	Pex11a	peroxisomal biogenesis factor 11a	0.957	0.3953	0.0076	0.9322	No	1.0202	0.621	0.008	0.406	No	No
103662_at	Ncf4	neutrophil cytosolic factor 4	0.9267	0.0645	0.0116	0.1989	No	0.9712	0.783	0.707	0.252	No	No
103663_at	Pomgnt1	protein O-linked mannose beta1,2-N-acetylglucosaminyltransferase	1.0366	0.4526	0.0644	0.0216	No	0.997	0.89	0.0514	0.0418	No	No
103664_r_at	2810452K22 Rik	RIKEN cDNA 2810452K22 gene	0.8755	0.1372	0.5561	0.1431	No	0.9621	0.321	0.0384	0.753	No	No
103665_at	Elovl6	ELOVL family member 6, elongation of long chain fatty acids (yeast)	1.0335	0.1694	0.468	0.4691	No	1.079	0.1	0.0275	0.681	No	No
103666_at	Hoxb5	homeo box B5	1.0123	0.6638	0.5458	0.2272	No	1.0408	0.315	0.194	0.767	No	No
103667_at	Atp13a3	ATPase type 13A3	0.9159	0.4531	0.0067	0.2383	No	0.8592	0.206	0.0045	0.489	No	No
103668_at	Supt6h	suppressor of Ty 6 homolog (S. cerevisiae)	1.0042	0.9502	0.072	0.5673	No	1.0151	0.432	0.0103	0.0567	No	No
103669_at	E330034G19Rik	RIKEN cDNA E330034G19 gene	1.0482	0.4408	0.3305	0.4789	No	1.0648	0.153	0.0299	0.179	No	No
103670_at	Cyp2a12	cytochrome P450, family 2, subfamily a, polypeptide 12	1.0617	0.1505	0.3808	0.0797	No	1.1301	0.0142	0.462	0.0088	No	No
103671_at	Htatip2	HIV-1 tat interactive protein 2, homolog (human)	0.9959	0.9496	0.6234	0.1916	No	0.9706	0.169	0.481	0.0208	No	No
103672_at	Phf23	PHD finger protein 23	0.9811	0.6018	0.2987	0.0754	No	0.9398	0.228	0.579	0.0259	No	No
103673_at	C2	complement component 2 (within H-2S)	0.9688	0.5736	0.3142	0.9394	No	0.9911	0.915	0.0239	0.863	No	No
103674_f_at	Eif2s3y	eukaryotic translation initiation factor 2, subunit 3, structural gene Y-linked	0.8577	0.0444	0.0018	0.0099	No	0.9285	0.0911	0.0022	0.0075	No	No
103675_at	Robo1	roundabout homolog 1 (Drosophila)	1.0089	0.8574	0.0046	0.3117	No	0.9463	0.657	0.0019	0.414	No	No
103676_at	C1qtnf1	C1q and tumor necrosis factor related protein 1	0.9441	0.3848	0.0884	0.2788	No	0.9565	0.307	0.0558	0.163	No	No
103678_at	Sbno1	sno, strawberry notch homolog 1 (Drosophila)	1.0197	0.5641	0.0047	0.8978	No	1.0505	0.225	0.702	0.411	No	No
103679_at	Cdc23	CDC23 (cell division cycle 23, yeast, homolog)	0.9732	0.6826	0.2874	0.4912	No	1.097	0.178	0.042	0.587	No	No
103680_at	Srpr	signal recognition particle receptor ('docking protein')	1.0411	0.3778	0.1157	0.3015	No	1.0114	0.756	0.0638	0.161	No	No
103681_at	Atp6v0a2	ATPase, H+ transporting, lysosomal V0 subunit A2	1.0059	0.8459	0.0006	0.5938	No	0.9657	0.358	0.0017	0.134	No	No
103682_at	C80913	expressed sequence C80913	0.9513	0.3601	0.0153	0.6371	No	0.9727	0.716	0.008	0.818	No	No
103683_at	Dhodh	dihydroorotate dehydrogenase	0.9966	0.8423	0.0075	0.0011	No	1.0072	0.747	0.239	0.052	No	No
103684_at	Tekt2	tektin 2	0.9908	0.7843	0.0054	0.8688	No	0.9507	0.0629	0.01	0.595	No	No
103685_at	---	Transcribed locus	0.9758	0.4814	0	0.2233	No	1.0232	0.632	0.0002	0.754	No	No
103686_at	Mcoln2	mucolipin 2	0.9335	0.0963	0.1091	0.1136	No	1.0183	0.639	0.194	0.135	No	No
103688_at	Ankrd43	ankyrin repeat domain 43	0.9845	0.7183	0.0004	0.2069	No	1.0579	0.0592	0	0.0666	No	No
103689_at	Abcc3	ATP-binding cassette, sub-family C (CFTR/MRP), member 3	1.0147	0.4584	0.3688	0.0217	No	0.9891	0.708	0.0508	0.448	No	No
103690_at	Lat2	linker for activation of T cells family, member 2	1.0479	0.5085	0.1196	0.1707	No	1.1112	0.161	0.0122	0.284	No	No
103691_at	Trim13	tripartite motif protein 13	0.9706	0.3416	0.0233	0.226	No	1.0187	0.672	0.0564	0.969	No	No
103692_at	Efnb3	ephrin B3	1.0069	0.7881	0.0028	0.3918	No	1.0473	0.005	0.0001	0.695	Yes	No
103693_at	Tirap	toll-interleukin 1 receptor (TIR) domain-containing adaptor protein	0.935	0.3469	0.3052	0.4957	No	1.0887	0.137	0.186	0.899	No	No
103694_at	Nup188	nucleoporin 188	0.9593	0.3058	0.0171	0.95	No	0.946	0.2	0.183	0.337	No	No
103695_f_at	C330007P06 Rik	RIKEN cDNA C330007P06 gene	1.0981	0.126	0.001	0.2928	No	1.134	0.097	0.0015	0.215	No	No
103696_r_at	C330007P06 Rik	RIKEN cDNA C330007P06 gene	1.0936	0.0492	0.0011	0.1183	No	1.2568	0.112	0.0118	0.0983	No	No
103697_at	AW061234	expressed sequence AW061234	0.9492	0.1468	0	0.0761	No	0.9814	0.762	0	0.461	No	No
103699_i_at	Frat2	frequently rearranged in advanced T-cell lymphomas 2	1.0501	0.0716	0.0003	0.0273	No	1.0371	0.348	0.0056	0.185	No	No
103700_r_at	Frat2	Frequently rearranged in advanced T-cell lymphomas 2	1.0713	0.0587	0.4527	0.0668	No	1.0775	0.344	0.155	0.195	No	No
103701_at	Ccdc52	coiled-coil domain containing 52	0.9591	0.0151	0.0758	0.0064	No	0.961	0.537	0.226	0.487	No	No
103702_i_at	LOC623195	similar to integral membrane transport protein UST1R	1.1082	0.3442	0.1543	0.0856	No	1.2252	0.184	0.0742	0.0992	No	No
103703_f_at	C730048C13Rik /// LOC434674 /// LOC623195 /// LOC674021	RIKEN cDNA C730048C13 gene /// similar to integral membrane transport protein UST1R /// similar to integral membrane transport protein UST1R /// similar to integral membrane transport protein UST1R	1.0851	0.0946	0.1596	0.2785	No	1.0004	0.935	0.172	0.0729	No	No
103704_at	Ddhd2	DDHD domain containing 2	0.9391	0.1584	0.0084	0.8595	No	0.9267	0.055	0.0024	0.184	No	No
103706_at	Sfxn2 /// LOC677143	sideroflexin 2 /// similar to Sideroflexin-2	0.9423	0.1111	0.0153	0.1842	No	0.9152	0.142	0.0051	0.671	No	No
103707_at	C3ar1	complement component 3a receptor 1	1.0272	0.4235	0.0018	0.3101	No	1.0464	0.187	0.369	0.361	No	No
103708_at	Eif1a	eukaryotic translation initiation factor 1A	0.9413	0.1753	0.3209	0.8138	No	1.022	0.702	0.0058	0.758	No	No
103709_at	Col1a1	procollagen, type I, alpha 1	0.7665	0.0549	0.0081	0.0332	No	0.7171	0.0932	0.0374	0.0444	No	No
103710_at	MGC107415	hypothetical protein LOC383216	1.0076	0.9363	0.4264	0.935	No	1.0098	0.856	0.0002	0.791	No	No
103711_at	Casp9	caspase 9	0.9633	0.4741	0.018	0.4996	No	1.0182	0.623	0.0019	0.996	No	No
103712_at	R3hdm1	R3H domain 1 (binds single-stranded nucleic acids)	0.98	0.7142	0.0024	0.3707	No	0.9907	0.82	0.001	0.0946	No	No
103713_at	Usp9x	ubiquitin specific peptidase 9, X chromosome	0.9839	0.8396	0.0022	0.1553	No	0.9848	0.689	0.0013	0.667	No	No
103714_at	0610009A07 Rik	RIKEN cDNA 0610009A07 gene	0.9479	0.2946	0.1987	0.5089	No	0.9596	0.451	0.38	0.966	No	No
103715_at	Scin	scinderin	0.9472	0.1556	0.0001	0.051	No	0.9303	0.384	0.0005	0.0581	No	No
103716_at	Cep350	centrosomal protein 350	0.9591	0.5793	0.013	0.369	No	0.9679	0.694	0.0598	0.184	No	No
103717_at	Wwp2	WW domain containing E3 ubiquitin protein ligase 2	0.9481	0.0448	0.0001	0.2181	No	1.026	0.311	0.001	0.292	No	No
103718_at	Pcgf2	polycomb group ring finger 2	1.0307	0.2334	0.0022	0.389	No	1.2577	0.046	0.0012	0.111	No	No
103719_at	Msh5	mutS homolog 5 (E. coli)	1.0093	0.8791	0.0674	0.263	No	1.061	0.179	0.0175	0.0339	No	No
103720_at	Rest	RE1-silencing transcription factor	1.0273	0.6858	0.0004	0.2742	No	1.1029	0.0181	0.0001	0.0299	No	No
103721_at	Npnt	Nephronectin	0.8575	0.0086	0.8808	0.2853	Yes	0.8901	0.151	0.25	0.989	No	No
103723_at	Il13ra1	interleukin 13 receptor, alpha 1	0.9196	0.0918	0.13	0.4531	No	0.9528	0.136	0.898	0.163	No	No
103726_at	Yipf5	Yip1 domain family, member 5	1.0603	0.3479	0.0044	0.7555	No	1.0535	0.493	0.0333	0.509	No	No
103727_at	Hrb	HIV-1 Rev binding protein	1.0068	0.9242	0.0069	0.9159	No	1.0591	0.0812	0.001	0.861	No	No
103728_at	---	Transcribed locus	1.0283	0.4423	0.8749	0.5551	No	0.9411	0.504	0.852	0.499	No	No
103729_at	Lama1	laminin, alpha 1	0.8741	0.0455	0.5289	0.8938	No	0.9332	0.337	0.0963	0.443	No	No
103730_at	Col22a1	collagen, type XXII, alpha 1	0.9502	0.5315	0.1578	0.8454	No	1.1808	0.138	0.0765	0.0759	No	No
103731_at	Tsc1	tuberous sclerosis 1	0.984	0.548	0.1388	0.3303	No	0.9829	0.588	0.522	0.0418	No	No
103732_at	Pib5pa	phosphatidylinositol (4,5) bisphosphate 5-phosphatase, A	1.0376	0.3936	0.065	0.9079	No	0.9962	0.761	0.0031	0.14	No	No
103733_at	2900006A08 Rik	RIKEN cDNA 2900006A08 gene	1.0039	0.9062	0.2668	0.1571	No	1.059	0.366	0.33	0.235	No	No
103734_at	Ahi1	Abelson helper integration site	1.0432	0.6329	0.0179	0.5832	No	0.9016	0.0874	0.555	0.118	No	No
103735_at	Wnt6	wingless-related MMTV integration site 6	0.9199	0.0495	0.0535	0.013	No	0.9233	0.144	0.159	0.98	No	No
103736_at	Sash1	SAM and SH3 domain containing 1	1.039	0.4496	0.0008	0.178	No	0.8827	0.127	0.0001	0.0435	No	No
103737_at	Gfpt2	glutamine fructose-6-phosphate transaminase 2	0.9723	0.5341	0.07	0.1172	No	0.9637	0.318	0.137	0.107	No	No
103738_at	BC037034	cDNA sequence BC037034	0.9593	0.5443	0.4015	0.3592	No	1.0215	0.634	0.0578	0.264	No	No
103739_at	Glce	glucuronyl C5-epimerase	0.9467	0.2314	0.2284	0.5742	No	0.9311	0.206	0.482	0.372	No	No
103741_at	Hoxd10	homeo box D10	0.943	0.4171	0.755	0.8607	No	0.9845	0.718	0.0055	0.411	No	No
103742_at	Unc5c	unc-5 homolog C (C. elegans)	1.0287	0.59	0.0308	0.9578	No	1.0032	0.926	0.0002	0.992	No	No
103743_at	3732412D22 Rik	RIKEN cDNA 3732412D22 gene	0.9222	0.1263	0.0111	0.5687	No	0.9038	0.0375	0.0017	0.682	No	No
103744_at	Sh3bgrl2	SH3 domain binding glutamic acid-rich protein like 2	0.9411	0.1076	0.0021	0.0601	No	0.9677	0.516	0.861	0.515	No	No
103745_at	Snx13	sorting nexin 13	0.9788	0.7756	0.2001	0.2122	No	1.0073	0.773	0.0072	0.405	No	No
103746_at	Suz12	suppressor of zeste 12 homolog (Drosophila)	0.9856	0.8985	0.1444	0.8947	No	1.001	0.992	0.918	0.325	No	No
103748_at	4933407C03 Rik	RIKEN cDNA 4933407C03 gene	1.0194	0.6563	0.0187	0.9459	No	0.9973	0.963	0.0088	0.362	No	No
103751_at	AA409316	expressed sequence AA409316	1.0201	0.5918	0.1477	0.2737	No	1.0324	0.378	0.975	0.212	No	No
103752_r_at	Strn	striatin, calmodulin binding protein	1.0327	0.6316	0.0239	0.756	No	0.9567	0.357	0.0368	0.344	No	No
103753_at	Zzz3	zinc finger, ZZ domain containing 3	1.1264	0.114	0.0003	0.9988	No	1.0403	0.483	0.594	0.257	No	No
103754_at	Ube2d3	ubiquitin-conjugating enzyme E2D 3 (UBC4/5 homolog, yeast)	1.0531	0.4455	0.0616	0.4012	No	0.9436	0.37	0.0072	0.593	No	No
103755_at	Sh3d19	SH3 domain protein D19	0.9499	0.2657	0.8718	0.3222	No	1.0229	0.471	0.203	0.047	No	No
103756_at	BC023829	cDNA sequence BC023829	0.9716	0.0419	0.0003	0.0081	No	0.9865	0.732	0.083	0.183	No	No
103757_at	Mizf	MBD2-interacting zinc finger	1.0434	0.1413	0.1207	0.009	No	1.0568	0.152	0.0065	0.0289	No	No
103759_at	Bst1	bone marrow stromal cell antigen 1	0.957	0.6585	0.9801	0.3763	No	1.0112	0.957	0.602	0.455	No	No
103760_at	0610039J04 Rik	RIKEN cDNA 0610039J04 gene	0.95	0.0699	0.0636	0.0288	No	0.9359	0.162	0.146	0.791	No	No
103761_at	Tcfcp2l1	transcription factor CP2-like 1	0.8958	0.1	0.015	0.1881	No	1.0523	0.399	0.0633	0.0205	No	No
103762_at	Gtf2f1	general transcription factor IIF, polypeptide 1	0.9249	0.0576	0.8903	0.153	No	0.9989	0.944	0.706	0.783	No	No
103763_at	Ash1l	ash1 (absent, small, or homeotic)-like (Drosophila)	1.0212	0.6435	0.0032	0.5242	No	0.9662	0.564	0.0007	0.837	No	No
103765_at	Hkr3	GLI-Kruppel family member HKR3	0.96	0.3769	0.1762	0.1228	No	1.0891	0.0938	0.0041	0.536	No	No

103766_at	Sema5a	sema domain, seven thrombospondin repeats (type 1 and type 1-like), transmembrane domain (TM) and short cytoplasmic domain, (semaphorin) 5A	1.0423	0.1507	0.0003	0.0088	No	1.0823	0.156	0.0019	0.326	No	No
103767_f_at	Rrm2b	ribonucleotide reductase M2 B (TP53 inducible)	0.9728	0.5914	0.2842	0.1467	No	0.9595	0.444	0.686	0.109	No	No
103768_at	Ccdc86	coiled-coil domain containing 86	0.9842	0.7455	0.011	0.3762	No	1.0271	0.644	0.0048	0.748	No	No
103769_at	Il1rap	interleukin 1 receptor accessory protein	1.0073	0.8724	0.1126	0.0801	No	1.2992	0.131	0.0849	0.817	No	No
103770_at	2310014L17 Rik	RIKEN cDNA 2310014L17 gene	1.0056	0.8367	0.0076	0.0425	No	1.0551	0.357	0.0009	0.238	No	No
103771_at	1110061N23 Rik	RIKEN cDNA 1110061N23 gene	1.0415	0.0773	0.7504	0.2954	No	0.9706	0.234	0.896	0.483	No	No
103773_at	F830021D11 Rik /// LOC639135	RIKEN cDNA F830021D11 gene /// similar to UHRF1-binding protein 1 (Ubiquitin-like containing PHD and RING finger domains 1-binding protein 1) (ICBP90-binding protein 1)	1.0403	0.3249	0.0012	0.1932	No	1.0804	0.0791	0.0004	0.958	No	No
103774_at	Ankhd1	ankyrin repeat and KH domain containing 1	1.1128	0.1384	0.0577	0.5197	No	1.1728	0.0024	0.334	0.0087	No	No
103776_at	AI593864	expressed sequence AI593864	1.0085	0.8301	0.0646	0.0983	No	1.0621	0.0028	0.0011	0.0001	No	No
103778_at	C76336	expressed sequence C76336	1.1308	0.0345	0.0896	0.2892	No	1.2755	0.0158	0.0107	0.939	No	No
103779_at	Mkl1	MKL (megakaryoblastic leukemia)/myocardin-like 1	0.9514	0.1869	0.1204	0.1626	No	0.9861	0.714	0.0048	0.292	No	No
103780_at	1700021F05 Rik	RIKEN cDNA 1700021F05 gene	0.8986	0.038	0.3045	0.1326	No	0.9905	0.873	0.0749	0.323	No	No
103781_at	Stx4a	syntaxin 4A (placental)	1.0312	0.4914	0.5978	0.0334	No	1.1041	0.0798	0.0034	0.957	No	No
103782_at	Clcnka	chloride channel Ka	1.0018	0.9656	0.1117	0.0913	No	1.0462	0.431	0.449	0.2	No	No
103783_at	Xpr1	xenotropic and polytropic retrovirus receptor 1	1.0121	0.8138	0.0034	0.953	No	0.9495	0.517	0.4	0.143	No	No
103784_at	Gemin5	gem (nuclear organelle) associated protein 5	0.9293	0.4237	0.7651	0.7668	No	0.9359	0.557	0.0547	0.497	No	No
103786_at	D5Ertd135e	DNA segment, Chr 5, ERATO Doi 135, expressed	1.0212	0.6159	0.4471	0.2133	No	0.9954	0.868	0.0115	0.382	No	No
103787_at	Kcna1	potassium voltage-gated channel, shaker-related subfamily, member 1	1.0562	0.2615	0.0016	0.6538	No	1.0558	0.322	0.0064	0.968	No	No
103789_at	Brd4	bromodomain containing 4	0.8795	0.1357	0.0021	0.0435	No	1.0599	0.274	0.354	0.821	No	No
103790_at	Alg3	asparagine-linked glycosylation 3 homolog (yeast, alpha-1,3-mannosyltransferase)	1.0249	0.2336	0.2584	0.3081	No	1	0.903	0.0248	0.584	No	No
103791_at	Narg1	NMDA receptor-regulated gene 1	1.0686	0.2844	0.0006	0.0867	No	0.9003	0.545	0.0071	0.602	No	No
103792_at	Rab35	RAB35, member RAS oncogene family	0.948	0.3781	0.2548	0.7135	No	0.9517	0.0212	0.0576	0.221	No	No
103793_at	Mvp	major vault protein	1.0285	0.3433	0.0049	0.466	No	1.0208	0.546	0.002	0.263	No	No
103794_i_at	Timd2	T-cell immunoglobulin and mucin domain containing 2	1.0445	0.4564	0.2113	0.1343	No	1.0464	0.47	0.255	0.0861	No	No
103795_f_at	Timd2	T-cell immunoglobulin and mucin domain containing 2	1.0882	0.0527	0.7777	0.166	No	1.1019	0.0415	0.0029	0.228	No	No
103796_at	Apaf1	apoptotic peptidase activating factor 1	0.8768	0.0193	0.0005	0.1881	No	0.8924	0.098	0.0165	0.302	No	No
103797_at	Cdc7	cell division cycle 7 (S. cerevisiae)	1.0447	0.4867	0.0165	0.8177	No	1.0342	0.0317	0.0002	0.0008	No	No
103799_at	Mtmr9	myotubularin related protein 9	1.0472	0.2089	0.6382	0.275	No	1.011	0.836	0.47	0.88	No	No
103800_at	Abcc5	ATP-binding cassette, sub-family C (CFTR/MRP), member 5	0.9536	0.2419	0.1264	0.735	No	0.9637	0.544	0.0344	0.516	No	No
103801_at	Rfxank	regulatory factor X-associated ankyrin-containing protein	0.9577	0.2217	0.0024	0.3581	No	0.9682	0.444	0.0071	0.47	No	No
103803_at	Padi3	peptidyl arginine deiminase, type III	0.9241	0.1152	0.0479	0.2644	No	0.9729	0.522	0.0094	0.37	No	No
103804_at	Reck	reversion-inducing-cysteine-rich protein with kazal motifs	1.0366	0.5457	0.0055	0.9848	No	0.9429	0.0834	0.0307	0.487	No	No
103805_at	Nbn	nibrin	1.0086	0.865	0.5488	0.2395	No	0.9655	0.384	0.282	0.412	No	No
103806_at	Lrp5 /// LOC670589	low density lipoprotein receptor-related protein 5 /// similar to Low-density lipoprotein receptor-related protein 5 precursor (LRP7) (Lr3)	0.9474	0.1309	0.0002	0.2257	No	0.9472	0.0926	0.0001	0.232	No	No
103807_at	Wiz	widely-interspaced zinc finger motifs	0.9698	0.4309	0.1385	0.7099	No	0.9319	0.146	0.0189	0.132	No	No
103808_at	Golga5	golgi autoantigen, golgin subfamily a, 5	0.9905	0.8044	0.4641	0.6307	No	1.0182	0.721	0.136	0.866	No	No
103809_r_at	Dync1i1	dynein cytoplasmic 1 intermediate chain 1	1.0289	0.4245	0.3073	0.0838	No	1.0429	0.18	0.196	0.0096	No	No
103810_at	Trp63	transformation related protein 63	0.9964	0.9191	0.0023	0.2857	No	1.0929	0.0826	0.126	0.129	No	No
103811_at	Invs	inversin	0.9148	0.0118	0.0603	0.4713	No	0.9249	0.181	0.94	0.956	No	No
103812_at	Clca1	chloride channel calcium activated 1	0.9187	0.1303	0.0148	0.2627	No	1.0101	0.813	0.729	0.086	No	No
103813_at	Sh3yl1	Sh3 domain YSC-like 1	0.9343	0.0539	0.56	0.1801	No	0.7888	0.0619	0.733	0.12	No	No
103814_at	Pex11b	peroxisomal biogenesis factor 11b	0.9619	0.4516	0.3987	0.6974	No	1.0179	0.363	0.0017	0.351	No	No
103815_at	Pex11b	peroxisomal biogenesis factor 11b	1.0518	0.3883	0.2017	0.6675	No	0.9762	0.584	0.23	0.0801	No	No
103816_at	F11r	F11 receptor	1.0374	0.2743	0.0467	0.3952	No	1.0091	0.742	0.0274	0.384	No	No
103817_at	Crtap	cartilage associated protein	1.0447	0.2093	0.0015	0.5949	No	1.041	0.462	0.0101	0.822	No	No
103818_at	Slc7a7	solute carrier family 7 (cationic amino acid transporter, y+ system), member 7	1.0172	0.5205	0.0117	0.8882	No	1.0017	0.976	0.0677	0.715	No	No
103819_at	Shd	src homology 2 domain-containing transforming protein D	1.016	0.7654	0.3393	0.6731	No	1.0096	0.941	0.032	0.222	No	No
103821_at	Cdc6	cell division cycle 6 homolog (S. cerevisiae)	1.1575	0.004	0.0002	0.0096	No	1.1396	0.0803	0.0856	0.87	No	No
103822_at	Sfrs16	splicing factor, arginine/serine-rich 16 (suppressor-of-white-apricot homolog, Drosophila)	0.981	0.6154	0.0049	0.5794	No	1.0736	0.139	0.0025	0.398	No	No
103823_at	Top3b	topoisomerase (DNA) III beta	0.9628	0.0206	0.002	0.0064	No	0.9183	0.0008	0.0001	0.0001	No	No
103824_at	Wfs1	Wolfram syndrome 1 homolog (human)	1.006	0.7981	0.6168	0.8517	No	1.0087	0.947	0.138	0.368	No	No
103825_at	Rps6kb2	ribosomal protein S6 kinase, polypeptide 2	0.9578	0.0971	0.5233	0.6133	No	1.0105	0.833	0.841	0.0379	No	No
103826_at	Zbtb7a	zinc finger and BTB domain containing 7a	0.9137	0.132	0.1328	0.0918	No	0.971	0.802	0.0568	0.332	No	No
103828_at	Vps52 /// LOC631721	vacuolar protein sorting 52 (yeast) /// similar to vacuolar protein sorting 52	1.0116	0.8031	0.2244	0.4114	No	1.035	0.159	0.0521	0.0711	No	No
103829_at	Herc2	hect (homologous to the E6-AP (UBE3A) carboxyl terminus) domain and RCC1 (CHC1)-like domain (RLD) 2	0.9277	0.2677	0.0037	0.7617	No	0.9497	0.426	0.0022	0.679	No	No
103830_at	Snai1	snail homolog 1 (Drosophila)	1.0556	0.48	0.0029	0.3952	No	0.9952	0.868	0.34	0.0746	No	No
103831_at	Mkln1	muskelin 1, intracellular mediator containing kelch motifs	0.9975	0.9519	0.3512	0.1098	No	1.0952	0.255	0.706	0.113	No	No
103832_at	Tfip11	tuftelin interacting protein 11	0.8713	0.0498	0.7102	0.1379	No	1.0397	0.771	0.0654	0.0432	No	No
103833_at	Hipk2	homeodomain interacting protein kinase 2	0.9842	0.8235	0.1714	0.4935	No	0.9936	0.86	0.0582	0.445	No	No
103835_f_at	Hpcal1	hippocalcin-like 1	0.9747	0.2753	0.0016	0.1271	No	0.9952	0.619	0.0001	0.327	No	No
103836_at	Wbp4	WW domain binding protein 4	0.9776	0.4044	0.0019	0.019	No	0.9596	0.495	0.0173	0.449	No	No
103837_at	Casp14	caspase 14	1.0523	0.1657	0.0369	0.1796	No	1.1431	0.0654	0.0396	0.0624	No	No
103838_at	Sypl2	synaptophysin-like 2	0.9315	0.1324	0.6464	0.0857	No	0.994	0.831	0.0125	0.675	No	No
103839_at	Sphk1	sphingosine kinase 1	0.9763	0.4025	0.0003	0.552	No	0.9642	0.154	0.0002	0.478	No	No
103840_at	Rad17	RAD17 homolog (S. pombe)	1.0708	0.3521	0.0536	0.059	No	1.2218	0.0571	0.275	0.0864	No	No
103841_at	Zfp64	zinc finger protein 64	0.8784	0.1068	0.1173	0.0615	No	0.9462	0.541	0.0608	0.311	No	No
103842_at	Ddx3y	DEAD (Asp-Glu-Ala-Asp) box polypeptide 3, Y-linked	0.9325	0.5813	0.0042	0.1581	No	0.9877	0.416	0.0038	0.0653	No	No
103843_at	Gnao1	guanine nucleotide binding protein, alpha o	1.0491	0.3618	0.0009	0.646	No	0.9328	0.0827	0.0009	0.0431	No	No
103844_at	Gnao1	guanine nucleotide binding protein, alpha o	0.9982	0.9769	0.0115	0.7652	No	1.0279	0.319	0.0002	0.0087	No	No
103845_at	Slc31a1	solute carrier family 31, member 1	1.0364	0.1226	0.0033	0.485	No	1.0024	0.807	0.572	0.0032	No	No
103846_at	Lgals7	lectin, galactose binding, soluble 7	0.9018	0.0243	0.0003	0.0026	No	0.8299	0.0218	0.415	0.0738	No	No
103847_at	LOC435023	similar to spermiogenesis specific transcript on the Y 2	0.9796	0.7208	0.3849	0.7128	No	1.0037	0.642	0.0035	0.0522	No	No
103848_at	Crkl	v-crk sarcoma virus CT10 oncogene homolog (avian)-like	0.9951	0.8022	0	0.0858	No	0.9186	0.118	0.0005	0.596	No	No
103849_at	Crkl	v-crk sarcoma virus CT10 oncogene homolog (avian)-like	0.9887	0.8602	0.4215	0.886	No	0.9374	0.0094	0.0101	0.0098	No	No
103850_at	Loxl1	lysyl oxidase-like 1	0.9438	0.0828	0.0351	0.0258	No	0.9266	0.421	0.737	0.466	No	No
103852_at	2900001O04 Rik	RIKEN cDNA 2900001O04 gene	1.0768	0.5722	0.0313	0.6231	No	1.054	0.25	0.0021	0.799	No	No
103853_at	Grwd1	glutamate-rich WD repeat containing 1	1.017	0.6201	0.9669	0.0086	No	0.941	0.459	0.0851	0.143	No	No
103854_at	Arl2	ADP-ribosylation factor-like 2	0.9443	0.0967	0.0126	0.9511	No	1.0421	0.225	0.0115	0.685	No	No
103855_at	Plec1	plectin 1	0.9375	0.4282	0.0772	0.4321	No	0.9895	0.71	0.0063	0.37	No	No
103858_at	Eral1	Era (G-protein)-like 1 (E. coli)	0.938	0.314	0.0279	0.6955	No	0.957	0.0956	0.499	0.706	No	No
103859_at	Eral1	Era (G-protein)-like 1 (E. coli)	0.9439	0.3695	0.0423	0.5047	No	0.931	0.19	0.003	0.54	No	No
103860_at	D7Wsu128e	DNA segment, Chr 7, Wayne State University 128, expressed	1.0671	0.0717	0.7908	0.0125	No	1.1293	0.378	0.0012	0.166	No	No
103861_s_at	D7Wsu128e	DNA segment, Chr 7, Wayne State University 128, expressed	1.0225	0.5518	0.0031	0.0483	No	1.0413	0.394	0.0361	0.176	No	No
103862_r_at	D7Wsu128e	DNA segment, Chr 7, Wayne State University 128, expressed	0.9863	0.7527	0.6038	0.4066	No	0.9985	0.972	0.178	0.41	No	No
103863_at	5630401J11 Rik	RIKEN cDNA 5630401J11 gene	1.0655	0.0684	0.0421	0.1266	No	1.0666	0.0112	0.776	0.0316	No	No

103865_at	E330014E10 Rik /// LOC545761 /// AU018829 /// LOC546201 /// LOC621019 /// LOC622792 /// LOC664993 /// LOC665003 /// LOC665290 /// LOC665558 /// LOC665616 /// LOC665711 /// LOC665718 /// LOC665723 ///	RIKEN cDNA E330014E10 gene /// similar to PRAME family member 8 /// expressed sequence AU018829 /// similar to PRAME family member 8 /// similar to PRAME family member 8 /// similar to PRAME family member 8 /// similar to PRAME family member 8 /// similar to PRAME family member 8 /// similar to PRAME family member 8 /// similar to PRAME family member 8 /// similar to PRAME family member 8 /// similar to PRAME family member 8 /// similar to PRAME family member 8 /// similar to PRAME family member 8 /// similar to PRAME family member 8	0.9649	0.6862	0.2063	0.7957	No	0.9386	0.347	0.215	0.854	No	No
103866_at	Pik3cd	phosphatidylinositol 3-kinase catalytic delta polypeptide	1.0166	0.7295	0.0077	0.348	No	1.104	0.0454	0.0005	0.0017	No	No
103867_at	2210409E12 Rik	RIKEN cDNA 2210409E12 gene	1.0673	0.3453	0.7901	0.8277	No	1.0215	0.76	0.851	0.797	No	No
103868_at	Nufip1	nuclear fragile X mental retardation protein interacting protein 1	0.944	0.2563	0.9578	0.1671	No	0.9419	0.476	0.0033	0.861	No	No
103869_at	Thbs3	thrombospondin 3	0.9449	0.002	0	0.0021	No	0.9396	0.0572	0	0.0882	No	No
103871_at	Sec23ip	Sec23 interacting protein	0.9166	0.0028	0.1316	0.0093	No	0.9298	0.0117	0.0022	0.0681	No	No
103872_r_at	---	---	0.9022	0.0305	0.0669	0.4619	No	0.8947	0.141	0.065	0.491	No	No
103873_i_at	2310015N07 Rik	RIKEN cDNA 2310015N07 gene	1.0537	0.4822	0.001	0.2454	No	1.0784	0.526	0.0057	0.195	No	No
103874_r_at	2310015N07 Rik	RIKEN cDNA 2310015N07 gene	0.9956	0.8427	0.0065	0.1091	No	1.1059	0.165	0.595	0.0831	No	No
103875_at	Ngrn	neugrin, neurite outgrowth associated	0.9887	0.7849	0.4815	0.1124	No	0.951	0.408	0.141	0.144	No	No
103876_at	Crlz1	charged amino acid rich leucine zipper 1	0.9895	0.7458	0.2262	0.1361	No	0.9266	0.101	0.0024	0.145	No	No
103877_at	Slc35c2	solute carrier family 35, member C2	1.0438	0.3867	0.0215	0.8587	No	1.0184	0.41	0.0074	0.97	No	No
103878_at	Ap3b1	adaptor-related protein complex 3, beta 1 subunit	0.9622	0.0482	0.0541	0.7987	No	0.9793	0.447	0.446	0.465	No	No
103879_at	Foxred1	FAD-dependent oxidoreductase domain containing 1	0.9738	0.4334	0.4263	0.404	No	0.9864	0.413	0.391	0.437	No	No
103881_at	Ppa2	pyrophosphatase (inorganic) 2	0.9737	0.6102	0.0014	0.866	No	0.9909	0.769	0.0083	0.286	No	No
103882_at	Cpn1	carboxypeptidase N, polypeptide 1	0.9649	0.2911	0.0027	0.5649	No	1.1306	0.1	0.0859	0.267	No	No
103884_at	Atpbd1c	ATP binding domain 1 family, member C	0.9806	0.6214	0.1636	0.1166	No	1.0057	0.834	0.017	0.435	No	No
103885_at	Prpf31	PRP31 pre-mRNA processing factor 31 homolog (yeast)	0.8907	0.0308	0.2052	0.0346	No	0.8979	0.0416	0.511	0.62	No	No
103886_at	Reep5	receptor accessory protein 5	1.0329	0.7456	0.1151	0.8949	No	0.9946	0.928	0.0165	0.777	No	No
103887_at	S100a9	S100 calcium binding protein A9 (calgranulin B)	0.8872	0.0157	0.51	0.3918	No	0.9603	0.463	0.292	0.335	No	No
103888_at	Rbpms	RNA binding protein gene with multiple splicing	0.9715	0.5333	0.0048	0.2343	No	1.0933	0.0623	0.121	0.144	No	No
103889_at	Tbl1xr1	transducin (beta)-like 1X-linked receptor 1	0.984	0.749	0.001	0.1694	No	0.9494	0.558	0.0026	0.148	No	No
103890_at	Unc45a	unc-45 homolog A (C. elegans)	0.9677	0.3246	0.0199	0.2012	No	0.9493	0.313	0.0063	0.652	No	No
103891_i_at	Ell2	elongation factor RNA polymerase II 2	0.9399	0.1777	0.0004	0.4742	No	0.9557	0.528	0.0022	0.762	No	No
103892_r_at	Ell2	elongation factor RNA polymerase II 2	0.9702	0.4013	0.0025	0.4271	No	0.9264	0.329	0.0224	0.209	No	No
103893_at	Fbxo38	F-box protein 38	0.9954	0.8843	0.001	0.6016	No	0.9794	0.571	0.676	0.448	No	No
103894_at	Shkbp1	Sh3kbp1 binding protein 1	1.0673	0.0376	0.0002	0.8743	No	0.9775	0.844	0.0001	0.0609	No	No
103895_at	AW549877	expressed sequence AW549877	0.9254	0.4283	0.0162	0.6319	No	0.9295	0.395	0.0805	0.957	No	No
103896_f_at	Igfbp1	insulin-like growth factor binding protein 1	1.0378	0.3435	0.5555	0.8315	No	0.9889	0.886	0.15	0.197	No	No
103897_r_at	Igfbp1	insulin-like growth factor binding protein 1	1.017	0.8056	0.0456	0.2339	No	1.1311	0.0476	0.0005	0.0099	No	No
103899_at	Atp11a	ATPase, class VI, type 11A	1.0235	0.7536	0.0005	0.9227	No	1.0066	0.895	0.0001	0.943	No	No
103900_at	Centg3	centaurin, gamma 3	0.985	0.5759	0.0002	0.5403	No	0.9796	0.62	0.0004	0.119	No	No
103901_at	Tbc1d23	TBC1 domain family, member 23	1.0102	0.924	0.4268	0.5175	No	1.0214	0.688	0.958	0.836	No	No
103903_at	Dnajc11	DnaJ (Hsp40) homolog, subfamily C, member 11	0.9795	0.6341	0.9067	0.0765	No	0.9426	0.48	0.0369	0.98	No	No
103904_at	Igfbp6	insulin-like growth factor binding protein 6	0.8543	0.0196	0.022	0.0151	No	0.9071	0.119	0.0016	0.698	No	No
103905_at	Car12	carbonic anyhydrase 12	1.0931	0.083	0.235	0.4274	No	1.1131	0.0377	0.957	0.122	No	No
103906_f_at	Nedd4l	neural precursor cell expressed, developmentally down-regulated gene 4-like	1.0115	0.8841	0.2335	0.1587	No	1.013	0.884	0.0125	0.036	No	No
103907_at	Nedd4l	neural precursor cell expressed, developmentally down-regulated gene 4-like	0.9346	0.008	0.0123	0.0649	Yes	0.9956	0.81	0.329	0.391	No	No
103908_at	Sdccag3	serologically defined colon cancer antigen 3	0.9945	0.9045	0.0975	0.6328	No	0.98	0.732	0.544	0.368	No	No
103909_at	Ccs	copper chaperone for superoxide dismutase	1.0535	0.2453	0.0008	0.406	No	1.0223	0.551	0.0057	0.617	No	No
103910_at	Taf10	TAF10 RNA polymerase II, TATA box binding protein (TBP)-associated factor	1.0825	0.0409	0	0.0315	No	1.0723	0.0254	0	0.0131	No	No
103911_at	Sumf1	sulfatase modifying factor 1	0.8733	0.1535	0.1261	0.1131	No	0.916	0.084	0.0203	0.0109	No	No
103912_at	Ppp2r1b	protein phosphatase 2 (formerly 2A), regulatory subunit A (PR 65), beta isoform	1.0057	0.8554	0.0029	0.0607	No	1.01	0.507	0.0012	0.0325	No	No
103913_at	Sec61a2	Sec61, alpha subunit 2 (S. cerevisiae)	0.9704	0.5971	0.0242	0.4212	No	0.9972	0.989	0.036	0.393	No	No
103914_at	Pcyt2	phosphate cytidylyltransferase 2, ethanolamine	0.9578	0.0726	0.0026	0.0004	No	0.9935	0.922	0.0127	0.426	No	No
103916_at	8430420C20 Rik	RIKEN cDNA 8430420C20 gene	0.9472	0.447	0.0021	0.0772	No	0.9722	0.331	0.007	0.0339	No	No
103918_at	Slc15a2	solute carrier family 15 (H+/peptide transporter), member 2	1.0819	0.1443	0.0048	0.7083	No	1.1092	0.175	0.157	0.547	No	No
103921_i_at	Cyb5r1	cytochrome b5 reductase 1	0.986	0.7471	0.0238	0.3148	No	1.0037	0.865	0.0323	0.187	No	No
103922_f_at	Cyb5r1	cytochrome b5 reductase 1	0.9744	0.5396	0.0789	0.4352	No	0.9984	0.999	0.358	0.985	No	No
103923_at	Gpr137b	G protein-coupled receptor 137B	0.9626	0.3904	0.0255	0.3883	No	1.0017	0.906	0.068	0.157	No	No
103924_at	Agpat5	1-acylglycerol-3-phosphate O-acyltransferase 5 (lysophosphatidic acid acyltransferase, epsilon)	1.0096	0.4666	0.0032	0.1096	No	1.0129	0.773	0.0007	0.189	No	No
103925_at	Mllt3	myeloid/lymphoid or mixed lineage-leukemia translocation to 3 homolog (Drosophila)	1.0981	0.0128	0	0.003	No	1.0514	0.103	0.0002	0.0283	No	No
103926_at	Eif4g1	eukaryotic translation initiation factor 4, gamma 1	0.9362	0.1347	0.0137	0.0463	No	0.9868	0.575	0.0208	0.0159	No	No
103927_at	Zfp568	zinc finger protein 568	0.9374	0.0575	0.0005	0.1142	No	0.9373	0.0833	0.0005	0.423	No	No
103928_at	Sgol1	shugoshin-like 1 (S. pombe)	1.0677	0.1457	0.0213	0.0304	No	1.0892	0.343	0.156	0.333	No	No
103930_at	BC004022	cDNA sequence BC004022	0.8735	0.0874	0.0026	0.2547	No	0.8953	0.0001	0	0.0003	No	No
103931_at	Gpr162	G protein-coupled receptor 162	1.0843	0.0346	0.0087	0.1721	No	1.066	0.271	0.12	0.435	No	No
103932_at	Wdsof1	WD repeats and SOF domain containing 1	0.9961	0.9416	0.0256	0.757	No	1.04	0.353	0.0235	0.567	No	No
103933_at	Siae	sialic acid acetylesterase	0.9864	0.7534	0.0035	0.053	No	1.1132	0.143	0.0066	0.0394	No	No
103934_at	Slc6a13	solute carrier family 6 (neurotransmitter transporter, GABA), member 13	0.9953	0.8482	0.2326	0.4811	No	1.0386	0.234	0.0077	0.0495	No	No
103935_at	Atp2a3	ATPase, Ca++ transporting, ubiquitous	1.0194	0.6181	0.1583	0.2802	No	1.0593	0.0484	0.23	0.155	No	No
103939_at	Hibch	3-hydroxyisobutyryl-Coenzyme A hydrolase	0.9348	0.1915	0.0047	0.6485	No	0.91	0.3	0.0293	0.788	No	No
103941_at	LOC630963	similar to spectrin alpha 1	1.0434	0.6111	0.2569	0.3919	No	1.1918	0.17	0.287	0.67	No	No
103942_at	Gucy2c	guanylate cyclase 2c	1.0017	0.9449	0.4007	0.0164	No	1.0091	0.788	0.499	0.734	No	No
103943_at	Ppfia4	protein tyrosine phosphatase, receptor type, f polypeptide (PTPRF), interacting protein (liprin), alpha 4	1.0595	0.1598	0.0836	0.5765	No	1.0315	0.472	0.1	0.914	No	No
103944_at	Rad51l1	RAD51-like 1 (S. cerevisiae)	1.0108	0.8979	0.9772	0.8959	No	1.0822	0.0268	0.0366	0.0094	No	No
103945_at	Cfc1	cripto, FRL-1, cryptic family 1	0.9941	0.8879	0.264	0.044	No	1.0009	1	0.0905	0.677	No	No
103946_at	Pstpip1	proline-serine-threonine phosphatase-interacting protein 1	0.9547	0.3573	0.0014	0.1424	No	0.9902	0.871	0.0003	0.0374	No	No
103947_at	Ppp6c	protein phosphatase 6, catalytic subunit	1.0008	0.9864	0.0019	0.738	No	0.9957	0.941	0.0001	0.0312	No	No

103948_at	Tlx3	T-cell leukemia, homeobox 3	0.9905	0.6807	0.017	0.1108	No	0.9968	0.993	0.0123	0.381	No	No
103949_at	Ihh	Indian hedgehog	1.1102	0.1814	0.0009	0.4939	No	1.0356	0.523	0.134	0.436	No	No
103950_at	Aqr	aquarius	1.042	0.0355	0.883	0.3058	No	0.8365	0.108	0.456	0.235	No	No
103952_at	Hoxb9	Homeo box B9	0.9205	0.238	0.024	0.8832	No	0.9734	0.709	0.206	0.362	No	No
103953_at	Sec22b	SEC22 vesicle trafficking protein homolog B (S. cerevisiae)	1.1501	0.0653	0.0059	0.9847	No	1.057	0.0293	0.0001	0.0251	No	No
103954_at	Reg3a	regenerating islet-derived 3 alpha	1.0767	0.2662	0.7698	0.2568	No	0.9199	0.0688	0.0151	0.363	No	No
103955_at	Cryl1	crystallin, lamda 1	1.0053	0.8801	0.0009	0.0268	No	0.9835	0.816	0.0075	0.103	No	No
103956_at	Azi1	5-azacytidine induced gene 1	0.9674	0.3896	0.0111	0.3269	No	0.9361	0.183	0.0081	0.0865	No	No
103957_at	Tfrc	transferrin receptor	1.0094	0.8977	0.2111	0.5874	No	0.9994	0.919	0.0502	0.205	No	No
103958_g_at	Tfrc	transferrin receptor	0.9755	0.6133	0.0097	0.3908	No	1.0003	0.986	0.0323	0.785	No	No
103959_at	Phf13	PHD finger protein 13	1.0049	0.9488	0.0512	0.3483	No	1.0633	0.386	0.0222	0.649	No	No
103960_at	Rap2ip	Rap2 interacting protein	1.023	0.6006	0.1381	0.3211	No	0.9599	0.273	0.246	0.909	No	No
103961_s_at	Dntt	deoxynucleotidyltransferase, terminal	1.0558	0.5004	0.4079	0.3675	No	1.1636	0.158	0.397	0.116	No	No
103962_at	Dntt	deoxynucleotidyltransferase, terminal	1.0496	0.4484	0.0498	0.4136	No	1.0112	0.92	0.123	0.633	No	No
103963_f_at	Iigp1	interferon inducible GTPase 1	1.0154	0.7203	0.7284	0.5264	No	1.0857	0.134	0.0055	0.217	No	No
103964_at	Esrra	estrogen related receptor, alpha	1.0179	0.7379	0.4842	0.166	No	1.0399	0.271	0.092	0.0106	No	No
103965_at	Entpd6	ectonucleoside triphosphate diphosphohydrolase 6	1.0297	0.4513	0.1793	0.4486	No	0.9984	0.94	0.444	0.672	No	No
103967_at	Mid2	Midline 2	0.9115	0.3327	0.6537	0.3873	No	0.7717	0.0308	0.0073	0.421	No	No
103968_at	Mid2	midline 2	0.9149	0.1003	0.0096	0.9437	No	0.9609	0.658	0.004	0.0792	No	No
103969_at	Mll5	myeloid/lymphoid or mixed-lineage leukemia 5	0.9873	0.8355	0.0007	0.8153	No	0.9438	0.451	0.0018	0.821	No	No
103970_at	Arid3b	AT rich interactive domain 3B (Bright like)	1.121	0.0754	0.8285	0.1407	No	1.1368	0.0006	0.353	0.0001	No	No
103971_at	P2rx1	purinergic receptor P2X, ligand-gated ion channel, 1	0.9876	0.7536	0.9887	0.5465	No	1.026	0.754	0.138	0.726	No	No
103972_at	Kcnj1	potassium inwardly-rectifying channel, subfamily J, member 1	0.9019	0.3153	0.0399	0.4106	No	0.9247	0.466	0.0808	0.516	No	No
103973_at	Kcnj1	potassium inwardly-rectifying channel, subfamily J, member 1	0.9161	0.331	0.059	0.5752	No	1.0572	0.0944	0.0013	0.0049	No	No
103974_at	Tmprss2	transmembrane protease, serine 2	0.9548	0.096	0.1693	0.0216	No	0.9306	0.384	0.323	0.122	No	No
103975_at	Grem2	gremlin 2 homolog, cysteine knot superfamily (Xenopus laevis)	0.9563	0.0917	0.6108	0.0242	No	1.0285	0.737	0.0261	0.135	No	No
103976_at	---	Transcribed locus	0.9447	0.0085	0.0005	0.1534	Yes	1.0244	0.562	0.0768	0.417	No	No
103977_at	F10	coagulation factor X	0.8953	0.1133	0.0065	0.5887	No	0.8768	0.418	0.103	0.218	No	No
103978_at	Zc3h11a	zinc finger CCCH type containing 11A	0.8338	0.0011	0.0028	0.0288	Yes	0.8609	0.0524	0.16	0.333	No	No
103980_at	Epha2	Eph receptor A2	0.9598	0.3745	0.0657	0.8967	No	1.0378	0.18	0.0029	0.0291	No	No
103981_at	U2af1-rs2	U2 small nuclear ribonucleoprotein auxiliary factor (U2AF) 1, related sequence 2	1.2747	0.0131	0.0095	0.0843	No	1.239	0.006	0.0026	0.0161	Yes	Yes
103982_s_at	Adh4	alcohol dehydrogenase 4 (class II), pi polypeptide	1.0662	0.4368	0.0879	0.6357	No	1.0556	0.534	0.166	0.693	No	No
103983_at	Adh4	alcohol dehydrogenase 4 (class II), pi polypeptide	0.9955	0.9162	0.0053	0.1509	No	1.0564	0.55	0.0233	0.707	No	No
103984_at	Pkhd1	polycystic kidney and hepatic disease 1	1.0255	0.5096	0.7924	0.3061	No	1.0973	0.503	0.836	0.293	No	No
103985_at	Map4k2	mitogen activated protein kinase kinase kinase kinase 2	1.0309	0.659	0.0104	0.4296	No	0.9875	0.528	0.0001	0.328	No	No
103986_at	Inhbc	inhibin beta-C	1.0829	0.266	0.6616	0.6649	No	1.0338	0.237	0.0277	0.924	No	No
103987_at	Mog	myelin oligodendrocyte glycoprotein	0.8178	0.0868	0.0121	0.3636	No	0.9473	0.469	0	0.416	No	No
103988_at	1810013C15 Rik	RIKEN cDNA 1810013C15 gene	0.9431	0.0144	0.0159	0.0192	No	1.0458	0.0304	0.497	0.0068	No	No
103989_at	Prdm5	PR domain containing 5	0.8079	0.0214	0.1225	0.0605	No	0.9504	0.141	0.0587	0.348	No	No
103990_at	Fosb	FBJ osteosarcoma oncogene B	1.1167	0.045	0.1812	0.1973	No	1.0784	0.211	0.0317	0.545	No	No
103991_at	Akp5	alkaline phosphatase 5	1.0363	0.3735	0.0502	0.6042	No	0.9791	0.73	0.228	0.858	No	No
103992_f_at	Ndg1 /// LOC433395 /// LOC623189	Nur77 downstream gene 1 /// similar to chromosome 6 open reading frame 148 /// similar to chromosome 6 open reading frame 148	0.9856	0.3891	0.0122	0.8219	No	0.9701	0.815	0.0864	0.561	No	No
103993_at	Spsb2	splA/ryanodine receptor domain and SOCS box containing 2	1.0112	0.7517	0.0311	0.3973	No	1.0409	0.527	0.0268	0.318	No	No
103994_at	Eif2c2	eukaryotic translation initiation factor 2C, 2	1.1055	0.022	0.0002	0.15	No	1.0488	0.0789	0.0003	0.74	No	No
103995_at	Fgfbp1	fibroblast growth factor binding protein 1	0.9993	0.9918	0.1476	0.6996	No	0.9706	0.582	0.121	0.516	No	No
103997_at	Epor	erythropoietin receptor	1.0571	0.2241	0.0038	0.2256	No	1.0714	0.227	0.0016	0.977	No	No
103998_at	Kif16b	kinesin family member 16B	1.1182	0.0591	0.0002	0.0578	No	1.0363	0.515	0.0004	0.0274	No	No
103999_at	4932416N17 Rik	RIKEN cDNA 4932416N17 gene	0.9608	0.0031	0.0006	0.0087	No	0.9447	0.162	0.0029	0.939	No	No
104000_at	2210023G05 Rik	RIKEN cDNA 2210023G05 gene	1.044	0.2722	0.0315	0.3231	No	0.9677	0.46	0.0067	0.988	No	No
104001_at	Rfc5	replication factor C (activator 1) 5	1.0954	0.1633	0.4835	0.0845	No	1.0601	0.349	0.438	0.212	No	No
104002_at	Zfp275	zinc finger protein 275	0.9812	0.5934	0.0018	0.0357	No	0.9133	0.0485	0.0006	0.866	No	No
104003_at	Phka2	phosphorylase kinase alpha 2	0.9261	0.0964	0.0082	0.1511	No	0.9746	0.826	0.0245	0.0436	No	No
104004_at	Golph4	golgi phosphoprotein 4	1.1418	0.0191	0.0003	0.0388	No	1.0966	0.016	0	0.179	No	No
104005_at	B4galt2	UDP-Gal:betaGlcNAc beta 1,4- galactosyltransferase, polypeptide 2	1.0895	0.2118	0.0179	0.5784	No	1.0414	0.215	0.0085	0.121	No	No
104006_at	Eps15	epidermal growth factor receptor pathway substrate 15	1.0068	0.6102	0.048	0.0339	No	0.9881	0.747	0.086	0.0938	No	No
104007_at	Slc25a15	solute carrier family 25 (mitochondrial carrier ornithine transporter), member 15	1.029	0.4043	0.1294	0.9493	No	1.0134	0.307	0.0582	0.461	No	No
104008_at	Spdef	SAM pointed domain containing ets transcription factor	0.957	0.4062	0.1556	0.5138	No	1.005	0.936	0.722	0.731	No	No
104010_at	Zfp99	zinc finger protein 99	0.969	0.2196	0.1032	0.483	No	1.0088	0.651	0.562	0.197	No	No
104011_at	Aox1	aldehyde oxidase 1	0.9138	0.2474	0.591	0.3043	No	0.9698	0.551	0.124	0.79	No	No
104012_at	Gnrhr	gonadotropin releasing hormone receptor	1.0357	0.5831	0.0758	0.5259	No	0.9842	0.875	0.269	0.854	No	No
104013_at	Gnrhr	gonadotropin releasing hormone receptor	0.9549	0.3903	0.4019	0.7966	No	0.9031	0.0873	0.0207	0.65	No	No
104014_at	Hfe	hemochromatosis	1.0141	0.6468	0.056	0.0409	No	0.9982	0.969	0.25	0.644	No	No
104015_at	Metap1	methionyl aminopeptidase 1	0.8937	0.1159	0.0919	0.0684	No	0.9872	0.504	0.258	0.0804	No	No
104016_at	Gjb5	gap junction membrane channel protein beta 5	0.9793	0.6556	0.1181	0.6834	No	0.9441	0.285	0.858	0.635	No	No
104017_at	Acsl4	acyl-CoA synthetase long-chain family member 4	1.028	0.742	0.3512	0.9465	No	1.0257	0.529	0.0128	0.146	No	No
104018_at	Lct	lactase	1.1111	0.1164	0.1937	0.0485	No	0.9506	0.342	0.0482	0.548	No	No
104019_at	---	---	0.9946	0.9087	0.0295	0.3764	No	1.027	0.683	0.0576	0.782	No	No
104020_at	Rap2b	RAP2B, member of RAS oncogene family	1.0263	0.5123	0.0637	0.7786	No	0.971	0.366	0.0032	0.503	No	No
104021_at	Hoxa11	homeo box A11	0.9738	0.7665	0.47	0.3238	No	1.0156	0.788	0.503	0.146	No	No
104022_at	Mphosph10	M-phase phosphoprotein 10 (U3 small nucleolar ribonucleoprotein)	0.9412	0.0292	0.5865	0.3223	No	0.9641	0.302	0.0588	0.119	No	No
104023_at	Cd300d /// Cd300a	Cd300D antigen /// CD300A antigen	1.0436	0.3029	0.0409	0.2498	No	1.0012	0.914	0.002	0.61	No	No
104024_at	Cyp3a25 /// LOC622249	cytochrome P450, family 3, subfamily a, polypeptide 25 /// similar to cytochrome P450, family 3, subfamily a, polypeptide 25	1.0218	0.5678	0.9947	0.1524	No	1.2799	0.106	0.149	0.498	No	No
104025_at	Thop1	thimet oligopeptidase 1	1.0048	0.9091	0.0028	0.1788	No	1.045	0.242	0.0014	0.175	No	No
104029_at	Clgn	calmegin	1.0209	0.7204	0.7475	0.8136	No	1.1202	0.0672	0.0317	0.139	No	No
104030_at	Ptch1	patched homolog 1	1.0136	0.876	0.0039	0.3317	No	1.0062	0.464	0.0006	0.0061	No	No
104031_at	Ptch1	patched homolog 1	1.0064	0.9005	0.0157	0.2027	No	1.1128	0.0259	0.0117	0.0239	No	No
104032_at	Mast3	microtubule associated serine/threonine kinase 3	0.8552	0.0245	0.3415	0.2775	No	0.9012	0.0353	0.232	0.0614	No	No
104033_at	Mgea6	meningioma expressed antigen 6 (coiled-coil proline-rich)	0.962	0.6547	0.0058	0.469	No	0.9813	0.637	0.0074	0.241	No	No
104034_at	AI464131	expressed sequence AI464131	0.9537	0.0802	0.0039	0.0988	No	0.9554	0.178	0.108	0.838	No	No
104035_at	9130404D08 Rik	RIKEN cDNA 9130404D08 gene	0.9556	0.3625	0.0045	0.1206	No	0.9681	0.624	0.0262	0.269	No	No
104036_at	Dpp7	dipeptidylpeptidase 7	1.018	0.8484	0.3387	0.9386	No	1.1449	0.132	0.155	0.852	No	No
104037_at	Rwdd4a	RWD domain containing 4A	0.9985	0.9686	0.344	0.1381	No	1.0131	0.718	0.286	0.292	No	No
104038_at	4933434E20 Rik	RIKEN cDNA 4933434E20 gene	1.0117	0.5603	0.6621	0.1647	No	1.0046	0.895	0.104	0.296	No	No
104039_at	AU021838	expressed sequence AU021838	0.9714	0.5112	0.0248	0.8623	No	0.9455	0.193	0.0053	0.0702	No	No
104040_at	2600009E05 Rik	RIKEN cDNA 2600009E05 gene	0.9392	0.5443	0.3568	0.5855	No	1.0602	0.495	0.224	0.35	No	No
104041_at	Zubr1	zinc finger, UBR1 type 1	0.9712	0.6577	0.0284	0.0863	No	0.9588	0.486	0.0018	0.0346	No	No
104042_at	Slc35b2	solute carrier family 35, member B2	0.9395	0.2298	0.0024	0.0254	No	1.0526	0.346	0.0042	0.576	No	No
104044_at	B230118G17 Rik	RIKEN cDNA B230118G17 gene	1.0296	0.258	0.0034	0.5796	No	1.0495	0.214	0.0224	0.805	No	No
104045_at	Tspyl4	TSPY-like 4	1.0013	0.9877	0.0006	0.4262	No	1.0342	0.757	0.0008	0.513	No	No
104046_at	Arih1	ariadne ubiquitin-conjugating enzyme E2 binding protein homolog 1 (Drosophila)	1.0329	0.6749	0.0019	0.8871	No	1.0445	0.64	0.0054	0.927	No	No

104047_at	Mapk8	mitogen activated protein kinase 8	1.0079	0.8921	0.0047	0.265	No	1.0344	0.612	0.255	0.32	No	No
104048_at	Cars	cysteinyl-tRNA synthetase	0.8836	0.0013	0.1021	0.0016	No	0.8873	0.0069	0.0276	0.341	Yes	No
104049_at	Rnf6	ring finger protein (C3H2C3 type) 6	0.8763	0.0502	0.0129	0.842	No	0.9021	0.156	0.66	0.87	No	No
104050_at	Zfr	zinc finger RNA binding protein	0.9849	0.8275	0.7251	0.1872	No	0.9482	0.309	0.0342	0.408	No	No
104052_at	Tiprl	TIP41, TOR signalling pathway regulator-like (S. cerevisiae)	0.9853	0.657	0.0228	0.0313	No	1.0256	0.598	0.143	0.804	No	No
104053_at	Trio	triple functional domain (PTPRF interacting)	0.9005	0.122	0.4482	0.3107	No	0.9153	0.14	0.394	0.925	No	No
104055_at	D16Bwg1543e	DNA segment, Chr 16, Brigham & Women's Genetics 1543 expressed	0.9443	0.1883	0.492	0.4602	No	0.9348	0.602	0.877	0.307	No	No
104056_at	D16Bwg1543e	DNA segment, Chr 16, Brigham & Women's Genetics 1543 expressed	0.9867	0.8891	0.0806	0.6202	No	0.9404	0.366	0.0886	0.211	No	No
104057_at	Grpel1	GrpE-like 1, mitochondrial	0.9827	0.6824	0.4273	0.0597	No	0.9553	0.409	0.95	0.0155	No	No
104058_at	1110018J12Rik	RIKEN cDNA 1110018J12 gene	0.9469	0.1239	0.0116	0.7939	No	0.9472	0.0658	0.0132	0.303	No	No
104059_at	Chd8	chromodomain helicase DNA binding protein 8	0.9068	0.0073	0.012	0.2867	Yes	0.9117	0.0304	0.0074	0.129	No	Yes
104060_at	Zcrb1	zinc finger CCHC-type and RNA binding motif 1	1.0581	0.1071	0.0054	0.8947	No	1.014	0.923	0.0827	0.393	No	No
104063_at	Tom1l1	target of myb1-like 1 (chicken)	1.0293	0.6626	0.0009	0.1248	No	1.0455	0.707	0.0006	0.292	No	No
104064_at	Slc9a3r2	solute carrier family 9 (sodium/hydrogen exchanger), isoform 3 regulator 2	1.0356	0.0896	0.0526	0.0223	No	0.9769	0.236	0.0012	0.0385	No	No
104065_at	Edem1	ER degradation enhancer, mannosidase alpha-like 1	1.0099	0.8547	0.0417	0.8602	No	1.0353	0.585	0.0975	0.553	No	No
104066_at	AW548124	expressed sequence AW548124	1.099	0.1235	0.0004	0.1965	No	1.0677	0.0411	0	0.332	No	No
104067_at	Tubgcp3	tubulin, gamma complex associated protein 3	1.0561	0.5124	0.1142	0.6703	No	1.0994	0.158	0.101	0.233	No	No
104068_at	Strn4	Striatin, calmodulin binding protein 4	0.9666	0.1806	0.063	0.2415	No	1.0706	0.135	0.0923	0.0306	No	No
104069_at	Spats2	spermatogenesis associated, serine-rich 2	0.9769	0.5623	0.0117	0.9606	No	0.9636	0.365	0.259	0.774	No	No
104070_at	Pcaf	p300/CBP-associated factor	0.8406	0.1121	0.005	0.2132	No	0.9478	0.371	0.0011	0.936	No	No
104071_at	Tnpo2	transportin 2 (importin 3, karyopherin beta 2b)	0.9549	0.343	0.0727	0.7837	No	0.9891	0.85	0.197	0.551	No	No
104072_at	Apcs	serum amyloid P-component	0.9652	0.1463	0.0164	0.6492	No	1.0801	0.176	0.0178	0.0245	No	No
104073_at	Cugbp1	CUG triplet repeat, RNA binding protein 1	1.0027	0.8889	0.9133	0.1024	No	1.0463	0.0235	0.0027	0.0231	No	No
104074_at	Map3k3	mitogen activated protein kinase kinase kinase 3	0.9578	0.0942	0.0008	0.878	No	0.9934	0.844	0.0041	0.655	No	No
104076_at	1190017O12Rik	RIKEN cDNA 1190017O12 gene	0.8517	0.0201	0.2247	0.0068	No	0.9388	0.345	0.688	0.158	No	No
104077_at	1110049G11Rik	RIKEN cDNA 1110049G11 gene	0.9821	0.4598	0.0015	0.1755	No	1.0908	0.0817	0.0058	0.0441	No	No
104078_g_at	1110049G11Rik	RIKEN cDNA 1110049G11 gene	0.987	0.8433	0.0128	0.1003	No	0.9968	0.857	0.0573	0.127	No	No
104079_at	Dyrk1a	dual-specificity tyrosine-(Y)-phosphorylation regulated kinase 1a	1.121	0.0476	0.0009	0.1373	No	1.0596	0.247	0.0181	0.0942	No	No
104080_at	Pdap1	PDGFA associated protein 1	1.09	0.1812	0.3578	0.2725	No	1.1211	0.101	0.59	0.13	No	No
104082_at	Rab12	RAB12, member RAS oncogene family	0.9723	0.5315	0.0056	0.1501	No	1.0103	0.966	0.0003	0.392	No	No
104083_at	Cdh5	cadherin 5	0.894	0.1975	0.0171	0.5046	No	0.9131	0.358	0.0044	0.882	No	No
104085_at	2900042B11Rik	RIKEN cDNA 2900042B11 gene	0.9396	0.2837	0.0252	0.5078	No	0.9667	0.641	0.0981	0.854	No	No
104086_at	Dmgdh	dimethylglycine dehydrogenase precursor	0.9642	0.3065	0.0842	0.131	No	1.0025	0.888	0.114	0.143	No	No
104087_at	D10Ertd641e	DNA segment, Chr 10, ERATO Doi 641, expressed	0.8341	0.0051	0.0739	0.0225	Yes	0.9377	0.239	0.178	0.388	No	No
104088_at	Bnip1	BCL2/adenovirus E1B interacting protein 1, NIP1	0.9497	0.2152	0.0245	0.5001	No	1.1018	0.0867	0.0093	0.309	No	No
104089_at	2810026P18Rik	RIKEN cDNA 2810026P18 gene	1.0223	0.8412	0.1286	0.8584	No	1.1055	0.245	0.058	0.297	No	No
104090_at	Cdc23	CDC23 (cell division cycle 23, yeast, homolog)	1.0222	0.4957	0.0262	0.2941	No	1.0245	0.492	0.936	0.0569	No	No
104091_at	Tspan9	tetraspanin 9	1.05	0.3028	0.1806	0.6186	No	1.0885	0.108	0.378	0.178	No	No
104092_at	Usp48 /// LOC674195	ubiquitin specific peptidase 48 /// hypothetical protein LOC674195	0.9819	0.7224	0.0052	0.8375	No	1.013	0.566	0.001	0.22	No	No
104093_at	Lsp1	lymphocyte specific 1	0.904	0.0926	0.0218	0.4977	No	0.9085	0.202	0.102	0.894	No	No
104094_at	Pdlim4	PDZ and LIM domain 4	1.0123	0.8719	0.0552	0.7584	No	1.0735	0.154	0.0027	0.926	No	No
104095_at	AU040320	expressed sequence AU040320	0.9741	0.5469	0.5082	0.9268	No	0.9982	0.944	0.162	0.687	No	No
104096_at	Orc4l	origin recognition complex, subunit 4-like (S. cerevisiae)	1.0088	0.8898	0.0225	0.0406	No	1.0426	0.333	0.019	0.0926	No	No
104097_at	Bub1	budding uninhibited by benzimidazoles 1 homolog (S. cerevisiae)	0.9806	0.8202	0.6164	0.2649	No	1.0408	0.563	0.686	0.142	No	No
104098_at	Pxmp2	peroxisomal membrane protein 2	0.9484	0.1427	0.0546	0.5739	No	0.9026	0.177	0.166	0.672	No	No
104099_at	Pglyrp1	peptidoglycan recognition protein 1	0.9178	0.0525	0.0001	0.0041	No	0.97	0.373	0.0049	0.014	No	No
104100_at	Ptrf	polymerase I and transcript release factor	0.8675	0.093	0.1923	0.4322	No	0.8739	0.0916	0.681	0.971	No	No
104101_at	Slc9a8	solute carrier family 9 (sodium/hydrogen exchanger), member 8	1.0241	0.4099	0.684	0.5945	No	1.0058	0.914	0.333	0.4	No	No
104102_at	Htra2	HtrA serine peptidase 2	0.8786	0.0301	0.2869	0.0682	No	0.9324	0.0999	0.227	0.968	No	No
104103_at	Atad3a	ATPase family, AAA domain containing 3A	1.0485	0.4245	0.8447	0.2879	No	1.1059	0.0404	0.369	0.0859	No	No
104104_at	Crxos1	Crx opposite strand transcript 1	0.9835	0.8344	0.0916	0.5452	No	0.8732	0.572	0.0021	0.13	No	No
104105_at	Xpo6	exportin 6	1.0637	0.1014	0.3054	0.7697	No	1.1126	0.0276	0.108	0.312	No	No
104106_at	Sbno1	sno, strawberry notch homolog 1 (Drosophila)	1.0335	0.6222	0.0003	0.0128	No	1.0009	0.132	0	0.0196	No	No
104108_at	Rab6ip1	Rab6 interacting protein 1	0.9455	0.3143	0.1009	0.0827	No	0.9216	0.195	0.0076	0.182	No	No
104109_at	Fbxo21	F-box only protein 21	1.021	0.6477	0.0077	0.94	No	1.0336	0.426	0.257	0.357	No	No
104110_at	Gatad2a	GATA zinc finger domain containing 2A	1.0683	0.0875	0.0036	0.2019	No	1.0552	0.307	0.0037	0.575	No	No
104112_at	Rab21	RAB21, member RAS oncogene family	0.9515	0.2646	0.6198	0.5322	No	1.1433	0.0533	0.134	0.0527	No	No
104114_at	Edem3	ER degradation enhancer, mannosidase alpha-like 3	0.9481	0.2156	0.3193	0.4165	No	0.9655	0.434	0.224	0.268	No	No
104115_at	Psme4	proteasome (prosome, macropain) activator subunit 4	1.0124	0.842	0.005	0.4081	No	1.0298	0.697	0.0192	0.775	No	No
104116_at	D5Ertd593e	DNA segment, Chr 5, ERATO Doi 593, expressed	0.8934	0.0232	0.0181	0.0018	No	0.8872	0.0069	0.174	0.0005	No	No
104117_at	2310040A13Rik	RIKEN cDNA 2310040A13 gene	1.0995	0.2758	0.0025	0.3826	No	1.0878	0.245	0.0001	0.0168	No	No
104118_at	2810037C14Rik	RIKEN cDNA 2810037C14 gene	0.9222	0.0183	0.0577	0.0438	No	0.9598	0.64	0.0155	0.903	No	No
104119_at	2610024E20Rik	RIKEN cDNA 2610024E20 gene	0.9281	0.4035	0.0019	0.4942	No	0.9355	0.355	0.0046	0.225	No	No
104120_at	5330431N19Rik	RIKEN cDNA 5330431N19 gene	0.9613	0.3292	0.7403	0.3837	No	0.9927	0.841	0.0145	0.305	No	No
104121_at	Jup	junction plakoglobin	1.023	0.7514	0.0945	0.6963	No	1.0102	0.853	0.006	0.148	No	No
104122_at	D330001F17Rik	RIKEN cDNA D330001F17 gene	1.0111	0.6086	0.015	0.5392	No	1.0141	0.674	0.126	0.896	No	No
104123_at	Fcho1	FCH domain only 1	1.0382	0.4516	0.0097	0.4588	No	0.971	0.68	0.853	0.49	No	No
104124_at	Blvra	biliverdin reductase A	1.0218	0.558	0.6367	0.9479	No	1.0038	0.926	0.36	0.948	No	No
104125_at	Rnf12	ring finger protein 12	0.982	0.7483	0.0186	0.0946	No	1.0122	0.132	0.0007	0.0001	No	No
104126_at	Cstf2t	cleavage stimulation factor, 3' pre-RNA subunit 2, tau	1.0639	0.189	0.1811	0.9338	No	0.9946	0.866	0.0373	0.59	No	No
104128_at	D15Mgi27	DNA Segment, Chr 15, Mouse Genome Informatics 27	1.0723	0.0452	0.1918	0.6658	No	1.0172	0.506	0.0038	0.406	No	No
104129_at	Cyp4f13	cytochrome P450, family 4, subfamily f, polypeptide 13	0.9598	0.5308	0.0067	0.7238	No	0.9419	0.344	0.0016	0.233	No	No
104131_at	Gpr175	G protein-coupled receptor 175	0.9822	0.7957	0.0284	0.5468	No	1.0529	0.401	0.616	0.111	No	No
104132_at	Cox4nb	COX4 neighbor	1.0182	0.6185	0.0082	0.0282	No	0.9955	0.945	0.0009	0.433	No	No
104134_at	Gdap2	ganglioside-induced differentiation-associated-protein 2	1.0061	0.8562	0.0063	0.0698	No	0.9866	0.819	0.0011	0.0174	No	No
104135_at	Arl3	ADP-ribosylation factor-like 3	1.0137	0.8641	0.0076	0.9379	No	1.0238	0.376	0.0048	0.468	No	No
104136_at	Dlgap4	discs, large homolog-associated protein 4 (Drosophila)	0.9925	0.8441	0.2295	0.2489	No	1.0143	0.586	0.0063	0.233	No	No
104137_at	Abca2	ATP-binding cassette, sub-family A (ABC1), member 2	1.0042	0.9137	0.1435	0.9731	No	0.9033	0.0732	0.009	0.026	No	No
104138_at	Pafah2	platelet-activating factor acetylhydrolase 2	0.9326	0.1425	0.0136	0.1291	No	0.9726	0.526	0.302	0.786	No	No
104139_at	P4ha1	procollagen-proline, 2-oxoglutarate 4-dioxygenase (proline 4-hydroxylase), alpha 1 polypeptide	0.9816	0.5655	0.0245	0.0675	No	0.9667	0.306	0.0033	0.162	No	No
104140_s_at	Nomo1	nodal modulator 1	1.0383	0.4043	0.0391	0.9665	No	1.0028	0.871	0.0013	0.593	No	No
104141_at	D15Wsu75e	DNA segment, Chr 15, Wayne State University 75, expressed	0.9405	0.047	0.0045	0.867	No	0.8994	0.0553	0.0081	0.0869	No	No
104142_at	Ints8	integrator complex subunit 8	1.031	0.423	0.0018	0.7134	No	1.0107	0.751	0.0008	0.578	No	No
104143_at	Copz2	coatomer protein complex, subunit zeta 2	1.077	0.1133	0.0008	0.1311	No	1.0549	0.0392	0	0.0165	No	No
104144_at	Gtpbp2	GTP binding protein 2	0.9628	0.1965	0.0441	0.3681	No	0.9317	0.02	0.632	0.0219	No	No
104145_at	Tcof1	Treacher Collins Franceschetti syndrome 1, homolog	0.9556	0.1274	0.0062	0.9847	No	0.963	0.435	0.657	0.366	No	No
104146_at	Rasip1	Ras interacting protein 1	0.9473	0.3187	0.0036	0.0909	No	1.1085	0.0518	0.0084	0.0063	No	No

104147_at	Nans	N-acetylneuraminic acid synthase (sialic acid synthase)	1.0036	0.9304	0.0016	0.0687	No	1.0367	0.418	0.0001	0.0414	No	No
104148_at	H6pd	hexose-6-phosphate dehydrogenase (glucose 1-dehydrogenase)	0.9399	0.0774	0.0874	0.2211	No	0.9771	0.423	0.321	0.654	No	No
104149_at	Nfkbia	nuclear factor of kappa light chain gene enhancer in B-cells inhibitor, alpha	0.769	0.0129	0.0701	0.1676	No	0.7568	0.0035	0.0733	0.688	Yes	Yes
104150_at	Lrch4	leucine-rich repeats and calponin homology (CH) domain containing 4	1.0437	0.2151	0.0855	0.9923	No	1.0035	0.918	0.0443	0.669	No	No
104151_at	Lrch4	leucine-rich repeats and calponin homology (CH) domain containing 4	0.9653	0.0722	0.7664	0.8335	No	0.9356	0.117	0.962	0.628	No	No
104152_at	Stk40	serine/threonine kinase 40	0.9821	0.6747	0.6741	0.9841	No	0.9923	0.816	0.11	0.816	No	No
104153_at	Ivd	isovaleryl coenzyme A dehydrogenase	0.9486	0.1142	0.1902	0.124	No	0.9369	0.0296	0.128	0.0138	No	No
104154_at	Trp53	transformation related protein 53	1.0532	0.4977	0.2501	0.3937	No	1.0146	0.813	0.149	0.542	No	No
104155_f_at	Atf3	activating transcription factor 3	1.0356	0.4828	0.1902	0.8146	No	1.0118	0.524	0.0041	0.121	No	No
104156_r_at	Atf3	activating transcription factor 3	0.9927	0.8474	0.0028	0.6459	No	1.0776	0.183	0.0008	0.0474	No	No
104157_at	C78212	expressed sequence C78212	0.9434	0.4401	0.9719	0.3671	No	0.9987	0.983	0.914	0.89	No	No
104158_at	Snw1	SNW domain containing 1	0.8925	0.151	0.9698	0.0615	No	0.9025	0.0534	0.0044	0.0068	No	No
104159_at	Ceacam9	CEA-related cell adhesion molecule 9	1.0026	0.9052	0.4943	0.8531	No	1.1456	0.172	0.0294	0.255	No	No
104160_at	Acp1	acid phosphatase 1, soluble	0.9614	0.5083	0.1477	0.5474	No	0.9946	0.904	0.536	0.65	No	No
104161_at	Cpsf2	cleavage and polyadenylation specific factor 2	0.9558	0.4451	0.0053	0.3744	No	0.9582	0.181	0.0105	0.0502	No	No
104163_at	Ipo8	importin 8	0.9487	0.4515	0.0217	0.5149	No	1.0059	0.651	0.0007	0.0961	No	No
104164_at	Tysnd1	trypsin domain containing 1	0.9522	0.4124	0.0466	0.0691	No	0.9311	0.0071	0.858	0.94	Yes	No
104165_at	Vnn1	vanin 1	0.9317	0.3371	0.0648	0.8135	No	0.933	0.221	0.0526	0.237	No	No
104166_at	Rage	renal tumor antigen	0.9631	0.614	0.2734	0.6415	No	0.9498	0.331	0.0281	0.238	No	No
104168_at	Actr2	ARP2 actin-related protein 2 homolog (yeast)	1.1284	0.0452	0.4295	0.7621	No	1.0996	0.352	0.0469	0.863	No	No
104169_at	Zic1	zinc finger protein of the cerebellum 1	1.1241	0.1673	0.0011	0.3087	No	1.1143	0.0616	0.0002	0.77	No	No
104170_at	Mapk8ip1	mitogen activated protein kinase 8 interacting protein 1	1.0464	0.3189	0.0367	0.3413	No	0.9732	0.0512	0.0001	0.0102	No	No
104171_f_at	Crygd	crystallin, gamma D	1.0063	0.9102	0.008	0.4209	No	1.0116	0.907	0.605	0.576	No	No
104172_at	Folr2	folate receptor 2 (fetal)	0.9813	0.5582	0.5094	0.6006	No	1.0358	0.517	0.503	0.992	No	No
104173_at	Ms4a1	membrane-spanning 4-domains, subfamily A, member 1	0.9819	0.5806	0.7968	0.2375	No	1.0158	0.843	0.749	0.888	No	No
104174_at	Enpp1	ectonucleotide pyrophosphatase/phosphodiesterase 1	0.8728	0.0093	0.0088	0.0082	No	0.8502	0.0364	0.168	0.0486	No	No
104175_at	Dlgh4	discs, large homolog 4 (Drosophila)	0.9822	0.6932	0.0634	0.1244	No	1.0844	0.155	0.317	0.0333	No	No
104176_at	LOC637741	hypothetical protein LOC637741	0.985	0.8109	0.4685	0.6128	No	0.9989	0.964	0.42	0.404	No	No
104177_at	Rsad2	radical S-adenosyl methionine domain containing 2	0.9671	0.3851	0.0004	0.8021	No	0.9395	0.593	0.186	0.698	No	No
104179_at	AI788669	expressed sequence AI788669	1.0643	0.6744	0.093	0.9815	No	1.0319	0.85	0.392	0.342	No	No
104180_at	Rac3	RAS-related C3 botulinum substrate 3	1.0435	0.2502	0.0124	0.069	No	0.9959	0.908	0.0873	0.51	No	No
104181_at	Vnn3	vanin 3	0.9848	0.7045	0.2167	0.2889	No	1.1568	0.0938	0.0441	0.151	No	No
104182_at	Hgfac	hepatocyte growth factor activator	1.0177	0.6174	0.5655	0.5566	No	1.0508	0.419	0.942	0.572	No	No
104183_at	---	---	0.9904	0.8768	0.0341	0.1999	No	0.9839	0.516	0.0023	0.114	No	No
104184_at	Nppb	natriuretic peptide precursor type B	0.9862	0.7904	0.5917	0.0992	No	0.9648	0.667	0.943	0.755	No	No
104185_at	Prss32	protease, serine, 32	1.0329	0.5161	0.3027	0.8851	No	1.0426	0.455	0.676	0.223	No	No
104186_at	Msl2l1	male-specific lethal 2-like 1 (Drosophila)	0.9236	0.336	0.0162	0.5402	No	0.9612	0.42	0.0028	0.297	No	No
104187_at	Mbd6	methyl-CpG binding domain protein 6	1.0273	0.4342	0.0062	0.9259	No	0.9825	0.394	0.0103	0.29	No	No
104188_at	Notch2	Notch gene homolog 2 (Drosophila)	0.9095	0.1938	0.0038	0.234	No	0.947	0.493	0.746	0.0137	No	No
104189_at	Traf6	Tnf receptor-associated factor 6	0.9664	0.5902	0.3926	0.9259	No	0.9489	0.381	0.653	0.58	No	No
104190_at	Traf6	Tnf receptor-associated factor 6	1.0766	0.1963	0.0948	0.1985	No	1.1173	0.165	0.116	0.652	No	No
104191_at	Zxda	zinc finger, X-linked, duplicated A	1.0254	0.5258	0.0265	0.1357	No	1.0471	0.331	0.353	0.177	No	No
104192_at	Tpr	translocated promoter region	0.8259	0.0682	0.0025	0.6985	No	0.8332	0.228	0.0049	0.883	No	No
104193_at	2810485I05 Rik	RIKEN cDNA 2810485I05 gene	0.9591	0.4547	0.0293	0.0707	No	0.9686	0.769	0.018	0.0935	No	No
104194_at	Heph	hephaestin	0.9722	0.7333	0.0449	0.6061	No	0.998	0.913	0.339	0.273	No	No
104195_at	5730408K05 Rik	RIKEN cDNA 5730408K05 gene	0.909	0.0125	0.0043	0.4831	No	0.9915	0.698	0.0055	0.823	No	No
104196_at	Zmym4	zinc finger, MYM-type 4	0.9873	0.4202	0	0.0227	No	0.994	0.903	0.0001	0.172	No	No
104197_at	Rbm9	RNA binding motif protein 9	1.0034	0.9349	0.0639	0.7843	No	0.9288	0.195	0.669	0.227	No	No
104198_at	Cdcp1	CUB domain containing protein 1	1.0522	0.2438	0.6824	0.2204	No	1.1227	0.0336	0.0171	0.0057	No	No
104199_at	Tigd5	tigger transposable element derived 5	1.0249	0.7042	0.748	0.5011	No	1.0675	0.281	0.136	0.18	No	No
104200_at	Slc5a6	solute carrier family 5 (sodium-dependent vitamin transporter), member 6	1.0205	0.6474	0.4612	0.5369	No	1.0361	0.434	0.16	0.833	No	No
104201_at	Plac1	placental specific protein 1	1.0036	0.9393	0.0691	0.8228	No	1.0292	0.547	0.25	0.136	No	No
104202_at	Tmem1	transmembrane protein 1	1.0103	0.8298	0.0346	0.6851	No	0.975	0.353	0.0025	0.125	No	No
104205_at	Agc1	aggrecan 1	0.8628	0.025	0.0033	0.6038	No	0.8682	0.0077	0.0002	0.0662	Yes	Yes
104206_at	5730557B15 Rik	RIKEN cDNA 5730557B15 gene	0.995	0.8886	0.3403	0.9343	No	1.0667	0.102	0.0208	0.0624	No	No
104207_at	6330505N24 Rik	RIKEN cDNA 6330505N24 gene	1.0363	0.5652	0.7787	0.5371	No	1.04	0.613	0.473	0.784	No	No
104208_at	Pik4ca	phosphatidylinositol 4-kinase, catalytic, alpha polypeptide	1.0272	0.3477	0.0074	0.2615	No	0.973	0.326	0.0015	0.0472	No	No
104209_at	Cyhr1	cysteine and histidine rich 1	0.9927	0.845	0.0119	0.6447	No	0.9817	0.752	0.692	0.137	No	No
104210_at	Itga3	integrin alpha 3	1.0456	0.1553	0.0195	0.2617	No	1.0847	0.16	0.0161	0.0325	No	No
104211_at	Itga3	integrin alpha 3	0.9851	0.8154	0.061	0.9165	No	0.991	0.936	0.0482	0.454	No	No
104212_at	Lrpprc	leucine-rich PPR-motif containing	1.0563	0.2019	0.003	0.5106	No	0.9844	0.675	0.0134	0.274	No	No
104213_at	---	---	1.0119	0.8964	0.1626	0.484	No	1.0654	0.223	0.0036	0.391	No	No
104214_at	Slc7a8	solute carrier family 7 (cationic amino acid transporter, y+ system), member 8	1.0295	0.4099	0.567	0.968	No	1.012	0.838	0.0283	0.13	No	No
104215_at	Atf6	activating transcription factor 6	0.9234	0.4219	0.594	0.8687	No	0.9126	0.238	0.52	0.996	No	No
104216_at	Shoc2	soc-2 (suppressor of clear) homolog (C. elegans)	0.8702	0.2386	0.0156	0.5069	No	0.8671	0.0763	0.0068	0.881	No	No
104217_at	Gdpd3	glycerophosphodiester phosphodiesterase domain containing 3	0.9174	0.0658	0.0174	0.0801	No	0.8986	0.091	0.438	0.0737	No	No
104218_s_at	Pb1	polybromo 1	0.975	0.7682	0.021	0.4472	No	0.9921	0.795	0.0399	0.239	No	No
104219_f_at	2610205E22 Rik	RIKEN cDNA 2610205E22 gene	0.959	0.5487	0.9843	0.9027	No	0.9463	0.447	0.881	0.365	No	No
104220_at	LOC670044	similar to Mothers against decapentaplegic homolog 6 (SMAD 6) (Mothers against DPP homolog 6) (Smad6) (Mad homolog 7)	0.9781	0.6414	0.0244	0.023	No	0.9423	0.337	0.0038	0.0085	No	No
104221_at	Slc7a5	solute carrier family 7 (cationic amino acid transporter, y+ system), member 5	0.8906	0.0855	0.4389	0.4421	No	0.9242	0.126	0.734	0.464	No	No
104222_f_at	Ggps1	geranylgeranyl diphosphate synthase 1	1.0108	0.7732	0.0599	0.3652	No	1.074	0.175	0.0119	0.342	No	No
104225_at	Snx5	sorting nexin 5	1.0826	0.12	0.0004	0.0325	No	1.0109	0.685	0.0025	0.26	No	No
104227_at	Gpr97	G protein-coupled receptor 97	0.9639	0.2043	0.9659	0.1066	No	1.1433	0.0782	0.0103	0.181	No	No
104228_at	---	PREDICTED: Mus musculus similar to Rap guanine nucleotide exchange factor 2 (PDZ domain containing guanine nucleotide exchange factor 1) (PDZ-GEF1) (LOC620996), mRNA	0.8158	0.2264	0.0027	0.9399	No	0.7587	0.16	0.0043	0.906	No	No
104229_at	2610200G18 Rik	RIKEN cDNA 2610200G18 gene	0.9937	0.895	0.1198	0.5829	No	1.0104	0.614	0.0054	0.0429	No	No
104230_at	Dclre1a	DNA cross-link repair 1A, PSO2 homolog (S. cerevisiae)	0.9238	0.1851	0.0291	0.8771	No	0.9181	0.132	0.0188	0.179	No	No
104231_at	Hnrph3 /// LOC669773	heterogeneous nuclear ribonucleoprotein H3 /// similar to heterogeneous nuclear ribonucleoprotein H3 isoform a	1.0711	0.1601	0.6696	0.1347	No	1.1138	0.0095	0.395	0.183	Yes	No
104232_at	Gjb3	gap junction membrane channel protein beta 3	0.9637	0.3715	0.3165	0.0959	No	1.0599	0.0719	0.0005	0.0542	No	No
104233_at	Tead1	TEA domain family member 1	1.0784	0.1903	0.7788	0.136	No	1.103	0.12	0.503	0.0479	No	No
104234_at	Mrps25	mitochondrial ribosomal protein S25	1.0113	0.9029	0.0735	0.2894	No	1.1264	0.0638	0.0006	0.417	No	No
104235_at	Unc84b	unc-84 homolog B (C. elegans)	0.8334	0.0197	0.0123	0.1997	No	0.8586	0.0784	0.398	0.626	No	No
104237_at	Pcbd2	pterin 4 alpha carbinolamine dehydratase/dimerization cofactor of hepatocyte nuclear factor 1 alpha (TCF1) 2	0.9836	0.7349	0.5849	0.1191	No	0.9474	0.0855	0.0081	0.552	No	No
104238_at	Art5	ADP-ribosyltransferase 5	0.9315	0.227	0.2523	0.8811	No	0.9425	0.183	0.0395	0.56	No	No
104239_at	Tspan32	tetraspanin 32	1.0337	0.3125	0.0029	0.0529	No	1.0356	0.254	0.0008	0.403	No	No
104240_at	Cutl1	cut-like 1 (Drosophila)	1.0078	0.928	0.1645	0.8671	No	1.0019	0.708	0.0016	0.177	No	No
104241_at	Mef2d	myocyte enhancer factor 2D	1.0229	0.7436	0.0012	0.1153	No	1.0244	0.547	0.0007	0.551	No	No

104242_f_at	6530401N04 Rik	RIKEN cDNA 6530401N04 gene	1.0107	0.9144	0.0166	0.4903	No	1.0056	0.838	0.0052	0.358	No	No
104243_r_at	6530401N04 Rik	RIKEN cDNA 6530401N04 gene	1.0468	0.1553	0.0024	0.0906	No	0.9463	0.265	0.0603	0.755	No	No
104244_at	Mark2	MAP/microtubule affinity-regulating kinase 2	1.0191	0.5937	0.2101	0.4818	No	1.0141	0.597	0.109	0.785	No	No
104245_at	Rsn	restin (Reed-Steinberg cell-expressed intermediate filament-associated protein)	1.0068	0.7281	0.0095	0.5205	No	1.105	0.0517	0.0147	0.0205	No	No
104246_at	Asxl1	additional sex combs like 1 (Drosophila)	1.0527	0.0318	0.001	0.1708	No	1.0395	0.12	0.0009	0.174	No	No
104247_at	Zfyve19	zinc finger, FYVE domain containing 19	0.9885	0.7585	0.0027	0.2103	No	1.0696	0.0792	0.0008	0.0523	No	No
104248_at	Ssr3	signal sequence receptor, gamma	1.0424	0.5567	0.096	0.2268	No	0.9923	0.851	0.476	0.229	No	No
104249_g_at	Ssr3	signal sequence receptor, gamma	0.9459	0.4825	0.1453	0.1601	No	0.941	0.423	0.262	0.291	No	No
104250_at	Lrrc8a	leucine rich repeat containing 8A	0.8541	0.0157	0.0832	0.3615	No	0.871	0.156	0.277	0.789	No	No
104252_at	AU020206	expressed sequence AU020206	0.8573	0.0096	0.0159	0.9137	Yes	0.8622	0.0091	0.0358	0.723	Yes	Yes
104253_at	Mllt1	myeloid/lymphoid or mixed lineage-leukemia translocation to 1 homolog (Drosophila)	0.9771	0.2492	0.0111	0.5183	No	0.9629	0.564	0.085	0.536	No	No
104254_at	Ralgps2	Ral GEF with PH domain and SH3 binding motif 2	0.8578	0.0168	0.0108	0.5326	No	0.8965	0.0498	0.0018	0.771	No	No
104255_at	Fhod3	formin homology 2 domain containing 3	1.0777	0.2756	0.0123	0.7574	No	1.0863	0.0891	0.0069	0.148	No	No
104256_at	Pscdbp	pleckstrin homology, Sec7 and coiled-coil domains, binding protein	0.8495	0.0171	0.0312	0.1187	No	0.9783	0.685	0.0832	0.801	No	No
104257_g_at	Pscdbp	pleckstrin homology, Sec7 and coiled-coil domains, binding protein	0.9476	0.412	0.0124	0.0225	No	0.8908	0.0661	0.0303	0.0459	No	No
104258_at	Acyp2	acylphosphatase 2, muscle type	0.9867	0.6828	0.0008	0.2904	No	0.9994	0.665	0.0009	0.0327	No	No
104259_at	Cbx5	chromobox homolog 5 (Drosophila HP1a)	1.0563	0.2378	0.7181	0.2305	No	1.0025	0.866	0.124	0.043	No	No
104260_at	AW112037	expressed sequence AW112037	0.9986	0.9829	0.5897	0.9588	No	1.1195	0.0686	0.209	0.0959	No	No
104261_at	Asxl1	additional sex combs like 1 (Drosophila)	1.0285	0.3017	0.0887	0.1064	No	1.0549	0.473	0.203	0.307	No	No
104262_at	Pthlh	parathyroid hormone-like peptide	0.9626	0.5913	0.008	0.8234	No	0.961	0.511	0.0007	0.325	No	No
104263_at	Zmym6	zinc finger, MYM-type 6	0.9895	0.9102	0.1424	0.8253	No	0.9863	0.871	0.0621	0.535	No	No
104264_at	Lrba	LPS-responsive beige-like anchor	0.9546	0.2035	0.0017	0.5066	No	0.9825	0.57	0.0003	0.0783	No	No
104265_at	Kdr	kinase insert domain protein receptor	1.0418	0.0008	0	0.0032	No	0.9929	0.829	0.0246	0.638	No	No
104266_at	Gm98	gene model 98, (NCBI)	0.9549	0.0023	0.6027	0.0401	Yes	1.0061	0.978	0.003	0.052	No	No
104267_at	Slc23a2	solute carrier family 23 (nucleobase transporters), member 2	0.9632	0.3236	0.0008	0.2523	No	1.0415	0.167	0.0194	0.0352	No	No
104268_at	Il6ra	interleukin 6 receptor, alpha	0.9295	0.0738	0.0402	0.8755	No	0.9805	0.746	0.011	0.0942	No	No
104269_at	Syt5	synaptotagmin V	0.9445	0.3405	0.1906	0.9373	No	0.9838	0.145	0.0001	0.0324	No	No
104270_at	Adrbk1	adrenergic receptor kinase, beta 1	0.9711	0.5045	0.0451	0.2568	No	1.0536	0.294	0.0227	0.805	No	No
104271_at	Admr	adrenomedullin receptor	0.9898	0.5601	0.0689	0.0856	No	1.0012	0.972	0.0936	0.587	No	No
104272_s_at	Map3k4	mitogen activated protein kinase kinase kinase 4	0.9876	0.8104	0.0233	0.2572	No	0.9122	0.172	0.0057	0.379	No	No
104273_at	Baat	bile acid-Coenzyme A: amino acid N-acyltransferase	0.987	0.7993	0.2845	0.7813	No	0.9881	0.939	0.0269	0.0217	No	No
104274_at	Cpne3	copine III	0.9555	0.1009	0.0001	0.1154	No	0.9406	0.0142	0	0.861	No	No
104275_g_at	Trp53	transformation related protein 53	0.9497	0.3458	0.0043	0.898	No	0.9285	0.024	0.0001	0.61	No	No
104276_at	Glipr2	GLI pathogenesis-related 2	1.0151	0.6939	0.0213	0.9543	No	1.0862	0.0112	0.0006	0.019	No	No
104277_at	Alg2	asparagine-linked glycosylation 2 homolog (yeast, alpha-1,3-mannosyltransferase)	1.0608	0.1082	0.0615	0.1147	No	1.0976	0.0058	0.0108	0.0046	No	No
104279_at	Polr2f	polymerase (RNA) II (DNA directed) polypeptide F	1.0048	0.8959	0.0003	0.0445	No	1.0199	0.315	0.0004	0.0398	No	No
104280_at	Sncg	synuclein, gamma	0.9793	0.7435	0.0023	0.4706	No	1.0206	0.831	0.0008	0.609	No	No
104282_at	AW112037	expressed sequence AW112037	1.0236	0.6325	0.0768	0.0609	No	0.9604	0.344	0.252	0.0185	No	No
104283_at	Tbc1d15	TBC1 domain family, member 15	0.9535	0.5777	0.9065	0.4323	No	0.9151	0.199	0.269	0.938	No	No
104284_at	5031439G07 Rik	RIKEN cDNA 5031439G07 gene	1.0506	0.0295	0.0621	0.0052	No	0.9653	0.379	0.0441	0.0526	No	No
104285_at	Hmgcr	3-hydroxy-3-methylglutaryl-Coenzyme A reductase	1.0218	0.7768	0.0342	0.9479	No	1.0013	0.979	0.0341	0.541	No	No
104286_at	Slc38a4	solute carrier family 38, member 4	0.9536	0.4262	0.0344	0.9348	No	0.9491	0.0644	0.618	0.0957	No	No
104287_at	Senp3	SUMO/sentrin specific peptidase 3	1.0599	0.0767	0.0016	0.256	No	1.0114	0.516	0.0008	0.216	No	No
104288_at	Cul4a	cullin 4A	0.941	0.1458	0.0132	0.2369	No	0.9396	0.302	0.0756	0.471	No	No
104289_at	Abcb8	ATP-binding cassette, sub-family B (MDR/TAP), member 8	1.0104	0.8034	0.0116	0.8437	No	1.0399	0.332	0.0326	0.146	No	No
104290_at	Casp8ap2	caspase 8 associated protein 2	0.9901	0.8805	0.0637	0.8842	No	1.0605	0.408	0.16	0.667	No	No
104291_at	Casp8ap2	caspase 8 associated protein 2	1.1904	0.0007	0.0007	0.0235	Yes	1.1121	0.0601	0.0301	0.087	No	No
104292_at	Eya2	eyes absent 2 homolog (Drosophila)	0.9415	0.2299	0.0275	0.8488	No	0.9719	0.426	0.0013	0.904	No	No
104293_at	Klhl21	kelch-like 21 (Drosophila)	0.8936	0.0007	0	0.0063	No	0.8814	0.0063	0.0004	0.0176	Yes	No
104294_at	Ibtk	inhibitor of Bruton agammaglobulinemia tyrosine kinase	0.9761	0.7388	0.0068	0.328	No	0.9874	0.771	0.0001	0.119	No	No
104296_at	Hnrpul2	heterogeneous nuclear ribonucleoprotein U-like 2	0.9366	0.4595	0.1231	0.3352	No	0.9005	0.215	0.685	0.638	No	No
104297_at	Ipo11	importin 11	0.9576	0.5152	0.0564	0.5743	No	1.0018	0.915	0.0115	0.677	No	No
104298_at	---	---	0.9308	0.237	0.0273	0.6603	No	0.9832	0.771	0.13	0.249	No	No
104299_at	Zdhhc14	zinc finger, DHHC domain containing 14	0.9957	0.9204	0.0329	0.7712	No	1.158	0.0448	0.0486	0.344	No	No
104300_at	Iqgap1	IQ motif containing GTPase activating protein 1	0.9127	0.0019	0.0023	0.0569	Yes	0.933	0.0016	0.0014	0.0206	Yes	Yes
104301_at	2410018G20 Rik	RIKEN cDNA 2410018G20 gene	0.9757	0.5532	0.3263	0.1425	No	0.9565	0.22	0.328	0.0394	No	No
104302_f_at	Commd9	COMM domain containing 9	0.9472	0.1309	0.0632	0.0112	No	0.9935	0.765	0.0887	0.0382	No	No
104303_i_at	Polr3k	polymerase (RNA) III (DNA directed) polypeptide K	1.2465	0.0188	0.4105	0.3881	No	1.2276	0.0828	0.0421	0.175	No	No
104304_r_at	Polr3k	polymerase (RNA) III (DNA directed) polypeptide K	1.2744	0.0538	0.0062	0.3195	No	1.2225	0.0025	0.0001	0.283	Yes	No
104305_at	Rarsl	arginyl-tRNA synthetase-like	0.9829	0.5867	0.0003	0.0621	No	1.0212	0.645	0.0013	0.149	No	No
104306_at	Dpp3	dipeptidylpeptidase 3	1.018	0.5625	0.0331	0.1114	No	0.9882	0.841	0.26	0.134	No	No
104308_at	Itgax	integrin alpha X	0.9892	0.8311	0.9488	0.4556	No	0.937	0.177	0.0108	0.439	No	No
104310_at	G6pc3	glucose 6 phosphatase, catalytic, 3	0.9995	0.9901	0.1966	0.7009	No	1.006	0.678	0.0137	0.0191	No	No
104311_at	Ubxd2	UBX domain containing 2	0.9677	0.428	0.0012	0.0824	No	0.8979	0.195	0.758	0.222	No	No
104312_at	Abhd14a	abhydrolase domain containing 14A	0.9441	0.1883	0.0041	0.5656	No	0.9612	0.0367	0	0.0647	No	No
104313_at	Pgm2	phosphoglucomutase 2	0.9932	0.7985	0.6961	0.0611	No	0.9767	0.618	0.452	0.533	No	No
104314_r_at	1110032A03 Rik	RIKEN cDNA 1110032A03 gene	1.1192	0.1007	0.0006	0.0223	No	1.0591	0.0534	0.0003	0.005	No	No
104315_at	Arhgap1	Rho GTPase activating protein 1	0.9976	0.9187	0.3077	0.1567	No	1.1054	0.138	0.0529	0.438	No	No
104316_at	Gna13	guanine nucleotide binding protein, alpha 13	0.9983	0.9767	0.7211	0.9316	No	0.9408	0.263	0.449	0.49	No	No
104317_at	Cse1l	chromosome segregation 1-like (S. cerevisiae)	1.0391	0.3932	0.0038	0.9597	No	1.0091	0.964	0.233	0.912	No	No
104318_at	Pex5	peroxisome biogenesis factor 5	0.9822	0.5703	0.0088	0.1118	No	0.9945	0.835	0.19	0.0479	No	No
104320_at	Pdxk	pyridoxal (pyridoxine, vitamin B6) kinase	0.9882	0.615	0.0002	0.7698	No	0.9944	0.891	0.937	0.562	No	No
104322_at	Ckap2	cytoskeleton associated protein 2	0.9754	0.4864	0.0006	0.4036	No	0.9465	0.184	0.002	0.136	No	No
104323_at	Khk	ketohexokinase	0.9659	0.3537	0.9002	0.0136	No	1.0015	0.964	0.697	0.0959	No	No
104324_at	Tardbp	TAR DNA binding protein	1.0175	0.8212	0.0021	0.1092	No	0.9096	0.0179	0.0008	0.0467	No	No
104325_at	Mrap	melanocortin 2 receptor accessory protein	0.9739	0.6138	0.5045	0.4715	No	1.0473	0.605	0.81	0.448	No	No
104326_at	Adpgk	ADP-dependent glucokinase	1.0762	0.0992	0.6634	0.3082	No	1.073	0.196	0.927	0.685	No	No
104327_at	AI265322	expressed sequence AI265322	1.0025	0.9816	0.0147	0.3348	No	0.9506	0.678	0.0004	0.1	No	No
104328_at	Aqp9	aquaporin 9	1.0106	0.912	0.2014	0.9686	No	1.0726	0.219	0.219	0.871	No	No
104329_at	Arid1a /// LOC669477 /// LOC675933	AT rich interactive domain 1A (Swi1 like) /// similar to AT rich interactive domain 1A isoform a /// similar to AT rich interactive domain 1A isoform a	1.0258	0.717	0.9154	0.4516	No	0.9741	0.776	0.0565	0.756	No	No
104330_g_at	Arid1a /// LOC669477 /// LOC675933	AT rich interactive domain 1A (Swi1 like) /// similar to AT rich interactive domain 1A isoform a /// similar to AT rich interactive domain 1A isoform a	1.0358	0.6198	0.017	0.3245	No	0.9677	0.412	0.17	0.192	No	No
104331_at	Arid1a /// LOC669477 /// LOC675933	AT rich interactive domain 1A (Swi1 like) /// similar to AT rich interactive domain 1A isoform a /// similar to AT rich interactive domain 1A isoform a	1.0234	0.5672	0.0005	0.3352	No	1.0507	0.0545	0.0043	0.0643	No	No
104332_at	Ptcd2	pentatricopeptide repeat domain 2	0.9018	0.0235	0.1641	0.0263	No	0.8892	0.0267	0.193	0.51	No	No
104333_at	D17H6S56E-5	DNA segment, Chr 17, human D6S56E 5	0.9622	0.2185	0.0131	0.0533	No	0.9077	0.168	0.0759	0.357	No	No
104334_at	2310004I24 Rik	RIKEN cDNA 2310004I24 gene	0.9353	0.0312	0.774	0.6588	No	0.9956	0.946	0.0208	0.118	No	No
104335_at	Kifc3	kinesin family member C3	1.0366	0.3839	0.9877	0.3561	No	0.9338	0.0411	0.001	0.282	No	No
104336_at	Rbks	ribokinase	0.9563	0.3566	0.941	0.7309	No	1.0009	0.969	0.941	0.771	No	No
104337_f_at	Pkp2	plakophilin 2	0.9915	0.7901	0.0006	0.0199	No	0.9992	0.442	0.0004	0.0022	No	No
104338_r_at	Pkp2	plakophilin 2	1.0056	0.9171	0.05	0.7208	No	1.002	0.953	0.116	0.198	No	No

104339_at	Pygo2	pygopus 2	1.0103	0.8056	0.0136	0.1555	No	1.0028	0.975	0.0032	0.892	No	No
104340_at	Mbd1	methyl-CpG binding domain protein 1	1.0084	0.8934	0.8903	0.7033	No	1.0027	0.983	0.113	0.523	No	No
104341_at	2310014H01 Rik	RIKEN cDNA 2310014H01 gene	1.0104	0.6995	0.0745	0.8174	No	0.9321	0.0523	0.419	0.0317	No	No
104342_i_at	Pla2g12a	phospholipase A2, group XIIA	1.0188	0.6506	0.0001	0.2317	No	1.0488	0.354	0.0001	0.557	No	No
104343_f_at	Pla2g12a	phospholipase A2, group XIIA	1.1223	0.0006	0	0.0003	No	1.0834	0.0716	0	0.0228	No	No
104344_at	Klkb1	kallikrein B, plasma 1	1.0231	0.6932	0.3428	0.9622	No	0.9848	0.957	0.265	0.816	No	No
104345_at	Exoc7	exocyst complex component 7	1.024	0.5223	0.1039	0.8133	No	1.0469	0.0384	0.0342	0.234	No	No
104346_at	Acbd6	acyl-Coenzyme A binding domain containing 6	0.9617	0.6253	0.0446	0.7942	No	1.2951	0.0049	0.0015	0.0383	Yes	No
104348_at	Bre	brain and reproductive organ-expressed protein	0.9287	0.0757	0.4097	0.0935	No	0.9866	0.692	0.222	0.0205	No	No
104349_at	Snip1	Smad nuclear interacting protein 1	0.9959	0.8758	0.6834	0.465	No	0.9937	0.398	0.0026	0.0037	No	No
104350_at	Shc1	src homology 2 domain-containing transforming protein C1	1.0229	0.7665	0.0181	0.6238	No	0.9814	0.796	0.0022	0.787	No	No
104351_at	Gmppb	GDP-mannose pyrophosphorylase B	1.0461	0.1089	0.3768	0.3913	No	0.9548	0.2	0.0088	0.456	No	No
104352_at	Brd4	bromodomain containing 4	0.9806	0.7016	0.0138	0.0808	No	1.0158	0.763	0.0002	0.419	No	No
104353_at	2500002G23 Rik	RIKEN cDNA 2500002G23 gene	1.0088	0.9063	0.0249	0.1117	No	1.1009	0.204	0.0073	0.0368	No	No
104354_at	Csf1r	colony stimulating factor 1 receptor	0.9039	0.2143	0.6556	0.8959	No	1.0051	0.855	0.271	0.048	No	No
104355_at	Fus	fusion, derived from t(12;16) malignant liposarcoma (human)	0.9439	0.3959	0.9457	0.972	No	1.2491	0.436	0.162	0.603	No	No
104356_at	Taf9	TAF9 RNA polymerase II, TATA box binding protein (TBP)-associated factor	0.945	0.2557	0.4459	0.1897	No	0.9524	0.486	0.248	0.587	No	No
104358_at	D10Ertd610 e	DNA segment, Chr 10, ERATO Doi 610, expressed	1.0323	0.4391	0.0247	0.8833	No	1.0545	0.101	0.0621	0.462	No	No
104360_at	Svil	supervillin	0.949	0.2744	0.0044	0.8762	No	0.9261	0.01	0.0009	0.0309	Yes	No
104361_at	Zfp637	zinc finger protein 637	0.9194	0.1079	0.0207	0.0448	No	0.9089	0.0263	0.0953	0.0081	No	No
104362_at	Ube2o	ubiquitin-conjugating enzyme E2O	0.9725	0.5084	0.9368	0.4938	No	1.0279	0.246	0.0183	0.0093	No	No
104363_at	Cdsn	corneodesmosin	0.9235	0.1672	0.0524	0.7378	No	0.9475	0.378	0.0969	0.984	No	No
104364_at	Mapkapk5	MAP kinase-activated protein kinase 5	0.9679	0.455	0.0021	0.9609	No	0.9724	0.118	0.0009	0.0245	No	No
104365_at	Scamp2	secretory carrier membrane protein 2	0.997	0.9628	0.0226	0.3919	No	1.0376	0.348	0.0154	0.294	No	No
104366_at	BC039093	cDNA sequence BC039093	1.0844	0.2394	0.0957	0.6641	No	1.0497	0.403	0.0333	0.628	No	No
104367_at	---	---	1.1287	0.0209	0.0002	0.8572	No	1.1008	0.0526	0.0005	0.641	No	No
104368_at	Mapre3	microtubule-associated protein, RP/EB family, member 3	1.05	0.1097	0.1582	0.2559	No	0.9947	0.977	0.259	0.172	No	No
104369_at	Krt2-6a	keratin complex 2, basic, gene 6a	1.0106	0.8384	0.0074	0.0804	No	1.0221	0.664	0.0067	0.87	No	No
104370_s_at	Krt2-6a	keratin complex 2, basic, gene 6a	0.9711	0.5783	0.0035	0.0624	No	0.9759	0.91	0.0078	0.163	No	No
104371_at	Dgat1	diacylglycerol O-acyltransferase 1	0.9855	0.8237	0.2618	0.5752	No	0.9833	0.405	0.0005	0.0775	No	No
104372_at	Abhd8	abhydrolase domain containing 8	1.0901	0.0168	0.0025	0.9405	No	1.0251	0.354	0.098	0.395	No	No
104373_at	Aprin	androgen-induced proliferation inhibitor	1.0501	0.5357	0.0219	0.2281	No	0.8593	0.219	0.585	0.576	No	No
104374_at	Serpina3n	serine (or cysteine) peptidase inhibitor, clade A, member 3N	0.7825	0.0004	0	0.0025	No	0.8099	0.0005	0	0.0024	No	No
104375_at	Spock2	sparc/osteonectin, cwcv and kazal-like domains proteoglycan 2	0.8841	0.0007	0.0009	0.0009	No	0.9086	0.0381	0.559	0.0828	No	No
104376_at	Hdac5	histone deacetylase 5	0.9323	0.0667	0.0091	0.1296	No	1.0207	0.704	0.322	0.102	No	No
104378_at	Pon2	paraoxonase 2	1.0448	0.5046	0.0564	0.7329	No	1.01	0.743	0.0001	0.643	No	No
104380_at	Slc35a1	solute carrier family 35 (CMP-sialic acid transporter), member 1	1.0224	0.7908	0.511	0.8165	No	1.0505	0.298	0.819	0.14	No	No
104381_at	Nr1h3	nuclear receptor subfamily 1, group H, member 3	0.9682	0.2986	0.5631	0.5738	No	0.9448	0.104	0.0082	0.0138	No	No
104382_at	Mcpt4	mast cell protease 4	0.9406	0.1433	0.1287	0.2012	No	1.01	0.635	0.0194	0.219	No	No
104383_at	Crmp1	collapsin response mediator protein 1	1.0474	0.1891	0.0083	0.093	No	1.016	0.563	0.0423	0.397	No	No
104385_i_at	Itgav	Integrin alpha V	1.0303	0.7866	0.2957	0.2699	No	0.8997	0.647	0.067	0.468	No	No
104386_f_at	Itgav	integrin alpha V	0.9067	0.2757	0.2641	0.6058	No	0.8865	0.205	0.075	0.578	No	No
104387_at	Slc23a1	solute carrier family 23 (nucleobase transporters), member 1	0.9946	0.9204	0.162	0.6779	No	1.0294	0.599	0.541	0.376	No	No
104388_at	Ccl9	chemokine (C-C motif) ligand 9	0.8104	0.004	0.0024	0.025	Yes	0.8048	0.0001	0	0.0006	No	No
104389_at	1700017B05 Rik /// LOC640563	RIKEN cDNA 1700017B05 gene /// hypothetical protein LOC640563	1.0213	0.6777	0.6999	0.829	No	1.14	0.271	0.493	0.251	No	No
104390_at	Anp32a	acidic (leucine-rich) nuclear phosphoprotein 32 family, member A	1.0296	0.5387	0.1077	0.6954	No	1.1254	0.0944	0.0894	0.0573	No	No
104391_s_at	Clcn7	chloride channel 7	0.9071	0.0162	0.0005	0.0771	No	0.8925	0.0108	0.0002	0.025	No	No
104392_at	Clcn7	chloride channel 7	1.002	0.9315	0.033	0.049	No	0.9877	0.744	0.0038	0.548	No	No
104393_at	2810453I06 Rik	RIKEN cDNA 2810453I06 gene	0.938	0.1474	0.38	0.6287	No	0.961	0.303	0.176	0.59	No	No
104394_at	Abcb10	ATP-binding cassette, sub-family B (MDR/TAP), member 10	0.9558	0.1282	0.0615	0.6352	No	0.8684	0.0385	0.665	0.0868	No	No
104395_at	Itpkb	inositol 1,4,5-trisphosphate 3-kinase B	1.031	0.429	0.8044	0.0521	No	1.0381	0.367	0.872	0.0485	No	No
104396_at	Znrf2	zinc and ring finger 2	0.9777	0.6955	0.2546	0.7809	No	1.0859	0.433	0.121	0.637	No	No
104398_at	Tspan33	tetraspanin 33	0.966	0.2868	0.5804	0.0814	No	0.9517	0.521	0.887	0.799	No	No
104399_at	Cstf2	cleavage stimulation factor, 3' pre-RNA subunit 2	1.0411	0.3767	0.0007	0.1521	No	1.0029	0.583	0.0009	0.0328	No	No
104400_at	Ykt6	YKT6 homolog (S. Cerevisiae)	1.1163	0.0388	0.032	0.037	No	1.0732	0.146	0.106	0.0314	No	No
104401_at	6430526N21 Rik	RIKEN cDNA 6430526N21 gene	0.9322	0.0551	0.0723	0.2608	No	0.9347	0.408	0.0234	0.364	No	No
104402_at	Gtf3c4	general transcription factor IIIC, polypeptide 4	0.9992	0.977	0.001	0.0469	No	0.9862	0.62	0.0058	0.192	No	No
104403_at	---	---	0.9293	0.4157	0.0061	0.1981	No	0.9543	0.0488	0	0.0009	No	No
104404_at	Nat12	N-acetyltransferase 12	1.0094	0.86	0.0612	0.3765	No	0.9897	0.885	0.097	0.381	No	No
104405_at	---	Adult male pituitary gland cDNA, RIKEN full-length enriched library, clone:5330439J20 product:unclassifiable, full insert sequence	0.9423	0.1479	0.8961	0.9647	No	0.8041	0.003	0.0215	0.0137	Yes	No
104406_at	Ptges	prostaglandin E synthase	1.0921	0.0181	0.7724	0.2706	No	1.0479	0.0816	0.943	0.605	No	No
104407_at	Alcam	activated leukocyte cell adhesion molecule	0.9186	0.4896	0.0004	0.834	No	0.9595	0.49	0.0004	0.458	No	No
104408_s_at	Sox18	SRY-box containing gene 18	1.0006	0.9863	0.2827	0.0506	No	0.9585	0.195	0.0614	0.0265	No	No
104409_at	Grik5	glutamate receptor, ionotropic, kainate 5 (gamma 2)	0.9521	0.2039	0.0181	0.3784	No	1.0033	0.868	0.0275	0.269	No	No
104410_at	Midn	midnolin	0.9909	0.7992	0.0206	0.6343	No	1.0295	0.574	0.0201	0.208	No	No
104411_at	D930048N1 4Rik	RIKEN cDNA D930048N14 gene	1.0204	0.7893	0.0237	0.4601	No	1.0685	0.337	0.0068	0.83	No	No
104412_at	Gnai1	guanine nucleotide binding protein, alpha inhibiting 1	1.0318	0.6007	0.6345	0.7907	No	1.041	0.397	0.495	0.406	No	No
104413_at	Slc35d1	solute carrier family 35 (UDP-glucuronic acid/UDP-N-acetylgalactosamine dual transporter), member D1	0.9851	0.5783	0.0022	0.7009	No	1.0223	0.385	0.0006	0.745	No	No
104414_at	Gnao1	Guanine nucleotide binding protein, alpha o	1.0011	0.9751	0.9511	0.6552	No	1.0542	0.286	0.0633	0.281	No	No
104415_at	---	Adult male corpora quadrigemina cDNA, RIKEN full-length enriched library, clone:B230341P20 product:inferred: forkhead box P1, full insert sequence	0.9283	0.1302	0.0049	0.8194	No	0.9196	0.248	0.0093	0.511	No	No
104416_at	Flt4	FMS-like tyrosine kinase 4	0.9902	0.6684	0.1607	0.5778	No	1.0997	0.0185	0.0035	0.0483	No	No
104417_at	Flt4	FMS-like tyrosine kinase 4	1.0646	0.2102	0.0321	0.1065	No	1.0877	0.014	0.184	0.0866	No	No
104418_at	Znrf2	zinc and ring finger 2	1.0955	0.0158	0.0019	0.2616	No	1.0374	0.539	0.12	0.369	No	No
104419_at	Fndc3a	fibronectin type III domain containing 3a	1.0465	0.6257	0.0096	0.8017	No	1.004	0.885	0.0012	0.554	No	No
104420_at	Nos2	nitric oxide synthase 2, inducible, macrophage	0.9451	0.5237	0.8281	0.8322	No	0.8892	0.0069	0.0059	0.149	Yes	No
104421_at	Fmo3	flavin containing monooxygenase 3	0.9791	0.6673	0.5979	0.4013	No	1.0074	0.91	0.892	0.432	No	No
104422_at	Ptprn /// LOC669060	protein tyrosine phosphatase, receptor type, N /// similar to Receptor-type tyrosine-protein phosphatase-like N precursor (R-PTP-N) (PTP IA-2)	0.9999	0.9984	0.0676	0.2481	No	1.0168	0.546	0.0106	0.553	No	No
104423_at	Dtl	denticleless homolog (Drosophila)	1.1354	0.0109	0.0006	0.0054	No	1.1553	0.0102	0.0008	0.0092	No	No
104424_at	C9	complement component 9	1.0188	0.7178	0.2316	0.3176	No	1.034	0.643	0.0584	0.818	No	No
104425_at	Inadl	InaD-like (Drosophila)	0.9077	0.1439	0.0258	0.6947	No	0.8215	0.0272	0.592	0.619	No	No
104427_at	Sbf2	SET binding factor 2	0.9997	0.995	0.0003	0.6155	No	0.9939	0.717	0.0009	0.259	No	No
104428_s_at	Matk	megakaryocyte-associated tyrosine kinase	1.0689	0.2676	0.6334	0.8432	No	1.2075	0.0631	0.0714	0.174	No	No
104429_at	H2-Ob	histocompatibility 2, O region beta locus	1.0344	0.3711	0.0915	0.1143	No	1.0743	0.147	0.772	0.146	No	No
104430_at	Cd96	CD96 antigen	1.0266	0.6755	0.1591	0.3005	No	1.0644	0.0261	0.0051	0.003	No	No
104431_at	Prkcq	protein kinase C, theta	0.936	0.0605	0.1493	0.3525	No	0.9877	0.85	0.0426	0.6	No	No
104432_at	Rnd2	Rho family GTPase 2	1.0416	0.4705	0.507	0.3732	No	1.0747	0.178	0.455	0.127	No	No

104433_at	Atp13a2 /// LOC676620	ATPase type 13A2 /// similar to ATPase type 13A2	0.9947	0.8859	0.0874	0.1295	No	0.9758	0.33	0.902	0.192	No	No
104434_at	AA407659	expressed sequence AA407659	1.0497	0.4069	0.9606	0.4573	No	1.0812	0.156	0.671	0.379	No	No
104435_at	Igsf10	immunoglobulin superfamily, member 10	1.0269	0.5077	0.0022	0.2559	No	1.109	0.067	0.0056	0.101	No	No
104436_at	Il1f5	interleukin 1 family, member 5 (delta)	0.9567	0.469	0.3218	0.9383	No	0.8025	0.554	0.155	0.342	No	No
104437_at	Zfp30	zinc finger protein 30	0.9453	0.2612	0.0065	0.4037	No	0.9446	0.328	0.0174	0.368	No	No
104438_at	Zfp30	zinc finger protein 30	1.0371	0.4709	0.0205	0.0783	No	1.0482	0.264	0.004	0.0077	No	No
104439_at	Nat6	N-acetyltransferase 6	0.9767	0.695	0.0578	0.1903	No	0.9559	0.299	0.0062	0.895	No	No
104440_at	Ybx2	Y box protein 2	0.9465	0.4009	0.0847	0.2843	No	0.9127	0.149	0.133	0.091	No	No
104441_at	Trim3	tripartite motif protein 3	1.0673	0.1786	0.9062	0.0561	No	1.093	0.14	0.0252	0.0178	No	No
104442_at	Rab14	RAB14, member RAS oncogene family	0.9757	0.5762	0.0023	0.1543	No	1.0523	0.55	0.438	0.265	No	No
104443_at	Ccr7	chemokine (C-C motif) receptor 7	0.9879	0.873	0.0257	0.1487	No	1.1176	0.49	0.0605	0.694	No	No
104444_at	9430098E02 Rik	RIKEN cDNA 9430098E02 gene	0.9629	0.4427	0.2182	0.019	No	0.9812	0.442	0.0564	0.0993	No	No
104445_at	4631408O11 Rik	RIKEN cDNA 4631408O11 gene	1.0211	0.6354	0.0003	0.1621	No	1.02	0.976	0.0002	0.0983	No	No
104446_f_at	4933428G09 Rik	RIKEN cDNA 4933428G09 gene	1.0414	0.675	0.9586	0.1048	No	1.0273	0.69	0.0252	0.815	No	No
104447_r_at	4933428G09 Rik	RIKEN cDNA 4933428G09 gene	1.0884	0.0085	0.0006	0.1991	Yes	0.9905	0.749	0.0267	0.0972	No	No
104448_at	Mttp	microsomal triglyceride transfer protein	0.9996	0.9867	0.7299	0.2911	No	0.8372	0.175	0.078	0.166	No	No
104449_at	Glrb	glycine receptor, beta subunit	1.0576	0.2641	0.0006	0.5036	No	0.986	0.964	0.0045	0.319	No	No
104450_at	---	(ORF 1) and reverse transcriptase (ORF 2) genes	1.0117	0.7563	0.3048	0.3873	No	1.309	0.0249	0.505	0.471	No	No
104451_at	Slc11a2	solute carrier family 11 (proton-coupled divalent metal ion transporters), member 2	1.0061	0.8976	0.6132	0.5783	No	1.0396	0.795	0.178	0.418	No	No
104452_at	Fstl3	follistatin-like 3	1.0656	0.0477	0.2295	0.169	No	1.1132	0.004	0.344	0.0044	No	No
104453_at	Sap30l	SAP30-like	0.9466	0.2083	0.0063	0.019	No	0.908	0.0547	0.0012	0.0021	No	No
104454_at	Pms2	postmeiotic segregation increased 2 (S. cerevisiae)	0.9349	0.2372	0.6711	0.9051	No	0.9713	0.321	0.003	0.142	No	No
104455_at	Camk4	calcium/calmodulin-dependent protein kinase IV	1.0291	0.3811	0.3236	0.0333	No	1.1439	0.0453	0.514	0.011	No	No
104456_at	5730589K01 Rik	RIKEN cDNA 5730589K01 gene	0.8684	0.1162	0.0372	0.2517	No	0.9598	0.339	0.0252	0.0567	No	No
104457_at	Vps13c	Vacuolar protein sorting 13C (yeast)	1.044	0.2611	0.0053	0.5926	No	0.9861	0.923	0.114	0.746	No	No
104458_at	Tcf3	transcription factor 3	0.9824	0.4601	0.0823	0.3607	No	1.0486	0.294	0.188	0.101	No	No
104460_at	Cacna1g	calcium channel, voltage-dependent, T type, alpha 1G subunit	1.0216	0.636	0.0093	0.9879	No	1.2287	0.195	0.0701	0.872	No	No
104461_at	Pik3ca	phosphatidylinositol 3-kinase, catalytic, alpha polypeptide	0.9375	0.2586	0.044	0.709	No	0.911	0.0319	0.945	0.0289	No	No
104462_at	Hic1	hypermethylated in cancer 1	1.1227	0.0225	0.0029	0.1398	No	1.1315	0.0197	0.0022	0.132	No	No
104463_at	Utp20	UTP20, small subunit (SSU) processome component, homolog (yeast)	1.0086	0.836	0.7827	0.4351	No	0.8568	0.0058	0.0689	0.0104	Yes	No
104464_s_at	Kdelr3	KDEL (Lys-Asp-Glu-Leu) endoplasmic reticulum protein retention receptor 3	1.1635	0.1111	0.0421	0.3561	No	1.1252	0.0678	0.0023	0.229	No	No
104466_at	D4Wsu114e	DNA segment, Chr 4, Wayne State University 114, expressed	0.9911	0.7848	0.0962	0.6297	No	1.1781	0.0011	0.204	0.193	Yes	No
104467_at	Cpd	carboxypeptidase D	0.9473	0.3575	0.0026	0.0579	No	0.9561	0.545	0.0097	0.0755	No	No
104468_at	Incenp	inner centromere protein	0.9918	0.7125	0.002	0.4617	No	1.2426	0.0386	0.0013	0.0168	No	No
104469_at	Pdpn	podoplanin	1.0327	0.3981	0.0029	0.2761	No	0.9785	0.725	0.0018	0.828	No	No
104470_at	Utp14a	UTP14, U3 small nucleolar ribonucleoprotein, homolog A (yeast)	0.9433	0.142	0.7519	0.4371	No	1.0035	0.996	0.102	0.119	No	No
104471_at	LOC669168	similar to Histone deacetylase 6 (HD6) (Histone deacetylase mHDA2)	1.0373	0.4551	0.7966	0.8575	No	1.0547	0.0957	0.263	0.349	No	No
104473_at	Atpbd1b	ATP binding domain 1 family, member B	1.0324	0.3502	0.3654	0.2635	No	1.0048	0.836	0.001	0.744	No	No
104474_s_at	Oprl1	opioid receptor-like 1	0.9751	0.2009	0.0986	0.64	No	0.9349	0.0327	0.912	0.0502	No	No
104475_at	AA408278	expressed sequence AA408278	0.9555	0.4331	0.0963	0.3542	No	0.9528	0.137	0.106	0.0221	No	No
104476_at	Rbl1	retinoblastoma-like 1 (p107)	1.0121	0.7905	0.0057	0.2722	No	1.0764	0.241	0.12	0.475	No	No
104477_at	---	Transcribed locus	1.0029	0.9741	0.0008	0.2205	No	1.035	0.387	0.0001	0.0056	No	No
104479_at	P2ry2	purinergic receptor P2Y, G-protein coupled 2	0.9689	0.5776	0.0615	0.5068	No	0.9446	0.392	0.0303	0.683	No	No
104480_at	Dsg2	desmoglein 2	1.0234	0.2624	0.639	0.0032	No	1.1114	0.0138	0.0416	0.0167	No	No
104481_at	Eef2k	eukaryotic elongation factor-2 kinase	0.9387	0.2735	0.0079	0.0779	No	0.9561	0.425	0.0173	0.14	No	No
104482_at	Epim	epimorphin	0.9804	0.5299	0.6471	0.828	No	0.9763	0.174	0.0036	0.338	No	No
104483_at	Col9a1	procollagen, type IX, alpha 1	0.9165	0.028	0	0.4468	No	0.9082	0.0485	0.0001	0.18	No	No
104484_at	Sult2a2	sulfotransferase family 2A, dehydroepiandrosterone (DHEA)-preferring, member 2	0.9908	0.867	0.4678	0.2889	No	1.0117	0.847	0.605	0.684	No	No
104485_at	4930527D15 Rik	RIKEN cDNA 4930527D15 gene	0.9542	0.2096	0.3906	0.1301	No	1.0122	0.82	0.084	0.809	No	No
104486_at	A2m	alpha-2-macroglobulin	0.8855	0.0807	0.0273	0.0744	No	0.8522	0.029	0.129	0.0351	No	No
104487_at	H2afj	H2A histone family, member J	0.9428	0.2697	0.1348	0.6277	No	0.9806	0.919	0.0056	0.165	No	No
104488_at	5730407K14 Rik	RIKEN cDNA 5730407K14 gene	0.944	0.3223	0.0204	0.1451	No	0.9533	0.0625	0.0489	0.273	No	No
104489_at	Sntb2	syntrophin, basic 2	0.9746	0.1726	0.2648	0.4472	No	0.929	0.043	0.021	0.0681	No	No
104491_at	Phtf2	putative homeodomain transcription factor 2	1.0489	0.4828	0.0302	0.317	No	1.0808	0.41	0.206	0.478	No	No
104492_at	Ebf3	early B-cell factor 3	1.0461	0.4833	0.0134	0.0469	No	0.9925	0.88	0.0059	0.0598	No	No
104493_at	Nrp2	neuropilin 2	1.0443	0.0873	0.3118	0.0462	No	1.0921	0.31	0.754	0.567	No	No
104494_at	C820004L04 Rik	RIKEN cDNA C820004L04 gene	1.0964	0.1905	0.0595	0.3419	No	1.016	0.702	0.0351	0.456	No	No
104495_f_at	Klk1b5	kallikrein 1-related peptidase b5	0.8447	0.1154	0.1101	0.1843	No	0.9231	0.318	0.0059	0.633	No	No
104496_i_at	Klk1b8	kallikrein 1-related peptidase b8	0.9208	0.1758	0.1967	0.3085	No	0.8777	0.0254	0.529	0.0344	No	No
104497_f_at	Klk1b8	kallikrein 1-related peptidase b8	1.0246	0.738	0.1086	0.9767	No	0.9269	0.477	0.0059	0.183	No	No
104498_at	Homer1	homer homolog 1 (Drosophila)	1.0289	0.6434	0.0799	0.5062	No	1.0903	0.364	0.0532	0.315	No	No
104499_at	Homer1	homer homolog 1 (Drosophila)	0.8943	0.1921	0.2287	0.8954	No	0.9364	0.515	0.0005	0.0404	No	No
104500_at	Idua	iduronidase, alpha-L-	1.0348	0.48	0.0035	0.4309	No	1.0526	0.058	0.0002	0.0212	No	No
104501_at	Vapb	vesicle-associated membrane protein, associated protein B and C	1.0817	0.2373	0.0028	0.7348	No	1.0711	0.017	0.0056	0.498	No	No
104502_f_at	Ccdc94	coiled-coil domain containing 94	0.9672	0.2541	0.0213	0.9082	No	0.9732	0.256	0.0006	0.0759	No	No
104503_at	Pkn2	protein kinase N2	0.9366	0.0198	0.0009	0.1028	No	0.963	0.105	0.432	0.0747	No	No
104504_at	Scn8a	sodium channel, voltage-gated, type VIII, alpha	0.9238	0.1141	0.517	0.2614	No	0.8761	0.0706	0.0458	0.0443	No	No
104505_at	Scn8a	sodium channel, voltage-gated, type VIII, alpha	1.0665	0.2798	0.9053	0.4023	No	0.9787	0.522	0.0022	0.227	No	No
104506_at	Nr1i3	nuclear receptor subfamily 1, group I, member 3	1.0875	0.1949	0.4876	0.1129	No	1.0266	0.638	0.912	0.23	No	No
104507_g_at	Nr1i3	nuclear receptor subfamily 1, group I, member 3	0.9978	0.9719	0.0279	0.7764	No	0.991	0.524	0.0002	0.534	No	No
104508_at	Nckap1l	NCK associated protein 1 like	0.988	0.4683	0.0002	0.0029	No	0.9972	0.993	0.0544	0.553	No	No
104509_at	Ch25h	cholesterol 25-hydroxylase	1.0844	0.0053	0.5617	0.0029	No	1.036	0.627	0.608	0.0822	No	No
104510_at	Cacna2d1	calcium channel, voltage-dependent, alpha2/delta subunit 1	0.9156	0.0296	0.022	0.9982	No	0.9467	0.17	0.0286	0.0686	No	No
104512_at	Upk3b	uroplakin 3B	0.9839	0.779	0.0541	0.995	No	1.0239	0.728	0.0597	0.522	No	No
104513_at	2410004N09 Rik	RIKEN cDNA 2410004N09 gene	1.0872	0.3058	0.0382	0.6641	No	1.0276	0.72	0.204	0.938	No	No
104514_at	Epn1	epsin 1	0.9923	0.6713	0.0939	0.2612	No	0.9917	0.722	0.02	0.335	No	No
104515_at	5730508B09 Rik	RIKEN cDNA 5730508B09 gene	1.1308	0.1624	0.0391	0.0443	No	1.0995	0.131	0.0146	0.0413	No	No
104516_at	Cldn5	claudin 5	1.0353	0.416	0.6561	0.0106	No	1.021	0.554	0.0459	0.528	No	No
104518_at	Cryba1	crystallin, beta A1	1.0152	0.6938	0.0083	0.0989	No	0.9902	0.912	0.866	0.902	No	No
104519_at	Itih2	inter-alpha trypsin inhibitor, heavy chain 2	0.976	0.5591	0.0026	0.4479	No	0.9759	0.771	0.0092	0.392	No	No
104522_at	Itsn1	intersectin 1 (SH3 domain protein 1A)	1.0346	0.23	0.0236	0.9139	No	1.0386	0.0154	0.0016	0.0045	No	No
104523_at	Lrrc8c	leucine rich repeat containing 8 family, member C	0.9511	0.4164	0.6454	0.2079	No	0.8604	0.0907	0.0003	0.767	No	No
104524_at	Gltp	glycolipid transfer protein	1.0119	0.81	0.0067	0.0859	No	0.9721	0.872	0.0012	0.0151	No	No
104525_at	Ppm1m	protein phosphatase 1M	1.0521	0.0652	0.0004	0.0202	No	1.0096	0.329	0.0004	0.0053	No	No
104526_at	Tcfeb	transcription factor EB	0.9258	0.3213	0.2567	0.7144	No	0.9873	0.944	0.0408	0.511	No	No
104527_at	Rad51	RAD51 homolog (S. cerevisiae)	1.1221	0.0481	0.0731	0.0153	No	1.1108	0.0112	0.001	0.0034	No	No
104528_at	Znhit2	zinc finger, HIT domain containing 2	0.9873	0.7167	0.0645	0.8258	No	1.0157	0.695	0.0291	0.0417	No	No
104529_at	Caml	calcium modulating ligand	1.0654	0.0967	0.7674	0.4746	No	1.0281	0.652	0.249	0.156	No	No
104531_at	Prkcd	protein kinase C, delta	0.9947	0.7075	0.1732	0.0677	No	1.0291	0.286	0.0216	0.818	No	No
104532_at	Tomm34	translocase of outer mitochondrial membrane 34	1.0582	0.004	0.888	0.0069	No	0.9562	0.272	0.153	0.147	No	No
104533_at	Pim1	proviral integration site 1	0.9588	0.3767	0.2459	0.7365	No	1.0195	0.518	0.269	0.854	No	No
104534_at	Pgm1	phosphoglucomutase 1	1.2018	0.1126	0.101	0.8149	No	1.1494	0.0717	0.0132	0.603	No	No
104535_at	Yme1l1	YME1-like 1 (S. cerevisiae)	0.9778	0.6306	0.2355	0.4003	No	0.9667	0.721	0.0067	0.12	No	No

104536_at	Smad2	MAD homolog 2 (Drosophila)	1.0309	0.426	0.0025	0.8026	No	0.9865	0.331	0	0.109	No	No
104537_at	0610042C05 Rik	RIKEN cDNA 0610042C05 gene	0.9873	0.8659	0.3996	0.5034	No	1.0006	0.988	0.638	0.242	No	No
104538_at	Ptgis	prostaglandin I2 (prostacyclin) synthase	0.8263	0.0047	0.0001	0.1295	Yes	0.7952	0.0235	0.0002	0.0427	No	Yes
104539_at	Sult1b1	sulfotransferase family 1B, member 1	0.9334	0.3516	0.1433	0.549	No	0.9141	0.388	0.106	0.191	No	No
104541_at	Prtn3	proteinase 3	0.9507	0.5383	0.0155	0.5926	No	1.0088	0.881	0.031	0.356	No	No
104544_at	4930517K11 Rik	RIKEN cDNA 4930517K11 gene	0.9594	0.1007	0.1053	0.0312	No	1.0048	0.856	0.0468	0.372	No	No
104545_at	Csnk2a1	casein kinase II, alpha 1 polypeptide	0.9087	0.1115	0.0297	0.0855	No	0.974	0.511	0.258	0.62	No	No
104546_g_at	Csnk2a1	casein kinase II, alpha 1 polypeptide	0.9914	0.842	0.0195	0.8313	No	1.0621	0.0615	0.0131	0.0493	No	No
104547_at	Dhfr	dihydrofolate reductase	1.0122	0.6331	0.0044	0.6175	No	0.9785	0.607	0.5	0.655	No	No
104548_at	Phlda2	pleckstrin homology-like domain, family A, member 2	1.063	0.3076	0.0574	0.1961	No	1.0463	0.544	0.234	0.416	No	No
104549_at	Aqr	aquarius	0.9534	0.2374	0.0008	0.0216	No	0.9104	0.112	0.0133	0.825	No	No
104550_at	Cyp2s1	cytochrome P450, family 2, subfamily s, polypeptide 1	1.0587	0.3472	0.0084	0.3369	No	1.1103	0.323	0.126	0.104	No	No
104551_at	Cul7	cullin 7	1.1406	0.0596	0.1488	0.0502	No	1.089	0.0658	0.141	0.187	No	No
104554_at	Nr2f6	nuclear receptor subfamily 2, group F, member 6	1.0778	0.1227	0.0029	0.3796	No	1.0907	0.0207	0.0019	0.0277	No	No
104555_at	Myoz1	myozenin 1	0.8066	0.0079	0.8296	0.0112	Yes	0.8355	0.021	0.17	0.0417	No	Yes
104556_at	4933426K21 Rik	RIKEN cDNA 4933426K21 gene	1.1807	0.0238	0.0226	0.8414	No	1.0344	0.282	0.001	0.826	No	No
104557_at	Pitpnb	phosphatidylinositol transfer protein, beta	1.0485	0.2528	0.097	0.0778	No	1.0671	0.0776	0.11	0.051	No	No
104558_at	Rcor1	REST corepressor 1	0.9626	0.588	0.4474	0.8393	No	0.9825	0.845	0.221	0.56	No	No
104559_at	BC003940	cDNA sequence BC003940	1.0537	0.0837	0.9347	0.8741	No	1.0416	0.0483	0.0061	0.568	No	No
104560_at	Slc25a28	solute carrier family 25, member 28	1.044	0.4254	0.4403	0.7039	No	1.0092	0.858	0.609	0.97	No	No
104561_at	Gtf3c5	general transcription factor IIIC, polypeptide 5	0.9582	0.1274	0.4571	0.8466	No	0.993	0.872	0.295	0.73	No	No
104562_at	5730403M16 Rik	RIKEN cDNA 5730403M16 gene	1.0336	0.5227	0.9548	0.9779	No	1.0112	0.796	0.355	0.421	No	No
104564_at	Scg3	secretogranin III	0.9555	0.2913	0.1724	0.0906	No	0.9142	0.174	0.0535	0.107	No	No
104565_at	Ap4s1	adaptor-related protein complex AP-4, sigma 1	1.0948	0.1515	0.0023	0.3264	No	1.1088	0.094	0.0023	0.366	No	No
104566_at	Asl	argininosuccinate lyase	0.9469	0.1709	0.0865	0.0425	No	0.9934	0.824	0.0702	0.304	No	No
104567_at	Mrpl46	mitochondrial ribosomal protein L46	1.0104	0.7423	0.4384	0.3232	No	1.0161	0.583	0.0196	0.464	No	No
104568_at	Mll1	myeloid/lymphoid or mixed-lineage leukemia 1	1.0055	0.8253	0.0122	0.0133	No	1.0813	0.0283	0.0261	0.0052	No	No
104569_at	Mll1	myeloid/lymphoid or mixed-lineage leukemia 1	0.9924	0.9131	0.0042	0.1198	No	0.9144	0.271	0.0018	0.318	No	No
104571_at	Cxcl4	chemokine (C-X-C motif) ligand 4	1.0353	0.3826	0.0097	0.5076	No	1.1337	0.0244	0.0002	0.0353	No	No
104572_at	Zcchc6	zinc finger, CCHC domain containing 6	1.0152	0.3349	0.0001	0.5357	No	0.9956	0.854	0.0005	0.949	No	No
104573_at	Bola2	bolA-like 2 (E. coli)	0.9585	0.5286	0.0104	0.0346	No	0.967	0.859	0.0081	0.0373	No	No
104574_at	5730453I16 Rik	RIKEN cDNA 5730453I16 gene	0.9704	0.2246	0.0012	0.0022	No	0.9448	0.0398	0.0262	0.104	No	No
104576_at	Ski	Sloan-Kettering viral oncogene homolog	0.9691	0.4858	0.5675	0.9714	No	0.9081	0.0921	0.0529	0.241	No	No
104577_at	Mlh1	mutL homolog 1 (E. coli)	0.9554	0.48	0.2708	0.8764	No	1.0734	0.51	0.11	0.176	No	No
104578_f_at	Actn1	actinin, alpha 1	0.978	0.6563	0.002	0.9103	No	0.9317	0.111	0.0063	0.829	No	No
104579_r_at	Actn1	actinin, alpha 1	1.0509	0.2672	0.7246	0.0714	No	0.9342	0.384	0.251	0.145	No	No
104580_at	Plcd1	phospholipase C, delta 1	0.9626	0.3436	0.0042	0.0871	No	0.9495	0.159	0.0011	0.0178	No	No
104581_at	Zdhhc6 /// LOC669170	zinc finger, DHHC domain containing 6 /// similar to Probable palmitoyltransferase ZDHHC6 (Zinc finger DHHC domain-containing protein 6) (DHHC-6) (H4 homolog)	0.9983	0.9123	0.2111	0.0798	No	1.1422	0.181	0.484	0.0875	No	No
104582_g_at	Zdhhc6	zinc finger, DHHC domain containing 6	0.9993	0.9896	0.5012	0.9434	No	1.2897	0.0193	0.921	0.0144	No	No
104583_at	Zdhhc6	zinc finger, DHHC domain containing 6	1.0202	0.6741	0.0378	0.1218	No	1.045	0.269	0.01	0.327	No	No
104584_f_at	Crb3	crumbs homolog 3 (Drosophila)	1.0226	0.6876	0.7404	0.81	No	1.025	0.537	0.617	0.0518	No	No
104585_at	Crb3	crumbs homolog 3 (Drosophila)	1.0558	0.5351	0.1384	0.7953	No	1.0881	0.201	0.0032	0.847	No	No
104586_at	Sfrs1	splicing factor, arginine/serine-rich 1 (ASF/SF2)	1.0266	0.7395	0.0519	0.229	No	1.0046	0.607	0.0121	0.0059	No	No
104587_at	Lama4	laminin, alpha 4	0.9716	0.3517	0.0017	0.0636	No	0.9461	0.055	0.0034	0.784	No	No
104588_at	Hamp1	hepcidin antimicrobial peptide 1	0.954	0.5548	0.2969	0.3376	No	1.0252	0.715	0.0623	0.45	No	No
104589_at	C80913	expressed sequence C80913	1.0719	0.165	0.0001	0.112	No	1.021	0.726	0.0036	0.834	No	No
104590_at	Mef2c	myocyte enhancer factor 2C	1.0639	0.1339	0.0002	0.4307	No	0.9963	0.882	0.0013	0.782	No	No
104591_g_at	Mef2c	myocyte enhancer factor 2C	1.1607	0.086	0.0011	0.3164	No	1.0345	0.385	0.0004	0.683	No	No
104592_i_at	Mef2c	myocyte enhancer factor 2C	1.1411	0.0143	0.0001	0.2279	No	1.0891	0.0224	0	0.83	No	No
104593_at	Ccdc101	coiled-coil domain containing 101	0.946	0.2871	0.0477	0.7682	No	0.9554	0.479	0.0619	0.441	No	No
104594_at	S100a14	S100 calcium binding protein A14	0.9061	0.0535	0.5777	0.6228	No	1.0104	0.797	0.0025	0.996	No	No
104595_at	Stag2	stromal antigen 2	1.0309	0.7223	0.0157	0.8945	No	0.9582	0.62	0.0077	0.916	No	No
104597_at	Gbp2	guanylate nucleotide binding protein 2	0.991	0.7143	0.1806	0.0897	No	1.0503	0.156	0.0082	0.981	No	No
104598_at	Dusp1	dual specificity phosphatase 1	0.7776	0.0013	0.6554	0.1138	Yes	0.8154	0.0113	0.0204	0.0708	No	Yes
104600_at	Fip1l1	FIP1 like 1 (S. cerevisiae)	0.9446	0.2507	0.0433	0.1445	No	0.9074	0.108	0.168	0.966	No	No
104601_at	Thbd	thrombomodulin	1.0052	0.9656	0.1333	0.628	No	1.0323	0.758	0.009	0.662	No	No
104602_at	Waspip	Wiskott-Aldrich syndrome protein interacting protein	1.0131	0.7636	0.0013	0.1657	No	0.9297	0.337	0.073	0.191	No	No
104603_at	Gstt2	glutathione S-transferase, theta 2	1.1018	0.0191	0.0524	0.7008	No	1.0177	0.414	0.0537	0.0803	No	No
104604_at	Zfp96	zinc finger protein 96	0.9008	0.2121	0.7296	0.7591	No	0.9456	0.451	0.392	0.304	No	No
104605_at	Adipor2	adiponectin receptor 2	0.9806	0.6908	0.4986	0.5785	No	0.977	0.88	0.373	0.55	No	No
104606_at	Cd52	CD52 antigen	1.0794	0.0364	0.1004	0.0414	No	1.1059	0.0624	0.521	0.0211	No	No
104607_at	Rtn2	reticulon 2 (Z-band associated protein)	0.7826	0.0026	0.5284	0.002	No	0.7699	0.011	0.464	0.0055	No	No
104608_at	Tm2d1	TM2 domain containing 1	1.157	0.121	0.0008	0.4804	No	0.9853	0.992	0.0846	0.235	No	No
104609_at	Hnrpul2	heterogeneous nuclear ribonucleoprotein U-like 2	0.9352	0.3646	0.0282	0.8327	No	0.9631	0.353	0.0073	0.746	No	No
104610_at	Pprc1	peroxisome proliferative activated receptor, gamma, coactivator-related 1	0.9866	0.7401	0.0152	0.0956	No	1.0374	0.623	0.0189	0.0757	No	No
104611_at	Wdr26	WD repeat domain 26	0.9405	0.0862	0.0546	0.9822	No	0.9577	0.139	0.588	0.275	No	No
104612_g_at	Wdr26	WD repeat domain 26	0.9306	0.3252	0.0149	0.9223	No	0.9474	0.0996	0.0033	0.0663	No	No
104614_at	Gpc1	glypican 1	0.9641	0.4006	0.0102	0.965	No	0.9887	0.753	0.464	0.0758	No	No
104615_at	Galt	galactose-1-phosphate uridyl transferase	0.9438	0.4834	0.0511	0.8509	No	0.9433	0.309	0.0037	0.74	No	No
104616_g_at	Galt	galactose-1-phosphate uridyl transferase	1.0437	0.595	0.7642	0.6948	No	1.0482	0.499	0.932	0.208	No	No
104617_at	0610005C13 Rik	RIKEN cDNA 0610005C13 gene	0.9301	0.3885	0.6592	0.8768	No	0.9352	0.511	0.624	0.239	No	No
104618_at	Rbbp9	retinoblastoma binding protein 9	0.9353	0.263	0.7962	0.0961	No	0.9989	0.95	0.0192	0.705	No	No
104619_at	Sphk2	sphingosine kinase 2	0.9673	0.1869	0.0014	0.3461	No	1.0286	0.0777	0	0.922	No	No
104620_at	Tmem68	transmembrane protein 68	0.9357	0.2539	0.3101	0.2199	No	0.9671	0.439	0.0853	0.437	No	No
104621_at	38782	membrane-associated ring finger (C3HC4) 6	0.9357	0.346	0.0006	0.6804	No	0.994	0.773	0.0007	0.371	No	No
104622_at	Tcea2	transcription elongation factor A (SII), 2	0.9603	0.5206	0.1013	0.9073	No	1.0216	0.685	0.0563	0.8	No	No
104623_at	Tle3	transducin-like enhancer of split 3, homolog of Drosophila E(spl)	0.897	0.0372	0.1884	0.2464	No	0.8886	0.0164	0.0091	0.107	No	No
104624_at	AU024582	expressed sequence AU024582	1.0217	0.4656	0.0008	0.2757	No	0.9799	0.684	0.781	0.433	No	No
104625_at	Dnajb6	DnaJ (Hsp40) homolog, subfamily B, member 6	0.9603	0.1272	0.7991	0.0499	No	0.9663	0.463	0.3	0.599	No	No
104626_at	Cmtm8	CKLF-like MARVEL transmembrane domain containing 8	0.9549	0.5157	0.8738	0.651	No	0.9563	0.288	0.372	0.476	No	No
104627_at	Cds2	CDP-diacylglycerol synthase (phosphatidate cytidylyltransferase) 2	0.9498	0.2015	0.4304	0.1335	No	0.9611	0.252	0.201	0.0558	No	No
104628_at	Man2a1	mannosidase 2, alpha 1	0.8667	0.0093	0.0253	0.6709	Yes	0.8597	0.0074	0.964	0.251	Yes	Yes
104629_at	Asb3	ankyrin repeat and SOCS box-containing protein 3	0.9991	0.987	0.0274	0.1512	No	0.9774	0.554	0.0121	0.133	No	No
104630_at	BC021611	cDNA sequence BC021611	0.9836	0.5713	0.0005	0.9371	No	1.1932	0.04	0.0039	0.373	No	No
104631_g_at	BC021611	cDNA sequence BC021611	0.9726	0.4218	0.0431	0.1981	No	1.0143	0.657	0.223	0.046	No	No
104632_at	BC021611	cDNA sequence BC021611	0.9916	0.8412	0.0855	0.6296	No	0.9704	0.212	0.0017	0.707	No	No
104633_at	Dab2	disabled homolog 2 (Drosophila)	1.072	0.271	0.0021	0.2877	No	0.9662	0.521	0.0004	0.122	No	No
104634_at	Lims1	LIM and senescent cell antigen-like domains 1	1.0524	0.5239	0.0021	0.6551	No	1.1459	0.165	0.001	0.105	No	No
104635_r_at	Gja7	gap junction membrane channel protein alpha 7	1.0346	0.6413	0.0184	0.8106	No	1.0643	0.149	0.0004	0.626	No	No
104636_at	Gstm6	glutathione S-transferase, mu 6	0.9722	0.6753	0.1401	0.9208	No	1.1124	0.12	0.0003	0.595	No	No
104637_at	Gstm6	glutathione S-transferase, mu 6	0.9897	0.8857	0.0023	0.1767	No	0.9909	0.8	0.0918	0.967	No	No
104638_at	Art1	ADP-ribosyltransferase 1	0.7682	0.0012	0.0604	0.0013	No	0.8232	0.0155	0.0897	0.0132	No	No
104639_i_at	Josd3	Josephin domain containing 3	1.0603	0.2438	0.0021	0.1092	No	1.0661	0.111	0.0007	0.201	No	No
104640_f_at	Josd3	Josephin domain containing 3	1.0015	0.984	0.0059	0.4209	No	1.0377	0.308	0.0004	0.554	No	No

104641_f_at	LOC668645	similar to gonadotropin inducible ovarian transcription factor 1	1.1034	0.012	0.4162	0.0309	No	1.135	0.0399	0.243	0.0189	No	No
104642_at	Clec4f	C-type lectin domain family 4, member f	0.9499	0.3546	0.3175	0.54	No	0.9734	0.827	0.257	0.571	No	No
104643_at	Wwc1	WW, C2 and coiled-coil domain containing 1	0.9574	0.1868	0.0006	0.2474	No	0.9362	0.0822	0.0063	0.29	No	No
104644_at	Kif4	kinesin family member 4	1.0241	0.6485	0.024	0.7013	No	1.0163	0.932	0.003	0.837	No	No
104645_at	Klf7	Kruppel-like factor 7 (ubiquitous)	0.9526	0.2665	0.0001	0.0719	No	0.861	0.0546	0.0074	0.253	No	No
104646_at	Gdf15	growth differentiation factor 15	0.973	0.3151	0.0726	0.2408	No	0.9643	0.244	0.978	0.478	No	No
104647_at	Ptgs2	prostaglandin-endoperoxide synthase 2	0.9068	0.2069	0.3531	0.246	No	1.0398	0.543	0.458	0.297	No	No
104648_at	Pacs1	phosphofurin acidic cluster sorting protein 1	1.0223	0.5993	0.8572	0.8566	No	1.086	0.0953	0.0143	0.0098	No	No
104650_at	Ache	acetylcholinesterase	0.9138	0.0397	0.0219	0.0362	No	1.0043	0.943	0.208	0.601	No	No
104651_at	Snx14	sorting nexin 14	0.9497	0.5121	0.1108	0.2975	No	1.0671	0.343	0.0376	0.0692	No	No
104652_at	Kcnk2	potassium channel, subfamily K, member 2	0.9892	0.8006	0.0061	0.4506	No	1.0561	0.47	0.305	0.709	No	No
104653_at	Rab8b /// LOC670268	RAB8B, member RAS oncogene family /// similar to RAB8B, member RAS oncogene family	1.0534	0.0961	0.3004	0.5756	No	0.9871	0.653	0.0023	0.481	No	No
104654_at	Actl6b	actin-like 6B	1.0209	0.608	0.0972	0.5714	No	1.0796	0.262	0.0321	0.101	No	No
104655_at	Tbx2	T-box 2	1.0129	0.7419	0.8664	0.694	No	1.0574	0.345	0.0214	0.151	No	No
104657_at	Lifr	leukemia inhibitory factor receptor	0.9941	0.883	0.9217	0.2558	No	1.0722	0.0539	0.733	0.0043	No	No
104658_at	Lifr	leukemia inhibitory factor receptor	0.9208	0.004	0.0025	0.1092	Yes	0.9763	0.16	0.0852	0.149	No	No
104659_g_at	Lifr	leukemia inhibitory factor receptor	0.8877	0.0401	0.1419	0.7291	No	0.8936	0.198	0.228	0.476	No	No
104660_at	Sit1	suppression inducing transmembrane adaptor 1	0.955	0.3868	0.0384	0.6104	No	0.9235	0.153	0.0209	0.22	No	No
104661_at	A630051L19 Rik	RIKEN cDNA A630051L19 gene	1.0452	0.0722	0	0.0068	No	1.0031	0.775	0.0138	0.0514	No	No
104662_at	Tnfrsf18	tumor necrosis factor receptor superfamily, member 18	1.0733	0.0084	0.0004	0.0754	Yes	0.9885	0.376	0.0021	0.0098	No	No
104663_at	Pip5k1b	phosphatidylinositol-4-phosphate 5-kinase, type 1 beta	0.9771	0.614	0.0129	0.7556	No	1.0447	0.155	0.0114	0.291	No	No
104664_at	Ches1	checkpoint suppressor 1	0.9411	0.0926	0.0013	0.4766	No	0.9885	0.77	0.0444	0.534	No	No
104665_g_at	Ches1	checkpoint suppressor 1	1.015	0.6574	0.0211	0.2014	No	1.2282	0.0159	0.249	0.0091	No	No
104666_at	Zfp653	zinc finger protein 653	1.0276	0.6893	0.0696	0.9601	No	1.0238	0.696	0.651	0.147	No	No
104667_at	Lrrc23	leucine rich repeat containing 23	0.8984	0.1398	0.0011	0.5459	No	0.9661	0.475	0.0009	0.85	No	No
104669_at	Irf7	interferon regulatory factor 7	0.9435	0.2306	0.0435	0.742	No	0.9159	0.155	0.919	0.234	No	No
104670_at	1700065A05 Rik	RIKEN cDNA 1700065A05 gene	1.0203	0.7654	0.015	0.349	No	1.0376	0.581	0.0055	0.702	No	No
104671_at	Ampd3	AMP deaminase 3	0.8597	0.008	0.0458	0.016	Yes	0.8436	0.0009	0.0035	0.376	Yes	Yes
104672_at	Frzb	frizzled-related protein	1.011	0.7736	0.0027	0.0288	No	0.9752	0.501	0.0003	0.705	No	No
104673_at	Epha4	Eph receptor A4	0.9949	0.9016	0.0077	0.8262	No	0.9865	0.774	0.0214	0.308	No	No
104674_s_at	Pcsk4	proprotein convertase subtilisin/kexin type 4	0.96	0.2056	0.026	0.2181	No	0.9519	0.37	0.0143	0.242	No	No
104675_at	Pcsk4	proprotein convertase subtilisin/kexin type 4	1.0621	0.3812	0.743	0.8339	No	1.1066	0.0382	0.0724	0.0811	No	No
104677_at	Man1b1	mannosidase, alpha, class 1B, member 1	0.943	0.4127	0.0851	0.0817	No	1.0082	0.71	0.0123	0.153	No	No
104678_at	Gas7	growth arrest specific 7	0.9914	0.8258	0.4741	0.5457	No	0.8905	0.086	0.816	0.185	No	No
104679_at	Txk	TXK tyrosine kinase	1.0864	0.2667	0.467	0.135	No	1.0597	0.503	0.252	0.412	No	No
104680_at	Ramp1	receptor (calcitonin) activity modifying protein 1	0.9579	0.2278	0.0004	0.3405	No	0.9527	0.0952	0.0005	0.191	No	No
104681_at	Diap1	diaphanous homolog 1 (Drosophila)	1.054	0.4062	0.1751	0.4081	No	1.0021	0.996	0.39	0.0826	No	No
104682_at	Tuba8	tubulin, alpha 8	1.0199	0.5384	0.8354	0.4996	No	1.0085	0.82	0.0732	0.276	No	No
104683_at	Ankrd17	ankyrin repeat domain 17	0.9074	0.2818	0.0078	0.6298	No	0.8929	0.175	0.365	0.438	No	No
104684_at	Grin1	glutamate receptor, ionotropic, NMDA1 (zeta 1)	0.9757	0.621	0.2003	0.2996	No	0.9244	0.0847	0.403	0.874	No	No
104685_g_at	Grin1	glutamate receptor, ionotropic, NMDA1 (zeta 1)	1.0766	0.3608	0.0095	0.4715	No	1.0677	0.488	0.0095	0.442	No	No
104686_at	Grin1	glutamate receptor, ionotropic, NMDA1 (zeta 1)	0.9724	0.6076	0.169	0.8306	No	1.0427	0.507	0.0316	0.317	No	No
104687_at	Edg6	endothelial differentiation, G-protein-coupled receptor 6	1.0405	0.3529	0.2269	0.117	No	0.9755	0.645	0.0288	0.577	No	No
104688_at	Map4k1 /// LOC672367	mitogen activated protein kinase kinase kinase kinase 1 /// similar to Mitogen-activated protein kinase kinase kinase kinase 1 (MAPK/ERK kinase kinase kinase 1) (MEK kinase kinase 1) (MEKKK 1) (Hematopoietic progenitor kinase) (HPK)	0.9518	0.1493	0.3602	0.3328	No	1.1261	0.109	0.602	0.325	No	No
104689_at	E4f1	E4F transcription factor 1	0.9966	0.8303	0.2186	0.0192	No	1.057	0.164	0.0077	0.326	No	No
104690_at	Polm	polymerase (DNA directed), mu	1.0529	0.3368	0.0241	0.0424	No	1.0329	0.389	0.805	0.0658	No	No
104692_at	Selp	selectin, platelet	1.0136	0.8204	0.5853	0.1943	No	0.9704	0.625	0.34	0.225	No	No
104693_at	2600001M11 Rik	RIKEN cDNA 2600001M11 gene	0.9436	0.1269	0.8948	0.4339	No	0.8936	0.0423	0.261	0.563	No	No
104694_at	BC021442	cDNA sequence BC021442	1.1638	0.0688	0.0131	0.2364	No	1.0197	0.124	0.0004	0.0007	No	No
104695_at	2310040C09 Rik	RIKEN cDNA 2310040C09 gene	0.9654	0.4769	0.5701	0.6883	No	1.0091	0.862	0.57	0.882	No	No
104696_at	Ctse	cathepsin E	0.9294	0.0747	0.0177	0.1243	No	1.0794	0.0714	0.0006	0.0515	No	No
104697_at	Rhoj	ras homolog gene family, member J	0.9576	0.2721	0.3695	0.0254	No	0.9884	0.777	0.133	0.554	No	No
104698_at	Gata6	GATA binding protein 6	1.1148	0.1293	0.6563	0.1334	No	1.0419	0.551	0.585	0.347	No	No
104699_at	Pik3c3	phosphoinositide-3-kinase, class 3	0.878	0.0545	0.0073	0.0279	No	0.9336	0.0161	0.0206	0.0117	No	No
104700_at	Shmt1	serine hydroxymethyl transferase 1 (soluble)	0.9899	0.8587	0.1032	0.9063	No	1.037	0.528	0.874	0.243	No	No
104701_at	Bhlhb2	basic helix-loop-helix domain containing, class B2	1.0399	0.477	0.1209	0.5606	No	1.0745	0.0653	0.903	0.258	No	No
104702_at	Wdr46	WD repeat domain 46	0.9585	0.2697	0.0596	0.9077	No	1.0584	0.315	0.0693	0.232	No	No
104704_at	Clcn4-2	Chloride channel 4-2	0.9562	0.4422	0.0573	0.0214	No	0.9881	0.962	0.172	0.394	No	No
104706_at	Pex7	peroxisome biogenesis factor 7	1.0488	0.5248	0.0195	0.1111	No	1.128	0.227	0.0018	0.0632	No	No
104707_at	Tm4sf5	transmembrane 4 superfamily member 5	1.0677	0.2365	0.1214	0.5003	No	0.9384	0.144	0.0105	0.882	No	No
104708_at	Tbl1x	Transducin (beta)-like 1 X-linked	1.0088	0.8382	0.5139	0.1585	No	1.1176	0.0438	0.0202	0.36	No	No
104709_at	Sec23a	SEC23A (S. cerevisiae)	0.9953	0.8633	0.945	0.0205	No	0.9758	0.691	0.0084	0.53	No	No
104710_at	Bak1	BCL2-antagonist/killer 1	0.985	0.7922	0.0505	0.1923	No	1.0898	0.0197	0.688	0.0341	No	No
104711_at	Vps4a	vacuolar protein sorting 4a (yeast)	0.9407	0.1393	0.0184	0.8702	No	0.9892	0.819	0.943	0.919	No	No
104712_at	Myc	myelocytomatosis oncogene	0.8268	0.095	0.3136	0.9733	No	0.8606	0.18	0.218	0.514	No	No
104713_at	Prpf38a	PRP38 pre-mRNA processing factor 38 (yeast) domain containing A	0.9761	0.598	0.03	0.3778	No	0.9711	0.559	0.0295	0.186	No	No
104714_at	Dock9 /// LOC670309	dedicator of cytokinesis 9 /// similar to Dedicator of cytokinesis protein 9 (Cdc42 guanine nucleotide exchange factor zizimin 1)	0.9525	0.1605	0.0007	0.7099	No	0.986	0.905	0.0003	0.0233	No	No
104715_at	Ubap2	ubiquitin-associated protein 2	0.948	0.088	0.0019	0.9874	No	0.9689	0.453	0.0088	0.877	No	No
104716_at	Rbp1	retinol binding protein 1, cellular	1.1653	0.0144	0.5288	0.3284	No	1.1738	0.0026	0.0134	0.285	Yes	Yes
104717_at	Zfp644	zinc finger protein 644	1.0091	0.8264	0.0093	0.268	No	1.065	0.377	0.112	0.851	No	No
104719_at	Slc12a7	solute carrier family 12, member 7	0.999	0.9309	0	0.465	No	1.0903	0.0165	0.0024	0.188	No	No
104720_at	Ubn1	ubinuclein 1	0.9129	0.0177	0.0014	0.018	No	0.9654	0.476	0.0161	0.167	No	No
104722_at	Lmbr1l	limb region 1 like	0.9536	0.2494	0.2321	0.8344	No	1.0692	0.0263	0.0498	0.0287	No	No
104723_at	Aspscr1	Alveolar soft part sarcoma chromosome region, candidate 1 (human)	0.9829	0.6655	0.2622	0.1008	No	1.029	0.781	0.312	0.209	No	No
104725_at	Rhoq	ras homolog gene family, member Q	1.0111	0.8175	0.0002	0.3792	No	0.9954	0.53	0	0.186	No	No
104726_at	1700013L23 Rik	RIKEN cDNA 1700013L23 gene	0.9026	0.0016	0.0001	0.0887	Yes	0.9437	0.631	0.613	0.807	No	No
104727_at	AW495713	expressed sequence AW495713	0.97	0.4025	0.0111	0.7573	No	0.9927	0.793	0.0066	0.0929	No	No
104728_at	Pros1	protein S (alpha)	0.8757	0.0177	0.0621	0.6298	No	0.8713	0.0066	0.0205	0.413	Yes	Yes
104729_at	Gja4	gap junction membrane channel protein alpha 4	1.0187	0.6309	0.0096	0.363	No	1.0232	0.119	0.0004	0.632	No	No
104730_at	Mdn1	midasin homolog (yeast)	1.0179	0.623	0.8548	0.448	No	1.1041	0.187	0.535	0.835	No	No
104731_at	Zfp467	zinc finger protein 467	0.9985	0.9627	0.1405	0.0953	No	0.9979	0.992	0.0056	0.371	No	No
104733_at	Cetn2	centrin 2	1.1373	0.2028	0.1801	0.7236	No	1.0831	0.422	0.144	0.273	No	No
104735_at	Kctd12	potassium channel tetramerisation domain containing 12	1.1824	0.0687	0.0013	0.2178	No	1.087	0.119	0.0009	0.262	No	No
104737_at	2610528A11 Rik	RIKEN cDNA 2610528A11 gene	0.9811	0.7241	0.0078	0.3264	No	0.932	0.331	0.108	0.889	No	No
104738_at	Dnajc2	DnaJ (Hsp40) homolog, subfamily C, member 2	0.919	0.0353	0.5598	0.0227	No	0.9668	0.157	0.0103	0.0883	No	No
104739_at	Tcta	T-cell leukemia translocation altered gene	0.95	0.0834	0.0021	0.104	No	0.9858	0.374	0.0106	0.233	No	No
104740_at	Tada1l	transcriptional adaptor 1 (HFI1 homolog, yeast) like	0.9575	0.3816	0.0113	0.9621	No	0.9403	0.209	0.795	0.517	No	No
104741_at	Zdhhc9	zinc finger, DHHC domain containing 9	1.0253	0.4811	0.0035	0.8831	No	1.0412	0.328	0.0818	0.783	No	No
104742_at	Mgst2	microsomal glutathione S-transferase 2	1.0199	0.6022	0.9415	0.3683	No	0.9409	0.175	0.0459	0.0725	No	No
104743_at	Cdh13	cadherin 13	0.9217	0.1417	0.2685	0.1503	No	0.942	0.312	0.182	0.224	No	No
104744_at	Tmem55a	transmembrane protein 55A	0.9827	0.7956	0.3894	0.4673	No	0.9745	0.513	0.0129	0.141	No	No
104745_at	Arl6ip2	ADP-ribosylation factor-like 6 interacting protein 2	1.0924	0.1455	0.0044	0.0313	No	0.9771	0.583	0.936	0.72	No	No
104746_at	Fkbp7	FK506 binding protein 7	1.013	0.7697	0.0922	0.8672	No	1.0738	0.097	0.0749	0.911	No	No
104747_at	Slc1a1	solute carrier family 1 (neuronal/epithelial high affinity glutamate transporter, system Xag), member 1	0.9637	0.5108	0.3371	0.8143	No	1.0747	0.412	0.483	0.177	No	No

104748_s_at	Slc1a1	solute carrier family 1 (neuronal/epithelial high affinity glutamate transporter, system Xag), member 1	0.863	0.1233	0.0053	0.7407	No	1.0613	0.886	0.0002	0.229	No	No
104749_at	Rnf20	ring finger protein 20	0.9604	0.523	0.0207	0.2385	No	0.9547	0.657	0.0154	0.372	No	No
104750_at	Ifi47	interferon gamma inducible protein 47	1.0663	0.3909	0.479	0.5944	No	1.1385	0.186	0.157	0.27	No	No
104751_at	Prph1 /// LOC673890	peripherin 1 /// similar to peripherin 1	0.8694	0.1456	0.0011	0.5711	No	0.9389	0.028	0	0.033	No	No
104752_at	Apip	APAF1 interacting protein	0.9944	0.7586	0.004	0.577	No	0.9643	0.0974	0.0106	0.114	No	No
104754_at	Sfrs4	splicing factor, arginine/serine-rich 4 (SRp75)	1.0181	0.0862	0.0001	0.0065	No	1.0305	0.609	0.0217	0.863	No	No
104755_at	Tnip1	TNFAIP3 interacting protein 1	0.95	0.1614	0.0222	0.6573	No	1.0323	0.389	0.0022	0.579	No	No
104756_at	2310047M15 Rik	RIKEN cDNA 2310047M15 gene	0.9896	0.8283	0.0279	0.7028	No	1.0035	0.944	0.0098	0.968	No	No
104757_at	Zw10	ZW10 homolog (Drosophila), centromere/kinetochore protein	0.9943	0.9017	0.032	0.4804	No	1.0064	0.805	0.007	0.381	No	No
104758_at	Rpusd4	RNA pseudouridylate synthase domain containing 4	0.9318	0.0962	0.8694	0.1404	No	0.9526	0.131	0.816	0.852	No	No
104759_at	1200011I18 Rik	RIKEN cDNA 1200011I18 gene	1.0177	0.5202	0.0028	0.2104	No	1.0145	0.403	0.0063	0.52	No	No
104760_at	Ifrd2	interferon-related developmental regulator 2	1.0441	0.304	0.0144	0.1109	No	0.9279	0.0258	0.002	0.288	No	No
104761_at	Antxr2	anthrax toxin receptor 2	0.9414	0.4027	0.0248	0.7845	No	0.9338	0.292	0.073	0.356	No	No
104762_r_at	Crsp8	cofactor required for Sp1 transcriptional activation, subunit 8	0.9571	0.3637	0.0318	0.8155	No	0.9834	0.501	0.0058	0.442	No	No
104763_at	Agrn	agrin	1.016	0.7728	0.791	0.2057	No	1.0306	0.693	0.637	0.76	No	No
104766_at	Nola1	nucleolar protein family A, member 1 (H/ACA small nucleolar RNPs)	1.0811	0.101	0.0408	0.676	No	0.9872	0.819	0.0937	0.556	No	No
104767_f_at	Mrps18a	mitochondrial ribosomal protein S18A	0.9724	0.5589	0.1211	0.0787	No	1.0034	0.802	0.0078	0.294	No	No
160060_at	Znhit3	zinc finger, HIT type 3	0.9493	0.4324	0.0258	0.5411	No	0.9974	0.986	0.21	0.3	No	No
160061_at	Tmed1	transmembrane emp24 domain containing 1	0.9377	0.1642	0.0767	0.1113	No	0.9535	0.333	0.0841	0.0194	No	No
160062_i_at	Fdx1	Ferredoxin 1	0.9902	0.83	0.0042	0.5181	No	1.0768	0.1	0.0295	0.0914	No	No
160063_i_at	Gsta1	Glutathione S-transferase, alpha 1 (Ya)	1.0348	0.6164	0.6061	0.1409	No	0.9747	0.872	0.33	0.204	No	No
160064_at	Stx7	syntaxin 7	1.0203	0.6116	0.6586	0.4094	No	0.9772	0.641	0.577	0.195	No	No
160065_s_at	Csrp1	cysteine and glycine-rich protein 1	0.9193	0.3667	0.4278	0.4394	No	0.9026	0.298	0.0852	0.319	No	No
160066_at	Limd1	LIM domains containing 1	0.8887	0.0607	0.005	0.6638	No	0.986	0.711	0.0017	0.518	No	No
160067_at	2310057D15 Rik	RIKEN cDNA 2310057D15 gene	1.1277	0.0382	0.008	0.3814	No	0.9501	0.0857	0.0004	0.0716	No	No
160068_at	Sap30	sin3 associated polypeptide	0.9689	0.647	0.3169	0.6806	No	0.9254	0.0992	0.47	0.361	No	No
160069_at	Gmnn	geminin	1.112	0.0214	0.6845	0.0957	No	1.1435	0.011	0.0598	0.232	No	No
160070_at	Pnliprp2	pancreatic lipase-related protein 2	0.9438	0.1411	0.001	0.4505	No	1.0476	0.347	0.12	0.692	No	No
160071_at	Rpp30	ribonuclease P/MRP 30 subunit (human)	1.0009	0.9835	0.8596	0.1501	No	0.9802	0.614	0.897	0.302	No	No
160072_at	Rfc3	replication factor C (activator 1) 3	1.012	0.8869	0.1123	0.6193	No	0.9892	0.849	0.937	0.352	No	No
160073_i_at	Srp54	signal recognition particle 54	1.087	0.1287	0.0044	0.8932	No	1.0255	0.54	0.0443	0.483	No	No
160074_at	Ddc	dopa decarboxylase	0.8879	0.0097	0.0044	0.2692	Yes	0.9186	0.001	0.0001	0.789	Yes	Yes
160075_at	Nit1	nitrilase 1	1.0223	0.7645	0.3563	0.5188	No	0.9883	0.837	0.168	0.873	No	No
160076_at	Mtx2	metaxin 2	1.0121	0.8495	0.6234	0.5293	No	1.0172	0.787	0.702	0.556	No	No
160077_at	Rai12	retinoic acid induced 12	1.0307	0.1698	0.0005	0.3318	No	0.9835	0.583	0.0237	0.183	No	No
160078_at	Ppp1r14b	protein phosphatase 1, regulatory (inhibitor) subunit 14B	1.0291	0.6455	0.173	0.1166	No	1.006	0.605	0.0062	0.0146	No	No
160079_i_at	Wac	WW domain containing adaptor with coiled-coil	0.9342	0.1657	0.0001	0.1398	No	0.8956	0.103	0.0001	0.0608	No	No
160080_r_at	Ddah1	Dimethylarginine dimethylaminohydrolase 1	0.9836	0.8121	0.0069	0.0954	No	0.9674	0.42	0.003	0.162	No	No
160081_at	Rpl36a	ribosomal protein L36a	1.0311	0.6836	0.1341	0.7555	No	0.9361	0.593	0.259	0.379	No	No
160082_s_at	Arf4	ADP-ribosylation factor 4	1.076	0.2543	0.8982	0.6346	No	1.0568	0.513	0.222	0.92	No	No
160083_at	LOC669888	similar to Lipoprotein lipase precursor (LPL)	0.8213	0.0463	0.0197	0.9621	No	0.9121	0.0197	0.0023	0.14	No	No
160084_at	LOC668343 /// LOC673302	similar to Ornithine decarboxylase (ODC) /// similar to Ornithine decarboxylase (ODC)	1.1017	0.0224	0.04	0.1871	No	1.1626	0.0441	0.275	0.3	No	No
160085_at	Tst	thiosulfate sulfurtransferase, mitochondrial	0.982	0.6484	0.0006	0.1267	No	0.954	0.418	0.0017	0.417	No	No
160086_at	Itga7	integrin alpha 7	0.9777	0.5882	0.6454	0.8976	No	1.0642	0.0363	0.131	0.0142	No	No
160087_at	Preb	prolactin regulatory element binding	0.969	0.62	0.15	0.7303	No	1.0425	0.438	0.307	0.943	No	No
160088_at	Fmo5	flavin containing monooxygenase 5	0.9455	0.307	0.0193	0.3336	No	0.9293	0.0368	0.0067	0.59	No	No
160089_at	Lamp1	lysosomal membrane glycoprotein 1	0.9594	0.0603	0.0059	0.2552	No	0.9899	0.719	0.922	0.295	No	No
160090_f_at	Aldoa	aldolase 1, A isoform	0.9247	0.0831	0.1427	0.0078	No	0.8794	0.0042	0.0001	0.0017	No	No
160091_at	Pgam1 /// LOC667978 /// LOC668045 /// LOC669836 /// LOC672147 /// LOC675179 /// LOC677000	phosphoglycerate mutase 1 /// similar to Phosphoglycerate mutase 1 (Phosphoglycerate mutase isozyme B) (PGAM-B) (BPG-dependent PGAM 1) /// similar to Phosphoglycerate mutase 1 (Phosphoglycerate mutase isozyme B) (PGAM-B) (BPG-dependent PGAM 1) /// similar to Phosphoglycerate mutase 1 (Phosphoglycerate mutase isozyme B) (PGAM-B) (BPG-dependent PGAM 1) /// similar to Phosphoglycerate mutase 1 (Phosphoglycerate mutase isozyme B) (PGAM-B) (BPG-dependent PGAM 1) /// similar to Phosphoglycerate mutase 1 (Phosphoglycerate mutase isozyme B) (PGAM-B) (BPG-dependent PGAM 1) /// similar to Phosphoglycerate mutase 1 (Phosphoglycerate mutase isozyme B) (PGAM-B) (BPG-dependent PGAM 1)	1.0177	0.668	0.3974	0.3466	No	1.0581	0.104	0.252	0.862	No	No
160092_at	Ifrd1	interferon-related developmental regulator 1	1.1489	0.11	0.0249	0.3491	No	1.0707	0.38	0.0466	0.307	No	No
160093_r_at	Thyn1	thymocyte nuclear protein 1	1.0197	0.7157	0.1508	0.4133	No	1.0265	0.76	0.138	0.582	No	No
160094_at	Arpc4	actin related protein 2/3 complex, subunit 4	0.9673	0.7342	0.0228	0.8292	No	0.9415	0.528	0.0053	0.331	No	No
160095_at	Lox	lysyl oxidase	0.9205	0.1241	0.9746	0.0868	No	0.8492	0.0214	0.0087	0.986	No	No
160096_at	Spop	speckle-type POZ protein	0.9191	0.163	0.0147	0.5798	No	0.9229	0.194	0.59	0.242	No	No
160097_at	Nespas	neuroendocrine secretory protein antisense	0.9932	0.8831	0.0333	0.8642	No	1.02	0.508	0.0588	0.0747	No	No
160098_s_at	Faf1	Fas-associated factor 1	1.0576	0.3078	0.8866	0.1967	No	1.0415	0.267	0.57	0.0171	No	No
160099_at	Lgals4	lectin, galactose binding, soluble 4	0.8875	0.0057	0.0005	0.0399	Yes	1.0054	0.793	0.0009	0.51	No	No
160100_at	Efhd2	EF hand domain containing 2	1.0224	0.5216	0.4773	0.6522	No	0.9678	0.459	0.29	0.205	No	No
160101_at	Hmox1	heme oxygenase (decycling) 1	0.9692	0.094	0.0007	0.014	No	1.0006	0.619	0.0004	0.0112	No	No
160102_at	Cct8	chaperonin subunit 8 (theta)	1.1859	0.0912	0.0756	0.1551	No	1.0017	0.756	0.0095	0.074	No	No
160103_at	38783	membrane-associated ring finger (C3HC4) 7	0.9532	0.5384	0.0068	0.9735	No	0.9236	0.368	0.0341	0.943	No	No
160104_at	Hsd3b7	hydroxy-delta-5-steroid dehydrogenase, 3 beta- and steroid delta-isomerase 7	0.9922	0.6016	0.0029	0.0053	No	0.9641	0.531	0.0127	0.444	No	No
160105_r_at	Pigk	phosphatidylinositol glycan, class K	1.0152	0.6021	0	0.0106	No	1.0181	0.721	0.0002	0.0214	No	No
160106_at	Capg	capping protein (actin filament), gelsolin-like	0.9387	0.4163	0.041	0.1833	No	0.8955	0.0096	0.202	0.0052	No	No
160107_at	Hprt1	hypoxanthine guanine phosphoribosyl transferase 1	1.1463	0.0004	0.0037	0.3139	Yes	1.0602	0.184	0.837	0.794	No	No
160108_at	Nupr1	nuclear protein 1	0.9012	0.1984	0.4074	0.1807	No	0.9063	0.0585	0.722	0.19	No	No
160109_at	Sox4 /// LOC672274	SRY-box containing gene 4 /// similar to Transcription factor SOX-4	1.2376	0.0004	0	0.0004	No	1.2419	0.001	0.0001	0.0027	No	No
160110_at	Wwp2	WW domain containing E3 ubiquitin protein ligase 2	0.9797	0.4693	0	0.0326	No	0.9746	0.94	0	0.0362	No	No
160111_at	Eif1ay	eukaryotic translation initiation factor 1A, Y-linked	0.968	0.7432	0.0535	0.5275	No	0.9683	0.687	0.0093	0.989	No	No
160112_at	Chmp2b	chromatin modifying protein 2B	0.9929	0.9602	0.4299	0.4292	No	0.9427	0.403	0.0147	0.999	No	No
160113_at	Brap	BRCA1 associated protein	0.9442	0.0716	0.0713	0.6748	No	0.9365	0.176	0.25	0.236	No	No
160114_at	Gdi2	guanosine diphosphate (GDP) dissociation inhibitor 2	0.9724	0.6204	0.0337	0.5341	No	0.9649	0.519	0.924	0.712	No	No
160115_at	Txnl1	thioredoxin-like 1	1.0904	0.3336	0.0294	0.6387	No	1.17	0.184	0.0551	0.961	No	No
160116_at	Timp2	tissue inhibitor of metalloproteinase 2	0.9675	0.674	0.6988	0.8809	No	1.1201	0.0068	0.0055	0.0143	Yes	No
160117_at	Tef	thyrotroph embryonic factor	0.9649	0.1448	0.0037	0.4418	No	1.0086	0.658	0.662	0.0486	No	No
160118_at	Mmp14	matrix metallopeptidase 14 (membrane-inserted)	1.1047	0.0295	0.007	0.2445	No	1.1122	0.0029	0.0026	0.154	Yes	Yes
160119_at	Creld2	cysteine-rich with EGF-like domains 2	1.0598	0.2527	0.0412	0.537	No	0.9872	0.607	0.0053	0.241	No	No
160120_i_at	Pla2g1b	phospholipase A2, group IB, pancreas	1.0941	0.0334	0.0024	0.0103	No	1.1289	0.0282	0.0085	0.0185	No	No

160121_at	Galk2	galactokinase 2	0.9586	0.0308	0.0002	0.017	No	0.9528	0.15	0.0005	0.004	No	No
160122_at	Asrgl1	asparaginase like 1	1.0024	0.9318	0.5444	0.3318	No	1.0455	0.562	0.14	0.411	No	No
160123_at	Phpt1	phosphohistidine phosphatase 1	1.0218	0.6241	0.0252	0.1516	No	0.9892	0.76	0.0547	0.276	No	No
160124_r_at	Atp6v1c1	ATPase, H+ transporting, lysosomal V1 subunit C1	1.0704	0.3026	0.0003	0.8902	No	1.0944	0.0979	0.0001	0.887	No	No
160125_at	D11Ertd497e	DNA segment, Chr 11, ERATO Doi 497, expressed	0.8921	0.0027	0.0001	0.0375	Yes	0.9378	0.0776	0.0008	0.0829	No	No
160126_at	Map2k1ip1	mitogen-activated protein kinase kinase 1 interacting protein 1	0.9592	0.3578	0.3029	0.1551	No	0.9348	0.0822	0.0846	0.0278	No	No
160127_at	Ccng1	cyclin G1	0.9254	0.4288	0.0228	0.7066	No	0.9746	0.549	0.0612	0.396	No	No
160128_at	Pccb	propionyl Coenzyme A carboxylase, beta polypeptide	1.045	0.4116	0.0251	0.471	No	0.9963	0.529	0.0003	0.0214	No	No
160129_at	Eef1d	eukaryotic translation elongation factor 1 delta (guanine nucleotide exchange protein)	0.9458	0.2029	0.0015	0.0429	No	0.9542	0.757	0.0003	0.101	No	No
160130_at	Wdr26	WD repeat domain 26	1.1355	0.2505	0.0017	0.1195	No	1.1146	0.0574	0.0016	0.021	No	No
160131_at	Amotl2	angiomotin like 2	0.9156	0.2062	0.0228	0.4283	No	0.8639	0.0658	0.0006	0.441	No	No
160132_at	Clps	colipase, pancreatic	0.9145	0.0235	0.0168	0.1504	No	0.8871	0.0336	0.0075	0.0169	No	No
160133_at	Ltbp3	latent transforming growth factor beta binding protein 3	1.0361	0.0138	0.0002	0.0039	No	1.0521	0.156	0.0042	0.147	No	No
160134_at	Adipor1	adiponectin receptor 1	0.9855	0.6113	0.6394	0.1088	No	1.0179	0.62	0.296	0.279	No	No
160135_at	Nit2	nitrilase family, member 2	0.9685	0.3864	0.5506	0.0956	No	1.0177	0.44	0.303	0.0099	No	No
160136_r_at	Tloc1	translocation protein 1	0.9658	0.4725	0.1753	0.8533	No	1.0653	0.248	0.0183	0.549	No	No
160137_at	B3gnt2	UDP-GlcNAc:betaGal beta-1,3-N-acetylglucosaminyltransferase 2	0.8882	0.011	0.0144	0.0768	No	0.9191	0.211	0.128	0.41	No	No
160138_at	Mxi1	Max interacting protein 1	0.9412	0.3038	0.0025	0.9749	No	0.8926	0.17	0.0431	0.666	No	No
160139_at	Hspb8	heat shock protein 8	0.8032	0.0029	0.2195	0.1438	Yes	0.7901	0.0104	0.47	0.0787	No	Yes
160140_at	Tbce	tubulin-specific chaperone e	1.1287	0.0049	0.0013	0.7729	Yes	1.0901	0.179	0.034	0.952	No	No
160141_r_at	Sfrs1	splicing factor, arginine/serine-rich 1 (ASF/SF2)	1.2025	0.2713	0.0086	0.455	No	1.0647	0.813	0.0046	0.72	No	No
160142_at	Tusc2	tumor suppressor candidate 2	1.0381	0.6392	0.7536	0.8049	No	1.1163	0.064	0.45	0.497	No	No
160143_r_at	Sycp3	synaptonemal complex protein 3	1.0159	0.4101	0.037	0.0073	No	1.1115	0.383	0.42	0.155	No	No
160144_at	Dnajc10	DnaJ (Hsp40) homolog, subfamily C, member 10	1.0289	0.507	0.0223	0.625	No	1.0183	0.478	0.0008	0.952	No	No
160145_at	1810010M01 Rik	RIKEN cDNA 1810010M01 gene	1.0473	0.0889	0.0003	0.0155	No	1.1293	0.266	0.967	0.626	No	No
160146_r_at	Polr2c	polymerase (RNA) II (DNA directed) polypeptide C	1.1924	0.1536	0.0017	0.4853	No	1.1708	0.065	0.0007	0.741	No	No
160147_r_at	H1foo	H1 histone family, member O, oocyte-specific	0.9752	0.7351	0.0014	0.5001	No	1.0129	0.997	0.0003	0.641	No	No
160148_at	Prmt5	Protein arginine N-methyltransferase 5	1.029	0.6587	0.8549	0.2865	No	1.1163	0.102	0.411	0.0225	No	No
160149_at	Rab10	RAB10, member RAS oncogene family	1.0244	0.761	0.2821	0.4989	No	1.0127	0.857	0.479	0.878	No	No
160150_f_at	Cnn3	calponin 3, acidic	1.0914	0.0918	0.5539	0.4317	No	1.0715	0.273	0.0932	0.193	No	No
160151_i_at	Ergic2	ERGIC and golgi 2	1.0204	0.8395	0.0117	0.1759	No	0.9253	0.26	0.0094	0.133	No	No
160152_at	Psmc1	protease (prosome, macropain) 26S subunit, ATPase 1	0.9986	0.9693	0.152	0.6437	No	0.9487	0.109	0.692	0.0227	No	No
160153_at	Dnajc7	DnaJ (Hsp40) homolog, subfamily C, member 7	1.0419	0.5471	0.1883	0.3573	No	1.0163	0.631	0.0038	0.407	No	No
160154_at	Pop4	processing of precursor 4, ribonuclease P/MRP family, (S. cerevisiae)	1.0202	0.7687	0.2278	0.4912	No	1.0883	0.0343	0.0901	0.0083	No	No
160155_at	C86187	expressed sequence C86187	1.0044	0.9405	0.0254	0.5077	No	1.0188	0.725	0.557	0.454	No	No
160156_at	0910001A06 Rik	RIKEN cDNA 0910001A06 gene	1.1627	0.0719	0.0018	0.8868	No	1.2194	0.003	0.0012	0.521	Yes	No
160157_at	Mkrn2	makorin, ring finger protein, 2	0.9449	0.1089	0.1196	0.1931	No	0.8456	0.204	0.0291	0.124	No	No
160158_at	Gpc3	glypican 3	0.9826	0.8229	0.2926	0.4442	No	1.0079	0.987	0.0054	0.445	No	No
160159_at	Ccnb1-rs1 /// Ccnb1	cyclin B1, related sequence 1 /// cyclin B1	1.1135	0.2431	0.0302	0.4988	No	1.0676	0.0689	0.0008	0.0838	No	No
160160_at	Dedd	death effector domain-containing	0.981	0.7075	0.9691	0.911	No	0.8918	0.192	0.189	0.671	No	No
160161_at	Gtrgeo22	gene trap ROSA b-geo 22	0.9439	0.0718	0.0053	0.1781	No	0.9809	0.447	0.0003	0.142	No	No
160162_at	Tagln2	transgelin 2	0.9729	0.2967	0.0007	0.0438	No	0.9734	0.365	0.001	0.532	No	No
160163_at	2210010A19 Rik	RIKEN cDNA 2210010A19 gene	1.0295	0.5131	0.0015	0.1277	No	0.9823	0.311	0	0.0429	No	No
160164_at	Ube2v1 /// LOC545332 /// LOC666853	ubiquitin-conjugating enzyme E2 variant 1 /// similar to ubiquitin-conjugating enzyme E2 variant 1 /// similar to ubiquitin-conjugating enzyme E2 variant 1	1.0118	0.8063	0.1403	0.681	No	0.9638	0.495	0.0453	0.189	No	No
160165_at	Ube2m	ubiquitin-conjugating enzyme E2M (UBC12 homolog, yeast)	1.0721	0.1075	0.0003	0.2293	No	1.1451	0.111	0.0001	0.383	No	No
160166_r_at	2810409H07 Rik	RIKEN cDNA 2810409H07 gene	1.1329	0.2233	0.0246	0.3082	No	1.0339	0.663	0.13	0.404	No	No
160167_at	Nup62	nucleoporin 62	0.9714	0.5713	0.3385	0.8879	No	1.0338	0.305	0.55	0.391	No	No
160168_at	Fbxo3	F-box only protein 3	1.0007	0.9843	0.0516	0.1546	No	0.9926	0.923	0.0068	0.14	No	No
160169_at	Plaa	phospholipase A2, activating protein	0.9811	0.8322	0.776	0.2762	No	0.9956	0.992	0.603	0.406	No	No
160170_at	Stmn3	stathmin-like 3	1.0507	0.3609	0.0021	0.9675	No	1.1344	0.0882	0.0026	0.961	No	No
160171_f_at	Acot9	acyl-CoA thioesterase 9	0.9439	0.3134	0.4399	0.3202	No	0.9377	0.0404	0.0063	0.0309	No	No
160172_at	Gtl2	GTL2, imprinted maternally expressed untranslated mRNA	0.8548	0.0266	0.0541	0.8945	No	0.8358	0.0292	0.0599	0.987	No	No
160173_at	Gtl2	GTL2, imprinted maternally expressed untranslated mRNA	0.9674	0.7033	0.0131	0.4696	No	0.952	0.387	0.0009	0.126	No	No
160174_at	Xab2	XPA binding protein 2	0.9273	0.2559	0.0808	0.3057	No	1.0199	0.604	0.0076	0.476	No	No
160175_at	Ckm	creatine kinase, muscle	1.0406	0.3032	0.3909	0.5147	No	1.0624	0.241	0.293	0.312	No	No
160176_at	Hirip5	histone cell cycle regulation defective interacting protein 5	0.9114	0.1657	0.2238	0.0498	No	0.9167	0.255	0.0183	0.112	No	No
160177_at	Smu1	smu-1 suppressor of mec-8 and unc-52 homolog (C. elegans)	1.0219	0.4298	0.6017	0.5579	No	1.0689	0.0253	0.73	0.649	No	No
160178_r_at	---	---	0.9574	0.5264	0.0198	0.2721	No	1.0772	0.236	0.0043	0.797	No	No
160179_at	Tmem30a	transmembrane protein 30A	1.132	0.0144	0.0062	0.0735	No	1.035	0.556	0.129	0.269	No	No
160180_at	Canx	calnexin	0.9683	0.6467	0.0095	0.2193	No	0.9288	0.444	0.419	0.175	No	No
160181_at	Syp	synaptophysin	0.8141	0.1019	0.0619	0.3403	No	0.9455	0.259	0.0137	0.348	No	No
160182_at	Sfrs6	splicing factor, arginine/serine-rich 6	0.9879	0.8079	0.0256	0.1679	No	0.9776	0.567	0.0266	0.129	No	No
160183_f_at	Tmed7	transmembrane emp24 protein transport domain containing 7	1.1077	0.4205	0.0061	0.7071	No	0.9357	0.365	0.0271	0.332	No	No
160184_at	1200007D18 Rik	RIKEN cDNA 1200007D18 gene	1.0423	0.2025	0.0032	0.0311	No	0.9591	0.211	0.0035	0.086	No	No
160185_at	Tagln3	transgelin 3	0.9155	0.0894	0.0309	0.6905	No	1.0153	0.763	0.702	0.281	No	No
160186_at	C030004A17 Rik	RIKEN cDNA C030004A17 gene	1.0241	0.4194	0.4241	0.2328	No	0.8826	0.32	0.0893	0.116	No	No
160187_at	Pafah1b2	platelet-activating factor acetylhydrolase, isoform 1b, alpha2 subunit	1.0536	0.0851	0.0057	0.0359	No	1.0054	0.884	0.0052	0.569	No	No
160188_at	Nudt4	nudix (nucleoside diphosphate linked moiety X)-type motif 4	0.9562	0.2203	0.0285	0.7211	No	0.9693	0.636	0.0199	0.155	No	No
160189_at	Nudt4	nudix (nucleoside diphosphate linked moiety X)-type motif 4	0.9296	0.1363	0.0244	0.1256	No	0.9197	0.132	0.068	0.193	No	No
160190_at	Syt4	synaptotagmin IV	0.9926	0.9371	0.006	0.5946	No	1.118	0.632	0.0005	0.233	No	No
160191_at	Nat11	N-acetyltransferase 11	0.9757	0.5199	0.0007	0.2593	No	0.9804	0.706	0.106	0.893	No	No
160192_at	Rbmxrt	RNA binding motif protein, X chromosome retrogene	0.9915	0.8433	0.1366	0.9058	No	1.0644	0.175	0.0062	0.53	No	No
160193_at	Ormdl2	ORM1-like 2 (S. cerevisiae)	1.0477	0.3094	0.017	0.6791	No	1.0429	0.436	0.0131	0.212	No	No
160194_at	Gcdh	glutaryl-Coenzyme A dehydrogenase	0.9859	0.5797	0.6073	0.3896	No	0.958	0.182	0.28	0.0689	No	No
160195_at	1200013P24 Rik	RIKEN cDNA 1200013P24 gene	1.0846	0.2545	0.1867	0.9696	No	1.0985	0.0899	0.0394	0.824	No	No
160196_at	Smap1	stromal membrane-associated protein 1	1.0128	0.837	0.0009	0.5983	No	1.0403	0.513	0.0004	0.398	No	No
160197_at	Pycrl	pyrroline-5-carboxylate reductase-like	0.983	0.5053	0.0047	0.1005	No	1.0038	0.89	0.0058	0.704	No	No
160198_at	Klhl9	Kelch-like 9 (Drosophila)	0.8786	0.2836	0.5872	0.5269	No	0.9049	0.0441	0.108	0.0911	No	No
160199_at	Hnrpc /// LOC620883 /// LOC676606	heterogeneous nuclear ribonucleoprotein C /// similar to heterogeneous nuclear ribonucleoprotein C /// similar to heterogeneous nuclear ribonucleoprotein C	1.1003	0.4652	0.107	0.8371	No	1.1126	0.484	0.295	0.635	No	No

160200_at	3230401D17 Rik	RIKEN cDNA 3230401D17 gene	0.9938	0.955	0.0354	0.8357	No	0.9571	0.569	0.0111	0.795	No	No
160201_r_at	Slc33a1	solute carrier family 33 (acetyl-CoA transporter), member 1	1.0084	0.9046	0.0512	0.6665	No	0.9508	0.599	0.0083	0.389	No	No
160202_at	Atp6ap2	ATPase, H+ transporting, lysosomal accessory protein 2	1.0245	0.6969	0.0042	0.7236	No	0.9109	0.204	0.073	0.421	No	No
160203_at	Dnajc9	DnaJ (Hsp40) homolog, subfamily C, member 9	1.0859	0.457	0.5032	0.7675	No	1.1263	0.135	0.827	0.196	No	No
160204_at	Prr6	proline-rich polypeptide 6	1.0486	0.3667	0.0445	0.1454	No	0.96	0.581	0.0226	0.124	No	No
160205_f_at	Rnf11	ring finger protein 11	0.9846	0.8322	0.0134	0.5849	No	0.9876	0.769	0.0286	0.386	No	No
160206_at	Rcc2	regulator of chromosome condensation 2	1.0553	0.0739	0.0285	0.8851	No	1.078	0.188	0.166	0.959	No	No
160207_at	Acly	ATP citrate lyase	1.0779	0.1529	0.0042	0.3389	No	1.0445	0.525	0.0171	0.805	No	No
160208_at	Sf3b3	splicing factor 3b, subunit 3	0.9631	0.2941	0.1316	0.3138	No	0.9676	0.395	0.102	0.356	No	No
160209_at	Rnf44	ring finger protein 44	0.9661	0.6022	0.0099	0.6207	No	0.9647	0.168	0.0002	0.467	No	No
160210_at	4930539H15 Rik	RIKEN cDNA 4930539H15 gene	1.0422	0.2858	0.2962	0.158	No	1.0975	0.0893	0.156	0.0453	No	No
160211_at	Yipf3	Yip1 domain family, member 3	0.8857	0.0044	0.0012	0.0111	Yes	0.9296	0.0464	0.0021	0.091	No	Yes
160212_at	Ttc4	tetratricopeptide repeat domain 4	1.0559	0.0006	0.0001	0.0001	No	1.0354	0.0951	0.0011	0.042	No	No
160213_at	Reg1	regenerating islet-derived 1	1.0028	0.9099	0.0202	0.0902	No	1.0051	0.888	0.61	0.865	No	No
160214_at	Lancl2	LanC (bacterial lantibiotic synthetase component C)-like 2	1.1666	0.1141	0.0413	0.2576	No	1.0523	0.336	0.0131	0.0646	No	No
160215_at	Aes	amino-terminal enhancer of split	1.028	0.6782	0.0006	0.9213	No	1.0302	0.568	0.0007	0.607	No	No
160216_f_at	Obox1 /// LOC666466	oocyte specific homeobox 1 /// similar to oocyte specific homeobox 2	0.9914	0.6001	0.0185	0.1335	No	1.0404	0.966	0.0738	0.0657	No	No
160217_at	2310001A20 Rik	RIKEN cDNA 2310001A20 gene	1.0145	0.7273	0.0053	0.4424	No	0.9886	0.756	0.0008	0.76	No	No
160218_at	2310056P07 Rik	RIKEN cDNA 2310056P07 gene	1.0754	0.5094	0.8074	0.8402	No	1.022	0.844	0.718	0.476	No	No
160219_r_at	Meig1	meiosis expressed gene 1	0.9676	0.404	0.1388	0.1083	No	0.8716	0.272	0.925	0.225	No	No
160220_at	Zfp110	zinc finger protein 110	0.9421	0.3612	0.6542	0.2957	No	0.9828	0.736	0.117	0.307	No	No
160221_at	Lgals2	lectin, galactose-binding, soluble 2	1.0308	0.4958	0.0821	0.5211	No	0.9831	0.95	0.945	0.409	No	No
160222_i_at	Letm1	Leucine zipper-EF-hand containing transmembrane protein 1	1.0426	0.5095	0.0187	0.2219	No	1.1085	0.141	0.0279	0.061	No	No
160223_at	2610511O17 Rik	RIKEN cDNA 2610511O17 gene	0.9269	0.3219	0.1075	0.8687	No	0.8399	0.0803	0.297	0.265	No	No
160224_at	Slc35a4	Solute carrier family 35, member A4	0.9969	0.9723	0.3737	0.8367	No	0.9945	0.867	0.0214	0.109	No	No
160225_at	Gtf2b	general transcription factor IIB	0.9695	0.5118	0.0004	0.0264	No	0.952	0.391	0.0081	0.118	No	No
160226_at	Gfm1	G elongation factor, mitochondrial 1	0.9108	0.1267	0.0047	0.4652	No	0.9754	0.456	0.037	0.15	No	No
160227_s_at	Bysl	bystin-like	1.0408	0.3625	0.0475	0.0328	No	1.041	0.572	0.0235	0.0542	No	No
160228_at	Cldnd1	claudin domain containing 1	0.988	0.8231	0.0473	0.3066	No	0.9364	0.0733	0.101	0.166	No	No
160229_at	Osbpl9	oxysterol binding protein-like 9	1.1341	0.0892	0.7702	0.6023	No	1.1012	0.387	0.0469	0.768	No	No
160230_at	Cox17	cytochrome c oxidase, subunit XVII assembly protein homolog (yeast)	0.964	0.6506	0.0304	0.1863	No	0.9785	0.588	0.006	0.287	No	No
160231_at	Farsla	phenylalanine-tRNA synthetase-like, alpha subunit	1.0188	0.628	0.2123	0.181	No	1.0679	0.199	0.103	0.0714	No	No
160232_at	St6galnac4	ST6 (alpha-N-acetyl-neuraminyl-2,3-beta-galactosyl-1,3)-N-acetylgalactosaminide alpha-2,6-sialyltransferase 4	0.9675	0.4592	0.1749	0.3744	No	1.0705	0.163	0.127	0.179	No	No
160233_at	Ak3	adenylate kinase 3	0.8279	0.052	0.354	0.0487	No	0.8307	0.009	0.0468	0.0118	Yes	No
160234_at	Usp1	ubiquitin specific peptdiase 1	1.1618	0.1171	0.0075	0.6664	No	1.0363	0.874	0.0049	0.206	No	No
160235_at	Tmem147	transmembrane protein 147	1.1718	0.0124	0.0068	0.2186	No	1.0642	0.223	0.0326	0.446	No	No
160236_at	9630044O09 Rik	RIKEN cDNA 9630044O09 gene	0.962	0.5496	0.0166	0.5923	No	0.9229	0.501	0.0928	0.431	No	No
160237_at	Ndufa6	NADH dehydrogenase (ubiquinone) 1 alpha subcomplex, 6 (B14)	0.991	0.9302	0.6462	0.9944	No	1.0355	0.767	0.746	0.868	No	No
160238_at	Ciapin1	cytokine induced apoptosis inhibitor 1	1.021	0.6863	0.1404	0.1906	No	1.0013	0.936	0.102	0.472	No	No
160239_at	Psmd6	proteasome (prosome, macropain) 26S subunit, non-ATPase, 6	1.099	0.2006	0.3025	0.7865	No	1.0807	0.32	0.83	0.916	No	No
160240_at	1110003E01 Rik	RIKEN cDNA 1110003E01 gene	0.9667	0.4058	0.0325	0.2877	No	0.9382	0.358	0.116	0.261	No	No
160241_at	Adam17	a disintegrin and metallopeptidase domain 17	1.0261	0.4101	0.079	0.4488	No	0.9585	0.0968	0.805	0.574	No	No
160242_at	Flcn	folliculin	0.9617	0.2755	0.012	0.3883	No	0.935	0.0708	0.001	0.112	No	No
160243_r_at	Myh8	myosin, heavy polypeptide 8, skeletal muscle, perinatal	0.5061	0.0006	0.0007	0.0131	Yes	0.5686	0.0001	0.0002	0.001	No	No
160244_at	Fem1a	feminization 1 homolog a (C. elegans)	0.9077	0.0191	0.0146	0.0178	No	0.8996	0.121	0.0231	0.0244	No	No
160245_at	1110034O07 Rik	RIKEN cDNA 1110034O07 gene	0.9878	0.7928	0.146	0.3846	No	0.945	0.0803	0.0011	0.556	No	No
160246_at	Tnfaip8	tumor necrosis factor, alpha-induced protein 8	1.0173	0.7818	0.044	0.0954	No	0.9458	0.162	0.0004	0.829	No	No
160247_at	Ube2v2	ubiquitin-conjugating enzyme E2 variant 2	1.0465	0.3504	0.0021	0.6896	No	1.1562	0.0346	0.0018	0.0111	No	No
160248_at	Fubp1	far upstream element (FUSE) binding protein 1	0.9824	0.6765	0.5266	0.4046	No	1.0552	0.274	0.173	0.713	No	No
160249_at	Tpd52	tumor protein D52	1.0669	0.1707	0.0002	0.8814	No	1.0089	0.537	0.0005	0.0717	No	No
160250_at	---	---	1.0832	0.0016	0.0021	0.0002	No	1.0097	0.76	0.749	0.761	No	No
160251_at	2610312B22 Rik	RIKEN cDNA 2610312B22 gene	1.027	0.5911	0.1824	0.769	No	1.072	0.118	0.978	0.237	No	No
160252_at	Cltc	clathrin, heavy polypeptide (Hc)	0.9872	0.8002	0.0046	0.1061	No	0.9724	0.671	0.0104	0.129	No	No
160253_at	Ifitm3	interferon induced transmembrane protein 3	0.9882	0.7982	0.1633	0.0595	No	0.9689	0.307	0.0028	0.433	No	No
160254_at	Ifitm1	interferon induced transmembrane protein 1	1.0162	0.7185	0.0487	0.6227	No	1.0056	0.77	0.0026	0.0852	No	No
160255_at	Ahnak	AHNAK nucleoprotein (desmoyokin)	0.8546	0.0029	0.0796	0.4567	Yes	0.8507	0.0367	0.0221	0.378	No	Yes
160256_at	Tomm7	translocase of outer mitochondrial membrane 7 homolog (yeast)	0.9508	0.6549	0.7437	0.8058	No	0.9912	0.931	0.395	0.642	No	No
160257_at	Fkbp1a	FK506 binding protein 1a	1.0561	0.2493	0.5666	0.2674	No	1.0546	0.187	0.389	0.362	No	No
160258_at	Magmas	mitochondria-associated protein involved in granulocyte-macrophage colony-stimulating factor signal transduction	0.9655	0.5749	0.0306	0.5719	No	0.9518	0.308	0.0147	0.58	No	No
160259_at	Trim61	tripartite motif-containing 61	1.0871	0.3324	0.0658	0.8782	No	0.9517	0.781	0.0027	0.234	No	No
160260_at	Thoc7	THO complex 7 homolog (Drosophila)	0.9787	0.788	0.0299	0.7671	No	0.8764	0.054	0.0016	0.304	No	No
160261_i_at	Lats2	large tumor suppressor 2	0.8187	0.0643	0.0203	0.2527	No	0.8538	0.12	0.0251	0.411	No	No
160262_at	Mtch2	mitochondrial carrier homolog 2 (C. elegans)	1.0933	0.0489	0.3778	0.1395	No	1.062	0.192	0.0227	0.655	No	No
160263_r_at	Ndfip2	Nedd4 family interacting protein 2	1.109	0.0753	0.0008	0.9673	No	1.1392	0.0688	0.0011	0.395	No	No
160264_s_at	Pcp4l1	Purkinje cell protein 4-like 1	0.9334	0.3648	0.8747	0.0944	No	0.9691	0.589	0.0423	0.983	No	No
160265_at	Eif5	eukaryotic translation initiation factor 5	0.9483	0.1867	0.6446	0.5432	No	0.9614	0.289	0.0015	0.692	No	No
160266_r_at	Bxdc2	brix domain containing 2	1.0397	0.1992	0.842	0.1742	No	1.054	0.168	0.001	0.417	No	No
160267_at	Tmem93	transmembrane protein 93	0.9828	0.791	0.134	0.8182	No	1.031	0.694	0.232	0.443	No	No
160268_at	Pdcl3	phosducin-like 3	1.0095	0.7101	0.1107	0.0034	No	0.9983	0.986	0.114	0.207	No	No
160269_at	Gfer	growth factor, erv1 (S. cerevisiae)-like (augmenter of liver regeneration)	0.9511	0.1373	0.286	0.0027	No	0.9401	0.13	0.0028	0.0335	No	No
160270_at	Lman1	lectin, mannose-binding, 1	1.0084	0.8621	0.6567	0.5475	No	0.9355	0.188	0.909	0.427	No	No
160271_at	0610007C21 Rik	RIKEN cDNA 0610007C21 gene	0.9765	0.6753	0.2543	0.2358	No	0.913	0.229	0.668	0.125	No	No
160272_at	Cbx3 /// LOC629578 /// LOC633016 /// LOC672750	chromobox homolog 3 (Drosophila HP1 gamma) /// similar to chromobox homolog 3 /// similar to chromobox homolog 3 /// similar to chromobox homolog 3	0.8818	0.3213	0.0736	0.6976	No	0.9725	0.707	0.0094	0.955	No	No
160273_at	Zfp36l2	zinc finger protein 36, C3H type-like 2	0.9769	0.7642	0.0639	0.8963	No	0.9482	0.341	0.0159	0.7	No	No
160274_at	Krt1-4	keratin complex 1, acidic, gene 4	1.1095	0.1844	0.7572	0.145	No	1.1297	0.08	0.0581	0.152	No	No
160275_at	Sepx1	selenoprotein X 1	0.9674	0.3388	0.0225	0.6394	No	0.8854	0.0026	0.23	0.0107	Yes	No
160276_at	Cd97	CD97 antigen	0.991	0.8915	0.5523	0.0894	No	1.0017	0.94	0.718	0.0088	No	No
160277_at	Stx12	syntaxin 12	0.9541	0.3767	0.5096	0.521	No	0.9859	0.804	0.182	0.947	No	No
160278_at	2810428I15 Rik	RIKEN cDNA 2810428I15 gene	1.0317	0.5708	0.0022	0.2631	No	1.0414	0.273	0.0009	0.165	No	No
160279_at	Dnttip2	deoxynucleotidyltransferase, terminal, interacting protein 2	0.9703	0.5538	0.3903	0.1656	No	0.9842	0.806	0.521	0.385	No	No
160280_at	Cav1	caveolin, caveolae protein 1	0.9608	0.1114	0.0019	0.9227	No	0.9289	0.0175	0.0002	0.258	No	No
160281_at	Lsm14a	LSM14 homolog A (SCD6, S. cerevisiae)	1.0278	0.6376	0.0008	0.191	No	1.0144	0.935	0.0031	0.511	No	No

160282_at	4931406I20 Rik	RIKEN cDNA 4931406I20 gene	0.9567	0.2165	0.134	0.2434	No	1.0369	0.148	0.0017	0.195	No	No
160283_at	Rsl1d1	ribosomal L1 domain containing 1	0.9409	0.0208	0.0011	0.0342	No	0.9128	0.172	0.0196	0.462	No	No
160284_at	Ndufa10	NADH dehydrogenase (ubiquinone) 1 alpha subcomplex 10	1.0376	0.3668	0.0835	0.3752	No	0.9	0.181	0.0483	0.335	No	No
160285_at	Dhx40	DEAH (Asp-Glu-Ala-His) box polypeptide 40	0.8525	0.0105	0.0289	0.1132	No	0.8828	0.0021	0	0.255	Yes	Yes
160286_at	Dek	DEK oncogene (DNA binding)	1.0782	0.2446	0.114	0.0874	No	1.0704	0.317	0.0728	0.587	No	No
160287_at	Map1lc3b	microtubule-associated protein 1 light chain 3 beta	0.9164	0.1291	0.026	0.0178	No	0.9165	0.126	0.0371	0.0934	No	No
160288_at	Map1lc3b	microtubule-associated protein 1 light chain 3 beta	0.934	0.2076	0.3939	0.0448	No	0.9199	0.0434	0.0071	0.0605	No	No
160289_s_at	Dlst	dihydrolipoamide S-succinyltransferase (E2 component of 2-oxo-glutarate complex)	0.9966	0.933	0.2262	0.3661	No	1.0145	0.733	0.0046	0.763	No	No
160290_at	Ide	insulin degrading enzyme	0.9532	0.1719	0	0.9567	No	1.1012	0.19	0.0017	0.774	No	No
160291_at	Sec61a1	Sec61 alpha 1 subunit (S. cerevisiae)	1.1178	0.2347	0.0284	0.2391	No	1.0543	0.52	0.348	0.0849	No	No
160292_at	Ecgf1	endothelial cell growth factor 1 (platelet-derived)	0.9501	0.2525	0.0865	0.7727	No	0.9384	0.0828	0.0037	0.116	No	No
160293_at	Wdr61	WD repeat domain 61	1.0649	0.2787	0.736	0.9823	No	1.0329	0.407	0.0083	0.546	No	No
160294_at	Snapc2	small nuclear RNA activating complex, polypeptide 2	0.9969	0.8812	0.0014	0.4858	No	1.0386	0.062	0.0003	0.01	No	No
160295_at	Slc25a11	solute carrier family 25 (mitochondrial carrier oxoglutarate carrier), member 11	0.9705	0.5801	0.2728	0.0813	No	0.934	0.24	0.0104	0.596	No	No
160296_at	Wsb2	WD repeat and SOCS box-containing 2	1.0701	0.625	0.342	0.6402	No	0.9933	0.96	0.0039	0.0436	No	No
160297_at	Ccdc58	coiled-coil domain containing 58	1.0355	0.6976	0.8129	0.468	No	0.9849	0.832	0.0901	0.407	No	No
160298_at	Ccdc80	coiled-coil domain containing 80	0.976	0.4565	0.0329	0.815	No	0.9078	0.349	0.111	0.512	No	No
160299_at	Rwdd1	RWD domain containing 1	1.082	0.1515	0.0042	0.1114	No	1.0279	0.309	0.002	0.155	No	No
160300_at	Tff1	trefoil factor 1	1.0015	0.9467	0.9257	0.0036	No	1.0393	0.382	0.846	0.025	No	No
160301_at	Riok3	RIO kinase 3 (yeast)	0.9188	0.1824	0.139	0.6488	No	0.9532	0.255	0.0104	0.871	No	No
160302_at	1200003C05 Rik	RIKEN cDNA 1200003C05 gene	1.0124	0.8488	0.933	0.3706	No	0.9838	0.624	0.548	0.0477	No	No
160303_at	Acad9	acyl-Coenzyme A dehydrogenase family, member 9	0.9514	0.0354	0.0054	0.7231	No	0.9427	0.242	0.13	0.591	No	No
160304_r_at	Psmd11	Proteasome (prosome, macropain) 26S subunit, non-ATPase, 11	1.0078	0.8612	0.0194	0.0866	No	0.9386	0.0476	0.0741	0.151	No	No
160305_at	Psmd11	proteasome (prosome, macropain) 26S subunit, non-ATPase, 11	1.0776	0.1386	0.0008	0.1568	No	1.1562	0.0477	0.0016	0.652	No	No
160306_at	Thrsp	thyroid hormone responsive SPOT14 homolog (Rattus)	1.0247	0.6107	0.0045	0.0652	No	0.9099	0.211	0.0097	0.15	No	No
160307_at	Pi4k2a	phosphatidylinositol 4-kinase type 2 alpha	0.9175	0.0388	0.0379	0.0867	No	0.9342	0.0981	0.366	0.319	No	No
160308_at	Msn	moesin	0.9807	0.8215	0.0752	0.2869	No	1.0232	0.795	0.419	0.101	No	No
160309_at	Map3k7ip2	mitogen-activated protein kinase kinase kinase 7 interacting protein 2	0.9311	0.1988	0.0148	0.3542	No	0.9467	0.331	0.0137	0.635	No	No
160310_at	D19Bwg135 7e	DNA segment, Chr 19, Brigham & Women's Genetics 1357 expressed	0.9503	0.1941	0.5315	0.6364	No	0.9052	0.0574	0.068	0.319	No	No
160311_at	Plxnb2	plexin B2	0.9305	0.1767	0.3134	0.4752	No	0.9891	0.782	0.0423	0.295	No	No
160312_at	Cmtm6	CKLF-like MARVEL transmembrane domain containing 6	1.0012	0.9667	0.0344	0.5628	No	0.9914	0.887	0.971	0.344	No	No
160313_at	A730098D12 Rik	RIKEN cDNA A730098D12 gene	1.014	0.8638	0.006	0.2803	No	1.0026	0.731	0.0059	0.131	No	No
160314_at	Ppa1	pyrophosphatase (inorganic) 1	0.9036	0.0354	0.0001	0.0109	No	0.9104	0.0162	0	0.0259	No	No
160315_at	2310005N03 Rik /// AI503316	RIKEN cDNA 2310005N03 gene /// expressed sequence AI503316	1.0923	0.2375	0.0033	0.7002	No	0.9951	0.959	0	0.689	No	No
160316_at	Hnrpu	Heterogeneous nuclear ribonucleoprotein U	1.0628	0.4337	0.0288	0.1594	No	0.963	0.811	0.0081	0.178	No	No
160317_at	Rab34	RAB34, member of RAS oncogene family	0.9672	0.3804	0.0064	0.0196	No	0.9825	0.988	0.0023	0.0532	No	No
160318_at	Stub1	STIP1 homology and U-Box containing protein 1	1.0862	0.226	0.073	0.6067	No	1.1394	0.0306	0.0014	0.821	No	No
160319_at	Sparcl1	SPARC-like 1 (mast9, hevin)	1.1128	0.0045	0.0003	0.3235	Yes	1.211	0.0116	0.014	0.0817	No	Yes
160320_at	Sorbs1	sorbin and SH3 domain containing 1	0.9058	0.0516	0.066	0.4532	No	0.9082	0.0571	0.31	0.947	No	No
160321_at	Za20d2	zinc finger, A20 domain containing 2	1.0538	0.3797	0.0056	0.1937	No	1.0505	0.214	0.0008	0.9	No	No
160322_at	Tmco1	transmembrane and coiled-coil domains 1	1.0885	0.2597	0.0763	0.8356	No	0.9473	0.382	0.115	0.859	No	No
160323_at	Sra1	steroid receptor RNA activator 1	1.0714	0.1267	0.0345	0.1841	No	1.0707	0.3	0.0139	0.611	No	No
160324_at	Rpa3	replication protein A3	1.0163	0.7665	0.663	0.5475	No	1.129	0.019	0.0334	0.875	No	No
160325_r_at	Cdv3	carnitine deficiency-associated gene expressed in ventricle 3	0.995	0.8578	0.0133	0.0996	No	0.9187	0.0588	0.138	0.195	No	No
160326_at	Cdv3	carnitine deficiency-associated gene expressed in ventricle 3	0.9838	0.6244	0.0008	0.1347	No	1.0217	0.572	0.0012	0.608	No	No
160327_at	Dctn6	dynactin 6	0.9942	0.8716	0.4985	0.766	No	1.0182	0.552	0.14	0.391	No	No
160328_at	Prss15	protease, serine, 15	0.9356	0.1382	0.006	0.7432	No	0.9516	0.175	0.0014	0.309	No	No
160329_at	1110049F12 Rik	RIKEN cDNA 1110049F12 gene	0.9517	0.4403	0.9439	0.5717	No	1.0339	0.542	0.908	0.953	No	No
160330_at	Chordc1	cysteine and histidine-rich domain (CHORD)-containing, zinc-binding protein 1	0.9475	0.183	0.0031	0.0621	No	0.9545	0.516	0.008	0.154	No	No
160331_at	Zbtb7b	Vzinc finger and BTB domain containing 7B	1.0025	0.9473	0.0024	0.7653	No	0.8693	0.129	0.603	0.561	No	No
160332_at	Pdgfra	Platelet derived growth factor receptor, alpha polypeptide	0.9478	0.17	0.0122	0.1181	No	0.97	0.226	0.218	0.0313	No	No
160333_at	1110008F13 Rik	RIKEN cDNA 1110008F13 gene	0.9667	0.409	0.0056	0.1561	No	0.9835	0.895	0.0011	0.0744	No	No
160334_at	Tmem134	transmembrane protein 134	0.9311	0.3385	0.0418	0.6625	No	0.9114	0.0631	0.0106	0.0174	No	No
160335_at	Gclm	glutamate-cysteine ligase , modifier subunit	0.9345	0.0967	0.0063	0.0073	No	0.9211	0.0016	0.0001	0.0005	No	No
160336_at	Icmt	isoprenylcysteine carboxyl methyltransferase	1.0121	0.8278	0.4656	0.4198	No	1.0216	0.591	0.731	0.0629	No	No
160337_at	Isyna1	myo-inositol 1-phosphate synthase A1	1.0301	0.1324	0.0004	0.0564	No	1.0294	0.246	0.0021	0.0743	No	No
160338_at	Ankrd13a	ankyrin repeat domain 13a	1.0521	0.4179	0.4521	0.6287	No	0.9962	0.912	0.708	0.46	No	No
160339_at	Pycr2	pyrroline-5-carboxylate reductase family, member 2	1.0378	0.0522	0.0003	0.7836	No	1.033	0.453	0.0007	0.992	No	No
160340_at	D8Ertd738e	DNA segment, Chr 8, ERATO Doi 738, expressed	1.016	0.7573	0.5951	0.3111	No	1.0065	0.939	0.192	0.266	No	No
160341_at	Jtv1	JTV1 gene	0.9513	0.0029	0.0001	0.0013	No	0.9998	0.961	0.718	0.19	No	No
160342_r_at	Ndufb2	NADH dehydrogenase (ubiquinone) 1 beta subcomplex, 2	0.9203	0.4197	0.0016	0.2579	No	0.9258	0.262	0	0.0184	No	No
160343_at	Srp19	signal recognition particle 19	1.0374	0.4747	0.0006	0.0115	No	1.0483	0.393	0.0002	0.0011	No	No
160344_at	Npc2	Niemann Pick type C2	0.9915	0.7035	0.0035	0.2454	No	1.0059	0.838	0.0125	0.733	No	No
160345_at	Mrpl34	mitochondrial ribosomal protein L34	1.023	0.4067	0.0481	0.6662	No	1.0175	0.61	0.215	0.674	No	No
160346_at	Dalrd3	DALR anticodon binding domain containing 3	1.0002	0.9953	0.1053	0.2143	No	1.0314	0.495	0.468	0.141	No	No
160347_at	D15Ertd785 e	DNA segment, Chr 15, ERATO Doi 785, expressed	1.0332	0.4306	0.0173	0.8762	No	1.0894	0.0855	0.0297	0.335	No	No
160348_at	Ranbp3	RAN binding protein 3	0.9439	0.1499	0.3586	0.0461	No	0.9635	0.332	0.793	0.666	No	No
160349_at	Cndp2	CNDP dipeptidase 2 (metallopeptidase M20 family)	0.9892	0.8088	0.1765	0.4213	No	0.9933	0.884	0.0184	0.575	No	No
160350_at	Gstz1	glutathione transferase zeta 1 (maleylacetoacetate isomerase)	0.9388	0.1834	0.0086	0.1106	No	0.9414	0.352	0.0644	0.373	No	No
160351_at	Rnpep	arginyl aminopeptidase (aminopeptidase B)	0.9299	0.1594	0.0014	0.2909	No	1.0689	0.228	0.219	0.0625	No	No
160352_at	Pcbp4	poly(rC) binding protein 4	1.0358	0.3261	0.0999	0.3617	No	0.9698	0.339	0.936	0.219	No	No
160353_i_at	Mapkapk2	MAP kinase-activated protein kinase 2	1.1778	0.1697	0.0233	0.6135	No	1.1748	0.0242	0.0033	0.992	No	No
160354_at	Ldb3	LIM domain binding 3	0.8026	0.0075	0.7008	0.0066	No	0.857	0.0013	0.0547	0.0024	No	No
160355_at	Maz	MYC-associated zinc finger protein (purine-binding transcription factor)	0.9699	0.5787	0.0178	0.3667	No	0.9996	0.775	0.0047	0.0712	No	No
160356_at	Cmpk	cytidylate kinase	1.0804	0.1241	0.0083	0.1836	No	1.1502	0.0275	0.0003	0.496	No	No
160357_at	Dazl	deleted in azoospermia-like	0.9381	0.2217	0.0286	0.833	No	1.005	0.813	0.0003	0.782	No	No
160358_at	Cd34	CD34 antigen	0.9656	0.3057	0.0552	0.8951	No	0.949	0.106	0.007	0.0381	No	No
160359_at	1190002H23 Rik	RIKEN cDNA 1190002H23 gene	1.0161	0.8514	0.2452	0.2513	No	0.8824	0.0365	0.0124	0.175	No	No
160360_at	38975	selenoprotein	1.0705	0.0484	0.4221	0.005	No	0.9864	0.25	0.0003	0.0732	No	No
160361_at	Trappc4	trafficking protein particle complex 4	1.0202	0.6872	0.3563	0.032	No	1.0078	0.969	0.238	0.0283	No	No
160362_at	Mat2a	methionine adenosyltransferase II, alpha	0.9346	0.1425	0.005	0.9384	No	0.9136	0.161	0.0287	0.841	No	No
160363_at	Vps72	vacuolar protein sorting 72 (yeast)	0.921	0.0551	0.0024	0.0338	No	0.9331	0.0719	0.0014	0.0625	No	No
160364_at	Sfrs10	splicing factor, arginine/serine-rich 10 (transformer 2 homolog, Drosophila)	1.136	0.1444	0.0347	0.5071	No	1.1262	0.0698	0.0131	0.441	No	No
160365_at	Eif2s2	eukaryotic translation initiation factor 2, subunit 2 (beta)	0.9645	0.6018	0.0319	0.4079	No	1.0473	0.558	0.0477	0.883	No	No
160366_at	BC031181	cDNA sequence BC031181	0.9914	0.847	0.7335	0.1686	No	0.9643	0.515	0.14	0.716	No	No
160367_at	Pdlim7	PDZ and LIM domain 7	1.0081	0.8752	0.1367	0.2257	No	0.989	0.816	0.0132	0.765	No	No
160368_at	Sfrs7	splicing factor, arginine/serine-rich 7	0.978	0.6838	0.0633	0.524	No	0.9802	0.82	0.0372	0.725	No	No
160369_at	Dhcr24	24-dehydrocholesterol reductase	1.1133	0.1613	0.5925	0.0832	No	1.0594	0.415	0.302	0.672	No	No
160370_at	Dppa5	developmental pluripotency associated 5	1.0041	0.9439	0.0509	0.555	No	1.0754	0.333	0.609	0.146	No	No
160371_at	Arl6ip1	ADP-ribosylation factor-like 6 interacting protein 1	0.9987	0.9628	0.0205	0.6833	No	1.0126	0.755	0.0078	0.295	No	No
160372_at	Stk11	serine/threonine kinase 11	0.9842	0.7817	0.2622	0.7265	No	1.0043	0.982	0.0015	0.624	No	No
160373_i_at	Sdpr	serum deprivation response	0.9079	0.3075	0.9313	0.7817	No	0.793	0.105	0.145	0.262	No	No

160374_r_at	Ptbp2	polypyrimidine tract binding protein 2	1.2449	0.002	0	0.2275	Yes	1.4669	0.147	0.0126	0.599	No	No
160375_at	Car3	carbonic anhydrase 3	0.9954	0.9287	0.0011	0.3303	No	0.9947	0.719	0.0036	0.0784	No	No
160376_at	Trp53inp2	tumor protein p53 inducible nuclear protein 2	0.9138	0.0578	0.0026	0.1264	No	0.9373	0.0617	0.0002	0.18	No	No
160377_at	Tardbp	TAR DNA binding protein	1.0613	0.5185	0.0122	0.3422	No	1.0115	0.755	0.0033	0.449	No	No
160378_at	Tmem109	transmembrane protein 109	1.0331	0.3972	0.0003	0.4191	No	1.0276	0.567	0.0002	0.01	No	No
160379_at	Epb4.1	erythrocyte protein band 4.1	0.9854	0.7099	0.0061	0.4389	No	0.9464	0.432	0.444	0.475	No	No
160380_at	Becn1	beclin 1 (coiled-coil, myosin-like BCL2-interacting protein)	1.0376	0.3511	0.0474	0.6682	No	1.031	0.468	0.205	0.555	No	No
160381_at	Mrpl50	mitochondrial ribosomal protein L50	1.003	0.8398	0.011	0.1322	No	0.9465	0.265	0.0242	0.0836	No	No
160382_at	1110014J01 Rik	RIKEN cDNA 1110014J01 gene	0.977	0.3612	0.0319	0.0172	No	0.9825	0.62	0.0298	0.606	No	No
160383_at	Cox7a2l	cytochrome c oxidase subunit VIIa polypeptide 2-like	0.9603	0.1679	0.3658	0.0305	No	0.9484	0.142	0.0354	0.917	No	No
160384_at	Bat1a	HLA-B-associated transcript 1A	1.0487	0.0318	0.0083	0.4777	No	1.1174	0.0013	0.0008	0.0029	No	No
160385_at	Gcsh	glycine cleavage system protein H (aminomethyl carrier)	1.0106	0.8789	0.4072	0.8367	No	1.016	0.664	0.0528	0.188	No	No
160386_at	C130052I12 Rik	RIKEN cDNA C130052I12 gene	1.0644	0.1524	0.0089	0.2892	No	1.1032	0.022	0.0012	0.107	No	No
160387_at	Cd302	CD302 antigen	1.0324	0.7601	0.7354	0.5213	No	0.9752	0.8	0.586	0.0697	No	No
160388_at	Sc4mol	sterol-C4-methyl oxidase-like	1.3369	0.0298	0.146	0.7519	No	1.3298	0.0223	0.0218	0.717	No	No
160389_r_at	Rps27l	ribosomal protein S27-like	0.9036	0.1755	0.0003	0.9005	No	1.0282	0.722	0	0.0234	No	No
160390_at	Sdf4	stromal cell derived factor 4	1.0326	0.6822	0.0992	0.6382	No	0.9776	0.677	0.342	0.996	No	No
160391_at	Fads1	fatty acid desaturase 1	1.1401	0.0058	0.0002	0.7152	Yes	1.1004	0.112	0.0204	0.102	No	No
160392_at	B430110G05 Rik	RIKEN cDNA B430110G05 gene	1.0083	0.8885	0.0181	0.062	No	1.0131	0.487	0.0086	0.009	No	No
160393_at	Etnk1	ethanolamine kinase 1	1.0299	0.7441	0.0037	0.6695	No	0.9898	0.678	0.0043	0.153	No	No
160394_at	Ttc15	tetratricopeptide repeat domain 15	0.9806	0.826	0.0425	0.7258	No	1.0525	0.496	0.0159	0.697	No	No
160395_at	Nudcd2	NudC domain containing 2	1.1961	0.0221	0.0007	0.3799	No	1.2082	0.0208	0.0022	0.229	No	No
160396_at	Lass2	longevity assurance homolog 2 (S. cerevisiae)	1.0199	0.5121	0.0323	0.4133	No	1.098	0.271	0.0089	0.259	No	No
160397_at	Ik	IK cytokine	1.0386	0.6218	0.0483	0.7727	No	1.0426	0.594	0.946	0.99	No	No
160398_at	Nol7	nucleolar protein 7	1.0427	0.5847	0.0514	0.8847	No	0.9838	0.899	0.0453	0.377	No	No
160399_r_at	H2afy	H2A histone family, member Y	1.1809	0.1304	0.0016	0.7778	No	1.2181	0.197	0.038	0.698	No	No
160400_at	2810422B04 Rik	RIKEN cDNA 2810422B04 gene	0.9951	0.9393	0.7708	0.1959	No	1.0365	0.451	0.0531	0.469	No	No
160401_r_at	Cnot7	CCR4-NOT transcription complex, subunit 7	1.0389	0.4267	0.0213	0.3003	No	0.9802	0.61	0.0171	0.108	No	No
160402_at	Tceb2	transcription elongation factor B (SIII), polypeptide 2	1.0685	0.3966	0.0268	0.5226	No	1.0076	0.732	0.014	0.229	No	No
160403_at	Selk	selenoprotein K	1.0193	0.5522	0.0026	0.1722	No	0.8812	0.0912	0.471	0.0845	No	No
160404_at	Hils1	histone H1-like protein in spermatids 1	0.9529	0.0267	0.0011	0.1295	No	0.9223	0.172	0.188	0.047	No	No
160405_at	Dhps	deoxyhypusine synthase	1.0135	0.8591	0.5959	0.4975	No	1.1033	0.098	0.259	0.56	No	No
160406_at	Ctsk	cathepsin K	0.9146	0.244	0.6331	0.3301	No	0.9067	0.0747	0.0606	0.0883	No	No
160407_at	Actr1a	ARP1 actin-related protein 1 homolog A (yeast)	0.9527	0.4059	0.2014	0.5714	No	1.0099	0.98	0.0316	0.381	No	No
160408_at	Gpsm1	G-protein signalling modulator 1 (AGS3-like, C. elegans)	1.0512	0.2249	0.0035	0.6787	No	1.0245	0.619	0.99	0.692	No	No
160409_at	Pitpna	phosphatidylinositol transfer protein, alpha	1.0386	0.502	0.3826	0.9249	No	1.0445	0.463	0.0019	0.474	No	No
160410_at	Vps16	vacuolar protein sorting 16 (yeast)	0.9754	0.7013	0.6233	0.4863	No	0.99	0.902	0.252	0.2	No	No
160411_at	---	---	1.0389	0.4072	0.4088	0.1483	No	1.0747	0.545	0.157	0.531	No	No
160412_at	Pdcd2	programmed cell death 2	1.13	0.0526	0.0317	0.037	No	1.0655	0.19	0.437	0.0347	No	No
160413_at	Nsg2	neuron specific gene family member 2	1.0943	0.1886	0.6542	0.5904	No	1.0714	0.169	0.101	0.063	No	No
160414_at	1810073N04 Rik	RIKEN cDNA 1810073N04 gene	1.0017	0.9718	0.009	0.4487	No	1.0192	0.417	0.0001	0.383	No	No
160415_at	Cldn1	claudin 1	0.8476	0.0069	0.1591	0.2782	Yes	0.8271	0.0002	0.0101	0.301	Yes	Yes
160416_at	Fkbp3	FK506 binding protein 3	1.0602	0.3888	0.5722	0.7747	No	1.0361	0.682	0.751	0.926	No	No
160417_at	Kif5b	kinesin family member 5B	0.9815	0.8144	0.0016	0.7592	No	1.0066	0.744	0.0026	0.295	No	No
160418_at	Clcc1	chloride channel CLIC-like 1	1.0887	0.2487	0.199	0.9302	No	0.9301	0.248	0.19	0.0243	No	No
160419_r_at	Phgdhl1	Phosphoglycerate dehydrogenase like 1	1.1472	0.361	0.3607	0.8346	No	1.0989	0.543	0.665	0.679	No	No
160420_r_at	Tuba3	tubulin, alpha 3	0.9902	0.9116	0.2389	0.5135	No	0.9941	0.984	0.707	0.948	No	No
160421_r_at	Ctrb1	chymotrypsinogen B1	1.0044	0.9312	0.0043	0.8706	No	0.9526	0.161	0.001	0.901	No	No
160422_at	Ruvbl2	RuvB-like protein 2	0.979	0.5683	0.0063	0.7612	No	0.9639	0.604	0.213	0.821	No	No
160423_at	Mrps2	mitochondrial ribosomal protein S2	0.8909	0.1672	0.6573	0.4669	No	0.946	0.261	0.347	0.91	No	No
160424_f_at	Fdps /// LOC544723	farnesyl diphosphate synthetase /// similar to farnesyl diphosphate synthetase	1.1405	0.0427	0.0007	0.1489	No	1.1238	0.0648	0.0006	0.148	No	No
160425_at	Mterfd1	MTERF domain containing 1	1.1637	0.0176	0.0031	0.0251	No	1.1731	0.0876	0.0336	0.13	No	No
160426_at	Rpo1-1	RNA polymerase 1-1	0.9422	0.147	0.118	0.2013	No	0.9455	0.436	0.237	0.912	No	No
160427_at	Krr1	KRR1, small subunit (SSU) processome component, homolog (yeast)	0.9996	0.9909	0.9971	0.8131	No	0.9924	0.728	0.173	0.371	No	No
160428_at	Suclg2	succinate-Coenzyme A ligase, GDP-forming, beta subunit	1.0745	0.2108	0.9355	0.3594	No	1.0915	0.0573	0.571	0.321	No	No
160429_at	Nxt1	NTF2-related export protein 1	1.0613	0.3973	0.1388	0.6733	No	1.0281	0.571	0.0158	0.683	No	No
160430_at	Ctnnb1	catenin (cadherin associated protein), beta 1	1.0546	0.1633	0.0007	0.3108	No	0.9976	0.935	0.0007	0.911	No	No
160431_at	Mrpl12	mitochondrial ribosomal protein L12	0.9215	0.2389	0.1224	0.0631	No	0.9543	0.195	0.002	0.0081	No	No
160432_at	U2af1l4	U2 small nuclear RNA auxiliary factor 1-like 4	0.9404	0.213	0.004	0.2413	No	1.0474	0.0651	0.694	0.0144	No	No
160433_at	3110002H16 Rik	RIKEN cDNA 3110002H16 gene	0.9599	0.2159	0.2695	0.2901	No	0.9367	0.228	0.435	0.634	No	No
160434_at	Tmem9b	TMEM9 domain family, member B	1.1671	0.1364	0.6989	0.9897	No	1.0973	0.0436	0.955	0.145	No	No
160435_at	Cd99l2	Cd99 antigen-like 2	1.0251	0.4123	0.1681	0.0271	No	1.0067	0.893	0.798	0.0435	No	No
160436_at	Sulf2	sulfatase 2	1.0219	0.8277	0.0149	0.957	No	1.0277	0.76	0.0002	0.895	No	No
160437_at	Txnrd2	thioredoxin reductase 2	0.9505	0.3092	0.0093	0.0609	No	0.9213	0.18	0.119	0.225	No	No
160438_at	Cox5a	cytochrome c oxidase, subunit Va	1.0632	0.2418	0.0125	0.4046	No	0.9298	0.628	0.2	0.833	No	No
160439_at	Polg	polymerase (DNA directed), gamma	0.9627	0.5604	0.0156	0.0682	No	0.8734	0.0017	0	0.002	No	No
160440_at	Smad4	MAD homolog 4 (Drosophila)	0.9683	0.3094	0.0002	0.4244	No	0.901	0.0062	0.0001	0.33	Yes	No
160441_at	Ceacam11	CEA-related cell adhesion molecule 11	1.116	0.0397	0.0545	0.0212	No	1.0852	0.276	0.0685	0.081	No	No
160442_at	Cct2	chaperonin subunit 2 (beta)	1.1546	0.0647	0.2367	0.2769	No	1.1318	0.309	0.858	0.781	No	No
160443_at	Csh2	chorionic somatomammotropin hormone 2	1.0103	0.8762	0.0467	0.8685	No	1.0185	0.784	0.0774	0.348	No	No
160444_at	Pigx	phosphatidylinositol glycan, class X	1.0627	0.1801	0.2845	0.1651	No	1.1057	0.087	0.0147	0.26	No	No
160445_at	Zbed3	zinc finger, BED domain containing 3	0.9294	0.0674	0.0008	0.596	No	0.995	0.954	0.0243	0.923	No	No
160446_at	U46068	cDNA sequence U46068	0.9207	0.2821	0.9868	0.4435	No	0.9082	0.108	0.0245	0.904	No	No
160447_at	Hiat1	hippocampus abundant gene transcript 1	1.0545	0.5069	0.0125	0.8288	No	1.1117	0.282	0.0063	0.909	No	No
160448_at	Pdcd2l	programmed cell death 2-like	1.0569	0.0703	0.006	0.1606	No	1.0415	0.34	0.0049	0.727	No	No
160449_at	Dr1	down-regulator of transcription 1	0.8661	0.0024	0.0748	0.1594	Yes	0.8857	0.025	0.0567	0.902	No	Yes
160450_at	---	---	0.9822	0.6456	0.8073	0.898	No	1.0529	0.421	0.765	0.795	No	No
160451_at	Etf1	eukaryotic translation termination factor 1	0.9828	0.699	0.0075	0.3938	No	0.9632	0.462	0.0149	0.915	No	No
160452_at	Dnase1	deoxyribonuclease I	1.089	0.1268	0.0988	0.5639	No	1.0395	0.711	0.0248	0.585	No	No
160453_at	Ryk	receptor-like tyrosine kinase	0.9544	0.3185	0.0021	0.1206	No	0.9674	0.276	0.0072	0.967	No	No
160454_at	Ube2z	ubiquitin-conjugating enzyme E2Z (putative)	0.9214	0.0199	0.002	0.0173	No	0.9152	0.0232	0.0153	0.0047	No	No
160455_s_at	Zwint	ZW10 interactor	0.9438	0.2852	0.0223	0.1013	No	1.0013	0.991	0.251	0.366	No	No
160456_at	Ppih /// LOC433064 /// LOC665989 /// LOC669441 /// LOC676493	peptidyl prolyl isomerase H /// similar to peptidyl prolyl isomerase H /// similar to peptidylprolyl isomerase H /// similar to peptidylprolyl isomerase H /// similar to peptidylprolyl isomerase H	1.0056	0.8366	0.4893	0.2346	No	0.8865	0.137	0.373	0.971	No	No
160457_at	Mrfap1	Morf4 family associated protein 1	1.1121	0.0295	0.1095	0.2377	No	1.0565	0.239	0.012	0.131	No	No
160458_at	Mcam	melanoma cell adhesion molecule	1.0686	0.0412	0.3015	0.0172	No	1.0769	0.0409	0.0122	0.0111	No	No
160459_r_at	Cct6a	chaperonin subunit 6a (zeta)	1.0115	0.8422	0.0004	0.0913	No	1.0716	0.397	0.0049	0.0595	No	No
160460_at	Hoxd8	homeo box D8	1.0364	0.1773	0.065	0.0087	No	1.0293	0.746	0.0056	0.0175	No	No
160461_f_at	Tubb6	tubulin, beta 6	1.1	0.2581	0.2285	0.1133	No	1.0619	0.345	0.0451	0.493	No	No
160462_f_at	Tubb3	tubulin, beta 3	1.1021	0.0666	0.132	0.1161	No	1.0983	0.0889	0.0146	0.845	No	No
160463_at	Myd116	myeloid differentiation primary response gene 116	0.9654	0.1225	0.0082	0.4172	No	0.9882	0.641	0.0989	0.529	No	No

160464_s_at	Ndrl	N-myc downstream regulated-like	0.8168	0.0188	0.6609	0.0935	No	0.8485	0.0168	0.0035	0.0619	No	No
160465_at	---	Transcribed locus	1.1187	0.1149	0.9706	0.1481	No	0.9117	0.151	0.252	0.0846	No	No
160466_at	Rae1	RAE1 RNA export 1 homolog (S. pombe)	1.0615	0.1199	0.0101	0.0123	No	1.052	0.349	0.963	0.121	No	No
160467_at	Oaz3	ornithine decarboxylase antizyme 3	0.94	0.2065	0.2936	0.9228	No	0.9748	0.0794	0.008	0.184	No	No
160468_at	Mtpn	myotrophin	1.0571	0.503	0.0193	0.3719	No	1.0849	0.383	0.134	0.29	No	No
160469_at	Thbs1 /// LOC640441	thrombospondin 1 /// similar to thrombospondin 1	0.9917	0.8963	0.0022	0.0821	No	0.9357	0.499	0.01	0.195	No	No
160470_at	Mrps5	mitochondrial ribosomal protein S5	0.9888	0.8696	0.781	0.7869	No	0.9813	0.78	0.0801	0.811	No	No
160471_at	Slbp	stem-loop binding protein	1.0423	0.4604	0.0462	0.7154	No	0.9696	0.65	0.812	0.851	No	No
160472_r_at	Tmeff1	transmembrane protein with EGF-like and two follistatin-like domains 1	1.2248	0.0509	0.0373	0.1832	No	1.2871	0.123	0.462	0.276	No	No
160473_at	Nme4	expressed in non-metastatic cells 4, protein	1.0063	0.7299	0	0.0498	No	1.0614	0.0011	0	0.549	Yes	No
160474_at	Rpp14	ribonuclease P 14 subunit (human)	1.0283	0.5803	0.0042	0.3037	No	1.034	0.49	0.0113	0.283	No	No
160475_at	Yipf4	Yip1 domain family, member 4	0.8847	0.0685	0.0121	0.1966	No	0.8835	0.0354	0.0053	0.365	No	No
160476_f_at	Rpl18 /// LOC672898	ribosomal protein L18 /// similar to 60S ribosomal protein L18	1.0289	0.6031	0.0608	0.0646	No	0.9699	0.207	0.04	0.0034	No	No
160477_at	Ndufa4	NADH dehydrogenase (ubiquinone) 1 alpha subcomplex, 4	1.1131	0.0371	0.0794	0.0624	No	1.0753	0.116	0.0356	0.107	No	No
160478_r_at	Rpl32	Ribosomal protein L32	0.9367	0.6503	0.3324	0.5635	No	0.9126	0.522	0.224	0.589	No	No
160479_at	Cat	catalase	1.0236	0.7204	0.001	0.6255	No	0.932	0.423	0.0015	0.693	No	No
160480_at	Ptprs	protein tyrosine phosphatase, receptor type, S	1.0255	0.4735	0.4636	0.6562	No	1.0172	0.603	0.184	0.76	No	No
160481_at	Pck1	phosphoenolpyruvate carboxykinase 1, cytosolic	1.228	0.0038	0.0388	0.6929	Yes	1.0802	0.119	0.0407	0.414	No	No
160482_at	Acaa1a	acetyl-Coenzyme A acyltransferase 1A	1.0275	0.5706	0.0801	0.07	No	1.09	0.256	0.0234	0.826	No	No
160483_at	Tcf4	transcription factor 4	1.1233	0.1086	0.0209	0.0444	No	1.099	0.247	0.0518	0.108	No	No
160484_at	Rtn4	reticulon 4	1.0179	0.7582	0.0106	0.1724	No	0.9895	0.887	0.133	0.67	No	No
160485_r_at	Ywhae	tyrosine 3-monooxygenase/tryptophan 5-monooxygenase activation protein, epsilon polypeptide	1.1146	0.0569	0.0106	0.6416	No	1.1158	0.299	0.486	0.696	No	No
160486_at	Ccdc28b	coiled coil domain containing 28B	0.9642	0.6392	0.2576	0.5586	No	0.9576	0.3	0.0954	0.108	No	No
160487_at	Myl4	myosin, light polypeptide 4	0.8149	0.0472	0.0401	0.0584	No	0.8301	0.0783	0.0581	0.0451	No	No
160488_at	Msh3	mutS homolog 3 (E. coli)	1.0606	0.3178	0.4803	0.1277	No	1.0591	0.0937	0.133	0.0852	No	No
160489_at	Tnfaip2	tumor necrosis factor, alpha-induced protein 2	0.9632	0.4007	0.0917	0.4468	No	0.9585	0.225	0.115	0.0215	No	No
160490_at	Def8	differentially expressed in FDCP 8	0.9544	0.4527	0.2877	0.6158	No	0.9586	0.467	0.947	0.544	No	No
160491_at	---	---	0.9941	0.8211	0.3204	0.8089	No	0.9975	0.875	0.0044	0.624	No	No
160492_at	Ddx18	DEAD (Asp-Glu-Ala-Asp) box polypeptide 18	1.0024	0.9329	0.0308	0.2923	No	1.0988	0.0403	0.349	0.842	No	No
160493_at	Cd63	Cd63 antigen	1.1157	0.1873	0.9587	0.8029	No	1.0775	0.243	0.445	0.564	No	No
160494_at	Endog	endonuclease G	1.012	0.7952	0.0347	0.7281	No	0.9526	0.303	0.923	0.39	No	No
160495_at	Ahr	aryl-hydrocarbon receptor	1.0714	0.4856	0.0044	0.3629	No	1.1475	0.28	0.0066	0.912	No	No
160496_s_at	LOC671904	similar to DNA replication licensing factor MCM3 (DNA polymerase alpha holoenzyme-associated protein P1) (P1-MCM3)	1.125	0.0713	0.7096	0.4172	No	1.1093	0.0813	0.0019	0.788	No	No
160497_at	1810059G22 Rik	RIKEN cDNA 1810059G22 gene	0.9723	0.5961	0.0889	0.4046	No	1.0469	0.29	0.216	0.987	No	No
160498_at	Ldb1	LIM domain binding 1	0.9649	0.327	0.5462	0.6026	No	1.0189	0.493	0.0216	0.159	No	No
160499_at	Hsp90b1	heat shock protein 90kDa beta (Grp94), member 1	1.0473	0.0025	0	0.0001	No	0.9911	0.49	0.0002	0.0617	No	No
160500_at	Atp1b1	ATPase, Na+/K+ transporting, beta 1 polypeptide	1.0345	0.01	0.0005	0.0023	No	1.0197	0.577	0.272	0.168	No	No
160501_at	Kif20a	kinesin family member 20A	0.9822	0.4707	0.0242	0.7069	No	0.8917	0.0318	0.0033	0.622	No	No
160502_at	Creg1	cellular repressor of E1A-stimulated genes 1	0.9606	0.1538	0.0424	0.0834	No	0.97	0.307	0.186	0.0131	No	No
160503_at	Fbl	fibrillarin	1.021	0.6849	0.0294	0.8303	No	1.0156	0.698	0.0111	0.804	No	No
160504_at	Ceacam12	CEA-related cell adhesion molecule 12	0.9682	0.0276	0.0531	0.0263	No	1.0226	0.857	0.213	0.262	No	No
160505_at	4922502D21 Rik	RIKEN cDNA 4922502D21 gene	0.9767	0.3764	0.0032	0.2972	No	1.0842	0.0299	0.0001	0.095	No	No
160506_at	Ropn1l	ropporin 1-like	1.0447	0.345	0.0391	0.2284	No	0.9811	0.884	0.449	0.0642	No	No
160507_at	0610013E23 Rik	RIKEN cDNA 0610013E23 gene	0.9621	0.7147	0.2152	0.3198	No	0.9913	0.894	0.933	0.646	No	No
160508_at	Psca	prostate stem cell antigen	1.0379	0.1374	0.0019	0.7669	No	1.0133	0.546	0.0101	0.275	No	No
160509_at	Chia	chitinase, acidic	1.0341	0.5652	0.9139	0.4364	No	1.0395	0.494	0.346	0.106	No	No
160510_f_at	Krt1-13	keratin complex 1, acidic, gene 13	0.9396	0.3571	0.0145	0.1446	No	0.9613	0.29	0	0.0065	No	No
160511_at	Cxcl12	chemokine (C-X-C motif) ligand 12	1.1602	0.0461	0.0005	0.6769	No	1.2043	0.0088	0.0004	0.438	Yes	Yes
160512_at	BC017643	cDNA sequence BC017643	0.9947	0.8757	0.0028	0.0561	No	0.9842	0.776	0.007	0.127	No	No
160513_at	2210016F16 Rik	RIKEN cDNA 2210016F16 gene	0.9736	0.1752	0.0131	0.008	No	0.9728	0.238	0.0022	0.0053	No	No
160514_at	Rp9h	retinitis pigmentosa 9 homolog (human)	0.9801	0.3918	0.0003	0.2269	No	0.9737	0.397	0.0005	0.01	No	No
160515_at	Parn	poly(A)-specific ribonuclease (deadenylation nuclease)	0.965	0.2458	0.1168	0.7186	No	0.9359	0.212	0.424	0.769	No	No
160516_at	Gaa	glucosidase, alpha, acid	0.9535	0.022	0.0001	0.0042	No	0.9904	0.568	0.0157	0.188	No	No
160517_at	Lmnb1	lamin B1	0.9022	0.1401	0.0792	0.0861	No	1.015	0.863	0.0581	0.889	No	No
160518_at	Rer1	RER1 retention in endoplasmic reticulum 1 homolog (S. cerevisiae)	1.0058	0.9111	0.0257	0.5028	No	0.9782	0.574	0.742	0.339	No	No
160519_at	Timp3	tissue inhibitor of metalloproteinase 3	1.1022	0.1938	0.0575	0.6214	No	1.0687	0.429	0.566	0.947	No	No
160520_at	Yap1	yes-associated protein 1	0.9688	0.5356	0.5382	0.2588	No	1.0043	0.971	0.239	0.121	No	No
160521_at	Ltv1	LTV1 homolog (S. cerevisiae)	1.0257	0.6066	0.0103	0.1529	No	0.9684	0.735	0.0171	0.189	No	No
160522_at	D0H4S114	DNA segment, human D4S114	1.0095	0.837	0.0693	0.2517	No	1.007	0.836	0.138	0.405	No	No
160523_at	Mpa2 /// Mpa2l /// LOC547126 /// LOC626578 /// LOC634650 /// LOC671219 /// LOC673195	macrophage activation 2 /// macrophage activation 2 like /// similar to macrophage activation 2 /// similar to macrophage activation 2 like /// similar to macrophage activation 2 /// similar to guanylate binding protein family, member 6 /// similar to guanylate binding protein family, member 6	1.035	0.4623	0.4958	0.086	No	1.0442	0.546	0.122	0.488	No	No
160524_at	Rab3a	RAB3A, member RAS oncogene family	0.9507	0.395	0.1112	0.9804	No	0.9991	0.974	0.0143	0.652	No	No
160525_f_at	Prlpk	prolactin-like protein K	0.9732	0.7428	0.6492	0.8786	No	0.9925	0.986	0.889	0.974	No	No
160526_s_at	Crem	cAMP responsive element modulator	1.029	0.6511	0.6447	0.5639	No	1.0349	0.687	0.0594	0.274	No	No
160527_at	Igfbp7 /// LOC621527	insulin-like growth factor binding protein 7 /// similar to O-acyltransferase (membrane bound) domain containing 2	0.9235	0.1812	0.0702	0.1131	No	0.9595	0.523	0.0037	0.542	No	No
160528_at	Prm2	protamine 2	0.888	0.0756	0.0741	0.9386	No	1.0082	0.997	0.129	0.34	No	No
160529_r_at	Vdac2	voltage-dependent anion channel 2	1.2514	0.0075	0.0004	0.1221	Yes	1.1127	0.154	0.0018	0.836	No	No
160530_at	Ghitm	growth hormone inducible transmembrane protein	1.0586	0.6334	0.0986	0.5569	No	1.0611	0.334	0.401	0.0454	No	No
160531_at	Emg1	EMG1 nucleolar protein homolog (S. cerevisiae)	0.9865	0.7965	0.2027	0.2878	No	0.9771	0.758	0.437	0.847	No	No
160532_at	Tpm1	tropomyosin 1, alpha	0.9989	0.9792	0.0045	0.3432	No	0.9784	0.73	0.0085	0.828	No	No
160533_r_at	Tnp1	transition protein 1	1.1025	0.1422	0.0005	0.045	No	1.0386	0.949	0.0001	0.003	No	No
160534_at	Psmc5	protease (prosome, macropain) 26S subunit, ATPase 5	1.0085	0.7763	0.078	0.9085	No	0.9142	0.0416	0.146	0.487	No	No
160535_at	Nfe2l1	nuclear factor, erythroid derived 2,-like 1	0.9383	0.1387	0.7825	0.6043	No	0.9722	0.506	0.134	0.812	No	No
160536_at	Hras1	Harvey rat sarcoma virus oncogene 1	1.0445	0.2087	0.0473	0.1005	No	1.0572	0.314	0.0411	0.126	No	No
160537_at	Sult1d1	sulfotransferase family 1D, member 1	1.035	0.6039	0.6678	0.1986	No	1.0697	0.4	0.0608	0.731	No	No
160538_at	Cdk4 /// LOC640611	cyclin-dependent kinase 4 /// similar to Cell division protein kinase 4 (Cyclin-dependent kinase 4) (PSK-J3) (CRK3)	1.0454	0.0688	0.0002	0.4586	No	1.0724	0.17	0.0146	0.499	No	No
160539_at	Sfrs1	splicing factor, arginine/serine-rich 1 (ASF/SF2)	1.1751	0.1777	0.122	0.5408	No	1.1922	0.102	0.657	0.862	No	No
160540_at	Bag1	Bcl2-associated athanogene 1	1.051	0.2895	0.0033	0.5811	No	0.9792	0.605	0.0009	0.02	No	No
160541_at	Eif3s5	Eukaryotic translation initiation factor 3, subunit 5 (epsilon)	1.0148	0.7845	0.197	0.888	No	1.0147	0.838	0.0476	0.178	No	No
160542_at	Cpsf2	cleavage and polyadenylation specific factor 2	0.9726	0.1463	0.0004	0.099	No	0.8668	0.097	0.0029	0.834	No	No
160543_at	Snx3	sorting nexin 3	1.0523	0.2137	0.4908	0.5697	No	1.0425	0.352	0.0335	0.718	No	No
160544_at	Fabp5	fatty acid binding protein 5, epidermal	1.0773	0.3119	0.7084	0.4109	No	1.0986	0.126	0.0515	0.313	No	No
160545_at	Ccnd3	cyclin D3	0.8966	0.1205	0.0229	0.2154	No	0.8927	0.0318	0.0025	0.33	No	No
160546_at	Aldoc	aldolase 3, C isoform	1.0111	0.696	0.0012	0.1146	No	0.994	0.776	0	0.0114	No	No

160547_s_at	Txnip	thioredoxin interacting protein	0.8944	0.0152	0.0026	0.0106	No	0.8579	0.0127	0.0204	0.0259	No	No
160548_at	Txnip	Thioredoxin interacting protein	1.1228	0.094	0.0613	0.0907	No	1.2936	0.0608	0.0273	0.255	No	No
160549_at	Tparl	TPA regulated locus	0.9857	0.3404	0.0163	0.1375	No	0.9749	0.257	0.0179	0.91	No	No
160550_i_at	Magoh	mago-nashi homolog, proliferation-associated (Drosophila)	1.0555	0.6217	0.0929	0.9114	No	1.0469	0.535	0.0065	0.993	No	No
160551_at	Vdac3	voltage-dependent anion channel 3	1.0338	0.5228	0.098	0.513	No	1.0193	0.77	0.101	0.496	No	No
160552_at	Ap1s1	adaptor protein complex AP-1, sigma 1	0.9954	0.9356	0.3322	0.1176	No	0.9956	0.897	0.763	0.0906	No	No
160553_at	Ly6d	lymphocyte antigen 6 complex, locus D	0.9323	0.1362	0.0024	0.4563	No	0.9579	0.442	0.0028	0.0951	No	No
160554_at	Eif3s6	eukaryotic translation initiation factor 3, subunit 6	1.1282	0.198	0.0148	0.2279	No	1.0191	0.661	0.0458	0.524	No	No
160555_r_at	Snrpb	small nuclear ribonucleoprotein B	0.9979	0.9595	0.0671	0.3314	No	1.0065	0.846	0.0863	0.639	No	No
160556_at	1810020G14 Rik	RIKEN cDNA 1810020G14 gene	1.0445	0.6644	0.4417	0.6884	No	1.0024	0.969	0.762	0.371	No	No
160557_at	Tssc4	tumor-suppressing subchromosomal transferable fragment 4	0.9664	0.2255	0.0011	0.0779	No	0.9442	0.0878	0.0003	0.0378	No	No
160558_at	Akt2	thymoma viral proto-oncogene 2	1.0305	0.0127	0	0.3185	No	0.9851	0.814	0.0026	0.449	No	No
160559_at	Psmb5	proteasome (prosome, macropain) subunit, beta type 5	1.0104	0.8307	0.0217	0.2015	No	0.9471	0.466	0.0034	0.371	No	No
160560_at	Hbb-b1	Hemoglobin, beta adult major chain	0.9792	0.5067	0.0045	0.4117	No	0.9511	0.533	0.304	0.967	No	No
160561_at	Mdk	midkine	1.0312	0.5332	0.0047	0.9447	No	1.015	0.595	0.0004	0.433	No	No
160562_at	Cct7	chaperonin subunit 7 (eta)	1.0197	0.1579	0.0464	0.0097	No	1.016	0.68	0.0318	0.0227	No	No
160563_at	Serf2	small EDRK-rich factor 2	0.9972	0.966	0.8783	0.362	No	0.953	0.357	0.0921	0.235	No	No
160564_at	Lcn2	lipocalin 2	0.7959	0.0042	0.0013	0.0093	No	0.7329	0.0056	0.003	0.0429	Yes	No
160565_at	Ckmt1	creatine kinase, mitochondrial 1, ubiquitous	0.8375	0.1289	0.4166	0.037	No	0.7743	0.316	0.0013	0.402	No	No
160566_at	Prss8	protease, serine, 8 (prostasin)	0.9223	0.2982	0.1418	0.9214	No	0.9998	0.998	0.769	0.578	No	No
160567_at	Gdf9	growth differentiation factor 9	0.9959	0.9168	0.1699	0.5967	No	1.0035	0.959	0.0515	0.627	No	No
160568_at	Eno1 /// LOC433182 /// LOC671462	enolase 1, alpha non-neuron /// similar to enolase 1, alpha non-neuron /// similar to T21B10.2b	1.0131	0.6952	0.0004	0.9448	No	1.0055	0.596	0.0003	0.103	No	No
160569_at	2310008M10 Rik	RIKEN cDNA 2310008M10 gene	1.1365	0.102	0.0761	0.2746	No	1.1185	0.114	0.007	0.177	No	No
160570_at	Actn2	actinin alpha 2	0.8079	0.0011	0.1053	0.0072	No	0.7373	0.0033	0.161	0.0094	No	No
160571_at	Idh1	isocitrate dehydrogenase 1 (NADP+), soluble	0.985	0.8108	0.2528	0.4304	No	1.0817	0.079	0.736	0.567	No	No
160572_at	Dhx15	DEAH (Asp-Glu-Ala-His) box polypeptide 15	1.0322	0.347	0.0099	0.0321	No	1.1177	0.0015	0.0001	0.0244	Yes	No
160573_at	Hccs	holocytochrome c synthetase	0.9954	0.9613	0.0202	0.7565	No	0.9894	0.762	0.0185	0.331	No	No
160574_at	Es2el	expressed sequence 2 embryonic lethal	0.9484	0.47	0.3549	0.3558	No	0.9332	0.302	0.52	0.559	No	No
160575_at	Cggbp1	CGG triplet repeat binding protein 1	0.9394	0.3963	0.0419	0.397	No	0.9291	0.149	0.0037	0.0892	No	No
160576_at	Ctdp1	CTD (carboxy-terminal domain, RNA polymerase II, polypeptide A) phosphatase, subunit 1	1.0493	0.5241	0.4083	0.1434	No	1.037	0.224	0.0075	0.948	No	No
160577_at	Atp9a	ATPase, class II, type 9A	1.0042	0.8798	0.0331	0.0572	No	0.9757	0.58	0.0489	0.0325	No	No
160578_at	Pdpk1	3-phosphoinositide dependent protein kinase-1	0.9542	0.3975	0.0029	0.8378	No	0.9547	0.418	0.0079	0.813	No	No
160579_at	Man1a	mannosidase 1, alpha	0.8355	0.0306	0.3115	0.2913	No	0.8732	0.0499	0.563	0.098	No	No
160580_at	Man1a	mannosidase 1, alpha	0.9231	0.0226	0.0008	0.3663	No	0.9299	0.0926	0.916	0.421	No	No
160581_at	Atg16l1	autophagy-related 16-like 1 (yeast)	0.9902	0.8942	0.3329	0.6408	No	1.077	0.252	0.0835	0.184	No	No
160582_at	Mybpc3	myosin binding protein C, cardiac	1.0431	0.2898	0.1309	0.1279	No	1.1141	0.288	0.575	0.203	No	No
160583_at	Xlkd1	extra cellular link domain-containing 1	0.9103	0.2138	0.1189	0.6215	No	0.9592	0.299	0.14	0.439	No	No
160584_at	Rsu1	Ras suppressor protein 1	1.0494	0.3406	0.9498	0.6819	No	0.9609	0.57	0.0005	0.335	No	No
160585_at	Bxdc1	brix domain containing 1	1.0059	0.91	0.3898	0.0778	No	1.0152	0.781	0.543	0.0284	No	No
160586_r_at	Tchh	trichohyalin	1.0024	0.954	0.0995	0.0383	No	0.6775	0.718	0.0191	0.231	No	No
160587_at	Tssc1	tumor suppressing subtransferable candidate 1	1.0324	0.4746	0.0496	0.064	No	1.0883	0.227	0.0951	0.677	No	No
160588_at	Zfp131	zinc finger protein 131	1.1012	0.0697	0.0578	0.3267	No	1.0274	0.472	0.043	0.423	No	No
160589_at	Ppig	peptidyl-prolyl isomerase G (cyclophilin G)	0.8973	0.2917	0.9442	0.9559	No	0.9136	0.441	0.918	0.96	No	No
160590_r_at	Comtd1	catechol-O-methyltransferase domain containing 1	1.1773	0.0054	0.143	0.0011	No	1.0601	0.0886	0.0006	0.0676	No	No
160591_at	St7	Suppression of tumorigenicity 7	0.9617	0.2187	0.0018	0.9377	No	0.93	0.25	0.0058	0.527	No	No
160592_at	Tmc6	transmembrane channel-like gene family 6	1.014	0.1635	0.0003	0.7115	No	0.957	0.384	0.0712	0.479	No	No
160593_at	Snx9	sorting nexin 9	0.991	0.357	0.0276	0.1935	No	0.9861	0.793	0.925	0.548	No	No
160594_at	Col17a1	procollagen, type XVII, alpha 1	1.0152	0.772	0.0063	0.0886	No	1.006	0.977	0.0877	0.736	No	No
160595_at	D15Ertd682e	DNA segment, Chr 15, ERATO Doi 682, expressed	1.025	0.5592	0.0333	0.4304	No	1.0273	0.524	0.972	0.98	No	No
160596_at	Med11	mediator of RNA polymerase II transcription, subunit 11 homolog (S. cerevisiae)	0.9434	0.3872	0.058	0.6094	No	1.1724	0.202	0.0258	0.102	No	No
160597_at	D1Ertd622e	DNA segment, Chr 1, ERATO Doi 622, expressed	0.9299	0.1868	0.002	0.771	No	1.0511	0.28	0.0726	0.646	No	No
160598_at	A230106M15Rik	RIKEN cDNA A230106M15 gene	0.9961	0.9255	0.8862	0.4996	No	1.0757	0.0384	0.0098	0.008	No	No
160599_r_at	Tsga2	testis specific gene A2	0.9361	0.2037	0.0252	0.1839	No	0.9048	0.0957	0.0338	0.0587	No	No
160600_at	1700009P03 Rik	RIKEN cDNA 1700009P03 gene	1.0534	0.3792	0.0732	0.107	No	1.056	0.0505	0.0136	0.276	No	No
160601_at	Lfng	lunatic fringe gene homolog (Drosophila)	0.9756	0.6923	0.0578	0.6559	No	1.0457	0.504	0.202	0.108	No	No
160602_at	Pde6d	phosphodiesterase 6D, cGMP-specific, rod, delta	1.0166	0.4812	0.0006	0.0099	No	1.021	0.246	0.0002	0.145	No	No
160603_at	Pparbp	peroxisome proliferator activated receptor binding protein	0.9126	0.1567	0.0034	0.1951	No	0.9238	0.181	0.0028	0.952	No	No
160604_at	Foxc1	forkhead box C1	1.1296	0.2533	0.0071	0.0466	No	1.1055	0.108	0.0074	0.0227	No	No
160605_s_at	Usp38	ubiquitin specific peptidase 38	1.0326	0.284	0.8386	0.2811	No	1.0919	0.498	0.176	0.515	No	No
160606_r_at	Adamts1	a disintegrin-like and metallopeptidase (reprolysin type) with thrombospondin type 1 motif, 1	0.9051	0.0147	0.0005	0.0032	No	0.8917	0.732	0.0169	0.66	No	No
160607_at	Pard3	par-3 (partitioning defective 3) homolog (C. elegans)	1.0856	0.0948	0.0028	0.1594	No	1.0676	0.193	0.0021	0.141	No	No
160608_at	Rab20	RAB20, member RAS oncogene family	0.9773	0.6393	0.497	0.8943	No	1.0403	0.539	0.718	0.942	No	No
160609_at	Atp7a	ATPase, Cu++ transporting, alpha polypeptide	0.9745	0.4851	0.0001	0.4546	No	0.9293	0.166	0.0004	0.891	No	No
160610_at	Pcdha4 /// Pcdha6 /// Pcdha7 /// Pcdha5 /// Pcdha11 /// Pcdha10 /// Pcdha1 /// Pcdha9 /// Pcdha3 /// Pcdha12 /// Pcdha2 /// Pcdha8 /// Pcdhac1 /// Pcdhac2	protocadherin alpha 4 /// protocadherin alpha 6 /// protocadherin alpha 7 /// protocadherin alpha 5 /// protocadherin alpha 11 /// protocadherin alpha 10 /// protocadherin alpha 1 /// protocadherin alpha 9 /// protocadherin alpha 3 /// protocadherin alpha 12 /// protocadherin alpha 2 /// protocadherin alpha 8 /// protocadherin alpha subfamily C, 1 /// protocadherin alpha subfamily C, 2	1.1531	0.2291	0.0011	0.411	No	1.0443	0.497	0.0009	0.609	No	No
160611_at	Cyp4v3	cytochrome P450, family 4, subfamily v, polypeptide 3	0.8892	0.0216	0.0055	0.2743	No	0.9168	0.0956	0.0135	0.0605	No	No
160612_at	Abcg1	ATP-binding cassette, sub-family G (WHITE), member 1	0.952	0.0856	0.0093	0.5826	No	0.9657	0.117	0.0614	0.0137	No	No
160613_at	Tinagl	tubulointerstitial nephritis antigen-like	0.8863	0.0019	0.0002	0.0994	Yes	0.87	0.0024	0.0007	0.165	Yes	Yes
160614_at	Pten	phosphatase and tensin homolog	0.9018	0.1632	0.0003	0.7424	No	1.0156	0.781	0.0002	0.941	No	No
160615_at	Pias3	protein inhibitor of activated STAT 3	1.0194	0.7761	0.0045	0.5981	No	0.9645	0.456	0.0004	0.598	No	No
160616_at	Whsc2	Wolf-Hirschhorn syndrome candidate 2 (human)	1.0101	0.808	0.1467	0.7025	No	1.024	0.684	0.107	0.968	No	No
160617_at	Klf13	Kruppel-like factor 13	1.144	0.0081	0	0.0009	No	1.0494	0.0792	0.0004	0.0068	No	No
160618_at	Lgals8	lectin, galactose binding, soluble 8	0.9231	0.2562	0.3984	0.6506	No	0.9307	0.39	0.842	0.637	No	No
160619_at	Bsdc1	BSD domain containing 1	0.8878	0.006	0.0018	0.5799	Yes	0.9184	0.0878	0.897	0.631	No	No
160620_at	Gt(ROSA)26 Sor	gene trap ROSA 26, Philippe Soriano	1.2375	0.1016	0.1639	0.7271	No	1.1842	0.241	0.0395	0.869	No	No
160621_at	Mrps22	mitochondrial ribosomal protein S22	0.9518	0.509	0.0101	0.4104	No	1.056	0.261	0.0046	0.738	No	No
160622_at	BC011209	cDNA sequence BC011209	0.9715	0.6562	0.2203	0.6264	No	1.0189	0.859	0.416	0.505	No	No
160623_at	Cdkl2	cyclin-dependent kinase-like 2 (CDC2-related kinase)	1.1829	0.0962	0.1119	0.4153	No	1.021	0.734	0.0912	0.981	No	No
160624_at	Kpna6	karyopherin (importin) alpha 6	0.9893	0.906	0.0691	0.5371	No	0.9358	0.404	0.013	0.761	No	No
160625_f_at	Mettl8	methyltransferase like 8	0.9872	0.7157	0.1335	0.2427	No	1.0225	0.466	0.0629	0.566	No	No
160626_at	Myef2	myelin basic protein expression factor 2, repressor	0.9668	0.2485	0.0002	0.6488	No	0.955	0.174	0.0011	0.949	No	No
160627_at	Ddx52	DEAD (Asp-Glu-Ala-Asp) box polypeptide 52	0.9636	0.4838	0.0172	0.5643	No	0.9396	0.27	0.0232	0.366	No	No
160628_at	Gcat	glycine C-acetyltransferase (2-amino-3-ketobutyrate-coenzyme A ligase)	1.0037	0.9214	0.0155	0.0846	No	1.1583	0.1	0.0359	0.509	No	No

160629_at	Rgs10	regulator of G-protein signalling 10	0.9495	0.2893	0.328	0.1981	No	0.9556	0.0964	0.0048	0.323	No	No
160630_at	Sms	spermine synthase	1.1143	0.1017	0.0087	0.1969	No	1.0907	0.0061	0.0001	0.0045	No	No
160631_s_at	Sgca	sarcoglycan, alpha (dystrophin-associated glycoprotein)	0.8122	0.005	0.0061	0.0428	Yes	0.8183	0.0017	0.0066	0.0068	No	No
160632_at	Prkcn	protein kinase C, nu	0.9041	0.2954	0.13	0.7105	No	0.8955	0.12	0.0444	0.335	No	No
160633_at	Thoc4	THO complex 4	1.1209	0.265	0.3964	0.2469	No	1.0675	0.374	0.0334	0.246	No	No
160634_at	Pacrg	Park2 co-regulated	1.0393	0.4862	0.1623	0.2502	No	1.0142	0.545	0.119	0.432	No	No
160635_at	Stx18	syntaxin 18	0.9686	0.4029	0.0837	0.0744	No	1.0247	0.488	0.29	0.201	No	No
160636_at	AI413582	expressed sequence AI413582	0.9143	0.0102	0.0002	0.7957	No	0.9994	0.591	0.0003	0.0196	No	No
160637_at	Mocs2	molybdenum cofactor synthesis 2	1.1386	0.0129	0.1042	0.7091	No	1.1147	0.0363	0.0121	0.232	No	No
160638_at	Cdkn2c	cyclin-dependent kinase inhibitor 2C (p18, inhibits CDK4)	0.987	0.5999	0.1916	0.3261	No	1.018	0.659	0.591	0.0755	No	No
160639_at	Hpn	hepsin	1.003	0.9188	0.2104	0.1028	No	1.0175	0.713	0.0518	0.103	No	No
160640_at	Galnt11	UDP-N-acetyl-alpha-D-galactosamine:polypeptide N-acetylgalactosaminyltransferase 11	0.8712	0.0645	0.1531	0.6129	No	1.003	0.921	0.77	0.541	No	No
160641_at	Pfkfb3	6-phosphofructo-2-kinase/fructose-2,6-biphosphatase 3	0.9557	0.4264	0.8167	0.4778	No	1.025	0.691	0.0802	0.848	No	No
160642_at	Tmem5	transmembrane protein 5	1.0267	0.4265	0.0018	0.155	No	1.0507	0.589	0.338	0.215	No	No
160643_at	Hdac2	histone deacetylase 2	1.0318	0.6911	0.9558	0.6021	No	0.9988	0.942	0.524	0.582	No	No
160644_at	Ggnbp1	gametogenetin binding protein 1	0.9808	0.0785	0.0024	0.0105	No	1.0036	0.961	0.284	0.491	No	No
160645_at	Birc3	baculoviral IAP repeat-containing 3	0.9931	0.9176	0.0363	0.8803	No	1.0599	0.33	0.0971	0.186	No	No
160646_at	Gsr /// LOC630729	glutathione reductase 1 /// similar to Glutathione reductase, mitochondrial precursor (GR) (GRase)	1.0176	0.7016	0.2255	0.8798	No	0.9951	0.689	0.0085	0.294	No	No
160647_at	Car6	carbonic anhydrase 6	1.0876	0.083	0.2112	0.7248	No	0.9701	0.467	0.0286	0.191	No	No
160648_at	Fignl1	fidgetin-like 1	1.0947	0.2367	0.0277	0.3675	No	1.0298	0.564	0.0058	0.233	No	No
160649_at	Gp1bb	glycoprotein Ib, beta polypeptide	0.921	0.1802	0.1579	0.0258	No	1.0384	0.149	0.0007	0.138	No	No
160650_at	Polr3d	polymerase (RNA) III (DNA directed) polypeptide D	0.9928	0.9045	0.0129	0.2977	No	0.9901	0.866	0.128	0.123	No	No
160651_at	Tacstd2	tumor-associated calcium signal transducer 2	1.0432	0.4045	0.1053	0.1446	No	1.2112	0.0008	0.0006	0.0004	No	No
160652_at	Ctps2	cytidine 5'-triphosphate synthase 2	0.9504	0.2181	0.0627	0.8195	No	0.9629	0.577	0.0382	0.895	No	No
160653_at	Tomm40	translocase of outer mitochondrial membrane 40 homolog (yeast)	1.0295	0.453	0.0301	0.4113	No	1.0286	0.451	0.0236	0.181	No	No
160654_at	2310073E15 Rik	RIKEN cDNA 2310073E15 gene	0.9711	0.6158	0.3712	0.4948	No	1.0234	0.183	0.0037	0.0184	No	No
160655_at	Cpd	carboxypeptidase D	0.9968	0.9554	0.0121	0.2307	No	1.0984	0.0599	0.971	0.0393	No	No
160656_i_at	Fem1b	feminization 1 homolog b (C. elegans)	1.0108	0.8866	0.0389	0.052	No	1.0398	0.546	0.832	0.586	No	No
160657_at	Ilf3	interleukin enhancer binding factor 3	1.1899	0.0254	0.0178	0.0865	No	1.0685	0.384	0.0149	0.888	No	No
160658_at	Cdk7	cyclin-dependent kinase 7 (homolog of Xenopus MO15 cdk-activating kinase)	0.9544	0.2499	0.463	0.6309	No	0.971	0.651	0.759	0.879	No	No
160659_at	Hddc2	HD domain containing 2	1.0329	0.5242	0.639	0.3467	No	0.9968	0.989	0.208	0.268	No	No
160660_r_at	Zfp503	zinc finger protein 503	1.0046	0.899	0.074	0.6863	No	1.1983	0.343	0.251	0.108	No	No
160661_at	5730472N09 Rik	RIKEN cDNA 5730472N09 gene	0.9405	0.2674	0.8793	0.2944	No	0.8579	0.0477	0.532	0.0167	No	No
160662_r_at	Gata6	GATA binding protein 6	0.9783	0.5666	0.5473	0.4913	No	1.8655	0.228	0.131	0.347	No	No
160663_at	Ddx41	DEAD (Asp-Glu-Ala-Asp) box polypeptide 41	1.0516	0.1581	0.0026	0.8054	No	1.0211	0.592	0.0018	0.52	No	No
160664_at	1200004M23 Rik	RIKEN cDNA 1200004M23 gene	0.9577	0.2459	0.1242	0.9794	No	0.9709	0.345	0.0381	0.579	No	No
160665_at	Mmp24	matrix metallopeptidase 24	0.9435	0.37	0.0145	0.4479	No	1.0503	0.483	0.439	0.591	No	No
160666_at	Actr6	ARP6 actin-related protein 6 homolog (yeast)	0.9865	0.846	0.0039	0.129	No	0.964	0.284	0.0005	0.112	No	No
160667_at	Evl	Ena-vasodilator stimulated phosphoprotein	1.096	0.2452	0.8864	0.4882	No	1.0383	0.708	0.895	0.875	No	No
160668_at	Ogfr	opioid growth factor receptor	0.9563	0.4844	0.0333	0.0653	No	0.9501	0.494	0.0297	0.0508	No	No
160669_at	Htatsf1	HIV TAT specific factor 1	1.0195	0.5279	0.0072	0.2396	No	1.0149	0.706	0.967	0.226	No	No
160670_at	Tnfrsf19	tumor necrosis factor receptor superfamily, member 19	1.0551	0.3015	0.0947	0.4549	No	0.9596	0.386	0.0291	0.459	No	No
160671_at	Cln8	ceroid-lipofuscinosis, neuronal 8	0.958	0.0565	0.0057	0.1112	No	1.0369	0.236	0.021	0.477	No	No
160672_at	AI462438	expressed sequence AI462438	0.9539	0.1898	0.0091	0.1239	No	1.1889	0.022	0.714	0.0103	No	No
160673_at	BC020002	cDNA sequence BC020002	1.0136	0.8238	0.5478	0.1172	No	1.138	0.0955	0.574	0.0145	No	No
160674_at	Trfr2	transferrin receptor 2	1.0039	0.9006	0.1191	0.0278	No	1.0893	0.168	0.251	0.0176	No	No
160675_at	Smarcd2	SWI/SNF related, matrix associated, actin dependent regulator of chromatin, subfamily d, member 2	1.107	0.0448	0.0079	0.1035	No	1.0434	0.198	0.0013	0.102	No	No
160676_at	1810012N18 Rik	RIKEN cDNA 1810012N18 gene	0.9369	0.4405	0.0199	0.7324	No	0.9368	0.616	0.0246	0.884	No	No
160677_at	Gatad2a	GATA zinc finger domain containing 2A	1.0403	0.3578	0.0711	0.3217	No	0.955	0.213	0.824	0.382	No	No
160678_at	Tspan12	tetraspanin 12	0.9706	0.4176	0.8043	0.4536	No	0.9511	0.519	0.579	0.79	No	No
160679_at	Kif1c	Kinesin family member 1C	1.0018	0.9843	0.0464	0.9258	No	1.0116	0.859	0.0152	0.009	No	No
160680_at	Cpeb1	cytoplasmic polyadenylation element binding protein 1	1.0273	0.4396	0.0277	0.2943	No	1.1313	0.024	0.0111	0.0422	No	No
160681_at	Papola	poly (A) polymerase alpha	0.8958	0.0364	0.0062	0.5335	No	0.9086	0.0743	0.404	0.748	No	No
160682_at	C330011F01 Rik /// 6430706D22 Rik	RIKEN cDNA C330011F01 gene /// RIKEN cDNA 6430706D22 gene	0.9885	0.3664	0.0275	0.0024	No	0.9963	0.965	0.128	0.169	No	No
160683_at	Slc25a36	solute carrier family 25, member 36	0.9688	0.4799	0.0034	0.2039	No	0.9991	0.92	0.0032	0.516	No	No
160684_at	8430410A17 Rik	RIKEN cDNA 8430410A17 gene	0.9163	0.1058	0.1525	0.2693	No	0.9402	0.0695	0.0039	0.923	No	No
160685_at	Klhl7	kelch-like 7 (Drosophila)	1.0687	0.1082	0.0206	0.081	No	0.9928	0.948	0.139	0.923	No	No
160686_at	Sltm	SAFB-like, transcription modulator	0.919	0.2299	0.0732	0.8873	No	1.0602	0.265	0.0634	0.0147	No	No
160687_r_at	Dbf4	DBF4 homolog (S. cerevisiae)	1.0238	0.557	0.7389	0.0688	No	1.1952	0.284	0.0442	0.14	No	No
160688_at	Golph3	golgi phosphoprotein 3	0.9522	0.5747	0.0225	0.2599	No	0.9682	0.715	0.0425	0.908	No	No
160689_r_at	Tradd	TNFRSF1A-associated via death domain	0.9618	0.3744	0.014	0.3694	No	0.9992	0.97	0.0159	0.662	No	No
160690_at	Csnk2a1	casein kinase II, alpha 1 polypeptide	0.9576	0.5727	0.7865	0.477	No	0.935	0.353	0.482	0.926	No	No
160691_at	4930511A21 Rik /// LOC665355	RIKEN cDNA 4930511A21 gene /// similar to phosphatase subunit gene g4-1	1.0593	0.2383	0.2527	0.0486	No	1.0099	0.962	0.0028	0.303	No	No
160692_at	Fcer1g	Fc receptor, IgE, high affinity I, gamma polypeptide	0.9543	0.078	0.0003	0.5075	No	0.9893	0.879	0.723	0.552	No	No
160693_at	Pip5k2c	phosphatidylinositol-4-phosphate 5-kinase, type II, gamma	0.9992	0.984	0.5029	0.6692	No	1.0205	0.725	0.93	0.505	No	No
160694_at	Ifngr2	interferon gamma receptor 2	0.9832	0.7531	0.9034	0.4155	No	1.0218	0.319	0.0019	0.0481	No	No
160695_i_at	Homer2	homer homolog 2 (Drosophila)	1.0334	0.348	0.9392	0.2531	No	1.1938	0.0209	0.0179	0.019	No	No
160696_at	Tia1	cytotoxic granule-associated RNA binding protein 1	0.9055	0.1069	0.0006	0.846	No	0.9484	0.189	0.0001	0.591	No	No
160697_at	C77080	expressed sequence C77080	0.9612	0.2353	0.0264	0.5868	No	1.0529	0.214	0.0046	0.156	No	No
160698_s_at	Prkcd	protein kinase C, delta	0.9609	0.0472	0.001	0.1916	No	0.9778	0.461	0.0125	0.0756	No	No
160699_at	Cdca5	cell division cycle associated 5	0.9841	0.5343	0.0031	0.1983	No	1.0675	0.303	0.341	0.588	No	No
160700_i_at	Sema6c	sema domain, transmembrane domain (TM), and cytoplasmic domain, (semaphorin) 6C	1.0351	0.3864	0.0006	0.0388	No	1.045	0.536	0.439	0.0787	No	No
160701_at	Axin1	axin 1	1.0215	0.3413	0.0013	0.6739	No	0.9832	0.811	0.304	0.748	No	No
160702_at	Rbm35a	RNA binding motif protein 35A	0.9291	0.1346	0.1226	0.864	No	0.9666	0.0352	0.0836	0.0058	No	No
160703_at	Poldip3	polymerase (DNA-directed), delta interacting protein 3	0.9772	0.6748	0.0544	0.1592	No	1.0061	0.884	0.0593	0.761	No	No
160704_at	1110067D22 Rik	RIKEN cDNA 1110067D22 gene	0.9213	0.1115	0.0311	0.6175	No	0.8549	0.174	0.224	0.885	No	No
160705_at	Cited1	Cbp/p300-interacting transactivator with Glu/Asp-rich carboxy-terminal domain 1	0.988	0.8161	0.6047	0.3674	No	1.0672	0.17	0.0915	0.0852	No	No
160706_at	3110004L20 Rik	RIKEN cDNA 3110004L20 gene	0.963	0.5981	0.0658	0.2693	No	0.9824	0.429	0.0146	0.678	No	No
160707_at	D10Wsu102 e	DNA segment, Chr 10, Wayne State University 102, expressed	0.9806	0.6914	0.1206	0.5731	No	1.0407	0.313	0.0077	0.0765	No	No
160708_at	Schip1	schwannomin interacting protein 1	0.9767	0.5847	0.2001	0.2169	No	0.9779	0.497	0.243	0.135	No	No
160709_at	1110001A16 Rik	RIKEN cDNA 1110001A16 gene	1.1035	0.0985	0.8503	0.4021	No	1.0007	0.975	0.976	0.979	No	No
160710_at	Usp33	ubiquitin specific peptidase 33	0.9581	0.7799	0.0131	0.9468	No	1.0051	0.835	0.0019	0.67	No	No
160711_at	Decr1	2,4-dienoyl CoA reductase 1, mitochondrial	0.9996	0.996	0.7107	0.3432	No	1.1116	0.125	0.0588	0.0672	No	No
160712_r_at	E130012A19 Rik	RIKEN cDNA E130012A19 gene	0.9766	0.3527	0.002	0.3245	No	0.9372	0.133	0.835	0.207	No	No
160713_at	Zfand2a	zinc finger, AN1-type domain 2A	1.083	0.0344	0.105	0.6284	No	1.0526	0.214	0.227	0.776	No	No

160714_at	Gab1	growth factor receptor bound protein 2-associated protein 1	0.9894	0.8496	0.4351	0.8599	No	1.0261	0.63	0.393	0.844	No	No
160715_at	AW146242	expressed sequence AW146242	1.2006	0.0617	0.0808	0.9671	No	1.1205	0.059	0.0944	0.85	No	No
160716_at	2410018C17 Rik	RIKEN cDNA 2410018C17 gene	0.9553	0.415	0.0043	0.1071	No	0.957	0.395	0.0081	0.561	No	No
160717_at	1110059E24 Rik	RIKEN cDNA 1110059E24 gene	0.9453	0.3888	0.937	0.4955	No	0.9483	0.36	0.874	0.89	No	No
160718_at	Capn7	calpain 7	1.0427	0.6165	0.0813	0.1506	No	1.0168	0.956	0.0111	0.137	No	No
160719_at	Ptrh2	peptidyl-tRNA hydrolase 2	0.9547	0.3723	0.1177	0.4378	No	0.9935	0.851	0.678	0.228	No	No
160720_at	Nola1	Nucleolar protein family A, member 1 (H/ACA small nucleolar RNPs)	0.915	0.1568	0.7507	0.39	No	0.9263	0.508	0.871	0.531	No	No
160721_at	Elf1	E74-like factor 1	1.026	0.7494	0.7264	0.6947	No	1.0451	0.425	0.17	0.709	No	No
160722_at	Rnmtl1	RNA methyltransferase like 1	0.8835	0.0568	0.0426	0.0714	No	0.9348	0.489	0.694	0.121	No	No
160723_at	1500001M20 Rik	RIKEN cDNA 1500001M20 gene	1.0934	0.2624	0.0098	0.5639	No	0.8335	0.0154	0.0001	0.672	No	No
160724_at	Trfp	Trf (TATA binding protein-related factor)-proximal protein homolog (Drosophila)	0.8967	0.0009	0.1291	0.0004	No	0.8692	0.0131	0.0956	0.0254	No	No
160725_at	4930432O21 Rik	RIKEN cDNA 4930432O21 gene	1.036	0.6192	0.0844	0.8089	No	0.9834	0.579	0.0209	0.186	No	No
160726_at	Qk	quaking	0.8793	0.2711	0.0107	0.6681	No	0.9325	0.0949	0.0006	0.0352	No	No
160727_at	2410002F23 Rik	RIKEN cDNA 2410002F23 gene	1.0663	0.1245	0.0748	0.0583	No	1.0351	0.435	0.328	0.613	No	No
160728_r_at	Otud4	OTU domain containing 4	0.9712	0.5763	0.5779	0.2345	No	0.4134	0.196	0.156	0.113	No	No
160729_f_at	Fabp9	fatty acid binding protein 9, testis	1.0085	0.955	0.2629	0.8976	No	0.9115	0.509	0.272	0.838	No	No
160730_at	Dnajc17	DnaJ (Hsp40) homolog, subfamily C, member 17	1.0135	0.7473	0.606	0.5048	No	0.9369	0.165	0.129	0.949	No	No
160731_at	Rab25	RAB25, member RAS oncogene family	1.0326	0.2427	0.0003	0.0848	No	0.9909	0.866	0.0184	0.176	No	No
160732_at	Npepl1	aminopeptidase-like 1	0.9164	0.043	0.0073	0.1581	No	0.9509	0.426	0.268	0.273	No	No
160733_at	Akr1c21	aldo-keto reductase family 1, member C21	1.0719	0.0175	0.8865	0.1194	No	1.0863	0.0122	0.428	0.0101	No	No
160734_at	Ap3s1	adaptor-related protein complex 3, sigma 1 subunit	1.0688	0.4238	0.2581	0.4339	No	0.9974	0.996	0.181	0.583	No	No
160735_at	D230025D16Rik	RIKEN cDNA D230025D16 gene	1.0126	0.6225	0.3595	0.462	No	0.9289	0.109	0.319	0.0401	No	No
160736_at	4932432N11 Rik	RIKEN cDNA 4932432N11 gene	1.0522	0.343	0.085	0.9373	No	0.9962	0.872	0.0026	0.17	No	No
160737_at	Lss	lanosterol synthase	1.0649	0.0815	0.9398	0.1024	No	1.0226	0.205	0.0075	0.117	No	No
160738_at	Polrmt	polymerase (RNA) mitochondrial (DNA directed)	0.967	0.4386	0.0932	0.7107	No	0.8827	0.104	0.58	0.853	No	No
160739_at	Wnk1	WNK lysine deficient protein kinase 1	0.9805	0.7564	0.0015	0.2035	No	1.0117	0.889	0.0021	0.169	No	No
160740_at	Gm561	gene model 561, (NCBI)	1.0785	0.0152	0.0003	0.0122	No	1.0651	0.0371	0.0006	0.0288	No	No
160741_at	Recql	RecQ protein-like	1.0853	0.1871	0.023	0.4751	No	1.0051	0.813	0.25	0.18	No	No
160742_at	Plod3	procollagen-lysine, 2-oxoglutarate 5-dioxygenase 3	1.0385	0.1697	0.0005	0.3728	No	1.0693	0.172	0.0396	0.815	No	No
160743_at	---	---	1.0127	0.7934	0.3028	0.8849	No	1.0369	0.381	0.0182	0.226	No	No
160744_r_at	Ctrl	chymotrypsin-like	0.9568	0.3382	0.0002	0.2055	No	0.9255	0.0998	0.0003	0.546	No	No
160745_at	Gcn5l2	GCN5 general control of amino acid synthesis-like 2 (yeast)	0.9257	0.0606	0.0034	0.0417	No	0.8951	0.0396	0.894	0.0089	No	No
160746_at	Rock1	Rho-associated coiled-coil forming kinase 1	1.0109	0.8427	0.0002	0.1265	No	0.931	0.803	0.0003	0.0672	No	No
160747_at	Rgs3 /// LOC436235 /// LOC670092	regulator of G-protein signaling 3 /// similar to Regulator of G-protein signaling 3 (RGS3) (C2PA) /// similar to Regulator of G-protein signaling 3 (RGS3) (C2PA)	1.0273	0.2073	0.0003	0.3429	No	1.0083	0.919	0.0184	0.715	No	No
160748_at	Asb6	ankyrin repeat and SOCS box-containing protein 6	0.9935	0.7529	0.0326	0.071	No	1.0982	0.0378	0.0005	0.325	No	No
160749_at	1500011H22 Rik	RIKEN cDNA 1500011H22 gene	1.0228	0.3417	0.3047	0.4617	No	1.0204	0.687	0.76	0.963	No	No
160750_at	Prpsap2	phosphoribosyl pyrophosphate synthetase-associated protein 2	1.0008	0.9888	0.7096	0.1956	No	0.965	0.395	0.0065	0.0193	No	No
160751_i_at	Crnkl1	Crn, crooked neck-like 1 (Drosophila)	0.9483	0.2885	0.0332	0.1564	No	0.9408	0.19	0.0064	0.0376	No	No
160752_at	Lrrc57	leucine rich repeat containing 57	1.0012	0.9557	0.4941	0.0816	No	1.029	0.676	0.169	0.363	No	No
160753_at	Myh3	myosin, heavy polypeptide 3, skeletal muscle, embryonic	0.5758	0.0002	0.0015	0.0064	No	0.5817	0.0006	0.0109	0.0171	Yes	No
160754_at	Pygm	muscle glycogen phosphorylase	0.5603	0.0024	0.2438	0.0152	Yes	0.6345	0.0003	0.0341	0.0024	No	No
160755_at	Kif2c /// LOC631653	kinesin family member 2C /// similar to Kinesin-like protein KIF2C (Mitotic centromere-associated kinesin) (MCAK)	1.0036	0.9547	0.0581	0.8006	No	1.0946	0.18	0.043	0.156	No	No
160756_at	Ralb	v-ral simian leukemia viral oncogene homolog B (ras related)	0.9667	0.0218	0	0.0073	No	1.0691	0.453	0.0176	0.139	No	No
160757_at	Rnf38	ring finger protein 38	0.9574	0.1545	0.0005	0.2598	No	0.894	0.0202	0.0028	0.705	No	No
160758_at	Seh1l	SEH1-like (S. cerevisiae	1.1028	0.272	0.0413	0.1548	No	1.0174	0.698	0.0789	0.129	No	No
160759_at	Rfc2	replication factor C (activator 1) 2	1.02	0.47	0.7842	0.5797	No	1.0564	0.0024	0.0932	0.0011	No	No
160760_at	Ptprk	protein tyrosine phosphatase, receptor type, K	0.9712	0.4147	0.3588	0.2508	No	0.9555	0.216	0.834	0.163	No	No
160761_at	Pde4dip	phosphodiesterase 4D interacting protein (myomegalin)	0.7616	0.0017	0.1026	0.0045	No	0.938	0.507	0.986	0.25	No	No
160762_at	Abr	active BCR-related gene	0.9072	0.1214	0.5288	0.3502	No	0.963	0.579	0.597	0.365	No	No
160763_at	Mapk13	mitogen activated protein kinase 13	1.0225	0.6434	0.0114	0.788	No	1.1618	0.0715	0.014	0.933	No	No
160764_at	Fbxw11	F-box and WD-40 domain protein 11	1.036	0.4588	0.2065	0.7985	No	1.0243	0.751	0.0389	0.197	No	No
160765_at	Ptges2	prostaglandin E synthase 2	1.0662	0.098	0.1607	0.1342	No	1.0735	0.0326	0.518	0.0635	No	No
160766_at	Cd2bp2	CD2 antigen (cytoplasmic tail) binding protein 2	0.9831	0.4342	0.2576	0.8995	No	1.0166	0.677	0.156	0.839	No	No
160767_at	Soat1	sterol O-acyltransferase 1	0.9541	0.2515	0.0985	0.4509	No	0.9285	0.124	0.325	0.374	No	No
160768_at	D030056L22 Rik	RIKEN cDNA D030056L22 gene	1.035	0.6687	0.0216	0.7615	No	1.0647	0.383	0.168	0.912	No	No
160769_at	Derl2	Der1-like domain family, member 2	0.9855	0.8553	0.9466	0.8341	No	0.9428	0.36	0.0033	0.394	No	No
160770_at	Mvd	mevalonate (diphospho) decarboxylase	1.038	0.3326	0.5018	0.0325	No	1.0386	0.539	0.426	0.181	No	No
160771_r_at	Ppfibp2	protein tyrosine phosphatase, receptor-type, F interacting protein, binding protein 2	0.942	0.1694	0.0109	0.2118	No	0.891	0.0056	0.113	0.0177	Yes	No
160772_i_at	D11Ertd730e	DNA segment, Chr 11, ERATO Doi 730, expressed	0.931	0.145	0.0046	0.8493	No	1.0074	0.921	0.0011	0.49	No	No
160773_at	D4Wsu132e	DNA segment, Chr 4, Wayne State University 132, expressed	1.0377	0.1867	0.6574	0.3007	No	1.1075	0.0646	0.368	0.338	No	No
160774_at	Entpd1	ectonucleoside triphosphate diphosphohydrolase 1	0.9428	0.0701	0.0258	0.3052	No	0.9632	0.386	0.0429	0.319	No	No
160775_at	Tulp4	tubby like protein 4	0.9913	0.919	0.0082	0.6674	No	1.0252	0.652	0.007	0.125	No	No
160776_at	Wdr42a	WD repeat domain 42A	0.9377	0.2038	0.4861	0.0327	No	0.909	0.0965	0.001	0.0016	No	No
160777_at	Ttc7b	tetratricopeptide repeat domain 7B	1.0844	0.036	0.0082	0.297	No	1.0919	0.261	0.0449	0.327	No	No
160778_at	LOC677213	similar to U2AF homology motif (UHM) kinase 1	1.1186	0.2685	0.0109	0.1345	No	1.0764	0.335	0.0239	0.143	No	No
160779_at	Saps3	SAPS domain family, member 3	0.9391	0.0313	0.0011	0.1243	No	0.9643	0.479	0.167	0.67	No	No
160780_at	Tcf3	transcription factor 3	0.9779	0.482	0.1327	0.0163	No	0.9001	0.0786	0.0688	0.124	No	No
160781_r_at	Unc93b1	unc-93 homolog B1 (C. elegans)	1.0258	0.6793	0.8775	0.6941	No	1.0036	0.874	0.0995	0.272	No	No
160782_at	BC021395	cDNA sequence BC021395	1.0006	0.9871	0.002	0.0241	No	0.955	0.18	0.0001	0.08	No	No
160783_at	D14Ertd436e	DNA segment, Chr 14, ERATO Doi 436, expressed	0.8748	0.0443	0.7314	0.5143	No	0.9493	0.193	0.559	0.0756	No	No
160784_r_at	2610039C10 Rik	RIKEN cDNA 2610039C10 gene	1.1183	0.2416	0.0228	0.2258	No	0.9889	0.991	0.0211	0.936	No	No
160785_at	Arhgap21	Rho GTPase activating protein 21	1.0236	0.8062	0.0028	0.968	No	1.0568	0.196	0.0045	0.921	No	No
160786_f_at	Actr1b	ARP1 actin-related protein 1 homolog B (yeast)	0.9451	0.2432	0.4894	0.344	No	1.016	0.759	0.133	0.324	No	No
160787_at	1500010J02 Rik	RIKEN cDNA 1500010J02 gene	0.916	0.0123	0.0005	0.1721	No	0.9037	0.0167	0.0006	0.943	No	No
160788_at	Pes1	pescadillo homolog 1, containing BRCT domain (zebrafish)	0.9581	0.1146	0.8847	0.4188	No	0.9397	0.0991	0.0587	0.0111	No	No
160789_at	Edem2	ER degradation enhancer, mannosidase alpha-like 2	0.9455	0.2168	0.0148	0.6589	No	1.0013	0.958	0.113	0.659	No	No
160790_s_at	Btrc	beta-transducin repeat containing protein	0.9906	0.8109	0.0104	0.9613	No	0.9996	0.898	0.0006	0.45	No	No
160791_at	3300001P08 Rik	RIKEN cDNA 3300001P08 gene	0.8905	0.2669	0.319	0.0946	No	0.936	0.55	0.456	0.109	No	No
160792_at	Snapap	SNAP-associated protein	0.9074	0.0891	0.0465	0.4168	No	0.9442	0.182	0.0237	0.945	No	No
160793_at	Pou6f1	POU domain, class 6, transcription factor 1	0.9826	0.7986	0.7338	0.7433	No	0.9851	0.836	0.261	0.299	No	No
160794_at	Smyd2	SET and MYND domain containing 2	0.9572	0.2137	0.0002	0.0815	No	0.9863	0.951	0.0001	0.0382	No	No
160795_at	Scamp1	secretory carrier membrane protein 1	1.0093	0.7575	0.2894	0.6875	No	1.152	0.0438	0.293	0.119	No	No

160796_at	Tiam2	T-cell lymphoma invasion and metastasis 2	1.0055	0.943	0.6933	0.8313	No	1.0171	0.753	0.534	0.236	No	No
160797_r_at	Cbx1	chromobox homolog 1 (Drosophila HP1 beta)	1.1398	0.2692	0.6953	0.3371	No	1.3078	0.117	0.697	0.127	No	No
160798_at	Mecr	mitochondrial trans-2-enoyl-CoA reductase	0.9634	0.4988	0.2529	0.0931	No	0.9901	0.682	0.0083	0.172	No	No
160799_at	LOC668581 /// LOC673100	similar to NADH-ubiquinone oxidoreductase MWFE subunit (Complex I-MWFE) (CI-MWFE) /// hypothetical protein LOC673100	0.8929	0.0841	0.0121	0.9984	No	1.0147	0.935	0.0002	0.287	No	No
160800_at	Zranb1	zinc finger, RAN-binding domain containing 1	1.0196	0.7786	0.6454	0.8361	No	1.0019	0.817	0.0001	0.561	No	No
160801_at	Pqlc1	PQ loop repeat containing 1	0.9939	0.7988	0.0037	0.1895	No	1.0614	0.0137	0.884	0.164	No	No
160802_at	Ppan	peter pan homolog (Drosophila)	0.9995	0.9936	0.2335	0.261	No	0.9916	0.954	0.0089	0.237	No	No
160803_at	Ubxd6	UBX domain containing 6	0.854	0.0317	0.0537	0.0168	No	0.8626	0.0009	0.0001	0.0014	No	No
160804_at	Eif2b5	eukaryotic translation initiation factor 2B, subunit 5 epsilon	1.0128	0.8063	0.9208	0.5585	No	0.9672	0.263	0.13	0.211	No	No
160805_s_at	Mpdu1	mannose-P-dolichol utilization defect 1	0.9609	0.2211	0.1515	0.0667	No	0.9886	0.673	0.0113	0.175	No	No
160806_at	Stk39	serine/threonine kinase 39, STE20/SPS1 homolog (yeast)	1.0713	0.4706	0.0506	0.9491	No	1.0171	0.692	0.0113	0.226	No	No
160807_at	Agpat3	1-acylglycerol-3-phosphate O-acyltransferase 3	0.9692	0.4777	0.3622	0.4644	No	1.0632	0.0031	0.0001	0.927	Yes	No
160808_at	Prkab1	protein kinase, AMP-activated, beta 1 non-catalytic subunit	0.9837	0.7682	0.4615	0.1674	No	0.9032	0.199	0.0064	0.0241	No	No
160809_at	Tollip	toll interacting protein	0.9968	0.9092	0.1854	0.1498	No	1.0032	0.992	0.601	0.478	No	No
160810_r_at	2510022D24 Rik	RIKEN cDNA 2510022D24 gene	0.9762	0.5337	0.0211	0.721	No	1.0179	0.622	0.0962	0.524	No	No
160811_at	AU022870	expressed sequence AU022870	1.034	0.528	0.3231	0.5802	No	0.9888	0.599	0.0003	0.7	No	No
160812_at	Gga2	golgi associated, gamma adaptin ear containing, ARF binding protein 2	1.0479	0.3272	0.0186	0.7989	No	1.0029	0.807	0.0054	0.0945	No	No
160813_r_at	Cox6b2	cytochrome c oxidase subunit VIb polypeptide 2	1.0309	0.4869	0.0494	0.0243	No	1.0247	0.473	0.234	0.008	No	No
160814_at	Thap4	THAP domain containing 4	1.0384	0.1842	0.0049	0.6788	No	1.078	0.0654	0.0334	0.089	No	No
160815_at	BC032204	cDNA sequence BC032204	0.8431	0.0011	0.0016	0.0105	Yes	0.8995	0.0809	0.395	0.692	No	No
160816_at	Fjx1	four jointed box 1 (Drosophila)	0.9566	0.4282	0.0064	0.2775	No	0.9098	0.144	0.0061	0.052	No	No
160817_at	Tmem40	transmembrane protein 40	0.967	0.4752	0.0008	0.1607	No	1.0144	0.883	0.0234	0.0734	No	No
160818_at	Lrrfip2	leucine rich repeat (in FLII) interacting protein 2	0.9377	0.1953	0.2417	0.8928	No	0.997	0.896	0.182	0.279	No	No
160819_at	Ndrg4	N-myc downstream regulated gene 4	0.9804	0.4768	0.0024	0.5728	No	0.993	0.246	0.0001	0.0016	No	No
160820_at	Igsf8	immunoglobulin superfamily, member 8	1.0265	0.6565	0.3707	0.5624	No	1.0451	0.0571	0.0276	0.456	No	No
160821_r_at	Ppp6c	protein phosphatase 6, catalytic subunit	0.9734	0.6349	0.1987	0.6891	No	1.0519	0.365	0.598	0.362	No	No
160822_at	Rap1gap	Rap1 GTPase-activating protein	0.9829	0.6212	0.0316	0.1878	No	0.9671	0.118	0.13	0.0661	No	No
160823_at	Nedd1	neural precursor cell expressed, developmentally down-regulated gene 1	0.9204	0.0641	0.0196	0.0388	No	0.8908	0.0205	0.0052	0.0753	No	No
160824_at	Rreb1	ras responsive element binding protein 1	0.9095	0.2004	0.0018	0.5987	No	0.9224	0.265	0.0018	0.529	No	No
160825_at	Chfr	checkpoint with forkhead and ring finger domains	0.9888	0.7826	0.0421	0.2186	No	0.9997	0.987	0.439	0.891	No	No
160826_at	Nsmaf	neutral sphingomyelinase (N-SMase) activation associated factor	1.063	0.2757	0.2885	0.8275	No	0.9444	0.33	0.291	0.96	No	No
160827_at	Rin2	Ras and Rab interactor 2	0.9208	0.1628	0.6541	0.9388	No	0.9562	0.286	0.0725	0.971	No	No
160828_at	Inhbb	inhibin beta-B	1.001	0.9785	0.1777	0.3979	No	0.9484	0.773	0.0727	0.491	No	No
160829_at	Phlda1	pleckstrin homology-like domain, family A, member 1	1.1331	0.0995	0.412	0.6193	No	1.1605	0.0457	0.494	0.134	No	No
160830_at	Cnih2	cornichon homolog 2 (Drosophila)	1.0584	0.2482	0.0313	0.2159	No	1.0291	0.149	0.0017	0.605	No	No
160831_at	Atpbd1b	ATP binding domain 1 family, member B	0.9491	0.0643	0.0001	0.1032	No	0.9147	0.0327	0.0001	0.0289	No	No
160832_at	Ldlr	low density lipoprotein receptor	1.1881	0.0075	0.05	0.5172	Yes	1.1967	0.0023	0.0917	0.129	Yes	Yes
160833_at	Mbd2	methyl-CpG binding domain protein 2	1.02	0.7239	0.0121	0.925	No	1.0773	0.329	0.121	0.517	No	No
160834_at	Sertad1	SERTA domain containing 1	1.0158	0.8157	0.2828	0.1374	No	1.0419	0.406	0.162	0.0997	No	No
160835_i_at	1110007C09 Rik	RIKEN cDNA 1110007C09 gene	1.0691	0.1826	0.0038	0.1299	No	0.9472	0.437	0.0008	0.92	No	No
160836_at	Sema4d	sema domain, immunoglobulin domain (Ig), transmembrane domain (TM) and short cytoplasmic domain, (semaphorin) 4D	1.0258	0.5105	0.0123	0.1759	No	1.0493	0.483	0.291	0.421	No	No
160837_at	Pdlim7	PDZ and LIM domain 7	1	0.9996	0.0113	0.7876	No	0.9846	0.567	0.002	0.315	No	No
160838_at	Orc2l	origin recognition complex, subunit 2-like (S. cerevisiae)	1.0205	0.7249	0.3772	0.3012	No	1.0088	0.915	0.0834	0.183	No	No
160839_at	Slc2a5	solute carrier family 2 (facilitated glucose transporter), member 5	1.0148	0.578	0.3809	0.9367	No	0.9974	0.951	0.0119	0.741	No	No
160840_at	Arhgef3	Rho guanine nucleotide exchange factor (GEF) 3	0.9628	0.3443	0.0048	0.727	No	1.0006	0.746	0.0001	0.0652	No	No
160841_at	Dbp	D site albumin promoter binding protein	1.0241	0.6457	0.9382	0.1475	No	1.1092	0.07	0.186	0.491	No	No
160842_at	Kin	antigenic determinant of rec-A protein	0.9389	0.1669	0.0025	0.0232	No	1.0573	0.102	0.0001	0.0791	No	No
160843_at	Spin	spindlin	1.0279	0.7164	0.4281	0.2757	No	0.997	0.928	0.531	0.27	No	No
160844_at	Pts	6-pyruvoyl-tetrahydropterin synthase	1.0462	0.5728	0.6699	0.6369	No	0.8423	0.0712	0.0004	0.0377	No	No
160845_at	Ccdc99	coiled-coil domain containing 99	0.9426	0.2072	0.3949	0.4679	No	0.9354	0.121	0.475	0.145	No	No
160846_at	Nek6 /// LOC674247	NIMA (never in mitosis gene a)-related expressed kinase 6 /// similar to NIMA (never in mitosis gene a)-related expressed kinase 6	1.0415	0.5368	0.5213	0.5404	No	0.9227	0.196	0.0008	0.0793	No	No
160847_at	Trnt1	tRNA nucleotidyl transferase, CCA-adding, 1	0.9586	0.5536	0.0133	0.4382	No	0.952	0.305	0.0103	0.372	No	No
160848_at	Zhx1	zinc fingers and homeoboxes protein 1	0.9062	0.019	0.0003	0.4606	No	0.9807	0.665	0.0005	0.253	No	No
160849_at	Dlgh1	discs, large homolog 1 (Drosophila)	1.0308	0.5571	0.2579	0.6871	No	0.9908	0.787	0.186	0.612	No	No
160850_at	Fpgs	folylpolyglutamyl synthetase	1.0555	0.3757	0.0295	0.0849	No	1.0037	0.827	0.0017	0.486	No	No
160851_r_at	Cox8b	cytochrome c oxidase, subunit VIIIb	0.9696	0.5195	0.6162	0.8038	No	0.9997	0.96	0.0565	0.432	No	No
160852_at	Krt1-15	keratin complex 1, acidic, gene 15	1.0103	0.859	0.0024	0.193	No	0.8814	0.233	0.0041	0.046	No	No
160853_at	Ptdss2	phosphatidylserine synthase 2	0.9585	0.137	0.3898	0.3614	No	0.9813	0.655	0.021	0.278	No	No
160854_at	Map3k7	mitogen activated protein kinase kinase kinase 7	0.8982	0.1927	0.0145	0.7711	No	0.8174	0.0655	0.0013	0.263	No	No
160855_at	Tmem12	transmembrane protein 12	1.0033	0.9275	0.6052	0.9671	No	1.0981	0.414	0.392	0.532	No	No
160856_at	Ubl4	ubiquitin-like 4	1.0921	0.1246	0.8302	0.3327	No	1.0459	0.195	0.201	0.714	No	No
160857_at	Efnb2	ephrin B2	0.9024	0.1317	0.0076	0.0639	No	0.9096	0.0836	0.064	0.0442	No	No
160858_at	2310061C15 Rik	RIKEN cDNA 2310061C15 gene	1.0343	0.5032	0.1253	0.4594	No	0.9802	0.579	0.0042	0.257	No	No
160859_s_at	Nfib	nuclear factor I/B	0.9336	0.4869	0.0651	0.8557	No	0.9425	0.459	0.0099	0.513	No	No
160860_at	Gdf10	growth differentiation factor 10	1.0095	0.8317	0.039	0.0354	No	0.9917	0.741	0.076	0.0855	No	No
160861_s_at	Pde1a	phosphodiesterase 1A, calmodulin-dependent	0.9621	0.4723	0.352	0.8257	No	0.9681	0.242	0.532	0.335	No	No
160862_at	Ptp4a3	protein tyrosine phosphatase 4a3	1.0074	0.8412	0.0002	0.1175	No	1.0543	0.0219	0	0.0263	No	No
160863_at	Tulp2	tubby-like protein 2	1.024	0.6382	0.0046	0.1442	No	1.002	0.446	0	0.0263	No	No
160864_at	Rtkn	rhotekin	0.8949	0.0317	0.0018	0.0086	No	0.9272	0.0675	0.0055	0.0631	No	No
160865_at	Vldlr	very low density lipoprotein receptor	1.0227	0.3998	0.2333	0.0387	No	0.9489	0.116	0.0189	0.578	No	No
160866_at	Dffa	DNA fragmentation factor, alpha subunit	0.9469	0.2539	0.0631	0.3743	No	0.9972	0.96	0.011	0.098	No	No
160867_at	Pdgfrb	platelet derived growth factor receptor, beta polypeptide	0.8567	0.0747	0.4106	0.316	No	0.9405	0.581	0.261	0.537	No	No
160868_at	Rab3b	RAB3B, member RAS oncogene family	1.0106	0.8861	0.499	0.9082	No	0.9135	0.0502	0.173	0.153	No	No
160869_at	Sirt3	sirtuin 3 (silent mating type information regulation 2, homolog) 3 (S. cerevisiae)	0.9125	0.3207	0.3437	0.2726	No	0.9527	0.602	0.138	0.762	No	No
160870_at	Adcy3	adenylate cyclase 3	0.9432	0.0823	0.0074	0.1035	No	1.0331	0.402	0.0118	0.455	No	No
160871_at	Kif1b	kinesin family member 1B	1.0233	0.4333	0.7811	0.108	No	1.0943	0.0397	0.661	0.598	No	No
160872_f_at	2310008H09 Rik	RIKEN cDNA 2310008H09 gene	1.0214	0.7438	0.0154	0.9345	No	0.9603	0.262	0.0024	0.0614	No	No
160873_at	Ghrl	ghrelin	1.0869	0.0795	0.0508	0.0979	No	0.9837	0.598	0.0159	0.169	No	No
160874_r_at	Ruvbl1	RuvB-like protein 1	1.0085	0.9246	0.8916	0.5162	No	0.9967	0.987	0.18	0.0502	No	No
160875_at	Psmb1	Proteasome (prosome, macropain) subunit, beta type 1	1.0578	0.2506	0.0017	0.6026	No	1.0276	0.34	0.0055	0.387	No	No
160876_at	Bcap29	B-cell receptor-associated protein 29	0.9765	0.6817	0.1167	0.85	No	0.991	0.86	0.159	0.786	No	No
160877_at	Ogn	osteoglycin	0.9756	0.6713	0.7522	0.666	No	0.9748	0.531	0.267	0.941	No	No
160878_at	Bop1	block of proliferation 1	1.0159	0.789	0.1175	0.129	No	1.0665	0.034	0	0.516	No	No
160879_at	Terf1	telomeric repeat binding factor 1	1.0524	0.0755	0.0057	0.0088	No	0.9663	0.497	0.0004	0.0089	No	No
160880_at	Mapk8ip3	mitogen-activated protein kinase 8 interacting protein 3	1.0078	0.8673	0.0325	0.8617	No	1.0254	0.425	0.156	0.63	No	No
160881_at	Map3k12	mitogen activated protein kinase kinase kinase 12	1.0782	0.2506	0.1719	0.5098	No	1.006	0.892	0.0012	0.917	No	No
160882_at	Ogg1	8-oxoguanine DNA-glycosylase 1	1.0443	0.2398	0.262	0.2828	No	1.0649	0.444	0.165	0.075	No	No
160883_at	LOC673662	similar to CG3835-PA, isoform A	1.0745	0.2032	0.0118	0.1138	No	1.0399	0.44	0.0313	0.28	No	No
160884_at	Clic3	chloride intracellular channel 3	0.9749	0.5022	0.0027	0.5947	No	0.9317	0.0665	0.0006	0.006	No	No
160885_at	Nucks1	nuclear casein kinase and cyclin-dependent kinase substrate 1	1.0816	0.3947	0.1041	0.2052	No	1.0101	0.81	0.0855	0.251	No	No

160886_i_at	Fabp6	Fatty acid binding protein 6, ileal (gastrotropin)	0.9975	0.9694	0.153	0.6693	No	1.049	0.858	0.244	0.828	No	No
160887_at	Hes1	hairy and enhancer of split 1 (Drosophila)	1.019	0.6882	0.0759	0.4652	No	1.0166	0.492	0.0103	0.662	No	No
160888_at	Reps1	RalBP1 associated Eps domain containing protein	0.9807	0.7183	0.5298	0.4886	No	0.9249	0.0926	0.0627	0.337	No	No
160889_at	8430437G11 Rik	RIKEN cDNA 8430437G11 gene	0.9488	0.0701	0.0303	0.8347	No	0.9491	0.285	0.0448	0.897	No	No
160890_at	Lbr	lamin B receptor	1.024	0.6459	0.2378	0.1719	No	1.0375	0.484	0.402	0.0335	No	No
160891_at	Sftpb	surfactant associated protein B	0.959	0.555	0.8294	0.4131	No	0.9162	0.163	0.0355	0.24	No	No
160892_at	Dlgh3	discs, large homolog 3 (Drosophila)	0.9737	0.5867	0.255	0.366	No	1.0352	0.513	0.0407	0.103	No	No
160893_at	Gngt2	guanine nucleotide binding protein (G protein), gamma transducing activity polypeptide 2	0.8822	0.1549	0.103	0.0905	No	1.0188	0.785	0.0884	0.45	No	No
160894_at	Cebpd	CCAAT/enhancer binding protein (C/EBP), delta	0.6301	0.0019	0.0824	0.0303	Yes	0.5658	0.0004	0.0056	0.0258	Yes	Yes
160895_at	Fancc	Fanconi anemia, complementation group C	1.0036	0.6519	0.0487	0.1077	No	1.019	0.252	0.0072	0.588	No	No
160896_at	Rcn1	reticulocalbin 1	1.1078	0.1844	0.4997	0.9851	No	1.0432	0.507	0.409	0.818	No	No
160897_at	Aytl2	acyltransferase like 2	0.9773	0.6166	0.0054	0.2807	No	1.0732	0.407	0.0011	0.0287	No	No
160898_at	Abt1	activator of basal transcription	0.9889	0.8204	0.2905	0.3628	No	0.9813	0.697	0.0403	0.559	No	No
160899_at	Pcp4	Purkinje cell protein 4	1.0017	0.9492	0.0009	0.2218	No	1.0472	0.415	0.0291	0.392	No	No
160900_at	Gkap1 /// LOC673324	G kinase anchoring protein 1 /// similar to G kinase anchoring protein 1	0.9176	0.1809	0.0273	0.8224	No	0.9962	0.882	0.0396	0.166	No	No
160901_at	Fos	FBJ osteosarcoma oncogene	0.9675	0.5525	0.0012	0.0017	No	0.9985	0.118	0.0004	0.001	No	No
160902_at	F8a	factor 8-associated gene A	0.8692	0.0435	0.0383	0.1882	No	0.9697	0.343	0.101	0.657	No	No
160903_at	Apbb2	amyloid beta (A4) precursor protein-binding, family B, member 2	1.0007	0.9909	0.097	0.4324	No	1.0236	0.189	0.0003	0.0496	No	No
160904_at	B230317C12 Rik	RIKEN cDNA B230317C12 gene	1.0436	0.1463	0.0044	0.058	No	1.034	0.214	0.0044	0.0275	No	No
160905_s_at	A030009H04 Rik	RIKEN cDNA A030009H04 gene	1.0402	0.2129	0.0144	0.3196	No	1.0851	0.175	0.981	0.952	No	No
160906_i_at	Wiz /// Mtm1	Widely-interspaced zinc finger motifs /// X-linked myotubular myopathy gene 1	0.8744	0.2553	0.0054	0.8899	No	0.8673	0.463	0.002	0.182	No	No
160907_at	Slc38a5	solute carrier family 38, member 5	1.014	0.5289	0.0655	0.1157	No	1.1092	0.0067	0.0128	0.0018	No	No
160908_r_at	2510042P03 Rik	RIKEN cDNA 2510042P03 gene	1.0745	0.3233	0.2572	0.6708	No	1.042	0.357	0.434	0.395	No	No
160909_at	Sprr1a	small proline-rich protein 1A	1.0168	0.6463	0.2024	0.2586	No	1.1912	0.0589	0.0037	0.19	No	No
160910_at	Ghrh	growth hormone releasing hormone	1.113	0.0295	0.0039	0.033	No	1.2869	0.0008	0.0432	0.0021	No	No
160911_at	Sos1	Son of sevenless homolog 1 (Drosophila)	1.0765	0.1432	0.0023	0.3718	No	1.0835	0.241	0.0037	0.593	No	No
160912_i_at	Pias2	protein inhibitor of activated STAT 2	1.0389	0.0698	0.0003	0.003	No	1.1427	0.161	0.0052	0.179	No	No
160913_at	Nrap	nebulin-related anchoring protein	0.7407	0.0001	0.3379	0.0004	No	0.7487	0.0051	0.989	0.0219	Yes	No
160914_at	Wfdc12	WAP four-disulfide core domain 12	0.952	0.0948	0.292	0.1729	No	0.9993	0.983	0.0852	0.938	No	No
160915_at	B4galt3	UDP-Gal:betaGlcNAc beta 1,4-galactosyltransferase, polypeptide 3	1.066	0.0933	0.0479	0.0897	No	1.0952	0.025	0.01	0.726	No	No
160916_at	2310005P05 Rik	RIKEN cDNA 2310005P05 gene	0.9588	0.5702	0.3568	0.5721	No	0.888	0.321	0.842	0.558	No	No
160917_r_at	Mageh1	melanoma antigen, family H, 1	1.2184	0.0845	0.004	0.7475	No	1.4041	0.212	0.0148	0.42	No	No
160918_at	S100g	S100 calcium binding protein G	0.9183	0.322	0.7615	0.9813	No	0.9927	0.944	0.773	0.588	No	No
160919_r_at	Fgfr3	fibroblast growth factor receptor 3	1.1478	0.1367	0.0304	0.1401	No	1.2904	0.179	0.457	0.374	No	No
160920_at	Bcl2l2	Bcl2-like 2	0.9706	0.2703	0.6293	0.1766	No	0.9549	0.106	0.282	0.923	No	No
160921_at	Acss1	acyl-CoA synthetase short-chain family member 1	1.0337	0.7159	0.0238	0.6347	No	1.0709	0.331	0.0386	0.306	No	No
160922_at	Ogfod2	2-oxoglutarate and iron-dependent oxygenase domain containing 2	0.909	0.2629	0.0756	0.9152	No	0.9569	0.391	0.0185	0.366	No	No
160923_at	Abp1	amiloride binding protein 1 (amine oxidase, copper-containing)	0.9794	0.7075	0.2189	0.1396	No	0.9694	0.542	0.243	0.189	No	No
160924_at	D14Ertd231 e	DNA segment, Chr 14, ERATO Doi 231, expressed	0.9051	0.1548	0.7441	0.944	No	0.9155	0.0194	0.263	0.0651	No	No
160925_at	Nras	neuroblastoma ras oncogene	1.0319	0.6561	0.0353	0.1185	No	1.0296	0.661	0.0125	0.754	No	No
160926_r_at	---	Transcribed locus	1.0199	0.7486	0.0005	0.2475	No	1.1111	0.322	0.0001	0.576	No	No
160927_at	Ace	angiotensin I converting enzyme (peptidyl-dipeptidase A) 1	0.9454	0.2447	0.8484	0.4308	No	0.8888	0.148	0.0957	0.265	No	No
160928_at	Tfpt	TCF3 (E2A) fusion partner	1.0156	0.5318	0.5224	0.3108	No	0.9944	0.911	0.705	0.467	No	No
160929_at	Mettl3	methyltransferase-like 3	0.9456	0.291	0.075	0.7747	No	0.9044	0.0071	0.0938	0.0158	Yes	No
160930_at	Sacm1l	SAC1 (suppressor of actin mutations 1, homolog)-like (S. cerevisiae)	0.9621	0.2767	0.1222	0.1216	No	0.9781	0.513	0.0184	0.169	No	No
160931_at	Dnm1l	dynamin 1-like	0.9225	0.2181	0.0878	0.7302	No	0.9107	0.152	0.367	0.435	No	No
160932_at	Nck1	non-catalytic region of tyrosine kinase adaptor protein 1	0.972	0.4854	0.0039	0.9568	No	1.0658	0.336	0.0283	0.312	No	No
160933_at	Iigp	interferon gamma induced GTPase	1.0103	0.7147	0.0807	0.6558	No	1.028	0.51	0.733	0.0937	No	No
160934_s_at	Gag	Gag	0.9781	0.6369	0.0009	0.2621	No	1.1063	0.0397	0.0005	0.461	No	No
160935_at	Ddhd1	DDHD domain containing 1	1.0065	0.9013	0.2067	0.2069	No	1.0033	0.852	0.248	0.0228	No	No
160936_at	Tram1	translocating chain-associating membrane protein 1	1.0106	0.7691	0.7701	0.5517	No	0.9712	0.676	0.692	0.622	No	No
160937_at	Crym	crystallin, mu	1.2269	0.002	0.3592	0.4225	Yes	1.211	0.0252	0.627	0.265	No	Yes
160938_at	Mast2	microtubule associated serine/threonine kinase 2	0.9663	0.3826	0.4833	0.9317	No	0.934	0.145	0.0357	0.0497	No	No
160939_at	Twist2	twist homolog 2 (Drosophila)	0.9995	0.9782	0.0984	0.0609	No	0.9624	0.148	0.602	0.0276	No	No
160940_at	Sfrs14	splicing factor, arginine/serine-rich 14	1.0218	0.1926	0.8145	0.853	No	0.9877	0.17	0.0004	0.0232	No	No
160941_at	Pde8a	phosphodiesterase 8A	0.9298	0.2129	0.0268	0.8595	No	0.9674	0.646	0.0269	0.63	No	No
160942_at	Cbx6	chromobox homolog 6	0.9972	0.9552	0.0278	0.2509	No	1.0101	0.907	0.0253	0.0092	No	No
160943_at	Stag3	stromal antigen 3	0.9356	0.112	0.0266	0.5225	No	1.0335	0.615	0.343	0.851	No	No
160944_at	Pkp3	plakophilin 3	0.9078	0.1485	0.2109	0.2999	No	0.9311	0.202	0.0117	0.349	No	No
160945_at	C80008	expressed sequence C80008	1.0253	0.4829	0.0429	0.2184	No	1.0506	0.786	0.0611	0.468	No	No
160946_at	Usp12	ubiquitin specific peptidase 12	0.9902	0.6384	0.7455	0.0587	No	0.9841	0.889	0.6	0.546	No	No
160947_at	Centd3	centaurin, delta 3	1.0645	0.09	0.2327	0.6907	No	1.0224	0.572	0.0874	0.988	No	No
160948_at	Ppp3cc	protein phosphatase 3, catalytic subunit, gamma isoform	0.9956	0.8895	0.0612	0.3486	No	1.0418	0.644	0.71	0.311	No	No
160949_at	Parg	poly (ADP-ribose) glycohydrolase	1.0464	0.764	0.0439	0.5015	No	1.0141	0.853	0.0465	0.874	No	No
160950_at	Gapdhs	glyceraldehyde-3-phosphate dehydrogenase, spermatogenic	0.9757	0.8202	0.0663	0.9344	No	0.9114	0.208	0.111	0.573	No	No
160951_at	Serpina12	serine (or cysteine) peptidase inhibitor, clade A (alpha-1 antiproteinase, antitrypsin), member 12	0.8732	0.0203	0.0052	0.3358	No	0.9298	0.153	0.0223	0.254	No	No
160952_r_at	Lrrc6	leucine rich repeat containing 6 (testis)	1.1312	0.0884	0.3719	0.2776	No	1.3606	0.0897	0.0834	0.192	No	No
160953_at	Cacna1h	calcium channel, voltage-dependent, T type, alpha 1H subunit	1.0195	0.7859	0.48	0.3037	No	0.9428	0.13	0.0074	0.794	No	No
160954_at	Syn2	synapsin II	1.0011	0.9858	0.039	0.1129	No	1.0229	0.643	0.0629	0.206	No	No
160955_at	2010309E21 Rik	RIKEN cDNA 2010309E21 gene	0.9522	0.4467	0.0373	0.0403	No	0.9274	0.322	0.0131	0.0341	No	No
160956_r_at	Guf1	GUF1 GTPase homolog (S. cerevisiae)	0.9613	0.6548	0.0151	0.9417	No	1.0052	0.835	0.0123	0.706	No	No
160957_at	Dicer1	Dicer1, Dcr-1 homolog (Drosophila)	0.9086	0.2306	0.0059	0.1364	No	1.0033	0.689	0.0001	0.0025	No	No
160958_at	9430016H08 Rik	RIKEN cDNA 9430016H08 gene	0.9618	0.6552	0.2872	0.5678	No	0.951	0.279	0.004	0.709	No	No
160959_at	Rcbtb1	regulator of chromosome condensation (RCC1) and BTB (POZ) domain containing protein 1	1.0481	0.4341	0.0776	0.5745	No	1.0737	0.341	0.139	0.281	No	No
160960_at	Atp6v0a2	ATPase, H+ transporting, lysosomal V0 subunit A2	1.0226	0.6202	0.0587	0.6693	No	1.1007	0.102	0.923	0.0718	No	No
160961_at	Sipa1l2	signal-induced proliferation-associated 1 like 2	1.0471	0.37	0.8937	0.606	No	1.0227	0.532	0.0059	0.916	No	No
160962_at	Bag2	Bcl2-associated athanogene 2	1.0832	0.0279	0.0138	0.1053	No	1.0267	0.624	0.213	0.869	No	No
160963_at	9630050M13 Rik	RIKEN cDNA 9630050M13 gene	1.1026	0.0628	0.0422	0.0159	No	1.1008	0.0343	0.4	0.0106	No	No
160964_at	D16Bwg149 4e	DNA segment, Chr 16, Brigham & Women's Genetics 1494 expressed	1.0642	0.0503	0.0001	0.0287	No	0.9954	0.857	0.0016	0.162	No	No
160965_at	Rasa4	RAS p21 protein activator 4	0.921	0.0022	0.0117	0.2152	Yes	0.9113	0.0067	0.0506	0.152	Yes	Yes
160966_at	Zfp187	zinc finger protein 187	0.8589	0.0531	0.0625	0.0704	No	0.8597	0.0501	0.0177	0.122	No	No
160967_at	Sfxn2	sideroflexin 2	0.9782	0.664	0.3689	0.7547	No	1.0174	0.68	0.0223	0.482	No	No
160968_at	Aard	alanine and arginine rich domain containing protein	1.0274	0.4938	0.0245	0.4105	No	0.9538	0.394	0.0747	0.819	No	No
160969_at	Foxk1	forkhead box K1	0.9171	0.2415	0.0139	0.3317	No	0.9322	0.145	0.0487	0.642	No	No
160970_at	Odf2	outer dense fiber of sperm tails 2	0.9668	0.4053	0.0008	0.3352	No	1.0237	0.235	0.0001	0.0839	No	No
160971_at	Kazald1	Kazal-type serine peptidase inhibitor domain 1	0.9657	0.4353	0.1122	0.0393	No	1.0133	0.7	0.0737	0.136	No	No
160972_at	Serpinb9e	serine (or cysteine) peptidase inhibitor, clade B, member 9e	0.9266	0.4273	0.3784	0.6481	No	1.0584	0.292	0.0155	0.98	No	No

160973_at	C330027C09Rik	RIKEN cDNA C330027C09 gene	0.9455	0.4164	0.0552	0.5329	No	0.9503	0.568	0.0733	0.517	No	No
160974_at	AA408556	expressed sequence AA408556	1.0071	0.9178	0.0829	0.9084	No	0.9936	0.697	0.64	0.194	No	No
160975_at	---	Transcribed locus	1.0838	0.4432	0.0013	0.4015	No	1.0301	0.472	0.0012	0.279	No	No
160976_at	Diap1 /// LOC671545	diaphanous homolog 1 (Drosophila) /// similar to Protein diaphanous homolog 1 (Diaphanous-related formin-1) (DRF1) (mDIA1) (p140mDIA)	0.9243	0.259	0.0129	0.135	No	0.8793	0.0489	0.0065	0.347	No	No
160977_at	Arhgef5	Rho guanine nucleotide exchange factor (GEF) 5	0.9933	0.9053	0.9953	0.815	No	0.9695	0.241	0.984	0.283	No	No
160978_at	Osta	organic solute transporter alpha	0.9673	0.645	0.4716	0.6812	No	0.894	0.491	0.076	0.832	No	No
160979_at	Ctbp2 /// Zranb1	C-terminal binding protein 2 /// zinc finger, RAN-binding domain containing 1	1.0484	0.7821	0.0104	0.7198	No	1.1004	0.4	0.0014	0.191	No	No
160980_at	2700097O09Rik	RIKEN cDNA 2700097O09 gene	1.0802	0.2546	0.8574	0.8961	No	0.9081	0.136	0.0084	0.395	No	No
160981_at	Atxn10	ataxin 10	1.0018	0.9735	0.0299	0.8258	No	1.0743	0.404	0.733	0.962	No	No
160982_at	4921526G09Rik	RIKEN cDNA 4921526G09 gene	0.9994	0.9957	0.16	0.6832	No	0.9445	0.323	0.153	0.443	No	No
160983_at	1810044D09Rik	RIKEN cDNA 1810044D09 gene	0.9527	0.3367	0.5034	0.4838	No	0.9564	0.355	0.381	0.448	No	No
160984_r_at	Odf2l	outer dense fiber of sperm tails 2-like	1.0879	0.1077	0.0878	0.53	No	0.8014	0.0002	0.071	0.0005	No	No
160985_at	4930579C15Rik	RIKEN cDNA 4930579C15 gene	0.9148	0.2717	0.0196	0.7789	No	0.8335	0.172	0.115	0.669	No	No
160986_r_at	Ace2	angiotensin I converting enzyme (peptidyl-dipeptidase A) 2	0.976	0.0275	0.0003	0.001	No	0.8683	0.0522	0.124	0.492	No	No
160987_r_at	1110001A07Rik	RIKEN cDNA 1110001A07 gene	1.1494	0.0205	0.7577	0.0135	No	1.3043	0.0643	0.0103	0.0513	No	No
160988_r_at	Fubp1	far upstream element (FUSE) binding protein 1	1.1068	0.0622	0.0045	0.2483	No	1.0785	0.195	0.0317	0.0701	No	No
160989_r_at	Npr3	natriuretic peptide receptor 3	0.9634	0.5122	0.0003	0.0605	No	0.913	0.807	0.0014	0.0935	No	No
160990_r_at	Rwdd4a	RWD domain containing 4A	0.9409	0.4988	0.0663	0.1761	No	0.8798	0.303	0.201	0.744	No	No
160991_at	Nkiras1	NFKB inhibitor interacting Ras-like protein 1	0.9425	0.0836	0.4299	0.0476	No	0.967	0.367	0.0458	0.373	No	No
160992_at	Ccdc93	coiled-coil domain containing 93	0.9603	0.4115	0.4817	0.2391	No	1.096	0.273	0.211	0.48	No	No
160993_at	Iqgap2	IQ motif containing GTPase activating protein 2	0.9931	0.7259	0.0018	0.074	No	1.0074	0.935	0.144	0.763	No	No
160994_at	S100pbp	S100P binding protein	0.9507	0.3971	0.02	0.1996	No	0.9942	0.993	0.0009	0.546	No	No
160995_at	Repin1	replication initiator 1	1.0419	0.2952	0.0006	0.0345	No	1.043	0.517	0.159	0.212	No	No
160996_at	Foxd1	forkhead box D1	0.9971	0.9654	0.0057	0.3149	No	1.0547	0.0316	0.0002	0.0025	No	No
160997_at	E130303B06Rik	RIKEN cDNA E130303B06 gene	0.9756	0.485	0.0036	0.262	No	0.9885	0.669	0.0045	0.0549	No	No
160998_at	Tmem38b	transmembrane protein 38B	0.952	0.2248	0.5064	0.2789	No	0.9766	0.404	0.0111	0.135	No	No
160999_r_at	2810422O20Rik	RIKEN cDNA 2810422O20 gene	1.0901	0.0224	0.837	0.0772	No	1.0758	0.159	0.141	0.103	No	No
161000_i_at	Nusap1	nucleolar and spindle associated protein 1	1.0071	0.9036	0.0353	0.7398	No	0.9556	0.511	0.108	0.62	No	No
161001_at	AA407270	expressed sequence AA407270	0.9676	0.0953	0.0021	0.0173	No	0.9774	0.61	0.0007	0.319	No	No
161002_at	Rbm15	RNA binding motif protein 15	0.9847	0.8561	0.0302	0.5048	No	1.0895	0.12	0.0315	0.188	No	No
161003_at	Kif3b	kinesin family member 3B	0.9419	0.249	0.4667	0.5667	No	1.1705	0.0955	0.142	0.041	No	No
161004_at	1700097N02Rik	RIKEN cDNA 1700097N02 gene	1.0816	0.2448	0.0606	0.1515	No	1.0027	0.989	0.311	0.0725	No	No
161005_at	5730420B22Rik	RIKEN cDNA 5730420B22 gene	0.9786	0.1735	0	0.0169	No	0.9341	0.111	0.0003	0.369	No	No
161006_at	Slco3a1	solute carrier organic anion transporter family, member 3a1	0.9814	0.5284	0.0314	0.9147	No	1.0102	0.854	0.0437	0.435	No	No
161007_at	---	---	1.0582	0.3055	0.745	0.1069	No	1.079	0.134	0.372	0.192	No	No
161008_at	AI428936	expressed sequence AI428936	0.9436	0.315	0.0532	0.0825	No	1.0344	0.756	0.0765	0.438	No	No
161009_at	Morc4	microrchidia 4	0.9564	0.2803	0.2391	0.3722	No	1.0043	0.97	0.352	0.0047	No	No
161010_r_at	Cdkn1b	cyclin-dependent kinase inhibitor 1B (P27)	1.0051	0.9098	0.0842	0.8279	No	0.8823	0.366	0.345	0.748	No	No
161011_at	Btbd4	BTB (POZ) domain containing 4	1.0381	0.5932	0.0033	0.1898	No	1.0591	0.461	0.0097	0.304	No	No
161012_at	Cd79b	CD79B antigen	0.8449	0.0003	0.002	0.004	No	0.9326	0.412	0.0549	0.453	No	No
161013_f_at	Spsb1	splA/ryanodine receptor domain and SOCS box containing 1	0.7807	0.0249	0.1023	0.0317	No	0.7551	0.0403	0.031	0.0389	No	No
161014_at	---	---	1.0496	0.3706	0.0027	0.8807	No	1.0069	0.802	0.0245	0.137	No	No
161015_at	4933402J24Rik	RIKEN cDNA 4933402J24 gene	1.0295	0.3869	0.4951	0.5548	No	1.0254	0.931	0.0202	0.011	No	No
161016_at	Galnt4	UDP-N-acetyl-alpha-D-galactosamine:polypeptide N-acetylgalactosaminyltransferase 4	1.0081	0.7929	0.2473	0.0453	No	0.9638	0.174	0.322	0.0473	No	No
161017_at	Kntc2 /// 2700099C18Rik	kinetochore associated 2 /// RIKEN cDNA 2700099C18 gene	0.9235	0.1589	0.3306	0.9821	No	0.9721	0.642	0.926	0.349	No	No
161018_at	2310010J17Rik	RIKEN cDNA 2310010J17 gene	0.9261	0.3145	0.0496	0.0788	No	0.9221	0.0813	0.133	0.0158	No	No
161019_at	2210018M03Rik	RIKEN cDNA 2210018M03 gene	0.9379	0.3377	0.3297	0.3468	No	0.9904	0.906	0.218	0.545	No	No
161020_r_at	Mnab	membrane associated DNA binding protein	1.0536	0.3018	0.0042	0.0859	No	1.0255	0.88	0.0024	0.627	No	No
161021_at	Pak3	p21 (CDKN1A)-activated kinase 3	1.0034	0.9309	0.0173	0.4679	No	1.0881	0.286	0.0903	0.613	No	No
161022_at	---	---	0.9692	0.2032	0.0528	0.0436	No	1.0249	0.576	0.0658	0.324	No	No
161023_at	Il15ra	interleukin 15 receptor, alpha chain	0.9393	0.3535	0.1708	0.4413	No	0.96	0.597	0.638	0.215	No	No
161024_at	Plekha7	pleckstrin homology domain containing, family A member 7	1.0266	0.67	0.0254	0.7322	No	0.9672	0.593	0.0414	0.49	No	No
161025_f_at	Sox18	SRY-box containing gene 18	1.0221	0.3682	0.0699	0.0383	No	1.0363	0.271	0.472	0.819	No	No
161026_s_at	Sytl4	synaptotagmin-like 4	0.9906	0.9051	0.1566	0.4163	No	1.0156	0.697	0.0103	0.535	No	No
161027_f_at	Plekha1	pleckstrin homology domain containing, family A (phosphoinositide binding specific) member 1	0.9787	0.6515	0.017	0.7405	No	1.0366	0.186	0.0003	0.0218	No	No
161028_at	Bmp6	bone morphogenetic protein 6	0.9792	0.6403	0.0012	0.3915	No	1.0397	0.329	0.0007	0.956	No	No
161029_at	6330416G13Rik	RIKEN cDNA 6330416G13 gene	1.0189	0.4564	0.0034	0.6003	No	0.9733	0.398	0.0056	0.0705	No	No
161030_at	Scx	scleraxis	0.9523	0.328	0.0046	0.0401	No	0.9333	0.188	0.0007	0.0288	No	No
161031_at	Zbtb25	zinc finger and BTB domain containing 25	1.0117	0.8543	0.411	0.6882	No	0.9965	0.971	0.127	0.573	No	No
161032_i_at	Nmu	neuromedin U	1.0173	0.7778	0.0192	0.1956	No	1.064	0.0513	0.0169	0.0021	No	No
161033_at	Papolb	poly (A) polymerase beta (testis specific)	0.9926	0.7164	0.5603	0.12	No	0.9748	0.532	0.442	0.125	No	No
161034_at	Pla2g10	phospholipase A2, group X	1.045	0.2832	0.4484	0.2651	No	1.0125	0.45	0.0016	0.244	No	No
161035_at	Kif9	kinesin family member 9	0.9615	0.562	0.1999	0.4473	No	0.9964	0.999	0.966	0.458	No	No
161036_at	Slc6a15	solute carrier family 6 (neurotransmitter transporter), member 15	1.1156	0.0401	0.0007	0.0424	No	1.0415	0.403	0.037	0.492	No	No
161037_at	Il15	interleukin 15	0.8756	0.0009	0.0982	0.572	Yes	0.7515	0.157	0.111	0.162	No	No
161038_at	Prps2	phosphoribosyl pyrophosphate synthetase 2	1.0342	0.5256	0.7283	0.9088	No	1.0619	0.435	0.95	0.244	No	No
161039_at	Adam22	a disintegrin and metallopeptidase domain 22	0.9239	0.0086	0.0001	0.7052	Yes	0.9291	0.195	0.0006	0.786	No	No
161040_at	Fzd1	frizzled homolog 1 (Drosophila)	1.077	0.0969	0.4138	0.182	No	1.0668	0.234	0.13	0.256	No	No
161041_at	Ddx25	DEAD (Asp-Glu-Ala-Asp) box polypeptide 25	0.9747	0.6227	0.7047	0.1716	No	1.0253	0.617	0.394	0.235	No	No
161042_at	Cbr3	carbonyl reductase 3	1.0023	0.939	0.0016	0.7606	No	1.0858	0.093	0.445	0.459	No	No
161043_r_at	Txnrd3	thioredoxin reductase 3	0.9845	0.8194	0.5232	0.1917	No	0.9489	0.395	0.0976	0.0952	No	No
161044_at	D030051N19Rik	RIKEN cDNA D030051N19 gene	0.8468	0.0195	0.0873	0.2615	No	0.8569	0.0212	0.389	0.422	No	No
161045_at	Lrrn1	leucine rich repeat protein 1, neuronal	1.0542	0.3498	0.0007	0.0703	No	0.9706	0.192	0.001	0.868	No	No
161046_at	Crlf1	cytokine receptor-like factor 1	0.9567	0.3102	0.0003	0.0165	No	0.9698	0.437	0.0001	0.015	No	No
161047_at	Atp5d	ATP synthase, H+ transporting, mitochondrial F1 complex, delta subunit	0.9701	0.7446	0.1414	0.4411	No	1.0116	0.906	0.242	0.256	No	No
161048_at	Prg2	proteoglycan 2, bone marrow	1.0511	0.5272	0.0201	0.624	No	1.002	0.961	0.343	0.0243	No	No
161049_at	Foxg1	forkhead box G1	1.0357	0.2755	0.0282	0.3823	No	1.129	0.0442	0.988	0.253	No	No
161050_at	E430014L09Rik	RIKEN cDNA E430014L09 gene	0.9106	0.0759	0.0021	0.1436	No	0.978	0.366	0.0043	0.46	No	No
161051_at	Hes5	hairy and enhancer of split 5 (Drosophila)	0.944	0.4046	0.0027	0.7836	No	1.011	0.993	0.0036	0.354	No	No

161052_r_at	Krt1-5	keratin complex 1, acidic, gene 5	1.0058	0.9064	0.079	0.5452	No	1.0209	0.416	0.0003	0.299	No	No
161053_at	LOC665276	similar to Y-linked testis-specific protein	0.8474	0.2456	0.0571	0.272	No	0.893	0.367	0.0196	0.162	No	No
161054_at	Spock1	sparc/osteonectin, cwcv and kazal-like domains proteoglycan 1	1.0115	0.8431	0.8809	0.9913	No	1.0553	0.262	0.319	0.0293	No	No
161055_r_at	6330409N04 Rik	RIKEN cDNA 6330409N04 gene	1.0576	0.0874	0.0239	0.0859	No	1.1629	0.002	0.0142	0.0306	Yes	No
161056_at	Gm1103	gene model 1103, (NCBI)	0.9835	0.6038	0.008	0.2673	No	0.9626	0.162	0.0012	0.0308	No	No
161057_at	2900086B20 Rik	RIKEN cDNA 2900086B20 gene	1.0521	0.3915	0.0469	0.1229	No	1.1282	0.0696	0.117	0.0846	No	No
161058_f_at	R74862	expressed sequence R74862	1.0735	0.0687	0.0916	0.5251	No	1.1355	0.321	0.738	0.843	No	No
161059_at	Slc6a1	solute carrier family 6 (neurotransmitter transporter, GABA), member 1	1.1931	0.0057	0.0014	0.0218	Yes	1.0892	0.0448	0.0067	0.0074	No	No
161060_i_at	Ddx51	DEAD (Asp-Glu-Ala-Asp) box polypeptide 51	0.9354	0.0756	0.063	0.0031	No	1.0005	0.811	0.0002	0.055	No	No
161061_r_at	Sh3rf1	SH3 domain containing ring finger 1	1.0569	0.3682	0.0136	0.649	No	1.0953	0.228	0.0064	0.161	No	No
161062_r_at	Rufy3	RUN and FYVE domain containing 3	1.1403	0.0203	0.0001	0.5066	No	1.1708	0.0231	0	0.537	No	No
161063_r_at	R74740	expressed sequence R74740	1.0261	0.4652	0.0352	0.7179	No	0.9787	0.164	0.0001	0.154	No	No
161064_f_at	Phf7	PHD finger protein 7	0.8362	0.0037	0.0016	0.0059	No	0.9196	0.359	0.849	0.17	No	No
161065_at	Dpep3	dipeptidase 3	0.9729	0.6742	0.253	0.4018	No	1.1026	0.281	0.0978	0.139	No	No
161066_at	Alg5	asparagine-linked glycosylation 5 homolog (yeast, dolichyl-phosphate beta-glucosyltransferase)	1.1074	0.4881	0.0684	0.4378	No	1.1292	0.353	0.0076	0.347	No	No
161067_at	Trib3	tribbles homolog 3 (Drosophila)	0.9468	0.2275	0.5309	0.072	No	0.9999	0.884	0.0156	0.132	No	No
161068_at	Fmr1nb	fragile X mental retardation 1 neighbor	1.0747	0.2351	0.0343	0.6457	No	1.17	0.0568	0.865	0.175	No	No
161069_at	Neurog1	neurogenin 1	1.0096	0.9035	0.1753	0.485	No	0.9824	0.952	0.237	0.226	No	No
161070_at	Spred2	sprouty-related, EVH1 domain containing 2	0.977	0.7965	0.304	0.5463	No	1.0265	0.45	0.0035	0.642	No	No
161071_at	Qrsl1	glutaminyl-tRNA synthase (glutamine-hydrolyzing)-like 1	1.085	0.1034	0.0642	0.3193	No	1.0509	0.456	0.442	0.698	No	No
161072_at	Nanog	Nanog homeobox	1.0601	0.4735	0.2243	0.1182	No	1.1087	0.265	0.288	0.0983	No	No
161073_at	C630002B14 Rik	RIKEN cDNA C630002B14 gene	0.9817	0.5947	0.1604	0.5663	No	1.0215	0.55	0.891	0.468	No	No
161074_at	Pcyt1a	phosphate cytidylyltransferase 1, choline, alpha isoform	1.0129	0.8264	0.0136	0.5954	No	0.9592	0.403	0.0113	0.475	No	No
161075_at	D9Ertd280e	DNA segment, Chr 9, ERATO Doi 280, expressed	1.1266	0.1262	0.1711	0.1668	No	1.1784	0.0615	0.0555	0.0728	No	No
161076_at	BC023882	cDNA sequence BC023882	0.8097	0.0116	0.1315	0.5589	No	0.8788	0.0332	0.0894	0.782	No	No
161077_f_at	Smarcd2	SWI/SNF related, matrix associated, actin dependent regulator of chromatin, subfamily d, member 2	0.9813	0.6948	0.798	0.1225	No	0.957	0.626	0.078	0.0562	No	No
161078_at	Krtap5-5	Keratin associated protein 5-5	1.0234	0.5512	0.091	0.6322	No	1.0187	0.771	0.0051	0.539	No	No
161079_at	Naalad2	N-acetylated alpha-linked acidic dipeptidase 2	1.0635	0.2927	0.011	0.2119	No	0.9998	0.979	0.932	0.823	No	No
161080_f_at	Gtf2ird2	GTF2I repeat domain containing 2	1.0744	0.4848	0.4004	0.7056	No	1.0537	0.14	0.617	0.0582	No	No
161081_at	Cpeb2	cytoplasmic polyadenylation element binding protein 2	1.1333	0.3405	0.0022	0.5795	No	1.0058	0.623	0.0015	0.234	No	No
161082_r_at	1500005I02 Rik	RIKEN cDNA 1500005I02 gene	0.9914	0.6992	0.0005	0.24	No	1.0387	0.452	0.712	0.109	No	No
161083_at	Taok3	TAO kinase 3	0.9714	0.7087	0.7891	0.645	No	1.0931	0.386	0.432	0.474	No	No
161084_at	Zfp612	zinc finger protein 612	0.9288	0.2989	0.4858	0.0874	No	0.8568	0.034	0.0773	0.0462	No	No
161085_r_at	A4galt	alpha 1,4-galactosyltransferase	0.9121	0.3913	0.0004	0.5806	No	0.938	0.159	0.0214	0.433	No	No
161086_at	Lin7c	lin-7 homolog C (C. elegans)	1.0022	0.9763	0.0116	0.1773	No	1.046	0.456	0.021	0.29	No	No
161087_r_at	---	---	1.129	0.269	0.0677	0.4966	No	1.2482	0.121	0.0087	0.237	No	No
161088_r_at	Akap8	A kinase (PRKA) anchor protein 8	1.0765	0.0949	0.3678	0.0245	No	1.0798	0.0908	0.0053	0.0966	No	No
161089_r_at	Akap8	A kinase (PRKA) anchor protein 8	0.9704	0.5375	0.6794	0.3152	No	1.487	0.413	0.786	0.606	No	No
161090_i_at	---	---	0.9713	0.6636	0.0318	0.4872	No	0.9555	0.38	0.0137	0.643	No	No
161091_r_at	---	---	0.9974	0.9503	0.0338	0.033	No	1.1306	0.0342	0	0.0438	No	No
161092_at	Umps	uridine monophosphate synthetase	0.949	0.4958	0.5595	0.9113	No	0.9555	0.236	0.571	0.96	No	No
161093_at	Ift81	intraflagellar transport 81 homolog (Chlamydomonas)	0.9874	0.8091	0.0036	0.5667	No	1.1323	0.106	0.0008	0.367	No	No
161094_r_at	Gdpd5	Glycerophosphodiester phosphodiesterase domain containing 5	1.0416	0.5089	0.576	0.0836	No	1.055	0.408	0.0326	0.0439	No	No
161095_i_at	---	---	0.9518	0.5317	0.038	0.6656	No	1.0085	0.919	0.0774	0.995	No	No
161096_at	Zfp532	zinc finger protein 532	0.9493	0.4628	0.0339	0.4125	No	0.9741	0.69	0.007	0.826	No	No
161097_at	1600014E20 Rik	RIKEN cDNA 1600014E20 gene	0.9973	0.9518	0.0185	0.9362	No	1.0715	0.272	0.0038	0.243	No	No
161098_at	Fbxl12	F-box and leucine-rich repeat protein 12	0.969	0.4593	0.0081	0.3535	No	1.0153	0.607	0.0004	0.579	No	No
161099_at	She	src homology 2 domain-containing transforming protein E	0.9959	0.9367	0.0562	0.3196	No	1.0565	0.331	0.29	0.273	No	No
161100_at	1110062M06 Rik	RIKEN cDNA 1110062M06 gene	0.8848	0.0243	0.9732	0.0768	No	1.1085	0.0861	0.0444	0.0276	No	No
161101_r_at	Btbd3	BTB (POZ) domain containing 3	0.9978	0.9483	0.0067	0.1156	No	0.9576	0.256	0.0018	0.188	No	No
161102_at	2810002I04 Rik	RIKEN cDNA 2810002I04 gene	0.9491	0.6417	0.0524	0.3179	No	0.91	0.557	0.0826	0.672	No	No
161103_at	Entpd7	ectonucleoside triphosphate diphosphohydrolase 7	0.9471	0.4737	0.9202	0.9933	No	1.0612	0.317	0.591	0.782	No	No
161104_at	38970	septin 10	1.0402	0.6841	0.0125	0.2432	No	1.0459	0.809	0.0022	0.198	No	No
161105_at	AA408650	expressed sequence AA408650	0.9591	0.151	0.0099	0.5546	No	0.9178	0.239	0.339	0.835	No	No
161106_r_at	AA415014	expressed sequence AA415014	0.9143	0.1747	0.0032	0.8462	No	0.9717	0.468	0.0015	0.71	No	No
161107_r_at	Mogat2	monoacylglycerol O-acyltransferase 2	1.1523	0.1342	0.0266	0.0384	No	1.2568	0.0432	0.0215	0.013	No	No
161108_r_at	Pla2g4f	phospholipase A2, group IVF	0.9289	0.1825	0.0082	0.0311	No	0.9912	0.502	0.0042	0.0105	No	No
161109_at	1810013L24 Rik	RIKEN cDNA 1810013L24 gene	1.0288	0.5617	0.0053	0.2265	No	0.9719	0.928	0.0066	0.628	No	No
161110_at	Tktl1	transketolase-like 1	0.9925	0.8895	0.5639	0.7002	No	1.102	0.0515	0.104	0.0581	No	No
161111_f_at	Dhh	desert hedgehog	1.0581	0.2535	0.0469	0.6944	No	1.0518	0.106	0.0045	0.198	No	No
161112_at	Parp16	poly (ADP-ribose) polymerase family, member 16	0.9025	0.0635	0.0833	0.0894	No	1.0433	0.191	0.0006	0.186	No	No
161113_at	Esr1	estrogen receptor 1 (alpha)	1.0411	0.1984	0.8973	0.3522	No	1.2696	0.0014	0.536	0.0017	No	No
161114_i_at	6330509M23 Rik	RIKEN cDNA 6330509M23 gene	0.9619	0.3313	0.0023	0.258	No	0.9573	0.559	0.648	0.439	No	No
161115_r_at	Serpinb9f /// Serpinb9e /// Serpinb9g /// LOC544923	serine (or cysteine) peptidase inhibitor, clade B, member 9f /// serine (or cysteine) peptidase inhibitor, clade B, member 9e /// serine (or cysteine) peptidase inhibitor, clade B, member 9g /// similar to serine (or cysteine) proteinase inhibitor, clade B, member 9f	0.9691	0.4403	0.27	0.4092	No	0.9697	0.6	0.353	0.262	No	No
161116_at	Pcaf	p300/CBP-associated factor	0.8815	0.0583	0.0192	0.1713	No	0.8992	0.0964	0.165	0.0332	No	No
161117_at	Dkk2	dickkopf homolog 2 (Xenopus laevis)	0.944	0.4622	0.266	0.6061	No	0.9691	0.323	0.399	0.258	No	No
161118_r_at	Bmp2	bone morphogenetic protein 2	1.0479	0.2457	0.5596	0.0374	No	1.2812	0.446	0.634	0.41	No	No
161119_at	Epha5	Eph receptor A5	1.1326	0.0321	0.0234	0.0396	No	1.1034	0.142	0.0118	0.249	No	No
161120_r_at	Tufm /// LOC669832	Tu translation elongation factor, mitochondrial /// similar to Tu translation elongation factor, mitochondrial	1.0618	0.0826	0.0432	0.0283	No	1.048	0.278	0.0337	0.81	No	No
161121_f_at	S100a13	S100 calcium binding protein A13	0.9595	0.5305	0.0022	0.2116	No	0.9131	0.13	0.0004	0.0365	No	No
161122_f_at	Ndufab1	NADH dehydrogenase (ubiquinone) 1, alpha/beta subcomplex, 1	1.0428	0.3375	0.2271	0.0809	No	1.1488	0.145	0.122	0.286	No	No
161123_i_at	Nek1	NIMA (never in mitosis gene a)-related expressed kinase 1	0.9499	0.4298	0.4951	0.2953	No	0.9836	0.722	0.0538	0.363	No	No
161124_at	Pnpla1	Patatin-like phospholipase domain containing 1	1.0658	0.2274	0.006	0.0691	No	1.0717	0.199	0.0142	0.0378	No	No

161125_at	---	---	1.0353	0.4285	0.0748	0.1594	No	0.8942	0.169	0.02	0.553	No	No
161126_at	Gm288	gene model 288, (NCBI)	1.0794	0.0394	0.0064	0.4981	No	1.2007	0.0515	0.0405	0.0453	No	No
161127_i_at	Rpl24 /// LOC624993 /// LOC667996	ribosomal protein L24 /// similar to ribosomal protein L24 /// similar to ribosomal protein L24	1.1601	0.0292	0.0236	0.926	No	1.1596	0.0751	0.122	0.791	No	No
161128_r_at	6030426L16 Rik /// LOC245297 /// LOC627899 /// LOC628979 /// LOC668413 /// LOC668594	RIKEN cDNA 6030426L16 gene /// similar to RIKEN cDNA 6030426L16 /// similar to gonadotropin inducible ovarian transcription factor 1 /// similar to Hippocalcin-like protein 1 (Visinin-like protein 3) (VILIP 3) (Neural visinin-like protein 3) (NVL-3) (NVP-3) /// similar to Hippocalcin-like protein 1 (Visinin-like protein 3) (VILIP-3) (Neural visinin-like protein 3) (NVL 3) (NVP-3) /// similar to Hippocalcin-like protein 1 (Visinin-like protein 3) (VILIP-3) (Neural visinin-like protein 3) (NVL-3) (NVP-3)	1.0122	0.8094	0.4631	0.225	No	0.8682	0.0801	0.0013	0.501	No	No
161129_r_at	Bag2	Bcl2-associated athanogene 2	0.9503	0.1516	0.0106	0.3839	No	0.9782	0.614	0.0188	0.753	No	No
161130_f_at	Klhdc4 /// LOC384360	kelch domain containing 4 /// hypothetical gene supported by NM_145605	0.9841	0.6929	0.1473	0.1686	No	1.04	0.152	0.0842	0.922	No	No
161131_r_at	---	---	1.0168	0.7972	0.6962	0.9896	No	1.0278	0.635	0.0394	0.431	No	No
161132_at	Scel	sciellin	1.0122	0.8544	0.2974	0.64	No	1.0358	0.815	0.26	0.359	No	No
161133_at	Calm2	Calmodulin 2	1.161	0.012	0.2668	0.059	No	1.0048	0.894	0.0259	0.648	No	No
161134_at	---	---	0.9557	0.3541	0.1994	0.854	No	0.998	0.93	0.0757	0.47	No	No
161135_f_at	Sac3d1	SAC3 domain containing 1	0.922	0.4166	0.3491	0.1206	No	1.001	0.931	0.261	0.0734	No	No
161136_r_at	2610001J05 Rik	RIKEN cDNA 2610001J05 gene	1.0284	0.527	0.1332	0.0112	No	1.1939	0.164	0.0956	0.04	No	No
161137_r_at	Ube2c	ubiquitin-conjugating enzyme E2C	1.1386	0.0144	0.0135	0.0645	No	1.0426	0.571	0.0346	0.138	No	No
161138_r_at	Cmas	cytidine monophospho-N-acetylneuraminic acid synthetase	1.1017	0.0553	0.0591	0.0518	No	1.1409	0.0122	0.049	0.0107	No	No
161139_f_at	Ddef1	development and differentiation enhancing	1.0359	0.3573	0.0103	0.9849	No	1.0637	0.243	0.01	0.62	No	No
161140_r_at	Hspd1	heat shock protein 1 (chaperonin) /// chaperonin	1.0135	0.8453	0.0028	0.1884	No	0.9718	0.755	0.0008	0.0396	No	No
161141_r_at	---	Transcribed locus	1.1279	0.1631	0.6718	0.0857	No	1.0905	0.904	0.804	0.0232	No	No
161142_at	4632417K18 Rik	RIKEN cDNA 4632417K18 gene	0.9921	0.9132	0.9546	0.5785	No	1.0272	0.766	0.16	0.888	No	No
161143_r_at	---	---	1.0269	0.7128	0.2961	0.9625	No	1.077	0.177	0.141	0.97	No	No
161144_r_at	Gdf3	growth differentiation factor 3	1.0321	0.5575	0.1085	0.0999	No	1.0885	0.146	0.104	0.0043	No	No
161145_f_at	Atp5d	ATP synthase, H+ transporting, mitochondrial F1 complex, delta subunit	0.9655	0.5837	0.0137	0.1177	No	0.9415	0.756	0.0302	0.0495	No	No
161146_r_at	---	---	1.077	0.521	0.2898	0.6529	No	1.1382	0.0263	0.0361	0.0142	No	No
161147_f_at	---	---	0.9937	0.8512	0.0064	0.8123	No	1.0319	0.546	0.201	0.772	No	No
161148_f_at	Ing4	inhibitor of growth family, member 4	1.0149	0.6323	0.076	0.5773	No	1.0792	0.0693	0.159	0.583	No	No
161149_r_at	Slc23a3	solute carrier family 23 (nucleobase transporters), member 3	1.0261	0.7469	0	0.4566	No	1.0546	0.596	0.0004	0.524	No	No
161150_at	Tk1 /// LOC619653 /// LOC638277	thymidine kinase 1 /// similar to Thymidine kinase, cytosolic /// similar to Thymidine kinase, cytosolic	0.9685	0.6339	0.0467	0.565	No	0.9448	0.403	0.0361	0.623	No	No
161151_at	Epb4.2	erythrocyte protein band 4.2	0.9821	0.7508	0.5346	0.9506	No	1.0822	0.0878	0.0099	0.0495	No	No
161152_r_at	AL033326	expressed sequence AL033326	0.9829	0.7942	0.1081	0.0439	No	1.0203	0.644	0.0412	0.0666	No	No
161153_r_at	---	---	1.1011	0.1099	0.0486	0.0038	No	1.0562	0.577	0.0131	0.379	No	No
161154_at	---	---	0.9501	0.2195	0.7492	0.154	No	0.9625	0.348	0.039	0.358	No	No
161155_r_at	Enpep	glutamyl aminopeptidase	1.0834	0.121	0.3777	0.0127	No	1.0501	0.352	0.708	0.0295	No	No
161156_r_at	---	---	0.9793	0.6666	0.986	0.1841	No	1.0004	0.991	0.514	0.176	No	No
161157_r_at	Tgfbi	transforming growth factor, beta induced	1.0952	0.0969	0.1765	0.1083	No	0.856	0.267	0.221	0.735	No	No
161158_f_at	Ereg	epiregulin	1.0035	0.947	0.5091	0.5605	No	1.1826	0.353	0.866	0.221	No	No
161159_r_at	Pou5f1	POU domain, class 5, transcription factor 1	0.9464	0.425	0.1604	0.7626	No	1.2198	0.0187	0.0342	0.0177	No	No
161160_f_at	Mybl2	myeloblastosis oncogene-like 2	1.0128	0.8602	0.4889	0.2183	No	1.0618	0.495	0.423	0.11	No	No
161161_r_at	Nme1	expressed in non-metastatic cells 1, protein	1.247	0.0234	0.0126	0.0216	No	1.0737	0.206	0.0149	0.566	No	No
161162_at	Rbm35b	RNA binding motif protein 35b	1.1009	0.0172	0.0024	0.0719	No	1.0818	0.0453	0.0053	0.422	No	No
161163_at	Apbb2	amyloid beta (A4) precursor protein-binding, family B, member 2	0.8556	0.0189	0.0121	0.0282	No	0.944	0.393	0.0062	0.808	No	No
161164_r_at	Penk1	preproenkephalin 1	1.0674	0.3636	0.4469	0.1514	No	1.3366	0.22	0.219	0.0929	No	No
161165_f_at	Lpin2	lipin 2	0.9935	0.8666	0.5373	0.1442	No	0.9794	0.981	0.205	0.253	No	No
161166_i_at	---	---	1.0238	0.6089	0.2865	0.269	No	1.3183	0.0985	0.433	0.345	No	No
161167_r_at	Uck1	uridine-cytidine kinase 1	1.128	0.2091	0.0959	0.0965	No	1.2225	0.0573	0.247	0.0259	No	No
161168_at	---	---	0.9567	0.3118	0.0104	0.0369	No	1.1002	0.414	0.158	0.0344	No	No
161169_f_at	1810030O07 Rik	RIKEN cDNA 1810030O07 gene	1.1304	0.0464	0.1632	0.0283	No	1.1091	0.0711	0.0105	0.196	No	No
161170_r_at	Dnmt3a	DNA methyltransferase 3A	1.0443	0.5837	0.5842	0.1332	No	1.1023	0.498	0.121	0.17	No	No
161171_at	Dusp8	dual specificity phosphatase 8	1.1047	0.0807	0.0335	0.5993	No	1.1348	0.0415	0.0502	0.066	No	No
161172_f_at	Brrn1	barren homolog (Drosophila)	1.1606	0.032	0.0027	0.0719	No	1.1692	0.0748	0.0045	0.2	No	No
161173_f_at	Ifi202b	interferon activated gene 202B	0.8606	0.085	0.1481	0.37	No	0.7095	0.0434	0.64	0.277	No	No
161174_i_at	Krt2-19	keratin complex 2, basic, gene 19	0.8784	0.0605	0.0187	0.7396	No	0.8852	0.039	0.0248	0.339	No	No
161175_r_at	Krt2-19	keratin complex 2, basic, gene 19	1.0093	0.7924	0.0309	0.0156	No	0.6128	0.517	0.109	0.254	No	No
161176_r_at	Stom	stomatin	1.1085	0.0888	0.0499	0.3705	No	1.1415	0.0636	0.0441	0.454	No	No
161177_f_at	---	---	1.0168	0.8025	0.2246	0.2126	No	0.969	0.775	0.0462	0.383	No	No
161178_at	Centd3	centaurin, delta 3	1.0277	0.533	0.2724	0.9892	No	0.4257	0.12	0.464	0.733	No	No
161179_at	---	---	1.2044	0.0136	0.0476	0.0714	No	1.0835	0.078	0.0174	0.0351	No	No
161180_r_at	Ifi204 /// LOC672547	interferon activated gene 204 /// similar to Interferon-activatable protein 204 (Ifi-204) (Interferon-inducible protein p204)	0.9825	0.7128	0.1323	0.4299	No	0.9758	0.583	0.48	0.371	No	No
161181_f_at	Dusp16	dual specificity phosphatase 16	0.9449	0.5637	0.6053	0.8613	No	0.957	0.893	0.0735	0.486	No	No
161182_r_at	Set	SET translocation	1.0028	0.8985	0.3671	0.018	No	2.9681	0.069	0.145	0.362	No	No
161183_at	Dag1	dystroglycan 1	0.9482	0.4482	0.114	0.3703	No	1.0492	0.631	0.0175	0.848	No	No
161184_f_at	Tie1	tyrosine kinase receptor 1	0.9499	0.3422	0.0239	0.1012	No	0.8782	0.296	0.0167	0.103	No	No
161185_i_at	Klf4	Kruppel-like factor 4 (gut)	1.0448	0.3769	0.0393	0.0718	No	1.0268	0.976	0.126	0.423	No	No
161186_f_at	Tyki	thymidylate kinase family LPS-inducible member	0.977	0.6045	0.455	0.6259	No	1.0657	0.415	0.681	0.411	No	No
161187_f_at	5730589K01 Rik	RIKEN cDNA 5730589K01 gene	1.0005	0.9952	0.1809	0.7907	No	1.0427	0.372	0	0.933	No	No
161188_f_at	Lamb3	laminin, beta 3	0.9536	0.5305	0.1119	0.8282	No	0.9674	0.539	0.915	0.228	No	No
161189_r_at	---	---	1.1418	0.0594	0.0228	0.012	No	1.1814	0.0353	0.0162	0.0671	No	No
161190_r_at	1110057K04 Rik	RIKEN cDNA 1110057K04 gene	1.2273	0.1786	0.3393	0.1607	No	1.3203	0.0586	0.0844	0.0647	No	No
161191_i_at	Grsf1	G-rich RNA sequence binding factor 1	1.0792	0.1612	0.0203	0.4523	No	1.0608	0.227	0.611	0.0717	No	No
161192_at	Il4ra	Interleukin 4 receptor, alpha	1.1003	0.1961	0.2812	0.069	No	1.1182	0.107	0.13	0.0179	No	No
161193_r_at	---	---	0.9076	0.402	0.0001	0.5771	No	0.962	0.589	0.0001	0.831	No	No

161194_r_at	Tnfrsf1a	tumor necrosis factor receptor superfamily, member 1a	1.09	0.2663	0.2572	0.5058	No	1.1031	0.105	0.0207	0.124	No	No
161195_i_at	Snw1	SNW domain containing 1	1.0134	0.821	0.7351	0.0976	No	1.0486	0.104	0.0034	0.0077	No	No
161196_r_at	Adam3	a disintegrin and metallopeptidase domain 3 (cyritestin)	1.1217	0.0207	0.0093	0.0069	No	1.0637	0.105	0.0011	0.0016	No	No
161197_r_at	D10Wsu52e	DNA segment, Chr 10, Wayne State University 52, expressed	1.1873	0.0569	0.004	0.0709	No	1.0115	0.938	0.0008	0.627	No	No
161198_r_at	---	---	0.9983	0.9555	0.006	0.6911	No	0.9812	0.482	0.0098	0.691	No	No
161199_at	Ubxd5	UBX domain containing 5	1.0105	0.502	0.36	0.2038	No	1.058	0.433	0.388	0.0724	No	No
161200_i_at	---	---	0.9025	0.2328	0.273	0.8501	No	0.8665	0.0189	0.171	0.0532	No	No
161201_r_at	Slc9a1	solute carrier family 9 (sodium/hydrogen exchanger), member 1	1.0399	0.1097	0.0014	0.0497	No	1.0009	0.95	0.0516	0.122	No	No
161202_r_at	Pdgfra	platelet derived growth factor receptor, alpha polypeptide	1.003	0.9534	0.2011	0.5363	No	0.9367	0.864	0.649	0.711	No	No
161203_f_at	Tanc1	tetratricopeptide repeat, ankyrin repeat and coiled-coil containing 1	1.0987	0.1199	0.0139	0.1536	No	1.0959	0.338	0.0195	0.813	No	No
161204_r_at	Rabgap1l	RAB GTPase activating protein 1-like	1.0382	0.2396	0.2062	0.2152	No	3.3854	0.258	0.0983	0.758	No	No
161205_at	Cox8b	Cytochrome c oxidase, subunit VIIIb	1.0444	0.5707	0.2244	0.0612	No	1.0847	0.126	0.0111	0.358	No	No
161206_at	Plekhh1	pleckstrin homology domain containing, family H (with MyTH4 domain) member 1	1.065	0.3729	0.7417	0.4515	No	1.1373	0.0273	0.135	0.226	No	No
161207_at	---	---	0.9819	0.7977	0.8406	0.1392	No	1.2085	0.211	0.892	0.0383	No	No
161208_r_at	Calu	Calumenin	1.2054	0.1081	0.1086	0.1388	No	1.1944	0.0427	0.143	0.0338	No	No
161209_r_at	Rab3ip	RAB3A interacting protein	0.9391	0.1007	0.1692	0.4448	No	0.9272	0.173	0.0055	0.701	No	No
161210_f_at	Cnga2	cyclic nucleotide gated channel alpha 2	1.0235	0.7219	0.3679	0.6101	No	1.0417	0.622	0.111	0.705	No	No
161211_r_at	Sox6	SRY-box containing gene 6	0.9785	0.6483	0.725	0.6806	No	0.9464	0.293	0.417	0.441	No	No
161212_r_at	Agtr2	angiotensin II receptor, type 2	0.9432	0.2805	0.1567	0.4562	No	0.8537	0.145	0.38	0.0547	No	No
161213_r_at	---	---	1.1554	0.1531	0.1117	0.0655	No	1.3336	0.0172	0.112	0.0802	No	No
161214_r_at	BC037034	cDNA sequence BC037034	0.9815	0.8421	0.0149	0.8776	No	0.9972	0.992	0.011	0.992	No	No
161215_at	Chrng	cholinergic receptor, nicotinic, gamma polypeptide	1.045	0.1651	0.6841	0.0596	No	0.9939	0.864	0.821	0.114	No	No
161216_at	Tnp1	transition protein 1	1.0104	0.7496	0.3823	0.3097	No	1.0859	0.118	0.171	0.352	No	No
161217_r_at	---	---	1.1671	0.0282	0.0351	0.0899	No	1.1341	0.0498	0.0235	0.151	No	No
161218_r_at	Pafah1b3	platelet-activating factor acetylhydrolase, isoform 1b, alpha1 subunit	0.9537	0.3768	0.6756	0.0312	No	0.8626	0.471	0.09	0.303	No	No
161219_r_at	---	---	1.0577	0.4492	0.5112	0.0465	No	0.9816	0.698	0.0195	0.067	No	No
161220_f_at	Ubn1	ubinuclein 1	1.0771	0.3883	0.0314	0.5479	No	1.1204	0.197	0.187	0.855	No	No
161221_f_at	Asns	asparagine synthetase	1.0821	0.2239	0.0359	0.2809	No	1.1523	0.0269	0.0011	0.297	No	No
161222_r_at	Tmem50b	transmembrane protein 50B	0.9435	0.3263	0.2073	0.0479	No	0.9429	0.102	0.0934	0.0023	No	No
161223_r_at	Flnb	filamin, beta	0.9886	0.8785	0.9414	0.2985	No	0.9865	0.76	0.176	0.246	No	No
161224_f_at	Ace	angiotensin I converting enzyme (peptidyl-dipeptidase A) 1	0.8477	0.1014	0.027	0.6385	No	0.8755	0.307	0.0277	0.609	No	No
161225_r_at	Cry1	cryptochrome 1 (photolyase-like)	1.0461	0.3731	0.2122	0.2485	No	2.2462	0.207	0.107	0.169	No	No
161226_f_at	1700073E17Rik	RIKEN cDNA 1700073E17 gene	1.1627	0.0181	0.6141	0.222	No	1.1037	0.241	0.0572	0.355	No	No
161227_r_at	---	---	1.063	0.4054	0.3556	0.6581	No	1.0806	0.279	0.237	0.496	No	No
161228_f_at	Enah	Enabled homolog (Drosophila)	1.079	0.1646	0.0327	0.8226	No	1.0033	0.977	0.143	0.96	No	No
161229_at	Prm2	Protamine 2	0.9037	0.4694	0.1355	0.1962	No	0.9557	0.927	0.196	0.396	No	No
161230_r_at	Slc30a4	solute carrier family 30 (zinc transporter), member 4	0.9251	0.1293	0.0222	0.3221	No	0.9778	0.842	0.556	0.436	No	No
161231_r_at	Reln	reelin	0.934	0.4959	0.1388	0.5302	No	0.8673	0.246	0.0582	0.581	No	No
161232_r_at	Gpiap1	GPI-anchored membrane protein 1	0.9865	0.7976	0.6485	0.1073	No	0.972	0.873	0.288	0.241	No	No
161233_at	Pim2	proviral integration site 2	0.9068	0.3773	0.5902	0.3746	No	1.0223	0.723	0.298	0.154	No	No
161234_f_at	38964	septin 4	0.9525	0.3872	0.3498	0.9904	No	1.0075	0.866	0.235	0.721	No	No
161235_f_at	Bat4	HLA-B associated transcript 4	0.9443	0.0374	0.0141	0.0167	No	1.1096	0.0049	0.0001	0.0466	Yes	Yes
161236_r_at	2610209M04Rik	RIKEN cDNA 2610209M04 gene	1.0427	0.2286	0.0523	0.0088	No	1.2204	0.158	0.0684	0.0501	No	No
161237_at	Sbno1	sno, strawberry notch homolog 1 (Drosophila)	1.0474	0.3405	0.7645	0.0092	No	1.0224	0.66	0.334	0.0495	No	No
161238_f_at	Cct3	chaperonin subunit 3 (gamma)	1.0492	0.1995	0.8795	0.8193	No	1.0237	0.291	0.622	0.679	No	No
161239_r_at	Chl1	Cell adhesion molecule with homology to L1CAM	1.0162	0.662	0.5501	0.0913	No	0.9201	0.801	0.117	0.795	No	No
161240_f_at	---	---	1.0638	0.5285	0.552	0.8246	No	1.059	0.39	0.206	0.101	No	No
161241_at	Noc4l	Nucleolar complex associated 4 homolog (S. cerevisiae)	1.1613	0.0508	0.5056	0.0516	No	1.1983	0.0219	0.315	0.0206	No	No
161242_f_at	Usp39	ubiquitin specific peptidase 39	1.0804	0.0035	0.0022	0.2341	Yes	1.0709	0.222	0.256	0.708	No	No
161243_f_at	Abhd8	abhydrolase domain containing 8	1.0349	0.4018	0.042	0.0397	No	1.0148	0.662	0.997	0.567	No	No
161244_f_at	Pstpip1	proline-serine-threonine phosphatase-interacting protein 1	1.0032	0.9196	0.8174	0.775	No	1.0251	0.715	0.074	0.885	No	No
161245_r_at	Klhl21	kelch-like 21 (Drosophila)	1.1071	0.0608	0.0347	0.0221	No	1.4434	0.0625	0.772	0.281	No	No
161246_at	Arg2	Arginase type II	1.0736	0.2466	0.8663	0.157	No	1.1883	0.0119	0.32	0.0622	No	No
161247_f_at	Dgcr2	DiGeorge syndrome critical region gene 2	1.1343	0.1207	0.0009	0.805	No	1.1297	0.372	0.0003	0.141	No	No
161248_r_at	---	---	0.9772	0.6737	0.0092	0.9494	No	0.9756	0.334	0.0005	0.265	No	No
161249_at	Ap1b1	adaptor protein complex AP-1, beta 1 subunit	1.0884	0.1569	0.0495	0.3941	No	1.0675	0.132	0.0001	0.0811	No	No
161250_at	Ipo4	importin 4	1.0201	0.294	0.0004	0.0207	No	1.1584	0.0084	0.0001	0.349	Yes	No
161251_f_at	---	---	1.1249	0.0017	0.0212	0.033	Yes	0.9838	0.529	0.0014	0.845	No	No
161252_r_at	Trim25	tripartite motif protein 25	1.0169	0.1954	0.0002	0.0003	No	1.0462	0.396	0.0179	0.0561	No	No
161253_i_at	Plod3	procollagen-lysine, 2-oxoglutarate 5-dioxygenase 3	1.0713	0.2436	0.102	0.0579	No	1.0525	0.639	0.772	0.199	No	No
161254_r_at	Ctnna1	catenin (cadherin associated protein), alpha 1	1.191	0.0248	0.453	0.0824	No	1.2738	0.0005	0.968	0.002	No	No
161255_at	Vdac2	voltage-dependent anion channel 2	1.0899	0.1287	0.3357	0.8382	No	1.0443	0.252	0.0173	0.21	No	No
161256_at	Orc5l	origin recognition complex, subunit 5-like (S. cerevisiae)	1.0304	0.5475	0.5987	0.7893	No	1.1941	0.312	0.18	0.224	No	No
161257_r_at	Snx17	sorting nexin 17	1.0572	0.477	0.0074	0.1872	No	1.0237	0.609	0.0067	0.197	No	No
161258_at	---	---	1.0526	0.1442	0.001	0.0568	No	1.0237	0.117	0.0002	0.0074	No	No
161259_f_at	Serpina5	serine (or cysteine) peptidase inhibitor, clade A, member 5	1.0474	0.3066	0.0186	0.2684	No	0.9832	0.749	0.0173	0.315	No	No
161260_f_at	Rgl1	ral guanine nucleotide dissociation stimulator,-like 1	1.0522	0.3174	0.027	0.1117	No	0.9209	0.03	0.0362	0.0048	No	No

161261_f_at	Hsd3b1	hydroxysteroid dehydrogenase-1, delta<5>-3-beta	1.178	0.0139	0.3764	0.0053	No	1.2353	0.161	0.718	0.431	No	No
161262_r_at	Odf2	outer dense fiber of sperm tails 2	1.1375	0.4634	0.0009	0.1832	No	1.1743	0.561	0.0004	0.541	No	No
161263_f_at	Casp2	caspase 2	1.0962	0.1988	0.0084	0.5903	No	1.0577	0.329	0.0033	0.936	No	No
161264_f_at	---	---	0.9987	0.9703	0.3247	0.8045	No	0.9991	0.971	0.938	0.262	No	No
161265_f_at	Lck	lymphocyte protein tyrosine kinase	1.0346	0.0914	0.3454	0.789	No	0.9415	0.337	0.758	0.1	No	No
161266_r_at	Ccdc52	coiled-coil domain containing 52	1.0142	0.6978	0.9168	0.0501	No	1.0736	0.364	0.934	0.892	No	No
161267_f_at	B3gnt2	UDP-GlcNAc:betaGal beta-1,3-N-acetylglucosaminyltransferase 2	1.0538	0.5525	0.3114	0.7066	No	1.0483	0.519	0.888	0.986	No	No
161268_f_at	---	---	1.0049	0.8725	0.0036	0.006	No	1.163	0.032	0.0231	0.0517	No	No
161269_i_at	Ren1 /// Ren2	renin 1 structural /// renin 2 tandem duplication of Ren1	0.9281	0.0889	0.0151	0.3327	No	1.2228	0.367	0.188	0.438	No	No
161270_i_at	Wnk1	WNK lysine deficient protein kinase 1	0.5889	0.0049	0.0001	0.0165	Yes	0.7297	0.0046	0.0002	0.0061	No	No
161271_r_at	Ppox	protoporphyrinogen oxidase	0.8213	0.1311	0.0008	0.1108	No	0.8556	0.181	0.0001	0.371	No	No
161272_f_at	---	---	0.9882	0.7428	0.0788	0.0836	No	1.0203	0.614	0.615	0.0451	No	No
161273_f_at	Ddx3x	DEAD/H (Asp-Glu-Ala-Asp/His) box polypeptide 3, X-linked	1.095	0.259	0.1309	0.3045	No	1.1821	0.102	0.0268	0.582	No	No
161274_at	---	---	1.031	0.2941	0.0634	0.999	No	1.0295	0.629	0.13	0.583	No	No
161275_at	Kif3a	kinesin family member 3A	0.8847	0.1481	0.2135	0.7129	No	0.9105	0.241	0.0122	0.415	No	No
161276_i_at	Sox3	SRY-box containing gene 3	1.0631	0.5969	0.7955	0.5529	No	1.048	0.654	0.763	0.633	No	No
161277_r_at	Tor3a	torsin family 3, member A	1.0285	0.736	0.2757	0.3284	No	1.0874	0.612	0.215	0.755	No	No
161278_r_at	Oat	ornithine aminotransferase	0.9079	0.2208	0.0195	0.3421	No	0.9354	0.416	0.0219	0.67	No	No
161279_f_at	---	---	1.0868	0.1	0.0164	0.8159	No	0.9803	0.988	0.992	0.679	No	No
161280_r_at	Pelo	pelota homolog (Drosophila)	1.0728	0.034	0.0035	0.0083	No	1.0571	0.0163	0.0002	0.0118	No	No
161281_f_at	---	---	1.158	0.0236	0.0007	0.3559	No	1.0188	0.97	0.0007	0.484	No	No
161282_r_at	Krt2-6g	Keratin complex 2, basic, gene 6g	1.1672	0.0171	0.0089	0.0095	No	1.0862	0.108	0.0665	0.0312	No	No
161283_i_at	---	---	1.0098	0.881	0.0216	0.6062	No	0.9511	0.516	0.0185	0.913	No	No
161284_r_at	Txnrd3	Thioredoxin reductase 3	0.8299	0.0589	0.0094	0.3364	No	0.8573	0.1	0.0073	0.143	No	No
161285_r_at	Stt3a	STT3, subunit of the oligosaccharyltransferase complex, homolog A (S. cerevisiae)	1.0293	0.6917	0.362	0.0369	No	0.9171	0.517	0.0931	0.0632	No	No
161286_f_at	Arsa	arylsulfatase A	0.9469	0.464	0.0337	0.6751	No	1.0709	0.371	0.0004	0.0957	No	No
161287_f_at	Mybbp1a	MYB binding protein (P160) 1a	1.0152	0.6505	0.0677	0.0895	No	1.0485	0.476	0.24	0.219	No	No
161288_r_at	Lman1	lectin, mannose-binding, 1	1.0954	0.1533	0.578	0.0256	No	1.1008	0.544	0.19	0.0557	No	No
161289_at	---	---	1.0614	0.45	0.0564	0.2307	No	1.2686	0.196	0.101	0.174	No	No
161290_r_at	Brd7 /// LOC634327	bromodomain containing 7 /// similar to bromodomain containing 7	0.9861	0.5459	0.0921	0.0269	No	0.8813	0.604	0.0744	0.143	No	No
161291_at	Dab1	disabled homolog 1 (Drosophila)	1.0535	0.431	0.0892	0.7385	No	1.0907	0.482	0.321	0.338	No	No
161292_f_at	Plk1	polo-like kinase 1 (Drosophila)	1.0957	0.0256	0.009	0.0389	No	1.1638	0.0018	0.0431	0.0021	No	No
161293_r_at	Psme1	proteasome (prosome, macropain) 28 subunit, alpha	1.0778	0.1248	0.0117	0.0536	No	1.2079	0.023	0.102	0.0116	No	No
161294_f_at	Clu	clusterin	1.189	0.0026	0.0019	0.0035	No	1.2096	0.0297	0.0666	0.0655	No	No
161295_r_at	Map4k4	mitogen-activated protein kinase kinase kinase kinase 4	0.9363	0.2282	0.277	0.2307	No	0.8475	0.451	0.341	0.141	No	No
161296_r_at	---	---	0.9936	0.8924	0.1035	0.266	No	1.0251	0.788	0.0352	0.409	No	No
161297_f_at	Vpreb1	pre-B lymphocyte gene 1	1.0894	0.1289	0.0318	0.0564	No	1.1111	0.0841	0.0469	0.0903	No	No
161298_i_at	---	---	1.0319	0.6532	0.0032	0.0979	No	1.0004	0.881	0.0002	0.687	No	No
161299_r_at	---	---	1.073	0.2857	0.8289	0.1947	No	1.0889	0.307	0.823	0.158	No	No
161300_r_at	---	---	0.9767	0.4091	0.0235	0.1478	No	1.0002	0.99	0.171	0.494	No	No
161301_f_at	Lgals9	lectin, galactose binding, soluble 9	1.0201	0.7566	0.0094	0.3018	No	0.9901	0.992	0.0259	0.336	No	No
161302_r_at	Reg2	regenerating islet-derived 2	1.0549	0.5496	0.0001	0.4134	No	1.069	0.69	0.0001	0.406	No	No
161303_at	---	---	1.0183	0.7096	0.0043	0.9709	No	0.9523	0.348	0.0014	0.0307	No	No
161304_r_at	Gna-rs1	guanine nucleotide binding protein, related sequence 1	1.0286	0.6823	0.3775	0.9529	No	1.0204	0.512	0.052	0.475	No	No
161305_r_at	Usp4	ubiquitin specific peptidase 4 (proto-oncogene)	1.0067	0.8974	0.0229	0.1186	No	1.0972	0.232	0.0113	0.312	No	No
161306_r_at	1810054D07 Rik	RIKEN cDNA 1810054D07 gene	0.9861	0.4624	0.0212	0.3715	No	0.8925	0.522	0.152	0.377	No	No
161307_f_at	Capn10	calpain 10	1.0257	0.7316	0.0884	0.5503	No	1.0266	0.688	0.0158	0.927	No	No
161308_f_at	Yap1	yes-associated protein 1	1.0975	0.1996	0.1629	0.2768	No	1.0294	0.659	0.001	0.693	No	No
161309_r_at	Rgs11	regulator of G-protein signaling 11	1.0294	0.5471	0.0143	0.0173	No	1.1255	0.179	0.0088	0.117	No	No
161310_at	---	---	0.8884	0.1521	0.0235	0.2777	No	0.8567	0.018	0.0013	0.0104	No	No
161311_at	Snrpe	Small nuclear ribonucleoprotein E	0.8001	0.0643	0.0115	0.0442	No	0.8886	0.133	0.0001	0.0088	No	No
161312_r_at	---	---	1.0842	0.2195	0.884	0.0409	No	1.0802	0.541	0.509	0.0759	No	No
161313_at	Hk2	hexokinase 2	0.9455	0.6102	0.0678	0.4949	No	0.9849	0.97	0.141	0.481	No	No
161314_r_at	Zubr1	zinc finger, UBR1 type 1	1.3527	0.0135	0.0062	0.0118	No	1.2611	0.0161	0.0143	0.0064	No	No
161315_i_at	Tm4sf1	transmembrane 4 superfamily member 1	1.0536	0.6261	0.4756	0.1888	No	1.1595	0.184	0.144	0.198	No	No
161316_f_at	---	---	1.0107	0.8329	0.1137	0.1584	No	1.0358	0.0753	0.0005	0.0068	No	No
161317_r_at	---	---	0.9437	0.1708	0.1003	0.3315	No	0.8797	0.272	0.196	0.056	No	No
161318_f_at	Ctnna2	catenin (cadherin associated protein), alpha 2	0.9807	0.7568	0.0274	0.0263	No	1.0603	0.147	0.014	0.0116	No	No
161319_at	Copz1	coatomer protein complex, subunit zeta 1	0.9993	0.9812	0.1341	0.0303	No	0.8575	0.122	0.494	0.346	No	No
161320_r_at	Tpm2	tropomyosin 2, beta	1.0074	0.9532	0.0488	0.3732	No	1.1459	0.0554	0.0137	0.0075	No	No
161321_i_at	Apoe	apolipoprotein E	1.0603	0.413	0.0485	0.5398	No	1.2218	0.0614	0.0793	0.586	No	No
161322_r_at	Atad3a	ATPase family, AAA domain containing 3A	1.0278	0.5986	0.2662	0.0647	No	1.1116	0.427	0.0514	0.0741	No	No
161323_f_at	Cad	carbamoyl-phosphate synthetase 2, aspartate transcarbamylase, and dihydroorotase	0.9388	0.4798	0.151	0.5535	No	0.8552	0.0799	0.0032	0.42	No	No

161324_r_at	Atp5g1 /// LOC625672	ATP synthase, H+ transporting, mitochondrial F0 complex, subunit c (subunit 9), isoform 1 /// similar to ATP synthase lipid-binding protein, mitochondrial precursor (ATP synthase proteolipid P1) (ATPase protein 9) (ATPase subunit C)	1.0078	0.8679	0.4972	0.6383	No	0.9713	0.168	0.0398	0.0069	No	No
161325_at	Upp1	uridine phosphorylase 1	1.0631	0.0498	0.1672	0.0066	No	1.0438	0.187	0.253	0.0934	No	No
161326_f_at	Serpina6	serine (or cysteine) peptidase inhibitor, clade A, member 6	1.0433	0.6784	0.113	0.5941	No	1.0894	0.253	0.761	0.254	No	No
161327_f_at	Rpl10a /// LOC546651 /// LOC625861 /// LOC628769 /// LOC639622	ribosomal protein L10A /// similar to ribosomal protein L10a /// similar to ribosomal protein L10a /// similar to ribosomal protein L10a /// similar to ribosomal protein L10a	1.0376	0.4651	0.0219	0.6531	No	1.0336	0.603	0.043	0.402	No	No
161328_i_at	Urod	uroporphyrinogen decarboxylase	0.9758	0.6087	0.0079	0.1906	No	0.9517	0.278	0.282	0.0365	No	No
161329_f_at	Capzb	capping protein (actin filament) muscle Z-line, beta	0.901	0.3411	0.0211	0.9658	No	0.9379	0.535	0.0182	0.811	No	No
161330_r_at	Aprt	adenine phosphoribosyl transferase	0.944	0.026	0.0001	0.022	No	0.9209	0.0344	0.0004	0.0404	No	No
161331_r_at	---	---	1.0538	0.5824	0.0924	0.3494	No	0.9588	0.896	0.0001	0.11	No	No
161332_f_at	Cdca5	cell division cycle associated 5	1.1102	0.0675	0.3813	0.1041	No	1.1127	0.1	0.433	0.0701	No	No
161333_f_at	D1Ertd161e	DNA segment, Chr 1, ERATO Doi 161, expressed	0.9579	0.2834	0.5749	0.6802	No	0.9611	0.513	0.0013	0.421	No	No
161334_r_at	Zfp277	zinc finger protein 277	1.0814	0.2804	0.0202	0.04	No	1.0782	0.0935	0.0025	0.0525	No	No
161335_r_at	Snrpb	small nuclear ribonucleoprotein B	1.0633	0.3295	0.1606	0.1163	No	1.3022	0.0948	0.0342	0.102	No	No
161336_r_at	Ndst2	N-deacetylase/N-sulfotransferase (heparan glucosaminyl) 2	0.8085	0.016	0.0024	0.0546	No	0.9162	0.173	0.0134	0.392	No	No
161337_f_at	Wars	tryptophanyl-tRNA synthetase	1.0148	0.7651	0.5811	0.9926	No	1.0099	0.829	0.104	0.0164	No	No
161338_i_at	Uqcr	ubiquinol-cytochrome c reductase (6.4kD) subunit	0.9973	0.9274	0.0002	0.2423	No	1.0105	0.612	0.0002	0.389	No	No
161339_f_at	Sars2	seryl-aminoacyl-tRNA synthetase 2	0.9607	0.2286	0.0599	0.0125	No	1.0059	0.628	0.0192	0.0075	No	No
161340_r_at	Pitx2	paired-like homeodomain transcription factor 2	1.1477	0.0976	0.1489	0.0333	No	1.2459	0.0697	0.576	0.254	No	No
161341_f_at	Gabpb1	GA repeat binding protein, beta 1	1.156	0.1084	0.1005	0.2449	No	0.9794	0.769	0.816	0.88	No	No
161342_r_at	Eif4b	eukaryotic translation initiation factor 4B	0.9768	0.6921	0.001	0.4904	No	0.997	0.833	0.003	0.579	No	No
161343_r_at	---	---	1.0493	0.5067	0.1383	0.5839	No	1.0673	0.251	0.0902	0.8	No	No
161344_r_at	Etf1	eukaryotic translation termination factor 1	1.1079	0.1387	0.0957	0.0532	No	1.044	0.553	0.19	0.175	No	No
161345_f_at	Cyp7b1	Cytochrome P450, family 7, subfamily b, polypeptide 1	1.0949	0.0767	0.0191	0.4931	No	1.0004	0.931	0.381	0.28	No	No
161346_f_at	Klk8	kallikrein 8	1.0059	0.9136	0.0043	0.0858	No	1.0206	0.576	0.0032	0.279	No	No
161347_r_at	Rpo1-1	RNA polymerase 1-1	1.258	0.0198	0.0046	0.0565	No	1.1223	0.0447	0.0017	0.0217	No	No
161348_r_at	Pdlim1 /// LOC545743	PDZ and LIM domain 1 (elfin) /// similar to PDZ and LIM domain protein 1 (Elfin) (LIM domain protein CLP-36) (C-terminal LIM domain protein 1)	1.066	0.1275	0.0061	0.1279	No	0.9731	0.425	0.0282	0.0228	No	No
161349_f_at	Dpm2	Dolichol-phosphate (beta-D) mannosyltransferase 2	0.9759	0.7226	0.0139	0.0604	No	0.9497	0.503	0.0156	0.0386	No	No
161350_f_at	Enpp2	ectonucleotide pyrophosphatase/phosphodiesterase 2	1.1473	0.0172	0.0535	0.0498	No	1.2311	0.0421	0.347	0.0157	No	No
161351_r_at	---	---	0.9668	0.3229	0.3917	0.6345	No	1.0215	0.936	0.0538	0.343	No	No
161352_r_at	---	---	1.0923	0.3064	0.0747	0.0288	No	1.0666	0.565	0.0002	0.0358	No	No
161353_r_at	1110007M04Rik	RIKEN cDNA 1110007M04 gene	0.9727	0.7671	0.0597	0.4488	No	0.8251	0.535	0.173	0.615	No	No
161354_f_at	6330403K07Rik	RIKEN cDNA 6330403K07 gene	1.115	0.0819	0.0294	0.7175	No	1.1193	0.395	0.0521	0.573	No	No
161355_f_at	6030458H05	hypothetical protein 6030458H05	1.0901	0.1953	0.8721	0.9825	No	0.9835	0.907	0.0999	0.22	No	No
161356_at	---	---	1.0927	0.0616	0.0066	0.4215	No	0.986	0.791	0.0988	0.519	No	No
161357_r_at	Gstm2 /// LOC626327 /// LOC670888	glutathione S-transferase, mu 2 /// similar to Glutathione S-transferase Mu 2 (GST class-mu 2) (Glutathione S-transferase pmGT2) (GST 5-5) /// similar to Glutathione S-transferase Mu 2 (GST class-mu 2) (Glutathione S-transferase pmGT2) (GST 5-5)	1.1147	0.0158	0.0028	0.0029	No	1.1117	0.019	0.003	0.0025	No	No
161358_r_at	Dpep3	dipeptidase 3	0.9838	0.8222	0.4431	0.1951	No	1.0119	0.816	0.0018	0.0544	No	No
161359_s_at	Apoa1bp	apolipoprotein A-I binding protein	1.024	0.44	0.0067	0.006	No	1.051	0.232	0.004	0.199	No	No
161360_at	Oaz1	ornithine decarboxylase antizyme	1.0391	0.4459	0.0211	0.0996	No	1.0864	0.0994	0.927	0.859	No	No
161361_s_at	Tnnt1	troponin T1, skeletal, slow	0.7786	0.0003	0.0082	0.0002	No	0.7956	0.0002	0.0025	0.0001	No	No
161362_at	---	---	0.923	0.2089	0.6702	0.2093	No	0.9668	0.596	0.663	0.306	No	No
161363_r_at	---	---	1.0794	0.0732	0.2443	0.123	No	0.8921	0.012	0.0007	0.0083	No	No
161364_f_at	Faf1	Fas-associated factor 1	1.038	0.5892	0.1671	0.2714	No	1.0445	0.0948	0.0793	0.0093	No	No
161365_r_at	---	---	0.9456	0.1283	0.01	0.4385	No	0.9237	0.0131	0.0004	0.867	No	No
161366_r_at	Cdh15 /// LOC672914	cadherin 15 /// similar to Muscle-cadherin precursor (M-cadherin) (Cadherin-15) (Cadherin-14)	0.9397	0.2199	0.0002	0.0805	No	0.9386	0.0921	0	0.0096	No	No
161367_f_at	Hps1	Hermansky-Pudlak syndrome 1 homolog (human)	1.0165	0.7447	0.6545	0.5236	No	0.9332	0.466	0.0808	0.222	No	No
161368_r_at	Cyp1b1	cytochrome P450, family 1, subfamily b, polypeptide 1	0.9791	0.2124	0.6042	0.0486	No	0.6508	0.702	0.832	0.543	No	No
161369_r_at	---	---	1.1347	0.1479	0.0098	0.0122	No	1.4179	0.0184	0.0055	0.0186	No	No
161370_f_at	Sdc1	syndecan 1	0.9213	0.0486	0.0683	0.6	No	1.0334	0.0669	0.09	0.0374	No	No
161371_r_at	Ptprk	protein tyrosine phosphatase, receptor type, K	1.016	0.7605	0.3188	0.9758	No	1.3421	0.209	0.367	0.0597	No	No
161372_f_at	Hnrpdl	heterogeneous nuclear ribonucleoprotein D-like	1.0069	0.7075	0.6233	0.6894	No	0.9649	0.615	0.682	0.373	No	No
161373_r_at	Fmod	fibromodulin	0.98	0.7185	0.0019	0.0419	No	0.9681	0.0079	0	0.0005	No	No
161374_f_at	Oxnad1	oxidoreductase NAD-binding domain containing 1	1.1018	0.0186	0.4176	0.3613	No	0.9984	0.967	0.0502	0.69	No	No
161375_at	---	---	1.0025	0.9721	0.4875	0.1527	No	0.9318	0.469	0.299	0.0966	No	No
161376_f_at	---	---	0.9811	0.8205	0.8619	0.7318	No	0.9198	0.507	0.529	0.707	No	No
161377_at	Emr1	EGF-like module containing, mucin-like, hormone receptor-like sequence 1	1.0224	0.7547	0.0726	0.4034	No	1.0736	0.486	0.0265	0.494	No	No
161378_r_at	Runx2	runt related transcription factor 2	1.0935	0.0281	0.587	0.0212	No	1.0187	0.164	0.0011	0.0499	No	No
161379_at	Rpo1-4	RNA polymerase 1-4	0.962	0.3952	0.0832	0.1553	No	0.9572	0.45	0.709	0.602	No	No

161380_f_at	Rab31	RAB31, member RAS oncogene family	1.1428	0.1678	0.1614	0.7905	No	1.149	0.192	0.0102	0.402	No	No
161381_r_at	Ncoa4	nuclear receptor coactivator 4	0.9971	0.9702	0.9997	0.6308	No	0.8659	0.399	0.724	0.405	No	No
161382_at	Tgfb3	transforming growth factor, beta 3	1.0428	0.604	0.0111	0.1368	No	1.1103	0.0051	0.0001	0.338	Yes	No
161383_r_at	Tead1	TEA domain family member 1	1.0464	0.134	0.002	0.0369	No	0.9881	0.763	0.0801	0.134	No	No
161384_r_at	---	Transcribed locus	1.1958	0.0735	0.3149	0.0193	No	1.1378	0.0278	0.0108	0.0249	No	No
161385_r_at	Tph1	tryptophan hydroxylase 1	1.0335	0.7666	0.2801	0.0667	No	1.3435	0.0111	0.0051	0.0023	No	No
161386_f_at	Ddx1	DEAD (Asp-Glu-Ala-Asp) box polypeptide 1	1.0265	0.7627	0.2468	0.5823	No	1.1012	0.236	0.0351	0.651	No	No
161387_i_at	Sltm	SAFB-like, transcription modulator	1.1209	0.0178	0.2666	0.0043	No	1.2798	0.0041	0.405	0.0037	No	No
161388_f_at	Tenr	testis nuclear RNA binding protein	1.0535	0.4456	0.8676	0.067	No	1.1328	0.0502	0.0058	0.0459	No	No
161389_f_at	Asnsd1	asparagine synthetase domain containing 1	1.1544	0.0324	0.0297	0.4914	No	1.0921	0.228	0.0995	0.3	No	No
161390_r_at	Pou6f1	POU domain, class 6, transcription factor 1	0.9053	0.2524	0.093	0.6981	No	0.8659	0.199	0.13	0.456	No	No
161391_r_at	Aldh18a1	aldehyde dehydrogenase 18 family, member A1	1.0435	0.3607	0.0051	0.0178	No	1.1316	0.21	0.0577	0.0334	No	No
161392_f_at	Lrrc23	leucine rich repeat containing 23	1.0756	0.2389	0.8483	0.3504	No	0.972	0.785	0.612	0.234	No	No
161393_at	---	---	0.8978	0.3013	0.5641	0.0776	No	0.8406	0.232	0.0447	0.366	No	No
161394_f_at	Hist1h2bp	histone 1, H2bp	0.9839	0.8054	0.0188	0.1947	No	1.0765	0.0449	0.0001	0.398	No	No
161395_i_at	---	---	1.1255	0.1548	0.0887	0.0091	No	1.0856	0.115	0.0248	0.0095	No	No
161396_f_at	Ube2o	ubiquitin-conjugating enzyme E2O	0.8486	0.0251	0.0027	0.1366	No	0.9292	0.165	0.0085	0.487	No	No
161397_r_at	Dhodh	dihydroorotate dehydrogenase	1.0014	0.9736	0.1174	0.7187	No	0.9883	0.795	0.053	0.855	No	No
161398_at	Dnahc8	dynein, axonemal, heavy chain 8	1.0926	0.0774	0.3596	0.4798	No	1.0442	0.514	0.121	0.629	No	No
161399_r_at	Gpld1	glycosylphosphatidylinositol specific phospholipase D1	0.9513	0.4509	0.0424	0.9953	No	1.0485	0.492	0.0436	0.157	No	No
161400_f_at	Rpn1	ribophorin I	1.0435	0.3764	0.1749	0.5603	No	1.0801	0.369	0.978	0.502	No	No
161401_f_at	Aldh3a2	aldehyde dehydrogenase family 3, subfamily A2	0.9951	0.9354	0.0267	0.2023	No	1.0402	0.777	0.0249	0.382	No	No
161402_r_at	Lrrc35	leucine rich repeat containing 35	1.1751	0.1471	0.8803	0.0502	No	1.3938	0.061	0.0829	0.0554	No	No
161403_r_at	Acrv1	acrosomal vesicle protein 1	0.9957	0.9274	0.1078	0.3556	No	0.9845	0.828	0.0063	0.953	No	No
161404_at	Bhmt /// LOC673844	betaine-homocysteine methyltransferase /// similar to Betaine--homocysteine S-methyltransferase	0.9714	0.4704	0.7052	0.2476	No	0.8805	0.0257	0.131	0.102	No	No
161405_r_at	2610019F03 Rik	RIKEN cDNA 2610019F03 gene	0.9388	0.4423	0.5262	0.2127	No	1.1334	0.0016	0.0026	0.0004	No	No
161406_at	---	Transcribed locus	1.1388	0.2201	0.3349	0.2718	No	1.1188	0.11	0.116	0.0552	No	No
161407_i_at	Inpp1	inositol polyphosphate-1-phosphatase	1.0515	0.3566	0.4604	0.1375	No	1.0224	0.564	0.0645	0.497	No	No
161408_r_at	---	---	0.9494	0.4389	0.0218	0.1939	No	1.0359	0.319	0.0033	0.0413	No	No
161409_f_at	Dntt	deoxynucleotidyltransferase, terminal	1.0007	0.969	0.0402	0.1579	No	1.113	0.274	0.169	0.353	No	No
161410_r_at	---	---	1.0687	0.0169	0.0001	0.0008	No	1.1268	0.0298	0.0044	0.0186	No	No
161411_i_at	---	---	1.1463	0.2199	0.1703	0.1421	No	1.2383	0.0468	0.0898	0.0282	No	No
161412_r_at	Cdc25a	Cell division cycle 25 homolog A (S. cerevisiae)	0.9309	0.58	0.3592	0.2382	No	0.9735	0.918	0.209	0.128	No	No
161413_f_at	Metap1	methionyl aminopeptidase 1	1.1031	0.2002	0.1339	0.0981	No	1.1845	0.0703	0.138	0.0691	No	No
161414_f_at	Smarcd2	SWI/SNF related, matrix associated, actin dependent regulator of chromatin, subfamily d, member 2	1.0484	0.1244	0.1393	0.184	No	1.0006	0.981	0.221	0.43	No	No
161415_r_at	Pvrl3	poliovirus receptor-related 3	0.9948	0.943	0.2124	0.8453	No	1.049	0.403	0.0002	0.0196	No	No
161416_r_at	Trove2	TROVE domain family, member 2	1.0597	0.4101	0.5514	0.3532	No	1.0584	0.665	0.856	0.0451	No	No
161417_r_at	Mycn	v-myc myelocytomatosis viral related oncogene, neuroblastoma derived (avian)	0.9847	0.7119	0.0216	0.1657	No	0.9107	0.307	0.0825	0.122	No	No
161418_r_at	Nr5a1	nuclear receptor subfamily 5, group A, member 1	1.2604	0.0089	0.0007	0.005	No	1.1222	0.0359	0.0004	0.0727	No	No
161419_r_at	Rps6ka1	ribosomal protein S6 kinase polypeptide 1	1.0366	0.3408	0.0344	0.3519	No	1.0366	0.613	0.0643	0.754	No	No
161420_r_at	Dpagt1	Dolichyl-phosphate (UDP-N-acetylglucosamine) acetylglucosaminephosphotransferase 1 (GlcNAc-1-P transferase)	1.0585	0.3554	0.1016	0.5105	No	0.8424	0.565	0.269	0.432	No	No
161421_r_at	Grwd1	glutamate-rich WD repeat containing 1	1.1266	0.1746	0.4614	0.1064	No	1.2203	0.0958	0.661	0.0233	No	No
161422_f_at	Ucp1	uncoupling protein 1 (mitochondrial, proton carrier)	1.5028	0.0095	0.0309	0.8112	Yes	2.3119	0.0001	0.0001	0.0106	Yes	Yes
161423_r_at	---	---	1.0438	0.3269	0.852	0.0729	No	1.0598	0.419	0.978	0.169	No	No
161424_f_at	1110049F12 Rik	RIKEN cDNA 1110049F12 gene	0.9915	0.8173	0.0976	0.6827	No	0.9565	0.509	0.0687	0.643	No	No
161425_r_at	D4Wsu132e	DNA segment, Chr 4, Wayne State University 132, expressed	1.0247	0.7146	0.7294	0.3959	No	0.9709	0.444	0.04	0.886	No	No
161426_at	D3Wsu161e	DNA segment, Chr 3, Wayne State University 161, expressed	1.0062	0.9081	0.0046	0.3296	No	0.9325	0.177	0.76	0.105	No	No
161427_f_at	E2f1	E2F transcription factor 1	1.0619	0.207	0.0292	0.4528	No	1.0977	0.36	0.206	0.203	No	No
161428_at	---	---	1.0842	0.0222	0.0008	0.0079	No	1.0375	0.385	0.103	0.177	No	No
161429_at	AA408420	expressed sequence AA408420	1.0227	0.6063	0.0156	0.8805	No	0.9995	0.93	0.0093	0.212	No	No
161430_at	Preb	prolactin regulatory element binding	1.068	0.3552	0.1989	0.4161	No	1.0693	0.166	0.0279	0.997	No	No
161431_i_at	C80913	expressed sequence C80913	0.9287	0.1869	0.0007	0.2255	No	0.8997	0.112	0.0004	0.208	No	No
161432_f_at	Sart3	squamous cell carcinoma antigen recognized by T-cells 3	0.9347	0.1578	0.0319	0.9148	No	0.9531	0.203	0.0043	0.108	No	No
161433_f_at	Piga	phosphatidylinositol glycan, class A	1.082	0.1759	0.0089	0.4098	No	0.9935	0.882	0.0106	0.484	No	No
161434_r_at	Coro2b	coronin, actin binding protein, 2B	0.9574	0.1327	0.0357	0.7901	No	1.0354	0.358	0.0421	0.521	No	No
161435_i_at	1810013D10 Rik	RIKEN cDNA 1810013D10 gene	1.0593	0.1526	0.2439	0.1157	No	1.2406	0.158	0.0733	0.0879	No	No
161436_s_at	Adarb1	adenosine deaminase, RNA-specific, B1	0.8377	0.0542	0.025	0.1059	No	0.7989	0.0045	0.0192	0.0374	Yes	No
161437_f_at	6530401N04 Rik /// LOC638977	RIKEN cDNA 6530401N04 gene /// hypothetical protein LOC638977	1.0033	0.9335	0.007	0.2547	No	0.953	0.467	0.049	0.0987	No	No
161438_r_at	---	---	0.9835	0.7621	0.6452	0.2637	No	1.2101	0.386	0.421	0.158	No	No
161439_f_at	Ap1m1	adaptor-related protein complex AP-1, mu subunit 1	1.0651	0.0442	0.0014	0.2453	No	0.9327	0.229	0.0007	0.0175	No	No
161440_r_at	Suv420h2	suppressor of variegation 4-20 homolog 2 (Drosophila)	1.06	0.467	0.0135	0.0951	No	1.1332	0.171	0.216	0.126	No	No
161441_f_at	---	---	1.1551	0.0134	0.1939	0.0615	No	1.1509	0.0025	0.0501	0.449	Yes	Yes
161442_at	Pmp22	peripheral myelin protein	1.0865	0.3918	0.069	0.3747	No	0.9989	0.967	0.0033	0.834	No	No

161443_r_at	Adfp	Adipose differentiation related protein	1.0503	0.2956	0.5701	0.1403	No	0.8971	0.705	0.26	0.207	No	No
161444_f_at	Ints5	integrator complex subunit 5	0.9292	0.4919	0.0499	0.3778	No	0.9378	0.581	0.0804	0.384	No	No
161445_at	Ankrd13c	ankyrin repeat domain 13c	1.1454	0.0908	0.0467	0.2676	No	1.0078	0.461	0.0031	0.0552	No	No
161446_r_at	Htra2	HtrA serine peptidase 2	1.0952	0.1468	0.0137	0.0818	No	1.0787	0.123	0.0173	0.0461	No	No
161447_f_at	---	---	1.0379	0.3932	0.0255	0.2818	No	1.1082	0.237	0.0456	0.123	No	No
161448_f_at	---	---	0.9856	0.8497	0.9861	0.6994	No	1.0155	0.746	0.134	0.0647	No	No
161449_f_at	Cwf19l1	CWF19-like 1, cell cycle control (S. pombe)	0.9449	0.3684	0.1334	0.3499	No	0.9324	0.213	0.716	0.148	No	No
161450_r_at	Psen1	presenilin 1	1.1874	0.0028	0.0903	0.0113	Yes	1.3632	0.0602	0.274	0.0625	No	No
161451_r_at	Clcn5	chloride channel 5	1.0277	0.5788	0.1234	0.8235	No	1.0058	0.852	0.0032	0.0613	No	No
161452_f_at	---	---	1.0844	0.1737	0.308	0.503	No	1.0948	0.473	0.445	0.51	No	No
161453_r_at	Grsf1	G-rich RNA sequence binding factor 1	1.0169	0.6044	0.0148	0.0915	No	1.1204	0.208	0.0151	0.14	No	No
161454_r_at	2610312B22 Rik /// LOC671051	RIKEN cDNA 2610312B22 gene /// similar to brain zinc finger protein	0.9615	0.438	0.0481	0.1586	No	0.9157	0.175	0.0212	0.0963	No	No
161455_r_at	Rae1	RAE1 RNA export 1 homolog (S. pombe)	1.0431	0.2891	0.2205	0.1066	No	1.4545	0.0041	0.0003	0.453	Yes	No
161456_f_at	---	---	1.0385	0.4078	0.0757	0.6111	No	0.9675	0.692	0.301	0.19	No	No
161457_at	Cdc2l1	cell division cycle 2-like 1	0.9881	0.7692	0.2131	0.9692	No	1.0103	0.865	0.428	0.253	No	No
161458_at	F7	coagulation factor VII	1.0249	0.2791	0.0011	0.1791	No	1.0301	0.336	0.0095	0.0951	No	No
161459_f_at	Prep	prolyl endopeptidase	0.8877	0.0007	0.0018	0.001	No	0.932	0.0967	0.0048	0.0089	No	No
161460_r_at	Egf	epidermal growth factor	1.0171	0.735	0.5317	0.5317	No	1.0946	0.438	0.15	0.516	No	No
161461_at	---	---	1.2288	0.0011	0.6888	0.1812	Yes	1.1824	0.0464	0.481	0.826	No	Yes
161462_r_at	2610005L07 Rik	RIKEN cDNA 2610005L07 gene	1.0602	0.6038	0.3708	0.0762	No	1.1084	0.157	0.0792	0.0173	No	No
161463_f_at	Nsg1	neuron specific gene family member 1	0.9468	0.4033	0.0303	0.9649	No	1.094	0.0705	0.0018	0.0534	No	No
161464_f_at	Stk24	serine/threonine kinase 24 (STE20 homolog, yeast)	1.0437	0.066	0.0084	0.1965	No	1.0088	0.797	0.13	0.0101	No	No
161465_r_at	---	---	0.9875	0.8145	0.7875	0.6937	No	0.9568	0.295	0.392	0.693	No	No
161466_r_at	Asb3	ankyrin repeat and SOCS box-containing protein 3	0.9907	0.8892	0.0075	0.9531	No	1.0078	0.922	0.0037	0.838	No	No
161467_f_at	Atp1b1	ATPase, Na+/K+ transporting, beta 1 polypeptide	0.987	0.7095	0.0301	0.0325	No	0.9763	0.191	0.0007	0.0197	No	No
161468_f_at	Hand1	Heart and neural crest derivatives expressed transcript 1	1.0446	0.0882	0.0058	0.0042	No	0.8892	0.0491	0.0199	0.581	No	No
161469_r_at	Acads	acyl-Coenzyme A dehydrogenase, short chain	1.0574	0.341	0.7642	0.1742	No	1.0132	0.811	0.0948	0.965	No	No
161470_r_at	Mtf2	Metal response element binding transcription factor 2	0.9575	0.3447	0.4197	0.87	No	1.0152	0.793	0.768	0.876	No	No
161471_f_at	Rcor1	REST corepressor 1	1.0059	0.9208	0.0473	0.7947	No	1.058	0.341	0.006	0.312	No	No
161472_r_at	Lman2	lectin, mannose-binding 2	1.0238	0.7602	0.1484	0.1673	No	1.0832	0.386	0.12	0.122	No	No
161473_f_at	Gng11	guanine nucleotide binding protein (G protein), gamma 11	0.9992	0.9849	0.0074	0.5475	No	1.1402	0.355	0.368	0.253	No	No
161474_r_at	Dpep3	dipeptidase 3	1.0094	0.8692	0.0174	0.8422	No	1.0444	0.454	0.026	0.134	No	No
161475_f_at	Cox8c	cytochrome c oxidase, subunit VIIIc	1.0249	0.7435	0.403	0.4094	No	1.093	0.103	0.0065	0.161	No	No
161476_at	Psap	prosaposin	1.0282	0.462	0.0544	0.1014	No	0.9036	0.151	0.66	0.862	No	No
161477_r_at	---	---	1.039	0.4399	0.4878	0.3621	No	1.0568	0.242	0.0241	0.0253	No	No
161478_at	Mthfd2	methylenetetrahydrofolate dehydrogenase (NAD+ dependent), methenyltetrahydrofolate cyclohydrolase	1.0269	0.5272	0.8328	0.0231	No	1.0739	0.0637	0.152	0.01	No	No
161479_f_at	Ctf1	cardiotrophin 1	0.9185	0.2926	0.0347	0.8287	No	0.934	0.268	0.0107	0.619	No	No
161480_i_at	---	---	1.0702	0.2369	0.0173	0.535	No	1.0338	0.438	0.0118	0.462	No	No
161481_f_at	Bnip1	BCL2/adenovirus E1B interacting protein 1, NIP1	1.207	0.0618	0.3418	0.1206	No	1.1946	0.0543	0.0683	0.0513	No	No
161482_f_at	LOC673890	similar to peripherin 1	0.977	0.8487	0.0212	0.8607	No	1.024	0.467	0	0.0922	No	No
161483_r_at	---	---	1.0967	0.1839	0.328	0.0796	No	1.1815	0.0874	0.0402	0.0536	No	No
161484_r_at	Trim47	Tripartite motif protein 47	1.0234	0.5406	0.0142	0.4166	No	1.0969	0.306	0.13	0.113	No	No
161485_r_at	---	---	1.1404	0.0946	0.1058	0.0216	No	1.0748	0.139	0.014	0.0145	No	No
161486_f_at	Igh-VJ558 /// LOC238447 /// LOC544903 /// LOC544907	immunoglobulin heavy chain (J558 family) /// similar to immunoglobulin heavy chain variable region /// similar to immunoglobulin mu-chain /// similar to anti-poly(dC) monoclonal antibody heavy chain	1.0002	0.9982	0.049	0.9543	No	0.9395	0.31	0.0073	0.586	No	No
161487_f_at	---	---	1.0109	0.7884	0.1209	0.2221	No	1.0425	0.139	0.246	0.0023	No	No
161488_r_at	Mrpl36	mitochondrial ribosomal protein L36	0.9607	0.665	0.658	0.9366	No	0.9844	0.754	0.45	0.575	No	No
161489_r_at	Tmem109	transmembrane protein 109	1.088	0.5118	0.0918	0.774	No	1.1228	0.584	0.1	0.718	No	No
161490_at	Cox5a	cytochrome c oxidase, subunit Va	0.9736	0.6366	0.1704	0.2575	No	0.9389	0.319	0.13	0.0464	No	No
161491_r_at	Fbxo3	F-box only protein 3	1.0835	0.3812	0.1802	0.3922	No	1.0968	0.307	0.273	0.528	No	No
161492_i_at	Mgat1	mannoside acetylglucosaminyltransferase 1	1.0707	0.2363	0.0076	0.0814	No	1.0678	0.413	0.0083	0.217	No	No
161493_at	---	---	1.0929	0.1891	0.0948	0.208	No	0.9823	0.843	0.0404	0.683	No	No
161494_f_at	Atp5d	ATP synthase, H+ transporting, mitochondrial F1 complex, delta subunit	1.1049	0.0065	0.0008	0.0991	Yes	0.9827	0.803	0.105	0.323	No	No
161495_r_at	Ckmt1	creatine kinase, mitochondrial 1, ubiquitous	1.3235	0.1109	0.6193	0.088	No	1.1375	0.136	0.224	0.051	No	No
161496_r_at	Krt1-17	keratin complex 1, acidic, gene 17	1.1233	0.0096	0.0011	0.0214	Yes	1.1119	0.125	0.0028	0.0803	No	No
161497_f_at	Itga7	integrin alpha 7	0.96	0.4501	0.1355	0.5302	No	1.0138	0.769	0.0184	0.729	No	No
161498_at	Rpl23	ribosomal protein L23	1.0285	0.1562	0.0005	0.5087	No	1.0027	0.791	0.0197	0.0752	No	No
161499_f_at	---	---	1.0367	0.6668	0.5284	0.8972	No	1.0765	0.306	0.18	0.672	No	No
161500_i_at	Bad	Bcl-associated death promoter	1.1291	0.1536	0.1086	0.1536	No	1.085	0.151	0.0078	0.071	No	No
161501_at	---	---	1.1048	0.0828	0.0463	0.0573	No	1.1065	0.026	0.0263	0.754	No	No
161502_r_at	Smyd5	SET and MYND domain containing 5	1.0159	0.7585	0.1239	0.0534	No	1.0761	0.126	0.0492	0.0151	No	No
161503_f_at	---	---	1.0246	0.3607	0.1337	0.0722	No	1.0636	0.155	0.0233	0.101	No	No
161504_i_at	D10Ertd214e	DNA segment, Chr 10, ERATO Doi 214, expressed	1.3581	0.0263	0.0374	0.0271	No	1.327	0.0169	0.0396	0.0228	No	No

161505_i_at	---	Transcribed locus	1.0299	0.815	0.0946	0.7768	No	1.1157	0.235	0.0125	0.265	No	No
161506_r_at	Ccs	copper chaperone for superoxide dismutase	1.0454	0.3834	0.1041	0.5593	No	1.0532	0.314	0.781	0.11	No	No
161507_f_at	Ndufaf1	NADH dehydrogenase (ubiquinone) 1 alpha subcomplex, assembly factor 1	0.9995	0.9879	0.025	0.896	No	1.0175	0.9	0.0026	0.257	No	No
161508_at	Sub1 /// LOC676541	SUB1 homolog (S. cerevisia /// similar to Activated RNA polymerase II transcriptional coactivator p15 precursor (SUB1 homolog) (Positive cofactor 4) (PC4) (p14) (Single-stranded DNA-binding protein p9)	0.9737	0.6458	0.6007	0.2776	No	0.9343	0.377	0.109	0.232	No	No
161509_at	Mmp2	matrix metallopeptidase 2	0.9919	0.716	0.1793	0.8523	No	1.0336	0.384	0.0108	0.562	No	No
161510_f_at	---	---	0.9711	0.25	0.0189	0.7637	No	0.9706	0.13	0.124	0.144	No	No
161511_f_at	LOC677168	hypothetical protein LOC677168	1.0626	0.1311	0.0421	0.1425	No	0.9325	0.527	0.0011	0.135	No	No
161512_r_at	Dgcr6	DiGeorge syndrome critical region gene 6	0.9824	0.6583	0.0439	0.7457	No	0.9899	0.808	0.247	0.368	No	No
161513_r_at	Chmp1b	chromatin modifying protein 1B	1.0313	0.4962	0.2066	0.8111	No	1.0891	0.167	0.218	0.317	No	No
161514_at	---	---	1.071	0.1187	0.0681	0.1597	No	1.0725	0.441	0.0601	0.111	No	No
161515_i_at	Timp2	tissue inhibitor of metalloproteinase 2	1.3368	0.0108	0.0032	0.0108	No	1.2933	0.0042	0.0018	0.005	No	No
161516_r_at	Vapa	vesicle-associated membrane protein, associated protein A	1.1899	0.0455	0.0035	0.1787	No	1.2797	0.166	0.0139	0.33	No	No
161517_at	---	---	1.0884	0.2151	0.0392	0.0539	No	1.1376	0.0871	0.01	0.228	No	No
161518_r_at	Angptl4	angiopoietin-like 4	0.95	0.4876	0.0696	0.8949	No	0.9255	0.461	0.4	0.227	No	No
161519_f_at	---	---	1.1543	0.0265	0.0162	0.0269	No	1.1371	0.0266	0.0057	0.0283	No	No
161520_at	2610029G23 Rik	RIKEN cDNA 2610029G23 gene	0.9215	0.3251	0.0906	0.5413	No	0.6861	0.254	0.178	0.209	No	No
161521_at	Ppp1r12c	protein phosphatase 1, regulatory (inhibitor) subunit 12C	1.0263	0.5806	0.2244	0.908	No	1.035	0.611	0.789	0.818	No	No
161522_i_at	Cst3	cystatin C	0.9187	0.0128	0.0498	0.6226	No	0.8392	0.0654	0.388	0.179	No	No
161523_r_at	Calm2	calmodulin 2	1.0963	0.1755	0.006	0.0827	No	1.0548	0.455	0.0147	0.126	No	No
161524_r_at	Gpi1 /// LOC669429 /// LOC676596	glucose phosphate isomerase 1 /// hypothetical protein LOC669429 /// similar to Glucose-6-phosphate isomerase (GPI) (Phosphoglucose isomerase) (PGI) (Phosphohexose isomerase) (PHI) (Neuroleukin) (NLK)	1.1593	0.1152	0.0348	0.0655	No	1.1052	0.0805	0.0087	0.0815	No	No
161525_f_at	Rom1	rod outer segment membrane protein 1	0.9731	0.711	0.1873	0.4788	No	0.8963	0.671	0.0877	0.581	No	No
161526_r_at	---	---	0.9968	0.9547	0.2821	0.586	No	1.108	0.185	0.931	0.134	No	No
161527_r_at	Ccni	cyclin I	1.0731	0.1407	0.3286	0.2066	No	1.8432	0.0311	0.0365	0.0248	No	No
161528_r_at	Pold1	polymerase (DNA directed), delta 1, catalytic subunit	1.2899	0.0024	0.002	0.0023	No	1.2737	0.0213	0.0116	0.0081	No	No
161529_r_at	Cldn11	claudin 11	1.3829	0.0307	0.0119	0.0183	No	1.2347	0.0818	0.0082	0.1	No	No
161530_r_at	Sema4a	sema domain, immunoglobulin domain (Ig), transmembrane domain (TM) and short cytoplasmic domain, (semaphorin) 4A	1.5555	0.0008	0.4196	0.0007	No	1.39	0.0088	0.438	0.0076	No	No
161531_r_at	Egr2	Early growth response 2	1.0693	0.2716	0.6047	0.095	No	1.691	0.0191	0.0367	0.0241	No	No
161532_f_at	2610304G08 Rik	RIKEN cDNA 2610304G08 gene	0.9888	0.7491	0.9208	0.1623	No	0.9549	0.541	0.398	0.196	No	No
161533_at	---	---	1.0257	0.747	0.9855	0.3427	No	1.1073	0.315	0.377	0.156	No	No
161534_f_at	1110038D17 Rik	RIKEN cDNA 1110038D17 gene	0.9176	0.2225	0.1118	0.76	No	0.9711	0.535	0.0257	0.519	No	No
161535_at	---	---	1.0183	0.5401	0.0089	0.0854	No	0.9652	0.314	0.132	0.946	No	No
161536_r_at	Dusp6	dual specificity phosphatase 6	1.0032	0.9684	0.0547	0.0338	No	1.0215	0.1	0.001	0.0017	No	No
161537_f_at	Tpp2	tripeptidyl peptidase II	0.9862	0.6682	0.0055	0.0282	No	0.9202	0.341	0.0073	0.896	No	No
161538_r_at	Tyrp1	tyrosinase-related protein 1	0.9976	0.9588	0.8153	0.315	No	1.1353	0.26	0.167	0.0162	No	No
161539_f_at	Tchh	trichohyalin	0.9398	0.1565	0.2672	0.5418	No	0.9563	0.879	0.169	0.468	No	No
161540_r_at	Tmem77	transmembrane protein 77	0.9751	0.515	0.0239	0.041	No	0.3067	0.993	0.232	0.187	No	No
161541_r_at	Tchh	trichohyalin	0.9745	0.7481	0.0723	0.7268	No	0.9327	0.235	0.0089	0.976	No	No
161542_r_at	Tmem129	transmembrane protein 129	1.0807	0.2457	0.0276	0.2216	No	1.0913	0.301	0.0101	0.0375	No	No
161543_at	---	---	1.033	0.3821	0.3333	0.0124	No	1.1193	0.133	0.639	0.244	No	No
161544_r_at	Csf2rb2	colony stimulating factor 2 receptor, beta 2, low-affinity (granulocyte-macrophage)	0.9788	0.6592	0.0356	0.0853	No	1.3516	0.0937	0.0712	0.0648	No	No
161545_r_at	Myh4	myosin, heavy polypeptide 4, skeletal muscle	0.9725	0.373	0.7368	0.0198	No	0.9672	0.508	0.722	0.395	No	No
161546_r_at	---	---	1.0084	0.8132	0.0457	0.5367	No	0.623	0.0037	0.0026	0.0016	No	No
161547_f_at	---	---	0.9781	0.6587	0.0037	0.7508	No	0.9613	0.192	0.0011	0.066	No	No
161548_r_at	Ryk	receptor-like tyrosine kinase	1.1204	0.1104	0.0841	0.1774	No	1.0654	0.291	0.0351	0.214	No	No
161549_f_at	C1qtnf1	C1q and tumor necrosis factor related protein 1	1.1816	0.031	0.1915	0.0236	No	1.0557	0.486	0.0034	0.117	No	No
161550_r_at	Vcp /// LOC675857	valosin containing protein /// similar to Transitional endoplasmic reticulum ATPase (TER ATPase) (15S Mg(2+)-ATPase p97 subunit) (Valosin-containing protein) (VCP)	0.9934	0.8665	0.7216	0.0814	No	0.663	0.0396	0.0047	0.0135	No	No
161551_f_at	Riok3	RIO kinase 3 (yeast)	1.1507	0.0887	0.0017	0.1546	No	1.037	0.718	0.0027	0.894	No	No
161552_i_at	Xpa	xeroderma pigmentosum, complementation group A	0.7945	0.1375	0.0476	0.1054	No	0.5678	0.247	0.0698	0.198	No	No
161553_i_at	A030009H04 Rik	RIKEN cDNA A030009H04 gene	1.1683	0.1223	0.997	0.0889	No	1.0902	0.397	0.858	0.158	No	No
161554_r_at	Coasy	Coenzyme A synthase	0.926	0.1498	0.0326	0.5525	No	0.925	0.199	0.0302	0.422	No	No
161555_f_at	Slc2a8	solute carrier family 2, (facilitated glucose transporter), member 8	0.9803	0.8349	0.2397	0.8881	No	0.9637	0.364	0.0138	0.542	No	No
161556_at	Usp52	ubiquitin specific peptidase 52	0.9765	0.5965	0.3378	0.8974	No	0.9317	0.137	0.0997	0.0437	No	No
161557_at	Mnat1	menage a trois 1	1.0863	0.0996	0.2307	0.3148	No	1.0838	0.0336	0.0034	0.582	No	No
161558_f_at	---	---	1.0305	0.4265	0.2135	0.1313	No	1.0862	0.249	0.948	0.175	No	No
161559_i_at	---	---	0.8932	0.1273	0.0057	0.4453	No	0.9087	0.0583	0.0049	0.276	No	No
161560_f_at	Xrcc1	X-ray repair complementing defective repair in Chinese hamster cells 1	1.0018	0.9614	0.007	0.9816	No	1.02	0.467	0.0431	0.151	No	No
161561_r_at	Surf5	Surfeit gene 5	0.9074	0.1963	0.001	0.3795	No	0.9058	0.195	0.0002	0.0286	No	No
161562_f_at	2210018M03 Rik	RIKEN cDNA 2210018M03 gene	0.9778	0.1008	0.0343	0.0533	No	1.0448	0.585	0.0418	0.76	No	No
161563_r_at	---	---	0.904	0.1626	0.0959	0.096	No	0.9989	0.952	0.401	0.915	No	No
161564_r_at	X99384	cDNA sequence X99384	1.018	0.7618	0.0413	0.0991	No	0.9463	0.362	0.449	0.661	No	No

161565_r_at	---	---	1.0007	0.9888	0.8282	0.5366	No	1.0638	0.383	0.311	0.937	No	No
161566_r_at	Asgr1	asialoglycoprotein receptor 1	0.8693	0.0132	0.0025	0.8328	No	0.9323	0.294	0.0058	0.328	No	No
161567_r_at	Tob1	transducer of ErbB-2.1	1.09	0.0465	0.0488	0.0818	No	0.9883	0.895	0.0292	0.438	No	No
161568_f_at	Map3k4	mitogen activated protein kinase kinase kinase 4	1.0454	0.1581	0.0756	0.0486	No	0.9694	0.583	0.947	0.0428	No	No
161569_f_at	Ckm	creatine kinase, muscle	0.761	0.0078	0.16	0.0018	No	0.5741	0.001	0.0021	0.0004	No	No
161570_r_at	4921531G14 Rik	RIKEN cDNA 4921531G14 gene	1.0278	0.3054	0.035	0.6392	No	0.9949	0.973	0.469	0.591	No	No
161571_f_at	C1r /// LOC667277 /// LOC677405	complement component 1, r subcomponent /// similar to Complement C1r-B subcomponent precursor (Complement component 1, r-B subcomponent) /// similar to complement component 1, r subcomponent	1.2069	0.1461	0.0933	0.1908	No	1.1799	0.296	0.0424	0.681	No	No
161572_r_at	---	---	1.0365	0.5887	0.7239	0.6234	No	1.027	0.696	0.637	0.298	No	No
161573_at	Slc4a7	solute carrier family 4, sodium bicarbonate cotransporter, member 7	0.9733	0.5129	0.0297	0.6173	No	1.0587	0.193	0.35	0.0133	No	No
161574_r_at	Atp2c1	ATPase, Ca++-sequestering	1.059	0.3394	0.7837	0.1624	No	1.218	0.46	0.49	0.802	No	No
161575_f_at	Mapk10	mitogen activated protein kinase 10	0.9466	0.2919	0.4492	0.1337	No	1.0176	0.644	0.0139	0.124	No	No
161576_f_at	Trafd1	TRAF type zinc finger domain containing 1	1.001	0.9863	0.0344	0.4378	No	1.0575	0.0492	0.003	0.15	No	No
161577_f_at	Ccdc96	coiled-coil domain containing 96	1.0219	0.5745	0.4659	0.3279	No	1.0501	0.372	0.0472	0.0341	No	No
161578_r_at	---	---	0.868	0.0091	0.0002	0.0161	Yes	0.9361	0.0794	0.0009	0.0111	No	No
161579_r_at	Sfpq	Splicing factor proline/glutamine rich (polypyrimidine tract binding protein associated)	1.004	0.8902	0.0001	0.5491	No	1.0339	0.342	0	0.16	No	No
161580_f_at	6330412F12 Rik	RIKEN cDNA 6330412F12 gene	0.9503	0.4553	0.2006	0.4727	No	0.9629	0.565	0.0002	0.398	No	No
161581_r_at	Mizf	MBD2-interacting zinc finger	1.0933	0.1448	0.454	0.0362	No	1.0018	0.987	0.148	0.321	No	No
161582_r_at	Wbp11	WW domain binding protein 11	1.1643	0.1025	0.1591	0.2719	No	1.6198	0.0136	0.0407	0.0135	No	No
161583_at	Mapk1	mitogen activated protein kinase 1	0.9314	0.287	0.5293	0.1855	No	0.9291	0.998	0.0931	0.305	No	No
161584_r_at	Elf1	E74-like factor 1	1.0096	0.7968	0.0793	0.2723	No	0.6346	0.493	0.0125	0.941	No	No
161585_at	Gp5	glycoprotein 5 (platelet)	0.9902	0.5776	0.0847	0.0559	No	1.0391	0.825	0.345	0.378	No	No
161586_f_at	Cenpe	centromere protein E	0.9913	0.7668	0.8244	0.5241	No	1.0567	0.116	0.213	0.797	No	No
161587_at	Hrsp12	heat-responsive protein 12	1.1236	0.0967	0.2445	0.0384	No	1.1774	0.289	0.397	0.0977	No	No
161588_r_at	Dhh	desert hedgehog	0.9096	0.1984	0.0016	0.7645	No	0.9612	0.181	0.0001	0.204	No	No
161589_at	Ard1	N-acetyltransferase ARD1 homolog (S. cerevisiae)	1.1208	0.1955	0.054	0.2742	No	1.0329	0.698	0.0518	0.951	No	No
161590_r_at	Plf /// Plf2 /// Mrpplf3	proliferin /// proliferin 2 /// mitogen regulated protein, proliferin 3	1.0565	0.4688	0.0506	0.1112	No	1.2083	0.168	0.0383	0.0677	No	No
161591_r_at	Gal	galanin	1.1228	0.2096	0.0399	0.0856	No	0.9991	0.979	0.446	0.495	No	No
161592_at	Tuba4	Tubulin, alpha 4	1.1483	0.0701	0.0101	0.1139	No	1.2339	0.203	0.0183	0.0564	No	No
161593_r_at	Ap1g1	adaptor protein complex AP-1, gamma 1 subunit	0.9895	0.804	0.0298	0.1242	No	1.008	0.47	0.105	0.0087	No	No
161594_f_at	Galm	galactose mutarotase	1.1015	0.0315	0.0078	0.0056	No	1.0218	0.312	0.0013	0.0188	No	No
161595_at	---	---	1.0736	0.3858	0.9942	0.0738	No	1.0792	0.114	0.0208	0.0337	No	No
161596_f_at	Akap8	A kinase (PRKA) anchor protein 8	0.9944	0.9071	0.0852	0.0892	No	0.9959	0.985	0.0383	0.0341	No	No
161597_r_at	Mrpl49	mitochondrial ribosomal protein L49	0.9637	0.5346	0.2857	0.6685	No	1.0643	0.449	0.164	0.226	No	No
161598_at	Gdnf	glial cell line derived neurotrophic factor	0.9892	0.7723	0.7575	0.277	No	1.0586	0.318	0.104	0.0982	No	No
161599_i_at	Bop1	block of proliferation 1	0.9471	0.5204	0.4079	0.4211	No	0.9321	0.497	0.58	0.596	No	No
161600_r_at	---	---	0.7642	0.0444	0.6087	0.1901	No	0.8327	0.041	0.788	0.124	No	No
161601_i_at	Dnmt1	DNA methyltransferase (cytosine-5) 1	0.8161	0.0096	0	0.0952	Yes	0.9354	0.171	0	0.263	No	No
161602_at	Gbl	G protein beta subunit-like	1.0938	0.0439	0.0799	0.8942	No	1.1259	0.185	0.894	0.448	No	No
161603_r_at	Epb4.1l4a	erythrocyte protein band 4.1-like 4a	1.1698	0.0425	0.0033	0.8524	No	1.1322	0.0571	0.0071	0.219	No	No
161604_r_at	Kifap3	Kinesin-associated protein 3	1.0244	0.7926	0.8896	0.3489	No	1.057	0.73	0.796	0.241	No	No
161605_f_at	Zfp37	Zinc finger protein 37	1.0992	0.3312	0.0101	0.0803	No	1.1481	0.172	0.0046	0.352	No	No
161606_f_at	Tigd5	tigger transposable element derived 5	1.001	0.9867	0.0911	0.5498	No	1.0451	0.144	0.0009	0.192	No	No
161607_r_at	Gjb6	gap junction membrane channel protein beta 6	0.9727	0.5038	0.5673	0.8034	No	0.7791	0.268	0.692	0.218	No	No
161608_r_at	Hyal2	hyaluronoglucosaminidase 2	0.8918	0.2996	0.03	0.4198	No	1.0298	0.777	0.591	0.302	No	No
161609_at	Rgs16	regulator of G-protein signaling 16	0.9916	0.8835	0.0517	0.1481	No	1.0012	0.952	0.396	0.286	No	No
161610_at	Ndrg2	N-myc downstream regulated gene 2	0.8138	0.0004	0.0015	0.0002	No	0.7217	0.0018	0.436	0.0045	No	No
161611_f_at	Emid1	EMI domain containing 1	1.0014	0.9812	0.0568	0.7602	No	0.9992	0.906	0.0145	0.41	No	No
161612_f_at	Tubb3 /// LOC545887 /// LOC632223	tubulin, beta 3 /// similar to tubulin, beta 3 /// similar to tubulin, beta 3	0.9814	0.6122	0.0354	0.56	No	1.0251	0.158	0.0078	0.345	No	No
161613_at	Chmp7	CHMP family, member 7	0.9617	0.3927	0.0004	0.3166	No	0.9525	0.312	0.0012	0.46	No	No
161614_r_at	Tubb2a	tubulin, beta 2a	1.0463	0.4366	0.526	0.8543	No	1.0954	0.088	0.0884	0.452	No	No
161615_f_at	---	---	1.0418	0.3804	0.63	0.3864	No	1.056	0.265	0.0409	0.88	No	No
161616_f_at	Rprm	reprimo, TP53 dependent G2 arrest mediator candidate	0.9504	0.2197	0.9987	0.0451	No	1.0475	0.517	0.0238	0.0923	No	No
161617_f_at	---	---	0.9777	0.7285	0.1615	0.1135	No	1.0721	0.58	0.055	0.664	No	No
161618_r_at	---	---	1.0193	0.8045	0.2252	0.5498	No	1.0781	0.347	0.547	0.305	No	No
161619_f_at	Mapk8ip1	mitogen activated protein kinase 8 interacting protein 1	0.9975	0.965	0.0144	0.1673	No	0.9347	0.299	0.0226	0.202	No	No
161620_f_at	Cwf19l1	CWF19-like 1, cell cycle control (S. pombe)	0.9466	0.2771	0.1051	0.4269	No	0.948	0.659	0.0474	0.949	No	No
161621_r_at	Cggbp1	CGG triplet repeat binding protein 1	1.0328	0.4272	0.1553	0.0199	No	1.1273	0.0307	0.018	0.223	No	No
161622_f_at	---	---	1.0152	0.7319	0.0066	0.6495	No	1.2082	0.308	0.0223	0.999	No	No
161623_at	Acadvl	acyl-Coenzyme A dehydrogenase, very long chain	1.0047	0.9669	0.1049	0.789	No	1.0105	0.911	0.0375	0.511	No	No
161624_r_at	---	---	0.9831	0.527	0.1373	0.0352	No	0.905	0.155	0.0326	0.0976	No	No
161625_r_at	Lrp10	low-density lipoprotein receptor-related protein 10	1.0964	0.005	0.0043	0.0043	No	1.1145	0.0074	0.0852	0.0222	Yes	No
161626_f_at	Plg	plasminogen	1.143	0.0376	0.0021	0.5598	No	1.1381	0.179	0.0133	0.173	No	No
161627_r_at	Prkd2	Protein kinase D2	0.9855	0.7466	0.2293	0.3766	No	1.0925	0.149	0.136	0.102	No	No
161628_r_at	Fbln1	fibulin 1	0.9773	0.457	0.0043	0.1493	No	0.9445	0.073	0.0293	0.519	No	No

161629_i_at	Afp	alpha fetoprotein	1.1051	0.452	0.2235	0.138	No	1.0847	0.426	0.15	0.176	No	No
161630_i_at	---	---	1.1254	0.2928	0.5406	0.1936	No	1.1261	0.34	0.587	0.256	No	No
161631_f_at	Cstf1	Cleavage stimulation factor, 3' pre-RNA, subunit 1	1.0153	0.6851	0.5755	0.7304	No	1.0852	0.302	0.111	0.26	No	No
161632_r_at	Phf7	PHD finger protein 7	1.0844	0.0949	0.0354	0.7424	No	0.8781	0.0799	0.0013	0.701	No	No
161633_r_at	---	---	0.9309	0.1532	0.5016	0.0763	No	0.911	0.0922	0.0364	0.831	No	No
161634_r_at	Dll1	delta-like 1 (Drosophila)	1.0176	0.5328	0.0051	0.7106	No	1.0694	0.416	0.0082	0.273	No	No
161635_f_at	1110008H02 Rik	RIKEN cDNA 1110008H02 gene	1.0456	0.6165	0.5897	0.6881	No	1.1544	0.195	0.131	0.414	No	No
161636_r_at	Plk3	polo-like kinase 3 (Drosophila)	1.051	0.238	0.0542	0.0668	No	1.0158	0.652	0.023	0.737	No	No
161637_f_at	Egfbp2 /// Klk1 /// Klk1b26 /// Klk1b5	epidermal growth factor binding protein type B /// kallikrein 1 /// kallikrein 1-related petidase b26 /// kallikrein 1-related peptidase b5	0.9368	0.4583	0.0843	0.7401	No	1.035	0.913	0.609	0.144	No	No
161638_f_at	Pick1	protein interacting with C kinase 1	0.9671	0.6796	0.4505	0.657	No	0.9872	0.71	0.0118	0.286	No	No
161639_f_at	Gast	gastrin	0.8632	0.0654	0.0104	0.8231	No	0.8576	0.0156	0.0522	0.0803	No	No
161640_at	AW491445	expressed sequence AW491445	0.9645	0.309	0.8284	0.7502	No	0.9519	0.214	0.387	0.745	No	No
161641_at	---	---	0.9937	0.9248	0.3679	0.5104	No	1.1508	0.0912	0.208	0.186	No	No
161642_f_at	Reg3a	regenerating islet-derived 3 alpha	1.0929	0.0514	0.0655	0.4316	No	1.1454	0.254	0.389	0.416	No	No
161643_i_at	---	---	0.986	0.5125	0.0889	0.0309	No	1.0395	0.557	0.118	0.141	No	No
161644_f_at	Gamt	guanidinoacetate methyltransferase	1.0833	0.0184	0.0668	0.0708	No	1.0184	0.51	0.0359	0.277	No	No
161645_r_at	Atf3	activating transcription factor 3	0.9752	0.8138	0.082	0.6955	No	0.998	0.992	0.018	0.858	No	No
161646_r_at	Klhdc2	kelch domain containing 2	1.0002	0.9908	0.0689	0.0268	No	1.1634	0.593	0.524	0.0383	No	No
161647_f_at	Krt2-4	keratin complex 2, basic, gene 4	0.9974	0.9413	0.0657	0.2494	No	1.0743	0.418	0.462	0.686	No	No
161648_at	Crry	complement receptor related protein	0.9172	0.3364	0.0517	0.6185	No	0.9553	0.345	0.0053	0.951	No	No
161649_f_at	---	---	0.9916	0.9047	0.0941	0.4867	No	1.0162	0.823	0.412	0.582	No	No
161650_at	Slpi	secretory leukocyte peptidase inhibitor	1.0043	0.9122	0.0079	0.3444	No	0.9035	0.0009	0.0002	0.0036	No	No
161651_f_at	---	---	1.0432	0.3391	0.0896	0.6172	No	1.0374	0.272	0.0002	0.153	No	No
161652_r_at	Idh3a	isocitrate dehydrogenase 3 (NAD+) alpha	0.9905	0.8335	0.2641	0.5315	No	1.3953	0.0222	0.0018	0.0258	No	No
161653_f_at	Nanog	Nanog homeobox	1.0784	0.1994	0.6383	0.2185	No	1.0292	0.696	0.0281	0.877	No	No
161654_r_at	---	PREDICTED: Mus musculus similar to 40S ribosomal protein S4, X isoform, transcript variant 1 (LOC225134), mRNA	1.0443	0.7046	0.0239	0.8511	No	1.0358	0.819	0.0088	0.445	No	No
161655_at	Dad1	defender against cell death 1	1.0488	0.3061	0.7159	0.7927	No	1.0908	0.202	0.0637	0.768	No	No
161656_r_at	Proc	protein C	1.094	0.0723	0.012	0.0382	No	1.0159	0.814	0.0228	0.024	No	No
161657_f_at	Rpl10 /// LOC234703 /// LOC434434 /// LOC625219 /// LOC638133 /// LOC668946 /// LOC670252 /// LOC675230	ribosomal protein 10 /// similar to ribosomal protein L10 /// similar to ribosomal protein L10 /// similar to ribosomal protein L10 /// similar to 60S ribosomal protein L10 (QM protein) (Tumor suppressor QM) (Laminin receptor homolog) /// similar to 60S ribosomal protein L10 (QM protein) (Tumor suppressor QM) (Laminin receptor homolog) /// similar to 60S ribosomal protein L10 (QM protein) (Tumor suppressor QM) (Laminin receptor homolog)	0.9747	0.5776	0.0873	0.2335	No	0.938	0.136	0.0043	0.0945	No	No
161658_at	Map3k11	mitogen activated protein kinase kinase kinase 11	0.9966	0.9419	0.0122	0.7272	No	0.9586	0.103	0.0009	0.0765	No	No
161659_f_at	Acp1 /// LOC631286	acid phosphatase 1, soluble /// similar to acid phosphatase 1, soluble	1.0119	0.8922	0.2682	0.2535	No	1.0083	0.869	0.104	0.651	No	No
161660_r_at	---	---	1.2408	0.0128	0.0325	0.0529	No	1.6272	0.0543	0.0747	0.807	No	No
161661_i_at	Itpr1	Inositol 1,4,5-triphosphate receptor 1	0.9855	0.9288	0.3212	0.5554	No	1.0609	0.692	0.05	0.648	No	No
161662_f_at	Sap30bp /// LOC639905	SAP30 binding protein /// similar to transcriptional regulator protein	1.0382	0.629	0.2839	0.6038	No	0.9851	0.83	0.224	0.219	No	No
161663_f_at	Dnajc19 /// LOC624405	DnaJ (Hsp40) homolog, subfamily C, member 19 /// similar to translocase of the inner mitochondrial membrane 14 isoform 1	0.9928	0.8995	0.7398	0.5129	No	1.0496	0.455	0.732	0.387	No	No
161664_at	Mrpl50	mitochondrial ribosomal protein L50	1.1474	0.0061	0.0002	0.0227	Yes	1.1391	0.207	0.0018	0.497	No	No
161665_at	Mark1	MAP/microtubule affinity-regulating kinase 1	0.9853	0.774	0.0869	0.9596	No	0.9479	0.308	0.0137	0.158	No	No
161666_f_at	Gadd45b	growth arrest and DNA-damage-inducible 45 beta	0.9752	0.6833	0.0075	0.1138	No	0.973	0.851	0.0016	0.475	No	No
161667_r_at	Tmem59	transmembrane protein 59	0.9654	0.4245	0.0277	0.426	No	0.9753	0.752	0.663	0.514	No	No
161668_f_at	Por	P450 (cytochrome) oxidoreductase	0.9815	0.6662	0.017	0.9144	No	0.9458	0.116	0.234	0.427	No	No
161669_r_at	Naca /// LOC628277 /// LOC633817 /// LOC669373	nascent polypeptide-associated complex alpha polypeptide /// similar to nascent polypeptide-associated complex alpha polypeptide /// similar to nascent polypeptide-associated complex alpha polypeptide /// similar to nascent polypeptide-associated complex alpha polypeptide	1.0243	0.2062	0.0202	0.0622	No	0.7972	0.598	0.482	0.461	No	No
161670_f_at	Hoxa11os	homeo box A11, opposite strand transcript	0.9752	0.7276	0.1285	0.6822	No	0.9973	0.902	0.279	0.686	No	No
161671_at	---	---	0.9658	0.5198	0.1671	0.6781	No	1.1303	0.172	0.105	0.217	No	No
161672_at	Map3k7	mitogen activated protein kinase kinase kinase 7	1.0092	0.791	0.1447	0.8768	No	0.9556	0.405	0.177	0.0729	No	No
161673_r_at	---	---	1.0078	0.8999	0.3782	0.0919	No	1.0506	0.77	0.677	0.215	No	No
161674_i_at	---	---	1.0666	0.2455	0.0876	0.1948	No	1.0682	0.277	0.323	0.772	No	No
161675_f_at	---	---	0.9784	0.5931	0.0019	0.5904	No	0.9435	0.292	0.0005	0.205	No	No
161676_at	Sgol1	shugoshin-like 1 (S. pombe)	0.9746	0.6596	0.0406	0.7265	No	0.9095	0.0304	0.0305	0.0543	No	No
161677_r_at	---	---	0.9282	0.2809	0.0043	0.737	No	0.9604	0.273	0.001	0.894	No	No
161678_at	Inpp5d	inositol polyphosphate-5-phosphatase D	1.1097	0.0193	0.5954	0.0333	No	1.1861	0.0016	0.0301	0.013	Yes	Yes
161679_r_at	---	---	1.1257	0.0361	0.156	0.017	No	1.1514	0.132	0.0259	0.0195	No	No
161680_r_at	Nr1h2	nuclear receptor subfamily 1, group H, member 2	1.0127	0.7934	0.0867	0.3356	No	1.0543	0.201	0.0859	0.0486	No	No
161681_i_at	Sars2	seryl-aminoacyl-tRNA synthetase 2	1.095	0.3234	0.0888	0.1784	No	1.1597	0.149	0.455	0.0643	No	No
161682_f_at	Gpaa1	GPI anchor attachment protein 1	1.0214	0.4328	0.836	0.1853	No	0.9967	0.948	0.0027	0.291	No	No
161683_r_at	Gtpbp1	GTP binding protein 1	1.1044	0.1529	0.1216	0.1365	No	1.0696	0.224	0.0773	0.216	No	No
161684_r_at	Lcn2	lipocalin 2	1.0193	0.5901	0.0005	0.2526	No	1.1703	0.0002	0.0001	0.0011	No	No

161685_r_at	Nufip1	Nuclear fragile X mental retardation protein interacting protein 1	1.1042	0.2224	0.5424	0.1219	No	1.2011	0.15	0.108	0.112	No	No
161686_i_at	Xrcc5	X-ray repair complementing defective repair in Chinese hamster cells 5	0.9916	0.8892	0.8083	0.7012	No	0.9642	0.761	0.624	0.905	No	No
161687_r_at	Slc29a1	solute carrier family 29 (nucleoside transporters), member 1	1.1291	0.1395	0.0087	0.0877	No	1.0689	0.544	0.0176	0.473	No	No
161688_r_at	---	---	0.9451	0.1126	0.0015	0.8724	No	0.9446	0.131	0.0005	0.624	No	No
161689_f_at	Il1r2	interleukin 1 receptor, type II	0.8021	0.1046	0.0175	0.487	No	0.9321	0.228	0.0049	0.22	No	No
161690_at	Gzmg	granzyme G	0.9361	0.5335	0.4685	0.5392	No	0.9654	0.898	0.989	0.617	No	No
161691_at	Htatsf1	HIV TAT specific factor 1	1.0094	0.9238	0.0905	0.7466	No	0.968	0.746	0.033	0.281	No	No
161692_r_at	---	---	0.9421	0.3224	0.0935	0.1912	No	1.0642	0.31	0.015	0.0615	No	No
161693_r_at	D1Ertd161e	DNA segment, Chr 1, ERATO Doi 161, expressed	1.0426	0.5256	0.0181	0.3604	No	0.8508	0.118	0.187	0.0564	No	No
161694_f_at	Ptgs1	prostaglandin-endoperoxide synthase 1	0.9667	0.5006	0.152	0.7441	No	1.0183	0.19	0.439	0.026	No	No
161695_f_at	Slc6a4	solute carrier family 6 (neurotransmitter transporter, serotonin), member 4	0.9953	0.9172	0.0905	0.3473	No	1.1065	0.009	0.0018	0.002	No	No
161696_f_at	C77080	expressed sequence C77080	1.1117	0.1394	0.0015	0.3464	No	1.1383	0.145	0.0015	0.882	No	No
161697_r_at	D14Ertd436e	DNA segment, Chr 14, ERATO Doi 436, expressed	1.0074	0.9099	0.3469	0.1805	No	1.0629	0.191	0.557	0.0127	No	No
161698_f_at	Rprc1	arginine/proline rich coiled-coil 1	0.979	0.7433	0.8418	0.2366	No	0.9713	0.437	0.0019	0.179	No	No
161699_i_at	Irf6	interferon regulatory factor 6	1.1966	0.1078	0.0616	0.1536	No	1.2001	0.104	0.0367	0.169	No	No
161700_i_at	Nsmaf	neutral sphingomyelinase (N-SMase) activation associated factor	0.9491	0.4705	0.0276	0.2714	No	0.985	0.923	0.0425	0.515	No	No
161701_at	---	---	0.9382	0.1635	0.2201	0.9753	No	1.0166	0.614	0.0003	0.874	No	No
161702_f_at	Lama5 /// LOC671909	laminin, alpha 5 /// similar to Laminin alpha-5 chain precursor	0.9437	0.4152	0.0056	0.2798	No	1.0483	0.486	0.125	0.555	No	No
161703_f_at	Anxa1	annexin A1	0.8855	0.0707	0.6182	0.0204	No	0.876	0.292	0.589	0.145	No	No
161704_r_at	Cd36	CD36 antigen	1.0765	0.1523	0.1921	0.0105	No	1.0897	0.925	0.0908	0.066	No	No
161705_r_at	---	---	1.1223	0.2371	0.3689	0.2397	No	0.9764	0.739	0.0244	0.587	No	No
161706_f_at	Lamc1	laminin, gamma 1	0.9992	0.966	0.0001	0.3019	No	0.9708	0.401	0.0015	0.147	No	No
161707_f_at	---	---	1.1944	0.0238	0.0075	0.0186	No	1.1128	0.0619	0.0041	0.0574	No	No
161708_f_at	Mpdz	multiple PDZ domain protein	0.9202	0.0156	0.0091	0.0046	No	0.9169	0.0556	0.736	0.0052	No	No
161709_at	Fancc	Fanconi anemia, complementation group C	1.0454	0.3319	0.0524	0.8408	No	1.0973	0.15	0.82	0.146	No	No
161710_r_at	1810073N04Rik	RIKEN cDNA 1810073N04 gene	1.0216	0.5456	0.0194	0.527	No	0.7295	0.218	0.0021	0.0218	No	No
161711_f_at	Chx10	C. elegans ceh-10 homeo domain containing homolog	1.086	0.0571	0.0019	0.1018	No	0.9466	0.814	0.0002	0.0589	No	No
161712_r_at	Bnip2	BCL2/adenovirus E1B interacting protein 1, NIP2	0.9937	0.9106	0.2663	0.9678	No	0.9929	0.928	0.37	0.681	No	No
161713_f_at	Ptgfr	prostaglandin F receptor	1.1038	0.1929	0.1338	0.4181	No	0.9994	0.88	0.06	0.116	No	No
161714_f_at	Maoa	monoamine oxidase A	0.9618	0.5109	0.2877	0.7589	No	0.8597	0.529	0.0254	0.181	No	No
161715_f_at	D4Ertd196e	DNA segment, Chr 4, ERATO Doi 196, expressed	1.0309	0.6475	0.6234	0.7886	No	1.0308	0.397	0.0153	0.858	No	No
161716_at	Fos	FBJ osteosarcoma oncogene	1.1569	0.0962	0.0185	0.1132	No	1.1336	0.0941	0.0314	0.315	No	No
161717_i_at	Zfp26	zinc finger protein 26	1.0218	0.825	0.68	0.9208	No	1.0989	0.295	0.248	0.329	No	No
161718_at	Braf	Braf transforming gene	1.0607	0.3343	0.1948	0.8221	No	1.0649	0.369	0.478	0.545	No	No
161719_f_at	1500034J01Rik	RIKEN cDNA 1500034J01 gene	1.0608	0.204	0.056	0.0463	No	1.0827	0.0644	0.147	0.0493	No	No
161720_r_at	Acrbp	proacrosin binding protein	1.0579	0.5701	0.0444	0.1107	No	1.2672	0.024	0.0096	0.0425	No	No
161721_f_at	Cyp17a1	cytochrome P450, family 17, subfamily a, polypeptide 1	0.9615	0.5981	0.0733	0.7983	No	0.7737	0.529	0.355	0.701	No	No
161722_f_at	Gstm5	glutathione S-transferase, mu 5	0.9916	0.8894	0.0253	0.1895	No	0.9418	0.518	0.594	0.524	No	No
161723_at	Wdfy2	WD repeat and FYVE domain containing 2	0.9374	0.1179	0.3633	0.4114	No	0.9579	0.204	0.0067	0.0351	No	No
161724_r_at	Tgoln1 /// Tgoln2	trans-golgi network protein /// trans-golgi network protein 2	1.089	0.0726	0.0226	0.01	No	0.8657	0.426	0.0377	0.0575	No	No
161725_r_at	Ube2v2	ubiquitin-conjugating enzyme E2 variant 2	0.9857	0.5817	0.2302	0.0088	No	0.9303	0.854	0.385	0.56	No	No
161726_f_at	Dnm2	dynamin 2	1.0124	0.7688	0.2389	0.0431	No	0.9616	0.335	0.844	0.17	No	No
161727_r_at	Rad52	RAD52 homolog (S. cerevisiae)	1.0744	0.2076	0.6145	0.1683	No	1.1849	0.284	0.344	0.0475	No	No
161728_f_at	Pde4dip	phosphodiesterase 4D interacting protein (myomegalin)	0.9594	0.4533	0.4801	0.0723	No	0.8876	0.119	0.553	0.0326	No	No
161729_f_at	Josd1	Josephin domain containing 1	1	0.9989	0.0482	0.1529	No	0.9914	0.939	0.0135	0.368	No	No
161730_f_at	Itgae	integrin, alpha E, epithelial-associated	0.9853	0.8783	0.0898	0.4345	No	0.8796	0.105	0.0677	0.0831	No	No
161731_at	Rrm1	ribonucleotide reductase M1	1.008	0.9321	0.2867	0.3853	No	1.078	0.41	0.0504	0.215	No	No
161732_at	---	---	0.9519	0.5609	0.316	0.5072	No	1.0378	0.737	0.164	0.663	No	No
161733_at	Sqrdl	sulfide quinone reductase-like (yeast)	1.0008	0.9872	0.1592	0.4226	No	0.9579	0.771	0.366	0.19	No	No
161734_r_at	Clgn	Calmegin	1.0839	0.343	0.3314	0.2112	No	1.053	0.52	0.216	0.132	No	No
161735_r_at	5730469M10Rik	RIKEN cDNA 5730469M10 gene	1.1585	0.0201	0.0277	0.014	No	1.2199	0.0093	0.0506	0.0103	Yes	Yes
161736_r_at	---	---	1.0616	0.0067	0.315	0.001	No	1.0584	0.529	0.568	0.0123	No	No
161737_at	Utp18	UTP18, small subunit (SSU) processome component, homolog (yeast)	1.0611	0.0937	0.6094	0.0451	No	1.0695	0.0502	0.869	0.019	No	No
161738_f_at	Ilvbl	ilvB (bacterial acetolactate synthase)-like	0.9642	0.1531	0.0451	0.0231	No	0.9282	0.018	0.0853	0.0043	No	No
161739_r_at	Pola2	polymerase (DNA directed), alpha 2	1.0416	0.6018	0.4903	0.2515	No	1.014	0.696	0.0228	0.0292	No	No
161740_r_at	Rag1	recombination activating gene 1	1.0676	0.1043	0.1014	0.8389	No	1.1199	0.184	0.0348	0.822	No	No
161741_r_at	Zfp503	zinc finger protein 503	1.0198	0.6759	0.0498	0.2343	No	1.0256	0.766	0.0042	0.892	No	No
161742_r_at	---	---	1.0338	0.3447	0.0005	0.9188	No	1.0371	0.851	0.0026	0.139	No	No
161743_i_at	Eif3s9	eukaryotic translation initiation factor 3, subunit 9 (eta)	0.9954	0.9499	0.1571	0.3433	No	1.0719	0.107	0.191	0.236	No	No
161744_f_at	Mark3	MAP/microtubule affinity-regulating kinase 3	0.8482	0.3712	0.1222	0.7196	No	0.7147	0.208	0.303	0.555	No	No
161745_f_at	Hspa4	heat shock protein 4	0.9962	0.9488	0.5902	0.8542	No	1.0483	0.573	0.604	0.503	No	No
161746_i_at	Bxdc2	brix domain containing 2	0.8331	0.27	0.1792	0.5553	No	0.6189	0.12	0.029	0.573	No	No
161747_i_at	Myo5b	Myosin Vb	1.0743	0.063	0.0051	0.0675	No	1.0999	0.0069	0.0021	0.0715	Yes	No
161748_r_at	---	---	0.9758	0.8398	0.0607	0.9395	No	0.7752	0.136	0.0019	0.0403	No	No
161749_r_at	Tug1	taurine upregulated gene 1	1.0597	0.4821	0.6597	0.1864	No	1.0412	0.347	0.341	0.0312	No	No

161750_f_at	Ncam1	neural cell adhesion molecule 1	0.8359	0.0023	0.0397	0.9497	Yes	0.8771	0.0128	0.0005	0.0454	No	Yes
161751_f_at	R3hcc1	R3H domain and coiled-coil containing 1	1.0375	0.6954	0.0578	0.7764	No	1.0517	0.737	0.0685	0.703	No	No
161752_r_at	Cx3cl1	chemokine (C-X3-C motif) ligand 1	0.9847	0.7135	0.3612	0.5491	No	1.127	0.0381	0.308	0.366	No	No
161753_f_at	Gpd1	glycerol-3-phosphate dehydrogenase 1 (soluble)	1.1445	0.0285	0.1131	0.8861	No	1.0684	0.211	0.016	0.576	No	No
161754_f_at	Glb1	galactosidase, beta 1	0.9964	0.9303	0.1976	0.4814	No	0.9998	0.999	0.211	0.402	No	No
161755_at	Erp29	endoplasmic reticulum protein 29	1.1159	0.0327	0.007	0.2531	No	1.1065	0.0946	0.146	0.0468	No	No
161756_at	Rnmtl1	RNA methyltransferase like 1	1.0663	0.6051	0.1274	0.2992	No	1.0911	0.136	0.006	0.0311	No	No
161757_f_at	Ranbp5	RAN binding protein 5	1.0235	0.65	0.7988	0.7068	No	1.1246	0.119	0.433	0.116	No	No
161758_r_at	Rbm9	RNA binding motif protein 9	1.0776	0.4486	0.3428	0.114	No	1.0412	0.563	0.151	0.114	No	No
161759_r_at	Lcat	lecithin cholesterol acyltransferase	1.075	0.1246	0.0031	0.1074	No	1.1654	0.0887	0.002	0.0586	No	No
161760_s_at	Rbbp6	retinoblastoma binding protein 6	0.9197	0.117	0.0084	0.176	No	0.9539	0.0361	0.0001	0.0083	No	No
161761_r_at	Hsp90ab1	heat shock protein 90kDa alpha (cytosolic), class B member 1	0.9009	0.308	0.0002	0.0839	No	0.9147	0.0561	0.0812	0.754	No	No
161762_at	---	---	0.9211	0.0598	0.0019	0.8605	No	0.959	0.615	0.0109	0.652	No	No
161763_r_at	Pip5k2c	phosphatidylinositol-4-phosphate 5-kinase, type II, gamma	1.0763	0.5	0.5498	0.3492	No	1.0648	0.519	0.221	0.331	No	No
161764_r_at	Stac	src homology three (SH3) and cysteine rich domain	0.9541	0.5331	0.3132	0.826	No	1.613	0.895	0.148	0.455	No	No
161765_f_at	Rgs10	regulator of G-protein signalling 10	1.005	0.8025	0.032	0.6578	No	1.0118	0.956	0.0228	0.0705	No	No
161766_i_at	Dalrd3	DALR anticodon binding domain containing 3	0.9855	0.7951	0.0204	0.3081	No	1.1256	0.204	0.0362	0.276	No	No
161767_r_at	Mrps18a	mitochondrial ribosomal protein S18A	0.9828	0.7903	0.0054	0.8297	No	0.9811	0.846	0.0012	0.172	No	No
161768_r_at	Map3k8	mitogen activated protein kinase kinase kinase 8	1.1016	0.2299	0.0158	0.4988	No	1.0259	0.789	0.0243	0.352	No	No
161769_r_at	Bphl	biphenyl hydrolase-like (serine hydrolase, breast epithelial mucin-associated antigen)	0.9981	0.9782	0.0559	0.1116	No	0.9467	0.326	0.0005	0.58	No	No
161770_f_at	D1Ertd161e	DNA segment, Chr 1, ERATO Doi 161, expressed	0.8723	0.1434	0.6387	0.2642	No	0.9404	0.162	0.0253	0.0547	No	No
161771_r_at	Gpr162	G protein-coupled receptor 162	1.0329	0.1692	0.002	0.0208	No	1.0675	0.123	0.0136	0.128	No	No
161772_i_at	Gtf2ird2	GTF2I repeat domain containing 2	0.976	0.7223	0.3703	0.1868	No	1.1459	0.0775	0.323	0.0295	No	No
161773_i_at	Cds2	CDP-diacylglycerol synthase (phosphatidate cytidylyltransferase) 2	1.1014	0.2141	0.711	0.12	No	1.0282	0.662	0.409	0.79	No	No
161774_f_at	Lypla1	lysophospholipase 1	0.9755	0.6772	0.006	0.2976	No	1.0851	0.147	0.0007	0.966	No	No
161775_f_at	---	---	1.0309	0.6217	0.7589	0.5422	No	1.004	0.888	0.004	0.859	No	No
161776_at	Matn2	matrilin 2	1.105	0.0233	0.0069	0.0153	No	1.1076	0.154	0.13	0.052	No	No
161777_f_at	Ercc5	excision repair cross-complementing rodent repair deficiency, complementation group 5	0.8995	0.0928	0.1151	0.2318	No	0.9193	0.265	0.372	0.585	No	No
161778_i_at	Ralgds	ral guanine nucleotide dissociation stimulator	1.0108	0.8885	0.5179	0.6883	No	0.9608	0.731	0.977	0.95	No	No
161779_r_at	Cryl1	crystallin, lamda 1	0.8934	0.2748	0.2893	0.3161	No	0.9385	0.479	0.0367	0.744	No	No
161780_f_at	Crygb	crystallin, gamma B	0.8265	0.0449	0.0072	0.0764	No	0.7818	0.0468	0.0193	0.0566	No	No
161781_at	Mtap4	microtubule-associated protein 4	0.9924	0.9099	0.0464	0.2341	No	1.0194	0.595	0.0142	0.478	No	No
161782_r_at	Zdhhc14	Zinc finger, DHHC domain containing 14	1.1169	0.3904	0.8252	0.1088	No	1.2876	0.0326	0.735	0.014	No	No
161783_at	Aldh7a1	Aldehyde dehydrogenase family 7, member A1	1.0134	0.8859	0.6859	0.8284	No	0.9963	0.88	0.0004	0.677	No	No
161784_f_at	---	---	0.9776	0.6316	0.8478	0.3228	No	0.9414	0.294	0.776	0.815	No	No
161785_f_at	Bcdin3	bin3, bicoid-interacting 3, homolog (Drosophila)	0.9523	0.537	0.0251	0.3765	No	0.8812	0.36	0.175	0.406	No	No
161786_f_at	---	---	1.017	0.6598	0.076	0.7811	No	0.9678	0.452	0.0211	0.0393	No	No
161787_f_at	Cdt1	chromatin licensing and DNA replication factor 1	1.0255	0.4197	0.0012	0.17	No	1.0426	0.309	0.0026	0.785	No	No
161788_f_at	Edg1	endothelial differentiation sphingolipid G-protein-coupled receptor 1	1.1456	0.0599	0.0848	0.8243	No	0.9549	0.432	0.0071	0.169	No	No
161789_r_at	---	---	1.0719	0.6374	0.0017	0.6812	No	1.0298	0.851	0.0001	0.779	No	No
161790_at	Rab3a	RAB3A, member RAS oncogene family	1.0864	0.2262	0.0429	0.1834	No	1.2198	0.142	0.444	0.0576	No	No
161791_r_at	Tbc1d10a	TBC1 domain family, member 10a	0.9852	0.6911	0.7087	0.1055	No	0.9687	0.467	0.968	0.192	No	No
161792_f_at	Nr2f2	nuclear receptor subfamily 2, group F, member 2	1.1054	0.0448	0.0601	0.3346	No	1.3288	0.117	0.0085	0.969	No	No
161793_at	Lama4	laminin, alpha 4	0.903	0.1261	0.2335	0.5979	No	1.0394	0.818	0.29	0.562	No	No
161794_i_at	D16Bwg1494e	DNA segment, Chr 16, Brigham & Women's Genetics 1494 expressed	1.0101	0.8641	0.0393	0.6851	No	0.9991	0.907	0.31	0.253	No	No
161795_r_at	Prpf39	PRP39 pre-mRNA processing factor 39 homolog (yeast)	1.0699	0.376	0.2128	0.0826	No	1.1165	0.23	0.0529	0.0839	No	No
161796_r_at	Kcnq1	potassium voltage-gated channel, subfamily Q, member 1	1.0588	0.194	0.0089	0.0199	No	1.0561	0.0198	0.0001	0.0055	No	No
161797_r_at	Xpot	exportin, tRNA (nuclear export receptor for tRNAs)	1.0923	0.5375	0.0234	0.0539	No	1.1027	0.0682	0.003	0.007	No	No
161798_r_at	Gtf3c4	general transcription factor IIIC, polypeptide 4	1.1156	0.0851	0.0961	0.0085	No	1.1057	0.12	0.261	0.0066	No	No
161799_r_at	Kcnj9	potassium inwardly-rectifying channel, subfamily J, member 9	1.002	0.9835	0	0.3992	No	1.0174	0.78	0.471	0.128	No	No
161800_r_at	Ddx50	DEAD (Asp-Glu-Ala-Asp) box polypeptide 50	0.9857	0.7786	0.1451	0.0837	No	1.0699	0.218	0.0869	0.0507	No	No
161801_r_at	Sema3b	sema domain, immunoglobulin domain (Ig), short basic domain, secreted, (semaphorin) 3B	0.9533	0.7232	0.1321	0.141	No	1.0269	0.722	0.299	0.356	No	No
161802_i_at	Egr1	Early growth response 1	1.1027	0.2931	0.2959	0.1782	No	1.2112	0.112	0.25	0.078	No	No
161803_r_at	---	---	1.0206	0.7403	0.0088	0.4279	No	0.9812	0.727	0.0085	0.865	No	No
161804_r_at	Bphl	biphenyl hydrolase-like (serine hydrolase, breast epithelial mucin-associated antigen)	0.9624	0.3621	0.137	0.5885	No	1.0865	0.948	0.012	0.0276	No	No
161805_r_at	Nsun2	NOL1/NOP2/Sun domain family 2	1.1103	0.2222	0.8531	0.0987	No	1.0696	0.134	0.0084	0.008	No	No
161806_r_at	Atp6ap1	ATPase, H+ transporting, lysosomal accessory protein 1	0.8509	0.0563	0.0001	0.5986	No	0.9142	0.0777	0	0.302	No	No
161807_i_at	Tbc1d22a	TBC1 domain family, member 22a	0.9927	0.961	0.1164	0.4222	No	0.9494	0.953	0.0499	0.433	No	No
161808_f_at	Evl	Ena-vasodilator stimulated phosphoprotein	1.0069	0.7894	0.0611	0.2005	No	0.9858	0.64	0.0009	0.0252	No	No
161809_r_at	Rnf4	ring finger protein 4	1.022	0.732	0.7235	0.2675	No	0.9963	0.851	0.387	0.139	No	No
161810_r_at	---	---	0.9806	0.766	0.1631	0.4147	No	1.0465	0.186	0.0007	0.0939	No	No
161811_f_at	Ptpra	protein tyrosine phosphatase, receptor type, A	1.0268	0.6088	0.3675	0.481	No	0.9735	0.738	0.591	0.877	No	No
161812_r_at	---	---	0.9769	0.6104	0.8954	0.6099	No	0.9313	0.0876	0.984	0.279	No	No
161813_i_at	---	---	1.0001	0.999	0.6191	0.8144	No	1.0365	0.717	0.556	0.821	No	No

161814_f_at	Rnf19	Ring finger protein (C3HC4 type) 19	1.0822	0.2852	0.0484	0.2672	No	0.9854	0.963	0.0257	0.174	No	No
161815_f_at	Mup1 /// Mup2 /// Mup3 /// MGC107671 /// LOC670615	major urinary protein 1 /// major urinary protein 2 /// major urinary protein 3 /// similar to alpha-2u-globulin V precursor - mouse /// similar to alpha-2u globulin PGCL3	1.0158	0.8348	0.0268	0.2375	No	1.0958	0.0846	0.0013	0.101	No	No
161816_r_at	Gltp	glycolipid transfer protein	1.007	0.8757	0.4439	0.4146	No	1.0387	0.194	0.479	0.0137	No	No
161817_f_at	Spsb1	splA/ryanodine receptor domain and SOCS box containing 1	0.8967	0.1792	0.2381	0.0238	No	0.8282	0.0985	0.265	0.0286	No	No
161818_f_at	Car4	carbonic anhydrase 4	1.0021	0.9608	0.0973	0.7008	No	0.983	0.825	0.411	0.44	No	No
161819_f_at	Laptm5	lysosomal-associated protein transmembrane 5	1.0226	0.4916	0.001	0.0728	No	1.0105	0.494	0.0003	0.107	No	No
161820_f_at	Gata2	GATA binding protein 2	1.0044	0.9488	0.2165	0.3486	No	0.937	0.682	0.457	0.922	No	No
161821_f_at	Entpd2	ectonucleoside triphosphate diphosphohydrolase 2	1.0155	0.7142	0.8797	0.6321	No	1.0209	0.674	0.113	0.154	No	No
161822_at	Svp2	seminal vesicle protein 2	0.9778	0.6139	0.2751	0.2681	No	1.0055	0.996	0.0024	0.492	No	No
161823_r_at	Cd151	CD151 antigen	0.9567	0.235	0.1301	0.1885	No	1.1428	0.0011	0.0001	0.0052	No	No
161824_r_at	Indo	indoleamine-pyrrole 2,3 dioxygenase	1.023	0.7785	0.6943	0.6527	No	1.0255	0.659	0.325	0.299	No	No
161825_f_at	Ceacam10	CEA-related cell adhesion molecule 10	1.0087	0.8616	0.0256	0.4253	No	1.0078	0.876	0.0032	0.675	No	No
161826_r_at	Glul	glutamate-ammonia ligase (glutamine synthetase)	1.0971	0.2155	0.0309	0.3382	No	0.9647	0.661	0.718	0.576	No	No
161827_f_at	Hpxn	hemopexin	0.9572	0.1133	0.0388	0.2453	No	1.0367	0.555	0.501	0.149	No	No
161828_r_at	---	---	1.057	0.2194	0.0082	0.0128	No	0.8387	0.0767	0.0001	0.565	No	No
161829_at	Nes	nestin	0.9654	0.3468	0.2108	0.1964	No	1.024	0.641	0.112	0.0955	No	No
161830_f_at	Mocos	molybdenum cofactor sulfurase	0.949	0.4502	0.5499	0.2712	No	1.047	0.28	0.119	0.402	No	No
161831_at	Cyp1a1	cytochrome P450, family 1, subfamily a, polypeptide 1	1.0733	0.1118	0.0003	0.0026	No	1.0406	0.169	0.001	0.018	No	No
161832_r_at	Csf1r	colony stimulating factor 1 receptor	1.0375	0.3832	0.0001	0.6105	No	0.8503	0.0644	0.0002	0.981	No	No
161833_r_at	---	---	1.0068	0.95	0.7202	0.8886	No	1.0144	0.878	0.228	0.782	No	No
161834_at	Gzmc	granzyme C	0.9393	0.4265	0.0169	0.6691	No	0.9506	0.41	0.0212	0.904	No	No
161835_at	S100g	S100 calcium binding protein G	0.9661	0.6395	0.9757	0.3331	No	0.9292	0.386	0.803	0.535	No	No
161836_r_at	Zhx1	zinc fingers and homeoboxes protein 1	0.8516	0.0675	0.0574	0.6158	No	0.6879	0.0023	0.0328	0.0683	Yes	No
161837_r_at	1700013L23 Rik	RIKEN cDNA 1700013L23 gene	0.9702	0.7382	0.2971	0.9459	No	1.0551	0.468	0.0253	0.316	No	No
161838_f_at	Arpc5l	actin related protein 2/3 complex, subunit 5-like	0.9696	0.6235	0.2126	0.387	No	0.8831	0.0881	0.0025	0.36	No	No
161839_f_at	Rasl2-9 /// LOC626393	RAS-like, family 2, locus 9 /// similar to RAN, member RAS oncogene family	0.9246	0.2625	0.1942	0.5033	No	0.9918	0.79	0.0849	0.153	No	No
161840_f_at	Klk1b3	kallikrein 1-related peptidase b3	0.9757	0.6952	0.0332	0.2732	No	1.0452	0.696	0.453	0.756	No	No
161841_r_at	2310073E15 Rik	RIKEN cDNA 2310073E15 gene	1.1914	0.0277	0.0079	0.006	No	1.0844	0.0538	0.0022	0.0061	No	No
161842_r_at	Etv4	Ets variant gene 4 (E1A enhancer binding protein, E1AF)	0.9608	0.4848	0.2077	0.8731	No	0.939	0.141	0.743	0.105	No	No
161843_at	---	---	1.0029	0.9633	0.043	0.0391	No	0.9701	0.638	0.022	0.155	No	No
161844_at	---	---	1.0495	0.3493	0.4285	0.4732	No	1.13	0.0248	0.996	0.213	No	No
161845_at	Tiam1	T-cell lymphoma invasion and metastasis 1	1.0611	0.4505	0.98	0.5452	No	0.9995	0.992	0.0753	0.655	No	No
161846_r_at	Mag	myelin-associated glycoprotein	1.0243	0.6702	0.476	0.0405	No	1.0455	0.462	0.243	0.0653	No	No
161847_r_at	2210016L21 Rik	RIKEN cDNA 2210016L21 gene	1.028	0.2326	0.9173	0.6595	No	1.034	0.35	0.0276	0.0866	No	No
161848_r_at	Ptpn22	protein tyrosine phosphatase, non-receptor type 22 (lymphoid)	0.9634	0.5327	0.0594	0.4845	No	1.3045	0.321	0.605	0.0572	No	No
161849_r_at	Ins2	insulin II	0.9048	0.2151	0.0071	0.8808	No	0.9473	0.102	0.0001	0.205	No	No
161850_at	---	---	1.0123	0.8966	0.0251	0.6142	No	1.0586	0.234	0.0036	0.189	No	No
161851_r_at	Bphl	biphenyl hydrolase-like (serine hydrolase, breast epithelial mucin-associated antigen)	1.1004	0.1712	0.1311	0.0537	No	1.03	0.611	0.745	0.15	No	No
161852_i_at	Rpo1-3	RNA polymerase 1-3	1.0543	0.6977	0.7734	0.9663	No	0.989	0.93	0.0341	0.922	No	No
161853_f_at	Prkcq	protein kinase C, theta	0.8311	0.0146	0.4113	0.0695	No	0.8909	0.0072	0.94	0.024	Yes	Yes
161854_f_at	Gatad2a	GATA zinc finger domain containing 2A	1.0311	0.6869	0.1788	0.2357	No	1.0074	0.921	0.905	0.595	No	No
161855_at	---	---	1.1417	0.0802	0.1878	0.0258	No	1.1668	0.145	0.108	0.0191	No	No
161856_f_at	Kif20a	kinesin family member 20A	1.0167	0.7373	0.0146	0.9815	No	0.9944	0.876	0.0144	0.722	No	No
161857_r_at	AI428936	expressed sequence AI428936	1.0313	0.4551	0.0002	0.1713	No	1.0526	0.0985	0	0.0219	No	No
161858_f_at	Rb1	retinoblastoma 1	1.0485	0.4117	0.005	0.3145	No	1.0544	0.571	0.0009	0.965	No	No
161859_f_at	Sncg	synuclein, gamma	0.945	0.0991	0.0006	0.8219	No	0.9015	0.0396	0.0002	0.768	No	No
161860_f_at	Lsmd1	LSM domain containing 1	1.1438	0.0362	0.0658	0.942	No	1.022	0.387	0.0002	0.4	No	No
161861_i_at	Cd82	CD82 antigen	1.0403	0.4276	0.0092	0.2202	No	1.0267	0.518	0.0047	0.192	No	No
161862_i_at	S100a13	S100 calcium binding protein A13	0.8402	0.1586	0.1188	0.533	No	0.8551	0.193	0.702	0.594	No	No
161863_r_at	Cd3d	CD3 antigen, delta polypeptide	1.0219	0.686	0.6035	0.0666	No	2.2399	0.703	0.88	0.514	No	No
161864_f_at	Ptdss1	phosphatidylserine synthase 1	1.1267	0.1211	0.0031	0.2275	No	1.1181	0.148	0.0052	0.353	No	No
161865_r_at	Mgst2	microsomal glutathione S-transferase 2	0.8992	0.3217	0.0008	0.1443	No	1.1432	0.261	0.0086	0.289	No	No
161866_at	Epha4	Eph receptor A4	0.9646	0.6423	0.0652	0.3528	No	1.0537	0.294	0.446	0.0528	No	No
161867_f_at	Mos	Moloney sarcoma oncogene	0.9803	0.752	0.001	0.3332	No	1.0447	0.378	0.0003	0.743	No	No
161868_r_at	Dnajc3	DnaJ (Hsp40) homolog, subfamily C, member 3	1.0639	0.416	0.1786	0.2015	No	0.8938	0.066	0.0002	0.15	No	No
161869_i_at	Hsp90aa1 /// LOC670148	heat shock protein 90kDa alpha (cytosolic), class A member 1 /// similar to heat shock protein 1, alpha	0.9005	0.4093	0.6109	0.6517	No	1.064	0.259	0.821	0.18	No	No
161870_at	Usp15	ubiquitin specific peptidase 15	1.0587	0.3217	0.002	0.0983	No	1.0545	0.388	0.0318	0.0999	No	No
161871_f_at	Rho	rhodopsin	1.0181	0.7585	0.0019	0.0261	No	1.0006	0.868	0.0871	0.154	No	No
161872_f_at	---	---	1.0488	0.1962	0.0046	0.0147	No	1.0678	0.604	0.0015	0.0079	No	No
161873_r_at	Gns	glucosamine (N-acetyl)-6-sulfatase	1.0038	0.9538	0.0161	0.9517	No	0.9272	0.0691	0	0.316	No	No
161874_r_at	---	---	0.9808	0.7783	0.3263	0.6271	No	1.0282	0.613	0.461	0.31	No	No
161875_at	Fmr1	fragile X mental retardation syndrome 1 homolog	1.1773	0.0708	0.6063	0.0695	No	1.1958	0.0557	0.828	0.0677	No	No
161876_r_at	Ckmt2	creatine kinase, mitochondrial 2	1.0811	0.1624	0.4894	0.2729	No	1.0334	0.62	0.179	0.128	No	No

161877_f_at	Cat	Catalase	1.032	0.386	0.0362	0.3569	No	0.9902	0.795	0.0755	0.68	No	No
161878_r_at	Reep1	receptor accessory protein 1	0.9166	0.4214	0.007	0.2005	No	0.9529	0.159	0.0028	0.193	No	No
161879_r_at	Ctdsp2	CTD (carboxy-terminal domain, RNA polymerase II, polypeptide A) small phosphatase 2	1.0134	0.7197	0.6072	0.8193	No	1.0231	0.52	0.285	0.357	No	No
161880_r_at	---	---	0.9736	0.5501	0.7397	0.399	No	1.0879	0.51	0.246	0.355	No	No
161881_f_at	Zfp259	zinc finger protein 259	1.0175	0.6682	0.2233	0.9874	No	0.962	0.163	0.0013	0.164	No	No
161882_f_at	Rdh13	Retinol dehydrogenase 13 (all-trans and 9-cis)	0.8609	0.258	0.0378	0.9144	No	0.8429	0.0237	0.0002	0.151	No	No
161883_f_at	Birc2	baculoviral IAP repeat-containing 2	0.9646	0.5875	0.6971	0.3421	No	1.0192	0.648	0.0216	0.165	No	No
161884_r_at	Fxr1h	fragile X mental retardation gene 1, autosomal homolog	0.9826	0.7911	0.8637	0.4355	No	1.0026	0.966	0.564	0.336	No	No
161885_f_at	Gramd3	GRAM domain containing 3	1.1534	0.1207	0.6152	0.0146	No	1.0484	0.583	0.349	0.0796	No	No
161886_at	2410018L13 Rik /// 3110053B16 Rik /// LOC546752 /// LOC632781 /// LOC668594 /// LOC668600 /// LOC668646 /// LOC668660 /// LOC668682 /// LOC673264 /// LOC673929 /// LOC675641 /// LOC677338	RIKEN cDNA 2410018L13 gene /// RIKEN cDNA 3110053B16 gene /// similar to Hippocalcin-like protein 1 (Visinin-like protein 3) (VILIP-3) (Neural visinin-like protein 3) (NVL-3) (NVP-3) /// similar to Hippocalcin-like protein 1 (Visinin-like protein 3) (VILIP-3) (Neural visinin-like protein 3) (NVL-3) (NVP-3) /// similar to Hippocalcin-like protein 1 (Visinin-like protein 3) (VILIP-3) (Neural visinin-like protein 3) (NVL-3) (NVP-3) /// similar to Hippocalcin-like protein 1 (Visinin-like protein 3) (VILIP-3) (Neural visinin-like protein 3) (NVL-3) (NVP-3) /// similar to Hippocalcin-like protein 1 (Visinin-like protein 3) (VILIP-3) (Neural visinin-like protein 3) (NVL-3) (NVP-3) /// similar to Hippocalcin-like protein 1 (Visinin-like protein 3) (VILIP-3) (Neural visinin-like protein 3) (NVL-3) (NVP-3) /// similar to development- and differentiation-enhancing factor 2 /// similar to Hippocalcin-like protein 1 (Visinin-like protein 3) (VILIP-3) (Neural visinin-like protein 3) (NVL-3) (NVP-3) /// similar to Hippocalcin-like protein 1 (Visinin-like protein 3) (VILIP-3) (Neural visinin-like protein 3) (NVL-3) (NVP-3	1.0365	0.4064	0.0215	0.081	No	0.9975	0.995	0.171	0.493	No	No
161887_r_at	Ttc1	tetratricopeptide repeat domain 1	1.0623	0.4212	0.0239	0.0984	No	1.1265	0.139	0.0051	0.119	No	No
161888_r_at	Commd9	COMM domain containing 9	1.0103	0.8674	0.0772	0.2781	No	0.99	0.775	0.25	0.316	No	No
161889_f_at	Aldoa	Aldolase 1, A isoform	0.964	0.6725	0.3299	0.0826	No	0.8587	0.0659	0.244	0.0117	No	No
161890_f_at	Pap	pancreatitis-associated protein	0.803	0.123	0.0204	0.2462	No	0.854	0.096	0.0159	0.103	No	No
161891_r_at	---	---	0.9853	0.8195	0.1556	0.0727	No	1.1345	0.147	0.0196	0.0404	No	No
161892_r_at	Dsp	desmoplakin	0.919	0.3134	0.861	0.7452	No	0.6967	0.183	0.23	0.617	No	No
161893_i_at	Pemt	phosphatidylethanolamine N-methyltransferase	1.0356	0.6609	0.4289	0.291	No	0.9948	0.951	0.0056	0.277	No	No
161894_r_at	Sf1	splicing factor 1	0.9421	0.4568	0.0026	0.3334	No	0.9646	0.28	0.0005	0.117	No	No
161895_s_at	Wdr81	WD repeat domain 81	0.9111	0.058	0.0003	0.0191	No	0.925	0.0702	0.0003	0.0408	No	No
161896_at	Dad1	defender against cell death 1	1.0377	0.0461	0.0002	0.0079	No	1.0011	0.86	0.0237	0.0819	No	No
161897_f_at	Prps1 /// AU021838	phosphoribosyl pyrophosphate synthetase 1 /// expressed sequence AU021838	0.9419	0.2843	0.0299	0.059	No	0.9032	0.0177	0.0154	0.0028	No	No
161898_i_at	Mtx1	metaxin 1	1.12	0.1978	0.453	0.4207	No	1.0458	0.109	0.0138	0.0229	No	No
161899_f_at	Lat2	linker for activation of T cells family, member 2	1.1165	0.2462	0.0003	0.1652	No	1.1528	0.058	0.0009	0.0478	No	No
161900_f_at	Adrb3	adrenergic receptor, beta 3	1.1511	0.1275	0.3892	0.1965	No	1.3563	0.0895	0.418	0.335	No	No
161901_r_at	Bmp2	bone morphogenetic protein 2	1.0539	0.3982	0.0772	0.0453	No	1.0119	0.795	0.14	0.444	No	No
161902_f_at	Gnao1	guanine nucleotide binding protein, alpha o	1.0509	0.1926	0.0014	0.0688	No	1.0844	0.0517	0.0011	0.0157	No	No
161903_f_at	Nfkbiz	nuclear factor of kappa light polypeptide gene enhancer in B-cells inhibitor, zeta	1.005	0.8814	0.184	0.6633	No	0.9511	0.62	0.204	0.454	No	No
161904_f_at	Cftr	cystic fibrosis transmembrane conductance regulator homolog	0.9108	0.0005	0.0365	0.0013	No	1.1816	0.295	0.544	0.0232	No	No
161905_r_at	---	---	1.0267	0.4269	0.0396	0.0583	No	1.1211	0.124	0.0265	0.0988	No	No
161906_f_at	2410022L05 Rik	RIKEN cDNA 2410022L05 gene	1.1147	0.0118	0.0423	0.7016	No	1.031	0.453	0.006	0.16	No	No
161907_s_at	Tnxb	tenascin XB	0.8491	0.0064	0.0713	0.0129	Yes	0.8808	0.0143	0.0157	0.0249	No	Yes
161908_i_at	Ufm1	ubiquitin-fold modifier 1	1.0971	0.367	0.3339	0.7458	No	1.0769	0.314	0.129	0.539	No	No
161909_r_at	---	---	1.0638	0.439	0.2381	0.7294	No	1.0321	0.623	0.14	0.447	No	No
161910_at	Mrpl2	mitochondrial ribosomal protein L2	0.9311	0.4498	0.792	0.82	No	0.9833	0.798	0.755	0.671	No	No
161911_f_at	Eif4e2	eukaryotic translation initiation factor 4E member 2	0.9367	0.1123	0.22	0.9162	No	0.9101	0.0468	0.255	0.597	No	No
161912_r_at	Numb	numb gene homolog (Drosophila)	1.4148	0.0034	0.0077	0.0014	No	1.2845	0.0025	0.337	0.0122	Yes	No
161913_r_at	Fth1	Ferritin heavy chain 1	0.9531	0.4519	0.1073	0.8778	No	0.9465	0.095	0.0079	0.145	No	No
161914_s_at	Lsp1	lymphocyte specific 1	0.9298	0.0599	0	0.0248	No	0.9216	0.0601	0.0001	0.0044	No	No
161915_f_at	---	Transcribed locus, strongly similar to XP_215069.3 PREDICTED: similar to Tu translation elongation factor, mitochondrial [Rattus norvegicus]	0.9432	0.0784	0.4451	0.0977	No	0.9274	0.145	0.782	0.111	No	No
161916_r_at	Serpinf2	serine (or cysteine) peptidase inhibitor, clade F, member 2	0.9695	0.6427	0.0417	0.7419	No	0.9606	0.508	0.0118	0.845	No	No
161917_i_at	Pnmt	phenylethanolamine-N-methyltransferase	1.0378	0.1106	0.1969	0.0063	No	1.0803	0.502	0.0181	0.188	No	No
161918_at	Akr1b7	aldo-keto reductase family 1, member B7	1.3285	0.0016	0.1415	0.0033	No	1.1993	0.066	0.0435	0.103	No	No
161919_r_at	---	---	1.0513	0.4454	0.4579	0.2342	No	1.0386	0.373	0.614	0.444	No	No
161920_r_at	Smarcad1	SWI/SNF-related, matrix-associated actin-dependent regulator of chromatin, subfamily a, containing DEAD/H box 1`	1.0417	0.4395	0.0691	0.1459	No	1.0926	0.0519	0.0016	0.0519	No	No
161921_f_at	Psmc5	protease (prosome, macropain) 26S subunit, ATPase 5	1.0649	0.1034	0.0017	0.0256	No	0.8337	0.0229	0.0004	0.933	No	No
161922_r_at	---	---	1.0866	0.0432	0.0537	0.0586	No	1.3201	0.0221	0.301	0.397	No	No
161923_at	Psmb1	Proteasome (prosome, macropain) subunit, beta type 1	0.9422	0.2319	0.0916	0.5436	No	1.0327	0.39	0.0045	0.123	No	No
161924_f_at	Apoa2	apolipoprotein A-II	0.9471	0.4618	0.5517	0.2659	No	1.0382	0.585	0.834	0.981	No	No
161925_at	Smg5	Smg-5 homolog, nonsense mediated mRNA decay factor (C. elegans)	0.9836	0.8417	0.0825	0.4669	No	0.9843	0.769	0.0296	0.793	No	No

161926_r_at	P4hb	prolyl 4-hydroxylase, beta polypeptide	0.9692	0.286	0.2008	0.7216	No	1.0468	0.11	0.0521	0.332	No	No
161927_at	Zfp622	zinc finger protein 622	0.972	0.4761	0.368	0.2366	No	0.9595	0.103	0.0016	0.0128	No	No
161928_at	3110079O15 Rik	RIKEN cDNA 3110079O15 gene	0.9793	0.7169	0.0014	0.0532	No	0.9716	0.876	0.0006	0.31	No	No
161929_at	---	---	0.954	0.6287	0.1708	0.9858	No	0.937	0.613	0.0779	0.831	No	No
161930_r_at	---	---	0.9163	0.2703	0.0755	0.6329	No	1.1138	0.415	0.874	0.363	No	No
161931_r_at	Mki67 /// LOC638774	antigen identified by monoclonal antibody Ki 67 /// similar to Antigen KI-67	0.9731	0.6521	0.1963	0.1147	No	0.9582	0.539	0.0004	0.587	No	No
161932_r_at	---	---	1.0074	0.8882	0.8924	0.3785	No	0.8785	0.383	0.0224	0.244	No	No
161933_r_at	Ddx24	DEAD (Asp-Glu-Ala-Asp) box polypeptide 24	1.078	0.1396	0.0764	0.2794	No	1.0089	0.732	0.0629	0.179	No	No
161934_f_at	Stmn3	Stathmin-like 3	0.9852	0.8598	0.2201	0.4474	No	0.9391	0.236	0.0156	0.0638	No	No
161935_r_at	Sh3bgr	SH3-binding domain glutamic acid-rich protein	1.055	0.265	0.0179	0.2345	No	0.9758	0.69	0.0229	0.958	No	No
161936_at	---	---	0.9498	0.5691	0.245	0.8885	No	1.0133	0.895	0.238	0.778	No	No
161937_r_at	4931406I20 Rik	RIKEN cDNA 4931406I20 gene	0.8879	0.0334	0.0648	0.7066	No	0.916	0.149	0.026	0.375	No	No
161938_r_at	Psme2 /// Psme2b-ps	proteasome (prosome, macropain) 28 subunit, beta /// protease (prosome, macropain) 28 subunit beta B, pseudogene	1.0888	0.2744	0.0082	0.526	No	0.9943	0.871	0.0005	0.771	No	No
161939_f_at	Sec23b	SEC23B (S. cerevisiae)	1.1122	0.1288	0.0161	0.199	No	1.1122	0.0493	0.007	0.0903	No	No
161940_r_at	Rnf103	ring finger protein 103	1.0213	0.5835	0.3928	0.1236	No	1.0843	0.56	0.162	0.338	No	No
161941_r_at	---	---	1.0846	0.1792	0.1315	0.0904	No	1.0309	0.417	0.133	0.111	No	No
161942_f_at	Cwf19l1	CWF19-like 1, cell cycle control (S. pombe)	0.9551	0.3948	0.0889	0.298	No	1.0203	0.953	0.0698	0.309	No	No
161943_at	Hs3st3a1	heparan sulfate (glucosamine) 3-O-sulfotransferase 3A1	1.0156	0.6954	0.0133	0.1234	No	1.0235	0.482	0.0125	0.134	No	No
161944_at	---	Transcribed locus	1.0245	0.429	0.7084	0.1502	No	1.0624	0.701	0.281	0.0586	No	No
161945_at	Ldhb /// LOC433229	lactate dehydrogenase B /// similar to L-lactate dehydrogenase B chain (LDH-B) (LDH heart subunit) (LDH-H)	1.0521	0.4694	0.2606	0.2588	No	0.9695	0.594	0.0126	0.728	No	No
161946_r_at	Sorbs1	sorbin and SH3 domain containing 1	1.3651	0.0537	0.0714	0.1274	No	1.2544	0.048	0.12	0.0622	No	No
161947_f_at	Alox12e	arachidonate lipoxygenase, epidermal	1	0.9996	0.9509	0.2155	No	1.0952	0.109	0.002	0.142	No	No
161948_f_at	---	---	1.035	0.7587	0.0495	0.4448	No	1.0088	0.836	0.0217	0.623	No	No
161949_at	1500035H01 Rik	RIKEN cDNA 1500035H01 gene	0.9402	0.0244	0.0031	0.0756	No	0.9697	0.515	0.0728	0.8	No	No
161950_at	Sfrs14	splicing factor, arginine/serine-rich 14	1.0798	0.3727	0.1667	0.2629	No	1.1248	0.187	0.195	0.129	No	No
161951_f_at	Mcart1	mitochondrial carrier triple repeat 1	1.007	0.7563	0.0413	0.0898	No	1.0262	0.68	0.313	0.612	No	No
161952_r_at	Rnf20	Ring finger protein 20	1.0192	0.3127	0.0352	0.0378	No	0.7598	0.667	0.475	0.237	No	No
161953_at	Gdpd5	glycerophosphodiester phosphodiesterase domain containing 5	1.0111	0.7365	0.0001	0.0916	No	1.0704	0.467	0.0015	0.0731	No	No
161954_r_at	---	---	0.9985	0.979	0.2331	0.6458	No	0.9825	0.742	0.0046	0.078	No	No
161955_f_at	---	---	1.1282	0.1465	0.0463	0.057	No	1.0428	0.284	0.0089	0.0254	No	No
161956_at	Aldh1a1	aldehyde dehydrogenase family 1, subfamily A1	1.0613	0.3141	0.1273	0.0416	No	1.0737	0.254	0.0444	0.75	No	No
161957_r_at	---	---	0.9909	0.7728	0.4835	0.1245	No	0.713	0.683	0.702	0.245	No	No
161958_at	Eef1g	Eukaryotic translation elongation factor 1 gamma	0.9623	0.3796	0.0206	0.3483	No	1.0449	0.259	0.0004	0.143	No	No
161959_f_at	Lrrn1	leucine rich repeat protein 1, neuronal	1.0011	0.975	0.0013	0.3266	No	1.2873	0.292	0.0039	0.63	No	No
161960_f_at	Dynll2	dynein light chain LC8-type 2	1.023	0.4615	0.7266	0.2109	No	0.9372	0.421	0.0626	0.239	No	No
161961_at	Rpl19	Ribosomal protein L19	1.0493	0.4355	0.9781	0.7201	No	1.0202	0.732	0.671	0.137	No	No
161962_f_at	Mfap4	microfibrillar-associated protein 4	1.0238	0.5131	0.0028	0.028	No	0.9583	0.105	0.0018	0.0973	No	No
161963_f_at	C330007P06 Rik	RIKEN cDNA C330007P06 gene	1.0763	0.082	0.1025	0.0198	No	1.0255	0.477	0.128	0.0176	No	No
161964_r_at	Prkcz	protein kinase C, zeta	1.0747	0.0509	0.0822	0.2961	No	1.0343	0.578	0.367	0.172	No	No
161965_r_at	Smarcd2	SWI/SNF related, matrix associated, actin dependent regulator of chromatin, subfamily d, member 2	0.9476	0.1915	0.009	0.6408	No	0.9551	0.158	0.0083	0.383	No	No
161966_f_at	Entpd6	ectonucleoside triphosphate diphosphohydrolase 6	1.0192	0.6965	0.1762	0.6561	No	1.0384	0.73	0.544	0.851	No	No
161967_at	---	---	0.9668	0.4962	0.002	0.6676	No	1.0079	0.711	0.0024	0.28	No	No
161968_f_at	Ccr5	chemokine (C-C motif) receptor 5	0.9916	0.852	0.4701	0.1158	No	0.9894	0.811	0.232	0.159	No	No
161969_f_at	Capg	capping protein (actin filament), gelsolin-like	0.9468	0.0696	0.0117	0.0244	No	0.8845	0.101	0.0224	0.022	No	No
161970_f_at	Hmgcl	3-hydroxy-3-methylglutaryl-Coenzyme A lyase	1.0048	0.9043	0.794	0.7366	No	0.8045	0.0493	0.0779	0.0875	No	No
161971_r_at	Btk	Bruton agammaglobulinemia tyrosine kinase	1.1953	0.017	0.4951	0.008	No	1.2914	0.0974	0.253	0.045	No	No
161972_r_at	Slc4a1	solute carrier family 4 (anion exchanger), member 1	0.9642	0.7838	0.0463	0.8681	No	1.0178	0.932	0.0104	0.456	No	No
161973_r_at	BC053401	CDNA sequence BC053401	1.0281	0.7455	0.2158	0.5049	No	1.0161	0.811	0.243	0.742	No	No
161974_r_at	2810417H13 Rik	RIKEN cDNA 2810417H13 gene	1.1271	0.1794	0.7943	0.095	No	1.452	0.0416	0.0459	0.028	No	No
161975_r_at	Pgd	phosphogluconate dehydrogenase	1.13	0.0286	0.0116	0.0076	No	1.0494	0.187	0.0015	0.0561	No	No
161976_r_at	Higd1a	HIG1 domain family, member 1A	1.0332	0.359	0.8482	0.014	No	0.7841	0.238	0.212	0.58	No	No
161977_r_at	Scin	scinderin	1.1944	0.0375	0.8703	0.0082	No	1.1789	0.0446	0.0012	0.0688	No	No
161978_r_at	Cpt2	carnitine palmitoyltransferase 2	0.9687	0.5212	0.0348	0.1398	No	1.1911	0.181	0.149	0.531	No	No
161979_f_at	Tmem41b	transmembrane protein 41B	0.8706	0.2459	0.119	0.4499	No	0.616	0.652	0.0394	0.0374	No	No
161980_f_at	Bag3	Bcl2-associated athanogene 3	0.9456	0.6133	0.0026	0.2589	No	0.9549	0.458	0.0017	0.364	No	No
161981_r_at	Flg /// LOC620355 /// LOC675741	filaggrin /// similar to Filaggrin /// similar to Filaggrin	0.9607	0.2523	0.0382	0.5144	No	1.0013	0.803	0.256	0.0403	No	No
161982_at	Ptk2	PTK2 protein tyrosine kinase 2	0.9653	0.4724	0.0586	0.7891	No	1.0093	0.891	0.126	0.611	No	No
161983_f_at	Rnh1	ribonuclease/angiogenin inhibitor 1	0.9874	0.5317	0.0166	0.1353	No	0.9522	0.246	0.0656	0.597	No	No
161984_f_at	Col3a1	procollagen, type III, alpha 1	1.0832	0.287	0.0006	0.6723	No	1.0251	0.749	0.0022	0.994	No	No
161985_f_at	Ktn1	kinectin 1	0.9299	0.3391	0.2392	0.5999	No	0.9667	0.701	0.315	0.633	No	No
161986_f_at	Gpx5	glutathione peroxidase 5	0.9568	0.283	0.0341	0.8072	No	0.9908	0.795	0.0022	0.975	No	No
161987_at	Pdyn	prodynorphin	1.0424	0.4222	0.2026	0.6432	No	0.985	0.697	0.495	0.205	No	No
161988_f_at	Lrpap1	low density lipoprotein receptor-related protein associated protein 1	1.0149	0.7024	0.0075	0.6702	No	1.0055	0.999	0.019	0.716	No	No

161989_f_at	Crat	carnitine acetyltransferase	0.9541	0.2081	0.0765	0.0596	No	0.9496	0.215	0.0171	0.405	No	No
161990_f_at	Diap1	Diaphanous homolog 1 (Drosophila)	1.0585	0.2624	0.9137	0.6824	No	1.0577	0.0561	0.72	0.173	No	No
161991_at	---	---	1.0515	0.4529	0.8863	0.3764	No	1.033	0.455	0.288	0.514	No	No
161992_at	Ppm1m	protein phosphatase 1M	0.9797	0.5801	0.0099	0.4513	No	0.9754	0.522	0.0031	0.478	No	No
161993_r_at	6330403K07 Rik	RIKEN cDNA 6330403K07 gene	1.1536	0.0162	0.0943	0.0136	No	1.1658	0.31	0.109	0.0977	No	No
161994_f_at	Pias3	protein inhibitor of activated STAT 3	1.0757	0.2441	0.6322	0.2042	No	1.092	0.172	0.569	0.354	No	No
161995_r_at	Got2 /// LOC640847	glutamate oxaloacetate transaminase 2, mitochondrial /// similar to Aspartate aminotransferase, mitochondrial precursor (Transaminase A) (Glutamate oxaloacetate transaminase 2)	1.0372	0.5513	0.2119	0.1859	No	0.9856	0.605	0.0501	0.653	No	No
161996_f_at	---	---	1.2625	0.0006	0	0.1611	Yes	1.1375	0.121	0	0.4	No	No
161997_f_at	---	---	1.0776	0.0866	0.0005	0.4632	No	1.2015	0.0574	0.0308	0.252	No	No
161998_f_at	Ndrg4	N-myc downstream regulated gene 4	1.0599	0.2473	0.0013	0.5953	No	1.0648	0.0875	0.0002	0.126	No	No
161999_at	---	---	1.1107	0.1309	0.8202	0.3213	No	1.1289	0.0182	0.707	0.196	No	No
162000_r_at	Clpb	ClpB caseinolytic peptidase B homolog (E. coli)	1.0612	0.1999	0.0267	0.7563	No	1.0559	0.713	0.34	0.747	No	No
162001_f_at	Csnk1d	casein kinase 1, delta	1.0021	0.9645	0.011	0.3736	No	1.0276	0.821	0.0095	0.348	No	No
162002_r_at	---	---	0.9351	0.1951	0.0003	0.1697	No	0.9929	0.904	0.0003	0.142	No	No
162003_at	Rsl1d1	ribosomal L1 domain containing 1	1.1299	0.0433	0.4952	0.0648	No	1.0856	0.116	0.0046	0.139	No	No
162004_i_at	Lig1	Ligase I, DNA, ATP-dependent	1.1518	0.1075	0.7928	0.0824	No	1.0917	0.0014	0.0583	0.0001	No	No
162005_at	Cdk5rap3	CDK5 regulatory subunit associated protein 3	0.9901	0.6734	0.2624	0.1109	No	1.1771	0.0387	0.125	0.667	No	No
162006_r_at	Immt	inner membrane protein, mitochondrial	1.0783	0.0051	0.01	0.0063	No	1.0702	0.0093	0.0011	0.0047	No	No
162007_i_at	Sipa1	Signal-induced proliferation associated gene 1	0.9865	0.8199	0.0745	0.1917	No	1.0234	0.704	0.0228	0.604	No	No
162008_r_at	---	---	1.0508	0.1217	0.0264	0.016	No	1.1635	0.373	0.0966	0.211	No	No
162009_f_at	Msh2	mutS homolog 2 (E. coli)	0.9409	0.2827	0.9351	0.7479	No	0.9512	0.105	0.332	0.175	No	No
162010_r_at	Atf2	activating transcription factor 2	0.9908	0.8979	0.0064	0.3842	No	1.2132	0.198	0.0039	0.582	No	No
162011_f_at	Rhou	ras homolog gene family, member U	0.9475	0.0087	0.0011	0.0028	No	0.9082	0.0646	0.0126	0.0623	No	No
162012_r_at	Cwf19l1	CWF19-like 1, cell cycle control (S. pombe)	0.9737	0.5526	0.1579	0.1074	No	0.8186	0.469	0.948	0.072	No	No
162013_f_at	Mrpl48	Mitochondrial ribosomal protein L48	0.9827	0.5769	0.1407	0.9861	No	0.974	0.438	0.688	0.177	No	No
162014_i_at	Tax1bp3 /// LOC634896	Tax1 (human T-cell leukemia virus type I) binding protein 3 /// similar to Tax1 (human T-cell leukemia virus type I) binding protein 3	1.1259	0.0082	0.0523	0.045	Yes	1.06	0.203	0.0054	0.347	No	No
162015_f_at	Ckmt2	creatine kinase, mitochondrial 2	0.9957	0.9679	0.115	0.0315	No	0.9055	0.214	0.324	0.0355	No	No
162016_f_at	Foxc2	forkhead box C2	1.0398	0.5043	0.4485	0.4441	No	1.0194	0.744	0.885	0.105	No	No
162017_at	Krtap5-5	Keratin associated protein 5-5	0.9794	0.7967	0.072	0.6351	No	1.0136	0.807	0.0848	0.113	No	No
162018_r_at	Cryge /// Crygf	crystallin, gamma E /// crystallin, gamma F	1.0442	0.2791	0.832	0.0235	No	1.0459	0.417	0.29	0.246	No	No
162019_r_at	Crybb2	crystallin, beta B2	1.0067	0.8986	0.0148	0.2275	No	1.0072	0.798	0.0251	0.128	No	No
162020_at	---	---	1.078	0.0036	0.019	0.7465	Yes	1.0812	0.347	0.209	0.129	No	No
162021_r_at	Bcl2l10	Bcl2-like 10	1.0184	0.6065	0.0003	0.0959	No	1.0523	0.0566	0.0007	0.0153	No	No
162022_f_at	---	---	0.9757	0.6057	0.549	0.7127	No	0.9165	0.0574	0.0145	0.101	No	No
162023_f_at	Thbd	thrombomodulin	0.9504	0.6451	0.059	0.5166	No	1.0475	0.86	0.0023	0.613	No	No
162024_at	Fmn1	formin 1	0.9693	0.5809	0.001	0.0517	No	0.9546	0.676	0.0008	0.284	No	No
162025_r_at	---	---	1.055	0.2231	0.2283	0.0355	No	1.2773	0.0916	0.0834	0.0424	No	No
162026_r_at	Snrpb2 /// LOC638698	U2 small nuclear ribonucleoprotein B /// similar to U2 small nuclear ribonucleoprotein B	0.8834	0.0716	0.0379	0.2114	No	0.9628	0.857	0.0045	0.11	No	No
162027_f_at	---	---	0.942	0.0002	0	0.0001	No	1.0144	0.665	0.0054	0.71	No	No
162028_f_at	D4Wsu114e	DNA segment, Chr 4, Wayne State University 114, expressed	0.9859	0.7473	0.3236	0.7137	No	1.0138	0.677	0.581	0.429	No	No
162029_r_at	BC038311	cDNA sequence BC038311	1.0838	0.0725	0.1465	0.0104	No	1.3207	0.0069	0.0036	0.0031	No	No
162030_r_at	Selenbp1 /// Selenbp2	selenium binding protein 1 /// selenium binding protein 2	0.9245	0.4161	0.0001	0.5163	No	0.9586	0.498	0.0001	0.653	No	No
162031_f_at	Galm	galactose mutarotase	0.9231	0.173	0.7829	0.452	No	0.9645	0.45	0.82	0.773	No	No
162032_f_at	Pkm2 /// LOC671267	pyruvate kinase, muscle /// similar to Pyruvate kinase isozyme M2	0.9616	0.2057	0.0183	0.0414	No	0.9728	0.587	0.222	0.0364	No	No
162033_f_at	D2Wsu81e	DNA segment, Chr 2, Wayne State University 81, expressed	1.0235	0.3213	0.0092	0.8926	No	1.0183	0.179	0.0007	0.0285	No	No
162034_r_at	Antxr2	Anthrax toxin receptor 2	1.0396	0.0856	0.8915	0.0106	No	0.9863	0.868	0.741	0.975	No	No
162035_f_at	Vav2	Vav2 oncogene	1.0092	0.913	0.6083	0.748	No	0.9281	0.506	0.143	0.871	No	No
162036_r_at	D4Wsu114e	DNA segment, Chr 4, Wayne State University 114, expressed	0.9607	0.3585	0.7423	0.7012	No	0.9799	0.798	0.0311	0.597	No	No
162037_f_at	Npy2r	neuropeptide Y receptor Y2	1.0739	0.2607	0.0873	0.077	No	1.0185	0.61	0.0086	0.926	No	No
162038_f_at	Galm	galactose mutarotase	0.8849	0.0547	0.0755	0.9669	No	0.8691	0.0025	0.0002	0.0191	Yes	No
162039_f_at	Scamp2	secretory carrier membrane protein 2	1.0629	0.3611	0.4242	0.1097	No	1.0507	0.0927	0.0833	0.0068	No	No
162040_r_at	---	---	1.1716	0.1162	0.7239	0.0541	No	1.1956	0.0669	0.0478	0.0088	No	No
162041_f_at	Muc13	mucin 13, epithelial transmembrane	1.0485	0.4234	0.065	0.2008	No	1.0416	0.464	0.441	0.572	No	No
162042_i_at	---	---	1.0066	0.9227	0.0007	0.32	No	1.1499	0.286	0.0002	0.485	No	No
162043_i_at	Pld4	phospholipase D family, member 4	0.9929	0.9004	0.043	0.6548	No	1.0763	0.475	0.152	0.185	No	No
162044_f_at	Cyp4b1 /// LOC631037	cytochrome P450, family 4, subfamily b, polypeptide 1 /// similar to Cytochrome P450 4B1 (CYPIVB1)	1.2478	0.0262	0.0032	0.1004	No	1.1535	0.0613	0.0013	0.166	No	No
162045_r_at	---	---	1.092	0.2248	0.2844	0.0344	No	1.0894	0.028	0.0683	0.0012	No	No
162046_at	Nt5c3l	5'-nucleotidase, cytosolic III-like	1.0316	0.3829	0.1117	0.0115	No	1.3827	0.658	0.023	0.234	No	No
162047_f_at	---	---	0.9605	0.3797	0.0013	0.0676	No	0.9224	0.246	0.0844	0.0364	No	No
162048_r_at	Arfgef1	ADP-ribosylation factor guanine nucleotide-exchange factor 1(brefeldin A-inhibited)	1.034	0.5173	0.9689	0.4972	No	1.1294	0.0015	0.0003	0.167	Yes	No
162049_f_at	Gdpd5	glycerophosphodiester phosphodiesterase domain containing 5	0.9808	0.7745	0.0498	0.8462	No	1.0029	0.912	0.0188	0.752	No	No
162050_at	Fbxo6b	F-box only protein 6b	0.9897	0.8614	0.9367	0.7375	No	1.0523	0.39	0.101	0.835	No	No
162051_r_at	Svs6	Seminal vesicle secretion 6	1.1169	0.1259	0.9904	0.9844	No	#DIV/0!	0.0204	0.154	0.154	No	No

162052_i_at	Ccs	copper chaperone for superoxide dismutase	1.3238	0.0131	0.9951	0.0163	No	1.3435	0.0586	0.129	0.034	No	No
162053_i_at	Ptpn5	protein tyrosine phosphatase, non-receptor type 5	1.1049	0.0595	0.0659	0.0025	No	1.1115	0.147	0.193	0.0145	No	No
162054_r_at	Snapc3	Small nuclear RNA activating complex, polypeptide 3	0.9832	0.6523	0.7495	0.0915	No	0.9418	0.205	0.0078	0.469	No	No
162055_f_at	Serf2	small EDRK-rich factor 2	0.9387	0.3301	0.2082	0.2831	No	0.9239	0.298	0.5	0.203	No	No
162056_f_at	---	---	0.9314	0.16	0.2565	0.2123	No	1.0279	0.0904	0.0691	0.0191	No	No
162057_f_at	Mtdh	Metadherin	0.7784	0.0427	0.9076	0.1506	No	0.7969	0.0689	0.954	0.199	No	No
162058_f_at	---	---	0.9148	0.2329	0.0177	0.2887	No	0.9742	0.674	0.0286	0.472	No	No
162059_r_at	Usp14	ubiquitin specific peptidase 14	0.9738	0.6339	0.0606	0.114	No	0.0963	0.0475	0.354	0.373	No	No
162060_r_at	Lig3	ligase III, DNA, ATP-dependent	0.9629	0.5173	0.0022	0.9935	No	0.9406	0.0265	0.0086	0.0121	No	No
162061_f_at	Ppp2r4	protein phosphatase 2A, regulatory subunit B (PR 53)	0.9641	0.5219	0.0018	0.118	No	1.0305	0.711	0.894	0.684	No	No
162062_r_at	Adam2	A disintegrin and metallopeptidase domain 2	1.0471	0.0589	0.4423	0.0291	No	1.2448	0.0796	0.936	0.017	No	No
162063_f_at	Mapkapk5 /// Adam1a	MAP kinase-activated protein kinase 5 /// a disintegrin and metallopeptidase domain 1a	1.1699	0.1082	0.0714	0.0478	No	1.1841	0.182	0.215	0.0236	No	No
162064_at	---	---	1.0167	0.7464	0.1249	0.1042	No	1.0215	0.535	0.113	0.658	No	No
162065_r_at	Dusp9	dual specificity phosphatase 9	1.0096	0.8634	0.0104	0.8007	No	1.0677	0.58	0.0304	0.225	No	No
162066_f_at	AA536749	expressed sequence AA536749	1.0346	0.5357	0.5516	0.7526	No	1.0279	0.565	0.212	0.423	No	No
162067_at	Rbm14	RNA binding motif protein 14	1.0747	0.3165	0.0239	0.4663	No	1.0113	0.814	0.0104	0.998	No	No
162068_r_at	Emb	embigin	1.0087	0.8709	0.0854	0.0808	No	1.0113	0.985	0.565	0.123	No	No
162069_at	Hes5	Hairy and enhancer of split 5 (Drosophila)	0.9816	0.6412	0.0084	0.8049	No	1.028	0.615	0.345	0.618	No	No
162070_r_at	Cyp24a1	Cytochrome P450, family 24, subfamily a, polypeptide 1	1.2198	0.1076	0.1329	0.1082	No	1.2553	0.0916	0.941	0.0948	No	No
162071_i_at	Umps	uridine monophosphate synthetase	0.8801	0.0448	0.0019	0.6268	No	0.9916	0.235	0	0.0161	No	No
162072_at	---	---	0.9294	0.4886	0.0037	0.1402	No	0.9979	0.912	0.0003	0.497	No	No
162073_r_at	Tsc1	Tuberous sclerosis 1	1.0883	0.0562	0.0465	0.1913	No	1.1122	0.233	0.2	0.0708	No	No
162074_r_at	---	---	1.004	0.8915	0.02	0.1179	No	0.9766	0.492	0.269	0.12	No	No
162075_r_at	Mpeg1 /// LOC671359	macrophage expressed gene 1 /// similar to macrophage expressed gene 1	1.0135	0.683	0.8909	0.2876	No	0.8866	0.877	0.609	0.116	No	No
162076_r_at	Gria2	glutamate receptor, ionotropic, AMPA2 (alpha 2)	0.9963	0.862	0.3583	0.8384	No	0.9135	0.945	0.156	0.337	No	No
162077_f_at	Scd2	stearoyl-Coenzyme A desaturase 2	1.0828	0.0777	0.0011	0.4366	No	1.0652	0.243	0.0069	0.748	No	No
162078_at	Sord	sorbitol dehydrogenase	0.9854	0.8462	0.5642	0.8107	No	0.8857	0.119	0.767	0.517	No	No
162079_at	Mbc2	membrane bound C2 domain containing protein	0.9194	0.3419	0.3771	0.2608	No	0.9493	0.554	0.652	0.255	No	No
162080_f_at	Kctd17	potassium channel tetramerisation domain containing 17	0.9449	0.5144	0.0422	0.5129	No	0.9434	0.35	0.0684	0.528	No	No
162081_f_at	---	---	1.0266	0.506	0.1529	0.2638	No	0.9656	0.0559	0.0217	0.291	No	No
162082_r_at	---	---	1.1696	0.0362	0.8961	0.1369	No	1.17	0.104	0.115	0.0822	No	No
162083_f_at	Ntan1	N-terminal Asn amidase	1.025	0.4119	0.0033	0.0345	No	1.1124	0.12	0.0049	0.225	No	No
162084_i_at	---	---	0.8596	0.1327	0.2051	0.1658	No	0.9902	0.715	0.0018	0.482	No	No
162085_r_at	2900086B20 Rik	RIKEN cDNA 2900086B20 gene	0.9914	0.8505	0.3634	0.9382	No	1.4071	0.223	0.217	0.584	No	No
162086_r_at	Trim30	tripartite motif protein 30	0.9958	0.9092	0.0359	0.0412	No	1.0885	0.396	0.101	0.169	No	No
162087_f_at	Kcnab1	potassium voltage-gated channel, shaker-related subfamily, beta member 1	0.9958	0.9168	0.0155	0.82	No	1.0858	0.0915	0.0665	0.157	No	No
162088_r_at	Cfl2	cofilin 2, muscle	0.9443	0.3883	0.4684	0.5754	No	1.174	0.459	0.173	0.652	No	No
162089_r_at	Tln1	talin 1	0.9979	0.944	0.5399	0.1792	No	1.0144	0.702	0.122	0.517	No	No
162090_i_at	D1Ertd161e	DNA segment, Chr 1, ERATO Doi 161, expressed	0.9229	0.2066	0.0064	0.8287	No	1.0665	0.753	0.0214	0.389	No	No
162091_f_at	Sf3b2	Splicing factor 3b, subunit 2	1.0591	0.4417	0.6243	0.229	No	1.0973	0.0351	0.0083	0.0219	No	No
162092_f_at	Ihpk1	inositol hexaphosphate kinase 1	1.0241	0.7859	0.2348	0.2448	No	1.0472	0.572	0.681	0.484	No	No
162093_at	Yars	Tyrosyl-tRNA synthetase	1.0695	0.1395	0.0565	0.0563	No	1.0747	0.213	0.0045	0.749	No	No
162094_f_at	---	---	1.1618	0.2467	0.0163	0.7013	No	1.0687	0.423	0.0193	0.271	No	No
162095_f_at	Gas7	growth arrest specific 7	0.89	0.0562	0.004	0.2101	No	0.8782	0.0713	0.0009	0.0655	No	No
162096_at	---	---	0.9689	0.5495	0.0347	0.6235	No	0.9478	0.233	0.0193	0.918	No	No
162097_r_at	Cdh3	cadherin 3	1.0197	0.7453	0.1688	0.165	No	0.9942	0.918	0.455	0.176	No	No
162098_i_at	Cdc91l1	CDC91 cell division cycle 91-like 1 (S. cerevisiae)	0.9418	0.1272	0.3229	0.8862	No	0.9516	0.763	0.64	0.853	No	No
162099_f_at	Cd2bp2	CD2 antigen (cytoplasmic tail) binding protein 2	1.0246	0.165	0.001	0.0262	No	1.0395	0.324	0.685	0.588	No	No
162100_r_at	Tcta	T-cell leukemia translocation altered gene	1.1476	0.1528	0.5247	0.0489	No	1.0717	0.0702	0.213	0.0022	No	No
162101_f_at	Mylpf /// LOC676912	myosin light chain, phosphorylatable, fast skeletal muscle /// similar to Myosin regulatory light chain 2, skeletal muscle isoform (G2) (DTNB) (MLC-2)	0.8541	0.1359	0.0214	0.012	No	0.7603	0.0014	0.0006	0.0007	No	No
162102_r_at	Mcm5	minichromosome maintenance deficient 5, cell division cycle 46 (S. cerevisiae)	0.9863	0.8757	0.4433	0.3794	No	0.9937	0.878	0.0696	0.842	No	No
162103_i_at	Col9a3	procollagen, type IX, alpha 3	0.9411	0.4373	0.2242	0.8292	No	0.9256	0.343	0.0341	0.894	No	No
162104_f_at	Tapbp	TAP binding protein	1.0296	0.3291	0.3756	0.9728	No	0.9602	0.056	0.011	0.0047	No	No
162105_r_at	Zfp93	Zinc finger protein 93	0.9536	0.4724	0.0075	0.1637	No	0.9906	0.906	0.141	0.438	No	No
162106_f_at	Krt1-3	keratin complex 1, acidic, gene 3	1.0209	0.7581	0.0955	0.3119	No	1.0821	0.265	0.75	0.15	No	No
162107_r_at	---	---	1.222	0.1256	0.706	0.9983	No	1.4806	0.0736	0.28	0.0717	No	No
162108_f_at	Spt1	salivary protein 1	0.9579	0.4825	0.5334	0.1824	No	0.9023	0.187	0.0055	0.0127	No	No
162109_f_at	Sh3rf1	SH3 domain containing ring finger 1	0.9113	0.2127	0.5964	0.7672	No	0.8867	0.181	0.526	0.316	No	No
162110_f_at	Vps45	vacuolar protein sorting 45 (yeast)	0.9942	0.9028	0.0795	0.4118	No	0.9941	0.77	0.102	0.0716	No	No
162111_r_at	BC021395	cDNA sequence BC021395	1.0828	0.0458	0.0059	0.0055	No	1.1612	0.223	0.139	0.0583	No	No
162112_i_at	Capn2	calpain 2	1.0476	0.2432	0.1347	0.0149	No	1.2201	0.0939	0.925	0.0312	No	No
162113_r_at	Sybl1	synaptobrevin like 1	1.1314	0.0742	0.2036	0.0035	No	1.1503	0.0073	0.027	0.0007	No	No
162114_f_at	Trfp	Trf (TATA binding protein-related factor)-proximal protein homolog (Drosophila)	0.9766	0.4496	0.8352	0.4955	No	1.0038	0.867	0.297	0.122	No	No
162115_i_at	Ftsj3	FtsJ homolog 3 (E. coli)	0.9379	0.6161	0.5751	0.2244	No	0.9528	0.602	0.0348	0.11	No	No
162116_r_at	Derl2	Der1-like domain family, member 2	0.9669	0.5414	0.0264	0.3789	No	#DIV/0!	1	1	1	No	No
162117_f_at	Plac1	placental specific protein 1	0.9518	0.4061	0.0511	0.6006	No	1.0387	0.647	0.232	0.953	No	No

162118_f_at	Btbd14a	BTB (POZ) domain containing 14A	0.9495	0.1852	0.04	0.4534	No	1.075	0.385	0.404	0.14	No	No
162119_r_at	1500003O03 Rik /// LOC638632	RIKEN cDNA 1500003O03 gene /// similar to calcium binding protein P22	1.1525	0.0431	0.1291	0.0865	No	1.747	0.0818	0.0332	0.188	No	No
162120_at	---	---	1.0783	0.197	0.9303	0.0706	No	1.1926	0.517	0.504	0.211	No	No
162121_at	Krt1-10	keratin complex 1, acidic, gene 10	0.8637	0.0459	0.0864	0.626	No	0.9237	0.127	0.0093	0.0153	No	No
162122_f_at	Fdxr	ferredoxin reductase	0.9809	0.7105	0.1812	0.1575	No	0.9627	0.313	0.0077	0.515	No	No
162123_f_at	Slc19a1	solute carrier family 19 (sodium/hydrogen exchanger), member 1	1.0225	0.5415	0.6913	0.8352	No	1.0176	0.602	0.217	0.896	No	No
162124_r_at	Rusc1	RUN and SH3 domain containing 1	1.0636	0.4353	0.1151	0.0835	No	1.2113	0.0809	0.528	0.0646	No	No
162125_f_at	Ubc	ubiquitin C	0.9424	0.2053	0.2678	0.1325	No	0.8558	0.052	0.0093	0.116	No	No
162126_r_at	C130006E23	Hypothetical protein C130006E23	1.0224	0.5103	0.223	0.0746	No	1.0106	0.641	0.0689	0.155	No	No
162127_r_at	Pa2g4	proliferation-associated 2G4	1.0088	0.7914	0.2376	0.5148	No	0.986	0.785	0.331	0.665	No	No
162128_i_at	---	---	1.0107	0.7822	0.001	0.4787	No	1.078	0.0228	0.0003	0.108	No	No
162129_f_at	Ppfibp2	protein tyrosine phosphatase, receptor-type, F interacting protein, binding protein 2	0.8873	0.0627	0.0181	0.1196	No	0.9821	0.515	0.0034	0.0727	No	No
162130_r_at	Sult1a1	sulfotransferase family 1A, phenol-preferring, member 1	1.0626	0.4065	0.5162	0.1702	No	1.0762	0.705	0.565	0.435	No	No
162131_f_at	---	---	1.0078	0.8795	0.1552	0.3812	No	1.0042	0.888	0.221	0.561	No	No
162132_f_at	---	---	0.997	0.9243	0.0016	0.0302	No	1.1327	0.108	0.0142	0.091	No	No
162133_at	Sema4b	sema domain, immunoglobulin domain (Ig), transmembrane domain (TM) and short cytoplasmic domain, (semaphorin) 4B	1.1335	0.0049	0.1316	0.0046	No	1.0743	0.0133	0.0015	0.0308	No	No
162134_r_at	2010111I01R ik	RIKEN cDNA 2010111I01 gene	1.0069	0.8163	0.8047	0.9456	No	0.9849	0.61	0.0793	0.609	No	No
162135_r_at	Slc38a2	solute carrier family 38, member 2	0.9824	0.5293	0.646	0.1269	No	1.8027	0.249	0.167	0.457	No	No
162136_r_at	Tbxas1	Thromboxane A synthase 1, platelet	1.1326	0.0726	0.6621	0.6322	No	1.1785	0.0946	0.398	0.175	No	No
162137_f_at	Txk	TXK tyrosine kinase	1.0512	0.4311	0.0201	0.3531	No	1.0748	0.399	0.0065	0.409	No	No
162138_s_at	Cbx6	chromobox homolog 6	0.9926	0.9014	0.0004	0.3135	No	1.0306	0.342	0.0001	0.631	No	No
162139_r_at	2310044H10 Rik	RIKEN cDNA 2310044H10 gene	0.9566	0.3475	0.0869	0.1033	No	1.2528	0.0286	0.327	0.0218	No	No
162140_i_at	Dhx30	DEAH (Asp-Glu-Ala-His) box polypeptide 30	1.0424	0.5994	0.2281	0.8388	No	1.1021	0.121	0.105	0.387	No	No
162141_r_at	Rab2	RAB2, member RAS oncogene family	1.0093	0.8411	0.433	0.8892	No	1.0296	0.851	0.36	0.301	No	No
162142_f_at	Prrg2	proline-rich Gla (G-carboxyglutamic acid) polypeptide 2	0.9977	0.9393	0.0007	0.8945	No	0.9387	0.467	0.339	0.629	No	No
162143_f_at	Prkrir	protein-kinase, interferon-inducible double stranded RNA dependent inhibitor, repressor of (P58 repressor)	1.0509	0.4593	0.0087	0.3376	No	1.0895	0.174	0.016	0.0764	No	No
162144_at	Etfb	electron transferring flavoprotein, beta polypeptide	0.959	0.475	0.7744	0.1032	No	0.985	0.831	0.344	0.966	No	No
162145_r_at	Pgs1	phosphatidylglycerophosphate synthase 1	0.9359	0.0861	0.9944	0.6923	No	0.9098	0.0995	0.26	0.736	No	No
162146_r_at	4932416N17 Rik	RIKEN cDNA 4932416N17 gene	0.9888	0.8747	0.731	0.7325	No	0.9246	0.58	0.334	0.384	No	No
162147_f_at	Snta1	syntrophin, acidic 1	0.9609	0.2618	0.0242	0.2337	No	0.9918	0.891	0.122	0.861	No	No
162148_r_at	---	---	1.0098	0.7606	0.0129	0.6599	No	0.9312	0.453	0.0141	0.146	No	No
162149_i_at	---	---	0.9592	0.6814	0.0437	0.6035	No	0.8871	0.0585	0.621	0.0688	No	No
162150_r_at	Man2b1	mannosidase 2, alpha B1	1.0667	0.4548	0.4809	0.3258	No	1.0158	0.816	0.0215	0.42	No	No
162151_i_at	---	---	0.9665	0.7158	0.0924	0.2949	No	0.8471	0.382	0.101	0.235	No	No
162152_r_at	Crhr2	corticotropin releasing hormone receptor 2	1.1639	0.0598	0.6742	0.0383	No	1.5209	0.115	0.314	0.284	No	No
162153_i_at	Exosc5	exosome component 5	1.0342	0.4818	0.7446	0.3333	No	1.0605	0.0611	0.0065	0.345	No	No
162154_i_at	Sash1	SAM and SH3 domain containing 1	0.9771	0.7366	0.9969	0.3897	No	0.9625	0.64	0.608	0.358	No	No
162155_f_at	Msh3	mutS homolog 3 (E. coli)	1.0362	0.2571	0.9483	0.7981	No	0.991	0.957	0.258	0.595	No	No
162156_f_at	Srpx	sushi-repeat-containing protein	1.1454	0.0074	0.0014	0.088	Yes	1.0144	0.683	0.0069	0.417	No	No
162157_f_at	Stk16	serine/threonine kinase 16	1.0293	0.7486	0.9908	0.6823	No	1.0853	0.408	0.0138	0.614	No	No
162158_r_at	Acox1	acyl-Coenzyme A oxidase 1, palmitoyl	1.0322	0.5521	0.8232	0.2822	No	1.1498	0.0839	0.418	0.0344	No	No
162159_i_at	Tcf2	transcription factor 2	0.9634	0.3389	0.0048	0.9163	No	1.0105	0.779	0.0005	0.249	No	No
162160_at	Ide	insulin degrading enzyme	1.2873	0.0789	0.1343	0.0379	No	1.1683	0.0364	0.175	0.0255	No	No
162161_r_at	---	---	1.0175	0.5651	0.1948	0.0605	No	0.978	0.884	0.543	0.251	No	No
162162_f_at	2900006F19 Rik	RIKEN cDNA 2900006F19 gene	1.0348	0.1004	0.0068	0.4746	No	0.9953	0.948	0.41	0.559	No	No
162163_at	Sct	secretin	1.0868	0.2892	0.8016	0.6161	No	1.0709	0.422	0.121	0.608	No	No
162164_f_at	Actn3	actinin alpha 3	0.8941	0.057	0.0542	0.0267	No	0.8564	0.0032	0.0032	0.0013	No	No
162165_f_at	2900092E17 Rik	RIKEN cDNA 2900092E17 gene	0.9244	0.0847	0.0274	0.033	No	0.944	0.033	0.0011	0.0008	No	No
162166_f_at	Vmp	vesicular membrane protein p24	1.0132	0.7898	0.9225	0.7761	No	1.0192	0.731	0.356	0.441	No	No
162167_f_at	Pde6b	phosphodiesterase 6B, cGMP, rod receptor, beta polypeptide	1.0724	0.1191	0.0135	0.8144	No	0.9644	0.265	0.0023	0.433	No	No

162168_r_at	Phgdh /// LOC382931 /// LOC384524 /// LOC385344 /// LOC627427 /// LOC666422 /// LOC666875 /// LOC668771 /// LOC669671 /// LOC669985 /// LOC670155 /// LOC670187 /// LOC671102 /// LOC671972 ///	3-phosphoglycerate dehydrogenase /// similar to 3-phosphoglycerate dehydrogenase /// similar to 3-phosphoglycerate dehydrogenase /// similar to 3-phosphoglycerate dehydrogenase /// similar to 3-phosphoglycerate dehydrogenase /// similar to 3-phosphoglycerate dehydrogenase /// similar to 3-phosphoglycerate dehydrogenase /// similar to 3-phosphoglycerate dehydrogenase /// similar to 3-phosphoglycerate dehydrogenase /// similar to 3-phosphoglycerate dehydrogenase /// similar to 3-phosphoglycerate dehydrogenase /// similar to 3-phosphoglycerate dehydrogenase /// similar to 3-phosphoglycerate dehydrogenase /// similar to 3-phosphoglycerate dehydrogenase	1.0454	0.3044	0.2259	0.6131	No	1.0227	0.583	0.98	0.082	No
162169_r_at	Hist1h2bp	Histone 1, H2bp	0.9443	0.5378	0.3048	0.9641	No	0.9406	0.327	0.241	0.746	No
162170_r_at	Fbn1	fibrillin 1	1.0033	0.9547	0.5584	0.6536	No	1.0497	0.372	0.106	0.796	No
162171_f_at	Fgfr2	fibroblast growth factor receptor 2	0.9815	0.8028	0.1081	0.5094	No	1.2446	0.127	0.0695	0.273	No
162172_f_at	Nedd4	neural precursor cell expressed, developmentally down-requlted gene 4	1.0169	0.7435	0.0928	0.2829	No	0.9886	0.954	0.107	0.226	No
162173_i_at	---	---	0.9168	0.0751	0.0047	0.1931	No	0.9886	0.953	0.0029	0.549	No
162174_at	Cyp2d9	cytochrome P450, family 2, subfamily d, polypeptide 9	0.9598	0.3188	0.0005	0.0486	No	1.0482	0.0719	0.0009	0.0046	No
162175_at	Dad1	defender against cell death 1	0.9292	0.2962	0.0022	0.6638	No	0.9452	0.54	0.0074	0.0429	No
162176_r_at	LOC14433 /// LOC219102 /// LOC224508 /// LOC380687 /// AK190093 /// LOC384643 /// LOC384808 /// Gapdh /// LOC432919 /// LOC433184 /// LOC433273 /// LOC435171 /// LOC435963 /// LOC545984 /// LOC546736 ///	similar to glyceraldehyde-3-phosphate dehydrogenase /// similar to glyceraldehyde-3-phosphate dehydrogenase (phosphorylating)-like /// similar to Glyceraldehyde-3-phosphate dehydrogenase (GAPDH) (38 kDa BFA-dependent ADP-ribosylation substrate) (BARS-38) /// similar to glyceraldehyde-3-phosphate dehydrogenase /// cDNA sequence AK190093 /// similar to glyceraldehyde-3-phosphate dehydrogenase /// similar to Glyceraldehyde-3-phosphate dehydrogenase (GAPDH) (38 kDa BFA-dependent ADP-ribosylation substrate) (BARS-38) /// glyceraldehyde-3-phosphate dehydrogenase /// similar to Glyceraldehyde-3-phosphate dehydrogenase (GAPDH) /// similar to Glyceraldehyde-3-phosphate dehydrogenase (GAPDH) /// similar to Glyceraldehyde-3-phosphate dehydrogenase (GAPDH) /// similar to Glyceraldehyde-3-phosphate dehydrogenase (GAPDH) /// similar to Glyceraldehyde-3-phosphate dehydrogenase (GAPDH) /// similar to Glyceraldehyde-3-phosphate dehydrogenase (GAPDH) /// similar to Glyceraldehyde-3-phosphate dehydrogenase (GAPDH) /// similar to Glyceraldehyde	1.0269	0.3846	0.0005	0.0088	No	1.051	0.429	0.0165	0.0141	No
162177_f_at	Rpl3 /// LOC242809 /// LOC433745 /// LOC545864 /// LOC635340 /// LOC668219 /// LOC669672 /// LOC670435 /// LOC670753 /// LOC674810 /// LOC674874	ribosomal protein L3 /// similar to 60S ribosomal protein L3 (J1 protein) /// similar to 60S ribosomal protein L3 (L4) /// similar to 60S ribosomal protein L3 (J1 protein) /// similar to 60S ribosomal protein L3 (L4) /// similar to ribosomal protein L3 isoform b /// similar to 60S ribosomal protein L3 (J1 protein) /// similar to 60S ribosomal protein L3 (J1 protein) /// similar to 60S ribosomal protein L3 (J1 protein) /// similar to 60S ribosomal protein L3 (J1 protein) /// similar to ribosomal protein L3 isoform b	1.0728	0.0899	0.0225	0.0348	No	1.0901	0.0056	0.035	0.0007	No
162178_r_at	---	---	1.0153	0.7756	0.0005	0.4474	No	1.0442	0.179	0.0001	0.611	No
162179_r_at	Blmh	Bleomycin hydrolase	1.1024	0.1448	0.0105	0.0035	No	1.183	0.152	0.0467	0.03	No
162180_r_at	---	---	1.0524	0.2151	0.0745	0.0921	No	0.9321	0.0539	0.147	0.0542	No
162181_f_at	Fcer1g	Fc receptor, IgE, high affinity I, gamma polypeptide	1.0309	0.6383	0.0198	0.7126	No	0.9468	0.374	0.0097	0.605	No
162182_f_at	Kcnab2	potassium voltage-gated channel, shaker-related subfamily, beta member 2	1.1041	0.0648	0.0544	0.6035	No	1.0104	0.727	0.12	0.0258	No
162183_at	C630002B14 Rik	RIKEN cDNA C630002B14 gene	1.105	0.2527	0.1822	0.489	No	1.0904	0.111	0.218	0.135	No
162184_at	Galm	galactose mutarotase	0.9332	0.4697	0.1217	0.9961	No	0.8098	0.392	0.158	0.591	No
162185_i_at	Poldip3	polymerase (DNA-directed), delta interacting protein 3	0.9449	0.1935	0.0016	0.0021	No	0.9311	0.346	0.0154	0.0655	No
162186_r_at	Mep1b	Meprin 1 beta	0.9357	0.2763	0.0017	0.3202	No	1.0043	0.905	0.0967	0.736	No
162187_f_at	Reg3g	regenerating islet-derived 3 gamma	0.9653	0.5694	0.0009	0.0339	No	0.9701	0.377	0.0001	0.0119	No
162188_f_at	P4ha2	procollagen-proline, 2-oxoglutarate 4-dioxygenase (proline 4-hydroxylase), alpha II polypeptide	0.8929	0.0252	0.0041	0.1714	No	0.9079	0.0802	0.0127	0.458	No
162189_r_at	---	---	1.1141	0.0285	0.8241	0.0153	No	1.1386	0.027	0.0984	0.056	No
162190_r_at	Lmbr1l	limb region 1 like	1.0758	0.0343	0	0.1512	No	1.0538	0.134	0	0.124	No
162191_at	Krt1-18 /// LOC382807	keratin complex 1, acidic, gene 18 /// hypothetical LOC382807	1.0335	0.2681	0.1077	0.2367	No	1.1393	0.109	0.255	0.804	No
162192_f_at	Chga	chromogranin A	1.0601	0.4118	0.0721	0.1859	No	1.1097	0.0718	0.0406	0.0404	No
162193_f_at	Itgb7	integrin beta 7	1.1173	0.1107	0.1059	0.2631	No	1.0386	0.646	0.9	0.0593	No

162194_r_at	Pkp2	plakophilin 2	1.0322	0.6046	0.1149	0.6642	No	1.2157	0.383	0.251	0.339	No	No
162195_i_at	---	---	1.1937	0.1199	0.8691	0.0571	No	1.144	0.291	0.869	0.52	No	No
162196_f_at	Cuzd1	CUB and zona pellucida-like domains 1	0.9625	0.5315	0.0216	0.6459	No	1.0199	0.546	0.879	0.0088	No	No
162197_at	Imp4	IMP4, U3 small nucleolar ribonucleoprotein, homolog (yeast)	1.1682	0.0528	0.1277	0.0482	No	1.2314	0.0405	0.0175	0.0719	No	No
162198_f_at	---	---	0.9531	0.4299	0.0236	0.8453	No	0.9677	0.527	0.0139	0.583	No	No
162199_r_at	Mcpt8	mast cell protease 8	1.3246	0.01	0.102	0.0124	No	1.1269	0.456	0.0007	0.379	No	No
162200_r_at	Agr2	anterior gradient 2 (Xenopus laevis)	1.001	0.9678	0.4081	0.0105	No	1.138	0.234	0.749	0.262	No	No
162201_r_at	---	---	1.0056	0.9533	0.0182	0.4692	No	1.0768	0.451	0.0042	0.573	No	No
162202_f_at	Irf7	interferon regulatory factor 7	0.9932	0.9008	0.0248	0.157	No	1.0035	0.959	0.452	0.165	No	No
162203_r_at	D030051N19Rik	RIKEN cDNA D030051N19 gene	1.0424	0.4709	0.2126	0.0791	No	1.0852	0.408	0.14	0.133	No	No
162204_r_at	Notch1	Notch gene homolog 1 (Drosophila)	0.9941	0.8236	0.0092	0.0028	No	1.0325	0.351	0.152	0.0267	No	No
162205_f_at	Prss32	protease, serine, 32	1.0074	0.8557	0.1735	0.5461	No	0.946	0.353	0.3	0.421	No	No
162206_f_at	Socs3	suppressor of cytokine signaling 3	1.0417	0.2764	0.001	0.3447	No	1.0572	0.201	0.0026	0.638	No	No
162207_f_at	Clcn7	Chloride channel 7	1.0166	0.7694	0.2098	0.2296	No	1.0273	0.468	0.161	0.28	No	No
162208_f_at	Vpreb3	pre-B lymphocyte gene 3	1.0472	0.436	0.6972	0.4761	No	1.0206	0.754	0.172	0.642	No	No
162209_r_at	Ptprr	protein tyrosine phosphatase, receptor type, R	1.0154	0.6977	0.0508	0.2947	No	1.1487	0.468	0.688	0.27	No	No
162210_r_at	---	---	1.0168	0.7533	0.2419	0.0371	No	1.1687	0.287	0.261	0.0749	No	No
162211_r_at	BC052328	cDNA sequence BC052328	1.0554	0.1416	0.2099	0.0045	No	0.9177	0.4	0.0324	0.908	No	No
162212_at	---	---	0.8869	0.3301	0.1784	0.3154	No	1.0019	0.857	0.0219	0.312	No	No
162213_i_at	Tmbim1	transmembrane BAX inhibitor motif containing 1	1.1563	0.2717	0.0323	0.3894	No	1.1462	0.124	0.0151	0.119	No	No
162214_r_at	Krt1-2	keratin complex 1, acidic, gene 2	1.1017	0.1421	0.2161	0.0496	No	1.1264	0.143	0.0741	0.0488	No	No
162215_f_at	Sdc2	syndecan 2	1.1198	0.1196	0.8076	0.1593	No	1.1513	0.0747	0.0537	0.0123	No	No
162216_i_at	Ly6d	lymphocyte antigen 6 complex, locus D	1.0692	0.3008	0.0648	0.3857	No	1.0772	0.393	0.144	0.995	No	No
162217_r_at	Fdps /// LOC544723 /// LOC671145	farnesyl diphosphate synthetase /// similar to farnesyl diphosphate synthetase /// similar to farnesyl diphosphate synthetase	1.0388	0.4582	0.9777	0.135	No	1.1126	0.0648	0.0368	0.0039	No	No
162218_f_at	Itgb5	integrin beta 5	0.9999	0.9982	0.1794	0.1173	No	0.9825	0.783	0.0009	0.236	No	No
162219_f_at	---	Transcribed locus	0.9984	0.9808	0.0065	0.8649	No	1.0344	0.723	0.0004	0.513	No	No
162220_r_at	Dnajb13	DnaJ (Hsp40) related, subfamily B, member 13	0.9146	0.5467	0.0005	0.6973	No	0.9585	0.578	0.0004	0.674	No	No
162221_i_at	Blmh	bleomycin hydrolase	1.1468	0.0422	0.0462	0.0471	No	1.2361	0.0054	0.0249	0.25	Yes	Yes
162222_r_at	Eif4e /// LOC630527	eukaryotic translation initiation factor 4E /// hypothetical LOC630527	1.1838	0.0554	0.013	0.0092	No	1.055	0.349	0.114	0.0318	No	No
162223_f_at	Atp2a1	ATPase, Ca++ transporting, cardiac muscle, fast twitch 1	0.5339	0.0003	0.0008	0.0002	No	0.6219	0.0006	0.0043	0.0006	No	No
162224_r_at	Aplp2	amyloid beta (A4) precursor-like protein 2	1.3541	0.0654	0.0102	0.1005	No	1.1664	0.02	0.0009	0.0085	No	No
162225_f_at	---	---	1.0577	0.1	0.0402	0.0971	No	1.0054	0.825	0.0007	0.514	No	No
162226_r_at	Tm9sf3	transmembrane 9 superfamily member 3	1.0761	0.0497	0.0006	0.0171	No	1.0045	0.403	0.0002	0.0183	No	No
162227_r_at	Ung	Uracil DNA glycosylase	1.0375	0.4936	0.0368	0.295	No	1.0224	0.38	0.682	0.0154	No	No
162228_f_at	Stard3	START domain containing 3	1.1205	0.2217	0.0013	0.1613	No	0.931	0.739	0.0185	0.054	No	No
162229_at	---	Transcribed locus, strongly similar to XP_537399.1 PREDICTED: similar to 40S ribosomal protein S4, X isoform [Canis familiaris]	1.0062	0.9321	0.7082	0.5856	No	1.0093	0.861	0.161	0.166	No	No
162230_r_at	Rbbp9 /// LOC677524	retinoblastoma binding protein 9 /// similar to Retinoblastoma-binding protein 9 (RBBP-9) (B5T overexpressed gene protein) (Bog protein)	1.009	0.4867	0.4685	0.5602	No	1.0857	0.578	0.549	0.694	No	No
162231_r_at	Mesdc2	mesoderm development candiate 2	0.9508	0.6757	0.5183	0.8373	No	0.9459	0.56	0.568	0.741	No	No
162232_r_at	Rad51	RAD51 homolog (S. cerevisiae)	1.0425	0.6428	0.4973	0.1403	No	1.4906	0.0551	0.589	0.0539	No	No
162233_r_at	Slc38a2	solute carrier family 38, member 2	1.0042	0.9482	0.1178	0.5024	No	1.0199	0.995	0.0563	0.663	No	No
162234_f_at	Cxcl12	chemokine (C-X-C motif) ligand 12	1.1177	0.0109	0.7307	0.0183	No	1.1719	0.18	0.0007	0.862	No	No
162235_f_at	Prpf19	PRP19/PSO4 pre-mRNA processing factor 19 homolog (S. cerevisiae)	0.9851	0.6963	0.1691	0.3835	No	0.9708	0.603	0.136	0.489	No	No
162236_f_at	Nelf	nasal embryonic LHRH factor	1.0419	0.0926	0.2729	0.466	No	0.9968	0.912	0.0004	0.0076	No	No
162237_f_at	Scg3	Secretogranin III	1.0713	0.2543	0.006	0.3063	No	1.0681	0.251	0.0193	0.129	No	No
162238_r_at	Shkbp1	Sh3kbp1 binding protein 1	1.1072	0.1028	0.2323	0.1113	No	1.0802	0.0459	0.0119	0.005	No	No
162239_at	4933407C03Rik	RIKEN cDNA 4933407C03 gene	0.8853	0.0163	0.0013	0.0403	No	0.8971	0.0922	0.0042	0.631	No	No
162240_r_at	---	---	0.9132	0.1451	0.0603	0.1613	No	0.9034	0.34	0.0198	0.422	No	No
162241_r_at	Gpr178	G protein-coupled receptor 178	1.1193	0.316	0.338	0.3623	No	1.4544	0.184	0.186	0.0913	No	No
162242_at	---	---	0.9058	0.1386	0.0563	0.246	No	0.9994	0.803	0.0058	0.0516	No	No
162243_f_at	Rtkn	rhotekin	1.0093	0.7952	0.0015	0.0915	No	0.8643	0.0542	0.0029	0.0244	No	No

162244_r_at	Rps4x /// LOC225134 /// LOC229746 /// LOC434180 /// LOC546380 /// LOC637070 /// LOC667398 /// LOC668668 /// LOC670119 /// LOC672743 /// LOC674228	ribosomal protein S4, X-linked /// similar to 40S ribosomal protein S4, X isoform /// similar to 40S ribosomal protein S4, X isoform /// similar to 40S ribosomal protein S4, X isoform /// similar to 40S ribosomal protein S4, X isoform /// similar to 40S ribosomal protein S4, X isoform /// similar to 40S ribosomal protein S4, X isoform /// similar to 40S ribosomal protein S4, X isoform /// similar to 40S ribosomal protein S4, X isoform /// similar to 40S ribosomal protein S4, X isoform /// similar to 40S ribosomal protein S4, X isoform	0.8833	0.1729	0.4749	0.4557	No	0.8715	0.0292	0.52	0.0646	No	No
162245_f_at	Myo6	myosin VI	0.996	0.9537	0.1533	0.7286	No	0.9593	0.332	0.292	0.0349	No	No
162246_r_at	Gss	glutathione synthetase	1.0045	0.9338	0.7949	0.2388	No	1.0786	0.153	0.0216	0.0795	No	No
162247_r_at	Tcea2	Transcription elongation factor A (SII), 2	0.9192	0.3263	0.3934	0.8335	No	1.0127	0.703	0.0002	0.136	No	No
162248_f_at	Bphl	biphenyl hydrolase-like (serine hydrolase, breast epithelial mucin-associated antigen)	1.1054	0.0581	0.6645	0.4567	No	1.0868	0.191	0.0758	0.198	No	No
162249_f_at	2610200G18 Rik	RIKEN cDNA 2610200G18 gene	1.0525	0.1673	0.0235	0.1363	No	1.0808	0.415	0.542	0.941	No	No
162250_f_at	Zcchc6	Zinc finger, CCHC domain containing 6	1.0223	0.7117	0.0375	0.4587	No	0.9188	0.122	0.0017	0.344	No	No
162251_f_at	Centg2	centaurin, gamma 2	1.0818	0.1639	0.0032	0.0539	No	1.0404	0.278	0.0012	0.0678	No	No
162252_f_at	Plekha1	pleckstrin homology domain containing, family A (phosphoinositide binding specific) member 1	0.9274	0.0321	0.0001	0.6813	No	1.0117	0.804	0.0006	0.261	No	No
162253_i_at	Fgfr3	fibroblast growth factor receptor 3	1.1003	0.0825	0.0059	0.5159	No	1.1156	0.0263	0.0036	0.52	No	No
162254_f_at	Cyb5r1	cytochrome b5 reductase 1	0.968	0.3027	0.0115	0.7696	No	1.0093	0.942	0.235	0.452	No	No
162255_s_at	Scn1a	sodium channel, voltage-gated, type I, alpha	1.1184	0.262	0.0014	0.5359	No	1.1389	0.286	0.0008	0.927	No	No
162256_r_at	Cwf19l1	CWF19-like 1, cell cycle control (S. pombe)	1.0045	0.9397	0.2125	0.2703	No	1.0522	0.248	0.061	0.0757	No	No
162257_i_at	---	---	0.9845	0.8527	0.1316	0.9413	No	1.2006	0.0747	0.307	0.0396	No	No
162258_f_at	---	---	0.9865	0.5908	0.0031	0.2019	No	0.9997	0.944	0.014	0.889	No	No
162259_f_at	Fmo5	flavin containing monooxygenase 5	0.9032	0.4053	0.1428	0.1423	No	0.9387	0.324	0.271	0.0561	No	No
162260_at	6330407G11 Rik	RIKEN cDNA 6330407G11 gene	1.0484	0.2634	0.3845	0.0668	No	1.0006	0.873	0.0001	0.463	No	No
162261_f_at	Zp2	Zona pellucida glycoprotein 2	1.0656	0.4933	0.2968	0.422	No	1.0476	0.273	0.0075	0.411	No	No
162262_f_at	Gyg	glycogenin	0.9886	0.7548	0.0012	0.0036	No	0.8949	0.122	0.0625	0.0108	No	No
162263_f_at	Lamb1-1	laminin B1 subunit 1	0.9882	0.8866	0.0445	0.1949	No	1.0178	0.998	0.0094	0.29	No	No
162264_s_at	Bub1	budding uninhibited by benzimidazoles 1 homolog (S. cerevisiae)	0.8801	0.0796	0.1026	0.1733	No	0.9579	0.718	0.538	0.412	No	No
162265_r_at	Klf17	Kruppel-like factor 17	0.9662	0.3183	0.0866	0.5585	No	1.0324	0.638	0.871	0.0778	No	No
162266_f_at	---	---	1.1719	0.0713	0.7306	0.2442	No	1.1164	0.191	0.542	0.221	No	No
162267_r_at	Pdc	phosducin	0.9671	0.7351	0.0198	0.1242	No	1.0212	0.844	0.0003	0.0028	No	No
162268_at	Crygd	crystallin, gamma D	0.9839	0.8652	0.7182	0.401	No	1.1245	0.372	0.74	0.282	No	No
162269_at	Smyd2	SET and MYND domain containing 2	1.0617	0.2362	0.6766	0.8217	No	1.0797	0.172	0.0517	0.122	No	No
162270_r_at	Bag2	Bcl2-associated athanogene 2	1.0895	0.1833	0.5305	0.1052	No	1.3302	0.039	0.0839	0.0205	No	No
162271_f_at	Ctsb	cathepsin B	1.0695	0.1911	0.0785	0.4011	No	1.1316	0.0959	0.239	0.119	No	No
162272_r_at	Stxbp2	syntaxin binding protein 2	0.9445	0.2947	0.0324	0.2736	No	1.05	0.395	0.208	0.0351	No	No
162273_at	Repin1	Replication initiator 1	0.9017	0.0715	0.0111	0.3191	No	0.9104	0.0511	0.0056	0.281	No	No
162274_f_at	Lsr	lipolysis stimulated lipoprotein receptor	1.0781	0.4117	0.0234	0.6704	No	1.0142	0.804	0.0031	0.163	No	No
162275_f_at	Upf3b	UPF3 regulator of nonsense transcripts homolog B (yeast)	1.0179	0.7515	0.0462	0.7197	No	0.9772	0.728	0.0539	0.621	No	No
162276_i_at	C1qb	complement component 1, q subcomponent, beta polypeptide	1.0002	0.9966	0.0015	0.302	No	1.0045	0.791	0.0018	0.327	No	No
162277_r_at	Polg2	polymerase (DNA directed), gamma 2, accessory subunit	1.1074	0.3778	0.1054	0.2766	No	1.1432	0.509	0.0248	0.542	No	No
162278_r_at	Tap1	transporter 1, ATP-binding cassette, sub-family B (MDR/TAP)	1.0074	0.8488	0.0598	0.0273	No	1.1094	0.0935	0.0266	0.0343	No	No
162279_f_at	Cct6a	Chaperonin subunit 6a (zeta)	0.9997	0.9928	0.1548	0.0446	No	0.945	0.0632	0.0612	0.0042	No	No
162280_f_at	Ndufb10	NADH dehydrogenase (ubiquinone) 1 beta subcomplex, 10	0.9311	0.4218	0.695	0.3692	No	0.9753	0.718	0.426	0.695	No	No
162281_at	Fdps /// LOC544723 /// LOC671145	farnesyl diphosphate synthetase /// similar to farnesyl diphosphate synthetase /// similar to farnesyl diphosphate synthetase	0.8966	0.049	0.5118	0.2938	No	0.8789	0.0563	0.0936	0.808	No	No
162282_f_at	Tyro3	TYRO3 protein tyrosine kinase 3	0.9583	0.5246	0.2617	0.6999	No	1.0564	0.11	0.117	0.0545	No	No
162283_r_at	Eif3s6ip	eukaryotic translation initiation factor 3, subunit 6 interacting protein	1.0396	0.1191	0.2864	0.1195	No	1.1085	0.24	0.267	0.56	No	No
162284_r_at	Slc39a4	solute carrier family 39 (zinc transporter), member 4	1.0116	0.6434	0.0191	0.548	No	1.0192	0.702	0.78	0.79	No	No
162285_r_at	Slc44a4	solute carrier family 44, member 4	1.0319	0.6758	0.9864	0.3806	No	1.2439	0.126	0.0812	0.193	No	No
162286_r_at	Fcgbp	Fc fragment of IgG binding protein	1.0739	0.2321	0.0566	0.7746	No	0.8133	0.0879	0.0819	0.133	No	No
162287_r_at	Clca3	chloride channel calcium activated 3	1.114	0.1873	0.4998	0.0442	No	1.167	0.323	0.797	0.101	No	No
162288_f_at	Pcx	pyruvate carboxylase	0.9821	0.8657	0.5716	0.7632	No	1.0791	0.225	0.183	0.0394	No	No
162289_at	Fgl2	fibrinogen-like protein 2	1.1159	0.1143	0.1141	0.442	No	1.3271	0.0524	0.435	0.993	No	No
162290_f_at	---	---	0.9997	0.9919	0.3564	0.0806	No	0.9893	0.939	0.329	0.761	No	No
162291_r_at	8430437G11 Rik	RIKEN cDNA 8430437G11 gene	1.0017	0.9674	0.4493	0.2536	No	0.8308	0.143	0.0958	0.0278	No	No
162292_r_at	---	---	0.994	0.7384	0.002	0.0934	No	1.0073	0.868	0.0012	0.0253	No	No
162293_r_at	---	---	0.9693	0.5727	0.1617	0.3425	No	1.0019	0.896	0.309	0.0524	No	No
162294_f_at	Maoa	monoamine oxidase A	1.0073	0.8307	0.2987	0.1146	No	0.9146	0.335	0.507	0.117	No	No
162295_at	---	---	1.0249	0.6178	0.3001	0.3381	No	0.9844	0.748	0.04	0.642	No	No

162296_at	Bglap-rs1 /// Bglap1 /// Bglap2	bone gamma-carboxyglutamate protein, related sequence 1 /// bone gamma carboxyglutamate protein 1 /// bone gamma-carboxyglutamate protein 2	0.9561	0.5809	0.3495	0.6632	No	1.0649	0.334	0.101	0.384	No	No
162297_s_at	Cav3	caveolin 3	0.7059	0.0142	0.0878	0.0072	No	0.7524	0.0101	0.167	0.0021	No	No
162298_r_at	Tjp1	tight junction protein 1	0.9827	0.4003	0.0017	0.8623	No	0.8792	0.124	0.28	0.0723	No	No
162299_f_at	Pebp1	phosphatidylethanolamine binding protein 1	1.0597	0.4356	0.0828	0.9466	No	1.0688	0.0696	0.0147	0.38	No	No
162300_at	Pcp4	Purkinje cell protein 4	0.9316	0.5248	0.7406	0.9548	No	0.909	0.343	0.0683	0.191	No	No
162301_f_at	2310016M24Rik	RIKEN cDNA 2310016M24 gene	1.0845	0.2549	0.609	0.5883	No	1.014	0.946	0.0774	0.0628	No	No
162302_f_at	Folr1	folate receptor 1 (adult)	1.0465	0.5394	0.2489	0.6807	No	1.1062	0.177	0.498	0.194	No	No
162303_f_at	Prlpf	prolactin-like protein F	1.0169	0.6522	0.1331	0.1663	No	1.1889	0.0167	0.105	0.377	No	No
162304_r_at	Phf7	PHD finger protein 7	0.896	0.1068	0.4526	0.891	No	0.8015	0.0367	0.125	0.284	No	No
162305_f_at	Klk1b9 /// Klk1b1	kallikrein 1-related peptidase b9 /// kallikrein 1-related peptidase b1	0.9249	0.3151	0.2022	0.7189	No	1.0111	0.727	0.0226	0.603	No	No
162306_at	Ubxd5	UBX domain containing 5	0.985	0.8746	0.0172	0.3412	No	1.0345	0.811	0.294	0.598	No	No
162307_at	Polk	polymerase (DNA directed), kappa	1.0348	0.4224	0.0623	0.0908	No	1.0971	0.0699	0.0448	0.0046	No	No
162308_f_at	Cryab	crystallin, alpha B	0.9177	0.0744	0.0271	0.1621	No	0.9428	0.0525	0.0327	0.0099	No	No
162309_at	Lyzs /// Lzp-s	lysozyme /// P lysozyme structural	1.0347	0.6552	0.1601	0.1324	No	0.9076	0.19	0.879	0.0373	No	No
162310_r_at	G630024C07Rik	RIKEN cDNA G630024C07 gene	0.9605	0.3862	0.023	0.2029	No	0.9672	0.605	0.0041	0.132	No	No
162311_f_at	---	---	1.0438	0.311	0.1686	0.2731	No	1.0423	0.143	0.106	0.0925	No	No
162312_f_at	Reg1	regenerating islet-derived 1	1.0972	0.3046	0.2961	0.6876	No	1.1187	0.401	0.394	0.153	No	No
162313_f_at	---	---	1.0478	0.3861	0.0107	0.2064	No	1.029	0.695	0.28	0.381	No	No
162314_at	Cnot7	CCR4-NOT transcription complex, subunit 7	0.9974	0.9422	0.0334	0.2009	No	1.0173	0.455	0.0268	0.386	No	No
162315_f_at	Cldn3	claudin 3	0.9741	0.6229	0.0065	0.1213	No	0.976	0.6	0.0806	0.191	No	No
162316_f_at	Dgat1	diacylglycerol O-acyltransferase 1	0.9954	0.9363	0.6081	0.7024	No	0.8978	0.13	0.0828	0.419	No	No
162317_r_at	---	---	1.0793	0.0944	0.017	0.0775	No	1.0082	0.883	0.0003	0.38	No	No
162318_r_at	---	---	1.2412	0.0571	0.0145	0.0253	No	1.2715	0.0308	0.014	0.11	No	No
162319_i_at	---	---	1.1465	0.1103	0.0378	0.1944	No	1.1371	0.0474	0.0091	0.0459	No	No
162320_at	---	---	1.0598	0.1194	0.012	0.009	No	0.995	0.94	0.0024	0.195	No	No
162321_f_at	Barx1	BarH-like homeobox 1	1.0618	0.0755	0.0018	0.5409	No	1.0392	0.361	0.0038	0.764	No	No
162322_r_at	Krt1-13	Keratin complex 1, acidic, gene 13	1.0469	0.5874	0.4397	0.0392	No	1.1544	0.0349	0.623	0.0256	No	No
162323_at	Ndufb7	NADH dehydrogenase (ubiquinone) 1 beta subcomplex, 7	1.0034	0.9598	0.6184	0.836	No	0.9633	0.722	0.965	0.769	No	No
162324_at	---	---	1.004	0.9044	0.0359	0.1366	No	1.0795	0.305	0.126	0.0709	No	No
162325_f_at	Tnnc2	troponin C2, fast	0.7704	0.0064	0.0087	0.0023	No	0.7948	0.0003	0.0022	0.0001	No	No
162326_at	---	---	1.0857	0.0149	0.001	0.0823	No	1.0108	0.748	0.046	0.749	No	No
162327_f_at	Ndufv2	NADH dehydrogenase (ubiquinone) flavoprotein 2	1.0963	0.4075	0.7606	0.7273	No	0.9807	0.94	0.082	0.0622	No	No
162328_f_at	Krt2-16	keratin complex 2, basic, gene 16	0.996	0.9243	0.1073	0.1236	No	1.0446	0.0093	0.001	0.0003	No	No
162329_r_at	Slpi	secretory leukocyte peptidase inhibitor	1.1507	0.0663	0.0748	0.0963	No	1.0984	0.186	0.038	0.092	No	No
162330_f_at	---	---	0.9506	0.1707	0.276	0.5428	No	1.0048	0.989	0.0076	0.772	No	No
162331_f_at	Aldh3a1	aldehyde dehydrogenase family 3, subfamily A1	1.0048	0.8517	0.0718	0.3065	No	1.0691	0.317	0.0455	0.0937	No	No
162332_f_at	Mapre3	microtubule-associated protein, RP/EB family, member 3	0.9411	0.0714	0.0003	0.1084	No	0.9409	0.0756	0.0003	0.952	No	No
162333_r_at	Slpi	secretory leukocyte peptidase inhibitor	1.0782	0.2348	0.4062	0.2424	No	1.1625	0.0881	0.0711	0.0427	No	No
162334_r_at	Llgl1	Lethal giant larvae homolog 1 (Drosophila)	0.8201	0.1196	0.6306	0.4372	No	0.9405	0.456	0.609	0.499	No	No
162335_at	Rps29 /// LOC435996 /// LOC620161 /// LOC621967 /// LOC632013 /// LOC635561 /// LOC640377 /// LOC665395 /// LOC675444	ribosomal protein S29 /// similar to 40S ribosomal protein S29 /// similar to 40S ribosomal protein S29 /// similar to 40S ribosomal protein S29 /// similar to 40S ribosomal protein S29 /// similar to 40S ribosomal protein S29 /// similar to 40S ribosomal protein S29 /// similar to 40S ribosomal protein S29 /// similar to 40S ribosomal protein S29	0.9214	0.1955	0.0219	0.2017	No	0.9691	0.777	0.0162	0.709	No	No
162336_r_at	Acp6	Acid phosphatase 6, lysophosphatidic	0.9843	0.8047	0.0691	0.1232	No	1.0181	0.599	0.0018	0.361	No	No
162337_f_at	Alg3	Asparagine-linked glycosylation 3 homolog (yeast, alpha-1,3-mannosyltransferase)	1.0835	0.127	0.1003	0.2118	No	1.1151	0.0957	0.221	0.132	No	No
162338_r_at	---	---	0.9987	0.9758	0.9313	0.3551	No	1.0082	0.788	0.219	0.325	No	No
162339_r_at	Cdc34	cell division cycle 34 homolog (S. cerevisiae)	1.0975	0.0135	0.0007	0.058	No	1.1383	0.0143	0.0246	0.0442	No	No
162340_r_at	S100a10	S100 calcium binding protein A10 (calpactin)	0.9697	0.4843	0.1256	0.9374	No	1.0229	0.602	0.732	0.578	No	No
162341_r_at	Akr1b3	aldo-keto reductase family 1, member B3 (aldose reductase)	1.0116	0.6154	0.104	0.0192	No	1.0911	0.0284	0.214	0.296	No	No
162342_at	Fabp1	Fatty acid binding protein 1, liver	1.0516	0.2623	0.0176	0.3299	No	1.0629	0.455	0.0458	0.748	No	No
162343_f_at	Atp5d	ATP synthase, H+ transporting, mitochondrial F1 complex, delta subunit	0.9861	0.7332	0.008	0.0294	No	0.9934	0.948	0.0044	0.0975	No	No
162344_at	---	---	1.0851	0.2628	0.0102	0.3274	No	1.1963	0.298	0.018	0.317	No	No
162345_at	1110059E24Rik	RIKEN cDNA 1110059E24 gene	1.0199	0.6826	0.0004	0.982	No	1.0683	0.261	0.0001	0.393	No	No
162346_f_at	H2-DMa	histocompatibility 2, class II, locus DMa	1.0118	0.5752	0.0197	0.0122	No	1.0508	0.197	0.0245	0.335	No	No
162347_f_at	Bgn	biglycan	1.0069	0.3084	0.0331	0.4298	No	1.0135	0.688	0.642	0.61	No	No
162348_r_at	Cpa3	carboxypeptidase A3, mast cell	0.9197	0.3426	0.0532	0.8182	No	0.9002	0.119	0.0054	0.0719	No	No
162349_i_at	---	---	1.0645	0.4739	0.0368	0.3113	No	1.0577	0.509	0.0918	0.792	No	No
162350_at	D330001F17Rik	RIKEN cDNA D330001F17 gene	1.0215	0.3203	0.0013	0.1327	No	0.9588	0.303	0.253	0.202	No	No
162351_f_at	Ccs	copper chaperone for superoxide dismutase	0.9697	0.401	0.0175	0.3868	No	0.979	0.673	0.0486	0.534	No	No
162352_r_at	Rprm	reprimo, TP53 dependent G2 arrest mediator candidate	0.9845	0.6718	0.9533	0.101	No	1.1537	0.0669	0.0563	0.0025	No	No
162353_at	---	---	1.1274	0.0223	0.3104	0.1934	No	1.0515	0.391	0.971	0.582	No	No

162354_f_at	Igfbpl1	insulin-like growth factor binding protein-like 1	0.9225	0.279	0.0037	0.1287	No	0.8926	0.315	0.0001	0.0345	No	No
162355_at	---	---	1.0237	0.4567	0.6144	0.0127	No	0.9752	0.685	0.843	0.0606	No	No
162356_r_at	1810042K04 Rik	RIKEN cDNA 1810042K04 gene	1.0084	0.8633	0.0326	0.461	No	1.0311	0.454	0.252	0.0949	No	No
162357_at	Ebi3	Epstein-Barr virus induced gene 3	0.9533	0.1758	0.4863	0.0317	No	0.859	0.0506	0.18	0.0938	No	No
162358_i_at	Slc25a1	solute carrier family 25 (mitochondrial carrier, citrate transporter), member 1	1.0522	0.016	0	0.033	No	1.1426	0.0299	0.001	0.491	No	No
162359_r_at	Gkap1	G kinase anchoring protein 1	0.9806	0.3157	0.2065	0.0107	No	1.0906	0.0352	0.0013	0.672	No	No
162360_f_at	1110061O04 Rik	RIKEN cDNA 1110061O04 gene	0.8562	0.1215	0.5406	0.367	No	0.9003	0.252	0.2	0.4	No	No
162361_at	Sdsl	serine dehydratase-like	1.0183	0.6343	0.0091	0.2906	No	1.0536	0.451	0.0341	0.653	No	No
162362_f_at	Tnc	tenascin C	0.8598	0.1424	0.0195	0.1963	No	1.0014	0.547	0.0173	0.0481	No	No
162363_at	---	---	0.9767	0.7448	0.0633	0.7052	No	0.95	0.46	0.0641	0.601	No	No
162364_f_at	Glud1	glutamate dehydrogenase 1	0.9671	0.477	0.003	0.4733	No	1.2145	0.0859	0.0135	0.223	No	No
162365_i_at	---	---	0.8909	0.1053	0.1555	0.2493	No	0.8865	0.106	0.768	0.164	No	No
162366_r_at	C2	complement component 2 (within H-2S)	0.9889	0.8789	0.0607	0.5807	No	0.97	0.696	0.0399	0.65	No	No
162367_f_at	Seh1l	SEH1-like (S. cerevisiae	1.0553	0.1301	0.7156	0.0171	No	1.2163	0.0367	0.0018	0.0782	No	No
162368_r_at	---	---	1.1814	0.1609	0.0864	0.0256	No	1.2602	0.0012	0.113	0.0004	No	No
162369_f_at	Mmp9	matrix metallopeptidase 9	1.0149	0.8403	0.0553	0.6362	No	1.0208	0.751	0.0013	0.818	No	No
162370_r_at	---	---	1.0765	0.0133	0.0779	0.0008	No	1.0333	0.051	0.0009	0.001	No	No
162371_r_at	Ephb6	Eph receptor B6	0.9431	0.2485	0.4321	0.5374	No	0.9475	0.182	0.0099	0.406	No	No
162372_f_at	Relb	avian reticuloendotheliosis viral (v-rel) oncogene related B	0.9642	0.3852	0.0023	0.1395	No	0.9576	0.189	0.0016	0.564	No	No
162373_r_at	Sh3bp2	SH3-domain binding protein 2	0.9776	0.4475	0	0.0652	No	0.93	0.0489	0	0.13	No	No
162374_r_at	Myh8	myosin, heavy polypeptide 8, skeletal muscle, perinatal	1.0083	0.9248	0.0022	0.7749	No	0.9179	0.054	0.0159	0.0158	No	No
162375_i_at	Krt2-19	keratin complex 2, basic, gene 19	1.0732	0.3543	0.9066	0.0944	No	1.2535	0.249	0.587	0.0909	No	No
162376_r_at	---	---	1.0162	0.7327	0.2986	0.092	No	1.0926	0.405	0.867	0.0641	No	No
162377_f_at	Ssbp4	single stranded DNA binding protein 4	0.9814	0.7523	0.1121	0.7696	No	1.0309	0.573	0.295	0.344	No	No
162378_r_at	8430437G11 Rik	RIKEN cDNA 8430437G11 gene	0.9942	0.8951	0.144	0.1957	No	1.0422	0.762	0.0273	0.517	No	No
162379_r_at	---	---	1.0134	0.6928	0.1669	0.0183	No	0.9983	0.989	0.766	0.551	No	No
162380_r_at	LOC637741	hypothetical protein LOC637741	0.9872	0.6945	0.1917	0.8706	No	0.9679	0.417	0.454	0.292	No	No
162381_f_at	Sphk2	Sphingosine kinase 2	1.0278	0.5314	0.2621	0.2458	No	0.949	0.118	0.0524	0.44	No	No
162382_f_at	Ccs	copper chaperone for superoxide dismutase	1.1312	0.1218	0.4001	0.7598	No	1.0369	0.643	0.825	0.759	No	No
162383_r_at	---	---	1.0837	0.2095	0.1585	0.4708	No	1.1017	0.252	0.221	0.426	No	No
162384_f_at	Ccrn4l	CCR4 carbon catabolite repression 4-like (S. cerevisiae)	0.9228	0.4407	0.0556	0.0803	No	1.1204	0.0602	0	0.0211	No	No
162385_i_at	Slc2a4	solute carrier family 2 (facilitated glucose transporter), member 4	1.0331	0.5348	0.0776	0.8692	No	1.1198	0.185	0.226	0.531	No	No
162386_at	---	---	0.9257	0.4249	0.2073	0.6577	No	0.9506	0.594	0.185	0.318	No	No
162387_f_at	Mfn1	mitofusin 1	1.0291	0.5901	0.0378	0.3357	No	1.1286	0.209	0.0098	0.179	No	No
162388_r_at	Adam5	a disintegrin and metallopeptidase domain 5	0.889	0.0393	0.1824	0.0627	No	0.9352	0.227	0.104	0.08	No	No
162389_at	D14Ertd209 e	DNA segment, Chr 14, ERATO Doi 209, expressed	0.9416	0.4854	0.3614	0.3206	No	0.9698	0.747	0.349	0.743	No	No
162390_r_at	C4b /// C4a /// LOC672587 /// LOC675521	complement component 4B (Childo blood group) /// complement component 4A (Rodgers blood group) /// similar to sex-limited protein /// similar to Complement C4 precursor	1.0075	0.9297	0.3169	0.9461	No	1.3377	0.0211	0.352	0.364	No	No
162391_r_at	Ltc4s	leukotriene C4 synthase	0.9797	0.5823	0.5836	0.0189	No	1.1702	0.064	0.0229	0.0085	No	No
162392_r_at	Ift88	intraflagellar transport 88 homolog (Chlamydomonas)	1.0124	0.7682	0.2024	0.0427	No	1.0427	0.269	0.349	0.0251	No	No
162393_at	---	---	1.04	0.3988	0.0853	0.0418	No	1.0651	0.239	0.188	0.0449	No	No
162394_r_at	Jak3	Janus kinase 3	1.0009	0.9921	0.6682	0.687	No	1.0261	0.761	0.302	0.759	No	No
162395_r_at	---	---	1.0594	0.4414	0.0536	0.0685	No	1.1695	0.227	0.0797	0.419	No	No
162396_at	---	---	1.0751	0.2897	0.4092	0.0523	No	1.1408	0.102	0.51	0.0369	No	No
162397_r_at	Ptbp1	polypyrimidine tract binding protein 1	0.8497	0.0958	0.0001	0.3443	No	1.0268	0.613	0.0563	0.469	No	No
162398_at	---	---	1.0729	0.0974	0.0026	0.8905	No	1.0167	0.477	0.0001	0.431	No	No
162399_f_at	Atxn2	ataxin 2	0.9821	0.8083	0.0002	0.0358	No	1.0612	0.678	0.0001	0.0231	No	No
162400_f_at	Mms19l	MMS19 (MET18 S. cerevisiae)-like	1.0301	0.5658	0.0229	0.2931	No	1.0799	0.533	0.0246	0.113	No	No
162401_f_at	Isyna1	myo-inositol 1-phosphate synthase A1	1.012	0.8978	0.0101	0.1827	No	0.996	0.908	0.0031	0.103	No	No
162402_r_at	Hoxa4	homeo box A4	1.0448	0.5908	0.0593	0.0864	No	1.1042	0.0377	0.0021	0.0041	No	No
162403_at	---	---	1.0703	0.5448	0.4438	0.205	No	1.0556	0.445	0.114	0.177	No	No
162404_i_at	---	---	0.9766	0.8001	0.1747	0.895	No	1.0357	0.747	0.221	0.401	No	No
162405_at	2810449G22 Rik	RIKEN cDNA 2810449G22 gene	0.9815	0.7494	0.2461	0.1373	No	1.1018	0.299	0.329	0.0741	No	No
162406_f_at	Stk38	serine/threonine kinase 38	0.9641	0.3523	0.1078	0.9057	No	0.9122	0.356	0.0287	0.56	No	No
162407_at	Traip	TRAF-interacting protein	1.0529	0.0279	0.0004	0.0273	No	0.9595	0.0241	0.0005	0.0129	No	No
162408_f_at	Il12rb1	interleukin 12 receptor, beta 1	0.9591	0.3912	0.0067	0.6513	No	1.0044	0.975	0.0006	0.502	No	No
162409_r_at	Zfml	zinc finger, matrin-like	1.1372	0.2234	0.2879	0.0847	No	1.1683	0.165	0.0738	0.0583	No	No
162410_s_at	Cd8b1	CD8 antigen, beta chain 1	0.9195	0.1495	0.1972	0.0473	No	0.9466	0.346	0.957	0.289	No	No
162411_f_at	Myb	myeloblastosis oncogene	1.0216	0.7453	0.7162	0.0393	No	1.0278	0.529	0.121	0.0184	No	No
162412_r_at	---	---	0.9515	0.4602	0.8259	0.922	No	1.9062	0.236	0.273	0.461	No	No
162413_r_at	Nr0b1	nuclear receptor subfamily 0, group B, member 1	1.1994	0.1285	0.345	0.0634	No	1.1179	0.116	0.0198	0.0203	No	No
162414_f_at	Fkbp10	FK506 binding protein 10	1.0179	0.7822	0.0469	0.9755	No	1.0214	0.817	0.0131	0.455	No	No
162415_f_at	Cant1	calcium activated nucleotidase 1	0.9232	0.0797	0.1177	0.221	No	0.9071	0.0394	0.123	0.045	No	No
162416_at	Cdca4	cell division cycle associated 4	1.068	0.3644	0.295	0.0429	No	1.2764	0.0455	0.0128	0.0505	No	No
162417_at	1500001M20 Rik	RIKEN cDNA 1500001M20 gene	0.9347	0.18	0.3853	0.5884	No	0.9396	0.0572	0.305	0.664	No	No

162418_r_at	Atad3a	ATPase family, AAA domain containing 3A	1.0944	0.0662	0.0261	0.1194	No	0.95	0.402	0.0453	0.431	No	No
162419_r_at	---	---	1.0498	0.1765	0.008	0.0097	No	1.3102	0.0048	0.0006	0.0035	No	No
162420_r_at	Selk	selenoprotein K	0.9935	0.9448	0.0189	0.6522	No	0.9021	0.133	0.0175	0.051	No	No
162421_r_at	Ctla2a	cytotoxic T lymphocyte-associated protein 2 alpha	1.0757	0.059	0.3	0.4018	No	1.0607	0.61	0.0837	0.591	No	No
162422_r_at	---	---	1.0055	0.836	0.3775	0.4296	No	1.0094	0.543	0.269	0.0438	No	No
162423_f_at	Nbr1	neighbor of Brca1 gene 1	1.1452	0.1719	0.0002	0.1429	No	1.1195	0.605	0.0001	0.287	No	No
162424_f_at	Ddx17	DEAD (Asp-Glu-Ala-Asp) box polypeptide 17	1.163	0.0677	0.001	0.2427	No	1.0584	0.198	0.465	0.0801	No	No
162425_f_at	Irf3	interferon regulatory factor 3	1.0362	0.4668	0.0607	0.0689	No	1.0282	0.871	0.127	0.178	No	No
162426_f_at	Scamp1	secretory carrier membrane protein 1	0.9746	0.3763	0.0016	0.0251	No	0.9776	0.384	0.141	0.104	No	No
162427_r_at	Efhd2	EF hand domain containing 2	1.1498	0.0098	0.0041	0.0009	No	1.1614	0.0304	0.473	0.0041	No	No
162428_i_at	S100a14	S100 calcium binding protein A14	1.0127	0.8091	0.2273	0.8443	No	1.0164	0.694	0.0487	0.727	No	No
162429_r_at	Serpinf1	serine (or cysteine) peptidase inhibitor, clade F, member 1	1.0134	0.758	0.0179	0.3017	No	1.0341	0.0519	0.0002	0.906	No	No
162430_at	---	---	1.0963	0.1476	0.0045	0.4999	No	1.0133	0.809	0.438	0.687	No	No
162431_at	---	---	1.0404	0.3739	0.0369	0.4862	No	1.0024	0.997	0.0168	0.444	No	No
162432_f_at	Mogat2	monoacylglycerol O-acyltransferase 2	0.8805	0.1831	0.0849	0.7777	No	1.0422	0.313	0.0129	0.157	No	No
162433_f_at	Nol5a /// LOC383425 /// LOC627681 /// LOC639387	nucleolar protein 5A /// hypothetical gene supported by AK037405; NM_024193 /// similar to Nucleolar protein Nop56 (Nucleolar protein 5A) /// hypothetical LOC639387	1.0107	0.8468	0.1387	0.096	No	0.9887	0.826	0.48	0.0416	No	No
162434_i_at	---	---	1.0241	0.6843	0.1037	0.0868	No	1.0024	0.678	0.0139	0.0314	No	No
162435_f_at	Fez1	fasciculation and elongation protein zeta 1 (zygin I)	1.0453	0.1317	0.5156	0.0115	No	0.9937	0.837	0.0055	0.823	No	No
162436_at	Mrpl46	mitochondrial ribosomal protein L46	1.0146	0.803	0.0045	0.329	No	0.9702	0.723	0.0844	0.521	No	No
162437_at	---	---	1.0833	0.4645	0.2539	0.2022	No	1.0724	0.321	0.082	0.0486	No	No
162438_f_at	D15Wsu75e	DNA segment, Chr 15, Wayne State University 75, expressed	0.9782	0.6082	0.1053	0.673	No	1.1594	0.122	0.168	0.115	No	No
162439_at	Pou2af1	POU domain, class 2, associating factor 1	0.9066	0.4311	0.354	0.5447	No	0.8775	0.192	0.141	0.248	No	No
162440_r_at	---	---	0.9654	0.3674	0.0099	0.1474	No	1.0324	0.235	0.0005	0.124	No	No
162441_i_at	Gaa	glucosidase, alpha, acid	1.0561	0.4198	0.1566	0.7345	No	1.0687	0.423	0.0308	0.24	No	No
162442_r_at	---	---	1.0741	0.3214	0.316	0.3424	No	1.0603	0.455	0.14	0.0949	No	No
162443_at	Tcp1	t-complex protein 1	1.102	0.0377	0.713	0.0527	No	1.0986	0.146	0.991	0.607	No	No
162444_r_at	Tinf2	Terf1 (TRF1)-interacting nuclear factor 2	1.232	0.158	0.0092	0.055	No	1.2195	0.0581	0.0046	0.0428	No	No
162445_at	---	---	0.9676	0.3359	0.0807	0.3496	No	0.9545	0.18	0.0287	0.0332	No	No
162446_at	Chfr	checkpoint with forkhead and ring finger domains	1.0061	0.9262	0.4712	0.3836	No	1.0828	0.332	0.284	0.11	No	No
162447_f_at	Mvp	Major vault protein	0.9957	0.8447	0.1238	0.2153	No	0.9858	0.501	0.0755	0.0461	No	No
162448_f_at	Hspb8	heat shock protein 8	0.8884	0.0805	0.0067	0.1249	No	0.9118	0.115	0.0066	0.0747	No	No
162449_f_at	Admr	Adrenomedullin receptor	0.9326	0.1884	0.0148	0.27	No	0.909	0.187	0.012	0.0407	No	No
162450_f_at	Igfals	insulin-like growth factor binding protein, acid labile subunit	1.0252	0.7366	0.0068	0.2404	No	1.0203	0.416	0.002	0.0558	No	No
162451_r_at	Fbxo3	F-box only protein 3	1.0084	0.8311	0.5517	0.5298	No	0.2122	0.532	0.574	0.689	No	No
162452_at	Pcp2	Purkinje cell protein 2 (L7)	1.077	0.0466	0.0841	0.0693	No	1.1104	0.0081	0.0633	0.0065	No	No
162453_at	---	---	0.9892	0.7875	0.122	0.7169	No	1.0127	0.813	0.394	0.748	No	No
162454_f_at	---	---	1.0117	0.8562	0.204	0.9986	No	1.0842	0.19	0.308	0.34	No	No
162455_f_at	1110039B18Rik	RIKEN cDNA 1110039B18 gene	1.0124	0.7913	0.2227	0.444	No	0.9627	0.314	0.0711	0.367	No	No
162456_f_at	Klk1b21 /// Klk1b24	kallikrein 1-related peptidase b21 /// kallikrein 1-related peptidase b24	1.0257	0.4152	0.0003	0.757	No	0.9415	0.31	0.0029	0.468	No	No
162457_f_at	Hba-a1	hemoglobin alpha, adult chain 1	0.9916	0.8819	0.0052	0.8365	No	0.961	0.252	0.0021	0.349	No	No
162458_i_at	Shmt2	serine hydroxymethyl transferase 2 (mitochondrial)	1.036	0.4981	0.5431	0.1442	No	1.0878	0.0168	0.438	0.0269	No	No
162459_f_at	Col6a1	procollagen, type VI, alpha 1	0.9981	0.9786	0.16	0.5235	No	1.047	0.828	0.583	0.986	No	No
162460_f_at	Igbp1	immunoglobulin (CD79A) binding protein 1	1.084	0.2664	0.0056	0.9545	No	1.0426	0.808	0.0121	0.0815	No	No
162461_at	---	---	0.9989	0.9849	0.0152	0.171	No	1.0927	0.218	0.175	0.0298	No	No
162462_r_at	Pck2	phosphoenolpyruvate carboxykinase 2 (mitochondrial)	0.8571	0.1827	0.0693	0.0699	No	0.8791	0.0069	0	0.0035	No	No
162463_at	Tpd52	tumor protein D52	1.2348	0.0791	0.0801	0.1066	No	1.1544	0.0533	0.611	0.0938	No	No
162464_f_at	Ppy	Pancreatic polypeptide	1.0949	0.1656	0.5845	0.0568	No	1.1266	0.104	0.806	0.0578	No	No
162465_i_at	Klhdc4	kelch domain containing 4	0.9986	0.9632	0.5167	0.3311	No	1.0767	0.0345	0.001	0.0047	No	No
162466_at	Anapc13	anaphase promoting complex subunit 13	1.0539	0.1343	0.0032	0.356	No	1.0575	0.385	0.0791	0.331	No	No
162467_r_at	2700078K21Rik	RIKEN cDNA 2700078K21 gene	0.9668	0.748	0.0855	0.7964	No	1.0062	0.978	0.0405	0.897	No	No
162468_at	Rhoc	ras homolog gene family, member C	0.9142	0.0079	0.0023	0.0318	Yes	0.9746	0.431	0.0874	0.963	No	No
162469_r_at	Cyc1	cytochrome c-1	0.8933	0.0603	0.0135	0.5652	No	0.8588	0.0593	0.0091	0.575	No	No
162470_at	---	---	1.0659	0.1498	0.0085	0.2858	No	1.1524	0.0049	0.0006	0.69	Yes	No
162471_i_at	1110019N10Rik	RIKEN cDNA 1110019N10 gene	0.9411	0.3732	0.6645	0.1908	No	1.0124	0.936	0.469	0.825	No	No
162472_f_at	Gast	gastrin	0.9155	0.32	0.0884	0.5508	No	0.9399	0.502	0.0576	0.75	No	No
162473_r_at	Ndufb2	NADH dehydrogenase (ubiquinone) 1 beta subcomplex, 2	1.0809	0.4487	0.2073	0.2344	No	1.1424	0.104	0.188	0.37	No	No
162474_f_at	---	---	1.0429	0.5493	0.0135	0.5745	No	1.0235	0.715	0.0001	0.242	No	No
162475_f_at	Pglyrp1	peptidoglycan recognition protein 1	0.9064	0.0364	0.0001	0.0018	No	0.9498	0.145	0.0002	0.368	No	No
162476_r_at	---	---	1.1688	0.1305	0.005	0.8216	No	1.0937	0.404	0.0009	0.335	No	No
162477_r_at	Srp14	signal recognition particle 14	0.9995	0.9953	0.0214	0.9264	No	1.0097	0.996	0.0007	0.984	No	No
162478_r_at	Idh3g	isocitrate dehydrogenase 3 (NAD+), gamma	1.0443	0.5156	0.6624	0.3832	No	1.0257	0.656	0.512	0.248	No	No
162479_f_at	---	---	1.0515	0.3158	0.0223	0.2029	No	1.058	0.117	0.334	0.494	No	No
162480_f_at	Lefty1	left right determination factor 1	0.9859	0.757	0.0132	0.4155	No	1.0406	0.596	0.0626	0.789	No	No
162481_f_at	Arrb1	arrestin, beta 1	1.0186	0.3971	0.0019	0.0424	No	1.0712	0.301	0.0609	0.507	No	No
162482_at	---	---	1.0112	0.6166	0.0014	0.6465	No	1.0605	0.147	0.016	0.67	No	No
162483_f_at	Col18a1	Procollagen, type XVIII, alpha 1	1.0753	0.3935	0.1766	0.9546	No	1.1022	0.431	0.345	0.718	No	No

162484_r_at	Tmed1	transmembrane emp24 domain containing 1	1.0476	0.4951	0.2139	0.7531	No	0.9486	0.228	0.0858	0.815	No	No
162485_at	Zfp91	zinc finger protein 91	0.9879	0.9297	0.0165	0.4985	No	1.3171	0.751	0	0.0329	No	No
162486_f_at	Dscr3	Down syndrome critical region gene 3	0.8442	0.0753	0.8939	0.0416	No	0.9719	0.568	0.0225	0.181	No	No
162487_f_at	C1qtnf1	C1q and tumor necrosis factor related protein 1	1.0906	0.0646	0.9163	0.5219	No	1.0714	0.152	0.784	0.331	No	No
162488_at	Hexa	hexosaminidase A	1.0431	0.3113	0.9808	0.1856	No	1.2372	0.101	0.931	0.859	No	No
162489_i_at	Ddx47	DEAD (Asp-Glu-Ala-Asp) box polypeptide 47	0.993	0.7547	0.2561	0.3973	No	0.9923	0.75	0.201	0.0331	No	No
162490_f_at	Pdcd6	Programmed cell death 6	1.2269	0.0004	0.0007	0.1977	Yes	1.2697	0.0165	0.0141	0.0263	No	Yes
162491_f_at	Prr6	proline-rich polypeptide 6	1.0085	0.7693	0.2202	0.9148	No	1.0224	0.463	0.0023	0.881	No	No
162492_at	Zipro1	zinc finger proliferation 1	0.9674	0.6976	0.0867	0.4692	No	1.0237	0.632	0.205	0.612	No	No
162493_r_at	---	---	1.1487	0.3125	0.081	0.6643	No	1.1625	0.112	0.994	0.221	No	No
162494_at	Pdlim4	PDZ and LIM domain 4	1.0168	0.6835	0.1105	0.23	No	1.0638	0.853	0.0061	0.236	No	No
162495_r_at	Bet1	Blocked early in transport 1 homolog (S. cerevisiae)	1.0146	0.8394	0.2565	0.792	No	0.8345	0.273	0.142	0.51	No	No
162496_r_at	---	---	1	0.9996	0.5617	0.0629	No	0.9591	0.433	0.452	0.355	No	No
162497_r_at	Ube1x	ubiquitin-activating enzyme E1, Chr X	1.0806	0.0088	0.0216	0.0049	No	0.9915	0.491	0.0944	0.082	No	No
162498_r_at	Rps4x /// LOC225134 /// LOC229746 /// LOC434180 /// LOC546380 /// LOC637070 /// LOC667398 /// LOC668668 /// LOC670119 /// LOC672743 /// LOC674228	ribosomal protein S4, X-linked /// similar to 40S ribosomal protein S4, X isoform /// similar to 40S ribosomal protein S4, X isoform /// similar to 40S ribosomal protein S4, X isoform /// similar to 40S ribosomal protein S4, X isoform /// similar to 40S ribosomal protein S4, X isoform /// similar to 40S ribosomal protein S4, X isoform /// similar to 40S ribosomal protein S4, X isoform /// similar to 40S ribosomal protein S4, X isoform /// similar to 40S ribosomal protein S4, X isoform /// similar to 40S ribosomal protein S4, X isoform	1.0139	0.8492	0.0526	0.8194	No	1.1176	0.057	0.0015	0.116	No	No
162499_f_at	Ube2d2	ubiquitin-conjugating enzyme E2D 2	1.1931	0.0002	0	0.018	Yes	1.0388	0.168	0.0005	0.226	No	No
162500_f_at	---	---	0.9839	0.7407	0.5429	0.4913	No	0.9553	0.15	0.704	0.317	No	No
162501_at	---	---	0.9814	0.7965	0.0023	0.247	No	0.9776	0.983	0.0003	0.155	No	No
162502_f_at	Cga	glycoprotein hormones, alpha subunit	1.1933	0.0222	0.0009	0.0094	No	1.2253	0.0026	0.0044	0.052	Yes	No
162503_f_at	Pip5k2a	phosphatidylinositol-4-phosphate 5-kinase, type II, alpha	1.0152	0.5114	0.8921	0.8946	No	0.9965	0.964	0.697	0.594	No	No
162504_at	Csk	C-src tyrosine kinase	1.1902	0.0037	0.0057	0.0048	No	1.2213	0.005	0.0335	0.0026	No	No
92180_at	H1fx	H1 histone family, member X	1.0276	0.6694	0.0016	0.3962	No	1.0775	0.4	0.0038	0.717	No	No
92181_at	Ager	advanced glycosylation end product-specific receptor	0.9706	0.3895	0.0029	0.2287	No	0.9784	0.519	0.0151	0.924	No	No
92182_at	Rufy1	RUN and FYVE domain containing 1	0.9656	0.2026	0.9452	0.1457	No	0.9405	0.114	0.0876	0.0546	No	No
92183_at	Dtna	dystrobrevin alpha	1.0505	0.4752	0.0136	0.5761	No	1.0083	0.939	0.0616	0.747	No	No
92184_at	Dtna	dystrobrevin alpha	0.9821	0.7822	0.1155	0.2022	No	0.9797	0.65	0.0978	0.645	No	No
92185_at	Arl4c	ADP-ribosylation factor-like 4C	1.0001	0.9986	0.1092	0.6707	No	1.0482	0.104	0.01	0.365	No	No
92186_at	Arx	aristaless related homeobox gene (Drosophila)	0.9358	0.3587	0.3804	0.5545	No	0.9397	0.372	0.548	0.449	No	No
92188_s_at	Fes	feline sarcoma oncogene	0.9744	0.6158	0.5039	0.5922	No	0.9204	0.144	0.0912	0.829	No	No
92189_at	Ufm1	ubiquitin-fold modifier 1	0.9753	0.7206	0.0306	0.5048	No	1.0506	0.47	0.0896	0.114	No	No
92190_at	Nr2c1	nuclear receptor subfamily 2, group C, member 1	0.8993	0.0616	0.7554	0.2733	No	0.8898	0.0253	0.0047	0.553	No	No
92191_at	Mettl5	methyltransferase like 5	1.0127	0.8793	0.0178	0.7558	No	1.0247	0.589	0.0017	0.55	No	No
92192_s_at	Pcsk5	proprotein convertase subtilisin/kexin type 5	0.9535	0.5601	0.0212	0.9383	No	1.0079	0.841	0.968	0.0775	No	No
92193_r_at	Pcsk5	proprotein convertase subtilisin/kexin type 5	1.1154	0.2011	0.0007	0.0682	No	1.0492	0.294	0.002	0.109	No	No
92194_at	Pcsk5	proprotein convertase subtilisin/kexin type 5	1.044	0.2232	0.002	0.4969	No	0.9675	0.0109	0	0.0028	No	No
92195_at	Cebpg	CCAAT/enhancer binding protein (C/EBP), gamma	0.8739	0.0882	0.8773	0.3206	No	0.8838	0.296	0.498	0.99	No	No
92196_f_at	Sf3a2	splicing factor 3a, subunit 2	1.0016	0.9737	0.6479	0.3775	No	0.9967	0.872	0.162	0.0854	No	No
92197_r_at	Sf3a2	splicing factor 3a, subunit 2	0.9653	0.2657	0.4148	0.5944	No	0.9557	0.269	0.0437	0.519	No	No
92198_s_at	Daf2	decay accelerating factor 2	0.9436	0.1807	0.1301	0.0383	No	0.8888	0.0705	0.0039	0.0057	No	No
92199_at	Stat5b	signal transducer and activator of transcription 5B	0.9289	0.3873	0.0136	0.1939	No	0.924	0.255	0.265	0.725	No	No
92200_at	Adcyap1	adenylate cyclase activating polypeptide 1	0.9845	0.8273	0.0036	0.2799	No	1.0689	0.2	0.0181	0.0512	No	No
92201_at	Zbtb16	zinc finger and BTB domain containing 16	0.9935	0.9154	0.068	0.9181	No	0.9678	0.724	0.0025	0.0452	No	No
92202_g_at	Zbtb16	zinc finger and BTB domain containing 16	0.4883	0.0002	0.0046	0.3735	Yes	0.5135	0.0003	0.0067	0.806	Yes	Yes
92203_s_at	Cd6	CD6 antigen	0.9299	0.3513	0.3861	0.3177	No	1.0628	0.376	0.0206	0.0169	No	No
92204_at	Cd6	CD6 antigen	1.011	0.6728	0.5732	0.5936	No	0.9022	0.17	0.0788	0.419	No	No
92205_at	Irs2	insulin receptor substrate 2	0.9127	0.2093	0.4244	0.527	No	0.9429	0.449	0.988	0.613	No	No
92206_at	A430107D22 Rik	RIKEN cDNA A430107D22 gene	0.972	0.7339	0.1854	0.4014	No	0.9871	0.877	0.166	0.463	No	No
92207_at	Eln	elastin	0.7458	0.0244	0.2848	0.9984	No	0.8391	0.0324	0.202	0.321	No	No
92208_at	C1qdc1	C1q domain containing 1	0.9279	0.2104	0.0227	0.1173	No	0.9321	0.233	0.0276	0.379	No	No
92209_at	Ulk1	Unc-51 like kinase 1 (C. elegans)	0.9146	0.0481	0.335	0.3195	No	0.9002	0.0934	0.661	0.0704	No	No
92210_at	Angpt2	angiopoietin 2	0.8827	0.0082	0.0095	0.0213	Yes	0.9399	0.207	0.472	0.182	No	No
92211_at	AF013969 /// LOC665775 /// LOC675766	expressed sequence AF013969 /// similar to CG5514-PB, isoform B /// region containing expressed sequence AF013969; RIKEN cDNA A230054D04 gene	0.882	0.0608	0.0053	0.8183	No	0.8769	0.0078	0.0006	0.449	Yes	No
92212_at	Rap2a	RAS related protein 2a	0.9895	0.7575	0.5488	0.0979	No	0.9873	0.998	0.275	0.0518	No	No
92213_at	Star	steroidogenic acute regulatory protein	0.9004	0.0915	0.4117	0.8058	No	0.9069	0.0737	0.737	0.0555	No	No
92214_at	Ctsw	cathepsin W	1.0815	0.249	0.0039	0.8788	No	0.9259	0.0856	0.0046	0.271	No	No
92215_at	Krtap12-1	keratin associated protein 12-1	1.0255	0.5986	0.0336	0.9262	No	0.9955	0.945	0.589	0.366	No	No
92216_at	Smad7	MAD homolog 7 (Drosophila)	1.0111	0.8502	0.0564	0.2423	No	0.9656	0.121	0.0105	0.0176	No	No
92217_s_at	Gp49a /// Lilrb4	glycoprotein 49 A /// leukocyte immunoglobulin-like receptor, subfamily B, member 4	0.7652	0.0397	0.6331	0.118	No	0.8249	0.13	0.144	0.225	No	No
92218_at	Myh2	myosin, heavy polypeptide 2, skeletal muscle, adult	1.0631	0.3106	0.6365	0.9746	No	1.3602	0.0231	0.622	0.106	No	No
92219_s_at	Mid1 /// LOC670952 /// LOC677632	midline 1 /// similar to Midline-1 (Tripartite motif protein 18) (Midin) /// similar to midline 1	0.9558	0.5779	0.0134	0.2453	No	0.9856	0.901	0.11	0.571	No	No
92220_s_at	Bin1	bridging integrator 1	0.9012	0.0004	0.0004	0.0003	No	0.9236	0.0414	0.0327	0.0045	No	No
92222_f_at	H2-Q1	histocompatibility 2, Q region locus 1	0.9857	0.6898	0.498	0.1279	No	1.1615	0.0282	0.0004	0.0108	No	No
92223_at	C1qc	complement component 1, q subcomponent, C chain	0.8156	0.0078	0.0291	0.0717	Yes	0.854	0.0218	0.0617	0.289	No	Yes
92224_at	Clec3b	C-type lectin domain family 3, member b	0.8188	0.0216	0.0188	0.0381	No	0.7956	0.039	0.694	0.122	No	No
92225_f_at	Rpo1-2 /// LOC671429	RNA polymerase 1-2 /// similar to DNA-directed RNA polymerase I 135 kDa polypeptide (RNA polymerase I subunit 2) (RPA135)	1.0294	0.571	0.0557	0.5338	No	1.032	0.545	0.111	0.396	No	No
92226_at	Ddx50	DEAD (Asp-Glu-Ala-Asp) box polypeptide 50	0.9646	0.4558	0.4675	0.9539	No	1.0103	0.999	0.0588	0.0604	No	No
92227_s_at	Ctnna2	catenin (cadherin associated protein), alpha 2	1.0429	0.0374	0.0001	0.0005	No	1.0717	0.124	0.0173	0.0603	No	No
92228_at	Ctnna2	catenin (cadherin associated protein), alpha 2	1.0288	0.5373	0.0018	0.1706	No	0.9939	0.773	0.0001	0.0267	No	No
92229_at	Zfp312	zinc finger protein 312	0.9832	0.5846	0.002	0.0332	No	0.9298	0.221	0.561	0.707	No	No
92230_at	Nln	neurolysin (metallopeptidase M3 family)	0.9412	0.398	0.0856	0.8614	No	0.9447	0.403	0.0212	0.634	No	No
92231_at	Epb4.1l4a	erythrocyte protein band 4.1-like 4a	0.9712	0.3517	0.212	0.1758	No	0.997	0.909	0.293	0.739	No	No
92232_at	Socs3	suppressor of cytokine signaling 3	0.9493	0.1055	0.0019	0.5965	No	1.0278	0.481	0.0251	0.515	No	No
92233_at	1810007M14 Rik	RIKEN cDNA 1810007M14 gene	1.0152	0.8401	0.0258	0.3428	No	1.0548	0.304	0.0118	0.199	No	No
92234_at	Rxra	retinoid X receptor alpha	1.0718	0.1126	0.2048	0.5218	No	1.0208	0.621	0.5	0.734	No	No
92235_g_at	Rxra	retinoid X receptor alpha	1.0245	0.5144	0.5062	0.1944	No	1.0613	0.109	0.069	0.268	No	No
92237_at	Rxrg	retinoid X receptor gamma	0.9695	0.4605	0.1444	0.1548	No	0.9498	0.322	0.0051	0.197	No	No

92238_at	Ppp1r12c	protein phosphatase 1, regulatory (inhibitor) subunit 12C	1.0233	0.4161	0.1754	0.4076	No	0.9823	0.749	0.773	0.372	No	No
92239_at	Elavl3	ELAV (embryonic lethal, abnormal vision, Drosophila)-like 3 (Hu antigen C)	1.1273	0.0335	0.0106	0.0148	No	1.1418	0.198	0.311	0.109	No	No
92240_at	Krtap5-5 /// LOC665305 /// LOC672423	keratin associated protein 5-5 /// hypothetical protein LOC665305 /// hypothetical protein LOC672423	1.0096	0.9047	0.318	0.1626	No	0.9091	0.13	0.004	0.429	No	No
92241_at	Nfic	nuclear factor I/C	1.0898	0.0671	0.0008	0.0426	No	1.1798	0.0053	0.0001	0.0884	Yes	No
92242_at	Saa4	serum amyloid A 4	0.9786	0.4752	0.087	0.0618	No	0.9654	0.486	0.186	0.711	No	No
92243_at	B930007L02Rik	RIKEN cDNA B930007L02 gene	1.0125	0.8476	0.0177	0.9185	No	1.0195	0.793	0.0103	0.927	No	No
92244_at	Exo1	exonuclease 1	0.8462	0.0783	0.1847	0.2355	No	1.044	0.451	0.0055	0.66	No	No
92245_at	Ddx50	DEAD (Asp-Glu-Ala-Asp) box polypeptide 50	1.0625	0.4503	0.1031	0.441	No	1.0076	0.937	0.174	0.82	No	No
92246_at	Krtap14	keratin associated protein 14	1.0802	0.2853	0.0505	0.2008	No	1.0489	0.619	0.208	0.179	No	No
92247_at	Arhgap5	Rho GTPase activating protein 5	0.9651	0.6502	0.5575	0.1757	No	0.8916	0.219	0.509	0.431	No	No
92248_at	Nr4a2	nuclear receptor subfamily 4, group A, member 2	0.9379	0.4156	0.5819	0.5903	No	0.9405	0.525	0.136	0.987	No	No
92249_g_at	Nr4a2	nuclear receptor subfamily 4, group A, member 2	0.9345	0.1318	0.3225	0.2284	No	1.083	0.122	0.0617	0.0417	No	No
92250_s_at	Prcc	papillary renal cell carcinoma (translocation-associated)	0.9905	0.6805	0	0.5329	No	0.9157	0.0963	0.0004	0.438	No	No
92251_f_at	AI607873	expressed sequence AI607873	0.9734	0.7396	0.1509	0.3672	No	0.9336	0.395	0.001	0.933	No	No
92252_at	Cckar	cholecystokinin A receptor	1.0013	0.9817	0.1016	0.4043	No	0.9924	0.983	0.0008	0.29	No	No
92253_at	Krtap6-2	keratin associated protein 6-2	0.9291	0.416	0.5526	0.9891	No	1.0392	0.706	0.144	0.622	No	No
92254_at	Myo5b	myosin Vb	1.0187	0.5573	0.0031	0.4196	No	1.0385	0.0482	0.617	0.836	No	No
92255_at	Hoxb4	homeo box B4	1.0595	0.1407	0.0146	0.5154	No	0.9766	0.614	0.0236	0.688	No	No
92256_at	Ctsb	cathepsin B	1.0081	0.6396	0.0821	0.0093	No	1.0471	0.0974	0.0007	0.31	No	No
92257_at	Clock	circadian locomoter output cycles kaput	0.9808	0.5924	0.001	0.0854	No	0.9967	0.98	0.0238	0.362	No	No
92258_at	Dppa4	developmental pluripotency associated 4	1.0441	0.4687	0.7577	0.3192	No	0.9908	0.861	0.685	0.587	No	No
92259_at	Tbl3	transducin (beta)-like 3	1.0227	0.7001	0.6893	0.0992	No	1.0824	0.18	0.236	0.0122	No	No
92260_at	---	---	1.0389	0.6787	0.7092	0.6561	No	0.9964	0.959	0.869	0.554	No	No
92261_at	Coq4	coenzyme Q4 homolog (yeast)	0.9757	0.4918	0.4584	0.3712	No	0.952	0.529	0.235	0.496	No	No
92262_at	Wig1	wild-type p53-induced gene 1	0.9875	0.6453	0.0001	0.3937	No	1.0964	0.0787	0.0007	0.434	No	No
92263_at	Leprel2	leprecan-like 2	1.1019	0.0258	0.0001	0.3022	No	1.0766	0.172	0.001	0.28	No	No
92264_at	Sox3	SRY-box containing gene 3	0.9061	0.1063	0.0547	0.8072	No	0.9258	0.331	0.0854	0.636	No	No
92265_f_at	Trove2	TROVE domain family, member 2	0.912	0.3558	0.0331	0.6077	No	0.9835	0.86	0.0078	0.566	No	No
92266_at	Il11	interleukin 11	0.9436	0.4066	0.0076	0.0531	No	0.8855	0.0188	0.337	0.0052	No	No
92267_at	F2r	coagulation factor II (thrombin) receptor	1.0177	0.7109	0.5429	0.0825	No	0.9603	0.556	0.166	0.447	No	No
92268_at	2700007P21Rik	RIKEN cDNA 2700007P21 gene	0.843	0.0576	0.4911	0.5589	No	0.8798	0.0748	0.274	0.925	No	No
92269_r_at	Peg10	paternally expressed 10	1.1259	0.1067	0.1124	0.0354	No	1.1321	0.0991	0.313	0.0375	No	No
92270_at	Tro	trophinin	0.9807	0.2636	0.0008	0.739	No	0.9841	0.262	0.002	0.0046	No	No
92271_at	Pax6	paired box gene 6	1.0728	0.129	0.0007	0.5405	No	0.9871	0.902	0.0038	0.0832	No	No
92272_at	Rbm16	RNA binding motif protein 16	0.9295	0.0874	0.0709	0.6896	No	0.8661	0.133	0.456	0.847	No	No
92273_at	Ptpn18	protein tyrosine phosphatase, non-receptor type 18	1.0171	0.5275	0.0179	0.9185	No	1.0143	0.636	0.0562	0.242	No	No
92274_at	Prlpj	prolactin-like protein J	0.936	0.0836	0.1566	0.074	No	0.9604	0.67	0.739	0.734	No	No
92275_at	Tcfap2c	transcription factor AP-2, gamma	1.0305	0.4826	0.1336	0.4193	No	1.123	0.052	0.626	0.258	No	No
92276_at	Map3k6	mitogen-activated protein kinase kinase kinase 6	0.7865	0.0584	0.2639	0.3775	No	0.827	0.0101	0.037	0.358	No	No
92277_at	Myo1e	myosin IE	0.9767	0.6409	0.0334	0.7596	No	1.0202	0.828	0.0207	0.502	No	No
92278_at	Ercc2	excision repair cross-complementing rodent repair deficiency, complementation group 2	1.075	0.2005	0.3424	0.5318	No	1.0374	0.361	0.0697	0.471	No	No
92279_at	Golt1b	golgi transport 1 homolog B (S. cerevisiae)	1.0329	0.6014	0.0165	0.8438	No	0.9895	0.906	0.0107	0.182	No	No
92280_at	Actn1	actinin, alpha 1	0.9738	0.3622	0.783	0.2506	No	0.8989	0.0099	0.0306	0.225	Yes	No
92281_at	2510002D24Rik	RIKEN cDNA 2510002D24 gene	0.9329	0.0951	0.1705	0.0679	No	0.9484	0.118	0.0533	0.189	No	No
92282_at	2610304G08Rik	RIKEN cDNA 2610304G08 gene	0.8578	0.073	0.2166	0.9357	No	0.8963	0.0993	0.339	0.272	No	No
92283_s_at	Il4	interleukin 4	0.9629	0.6246	0.0502	0.9533	No	0.9774	0.911	0.0613	0.514	No	No
92284_r_at	Il4	interleukin 4	1.0655	0.1001	0.044	0.0332	No	1.14	0.0776	0.73	0.0153	No	No
92285_at	Il4	Interleukin 4	0.9316	0.3246	0.129	0.9862	No	0.9624	0.711	0.806	0.842	No	No
92286_g_at	Il4	interleukin 4	0.9456	0.6512	0.8788	0.8464	No	1.0645	0.287	0.0092	0.396	No	No
92287_at	Socs5	suppressor of cytokine signaling 5	0.9786	0.4035	0.1603	0.1526	No	1.003	0.995	0.129	0.601	No	No
92288_at	Ap1g1	adaptor protein complex AP-1, gamma 1 subunit	0.8769	0.038	0.4355	0.2069	No	0.9066	0.0998	0.943	0.283	No	No
92289_at	Ptprb	protein tyrosine phosphatase, receptor type, B	0.8745	0.203	0.5911	0.5721	No	0.8831	0.108	0.326	0.177	No	No
92290_at	Siah1b	seven in absentia 1B	1.1799	0.0022	0.0001	0.001	No	1.1464	0.0022	0.0001	0.0028	No	No
92291_f_at	Cfhr1	complement factor H-related 1	0.9541	0.3852	0.0006	0.2266	No	0.8413	0.124	0.0005	0.959	No	No
92292_at	Slc2a3	solute carrier family 2 (facilitated glucose transporter), member 3	1.0223	0.6985	0.0193	0.2152	No	0.9477	0.209	0	0.133	No	No
92293_at	C130076O07Rik	RIKEN cDNA C130076O07 gene	1.0329	0.556	0.0232	0.0827	No	0.9949	0.995	0.0384	0.0811	No	No
92294_at	Arl5a	ADP-ribosylation factor-like 5A	1.1072	0.1222	0.0022	0.437	No	0.9298	0.295	0.0004	0.172	No	No
92295_at	Sema3e	sema domain, immunoglobulin domain (Ig), short basic domain, secreted, (semaphorin) 3E	0.8662	0.0101	0.0035	0.0484	No	1.0011	0.952	0.988	0.325	No	No
92296_at	Pign	phosphatidylinositol glycan, class N	1.121	0.1079	0.2038	0.6432	No	1.0877	0.395	0.817	0.789	No	No
92299_at	Jarid1a	jumonji, AT rich interactive domain 1A (Rbp2 like)	0.9695	0.6765	0.0324	0.3152	No	0.9432	0.507	0.0343	0.618	No	No
92300_at	Mnt	max binding protein	1.0211	0.5145	0.6623	0.439	No	1.0634	0.0574	0.094	0.458	No	No
92301_at	Ikbke	inhibitor of kappaB kinase epsilon	0.9925	0.8931	0.3892	0.1601	No	1.0484	0.511	0.389	0.0633	No	No
92302_at	Sos2	Son of sevenless homolog 2 (Drosophila)	1.0885	0.145	0.0004	0.6942	No	1.0331	0.501	0.0002	0.543	No	No
92303_at	Ptprr	protein tyrosine phosphatase, receptor type, R	1.0486	0.3818	0.0327	0.0298	No	1.0487	0.569	0.3	0.185	No	No
92304_at	Piga	phosphatidylinositol glycan, class A	0.908	0.0214	0.0001	0.6013	No	0.9542	0.352	0.0001	0.112	No	No
92305_s_at	Pou2f2	POU domain, class 2, transcription factor 2	0.9521	0.3078	0.004	0.1032	No	0.9008	0.0171	0.0009	0.0156	No	No
92306_at	Ott /// LOC382243 /// LOC434863 /// LOC434864 /// LOC434865 /// LOC666184 /// LOC666260	ovary testis transcribed /// similar to ovary testis transcribed /// similar to ovary testis transcribed /// similar to ovary testis transcribed /// similar to ovary testis transcribed /// similar to ovary testis transcribed /// similar to ovary testis transcribed	1.0037	0.9486	0.0773	0.0733	No	1.059	0.604	0.389	0.0892	No	No
92307_at	Ng23	Ng23 protein	0.8924	0.0791	0.0242	0.7146	No	0.8327	0.0464	0.0135	0.25	No	No
92308_at	Ptprm	protein tyrosine phosphatase, receptor type, M	1.1303	0.2155	0.025	0.3458	No	1.0505	0.162	0.0034	0.0368	No	No
92309_i_at	Ptprm	protein tyrosine phosphatase, receptor type, M	0.9134	0.0706	0.0548	0.8064	No	1.0054	0.741	0.0288	0.0671	No	No
92310_at	Plk2	polo-like kinase 2 (Drosophila)	1.0574	0.0677	0.0037	0.2136	No	1.1108	0.0122	0.0023	0.0841	No	No
92311_s_at	Pik3c2a	phosphatidylinositol 3-kinase, C2 domain containing, alpha polypeptide	0.9086	0.3842	0.1292	0.8599	No	0.919	0.296	0.24	0.721	No	No
92312_at	Pik3c2a	phosphatidylinositol 3-kinase, C2 domain containing, alpha polypeptide	0.9849	0.7666	0.3771	0.3612	No	0.9084	0.253	0.172	0.949	No	No
92313_at	Col12a1	Procollagen, type XII, alpha 1	1.0928	0.4273	0.0903	0.5276	No	1.063	0.321	0.0098	0.733	No	No
92314_at	Col12a1	procollagen, type XII, alpha 1	1.015	0.5253	0.3333	0.8468	No	0.9686	0.651	0.283	0.972	No	No
92315_at	Slfn4	schlafen 4	1.0348	0.5891	0.1748	0.5133	No	1.089	0.392	0.353	0.477	No	No
92316_f_at	Igl-V1 /// 2010309G21Rik /// LOC207685 /// LOC386520	immunoglobulin lambda chain, variable 1 /// RIKEN cDNA 2010309G21 gene /// hypothetical protein LOC207685 /// similar to Ig lambda-3 chain C region - mouse	0.9496	0.4682	0.1033	0.9171	No	0.9984	0.889	0.0014	0.596	No	No
92317_at	Elavl2	ELAV (embryonic lethal, abnormal vision, Drosophila)-like 2 (Hu antigen B)	0.9956	0.9616	0.0019	0.1993	No	1.0818	0.248	0.0118	0.112	No	No
92318_at	2010301N04Rik	RIKEN cDNA 2010301N04 gene	0.9879	0.7923	0.0626	0.1776	No	1.0245	0.913	0.009	0.151	No	No
92319_i_at	LOC544782	Similar to Poly(A) binding protein, cytoplasmic 4, isoform 1	1.0727	0.3892	0.0678	0.6104	No	1.3181	0.0143	0.0669	0.101	No	No
92320_f_at	---	---	1.0073	0.8395	0.0857	0.8134	No	1.2308	0.19	0.0902	0.144	No	No
92321_r_at	---	---	0.9758	0.7273	0.99	0.6441	No	1.0558	0.336	0.0113	0.292	No	No
92322_at	Camp	cathelicidin antimicrobial peptide	1.0167	0.5556	0.489	0.1507	No	0.9937	0.791	0.779	0.0161	No	No
92323_at	Mapk12	mitogen-activated protein kinase 12	0.9633	0.4738	0.1375	0.6224	No	0.9825	0.565	0.0631	0.176	No	No
92324_at	Klf17	Kruppel-like factor 17	0.9428	0.6566	0.1113	0.3466	No	0.7963	0.0829	0.0239	0.0101	No	No
92325_at	Ptk7	PTK7 protein tyrosine kinase 7	1.046	0.3444	0.0069	0.9387	No	1.1125	0.103	0.0049	0.641	No	No

92328_at	A630038E17 Rik	RIKEN cDNA A630038E17 gene	0.9472	0.4397	0.5708	0.4917	No	1.0292	0.622	0.958	0.902	No	No
92329_at	Rab17	RAB17, member RAS oncogene family	0.9919	0.897	0.0584	0.5002	No	0.9323	0.131	0.0082	0.436	No	No
92330_r_at	---	---	1.0267	0.8036	0.0005	0.8968	No	1.0397	0.721	0.0003	0.0347	No	No
92331_at	Mme	membrane metallo endopeptidase	0.9881	0.8662	0.0127	0.6958	No	1.0244	0.604	0.0062	0.974	No	No
92332_at	Dlx2	distal-less homeobox 2	0.7645	0.0455	0.4391	0.464	No	0.6373	0.0219	0.455	0.506	No	No
92333_at	Sirt1	Sirtuin 1 ((silent mating type information regulation 2, homolog) 1 (S. cerevisiae)	0.9324	0.2538	0.9238	0.6425	No	0.9908	0.974	0.0029	0.0256	No	No
92334_at	Sap30bp	SAP30 binding protein	1.0246	0.6356	0.0092	0.1898	No	1.0636	0.295	0.0153	0.0604	No	No
92335_at	Ltbp2	latent transforming growth factor beta binding protein 2	0.9525	0.1192	0.9989	0.3129	No	0.9025	0.0136	0.0065	0.714	No	No
92336_at	Polrmt	polymerase (RNA) mitochondrial (DNA directed)	0.9863	0.7892	0.0052	0.1163	No	0.9777	0.532	0.0149	0.56	No	No
92337_g_at	Polrmt	polymerase (RNA) mitochondrial (DNA directed)	0.9658	0.3168	0.6483	0.6057	No	0.9821	0.477	0.0198	0.599	No	No
92338_f_at	2310001H12 Rik /// LOC636362 /// LOC666849	RIKEN cDNA 2310001H12 gene /// similar to zinc finger protein 709 /// similar to zinc finger protein 665	1.1337	0.3963	0.1423	0.9763	No	1.0687	0.53	0.106	0.38	No	No
92339_at	Taf1a	TATA box binding protein (Tbp)-associated factor, RNA polymerase I, A	1.0183	0.5189	0.5699	0.6008	No	1.0314	0.0543	0.0006	0.671	No	No
92340_at	Adra1b	adrenergic receptor, alpha 1b	0.9015	0.1823	0.0132	0.8307	No	0.9653	0.293	0.0003	0.306	No	No
92341_at	B3galt2	UDP-Gal:betaGlcNAc beta 1,3-galactosyltransferase, polypeptide 2	0.9323	0.0846	0.1514	0.9442	No	0.9289	0.142	0.504	0.0156	No	No
92342_at	Sox1	SRY-box containing gene 1	1.0079	0.7887	0.0011	0.0614	No	1.026	0.472	0.638	0.315	No	No
92343_at	Smarca3	SWI/SNF related, matrix associated, actin dependent regulator of chromatin, subfamily a, member 3	0.9786	0.6015	0.4443	0.0276	No	0.9105	0.197	0.547	0.574	No	No
92344_at	Smarca3	SWI/SNF related, matrix associated, actin dependent regulator of chromatin, subfamily a, member 3	1.056	0.0784	0.0053	0.0022	No	1.1041	0.136	0.0383	0.023	No	No
92345_g_at	Smarca3	SWI/SNF related, matrix associated, actin dependent regulator of chromatin, subfamily a, member 3	0.9025	0.1071	0.0108	0.7405	No	0.9264	0.555	0.0008	0.0548	No	No
92346_at	Nef3	neurofilament 3, medium	0.9235	0.1598	0.0011	0.2575	No	1.0342	0.793	0.0004	0.419	No	No
92347_at	Kcnc3	potassium voltage gated channel, Shaw-related subfamily, member 3	0.9919	0.8704	0.5276	0.9308	No	0.9998	0.923	0.269	0.257	No	No
92348_at	Thra	thyroid hormone receptor alpha	0.9378	0.1002	0.0006	0.0091	No	0.9876	0.814	0.0005	0.0485	No	No
92349_at	Igfbpl1	insulin-like growth factor binding protein-like 1	1.0343	0.5473	0.2746	0.4647	No	1.019	0.987	0.0003	0.0203	No	No
92350_at	Mapre1	microtubule-associated protein, RP/EB family, member 1	1.0027	0.9352	0.0162	0.0079	No	1.0734	0.0901	0.183	0.004	No	No
92351_at	Dnm2	dynamin 2	1.0238	0.7207	0.8931	0.8473	No	1.0341	0.539	0.297	0.598	No	No
92352_at	Edg3	endothelial differentiation, sphingolipid G-protein-coupled receptor, 3	0.9774	0.14	0.5817	0.0244	No	1.0394	0.145	0.0123	0.295	No	No
92353_at	Klk6	kallikrein 6	0.9366	0.51	0.1882	0.5267	No	1.0237	0.73	0.209	0.352	No	No
92354_at	Maf	avian musculoaponeurotic fibrosarcoma (v-maf) AS42 oncogene homolog	1.025	0.653	0.2076	0.1105	No	1.033	0.658	0.532	0.364	No	No
92355_at	Zfpm2	zinc finger protein, multitype 2	1.0171	0.5919	0.0028	0.8057	No	0.9241	0.0453	0.538	0.11	No	No
92356_at	Ptpn22	protein tyrosine phosphatase, non-receptor type 22 (lymphoid)	0.9247	0.1068	0.9269	0.6943	No	0.9069	0.0825	0.421	0.0438	No	No
92357_at	Adam23	a disintegrin and metallopeptidase domain 23	0.9828	0.7243	0.0024	0.6657	No	0.9933	0.949	0.0013	0.429	No	No
92358_at	Nell2	NEL-like 2 (chicken)	1.0016	0.9664	0.0049	0.0408	No	1.0917	0.103	0.0036	0.163	No	No
92359_at	Blk	B lymphoid kinase	0.9703	0.8186	0.0614	0.969	No	0.8905	0.217	0.146	0.93	No	No
92360_at	Stam	signal transducing adaptor molecule (SH3 domain and ITAM motif) 1	0.9517	0.2936	0.019	0.8408	No	0.9713	0.607	0.0543	0.485	No	No
92361_at	Elavl4	ELAV (embryonic lethal, abnormal vision, Drosophila)-like 4 (Hu antigen D)	1.0647	0.3972	0.3	0.7998	No	1.1323	0.0486	0.0271	0.16	No	No
92362_at	Dusp8	dual specificity phosphatase 8	1.0028	0.9177	0.0677	0.6156	No	1.0503	0.635	0.416	0.296	No	No
92363_at	Celsr2	cadherin EGF LAG seven-pass G-type receptor 2	1.0334	0.0438	0.0031	0.0896	No	1.0573	0.0634	0.434	0.155	No	No
92364_at	Celsr2	cadherin EGF LAG seven-pass G-type receptor 2	0.9331	0.0816	0.032	0.0772	No	0.9344	0.192	0.0072	0.169	No	No
92365_at	Figf	c-fos induced growth factor	0.9898	0.8797	0.1082	0.3316	No	0.977	0.398	0.0068	0.588	No	No
92366_at	Lama2	laminin, alpha 2	0.9164	0.0116	0	0.5316	No	0.9172	0.156	0.0018	0.688	No	No
92367_at	Stil	Scl/Tal1 interrupting locus	1.0639	0.0923	0.5983	0.3016	No	0.9585	0.279	0.0043	0.511	No	No
92368_at	Ramp3	receptor (calcitonin) activity modifying protein 3	0.9397	0.2115	0.7723	0.3458	No	0.9807	0.292	0.0019	0.0019	No	No
92369_at	Tgfa	transforming growth factor alpha	1.006	0.9477	0.5989	0.9478	No	0.9462	0.0468	0.0046	0.961	No	No
92370_at	Arl10	ADP-ribosylation factor-like 10	1.0362	0.3566	0.2254	0.9559	No	1.021	0.601	0.0464	0.61	No	No
92371_at	Hrc	histidine rich calcium binding protein	0.9696	0.152	0.0108	0.01	No	0.9022	0.0091	0.0776	0.0046	No	No
92372_at	Bmp6	bone morphogenetic protein 6	0.9171	0.3302	0.7849	0.2209	No	1.0135	0.884	0.221	0.536	No	No
92374_at	Adora1	adenosine A1 receptor	0.9456	0.1078	0.0172	0.7987	No	0.8503	0.0906	0.758	0.437	No	No
92375_at	Ascc1	activating signal cointegrator 1 complex subunit 1	0.8706	0.0968	0.1469	0.8256	No	0.8663	0.0067	0.0124	0.148	Yes	No
92376_at	Rit1	Ras-like without CAAX 1	0.9627	0.0526	0.0683	0.0027	No	0.9403	0.138	0.319	0.0371	No	No
92377_at	Gm1060	gene model 1060, (NCBI)	1.0063	0.8987	0.0443	0.8237	No	1.0089	0.598	0.0015	0.0992	No	No
92378_at	Ptprz1	protein tyrosine phosphatase, receptor type Z, polypeptide 1	0.9267	0.007	0.0041	0.7062	Yes	1.0586	0.379	0.111	0.134	No	No
92379_f_at	Ptprz1	protein tyrosine phosphatase, receptor type Z, polypeptide 1	1.1167	0.2308	0.0069	0.1808	No	1.1064	0.022	0.0005	0.0153	No	No
92380_r_at	Ptprz1	protein tyrosine phosphatase, receptor type Z, polypeptide 1	1.004	0.8727	0.5641	0.633	No	0.7461	0.748	0.225	0.656	No	No
92381_at	Baz1b	bromodomain adjacent to zinc finger domain, 1B	0.9383	0.1271	0.0226	0.3406	No	0.9691	0.381	0.0023	0.278	No	No
92382_at	Myo6	myosin VI	0.8988	0.1264	0.0252	0.5332	No	0.9818	0.786	0.0168	0.962	No	No
92383_at	Dyrk1a	dual-specificity tyrosine-(Y)-phosphorylation regulated kinase 1a	0.8962	0.0725	0.9912	0.1079	No	1.3071	0.0064	0.514	0.0035	No	No
92384_at	Xpa	xeroderma pigmentosum, complementation group A	1.0554	0.2333	0.0249	0.0702	No	1.0241	0.753	0.0899	0.145	No	No
92385_at	Clcn2	Chloride channel 2	0.966	0.3667	0.0033	0.321	No	0.899	0.0448	0.0129	0.887	No	No
92386_at	Hps4	Hermansky-Pudlak syndrome 4 homolog (human)	1.0451	0.4062	0.9768	0.7448	No	0.9314	0.258	0.167	0.258	No	No
92387_at	Tbxas1	thromboxane A synthase 1, platelet	0.9805	0.5981	0.035	0.7551	No	1.0016	0.939	0.0466	0.434	No	No
92388_at	BC038311	cDNA sequence BC038311	1.0546	0.1427	0.0395	0.5291	No	1.1043	0.0211	0.886	0.0319	No	No
92389_at	Odz2	odd Oz/ten-m homolog 2 (Drosophila)	0.9367	0.3334	0.43	0.4591	No	0.9523	0.457	0.55	0.944	No	No
92390_at	Pik3cd	phosphatidylinositol 3-kinase catalytic delta polypeptide	0.901	0.0253	0.0025	0.3358	No	1.0298	0.237	0.0081	0.0923	No	No
92391_at	Cyb561	cytochrome b-561	0.9954	0.9015	0.0738	0.132	No	1.0449	0.222	0.0111	0.136	No	No
92392_at	---	Transcribed locus	1.0179	0.6068	0.0198	0.3476	No	0.7314	0.0345	0.199	0.146	No	No
92393_at	Kcna3	potassium voltage-gated channel, shaker-related subfamily, member 3	0.9487	0.0524	0.03	0.414	No	1.1666	0.084	0.141	0.551	No	No
92394_f_at	Crebl1	cAMP responsive element binding protein-like 1	1.1001	0.0908	0.5047	0.1064	No	1.0551	0.406	0.055	0.0693	No	No
92395_r_at	Crebl1	cAMP responsive element binding protein-like 1	0.9285	0.0103	0	0.0424	No	0.9644	0.342	0.0011	0.971	No	No
92396_at	Insig1	Insulin induced gene 1	1.0395	0.6146	0.0367	0.8177	No	1.1867	0.0207	0.242	0.0093	No	No
92397_at	Centg2	centaurin, gamma 2	1.05	0.0998	0.001	0.2287	No	1.0376	0.397	0.0005	0.681	No	No
92398_at	Vps37b	vacuolar protein sorting 37B (yeast)	1.0043	0.9188	0.0025	0.7511	No	0.9953	0.811	0.0023	0.44	No	No
92399_at	Runx1	runt related transcription factor 1	0.9525	0.1012	0.0013	0.0348	No	1.0449	0.194	0.434	0.0598	No	No
92400_at	Ndst2	N-deacetylase/N-sulfotransferase (heparan glucosaminyl) 2	0.8738	0.0371	0.0032	0.1329	No	0.8439	0.0036	0.0007	0.0192	Yes	Yes
92401_at	Ltc4s	leukotriene C4 synthase	0.9528	0.0263	0.0639	0.0201	No	0.8548	0.0076	0.003	0.0432	Yes	Yes
92402_at	LOC666623	similar to putative retrovirus-related gag protein	0.9437	0.465	0.3844	0.5001	No	0.9269	0.152	0.785	0.0488	No	No
92403_at	St6galnac5	ST6 (alpha-N-acetyl-neuraminyl-2,3-beta-galactosyl-1,3)-N-acetylgalactosaminide alpha-2,6-sialyltransferase 5	1.111	0.0945	0.0246	0.079	No	1.0611	0.0867	0.0907	0.416	No	No
92404_at	Wnt7b	wingless-related MMTV integration site 7B	0.924	0.0678	0.164	0.0519	No	1.0759	0.298	0.95	0.984	No	No
92405_at	Kpna6	karyopherin (importin) alpha 6	1.0242	0.1843	0.0484	0.0051	No	1.0292	0.533	0.0193	0.466	No	No
92406_at	Cd7	CD7 antigen	1.0625	0.0607	0.248	0.9449	No	1.08	0.0807	0.108	0.088	No	No
92407_at	Myom1	myomesin 1	0.7527	0.0004	0.0002	0.0003	No	0.7821	0.0002	0.0001	0.0002	No	No
92408_at	C130065N1 0Rik	RIKEN cDNA C130065N10 gene	1.0499	0.5347	0.0043	0.2505	No	0.991	0.9	0.0025	0.397	No	No
92409_at	LOC635007	similar to Zinc finger protein OZF (Zinc finger protein 260) (Zfp-260)	0.947	0.4655	0.056	0.3124	No	0.9853	0.755	0.182	0.264	No	No
92410_at	Rad23a	RAD23a homolog (S. cerevisiae)	0.9321	0.1322	0.2946	0.0474	No	0.9146	0.0689	0.003	0.116	No	No
92411_at	Hs1bp3	HS1 binding protein 3	0.9575	0.3898	0.0333	0.0751	No	0.9819	0.495	0.451	0.0355	No	No
92412_s_at	Spag5	sperm associated antigen 5	0.9442	0.4281	0.5688	0.5266	No	1.0183	0.797	0.104	0.803	No	No
92413_at	Foxk2	forkhead box K2	1.1031	0.0463	0.1566	0.036	No	1.2393	0.0046	0.0904	0.275	Yes	Yes
92414_at	Adam12	a disintegrin and metallopeptidase domain 12 (meltrin alpha)	0.97	0.2808	0.0004	0.2622	No	1.016	0.17	0	0.0113	No	No
92415_at	Tnfsf9	tumor necrosis factor (ligand) superfamily, member 9	1.0271	0.7101	0.1894	0.2728	No	1.0961	0.321	0.185	0.0988	No	No
92416_at	Freq	frequenin homolog (Drosophila)	0.9629	0.4348	0.1117	0.7607	No	0.9497	0.115	0.0047	0.0443	No	No
92417_at	Rasal1	RAS protein activator like 1 (GAP1 like)	0.9905	0.7537	0.0787	0.1546	No	0.9937	0.834	0.186	0.477	No	No

92418_at	Abcb9	ATP-binding cassette, sub-family B (MDR/TAP), member 9	0.9312	0.248	0.5646	0.6549	No	0.9737	0.377	0.0337	0.195	No	No
92419_at	Cct6b	chaperonin subunit 6b (zeta)	1.0662	0.5912	0.6783	0.3862	No	1.0158	0.847	0.161	0.181	No	No
92420_at	Ntf3	neurotrophin 3	1.0808	0.0781	0.3584	0.4078	No	1.0567	0.437	0.156	0.173	No	No
92421_at	Wdsub1	WD repeat, SAM and U-box domain containing 1	0.9585	0.3429	0.0468	0.343	No	0.9391	0.0451	0.0179	0.58	No	No
92422_at	Chga	chromogranin A	0.9267	0.1159	0.1034	0.2305	No	1.0096	0.481	0.0001	0.0889	No	No
92423_at	Pard6a	par-6 (partitioning defective 6,) homolog alpha (C. elegans)	0.9651	0.258	0.0752	0.0393	No	0.9533	0.102	0.0024	0.0541	No	No
92424_at	Zfp692	zinc finger protein 692	0.9635	0.4281	0.2459	0.8792	No	0.9287	0.105	0.489	0.38	No	No
92425_at	Chd1l	chromodomain helicase DNA binding protein 1-like	0.9865	0.8276	0.0596	0.26	No	1.0405	0.624	0.249	0.172	No	No
92426_at	Tspan5	tetraspanin 5	1.0452	0.6223	0.2089	0.7429	No	1.0371	0.483	0.129	0.935	No	No
92427_at	Tgfbr1	transforming growth factor, beta receptor I	1.0131	0.7436	0.0233	0.8575	No	0.9665	0.53	0.028	0.569	No	No
92428_at	Asna1	arsA (bacterial) arsenite transporter, ATP-binding, homolog 1	0.9269	0.2718	0.0388	0.2905	No	0.9601	0.504	0.0107	0.323	No	No
92429_at	Mc2r	melanocortin 2 receptor	0.9562	0.53	0.9937	0.2928	No	0.998	0.917	0.691	0.282	No	No
92430_at	Chchd5	coiled-coil-helix-coiled-coil-helix domain containing 5	0.9172	0.1246	0.0853	0.3394	No	1.2217	0.0642	0.0489	0.0098	No	No
92432_at	Zfp574	zinc finger protein 574	0.9372	0.3057	0.0162	0.7468	No	0.9908	0.888	0.0552	0.916	No	No
92433_at	Kif5c	kinesin family member 5C	1.0364	0.4823	0.0594	0.2181	No	1.0882	0.17	0.0867	0.73	No	No
92434_at	Traip	TRAF-interacting protein	0.9565	0.0721	0.0539	0.0401	No	0.9745	0.674	0.315	0.0894	No	No
92435_at	Rlbp1	retinaldehyde binding protein 1	1.064	0.1706	0.0032	0.1337	No	0.9574	0.288	0.0497	0.351	No	No
92436_at	Stk23	serine/threonine kinase 23	0.8876	0.0424	0.0203	0.0218	No	0.8421	0.0315	0.143	0.0344	No	No
92437_at	Tm7sf2	transmembrane 7 superfamily member 2	0.9366	0.2938	0.4696	0.9185	No	0.956	0.0714	0.0003	0.295	No	No
92439_at	Oc90	otoconin 90	1.102	0.1058	0.0126	0.6017	No	1.0364	0.138	0.0086	0.313	No	No
92440_at	Irf6	interferon regulatory factor 6	1.0437	0.3226	0.0277	0.3439	No	1.0672	0.169	0.0634	0.318	No	No
92441_at	Fap	fibroblast activation protein	0.8569	0.014	0.0848	0.4334	No	0.8414	0.0864	0.668	0.799	No	No
92442_at	1500005P14 Rik	RIKEN cDNA 1500005P14 gene	0.9815	0.5348	0.0111	0.0276	No	1.0655	0.14	0.0015	0.131	No	No
92443_i_at	Zfp1	zinc finger protein 1	1.0144	0.7233	0.039	0.8285	No	1.0555	0.158	0.0007	0.153	No	No
92444_f_at	Zfp1	zinc finger protein 1	0.9252	0.0285	0.0011	0.1656	No	1.0268	0.367	0.0959	0.0782	No	No
92445_at	Cacna1a	calcium channel, voltage-dependent, P/Q type, alpha 1A subunit	0.9774	0.615	0.0039	0.0224	No	1.0199	0.664	0.618	0.772	No	No
92448_s_at	Gfra2	glial cell line derived neurotrophic factor family receptor alpha 2	1.0588	0.2423	0.0253	0.435	No	0.9677	0.475	0.0968	0.953	No	No
92449_at	Gfra2	glial cell line derived neurotrophic factor family receptor alpha 2	0.9849	0.8712	0.0425	0.4861	No	0.9631	0.772	0.0028	0.166	No	No
92450_at	Slc12a4	solute carrier family 12, member 4	1.0199	0.6905	0.0105	0.6175	No	1.0603	0.0169	0.0009	0.0061	No	No
92451_at	Ankrd11	Ankyrin repeat domain 11	1.0135	0.6858	0.4166	0.9076	No	1.057	0.241	0.48	0.393	No	No
92452_at	Pik3ca	phosphatidylinositol 3-kinase, catalytic, alpha polypeptide	0.9057	0.2006	0.1894	0.061	No	0.8385	0.0016	0.0014	0.0132	Yes	No
92453_at	Eya4	eyes absent 4 homolog (Drosophila)	0.9555	0.3805	0.6503	0.4627	No	0.8443	0.318	0.656	0.933	No	No
92454_at	Slc6a20	solute carrier family 6 (neurotransmitter transporter), member 20	1.0139	0.7954	0.7078	0.821	No	1.0185	0.77	0.633	0.926	No	No
92455_at	Ddc8	testis specific protein, Ddc8	0.9973	0.9541	0.9856	0.8285	No	1.0653	0.226	0.91	0.075	No	No
92456_at	Taf1c	TATA box binding protein (Tbp)-associated factor, RNA polymerase I, C	0.9106	0.0781	0.0248	0.0758	No	0.9346	0.0546	0.0032	0.0086	No	No
92457_at	E130201N16 Rik	RIKEN cDNA E130201N16 gene	0.9908	0.767	0.1605	0.5833	No	0.952	0.672	0.503	0.173	No	No
92458_at	Orc1l	origin recognition complex, subunit 1-like (S.cereviaiae)	0.985	0.7329	0.31	0.2033	No	1.0089	0.63	0.0001	0.0125	No	No
92459_at	Ccl8	chemokine (C-C motif) ligand 8	1.0501	0.3341	0.0292	0.8497	No	1.1	0.078	0.341	0.206	No	No
92460_at	Sema4f	sema domain, immunoglobulin domain (Ig), TM domain, and short cytoplasmic domain	0.9201	0.2475	0.1386	0.3567	No	1.0889	0.27	0.336	0.178	No	No
92461_at	Mmp17	matrix metallopeptidase 17	0.9509	0.205	0.6041	0.7901	No	1.0008	0.966	0.143	0.281	No	No
92462_at	38966	septin 6	0.8987	0.1969	0.9078	0.7447	No	0.9454	0.0862	0.01	0.333	No	No
92464_at	Mknk1	MAP kinase-interacting serine/threonine kinase 1	1.014	0.563	0.0023	0.8432	No	0.8903	0.0184	0.466	0.013	No	No
92465_at	Plcb1	phospholipase C, beta 1	0.9696	0.228	0.9865	0.8578	No	1.0121	0.858	0.639	0.403	No	No
92466_at	Plcb1	phospholipase C, beta 1	0.9968	0.9324	0.0199	0.9722	No	1.0139	0.625	0.0548	0.0481	No	No
92467_g_at	Plcb1	phospholipase C, beta 1	1.0459	0.4944	0.3014	0.5869	No	0.962	0.715	0.322	0.391	No	No
92468_at	Ankrd49	ankyrin repeat domain 49	0.9499	0.4657	0.4152	0.6409	No	0.9866	0.597	0.0076	0.644	No	No
92469_at	Sfrp4	secreted frizzled-related sequence protein 4	0.9246	0.1074	0.2909	0.1821	No	1.0536	0.274	0.0384	0.548	No	No
92470_f_at	Csf2ra /// Igh VJ558 /// AB069917 /// LOC619833 /// LOC619916 /// LOC636044 /// LOC636244 /// LOC668549 /// LOC673286	colony stimulating factor 2 receptor, alpha, low-affinity (granulocyte-macrophage) /// immunoglobulin heavy chain (J558 family) /// cDNA sequence AB069917 /// similar to Ig heavy chain V region 102 precursor /// similar to Ig heavy chain V region B1-8/186-2 precursor /// similar to Ig heavy chain V region 23 precursor /// similar to Ig heavy chain V region 102 precursor /// similar to Ig heavy chain V region 145 precursor /// similar to Ig heavy chain V region 102 precursor	1.0545	0.4153	0.7265	0.2356	No	1.0536	0.295	0.0591	0.382	No	No
92471_i_at	Slfn2	schlafen 2	0.9197	0.1332	0.0054	0.6839	No	0.9334	0.0433	0.0058	0.71	No	No
92472_f_at	Slfn2	schlafen 2	0.8077	0.0578	0.3194	0.2791	No	1.0674	0.648	0.729	0.993	No	No
92473_at	Mknk2	MAP kinase-interacting serine/threonine kinase 2	0.9206	0.0182	0.0006	0.4075	No	0.9553	0.317	0.0641	0.481	No	No
92474_at	Pld1	phospholipase D1	1.0089	0.8484	0.0572	0.8374	No	0.9446	0.371	0.252	0.213	No	No
92475_g_at	Pld1	phospholipase D1	0.8988	0.0968	0.7059	0.3924	No	0.8659	0.0375	0.0112	0.0411	No	No
92476_at	Gdf3	growth differentiation factor 3	0.9849	0.7316	0.0797	0.9282	No	0.9759	0.345	0.0003	0.0107	No	No
92477_at	Spin	spindlin	0.9627	0.6348	0.0561	0.5947	No	0.9607	0.646	0.486	0.41	No	No
92478_at	Stag1	stromal antigen 1	1.0747	0.3135	0.2725	0.3882	No	1.0758	0.196	0.0486	0.031	No	No
92480_f_at	Zfp53	zinc finger protein 53	1.0866	0.4252	0.0395	0.9084	No	1.0369	0.661	0.106	0.975	No	No
92481_at	Chek2	CHK2 checkpoint homolog (S. pombe)	0.9751	0.4504	0.0286	0.3919	No	1.0186	0.404	0.0393	0.333	No	No
92482_at	Fmnl1	formin-like 1	0.9876	0.7142	0.0018	0.0161	No	0.9886	0.88	0.537	0.544	No	No
92483_g_at	Fmnl1	formin-like 1	1.0367	0.6916	0.0245	0.6862	No	1.0736	0.256	0.0629	0.685	No	No
92484_at	Hivep2	human immunodeficiency virus type I enhancer binding protein 2	1.0432	0.6667	0.0244	0.4308	No	0.9983	0.941	0.0013	0.681	No	No
92485_at	Bmp15	bone morphogenetic protein 15	1.0704	0.4197	0.749	0.2243	No	1.0152	0.917	0.591	0.636	No	No
92486_at	Slc22a21	solute carrier family 22 (organic cation transporter), member 21	1.018	0.6065	0.0035	0.3119	No	0.916	0.296	0.0341	0.524	No	No
92487_at	Sox7	SRY-box containing gene 7	1.0163	0.7746	0.2132	0.6626	No	0.9539	0.508	0.0999	0.218	No	No
92488_at	Casp12	caspase 12	0.9349	0.1572	0.059	0.0568	No	0.809	0.0486	0.527	0.88	No	No
92489_at	Gimap1	GTPase, IMAP family member 1	1.0844	0.0898	0.6143	0.0174	No	1.0209	0.731	0.183	0.215	No	No
92490_at	Kif9	kinesin family member 9	1.0038	0.8871	0.7934	0.7745	No	0.906	0.138	0.0453	0.486	No	No
92492_at	Ak3	adenylate kinase 3	0.8427	0.0886	0.021	0.5811	No	0.8495	0.0569	0.0238	0.432	No	No
92493_at	Rbpsuhl	recombining binding protein suppressor of hairless-like (Drosophila)	0.984	0.8562	0.5234	0.9988	No	0.9425	0.704	0.46	0.169	No	No
92494_at	Anxa10	annexin A10	0.9571	0.3983	0.3886	0.6022	No	0.959	0.102	0.0414	0.698	No	No
92495_at	Cnot4	CCR4-NOT transcription complex, subunit 4	0.981	0.6787	0.0246	0.0484	No	0.9861	0.821	0.0933	0.0529	No	No
92496_at	Vamp5	vesicle-associated membrane protein 5	0.9494	0.1464	0.9112	0.1106	No	1.0464	0.0571	0.0005	0.089	No	No
92497_at	Slc22a4	solute carrier family 22 (organic cation transporter), member 4	0.9812	0.5032	0.0049	0.2804	No	1.0271	0.757	0.0475	0.528	No	No
92498_at	Unc13b	unc-13 homolog B (C. elegans)	1.0901	0.1563	0.0328	0.3843	No	1.151	0.0175	0.361	0.157	No	No
92499_at	Uncx4.1	Unc4.1 homeobox (C. elegans)	1.1086	0.0597	0.2288	0.653	No	1.2385	0.0842	0.71	0.479	No	No
92500_at	Odz3	odd Oz/ten-m homolog 3 (Drosophila)	1.0263	0.7898	0.0073	0.304	No	0.9716	0.387	0.0108	0.478	No	No
92501_s_at	Plagl1	pleiomorphic adenoma gene-like 1	0.9583	0.6146	0.3362	0.2702	No	0.8883	0.261	0.568	0.431	No	No
92502_at	Plagl1	pleiomorphic adenoma gene-like 1	0.9561	0.4911	0.0334	0.0222	No	0.9742	0.304	0.0034	0.0114	No	No
92503_at	Hus1	Hus1 homolog (S. pombe)	0.9978	0.9751	0.026	0.9497	No	1.0391	0.109	0.0001	0.176	No	No
92504_at	Hus1	Hus1 homolog (S. pombe)	0.9511	0.2875	0.0332	0.2494	No	0.9213	0.0167	0.0018	0.937	No	No
92505_g_at	Hus1	Hus1 homolog (S. pombe)	1.0092	0.841	0.0875	0.7959	No	1.07	0.443	0.103	0.592	No	No
92506_at	Hapln1	hyaluronan and proteoglycan link protein 1	0.9979	0.9471	0.0002	0.0271	No	0.8812	0.0961	0.0005	0.409	No	No
92507_at	Utrn	utrophin	0.8929	0.4183	0.5332	0.6147	No	0.9476	0.543	0.569	0.315	No	No
92508_s_at	Utrn	utrophin	0.9514	0.383	0.7782	0.3333	No	0.9333	0.226	0.242	0.523	No	No
92509_at	F12	coagulation factor XII (Hageman factor)	0.9831	0.6149	0.017	0.1038	No	1.046	0.304	0.0249	0.237	No	No
92510_at	Jag2	jagged 2	1.0294	0.4693	0.0025	0.0384	No	0.9003	0.0254	0.0031	0.0124	No	No
92511_at	Aanat	arylalkylamine N-acetyltransferase	0.9675	0.431	0.0174	0.0252	No	0.9598	0.498	0.0044	0.449	No	No
92512_g_at	Aanat	arylalkylamine N-acetyltransferase	0.9675	0.5167	0.4267	0.6673	No	0.931	0.188	0.207	0.466	No	No
92513_at	Stag2	stromal antigen 2	0.8887	0.1494	0.9653	0.6361	No	0.8882	0.026	0.322	0.0663	No	No

92514_at	Barx1	BarH-like homeobox 1	0.998	0.9737	0.0065	0.6111	No	0.9901	0.992	0.0018	0.099	No	No
92515_at	Isl1	ISL1 transcription factor, LIM/homeodomain	0.9714	0.4652	0.0004	0.5007	No	1.0142	0.804	0.0005	0.894	No	No
92516_at	Ndst1	N-deacetylase/N-sulfotransferase (heparan glucosaminyl) 1	1.0428	0.3504	0.5504	0.9393	No	1.0738	0.306	0.0974	0.608	No	No
92517_at	Rbms2	RNA binding motif, single stranded interacting protein 2	0.8765	0.0334	0.0012	0.0956	No	0.9506	0.282	0.0005	0.705	No	No
92518_at	Neo1	neogenin	1.0408	0.52	0.0711	0.8983	No	1.112	0.0136	0.0094	0.148	No	No
92519_at	Phka1	phosphorylase kinase alpha 1	1.0409	0.5732	0.0228	0.2885	No	0.9989	0.803	0.0157	0.0238	No	No
92520_at	C80425	expressed sequence C80425	1.0124	0.6718	0.0274	0.6898	No	0.9595	0.337	0.0039	0.512	No	No
92521_at	Atbf1	AT motif binding factor 1	0.9673	0.1598	0.1096	0.1509	No	0.9516	0.143	0.0228	0.225	No	No
92522_at	Kcnj4	potassium inwardly-rectifying channel, subfamily J, member 4	0.9675	0.4285	0.0173	0.176	No	0.9844	0.609	0.129	0.161	No	No
92523_at	Kcnj6	potassium inwardly-rectifying channel, subfamily J, member 6	1.0978	0.0937	0.1202	0.3706	No	1.014	0.754	0.111	0.375	No	No
92524_at	Kcnj6	potassium inwardly-rectifying channel, subfamily J, member 6	1.0149	0.5277	0.056	0.8518	No	1.0534	0.374	0.0415	0.287	No	No
92525_i_at	Btbd14a	BTB (POZ) domain containing 14A	0.9441	0.2974	0.1804	0.5551	No	1.0695	0.447	0.829	0.94	No	No
92526_f_at	Btbd14a	BTB (POZ) domain containing 14A	0.9267	0.2535	0.0047	0.1278	No	0.9323	0.282	0.804	0.0154	No	No
92527_at	Adcy9	adenylate cyclase 9	1.0751	0.1121	0.0712	0.6271	No	1.0587	0.505	0.336	0.333	No	No
92528_at	Bai1	brain-specific angiogenesis inhibitor 1	1.0085	0.596	0.0055	0.0053	No	1.077	0.0565	0.0024	0.0731	No	No
92529_s_at	Arnt	aryl hydrocarbon receptor nuclear translocator	0.9317	0.3202	0.6749	0.5907	No	1.0641	0.281	0.0713	0.382	No	No
92531_at	Tloc1	translocation protein 1	0.9587	0.3392	0.0009	0.8667	No	1.0178	0.462	0.0011	0.09	No	No
92532_at	Avpr1a	arginine vasopressin receptor 1A	0.9696	0.6376	0.0227	0.6343	No	0.935	0.248	0.0104	0.843	No	No
92533_at	Calca	calcitonin/calcitonin-related polypeptide, alpha	1.082	0.1559	0.4526	0.1531	No	1.0168	0.755	0.121	0.319	No	No
92534_at	Gem	GTP binding protein (gene overexpressed in skeletal muscle)	0.9362	0.0251	0.292	0.0267	No	0.8951	0.0398	0.51	0.0421	No	No
92535_at	Ebf1	early B-cell factor 1	1.0899	0.0733	0.0001	0.8087	No	1.0644	0.233	0.0001	0.265	No	No
92536_at	Adrb3	adrenergic receptor, beta 3	1.1792	0.0208	0.0143	0.0292	No	1.2485	0.0042	0.0006	0.0089	No	No
92537_g_at	Adrb3	adrenergic receptor, beta 3	1.0236	0.7045	0.7527	0.3766	No	1.0725	0.0414	0.0387	0.0105	No	No
92538_at	Adrb3	adrenergic receptor, beta 3	0.9154	0.3251	0.4339	0.6746	No	1.0197	0.619	0.5	0.592	No	No
92539_at	S100a10	S100 calcium binding protein A10 (calpactin)	0.9216	0.1837	0.1878	0.1129	No	0.9268	0.167	0.0153	0.14	No	No
92540_f_at	Srm	spermidine synthase	1.1325	0.0197	0.0006	0.4246	No	1.0948	0.0199	0.0001	0.205	No	No
92541_at	Myl1	myosin, light polypeptide 1	0.7131	0.0004	0.0027	0.0004	No	0.8029	0.0005	0.0402	0.0002	No	No
92542_at	D4Wsu53e	DNA segment, Chr 4, Wayne State University 53, expressed	0.8459	0.1217	0.1324	0.5613	No	0.8241	0.0679	0.246	0.668	No	No
92543_at	Map2k2	mitogen activated protein kinase kinase 2	1.02	0.6924	0.3104	0.1426	No	0.9562	0.202	0.0742	0.0174	No	No
92544_f_at	Psma3	proteasome (prosome, macropain) subunit, alpha type 3	1.1627	0.0329	0.3071	0.387	No	1.1689	0.0277	0.848	0.183	No	No
92545_f_at	Ptgds	prostaglandin D2 synthase (brain)	0.9583	0.4276	0.0608	0.6631	No	0.98	0.815	0.0293	0.369	No	No
92546_r_at	Ptgds	prostaglandin D2 synthase (brain)	0.9631	0.2468	0.0088	0.0269	No	1.0122	0.643	0.414	0.0766	No	No
92547_at	Hip2	huntingtin interacting protein 2	1.0607	0.4359	0.0063	0.9723	No	1.0932	0.193	0.0033	0.692	No	No
92548_g_at	Hip2	huntingtin interacting protein 2	0.9161	0.1645	0.0649	0.9332	No	0.9728	0.538	0.0433	0.679	No	No
92549_at	Pkig	protein kinase inhibitor, gamma	1.0117	0.8463	0.5826	0.5574	No	1.0221	0.308	0.0097	0.869	No	No
92550_at	Krt1-19	keratin complex 1, acidic, gene 19	0.9462	0.2196	0.0022	0.0153	No	0.9633	0.782	0.0015	0.0114	No	No
92551_at	Lig1	ligase I, DNA, ATP-dependent	1.093	0.0145	0.0032	0.0807	No	1.1081	0.0004	0.0003	0.0008	No	No
92553_at	Esd	esterase D/formylglutathione hydrolase	1.0829	0.0939	0.0055	0.9508	No	1.0697	0.0922	0.0046	0.678	No	No
92554_at	Ctbp2	C-terminal binding protein 2	0.959	0.3376	0.0068	0.4153	No	1.0033	0.991	0.001	0.603	No	No
92555_at	Tspan6	tetraspanin 6	1.1353	0.0951	0.0729	0.4569	No	1.0375	0.494	0.0549	0.716	No	No
92556_at	Akr1c6	aldo-keto reductase family 1, member C6	1.1383	0.0336	0.0751	0.0978	No	1.116	0.0393	0.155	0.0107	No	No
92557_at	Hsd17b1	hydroxysteroid (17-beta) dehydrogenase 1	1.044	0.6775	0.4185	0.9005	No	0.9342	0.368	0.0006	0.772	No	No
92558_at	Vcam1	vascular cell adhesion molecule 1	0.7562	0.0014	0.0003	0.0512	Yes	0.9528	0.678	0.104	0.693	No	No
92559_at	Vcam1	vascular cell adhesion molecule 1	0.8966	0.3025	0.773	0.0831	No	0.8856	0.247	0.689	0.0902	No	No
92560_g_at	Vcam1	vascular cell adhesion molecule 1	0.8371	0.0021	0.0559	0.0054	No	0.9244	0.116	0.353	0.031	No	No
92561_at	Entpd5	ectonucleoside triphosphate diphosphohydrolase 5	1.034	0.3811	0.0624	0.7021	No	1.0232	0.616	0.0686	0.365	No	No
92562_at	Nfe2l2	nuclear factor, erythroid derived 2, like 2	0.9507	0.4673	0.2796	0.388	No	0.9194	0.132	0.206	0.14	No	No
92563_at	Lrrfip1	leucine rich repeat (in FLII) interacting protein 1	0.9193	0.0673	0.0628	0.9648	No	1.0328	0.225	0.005	0.0174	No	No
92564_at	Lrrfip1	leucine rich repeat (in FLII) interacting protein 1	1.0948	0.4052	0.0073	0.4765	No	1.0847	0.229	0.0004	0.671	No	No
92565_at	1110005A23 Rik	RIKEN cDNA 1110005A23 gene	1.0479	0.7095	0.9748	0.8127	No	1.0362	0.808	0.462	0.865	No	No
92566_at	Dapk3	death-associated kinase 3	0.9729	0.6219	0.1059	0.0609	No	0.8948	0.213	0.742	0.668	No	No
92567_at	Col5a2	procollagen, type V, alpha 2	0.9505	0.3133	0.0137	0.3555	No	0.9642	0.592	0.0516	0.185	No	No
92568_at	Tfb2m /// Gm1818	transcription factor B2, mitochondrial /// gene model 1818, (NCBI)	0.9695	0.4622	0.0016	0.2177	No	1.0021	0.998	0.247	0.776	No	No
92569_f_at	Nol5	nucleolar protein 5	1.0904	0.0592	0.314	0.0065	No	1.1734	0.0324	0.0125	0.91	No	No
92570_at	Atp5g2 /// LOC626403	ATP synthase, H+ transporting, mitochondrial F0 complex, subunit c (subunit 9), isoform 2 /// similar to ATP synthase, H+ transporting, mitochondrial F0 complex, subunit c (subunit 9), isoform 2	1.0821	0.2202	0.3239	0.0243	No	1.0581	0.234	0.592	0.0564	No	No
92571_at	Hspa4	heat shock protein 4	1.078	0.2761	0.0384	0.9775	No	0.9864	0.855	0.188	0.557	No	No
92572_at	Tnfaip1	tumor necrosis factor, alpha-induced protein 1 (endothelial)	0.9696	0.6207	0.0616	0.9444	No	0.9881	0.535	0.0001	0.315	No	No
92573_at	Ppp2r1a	protein phosphatase 2 (formerly 2A), regulatory subunit A (PR 65), alpha isoform	1.0766	0.1141	0.1954	0.5075	No	1.0633	0.0199	0.0205	0.513	No	No
92574_at	Sdhd	succinate dehydrogenase complex, subunit D, integral membrane protein	1.0615	0.0327	0.0058	0.0047	No	1.0126	0.636	0.0355	0.0571	No	No
92575_at	Aip	aryl-hydrocarbon receptor-interacting protein	1.0107	0.8069	0.0067	0.6614	No	1.0192	0.572	0.0015	0.412	No	No
92577_f_at	Rpl37	ribosomal protein L37	1.0393	0.3626	0.011	0.6246	No	0.9785	0.583	0.0077	0.0993	No	No
92578_at	Scye1	small inducible cytokine subfamily E, member 1	1.0336	0.5315	0.6064	0.1964	No	1.0307	0.591	0.184	0.264	No	No
92579_at	Ssb	Sjogren syndrome antigen B	1.1654	0.1947	0.0012	0.5747	No	1.2098	0.271	0.0088	0.714	No	No
92580_at	Hars	histidyl-tRNA synthetase	1.0875	0.0977	0.0576	0.5127	No	1.0462	0.0977	0.0008	0.686	No	No
92581_at	Acadm	acyl-Coenzyme A dehydrogenase, medium chain	1.07	0.2954	0.7649	0.5931	No	1.0115	0.803	0.133	0.791	No	No
92582_at	Slc1a5	solute carrier family 1 (neutral amino acid transporter), member 5	1.0096	0.7801	0.001	0.6421	No	1.0181	0.845	0.0039	0.316	No	No
92583_at	Serpina3k	serine (or cysteine) peptidase inhibitor, clade A, member 3K	1.0367	0.5125	0.1811	0.7768	No	1.0275	0.502	0.462	0.56	No	No
92584_at	Nkiras2	NFKB inhibitor interacting Ras-like protein 2	0.9921	0.6799	0.0034	0.5762	No	0.9622	0.0473	0.0001	0.234	No	No
92585_at	Map2k1	mitogen activated protein kinase kinase 1	1.0367	0.2773	0.0045	0.1099	No	1.032	0.565	0.0211	0.664	No	No
92586_at	Glud1	glutamate dehydrogenase 1	0.9672	0.616	0.0307	0.8767	No	0.952	0.27	0.0608	0.0231	No	No
92587_at	Fdx1	ferredoxin 1	1.0239	0.609	0.0477	0.1032	No	0.9722	0.378	0.131	0.0233	No	No
92589_at	Psph	phosphoserine phosphatase	1.0484	0.255	0.0078	0.8961	No	1.0627	0.307	0.0364	0.746	No	No
92590_at	Hmgcs2	3-hydroxy-3-methylglutaryl-Coenzyme A synthase 2	0.937	0.2972	0.0564	0.768	No	1.0024	0.996	0.0148	0.87	No	No
92592_at	Gpd1	glycerol-3-phosphate dehydrogenase 1 (soluble)	1.0647	0.1092	0.0113	0.9054	No	1.0336	0.158	0.0072	0.368	No	No
92593_at	Postn	periostin, osteoblast specific factor	0.9762	0.2317	0.142	0.009	No	0.9588	0.303	0.0166	0.0944	No	No
92594_at	Ap1g2	adaptor protein complex AP-1, gamma 2 subunit	1.0381	0.5317	0.0038	0.4794	No	1.0083	0.764	0.216	0.0059	No	No
92595_r_at	Fech	ferrochelatase	1.0053	0.9376	0.0042	0.1149	No	1.2313	0.33	0.0048	0.968	No	No
92596_at	Cacybp	calcyclin binding protein	1.2256	0.0312	0.1421	0.8544	No	1.0581	0.147	0.0305	0.247	No	No
92597_s_at	Atp6v1b2	ATPase, H+ transporting, lysosomal V1 subunit B2	1.0551	0.4803	0.0088	0.4206	No	1.0415	0.552	0.874	0.0603	No	No
92598_at	Atp6v1b2	ATPase, H+ transporting, lysosomal V1 subunit B2	0.9021	0.0042	0.0004	0.6139	Yes	0.9239	0.0663	0.0297	0.495	No	No
92599_at	Pgam2	phosphoglycerate mutase 2	0.7056	0.0013	0.0304	0.0006	No	0.7725	0.0089	0.475	0.0031	No	No
92600_f_at	Cyp4a10	cytochrome P450, family 4, subfamily a, polypeptide 10	0.9775	0.5957	0.1507	0.2962	No	0.9231	0.508	0.892	0.182	No	No
92601_at	Pnliprp1	pancreatic lipase related protein 1	0.9541	0.3026	0.8543	0.5254	No	0.9706	0.293	0.0035	0.0453	No	No
92602_at	Tcap	titin-cap	0.9197	0.0473	0.0039	0.0103	No	0.8563	0.24	0.63	0.184	No	No
92603_at	Atp6v0d1	ATPase, H+ transporting, lysosomal V0 subunit D1	0.9614	0.5638	0.7859	0.7718	No	0.9741	0.696	0.618	0.698	No	No
92605_at	Umod	uromodulin	0.9836	0.555	0.6632	0.1003	No	1.3925	0.0049	0.247	0.0178	Yes	No
92606_at	Uox	urate oxidase	0.9256	0.1238	0.5196	0.2304	No	0.9212	0.127	0.127	0.0227	No	No
92607_at	Mest	mesoderm specific transcript	1.1444	0.092	0.0777	0.2793	No	1.1363	0.0487	0.769	0.0976	No	No
92608_at	Csrp1	cysteine and glycine-rich protein 1	0.9559	0.4771	0.4773	0.0716	No	0.9106	0.185	0.0159	0.0657	No	No
92610_at	Rdbp	RD RNA-binding protein	1.0608	0.0799	0.0002	0.3224	No	1.0598	0.0932	0.0001	0.641	No	No
92611_at	Gpiap1	GPI-anchored membrane protein 1	0.9987	0.9891	0.2913	0.3279	No	0.989	0.451	0.0009	0.0238	No	No
92612_at	Slc27a5	solute carrier family 27 (fatty acid transporter), member 5	1.0154	0.7698	0.0183	0.576	No	1.1372	0.164	0.0546	0.784	No	No
92614_at	Id3	inhibitor of DNA binding 3	0.9655	0.1988	0.0005	0.0292	No	0.9596	0.385	0.0011	0.0558	No	No
92615_at	Ndufb6	NADH dehydrogenase (ubiquinone) 1 beta subcomplex, 6	1.1128	0.0418	0.0316	0.865	No	1.1346	0.0091	0.0103	0.0483	Yes	Yes
92616_at	Ube1x	ubiquitin-activating enzyme E1, Chr X	1.0401	0.2969	0.0056	0.0681	No	1.0076	0.813	0.153	0.456	No	No
92617_at	Ube1y1	ubiquitin-activating enzyme E1, Chr Y 1	0.9466	0.06	0.0689	0.8029	No	0.8492	0.0243	0.372	0.323	No	No
92618_at	Serf2	small EDRK-rich factor 2	0.9106	0.447	0.3028	0.2605	No	0.9064	0.294	0.0542	0.327	No	No
92619_at	Wbp1	WW domain binding protein 1	0.9745	0.5396	0.2708	0.2816	No	0.9674	0.604	0.0146	0.477	No	No
92621_at	Pcbp2	poly(rC) binding protein 2	1.0222	0.6626	0.028	0.3831	No	1.0336	0.627	0.0856	0.281	No	No

92622_at	2210013K02 Rik	RIKEN cDNA 2210013K02 gene	1.0046	0.9101	0.2356	0.6039	No	1.0089	0.913	0.285	0.853	No	No
92623_at	Csde1	cold shock domain containing E1, RNA binding	0.9946	0.815	0.0242	0.0104	No	0.9733	0.139	0.627	0.0025	No	No
92624_r_at	Lor	loricrin	1.0093	0.8738	0.9323	0.1913	No	1.0166	0.709	0.516	0.13	No	No
92625_at	Nme2	expressed in non-metastatic cells 2, protein	1.0605	0.0805	0.0001	0.1128	No	1.0719	0.074	0.0003	0.111	No	No
92626_at	Npdc1	neural proliferation, differentiation and control gene 1	0.994	0.7796	0.0002	0.0233	No	1.0106	0.42	0.0001	0.0715	No	No
92628_at	LOC622534	similar to ribosomal protein L36	0.955	0.4314	0.0042	0.0828	No	0.9549	0.602	0.0064	0.119	No	No
92629_f_at	Hdgf	hepatoma-derived growth factor	1.0216	0.4603	0.0046	0.1053	No	1.0435	0.102	0.0033	0.154	No	No
92630_r_at	Hdgf	hepatoma-derived growth factor	0.9474	0.0759	0.0978	0.8963	No	0.9686	0.306	0.0017	0.9	No	No
92631_f_at	Calm3	calmodulin 3	0.984	0.562	0.0003	0.1452	No	0.9387	0.185	0.0052	0.736	No	No
92632_at	Calm3	calmodulin 3	1.0164	0.7676	0.0425	0.4811	No	1.0359	0.614	0.0142	0.645	No	No
92633_at	Ctsz	cathepsin Z	0.9381	0.4854	0.1409	0.292	No	0.9803	0.729	0.297	0.178	No	No
92634_at	Dpp4	dipeptidylpeptidase 4	0.9764	0.4826	0.1051	0.4975	No	0.9703	0.664	0.232	0.822	No	No
92635_at	Tuba4	tubulin, alpha 4	0.8745	0.0028	0.0038	0.0914	Yes	0.9888	0.88	0.594	0.799	No	No
92636_f_at	Sec61g	SEC61, gamma subunit	1.1281	0.1537	0.0308	0.6064	No	1.0821	0.208	0.0216	0.858	No	No
92637_at	Pfkl	phosphofructokinase, liver, B-type	1.1115	0.0152	0.0003	0.5501	No	1.1335	0.0397	0.0011	0.116	No	No
92638_at	Ppp2ca	protein phosphatase 2 (formerly 2A), catalytic subunit, alpha isoform	1.1269	0.2518	0.0092	0.3525	No	1.2035	0.0107	0.0007	0.0698	No	No
92639_at	Aurka	aurora kinase A	0.9395	0.253	0.2641	0.7168	No	0.9098	0.148	0.959	0.882	No	No
92640_at	Vps35	vacuolar protein sorting 35	1.0065	0.9135	0.0162	0.649	No	0.98	0.929	0.0273	0.478	No	No
92642_at	Car2	carbonic anhydrase 2	0.8828	0.0208	0.0058	0.5113	No	0.9227	0.0905	0.0328	0.146	No	No
92643_at	Nf2	neurofibromatosis 2	0.9718	0.2056	0.0022	0.0009	No	0.9677	0.475	0.377	0.0441	No	No
92644_s_at	Myb	myeloblastosis oncogene	0.9175	0.4385	0.1974	0.963	No	0.9692	0.664	0.0324	0.757	No	No
92645_at	Psmd2	proteasome (prosome, macropain) 26S subunit, non-ATPase, 2	0.998	0.9759	0.4079	0.3436	No	1.0367	0.413	0.0231	0.0638	No	No
92646_at	Mrpl23	mitochondrial ribosomal protein L23	0.9759	0.3834	0.0006	0.0059	No	0.9569	0.21	0.0017	0.0054	No	No
92647_at	Rbbp4	retinoblastoma binding protein 4	1.0933	0.3399	0.3278	0.5817	No	1.0842	0.345	0.611	0.689	No	No
92648_at	Stxbp3a	syntaxin binding protein 3A	0.985	0.8904	0.2274	0.4746	No	0.9766	0.678	0.221	0.134	No	No
92649_at	D0HXS9928 E	DNA segment, human DXS9928E	0.9808	0.7719	0.1045	0.2969	No	1.0138	0.9	0.0015	0.152	No	No
92650_at	Man1a2	mannosidase, alpha, class 1A, member 2	0.999	0.9886	0.2622	0.6917	No	0.9246	0.392	0.308	0.311	No	No
92652_at	Notch4	Notch gene homolog 4 (Drosophila)	0.8816	0.0236	0.0107	0.2438	No	0.9528	0.49	0.0744	0.633	No	No
92653_at	D530037H1 2Rik	RIKEN cDNA D530037H12 gene	0.947	0.5649	0.9192	0.8881	No	1.0484	0.365	0.403	0.0179	No	No
92654_at	Polr3a	polymerase (RNA) III (DNA directed) polypeptide A	1.0024	0.8127	0.0072	0.289	No	0.9722	0.458	0.0113	0.113	No	No
92655_at	Gcnt1 /// LOC635918	glucosaminyl (N-acetyl) transferase 1, core 2 /// similar to glucosaminyl (N-acetyl) transferase 1, core 2	1.0245	0.6634	0.9191	0.7632	No	0.995	0.81	0.63	0.196	No	No
92656_at	Zbtb12	zinc finger and BTB domain containing 12	1.0897	0.2829	0.0443	0.6956	No	0.9601	0.482	0.317	0.102	No	No
92658_at	Foxq1	forkhead box Q1	1.036	0.3659	0.1362	0.8513	No	1.0443	0.314	0.0096	0.912	No	No
92659_at	Rapgef4	Rap guanine nucleotide exchange factor (GEF) 4	1.0044	0.9103	0.0108	0.1715	No	1.0292	0.554	0.0094	0.723	No	No
92660_f_at	Ube2e1	ubiquitin-conjugating enzyme E2E 1, UBC4/5 homolog (yeast)	1.1081	0.1967	0.0027	0.0944	No	1.0367	0.763	0.0325	0.369	No	No
92661_at	Ptprv	protein tyrosine phosphatase, receptor type, V	0.9329	0.1174	0.9391	0.6761	No	0.9412	0.44	0.593	0.937	No	No
92662_g_at	Ptprv	protein tyrosine phosphatase, receptor type, V	1.075	0.0584	0.4699	0.0811	No	0.8732	0.0363	0.0158	0.232	No	No
92663_at	Ptprv	protein tyrosine phosphatase, receptor type, V	0.9827	0.6595	0.3268	0.1395	No	0.9121	0.0634	0.0059	0.0385	No	No
92664_at	2500002G23 Rik	RIKEN cDNA 2500002G23 gene	0.9593	0.1644	0.2214	0.0838	No	0.9606	0.302	0.181	0.14	No	No
92665_f_at	Xlr /// 3830403N18 Rik	X-linked lymphocyte-regulated complex /// RIKEN cDNA 3830403N18 gene	1.0869	0.2316	0.0172	0.4355	No	1.0748	0.292	0.0186	0.676	No	No
92666_at	Sh3bp1	SH3-domain binding protein 1	1.0308	0.402	0.2399	0.3945	No	1.0212	0.706	0.517	0.558	No	No
92667_at	Ar	androgen receptor	1.0656	0.0819	0.0197	0.0062	No	1.0129	0.56	0.0098	0.271	No	No
92668_at	Btk	Bruton agammaglobulinemia tyrosine kinase	0.9843	0.5693	0.0052	0.542	No	1.0084	0.807	0.0079	0.571	No	No
92670_at	Myl6b	myosin, light polypeptide 6B	0.916	0.0407	0.0031	0.0122	No	0.8948	0.335	0.286	0.592	No	No
92671_f_at	Rgl1	ral guanine nucleotide dissociation stimulator,-like 1	0.9573	0.6283	0.5265	0.2621	No	0.9229	0.204	0.0795	0.291	No	No
92672_at	Gnas	GNAS (guanine nucleotide binding protein, alpha stimulating) complex locus	1.0356	0.7072	0.261	1	No	1.0914	0.209	0.0806	0.0446	No	No
92673_at	Sh3gl2	SH3-domain GRB2-like 2	0.9549	0.5111	0.0287	0.605	No	0.9765	0.833	0.0087	0.0962	No	No
92674_at	Foxn1	forkhead box N1	1.0496	0.0786	0.0018	0.2787	No	1.0208	0.308	0.001	0.0491	No	No
92676_at	Runx2	runt related transcription factor 2	0.9662	0.6471	0.9876	0.2911	No	0.9606	0.798	0.272	0.607	No	No
92677_s_at	Runx2	runt related transcription factor 2	0.9792	0.5657	0.0251	0.7444	No	0.9148	0.269	0.0331	0.325	No	No
92678_at	Ddx25	DEAD (Asp-Glu-Ala-Asp) box polypeptide 25	1.2177	0.0038	0.1266	0.0339	Yes	1.1608	0.0017	0.0019	0.0019	No	No
92681_at	Magel2	melanoma antigen, family L, 2	1.0032	0.9548	0.1551	0.1558	No	1.0757	0.114	0.0161	0.169	No	No
92682_at	Syt8	synaptotagmin VIII	0.9796	0.6596	0.6715	0.4319	No	0.9356	0.0556	0.478	0.0084	No	No
92683_at	Cd3d	CD3 antigen, delta polypeptide	0.9209	0.2177	0.0027	0.096	No	0.9404	0.493	0.0248	0.959	No	No
92684_at	Prss15	protease, serine, 15	0.9318	0.3066	0.4887	0.7139	No	0.9544	0.192	0.0452	0.905	No	No
92685_at	Tbxa2r	thromboxane A2 receptor	1.0249	0.6579	0.2353	0.7944	No	0.9848	0.678	0.262	0.138	No	No
92686_at	Slc27a3	solute carrier family 27 (fatty acid transporter), member 3	1.0782	0.0529	0.0022	0.6015	No	1.1261	0.018	0.002	0.621	No	No
92687_at	Smarca4	SWI/SNF related, matrix associated, actin dependent regulator of chromatin, subfamily a, member 4	1.0474	0.4152	0.0825	0.4115	No	1.1536	0.0267	0.0932	0.0486	No	No
92688_at	Acp2	acid phosphatase 2, lysosomal	0.988	0.6532	0.2582	0.4194	No	1.024	0.408	0.245	0.668	No	No
92689_at	Il18bp	interleukin 18 binding protein	1.0235	0.5332	0.0892	0.16	No	0.9745	0.753	0.845	0.671	No	No
92690_at	Gif	gastric intrinsic factor	0.9878	0.7647	0.0031	0.3796	No	0.9543	0.609	0.818	0.781	No	No
92691_at	Ppp2r2b	protein phosphatase 2 (formerly 2A), regulatory subunit B (PR 52), beta isoform	0.9077	0.015	0.0853	0.6269	No	0.9263	0.0317	0.33	0.762	No	No
92692_at	Sycp1	synaptonemal complex protein 1	0.952	0.6668	0.2322	0.6492	No	1.0742	0.505	0.0329	0.969	No	No
92693_at	Nin	ninein	1.0289	0.5918	0.0547	0.2767	No	0.9905	0.851	0.0854	0.747	No	No
92694_at	Chi3l3	chitinase 3-like 3	0.9704	0.3169	0.7617	0.7469	No	1.0449	0.472	0.139	0.371	No	No
92695_at	Frat1	frequently rearranged in advanced T-cell lymphomas	0.9681	0.4852	0.0122	0.6344	No	1.0284	0.391	0.172	0.0865	No	No
92696_at	Nr6a1	nuclear receptor subfamily 6, group A, member 1	1.1432	0.0706	0.9971	0.0363	No	1.1621	0.0254	0.193	0.0389	No	No
92697_at	Foxa1	forkhead box A1	1.1215	0.0759	0.0686	0.7566	No	0.9814	0.312	0.0107	0.0131	No	No
92698_at	Mertk	c-mer proto-oncogene tyrosine kinase	0.9145	0.2337	0.098	0.3644	No	0.8566	0.0114	0.0002	0.301	No	No
92699_at	Slc7a9	solute carrier family 7 (cationic amino acid transporter, y+ system), member 9	1.149	0.0451	0.0564	0.9281	No	0.8674	0.0346	0.0001	0.0016	No	No
92700_at	Bcan	brevican	1.0548	0.2767	0.2996	0.5407	No	1.049	0.568	0.328	0.146	No	No
92701_at	Bmp1	bone morphogenetic protein 1	1.0603	0.2081	0.0067	0.0534	No	1.0259	0.332	0.001	0.0753	No	No
92702_at	Astn1	astrotactin 1	1.027	0.0463	0	0.0024	No	1.0331	0.135	0.0004	0.0107	No	No
92703_at	Pb1	polybromo 1	1.0958	0.1508	0.0038	0.9862	No	1.0497	0.0757	0.0003	0.52	No	No
92704_at	Ercc4	excision repair cross-complementing rodent repair deficiency, complementation group 4	0.966	0.2603	0.0074	0.4243	No	1.0057	0.897	0.915	0.414	No	No
92705_at	Tbx2	T-box 2	0.9923	0.8545	0.0053	0.2491	No	0.8843	0.0687	0.36	0.0321	No	No
92706_at	Pvt1	plasmacytoma variant translocation 1	1.0246	0.4811	0.188	0.0441	No	0.9591	0.388	0.0144	0.16	No	No
92707_at	Pvt1	plasmacytoma variant translocation 1	0.9619	0.3543	0.9729	0.2806	No	1.0493	0.467	0.0574	0.698	No	No
92708_at	Ddx19b	DEAD (Asp-Glu-Ala-Asp) box polypeptide 19b	0.9966	0.9312	0.0792	0.4141	No	0.9587	0.183	0.582	0.957	No	No
92710_at	Rgnef	Rho-guanine nucleotide exchange factor	1.0114	0.8023	0.2278	0.4944	No	0.9846	0.3	0.001	0.009	No	No
92711_at	Mcpt6	mast cell protease 6	1.0084	0.9028	0.6419	0.6407	No	0.933	0.209	0.391	0.319	No	No
92712_at	1810009J06 Rik	RIKEN cDNA 1810009J06 gene	0.9767	0.7837	0.4635	0.6126	No	1.0525	0.365	0.105	0.0396	No	No
92713_at	Hnf4a	hepatic nuclear factor 4, alpha	0.8803	0.3086	0.2241	0.6264	No	0.9756	0.708	0.593	0.422	No	No
92714_at	Icam5	intercellular adhesion molecule 5, telencephalin	0.9582	0.4771	0.0238	0.8682	No	0.9778	0.761	0.127	0.625	No	No
92715_at	Ubd	ubiquitin D	0.9574	0.2511	0.0028	0.4714	No	0.9381	0.167	0.916	0.193	No	No
92717_at	Neurod1	neurogenic differentiation 1	1.0799	0.1764	0.7698	0.4783	No	1.1476	0.182	0.45	0.0786	No	No
92718_at	Ifi27	interferon, alpha-inducible protein 27	0.8705	0.0374	0.0942	0.5615	No	0.8684	0	0	0.0003	No	No
92722_f_at	Six1	sine oculis-related homeobox 1 homolog (Drosophila)	0.9833	0.8151	0.0033	0.8489	No	1.0115	0.797	0.0024	0.645	No	No
92724_at	Hnrpa1	heterogeneous nuclear ribonucleoprotein A1	0.9199	0.0698	0.283	0.044	No	0.9322	0.512	0.959	0.693	No	No
92725_at	Gas8	growth arrest specific 8	1.0374	0.5998	0.3631	0.2096	No	1.0105	0.819	0.932	0.764	No	No
92726_at	Sox6	SRY-box containing gene 6	0.9954	0.6383	0.0015	0.1166	No	0.9321	0.323	0.501	0.955	No	No
92727_at	Apba2	amyloid beta (A4) precursor protein-binding, family A, member 2	0.9748	0.6584	0.5293	0.9679	No	0.9752	0.452	0.661	0.733	No	No
92728_at	Prg2	proteoglycan 2, bone marrow	0.9256	0.2731	0.0087	0.1336	No	0.9131	0.199	0.0283	0.928	No	No
92730_at	Hbegf	heparin-binding EGF-like growth factor	0.9525	0.0736	0.1819	0.4786	No	1.0325	0.581	0.973	0.687	No	No
92731_at	Ptx3	pentraxin related gene	1.063	0.1818	0.0213	0.1119	No	1.0597	0.0883	0.0014	0.0392	No	No
92732_at	Adam2	a disintegrin and metallopeptidase domain 2	1.0287	0.5405	0.5848	0.0544	No	1.236	0.0563	0.557	0.0093	No	No
92733_at	Adam4	a disintegrin and metallopeptidase domain 4	1.0359	0.3177	0.1473	0.4015	No	1.145	0.0335	0.0031	0.0229	No	No
92734_at	Gpr83	G protein-coupled receptor 83	1.0913	0.0384	0.7076	0.0883	No	1.0314	0.6	0.182	0.349	No	No
92735_at	Pla2g2a	phospholipase A2, group IIA (platelets, synovial fluid)	1.0134	0.8344	0.3368	0.9955	No	0.977	0.896	0.905	0.194	No	No
92736_at	Slc7a2	solute carrier family 7 (cationic amino acid transporter, y+ system), member 2	1.0167	0.432	0.7121	0.205	No	1.0746	0.0301	0.0041	0.482	No	No

92737_at	Irf4	interferon regulatory factor 4	0.9913	0.6349	0.0104	0.0733	No	0.9881	0.993	0.0058	0.155	No	No
92738_at	Gdnf	glial cell line derived neurotrophic factor	1.0642	0.4053	0.5535	0.467	No	1.0207	0.738	0.0547	0.454	No	No
92739_at	Ivl	involucrin	0.8522	0.169	0.3352	0.7892	No	0.8791	0.186	0.322	0.656	No	No
92740_at	Igh-1a	Immunoglobulin heavy chain 1a (serum IgG2a)	0.9163	0.2916	0.2185	0.4096	No	0.9696	0.459	0.0083	0.106	No	No
92741_g_at	Igh-1a	Immunoglobulin heavy chain 1a (serum IgG2a)	0.9	0.2795	0.073	0.2828	No	0.8515	0.0939	0.002	0.0657	No	No
92742_at	Ccl11	small chemokine (C-C motif) ligand 11	1.0502	0.346	0.6135	0.6517	No	1.0952	0.128	0.0625	0.0514	No	No
92743_at	Spam1	sperm adhesion molecule 1	1.0397	0.6005	0.1196	0.9535	No	1.0349	0.481	0.568	0.48	No	No
92744_at	Apc2	adenomatosis polyposis coli 2	0.9799	0.7715	0.1293	0.2304	No	0.9376	0.0036	0.501	0.219	Yes	No
92745_at	Hoxa9	homeo box A9	1.162	0.0024	0.2898	0.003	No	1.1307	0.0362	0.756	0.0144	No	No
92746_at	Nat2	N-acetyltransferase 2 (arylamine N-acetyltransferase)	0.9218	0.0988	0.4544	0.6468	No	0.9021	0.149	0.116	0.45	No	No
92747_at	Nkx2-2	NK2 transcription factor related, locus 2 (Drosophila)	0.9589	0.5856	0.3859	0.6592	No	1.0943	0.108	0.0022	0.103	No	No
92749_at	Gabrb1 /// B230208N19 Rik	gamma-aminobutyric acid (GABA-A) receptor, subunit beta 1 /// RIKEN cDNA B230208N19 gene	1.1155	0.0157	0.2382	0.0089	No	1.0693	0.254	0.0736	0.361	No	No
92750_s_at	Wnt10b	wingless related MMTV integration site 10b	0.9781	0.7422	0.4166	0.2822	No	1.0095	0.921	0.174	0.14	No	No
92751_i_at	Wnt10b	wingless related MMTV integration site 10b	0.9802	0.656	0.8899	0.4172	No	1.0787	0.158	0.0066	0.0291	No	No
92752_r_at	Wnt10b	wingless related MMTV integration site 10b	1.1277	0.0395	0.0034	0.0273	No	1.1256	0.076	0.0037	0.0095	No	No
92753_at	Mrg2	myeloid ecotropic viral integration site-related gene 2	0.9313	0.2512	0.0083	0.7391	No	0.9999	0.995	0.396	0.452	No	No
92754_at	Fdxr	ferredoxin reductase	0.9863	0.581	0.0092	0.932	No	0.9561	0.44	0.0204	0.511	No	No
92755_f_at	Sct	secretin	0.9608	0.4885	0.3221	0.9198	No	0.917	0.0469	0.506	0.0248	No	No
92756_r_at	Sct	secretin	0.9784	0.4922	0.8573	0.6728	No	1.0015	0.962	0.183	0.565	No	No
92757_at	Sez6	seizure related gene 6	0.9068	0.4136	0.3601	0.4017	No	0.6852	0.267	0.876	0.182	No	No
92758_at	Dusp2	dual specificity phosphatase 2	1.0341	0.3675	0.0129	0.0454	No	0.9986	0.915	0.0827	0.14	No	No
92759_at	Lamb3	laminin, beta 3	0.9926	0.8806	0.0008	0.1399	No	1.0481	0.332	0.0048	0.829	No	No
92760_at	Was	Wiskott-Aldrich syndrome homolog (human)	1.0395	0.405	0.3289	0.5451	No	0.9656	0.358	0.0323	0.397	No	No
92761_at	---	---	0.9566	0.4321	0.1636	0.865	No	0.9169	0.239	0.413	0.696	No	No
92762_at	Clec4a2	C-type lectin domain family 4, member a2	0.9089	0.1039	0.166	0.9057	No	0.8693	0.0038	0.0234	0.151	Yes	No
92763_at	Abcb7	ATP-binding cassette, sub-family B (MDR/TAP), member 7	1.0485	0.3983	0.0177	0.29	No	1.0001	0.71	0.0114	0.157	No	No
92764_at	Ksr1	kinase suppressor of ras 1	0.9446	0.3344	0.6162	0.2603	No	1.1017	0.365	0.0373	0.252	No	No
92765_s_at	Ksr1	kinase suppressor of ras 1	1.05	0.2969	0.0139	0.0908	No	1.1411	0.0735	0.345	0.489	No	No
92766_at	Hand1	heart and neural crest derivatives expressed transcript 1	0.9822	0.4598	0.5564	0.068	No	1.0146	0.418	0.0039	0.0803	No	No
92767_at	Bmpr1a	bone morphogenetic protein receptor, type 1A	1.0105	0.8782	0.1628	0.7174	No	0.987	0.87	0.327	0.724	No	No
92768_s_at	Alas2	aminolevulinic acid synthase 2, erythroid	0.9609	0.1832	0.0001	0.0453	No	0.998	0.605	0.0001	0.0904	No	No
92769_at	Psmd3	proteasome (prosome, macropain) 26S subunit, non-ATPase, 3	1.0403	0.3433	0.0052	0.5236	No	1.0637	0.0243	0.827	0.0013	No	No
92770_at	S100a6	S100 calcium binding protein A6 (calcyclin)	0.9611	0.6565	0.0225	0.0819	No	0.9691	0.999	0.002	0.0177	No	No
92771_at	Zfp207	zinc finger protein 207	0.9529	0.4862	0.0146	0.2633	No	0.9733	0.666	0.0602	0.671	No	No
92773_at	Nrp1	neuropilin 1	0.9929	0.9533	0.0123	0.9298	No	0.947	0.774	0.0061	0.365	No	No
92774_at	LOC230010 /// Pabpc4 /// LOC432881 /// LOC544782 /// LOC624403 /// LOC624424 /// LOC673348 /// LOC673528	similar to poly(A) binding protein, cytoplasmic 4 isoform 1 /// poly A binding protein, cytoplasmic 4 /// similar to Poly(A) binding protein, cytoplasmic 4, isoform 1 /// similar to Poly(A) binding protein, cytoplasmic 4, isoform 1 /// similar to poly(A) binding protein, cytoplasmic 4 isoform 1 /// similar to poly(A) binding protein, cytoplasmic 4 isoform 1 /// similar to poly(A) binding protein, cytoplasmic 4 isoform 2 /// similar to poly(A) binding protein, cytoplasmic 4 isoform 1	0.9455	0.2771	0.0094	0.1402	No	1.0581	0.121	0	0.286	No	No
92775_at	Pabpc4	poly A binding protein, cytoplasmic 4	0.934	0.3088	0.0632	0.8843	No	0.9959	0.871	0.542	0.15	No	No
92777_at	Cyr61	cysteine rich protein 61	0.7393	0.0135	0.0026	0.3318	No	0.7182	0.0095	0.0872	0.994	Yes	Yes
92778_i_at	---	---	0.9633	0.5649	0.0023	0.6796	No	1.0119	0.38	0.0001	0.0836	No	No
92779_f_at	LOC634450	superantigen 3	0.9111	0.3084	0.3276	0.8561	No	1.0334	0.685	0.404	0.0717	No	No
92780_f_at	LOC625794	hypothetical protein LOC625794	0.9586	0.4234	0.0184	0.9593	No	0.9168	0.125	0.0043	0.501	No	No
92781_at	Tmpo	thymopoietin	1.1338	0.2393	0.6281	0.1313	No	1.0087	0.698	0.0352	0.0302	No	No
92782_at	Tmpo	thymopoietin	0.9498	0.3577	0.4109	0.5244	No	0.9699	0.388	0.108	0.204	No	No
92783_at	Gh	growth hormone	0.7396	0.0002	0	0	No	1.0223	0.0607	0.0005	0.0006	No	No
92784_at	Gba2	glucosidase beta 2	0.9597	0.5537	0.07	0.8354	No	1.0027	0.952	0.0039	0.416	No	No
92786_at	Efhd1	EF hand domain containing 1	1.1155	0.0487	0.0034	0.3979	No	1.0901	0.166	0.0076	0.661	No	No
92787_at	Ankrd10	ankyrin repeat domain 10	1.0085	0.5977	0.1161	0.1842	No	1.0189	0.676	0.0073	0.464	No	No
92788_f_at	Cetn3	centrin 3	0.9835	0.7388	0.2638	0.4904	No	1.0055	0.834	0.237	0.304	No	No
92789_r_at	Cetn3	centrin 3	1.1665	0.0519	0.0096	0.1661	No	1.2568	0.0667	0.0443	0.946	No	No
92790_at	Kpna2 /// LOC670551 /// LOC671639 /// LOC673776	karyopherin (importin) alpha 2 /// similar to Importin alpha-2 subunit (Karyopherin alpha-2 subunit) (SRP1-alpha) (RAG cohort protein 1) (Pendulin) (Pore targeting complex 58 kDa subunit) (PTAC58) (Importin alpha P1) /// similar to Importin alpha-2 subunit (Karyopherin alpha-2 subunit) (SRP1-alpha) (RAG cohort protein 1) (Pendulin) (Pore targeting complex 58 kDa subunit) (PTAC58) (Importin alpha P1) /// similar to Importin alpha-2 subunit (Karyopherin alpha-2 subunit) (SRP1-alpha) (RAG cohort protein 1) (Pendulin) (Pore targeting complex 58 kDa subunit) (PTAC58) (Importin alpha P1)	1.0842	0.3702	0.0086	0.7643	No	1.0465	0.316	0.001	0.973	No	No
92792_at	Ucp2	uncoupling protein 2 (mitochondrial, proton carrier)	1.0092	0.8899	0.3809	0.741	No	1.0113	0.97	0.161	0.904	No	No
92793_at	Tnfrsf1a	tumor necrosis factor receptor superfamily, member 1a	0.9563	0.3065	0.0047	0.0574	No	0.9423	0.352	0.0051	0.446	No	No
92794_f_at	Nme1	expressed in non-metastatic cells 1, protein	1.2863	0.0313	0.0094	0.7136	No	1.0678	0.0202	0.0012	0.0025	No	No
92795_at	Mtap4	microtubule-associated protein 4	0.9232	0.3828	0.3531	0.7961	No	0.8856	0.1	0.345	0.244	No	No
92796_at	Akp2	alkaline phosphatase 2, liver	0.8926	0.0573	0.0015	0.1936	No	0.8757	0.0348	0.0007	0.217	No	No
92797_at	Cul3	cullin 3	0.9205	0.1382	0.6607	0.3036	No	0.9224	0.122	0.482	0.299	No	No
92798_at	Atp5c1	ATP synthase, H+ transporting, mitochondrial F1 complex, gamma polypeptide 1	1.0627	0.1537	0.8693	0.4259	No	0.9208	0.204	0.0173	0.0239	No	No
92799_g_at	Atp5c1	ATP synthase, H+ transporting, mitochondrial F1 complex, gamma polypeptide 1	1.0553	0.1891	0.2722	0.1603	No	1.0455	0.266	0.409	0.0933	No	No
92800_i_at	Atp5c1 /// D13Mgi7	ATP synthase, H+ transporting, mitochondrial F1 complex, gamma polypeptide 1 /// DNA Segment, Chr 13, Mouse Genome Informatics 7	1.0567	0.4359	0.008	0.0886	No	1.1501	0.046	0.0619	0.0152	No	No
92801_at	---	---	0.9463	0.199	0.0003	0.9269	No	1.0187	0.432	0.0003	0.149	No	No
92802_s_at	Plp1	proteolipid protein (myelin) 1	0.9756	0.4125	0.0344	0.6223	No	1.1111	0.0705	0.0399	0.0826	No	No
92803_at	Prl	prolactin	1.0145	0.8189	0.2879	0.4801	No	1.0143	0.829	0.658	0.257	No	No
92804_at	Polr2h	polymerase (RNA) II (DNA directed) polypeptide H	0.9963	0.9137	0.0474	0.081	No	0.9866	0.746	0.515	0.018	No	No
92805_s_at	Arl4a	ADP-ribosylation factor-like 4A	1.0016	0.9591	0.3026	0.1018	No	0.9134	0.0294	0.0013	0.0026	No	No
92806_at	Pdrg1	p53 and DNA damage regulated 1	0.969	0.4061	0.0035	0.0509	No	1.0064	0.67	0.0029	0.0876	No	No
92807_at	Txn1	thioredoxin 1	1.0308	0.445	0.0216	0.6509	No	0.9773	0.528	0.593	0.221	No	No
92808_f_at	Fkbp4	FK506 binding protein 4	1.1351	0.0503	0.5805	0.1607	No	1.06	0.0608	0.125	0.276	No	No
92809_r_at	Fkbp4	FK506 binding protein 4	1.1103	0.2177	0.081	0.541	No	1.1607	0.0473	0.175	0.78	No	No
92810_at	Pdk3	pyruvate dehydrogenase kinase, isoenzyme 3	1.193	0.0258	0.3523	0.2481	No	1.1656	0.0185	0.0315	0.0563	No	No
92811_at	Rbp2	retinol binding protein 2, cellular	1.0016	0.9765	0.0048	0.9987	No	1.0044	0.586	0.0034	0.112	No	No
92812_f_at	Defcr3 /// Defcr4 /// Defcr6 /// Defcr17 /// Defcr20 /// Defcr23 /// Defcr24 /// LOC626708 /// LOC634825	defensin related cryptdin 3 /// defensin related cryptdin 4 /// defensin related cryptdin 6 /// defensin related cryptdin 17 /// defensin related cryptdin 20 /// defensin-related cryptdin 23 /// defensin-related cryptdin 24 /// defensin related cryptdin 26 /// similar to Defensin-related sequence cryptdin peptide precursor (Cryptdin-related protein 1C) (CRS1C)	0.9885	0.8337	0.0651	0.9874	No	0.9447	0.118	0.02	0.022	No	No
92813_at	Cyp2j6	cytochrome P450, family 2, subfamily j, polypeptide 6	0.9766	0.8228	0.1011	0.1726	No	0.8946	0.545	0.169	0.377	No	No
92814_at	Cyp2j5	cytochrome P450, family 2, subfamily j, polypeptide 5	1.0267	0.4966	0.2316	0.0268	No	1.0775	0.185	0.0495	0.133	No	No
92816_r_at	Eif4a1	eukaryotic translation initiation factor 4A1	1.0729	0.0946	0.0009	0.4012	No	0.9764	0.769	0.0003	0.0895	No	No
92817_at	Imp3	IMP3, U3 small nucleolar ribonucleoprotein, homolog (yeast)	0.8809	0.0086	0.0179	0.0487	Yes	0.9183	0.115	0.164	0.0628	No	No
92818_at	Ube1c	ubiquitin-activating enzyme E1C	1.0803	0.0849	0.0088	0.4033	No	1.0506	0.389	0.941	0.659	No	No
92820_at	Usp2	ubiquitin specific peptidase 2	0.876	0.021	0.0461	0.126	No	0.9955	0.947	0.0108	0.368	No	No

92821_at	Usp2	ubiquitin specific peptidase 2	0.9072	0.1343	0.0385	0.1845	No	0.9712	0.429	0.0161	0.0737	No	No
92824_at	Nme6	expressed in non-metastatic cells 6, protein	0.95	0.1807	0.0149	0.0319	No	1.0058	0.927	0.516	0.593	No	No
92825_at	Crisp2	cysteine-rich secretory protein 2	1.0117	0.6381	0.0139	0.382	No	1.2563	0.091	0.91	0.172	No	No
92826_at	Mrps33	mitochondrial ribosomal protein S33	1.0423	0.5003	0.2079	0.4055	No	1.0244	0.709	0.3	0.214	No	No
92827_at	Nsd1	nuclear receptor-binding SET-domain protein 1	1.0419	0.4781	0.0489	0.8797	No	1.0346	0.614	0.054	0.569	No	No
92828_at	Dpm1	dolichol-phosphate (beta-D) mannosyltransferase 1	1.0836	0.2443	0.0049	0.9429	No	1.1017	0.252	0.172	0.299	No	No
92829_at	Hspe1	heat shock protein 1 (chaperonin 10)	1.1285	0.0344	0.6309	0.1092	No	1.069	0.156	0.0418	0.138	No	No
92830_s_at	Zfp36	zinc finger protein 36	0.8672	0.0121	0.0076	0.5958	No	0.945	0.0274	0.94	0.112	No	No
92831_at	Sfxn1	sideroflexin 1	1.0523	0.4534	0.0104	0.5513	No	1.0089	0.856	0.282	0.867	No	No
92832_at	Socs1	suppressor of cytokine signaling 1	1.0001	0.9985	0.0111	0.3653	No	1.0042	0.818	0.0027	0.363	No	No
92833_at	Hal /// LOC638196	histidine ammonia lyase /// similar to Histidine ammonia-lyase (Histidase)	0.9168	0.2338	0.3122	0.6314	No	1.1032	0.314	0.087	0.0723	No	No
92834_at	Rpl13a	ribosomal protein L13a	1.0642	0.3595	0.0394	0.2852	No	1.0372	0.629	0.0077	0.36	No	No
92835_at	Cml1	camello-like 1	0.956	0.5055	0.5425	0.781	No	1.073	0.292	0.948	0.948	No	No
92836_at	Eln	elastin	0.8848	0.0007	0	0.0157	Yes	0.8649	0.0019	0	0.0403	Yes	Yes
92837_f_at	Mug-ps1 /// Mug1 /// Mug2 /// LOC434083 /// LOC640530 /// LOC669343	murinoglobulin, pseudogene 1 /// murinoglobulin 1 /// murinoglobulin 2 /// similar to murinoglobulin 1 /// similar to murinoglobulin 1 /// similar to murinoglobulin 1	0.9833	0.5589	0.6552	0.5707	No	0.8939	0.0789	0.21	0.328	No	No
92838_at	Fscn1	fascin homolog 1, actin bundling protein (Strongylocentrotus purpuratus)	0.9756	0.5567	0.0517	0.1568	No	0.9877	0.598	0.865	0.0483	No	No
92839_f_at	Snrpb2	U2 small nuclear ribonucleoprotein B	1.06	0.294	0.1655	0.7071	No	1.126	0.221	0.181	0.393	No	No
92840_at	Nup54	nucleoporin 54	0.9551	0.1749	0.1114	0.2589	No	0.9607	0.169	0.222	0.242	No	No
92841_f_at	Chgb	chromogranin B	1.0478	0.6994	0.0064	0.5637	No	1.2513	0.0403	0.0004	0.196	No	No
92842_r_at	---	---	1.0105	0.7885	0.1511	0.1669	No	1.4272	0.0253	0.0025	0.142	No	No
92843_r_at	Eif4g1	Eukaryotic translation initiation factor 4, gamma 1	1.0808	0.3016	0.0153	0.1987	No	1.0513	0.622	0.0114	0.0081	No	No
92845_at	---	---	0.9419	0.2557	0.0027	0.1321	No	0.9938	0.437	0.0001	0.0394	No	No
92846_at	M6pr	mannose-6-phosphate receptor, cation dependent	1.0825	0.3505	0.1099	0.284	No	1.1082	0.048	0.0021	0.0167	No	No
92847_s_at	M6pr /// M6pr-ps	mannose-6-phosphate receptor, cation dependent /// mannose-6-phosphate receptor, pseudogene	1.0283	0.7012	0.1392	0.3606	No	1.109	0.316	0.0253	0.915	No	No
92848_at	Oat	ornithine aminotransferase	1.0485	0.0972	0.59	0.7852	No	1.0719	0.118	0.0688	0.579	No	No
92849_at	Ccl6	chemokine (C-C motif) ligand 6	1.0502	0.458	0.006	0.0785	No	0.9939	0.776	0.127	0.03	No	No
92850_at	Rrbp1	ribosome binding protein 1	1.1037	0.0804	0.8396	0.5053	No	1.1194	0.118	0.156	0.63	No	No
92851_at	Cp	ceruloplasmin	0.7645	0.0103	0.0154	0.5728	No	0.9192	0.052	0.0065	0.544	No	No
92852_at	Fn1	fibronectin 1	1.0014	0.9726	0.013	0.1426	No	1.0553	0.258	0.0432	0.664	No	No
92853_at	Hspb1	heat shock protein 1	0.9565	0.2331	0.0057	0.1146	No	0.9935	0.973	0.0003	0.207	No	No
92854_at	Rab11a	RAB11a, member RAS oncogene family	1.0482	0.1926	0.9644	0.5052	No	1.0457	0.128	0.0494	0.991	No	No
92855_at	Eif1	eukaryotic translation initiation factor 1	0.9147	0.1018	0.9332	0.0731	No	0.9546	0.366	0.923	0.0591	No	No
92856_at	Rpl22	ribosomal protein L22	1.0213	0.6128	0.2724	0.1321	No	0.9992	0.943	0.0908	0.679	No	No
92857_at	Rpl22	ribosomal protein L22	1.0191	0.6731	0.019	0.2523	No	0.9865	0.817	0.0284	0.0943	No	No
92858_at	Slpi	secretory leukocyte peptidase inhibitor	0.939	0.0873	0.1586	0.8543	No	0.9772	0.404	0.0116	0.649	No	No
92860_at	Cox8a	cytochrome c oxidase, subunit VIIIa	1.0951	0.0383	0.0557	0.0183	No	1.1018	0.0373	0.0121	0.0643	No	No
92861_i_at	Krt1-17	keratin complex 1, acidic, gene 17	0.8702	0.0602	0.0041	0.0178	No	0.925	0.337	0.0032	0.0332	No	No
92862_f_at	Krt1-17	keratin complex 1, acidic, gene 17	0.9568	0.2079	0.0004	0.3186	No	0.9276	0.198	0.0086	0.0827	No	No
92863_at	Wbp2	WW domain binding protein 2	0.9556	0.4072	0.7907	0.5221	No	1.0004	0.984	0.0656	0.777	No	No
92864_at	Mat1a	methionine adenosyltransferase I, alpha	1.0308	0.3284	0.2679	0.788	No	0.9903	0.665	0.173	0.0576	No	No
92865_at	Dnase1	deoxyribonuclease I	0.9066	0.0251	0.2201	0.0573	No	1.0213	0.713	0.971	0.215	No	No
92866_at	H2-Aa	histocompatibility 2, class II antigen A, alpha	0.9364	0.0307	0.0069	0.1086	No	0.9327	0.296	0.98	0.962	No	No
92867_at	Phc2	polyhomeotic-like 2 (Drosophila)	0.9247	0.009	0.0001	0.0239	Yes	1.0209	0.774	0.0012	0.0264	No	No
92868_at	Tpst2	protein-tyrosine sulfotransferase 2	0.9861	0.7892	0.0107	0.3399	No	0.9583	0.566	0.0067	0.713	No	No
92869_at	Hsd3b4	hydroxysteroid dehydrogenase-4, delta<5>-3-beta	1.1181	0.0261	0.0027	0.1013	No	1.0132	0.939	0.0223	0.034	No	No
92870_at	Sel1h	Sel1 (suppressor of lin-12) 1 homolog (C. elegans)	1.0489	0.3522	0.0026	0.4347	No	1.0112	0.922	0.0023	0.442	No	No
92871_at	Sel1h	Sel1 (suppressor of lin-12) 1 homolog (C. elegans)	1.0628	0.5163	0.0079	0.1181	No	0.9975	0.886	0.0593	0.0125	No	No
92872_at	Necap1	NECAP endocytosis associated 1	0.966	0.459	0.5364	0.4656	No	0.9521	0.121	0.224	0.109	No	No
92873_f_at	Prss2	protease, serine, 2	0.9761	0.6552	0.1314	0.3994	No	1.0499	0.579	0.117	0.547	No	No
92874_f_at	Cops7a	COP9 (constitutive photomorphogenic) homolog, subunit 7a (Arabidopsis thaliana)	0.993	0.8451	0.1483	0.047	No	0.9457	0.346	0.212	0.19	No	No
92875_s_at	Cops7a	COP9 (constitutive photomorphogenic) homolog, subunit 7a (Arabidopsis thaliana)	1.0398	0.3765	0.2664	0.6575	No	0.9802	0.529	0.0028	0.347	No	No
92876_at	Ndufs4	NADH dehydrogenase (ubiquinone) Fe-S protein 4	1.1487	0.0367	0.2449	0.2143	No	1.1508	0.0016	0.791	0.0032	No	No
92877_at	Tgfbi	transforming growth factor, beta induced	1.0459	0.0978	0.0018	0.7629	No	1.0313	0.354	0.144	0.0475	No	No
92878_at	Commd3	COMM domain containing 3	1.0061	0.9176	0.3074	0.9098	No	0.9574	0.518	0.145	0.452	No	No
92879_at	Ppm1g	protein phosphatase 1G (formerly 2C), magnesium-dependent, gamma isoform	1.0494	0.1356	0.0254	0.0396	No	1.0372	0.423	0.102	0.244	No	No
92880_at	Mfge8	milk fat globule-EGF factor 8 protein	0.9236	0.0439	0.0003	0.0533	No	0.9623	0.267	0.0273	0.121	No	No
92881_at	Mylpf /// LOC676912	myosin light chain, phosphorylatable, fast skeletal muscle /// similar to Myosin regulatory light chain 2, skeletal muscle isoform (G2) (DTNB) (MLC-2)	0.8844	0.0052	0.3551	0.002	No	0.7272	0.0006	0.795	0.0002	No	No
92882_at	Rab1	RAB1, member RAS oncogene family	1.0436	0.1825	0.0027	0.0437	No	1.0207	0.734	0.119	0.716	No	No
92883_at	Rab1	RAB1, member RAS oncogene family	1.0009	0.9806	0.4757	0.3993	No	0.9713	0.379	0.004	0.486	No	No
92884_at	Gsk3a	glycogen synthase kinase 3 alpha	1.0306	0.3709	0.0499	0.0406	No	0.9989	0.939	0.43	0.191	No	No
92885_at	Tnnt3	troponin T3, skeletal, fast	0.652	0.0003	0.0249	0.0003	No	0.7421	0.0005	0.0107	0.0005	No	No
92887_at	Ddah2	dimethylarginine dimethylaminohydrolase 2	1.0817	0.0912	0.042	0.5034	No	1.0804	0.0121	0.0224	0.0138	No	No
92888_s_at	Styx	phosphoserine/threonine/tyrosine interaction protein	1.0934	0.2799	0.0379	0.4095	No	1.0404	0.249	0.0007	0.29	No	No
92889_r_at	Foxd3	forkhead box D3	0.9823	0.6391	0.3105	0.6427	No	0.9372	0.422	0.0061	0.978	No	No
92890_at	Kif1a	kinesin family member 1A	0.9808	0.6421	0.5229	0.3729	No	1.0113	0.755	0.144	0.191	No	No
92891_f_at	Hoxc9	homeo box C9	1.0508	0.1608	0.0106	0.1046	No	0.7958	0.384	0.0383	0.0262	No	No
92892_at	Vmp	vesicular membrane protein p24	0.926	0.1569	0.0424	0.3003	No	1.0609	0.101	0.0018	0.0184	No	No
92893_at	Nfia	nuclear factor I/A	1.0155	0.7623	0.0108	0.4041	No	1.0304	0.814	0.0001	0.258	No	No
92894_s_at	Nfia	nuclear factor I/A	0.9642	0.5345	0.0554	0.1114	No	0.959	0.587	0.481	0.384	No	No
92895_at	Sim1	single-minded homolog 1 (Drosophila)	0.9932	0.8768	0.3097	0.061	No	1.0167	0.879	0.382	0.487	No	No
92896_s_at	Sim2	single-minded homolog 2 (Drosophila)	0.9778	0.6142	0.2783	0.8139	No	1.0352	0.413	0.177	0.543	No	No
92897_at	Acrv1	acrosomal vesicle protein 1	0.9987	0.9815	0.1143	0.6544	No	0.9606	0.728	0.321	0.864	No	No
92898_at	Cyp7b1	cytochrome P450, family 7, subfamily b, polypeptide 1	0.9345	0.1788	0.0944	0.3106	No	0.966	0.331	0.357	0.295	No	No
92899_at	Gad2	glutamic acid decarboxylase 2	1.0334	0.5801	0.8487	0.1365	No	0.7485	0.0493	0.0002	0.344	No	No
92900_at	---	---	0.9378	0.1957	0.1533	0.2705	No	0.9768	0.184	0.352	0.005	No	No
92901_at	Rara	retinoic acid receptor, alpha	1.0454	0.5243	0.0853	0.3688	No	1.1206	0.0929	0.353	0.242	No	No
92902_at	Mybl1	myeloblastosis oncogene-like 1	0.9999	0.9985	0.2112	0.715	No	1.1967	0.1	0.794	0.333	No	No
92903_at	Tcfap2b	transcription factor AP-2 beta	1.1232	0.138	0.9365	0.2105	No	1.0668	0.129	0.623	0.14	No	No
92904_at	Prdm1	PR domain containing 1, with ZNF domain	1.0078	0.9008	0.8589	0.4512	No	1.1103	0.0916	0.165	0.373	No	No
92905_at	Epha7	Eph receptor A7	1.0193	0.6958	0.0433	0.7806	No	0.996	0.954	0.513	0.717	No	No
92906_at	Epha7	Eph receptor A7	0.9905	0.8604	0.0039	0.918	No	0.9605	0.613	0.0274	0.956	No	No
92907_at	Ocln	occludin	0.9805	0.5485	0.0106	0.1046	No	0.9784	0.582	0.0041	0.97	No	No
92908_at	Hivep1	human immunodeficiency virus type I enhancer binding protein 1	0.9729	0.6537	0.0125	0.0377	No	0.9609	0.6	0.0061	0.289	No	No
92909_at	Pgf	placental growth factor	0.9338	0.348	0.0413	0.1263	No	0.9585	0.548	0.0853	0.48	No	No
92910_at	Arnt2	aryl hydrocarbon receptor nuclear translocator 2	0.9879	0.8518	0.0654	0.7371	No	1.0612	0.169	0.0058	0.751	No	No
92911_at	Ccna1	cyclin A1	0.9453	0.2128	0.2266	0.4784	No	1.1407	0.102	0.989	0.011	No	No
92912_at	Msx3	homeo box, msh-like 3	1.0338	0.5999	0.4475	0.7941	No	0.9696	0.676	0.94	0.971	No	No
92913_at	Abcd2	ATP-binding cassette, sub-family D (ALD), member 2	0.8339	0.0047	0.0048	0.0258	Yes	1.0501	0.431	0.542	0.377	No	No
92914_at	---	---	0.9726	0.6824	0.0901	0.2591	No	1.0568	0.39	0.0652	0.591	No	No
92915_s_at	Hoxb8	homeo box B8	1.0506	0.2909	0.0043	0.0083	No	1.0395	0.158	0.0019	0.0121	No	No
92916_at	Syt3	synaptotagmin III	1.0074	0.9254	0.2799	0.6555	No	1.008	0.935	0.217	0.402	No	No
92917_at	Mmp7	matrix metallopeptidase 7	1.003	0.9436	0.1384	0.6674	No	1.06	0.38	0.0284	0.745	No	No
92918_at	F7	coagulation factor VII	0.9964	0.9428	0.4296	0.1688	No	1.0271	0.51	0.173	0.0583	No	No
92919_at	Htr3a	5-hydroxytryptamine (serotonin) receptor 3A	1.018	0.7007	0.1458	0.8165	No	0.9959	0.985	0.1	0.682	No	No
92920_at	Pitx1	paired-like homeodomain transcription factor 1	0.9826	0.7668	0.1083	0.4563	No	0.9007	0.124	0.215	0.226	No	No
92921_at	Col10a1	procollagen, type X, alpha 1	0.9679	0.4785	0.7966	0.3264	No	1.0257	0.595	0.152	0.0641	No	No
92922_at	Tcfap2a	transcription factor AP-2, alpha	1.0204	0.1917	0.0005	0.0003	No	1.0266	0.199	0.0007	0.008	No	No
92923_g_at	Tcfap2a	transcription factor AP-2, alpha	1.1095	0.0961	0.4905	0.0429	No	1.1211	0.246	0.506	0.671	No	No
92924_at	Ctsg	cathepsin G	0.9061	0.0581	0.248	0.4928	No	0.8136	0.0753	0.0381	0.348	No	No
92925_at	Cebpb	CCAAT/enhancer binding protein (C/EBP), beta	0.8643	0.021	0.0159	0.8938	No	0.8769	0.042	0.0188	0.777	No	No
92926_at	Mpl	myeloproliferative leukemia virus oncogene	1.0973	0.0731	0.0169	0.4678	No	1.0262	0.258	0.0002	0.647	No	No
92927_at	Etv1	ets variant gene 1	1.0119	0.776	0.0016	0.4165	No	1.0024	0.888	0.0134	0.527	No	No
92929_at	Cyct	cytochrome c, testis	0.9221	0.2689	0.2202	0.8665	No	0.9842	0.711	0.0322	0.903	No	No
92930_at	Dlx5	distal-less homeobox 5	0.9576	0.5024	0.9039	0.218	No	0.9485	0.0822	0.0133	0.597	No	No
92931_at	Dll1	delta-like 1 (Drosophila)	1.0228	0.0841	0.2035	0.2606	No	0.9783	0.662	0.118	0.3	No	No

92932_at	Cbln1	cerebellin 1 precursor protein	1.0704	0.1012	0.7345	0.9053	No	1.013	0.78	0.535	0.569	No	No
92933_at	Pou2f3	POU domain, class 2, transcription factor 3	1.0194	0.4946	0.2017	0.0145	No	1.0557	0.359	0.778	0.28	No	No
92934_at	Zfp90	zinc finger protein 90	0.9479	0.109	0.0015	0.023	No	0.9982	0.973	0.385	0.858	No	No
92935_at	Runx1t1	runt-related transcription factor 1; translocated to, 1 (cyclin D-related)	1.0165	0.7023	0.2221	0.4789	No	1.0397	0.39	0.315	0.612	No	No
92936_at	Cntn1	contactin 1	1.155	0.1857	0.0002	0.8489	No	0.9371	0.101	0.0002	0.0589	No	No
92937_at	Fgfr4	fibroblast growth factor receptor 4	1.0502	0.1485	0.1173	0.4537	No	1.0074	0.79	0.252	0.699	No	No
92938_at	Gabra1	gamma-aminobutyric acid (GABA-A) receptor, subunit alpha 1	1.0097	0.829	0.0221	0.3427	No	0.9518	0.739	0.0018	0.197	No	No
92939_at	Gabra6	gamma-aminobutyric acid (GABA-A) receptor, subunit alpha 6	1.0436	0.2163	0.0096	0.1293	No	1.1425	0.0875	0.889	0.0741	No	No
92940_s_at	Gabra6	gamma-aminobutyric acid (GABA-A) receptor, subunit alpha 6	1.0226	0.7996	0.1203	0.2722	No	1.0359	0.332	0.0039	0.0643	No	No
92941_at	1110054M08 Rik	RIKEN cDNA 1110054M08 gene	0.9546	0.141	0.0261	0.1446	No	0.8971	0.0765	0.892	0.125	No	No
92942_at	Trim21	tripartite motif protein 21	0.9911	0.7661	0.2977	0.2291	No	1.0635	0.1	0.27	0.137	No	No
92943_at	Gria1	glutamate receptor, ionotropic, AMPA1 (alpha 1)	0.978	0.7746	0.6195	0.6652	No	0.9324	0.402	0.705	0.261	No	No
92944_at	Gria1	glutamate receptor, ionotropic, AMPA1 (alpha 1)	0.9999	0.9981	0.0756	0.954	No	1.0595	0.167	0.787	0.857	No	No
92945_at	Gria2	glutamate receptor, ionotropic, AMPA2 (alpha 2)	1.0233	0.659	0.0055	0.4518	No	1.2084	0.0537	0.0019	0.0548	No	No
92946_f_at	Gria2	glutamate receptor, ionotropic, AMPA2 (alpha 2)	1.0053	0.6489	0.0002	0.0088	No	1.0389	0.485	0.0019	0.142	No	No
92947_s_at	Gria2	glutamate receptor, ionotropic, AMPA2 (alpha 2)	0.9953	0.9188	0.024	0.7469	No	0.9379	0.216	0.008	0.893	No	No
92948_at	Csf2	colony stimulating factor 2 (granulocyte-macrophage)	0.9445	0.2548	0.0787	0.6869	No	0.8488	0.212	0.12	0.532	No	No
92949_at	Pacsin1	protein kinase C and casein kinase substrate in neurons 1	1.0294	0.5467	0.1052	0.3777	No	0.9282	0.284	0.0197	0.209	No	No
92950_at	Slc29a2	solute carrier family 29 (nucleoside transporters), member 2	1.0117	0.7946	0.0718	0.4257	No	0.9501	0.237	0.25	0.584	No	No
92951_at	E230017H14 Rik	RIKEN cDNA E230017H14 gene	1.0057	0.9443	0.1035	0.1659	No	0.9209	0.0904	0.0828	0.665	No	No
92952_f_at	Napb	N-ethylmaleimide sensitive fusion protein attachment protein beta	0.9868	0.6673	0.0031	0.0943	No	1.0611	0.225	0.0094	0.106	No	No
92953_at	Fmn1	formin 1	1.0049	0.8998	0.6142	0.3519	No	1.0307	0.729	0.555	0.0954	No	No
92955_at	Il3ra	interleukin 3 receptor, alpha chain	0.9421	0.0962	0.01	0.3361	No	0.9284	0.137	0.0078	0.266	No	No
92956_at	Notch3	Notch gene homolog 3 (Drosophila)	0.9903	0.8016	0.2415	0.4223	No	0.9458	0.0053	0.0001	0.0256	Yes	No
92957_at	---	---	1.0918	0.2383	0.2828	0.4886	No	1.1094	0.132	0.0238	0.0499	No	No
92958_at	Foxo3a	forkhead box O3a	0.7431	0.0035	0.2423	0.6859	Yes	0.7449	0.0102	0.749	0.779	No	Yes
92959_at	Frk	fyn-related kinase	0.9677	0.4922	0.0023	0.2379	No	1.019	0.563	0.0106	0.0827	No	No
92960_at	Lif	leukemia inhibitory factor	1.0131	0.7603	0.115	0.6217	No	1.025	0.762	0.0184	0.43	No	No
92961_at	Lhx1	LIM homeobox protein 1	0.939	0.1363	0.0139	0.3861	No	0.9691	0.356	0.0733	0.622	No	No
92962_at	Cd40	CD40 antigen	0.923	0.3017	0.0239	0.6709	No	1.0083	0.631	0.0005	0.217	No	No
92963_at	Wdr81	WD repeat domain 81	0.9509	0.3123	0.0148	0.6691	No	1.0061	0.959	0.871	0.0638	No	No
92965_at	Chrne	cholinergic receptor, nicotinic, epsilon polypeptide	0.932	0.1719	0.9673	0.8178	No	1.0199	0.714	0.86	0.853	No	No
92966_g_at	Chrne	cholinergic receptor, nicotinic, epsilon polypeptide	0.9524	0.098	0.9315	0.3494	No	1.014	0.846	0.14	0.922	No	No
92967_r_at	Chrne	cholinergic receptor, nicotinic, epsilon polypeptide	0.9849	0.6123	0.3921	0.6002	No	0.9861	0.739	0.668	0.801	No	No
92968_at	Arf5	ADP-ribosylation factor 5	0.9741	0.6712	0.0102	0.2606	No	0.9694	0.678	0.055	0.568	No	No
92969_at	Ddr2	discoidin domain receptor family, member 2	1.1067	0.0357	0.0004	0.6817	No	1.222	0.0118	0.0201	0.0807	No	No
92970_at	Hoxa10	homeo box A10	1.0368	0.4749	0.1507	0.8718	No	0.9813	0.925	0.0149	0.931	No	No
92971_at	---	---	1.0277	0.5777	0.1014	0.2735	No	1.0462	0.527	0.135	0.955	No	No
92972_at	Vpreb1	pre-B lymphocyte gene 1	1.0093	0.8193	0.4205	0.2359	No	0.9107	0.157	0.0052	0.556	No	No
92974_at	Zfp37	zinc finger protein 37	0.9583	0.5648	0.0097	0.9997	No	1.053	0.436	0.0006	0.486	No	No
92975_at	Sh3bp2	SH3-domain binding protein 2	1.0437	0.3548	0.0293	0.8425	No	1.0332	0.635	0.0361	0.389	No	No
92976_at	Ndph	Norrie disease homolog	1.0109	0.8	0.0486	0.5846	No	0.9586	0.391	0.0036	0.464	No	No
92977_s_at	Hmx3	H6 homeo box 3	1.0123	0.5517	0.0004	0.0113	No	0.9646	0.296	0.0043	0.141	No	No
92978_s_at	Serpinb2	serine (or cysteine) peptidase inhibitor, clade B, member 2	1.0899	0.301	0.0543	0.0781	No	1.1597	0.0047	0.0003	0.0027	No	No
92979_at	Etv4	ets variant gene 4 (E1A enhancer binding protein, E1AF)	1.0841	0.3742	0.0343	0.5161	No	1.0029	0.933	0.0006	0.394	No	No
92980_at	---	---	1.0357	0.4666	0.0115	0.1024	No	1.0701	0.456	0.0205	0.561	No	No
92981_at	Scg2	secretogranin II	1.228	0.0114	0.0003	0.0189	No	1.3279	0.0052	0.0001	0.0397	Yes	Yes
92982_at	Bmp8a	bone morphogenetic protein 8a	0.9433	0.2857	0.0078	0.4038	No	0.9015	0.0412	0.0293	0.043	No	No
92983_at	Ptprj /// LOC627525 /// LOC668629	protein tyrosine phosphatase, receptor type, J /// similar to protein tyrosine phosphatase, receptor type, J /// similar to protein tyrosine phosphatase, receptor type, J	1.0329	0.5467	0.7232	0.2416	No	1.081	0.0204	0.0143	0.0019	No	No
92984_g_at	Ptprj	protein tyrosine phosphatase, receptor type, J	0.9324	0.5695	0.6852	0.6898	No	0.992	0.84	0.346	0.904	No	No
92985_at	Ptprj	protein tyrosine phosphatase, receptor type, J	0.985	0.7985	0.157	0.7039	No	0.9162	0.259	0.15	0.999	No	No
92986_g_at	Ptprj /// AW125753	protein tyrosine phosphatase, receptor type, J /// expressed sequence AW125753	1.0233	0.8055	0.3352	0.8942	No	1.0467	0.353	0.215	0.177	No	No
92987_at	Slc4a3	solute carrier family 4 (anion exchanger), member 3	0.9611	0.2499	0.0157	0.0785	No	0.9843	0.356	0.0064	0.0789	No	No
92988_i_at	Cadps	Ca<2+>dependent activator protein for secretion	1.2142	0.2436	0.0046	0.2557	No	1.1163	0.257	0.0069	0.431	No	No
92989_f_at	Cadps	Ca<2+>dependent activator protein for secretion	1.0307	0.5324	0.0001	0.8645	No	1.0176	0.447	0.0004	0.253	No	No
92990_at	Zfp93	zinc finger protein 93	1.0231	0.6192	0.023	0.4775	No	1.0379	0.301	0.342	0.0181	No	No
92991_at	Sp4	trans-acting transcription factor 4	0.9887	0.8292	0.8429	0.2567	No	0.969	0.692	0.817	0.884	No	No
92992_i_at	Sp4	trans-acting transcription factor 4	1.0016	0.9765	0.0047	0.0825	No	1.4042	0.191	0.0011	0.0891	No	No
92993_r_at	Sp4	trans-acting transcription factor 4	1.0822	0.1721	0.0011	0.477	No	1.3784	0.0239	0.0004	0.383	No	No
92994_at	Lalba	lactalbumin, alpha	0.9895	0.8698	0.1538	0.7129	No	0.9458	0.288	0.0196	0.177	No	No
92995_at	Vsnl1	visinin-like 1	1.0215	0.8022	0.0202	0.9437	No	1.0155	0.711	0.0026	0.449	No	No
92996_at	Sox17	SRY-box containing gene 17	0.8741	0.0657	0.8775	0.0293	No	0.9268	0.194	0.184	0.0174	No	No
92997_g_at	Sox17	SRY-box containing gene 17	0.9832	0.7626	0.0805	0.2431	No	0.9812	0.863	0.0978	0.195	No	No
92998_at	Vav2	Vav2 oncogene	1.0353	0.5642	0.1643	0.3269	No	1.1286	0.127	0.0146	0.367	No	No
92999_at	Six4	sine oculis-related homeobox 4 homolog (Drosophila)	0.9539	0.501	0.2983	0.937	No	0.9713	0.701	0.049	0.885	No	No
93000_g_at	Six4	sine oculis-related homeobox 4 homolog (Drosophila)	1.0501	0.4372	0.0248	0.5146	No	1.0591	0.144	0.0076	0.394	No	No
93001_at	Six4	sine oculis-related homeobox 4 homolog (Drosophila)	0.9872	0.874	0.0058	0.7253	No	1.0569	0.149	0.0103	0.496	No	No
93002_r_at	Tdgf1	teratocarcinoma-derived growth factor	1.0114	0.8031	0.0012	0.6806	No	0.9669	0.734	0	0.729	No	No
93004_r_at	BC018242	cDNA sequence BC018242	1.0914	0.018	0.3357	0.0067	No	1.1312	0.0048	0.0215	0.0283	Yes	No
93005_at	Syt1	synaptotagmin I	1.0656	0.4957	0.0019	0.4121	No	1.0514	0.309	0.0011	0.2	No	No
93006_at	Nfic	nuclear factor I/C	1.0151	0.8274	0.4407	0.7405	No	1.0509	0.135	0.581	0.0532	No	No
93007_at	Npy1r	neuropeptide Y receptor Y1	1.0089	0.9095	0.8804	0.8615	No	0.8972	0.0517	0.0063	0.0477	No	No
93008_at	Lsm4	LSM4 homolog, U6 small nuclear RNA associated (S. cerevisiae)	1.1091	0.0311	0.9672	0.1421	No	1.0304	0.117	0.706	0.328	No	No
93009_at	Gstm2	glutathione S-transferase, mu 2	1.0111	0.78	0.0496	0.304	No	0.9366	0.153	0.909	0.8	No	No
93010_at	Pqbp1	polyglutamine binding protein 1	0.961	0.3309	0.0005	0.0498	No	0.9248	0.418	0.0098	0.232	No	No
93011_at	Gabarapl1	gamma-aminobutyric acid (GABA(A)) receptor-associated protein-like 1	0.9515	0.4414	0.2083	0.4869	No	0.9253	0.0788	0.0022	0.942	No	No
93012_at	Tsn	translin	1.105	0.0376	0.0684	0.081	No	1.0975	0.0105	0.0016	0.117	No	No
93013_at	Id2	inhibitor of DNA binding 2	0.9565	0.5915	0.531	0.4994	No	0.9637	0.574	0.582	0.263	No	No
93014_at	Atp5l	ATP synthase, H+ transporting, mitochondrial F0 complex, subunit g	0.9765	0.7415	0.0637	0.6074	No	0.9565	0.491	0.0101	0.245	No	No
93015_at	Gsta3	glutathione S-transferase, alpha 3	1.0041	0.8974	0.5345	0.5639	No	0.9893	0.83	0.827	0.7	No	No
93016_at	Ywhaq /// LOC546165 /// LOC636369	tyrosine 3-monooxygenase/tryptophan 5-monooxygenase activation protein, theta polypeptide /// similar to 14-3-3 protein theta (14-3-3 protein tau) /// similar to 14-3-3 protein theta (14-3-3 protein tau)	1.0529	0.4939	0.0089	0.8992	No	0.9639	0.374	0.0003	0.465	No	No
93017_at	---	---	0.9641	0.676	0.0301	0.3965	No	0.9702	0.669	0.0415	0.387	No	No
93018_at	Ifitm2 /// LOC631287	interferon induced transmembrane protein 2 /// similar to interferon induced transmembrane protein 2	1.0754	0.0723	0.0011	0.0291	No	1.2052	0.0991	0.536	0.124	No	No
93019_at	H2afx	H2A histone family, member X	1.0466	0.2534	0.7363	0.6848	No	1.0433	0.593	0.1	0.779	No	No
93020_at	Bex1	brain expressed gene 1	0.9913	0.9126	0.1461	0.3707	No	1.0372	0.728	0.444	0.245	No	No
93021_at	Bex4	brain expressed gene 4	1.0175	0.5671	0.002	0.6937	No	1.0981	0.17	0.0475	0.515	No	No
93022_i_at	Hist1h3f	histone 1, H3f	1.2123	0.1194	0.3646	0.0523	No	1.1402	0.19	0.439	0.217	No	No

93023_f_at	Hist1h3g /// Hist1h3f /// Hist1h3c /// Hist1h3d /// Hist1h3b /// Hist1h3e /// Hist1h3h /// Hist1h3i /// Hist1h3a	histone 1, H3g /// histone 1, H3f /// histone1, H3c /// histone1, H3d /// histone 1, H3b /// histone 1, H3e /// histone 1, H3h /// histone 1, H3i /// histone 1, H3a	1.0043	0.8483	0.0017	0.9578	No	1.0514	0.0567	0.0005	0.0542	No	No
93024_at	Hist1h3g	histone 1, H3g	1.0271	0.5511	0.2981	0.0949	No	0.9391	0.0643	0.0126	0.0023	No	No
93025_at	Ndfip1	Nedd4 family interacting protein 1	1.0476	0.3647	0.0225	0.554	No	0.9978	0.999	0.39	0.319	No	No
93026_at	Mgst1	microsomal glutathione S-transferase 1	1.021	0.8392	0.0374	0.3058	No	1.017	0.983	0.025	0.21	No	No
93027_r_at	Rps13	Ribosomal protein S13	1.008	0.8965	0.0131	0.211	No	1.0138	0.583	0.0352	0.0051	No	No
93028_at	H19	H19 fetal liver mRNA	0.9786	0.3863	0.43	0.0666	No	0.9604	0.0221	0.111	0.154	No	No
93029_at	Idh3g	isocitrate dehydrogenase 3 (NAD+), gamma	1.0551	0.4359	0.1234	0.8254	No	1.0393	0.55	0.247	0.415	No	No
93030_at	Rps27a	ribosomal protein S27a	1.0171	0.7093	0.1353	0.5737	No	0.9704	0.261	0.315	0.39	No	No
93033_at	Ube2e3	ubiquitin-conjugating enzyme E2E 3, UBC4/5 homolog (yeast)	1.05	0.0407	0.1019	0.0136	No	1.0327	0.373	0.0043	0.0146	No	No
93037_i_at	Anxa1	annexin A1	0.9756	0.8182	0.1176	0.7917	No	0.944	0.81	0.0229	0.221	No	No
93038_f_at	Anxa1	annexin A1	0.9193	0.0849	0.893	0.0312	No	0.8843	0.125	0.158	0.0185	No	No
93039_at	Pgcp	plasma glutamate carboxypeptidase	1.0598	0.3143	0.0526	0.0815	No	1.0477	0.254	0.0018	0.87	No	No
93040_at	Fxyd1	FXYD domain-containing ion transport regulator 1	0.8981	0.0295	0.0012	0.0939	No	0.9803	0.464	0.0063	0.121	No	No
93041_at	Mcm4	minichromosome maintenance deficient 4 homolog (S. cerevisiae)	1.0413	0.3897	0.486	0.2707	No	1.0648	0.0225	0.0473	0.0341	No	No
93042_at	Bzrp	benzodiazepine receptor, peripheral	0.9866	0.5573	0.0114	0.0746	No	0.9991	0.985	0.0188	0.42	No	No
93043_at	Nptn	neuroplastin	1.042	0.5241	0.2001	0.7617	No	0.9723	0.634	0.562	0.329	No	No
93045_at	Abcd3	ATP-binding cassette, sub-family D (ALD), member 3	0.9686	0.6295	0.5117	0.7042	No	0.9698	0.604	0.173	0.908	No	No
93046_at	Nup50	nucleoporin 50	0.9945	0.916	0.0011	0.0905	No	0.9767	0.518	0.0006	0.996	No	No
93047_at	Nup50	nucleoporin 50	1.034	0.3418	0.0398	0.0632	No	1.0781	0.0024	0.0001	0.0003	No	No
93048_at	Clpp	caseinolytic peptidase, ATP-dependent, proteolytic subunit homolog (E. coli)	0.9996	0.9902	0.0032	0.0089	No	1.0744	0.0843	0.0007	0.453	No	No
93050_at	Myl2	myosin, light polypeptide 2, regulatory, cardiac, slow	0.812	0.0073	0.0335	0.0007	No	0.8194	0.0003	0.0029	0	No	No
93051_at	Ephx2	epoxide hydrolase 2, cytoplasmic	0.9203	0.0963	0.0745	0.0137	No	0.9441	0.255	0.0254	0.0888	No	No
93053_at	Casq2	calsequestrin 2	0.9611	0.3601	0.0051	0.682	No	1.034	0.712	0.0049	0.685	No	No
93054_at	Ankrd46	ankyrin repeat domain 46	0.9921	0.85	0.1752	0.6643	No	0.9653	0.454	0.371	0.419	No	No
93055_at	Ankrd46	ankyrin repeat domain 46	1.017	0.6982	0.146	0.0279	No	0.9795	0.701	0.238	0.75	No	No
93056_g_at	Ankrd46	ankyrin repeat domain 46	0.9669	0.3023	0.0716	0.3345	No	0.9548	0.3	0.0188	0.576	No	No
93057_at	Btf3	basic transcription factor 3	1.0293	0.6911	0.1059	0.7257	No	1.0122	0.892	0.0092	0.53	No	No
93058_at	Eif1a	eukaryotic translation initiation factor 1A	1.0022	0.9814	0.1453	0.8718	No	0.9552	0.631	0.442	0.825	No	No
93059_at	2610204K14 Rik	RIKEN cDNA 2610204K14 gene	1.0115	0.8242	0.2947	0.899	No	0.9804	0.755	0.731	0.765	No	No
93061_at	Itga7	integrin alpha 7	0.9001	0.0473	0.9223	0.3409	No	0.9669	0.405	0.0005	0.261	No	No
93062_at	Mrpl39	mitochondrial ribosomal protein L39	0.9596	0.5677	0.2907	0.7477	No	1.0338	0.5	0.266	0.141	No	No
93063_at	App	amyloid beta (A4) precursor protein	1.0203	0.4828	0.0019	0.3877	No	0.9776	0.433	0.0057	0.848	No	No
93064_at	Bnip2	BCL2/adenovirus E1B interacting protein 1, NIP2	0.8572	0.0404	0.0957	0.1653	No	0.9525	0.299	0.754	0.628	No	No
93065_at	Il11ra1	interleukin 11 receptor, alpha chain 1	1.0439	0.3722	0.5566	0.6047	No	1.0361	0.464	0.991	0.195	No	No
93066_at	Grn	granulin	0.9361	0.1687	0.0288	0.9526	No	0.9489	0.305	0.0372	0.534	No	No
93067_f_at	Hist2h3c1 /// Hist2h2aa1 /// Hist2h2aa2 /// LOC667728 /// LOC670497 /// LOC677006	histone 2, H3c1 /// histone 2, H2aa1 /// histone 2, H2aa2 /// similar to H2A histone family, member O /// similar to H2A histone family, member O /// similar to H2A histone family, member O	1.0055	0.8862	0.0791	0.0446	No	1.0632	0.0947	0.0018	0.821	No	No
93068_r_at	Hist2h3c1	histone 2, H3c1	0.9648	0.3323	0.006	0.1435	No	1.0056	0.915	0.964	0.721	No	No
93069_at	Ube2d2	ubiquitin-conjugating enzyme E2D 2	1.1041	0.1029	0.01	0.064	No	1.0664	0.0525	0.699	0.0226	No	No
93070_at	Ranbp5	RAN binding protein 5	1.1352	0.2032	0.0062	0.3299	No	1.0901	0.292	0.0095	0.857	No	No
93071_at	Trim28	tripartite motif protein 28	1.0537	0.1477	0.002	0.4553	No	1.0477	0.235	0.0011	0.457	No	No
93072_at	Cnp1	cyclic nucleotide phosphodiesterase 1	1.0243	0.7009	0.0399	0.882	No	1.0376	0.36	0.0036	0.339	No	No
93073_at	Nfatc2	nuclear factor of activated T-cells, cytoplasmic, calcineurin-dependent 2	1.0273	0.6744	0.5198	0.7322	No	1.006	0.858	0.182	0.193	No	No
93074_g_at	Nfatc2	nuclear factor of activated T-cells, cytoplasmic, calcineurin-dependent 2	1.006	0.8647	0.0873	0.4942	No	0.9712	0.402	0.0491	0.773	No	No
93075_r_at	Nfatc2	nuclear factor of activated T-cells, cytoplasmic, calcineurin-dependent 2	1.0975	0.2385	0.546	0.0441	No	1.1873	0.289	0.636	0.0759	No	No
93076_at	Csnk1a1	casein kinase 1, alpha 1	0.9688	0.3247	0.0067	0.3914	No	0.9562	0.468	0.0502	0.967	No	No
93077_s_at	Ly6c	lymphocyte antigen 6 complex, locus C	1.0047	0.9168	0.5084	0.1991	No	1.0452	0.386	0.146	0.284	No	No
93078_at	Ly6a	lymphocyte antigen 6 complex, locus A	0.8705	0.0033	0.0187	0.0048	No	1.0514	0.889	0.0113	0.101	No	No
93080_at	Bscl2	Bernardinelli-Seip congenital lipodystrophy 2 homolog (human)	0.9878	0.5794	0.3089	0.4775	No	0.9548	0.453	0.767	0.466	No	No
93081_at	Rbbp7	retinoblastoma binding protein 7	1.1003	0.1142	0.0021	0.8335	No	1.1015	0.0757	0.0066	0.991	No	No
93082_at	Za20d3	zinc finger, A20 domain containing 3	1.0376	0.511	0.0058	0.1148	No	1.0312	0.596	0.0219	0.288	No	No
93083_at	Anxa5	annexin A5	1.0314	0.3543	0.128	0.8033	No	1.012	0.601	0.433	0.196	No	No
93084_at	Slc25a4	solute carrier family 25 (mitochondrial carrier, adenine nucleotide translocator), member 4	1.0615	0.1985	0.2318	0.9023	No	1.0311	0.45	0.794	0.751	No	No
93085_at	Psmb9	proteosome (prosome, macropain) subunit, beta type 9 (large multifunctional peptidase 2)	0.972	0.4759	0.0029	0.5168	No	0.9171	0.0838	0.002	0.21	No	No
93086_at	Igk-V28 /// Igk-V8-16	immunoglobulin kappa chain variable 28 (V28) /// immunoglobulin kappa chain variable 8 (V8)-16	0.9451	0.1292	0.0025	0.8529	No	0.9827	0.623	0.0035	0.583	No	No
93087_r_at	Usp25	ubiquitin specific peptidase 25	0.9983	0.9757	0.2458	0.9211	No	1.0408	0.398	0.225	0.131	No	No
93088_at	B2m	beta-2 microglobulin	1.1131	0.0808	0.0579	0.636	No	1.0341	0.463	0.971	0.285	No	No
93089_at	Eif4a2	eukaryotic translation initiation factor 4A2	1.0044	0.9473	0.0939	0.0278	No	0.981	0.808	0.262	0.427	No	No
93090_at	Fgfr2	fibroblast growth factor receptor 2	0.9995	0.9866	0.1014	0.7073	No	0.9839	0.545	0.0868	0.859	No	No
93091_s_at	Fgfr2	fibroblast growth factor receptor 2	0.9025	0.19	0.0371	0.7969	No	0.9791	0.668	0.806	0.358	No	No
93092_at	H2-DMa	histocompatibility 2, class II, locus DMa	1.0633	0.0623	0.0546	0.3996	No	1.0367	0.535	0.0062	0.741	No	No
93093_at	Mcl1	myeloid cell leukemia sequence 1	0.8506	0.0912	0.6215	0.9783	No	0.8832	0.113	0.955	0.187	No	No
93094_at	Cdr2	cerebellar degeneration-related 2	1.0567	0.4495	0.0228	0.4144	No	0.9727	0.686	0.943	0.813	No	No
93095_at	Hmgb1 /// LOC667777 /// LOC669692	high mobility group box 1 /// similar to High mobility group protein 1 (HMG-1) (High mobility group protein B1) (Amphoterin) (Heparin-binding protein p30) /// similar to High mobility group protein 1 (HMG-1) (High mobility group protein B1) (Amphoterin) (Heparin-binding protein p30)	1.0107	0.8637	0.71	0.8157	No	0.9471	0.182	0.993	0.042	No	No
93096_at	Fgg	fibrinogen, gamma polypeptide	0.9818	0.5936	0.0037	0.1312	No	1.1319	0.371	0.0869	0.126	No	No
93097_at	Arg1	arginase 1, liver	1.0955	0.1706	0.3835	0.0473	No	1.095	0.0565	0.0015	0.0299	No	No
93098_at	Rpl35	ribosomal protein L35	1.0229	0.8425	0.3254	0.7962	No	1.0715	0.452	0.872	0.703	No	No
93099_f_at	Plk1	polo-like kinase 1 (Drosophila)	0.9638	0.2661	0.0004	0.3537	No	0.9759	0.533	0.0004	0.389	No	No
93100_at	Acta2	actin, alpha 2, smooth muscle, aorta	0.8832	0.1059	0.4576	0.0828	No	0.8651	0.0966	0.572	0.179	No	No
93101_s_at	Nedd4	neural precursor cell expressed, developmentally down-regulted gene 4	1.055	0.339	0.0471	0.363	No	0.9947	0.875	0.165	0.861	No	No
93102_f_at	Actg2	actin, gamma 2, smooth muscle, enteric	0.9983	0.9788	0.0047	0.8931	No	1.113	0.126	0.0005	0.543	No	No
93103_at	Ldhc	lactate dehydrogenase C	0.9535	0.2251	0.0574	0.9713	No	0.9109	0.0979	0.268	0.682	No	No
93104_at	Btg1	B-cell translocation gene 1, anti-proliferative	1.122	0.0291	0.3084	0.3249	No	1.1082	0.118	0.0327	0.939	No	No
93105_s_at	Tcrb-J /// Tcrb-V13 /// LOC665500 /// LOC665506 /// LOC669844 /// LOC669847 /// LOC674047 /// LOC674048 /// LOC676782	T-cell receptor beta, joining region /// T-cell receptor beta, variable 13 /// similar to T-cell receptor beta-1 chain C region /// similar to T-cell receptor beta-2 chain C region /// similar to T-cell receptor beta-1 chain C region /// similar to T-cell receptor beta-1 chain C region /// similar to T-cell receptor beta-2 chain C region /// similar to T-cell receptor beta-1 chain C region	0.9691	0.5436	0.0843	0.221	No	0.956	0.401	0.669	0.217	No	No

93106_i_at	Tcrb-J /// LOC665500 /// LOC669844 /// LOC674047 /// LOC676782	T-cell receptor beta, joining region /// similar to T-cell receptor beta-1 chain C region /// similar to T-cell receptor beta-1 chain C region /// similar to T-cell receptor beta-1 chain C region /// similar to T-cell receptor beta-1 chain C region	0.9586	0.5062	0.4133	0.5211	No	0.7566	0.716	0.403	0.834	No	No
93107_r_at	Tcrb-J /// LOC665500 /// LOC669844 /// LOC674047 /// LOC676782	T-cell receptor beta, joining region /// similar to T-cell receptor beta-1 chain C region /// similar to T-cell receptor beta-1 chain C region /// similar to T-cell receptor beta-1 chain C region /// similar to T-cell receptor beta-1 chain C region	0.9907	0.8572	0.5881	0.9469	No	1.2093	0.153	0.114	0.18	No	No
93108_at	P4hb	prolyl 4-hydroxylase, beta polypeptide	1.1233	0.0302	0.7159	0.249	No	1.1349	0.0493	0.173	0.49	No	No
93109_f_at	Serpina1a /// Serpina1b /// Serpina1c /// Serpina1d	serine (or cysteine) peptidase inhibitor, clade A, member 1a /// serine (or cysteine) preptidase inhibitor, clade A, member 1b /// serine (or cysteine) peptidase inhibitor, clade A, member 1c /// serine (or cysteine) peptidase inhibitor, clade A, member 1d	0.9896	0.8878	0.0531	0.7946	No	1.0458	0.461	0.405	0.536	No	No
93111_at	Kpnb1	karyopherin (importin) beta 1	0.976	0.4408	0.0046	0.049	No	0.9588	0.0989	0.004	0.0625	No	No
93112_at	Mcm2	minichromosome maintenance deficient 2 mitotin (S. cerevisiae)	1.0851	0.0305	0.0344	0.2163	No	1.0395	0.0713	0.0046	0.0227	No	No
93114_at	Atp5j2	ATP synthase, H+ transporting, mitochondrial F0 complex, subunit f, isoform 2	1.0096	0.8889	0.6268	0.1998	No	1.1226	0.295	0.0067	0.226	No	No
93116_at	Prkacb	protein kinase, cAMP dependent, catalytic, beta	1.0528	0.253	0.002	0.936	No	1.03	0.393	0.0261	0.366	No	No
93117_at	Hnrpa2b1	heterogeneous nuclear ribonucleoprotein A2/B1	1.2177	0.052	0.0016	0.1357	No	1.0895	0.0785	0.0003	0.0784	No	No
93118_at	Hnrpa2b1	heterogeneous nuclear ribonucleoprotein A2/B1	1.1796	0.2107	0.0214	0.8065	No	1.1085	0.331	0.0028	0.353	No	No
93119_at	Cox5b	cytochrome c oxidase, subunit Vb	1.0075	0.8649	0.1753	0.0968	No	1.007	0.871	0.8	0.0228	No	No
93120_f_at	H2-K1	histocompatibility 2, K1, K region	1.0345	0.2581	0.0108	0.5484	No	1.0009	0.979	0.0115	0.728	No	No
93121_at	Rps24	ribosomal protein S24	1.0499	0.4897	0.6688	0.6991	No	1.0436	0.366	0.0081	0.31	No	No
93122_at	Crisp1	cysteine-rich secretory protein 1	1.117	0.0116	0.0059	0.8614	No	1.2014	0.0029	0.0018	0.934	Yes	Yes
93124_at	Pebp1	phosphatidylethanolamine binding protein 1	1.0814	0.1845	0.5932	0.264	No	1.07	0.501	0.693	0.21	No	No
93126_at	Ckb	creatine kinase, brain	1.0012	0.961	0.0037	0.1138	No	0.9856	0.581	0.009	0.0819	No	No
93127_at	LOC670155	similar to 3-phosphoglycerate dehydrogenase	1.0984	0.1027	0.1314	0.0096	No	1.107	0.0231	0.0011	0.0022	No	No
93128_at	Npy1r	neuropeptide Y receptor Y1	1.0437	0.0058	0.0001	0	No	1.0357	0.218	0.0244	0.0059	No	No
93129_at	Cutl2	cut-like 2 (Drosophila)	0.918	0.3773	0.0802	0.3129	No	0.8915	0.0555	0.277	0.637	No	No
93130_at	2600005C20 Rik	RIKEN cDNA 2600005C20 gene	0.8801	0.0195	0.9819	0.6719	No	0.9501	0.0951	0.0122	0.349	No	No
93131_at	Galc	galactosylceramidase	1.1562	0.1248	0.9956	0.2919	No	1.1703	0.0405	0.16	0.0252	No	No
93132_at	Grik1	glutamate receptor, ionotropic, kainate 1	1.0649	0.3598	0.0084	0.077	No	1.0368	0.283	0.0007	0.1	No	No
93133_at	Slc7a3	solute carrier family 7 (cationic amino acid transporter, y+ system), member 3	1.014	0.8106	0.0936	0.9495	No	0.9762	0.751	0.277	0.464	No	No
93134_at	Nptx1	neuronal pentraxin 1	0.9756	0.5494	0.2655	0.0599	No	1.0431	0.321	0.596	0.019	No	No
93136_at	Dspg3	dermatan sulphate proteoglycan 3	1.105	0.0182	0.0004	0.029	No	1.1505	0.261	0.0074	0.428	No	No
93137_at	Ntsr2	neurotensin receptor 2	1.0114	0.8573	0.8527	0.9915	No	1.0109	0.695	0.0188	0.184	No	No
93138_at	2410012H22 Rik	RIKEN cDNA 2410012H22 gene	1.0163	0.6334	0.1331	0.5912	No	1.0263	0.725	0.306	0.839	No	No
93139_at	Sdsl	serine dehydratase-like	1.0593	0.1898	0.052	0.033	No	1.1473	0.0077	0.752	0.0016	No	No
93140_at	Tsx	testis specific X-linked gene	0.9464	0.2115	0.1101	0.2053	No	0.8474	0.131	0.0724	0.13	No	No
93141_at	Nr0b1	nuclear receptor subfamily 0, group B, member 1	1.0332	0.7284	0.5295	0.6665	No	0.9866	0.814	0.373	0.391	No	No
93142_at	Bach1	BTB and CNC homology 1	1.0059	0.8929	0.0295	0.3696	No	0.9577	0.0164	0.325	0.933	No	No
93143_at	1190005I06 Rik	RIKEN cDNA 1190005I06 gene	1.0618	0.0163	0.0016	0.0008	No	1.0558	0.025	0.365	0.0039	No	No
93144_at	AI317237	expressed sequence AI317237	1.0396	0.5131	0.0058	0.9243	No	1.0405	0.213	0.0007	0.266	No	No
93145_at	Gatad2a	GATA zinc finger domain containing 2A	0.9983	0.9773	0.7905	0.4014	No	0.9431	0.26	0.742	0.079	No	No
93146_at	Cldn8	claudin 8	0.929	0.0374	0.0567	0.1651	No	0.9914	0.708	0.0056	0.187	No	No
93147_f_at	Brunol4	bruno-like 4, RNA binding protein (Drosophila)	1.0834	0.2057	0.0077	0.8291	No	1.024	0.551	0.0194	0.383	No	No
93148_at	Ttc17	tetratricopeptide repeat domain 17	0.9806	0.3735	0.0447	0.0152	No	0.9282	0.176	0.2	0.203	No	No
93151_at	Cpne6	copine VI	0.9851	0.7676	0.0856	0.2414	No	0.9892	0.853	0.683	0.659	No	No
93153_at	Adarb1	adenosine deaminase, RNA-specific, B1	0.9636	0.1889	0.1277	0.5808	No	0.9496	0.325	0.0138	0.493	No	No
93155_at	A930004K21 Rik	RIKEN cDNA A930004K21 gene	1.1456	0.0723	0.0952	0.4536	No	1.1952	0.0795	0.109	0.329	No	No
93158_at	9130206N08 Rik	RIKEN cDNA 9130206N08 gene	1.0298	0.5034	0.35	0.3884	No	0.9839	0.677	0.002	0.629	No	No
93159_at	Syt2	synaptotagmin II	1.2322	0.0062	0.0005	0.822	Yes	1.0484	0.436	0.135	0.478	No	No
93162_f_at	ORF9	open reading frame 9	0.9257	0.4636	0.0228	0.2113	No	1.0092	0.888	0.268	0.4	No	No
93163_at	Gabrg2	gamma-aminobutyric acid (GABA-A) receptor, subunit gamma 2	1.0164	0.779	0.0243	0.7583	No	0.9015	0.188	0.0098	0.904	No	No
93164_at	Rnf2	ring finger protein 2	0.9472	0.2945	0.2098	0.0686	No	0.941	0.147	0.0043	0.16	No	No
93165_at	Lysmd1	LysM, putative peptidoglycan-binding, domain containing 1	0.9029	0.2728	0.7708	0.3469	No	0.9437	0.327	0.267	0.75	No	No
93166_at	---	PREDICTED: Mus musculus hypothetical protein LOC626765 (LOC626765), mRNA	1.026	0.633	0.4556	0.0639	No	1.0968	0.278	0.0957	0.106	No	No
93167_f_at	Olr1	oxidized low density lipoprotein (lectin-like) receptor 1	1.0429	0.1879	0.0165	0.1281	No	1.1172	0.597	0.449	0.343	No	No
93168_at	AV344025	expressed sequence AV344025	0.9242	0.1267	0.944	0.7508	No	1.0359	0.699	0.423	0.693	No	No
93169_at	---	---	1.0284	0.2092	0	0.007	No	1.0112	0.644	0.0002	0.485	No	No
93173_at	Slc39a3	solute carrier family 39 (zinc transporter), member 3	0.9911	0.8095	0.0461	0.9871	No	0.9795	0.584	0.103	0.495	No	No
93174_at	Cd276	CD276 antigen	1.0682	0.0883	0.0003	0.1509	No	1.0903	0.0214	0.0001	0.104	No	No
93175_at	A130022J15 Rik	RIKEN cDNA A130022J15 gene	1.0268	0.2456	0.0166	0.0266	No	1.0499	0.11	0.0988	0.365	No	No
93177_at	---	Transcribed locus	0.9633	0.5864	0.0015	0.6847	No	0.9601	0.638	0.0445	0.762	No	No
93178_at	Ngef	neuronal guanine nucleotide exchange factor	0.9622	0.3858	0.2501	0.4797	No	1.0792	0.0881	0.0589	0.239	No	No
93179_at	Tmem62	transmembrane protein 62	1.0195	0.6925	0.7858	0.6924	No	1.0076	0.908	0.571	0.056	No	No
93180_at	AI506816	expressed sequence AI506816	0.9779	0.6939	0.0689	0.5747	No	1.0983	0.11	0.0723	0.127	No	No
93181_r_at	Upf2	UPF2 regulator of nonsense transcripts homolog (yeast)	0.98	0.7718	0.1167	0.3461	No	1.0102	0.905	0.387	0.113	No	No
93182_at	Nkx6-2	NK6 transcription factor related, locus 2 (Drosophila)	1.006	0.9445	0.6506	0.906	No	0.9433	0.418	0.98	0.279	No	No
93183_at	Cdc91l1	CDC91 cell division cycle 91-like 1 (S. cerevisiae)	1.0016	0.9676	0.0068	0.9117	No	1.0769	0.242	0.0104	0.106	No	No
93184_at	Tbl2	transducin (beta)-like 2	1.1078	0.2831	0.2259	0.7546	No	1.042	0.279	0.0121	0.121	No	No
93185_at	AU021092	expressed sequence AU021092	0.9072	0.0563	0.0088	0.5162	No	1.1091	0.212	0.0032	0.0558	No	No
93186_at	Cxxc6	CXXC finger 6	1.0166	0.7232	0.3077	0.8183	No	0.8758	0.393	0.869	0.395	No	No
93187_at	Trim47	tripartite motif protein 47	0.943	0.1903	0.0006	0.4103	No	0.9846	0.736	0.0001	0.351	No	No
93188_at	Dkk3	dickkopf homolog 3 (Xenopus laevis)	0.9998	0.99	0.091	0.9038	No	0.9898	0.72	0.0825	0.73	No	No
93190_at	BC053401	cDNA sequence BC053401	1.0699	0.0902	0.0033	0.3612	No	1.0174	0.475	0.0542	0.431	No	No
93191_at	Vamp4	vesicle-associated membrane protein 4	0.8978	0.0556	0.003	0.1699	No	0.9381	0.0399	0.0015	0.0787	No	No
93193_at	Adrb2	adrenergic receptor, beta 2	0.9036	0.0634	0.1207	0.0232	No	0.9049	0.123	0.122	0.137	No	No
93194_at	Ly9	lymphocyte antigen 9	0.9897	0.8323	0.8243	0.1536	No	1.0313	0.429	0.331	0.038	No	No
93195_at	Mfhas1	malignant fibrous histiocytoma amplified sequence 1	0.9192	0.0193	0.0023	0.0822	No	0.9259	0.277	0.837	0.0641	No	No
93196_at	Vps37a	Vacuolar protein sorting 37A (yeast)	1.0091	0.8643	0.1337	0.5183	No	0.8997	0.172	0.295	0.559	No	No
93197_at	Ly6g6c	lymphocyte antigen 6 complex, locus G6C	0.9507	0.3522	0.904	0.2348	No	0.98	0.702	0.12	0.987	No	No
93198_at	Csf3r	colony stimulating factor 3 receptor (granulocyte)	0.9714	0.5694	0.0206	0.9052	No	1.187	0.0609	0.0821	0.0938	No	No
93199_at	---	---	0.9492	0.4478	0.3955	0.98	No	0.99	0.927	0.0003	0.009	No	No
93200_f_at	D1Pas1	DNA segment, Chr 1, Pasteur Institute 1	0.9951	0.8658	0.171	0.2702	No	1.0902	0.14	0.127	0.19	No	No
93201_at	D1Pas1	DNA segment, Chr 1, Pasteur Institute 1	0.989	0.8497	0.5752	0.1108	No	1.1622	0.0203	0.789	0.0058	No	No
93202_at	Nt5e	5' nucleotidase, ecto	0.9117	0.2358	0.1265	0.704	No	0.8642	0.115	0.0013	0.0321	No	No
93203_f_at	Foxn2	forkhead box N2	0.9747	0.4069	0.0134	0.1974	No	0.9251	0.0395	0.002	0.0063	No	No
93204_r_at	Foxn2	forkhead box N2	1.0161	0.6644	0.0042	0.1366	No	1.0616	0.254	0.0204	0.236	No	No
93205_at	Ada	adenosine deaminase	0.971	0.6313	0.0062	0.0548	No	0.9608	0.565	0.0057	0.0883	No	No
93206_g_at	Ada	adenosine deaminase	0.9714	0.617	0.0045	0.6724	No	0.9495	0.431	0.0061	0.75	No	No
93207_at	Acr	preproacrosin	0.9696	0.5821	0.1268	0.9218	No	0.9565	0.348	0.0118	0.296	No	No
93208_at	Prss3 /// Prss1 /// LOC436523	protease, serine, 3 /// protease, serine, 1 (trypsin 1) /// trypsinogen 12	1.1665	0.0562	0.7498	0.0779	No	1.2302	0.0141	0.307	0.0191	No	No
93209_at	Nek4	NIMA (never in mitosis gene a)-related expressed kinase 4	0.9687	0.519	0.6445	0.411	No	0.9704	0.43	0.065	0.123	No	No

93210_g_at	Nek4	NIMA (never in mitosis gene a)-related expressed kinase 4	0.9342	0.0438	0.2318	0.0162	No	1.0027	0.832	0.256	0.0539	No	No
93211_at	Dnajc5	DnaJ (Hsp40) homolog, subfamily C, member 5	1.0359	0.1696	0.6657	0.0559	No	1.0772	0.0044	0.104	0.0017	No	No
93212_at	Ptplad1	protein tyrosine phosphatase-like A domain containing 1	0.958	0.4138	0.3279	0.6252	No	0.9829	0.772	0.866	0.834	No	No
93213_at	LOC384413	Similar to immunoglobulin light chain variable region	1.0215	0.6355	0.5607	0.2681	No	1.1805	0.199	0.0376	0.744	No	No
93214_at	Camk2d	calcium/calmodulin-dependent protein kinase II, delta	0.9943	0.8151	0.0808	0.1658	No	1.0566	0.185	0.502	0.686	No	No
93215_at	Tnfaip1	tumor necrosis factor, alpha-induced protein 1 (endothelial)	0.9978	0.9565	0.2407	0.8635	No	1.0655	0.496	0.0941	0.549	No	No
93216_at	Fgf2	fibroblast growth factor 2	0.8762	0.1023	0.0342	0.4799	No	1.0808	0.248	0.381	0.628	No	No
93217_at	Fbxo45	F-box protein 45	1.1036	0.0396	0.0348	0.3024	No	1.0791	0.0624	0.202	0.368	No	No
93218_at	Swap70	SWA-70 protein	0.8149	0.0157	0.0127	0.0605	No	0.9454	0.379	0.129	0.423	No	No
93219_at	Acp1	acid phosphatase 1, soluble	0.9527	0.295	0.7026	0.3355	No	0.9812	0.634	0.023	0.949	No	No
93220_at	Col4a5	procollagen, type IV, alpha 5	0.9967	0.9148	0.0223	0.0357	No	0.9933	0.919	0.102	0.28	No	No
93221_at	Hoxd8	homeo box D8	1.0378	0.1947	0.0012	0.0356	No	1.1819	0.124	0.0846	0.0241	No	No
93222_at	Rbbp9 /// LOC677524	retinoblastoma binding protein 9 /// similar to Retinoblastoma-binding protein 9 (RBBP-9) (B5T overexpressed gene protein) (Bog protein)	0.9665	0.5916	0.1652	0.481	No	1.0479	0.518	0.0857	0.444	No	No
93223_at	LOC236118	similar to T-cell receptor alpha chain V region CTL-F3 precursor	1.0597	0.6179	0.1406	0.4225	No	1.0403	0.521	0.958	0.157	No	No
93224_at	Tcra-V8	T-cell receptor alpha, variable 8	0.9869	0.6779	0.0057	0.7612	No	1.0215	0.66	0.168	0.836	No	No
93225_s_at	Cyp21a1 /// LOC632302	cytochrome P450, family 21, subfamily a, polypeptide 1 /// similar to Cytochrome P450 XXI (Steroid 21-hydroxylase) (21-OHase) (P450-C21) (P-450c21)	1.0245	0.6532	0.2359	0.6883	No	1.0367	0.161	0.0051	0.456	No	No
93226_i_at	Igh-1a /// LOC380809 /// LOC633728	immunoglobulin heavy chain 1a (serum IgG2a) /// anti-lipoteichoic acid heavy chain variable region /// similar to Ig heavy chain V-III region VH26 precursor	1.0391	0.6029	0.4979	0.6181	No	1.2218	0.421	0.628	0.161	No	No
93227_f_at	LOC232065 /// LOC669091 /// LOC672427 /// LOC673751	similar to Ig kappa chain V-IV region precursor /// similar to Ig kappa chain V-IV region precursor /// similar to Ig kappa chain V-IV region precursor /// similar to Ig kappa chain V-IV region precursor	0.9672	0.377	0.0173	0.7731	No	1.0009	0.965	0.671	0.052	No	No
93228_at	Hells	helicase, lymphoid specific	1.1317	0.0203	0.1004	0.7472	No	1.205	0.0166	0.0155	0.528	No	No
93230_at	Neurog1	neurogenin 1	0.9841	0.8381	0.0514	0.6527	No	1.0759	0.0688	0.489	0.108	No	No
93231_at	Hic1	hypermethylated in cancer 1	0.9678	0.4441	0.0019	0.1136	No	0.9573	0.396	0.0002	0.0208	No	No
93232_at	Tmem141	transmembrane protein 141	1.0408	0.59	0.3056	0.8158	No	1.0703	0.191	0.0719	0.877	No	No
93234_at	Msc	musculin	1.0456	0.3735	0.1002	0.8278	No	1.0518	0.284	0.0199	0.0532	No	No
93235_at	BB128963	expressed sequence BB128963	1.0412	0.2112	0.0003	0.8779	No	1.009	0.814	0.007	0.711	No	No
93236_s_at	Tyms /// Tyms-ps	thymidylate synthase /// thymidylate synthase, pseudogene	1.1443	0	0.0001	0.9734	Yes	1.1124	0.0255	0.0066	0.736	No	Yes
93237_s_at	Tyms-ps	thymidylate synthase, pseudogene	1.1011	0.1095	0.0691	0.4476	No	1.0801	0.0149	0.0006	0.0821	No	No
93238_at	Dhrsx	dehydrogenase/reductase (SDR family) X chromosome	0.9724	0.3917	0.0733	0.4779	No	0.928	0.0723	0.255	0.605	No	No
93240_f_at	Upf3b	UPF3 regulator of nonsense transcripts homolog B (yeast)	0.9513	0.3964	0.0044	0.6005	No	0.9802	0.717	0.011	0.511	No	No
93241_r_at	Upf3b	UPF3 regulator of nonsense transcripts homolog B (yeast)	0.9981	0.943	0.0054	0.4907	No	1.3909	0.0088	0.0113	0.943	Yes	No
93242_at	Prm3	protamine 3	1.165	0.024	0.3125	0.0159	No	1.1122	0.235	0.084	0.351	No	No
93243_at	Bmp7	bone morphogenetic protein 7	1.0847	0.1054	0.0027	0.8466	No	1.0798	0.203	0.0224	0.914	No	No
93244_at	5730502D15 Rik	RIKEN cDNA 5730502D15 gene	0.963	0.3401	0.0005	0.7142	No	1.001	0.976	0.0669	0.972	No	No
93245_at	Pramel7	preferentially expressed antigen in melanoma like 7	0.9453	0.5739	0.8967	0.4462	No	0.9818	0.849	0.92	0.29	No	No
93246_at	Narg1	NMDA receptor-regulated gene 1	0.9028	0.1562	0.0006	0.0741	No	0.8615	0.0005	0.001	0.0002	No	No
93248_at	Thyn1	thymocyte nuclear protein 1	1.0571	0.1191	0.3046	0.5944	No	1.066	0.267	0.577	0.321	No	No
93250_r_at	Hmgb2 /// LOC678110 /// LOC678341	high mobility group box 2 /// high mobility group box 2 /// high mobility group box 2	1.1506	0.1786	0.471	0.1658	No	1.085	0.518	0.0639	0.0651	No	No
93251_at	Nipsnap1	4-nitrophenylphosphatase domain and non-neuronal SNAP25-like protein homolog 1 (C. elegans)	1.0059	0.8512	0.0361	0.0515	No	1.0093	0.684	0.151	0.0301	No	No
93252_at	Bcap31	B-cell receptor-associated protein 31	0.964	0.2564	0.9695	0.9331	No	1.0005	0.998	0.0433	0.32	No	No
93253_at	Mapk1	mitogen activated protein kinase 1	1.0863	0.2548	0.0308	0.9723	No	1.1052	0.0579	0.0121	0.342	No	No
93254_at	Mapk1	mitogen activated protein kinase 1	0.9976	0.9569	0.0214	0.9723	No	0.9623	0.256	0.009	0.521	No	No
93255_at	Ralbp1	ralA binding protein 1	0.9455	0.1664	0.0455	0.2514	No	0.9238	0.0067	0.001	0.0141	Yes	No
93256_at	Golga7 /// LOC628040	golgi autoantigen, golgin subfamily a, 7 /// similar to golgi autoantigen, golgin subfamily a, 7	1.0916	0.4111	0.2386	0.5847	No	1.2614	0.0871	0.352	0.0763	No	No
93257_at	Ddx1	DEAD (Asp-Glu-Ala-Asp) box polypeptide 1	1.1064	0.0775	0.0948	0.6686	No	1.1098	0.132	0.0319	0.541	No	No
93258_at	Hmbs	hydroxymethylbilane synthase	1.0459	0.2643	0.4733	0.0556	No	1.0042	0.888	0.452	0.26	No	No
93259_at	Tnnc2	troponin C2, fast	0.9589	0.4858	0.0337	0.3348	No	0.9057	0.14	0.386	0.0072	No	No
93261_at	Lgmn	legumain	0.9977	0.9566	0.1537	0.1051	No	0.9233	0.163	0.137	0.513	No	No
93264_at	Srebf1	sterol regulatory element binding factor 1	0.9598	0.4071	0.004	0.4707	No	1.0117	0.87	0.0206	0.704	No	No
93265_at	Tpm3	tropomyosin 3, gamma	1.0756	0.046	0.0007	0.0348	No	1.045	0.132	0.0001	0.738	No	No
93266_at	Tpm3	tropomyosin 3, gamma	0.9336	0.0217	0.0095	0.1848	No	1.058	0.249	0.0752	0.387	No	No
93267_at	Rnpc2	RNA-binding region (RNP1, RRM) containing 2	1.0819	0.2237	0.0119	0.0982	No	0.9423	0.253	0.0677	0.595	No	No
93268_at	Glo1	glyoxalase 1	1.1395	0.0774	0.0026	0.1626	No	1.055	0.467	0.0095	0.176	No	No
93269_at	Glo1	glyoxalase 1	1.0021	0.9395	0.0037	0.021	No	1.0708	0.0557	0.142	0.228	No	No
93270_at	Gars	glycyl-tRNA synthetase	1.0326	0.2772	0.122	0.0374	No	1.0541	0.0667	0.0771	0.161	No	No
93271_s_at	Gnas	GNAS (guanine nucleotide binding protein, alpha stimulating) complex locus	1.0229	0.0506	0.007	0.0129	No	1.0194	0.112	0.0021	0.293	No	No
93272_at	Stk16	serine/threonine kinase 16	1.0657	0.1446	0.1694	0.2054	No	1.0046	0.647	0.0032	0.0171	No	No
93273_at	Snca	synuclein, alpha	0.959	0.6938	0.0053	0.1546	No	0.9768	0.57	0.0743	0.101	No	No
93274_at	Clk1	CDC-like kinase 1	0.9355	0.2655	0.0246	0.2183	No	0.9282	0.296	0.02	0.618	No	No
93275_at	Sh3gl1	SH3-domain GRB2-like 1	1.0117	0.7211	0.0185	0.0924	No	0.9737	0.335	0.159	0.0249	No	No
93276_at	Hn1	hematological and neurological expressed sequence 1	1.0506	0.1332	0.004	0.4424	No	1.0447	0.265	0.0403	0.737	No	No
93277_at	Hspd1	heat shock protein 1 (chaperonin)	1.005	0.9002	0.6539	0.8914	No	0.9928	0.828	0.427	0.808	No	No
93278_at	Scp2	sterol carrier protein 2, liver	1.1367	0.0265	0.002	0.1655	No	1.0183	0.698	0.173	0.165	No	No
93279_at	Cryab	crystallin, alpha B	1.0314	0.0657	0.0001	0.0413	No	0.8973	0.135	0.0063	0.473	No	No
93281_at	Rcn2	reticulocalbin 2	1.0693	0.2433	0.0241	0.275	No	0.9613	0.303	0.0002	0.733	No	No
93282_at	Aprt	adenine phosphoribosyl transferase	1.028	0.5559	0.0299	0.0899	No	0.7311	0.0666	0.0019	0.0129	No	No
93283_at	Aprt	adenine phosphoribosyl transferase	0.9671	0.5515	0.083	0.7553	No	1.0399	0.575	0.191	0.928	No	No
93284_at	Cirbp	cold inducible RNA binding protein	1.0492	0.3634	0.1948	0.9389	No	1.0086	0.816	0.263	0.657	No	No
93285_at	Dusp6	dual specificity phosphatase 6	0.9466	0.3974	0.0071	0.2202	No	0.9341	0.333	0.005	0.117	No	No
93287_at	Bik	Bcl2-interacting killer	0.9303	0.1114	0.0458	0.2382	No	0.9736	0.618	0.0199	0.0554	No	No
93288_at	Arpc2	actin related protein 2/3 complex, subunit 2	1.0898	0.1312	0.0065	0.874	No	1.0126	0.802	0.402	0.532	No	No
93290_at	Pnp /// LOC545044 /// LOC667034	purine-nucleoside phosphorylase /// similar to purine-nucleoside phosphorylase /// similar to purine-nucleoside phosphorylase	0.9535	0.0604	0.0006	0.0047	No	0.9633	0.879	0.0181	0.038	No	No
93293_at	Calm2	calmodulin 2	1.0237	0.4137	0.0747	0.485	No	0.9871	0.527	0.074	0.75	No	No
93294_at	Ctgf	connective tissue growth factor	0.7582	0.0104	0.0045	0.1016	No	0.8332	0.0131	0.0051	0.387	No	No
93295_at	Cct5	chaperonin subunit 5 (epsilon)	1.0366	0.565	0.1118	0.2551	No	0.9929	0.971	0.0034	0.0716	No	No
93296_at	Tcl1	T-cell lymphoma breakpoint 1	0.9486	0.5767	0.1107	0.9721	No	1.0863	0.38	0.182	0.229	No	No
93298_at	Papss1	3'-phosphoadenosine 5'-phosphosulfate synthase 1	1.0255	0.3973	0.0031	0.4838	No	1.0379	0.0937	0.0026	0.102	No	No
93299_at	Msh6 /// LOC633695	mutS homolog 6 (E. coli) /// similar to mutS homolog 6	1.0393	0.5387	0.01	0.4071	No	1.0202	0.644	0.007	0.733	No	No
93300_at	Tgfb2	transforming growth factor, beta 2	1.143	0.0618	0.0009	0.3444	No	0.9614	0.302	0.006	0.371	No	No
93301_at	1300007B12 Rik	RIKEN cDNA 1300007B12 gene	0.958	0.5122	0.1011	0.4224	No	1.0297	0.362	0.103	0.3	No	No
93302_at	Tff2	trefoil factor 2 (spasmolytic protein 1)	0.9601	0.3889	0.0006	0.0234	No	0.9338	0.0638	0.0003	0.37	No	No
93303_at	Ufd1l	ubiquitin fusion degradation 1 like	1.0599	0.3048	0.0231	0.2512	No	1.1059	0.161	0.0333	0.207	No	No
93304_at	---	---	1.0386	0.3795	0.5268	0.1838	No	1.108	0.536	0.31	0.371	No	No
93305_f_at	Vamp8	vesicle-associated membrane protein 8	0.9523	0.4137	0.0157	0.1141	No	0.9384	0.193	0.0035	0.186	No	No
93306_at	Mapre1	microtubule-associated protein, RP/EB family, member 1	1.0964	0.3837	0.1374	0.7518	No	1.1015	0.147	0.212	0.983	No	No
93308_s_at	Pcx	pyruvate carboxylase	0.9527	0.0091	0.0003	0.0184	Yes	0.9338	0.176	0.214	0.88	No	No

93309_at	Ddx3x	DEAD/H (Asp-Glu-Ala-Asp/His) box polypeptide 3, X-linked	1.0719	0.5329	0.2316	0.0783	No	0.991	0.418	0.0009	0.0194	No	No
93310_at	Psmc6	proteasome (prosome, macropain) 26S subunit, ATPase, 6	1.0323	0.5209	0.0034	0.3581	No	1.0175	0.716	0.0093	0.919	No	No
93311_at	Clk3	CDC-like kinase 3	0.9278	0.0472	0.237	0.0039	No	0.9136	0.0009	0.0001	0.0003	No	No
93312_at	Ube2g1	ubiquitin-conjugating enzyme E2G 1 (UBC7 homolog, C. elegans)	1.0232	0.3638	0.0027	0.83	No	0.989	0.844	0.0928	0.632	No	No
93313_at	Map2k3	mitogen activated protein kinase kinase 3	0.9603	0.4103	0.0219	0.2797	No	1.0457	0.288	0.002	0.745	No	No
93314_g_at	Map2k3	mitogen activated protein kinase kinase 3	0.9741	0.3056	0.0002	0.0751	No	0.9631	0.25	0.0013	0.0668	No	No
93315_at	Map2k3	mitogen activated protein kinase kinase 3	0.9226	0.1255	0.1815	0.415	No	0.9928	0.846	0.0175	0.591	No	No
93316_at	Osbpl1a	oxysterol binding protein-like 1A	0.9731	0.7766	0.0236	0.567	No	0.9312	0.426	0.041	0.244	No	No
93317_at	Osbpl1a	oxysterol binding protein-like 1A	1.0292	0.3565	0.0051	0.4201	No	1.0107	0.802	0	0.0347	No	No
93318_at	Ninj1	ninjurin 1	0.9213	0.028	0.0003	0.0047	No	0.9185	0.0858	0.0015	0.0496	No	No
93319_at	Rasa3	RAS p21 protein activator 3	0.8853	0.0857	0.3917	0.0538	No	0.9132	0.184	0.895	0.0839	No	No
93320_at	Cpt1a	carnitine palmitoyltransferase 1a, liver	1.0228	0.5167	0.0005	0.0429	No	1.0482	0.184	0.0012	0.0375	No	No
93321_at	Ifi203	interferon activated gene 203	0.9796	0.6409	0.6486	0.8196	No	0.9133	0.013	0.999	0.346	No	No
93322_at	Pink1	PTEN induced putative kinase 1	0.9962	0.8943	0.5036	0.743	No	1.0524	0.629	0.195	0.533	No	No
93323_at	Plp2	proteolipid protein 2	0.9842	0.5256	0.0003	0.0412	No	0.9683	0.464	0.0002	0.034	No	No
93324_at	Zfp36l1	zinc finger protein 36, C3H type-like 1	0.8275	0.0232	0.0009	0.7796	No	0.8122	0.0141	0.0037	0.89	No	No
93325_at	Polr2e	polymerase (RNA) II (DNA directed) polypeptide E	1.06	0.4522	0.1881	0.242	No	1.1224	0.135	0.0647	0.424	No	No
93326_at	Tspan7	tetraspanin 7	0.9115	0.2356	0.013	0.397	No	0.9234	0.349	0.0663	0.35	No	No
93327_at	Tax1bp3	Tax1 (human T-cell leukemia virus type I) binding protein 3	0.935	0.1838	0.0118	0.0379	No	0.904	0.114	0.0094	0.0364	No	No
93328_at	Hdc	histidine decarboxylase	1.0023	0.9677	0.0339	0.3661	No	1.1278	0.0198	0.0288	0.0706	No	No
93330_at	Aqp1	aquaporin 1	0.9669	0.5348	0.2054	0.7899	No	0.9798	0.641	0.0729	0.67	No	No
93332_at	Cd36	CD36 antigen	1.0044	0.9373	0.1315	0.7643	No	1.2026	0.301	0.73	0.639	No	No
93333_at	Tbca	tubulin cofactor a	1.1383	0.1736	0.4739	0.7035	No	1.0232	0.607	0.0679	0.209	No	No
93334_at	Acadvl	acyl-Coenzyme A dehydrogenase, very long chain	1.0127	0.7348	0.1136	0.4909	No	1.1713	0.013	0.0317	0.0696	No	No
93336_at	Tmed10	transmembrane emp24-like trafficking protein 10 (yeast)	1.0432	0.1733	0.0006	0.0591	No	0.9185	0.115	0.0003	0.118	No	No
93337_at	Vps4b	vacuolar protein sorting 4b (yeast)	1.2102	0.0704	0.0019	0.2355	No	1.064	0.59	0.0011	0.61	No	No
93338_at	Mdm4	transformed mouse 3T3 cell double minute 4	1.0566	0.3724	0.5608	0.2068	No	0.9195	0.176	0.407	0.0149	No	No
93339_at	Mdm4	transformed mouse 3T3 cell double minute 4	0.9113	0.1828	0.0204	0.8369	No	0.9297	0.087	0.0466	0.0334	No	No
93340_f_at	Copb2	coatomer protein complex, subunit beta 2 (beta prime)	1.0503	0.6063	0.0434	0.6789	No	0.9468	0.271	0.0332	0.0966	No	No
93341_r_at	Copb2	coatomer protein complex, subunit beta 2 (beta prime)	1.0379	0.4677	0.0237	0.1118	No	0.9641	0.516	0.0078	0.0455	No	No
93342_at	Mki67ip	Mki67 (FHA domain) interacting nucleolar phosphoprotein	1.0148	0.8585	0.0955	0.2856	No	0.9716	0.821	0.0038	0.155	No	No
93346_at	Pgk1	phosphoglycerate kinase 1	1.0154	0.4916	0.016	0.5077	No	1.0449	0.394	0.624	0.739	No	No
93347_at	Rab24	RAB24, member RAS oncogene family	1.0776	0.0408	0.7686	0.0129	No	1.0553	0.18	0.104	0.0443	No	No
93348_at	Timm22	translocase of inner mitochondrial membrane 22 homolog (yeast)	0.9695	0.6814	0.7582	0.4033	No	1.0227	0.193	0.0002	0.0201	No	No
93349_at	Pcolce	procollagen C-endopeptidase enhancer protein	0.9422	0.26	0.0011	0.5645	No	0.9727	0.364	0.0005	0.164	No	No
93350_f_at	Zfp422	zinc finger protein 422	0.9563	0.1952	0.3034	0.0952	No	1.054	0.334	0.596	0.437	No	No
93351_at	Hpgd	hydroxyprostaglandin dehydrogenase 15 (NAD)	0.9546	0.4888	0.025	0.6583	No	0.9155	0.0402	0.0066	0.285	No	No
93352_at	Tgm2	transglutaminase 2, C polypeptide	0.984	0.6441	0.4317	0.0721	No	0.8601	0.007	0.964	0.25	Yes	No
93353_at	Lum	lumican	1.0343	0.5985	0.1273	0.1894	No	0.9787	0.691	0.0857	0.0965	No	No
93354_at	Apoc1	apolipoprotein C-I	0.975	0.3606	0.0331	0.165	No	1.0169	0.629	0.0055	0.178	No	No
93355_at	Got2	glutamate oxaloacetate transaminase 2, mitochondrial	1.1063	0.0391	0.0004	0.1203	No	0.9939	0.937	0	0.574	No	No
93356_at	Mcm7	minichromosome maintenance deficient 7 (S. cerevisiae)	1.0444	0.2135	0.0024	0.404	No	1.014	0.75	0.0117	0.651	No	No
93358_at	Eif1ay	eukaryotic translation initiation factor 1A, Y-linked	1.094	0.0794	0.0389	0.6273	No	1.0795	0.186	0.003	0.486	No	No
93359_at	Ndufc2	NADH dehydrogenase (ubiquinone) 1, subcomplex unknown, 2	0.9822	0.8529	0.5367	0.5472	No	1.0626	0.448	0.97	0.36	No	No
93360_at	Pmm1	phosphomannomutase 1	0.9473	0.189	0.0688	0.2974	No	0.9713	0.403	0.381	0.951	No	No
93362_at	Ap2m1	adaptor protein complex AP-2, mu1	1.056	0.2205	0.0064	0.3974	No	1.0196	0.505	0.0685	0.94	No	No
93364_at	Ctnna1	catenin (cadherin associated protein), alpha 1	0.9432	0.1351	0.0073	0.315	No	0.9789	0.69	0.862	0.327	No	No
93365_s_at	Sugt1	SGT1, suppressor of G2 allele of SKP1 (S. cerevisiae)	1.0008	0.9884	0.3233	0.1044	No	0.9732	0.676	0.558	0.0429	No	No
93366_r_at	Sugt1	SGT1, suppressor of G2 allele of SKP1 (S. cerevisiae)	0.9911	0.8456	0.0402	0.1957	No	0.9346	0.252	0.42	0.548	No	No
93367_at	Tep1	telomerase associated protein 1	0.9881	0.7861	0.0414	0.4004	No	0.9251	0.0979	0.014	0.463	No	No
93369_at	Omd	osteomodulin	0.862	0.0424	0.0036	0.0339	No	0.7771	0.0553	0.0155	0.381	No	No
93370_at	---	---	0.9563	0.3626	0.0041	0.3852	No	0.9724	0.666	0.0018	0.772	No	No
93371_at	Bnc1	basonuclin 1	1.0337	0.3691	0.0043	0.0209	No	1.0476	0.168	0.0003	0.679	No	No
93372_at	Anp32a	acidic (leucine-rich) nuclear phosphoprotein 32 family, member A	0.799	0.0262	0.0038	0.019	No	0.8361	0.0021	0.0002	0.0014	No	No
93373_at	Naglu	alpha-N-acetylglucosaminidase (Sanfilippo disease IIIB)	0.9396	0.0784	0.0006	0.0451	No	0.9814	0.433	0.0001	0.817	No	No
93374_at	Jph3	junctophilin 3	1.0192	0.6495	0.0009	0.4064	No	1.0421	0.537	0.0322	0.485	No	No
93375_at	Rspo2	R-spondin 2 homolog (Xenopus laevis)	1.0335	0.4513	0.0036	0.6361	No	1.0147	0.958	0.0163	0.259	No	No
93376_at	Bmp2k	BMP2 inducible kinase	1.0596	0.5075	0.0062	0.3887	No	1.0679	0.275	0.0021	0.942	No	No
93378_at	Hoxc8	homeo box C8	1.0614	0.4065	0.0574	0.0841	No	1.0941	0.179	0.0472	0.0242	No	No
93379_at	Dpysl4	dihydropyrimidinase-like 4	0.9746	0.0389	0.005	0.005	No	1.0609	0.0982	0.0102	0.0179	No	No
93380_at	Klrb1c	killer cell lectin-like receptor subfamily B member 1C	1.0373	0.5297	0.047	0.686	No	0.9738	0.399	0.104	0.383	No	No
93381_at	Avp	arginine vasopressin	0.9805	0.5301	0.1276	0.3062	No	0.9429	0.08	0.0787	0.253	No	No
93382_at	Pde1b	phosphodiesterase 1B, Ca2+-calmodulin dependent	0.9378	0.2655	0.1586	0.9282	No	0.9819	0.763	0.381	0.782	No	No
93383_at	Col7a1	procollagen, type VII, alpha 1	0.9826	0.3901	0.0004	0.0036	No	0.9901	0.871	0.164	0.482	No	No
93384_at	Sntb1	syntrophin, basic 1	1.0514	0.185	0.0046	0.3781	No	1.0046	0.958	0.0126	0.312	No	No
93385_at	Nthl1	nth (endonuclease III)-like 1 (E.coli)	1.0016	0.9466	0.004	0.2563	No	0.995	0.935	0.0084	0.522	No	No
93386_at	Amac1	acyl-malonyl condensing enzyme 1	1.0263	0.2353	0.0042	0.5711	No	0.9902	0.669	0.865	0.163	No	No
93387_at	Kera	keratocan	0.9552	0.536	0.9588	0.3291	No	0.9543	0.571	0.572	0.848	No	No
93388_s_at	Spib	Spi-B transcription factor (Spi-1/PU.1 related)	1.0212	0.6731	0.0173	0.4773	No	1.0054	0.914	0.648	0.928	No	No
93389_at	Prom1	prominin 1	0.933	0.23	0.5614	0.866	No	0.9805	0.765	0.422	0.849	No	No
93390_g_at	Prom1	prominin 1	1.0047	0.91	0.0015	0.3371	No	1.0416	0.304	0.0061	0.0299	No	No
93391_at	Cryqs	crystallin, gamma S	0.932	0.2732	0.0611	0.7221	No	1.0145	0.616	0.0189	0.272	No	No
93392_at	Ucp3	uncoupling protein 3 (mitochondrial, proton carrier)	0.8932	0.0611	0.0248	0.0765	No	0.9781	0.651	0.0063	0.859	No	No
93393_at	Eda	ectodysplasin-A	0.9058	0.164	0.3968	0.5221	No	0.9342	0.114	0.0063	0.393	No	No
93394_at	Eda /// LOC675952	ectodysplasin-A /// similar to Ectodysplasin-A (EDA protein homolog) (Tabby protein)	0.9836	0.1913	0.0021	0.0021	No	0.8143	0.647	0.227	0.213	No	No
93395_g_at	Eda /// LOC675952	ectodysplasin-A /// similar to Ectodysplasin-A (EDA protein homolog) (Tabby protein)	1.0142	0.7079	0.0329	0.1414	No	1.0265	0.67	0.0283	0.834	No	No
93396_at	Slc4a1ap	solute carrier family 4 (anion exchanger), member 1, adaptor protein	0.9746	0.6535	0.0275	0.4384	No	1.0135	0.843	0.0007	0.0546	No	No
93397_at	Ccr2	chemokine (C-C motif) receptor 2	1.0552	0.0443	0.0024	0.2273	No	0.9968	0.905	0.0273	0.0415	No	No
93398_at	U2af1-rs2	U2 small nuclear ribonucleoprotein auxiliary factor (U2AF) 1, related sequence 2	0.9959	0.9472	0.1132	0.8503	No	1.1032	0.504	0.664	0.288	No	No
93399_at	Rai2	retinoic acid induced 2	1.0423	0.4432	0.0273	0.3016	No	1.0542	0.02	0.758	0.0222	No	No
93400_at	Birc4	baculoviral IAP repeat-containing 4	1.0941	0.2851	0.4055	0.4189	No	1.0719	0.318	0.0564	0.251	No	No
93401_g_at	Birc4	baculoviral IAP repeat-containing 4	1.03	0.4304	0.5373	0.1642	No	1.2679	0.0808	0.164	0.0774	No	No
93402_i_at	Birc4	baculoviral IAP repeat-containing 4	1.0751	0.4856	0.0635	0.1252	No	1.0671	0.217	0.0143	0.0932	No	No
93403_at	Atp2a3	ATPase, Ca++ transporting, ubiquitous	0.9968	0.973	0.061	0.8675	No	1.0927	0.15	0.168	0.11	No	No
93404_g_at	Atp2a3	ATPase, Ca++ transporting, ubiquitous	1.0002	0.9947	0.2391	0.0588	No	1.1477	0.0091	0.307	0.003	No	No
93405_at	E2f3	E2F transcription factor 3	1.0041	0.8502	0.1413	0.0033	No	0.9946	0.915	0.0276	0.0135	No	No
93406_at	Mafg	v-maf musculoaponeurotic fibrosarcoma oncogene family, protein G (avian)	1.0086	0.8235	0.002	0.0251	No	1.0053	0.529	0.0014	0.0055	No	No
93407_at	Abcc6	ATP-binding cassette, sub-family C (CFTR/MRP), member 6	0.9043	0.287	0.5524	0.7172	No	1.0175	0.98	0.0059	0.347	No	No
93408_at	---	Transcribed locus	0.9867	0.6932	0.2252	0.1479	No	0.9284	0.0477	0.0069	0.0388	No	No
93409_at	---	---	0.9343	0.4423	0.8587	0.6062	No	1.0453	0.298	0.0147	0.285	No	No
93410_at	Wnk2	WNK lysine deficient protein kinase 2	0.9855	0.6165	0.5822	0.2744	No	1.1297	0.113	0.0221	0.0104	No	No
93411_at	Sema7a	sema domain, immunoglobulin domain (Ig), and GPI membrane anchor, (semaphorin) 7A	0.9528	0.111	0.0112	0.5456	No	0.9777	0.726	0.61	0.916	No	No
93412_at	Darc	Duffy blood group, chemokine receptor	0.9889	0.7363	0.3509	0.134	No	0.9517	0.337	0.475	0.973	No	No
93413_at	Terf2	telomeric repeat binding factor 2	0.9237	0.0978	0.0191	0.3233	No	0.9056	0.115	0.0158	0.183	No	No
93414_at	Abcb1b	ATP-binding cassette, sub-family B (MDR/TAP), member 1B	0.9754	0.4143	0.0362	0.0708	No	0.7849	0.0891	0.0317	0.302	No	No

93416_at	Tnfsf11	tumor necrosis factor (ligand) superfamily, member 11	1.0162	0.5614	0.066	0.0168	No	0.9607	0.229	0.924	0.0532	No	No
93417_at	Mef2b	myocyte enhancer factor 2B	0.9523	0.4823	0.107	0.5666	No	1.0068	0.838	0.0109	0.253	No	No
93418_g_at	Mef2b	myocyte enhancer factor 2B	1.0941	0.1058	0.0016	0.3744	No	1.0371	0.641	0.0079	0.0989	No	No
93419_at	1110061O04 Rik	RIKEN cDNA 1110061O04 gene	0.9005	0.0052	0.0616	0.0636	Yes	0.8732	0.001	0.151	0.0582	Yes	Yes
93421_at	Pftk1	PFTAIRE protein kinase 1	1.0746	0.2972	0.012	0.5416	No	0.9817	0.681	0.0523	0.261	No	No
93422_at	Pftk1	PFTAIRE protein kinase 1	1.033	0.2509	0.7777	0.0579	No	1.0096	0.853	0.643	0.861	No	No
93423_at	Ldoc1l	leucine zipper, down-regulated in cancer 1-like	0.959	0.145	0.0917	0.8511	No	1.0891	0.095	0.0731	0.246	No	No
93424_at	Phr1	pam, highwire, rpm 1	1.0159	0.7654	0.0005	0.5149	No	1.1058	0.0762	0.0001	0.626	No	No
93425_at	Irf5	interferon regulatory factor 5	1.0063	0.8193	0.012	0.0904	No	1.0525	0.212	0.004	0.0126	No	No
93426_at	D530031C13Rik	RIKEN cDNA D530031C13 gene	0.9708	0.181	0	0.1087	No	0.9529	0.0594	0.0002	0.471	No	No
93427_at	Myo1d	myosin ID	0.9252	0.2019	0.0098	0.0265	No	0.9132	0.0783	0.0217	0.12	No	No
93428_at	2610300B10 Rik	RIKEN cDNA 2610300B10 gene	1.0033	0.9222	0.0515	0.0652	No	1.0568	0.143	0.0032	0.0859	No	No
93429_at	Zp2	zona pellucida glycoprotein 2	0.9618	0.6619	0.3049	0.697	No	1.0809	0.0435	0.0091	0.0217	No	No
93430_at	Cmkor1	chemokine orphan receptor 1	1.0571	0.5752	0.006	0.1989	No	1.1165	0.163	0.003	0.108	No	No
93431_at	Dmpk	dystrophia myotonica-protein kinase	0.9674	0.4544	0.0041	0.9385	No	0.9193	0.418	0.539	0.314	No	No
93432_at	Zfp276	zinc finger protein (C2H2 type) 276	0.9949	0.8147	0.0424	0.5745	No	0.9581	0.0509	0.0096	0.369	No	No
93433_s_at	Asgr1	asialoglycoprotein receptor 1	1.0065	0.8371	0.1732	0.2946	No	0.9744	0.675	0.246	0.76	No	No
93434_at	Sult5a1	sulfotransferase family 5A, member 1	0.9277	0.2871	0.0893	0.5982	No	0.9463	0.187	0.322	0.0859	No	No
93435_at	Cyp24a1	cytochrome P450, family 24, subfamily a, polypeptide 1	1.0086	0.6541	0.0469	0.8946	No	0.9783	0.701	0.502	0.276	No	No
93436_i_at	4632419I22 Rik	RIKEN cDNA 4632419I22 gene	1.127	0.2215	0.0185	0.8736	No	1.1599	0.428	0.0235	0.552	No	No
93437_f_at	4632419I22 Rik	RIKEN cDNA 4632419I22 gene	0.8918	0.2383	0.2292	0.2075	No	0.9258	0.304	0.422	0.468	No	No
93439_f_at	---	---	0.9266	0.0509	0.0005	0.0341	No	0.9435	0.444	0.535	0.941	No	No
93440_at	Sec22b	SEC22 vesicle trafficking protein homolog B (S. cerevisiae)	0.9961	0.9247	0.0166	0.8331	No	1.0209	0.566	0.0178	0.713	No	No
93441_at	2700099C18 Rik	RIKEN cDNA 2700099C18 gene	1.0429	0.5988	0.0252	0.3274	No	1.0045	0.776	0.0012	0.316	No	No
93442_at	Zfp316	zinc finger protein 316	0.9198	0.1491	0.0574	0.1167	No	0.9585	0.364	0.0358	0.822	No	No
93443_at	Fndc1	fibronectin type III domain containing 1	1.0026	0.9326	0.0027	0.1404	No	0.9442	0.489	0.0193	0.77	No	No
93444_at	Batf	basic leucine zipper transcription factor, ATF-like	0.9892	0.6549	0.0416	0.2659	No	0.9902	0.683	0.144	0.425	No	No
93445_at	Cd5l	CD5 antigen-like	0.9121	0.1244	0.7508	0.1235	No	0.9812	0.566	0.529	0.428	No	No
93446_at	Zfp60	zinc finger protein 60	0.9853	0.7989	0.4702	0.585	No	0.9055	0.363	0.046	0.862	No	No
93447_at	---	---	1.0707	0.1271	0.9415	0.5379	No	1.078	0.291	0.813	0.543	No	No
93448_at	E330036I19 Rik	RIKEN cDNA E330036I19 gene	0.9192	0.1012	0.0917	0.2872	No	0.9664	0.352	0.0773	0.0469	No	No
93449_at	Rfpl4	ret finger protein-like 4	1.0114	0.6944	0.0687	0.0699	No	0.9026	0.0404	0.252	0.917	No	No
93450_at	Cenpe	centromere protein E	0.9918	0.8763	0.0058	0.3482	No	0.9981	0.278	0	0.006	No	No
93451_at	Lmo7	LIM domain only 7	0.7993	0.0503	0.0024	0.7015	No	0.8076	0.0588	0.0021	0.54	No	No
93452_at	Cer1	cerberus 1 homolog (Xenopus laevis)	1.1105	0.0491	0.3615	0.1042	No	1.0117	0.831	0.0507	0.871	No	No
93453_at	Rom1	rod outer segment membrane protein 1	0.9269	0.5608	0.4457	0.6525	No	0.9256	0.318	0.0009	0.172	No	No
93454_at	Cd93	CD93 antigen	0.9185	0.0603	0.3501	0.831	No	0.9265	0.0847	0.0383	0.521	No	No
93455_s_at	Bmp4	bone morphogenetic protein 4	0.9392	0.0937	0.0177	0.0994	No	0.9463	0.0956	0.0029	0.439	No	No
93456_r_at	Bmp4	bone morphogenetic protein 4	0.9721	0.7489	0.011	0.7082	No	0.9713	0.716	0.214	0.0973	No	No
93457_at	Crygb	crystallin, gamma B	0.9753	0.6846	0.2573	0.8256	No	0.7202	0.0221	0.113	0.852	No	No
93458_at	C330003B14 Rik	RIKEN cDNA C330003B14 gene	0.8477	0.2072	0.0369	0.2374	No	0.9205	0.127	0.0589	0.249	No	No
93459_s_at	Fzd4	frizzled homolog 4 (Drosophila)	1.0059	0.8855	0.0004	0.5735	No	0.9668	0.554	0.0607	0.583	No	No
93460_at	Acvr1	activin A receptor, type 1	1.0415	0.4873	0.1562	0.0629	No	1.0008	0.806	0.0002	0.125	No	No
93461_at	Birc1a	baculoviral IAP repeat-containing 1a	1.067	0.2471	0.0635	0.1671	No	1.076	0.446	0.497	0.951	No	No
93462_at	1810054D07 Rik	RIKEN cDNA 1810054D07 gene	1.0296	0.6132	0.4346	0.5266	No	1.0686	0.218	0.0053	0.145	No	No
93463_at	Usp19	ubiquitin specific peptidase 19	1.0861	0.2897	0.0804	0.4137	No	1.1545	0.0884	0.0482	0.105	No	No
93464_at	Akap9	A kinase (PRKA) anchor protein (yotiao) 9	0.7598	0.0737	0.0009	0.2211	No	0.7975	0.0647	0.0009	0.163	No	No
93465_at	9130211I03 Rik	RIKEN cDNA 9130211I03 gene	1.0295	0.3258	0.0014	0.7722	No	0.9982	0.958	0.0286	0.914	No	No
93466_at	Exoc4	exocyst complex component 4	1.0283	0.5185	0.0016	0.4778	No	1.0215	0.763	0.0481	0.441	No	No
93467_at	Cachd1	cache domain containing 1	0.9762	0.0832	0	0.8266	No	0.8842	0.0819	0.109	0.999	No	No
93468_at	Aff1	AF4/FMR2 family, member 1	0.9786	0.5264	0.8371	0.8756	No	0.958	0.505	0.862	0.704	No	No
93469_at	Ephb3	Eph receptor B3	1.0041	0.8443	0.0553	0.0066	No	1.2579	0.0047	0.0363	0.131	Yes	No
93470_at	Dnmt2	DNA methyltransferase 2	1.002	0.9784	0.725	0.6188	No	1.0127	0.794	0.0786	0.742	No	No
93471_at	Slc4a7	Solute carrier family 4, sodium bicarbonate cotransporter, member 7	1.1481	0.1819	0.0012	0.5978	No	1.1203	0.117	0.0005	0.379	No	No
93472_at	Col5a1	Procollagen, type V, alpha 1	0.9206	0.1687	0.0141	0.0162	No	0.9148	0.224	0.0182	0.0293	No	No
93475_at	Spef1	sperm flagellar 1	1.0356	0.5407	0.097	0.2706	No	0.9645	0.428	0.0268	0.274	No	No
93476_at	Prkdc	protein kinase, DNA activated, catalytic polypeptide	1.0404	0.4892	0.0803	0.2728	No	1.0467	0.349	0.0074	0.199	No	No
93478_at	4930461P20 Rik	RIKEN cDNA 4930461P20 gene	0.8569	0.0392	0.1822	0.0333	No	1.0527	0.374	0.69	0.417	No	No
93479_at	A930031D07 Rik	RIKEN cDNA A930031D07 gene	1.0222	0.6907	0.5057	0.5456	No	0.972	0.519	0.901	0.779	No	No
93480_at	Htra3	HtrA serine peptidase 3	0.9929	0.8098	0.0308	0.0514	No	1.0685	0.114	0.656	0.442	No	No
93481_at	Myh14 /// LOC672865	myosin, heavy polypeptide 14 /// similar to nonmuscle myosin heavy chain	1.0105	0.7163	0.0742	0.0626	No	0.9981	0.927	0.865	0.374	No	No
93482_at	Mylk	myosin, light polypeptide kinase	0.9759	0.3744	0.0113	0.7257	No	0.972	0.643	0.531	0.722	No	No
93483_at	Hck	hemopoietic cell kinase	0.9354	0.2897	0.2684	0.6809	No	0.9591	0.454	0.0606	0.162	No	No
93484_at	Gramd3	GRAM domain containing 3	1.0417	0.1924	0.01	0.5378	No	1.1355	0.27	0.326	0.822	No	No
93485_at	Ptprd	protein tyrosine phosphatase, receptor type, D	1.0269	0.7483	0.0022	0.1996	No	0.9217	0.369	0.0011	0.582	No	No
93486_at	Slc27a1	solute carrier family 27 (fatty acid transporter), member 1	1.0143	0.6979	0.249	0.7863	No	0.9985	0.973	0.401	0.636	No	No
93488_at	Tsr1	TSR1, 20S rRNA accumulation, homolog (yeast)	0.9259	0.1371	0.001	0.0892	No	0.9911	0.589	0.001	0.153	No	No
93489_at	Got1	glutamate oxaloacetate transaminase 1, soluble	0.9842	0.7419	0.2761	0.3785	No	1.1092	0.168	0.216	0.181	No	No
93490_at	Maf1	MAF1 homolog (S. cerevisiae)	0.9412	0.0002	0	0	No	0.937	0.0678	0.0021	0.223	No	No
93491_f_at	Ube2d3	ubiquitin-conjugating enzyme E2D 3 (UBC4/5 homolog, yeast)	1.1441	0.308	0.0101	0.6092	No	1.0803	0.476	0.0236	0.398	No	No
93492_at	Pscd2	pleckstrin homology, Sec7 and coiled-coil domains 2	0.9982	0.8164	0	0.0106	No	1.0593	0.242	0.018	0.376	No	No
93493_at	Ddx5	DEAD (Asp-Glu-Ala-Asp) box polypeptide 5	0.8955	0.1115	0.0062	0.1756	No	0.9576	0.26	0.0772	0.809	No	No
93495_at	Prdx4	peroxiredoxin 4	0.9342	0.1783	0.0264	0.0737	No	0.9767	0.928	0.0099	0.0232	No	No
93496_at	Elovl5	ELOVL family member 5, elongation of long chain fatty acids (yeast)	0.9584	0.5531	0.3372	0.4172	No	0.9804	0.778	0.556	0.203	No	No
93497_at	C3	complement component 3	0.9682	0.2194	0.6869	0.6248	No	0.987	0.927	0.195	0.905	No	No
93498_s_at	Aplp2	amyloid beta (A4) precursor-like protein 2	0.9326	0.4206	0.8341	0.6292	No	0.9355	0.301	0.0376	0.584	No	No
93499_at	Capza1	capping protein (actin filament) muscle Z-line, alpha 1	1.0431	0.216	0.0148	0.0337	No	1.1137	0.0373	0.0089	0.0154	No	No
93500_at	Alas1	aminolevulinic acid synthase 1	0.9877	0.7007	0.2064	0.1556	No	1.0237	0.601	0.192	0.933	No	No
93501_f_at	Sucla2	succinate-Coenzyme A ligase, ADP-forming, beta subunit	1.0698	0.2853	0.0294	0.0776	No	1.1228	0.104	0.0636	0.216	No	No
93502_r_at	Sucla2	succinate-Coenzyme A ligase, ADP-forming, beta subunit	1.0478	0.2	0.0005	0.0502	No	1.0419	0.466	0	0.0007	No	No
93503_at	Sfrp2	secreted frizzled-related protein 2	0.9439	0.0669	0.0404	0.0078	No	0.9294	0.168	0.157	0.344	No	No
93505_at	Metap2	methionine aminopeptidase 2	1.0316	0.6908	0.007	0.8999	No	1.0263	0.703	0.0018	0.885	No	No
93506_at	Slc25a3	solute carrier family 25 (mitochondrial carrier, phosphate carrier), member 3	0.9701	0.3351	0.9353	0.2975	No	1.0294	0.601	0.584	0.166	No	No
93507_at	Timp2	tissue inhibitor of metalloproteinase 2	1.1016	0.0434	0.0727	0.2244	No	1.0464	0.438	0.173	0.501	No	No
93509_at	Ube2b	ubiquitin-conjugating enzyme E2B, RAD6 homology (S. cerevisiae)	0.9103	0.1987	0.1634	0.4333	No	0.9177	0.116	0.819	0.0568	No	No
93511_at	Itm2a	integral membrane protein 2A	1.1151	0.1394	0.0155	0.1226	No	1.0336	0.465	0.603	0.692	No	No
93512_f_at	Adk	adenosine kinase	1.0537	0.401	0.0923	0.6039	No	1.0216	0.707	0.0607	0.785	No	No
93513_at	Rpl35a	ribosomal protein L35a	1.046	0.3465	0.1729	0.5123	No	1.0785	0.282	0.245	0.434	No	No
93514_at	Myl3	myosin, light polypeptide 3	0.8016	0.0123	0.9251	0.0073	No	0.7958	0.0095	0.579	0.0068	No	No
93515_at	Cdh16	cadherin 16	0.9253	0.1775	0.0062	0.5112	No	0.9793	0.758	0.571	0.802	No	No
93516_at	Dynlt1	dynein light chain Tctex-type 1	1.1415	0.1382	0.4738	0.1024	No	1.1201	0.213	0.166	0.0606	No	No
93517_at	Col6a2	procollagen, type VI, alpha 2	0.8138	0.0166	0.0036	0.4738	No	0.8411	0.01	0.0005	0.0082	No	No
93518_at	Rnps1	ribonucleic acid binding protein S1	0.9941	0.8093	0.0621	0.0562	No	0.9921	0.522	0.0364	0.0044	No	No
93519_s_at	Nedd8	neural precursor cell expressed, developmentally down-regulated gene 8	1.084	0.3085	0.0554	0.7818	No	1.062	0.314	0.0041	0.79	No	No
93520_at	Srrm1	serine/arginine repetitive matrix 1	1.0327	0.5635	0.0096	0.6716	No	1.0048	0.983	0.0034	0.742	No	No

93521_at	Srrm1	serine/arginine repetitive matrix 1	1.0507	0.3226	0.0187	0.7082	No	0.9895	0.821	0.0056	0.965	No	No
93522_at	Rad9	RAD9 homolog (S. pombe)	0.9923	0.9163	0.6623	0.3123	No	1.0049	0.891	0.205	0.708	No	No
93523_at	Calr	calreticulin	1.0666	0.0342	0.0067	0.0411	No	1.1288	0.0625	0.539	0.0304	No	No
93524_i_at	Ube2j2	Ubiquitin-conjugating enzyme E2, J2 homolog (yeast)	1.0141	0.5631	0.3161	0.9272	No	0.9798	0.938	0.0918	0.717	No	No
93525_f_at	Ube2j2 /// LOC545056	ubiquitin-conjugating enzyme E2, J2 homolog (yeast) /// ubiquitin-conjugating enzyme E2, J2 homolog pseudogene	1.0547	0.3476	0.015	0.4842	No	1.0636	0.168	0.0013	0.394	No	No
93526_at	Stra13	stimulated by retinoic acid 13	1.037	0.4278	0.0201	0.8792	No	1.0996	0.385	0.382	0.621	No	No
93527_at	Klf9	Kruppel-like factor 9	0.727	0.0001	0.0714	0.0119	Yes	0.8175	0.0059	0.0199	0.438	Yes	Yes
93528_s_at	Klf9	Kruppel-like factor 9	0.8375	0.0015	0	0.0025	No	0.8176	0.0038	0	0.0807	Yes	No
93529_at	Itfg1	integrin alpha FG-GAP repeat containing 1	1.0249	0.3884	0.0008	0.2275	No	0.9969	0.951	0.16	0.295	No	No
93530_at	---	---	1.0948	0.0169	0	0.0076	No	1.0666	0.192	0.0058	0.128	No	No
93531_at	Ndufa8	NADH dehydrogenase (ubiquinone) 1 alpha subcomplex, 8	0.9997	0.9933	0.07	0.1149	No	1.0664	0.104	0.0175	0.432	No	No
93532_at	Tnni2	troponin I, skeletal, fast 2	0.8893	0.0147	0.0947	0.0176	No	0.6786	0.0162	0.544	0.0056	No	No
93533_at	Ssu72	Ssu72 RNA polymerase II CTD phosphatase homolog (yeast)	0.9551	0.498	0.7271	0.0384	No	1.0003	0.684	0.006	0.0313	No	No
93534_at	Dcn	decorin	0.9925	0.857	0.1114	0.3749	No	0.9695	0.394	0.1	0.464	No	No
93535_at	Atxn2	ataxin 2	0.9605	0.1752	0	0.125	No	1.0302	0.488	0.001	0.693	No	No
93536_at	Bax	Bcl2-associated X protein	1.043	0.5872	0.3122	0.8054	No	0.9897	0.775	0.173	0.787	No	No
93538_at	Ttrap	Traf and Tnf receptor associated protein	1.0091	0.8847	0.0074	0.5561	No	0.8968	0.0135	0.0008	0.916	No	No
93539_at	Anapc13	anaphase promoting complex subunit 13	1.0808	0.0654	0.0661	0.3879	No	1.0192	0.534	0.0689	0.161	No	No
93540_at	Adprh	ADP-ribosylarginine hydrolase	0.9819	0.7331	0.1179	0.678	No	1.0243	0.64	0.222	0.559	No	No
93541_at	Tagln	transgelin	0.9172	0.1085	0.0032	0.8233	No	0.8814	0.0159	0.0751	0.0737	No	No
93542_at	Pter	phosphotriesterase related	1.0286	0.4904	0.0693	0.3277	No	1.0316	0.712	0.221	0.336	No	No
93543_f_at	Gstm1	glutathione S-transferase, mu 1	0.954	0.3326	0.0945	0.0359	No	0.99	0.59	0.0194	0.0966	No	No
93546_s_at	Cbfb	core binding factor beta	0.9781	0.1302	0.1593	0.0028	No	1.0058	0.658	0.371	0.0383	No	No
93547_at	Cbfb	core binding factor beta	1.0179	0.0105	0.0003	0.0005	No	1.0283	0.256	0.218	0.0942	No	No
93548_at	Sec61b	Sec61 beta subunit	1.0692	0.2437	0.0019	0.8598	No	1.0585	0.285	0.0014	0.724	No	No
93550_at	Csrp2	cysteine and glycine-rich protein 2	0.9545	0.3821	0.0034	0.2985	No	0.9059	0.185	0.0021	0.164	No	No
93551_at	Polr2l	polymerase (RNA) II (DNA directed) polypeptide L	1.0392	0.339	0.093	0.3577	No	1.0832	0.0169	0.001	0.0291	No	No
93555_at	Krt2-10	keratin complex 2, basic, gene 10	1.0449	0.2413	0.0057	0.4005	No	1.0641	0.155	0.0178	0.005	No	No
93556_at	Krt2-10	keratin complex 2, basic, gene 10	1.0773	0.0965	0.0228	0.0518	No	1.0168	0.235	0.0004	0.0071	No	No
93557_at	Sephs2	selenophosphate synthetase 2	1.2505	0.0882	0.0017	0.7871	No	1.1678	0.201	0.0015	0.635	No	No
93558_at	Slc35a2	solute carrier family 35 (UDP-galactose transporter), member 2	0.9502	0.2474	0.8993	0.07	No	1.0452	0.0507	0.0461	0.0066	No	No
93559_at	Apex1	apurinic/apyrimidinic endonuclease 1	1.0532	0.1498	0.0095	0.7119	No	1.0089	0.88	0.0266	0.688	No	No
93560_at	Acyp1	acylphosphatase 1, erythrocyte (common) type	0.9982	0.944	0.0024	0.0164	No	0.9525	0.345	0.0046	0.037	No	No
93561_at	Chchd6	coiled-coil-helix-coiled-coil-helix domain containing 6	1.068	0.0077	0	0.0028	No	0.9714	0.599	0.0039	0.0063	No	No
93562_at	Ndufb3	NADH dehydrogenase (ubiquinone) 1 beta subcomplex 3	1.0453	0.4605	0.0224	0.4396	No	1.0487	0.429	0.244	0.388	No	No
93563_s_at	Nid2	nidogen 2	1.1622	0.0951	0.0007	0.7842	No	1.1942	0.0786	0.0009	0.84	No	No
93564_at	Yars	tyrosyl-tRNA synthetase	0.9667	0.0803	0.2372	0.0027	No	0.9023	0.0358	0.882	0.0198	No	No
93565_at	Kns2	kinesin 2	1.1315	0.0622	0.0012	0.8685	No	1.1234	0.167	0.0144	0.682	No	No
93566_at	Gale	galactose-4-epimerase, UDP	1.0993	0.2049	0.3525	0.1181	No	1.2297	0.0226	0.102	0.0135	No	No
93567_at	Pfn2	profilin 2	1.0797	0.1684	0.908	0.1948	No	1.1119	0.183	0.0469	0.419	No	No
93568_i_at	LOC544988 /// LOC545007 /// 2610042L04 Rik /// LOC554327 /// LOC622916 /// LOC622931 /// LOC638853 /// LOC666193 /// LOC666329 /// LOC666442 /// LOC666684 /// LOC666772 /// LOC671957	hypothetical protein LOC544988 /// hypothetical protein LOC545007 /// RIKEN cDNA 2610042L04 gene /// hypothetical protein LOC554327 /// similar to Spetex-2F protein /// similar to Spetex-2H protein /// similar to Spetex-2F protein /// similar to Spetex-2H protein /// similar to Spetex-2H protein /// similar to Spetex-2H protein /// hypothetical protein LOC666772 /// hypothetical protein LOC671957	1.1327	0.4041	0.0298	0.7277	No	1.1886	0.138	0.0069	0.791	No	No
93569_f_at	LOC544988 /// B930046C15 Rik /// LOC545007 /// 2610042L04 Rik /// LOC554327 /// LOC622916 /// LOC622931 /// LOC666193 /// LOC666329 /// LOC666442 /// LOC666740 /// LOC666772	hypothetical protein LOC544988 /// RIKEN cDNA B930046C15 gene /// hypothetical protein LOC545007 /// RIKEN cDNA 2610042L04 gene /// hypothetical protein LOC554327 /// similar to Spetex-2F protein /// similar to Spetex-2H protein /// similar to Spetex-2F protein /// similar to Spetex-2H protein /// similar to Spetex-2H protein /// hypothetical protein LOC666740 /// hypothetical protein LOC666772	1.2633	0.05	0.0044	0.4061	No	1.1515	0.135	0.0234	0.482	No	No
93570_at	Slc12a3	solute carrier family 12, member 3	0.998	0.9678	0.156	0.8103	No	1.0287	0.568	0.352	0.192	No	No
93571_at	Spnb2	spectrin beta 2	0.9597	0.2439	0.0015	0.081	No	0.976	0.717	0.0031	0.14	No	No
93572_at	Ndufs1	NADH dehydrogenase (ubiquinone) Fe-S protein 1	1.0027	0.9368	0.0067	0.3332	No	0.9689	0.216	0.468	0.0765	No	No
93573_at	Mt1	metallothionein 1	0.7564	0.0062	0.0009	0.0248	Yes	0.7599	0.0027	0.0002	0.0095	No	No
93574_at	Serpinf1	serine (or cysteine) peptidase inhibitor, clade F, member 1	1.0408	0.4833	0.0164	0.8422	No	1.0423	0.284	0.0035	0.118	No	No
93575_at	Ggh /// LOC667301	gamma-glutamyl hydrolase /// similar to Gamma-glutamyl hydrolase precursor (Gamma-Glu-x carboxypeptidase) (Conjugase) (GH) (Folylpolyglutamate hydrolase) (FGPH)	0.9737	0.6971	0.1863	0.7317	No	0.9445	0.544	0.106	0.613	No	No
93578_at	Rpl15	Ribosomal protein L15	1.0703	0.4456	0.3719	0.7858	No	1.0471	0.653	0.0024	0.27	No	No
93579_at	Jagn1	jagunal homolog 1 (Drosophila)	1.0491	0.202	0.0016	0.037	No	1.0552	0.716	0.0017	0.13	No	No
93580_at	Pum2	pumilio 2 (Drosophila)	0.8835	0.009	0.0158	0.5206	Yes	0.9064	0.0827	0.0187	0.767	No	No
93581_at	Ndufb8	NADH dehydrogenase (ubiquinone) 1 beta subcomplex 8	1.0275	0.49	0.0001	0.0439	No	0.999	0.354	0	0.0569	No	No
93582_at	Coq7	demethyl-Q 7	1.026	0.5785	0.9787	0.2015	No	1.0103	0.729	0.801	0.225	No	No
93583_s_at	Igh-6	immunoglobulin heavy chain 6 (heavy chain of IgM)	0.9502	0.3757	0.0531	0.7509	No	1.0611	0.297	0.938	0.32	No	No
93584_at	Igh-6	immunoglobulin heavy chain 6 (heavy chain of IgM)	0.9207	0.0832	0.7916	0.3937	No	1.0092	0.793	0.555	0.152	No	No
93585_at	Cyp2c29	cytochrome P450, family 2, subfamily c, polypeptide 29	1.0146	0.6922	0.0157	0.0684	No	1.0308	0.519	0.0144	0.168	No	No
93586_at	Syngr2	synaptogyrin 2	0.9308	0.0368	0.1669	0.1248	No	0.899	0.0051	0.471	0.0018	No	No
93587_at	Anxa7	annexin A7	0.9633	0.1535	0.0716	0.146	No	0.9705	0.612	0.316	0.878	No	No
93588_at	Gtl3	gene trap locus 3	0.9881	0.7867	0.2461	0.3715	No	1.0015	0.832	0.0099	0.0715	No	No
93589_at	Entpd4	ectonucleoside triphosphate diphosphohydrolase 4	0.9914	0.7608	0.6044	0.1323	No	1.031	0.239	0.021	0.905	No	No

93590_at	Ndst1	N-deacetylase/N-sulfotransferase (heparan glucosaminyl) 1	0.9963	0.9618	0.3362	0.7035	No	1.0445	0.294	0.0258	0.553	No	No
93591_at	Pef1	penta-EF hand domain containing 1	1.0063	0.851	0.2483	0.7083	No	1.0584	0.241	0.0103	0.153	No	No
93592_at	Apod	apolipoprotein D	0.9196	0.1945	0.0019	0.1503	No	0.9165	0.0346	0.0012	0.0264	No	No
93593_f_at	Emp3	epithelial membrane protein 3	1.0459	0.2357	0.0014	0.0306	No	1.034	0.251	0.0021	0.0372	No	No
93594_r_at	Emp3	epithelial membrane protein 3	1.0295	0.174	0.0326	0.0047	No	1.0956	0.296	0.647	0.575	No	No
93595_at	Tpp1	tripeptidyl peptidase I	1.0763	0.1413	0.0005	0.2724	No	1.0294	0.832	0.0014	0.0539	No	No
93596_i_at	Atp5e	ATP synthase, H+ transporting, mitochondrial F1 complex, epsilon subunit	0.9432	0.3489	0.013	0.0216	No	0.9011	0.133	0.0003	0.0042	No	No
93597_at	Snx5	sorting nexin 5	0.9748	0.6593	0.3981	0.9523	No	1.0482	0.414	0.0303	0.196	No	No
93598_at	Snx5	sorting nexin 5	0.8996	0.1755	0.1904	0.3564	No	0.9243	0.185	0.0296	0.138	No	No
93599_at	2500003M10 Rik	RIKEN cDNA 2500003M10 gene	1.1374	0.3294	0.5069	0.9219	No	0.966	0.929	0.0037	0.134	No	No
93600_at	Leprot	leptin receptor overlapping transcript	1.0429	0.2823	0.0175	0.4161	No	1.0379	0.511	0.0007	0.566	No	No
93601_at	Keap1	kelch-like ECH-associated protein 1	0.982	0.59	0.0029	0.1336	No	0.9547	0.285	0.0015	0.746	No	No
93602_at	Rps6ka4	ribosomal protein S6 kinase, polypeptide 4	1.0347	0.3476	0.0012	0.627	No	1.0244	0.415	0.0019	0.114	No	No
93603_at	Mrpl40	mitochondrial ribosomal protein L40	0.9562	0.0384	0.0001	0.0011	No	0.9679	0.455	0.0001	0.0205	No	No
93604_f_at	Igsf4a	immunoglobulin superfamily, member 4A	1.0784	0.0742	0.0003	0.986	No	1.0551	0.211	0.0015	0.302	No	No
93605_r_at	Igsf4a	immunoglobulin superfamily, member 4A	1.0926	0.2345	0.0706	0.1814	No	1.0999	0.241	0.0158	0.229	No	No
93606_s_at	Igsf4a	immunoglobulin superfamily, member 4A	0.9538	0.1212	0.0042	0.6125	No	0.9818	0.665	0.0245	0.838	No	No
93608_at	Ebi3	Epstein-Barr virus induced gene 3	0.9962	0.9377	0.0557	0.7602	No	0.9521	0.305	0.74	0.0687	No	No
93609_at	Spg20	spastic paraplegia 20, spartin (Troyer syndrome) homolog (human)	0.9398	0.5251	0.1269	0.6952	No	0.9752	0.443	0	0	No	No
93611_at	Tbx6	T-box 6	0.9877	0.8987	0.0404	0.9153	No	0.9661	0.576	0.0031	0.534	No	No
93612_at	Mmp15	matrix metallopeptidase 15	1.0214	0.5918	0.2902	0.9159	No	0.9613	0.374	0.0508	0.992	No	No
93613_at	Smad3	MAD homolog 3 (Drosophila)	1.0355	0.2496	0.0065	0.8381	No	0.9393	0.157	0.258	0.682	No	No
93614_at	Rragd	Ras-related GTP binding D	1.0976	0.0412	0.0053	0.2496	No	1.1282	0.0053	0.0006	0.0013	No	No
93615_at	Pbx3	pre B-cell leukemia transcription factor 3	0.9785	0.7509	0.0266	0.0849	No	0.9866	0.989	0.0136	0.114	No	No
93616_g_at	Pbx3	pre B-cell leukemia transcription factor 3	1.0071	0.8954	0.5981	0.2833	No	1.0052	0.905	0.396	0.135	No	No
93617_at	Ccrl2	chemokine (C-C motif) receptor-like 2	0.9546	0.5445	0.2764	0.8665	No	0.9908	0.845	0.754	0.204	No	No
93618_at	Spnb3	spectrin beta 3	0.9951	0.7996	0.0554	0.0353	No	1.0606	0.145	0.2	0.127	No	No
93619_at	Per1	period homolog 1 (Drosophila)	0.7551	0.0007	0.0026	0.2174	Yes	0.8038	0.0032	0.119	0.0815	Yes	Yes
93620_at	Rpo1-4	RNA polymerase 1-4	0.895	0.0157	0.1065	0.0298	No	1.0319	0.541	0.0212	0.126	No	No
93621_at	Tgif2	TGFB-induced factor 2	1.064	0.0035	0.0784	0.0042	No	1.0801	0.777	0.162	0.342	No	No
93622_at	Mcpt6	mast cell protease 6	0.9922	0.5904	0.0016	0.9827	No	1.003	0.787	0.0249	0.801	No	No
93623_at	Dmd	dystrophin, muscular dystrophy	0.9647	0.4128	0.0005	0.0218	No	0.949	0.443	0	0.0034	No	No
93624_at	Slc10a6	solute carrier family 10 (sodium/bile acid cotransporter family), member 6	0.9186	0.1677	0.0918	0.6146	No	1.0472	0.217	0.003	0.727	No	No
93625_at	Agxt	alanine-glyoxylate aminotransferase	0.9308	0.1577	0.0051	0.0891	No	0.9472	0.351	0.0632	0.0122	No	No
93626_at	Abcg2	ATP-binding cassette, sub-family G (WHITE), member 2	0.9831	0.7599	0.0163	0.4601	No	0.8833	0.0359	0.046	0.0382	No	No
93627_at	Ankrd28	ankyrin repeat domain 28	0.8916	0.0547	0.0021	0.032	No	1.009	0.733	0.0019	0.233	No	No
93628_at	Prlpb	prolactin-like protein B	0.9545	0.0431	0.0006	0.0157	No	1.0331	0.569	0.0021	0.156	No	No
93629_s_at	Folh1	folate hydrolase	0.9506	0.2368	0.4155	0.3697	No	1.0203	0.731	0.0033	0.0212	No	No
93630_at	Cugbp1	CUG triplet repeat, RNA binding protein 1	1.0176	0.7227	0.7596	0.1662	No	1.0298	0.318	0.946	0.0049	No	No
93631_at	Arhgef2	rho/rac guanine nucleotide exchange factor (GEF) 2	0.9831	0.8527	0.0536	0.6521	No	0.9788	0.709	0.106	0.4	No	No
93632_g_at	Arhgef2	rho/rac guanine nucleotide exchange factor (GEF) 2	0.9669	0.6099	0.06	0.4689	No	0.9585	0.67	0.218	0.61	No	No
93633_at	Sergef	secretion regulating guanine nucleotide exchange factor	1.0777	0.0547	0.0016	0.7546	No	0.7943	0.271	0.37	0.648	No	No
93634_at	Fbxw11	F-box and WD-40 domain protein 11	0.9788	0.7993	0.0635	0.5992	No	0.956	0.445	0.0065	0.588	No	No
93635_at	Kif3c	kinesin family member 3C	0.9793	0.7259	0.0761	0.3299	No	0.8988	0.0791	0.144	0.286	No	No
93636_at	Rttn	rotatin	1.1368	0.0325	0.0021	0.0323	No	1.1846	0.0029	0.0029	0.0012	No	No
93637_at	Cd5	CD5 antigen	1.0111	0.8494	0.1877	0.9558	No	0.8976	0.0327	0.0285	0.41	No	No
93638_s_at	Igl-V1	immunoglobulin lambda chain, variable 1	1.0302	0.6691	0.0789	0.8271	No	1.1044	0.0829	0.0479	0.0787	No	No
93639_r_at	Igl-V1	immunoglobulin lambda chain, variable 1	0.9582	0.4651	0.2014	0.9995	No	0.9132	0.14	0.0252	0.196	No	No
93640_s_at	Tex21	testis expressed gene 21	1.0924	0.0964	0.9271	0.4032	No	1.6874	0.0078	0.0809	0.195	Yes	No
93642_at	Gdf1 /// Lass1	growth differentiation factor 1 /// longevity assurance homolog 1 (S. cerevisiae)	0.9962	0.9138	0.0118	0.5067	No	1.0442	0.428	0.0556	0.702	No	No
93643_at	Lhx9	LIM homeobox protein 9	1.0168	0.71	0.4174	0.7719	No	1.0064	0.96	0.647	0.259	No	No
93644_at	Lhx9	LIM homeobox protein 9	0.9759	0.7834	0.7002	0.5066	No	1.0984	0.258	0.0806	0.16	No	No
93645_at	Rgs7	regulator of G protein signaling 7	1.1234	0.0478	0.1137	0.0369	No	1.2064	0.0383	0.994	0.0616	No	No
93646_at	Ptk9	protein tyrosine kinase 9	0.9783	0.5013	0.0921	0.0631	No	1.1009	0.245	0.449	0.0092	No	No
93647_at	Gmip	Gem-interacting protein	0.8844	0.263	0.5038	0.2629	No	0.929	0.236	0.569	0.135	No	No
93648_at	Prkcc	protein kinase C, gamma	0.9734	0.4308	0.5467	0.9968	No	1.0161	0.805	0.453	0.749	No	No
93649_f_at	MP4	proline rich protein MP4	0.9798	0.7183	0.2765	0.5541	No	0.9658	0.576	0.984	0.987	No	No
93650_i_at	Prh1	proline rich protein HaeIII subfamily 1	0.9785	0.6281	0.2185	0.7962	No	1.0011	0.995	0.0167	0.233	No	No
93651_r_at	Prh1	proline rich protein HaeIII subfamily 1	0.9571	0.2804	0.0048	0.4537	No	0.9077	0.0905	0.105	0.098	No	No
93652_i_at	Vamp1	vesicle-associated membrane protein 1	0.9961	0.9186	0.004	0.1863	No	1.0494	0.0979	0.0012	0.0542	No	No
93653_r_at	Vamp1	vesicle-associated membrane protein 1	1.0632	0.2105	0.1216	0.2158	No	1.1172	0.241	0.731	0.142	No	No
93654_at	Fgf12	fibroblast growth factor 12	0.8858	0.0111	0.0045	0.0795	No	1.0026	0.962	0.0661	0.932	No	No
93655_at	Usf1	upstream transcription factor 1	1.1184	0.0598	0.1596	0.0186	No	1.0504	0.438	0.0833	0.242	No	No
93656_g_at	Usf1	upstream transcription factor 1	0.9948	0.8087	0.0021	0.4814	No	1.0403	0.348	0.584	0.015	No	No
93657_at	Spib	Spi-B transcription factor (Spi-1/PU.1 related)	0.9606	0.251	0.0883	0.0878	No	1.0508	0.17	0.379	0.145	No	No
93658_at	Ptpn20	protein tyrosine phosphatase, non-receptor type 20	1.0976	0.0132	0.0043	0.0258	No	1.2006	0.853	0.191	0.367	No	No
93659_at	Camk2a	calcium/calmodulin-dependent protein kinase II alpha	0.9511	0.7049	0.1032	0.3068	No	1.0667	0.366	0.0042	0.228	No	No
93660_at	Camk2a	calcium/calmodulin-dependent protein kinase II alpha	1.0139	0.723	0.0023	0.0765	No	0.9844	0.743	0.0001	0.004	No	No
93661_at	Zap70	zeta-chain (TCR) associated protein kinase	0.8705	0.048	0.3877	0.0364	No	0.8743	0.114	0.291	0.0977	No	No
93662_s_at	Zap70	zeta-chain (TCR) associated protein kinase	1.025	0.7079	0.9024	0.5299	No	0.9771	0.611	0.0035	0.0754	No	No
93663_r_at	Zap70	zeta-chain (TCR) associated protein kinase	1.0059	0.915	0.1715	0.6571	No	1.0173	0.388	0.0145	0.119	No	No
93664_at	Atp1b2	ATPase, Na+/K+ transporting, beta 2 polypeptide	0.9105	0.1011	0.6269	0.2409	No	0.9942	0.862	0.265	0.0285	No	No
93665_at	---	---	0.9314	0.3942	0.1522	0.5569	No	0.9834	0.766	0.0318	0.785	No	No
93666_at	Lmo2	LIM domain only 2	1.01	0.8149	0.1949	0.8244	No	1.1059	0.0577	0.141	0.366	No	No
93667_at	Fbxw7	F-box and WD-40 domain protein 7, archipelago homolog (Drosophila)	1.0085	0.68	0.0003	0.1927	No	1.1006	0.0432	0.0007	0.244	No	No
93668_at	Og9x	OG9 homeobox gene	1.0462	0.0188	0.0025	0.8122	No	1.051	0.207	0.28	0.766	No	No
93669_f_at	Sox11	SRY-box containing gene 11	1.0648	0.5648	0.0005	0.6203	No	1.1002	0.332	0.0001	0.275	No	No
93670_at	Erf	Ets2 repressor factor	0.8913	0.0351	0.0011	0.19	No	0.9492	0.328	0.589	0.973	No	No
93671_at	Erf	Ets2 repressor factor	0.988	0.6402	0.0015	0.0571	No	0.9831	0.84	0.826	0.89	No	No
93672_at	Eif2ak2	eukaryotic translation initiation factor 2-alpha kinase 2	1.0113	0.904	0.4413	0.3404	No	1.0365	0.451	0.0086	0.123	No	No
93673_at	Nrtn	neurturin	1.0198	0.4607	0.0007	0.0141	No	0.9921	0.672	0.0428	0.0166	No	No
93674_at	Fgd1	FYVE, RhoGEF and PH domain containing 1	1.1467	0.1832	0.244	0.8521	No	1.1036	0.0652	0.25	0.959	No	No
93675_at	Mak	male germ cell-associated kinase	0.9939	0.821	0.0054	0.805	No	0.9999	0.999	0.877	0.247	No	No
93676_at	Rad51ap1	RAD51 associated protein 1	1.0199	0.6929	0.3695	0.2974	No	1.0536	0.324	0.678	0.997	No	No
93677_at	Klrd1	killer cell lectin-like receptor, subfamily D, member 1	0.9895	0.8544	0.4761	0.1509	No	1.0699	0.209	0.458	0.748	No	No
93678_s_at	Klrk1	killer cell lectin-like receptor subfamily K, member 1	0.9653	0.316	0.0217	0.0725	No	0.4724	0.128	0.252	0.5	No	No
93679_at	Klrk1	killer cell lectin-like receptor subfamily K, member 1	1.062	0.2535	0.054	0.7207	No	1.0251	0.745	0.535	0.982	No	No
93680_at	Stk10	serine/threonine kinase 10	1.0759	0.3006	0.2172	0.1403	No	0.9476	0.346	0.0576	0.801	No	No
93681_at	Fzd2	frizzled homolog 2 (Drosophila)	1.0832	0.0308	0.0231	0.0557	No	1.089	0.0332	0.0028	0.24	No	No
93682_at	Ldb2	LIM domain binding 2	0.9694	0.2256	0.0309	0.4169	No	1.0488	0.296	0.521	0.465	No	No
93683_at	Rag1	recombination activating gene 1	1.0699	0.3947	0.4004	0.3509	No	1.0537	0.462	0.541	0.35	No	No
93685_at	1810013L24 Rik	RIKEN cDNA 1810013L24 gene	1.0214	0.7301	0.2083	0.7623	No	1.2113	0.0847	0.0748	0.21	No	No
93686_s_at	Cit	citron	0.9162	0.3281	0.4174	0.1011	No	0.8488	0.483	0.0467	0.0446	No	No
93687_r_at	Cit	citron	1.0216	0.6091	0.403	0.5792	No	1.1048	0.155	0.0478	0.221	No	No
93688_at	Cmah	cytidine monophospho-N-acetylneuraminic acid hydroxylase	0.9836	0.7339	0.1822	0.1861	No	1.0627	0.377	0.971	0.948	No	No
93689_at	Emid1	EMI domain containing 1	1.0436	0.2215	0.0035	0.5017	No	0.9683	0.456	0.0067	0.502	No	No
93690_at	Grb10	growth factor receptor bound protein 10	0.8667	0.0114	0.0031	0.2402	No	0.9339	0.292	0.78	0.948	No	No
93691_s_at	Grb10	growth factor receptor bound protein 10	0.9236	0.2862	0.9864	0.8975	No	0.931	0.305	0.494	0.332	No	No
93692_f_at	Ambn	ameloblastin	1.0712	0.3657	0.1878	0.447	No	1.1476	0.159	0.258	0.584	No	No
93693_at	Hmbox1	homeobox containing 1	0.9669	0.4432	0.0022	0.2093	No	0.9382	0.0574	0.0242	0.785	No	No
93694_at	Per2	period homolog 2 (Drosophila)	0.9712	0.424	0.0021	0.3495	No	0.9326	0.253	0.0286	0.24	No	No
93695_at	Adh7	alcohol dehydrogenase 7 (class IV), mu or sigma polypeptide	1.0008	0.9856	0.6834	0.5922	No	0.9951	0.901	0.377	0.593	No	No
93696_at	Nr1i2	nuclear receptor subfamily 1, group I, member 2	0.9834	0.7421	0.0556	0.131	No	0.9587	0.388	0.448	0.255	No	No
93697_at	Cbx4	chromobox homolog 4 (Drosophila Pc class)	0.9841	0.7424	0.0129	0.1525	No	1.0066	0.896	0.0084	0.737	No	No
93698_at	Sox13	SRY-box containing gene 13	1.0148	0.5537	0.0179	0.3078	No	0.986	0.43	0.0142	0.121	No	No

93699_at	Polg2	polymerase (DNA directed), gamma 2, accessory subunit	0.9373	0.2165	0.2643	0.1512	No	1.0232	0.398	0.0222	0.0098	No	No
93700_at	Arf3	ADP-ribosylation factor 3	1.0622	0.0437	0.0982	0.0212	No	0.9843	0.596	0.529	0.799	No	No
93701_at	Smarca5 /// 9230115A19 Rik /// LOC675704	SWI/SNF related, matrix associated, actin dependent regulator of chromatin, subfamily a, member 5 /// RIKEN cDNA 9230115A19 gene /// similar to SWI/SNF related, matrix associated, actin dependent regulator of chromatin, subfamily a, member 5	0.9348	0.2638	0.1527	0.5462	No	0.9432	0.0396	0.0286	0.0269	No	No
93702_at	Ptpn23	protein tyrosine phosphatase, non-receptor type 23	1.0737	0.0596	0.0017	0.2009	No	0.9964	0.919	0.237	0.641	No	No
93703_at	Cldn6	claudin 6	1.0996	0.0108	0.0365	0.1679	No	1.1142	0.0133	0.0619	0.105	No	No
93704_at	Foxf1a	forkhead box F1a	1.0005	0.9924	0.3355	0.3966	No	1.146	0.136	0.226	0.101	No	No
93705_at	Chrnb1	cholinergic receptor, nicotinic, beta polypeptide 1 (muscle)	1.0202	0.3184	0.02	0.2394	No	1.0169	0.85	0.155	0.123	No	No
93706_at	Ikbkb	inhibitor of kappaB kinase beta	0.9323	0.2174	0.0908	0.7962	No	1.0009	0.974	0.175	0.186	No	No
93707_f_at	G630024C07Rik	RIKEN cDNA G630024C07 gene	0.9684	0.546	0.0479	0.2348	No	1.0661	0.216	0.0297	0.31	No	No
93708_at	Pias3	protein inhibitor of activated STAT 3	0.9228	0.4148	0.2382	0.8932	No	1.0434	0.182	0.0001	0.406	No	No
93709_at	Tmprss8	transmembrane protease, serine 8 (intestinal)	1.0166	0.6852	0.1675	0.3097	No	0.9907	0.707	0.296	0.0455	No	No
93710_at	Krtap5-1	keratin associated protein 5-1	0.9856	0.7628	0.0843	0.7921	No	0.9748	0.55	0.0041	0.761	No	No
93711_at	Sec23a	SEC23A (S. cerevisiae)	0.9596	0.4748	0.0243	0.5599	No	0.9353	0.288	0.0077	0.653	No	No
93712_at	Ccnt1	cyclin T1	1.0194	0.6344	0.0043	0.8599	No	0.9542	0.262	0.0012	0.317	No	No
93713_at	Shc1	src homology 2 domain-containing transforming protein C1	0.9537	0.574	0.0642	0.8297	No	1.0219	0.454	0.0019	0.419	No	No
93714_f_at	LOC630499 /// LOC633617 /// LOC636948	similar to H-2 class I histocompatibility antigen, D-K alpha chain precursor (H-2D(K)) /// similar to H-2 class I histocompatibility antigen, D-K alpha chain precursor (H-2D(K)) /// similar to H-2 class I histocompatibility antigen, D-B alpha chain precursor (H-2D(B))	0.9989	0.9834	0.2029	0.6992	No	0.991	0.758	0.0166	0.648	No	No
93715_at	Mpz	myelin protein zero	1.0656	0.3155	0.8532	0.7714	No	1.0336	0.672	0.425	0.617	No	No
93716_at	Trim46	tripartite motif protein 46	1.0017	0.9788	0.9128	0.7527	No	0.9846	0.758	0.0163	0.917	No	No
93717_at	Ccl12	chemokine (C-C motif) ligand 12	0.9923	0.8933	0.8506	0.1372	No	1.0223	0.309	0.0001	0.662	No	No
93718_at	Rab23	RAB23, member RAS oncogene family	0.9168	0.2524	0.0404	0.5176	No	0.9911	0.908	0.0067	0.772	No	No
93719_at	Ccrl1	chemokine (C-C motif) receptor-like 1	0.977	0.7016	0.06	0.1525	No	0.9821	0.886	0.0012	0.22	No	No
93720_at	Agpat1	1-acylglycerol-3-phosphate O-acyltransferase 1 (lysophosphatidic acid acyltransferase, alpha)	1.2037	0.0172	0.0017	0.0153	No	1.0668	0.385	0.379	0.0473	No	No
93721_at	Cap1	CAP, adenylate cyclase-associated protein 1 (yeast)	1.0138	0.5542	0.0101	0.0153	No	1.0194	0.688	0.367	0.0785	No	No
93722_at	Ensa	endosulfine alpha	0.9297	0.3002	0.0073	0.6311	No	1.0219	0.696	0.0012	0.26	No	No
93723_at	Ror2	receptor tyrosine kinase-like orphan receptor 2	0.9455	0.2268	0.0051	0.146	No	0.9447	0.053	0.0229	0.516	No	No
93724_at	Ror2	receptor tyrosine kinase-like orphan receptor 2	1.0199	0.7877	0.0262	0.7712	No	1.0283	0.461	0.0033	0.281	No	No
93725_at	Cxadr	coxsackievirus and adenovirus receptor	0.9685	0.5575	0.2258	0.5837	No	1.0624	0.0615	0.003	0.0254	No	No
93727_at	Gspt1	G1 to S phase transition 1	0.9198	0.0199	0.2729	0.0819	No	0.9866	0.578	0.181	0.941	No	No
93728_at	Tsc22d1	TSC22 domain family, member 1	0.9549	0.4483	0.0378	0.9358	No	0.9222	0.322	0.544	0.911	No	No
93729_at	---	---	0.9419	0.4317	0.0449	0.6756	No	0.9876	0.658	0.0024	0.263	No	No
93730_at	Rps15a	ribosomal protein S15a	1.036	0.1663	0.0699	0.0965	No	0.9939	0.775	0.0001	0.0061	No	No
93731_at	Fkbp9	FK506 binding protein 9	1.0995	0.0714	0.03	0.7758	No	1.0626	0.155	0.012	0.152	No	No
93732_f_at	Gipc1	GIPC PDZ domain containing family, member 1	1.014	0.6273	0.0024	0.3168	No	1.021	0.404	0.0023	0.257	No	No
93733_r_at	Gipc1	GIPC PDZ domain containing family, member 1	1.0108	0.8682	0.0019	0.5011	No	1.0364	0.515	0.001	0.627	No	No
93734_i_at	Psmc3	proteasome (prosome, macropain) 26S subunit, ATPase 3	1.0681	0.2716	0.0133	0.7443	No	1.0193	0.656	0.0287	0.966	No	No
93735_f_at	Psmc3	proteasome (prosome, macropain) 26S subunit, ATPase 3	0.9596	0.0966	0.3792	0.001	No	1.0776	0.201	0.0481	0.167	No	No
93736_at	Tcn2	transcobalamin 2	0.918	0.0427	0.0583	0.1165	No	0.9404	0.0092	0.0002	0.114	Yes	Yes
93737_at	Eif2ak1	eukaryotic translation initiation factor 2 alpha kinase 1	0.9547	0.4691	0.9223	0.2352	No	0.9968	0.939	0.52	0.378	No	No
93738_at	Slc2a1	solute carrier family 2 (facilitated glucose transporter), member 1	0.9618	0.596	0.2651	0.3464	No	0.9189	0.0026	0.0001	0.903	Yes	No
93740_at	Ybx1	Y box protein 1	1.0871	0.0099	0.0017	0.035	Yes	1.0536	0.0377	0.0124	0.0178	No	Yes
93741_at	Nmd3	NMD3 homolog (S. cerevisiae)	1.138	0.0545	0.1122	0.1618	No	1.121	0.0177	0.0018	0.0184	No	No
93742_at	5730449L18 Rik	RIKEN cDNA 5730449L18 gene	0.9707	0.3574	0.0352	0.6368	No	0.8754	0.0163	0.346	0.0874	No	No
93743_at	Hsbp1	heat shock factor binding protein 1	0.9921	0.8299	0.024	0.9674	No	0.9724	0.329	0.0704	0.603	No	No
93744_at	Calm4	calmodulin 4	1.0149	0.7332	0.3182	0.7169	No	0.9517	0.647	0.0116	0.263	No	No
93747_at	Ptplad1	protein tyrosine phosphatase-like A domain containing 1	1.0402	0.3646	0.0036	0.8818	No	1.0253	0.634	0.0137	0.506	No	No
93748_at	Grinl1a	glutamate receptor, ionotropic, N-methyl D-aspartate-like 1A	0.9927	0.8592	0.1186	0.4841	No	1.0047	0.802	0.0026	0.443	No	No
93749_at	Maoa	monoamine oxidase A	1.0163	0.7003	0.0118	0.1421	No	0.967	0.291	0.0009	0.216	No	No
93750_at	Gsn	gelsolin	0.9995	0.9907	0.0141	0.2772	No	0.9684	0.429	0.0088	0.88	No	No
93751_at	Usp19	ubiquitin specific peptidase 19	0.967	0.4852	0.0375	0.8406	No	1.0485	0.25	0.108	0.641	No	No
93752_at	Iars	isoleucine-tRNA synthetase	0.9294	0.0505	0.0005	0.1007	No	0.9425	0.0374	0.0002	0.0086	No	No
93753_at	Litaf	LPS-induced TN factor	0.9439	0.2631	0.2556	0.8113	No	0.9986	0.97	0.0011	0.318	No	No
93754_at	Ech1	enoyl coenzyme A hydratase 1, peroxisomal	0.9786	0.5864	0.0091	0.0113	No	0.9713	0.849	0.0041	0.0421	No	No
93755_at	Retnlb	resistin like beta	1.1769	0.0464	0.1689	0.0688	No	1.0469	0.385	0.0014	0.425	No	No
93757_at	Srpr	signal recognition particle receptor ('docking protein')	0.9206	0.0976	0.0193	0.3251	No	0.9814	0.624	0.141	0.146	No	No
93758_at	Incenp	inner centromere protein	1.0206	0.5045	0.0059	0.5223	No	0.944	0.145	0.0001	0.44	No	No
93760_at	1200020A08 Rik	RIKEN cDNA 1200020A08 gene	0.9913	0.6712	0.2612	0.0076	No	0.9618	0.0535	0.0063	0.0463	No	No
93761_at	Hnrpu	heterogeneous nuclear ribonucleoprotein U	1.1472	0.0124	0.4731	0.5861	No	1.0653	0.402	0.0727	0.354	No	No
93762_at	Ppp2r4	protein phosphatase 2A, regulatory subunit B (PR 53)	1.0762	0.0851	0.0044	0.0444	No	1.0593	0.107	0.0138	0.182	No	No
93764_at	Ndufa13	NADH dehydrogenase (ubiquinone) 1 alpha subcomplex, 13	1.0024	0.9631	0.1869	0.0358	No	1.1522	0.0667	0.303	0.583	No	No
93765_at	Dcpp	demilune cell and parotid protein	0.8454	0.0257	0.0022	0.0134	No	0.5496	0.0033	0.001	0.0141	Yes	Yes
93766_at	Rgn	regucalcin	0.8993	0.0627	0.0514	0.7014	No	0.7898	0.0545	0.0103	0.949	No	No
93767_i_at	2700059D21 Rik	RIKEN cDNA 2700059D21 gene	1.0487	0.2709	0.8352	0.6288	No	1.0833	0.132	0.367	0.764	No	No
93768_f_at	2700059D21 Rik	RIKEN cDNA 2700059D21 gene	1.0232	0.6736	0.0007	0.5362	No	1.0186	0.943	0.0006	0.289	No	No
93769_at	Necap2	NECAP endocytosis associated 2	0.9613	0.1804	0.0004	0.0156	No	1.0052	0.962	0.88	0.534	No	No
93770_at	Cyp3a11	cytochrome P450, family 3, subfamily a, polypeptide 11	0.9887	0.6283	0.0037	0.0061	No	0.9488	0.512	0.875	0.0787	No	No
93771_at	Gjb1	gap junction membrane channel protein beta 1	0.9779	0.721	0.097	0.5944	No	0.9838	0.0351	0	0.674	No	No
93772_i_at	Zfp265	zinc finger protein 265	0.9393	0.4315	0.0063	0.4133	No	0.9233	0.345	0.0099	0.629	No	No
93773_f_at	Zfp265	zinc finger protein 265	1.2064	0.098	0.0021	0.8559	No	1.173	0.0697	0.0049	0.369	No	No
93774_at	2610524G07 Rik	RIKEN cDNA 2610524G07 gene	0.9459	0.1741	0.1122	0.3718	No	0.9811	0.951	0	0.139	No	No
93775_at	D12Ertd647e	DNA segment, Chr 12, ERATO Doi 647, expressed	1.0147	0.7598	0.0975	0.7242	No	1.0638	0.225	0.132	0.375	No	No
93776_at	1500001L15 Rik	RIKEN cDNA 1500001L15 gene	1.028	0.6152	0.5105	0.3686	No	0.9475	0.362	0.0023	0.326	No	No
93777_at	Golph3l	golgi phosphoprotein 3-like	0.9128	0.0196	0.1098	0.5969	No	0.9668	0.43	0.143	0.621	No	No
93778_at	Csprs	component of Sp100-rs	0.9886	0.8851	0.2783	0.6853	No	0.9943	0.95	0.576	0.0719	No	No
93779_at	C130026I21 Rik /// LOC673329 /// LOC673370	RIKEN cNDA C130026I21 gene /// similar to SP140 nuclear body protein (predicted) /// similar to SP140 nuclear body protein (predicted)	0.9839	0.759	0.1417	0.7578	No	1.0074	0.783	0.167	0.698	No	No
93780_at	Them2	thioesterase superfamily member 2	0.9429	0.3281	0.4197	0.2649	No	0.9616	0.274	0.0469	0.0653	No	No
93781_at	Miox	myo-inositol oxygenase	0.9988	0.9875	0.4448	0.6699	No	1.0334	0.565	0.542	0.77	No	No
93782_at	Rnf4	ring finger protein 4	1.0414	0.176	0.0005	0.2961	No	1.0338	0.275	0.0004	0.243	No	No
93783_at	Ela1	elastase 1, pancreatic	1.0349	0.3563	0.5562	0.1063	No	1.0047	0.829	0.268	0.0478	No	No
93784_at	Cfdp1	craniofacial development protein 1	1.0001	0.9968	0.1202	0.539	No	0.9883	0.826	0.791	0.765	No	No
93785_at	Folr1	folate receptor 1 (adult)	1.0801	0.0042	0.0548	0.6946	Yes	0.9737	0.486	0.0587	0.775	No	No
93786_i_at	Mrpl18 /// LOC433550	mitochondrial ribosomal protein L18 /// similar to mitochondrial ribosomal protein L18	1.2125	0.1039	0.0125	0.6182	No	1.1592	0.122	0.0089	0.611	No	No
93787_f_at	Mrpl18	mitochondrial ribosomal protein L18	1.0557	0.0846	0	0.0177	No	1.0705	0.0935	0	0.0056	No	No
93788_at	Cox4i1	cytochrome c oxidase subunit IV isoform 1	1.0633	0.2161	0.0008	0.1924	No	1.0628	0.0198	0	0.0003	No	No
93789_s_at	Sin3b	transcriptional regulator, SIN3B (yeast)	1.0106	0.6967	0.0196	0.0213	No	0.9661	0.133	0.0083	0.0073	No	No
93790_at	Sin3b	transcriptional regulator, SIN3B (yeast)	0.9672	0.6199	0.1921	0.5564	No	0.9305	0.0563	0.236	0.0547	No	No
93793_at	Lasp1	LIM and SH3 protein 1	1.0921	0.0681	0.0008	0.0912	No	1.0449	0.0033	0	0.0034	No	No

93794_at	Appbp1	amyloid beta precursor protein binding protein 1	1.0781	0.2394	0.5598	0.3985	No	1.0849	0.232	0.886	0.468	No	No
93795_at	Itpa	inosine triphosphatase (nucleoside triphosphate pyrophosphatase)	1.1057	0.0411	0.0191	0.8672	No	0.9121	0.0523	0.536	0.0101	No	No
93796_at	Atp1a1	ATPase, Na+/K+ transporting, alpha 1 polypeptide	0.9426	0.2278	0.6764	0.2448	No	0.972	0.76	0.0104	0.917	No	No
93797_g_at	Atp1a1	ATPase, Na+/K+ transporting, alpha 1 polypeptide	0.9205	0.07	0.1202	0.2578	No	0.9267	0.0572	0.0041	0.0571	No	No
93798_at	Atp1a1	ATPase, Na+/K+ transporting, alpha 1 polypeptide	0.9744	0.7402	0.0418	0.3364	No	0.9374	0.254	0.0013	0.463	No	No
93799_at	AI316787	expressed sequence AI316787	0.9914	0.7839	0.8952	0.8369	No	1.0016	0.957	0.435	0.685	No	No
93800_f_at	Krt1-c29	keratin complex-1, acidic, gene C29	0.9587	0.4633	0.2134	0.3953	No	1.0169	0.81	0.787	0.669	No	No
93801_at	4631426H08Rik	RIKEN cDNA 4631426H08 gene	1.0199	0.6639	0.9911	0.6329	No	1.1965	0.123	0.0278	0.201	No	No
93802_at	Mtdh	Metadherin	0.8557	0.1002	0.8616	0.1286	No	1.0027	0.827	0.0052	0.326	No	No
93803_at	Psme3	proteaseome (prosome, macropain) 28 subunit, 3	1.1613	0.0092	0.0003	0.0765	Yes	1.1342	0.145	0.0028	0.204	No	No
93804_at	Slc2a3	solute carrier family 2 (facilitated glucose transporter), member 3	1.0294	0.4507	0.0006	0.0862	No	1.0384	0.257	0.0019	0.006	No	No
93805_at	Ssna1	Sjogren's syndrome nuclear autoantigen 1	0.9657	0.3406	0.0009	0.1119	No	0.9794	0.771	0.0029	0.059	No	No
93806_at	Sh3bgrl	SH3-binding domain glutamic acid-rich protein like	1.0384	0.677	0.5106	0.3349	No	1.0017	0.98	0.376	0.302	No	No
93807_at	2310016E02Rik	RIKEN cDNA 2310016E02 gene	1.0087	0.8334	0.6644	0.1206	No	1.2002	0.131	0.501	0.0536	No	No
93808_at	Cbr2	carbonyl reductase 2	1.0191	0.8111	0.0664	0.4621	No	0.9296	0.224	0.64	0.436	No	No
93809_at	Aup1	ancient ubiquitous protein	1.0118	0.6128	0.0002	0.3463	No	1.0151	0.481	0.0001	0.776	No	No
93810_at	Ctsd	cathepsin D	0.9294	0.0365	0.0014	0.0259	No	0.9395	0.0682	0.701	0.0945	No	No
93812_at	Clns1a	chloride channel, nucleotide-sensitive, 1A	1.03	0.4444	0.0997	0.5973	No	0.9872	0.509	0.0006	0.085	No	No
93815_at	Chchd3	coiled-coil-helix-coiled-coil-helix domain containing 3	1.0783	0.2649	0.1903	0.1319	No	1.0351	0.385	0.489	0.0092	No	No
93817_at	Tmed2	transmembrane emp24 domain trafficking protein 2	0.9658	0.3042	0.4242	0.7127	No	1.012	0.947	0.327	0.39	No	No
93818_g_at	Tmed2	transmembrane emp24 domain trafficking protein 2	1.0814	0.1084	0.0936	0.6144	No	1.003	0.991	0.0369	0.466	No	No
93819_at	Tmed2	transmembrane emp24 domain trafficking protein 2	1.0476	0.4197	0.2973	0.2979	No	1.0278	0.601	0.352	0.209	No	No
93820_at	Cox7a2	cytochrome c oxidase, subunit VIIa 2	1.0127	0.8122	0.4574	0.2521	No	0.9958	0.986	0.0334	0.192	No	No
93821_at	Wwc2	WW, C2 and coiled-coil domain containing 2	1.0224	0.721	0.041	0.1844	No	0.8861	0.0067	0.134	0.0028	No	No
93822_at	Rpl37a	ribosomal protein L37a	0.9802	0.6629	0.0623	0.5337	No	0.9523	0.314	0.0072	0.995	No	No
93823_at	Ilf2	interleukin enhancer binding factor 2	1.0438	0.6797	0.475	0.4797	No	0.9996	0.938	0.192	0.862	No	No
93824_at	Cps1	carbamoyl-phosphate synthetase 1	1.0319	0.1536	0.0017	0.0984	No	1.1442	0.0818	0.0205	0.364	No	No
93826_at	Ppp2r5a	protein phosphatase 2, regulatory subunit B (B56), alpha isoform	1.0064	0.9411	0.6046	0.2651	No	0.9579	0.634	0.776	0.0492	No	No
93827_at	Tdo2	tryptophan 2,3-dioxygenase	1.0026	0.9514	0.1398	0.6698	No	1.0726	0.341	0.0764	0.641	No	No
93829_at	Rod1	ROD1 regulator of differentiation 1 (S. pombe)	1.0247	0.7374	0.0297	0.4676	No	0.8835	0.35	0.39	0.354	No	No
93830_at	Nono	non-POU-domain-containing, octamer binding protein	1.1264	0.0934	0.3318	0.5325	No	1.092	0.147	0.159	0.781	No	No
93831_at	Nono	non-POU-domain-containing, octamer binding protein	0.9407	0.4797	0.1653	0.814	No	0.9348	0.231	0.142	0.986	No	No
93832_at	Spag7	sperm associated antigen 7	1.0246	0.2634	0.0054	0.0593	No	1.037	0.379	0.0647	0.0927	No	No
93833_s_at	Hist1h2bc	histone 1, H2bc	0.9223	0.0944	0.017	0.0112	No	0.8745	0.14	0.449	0.0416	No	No
93834_at	Hist1h2bp	histone 1, H2bp	1.0992	0.187	0.1796	0.22	No	1.0812	0.283	0.6	0.0967	No	No
93835_at	Fuca1	fucosidase, alpha-L- 1, tissue	1.0053	0.9003	0.0628	0.295	No	0.9969	0.95	0.0136	0.0197	No	No
93836_at	Bnip3	BCL2/adenovirus E1B interacting protein 1, NIP3	0.8674	0.1076	0.6572	0.9175	No	0.9085	0.168	0.529	0.847	No	No
93837_at	Kng1	kininogen 1	0.9559	0.1713	0.0162	0.0724	No	0.9502	0.411	0.0087	0.369	No	No
93838_at	2700038C09Rik	RIKEN cDNA 2700038C09 gene	1.0842	0.0177	0.0001	0.0189	No	1.0861	0.0178	0.0001	0.0784	No	No
93839_at	Rtn3	reticulon 3	0.9961	0.9097	0.0314	0.8814	No	0.9885	0.828	0.822	0.262	No	No
93840_at	Apom	apolipoprotein M	0.8864	0.2298	0.3485	0.9583	No	1.0077	0.859	0.327	0.511	No	No
93841_at	Eif2a	eukaryotic translation initiation factor 2a	0.9664	0.3724	0.0478	0.588	No	0.9362	0.296	0.0274	0.198	No	No
93842_at	Dap	death-associated protein	1.0326	0.2768	0.0001	0.0352	No	1.03	0.301	0.0001	0.243	No	No
93843_at	Dhrs1	dehydrogenase/reductase (SDR family) member 1	1.0048	0.803	0.657	0.132	No	1.047	0.344	0.348	0.248	No	No
93844_at	Uqcrq	ubiquinol-cytochrome c reductase, complex III subunit VII	1.0337	0.0651	0.0003	0.0004	No	1.0133	0.163	0.0003	0.0008	No	No
93845_at	Abcf2	ATP-binding cassette, sub-family F (GCN20), member 2	0.9505	0.1872	0.0019	0.0406	No	1.0304	0.286	0.0007	0.334	No	No
93846_at	Snn	stannin	1.0496	0.2622	0.4179	0.9855	No	1.0506	0.522	0.0662	0.75	No	No
93847_at	Ptprd	protein tyrosine phosphatase, receptor type, D	0.9909	0.9106	0.3121	0.9595	No	0.968	0.661	0.393	0.656	No	No
93848_at	Map4k3	Mitogen-activated protein kinase kinase kinase kinase 3	1.0615	0.2634	0.7125	0.4851	No	1.2627	0.0981	0.27	0.729	No	No
93849_at	Tpk1	thiamin pyrophosphokinase	0.9652	0.4904	0.0394	0.2419	No	1.1663	0.234	0.0263	0.186	No	No
93850_at	Iqgap1	IQ motif containing GTPase activating protein 1	0.9498	0.3293	0.1784	0.3754	No	0.9179	0.0393	0.0007	0.455	No	No
93851_at	Rabggta	Rab geranylgeranyl transferase, a subunit	1.0344	0.4613	0.0518	0.802	No	0.9937	0.875	0.0374	0.822	No	No
93852_at	Mef2a	myocyte enhancer factor 2A	0.9504	0.3778	0.0228	0.7599	No	0.9236	0.207	0.0278	0.512	No	No
93853_at	Dnajb4	DnaJ (Hsp40) homolog, subfamily B, member 4	0.9824	0.6801	0.179	0.5021	No	0.9747	0.62	0.585	0.164	No	No
93855_at	Rem1	rad and gem related GTP binding protein 1	1	0.9993	0.1887	0.5456	No	0.9637	0.0589	0.0108	0.111	No	No
93856_at	Wt1	Wilms tumor homolog	0.9574	0.1025	0.905	0.2289	No	0.9899	0.838	0.768	0.923	No	No
93857_at	Kpna3	karyopherin (importin) alpha 3	1.0094	0.8307	0.726	0.6375	No	0.9677	0.831	0.0308	0.367	No	No
93858_at	Cxcl10	chemokine (C-X-C motif) ligand 10	0.9436	0.4669	0.3516	0.6332	No	1.0278	0.556	0.0754	0.0708	No	No
93859_at	Mtif2	mitochondrial translational initiation factor 2	1.0042	0.9491	0.1113	0.1431	No	0.9667	0.492	0.167	0.018	No	No
93860_i_at	Ctse /// LOC622147 /// Env /// LOC664798 /// LOC665955 /// LOC666402 /// LOC666404 /// LOC667328 /// LOC667538 /// LOC668227 /// LOC668269 /// LOC668639 /// LOC668769 /// LOC668842 /// LOC669098 ///	cathepsin E /// similar to melanoma antigen /// envelope polyprotein /// envelope polyprotein /// similar to melanoma antigen /// similar to melanoma antigen /// similar to melanoma antigen /// similar to melanoma antigen /// similar to melanoma antigen /// similar to melanoma antigen /// similar to melanoma antigen /// similar to melanoma antigen /// similar to melanoma antigen /// similar to melanoma antigen /// similar to melanoma antigen /// similar to melanoma antigen /// similar to melanoma antigen /// hypothetical protein LOC670510 /// similar to melanoma antigen /// similar to melanoma antigen /// similar to melanoma antigen /// similar to melanoma antigen	1.0026	0.9497	0.393	0.0134	No	1.1676	0.115	0.0311	0.0249	No	No

93861_f_at	Ctse /// LOC622147 /// Env /// LOC628577 /// LOC664798 /// LOC665955 /// LOC666402 /// LOC666404 /// LOC667328 /// LOC667538 /// LOC668227 /// LOC668269 /// LOC668639 /// LOC668769 /// LOC668842 ///	cathepsin E /// similar to melanoma antigen /// envelope polyprotein /// similar to melanoma antigen /// envelope polyprotein /// similar to melanoma antigen /// similar to melanoma antigen /// similar to melanoma antigen /// similar to melanoma antigen /// similar to melanoma antigen /// similar to melanoma antigen /// similar to melanoma antigen /// similar to melanoma antigen /// similar to melanoma antigen /// similar to melanoma antigen /// similar to melanoma antigen /// similar to melanoma antigen /// similar to melanoma antigen /// similar to melanoma antigen /// hypothetical protein LOC670510 /// similar to melanoma antigen /// similar to melanoma antigen /// similar to melanoma antigen /// similar to melanoma antigen	0.9246	0.3894	0.2514	0.2389	No	1.0014	0.899	0.0736	0.141	No	No
93862_i_at	Defcr4	Defensin related cryptdin 4	1.0373	0.2925	0.0015	0.0318	No	1.0741	0.0538	0.0007	0.0082	No	No
93863_f_at	Defcr3 /// Defcr6 /// Defcr17 /// Defcr23 /// Defcr24	defensin related cryptdin 3 /// defensin related cryptdin 6 /// defensin related cryptdin 17 /// defensin-related cryptdin 23 /// defensin-related cryptdin 24	0.9826	0.768	0.5038	0.2997	No	1.1039	0.0027	0.0019	0.016	Yes	No
93864_s_at	Enah	enabled homolog (Drosophila)	0.9516	0.4286	0.0064	0.7905	No	0.8861	0.0896	0.0209	0.821	No	No
93865_s_at	H2-T10 /// H2-T22 /// H2-T9	histocompatibility 2, T region locus 10 /// histocompatibility 2, T region locus 22 /// histocompatibility 2, T region locus 9	0.9453	0.0982	0.0801	0.0336	No	0.9935	0.895	0.0425	0.558	No	No
93866_s_at	Mgp	matrix Gla protein	0.8626	0.0048	0.0001	0.4821	Yes	0.898	0.0144	0.0001	0.6	No	Yes
93867_at	Abcd4	ATP-binding cassette, sub-family D (ALD), member 4	0.8799	0.2314	0.6851	0.2757	No	1.0111	0.776	0.0056	0.884	No	No
93868_at	Nsdhl	NAD(P) dependent steroid dehydrogenase-like	1.0034	0.962	0.0709	0.1263	No	0.9832	0.897	0.0203	0.077	No	No
93869_s_at	Bcl2a1a /// Bcl2a1c /// Bcl2a1d	B-cell leukemia/lymphoma 2 related protein A1a /// B-cell leukemia/lymphoma 2 related protein A1c /// B-cell leukemia/lymphoma 2 related protein A1d	0.9326	0.1917	0.3715	0.6968	No	0.9423	0.382	0.617	0.333	No	No
93870_at	Braf	Braf transforming gene	0.9524	0.1925	0.1419	0.8919	No	0.9816	0.798	0.0318	0.772	No	No
93871_at	Il1rn	interleukin 1 receptor antagonist	0.9423	0.0544	0.7869	0.2307	No	0.9803	0.798	0.971	0.737	No	No
93872_at	Gfra1	glial cell line derived neurotrophic factor family receptor alpha 1	0.9807	0.684	0.0221	0.0709	No	1.0416	0.509	0.041	0.297	No	No
93873_s_at	Hoxa11os	homeo box A11, opposite strand transcript	0.967	0.4142	0.0191	0.1217	No	0.9496	0.441	0.18	0.0424	No	No
93874_s_at	Il11ra1 /// Il11ra2 /// LOC621887	interleukin 11 receptor, alpha chain 1 /// interleukin 11 receptor, alpha chain 2 /// similar to interleukin 11 receptor, alpha chain 2	1.0155	0.7276	0.0202	0.3317	No	1.0319	0.272	0.0029	0.121	No	No
93875_at	Hspa1a	heat shock protein 1A	0.9327	0.1962	0.0073	0.2994	No	0.9111	0.172	0.0017	0.94	No	No
93877_at	2210419D22Rik	RIKEN cDNA 2210419D22 gene	0.9742	0.6298	0.0319	0.0282	No	0.9638	0.454	0.0559	0.0496	No	No
93878_at	Mllt10	myeloid/lymphoid or mixed lineage-leukemia translocation to 10 homolog (Drosophila)	0.92	0.1923	0.7698	0.9731	No	1.168	0.0967	0.0201	0.386	No	No
93879_f_at	Defcr17	defensin related cryptdin 17	1.0724	0.3673	0.2339	0.1357	No	1.1871	0.0997	0.0691	0.0979	No	No
93880_at	Eomes	eomesodermin homolog (Xenopus laevis)	1.0115	0.8461	0.9594	0.547	No	1.0579	0.357	0.0138	0.3	No	No
93881_i_at	Tgoln1 /// Tgoln2	trans-golgi network protein /// trans-golgi network protein 2	1.003	0.8552	0.0924	0.0782	No	0.9306	0.125	0.355	0.332	No	No
93882_f_at	Tgoln1 /// Tgoln2	trans-golgi network protein /// trans-golgi network protein 2	0.9836	0.7551	0.9391	0.4597	No	0.9688	0.735	0.0665	0.611	No	No
93883_at	Plf /// Plf2 /// Mrpplf3 /// Mrpplf4 /// LOC630663 /// LOC666317 /// LOC672240 /// LOC676034	proliferin /// proliferin 2 /// mitogen regulated protein, proliferin 3 /// mitogen regulated protein, proliferin 4 /// similar to Proliferin 3 precursor (Mitogen-regulated protein 3) /// similar to mitogen regulated protein, proliferin 4 /// similar to mitogen regulated protein, proliferin 4 /// similar to mitogen regulated protein, proliferin 4	0.9373	0.3381	0.3418	0.8637	No	0.9463	0.236	0.0056	0.498	No	No
93884_at	Emcn	endomucin	0.9899	0.8275	0.0264	0.6809	No	1.1203	0.18	0.0483	0.764	No	No
93885_g_at	Emcn	endomucin	1.024	0.5258	0.0099	0.9206	No	1.0274	0.412	0.003	0.67	No	No
93886_at	Tollip	toll interacting protein	0.9902	0.8467	0.4313	0.8073	No	1.0177	0.798	0.867	0.582	No	No
93887_at	Mpdz	multiple PDZ domain protein	0.9597	0.3049	0.0041	0.1048	No	0.9614	0.562	0.0082	0.313	No	No
93888_at	Hoxb1	homeo box B1	0.9449	0.211	0.1044	0.6038	No	0.9802	0.669	0.294	1	No	No
93889_f_at	Hist1h2ba	histone 1, H2ba	0.956	0.3558	0.0008	0.0586	No	0.9164	0.0278	0.0003	0.0223	No	No
93890_at	Kifc2	kinesin family member C2	0.9354	0.1962	0.0038	0.2481	No	1.0274	0.705	0.0129	0.896	No	No
93891_at	Kifc2	kinesin family member C2	1.0139	0.7472	0.6466	0.1755	No	1.0125	0.747	0.254	0.442	No	No
93892_at	Cugbp2	CUG triplet repeat, RNA binding protein 2	0.9989	0.977	0.1363	0.7538	No	1.0462	0.603	0.0809	0.634	No	No
93893_f_at	Klra3	killer cell lectin-like receptor, subfamily A, member 3	1.047	0.5432	0.071	0.3985	No	1.0611	0.634	0.103	0.669	No	No
93894_f_at	Klra3	killer cell lectin-like receptor, subfamily A, member 3	0.9396	0.3174	0.0207	0.5141	No	0.8534	0.254	0.296	0.773	No	No
93895_s_at	Itpr1	inositol 1,4,5-triphosphate receptor 1	0.9373	0.1128	0.0137	0.0542	No	0.909	0.0656	0.605	0.19	No	No
93896_at	Ptprd	protein tyrosine phosphatase, receptor type, D	0.9332	0.0986	0.0383	0.0955	No	0.9653	0.474	0.0065	0.877	No	No
93897_at	Theg	testicular haploid expressed gene	0.9307	0.3327	0.0259	0.7997	No	0.8725	0.0779	0.0098	0.28	No	No
93898_at	Sgcb	sarcoglycan, beta (dystrophin-associated glycoprotein)	1.0319	0.5465	0.5234	0.6258	No	0.9328	0.272	0.659	0.879	No	No
93899_at	Rps6kb1	ribosomal protein S6 kinase, polypeptide 1	0.9921	0.8077	0.7808	0.8465	No	0.9957	0.904	0.176	0.0729	No	No
93900_at	Bat2	HLA-B associated transcript 2	0.9536	0.5391	0.2416	0.2388	No	1.0227	0.496	0.0015	0.293	No	No
93901_at	Hps1	Hermansky-Pudlak syndrome 1 homolog (human)	1.0113	0.8161	0.0975	0.6691	No	1.0098	0.796	0.0143	0.552	No	No
93902_at	Gab1	growth factor receptor bound protein 2-associated protein 1	0.9767	0.5857	0.0209	0.9874	No	0.9877	0.887	0.0082	0.416	No	No
93903_at	Acvr2b	activin receptor IIB	0.9699	0.4316	0.4505	0.6715	No	1.0248	0.449	0.0158	0.682	No	No
93904_f_at	Igh-V3609N /// Igk-V1 /// LOC238418 /// LOC629826 /// LOC630322 /// LOC630348 /// LOC633539 /// LOC668417 /// LOC668421 /// LOC674073 /// LOC674108 /// LOC674140 ///	immunoglobulin heavy chain (V3609N non-productive) /// immunoglobulin kappa chain variable 1 (V1) /// similar to Ig H-chain (VDJ-region) precursor /// similar to Ig heavy chain V region 102 precursor /// similar to Ig heavy chain V region B1-8/186-2 precursor /// similar to Ig heavy chain V region 102 precursor /// similar to Ig heavy chain V region 186-1 precursor /// similar to Ig heavy chain V region MOPC 47A /// similar to Ig heavy chain V region 186-1 precursor /// similar to Ig heavy chain V region 186-1 precursor /// similar to Ig heavy chain V region 102 precursor /// similar to Ig heavy chain V region 186-1 precursor	0.9864	0.8032	0.9878	0.8004	No	0.9974	0.952	0.763	0.941	No	No
93905_at	Cd80	CD80 antigen	0.9869	0.7802	0.0048	0.134	No	0.9634	0.687	0.25	0.637	No	No
93906_s_at	Cd80	CD80 antigen	1.0284	0.6682	0.8406	0.7285	No	1.1027	0.0501	0.264	0.667	No	No

93907_f_at	Ccrn4l /// Sgip1 /// LOC280487 /// Gag /// LOC639390	CCR4 carbon catabolite repression 4-like (S. cerevisiae) /// SH3-domain GRB2-like (endophilin) interacting protein 1 /// pol polyprotein /// Gag /// gag protein	0.984	0.802	0.1045	0.4192	No	0.9853	0.779	0.0048	0.799	No	No
93908_f_at	Ccrn4l /// Sgip1 /// LOC280487 /// Gag /// LOC639390	CCR4 carbon catabolite repression 4-like (S. cerevisiae) /// SH3-domain GRB2-like (endophilin) interacting protein 1 /// pol polyprotein /// Gag /// gag protein	0.8936	0.1364	0.2128	0.9204	No	0.8766	0.187	0.269	0.73	No	No
93909_f_at	Ccrn4l /// Sgip1 /// LOC280487 /// LOC639390	CCR4 carbon catabolite repression 4-like (S. cerevisiae) /// SH3-domain GRB2-like (endophilin) interacting protein 1 /// pol polyprotein /// gag protein	0.9415	0.5296	0.2231	0.7866	No	0.9015	0.147	0.0056	0.923	No	No
93910_at	Pnck	pregnancy upregulated non-ubiquitously expressed CaM kinase	1.0192	0.7513	0.6281	0.7119	No	1.0677	0.299	0.0762	0.866	No	No
93911_at	Ncoa6ip	nuclear receptor coactivator 6 interacting protein	0.9637	0.5055	0.084	0.1674	No	0.9729	0.429	0.0827	0.188	No	No
93912_at	Bat3	HLA-B-associated transcript 3	0.9657	0.5923	0.2355	0.241	No	0.975	0.618	0.102	0.198	No	No
93913_at	Ebf3	early B-cell factor 3	0.9429	0.0523	0.4328	0.234	No	0.9433	0.41	0.549	0.208	No	No
93914_at	Il1r1	interleukin 1 receptor, type I	0.9684	0.6761	0.1469	0.8011	No	0.9271	0.31	0.127	0.751	No	No
93915_at	Pou2af1	POU domain, class 2, associating factor 1	1.0559	0.4194	0.1503	0.391	No	1.0799	0.181	0.519	0.782	No	No
93916_at	Smyd5	SET and MYND domain containing 5	0.9814	0.5976	0.3672	0.5957	No	0.9331	0.35	0.189	0.0385	No	No
93917_at	Tnfsf12	tumor necrosis factor (ligand) superfamily, member 12	0.9352	0.3403	0.7231	0.6523	No	0.9035	0.101	0.721	0.579	No	No
93918_at	Taf9	TAF9 RNA polymerase II, TATA box binding protein (TBP)-associated factor	1.1122	0.017	0.0109	0.2844	No	1.0796	0.0655	0.889	0.442	No	No
93919_at	Nrk	Nik related kinase	0.8194	0.0037	0.0117	0.2567	Yes	1.0857	0.295	0.551	0.0303	No	No
93920_at	---	---	1.0186	0.5463	0.1141	0.1017	No	0.9714	0.55	0.264	0.196	No	No
93921_at	Bat3	HLA-B-associated transcript 3	0.9407	0.2563	0.0137	0.7356	No	0.9295	0.223	0.028	0.434	No	No
93922_g_at	Bat3	HLA-B-associated transcript 3	1.0148	0.5067	0.0617	0.0676	No	0.9995	0.865	0.0501	0.311	No	No
93923_at	Atxn7l3	ataxin 7-like 3	0.9341	0.2739	0.4294	0.9269	No	1.0481	0.216	0.421	0.67	No	No
93924_f_at	Tuba7	tubulin, alpha 7	1.0859	0.2484	0.5792	0.8039	No	1.129	0.0666	0.228	0.621	No	No
93925_at	Gata6	GATA binding protein 6	1.0747	0.2854	0.0062	0.8908	No	0.9895	0.914	0.884	0.601	No	No
93926_at	Prlr	prolactin receptor	1.0233	0.6009	0.2471	0.2434	No	1.0896	0.216	0.356	0.059	No	No
93927_f_at	Igh /// Igh-1a /// AI324046 /// LOC544903 /// LOC628614 /// LOC629871 /// LOC629884 /// LOC634081 /// LOC634100 /// LOC634136 /// LOC634206 /// LOC634222 /// LOC634338 /// LOC634572 /// LOC637000	immunoglobulin heavy chain complex /// immunoglobulin heavy chain 1a (serum IgG2a) /// expressed sequence AI324046 /// similar to immunoglobulin mu-chain /// similar to Ig heavy chain V-I region V35 precursor /// similar to Ig heavy chain V-I region V35 precursor /// similar to Ig heavy chain V region AC38 205.12 /// similar to Ig heavy chain V-I region HG3 precursor /// similar to Ig heavy chain V region 108A precursor /// similar to Ig heavy chain V-I region V35 precursor /// similar to Ig heavy chain V-I region V35 precursor /// similar to Ig heavy chain V-I region V35 precursor /// similar to Ig heavy chain V-I region V35 precursor /// similar to Ig heavy chain V-I region V35 precursor /// similar to Ig heavy chain V region 108A precursor	1.0155	0.9006	0.3367	0.7566	No	1.0993	0.521	0.529	0.317	No	No
93928_f_at	/// LOC625794	hypothetical protein LOC625794	1.0075	0.8793	0.7159	0.1801	No	0.9519	0.379	0.0924	0.517	No	No
93929_s_at	Plf /// Plf2 /// Mrpplf3 /// Mrpplf4	proliferin /// proliferin 2 /// mitogen regulated protein, proliferin 3 /// mitogen regulated protein, proliferin 4	0.9445	0.2608	0.9866	0.0287	No	1.0007	0.985	0.951	0.0951	No	No
93930_at	Lasp1	LIM and SH3 protein 1	1.046	0.6119	0.5684	0.6902	No	0.985	0.534	0.207	0.498	No	No
93931_at	Prf1	perforin 1 (pore forming protein)	1.0536	0.0515	0.0007	0.0155	No	1.0507	0.277	0.61	0.0746	No	No
93932_at	---	---	1.1448	0.0607	0.1846	0.3208	No	1.1602	0.0668	0.348	0.187	No	No
93933_at	Ppp1r3c	protein phosphatase 1, regulatory (inhibitor) subunit 3C	0.9634	0.2429	0.3224	0.1135	No	0.9858	0.81	0.0128	0.186	No	No
93934_at	Cldn2	claudin 2	0.9799	0.7184	0.3336	0.2758	No	1.013	0.78	0.0054	0.0068	No	No
93935_at	Nlk	nemo like kinase	0.9506	0.2169	0.0023	0.614	No	0.9551	0.627	0.396	0.626	No	No
93936_at	Ptprg /// LOC632664	protein tyrosine phosphatase, receptor type, G /// similar to protein tyrosine phosphatase, receptor type, G	1.0882	0.1208	0.0115	0.2658	No	1.1116	0.0237	0.0191	0.301	No	No
93937_at	Gas8	growth arrest specific 8	0.9091	0.0748	0.199	0.9994	No	0.9794	0.455	0.0383	0.8	No	No
93938_at	Slc30a1	solute carrier family 30 (zinc transporter), member 1	1.1118	0.0842	0.0018	0.9599	No	1.1361	0.0928	0.0013	0.455	No	No
93939_at	Lnk	linker of T-cell receptor pathways	1.03	0.5545	0.9578	0.8978	No	1.0035	0.912	0.0826	0.937	No	No
93940_at	Pon3	paraoxonase 3	0.9432	0.2024	0.0129	0.1718	No	0.9219	0.0453	0.0018	0.0294	No	No
93941_at	T	brachyury	1.0021	0.9575	0.6389	0.0793	No	1.1174	0.203	0.745	0.0473	No	No
93942_at	Inpp1	inositol polyphosphate-1-phosphatase	0.9907	0.8519	0.1441	0.7176	No	1.1138	0.108	0.0039	0.857	No	No
93943_f_at	Zfp36l2	zinc finger protein 36, C3H type-like 2	1.0733	0.3105	0.0055	0.9941	No	1.0638	0.225	0.0015	0.671	No	No
93944_r_at	Zfp36l2	zinc finger protein 36, C3H type-like 2	0.9941	0.8176	0.0027	0.2916	No	0.9384	0.197	0.321	0.664	No	No
93945_at	Esr1	estrogen receptor 1 (alpha)	1.0963	0.1629	0.6507	0.2335	No	1.1244	0.0817	0.0027	0.751	No	No
93946_at	Esr1	estrogen receptor 1 (alpha)	1.0934	0.0626	0.1972	0.3044	No	1.0129	0.454	0.0001	0.205	No	No
93947_g_at	Esr1	estrogen receptor 1 (alpha)	0.9988	0.9814	0.0114	0.9681	No	1.07	0.346	0.0018	0.0434	No	No
93948_at	Nck2	non-catalytic region of tyrosine kinase adaptor protein 2	0.9959	0.9375	0.017	0.3964	No	1.0827	0.0358	0.0065	0.552	No	No
93949_at	Gnb4	guanine nucleotide binding protein, beta 4	0.9889	0.7045	0.0008	0.8438	No	1.0121	0.598	0.0537	0.505	No	No
93950_at	Foxa2	forkhead box A2	0.9801	0.5113	0.0002	0.9167	No	1.0425	0.493	0.529	0.122	No	No
93951_at	Golga3	golgi autoantigen, golgin subfamily a, 3	0.9388	0.2025	0.0443	0.9539	No	0.9904	0.897	0.0402	0.48	No	No
93952_r_at	2810417H13 Rik	RIKEN cDNA 2810417H13 gene	1.1438	0.1582	0.0571	0.0953	No	1.2368	0.0375	0.0029	0.0394	No	No
93953_at	Prss12	protease, serine, 12 neurotrypsin (motopsin)	0.9054	0.0496	0.3906	0.5804	No	0.8251	0.0017	0.0018	0.0056	No	No
93954_at	Gucy1b3	guanylate cyclase 1, soluble, beta 3	1.1048	0.0669	0.0009	0.4751	No	1.0709	0.385	0.0548	0.369	No	No
93955_at	Zpbp	zona pellucida binding protein	1.029	0.6311	0.1153	0.6917	No	1.0798	0.59	0.196	0.775	No	No
93956_at	Ifit3	interferon-induced protein with tetratricopeptide repeats 3	0.9696	0.5393	0.2656	0.2237	No	1.0814	0.493	0.745	0.381	No	No
93957_at	Vpreb3	pre-B lymphocyte gene 3	1.1262	0.0021	0.0001	0.1905	Yes	1.2054	0.0107	0.0018	0.272	No	Yes
93958_at	Rnf14	ring finger protein 14	0.9989	0.9687	0.013	0.0837	No	1.0103	0.713	0.0155	0.201	No	No
93959_at	2010107H07 Rik	RIKEN cDNA 2010107H07 gene	1.0647	0.4147	0.9432	0.8362	No	1.108	0.195	0.692	0.495	No	No
93961_at	Itgae	integrin, alpha E, epithelial-associated	1.0184	0.556	0.3285	0.7218	No	1.097	0.219	0.0067	0.0774	No	No
93962_at	---	---	0.9793	0.367	0.0003	0.2493	No	0.9797	0.594	0.0636	0.169	No	No
93963_at	Tmem71	transmembrane protein 71	1.072	0.3717	0.6428	0.7958	No	1.0552	0.436	0.34	0.392	No	No
93964_s_at	Ddx6	DEAD (Asp-Glu-Ala-Asp) box polypeptide 6	0.8158	0.076	0.0008	0.2357	No	0.9188	0.24	0.0199	0.81	No	No
93965_r_at	Ddx6	DEAD (Asp-Glu-Ala-Asp) box polypeptide 6	1.0328	0.6959	0.0005	0.378	No	0.7107	0.0044	0.0136	0.0076	No	No
93966_at	Ube4b	ubiquitination factor E4B, UFD2 homolog (S. cerevisiae)	0.9253	0.038	0.0004	0.1305	No	0.9111	0.0274	0.557	0.163	No	No
93967_at	Anapc11	anaphase promoting complex subunit 11 homolog (yeast)	1.1113	0.0689	0.0724	0.178	No	0.9896	0.807	0.1	0.563	No	No
93968_at	Pdcd5	programmed cell death 5	1.0378	0.6245	0.0226	0.4251	No	0.9876	0.964	0.092	0.149	No	No
93969_at	3930401K13 Rik	RIKEN cDNA 3930401K13 gene	1.0026	0.9491	0.2116	0.8565	No	1.1506	0.092	0.523	0.207	No	No
93970_at	Ipo7	importin 7	0.9882	0.9029	0.0052	0.5707	No	1.0535	0.44	0.0017	0.55	No	No
93971_f_at	Psmd12	proteasome (prosome, macropain) 26S subunit, non-ATPase, 12	0.9681	0.6016	0.0022	0.6217	No	0.943	0.351	0.0012	0.422	No	No
93972_at	Ndufs2	NADH dehydrogenase (ubiquinone) Fe-S protein 2	0.9866	0.876	0.8567	0.6991	No	0.9748	0.788	0.4	0.53	No	No

93973_at	Eif3s9	eukaryotic translation initiation factor 3, subunit 9 (eta)	1.0091	0.8525	0.0019	0.5137	No	1.0545	0.187	0.0031	0.837	No	No
93974_at	Errfi1	ERBB receptor feedback inhibitor 1	0.8323	0.0322	0.1453	0.3955	No	0.8422	0.0148	0.813	0.496	No	No
93975_at	Errfi1	ERBB receptor feedback inhibitor 1	0.8156	0.0017	0.0517	0.0815	Yes	0.8846	0.0655	0.594	0.099	No	No
93976_at	Cab39	calcium binding protein 39	0.999	0.99	0.0644	0.4505	No	0.9839	0.667	0.0049	0.294	No	No
93978_at	B3galnt2	UDP-GalNAc:betaGlcNAc beta 1,3-galactosaminyltransferase, polypeptide 2	0.9307	0.0432	0.287	0.0985	No	0.8484	0.0119	0.698	0.141	No	No
93980_at	Rbm13	RNA binding motif protein 13	1.1253	0.0294	0.0074	0.0307	No	1.1416	0.0538	0.007	0.0444	No	No
93981_at	Plat	plasminogen activator, tissue	0.9943	0.9417	0.0847	0.7831	No	0.8999	0.13	0.19	0.159	No	No
93982_at	Derl1	Der1-like domain family, member 1	0.9612	0.4839	0.0158	0.4945	No	1.0095	0.827	0.0589	0.819	No	No
93983_at	Derl1	Der1-like domain family, member 1	0.9879	0.6505	0.0788	0.1286	No	0.9999	0.967	0.141	0.809	No	No
93984_at	Atpif1	ATPase inhibitory factor 1	1.1196	0.0427	0.0214	0.7011	No	1.0742	0.115	0.0019	0.79	No	No
93985_at	Tiparp	TCDD-inducible poly(ADP-ribose) polymerase	0.9964	0.7924	0.0001	0.0227	No	1.0224	0.115	0.0001	0.0232	No	No
93986_at	2410003A14 Rik	RIKEN cDNA 2410003A14 gene	1.0854	0.092	0.2251	0.6787	No	1.0767	0.0511	0.0022	0.435	No	No
93987_f_at	Rpl22l1	ribosomal protein L22 like 1	0.975	0.5514	0.7814	0.3488	No	0.9711	0.105	0.156	0.209	No	No
93988_at	Psma7	proteasome (prosome, macropain) subunit, alpha type 7	1.1393	0.0516	0.0094	0.0598	No	1.1302	0.0555	0.0131	0.0114	No	No
93990_at	Hnrph1	heterogeneous nuclear ribonucleoprotein H1	1.0874	0.4877	0.5988	0.2772	No	1.0767	0.435	0.475	0.137	No	No
93991_at	Mdh2	malate dehydrogenase 2, NAD (mitochondrial)	1.0058	0.8879	1	0.091	No	1.0226	0.341	0.004	0.0264	No	No
93992_at	5430437P03 Rik	RIKEN cDNA 5430437P03 gene	0.9461	0.0668	0.0503	0.0591	No	1.0061	0.548	0.0004	0.0303	No	No
93993_at	Lman2	lectin, mannose-binding 2	1.0335	0.1166	0.0002	0.0115	No	1.0066	0.238	0	0.0049	No	No
93994_at	Chpt1	choline phosphotransferase 1	1.0302	0.6741	0.1892	0.1743	No	0.9897	0.93	0.924	0.192	No	No
93996_at	Cyp2e1	cytochrome P450, family 2, subfamily e, polypeptide 1	1.0274	0.6371	0.6879	0.3683	No	1.0852	0.0785	0.642	0.009	No	No
93997_at	Ifrg15	interferon alpha responsive gene	1.0079	0.9115	0.0284	0.9385	No	1.0283	0.652	0.128	0.487	No	No
93999_at	Snrpg /// LOC666609 /// LOC671824	small nuclear ribonucleoprotein polypeptide G /// similar to small nuclear ribonucleoprotein polypeptide G /// similar to small nuclear ribonucleoprotein polypeptide G	1.0408	0.4703	0.0023	0.8744	No	1.0382	0.557	0.0009	0.638	No	No
94000_at	Cd8b1	CD8 antigen, beta chain 1	0.9756	0.7113	0.1328	0.434	No	1.0101	0.774	0.0466	0.395	No	No
94001_at	Elavl1	ELAV (embryonic lethal, abnormal vision, Drosophila)-like 1 (Hu antigen R)	1.081	0.0122	0.0027	0.8224	No	1.1027	0.0079	0.0181	0.389	Yes	Yes
94002_at	Cul1	cullin 1	0.9698	0.4197	0.054	0.149	No	0.9821	0.718	0.0566	0.017	No	No
94003_at	Wnk1	WNK lysine deficient protein kinase 1	0.915	0.346	0.1739	0.5001	No	1.0242	0.949	0.0003	0.154	No	No
94004_at	Cnn2	calponin 2	1.0481	0.3766	0.0052	0.1584	No	1.0739	0.146	0.0018	0.102	No	No
94005_at	Pmpcb	peptidase (mitochondrial processing) beta	0.9883	0.9291	0.0337	0.8	No	0.9626	0.351	0.09	0.0464	No	No
94006_at	Azi2	5-azacytidine induced gene 2	0.856	0.0919	0.2425	0.5331	No	0.9529	0.462	0.169	0.733	No	No
94007_at	Morf4l1 /// LOC433955 /// A730098P11 Rik /// LOC627352 /// LOC635663	mortality factor 4 like 1 /// similar to mortality factor 4 like 1 isoform b /// RIKEN cDNA A730098P11 gene /// similar to mortality factor 4 like 1 /// similar to mortality factor 4 like 1 (predicted)	1.1405	0.021	0.8049	0.0525	No	1.1159	0.0945	0.291	0.54	No	No
94008_at	Tceb1	transcription elongation factor B (SIII), polypeptide 1	1.0487	0.47	0.0192	0.8778	No	1.0217	0.849	0.0327	0.562	No	No
94009_at	Tceb1	transcription elongation factor B (SIII), polypeptide 1	0.9878	0.7153	0.329	0.7485	No	0.9686	0.724	0.843	0.434	No	No
94010_g_at	Tceb1	transcription elongation factor B (SIII), polypeptide 1	1.0006	0.9926	0.1406	0.6858	No	1.0013	0.963	0.13	0.344	No	No
94011_at	3100004P22 Rik	RIKEN cDNA 3100004P22 gene	1.017	0.696	0.0017	0.1274	No	1.0007	0.83	0.0014	0.217	No	No
94012_at	Timm10	translocase of inner mitochondrial membrane 10 homolog (yeast)	1.0619	0.1698	0.0024	0.1982	No	0.932	0.159	0.0279	0.678	No	No
94014_at	2510048O06 Rik	RIKEN cDNA 2510048O06 gene	1.0229	0.1351	0.0002	0.0535	No	0.9987	0.972	0.0118	0.389	No	No
94015_at	Th1l	TH1-like homolog (Drosophila)	0.9801	0.4675	0.4765	0.0696	No	0.9939	0.708	0.109	0.0056	No	No
94016_at	Tubb2c	tubulin, beta 2c	1.0603	0.4363	0.0285	0.3072	No	0.9514	0.119	0.3	0.79	No	No
94017_s_at	Sfrs2	splicing factor, arginine/serine-rich 2 (SC-35)	1.1787	0.0093	0.0362	0.4734	Yes	1.1787	0.0082	0.0017	0.958	Yes	Yes
94018_at	Ubl3	ubiquitin-like 3	1.095	0.3337	0.0109	0.1168	No	1.0118	0.77	0.123	0.409	No	No
94019_at	---	---	1.0835	0.1777	0.1326	0.2171	No	1.0703	0.321	0.428	0.0782	No	No
94020_at	Ptk9l	protein tyrosine kinase 9-like (A6-related protein)	0.89	0.019	0.0008	0.0067	No	0.841	0.0082	0.0003	0.0027	No	No
94021_at	Trim11	tripartite motif protein 11	0.9404	0.2333	0.0395	0.3451	No	0.9797	0.74	0.487	0.708	No	No
94022_at	Gltscr2	glioma tumor suppressor candidate region gene 2	1.0004	0.9938	0.0047	0.3396	No	0.9879	0.817	0.0136	0.154	No	No
94024_at	Cdt1	chromatin licensing and DNA replication factor 1	1.1068	0.0567	0.0918	0.0712	No	1.064	0.182	0.0331	0.257	No	No
94025_at	Psmb3	proteasome (prosome, macropain) subunit, beta type 3	1.0926	0.0596	0.0032	0.6417	No	0.9863	0.92	0.0035	0.0226	No	No
94026_at	Pomc1	pro-opiomelanocortin-alpha	1.0242	0.7053	0.9715	0.0013	No	0.9666	0.184	0.0119	0.0018	No	No
94027_at	---	8 cells embryo 8 cells cDNA, RIKEN full-length enriched library, clone:E860019P17 product:unclassifiable, full insert sequence /// CDNA clone IMAGE:30033444 /// 2 cells egg cDNA, RIKEN full-length enriched library, clone:B020014N01 product:hypothetical protein, full insert sequence	1.0904	0.1639	0.0018	0.0165	No	1.1191	0.0234	0.0033	0.0045	No	No
94028_f_at	---	8 cells embryo 8 cells cDNA, RIKEN full-length enriched library, clone:E860019P17 product:unclassifiable, full insert sequence	0.9984	0.9623	0.0161	0.0063	No	1.0399	0.689	0.486	0.0697	No	No
94030_at	Commd2	COMM domain containing 2	1.0591	0.2384	0.7766	0.2479	No	1.0206	0.728	0.0814	0.925	No	No
94031_at	Rab2	RAB2, member RAS oncogene family	1.1365	0.1788	0.1384	0.8585	No	1.0536	0.372	0.412	0.748	No	No
94032_at	Apoa1bp	apolipoprotein A-I binding protein	0.9925	0.7723	0.0033	0.0807	No	1.0014	0.754	0.0011	0.0244	No	No
94034_at	Rexo2	REX2, RNA exonuclease 2 homolog (S. cerevisiae)	1.0587	0.5583	0.0176	0.8365	No	1.0283	0.439	0.0007	0.275	No	No
94035_at	---	---	1.0101	0.8237	0.6675	0.4936	No	1.0446	0.311	0.113	0.105	No	No
94036_at	Cdc42ep4	CDC42 effector protein (Rho GTPase binding) 4	0.9212	0.0742	0.0096	0.1591	No	0.9758	0.422	0.234	0.279	No	No
94037_at	Ela2a	elastase 2A	0.9922	0.8285	0.0158	0.1589	No	1.0236	0.453	0.0003	0.645	No	No
94038_at	2700060E02 Rik	RIKEN cDNA 2700060E02 gene	1.0904	0.0546	0.7724	0.4609	No	1.0393	0.398	0.0157	0.68	No	No
94039_at	---	Transcribed locus	1.1198	0.1364	0.0188	0.0703	No	1.1582	0.027	0.006	0.0578	No	No
94040_at	Erh	enhancer of rudimentary homolog (Drosophila)	1.1554	0.0142	0.1956	0.787	No	1.2034	0.0079	0.302	0.649	Yes	Yes
94041_at	Hnrpk	heterogeneous nuclear ribonucleoprotein K	1.2405	0.0413	0.0041	0.4133	No	1.1787	0.0298	0.0015	0.269	No	No
94042_f_at	Gng5	guanine nucleotide binding protein (G protein), gamma 5 subunit	1.1078	0.2077	0.2635	0.7452	No	1.0245	0.674	0.09	0.492	No	No
94043_at	Atp6ap1	ATPase, H+ transporting, lysosomal accessory protein 1	0.9954	0.9222	0.0397	0.5183	No	0.975	0.502	0.0119	0.239	No	No
94044_at	Suz12	suppressor of zeste 12 homolog (Drosophila)	1.0542	0.3389	0.043	0.3503	No	1.0919	0.488	0.0012	0.0771	No	No
94045_at	Ambp	alpha 1 microglobulin/bikunin	1.0202	0.4928	0.0238	0.0431	No	1.0406	0.934	0.0429	0.178	No	No
94046_at	Csk	c-src tyrosine kinase	0.9667	0.4283	0.0014	0.3818	No	1.054	0.276	0.0143	0.101	No	No
94047_at	0610031J06 Rik	RIKEN cDNA 0610031J06 gene	1.0319	0.237	0.2115	0.0689	No	1.0365	0.312	0.6	0.3	No	No
94048_at	Cdc34	cell division cycle 34 homolog (S. cerevisiae)	0.9155	0.1021	0.0299	0.014	No	0.9644	0.544	0.216	0.0413	No	No
94049_at	Bhmt	betaine-homocysteine methyltransferase	0.9184	0.2237	0.0684	0.2902	No	0.9592	0.554	0.783	0.881	No	No
94051_at	Edil3	EGF-like repeats and discoidin I-like domains 3	1.006	0.8896	0.3181	0.9672	No	1.1339	0.0708	0.49	0.497	No	No
94052_at	Dpm2	dolichol-phosphate (beta-D) mannosyltransferase 2	1.0196	0.6078	0.5247	0.5483	No	1.0264	0.122	0.164	0.0261	No	No
94053_at	Cpsf3l	cleavage and polyadenylation specific factor 3-like	0.9738	0.5345	0.4173	0.1903	No	1.0172	0.891	0.158	0.0936	No	No
94054_at	Cttn	cortactin	1.0201	0.1363	0.005	0.5924	No	0.9866	0.447	0.0125	0.0253	No	No
94055_at	Cttn	cortactin	0.9736	0.4378	0.0645	0.0772	No	1.008	0.763	0.0239	0.293	No	No
94056_at	Scd1	stearoyl-Coenzyme A desaturase 1	0.9748	0.7533	0.3027	0.8229	No	0.95	0.303	0.793	0.694	No	No
94057_g_at	Scd1	stearoyl-Coenzyme A desaturase 1	0.9745	0.6658	0.2927	0.6229	No	0.9688	0.546	0.0329	0.607	No	No
94058_r_at	Scd1	stearoyl-Coenzyme A desaturase 1	0.9882	0.8845	0	0.9311	No	0.9682	0.427	0	0.641	No	No
94059_at	Nrbp	nuclear receptor binding protein	0.9936	0.9009	0.1493	0.2779	No	0.9166	0.145	0.749	0.1	No	No
94060_at	Kctd10	potassium channel tetramerisation domain containing 10	0.9698	0.4654	0.0047	0.0388	No	0.9435	0.266	0.0019	0.269	No	No
94061_at	Crip1	cysteine-rich protein 1 (intestinal)	0.9312	0.2222	0.0015	0.1071	No	0.9795	0.725	0.0038	0.104	No	No
94062_at	Ndufv2	NADH dehydrogenase (ubiquinone) flavoprotein 2	1.0053	0.9469	0.9381	0.6076	No	0.9918	0.951	0.0003	0.2	No	No
94063_at	Sema4a	sema domain, immunoglobulin domain (Ig), transmembrane domain (TM) and short cytoplasmic domain, (semaphorin) 4A	0.928	0.0886	0.1529	0.958	No	0.871	0.0384	0.0788	0.665	No	No
94064_at	Zfp91	zinc finger protein 91	0.9273	0.2293	0.0932	0.2885	No	0.8581	0.0104	0.41	0.0257	No	No
94065_at	Crk	v-crk sarcoma virus CT10 oncogene homolog (avian)	0.9667	0.3451	0.1859	0.5136	No	0.9981	0.807	0.0012	0.006	No	No
94066_at	Rnf14	ring finger protein 14	0.9803	0.3484	0.258	0.3021	No	0.9803	0.495	0.0191	0.792	No	No
94067_at	Dcps	decapping enzyme, scavenger	0.9809	0.1446	0.0001	0.001	No	0.9863	0.492	0.0001	0.0755	No	No

94068_at	Rps19	ribosomal protein S19	0.9948	0.8749	0.0142	0.6193	No	0.9813	0.371	0.0032	0.0504	No	No
94069_r_at	2610204L23 Rik /// LOC666452 /// LOC677557	RIKEN cDNA 2610204L23 gene /// hypothetical protein LOC666452 /// hypothetical protein LOC677557	1.0452	0.2352	0.1561	0.1731	No	1.2131	0.0448	0.972	0.363	No	No
94070_at	Gosr2	golgi SNAP receptor complex member 2	0.9597	0.6057	0.2169	0.5971	No	1.0266	0.923	0.0465	0.441	No	No
94071_at	Gosr2	golgi SNAP receptor complex member 2	1.0921	0.0833	0.6017	0.1583	No	1.0471	0.242	0.0034	0.364	No	No
94072_g_at	Gosr2	golgi SNAP receptor complex member 2	0.9898	0.6147	0.0312	0.322	No	0.9773	0.388	0.261	0.122	No	No
94073_at	Polr2g	polymerase (RNA) II (DNA directed) polypeptide G	1.0334	0.3846	0.1442	0.5633	No	1.0322	0.266	0.0422	0.642	No	No
94074_at	Fcgr3	Fc receptor, IgG, low affinity III	0.9825	0.7462	0.6775	0.5137	No	1.0111	0.811	0.0032	0.682	No	No
94075_at	Fabp1	fatty acid binding protein 1, liver	0.9595	0.304	0.0075	0.2676	No	0.9529	0.319	0.0037	0.0508	No	No
94076_i_at	Rpn2	ribophorin II	0.9693	0.5511	0.263	0.5331	No	1.1383	0.122	0.0055	0.691	No	No
94077_f_at	Rpn2	ribophorin II	1.0734	0.0527	0.002	0.9475	No	1.0684	0.321	0.192	0.283	No	No
94078_at	1110020P15 Rik	RIKEN cDNA 1110020P15 gene	1.0462	0.2313	0.0119	0.0292	No	1.0296	0.275	0.0042	0.0259	No	No
94079_at	38964	septin 4	0.7865	0.001	0.0009	0.0067	No	0.8099	0.0003	0.0001	0.0072	No	No
94080_at	Sdha	succinate dehydrogenase complex, subunit A, flavoprotein (Fp)	1.0089	0.9378	0.006	0.6576	No	1.0252	0.841	0.0025	0.711	No	No
94081_at	Rbm17	RNA binding motif protein 17	0.9973	0.9364	0.2083	0.2829	No	0.8925	0.0568	0.0087	0.293	No	No
94084_at	Cdc26	cell division cycle 26	1.0018	0.9733	0.1375	0.1329	No	1.0121	0.737	0.355	0.068	No	No
94085_at	Prg1	proteoglycan 1, secretory granule	1.081	0.0975	0.0024	0.0126	No	1.0318	0.886	0.0166	0.0182	No	No
94086_at	Il3	interleukin 3	0.9814	0.7945	0.7278	0.5621	No	0.9423	0.391	0.008	0.609	No	No
94087_at	Prkcm	protein kinase C, mu	0.7916	0.0171	0.0374	0.2972	No	0.8765	0.0844	0.0213	0.231	No	No
94088_at	Ptbp2	polypyrimidine tract binding protein 2	1.0253	0.6607	0.0025	0.0746	No	1.0275	0.479	0.0099	0.0732	No	No
94089_at	Pkd1l3	Polycystic kidney disease 1 like 3	0.9967	0.9122	0.1667	0.3277	No	1.0423	0.552	0.303	0.314	No	No
94090_at	Tlm	T lymphoma oncogene	0.9704	0.5567	0.188	0.0705	No	1.0786	0.223	0.0746	0.0152	No	No
94095_at	---	---	1.1446	0.0613	0.6714	0.0235	No	1.2567	0.021	0.0592	0.0088	No	No
94097_at	Gli1	GLI-Kruppel family member GLI1	0.9503	0.1944	0.0234	0.3356	No	0.8659	0.134	0.272	0.0648	No	No
94098_at	Hcrt	hypocretin	0.9241	0.1253	0.829	0.365	No	0.9694	0.517	0.0098	0.701	No	No
94099_at	Nptx2	neuronal pentraxin 2	1.0074	0.6953	0.0416	0.3986	No	1.0401	0.347	0.512	0.534	No	No
94100_s_at	Trpc4	transient receptor potential cation channel, subfamily C, member 4	0.9787	0.7266	0.8615	0.251	No	1.1143	0.256	0.737	0.215	No	No
94101_at	Zfp98	zinc finger protein 98	0.9782	0.4311	0.0972	0.3494	No	0.9496	0.075	0.0582	0.0046	No	No
94102_at	Hmx1	H6 homeo box 1	1.0207	0.6943	0.1562	0.9552	No	0.9662	0.0555	0.0926	0.363	No	No
94103_at	Tspy-ps	testis specific protein-Y encoded, pseudogene	0.9552	0.3859	0.4855	0.7606	No	0.9965	0.903	0.0555	0.287	No	No
94104_at	Tert	telomerase reverse transcriptase	1.016	0.6722	0.0639	0.0311	No	1.0487	0.0343	0.0816	0.011	No	No
94105_at	Cdc42	cell division cycle 42 homolog (S. cerevisiae)	0.9963	0.9598	0.0662	0.5223	No	0.9925	0.904	0.0118	0.711	No	No
94106_at	---	Transcribed locus	1.0129	0.833	0.0035	0.3244	No	0.9528	0.393	0.0025	0.609	No	No
94107_at	Dnase1l3	deoxyribonuclease 1-like 3	1.0795	0.2127	0.0439	0.7127	No	1.0203	0.798	0.838	0.829	No	No
94108_at	Pik3c2g	phosphatidylinositol 3-kinase, C2 domain containing, gamma polypeptide	0.9897	0.7721	0.2429	0.0211	No	1.0688	0.267	0.0204	0.21	No	No
94109_at	Zfp281	zinc finger protein 281	0.9128	0.2012	0.0091	0.08	No	0.8957	0.0715	0.0517	0.0595	No	No
94110_f_at	D10627	cDNA sequence D10627	0.9948	0.9561	0.123	0.8326	No	1.0305	0.727	0.132	0.884	No	No
94111_r_at	D10627	cDNA sequence D10627	1.013	0.7387	0.2027	0.7588	No	1.0116	0.0767	0.0041	0.003	No	No
94112_at	Tnfsf10	tumor necrosis factor (ligand) superfamily, member 10	0.9491	0.2595	0.4329	0.2077	No	0.9956	0.913	0.361	0.623	No	No
94113_at	4933415E08 Rik	RIKEN cDNA 4933415E08 gene	1.0335	0.5612	0.0432	0.1587	No	1.0854	0.0234	0.0037	0.0215	No	No
94114_at	Hist1h1a	histone 1, H1a	1.0668	0.2701	0.0312	0.6805	No	1.0491	0.351	0.0066	0.893	No	No
94115_at	Npy5r	neuropeptide Y receptor Y5	0.9349	0.5418	0.972	0.975	No	0.9822	0.801	0.745	0.653	No	No
94116_at	Brs3	bombesin-like receptor 3	1.1322	0.0478	0.0054	0.0692	No	1.1059	0.196	0.0192	0.277	No	No
94117_f_at	Punc	putative neuronal cell adhesion molecule	1.1123	0.1836	0.4184	0.3999	No	0.9845	0.878	0.481	0.703	No	No
94118_r_at	Punc	putative neuronal cell adhesion molecule	0.959	0.3298	0.5448	0.5127	No	0.9285	0.395	0.815	0.267	No	No
94119_at	Pip5k1c	phosphatidylinositol-4-phosphate 5-kinase, type 1 gamma	0.9887	0.7673	0.0008	0.5524	No	0.9955	0.972	0.0001	0.0507	No	No
94120_s_at	Sprr2f	small proline-rich protein 2F	0.9422	0.3844	0.1904	0.8666	No	1.0499	0.466	0.828	0.411	No	No
94121_at	Sprr2h	small proline-rich protein 2H	1.0458	0.4259	0.4974	0.7135	No	1.1156	0.166	0.209	0.173	No	No
94122_at	Myoc	myocilin	0.9826	0.5338	0.0416	0.1039	No	0.9798	0.41	0.665	0.334	No	No
94123_at	Neurod4	neurogenic differentiation 4	1.0036	0.9106	0.2261	0.0905	No	1.0824	0.489	0.83	0.209	No	No
94124_at	Top3a	topoisomerase (DNA) III alpha	0.9697	0.6327	0.085	0.1808	No	0.9834	0.446	0.026	0.0348	No	No
94125_at	Eva1	epithelial V-like antigen 1	0.9803	0.4857	0.0115	0.3031	No	0.9425	0.198	0.002	0.357	No	No
94126_at	Wnt2b	wingless related MMTV integration site 2b	1.0023	0.9625	0.0572	0.7764	No	0.9892	0.869	0.0813	0.564	No	No
94127_at	Diap2	diaphanous homolog 2 (Drosophila)	1.0013	0.9675	0.0075	0.9133	No	0.9791	0.898	0.91	0.856	No	No
94128_at	Bard1	BRCA1 associated RING domain 1	0.9925	0.8455	0.7056	0.3686	No	0.9189	0.0391	0.0898	0.991	No	No
94129_at	---	Transcribed locus	0.9946	0.8421	0.0043	0.0752	No	1.2184	0.346	0.926	0.131	No	No
94130_at	P140	P140 gene	0.9147	0.4495	0.0364	0.8366	No	0.9243	0.46	0.0195	0.76	No	No
94131_at	Xpo1	exportin 1, CRM1 homolog (yeast)	0.948	0.3903	0.5549	0.8897	No	0.9654	0.818	0.335	0.525	No	No
94132_at	Gpx1	glutathione peroxidase 1	1.0869	0.3351	0.031	0.1777	No	1.0966	0.148	0.0057	0.0434	No	No
94133_r_at	Mef2a	myocyte enhancer factor 2A	0.944	0.3556	0.0259	0.7485	No	1.0109	0.94	0.0051	0.918	No	No
94134_at	Wnt1	wingless-related MMTV integration site 1	1.1189	0.0368	0.0561	0.1052	No	1.0122	0.762	0.0043	0.411	No	No
94135_at	Tcfcp2	transcription factor CP2	1.0479	0.4554	0.0056	0.7655	No	0.8783	0.0023	0.0085	0.0047	No	No
94136_at	5830431A10 Rik	RIKEN cDNA 5830431A10 gene	1.0516	0.2997	0.119	0.7532	No	1.2679	0.21	0.595	0.348	No	No
94137_at	Il8rb	interleukin 8 receptor, beta	0.9505	0.4537	0.9932	0.3849	No	1.0347	0.698	0.903	0.833	No	No
94138_s_at	Fut7	fucosyltransferase 7	1.0675	0.3401	0.1509	0.4795	No	1.122	0.0902	0.886	0.119	No	No
94139_at	Pdc	phosducin	0.9167	0.2931	0.7398	0.6882	No	0.8874	0.0895	0.209	0.231	No	No
94140_at	Msr1	macrophage scavenger receptor 1	1.0101	0.725	0.0102	0.3431	No	1.03	0.732	0.487	0.466	No	No
94141_at	Fcer2a	Fc receptor, IgE, low affinity II, alpha polypeptide	1.0041	0.8826	0.4111	0.3174	No	0.9521	0.0778	0.0066	0.0599	No	No
94142_at	Csf3	colony stimulating factor 3 (granulocyte)	0.9961	0.8017	0.8296	0.3066	No	1.0576	0.167	0.81	0.746	No	No
94143_at	Gfap	glial fibrillary acidic protein	1.1005	0.1959	0.3423	0.5582	No	1.0613	0.199	0.307	0.345	No	No
94144_g_at	Gfap	glial fibrillary acidic protein	1.0261	0.6537	0.3397	0.264	No	1.108	0.0412	0.764	0.0087	No	No
94145_at	Ifnb1	interferon beta 1, fibroblast	0.9646	0.7029	0.1057	0.5065	No	1.0215	0.725	0.084	0.562	No	No
94146_at	Ccl4	chemokine (C-C motif) ligand 4	0.9594	0.4206	0.2005	0.4589	No	0.9486	0.451	0.0207	0.264	No	No
94147_at	Serpine1	serine (or cysteine) peptidase inhibitor, clade E, member 1	0.9056	0.1155	0.0641	0.6207	No	1.1321	0.183	0.855	0.0302	No	No
94148_at	Ppy	pancreatic polypeptide	1.0279	0.8054	0.2445	0.4485	No	0.989	0.962	0.141	0.521	No	No
94149_at	Hivep3	human immunodeficiency virus type I enhancer binding protein 3	0.9988	0.9895	0.9463	0.7454	No	1.079	0.44	0.332	0.799	No	No
94150_at	Sag	retinal S-antigen	0.9882	0.6822	0.6637	0.7557	No	1.1056	0.139	0.416	0.729	No	No
94151_at	Pip	prolactin induced protein	1.0497	0.3755	0.2135	0.0851	No	1.2995	0.135	0.0833	0.89	No	No
94152_at	Spt1	salivary protein 1	0.9416	0.2609	0.0883	0.1834	No	0.949	0.734	0.559	0.744	No	No
94153_g_at	Spt1 /// Spt2	salivary protein 1 /// salivary protein 2	0.9307	0.0442	0.0422	0.796	No	1.0802	0.0046	0.0002	0.0014	No	No
94154_at	Tgn	thyroglobulin	1.0512	0.2708	0.4736	0.2178	No	1.0683	0.0675	0.0114	0.411	No	No
94155_at	Rgs4	regulator of G-protein signaling 4	0.886	0.1094	0.2084	0.5126	No	1.0949	0.178	0.112	0.0252	No	No
94156_at	Nppc	natriuretic peptide precursor type C	1.1062	0.0781	0.0035	0.3464	No	1.0353	0.648	0.0478	0.424	No	No
94157_i_at	Ptafr	platelet-activating factor receptor	0.9619	0.6139	0.5143	0.8395	No	0.845	0.552	0.876	0.277	No	No
94158_f_at	Ptafr	platelet-activating factor receptor	1.0303	0.5622	0.783	0.0933	No	1.0314	0.422	0.209	0.086	No	No
94159_at	Ptafr	platelet-activating factor receptor	0.9551	0.3735	0.5038	0.682	No	0.9927	0.905	0.627	0.122	No	No
94160_at	Efnb3	ephrin B3	1.0668	0.1658	0.0268	0.0881	No	0.9668	0.274	0.0015	0.646	No	No
94161_at	Prkce	protein kinase C, epsilon	1.0555	0.4478	0.9763	0.8446	No	0.987	0.724	0.443	0.278	No	No
94162_at	Ddx4	DEAD (Asp-Glu-Ala-Asp) box polypeptide 4	0.8881	0.1541	0.2964	0.7998	No	1.0182	0.701	0.0008	0.685	No	No
94163_at	Atp4a	ATPase, H+/K+ exchanging, gastric, alpha polypeptide	1.0211	0.6511	0.2084	0.4447	No	1.057	0.189	0.272	0.19	No	No
94164_at	St8sia1	ST8 alpha-N-acetyl-neuraminide alpha-2,8-sialyltransferase 1	1.0195	0.7758	0.6837	0.6068	No	1.0349	0.395	0.024	0.0204	No	No
94165_at	---	---	1.0602	0.2772	0.1046	0.3869	No	1.1156	0.0538	0.621	0.0375	No	No
94166_g_at	Ccl1	chemokine (C-C motif) ligand 1	0.9959	0.9333	0.6235	0.6003	No	0.9577	0.34	0.168	0.713	No	No
94167_at	Nos3	nitric oxide synthase 3, endothelial cell	0.899	0.0891	0.0068	0.3957	No	1.0323	0.394	0.872	0.172	No	No
94168_at	Il13	interleukin 13	1.048	0.4087	0.5791	0.1676	No	1.0511	0.426	0.307	0.0914	No	No
94169_at	Pcdh12	protocadherin 12	0.9198	0.1552	0.015	0.765	No	0.9297	0.143	0.0058	0.768	No	No
94170_at	Chst3	carbohydrate (chondroitin 6/keratan) sulfotransferase 3	0.9852	0.703	0.0514	0.8992	No	1.0246	0.571	0.0168	0.305	No	No
94171_at	Itgb2l	integrin beta 2-like	0.9562	0.5744	0.1405	0.9578	No	0.9767	0.746	0.521	0.616	No	No
94172_at	Xrcc2	X-ray repair complementing defective repair in Chinese hamster cells 2	1.0184	0.5663	0.1199	0.1765	No	1.1047	0.195	0.195	0.551	No	No
94173_at	Cxcr3	chemokine (C-X-C motif) receptor 3	0.9958	0.93	0.1449	0.246	No	1.0439	0.0501	0.0003	0.102	No	No
94174_at	Ctnnal1	catenin (cadherin associated protein), alpha-like 1	1.0487	0.2675	0.0183	0.4313	No	1.0629	0.511	0.0371	0.858	No	No
94175_at	T2	brachyury 2	0.932	0.1015	0.0997	0.0133	No	0.9879	0.647	0.0189	0.278	No	No
94176_at	Lhx6	LIM homeobox protein 6	0.9305	0.1351	0.0849	0.1326	No	0.9561	0.59	0.297	0.689	No	No
94177_at	Hsd17b7	hydroxysteroid (17-beta) dehydrogenase 7	1.1332	0.1805	0.1696	0.9131	No	1.0196	0.766	0.208	0.821	No	No

94178_at	Cd46	CD46 antigen, complement regulatory protein	0.9645	0.5029	0.1766	0.9585	No	0.9436	0.371	0.124	0.541	No	No
94179_at	Dach1	dachshund 1 (Drosophila)	1.0142	0.7203	0.7639	0.1594	No	1.1661	0.134	0.191	0.151	No	No
94180_s_at	Dach1	dachshund 1 (Drosophila)	0.8563	0.0128	0.5168	0.1786	No	0.9007	0.139	0.142	0.572	No	No
94181_at	Scrg1	scrapie responsive gene 1	0.9173	0.0853	0.0001	0.2683	No	0.9078	0.0604	0.0002	0.741	No	No
94182_at	Cacng2	calcium channel, voltage-dependent, gamma subunit 2	0.9296	0.3051	0.1088	0.12	No	1.0094	0.83	0.0163	0.606	No	No
94183_at	Krtap13	keratin associated protein 13	0.9667	0.4573	0.0565	0.0567	No	0.9696	0.698	0.19	0.0673	No	No
94184_at	Gpr33	G protein-coupled receptor 33	0.9447	0.4934	0.2359	0.9695	No	1.1714	0.0332	0.0225	0.0104	No	No
94185_at	Kcnk4	potassium channel, subfamily K, member 4	0.9504	0.1596	0.8512	0.3986	No	1.0086	0.802	0.279	0.105	No	No
94186_at	Traf1	Tnf receptor-associated factor 1	0.8615	0.0212	0.0319	0.0429	No	0.9239	0.273	0.502	0.909	No	No
94187_at	Gsc	goosecoid	0.9244	0.3613	0.0047	0.2034	No	0.9857	0.895	0.0006	0.0275	No	No
94188_at	Hcn3	hyperpolarization-activated, cyclic nucleotide-gated K+ 3	1.0635	0.3029	0.3654	0.9303	No	1.0645	0.282	0.0663	0.62	No	No
94189_at	Bcl6b	B-cell CLL/lymphoma 6, member B	1.0142	0.7971	0.3585	0.805	No	1.0623	0.19	0.0704	0.787	No	No
94190_at	Tnfrsf17	tumor necrosis factor receptor superfamily, member 17	1.0748	0.1824	0.0546	0.1602	No	1.1155	0.0662	0.431	0.236	No	No
94191_at	F2rl3	coagulation factor II (thrombin) receptor-like 3	1.0042	0.9259	0.1427	0.058	No	1.0662	0.179	0.171	0.11	No	No
94192_at	Gdap10	ganglioside-induced differentiation-associated-protein 10	1.0007	0.9891	0.0003	0.16	No	1.0881	0.0048	0	0.0015	No	No
94193_at	Kcna7	potassium voltage-gated channel, shaker-related subfamily, member 7	1.0437	0.6134	0.1846	0.0997	No	1.1167	0.276	0.166	0.749	No	No
94194_s_at	Hcn2	hyperpolarization-activated, cyclic nucleotide-gated K+ 2	0.9383	0.5345	0.6137	0.0854	No	0.9184	0.258	0.522	0.0204	No	No
94195_r_at	Hcn2	hyperpolarization-activated, cyclic nucleotide-gated K+ 2	0.9872	0.7999	0.0841	0.4447	No	0.9576	0.447	0.0277	0.492	No	No
94196_at	Ikbkg	inhibitor of kappaB kinase gamma	1.0624	0.0129	0.888	0.0276	No	1.0959	0.219	0.0957	0.0578	No	No
94197_at	Ugcg	UDP-glucose ceramide glucosyltransferase	0.9549	0.3762	0.0114	0.411	No	0.9039	0.0526	0.0049	0.435	No	No
94198_at	Ppard	peroxisome proliferator activator receptor delta	1.0027	0.9719	0.0883	0.6864	No	0.9814	0.809	0.024	0.81	No	No
94199_at	Kap	kidney androgen regulated protein	1.0177	0.6991	0.6393	0.3049	No	1.0161	0.583	0.0168	0.0791	No	No
94200_at	Gbx2	gastrulation brain homeobox 2	1.0043	0.9205	0.7385	0.1177	No	1.1336	0.0048	0.0002	0.0143	Yes	No
94201_at	Scn1a	sodium channel, voltage-gated, type I, alpha	0.8661	0.2023	0.0081	0.7536	No	0.9287	0.553	0.002	0.837	No	No
94202_at	Tcrb-V8.2	T-cell receptor beta, variable 8.2	0.9975	0.932	0.1043	0.1039	No	1.0114	0.715	0.0026	0.753	No	No
94203_at	A630025C20 Rik	RIKEN cDNA A630025C20 gene	0.9858	0.6539	0.0087	0.7611	No	0.944	0.433	0.371	0.698	No	No
94205_at	Epx	eosinophil peroxidase	1.0251	0.577	0.0195	0.8421	No	0.9759	0.703	0.0325	0.404	No	No
94206_at	Grcc10	gene rich cluster, C10 gene	1.0152	0.6188	0.0049	0.3185	No	1.0079	0.705	0.002	0.317	No	No
94207_at	Pdia6	protein disulfide isomerase associated 6	1.1225	0.0128	0.841	0.1649	No	1.0749	0.0602	0.357	0.372	No	No
94208_at	Pdia6	protein disulfide isomerase associated 6	1.051	0.4731	0.1306	0.3201	No	1.0537	0.449	0.168	0.442	No	No
94209_g_at	Pdia6	protein disulfide isomerase associated 6	1.0977	0.0705	0.0675	0.7159	No	1.0245	0.671	0.741	0.581	No	No
94210_at	Timm13	translocase of inner mitochondrial membrane 13 homolog (yeast)	1.0276	0.6245	0.2091	0.1421	No	1.0245	0.457	0.004	0.345	No	No
94211_at	6720460F02 Rik	RIKEN cDNA 6720460F02 gene	1.0212	0.5712	0.1445	0.4078	No	0.9334	0.198	0.0025	0.0331	No	No
94212_at	Tsc22d3	TSC22 domain family 3	0.9991	0.9831	0.0025	0.057	No	1.0469	0.374	0.0057	0.922	No	No
94214_at	Fabp3	fatty acid binding protein 3, muscle and heart	0.9854	0.7332	0.6669	0.0057	No	0.962	0.336	0.757	0.0043	No	No
94215_at	Zfp313	Zinc finger protein 313	1.0009	0.9898	0.0016	0.0918	No	1.0442	0.553	0.0018	0.844	No	No
94216_at	Sdhc	succinate dehydrogenase complex, subunit C, integral membrane protein	1.0312	0.5203	0.5801	0.8067	No	1.0012	0.925	0.0475	0.165	No	No
94217_f_at	Cdca3	cell division cycle associated 3	1.0556	0.2308	0.7907	0.5756	No	1.1298	0.0869	0.0006	0.413	No	No
94218_at	Tcp1	t-complex protein 1	1.0067	0.8424	0.2021	0.5364	No	0.8952	0.174	0.388	0.236	No	No
94219_at	Psmb2	proteasome (prosome, macropain) subunit, beta type 2	1.037	0.3615	0.0021	0.6137	No	1.0507	0.289	0.006	0.241	No	No
94220_at	Sh3bgrl3	SH3 domain binding glutamic acid-rich protein-like 3	1.1891	0.0262	0.0023	0.0209	No	1.0477	0.273	0.903	0.335	No	No
94222_at	Igfbp4	insulin-like growth factor binding protein 4	1.0454	0.037	0.0251	0.0204	No	0.9644	0.279	0.009	0.397	No	No
94223_at	Net1	neuroepithelial cell transforming gene 1	0.8269	0.052	0.2013	0.736	No	0.789	0.114	0.809	0.966	No	No
94224_s_at	Ifi203 /// Ifi204 /// LOC192690 /// Ifi205 /// Mnda /// LOC640890 /// LOC677184	interferon activated gene 203 /// interferon activated gene 204 /// similar to interferon activated gene 205 /// interferon activated gene 205 /// myeloid cell nuclear differentiation antigen /// similar to Interferon-activatable protein 205 (IFI-205) (D3 protein) /// similar to Interferon-activatable protein 204 (Ifi-204) (Interferon-inducible protein p204)	0.9132	0.0468	0.0039	0.7356	No	0.9103	0.0802	0.0184	0.65	No	No
94225_at	Atg5	autophagy-related 5 (yeast)	1.0043	0.9325	0.4388	0.2933	No	1.033	0.652	0.292	0.914	No	No
94226_at	Tex264	testis expressed gene 264	0.9057	0.1603	0.007	0.5909	No	0.9805	0.428	0.693	0.0413	No	No
94227_at	Krtap9-1	keratin associated protein 9-1	0.9409	0.3249	0.8738	0.6704	No	0.9722	0.435	0.928	0.557	No	No
94228_at	Xpo1	exportin 1, CRM1 homolog (yeast)	1.0803	0.5363	0.0307	0.4626	No	1.0615	0.239	0.0439	0.91	No	No
94229_at	Tsc22d4	TSC22 domain family 4	0.9073	0.0137	0.0001	0.0456	No	0.9279	0.0447	0	0.22	No	No
94231_at	Ccnd1	cyclin D1	0.9698	0.3499	0.001	0.3489	No	0.9758	0.645	0.0007	0.23	No	No
94232_at	Ccnd1	cyclin D1	1.0235	0.0295	0	0.0002	No	1.0496	0.617	0.353	0.454	No	No
94233_at	1110038F14 Rik	RIKEN cDNA 1110038F14 gene	0.9679	0.2637	0.0084	0.0095	No	1.0119	0.672	0.0235	0.108	No	No
94235_at	Trip12	thyroid hormone receptor interactor 12	0.9661	0.4481	0.0028	0.4788	No	1.0635	0.0065	0.0002	0.0207	Yes	No
94236_at	Nisch	nischarin	1.0414	0.3115	0.0012	0.5005	No	1.0013	0.968	0.0063	0.877	No	No
94237_at	Rpn1	ribophorin I	1.0439	0.3947	0.1021	0.5105	No	0.9788	0.601	0.199	0.133	No	No
94238_at	Prss23	protease, serine, 23	1.0987	0.3595	0.0617	0.2768	No	0.9528	0.678	0.0544	0.926	No	No
94239_at	---	---	1.1005	0.1333	0.0006	0.0586	No	1.027	0.296	0.0005	0.0596	No	No
94240_i_at	Rpl29 /// LOC240110 /// LOC432721 /// LOC433350 /// LOC433706 /// LOC433941 /// LOC622707 /// LOC626772 /// LOC628276 /// LOC634411 /// LOC665032 /// LOC666642 /// LOC667466 /// LOC669451 ///	ribosomal protein L29 /// similar to 60S ribosomal protein L29 /// similar to 60S ribosomal protein L29 /// similar to 60S ribosomal protein L29 /// similar to 60S ribosomal protein L29 /// similar to 60S ribosomal protein L29 /// similar to 60S ribosomal protein L29 /// similar to 60S ribosomal protein L29 /// hypothetical LOC628276 /// similar to 60S ribosomal protein L29 /// similar to 60S ribosomal protein L29 /// similar to 60S ribosomal protein L29 /// similar to 60S ribosomal protein L29 /// similar to 60S ribosomal protein L29 /// hypothetical protein LOC669999 /// similar to 60S ribosomal protein L29 /// similar to 60S ribosomal protein L29 /// similar to 60S ribosomal protein L29 /// hypothetical protein LOC675182 /// similar to 60S ribosomal protein L29	1.1043	0.3017	0.0219	0.9333	No	1.3675	0.0076	0.001	0.0921	Yes	No
94241_at	Coasy	Coenzyme A synthase	0.9785	0.6145	0.4519	0.1928	No	0.9895	0.668	0.0099	0.7	No	No
94242_at	Txnl5	thioredoxin-like 5	1.1554	0.0358	0.3341	0.5585	No	1.0596	0.185	0.072	0.657	No	No
94243_at	Ckap5	cytoskeleton associated protein 5	0.8799	0.1827	0.0181	0.7109	No	0.8808	0.0497	0.004	0.383	No	No
94244_at	Ihpk1	inositol hexaphosphate kinase 1	1.0323	0.5498	0.0079	0.3394	No	0.9676	0.426	0.139	0.0587	No	No
94245_at	---	---	1.129	0.1848	0.0266	0.28	No	1.0827	0.89	0.0045	0.263	No	No
94246_at	Ets2	E26 avian leukemia oncogene 2, 3' domain	1.0283	0.3868	0.0285	0.0814	No	1.0566	0.344	0.0071	0.442	No	No
94247_at	Dsp	desmoplakin	0.9937	0.8938	0.0032	0.0163	No	0.9385	0.162	0.0068	0.0178	No	No
94248_at	Ap1m1	adaptor-related protein complex AP-1, mu subunit 1	0.9729	0.0713	0.0445	0.0045	No	1.0335	0.0128	0.0003	0.0077	No	No
94249_at	Psenen	presenilin enhancer 2 homolog (C. elegans)	1.1058	0.0511	0.0068	0.847	No	1.0835	0.383	0.83	0.999	No	No
94250_at	Eif3s10	eukaryotic translation initiation factor 3, subunit 10 (theta)	0.9869	0.8328	0.991	0.5131	No	0.9907	0.91	0.0761	0.44	No	No
94252_at	Eif2s3x	eukaryotic translation initiation factor 2, subunit 3, structural gene X-linked	1.0351	0.4855	0.5824	0.1408	No	1.0551	0.099	0.442	0.0429	No	No
94253_at	Eif2s1	eukaryotic translation initiation factor 2, subunit 1 alpha	1.023	0.6016	0.288	0.0838	No	1.0051	0.932	0.276	0.331	No	No
94254_at	Clic4	chloride intracellular channel 4 (mitochondrial)	0.9536	0.2044	0.0326	0.9497	No	0.9246	0.0618	0.0012	0.493	No	No

94255_g_at	Clic4	chloride intracellular channel 4 (mitochondrial)	0.9841	0.8162	0.0704	0.1563	No	0.9398	0.388	0.161	0.311	No	No
94256_at	Clic4	chloride intracellular channel 4 (mitochondrial)	1.0085	0.8602	0.0054	0.1195	No	0.9542	0.4	0.0111	0.59	No	No
94257_at	Rraga	Ras-related GTP binding A	1.0565	0.5315	0.8798	0.8559	No	1.093	0.262	0.283	0.646	No	No
94258_at	Arhgdib	Rho, GDP dissociation inhibitor (GDI) beta	0.9194	0.148	0.0497	0.7916	No	1.0296	0.165	0.0464	0.0624	No	No
94259_at	Ptges3	prostaglandin E synthase 3 (cytosolic)	1.062	0.4263	0.5319	0.3288	No	1.0118	0.956	0.0429	0.131	No	No
94260_at	Larp1 /// LOC631268	La ribonucleoprotein domain family, member 1 /// similar to la related protein isoform 1	0.917	0.0276	0.0507	0.1616	No	1.0386	0.415	0.0058	0.195	No	No
94261_at	Thrap3	thyroid hormone receptor associated protein 3	0.9847	0.6637	0.1662	0.191	No	0.9684	0.301	0.0166	0.0249	No	No
94262_at	Thrap3	thyroid hormone receptor associated protein 3	0.9211	0.0426	0.0072	0.1413	No	0.9624	0.112	0.0016	0.859	No	No
94263_f_at	Psmb7	proteasome (prosome, macropain) subunit, beta type 7	1.0183	0.8121	0.1685	0.4796	No	1.0084	0.945	0.0428	0.119	No	No
94264_at	Raf1	v-raf-leukemia viral oncogene 1	0.9664	0.2433	0.5835	0.0036	No	0.9628	0.328	0.0177	0.0016	No	No
94266_at	Hcfc1r1	host cell factor C1 regulator 1 (XPO1-dependent)	0.9554	0.2038	0.0019	0.0347	No	0.9493	0.258	0.0011	0.0153	No	No
94267_i_at	Ubl5	ubiquitin-like 5	1.0635	0.5144	0.9709	0.3047	No	1.1254	0.172	0.0693	0.906	No	No
94268_f_at	Ubl5	ubiquitin-like 5	1.0523	0.2461	0.1595	0.9888	No	1.0458	0.26	0.0077	0.379	No	No
94269_at	Rabac1	Rab acceptor 1 (prenylated)	0.9834	0.6097	0.0007	0.054	No	0.9924	0.83	0.0007	0.145	No	No
94270_at	Krt1-18	keratin complex 1, acidic, gene 18	1.0455	0.314	0.1256	0.8425	No	0.9707	0.612	0.0194	0.105	No	No
94271_at	Dmtf1	cyclin D binding myb-like transcription factor 1	1.0031	0.9292	0.4543	0.5863	No	0.9294	0.589	0.0995	0.65	No	No
94272_g_at	Dmtf1	cyclin D binding myb-like transcription factor 1	0.971	0.4949	0.0396	0.243	No	0.9483	0.477	0.0171	0.338	No	No
94273_at	Yipf5	Yip1 domain family, member 5	1.1007	0.0741	0.0324	0.6568	No	1.093	0.111	0.0097	0.45	No	No
94274_at	Ube2s	ubiquitin-conjugating enzyme E2S	1.0662	0.4068	0.0469	0.8374	No	0.9893	0.705	0.526	0.947	No	No
94275_at	Urod	uroporphyrinogen decarboxylase	0.9726	0.5989	0.1943	0.3914	No	1.0385	0.591	0.265	0.928	No	No
94276_at	Hsd17b12	hydroxysteroid (17-beta) dehydrogenase 12	1.0369	0.6171	0.0864	0.7677	No	1.0315	0.446	0.253	0.191	No	No
94277_at	Mtx1	metaxin 1	1.0203	0.5943	0.013	0.0978	No	1.0126	0.268	0.0006	0.0026	No	No
94278_at	Lcp1	lymphocyte cytosolic protein 1	0.9969	0.9336	0.2128	0.8195	No	1.1037	0.0398	0.0105	0.0837	No	No
94279_at	Atp5d	ATP synthase, H+ transporting, mitochondrial F1 complex, delta subunit	0.9807	0.7698	0.8654	0.8731	No	0.9835	0.573	0.0496	0.762	No	No
94281_at	Cnot2	CCR4-NOT transcription complex, subunit 2	0.9212	0.0627	0.3764	0.9232	No	0.9241	0.0063	0.0103	0.198	Yes	No
94282_at	Asah1	N-acylsphingosine amidohydrolase 1	0.9762	0.6729	0.6982	0.3535	No	0.9393	0.0086	0.0025	0.0015	No	No
94283_at	---	---	0.942	0.5482	0.049	0.9788	No	0.9234	0.165	0.0094	0.438	No	No
94284_at	Cyb5r3	cytochrome b5 reductase 3	0.9941	0.8942	0.0258	0.2137	No	1.0265	0.518	0.0298	0.286	No	No
94285_at	H2-Eb1	histocompatibility 2, class II antigen E beta	0.9716	0.5015	0.7678	0.265	No	1.009	0.729	0.324	0.13	No	No
94286_at	9130011J15 Rik	RIKEN cDNA 9130011J15 gene	1.0531	0.6411	0.0211	0.4138	No	1.0906	0.369	0.0011	0.069	No	No
94288_at	Hist1h1c	histone 1, H1c	0.8673	0.005	0.0174	0.1352	Yes	0.8922	0.151	0.793	0.184	No	No
94289_r_at	Maged2	melanoma antigen, family D, 2	1.0978	0.1941	0.5139	0.5141	No	1.0812	0.278	0.536	0.784	No	No
94290_at	Akt1s1	AKT1 substrate 1 (proline-rich)	0.9819	0.6309	0.1439	0.1516	No	1.0296	0.658	0.0008	0.747	No	No
94291_at	Scgb1a1	secretoglobin, family 1A, member 1 (uteroglobin)	1.0844	0.2253	0.4694	0.2202	No	0.9778	0.61	0.173	0.659	No	No
94292_at	---	---	1.1613	0.1001	0.0019	0.2317	No	1.038	0.373	0.0007	0.605	No	No
94294_at	Ccnb2	cyclin B2	0.9616	0.5126	0.9697	0.2894	No	0.9541	0.124	0.0003	0.0264	No	No
94295_at	LOC669007	similar to General transcription factor II-I (GTFII-I) (TFII-I) (Bruton tyrosine kinase-associated protein 135) (BTK-associated protein 135) (BAP-135)	0.9458	0.1064	0.0426	0.2014	No	0.982	0.702	0.0093	0.677	No	No
94296_s_at	Gtf2i /// LOC669007	general transcription factor II I /// similar to General transcription factor II-I (GTFII-I) (TFII-I) (Bruton tyrosine kinase-associated protein 135) (BTK-associated protein 135) (BAP-135)	0.9488	0.3243	0.005	0.6051	No	0.923	0.074	0.0017	0.723	No	No
94297_at	Fkbp5	FK506 binding protein 5	0.5656	0.0001	0.0204	0.046	Yes	0.6349	0.0001	0.0129	0.0244	Yes	Yes
94298_at	Tsta3	tissue specific transplantation antigen P35B	0.9463	0.0771	0.0023	0.0083	No	0.9616	0.589	0.0162	0.112	No	No
94299_at	Dctn2	dynactin 2	0.9536	0.5024	0.0509	0.5977	No	0.9331	0.229	0.0046	0.0665	No	No
94300_f_at	Dctn2	dynactin 2	0.9634	0.6041	0.0669	0.4209	No	1.0002	0.999	0.29	0.807	No	No
94301_at	Atp6v0e	ATPase, H+ transporting, lysosomal V0 subunit E	0.9358	0.2488	0.0012	0.399	No	0.9522	0.496	0.0008	0.0274	No	No
94302_at	Psmd4	proteasome (prosome, macropain) 26S subunit, non-ATPase, 4	0.9779	0.5461	0.0456	0.6781	No	0.9981	0.749	0.0001	0.0992	No	No
94303_at	Hnrpd	heterogeneous nuclear ribonucleoprotein D	1.1035	0.0608	0.0096	0.0267	No	1.0094	0.658	0.0049	0.0977	No	No
94304_at	Anxa6	annexin A6	1.0937	0.109	0.0022	0.5729	No	1.0452	0.0289	0	0.0887	No	No
94305_at	Col1a1	procollagen, type I, alpha 1	0.8983	0.0385	0.0388	0.035	No	0.8867	0.0026	0.0023	0.0433	Yes	Yes
94307_at	Fbln1	fibulin 1	0.937	0.2221	0.1436	0.2196	No	0.9403	0.36	0.0415	0.228	No	No
94308_at	Fbln1	fibulin 1	0.9417	0.2591	0.1627	0.3549	No	0.9299	0.162	0.691	0.21	No	No
94309_g_at	Fbln1	fibulin 1	0.9265	0.0854	0.0106	0.7474	No	0.9569	0.0357	0.0012	0.637	No	No
94312_at	2210010A19 Rik	RIKEN cDNA 2210010A19 gene	1.0282	0.1717	0.1011	0.0392	No	1.0936	0.0434	0.003	0.375	No	No
94313_at	Snrp1c	U1 small nuclear ribonucleoprotein 1C	0.9964	0.9418	0.0445	0.5924	No	0.9903	0.817	0.0513	0.154	No	No
94316_at	Krt2-6b	keratin complex 2, basic, gene 6b	0.9979	0.966	0.5293	0.2054	No	1.0561	0.172	0.0023	0.568	No	No
94317_at	Krt2-5	keratin complex 2, basic, gene 5	0.9635	0.4054	0.004	0.3157	No	0.9041	0.0377	0.0992	0.216	No	No
94318_at	Apoh	apolipoprotein H	0.996	0.9593	0.0541	0.1703	No	1.0753	0.205	0.177	0.027	No	No
94319_at	Rab18	RAB18, member RAS oncogene family	0.9997	0.9955	0.0356	0.1239	No	1.0143	0.79	0.324	0.0803	No	No
94321_at	Krt1-10	keratin complex 1, acidic, gene 10	0.9266	0.3018	0.5986	0.3496	No	0.9831	0.95	0.0211	0.113	No	No
94322_at	Sqle	squalene epoxidase	1.2482	0.0075	0.2568	0.8731	Yes	1.1923	0.0201	0.105	0.769	No	Yes
94323_at	Nutf2 /// LOC621832 /// LOC668830 /// LOC670887 /// LOC675580	nuclear transport factor 2 /// similar to Nuclear transport factor 2 (NTF-2) /// similar to Nuclear transport factor 2 (NTF-2) /// similar to Nuclear transport factor 2 (NTF-2)	1.064	0.3068	0.0262	0.265	No	1.1297	0.0282	0.253	0.427	No	No
94324_f_at	Hmgcl	3-hydroxy-3-methylglutaryl-Coenzyme A lyase	0.9995	0.9781	0.0308	0.0578	No	0.9967	0.933	0.451	0.155	No	No
94325_at	Hmgcs1	3-hydroxy-3-methylglutaryl-Coenzyme A synthase 1	1.327	0.0011	0.0013	0.1079	Yes	1.3863	0.001	0.743	0.0144	Yes	Yes
94326_r_at	Mrps18a	mitochondrial ribosomal protein S18A	1.0616	0.1831	0.0058	0.0243	No	1.0788	0.242	0.0084	0.329	No	No
94327_at	Mrps18a	mitochondrial ribosomal protein S18A	1.0077	0.5892	0	0.4978	No	1.0204	0.548	0.0006	0.51	No	No
94330_at	Npl	N-acetylneuraminate pyruvate lyase	1.0749	0.3125	0.0084	0.824	No	1.0639	0.387	0.0075	0.369	No	No
94331_at	Stat6	signal transducer and activator of transcription 6	0.9482	0.1251	0.6734	0.7423	No	1.0039	0.915	0.222	0.904	No	No
94332_at	Ets1	E26 avian leukemia oncogene 1, 5' domain	1.0819	0.5606	0.0888	0.6732	No	0.9618	0.611	0.0436	0.675	No	No
94334_f_at	Ina	internexin neuronal intermediate filament protein, alpha	1.1629	0.0139	0.0056	0.1441	No	1.1396	0.0292	0.0909	0.733	No	No
94335_r_at	Ina	internexin neuronal intermediate filament protein, alpha	1	0.9996	0.0012	0.888	No	1.0115	0.918	0.0151	0.496	No	No
94336_at	Otub1	OTU domain, ubiquitin aldehyde binding 1	0.9587	0.3457	0.0361	0.2126	No	1.0124	0.874	0.0158	0.09	No	No
94337_at	Gas2	growth arrest specific 2	1.2613	0.085	0.3549	0.6922	No	1.2658	0.0906	0.0563	0.235	No	No
94338_g_at	Gas2	growth arrest specific 2	1.118	0.0553	0.0757	0.1085	No	1.1117	0.186	0.0344	0.0557	No	No
94339_at	D11Ertd18e	DNA segment, Chr 11, ERATO Doi 18, expressed	1	0.999	0.0387	0.01	No	0.9307	0.279	0.0891	0.0723	No	No
94340_at	Xpot	exportin, tRNA (nuclear export receptor for tRNAs)	1.0379	0.3733	0.0029	0.3056	No	1.027	0.505	0.0047	0.633	No	No
94341_at	Jarid2	jumonji, AT rich interactive domain 2	1.0779	0.1854	0.7336	0.8515	No	1.0667	0.0259	0.094	0.0648	No	No
94343_at	Dnajc3	DnaJ (Hsp40) homolog, subfamily C, member 3	1.0745	0.4413	0.0633	0.4522	No	1.0507	0.205	0.0013	0.213	No	No
94344_at	2410004B18 Rik	RIKEN cDNA 2410004B18 gene	0.9942	0.9299	0.7406	0.3932	No	0.9814	0.64	0.0516	0.142	No	No
94345_at	Il6st	interleukin 6 signal transducer	0.9535	0.6161	0.0419	0.3549	No	0.895	0.0525	0.0044	0.0369	No	No
94346_at	Wtap	Wilms' tumour 1-associating protein	1.101	0.3403	0.0185	0.5691	No	1.1282	0.0753	0.426	0.962	No	No
94347_i_at	Pcmt1	protein-L-isoaspartate (D-aspartate) O-methyltransferase 1	0.9799	0.8536	0.0194	0.6722	No	0.9206	0.22	0.98	0.306	No	No
94348_f_at	Pcmt1	protein-L-isoaspartate (D-aspartate) O-methyltransferase 1	0.9211	0.0421	0.0008	0.4536	No	0.898	0.272	0.0666	0.419	No	No
94349_at	AU024076	expressed sequence AU024076	1.0619	0.1364	0.4401	0.0107	No	1.0704	0.13	0.0447	0.0582	No	No
94350_f_at	Nqo1	NAD(P)H dehydrogenase, quinone 1	1.0548	0.4522	0.2313	0.7807	No	0.9285	0.179	0.0093	0.352	No	No
94351_r_at	Nqo1	NAD(P)H dehydrogenase, quinone 1	1.0543	0.4034	0.2023	0.2161	No	1.0149	0.919	0.0106	0.0483	No	No
94352_at	Mbtd1	mbt domain containing 1	0.8196	0.0174	0.0121	0.2224	No	0.8476	0.0078	0.0002	0.761	Yes	Yes
94353_at	Eif4ebp2	eukaryotic translation initiation factor 4E binding protein 2	1.0299	0.1099	0.0014	0.5516	No	1.0697	0.0252	0.0018	0.0241	No	No
94354_at	Abca1	ATP-binding cassette, sub-family A (ABC1), member 1	0.8205	0.1328	0.0011	0.7753	No	0.8446	0.0077	0.0002	0.0568	Yes	No
94355_at	---	---	1.0238	0.5422	0.001	0.0302	No	1.3446	0.112	0.022	0.263	No	No
94356_at	Trp53bp1	transformation related protein 53 binding protein 1	1.0169	0.6688	0.0022	0.2401	No	1.0485	0.232	0.001	0.773	No	No
94357_at	Slc5a1	solute carrier family 5 (sodium/glucose cotransporter), member 1	0.9447	0.0294	0.4959	0.5194	No	0.8819	0.26	0.592	0.533	No	No
94358_at	Spink4	serine peptidase inhibitor, Kazal type 4	0.9749	0.774	0.636	0.7069	No	1.0195	0.835	0.58	0.8	No	No
94359_at	Plekha1	pleckstrin homology domain containing, family A (phosphoinositide binding specific) member 1	1.0307	0.6077	0.0035	0.1469	No	1.152	0.0488	0.27	0.379	No	No
94360_at	2700029M09 Rik	RIKEN cDNA 2700029M09 gene	1.0411	0.341	0.0645	0.6966	No	1.0358	0.654	0.752	0.475	No	No
94361_at	Ddx21	DEAD (Asp-Glu-Ala-Asp) box polypeptide 21	1.0616	0.2835	0.1154	0.9381	No	1.0329	0.2	0.878	0.101	No	No

94362_at	Nras	neuroblastoma ras oncogene	1.026	0.5698	0.0004	0.4398	No	1.0123	0.763	0.0034	0.783	No	No
94363_at	Bms1l	BMS1-like, ribosome assembly protein (yeast)	0.885	0.0924	0.3279	0.3392	No	0.9856	0.776	0.777	0.831	No	No
94364_at	Glg1	golgi apparatus protein 1	0.9989	0.9875	0.001	0.4738	No	0.9668	0.275	0	0.585	No	No
94365_at	Hint2	histidine triad nucleotide binding protein 2	0.9597	0.2106	0.0188	0.0925	No	0.9981	0.911	0.0049	0.0289	No	No
94366_at	2310079N02Rik	RIKEN cDNA 2310079N02 gene	0.9598	0.4573	0.0341	0.2562	No	0.9719	0.554	0.0286	0.568	No	No
94367_at	Uck2	uridine-cytidine kinase 2	1.1403	0.0123	0.0122	0.9343	No	1.0374	0.249	0.0912	0.714	No	No
94368_at	Supv3l1	suppressor of var1, 3-like 1 (S. cerevisiae)	0.9132	0.1627	0.0466	0.5227	No	0.9441	0.055	0.0006	0.441	No	No
94369_at	Gnpnat1	glucosamine-phosphate N-acetyltransferase 1	1.0813	0.2371	0.0531	0.7007	No	1.0594	0.279	0.0712	0.762	No	No
94370_at	Oit1	oncoprotein induced transcript 1	0.9871	0.725	0.0056	0.0226	No	1.0422	0.091	0.0204	0.0149	No	No
94371_at	Pld2	phospholipase D2	0.9649	0.5382	0.0576	0.9457	No	0.9284	0.11	0.006	0.431	No	No
94372_at	Nudt1	nudix (nucleoside diphosphate linked moiety X)-type motif 1	0.9336	0.212	0.0126	0.0659	No	0.9811	0.66	0.0596	0.0116	No	No
94373_at	Dolpp1	dolichyl pyrophosphate phosphatase 1	1.0101	0.808	0.2101	0.9323	No	0.9868	0.802	0.729	0.522	No	No
94374_at	Wdr13	WD repeat domain 13	0.9929	0.858	0.0159	0.2259	No	0.9445	0.194	0.394	0.622	No	No
94375_at	Hk2	hexokinase 2	0.9744	0.4021	0.0009	0.011	No	1.051	0.379	0.084	0.139	No	No
94376_s_at	Mre11a	meiotic recombination 11 homolog A (S. cerevisiae)	0.9602	0.4281	0.5376	0.146	No	0.9823	0.743	0.0003	0.315	No	No
94377_at	Gng11	guanine nucleotide binding protein (G protein), gamma 11	0.9829	0.4557	0.0403	0.4793	No	1.0008	0.999	0.195	0.444	No	No
94378_at	Rgs16	regulator of G-protein signaling 16	1.0051	0.8771	0.5436	0.2094	No	1.0604	0.084	0.0025	0.047	No	No
94379_at	Kif1b	kinesin family member 1B	0.9415	0.2668	0.0137	0.1846	No	0.8375	0.0159	0.0032	0.223	No	No
94380_at	Ide	insulin degrading enzyme	0.899	0.0978	0.1922	0.3169	No	1.0406	0.0798	0.0001	0.334	No	No
94381_at	Uck1	uridine-cytidine kinase 1	1.0249	0.2911	0.1693	0.392	No	1.0411	0.479	0.103	0.831	No	No
94382_at	1110057K04Rik	RIKEN cDNA 1110057K04 gene	0.9383	0.3769	0.9701	0.6058	No	0.9429	0.33	0.149	0.268	No	No
94383_at	Tfpi2	tissue factor pathway inhibitor 2	0.9481	0.2988	0.177	0.8387	No	0.9571	0.272	0.676	0.0468	No	No
94384_at	Ier3	immediate early response 3	0.9225	0.0848	0.1251	0.1961	No	0.9783	0.357	0.0047	0.0401	No	No
94385_at	Cnih4	cornichon homolog 4 (Drosophila)	0.9838	0.6843	0.2923	0.6233	No	1.0706	0.139	0.458	0.466	No	No
94386_at	Son	Son cell proliferation protein	1.0897	0.0695	0.0003	0.2865	No	1.0133	0.736	0.0497	0.241	No	No
94387_at	Spata5	spermatogenesis associated 5	0.8779	0.0718	0.2214	0.05	No	0.9099	0.109	0.0094	0.0387	No	No
94388_at	Ap3s2	adaptor-related protein complex 3, sigma 2 subunit	1.1078	0.0229	0.001	0.1603	No	1.0074	0.748	0.0001	0.879	No	No
94389_at	LOC546689 /// LOC665903 /// LOC669241 /// LOC675489	similar to LSM5 homolog, U6 small nuclear RNA associated /// hypothetical protein LOC665903 /// hypothetical protein LOC669241 /// hypothetical protein LOC675489	0.9347	0.1261	0.9646	0.0233	No	0.9681	0.775	0.312	0.0881	No	No
94390_at	Akap8	A kinase (PRKA) anchor protein 8	1.0238	0.8372	0.0042	0.7241	No	1.021	0.592	0.0009	0.327	No	No
94391_at	Gjb6	gap junction membrane channel protein beta 6	1.2579	0.0033	0.0002	0.0119	Yes	0.9905	0.494	0.0071	0.0051	No	No
94392_f_at	Ang1	angiogenin, ribonuclease A family, member 1	0.921	0.2227	0.0269	0.6299	No	0.986	0.874	0.118	0.988	No	No
94393_r_at	Elovl2	elongation of very long chain fatty acids (FEN1/Elo2, SUR4/Elo3, yeast)-like 2	1.052	0.2457	0.0066	0.038	No	1.0522	0.552	0.0463	0.466	No	No
94394_at	Rras	Harvey rat sarcoma oncogene, subgroup R	0.9414	0.2506	0.2828	0.0265	No	0.9264	0.195	0.0394	0.149	No	No
94395_at	Fubp1	Far upstream element (FUSE) binding protein 1	1.0148	0.895	0.0029	0.2031	No	1.0242	0.449	0.0017	0.132	No	No
94396_at	Ing1	inhibitor of growth family, member 1	1.0003	0.9913	0.0563	0.2741	No	0.9758	0.512	0.297	0.104	No	No
94397_at	Usp52	ubiquitin specific peptidase 52	0.9708	0.502	0.1677	0.3605	No	0.9068	0.0713	0.266	0.0773	No	No
94398_s_at	Inpp5b	inositol polyphosphate-5-phosphatase B	0.9694	0.0376	0.0015	0.0009	No	0.9485	0.367	0.0192	0.655	No	No
94399_at	Inpp5b	inositol polyphosphate-5-phosphatase B	0.9688	0.5489	0.2398	0.0776	No	0.9246	0.0441	0.0077	0.234	No	No
94400_at	1110051M20Rik	RIKEN cDNA 1110051M20 gene	0.951	0.0028	0.0001	0.0611	Yes	1.0396	0.244	0.0004	0.0056	No	No
94401_s_at	Hemgn	hemogen	1.0086	0.8817	0.8106	0.1332	No	0.9475	0.848	0.417	0.239	No	No
94402_r_at	Hemgn	hemogen	0.9847	0.8833	0.1903	0.2129	No	1.0377	0.551	0.0998	0.0531	No	No
94403_at	Tbc1d14	TBC1 domain family, member 14	0.9592	0.3996	0.5636	0.6238	No	0.9993	0.995	0.525	0.522	No	No
94404_at	Vps45	vacuolar protein sorting 45 (yeast)	1.021	0.6392	0.3964	0.5989	No	1.0506	0.232	0.0924	0.158	No	No
94405_at	Slc6a6	solute carrier family 6 (neurotransmitter transporter, taurine), member 6	0.9514	0.6111	0.0587	0.3795	No	1.0082	0.997	0.0338	0.276	No	No
94406_at	Phtf1	putative homeodomain transcription factor 1	1.0628	0.1172	0.2318	0.4703	No	1.0765	0.171	0.0443	0.878	No	No
94407_at	B3gat3	beta-1,3-glucuronyltransferase 3 (glucuronosyltransferase I)	1.0099	0.812	0.0006	0.4331	No	1.0003	0.887	0.0005	0.435	No	No
94408_at	Nab1	Ngfi-A binding protein 1	0.9772	0.5	0.0567	0.5909	No	1.0225	0.258	0.189	0.837	No	No
94409_at	Ccdc12	coiled-coil domain containing 12	0.9729	0.6531	0.1192	0.4981	No	1.1665	0.085	0.967	0.227	No	No
94410_f_at	Ccdc12	coiled-coil domain containing 12	0.9897	0.8141	0.1602	0.4873	No	0.9848	0.813	0.156	0.13	No	No
94411_at	Cdk2	cyclin-dependent kinase 2	1.0519	0.3904	0.1512	0.5668	No	1.0076	0.946	0.516	0.532	No	No
94412_at	Cdk2	cyclin-dependent kinase 2	0.9837	0.6944	0.0083	0.3196	No	0.9607	0.388	0.0263	0.108	No	No
94413_at	Safb	scaffold attachment factor B	0.9163	0.1483	0.0129	0.0345	No	1.0261	0.99	0.0011	0.277	No	No
94414_at	Otc	ornithine transcarbamylase	1.0507	0.0719	0.1826	0.0716	No	1.0544	0.45	0.331	0.729	No	No
94415_at	Bach1	BTB and CNC homology 1	0.9208	0.3437	0.003	0.8884	No	0.9046	0.288	0.0053	0.718	No	No
94416_at	Rngtt	RNA guanylyltransferase and 5'-phosphatase	1.03	0.4534	0.0356	0.5268	No	1.0428	0.227	0.0142	0.996	No	No
94417_at	2010008E23Rik	RIKEN cDNA 2010008E23 gene	0.9783	0.7327	0.3293	0.2282	No	0.8439	0.0422	0.737	0.268	No	No
94418_at	Elovl6	ELOVL family member 6, elongation of long chain fatty acids (yeast)	1.202	0.0177	0.1472	0.5847	No	1.1823	0.0201	0.276	0.309	No	No
94419_at	Slc19a1	solute carrier family 19 (sodium/hydrogen exchanger), member 1	0.9744	0.4634	0.0071	0.5539	No	0.9932	0.84	0.0004	0.0385	No	No
94420_f_at	Cry1	cryptochrome 1 (photolyase-like)	1.0033	0.9442	0.0015	0.3509	No	0.9668	0.368	0.001	0.721	No	No
94421_r_at	Cry1	cryptochrome 1 (photolyase-like)	1.0157	0.5174	0.014	0.6324	No	0.89	0.234	0.018	0.139	No	No
94422_at	Dnajc13	DnaJ (Hsp40) homolog, subfamily C, member 13	0.9932	0.9393	0.0363	0.8152	No	0.9332	0.114	0.0107	0.105	No	No
94423_at	BC004728	cDNA sequence BC004728	0.9494	0.428	0.0951	0.1943	No	0.9324	0.117	0.0068	0.614	No	No
94424_at	Ankrd13c	ankyrin repeat domain 13c	0.9593	0.1144	0.0003	0.1239	No	0.9693	0.436	0.0274	0.361	No	No
94425_at	Ly86	lymphocyte antigen 86	0.927	0.1842	0.2801	0.3556	No	0.9654	0.467	0.424	0.0627	No	No
94426_at	Thumpd1	THUMP domain containing 1	0.989	0.8058	0.023	0.1972	No	1.0877	0.143	0.0222	0.612	No	No
94427_at	Copg	coatomer protein complex, subunit gamma	0.9626	0.1799	0.3708	0.6253	No	1.0496	0.502	0.0051	0.0083	No	No
94428_at	Ilvbl	ilvB (bacterial acetolactate synthase)-like	0.9072	0.0414	0.0229	0.0625	No	0.9521	0.214	0.0377	0.257	No	No
94429_at	Eef1a2	eukaryotic translation elongation factor 1 alpha 2	0.9506	0.2132	0.1543	0.0099	No	0.9716	0.146	0.0007	0.0007	No	No
94430_at	Zswim1	zinc finger, SWIM domain containing 1	0.9633	0.5604	0.411	0.6962	No	1.0225	0.557	0.0218	0.492	No	No
94431_at	St6gal1	beta galactoside alpha 2,6 sialyltransferase 1	0.9147	0.1573	0.6026	0.1338	No	0.9375	0.593	0.173	0.495	No	No
94432_at	St6gal1	beta galactoside alpha 2,6 sialyltransferase 1	0.9103	0.0375	0.0047	0.8017	No	0.8923	0.222	0.0431	0.579	No	No
94433_at	Slc38a2	solute carrier family 38, member 2	1.039	0.6502	0.0049	0.4305	No	1.0535	0.409	0.0435	0.44	No	No
94434_at	Zfp95	zinc finger protein 95	0.9968	0.7724	0.8679	0.0067	No	0.9825	0.755	0.0588	0.718	No	No
94435_at	D10Ertd438e	DNA segment, Chr 10, ERATO Doi 438, expressed	0.9324	0.0163	0.0012	0.7848	No	0.9736	0.225	0.0029	0.1	No	No
94438_at	Pfkm	phosphofructokinase, muscle	0.8554	0.0039	0.896	0.0258	Yes	0.8783	0.0318	0.167	0.218	No	Yes
94439_at	Osbpl11	oxysterol binding protein-like 11	1.1055	0.4331	0.0025	0.9964	No	0.9542	0.406	0.0004	0.603	No	No
94440_at	AI132487	expressed sequence AI132487	0.9467	0.1175	0.0089	0.027	No	0.9556	0.234	0.0444	0.477	No	No
94441_at	Gpsm3	G-protein signalling modulator 3 (AGS3-like, C. elegans)	0.8997	0.1088	0.015	0.89	No	0.9916	0.824	0.0073	0.523	No	No
94442_s_at	Gpsm3	G-protein signalling modulator 3 (AGS3-like, C. elegans)	0.9677	0.7084	0.5658	0.8669	No	1.1321	0.368	0.687	0.315	No	No
94445_at	Pls3	plastin 3 (T-isoform)	1.0899	0.1291	0.0012	0.2921	No	0.9111	0.24	0.0135	0.275	No	No
94447_at	4732484G22Rik	RIKEN cDNA 4732484G22 gene	1.029	0.7018	0.2114	0.8473	No	0.9389	0.232	0.208	0.224	No	No
94448_at	Bcl10	B-cell leukemia/lymphoma 10	1.0195	0.4348	0.0141	0.0191	No	0.9911	0.801	0.0766	0.0883	No	No

94449_at	Pcdhgb1 /// Pcdhgb2 /// Pcdhgb4 /// Pcdhgb5 /// Pcdhgb6 /// Pcdhgb7 /// Pcdhgb8 /// Pcdhgc3 /// Pcdhgc4 /// Pcdhgc5 /// Pcdhga1 /// Pcdhga2 /// Pcdhga3 /// Pcdhga4 /// Pcdhga5 /// Pcdhga6 /// Pcdhga7 /// Pcdhga8 /// Pcdhga9 /// Pcdhga10 /// Pcdhga11 /// Pcdhga12	protocadherin gamma subfamily B, 1 /// protocadherin gamma subfamily B, 2 /// protocadherin gamma subfamily B, 4 /// protocadherin gamma subfamily B, 5 /// protocadherin gamma subfamily B, 6 /// protocadherin gamma subfamily B, 7 /// protocadherin gamma subfamily B, 8 /// protocadherin gamma subfamily C, 3 /// protocadherin gamma subfamily C, 4 /// protocadherin gamma subfamily C, 5 /// protocadherin gamma subfamily A, 1 /// protocadherin gamma subfamily A, 2 /// protocadherin gamma subfamily A, 3 /// protocadherin gamma subfamily A, 4 /// protocadherin gamma subfamily A, 5 /// protocadherin gamma subfamily A, 6 /// protocadherin gamma subfamily A, 7 /// protocadherin gamma subfamily A, 8 /// protocadherin gamma subfamily A, 9 /// protocadherin gamma subfamily A, 10 /// protocadherin gamma subfamily A, 11 /// protocadherin gamma subfamily A, 12	1.0013	0.9555	0.4758	0.2279	No	0.9935	0.552	0.0051	0.773	No	No
94450_at	Nsun2	NOL1/NOP2/Sun domain family 2	1.206	0.0376	0.1666	0.6815	No	1.1372	0.271	0.702	0.622	No	No
94451_at	Nsun2	NOL1/NOP2/Sun domain family 2	1.0043	0.9089	0.2957	0.6336	No	0.9907	0.82	0.0314	0.161	No	No
94452_g_at	Nsun2	NOL1/NOP2/Sun domain family 2	1.0817	0.021	0.1171	0.7528	No	1.1015	0.0181	0.175	0.0446	No	No
94453_at	1810046J19 Rik	RIKEN cDNA 1810046J19 gene	0.9584	0.4844	0.0167	0.1201	No	1.0235	0.556	0.0033	0.726	No	No
94454_at	Dazap2	DAZ associated protein 2	0.9568	0.3268	0.0694	0.3657	No	0.9722	0.469	0.0009	0.442	No	No
94455_at	Lsm3	LSM3 homolog, U6 small nuclear RNA associated (S. cerevisiae)	1.0691	0.1123	0.0144	0.7712	No	1.0428	0.209	0.0004	0.475	No	No
94456_at	Set	SET translocation	1.3015	0.0134	0.0116	0.5423	No	1.1298	0.075	0.0137	0.624	No	No
94457_at	AL033326	expressed sequence AL033326	1.0128	0.5003	0.0005	0.0187	No	0.9789	0.637	0.0021	0.0283	No	No
94458_at	Casp6	caspase 6	1.0462	0.3033	0.0147	0.2658	No	0.9754	0.743	0.0775	0.413	No	No
94459_at	AI462493	expressed sequence AI462493	1.0301	0.6037	0.022	0.1084	No	1.0069	0.875	0.358	0.0738	No	No
94460_at	Stk38	serine/threonine kinase 38	0.9863	0.7917	0.0242	0.8268	No	0.8917	0.0277	0.0043	0.868	No	No
94461_at	Pbef1	pre-B-cell colony-enhancing factor 1	0.9598	0.3846	0.0005	0.7274	No	0.9846	0.82	0.0001	0.384	No	No
94462_at	Eif2b1	eukaryotic translation initiation factor 2B, subunit 1 (alpha)	0.9238	0.2504	0.0889	0.08	No	0.9395	0.105	0.0452	0.0505	No	No
94463_at	Clcn3	chloride channel 3	0.9302	0.2252	0.8372	0.5011	No	0.9307	0.248	0.0991	0.674	No	No
94464_at	Clcn3	chloride channel 3	0.9521	0.6672	0.0015	0.4767	No	0.9481	0.469	0.0016	0.648	No	No
94465_g_at	Clcn3	chloride channel 3	0.87	0.0386	0.0008	0.4118	No	0.8979	0.0804	0.0003	0.258	No	No
94466_f_at	Cebpz	CCAAT/enhancer binding protein zeta	0.9543	0.3125	0.0018	0.0908	No	0.972	0.0349	0	0.461	No	No
94467_at	Cebpz	CCAAT/enhancer binding protein zeta	0.9415	0.3937	0.0116	0.7843	No	1.0041	0.972	0.0126	0.445	No	No
94468_at	Mat2b	methionine adenosyltransferase II, beta	0.9945	0.9001	0.0471	0.2625	No	1.0397	0.317	0.0018	0.181	No	No
94469_at	Mat2b	methionine adenosyltransferase II, beta	1.0097	0.7655	0.0111	0.5957	No	1.0754	0.181	0.44	0.176	No	No
94470_i_at	Slc30a5	solute carrier family 30 (zinc transporter), member 5	0.9359	0.342	0.5187	0.1678	No	0.9113	0.0504	0.0185	0.0135	No	No
94471_r_at	Slc30a5	solute carrier family 30 (zinc transporter), member 5	1.0296	0.3409	0.0359	0.1004	No	0.9896	0.903	0.0459	0.456	No	No
94472_at	Spcs1	signal peptidase complex subunit 1 homolog (S. cerevisiae)	1.054	0.0576	0.3444	0.5263	No	1.0183	0.613	0.249	0.574	No	No
94473_at	Pttg1ip	pituitary tumor-transforming 1 interacting protein	1.0559	0.4161	0.036	0.4919	No	0.9751	0.643	0.391	0.0217	No	No
94476_at	Josd3	Josephin domain containing 3	1.0867	0.2365	0.2127	0.4404	No	1.014	0.836	0.159	0.781	No	No
94477_at	---	---	1.0304	0.5524	0.1439	0.227	No	0.9809	0.474	0.0076	0.0351	No	No
94478_at	Rab5a	RAB5A, member RAS oncogene family	1.0266	0.5881	0.0025	0.2603	No	1.0482	0.367	0.111	0.158	No	No
94479_at	D1Ertd161e	DNA segment, Chr 1, ERATO Doi 161, expressed	1.1068	0.0896	0.0193	0.2994	No	0.9987	0.794	0.0067	0.0615	No	No
94480_at	D1Ertd161e	DNA segment, Chr 1, ERATO Doi 161, expressed	0.9803	0.5957	0.1568	0.9924	No	0.9204	0.122	0.154	0.319	No	No
94481_at	Ugp2	UDP-glucose pyrophosphorylase 2	1.0226	0.7465	0.0802	0.5364	No	1.0046	0.891	0.0726	0.313	No	No
94482_at	Csnk2a2	casein kinase II, alpha 2, polypeptide	0.912	0.1217	0.312	0.4703	No	0.9151	0.134	0.0475	0.196	No	No
94483_at	Csnk2a2	casein kinase II, alpha 2, polypeptide	1.1021	0.0281	0.0001	0.321	No	1.1198	0.0123	0	0.136	No	No
94484_at	Hbs1l	Hbs1-like (S. cerevisiae)	0.9374	0.4791	0.9827	0.279	No	0.9561	0.35	0.198	0.189	No	No
94485_at	Peci	peroxisomal delta3, delta2-enoyl-Coenzyme A isomerase	0.9823	0.6014	0.0571	0.0143	No	0.9553	0.369	0.0167	0.0064	No	No
94486_at	Lrrc59	leucine rich repeat containing 59	1.1232	0.0121	0.025	0.6278	No	1.0763	0.017	0.0133	0.0389	No	No
94488_at	1110059P08 Rik	RIKEN cDNA 1110059P08 gene	1.0131	0.7931	0.1679	0.3365	No	1.0018	0.908	0.0065	0.324	No	No
94489_at	Ptp4a1 /// LOC433406 /// LOC627166 /// LOC627200 /// LOC667723 /// LOC669215	protein tyrosine phosphatase 4a1 /// protein tyrosine phosphatase 4a1-like /// protein tyrosine phosphatase 4a1 pseudogene /// protein tyrosine phosphatase 4a1-like /// similar to protein tyrosine phosphatase 4a1 /// similar to protein tyrosine phosphatase 4a1	1.0124	0.8972	0.2351	0.8078	No	1.0239	0.729	0.0309	0.996	No	No
94490_at	Cnot8	CCR4-NOT transcription complex, subunit 8	0.9825	0.8056	0.6283	0.434	No	0.9334	0.356	0.496	0.505	No	No
94491_at	Pex13	peroxisomal biogenesis factor 13	0.9283	0.348	0.0301	0.7269	No	0.9932	0.723	0.0475	0.0778	No	No
94492_at	Dstn	destrin	1.0379	0.4746	0.1336	0.4696	No	0.9967	0.997	0.542	0.354	No	No
94493_at	Cldn3	claudin 3	1.114	0.066	0.0246	0.2126	No	1.0262	0.568	0.013	0.687	No	No
94494_at	Farslb	phenylalanine-tRNA synthetase-like, beta subunit	1.1751	0.1634	0.0397	0.0818	No	1.0861	0.203	0.503	0.0835	No	No
94495_at	1810008A14 Rik	RIKEN cDNA 1810008A14 gene	1.0312	0.6129	0.1957	0.6063	No	1.0327	0.674	0.161	0.827	No	No
94499_at	Mgea5	meningioma expressed antigen 5 (hyaluronidase)	1.0641	0.2707	0.0001	0.9939	No	1.0235	0.76	0.0005	0.722	No	No
94501_at	Sgpp1	sphingosine-1-phosphate phosphatase 1	1.0279	0.6658	0.1291	0.3054	No	1.0744	0.15	0.554	0.039	No	No
94502_at	D13Wsu50e	DNA segment, Chr 13, Wayne State University 50, expressed	1.0974	0.0446	0.4044	0.0567	No	1.0239	0.514	0.0175	0.0989	No	No
94503_at	Rab8a	RAB8A, member RAS oncogene family	1.0541	0.3255	0.3095	0.8417	No	1.0677	0.207	0.0617	0.418	No	No
94504_at	4930570C03 Rik	RIKEN cDNA 4930570C03 gene	1.0025	0.9434	0.7189	0.018	No	1.0516	0.249	0.0242	0.654	No	No
94505_at	Peli1	pellino 1	0.9859	0.8055	0.002	0.3187	No	0.929	0.431	0.0258	0.81	No	No
94506_at	Nudt21	nudix (nucleoside diphosphate linked moiety X)-type motif 21	1.107	0.082	0.4303	0.9089	No	1.0575	0.246	0.106	0.667	No	No
94507_at	Acsl1	acyl-CoA synthetase long-chain family member 1	0.8635	0.0221	0.0405	0.0203	No	0.8776	0.0666	0.0648	0.0165	No	No
94508_at	Tmem126a	transmembrane protein 126A	1.1222	0.1479	0.4559	0.2636	No	0.9801	0.762	0.153	0.133	No	No
94509_at	Ncbp2	nuclear cap binding protein subunit 2	1.0831	0.2867	0.4427	0.3794	No	1.0222	0.85	0.0375	0.57	No	No
94510_at	Wdr77	WD repeat domain 77	1.0086	0.8677	0.7731	0.3856	No	1.0108	0.6	0.012	0.411	No	No
94511_at	LOC636537	similar to signal sequence receptor, alpha	1.0975	0.3776	0.2429	0.642	No	1.0867	0.23	0.484	0.225	No	No
94512_f_at	Arcn1	archain 1	1.0554	0.1191	0.003	0.4533	No	1.1388	0.179	0.0131	0.976	No	No
94513_r_at	Arcn1	Archain 1	0.9336	0.068	0	0.202	No	0.9565	0.345	0.0002	0.359	No	No
94514_s_at	Arcn1	archain 1	0.9984	0.9773	0.0116	0.2204	No	0.8604	0.0036	0.184	0.0039	No	No
94515_at	Sqrdl	sulfide quinone reductase-like (yeast)	0.9674	0.1339	0.0266	0.3642	No	1.0262	0.554	0.0357	0.525	No	No
94516_f_at	Penk1	preproenkephalin 1	1.0391	0.2797	0.0008	0.8805	No	1.0018	0.978	0.001	0.53	No	No
94517_r_at	Penk1	preproenkephalin 1	0.9606	0.6528	0.0009	0.0462	No	0.8743	0.0841	0.189	0.204	No	No
94518_at	Aurkaip1	aurora kinase A interacting protein 1	1.0102	0.6104	0.0025	0.4344	No	1.0082	0.88	0.106	0.447	No	No
94520_at	2610003J06 Rik	RIKEN cDNA 2610003J06 gene	1.0035	0.9506	0.1821	0.7819	No	1.0308	0.559	0.0216	0.449	No	No
94521_at	Cdkn2d	cyclin-dependent kinase inhibitor 2D (p19, inhibits CDK4)	0.9871	0.8022	0.6687	0.3329	No	1.0257	0.897	0.107	0.4	No	No
94522_at	Dctn3	dynactin 3	0.9911	0.8533	0.7119	0.4711	No	0.9912	0.853	0.411	0.306	No	No
94524_at	Dap3	death associated protein 3	0.9851	0.5106	0.4698	0.0647	No	0.952	0.121	0.0005	1	No	No
94526_at	D10Ertd214 e	DNA segment, Chr 10, ERATO Doi 214, expressed	1.0555	0.0962	0.743	0.1596	No	1.0867	0.113	0.66	0.319	No	No
94527_at	AI413782	expressed sequence AI413782	0.986	0.6061	0.0511	0.3162	No	1.031	0.744	0.126	0.138	No	No
94528_at	Nubp1	nucleotide binding protein 1	1.0113	0.7823	0.2273	0.8105	No	0.9874	0.696	0.0031	0.985	No	No
94530_at	Eif2b2	eukaryotic translation initiation factor 2B, subunit 2 beta	0.9929	0.7092	0.0024	0.5871	No	1.0015	0.963	0.471	0.476	No	No
94531_at	Coq9	coenzyme Q9 homolog (yeast)	0.9787	0.5365	0.6671	0.0244	No	0.9028	0.12	0.0742	0.0596	No	No
94532_at	Atp6v1e1	VATPase, H+ transporting, lysosomal V1 subunit E1	1.0098	0.7491	0.769	0.9161	No	0.9815	0.282	0.898	0.123	No	No
94534_at	Idh3a	isocitrate dehydrogenase 3 (NAD+) alpha	0.98	0.5809	0.0172	0.0397	No	0.9946	0.882	0.77	0.307	No	No
94535_at	Add1	adducin 1 (alpha)	0.9688	0.5504	0.1454	0.8624	No	0.9678	0.512	0.739	0.324	No	No

94536_s_at	2900073G15 Rik /// Mylc2b	RIKEN cDNA 2900073G15 gene /// myosin light chain, regulatory B	0.9977	0.9301	0.0007	0.4005	No	0.9677	0.526	0.0053	0.164	No	No
94537_at	Mylc2b	myosin light chain, regulatory B	1.0045	0.8948	0.1163	0.2413	No	0.9792	0.572	0.18	0.557	No	No
94539_f_at	Cyp2d9 /// 9030605E09 Rik	cytochrome P450, family 2, subfamily d, polypeptide 9 /// RIKEN cDNA 9030605E09 gene	1.0213	0.5518	0.8343	0.861	No	0.953	0.156	0.102	0.368	No	No
94540_at	Cyp2d26	cytochrome P450, family 2, subfamily d, polypeptide 26	0.9518	0.3327	0.1862	0.2334	No	0.9433	0.35	0.58	0.195	No	No
94541_at	Ndufb4	NADH dehydrogenase (ubiquinone) 1 beta subcomplex 4	1.1782	0.1817	0.715	0.167	No	1.1929	0.105	0.592	0.0528	No	No
94542_at	Mbtd1	mbt domain containing 1	0.9007	0.167	0.0038	0.1617	No	0.8921	0.165	0.0109	0.286	No	No
94543_at	1810042K04 Rik	RIKEN cDNA 1810042K04 gene	1.0845	0.1629	0.7321	0.0561	No	0.9632	0.515	0.0561	0.777	No	No
94545_at	Rtn1	reticulon 1	1.0819	0.3552	0.0077	0.3526	No	1.0533	0.322	0.002	0.306	No	No
94548_at	Mfsd1	major facilitator superfamily domain containing 1	0.9608	0.1888	0.9924	0.0656	No	0.9696	0.332	0.843	0.453	No	No
94549_at	Mfsd1	major facilitator superfamily domain containing 1	1.0076	0.7857	0.0186	0.4844	No	0.962	0.312	0.832	0.874	No	No
94550_at	Snx1	sorting nexin 1	0.9588	0.4745	0.094	0.1088	No	0.9354	0.232	0.151	0.0968	No	No
94551_at	Atg4b	autophagy-related 4B (yeast)	1.0302	0.0757	0.2703	0.387	No	1.0051	0.791	0.0162	0.17	No	No
94552_at	Pcbp1	poly(rC) binding protein 1	1.1174	0.0762	0.0175	0.7794	No	1.1246	0.0586	0.38	0.469	No	No
94554_at	Trappc5	trafficking protein particle complex 5	0.9767	0.5792	0.2678	0.0631	No	0.9472	0.356	0.0743	0.331	No	No
94555_at	Rnf167	ring finger protein 167	0.9855	0.7289	0.007	0.399	No	0.9856	0.476	0.0055	0.465	No	No
94556_at	Snx10	sorting nexin 10	0.8772	0.0596	0.0481	0.1793	No	0.942	0.189	0.307	0.123	No	No
94557_at	Gtf3a	general transcription factor III A	1.0226	0.7779	0.023	0.0723	No	1.1486	0.0653	0.0338	0.0077	No	No
94558_g_at	Gtf3a	general transcription factor III A	1.0242	0.4135	0.0237	0.8479	No	0.9882	0.926	0.0561	0.667	No	No
94559_at	Gtf3a	general transcription factor III A	1.0437	0.2995	0.307	0.4276	No	1.0645	0.0083	0.0001	0.0157	Yes	No
94561_at	Lima1	LIM domain and actin binding 1	0.9237	0.0589	0.8689	0.9025	No	0.9447	0.155	0.913	0.817	No	No
94562_at	Gnpat	glyceronephosphate O-acyltransferase	1.0398	0.5937	0.2844	0.8525	No	1.0942	0.303	0.934	0.35	No	No
94563_at	Afm	afamin	1.0442	0.4909	0.2992	0.8298	No	1.0847	0.218	0.696	0.989	No	No
94564_at	Sult4a1	sulfotransferase family 4A, member 1	0.9879	0.7872	0.8921	0.7712	No	0.9853	0.795	0.178	0.737	No	No
94565_at	Itpr2	inositol 1,4,5-triphosphate receptor 2	1.0092	0.88	0.5599	0.4105	No	1.0465	0.635	0.99	0.77	No	No
94566_at	Gpr132	G protein-coupled receptor 132	0.9993	0.9769	0.0156	0.0159	No	1.0364	0.264	0.0613	0.156	No	No
94568_at	Rgs6	regulator of G-protein signaling 6	1.0077	0.8472	0.0111	0.8071	No	1.0176	0.474	0.0009	0.518	No	No
94571_at	V1ra1	vomeronasal 1 receptor, A1	1.0165	0.8159	0.1546	0.424	No	1.0932	0.257	0.126	0.143	No	No
94618_at	Foxb2	forkhead box B2	0.9822	0.7305	0.0564	0.9	No	1.0203	0.747	0.522	0.748	No	No
94619_at	Grid1	glutamate receptor, ionotropic, delta 1	0.9675	0.4501	0.0132	0.7967	No	0.8702	0.0384	0.3	0.0788	No	No
94621_at	Foxd2	forkhead box D2	0.9902	0.7594	0.0041	0.1958	No	0.8365	0.023	0.0124	0.112	No	No
94622_at	Grm8	glutamate receptor, metabotropic 8	1.004	0.9599	0.3879	0.4385	No	0.9987	0.992	0.462	0.0796	No	No
94623_at	Enam	enamelin	0.9132	0.0845	0.163	0.1357	No	0.9052	0.0955	0.0782	0.0315	No	No
94624_at	Stk22s1	serine/threonine kinase 22 substrate 1	0.9889	0.8493	0.0068	0.6222	No	1.0382	0.821	0.174	0.181	No	No
94625_at	Otog	otogelin	1.0029	0.9546	0.003	0.8059	No	0.9903	0.97	0.0017	0.599	No	No
94626_at	Cntnap1	contactin associated protein 1	0.9314	0.4904	0.2534	0.5995	No	0.8602	0.0341	0.0016	0.0308	No	No
94627_at	Figla	folliculogenesis specific basic helix-loop-helix	1.0243	0.3879	0.0433	0.1843	No	1.2526	0.326	0.501	0.863	No	No
94628_r_at	Abpa	androgen binding protein alpha	1.0356	0.5512	0.6046	0.6164	No	1.0461	0.662	0.44	0.113	No	No
94630_at	Zfpn1a2	zinc finger protein, subfamily 1A, 2 (Helios)	0.9744	0.7	0.0068	0.664	No	0.9353	0.287	0.0027	0.72	No	No
94631_at	P2rxl1	purinergic receptor P2X-like 1, orphan receptor	1.0286	0.4965	0.1348	0.0894	No	1.0792	0.152	0.231	0.583	No	No
94632_at	Pbsn	probasin	1.0161	0.6563	0.3324	0.1753	No	1.1034	0.0944	0.73	0.0744	No	No
94633_at	Gcg	glucagon	0.9963	0.9536	0.0214	0.4161	No	0.9195	0.37	0.627	0.305	No	No
94634_at	Tacr2	tachykinin receptor 2	1.0254	0.7784	0.1303	0.442	No	0.9366	0.35	0.0112	0.357	No	No
94635_at	Tacr1	tachykinin receptor 1	1.0028	0.9402	0.6419	0.2073	No	1.05	0.325	0.699	0.177	No	No
94636_at	Hoxa13	homeo box A13	0.9525	0.3719	0.0059	0.1163	No	0.9882	0.704	0.0149	0.361	No	No
94637_at	Sema4b	sema domain, immunoglobulin domain (Ig), transmembrane domain (TM) and short cytoplasmic domain, (semaphorin) 4B	0.9967	0.9363	0.009	0.0967	No	1.0074	0.882	0.0234	0.745	No	No
94638_at	Hist2h2bb	histone 2, H2bb	0.9748	0.7045	0.1067	0.2422	No	0.9614	0.785	0.068	0.208	No	No
94639_at	Svs2	seminal vesicle protein, secretion 2	0.9952	0.9109	0.0402	0.5926	No	1.0371	0.563	0.388	0.522	No	No
94640_at	Cdh9	cadherin 9	0.9903	0.8306	0.8471	0.9578	No	1.0186	0.836	0.027	0.927	No	No
94641_at	Cdh10	cadherin 10	0.9462	0.6234	0.1565	0.6825	No	1.0532	0.37	0.56	0.414	No	No
94642_at	Gdi2	guanosine diphosphate (GDP) dissociation inhibitor 2	0.9723	0.1204	0.0001	0.0175	No	1.1305	0.383	0.491	0.662	No	No
94643_at	Pvr	poliovirus receptor	0.974	0.3309	0.349	0.6254	No	0.989	0.642	0.0881	0.0075	No	No
94644_at	Bche	butyrylcholinesterase	1.0437	0.1273	0.6235	0.1949	No	0.9742	0.404	0.0182	0.0038	No	No
94645_at	Gabra3	gamma-aminobutyric acid (GABA-A) receptor, subunit alpha 3	1.0201	0.7655	0.0743	0.7839	No	1.026	0.76	0.647	0.492	No	No
94657_at	D8Wsu26e	DNA segment, Chr 8, Wayne State University 26, expressed	0.8943	0.1774	0.1202	0.5521	No	0.8011	0.361	0.433	0.517	No	No
94662_at	4833441D16 Rik	RIKEN cDNA 4833441D16 gene	0.9896	0.8424	0.0053	0.7815	No	1.0326	0.0879	0.0001	0.0136	No	No
94663_at	Slco5a1	solute carrier organic anion transporter family, member 5A1	1.033	0.4808	0.0024	0.0449	No	1.0981	0.0152	0.0002	0.0064	No	No
94664_at	Irs3	insulin receptor substrate 3	0.9414	0.1549	0.1391	0.3168	No	1.0011	0.98	0.825	0.722	No	No
94665_at	Pla2g5	phospholipase A2, group V	0.9399	0.1274	0.0017	0.445	No	0.982	0.576	0.0015	0.0151	No	No
94667_at	AA408396	expressed sequence AA408396	1.0283	0.5242	0.0194	0.4023	No	1.0525	0.409	0.0196	0.513	No	No
94668_at	D3Wsu167e	DNA segment, Chr 3, Wayne State University 167, expressed	1.0699	0.4861	0.2431	0.7785	No	1.206	0.139	0.945	0.188	No	No
94674_at	D1Wsu158e	DNA segment, Chr 1, Wayne State University 158, expressed	0.9387	0.3287	0.2723	0.5372	No	0.9238	0.0956	0.0466	0.2	No	No
94683_at	AA516738	expressed sequence AA516738	1.0269	0.6878	0.052	0.3967	No	1.0534	0.291	0.35	0.266	No	No
94684_at	Denr	Density-regulated protein	0.9802	0.7945	0.7649	0.5186	No	1.1053	0.0693	0.0963	0.0231	No	No
94685_at	---	---	0.994	0.9215	0.9155	0.4322	No	1.0607	0.533	0.542	0.318	No	No
94686_at	C76533	expressed sequence C76533	1.0151	0.2736	0.0074	0.0131	No	0.9935	0.876	0.179	0.12	No	No
94687_at	Foxb1	forkhead box B1	1.0379	0.6518	0.1702	0.078	No	1.1347	0.173	0.4	0.123	No	No
94688_at	Mxd1	MAX dimerization protein 1	1.0022	0.9537	0.3414	0.5272	No	0.896	0.175	0.992	0.736	No	No
94689_at	C79248	expressed sequence C79248	0.732	0.0395	0.0004	0.1671	No	0.7866	0.0156	0.0003	0.0574	No	No
94690_at	C79490	expressed sequence C79490	1.0035	0.9245	0.0212	0.4772	No	1.0672	0.342	0.412	0.369	No	No
94691_at	---	---	0.964	0.5307	0.168	0.2057	No	0.9125	0.0631	0.0076	0.0656	No	No
94692_at	Gpx5	glutathione peroxidase 5	1.0701	0.3743	0.0271	0.8248	No	1.0496	0.516	0.0033	0.153	No	No
94693_at	Ogfrl1	Opioid growth factor receptor-like 1	0.9537	0.0891	0.9173	0.2316	No	0.8512	0.0075	0.0101	0.188	Yes	No
94694_at	Pcsk1	proprotein convertase subtilisin/kexin type 1	0.9963	0.9179	0.7149	0.1719	No	0.9138	0.242	0.0865	0.36	No	No
94695_at	C81521	expressed sequence C81521	1.0837	0	0	0	No	0.9169	0.163	0.0008	0.369	No	No
94696_at	Ccr4	chemokine (C-C motif) receptor 4	1.0459	0.5238	0.1984	0.3289	No	1.0442	0.629	0.775	0.417	No	No
94697_at	Fgr	Gardner-Rasheed feline sarcoma viral (Fgr) oncogene homolog	1.0386	0.3967	0.0529	0.2477	No	0.9964	0.946	0.103	0.586	No	No
94698_at	Fli1	Friend leukemia integration 1	0.9641	0.616	0.5088	0.9243	No	0.8926	0.302	0.581	0.145	No	No
94699_at	Ipp	IAP promoted placental gene	1.0129	0.7749	0.2658	0.9849	No	0.9471	0.218	0.0555	0.884	No	No
94700_at	Cga	glycoprotein hormones, alpha subunit	1.1947	0.0014	0.002	0.0005	No	1.1423	0.052	0.0184	0.0066	No	No
94701_at	Pde6b	phosphodiesterase 6B, cGMP, rod receptor, beta polypeptide	0.9891	0.8472	0.4516	0.2229	No	0.9887	0.852	0.803	0.802	No	No
94702_at	Tgfbr2	transforming growth factor, beta receptor II	1.0155	0.8916	0.0419	0.6322	No	0.8103	0.401	0.225	0.555	No	No
94703_at	Krt2-17	keratin complex 2, basic, gene 17	1.0033	0.9593	0.4034	0.0965	No	1.1515	0.219	0.336	0.417	No	No
94704_at	Wisp2	WNT1 inducible signaling pathway protein 2	0.9865	0.831	0.008	0.6901	No	1.0726	0.0939	0.0037	0.0238	No	No
94705_at	Eya1	eyes absent 1 homolog (Drosophila)	1.0397	0.4461	0.0424	0.4908	No	1.0058	0.839	0.193	0.167	No	No
94706_s_at	Eya1	eyes absent 1 homolog (Drosophila)	0.9935	0.877	0.1112	0.4789	No	1.0697	0.0228	0.279	0.11	No	No
94707_s_at	Amelx	amelogenin X chromosome	1.0013	0.9815	0.6508	0.9534	No	1.0687	0.27	0.0101	0.614	No	No
94708_at	Ptprn2	protein tyrosine phosphatase, receptor type, N polypeptide 2	0.9154	0.134	0.6328	0.6807	No	0.9591	0.475	0.0522	0.844	No	No
94709_at	Gcm2	glial cells missing homolog 2 (Drosophila)	1.0218	0.73	0.3842	0.0688	No	1.0661	0.467	0.919	0.108	No	No
94710_g_at	Gcm2	glial cells missing homolog 2 (Drosophila)	0.9461	0.4674	0.2304	0.7058	No	1.0783	0.186	0.0193	0.802	No	No
94711_at	Gcm1	glial cells missing homolog 1 (Drosophila)	1.0778	0.2359	0.3012	0.0723	No	1.081	0.121	0.0007	0.918	No	No
94712_at	Vegfc	vascular endothelial growth factor C	1.0338	0.6387	0.1167	0.5816	No	1.0606	0.237	0.0135	0.6	No	No
94713_at	Myo7a	myosin VIIa	0.9201	0.0017	0.0007	0.0119	Yes	0.964	0.143	0.0039	0.0731	No	No
94714_at	Klk1b3	kallikrein 1-related peptidase b3	0.9056	0.4117	0.3196	0.3298	No	1.004	0.919	0.0094	0.68	No	No
94715_at	Cyp1a1	cytochrome P450, family 1, subfamily a, polypeptide 1	0.9202	0.1952	0.7972	0.1291	No	0.8981	0.0848	0.009	0.735	No	No
94716_f_at	Klk1b9 /// Klk1 /// Klk1b1 /// Klk1b4 /// 1700127D06 Rik /// LOC668642	kallikrein 1-related peptidase b9 /// kallikrein 1 /// kallikrein 1-related peptidase b1 /// kallikrein 1-related pepidase b4 /// RIKEN cDNA 1700127D06 gene /// similar to Glandular kallikrein K3 precursor (Tissue kallikrein-3) (mGK-3) (7S nerve growth factor gamma chain) (Gamma-NGF)	1.0312	0.3443	0.9873	0.2039	No	1.0017	0.948	0.796	0.0796	No	No

94717_f_at	Ifna2	interferon alpha family, gene 2	1.0865	0.1775	0.3092	0.1407	No	1.1725	0.077	0.639	0.46	No	No
94718_at	Hist1h1e	histone 1, H1e	0.9531	0.2507	0.2034	0.305	No	0.9191	0.364	0.094	0.297	No	No
94719_at	Kcna6	potassium voltage-gated channel, shaker-related, subfamily, member 6	1.0035	0.9475	0.3038	0.4801	No	1.055	0.32	0.029	0.131	No	No
94720_at	Ets1	E26 avian leukemia oncogene 1, 5' domain	0.9793	0.2314	0.0303	0.0539	No	0.9559	0.232	0.732	0.0218	No	No
94721_at	Dspp /// LOC666279	dentin sialophosphoprotein /// similar to Dentin sialophosphoprotein precursor (Dentin matrix protein 3) (DMP-3)	0.9851	0.6684	0.0194	0.1071	No	0.9698	0.577	0.0775	0.664	No	No
94722_f_at	V2r10 /// V2r11	vomeronasal 2, receptor, 10 /// vomeronasal 2, receptor, 11	1.0004	0.9882	0.9781	0.1403	No	1.0971	0.214	0.0308	0.242	No	No
94723_at	Nat1	N-acetyltransferase 1 (arylamine N-acetyltransferase)	0.8914	0.1533	0.3885	0.4649	No	1.0194	0.729	0.0694	0.258	No	No
94724_at	Mmp10	matrix metallopeptidase 10	0.977	0.7557	0.8754	0.8348	No	1.0886	0.204	0.007	0.57	No	No
94725_f_at	Igkv4-91	immunoglobulin kappa chain variable 4-91	1.0063	0.908	0.1466	0.6241	No	1.0626	0.287	0.253	0.487	No	No
94726_at	Tcrb-V13 /// LOC621968	T-cell receptor beta, variable 13 /// similar to T-cell receptor beta chain V region E1 precursor	0.956	0.6693	0.1793	0.8461	No	1.1141	0.132	0.68	0.166	No	No
94727_f_at	Tcstv1	2-cell-stage, variable group, member 1	0.9389	0.1349	0.0048	0.0684	No	1.0522	0.506	0.0343	0.976	No	No
94728_f_at	Mcpt1	mast cell protease 1	0.9959	0.9356	0.7204	0.8158	No	1.0479	0.452	0.0569	0.522	No	No
94729_r_at	Mcpt1	mast cell protease 1	1.0035	0.9654	0.0051	0.9955	No	0.9954	0.602	0.0002	0.0583	No	No
94730_at	Crisp3	cysteine-rich secretory protein 3	0.9919	0.8792	0.3756	0.7968	No	1.0275	0.584	0.0938	0.0962	No	No
94731_at	Stac	src homology three (SH3) and cysteine rich domain	1.0796	0.0013	0.0003	0.5179	Yes	1.0482	0.517	0.469	0.563	No	No
94732_at	Rptn	repetin	1.031	0.1193	0.1803	0.0034	No	0.9974	0.842	0.0289	0.241	No	No
94733_at	Abcb4	ATP-binding cassette, sub-family B (MDR/TAP), member 4	1.0657	0.118	0.5003	0.8151	No	1.045	0.513	0.645	0.544	No	No
94734_at	Orm2	orosomucoid 2	0.9711	0.565	0.1644	0.6236	No	1.0039	0.962	0.774	0.951	No	No
94735_s_at	Mcpt9 /// Cma2	mast cell protease 9 /// chymase 2, mast cell	1.0419	0.0406	0	0.0042	No	0.955	0.759	0.0055	0.326	No	No
94736_at	Lyst	lysosomal trafficking regulator	1.0942	0.3821	0.7113	0.9242	No	1.0558	0.501	0.577	0.727	No	No
94737_at	Adcy8	adenylate cyclase 8	1.0374	0.1795	0.0041	0.0439	No	1.0687	0.049	0.0013	0.0533	No	No
94738_s_at	Defcr-rs10 /// Defcr-rs2	defensin related cryptdin, related sequence 10 /// defensin related cryptdin, related sequence 2	1.045	0.0795	0.4635	0.7803	No	0.9758	0.777	0.531	0.779	No	No
94739_at	Trpc1	transient receptor potential cation channel, subfamily C, member 1	0.9967	0.9501	0.1204	0.4091	No	0.9783	0.718	0.711	0.18	No	No
94740_g_at	Trpc1	transient receptor potential cation channel, subfamily C, member 1	0.9212	0.148	0.7748	0.9245	No	0.9351	0.359	0.997	0.569	No	No
94741_at	Tesp2	testicular serine protease 2	1.0366	0.4119	0.2027	0.5162	No	1.2065	0.0996	0.0375	0.836	No	No
94743_f_at	LOC214403	complement factor H-related protein	0.9261	0.1211	0.048	0.1212	No	0.8203	0.0182	0.877	0.0446	No	No
94744_at	Klrb1b	killer cell lectin-like receptor subfamily B member 1B	0.9881	0.8254	0.8371	0.7575	No	1.0717	0.556	0.0702	0.506	No	No
94745_f_at	Eif1a /// MGC107533 /// LOC435337 /// LOC623167 /// LOC641136 /// LOC666806 /// LOC666862	eukaryotic translation initiation factor 1A /// similar to Eukaryotic translation initiation factor 1A (eIF-1A) (eIF-4C) /// similar to Eukaryotic translation initiation factor 1A (eIF-1A) (eIF-4C) /// similar to X-linked eukaryotic translation initiation factor 1A /// similar to X-linked eukaryotic translation initiation factor 1A /// similar to X-linked eukaryotic translation initiation factor 1A /// similar to X-linked eukaryotic translation initiation factor 1A	0.9767	0.7052	0.3139	0.2242	No	1.009	0.914	0.015	0.0441	No	No
94746_at	H2-T24	histocompatibility 2, T region locus 24	1.028	0.7492	0.0349	0.5636	No	1.0665	0.113	0.471	0.657	No	No
94747_at	Csf2rb1	colony stimulating factor 2 receptor, beta 1, low-affinity (granulocyte-macrophage)	0.931	0.2593	0.3302	0.7745	No	1.0085	0.743	0.792	0.191	No	No
94748_g_at	Csf2rb1 /// Csf2rb2	colony stimulating factor 2 receptor, beta 1, low-affinity (granulocyte-macrophage) /// colony stimulating factor 2 receptor, beta 2, low-affinity (granulocyte-macrophage)	0.9037	0.329	0.0543	0.57	No	0.8796	0.159	0.009	0.741	No	No
94749_f_at	AF067061	cDNA sequence AF067061	1.0202	0.7925	0.2423	0.7503	No	1.0453	0.612	0.146	0.331	No	No
94750_at	Dazl	deleted in azoospermia-like	1.0823	0.0806	0.7905	0.0579	No	1.1312	0.212	0.33	0.207	No	No
94752_s_at	Skil	SKI-like	0.9863	0.8163	0.2669	0.7545	No	1.0146	0.725	0.119	0.739	No	No
94753_at	Gna15	guanine nucleotide binding protein, alpha 15	1.0334	0.5017	0.0166	0.0754	No	1.0389	0.324	0.0085	0.0376	No	No
94754_at	Lhx8	LIM homeobox protein 8	1.0121	0.5633	0.003	0.0553	No	1.1675	0.0702	0.0072	0.0318	No	No
94755_at	Il1a	interleukin 1 alpha	1.0746	0.2029	0.5946	0.1066	No	1.1149	0.0703	0.965	0.0151	No	No
94756_at	Hist2h3c1 /// Hist2h3c2 /// Hist1h3d /// Hist1h3b /// Hist1h3e /// Hist2h3b	histone 2, H3c1 /// histone 2, H3c2 /// histone1, H3d /// histone 1, H3b /// histone 1, H3e /// histone 2, H3b	1.0207	0.8238	0.0892	0.5304	No	1.0592	0.359	0.0281	0.214	No	No
94757_at	Cftr	cystic fibrosis transmembrane conductance regulator homolog	1.1834	0.0126	0.0055	0.1586	No	1.0525	0.518	0.0226	0.126	No	No
94758_s_at	Cftr	cystic fibrosis transmembrane conductance regulator homolog	0.9858	0.617	0.7184	0.7006	No	0.9692	0.792	0.0476	0.677	No	No
94759_at	2810402A17 Rik	RIKEN cDNA 2810402A17 gene	0.9701	0.0687	0.0048	0.0286	No	0.9913	0.843	0.513	0.313	No	No
94760_at	---	---	1.0266	0.2961	0.0199	0.1363	No	1.0646	0.219	0.121	0.0626	No	No
94761_at	Ccl7	chemokine (C-C motif) ligand 7	1.0374	0.1762	0.9099	0.0235	No	1.2066	0.0257	0.783	0.319	No	No
94762_at	F2rl2	coagulation factor II (thrombin) receptor-like 2	0.9924	0.9076	0.7237	0.3111	No	1.1192	0.0042	0.0176	0.0013	No	No
94763_at	Musk	muscle, skeletal, receptor tyrosine kinase	0.9284	0.0725	0.025	0.0359	No	0.8938	0.182	0.0493	0.488	No	No
94764_at	Itgal	integrin alpha L	0.9851	0.6262	0.0817	0.8152	No	0.9424	0.109	0.25	0.608	No	No
94765_at	Rdh13	retinol dehydrogenase 13 (all-trans and 9-cis)	1.0014	0.9797	0.0084	0.9303	No	0.9389	0.384	0.0079	0.967	No	No
94766_at	Eef1a1	eukaryotic translation elongation factor 1 alpha 1	1.0278	0.0645	0.0097	0.2678	No	1.032	0.15	0.654	0.293	No	No
94767_at	Rps11 /// LOC383992 /// LOC621119 /// LOC623114 /// LOC639478 /// LOC669543 /// LOC676877	ribosomal protein S11 /// similar to ribosomal protein S11 /// similar to ribosomal protein S11 /// similar to ribosomal protein S11 /// similar to ribosomal protein S11 /// similar to ribosomal protein S11 /// similar to ribosomal protein S11	1.051	0.3207	0.9018	0.4546	No	1.003	0.938	0.948	0.0761	No	No
94768_at	Rad21	RAD21 homolog (S. pombe)	1.0782	0.1969	0.0031	0.1774	No	0.9926	0.964	0.0304	0.394	No	No
94769_at	Mmp8	matrix metallopeptidase 8	1.0331	0.5887	0.594	0.8779	No	0.9659	0.552	0.555	0.0713	No	No
94770_at	Otud5	OTU domain containing 5	1.0338	0.4589	0.084	0.0857	No	1.0189	0.332	0.412	0.328	No	No
94771_at	Rassf2	Ras association (RalGDS/AF-6) domain family 2	0.9171	0.2256	0.5693	0.6928	No	1.0031	0.996	0.06	0.0036	No	No
94772_at	Klrb1a	killer cell lectin-like receptor subfamily B member 1A	1.0824	0.0724	0.1301	0.0254	No	1.1332	0.0933	0.667	0.0598	No	No
94773_at	Klk1b4	kallikrein 1-related pepidase b4	0.9256	0.2538	0.3723	0.5621	No	1.0705	0.604	0.123	0.813	No	No
94774_at	Ifi202b	interferon activated gene 202B	0.947	0.4388	0.9622	0.5039	No	0.7824	0.0395	0.0735	0.168	No	No
94775_at	Oxt	oxytocin	0.9774	0.6717	0.0419	0.9432	No	0.9952	0.939	0.697	0.783	No	No
94776_f_at	Klra4	killer cell lectin-like receptor, subfamily A, member 4	1.0026	0.9614	0.6922	0.3786	No	1.1008	0.316	0.0758	0.154	No	No
94777_at	Alb1	albumin 1	1.1171	0.0168	0.0053	0.0027	No	1.039	0.53	0.305	0.411	No	No
94778_at	Aldh1a7	aldehyde dehydrogenase family 1, subfamily A7	1.0057	0.8221	0.3456	0.0932	No	1.0074	0.781	0.157	0.0125	No	No
94779_f_at	Klra3 /// Klra9	killer cell lectin-like receptor, subfamily A, member 3 /// killer cell lectin-like receptor subfamily A, member 9	1.0479	0.5046	0.3138	0.3268	No	0.9903	0.999	0.124	0.129	No	No
94780_at	Zbtb20	zinc finger and BTB domain containing 20	0.8198	0.0019	0	0.2325	Yes	0.9258	0.22	0.0003	0.754	No	No
94781_at	Hba-a1	hemoglobin alpha, adult chain 1	0.9988	0.9711	0.0018	0.8511	No	0.9923	0.461	0.0001	0.25	No	No
94782_at	C78142	expressed sequence C78142	1.0467	0.4495	0.4246	0.8177	No	1.0313	0.648	0.0341	0.447	No	No
94783_at	---	---	0.9475	0.2157	0.0236	0.0678	No	0.9902	0.855	0.7	0.603	No	No
94784_at	Iqgap3	IQ motif containing GTPase activating protein 3	1.0246	0.6118	0.0208	0.1347	No	1.0157	0.56	0.0055	0.0675	No	No
94785_at	D7Ertd128e	DNA segment, Chr 7, ERATO Doi 128, expressed	0.9547	0.4937	0.083	0.8529	No	0.969	0.703	0.393	0.527	No	No
94786_at	C81272	expressed sequence C81272	0.9481	0.2894	0.8318	0.58	No	1.0486	0.643	0.253	0.749	No	No
94787_at	Auh	AU RNA binding protein/enoyl-coenzyme A hydratase	0.9754	0.4623	0.8314	0.0577	No	1.0189	0.656	0.264	0.285	No	No
94788_f_at	Tubb5	tubulin, beta 5	1.139	0.0135	0.0185	0.3171	No	1.1208	0.0293	0.017	0.814	No	No
94789_r_at	Tubb5	tubulin, beta 5	1.0651	0.15	0.0043	0.267	No	1.039	0.128	0.0007	0.975	No	No
94790_at	Agl	amylo-1,6-glucosidase, 4-alpha-glucanotransferase	0.8953	0.1165	0.002	0.2562	No	0.8575	0.132	0.0051	0.436	No	No
94791_s_at	Ltk	leukocyte tyrosine kinase	0.962	0.298	0.8779	0.2527	No	0.94	0.336	0.437	0.102	No	No
94792_at	Msr1	macrophage scavenger receptor 1	1.0197	0.7092	0.136	0.3737	No	1.0536	0.262	0.126	0.024	No	No

94793_at	Sephs1	selenophosphate synthetase 1	1.015	0.7444	0.094	0.6898	No	0.9544	0.636	0.0085	0.744	No	No
94794_at	Fth1	ferritin heavy chain 1	0.9096	0.0839	0.9785	0.2575	No	0.9762	0.541	0.0454	0.44	No	No
94795_at	Hsd3b5	hydroxysteroid dehydrogenase-5, delta<5>-3-beta	1.1818	0.069	0.0098	0.0471	No	1.1393	0.0664	0.0134	0.0121	No	No
94796_at	---	---	0.9826	0.6769	0.0096	0.9259	No	0.9654	0.449	0.027	0.508	No	No
94797_at	Slc26a1	solute carrier family 26 (sulfate transporter), member 1	0.972	0.5691	0.147	0.0399	No	0.9385	0.505	0.0783	0.143	No	No
94798_at	Abcb1b	ATP-binding cassette, sub-family B (MDR/TAP), member 1B	0.8903	0.0016	0.0472	0.6848	Yes	0.9585	0.948	0.0076	0.156	No	No
94799_at	F8	coagulation factor VIII	0.8827	0.0674	0.168	0.1166	No	0.8982	0.196	0.0343	0.178	No	No
94800_at	Mllt10	Myeloid/lymphoid or mixed lineage-leukemia translocation to 10 homolog (Drosophila)	1.0343	0.2557	0.0055	0.0637	No	0.9512	0.472	0.0157	0.61	No	No
94801_at	Pgrmc2	progesterone receptor membrane component 2	1.0256	0.5691	0.0355	0.6557	No	0.9772	0.643	0.177	0.275	No	No
94802_at	BC060632	cDNA sequence BC060632	1.0374	0.5153	0.0198	0.8127	No	1.0484	0.201	0.0412	0.101	No	No
94803_at	Pbx1	pre B-cell leukemia transcription factor 1	1.0476	0.0889	0.3903	0.1074	No	1.0307	0.332	0.132	0.729	No	No
94804_at	Pbx1 /// LOC676870	pre B-cell leukemia transcription factor 1 /// region containing RIKEN cDNA 2310056B04 gene; pre B-cell leukemia transcription factor 1	1.007	0.861	0.025	0.194	No	0.9402	0.0354	0.0001	0.137	No	No
94805_f_at	Hist3h2a /// Hist1h2ac /// Hist1h2ad /// Hist1h2ae /// Hist1h2ag /// Hist1h2ah /// Hist1h2ak /// Hist1h2an /// Hist1h2ao /// Hist1h2ab /// Hist1h2ai /// MGC73635	histone 3, H2a /// histone 1, H2ac /// histone 1, H2ad /// histone 1, H2ae /// histone 1, H2ag /// histone 1, H2ah /// histone 1, H2ak /// histone 1, H2an /// histone 1, H2ao /// histone 1, H2ab /// histone 1, H2ai /// similar to histone 2a	1.1681	0.0187	0.0143	0.0725	No	1.093	0.0081	0.0014	0.13	Yes	Yes
94806_at	Pdhb	pyruvate dehydrogenase (lipoamide) beta	1.0879	0.2093	0.194	0.2157	No	0.9851	0.899	0.0008	0.0324	No	No
94807_at	Slc25a1	solute carrier family 25 (mitochondrial carrier, citrate transporter), member 1	1.1704	0.0027	0.4287	0.9597	Yes	1.1401	0.0104	0.216	0.0313	No	Yes
94809_at	Tsg101	tumor susceptibility gene 101	1.021	0.7312	0.0248	0.3636	No	0.9687	0.578	0.0033	0.309	No	No
94810_at	Ewsr1	Ewing sarcoma breakpoint region 1	1.0087	0.4298	0.3336	0.779	No	0.956	0.326	0.144	0.44	No	No
94811_s_at	Gtf2h1 /// Ndn	general transcription factor II H, polypeptide 1 /// necdin	1.0415	0.4046	0.0184	0.8874	No	0.9097	0.223	0.793	0.893	No	No
94812_at	Gtf2h1	general transcription factor II H, polypeptide 1	0.9673	0.3876	0.0514	0.3475	No	0.9563	0.316	0.0094	0.357	No	No
94813_at	Gas1	growth arrest specific 1	0.9982	0.9777	0.0227	0.2398	No	0.9504	0.321	0.0252	0.145	No	No
94814_at	Gnai3	guanine nucleotide binding protein, alpha inhibiting 3	0.9646	0.2449	0.0043	0.32	No	0.9598	0.343	0.29	0.358	No	No
94815_at	Bpgm	2,3-bisphosphoglycerate mutase	0.9772	0.6914	0.002	0.0446	No	1.0446	0.411	0.0002	0.0056	No	No
94817_at	Serpinh1	serine (or cysteine) peptidase inhibitor, clade H, member 1	1.0787	0.1724	0.0228	0.1357	No	1.0405	0.206	0.0046	0.32	No	No
94818_at	Ogt	O-linked N-acetylglucosamine (GlcNAc) transferase (UDP-N-acetylglucosamine:polypeptide-N-acetylglucosaminyl transferase)	0.9233	0.4708	0.0033	0.2874	No	0.914	0.752	0.0015	0.122	No	No
94819_f_at	Ccni	cyclin I	0.9906	0.791	0.0026	0.2392	No	0.9529	0.328	0.0204	0.651	No	No
94820_r_at	Ccni	cyclin I	1.0746	0.1613	0.0011	0.037	No	1.0344	0.449	0.0243	0.256	No	No
94821_at	Xbp1	X-box binding protein 1	1.0794	0.3733	0.7405	0.463	No	1.1604	0.106	0.676	0.526	No	No
94822_at	Setd3 /// LOC622830 /// LOC633498 /// LOC671440	SET domain containing 3 /// hypothetical protein LOC622830 /// similar to CG32732-PA /// similar to CG32732-PA	1.0473	0.5537	0.0258	0.8113	No	1.0831	0.191	0.0475	0.0846	No	No
94823_at	Rpl23a	ribosomal protein L23a	1.0422	0.4611	0.1441	0.2613	No	1.0182	0.672	0.127	0.684	No	No
94825_at	Plaa	phospholipase A2, activating protein	1.0237	0.8045	0.7	0.6658	No	1.0779	0.532	0.462	0.98	No	No
94826_at	Itgb4bp	integrin beta 4 binding protein	1.06	0.2526	0.4722	0.3084	No	1.0376	0.0999	0.358	0.0904	No	No
94827_at	Fxyd2	FXYD domain-containing ion transport regulator 2	0.9856	0.7943	0.2512	0.1354	No	0.9805	0.667	0.17	0.0356	No	No
94828_at	Oprs1	opioid receptor, sigma 1	1.0102	0.718	0.3644	0.2694	No	1.0127	0.829	0.545	0.656	No	No
94829_at	Tmem70	transmembrane protein 70	0.9348	0.4218	0.3442	0.5633	No	0.9469	0.185	0.0079	0.091	No	No
94830_at	BC005537	cDNA sequence BC005537	1.0011	0.9915	0.0123	0.522	No	0.8842	0.0818	0.0039	0.904	No	No
94831_at	Ctsb	cathepsin B	0.9494	0.2058	0.3796	0.3746	No	0.9599	0.184	0.78	0.821	No	No
94832_at	Hnrph2	heterogeneous nuclear ribonucleoprotein H2	1.107	0.3418	0.0073	0.1301	No	0.9632	0.623	0.0098	0.699	No	No
94833_at	Fstl1	follistatin-like 1	1.0399	0.392	0.191	0.0494	No	1.0478	0.193	0.0902	0.0048	No	No
94834_at	Ctsh	cathepsin H	0.9076	0.1228	0.4999	0.8976	No	0.9344	0.0883	0.134	0.11	No	No
94835_f_at	Tubb2a	tubulin, beta 2a	1.0565	0.4214	0.1865	0.0735	No	1.0628	0.19	0.0064	0.151	No	No
94836_at	C79445	expressed sequence C79445	0.9974	0.9044	0.0893	0.1383	No	1.0672	0.0644	0.0056	0.007	No	No
94837_at	Eif3s8	eukaryotic translation initiation factor 3, subunit 8	1.012	0.8028	0.3567	0.4991	No	0.9732	0.306	0.0006	0.65	No	No
94838_r_at	Plf /// Plf2 /// LOC630663 /// LOC666317 /// LOC672240 /// LOC676034	proliferin /// proliferin 2 /// similar to Proliferin 3 precursor (Mitogen-regulated protein 3) /// similar to mitogen regulated protein, proliferin 4 /// similar to mitogen regulated protein, proliferin 4 /// similar to mitogen regulated protein, proliferin 4	1.0136	0.8795	0.0856	0.1675	No	1.0071	0.299	0.107	0.0834	No	No
94839_at	Nucb1	nucleobindin 1	0.975	0.1179	0	0.0102	No	0.9991	0.787	0.0017	0.0606	No	No
94840_at	Hexa	hexosaminidase A	0.9357	0.3511	0.0119	0.8912	No	0.8908	0.362	0.0206	0.309	No	No
94841_at	Psma5	proteasome (prosome, macropain) subunit, alpha type 5	1.0353	0.3597	0.6863	0.1171	No	0.9996	0.897	0.0036	0.31	No	No
94842_at	Blmh	bleomycin hydrolase	1.0107	0.6971	0.5312	0.4722	No	1.0198	0.613	0.913	0.86	No	No
94843_at	Pold4	polymerase (DNA-directed), delta 4	0.8938	0.0104	0.0268	0.0122	No	0.9885	0.615	0.148	0.267	No	No
94844_at	Rpl39	ribosomal protein L39	1.007	0.9084	0.1601	0.5935	No	0.9053	0.379	0.959	0.844	No	No
94845_at	Zc3h11a	zinc finger CCCH type containing 11A	1.0043	0.9173	0.0361	0.0496	No	1.0014	0.901	0.0006	0.523	No	No
94848_at	Mif /// LOC434371 /// LOC619750	macrophage migration inhibitory factor /// similar to Macrophage migration inhibitory factor (MIF) (Phenylpyruvate tautomerase) (Glycosylation-inhibiting factor) (GIF) (Delayed early response protein 6) (DER6) /// similar to Macrophage migration inhibitory factor (MIF) (Phenylpyruvate tautomerase) (Glycosylation-inhibiting factor) (GIF) (Delayed early response protein 6) (DER6)	1.2242	0.0887	0.0603	0.1628	No	1.1986	0.274	0.674	0.0787	No	No
94849_at	Mif	Macrophage migration inhibitory factor	1.0851	0.0572	0.9729	0.5717	No	0.9871	0.763	0.181	0.0373	No	No
94850_at	Acot9	acyl-CoA thioesterase 9	0.9813	0.6767	0.0392	0.365	No	0.9246	0.149	0.0506	0.847	No	No
94852_at	Glul	glutamate-ammonia ligase (glutamine synthetase)	0.5974	0.0006	0.0017	0.1737	Yes	0.6959	0.0008	0.59	0.67	Yes	Yes
94853_at	Gnb1	guanine nucleotide binding protein, beta 1	1.1458	0.0761	0.02	0.5296	No	1.0746	0.463	0.262	0.845	No	No
94854_g_at	Gnb1	guanine nucleotide binding protein, beta 1	1.0121	0.7948	0.009	0.5051	No	1.0473	0.27	0.0039	0.0919	No	No
94855_at	Phb	prohibitin	1.0285	0.5293	0.309	0.8974	No	1.0123	0.553	0.0316	0.171	No	No
94856_r_at	Wasf2	WAS protein family, member 2	0.9917	0.8699	0.0177	0.2552	No	1.0788	0.482	0.0007	0.0825	No	No
94857_at	Mpg	N-methylpurine-DNA glycosylase	0.9254	0.2592	0.1962	0.4338	No	0.9259	0.0704	0.0267	0.968	No	No
94860_at	Timm17a	translocase of inner mitochondrial membrane 17a	1.0671	0.3199	0.0591	0.3657	No	1.0946	0.246	0.0014	0.677	No	No
94861_at	4930453N24Rik	RIKEN cDNA 4930453N24 gene	1.0357	0.374	0.0269	0.4051	No	1.064	0.224	0.0181	0.87	No	No
94862_i_at	Dynlrb1	dynein light chain roadblock-type 1	0.988	0.7095	0.0027	0.0058	No	1.026	0.353	0.0005	0.107	No	No
94863_r_at	Dynlrb1	dynein light chain roadblock-type 1	1.1715	0.0144	0.012	0.6207	No	0.9721	0.764	0.0012	0.193	No	No
94865_at	Ublcp1	ubiquitin-like domain containing CTD phosphatase 1	1.0113	0.7747	0.0184	0.837	No	0.9713	0.0206	0	0.0013	No	No
94866_at	Mrps16	mitochondrial ribosomal protein S16	1.0456	0.1527	0.0002	0.6789	No	1.003	0.595	0.0009	0.0572	No	No
94868_at	Qars	glutaminyl-tRNA synthetase	0.9001	0.001	0.0036	0.0026	No	0.8985	0.0073	0.0082	0.003	No	No
94869_at	Aebp2	AE binding protein 2	0.9634	0.5696	0.1933	0.4967	No	1.0013	0.894	0.143	0.607	No	No
94870_f_at	Sar1b	SAR1 gene homolog B (S. cerevisiae)	1.0189	0.7498	0.0348	0.2347	No	1.0435	0.48	0.322	0.125	No	No
94871_r_at	Sar1b	SAR1 gene homolog B (S. cerevisiae)	1.1592	0.3215	0.1277	0.8405	No	1.0238	0.816	0.477	0.694	No	No
94872_at	Smpdl3a	sphingomyelin phosphodiesterase, acid-like 3A	0.9075	0.2176	0.0924	0.7445	No	0.9429	0.314	0.158	0.67	No	No

94874_at	Aplp1	amyloid beta (A4) precursor-like protein 1	1.0163	0.6946	0.0977	0.9506	No	1.0584	0.501	0.348	0.61	No	No
94875_at	Mrpl20	mitochondrial ribosomal protein L20	1.0614	0.2706	0.0279	0.4182	No	1.1174	0.0552	0.0088	0.251	No	No
94876_f_at	Gorasp2	golgi reassembly stacking protein 2	1.0963	0.0577	0.0095	0.2155	No	1.0224	0.52	0.401	0.802	No	No
94877_at	Btbd1 /// LOC545472 /// LOC672472 /// LOC677542	BTB (POZ) domain containing 1 /// similar to BTB (POZ) domain containing 1 (predicted) /// similar to BTB (POZ) domain containing 1 (predicted) /// similar to BTB (POZ) domain containing 1 (predicted)	1.0267	0.4866	0.033	0.0463	No	1.0255	0.798	0.026	0.172	No	No
94878_at	Btbd1	BTB (POZ) domain containing 1	1.06	0.267	0.0011	0.5048	No	1.0787	0.0447	0.0001	0.548	No	No
94881_at	Cdkn1a	cyclin-dependent kinase inhibitor 1A (P21)	0.8727	0.0654	0.0097	0.3802	No	0.8555	0.0187	0.0224	0.58	No	No
94882_at	Tmem30a	transmembrane protein 30A	1.0306	0.7222	0.1744	0.2919	No	1.0342	0.714	0.757	0.52	No	No
94885_at	Unc84a	unc-84 homolog A (C. elegans)	0.9358	0.0124	0.0862	0.0308	No	0.9212	0.0909	0.0912	0.278	No	No
94886_at	Canx	calnexin	1.1379	0.0231	0.021	0.0588	No	1.0314	0.31	0.0069	0.0305	No	No
94889_at	Vapa	vesicle-associated membrane protein, associated protein A	1.0409	0.4339	0.072	0.2335	No	0.9816	0.739	0.526	0.657	No	No
94890_at	Mea1	male enhanced antigen 1	0.9185	0.3277	0.0103	0.2578	No	1.0169	0.89	0.0098	0.0777	No	No
94891_s_at	Mea1	male enhanced antigen 1	0.9795	0.6193	0.3786	0.2701	No	0.9326	0.102	0.384	0.66	No	No
94892_r_at	Mea1	male enhanced antigen 1	0.9631	0.4369	0.2473	0.2499	No	1.0393	0.442	0.363	0.463	No	No
94893_at	2610301B20Rik	RIKEN cDNA 2610301B20 gene	1.0475	0.4003	0.8233	0.2535	No	1.0168	0.703	0.403	0.716	No	No
94895_at	Ythdf2	YTH domain family 2	1.1287	0.2108	0.0152	0.2958	No	1.0266	0.859	0.014	0.621	No	No
94896_at	Hnrpab	heterogeneous nuclear ribonucleoprotein A/B	1.0492	0.0455	0.4568	0.0615	No	1.0008	0.911	0.0539	0.09	No	No
94897_at	Gpx4	glutathione peroxidase 4	0.9654	0.2178	0.0052	0.0057	No	0.9604	0.576	0.0148	0.0276	No	No
94898_at	Rbm18	RNA binding motif protein 18	0.935	0.4865	0.0402	0.5919	No	0.9192	0.29	0.0775	0.193	No	No
94899_at	AA536749	expressed sequence AA536749	1.033	0.5629	0.0101	0.9884	No	1.0483	0.0706	0.0003	0.283	No	No
94900_at	Sftpc	surfactant associated protein C	0.9731	0.6067	0.0279	0.3905	No	0.9574	0.555	0.102	0.687	No	No
94902_at	Sod3	superoxide dismutase 3, extracellular	1.1055	0.0124	0.7776	0.8465	No	1.0723	0.0302	0.427	0.0564	No	No
94903_at	Tmed7	transmembrane emp24 protein transport domain containing 7	0.9863	0.8305	0.5819	0.9015	No	0.9625	0.607	0.623	0.745	No	No
94906_at	Adh1	alcohol dehydrogenase 1 (class I)	0.8334	0.012	0.5015	0.061	No	0.879	0.0352	0.3	0.0278	No	No
94907_f_at	1110001J03Rik	RIKEN cDNA 1110001J03 gene	0.9865	0.7328	0.1514	0.0178	No	1.0184	0.415	0.0187	0.0166	No	No
94908_r_at	1110001J03Rik	RIKEN cDNA 1110001J03 gene	1.0075	0.7493	0.0003	0.0123	No	1.024	0.326	0.704	0.592	No	No
94909_at	Mrps17	mitochondrial ribosomal protein S17	0.9936	0.8707	0.176	0.0249	No	1.0393	0.315	0.0586	0.039	No	No
94910_at	Nde1	nuclear distribution gene E homolog 1 (A nidulans)	0.9795	0.6353	0.5423	0.1549	No	0.9839	0.756	0.953	0.238	No	No
94912_at	Mrps21	mitochondrial ribosomal protein S21	1.0217	0.8107	0.9203	0.9235	No	0.9866	0.895	0.212	0.128	No	No
94913_at	E430034L04Rik	RIKEN cDNA E430034L04 gene	1.0887	0.0696	0.0224	0.0534	No	1.0602	0.384	0.674	0.804	No	No
94914_at	2210415F13Rik	RIKEN cDNA 2210415F13 gene	1.0576	0.1922	0.1706	0.0234	No	1.049	0.243	0.0086	0.064	No	No
94915_at	Ppib	peptidylprolyl isomerase B	1.0261	0.5834	0.0018	0.9823	No	1.015	0.768	0.0096	0.582	No	No
94916_at	Cyp51	cytochrome P450, family 51	1.1361	0.0058	0.0069	0.1157	Yes	1.194	0.0196	0.0141	0.525	No	Yes
94917_at	Fbxo8	F-box only protein 8	0.971	0.5147	0.2252	0.7237	No	0.9614	0.213	0.0085	0.123	No	No
94918_at	Aars	alanyl-tRNA synthetase	0.9809	0.4273	0.3883	0.0775	No	0.9736	0.366	0.301	0.222	No	No
94920_at	Pnpo	pyridoxine 5'-phosphate oxidase	0.9766	0.4139	0.0283	0.1278	No	0.957	0.2	0.769	0.0319	No	No
94921_i_at	Pnpo	Pyridoxine 5'-phosphate oxidase	1.0153	0.0551	0.364	0.0111	No	0.4991	0.266	0.151	0.336	No	No
94922_i_at	Otud4	OTU domain containing 4	0.9065	0.2132	0.0393	0.304	No	1.07	0.61	0.003	0.663	No	No
94923_f_at	Otud4	OTU domain containing 4	1.0361	0.6562	0.0021	0.2647	No	0.9476	0.268	0.0002	0.408	No	No
94924_at	Prrg2	proline-rich Gla (G-carboxyglutamic acid) polypeptide 2	0.9519	0.2066	0.0019	0.3568	No	1.041	0.446	0.0751	0.955	No	No
94925_at	Dnajc19	DnaJ (Hsp40) homolog, subfamily C, member 19	1.0379	0.6207	0.9701	0.5461	No	1.0088	0.774	0.049	0.268	No	No
94927_at	Fabp9	fatty acid binding protein 9, testis	0.9845	0.9015	0.0695	0.5536	No	1.0012	0.312	0	0.05	No	No
94928_at	Tnfrsf1b	tumor necrosis factor receptor superfamily, member 1b	1.0614	0.0508	0.8679	0.0286	No	1.001	0.987	0.566	0.19	No	No
94929_at	Ptpn1	protein tyrosine phosphatase, non-receptor type 1	1.0874	0.0563	0.2638	0.302	No	1.0763	0.085	0.0445	0.0357	No	No
94930_at	Thbs2	thrombospondin 2	1.0441	0.5306	0.0085	0.0308	No	0.9765	0.796	0.0079	0.0276	No	No
94931_at	Ufm1	ubiquitin-fold modifier 1	1.0655	0.2426	0.6745	0.0523	No	1.0177	0.681	0.123	0.0518	No	No
94932_at	Pdgfa	platelet derived growth factor, alpha	1.0214	0.3449	0.0603	0.1577	No	1.015	0.667	0.0764	0.662	No	No
94933_at	BC008155	cDNA sequence BC008155	0.9932	0.8159	0.0494	0.5991	No	1.0039	0.767	0.0135	0.0787	No	No
94934_at	Agtr2	angiotensin II receptor, type 2	0.9264	0.3712	0.0403	0.6887	No	0.9057	0.243	0.0541	0.79	No	No
94935_at	Tbl1x	Transducin (beta)-like 1 X-linked	1.0531	0.2787	0.0043	0.919	No	1.0711	0.219	0.0016	0.486	No	No
94936_at	Mep1b	meprin 1 beta	0.9837	0.7622	0.0107	0.9252	No	0.9638	0.337	0.0018	0.0803	No	No
94937_at	Zfp277	zinc finger protein 277	0.9981	0.9504	0.8114	0.9328	No	1.0039	0.928	0.242	0.902	No	No
94939_at	Cd53	CD53 antigen	0.9307	0.1619	0.0047	0.5369	No	0.8824	0.0894	0.0068	0.252	No	No
94940_at	Mccc1	methylcrotonoyl-Coenzyme A carboxylase 1 (alpha)	0.921	0.1162	0.0471	0.0965	No	0.959	0.348	0.0179	0.332	No	No
94941_at	Eif2ak4	eukaryotic translation initiation factor 2 alpha kinase 4	0.9536	0.2494	0.0883	0.438	No	0.946	0.462	0.694	0.877	No	No
94942_at	Cstf1	cleavage stimulation factor, 3' pre-RNA, subunit 1	0.9914	0.7386	0.1267	0.1035	No	0.9793	0.426	0.801	0.0117	No	No
94944_at	Prkar1b	protein kinase, cAMP dependent regulatory, type I beta	0.9576	0.3155	0.4002	0.7736	No	0.9892	0.358	0.0042	0.0118	No	No
94945_at	Commd5	COMM domain containing 5	0.9502	0.3117	0.0456	0.4039	No	0.9643	0.436	0.468	0.194	No	No
94946_at	Map3k3	mitogen activated protein kinase kinase kinase 3	0.9301	0.1786	0.7054	0.7603	No	0.9745	0.441	0.0298	0.257	No	No
94947_g_at	Map3k3	mitogen activated protein kinase kinase kinase 3	0.9431	0.143	0.0089	0.0938	No	0.9175	0.0877	0.0491	0.0475	No	No
94948_at	Trip6	thyroid hormone receptor interactor 6	0.9888	0.7327	0.003	0.2001	No	0.9734	0.503	0.0076	0.187	No	No
94951_at	Las1l	LAS1-like (S. cerevisiae)	0.957	0.3229	0.1159	0.2392	No	0.9856	0.801	0.775	0.789	No	No
94952_at	C330012H03Rik	RIKEN cDNA C330012H03 gene	1.0221	0.5862	0.0035	0.0243	No	1.0021	0.863	0.0011	0.353	No	No
94953_at	Racgap1	Rac GTPase-activating protein 1	1.0017	0.9735	0.0758	0.8983	No	1.0833	0.12	0.0124	0.315	No	No
94954_at	Anapc4	anaphase promoting complex subunit 4	0.9598	0.3109	0.0671	0.49	No	0.9658	0.554	0.0093	0.371	No	No
94955_at	Ankrd40	ankyrin repeat domain 40	1.0129	0.8419	0.4852	0.1857	No	0.8556	0.0937	0.0446	0.0279	No	No
94956_at	Dgcr6	DiGeorge syndrome critical region gene 6	1.0022	0.9557	0.0999	0.2608	No	1.0406	0.482	0.0255	0.708	No	No
94957_at	Prepl	prolyl endopeptidase-like	1.0577	0.5357	0.0106	0.2126	No	1.0121	0.547	0.0017	0.0618	No	No
94958_at	1110013L07Rik	RIKEN cDNA 1110013L07 gene	1.0145	0.6597	0.0107	0.5313	No	1.126	0.0661	0.0031	0.225	No	No
94961_at	Clpx	Caseinolytic peptidase X (E.coli)	0.9965	0.9429	0.295	0.8813	No	1.0079	0.826	0.0856	0.193	No	No
94962_g_at	Clpx	Caseinolytic peptidase X (E.coli)	1.0602	0.2482	0.645	0.4345	No	1.117	0.0682	0.607	0.0388	No	No
94963_at	Vcl	vinculin	0.911	0.1381	0.0037	0.2746	No	0.9097	0.103	0.0198	0.41	No	No
94964_at	Vcl	vinculin	0.9839	0.6563	0.1326	0.0787	No	0.9063	0.174	0.244	0.0901	No	No
94966_at	G6pdx	glucose-6-phosphate dehydrogenase X-linked	1.0051	0.9123	0.1168	0.2778	No	0.9594	0.0276	0.0464	0.0104	No	No
94967_at	D19Wsu12e	DNA segment, Chr 19, Wayne State University 12, expressed	1.0486	0.2421	0.0175	0.3138	No	1.1117	0.0737	0.258	0.431	No	No
94968_at	Nfyc	nuclear transcription factor-Y gamma	0.997	0.9442	0.0118	0.3533	No	0.9295	0.0116	0.0004	0.503	No	No
94969_at	Nfyc	nuclear transcription factor-Y gamma	0.9956	0.8334	0.0027	0.052	No	1.0018	0.975	0.0141	0.0788	No	No
94970_at	Ppp4r2	protein phosphatase 4, regulatory subunit 2	0.9957	0.9482	0.0306	0.984	No	1.0744	0.149	0.0018	0.923	No	No
94971_at	Cdkn3	cyclin-dependent kinase inhibitor 3	0.8734	0.0071	0.0125	0.1385	Yes	0.914	0.0022	0.114	0.221	Yes	Yes
94972_at	Rbms1	RNA binding motif, single stranded interacting protein 1	1.0027	0.9347	0.0124	0.4682	No	0.9975	0.967	0.107	0.759	No	No
94973_at	Jmjd1b	jumonji domain containing 1B	0.9345	0.0657	0.0006	0.1529	No	1.0262	0.887	0.0002	0.0132	No	No
94975_at	Ireb2	iron responsive element binding protein 2	0.9231	0.5615	0.0392	0.7228	No	0.9537	0.549	0.0634	0.617	No	No
94976_at	Numa1	nuclear mitotic apparatus protein 1	0.9253	0.033	0.0102	0.1074	No	0.9524	0.3	0.166	0.896	No	No
94977_at	Itpr1	inositol 1,4,5-triphosphate receptor 1	0.9032	0.0279	0.0007	0.9197	No	0.8477	0.0266	0.0117	0.255	No	No
94978_at	2310037I24Rik	RIKEN cDNA 2310037I24 gene	1.0858	0.0209	0.0217	0.9438	No	1.0155	0.575	0.112	0.186	No	No
94979_at	BC018507	cDNA sequence BC018507	1.0013	0.9811	0.0013	0.0619	No	0.9531	0.319	0.0043	0.225	No	No
94980_at	Dusp11	dual specificity phosphatase 11 (RNA/RNP complex 1-interacting)	0.9357	0.0309	0.0004	0.0487	No	0.9698	0.326	0.008	0.869	No	No
94981_i_at	Nme3	expressed in non-metastatic cells 3	0.8831	0.1366	0.3077	0.0271	No	1.0036	0.291	0	0.0081	No	No
94982_f_at	Nme3	expressed in non-metastatic cells 3	0.9767	0.6374	0.0685	0.0318	No	0.9679	0.551	0.0333	0.009	No	No
94983_at	1810073G14Rik	RIKEN cDNA 1810073G14 gene	0.8952	0.0667	0.0062	0.4809	No	0.922	0.0372	0.0269	0.0824	No	No
94985_at	Syncrip	synaptotagmin binding, cytoplasmic RNA interacting protein	0.9977	0.9736	0.0098	0.4198	No	1.0387	0.368	0.0618	0.36	No	No
94986_at	Gng3	guanine nucleotide binding protein (G protein), gamma 3 subunit	1.0016	0.9598	0.0004	0.8545	No	0.9993	0.977	0.0403	0.769	No	No
94987_at	Pemt	phosphatidylethanolamine N-methyltransferase	0.9695	0.5897	0.0065	0.3739	No	0.9934	0.852	0.0038	0.694	No	No
94988_at	Pten	phosphatase and tensin homolog	0.9654	0.2209	0.9313	0.1067	No	0.9586	0.418	0.0805	0.783	No	No
94989_at	Pten	phosphatase and tensin homolog	0.9992	0.9925	0.0933	0.5857	No	0.9266	0.0269	0.0545	0.831	No	No
94990_at	Tmem115	transmembrane protein 115	0.9568	0.2287	0.0141	0.1548	No	1.0048	0.895	0.012	0.638	No	No
94991_at	Synpo	synaptopodin	0.9509	0.3385	0.0075	0.0698	No	0.878	0.0151	0.0002	0.0041	No	No

94992_at	Copb1	coatomer protein complex, subunit beta 1	0.9985	0.9641	0.0209	0.5879	No	1.0017	0.942	0.712	0.772	No	No
94993_f_at	BC026782	cDNA sequence BC026782	0.9306	0.0866	0.0007	0.893	No	0.854	0.0287	0.0076	0.261	No	No
94994_at	BC026782	cDNA sequence BC026782	1.0847	0.361	0.3671	0.3231	No	1.1672	0.0807	0.221	0.166	No	No
94995_at	A030007L17Rik	RIKEN cDNA A030007L17 gene	1.0712	0.0723	0.0919	0.4957	No	0.9694	0.739	0.072	0.241	No	No
94997_at	Emcn	endomucin	1.0456	0.2441	0.0005	0.127	No	1.0784	0.0772	0.0007	0.674	No	No
94998_at	Rala	v-ral simian leukemia viral oncogene homolog A (ras related)	1.0993	0.1523	0.0247	0.61	No	0.9991	0.812	0.0113	0.0917	No	No
94999_at	Cubn	cubilin (intrinsic factor-cobalamin receptor)	1.0864	0.2649	0.6944	0.0837	No	1.0507	0.311	0.108	0.0921	No	No
95000_g_at	Cubn	cubilin (intrinsic factor-cobalamin receptor)	1.0572	0.2974	0.0189	0.0885	No	1.0162	0.597	0.0037	0.78	No	No
95001_at	Akap8	A kinase (PRKA) anchor protein 8	0.9531	0.3354	0.0076	0.1908	No	0.9903	0.791	0.139	0.628	No	No
95002_at	D17Wsu92e	DNA segment, Chr 17, Wayne State University 92, expressed	1.0073	0.9184	0.3852	0.661	No	0.9945	0.564	0.0002	0.0699	No	No
95003_at	Polr2k	polymerase (RNA) II (DNA directed) polypeptide K	1.2824	0.103	0.2021	0.7923	No	1.1006	0.339	0.0903	0.551	No	No
95004_at	Luc7l	Luc7 homolog (S. cerevisiae)-like	1.0126	0.7313	0.4573	0.6669	No	1.0992	0.155	0.0178	0.96	No	No
95007_at	Tpr	translocated promoter region	0.9639	0.3754	0.0062	0.7537	No	0.9254	0.0216	0.0113	0.0432	No	No
95009_at	Plod3	procollagen-lysine, 2-oxoglutarate 5-dioxygenase 3	1.0626	0.4217	0.3826	0.7845	No	1.0083	0.881	0.17	0.839	No	No
95010_at	Traf3	Tnf receptor-associated factor 3	1.0448	0.0643	0.6089	0.0183	No	1.0197	0.575	0.443	0.0735	No	No
95011_at	1810063B07Rik	RIKEN cDNA 1810063B07 gene	0.9375	0.3352	0.0686	0.1033	No	1.0297	0.688	0.178	0.452	No	No
95012_at	Slc22a17	solute carrier family 22 (organic cation transporter), member 17	1.0574	0.0155	0.2332	0.195	No	1.0582	0.0337	0.001	0.258	No	No
95014_at	Fbxo6b	F-box only protein 6b	0.9209	0.0075	0.0008	0.0087	No	0.9818	0.314	0.003	0.206	No	No
95015_at	Akr1c12	aldo-keto reductase family 1, member C12	0.9282	0.1322	0.4693	0.2264	No	0.9906	0.733	0.0791	0.0703	No	No
95016_at	Nrp1	neuropilin 1	0.9919	0.876	0.0023	0.7916	No	1.0534	0.177	0.0024	0.0391	No	No
95018_r_at	AU021838	expressed sequence AU021838	0.9693	0.5951	0.0035	0.9402	No	0.9947	0.693	0.0021	0.167	No	No
95019_at	Gstt1	glutathione S-transferase, theta 1	0.9816	0.5938	0.9133	0.0049	No	0.9975	0.872	0.0202	0.0056	No	No
95020_at	Cd47	CD47 antigen (Rh-related antigen, integrin-associated signal transducer)	0.9267	0.2077	0.1354	0.0321	No	0.9423	0.188	0.0173	0.0111	No	No
95021_at	9430010O03Rik	RIKEN cDNA 9430010O03 gene	0.865	0.1301	0.3705	0.711	No	0.8694	0.15	0.627	0.794	No	No
95022_at	Akap12	A kinase (PRKA) anchor protein (gravin) 12	1.0244	0.5268	0.0002	0.0045	No	1.0833	0.271	0.0312	0.261	No	No
95023_at	Sidt2	SID1 transmembrane family, member 2	0.9013	0.0108	0.0031	0.0058	No	0.9534	0.201	0.394	0.0463	No	No
95024_at	Usp18	ubiquitin specific peptidase 18	1.016	0.8341	0.4718	0.8981	No	1.0667	0.381	0.762	0.0259	No	No
95025_at	D16H22S680E	DNA segment, Chr 16, human D22S680E, expressed	0.9628	0.3769	0.06	0.8651	No	1.0643	0.484	0.185	0.511	No	No
95026_at	Retsat	retinol saturase (all trans retinol 13,14 reductase)	1.0166	0.7131	0.115	0.4356	No	1.0418	0.571	0.814	0.201	No	No
95027_at	Usp21	ubiquitin specific peptidase 21	0.9333	0.3151	0.0259	0.5381	No	0.9616	0.317	0.673	0.321	No	No
95028_r_at	3100002L24Rik /// 9230108I15Rik /// LOC329575 /// LOC626832 /// LOC627914 /// LOC628147 /// LOC665043 /// LOC665078 /// LOC665211 /// LOC668030 /// LOC668039 /// LOC668066	RIKEN cDNA 3100002L24 gene /// RIKEN cDNA 9230108I15 gene /// hypothetical LOC329575 /// similar to gonadotropin inducible ovarian transcription factor 1 /// similar to zinc finger protein 709 /// similar to zinc finger protein 709 /// similar to zinc finger protein 709 /// similar to zinc finger protein 709 /// similar to zinc finger protein 709 /// similar to gonadotropin inducible ovarian transcription factor 1 /// similar to zinc finger protein 97 /// similar to gonadotropin inducible ovarian transcription factor 1	0.9196	0.5056	0.2376	0.5321	No	0.8834	0.202	0.827	0.276	No	No
95029_at	Atp13a1	ATPase type 13A1	1.0016	0.9329	0.1246	0.0559	No	1.0334	0.501	0.583	0.919	No	No
95030_at	Prlr	prolactin receptor	0.991	0.8727	0.0473	0.3957	No	1.0084	0.935	0.715	0.814	No	No
95031_at	1110059H15Rik	RIKEN cDNA 1110059H15 gene	0.9054	0.3412	0.0104	0.251	No	0.951	0.767	0.004	0.632	No	No
95032_at	Prc1	protein regulator of cytokinesis 1	0.9853	0.8169	0.3576	0.4291	No	1.0286	0.331	0.0191	0.0529	No	No
95033_at	Jmjd1a	jumonji domain containing 1A	0.8788	0.1661	0.0114	0.2735	No	0.9109	0.0405	0.146	0.509	No	No
95034_f_at	Ipo4	importin 4	1.155	0.1133	0.0009	0.427	No	1.222	0.0185	0.0002	0.157	No	No
95035_at	Ipo4	importin 4	0.9911	0.8115	0.0095	0.3875	No	0.9992	0.972	0.539	0.542	No	No
95036_at	Calb2	calbindin 2	1.1876	0.0008	0.1539	0.2332	Yes	1.1633	0.0283	0.0611	0.28	No	Yes
95037_at	Cdk9	cyclin-dependent kinase 9 (CDC2-related kinase)	1.1069	0.0333	0.0388	0.2744	No	1.0302	0.563	0.101	0.476	No	No
95040_at	Pdcd6ip	programmed cell death 6 interacting protein	0.9267	0.5308	0.2914	0.8725	No	0.929	0.406	0.0681	0.875	No	No
95041_at	Josd2	Josephin domain containing 2	1.0083	0.8144	0.0008	0.565	No	1.0216	0.527	0.0009	0.778	No	No
95042_at	Yipf1	Yip1 domain family, member 1	1.0593	0.0791	0.0131	0.5054	No	0.9988	0.987	0.116	0.697	No	No
95043_at	Cyp2c70	cytochrome P450, family 2, subfamily c, polypeptide 70	0.9663	0.5086	0.4153	0.9528	No	0.953	0.287	0.54	0.614	No	No
95044_at	Med28	mediator of RNA polymerase II transcription, subunit 28 homolog (yeast)	0.9674	0.5474	0.1683	0.9856	No	0.9136	0.19	0.353	0.0336	No	No
95045_at	0610012D09Rik	RIKEN cDNA 0610012D09 gene	1.057	0.1128	0.4475	0.1888	No	1.0798	0.167	0.087	0.624	No	No
95046_s_at	Eif2b4	eukaryotic translation initiation factor 2B, subunit 4 delta	0.9765	0.4961	0.1441	0.2898	No	1.0013	0.992	0.039	0.245	No	No
95048_at	Sln	sarcolipin	1.0621	0.0581	0.0011	0.0467	No	1.0258	0.583	0.002	0.154	No	No
95049_at	Snrpd2	small nuclear ribonucleoprotein D2	0.9836	0.7607	0.056	0.0983	No	0.9731	0.629	0.0135	0.0237	No	No
95050_at	Chordc1	cysteine and histidine-rich domain (CHORD)-containing, zinc-binding protein 1	0.8737	0.0046	0.0005	0.6002	Yes	0.8601	0.0112	0.0045	0.469	No	Yes
95052_at	C1qdc2	C1q domain containing 2	0.9965	0.8692	0.0119	0.0981	No	1.035	0.448	0.0068	0.88	No	No
95053_s_at	Sdhb	succinate dehydrogenase complex, subunit B, iron sulfur (Ip)	1.0124	0.7558	0.0924	0.2136	No	1.043	0.247	0.0046	0.34	No	No
95054_at	Tars	threonyl-tRNA synthetase	1.0231	0.6013	0.2658	0.2328	No	1.0383	0.453	0.0807	0.471	No	No
95056_r_at	Dynlt3	dynein light chain Tctex-type 3	1.06	0.5061	0.8849	0.5767	No	1.0271	0.664	0.0086	0.787	No	No
95057_at	Herpud1	homocysteine-inducible, endoplasmic reticulum stress-inducible, ubiquitin-like domain member 1	0.9305	0.1081	0.7613	0.1346	No	1.1265	0.0485	0.0456	0.0384	No	No
95058_f_at	Brp44	brain protein 44	1.3188	0.0836	0.0122	0.257	No	1.0383	0.695	0.935	0.28	No	No
95059_at	Pnrc2	proline-rich nuclear receptor coactivator 2	1.0568	0.3895	0.399	0.5106	No	0.996	0.942	0.259	0.834	No	No
95060_at	Slc16a7	solute carrier family 16 (monocarboxylic acid transporters), member 7	0.9242	0.2964	0.7106	0.2312	No	0.9436	0.144	0.0147	0.908	No	No
95061_at	Bcas2	breast carcinoma amplified sequence 2	0.9503	0.614	0.3483	0.54	No	0.9439	0.385	0.597	0.62	No	No
95062_at	Cast	calpastatin	0.8581	0.1188	0.4317	0.5152	No	0.9778	0.749	0.0044	0.794	No	No
95063_at	Cdca7	cell division cycle associated 7	1.018	0.7967	0.0634	0.8809	No	1.0684	0.327	0.0168	0.842	No	No
95064_at	Acaa2	acetyl-Coenzyme A acyltransferase 2 (mitochondrial 3-oxoacyl-Coenzyme A thiolase)	1.0583	0.1027	0.0021	0.0051	No	1.0143	0.926	0.0216	0.0173	No	No
95065_at	Nfs1	nitrogen fixation gene 1 (S. cerevisiae)	0.9946	0.8358	0.0323	0.7998	No	1.0132	0.656	0.0412	0.631	No	No
95066_at	Taldo1	transaldolase 1	1.1617	0.039	0.3435	0.2448	No	1.1428	0.0721	0.339	0.329	No	No
95067_at	Mrpl2	mitochondrial ribosomal protein L2	1.0878	0.2878	0.4857	0.5068	No	1.0415	0.492	0.485	0.652	No	No
95068_at	Dnaja2	DnaJ (Hsp40) homolog, subfamily A, member 2	1.0297	0.4066	0.2902	0.1173	No	0.9806	0.807	0.479	0.723	No	No
95069_at	Ssrp1	structure specific recognition protein 1	1.0283	0.6564	0.2427	0.2722	No	1.0254	0.287	0.121	0.0429	No	No
95070_at	Nars	asparaginyl-tRNA synthetase	1.0091	0.9094	0.0162	0.7348	No	0.9521	0.449	0.0045	0.861	No	No
95071_at	Kcmf1	potassium channel modulatory factor 1	0.9778	0.4163	0.0754	0.0296	No	1.1128	0.0425	0.496	0.0446	No	No
95072_at	Cyc1	cytochrome c-1	1.1062	0.0381	0.1161	0.5897	No	1.0712	0.142	0.0832	0.553	No	No
95073_at	Pex19	peroxisome biogenesis factor 19	1.0242	0.7448	0.2764	0.264	No	1.2415	0.384	0.542	0.25	No	No
95074_at	Pex19	peroxisome biogenesis factor 19	1.0372	0.5379	0.1495	0.5943	No	1.0082	0.835	0.0098	0.778	No	No
95075_at	Nup35	nucleoporin 35	0.943	0.1656	0.2426	0.0163	No	0.8989	0.0339	0.37	0.002	No	No
95076_at	1500032L24Rik	RIKEN cDNA 1500032L24 gene	1.0254	0.4563	0.0128	0.3093	No	1.0758	0.0726	0.123	0.465	No	No
95077_at	Rabggtb	RAB geranylgeranyl transferase, b subunit	1.0029	0.9548	0.3123	0.3799	No	1.0017	0.683	0.0049	0.0153	No	No
95078_at	1200015F23Rik	RIKEN cDNA 1200015F23 gene	1.06	0.0086	0.0126	0.8722	Yes	0.9917	0.943	0.0844	0.145	No	No
95079_at	Pdgfra	platelet derived growth factor receptor, alpha polypeptide	0.9023	0.0055	0	0.0278	Yes	0.8894	0.0134	0.0003	0.5	No	Yes
95081_at	Exosc8	exosome component 8	1.095	0.1186	0.0019	0.0609	No	1.0172	0.644	0.0094	0.172	No	No

95082_at	Igfbp3	insulin-like growth factor binding protein 3	0.9513	0.4771	0.0035	0.9646	No	0.8539	0.0046	0.0001	0.981	Yes	No
95083_at	Igfbp3	insulin-like growth factor binding protein 3	0.8773	0.0108	0.0004	0.0084	No	0.8118	0.013	0.0004	0.0413	No	No
95084_f_at	Grhpr	glyoxylate reductase/hydroxypyruvate reductase	1.0287	0.7709	0.1921	0.5879	No	1.0203	0.713	0.038	0.323	No	No
95085_r_at	Grhpr	Glyoxylate reductase/hydroxypyruvate reductase	1.0805	0.537	0.0223	0.212	No	1.0947	0.371	0.0011	0.807	No	No
95086_at	2310036O22 Rik	RIKEN cDNA 2310036O22 gene	1.0684	0.0594	0.0083	0.2295	No	1.1759	0.0431	0.331	0.136	No	No
95090_at	Tmem134	transmembrane protein 134	1.0077	0.6679	0.0003	0.6065	No	1.0418	0.0963	0.0016	0.338	No	No
95091_at	Sec13l1	SEC13-like 1 (S. cerevisiae)	1.0901	0.0277	0.0432	0.1432	No	1.1022	0.0225	0.014	0.127	No	No
95092_at	Ppp3ca	protein phosphatase 3, catalytic subunit, alpha isoform	1.0583	0.5959	0.0357	0.3776	No	0.9291	0.47	0.262	0.566	No	No
95093_at	Ccar1	cell division cycle and apoptosis regulator 1	0.9061	0.1093	0.0006	0.1613	No	0.9123	0.253	0.0016	0.162	No	No
95094_g_at	Ccar1	cell division cycle and apoptosis regulator 1	0.8877	0.1724	0.0199	0.6735	No	0.8671	0.0579	0.0107	0.0802	No	No
95095_at	Flot1	flotillin 1	0.9441	0.2453	0.2028	0.3717	No	0.992	0.845	0.858	0.527	No	No
95096_at	Qk	quaking	1.0293	0.6227	0.4975	0.4086	No	0.9937	0.964	0.624	0.293	No	No
95097_at	Actr10	ARP10 actin-related protein 10 homolog (S. cerevisiae)	0.9755	0.6859	0.0158	0.1244	No	1.0217	0.787	0.149	0.0451	No	No
95098_at	Rnf26	ring finger protein 26	1.0293	0.1005	0.0807	0.648	No	0.9783	0.539	0.0553	0.819	No	No
95100_at	Anapc5	anaphase-promoting complex subunit 5	1.0326	0.086	0.0005	0.1505	No	1.0294	0.219	0.0012	0.0562	No	No
95101_at	Serinc1	serine incorporator 1	0.9979	0.9828	0.135	0.5912	No	0.9684	0.945	0.0951	0.345	No	No
95102_at	Scotin	scotin gene	1.0149	0.7199	0.0015	0.2949	No	0.9377	0.14	0.538	0.181	No	No
95103_at	2310065K24 Rik	RIKEN cDNA 2310065K24 gene	0.9702	0.4213	0.0099	0.0144	No	1.023	0.822	0.006	0.1	No	No
95104_at	Sdc2	syndecan 2	0.9728	0.3845	0.1851	0.5163	No	0.9433	0.0002	0.0016	0.0016	No	No
95105_at	Higd2a	HIG1 domain family, member 2A	0.9804	0.5842	0.1596	0.0607	No	1.0056	0.856	0.353	0.135	No	No
95108_at	2700055K07 Rik	RIKEN cDNA 2700055K07 gene	0.935	0.4613	0.1069	0.4548	No	0.9545	0.406	0.114	0.523	No	No
95109_at	Nol5a	nucleolar protein 5A	0.9177	0.2333	0.5042	0.0298	No	1.0093	0.924	0.0569	0.905	No	No
95110_at	Ppil2	peptidylprolyl isomerase (cyclophilin)-like 2	0.9533	0.0979	0.0451	0.1623	No	0.977	0.612	0.35	0.334	No	No
95111_i_at	Ppil2	peptidylprolyl isomerase (cyclophilin)-like 2	0.8652	0.2734	0.8091	0.6088	No	0.9099	0.115	0.0394	0.0788	No	No
95112_f_at	Ppil2	peptidylprolyl isomerase (cyclophilin)-like 2	0.9796	0.5321	0.0966	0.589	No	0.9626	0.379	0.0085	0.623	No	No
95113_at	Cuta	cutA divalent cation tolerance homolog (E. coli)	1.0576	0.3056	0.0552	0.1104	No	1.1028	0.046	0.058	0.0157	No	No
95114_s_at	Cuta	cutA divalent cation tolerance homolog (E. coli)	1.0279	0.2438	0.0016	0.598	No	1.1018	0.0028	0.0003	0.139	Yes	No
95117_at	Igf2r	insulin-like growth factor 2 receptor	0.9499	0.4755	0.1003	0.2308	No	0.9501	0.792	0.0137	0.337	No	No
95118_r_at	Kif22	kinesin family member 22	1.0804	0.1703	0.0017	0.1606	No	1.0622	0.226	0.0799	0.0798	No	No
95119_at	1110038D17 Rik	RIKEN cDNA 1110038D17 gene	0.9596	0.3384	0.0146	0.1616	No	0.9687	0.433	0.0157	0.378	No	No
95120_at	Tspan13	tetraspanin 13	1.0684	0.4811	0.0318	0.3709	No	0.9895	0.891	0.158	0.616	No	No
95121_at	Pole4	polymerase (DNA-directed), epsilon 4 (p12 subunit)	1.0374	0.5493	0.9712	0.4651	No	1.0641	0.608	0.183	0.888	No	No
95122_g_at	Pole4	polymerase (DNA-directed), epsilon 4 (p12 subunit)	1.0861	0.2452	0.7567	0.428	No	1.0429	0.468	0.419	0.406	No	No
95123_at	4930566A11 Rik	RIKEN cDNA 4930566A11 gene	1.1299	0.0498	0.2998	0.2717	No	1.0759	0.0613	0.0158	0.0369	No	No
95124_i_at	Rbx1	ring-box 1	1.0521	0.2923	0.0001	0.7358	No	1.0223	0.364	0	0.222	No	No
95125_f_at	Rbx1	ring-box 1	1.0865	0.456	0.0062	0.4297	No	1.0638	0.543	0.0023	0.992	No	No
95128_at	Anapc2	anaphase promoting complex subunit 2	0.9747	0.4677	0.9823	0.5481	No	0.9721	0.354	0.266	0.141	No	No
95129_at	Ncor2	nuclear receptor co-repressor 2	1.0781	0.048	0.0021	0.06	No	1.048	0.0424	0.0002	0.0151	No	No
95131_f_at	Ndufb2	NADH dehydrogenase (ubiquinone) 1 beta subcomplex, 2	0.9781	0.6387	0.8687	0.0324	No	0.9782	0.804	0.128	0.281	No	No
95132_r_at	Ndufb2	NADH dehydrogenase (ubiquinone) 1 beta subcomplex, 2	0.9667	0.4777	0.0112	0.1382	No	0.9502	0.215	0.0056	0.127	No	No
95133_at	Asns	asparagine synthetase	0.9785	0.4962	0.9379	0.2269	No	0.9864	0.72	0.523	0.734	No	No
95134_at	Mid1ip1	Mid1 interacting protein 1 (gastrulation specific G12-like (zebrafish))	1.0254	0.3549	0.5406	0.1937	No	0.9818	0.506	0.108	0.0056	No	No
95135_at	Mid1ip1	Mid1 interacting protein 1 (gastrulation specific G12-like (zebrafish))	1.079	0.2151	0.0152	0.3503	No	1.0602	0.26	0.0326	0.629	No	No
95136_at	Arl6ip4	ADP-ribosylation factor-like 6 interacting protein 4	0.9427	0.0796	0.2332	0.0135	No	0.9805	0.779	0.985	0.117	No	No
95137_at	Tmem97	transmembrane protein 97	1.0982	0.0539	0.0012	0.4921	No	1.1363	0.0074	0.0004	0.209	Yes	No
95138_at	Wipi2	WD repeat domain, phosphoinositide interacting 2	0.948	0.0222	0.0067	0.0068	No	0.9399	0.12	0.11	0.0788	No	No
95139_at	Wipi2	WD repeat domain, phosphoinositide interacting 2	1.0043	0.8974	0.0016	0.7548	No	0.997	0.919	0.0198	0.837	No	No
95140_at	5230400G24 Rik	RIKEN cDNA 5230400G24 gene	1.0031	0.94	0.0248	0.7524	No	1.0361	0.212	0.0016	0.101	No	No
95141_at	Capzb	capping protein (actin filament) muscle Z-line, beta	0.9937	0.9181	0.0706	0.8049	No	1.0498	0.217	0.015	0.323	No	No
95142_s_at	Capzb	capping protein (actin filament) muscle Z-line, beta	1.0179	0.7211	0.0039	0.3483	No	1.0321	0.621	0.0007	0.328	No	No
95144_at	Arpc1a	actin related protein 2/3 complex, subunit 1A	0.9284	0.0466	0.0121	0.9973	No	0.9534	0.193	0.0004	0.248	No	No
95146_at	Tmem66	transmembrane protein 66	0.8998	0.1696	0.8731	0.9145	No	0.9151	0.0958	0.779	0.443	No	No
95147_at	Pgls	6-phosphogluconolactonase	1.0752	0.0698	0.0002	0.1028	No	1.0383	0.0478	0	0.0319	No	No
95148_at	Ak2	adenylate kinase 2	1.0238	0.5339	0.0012	0.0263	No	1.0369	0.348	0.0025	0.209	No	No
95149_at	Copz1	coatomer protein complex, subunit zeta 1	1.0659	0.1634	0.0026	0.8507	No	1.0074	0.716	0.0018	0.273	No	No
95150_at	Plekho1	pleckstrin homology domain containing, family O member 1	0.926	0.0549	0.0067	0.1306	No	0.9995	0.965	0.004	0.975	No	No
95151_at	Plekho1	pleckstrin homology domain containing, family O member 1	0.9609	0.1348	0.0004	0.177	No	0.9379	0.122	0.0016	0.0764	No	No
95152_g_at	Plekho1	pleckstrin homology domain containing, family O member 1	0.9533	0.3097	0.017	0.7931	No	1.0454	0.337	0.056	0.558	No	No
95153_at	Cdc42se2	CDC42 small effector 2	1.0492	0.3235	0.0061	0.1547	No	1.0356	0.445	0.0024	0.296	No	No
95154_at	Scand1	SCAN domain-containing 1	0.973	0.7037	0.929	0.3677	No	1.0497	0.597	0.322	0.291	No	No
95155_at	Znrf1	zinc and ring finger 1	0.9887	0.6839	0.0007	0.2497	No	0.9866	0.848	0.0035	0.318	No	No
95156_g_at	Znrf1	zinc and ring finger 1	0.8202	0.0574	0.4626	0.6882	No	0.8666	0.0743	0.513	0.61	No	No
95157_at	Znrf1	zinc and ring finger 1	1.0029	0.9502	0.0066	0.7599	No	1.0302	0.295	0.0023	0.149	No	No
95158_at	H2-Ke2	H2-K region expressed gene 2	0.9987	0.9799	0.0362	0.7747	No	0.8947	0.0563	0.0082	0.742	No	No
95159_at	Mrps18b	mitochondrial ribosomal protein S18B	0.9696	0.5463	0.8197	0.6896	No	0.9407	0.268	0.782	0.196	No	No
95161_at	Ctdsp2	CTD (carboxy-terminal domain, RNA polymerase II, polypeptide A) small phosphatase 2	0.956	0.1103	0.0318	0.7385	No	0.9246	0.0288	0.02	0.0656	No	No
95164_at	D14Abb1e	DNA segment, Chr 14, Abbott 1 expressed	1.0274	0.4478	0.0267	0.7404	No	1.0123	0.778	0.0425	0.359	No	No
95165_at	Sema6d	sema domain, transmembrane domain (TM), and cytoplasmic domain, (semaphorin) 6D	1.0997	0.0594	0.0677	0.0419	No	1.0714	0.197	0.0248	0.485	No	No
95177_at	AA414993	expressed sequence AA414993	1.0944	0.2878	0.9806	0.2912	No	1.334	0.0526	0.281	0.107	No	No
95182_at	---	---	1.0301	0.2855	0.4661	0.2539	No	1.0208	0.623	0.162	0.374	No	No
95183_i_at	---	Transcribed locus	0.8902	0.3198	0.877	0.5846	No	0.9959	1	0.424	0.387	No	No
95184_f_at	---	Transcribed locus	0.9178	0.5525	0.9871	0.8737	No	0.9579	0.594	0.107	0.993	No	No
95215_f_at	Ubc	ubiquitin C	0.9327	0.1417	0.4443	0.1186	No	0.9292	0.0268	0.0016	0.0026	No	No
95228_f_at	Ang3	angiogenin, ribonuclease A family, member 3	0.9192	0.2503	0.0987	0.9366	No	0.9077	0.386	0.298	0.526	No	No
95232_at	Hnrpl	heterogeneous nuclear ribonucleoprotein L	0.8059	0.0402	0.0301	0.6902	No	0.9232	0.375	0.532	0.975	No	No
95244_at	Zfp40	zinc finger protein 40	1.0251	0.5499	0.0379	0.8321	No	1.1486	0.0392	0.0073	0.0798	No	No
95246_at	---	---	1.0619	0.3019	0.2219	0.0538	No	1.152	0.173	0.199	0.155	No	No
95247_at	BB001228	expressed sequence BB001228	1.0397	0.3606	0.3033	0.7914	No	1.083	0.339	0.167	0.534	No	No
95248_at	AA517841	expressed sequence AA517841	0.8518	0.0168	0.2917	0.9375	No	0.9261	0.158	0.332	0.823	No	No
95249_at	AA517858	expressed sequence AA517858	1.0631	0.4103	0.6113	0.0987	No	1.3438	0.0889	0.0001	0.344	No	No
95275_at	Il9r	interleukin 9 receptor	1.1555	0.0569	0.3157	0.4406	No	1.0823	0.384	0.296	0.589	No	No
95281_at	---	---	0.9883	0.7888	0.1298	0.9378	No	0.9098	0.237	0.948	0.0858	No	No
95282_at	Hsp90aa1	heat shock protein 90kDa alpha (cytosolic), class A member 1	1.0663	0.5243	0.0578	0.4087	No	1.0535	0.489	0.307	0.355	No	No
95283_at	Abcc2	ATP-binding cassette, sub-family C (CFTR/MRP), member 2	0.937	0.3367	0.3303	0.8101	No	0.9868	0.896	0.195	0.924	No	No
95284_at	---	---	0.8574	0.0951	0.0222	0.2227	No	0.9163	0.148	0.0249	0.973	No	No
95285_at	---	---	1.1478	0.0193	0.0003	0.0517	No	1.1187	0.0365	0.0017	0.0233	No	No
95286_at	Clu	clusterin	1.2118	0.0063	0.3467	0.1492	Yes	1.2016	0.0127	0.695	0.149	No	Yes
95287_at	Luc7l2	LUC7-like 2 (S. cerevisiae)	1.0497	0.2349	0.0002	0.0222	No	1.0042	0.25	0	0.0098	No	No
95288_i_at	A430106J12 Rik	RIKEN cDNA A430106J12 gene	1.1017	0.5126	0.0051	0.2174	No	1.0315	0.175	0.0002	0.025	No	No
95289_r_at	A430106J12 Rik	RIKEN cDNA A430106J12 gene	1.059	0.6433	0.0215	0.6006	No	1.087	0.396	0.0031	0.467	No	No
95290_at	Crhr1	corticotropin releasing hormone receptor 1	1.0101	0.7969	0.0072	0.6564	No	0.9577	0.546	0.542	0.369	No	No
95291_r_at	Taf15	TAF15 RNA polymerase II, TATA box binding protein (TBP)-associated factor	1.0055	0.8706	0.5999	0.0748	No	1.0902	0.77	0.827	0.411	No	No
95292_at	Itga4	integrin alpha 4	1.0173	0.8317	0.8127	0.7455	No	1.1314	0.178	0.12	0.0464	No	No
95293_at	Calr4	Calreticulin 4	0.9841	0.6728	0.5214	0.8731	No	0.9401	0.481	0.245	0.929	No	No
95294_at	Centg1	Centaurin, gamma 1	1.0154	0.6292	0.0156	0.6652	No	0.96	0.722	0.0013	0.024	No	No
95295_s_at	Flt3	FMS-like tyrosine kinase 3	0.9939	0.9376	0.5086	0.3925	No	0.9846	0.788	0.148	0.799	No	No
95296_r_at	Flt3	FMS-like tyrosine kinase 3	1.0052	0.9217	0.0091	0.3493	No	0.9986	0.996	0.12	0.711	No	No
95297_at	Hoxa1	homeo box A1	1.0703	0.3347	0.314	0.8288	No	1.0915	0.227	0.0598	0.403	No	No
95298_at	Epha3	Eph receptor A3	0.9648	0.6148	0.1172	0.3525	No	0.9826	0.651	0.0649	0.21	No	No

95299_at	Dnahc8 /// LOC547338	dynein, axonemal, heavy chain 8 /// similar to dynein, axonemal, heavy chain 8	1.0082	0.8687	0.3573	0.9641	No	1.1212	0.599	0.34	0.929	No	No
95300_at	Prg3	proteoglycan 3	0.9976	0.9243	0.0072	0.0329	No	1.1077	0.0582	0.0099	0.0247	No	No
95301_at	S100a5	S100 calcium binding protein A5	1.0625	0.3073	0.5306	0.933	No	1.0255	0.541	0.559	0.141	No	No
95302_at	Frap1	FK506 binding protein 12-rapamycin associated protein 1	1.0487	0.3223	0.2229	0.1477	No	1.0764	0.349	0.0291	0.0645	No	No
95303_at	Ifitm6	interferon induced transmembrane protein 6	0.9247	0.1278	0.3086	0.8119	No	0.911	0.0459	0.826	0.535	No	No
95304_at	Attp	signaling molecule ATTP	0.9071	0.0302	0.1136	0.3238	No	1.0619	0.512	0.749	0.159	No	No
95305_at	A630098A13 Rik	RIKEN cDNA A630098A13 gene	1.0386	0.5649	0.6538	0.2762	No	1.078	0.288	0.0202	0.0252	No	No
95306_at	Uts2r	urotensin 2 receptor	1.0276	0.5014	0.0558	0.0126	No	1.0451	0.381	0.389	0.428	No	No
95307_at	Srcrb4d	Scavenger receptor cysteine rich domain containing, group B (4 domains)	0.9565	0.2917	0.2167	0.6133	No	1.0941	0.154	0.397	0.543	No	No
95308_at	Adcy4	adenylate cyclase 4	0.945	0.0789	0.0134	0.163	No	1.057	0.029	0.0002	0.0208	No	No
95309_at	Dnahc8	Dynein, axonemal, heavy chain 8	0.9889	0.8158	0.7653	0.2613	No	1.0329	0.504	0.211	0.365	No	No
95310_at	Btc	betacellulin, epidermal growth factor family member	0.9167	0.2117	0.038	0.3946	No	0.7683	0.0299	0.0921	0.487	No	No
95311_at	Bat2	HLA-B associated transcript 2	1.0202	0.6525	0.0621	0.3819	No	0.9609	0.338	0.0849	0.178	No	No
95312_at	Hoxc5	homeo box C5	0.957	0.3531	0.4471	0.9934	No	1.0093	0.882	0.442	0.898	No	No
95313_at	F2r	coagulation factor II (thrombin) receptor	0.9587	0.5622	0.0515	0.4228	No	0.9679	0.692	0.256	0.591	No	No
95314_at	Ttl	tubulin tyrosine ligase	0.9556	0.4553	0.2004	0.0808	No	0.9655	0.566	0.33	0.154	No	No
95315_at	Zfp14	zinc finger protein 14	1.0298	0.5807	0.0256	0.662	No	1.0698	0.28	0.0107	0.648	No	No
95316_at	Fgf18	fibroblast growth factor 18	0.914	0.08	0.0128	0.6382	No	1.011	0.925	0.0153	0.45	No	No
95317_at	Bsn	bassoon	1.0172	0.7634	0.0569	0.136	No	1.0336	0.559	0.573	0.108	No	No
95318_at	Zfp105	zinc finger protein 105	0.9931	0.8856	0.0126	0.9599	No	0.9887	0.712	0.0041	0.844	No	No
95319_at	Ntsr1	neurotensin receptor 1	0.952	0.3964	0.3331	0.2212	No	0.9734	0.115	0.0569	0.0051	No	No
95320_at	Klrg1	killer cell lectin-like receptor subfamily G, member 1	1.0037	0.959	0.4926	0.3278	No	1.071	0.56	0.0385	0.808	No	No
95321_at	Htr4	5 hydroxytryptamine (serotonin) receptor 4	1.0872	0.4094	0.8417	0.7429	No	1.1029	0.368	0.13	0.176	No	No
95322_g_at	Htr4	5 hydroxytryptamine (serotonin) receptor 4	1.0315	0.5532	0.3011	0.0706	No	1.0451	0.429	0.315	0.17	No	No
95323_at	Htr4	5 hydroxytryptamine (serotonin) receptor 4	1.0774	0.1417	0.1798	0.1224	No	1.1058	0.086	0.0131	0.0424	No	No
95324_at	Atp2b2	ATPase, Ca++ transporting, plasma membrane 2	0.9744	0.4979	0.0346	0.0425	No	0.9807	0.807	0.822	0.767	No	No
95325_at	Pscd1	pleckstrin homology, Sec7 and coiled-coil domains 1	0.9857	0.7813	0.1995	0.7343	No	1.1016	0.179	0.656	0.112	No	No
95326_at	Tspyl1	testis-specific protein, Y-encoded-like 1	0.9956	0.9485	0.4391	0.6177	No	1.0114	0.869	0.252	0.781	No	No
95327_at	Sh3pxd2a	SH3 and PX domains 2A	0.9775	0.4323	0.0139	0.8402	No	0.9311	0.119	0.816	0.495	No	No
95328_at	Fut9	fucosyltransferase 9	0.8996	0.0899	0.2794	0.7721	No	0.8472	0.0813	0.113	0.649	No	No
95329_at	Ern2	endoplasmic reticulum (ER) to nucleus signalling 2	0.9978	0.9594	0.1766	0.4119	No	1.0332	0.485	0.0767	0.551	No	No
95330_at	G6pc2	glucose-6-phosphatase, catalytic, 2	1.018	0.7507	0.0555	0.3918	No	0.9441	0.607	0.0724	0.865	No	No
95331_at	Rgr	retinal G protein coupled receptor	0.9387	0.5457	0.2954	0.6176	No	0.9892	0.756	0.0063	0.487	No	No
95332_at	Htr2b	5-hydroxytryptamine (serotonin) receptor 2B	0.9742	0.6596	0.3609	0.9792	No	1.0594	0.436	0.317	0.101	No	No
95333_at	Il18rap	interleukin 18 receptor accessory protein	0.9143	0.0257	0.0343	0.3835	No	1.045	0.191	0.0103	0.0356	No	No
95334_at	Ntrk3	neurotrophic tyrosine kinase, receptor, type 3	0.8761	0.1029	0.0934	0.2537	No	1.0935	0.198	0.366	0.184	No	No
95335_at	Cx3cr1	chemokine (C-X3-C) receptor 1	1.0788	0.031	0.0143	0.0331	No	0.985	0.699	0.0001	0.368	No	No
95336_at	Hif3a	hypoxia inducible factor 3, alpha subunit	0.9155	0.2237	0.7646	0.423	No	0.9691	0.562	0.0048	0.848	No	No
95337_at	Uty	ubiquitously transcribed tetratricopeptide repeat gene, Y chromosome	1.1705	0.0146	0.006	0.3279	No	1.0906	0.0417	0.0004	0.132	No	No
95338_s_at	Mmp12	matrix metallopeptidase 12	0.9931	0.9284	0.5294	0.945	No	1.0005	0.68	0.004	0.0383	No	No
95339_r_at	Mmp12	matrix metallopeptidase 12	1.0392	0.3611	0.0004	0.0023	No	0.9478	0.986	0.0016	0.0157	No	No
95340_at	Mt3	metallothionein 3	0.9933	0.7676	0.0016	0.8322	No	0.9935	0.867	0.0145	0.568	No	No
95341_at	Tbrg4	transforming growth factor beta regulated gene 4	0.9805	0.6391	0.5543	0.6448	No	0.9384	0.377	0.0173	0.823	No	No
95342_at	V1ra2	vomeronasal 1 receptor, A2	1.0088	0.7092	0.9726	0.5106	No	1.1097	0.467	0.0648	0.0753	No	No
95343_at	Padi1	peptidyl arginine deiminase, type I	0.9458	0.184	0.1264	0.6086	No	0.9881	0.849	0.402	0.291	No	No
95344_at	Il13ra2	interleukin 13 receptor, alpha 2	1.0471	0.3079	0.3568	0.9969	No	1.0234	0.753	0.103	0.99	No	No
95345_at	Tpbg	trophoblast glycoprotein	1.018	0.507	0.1366	0.6652	No	1.0654	0.0957	0.31	0.138	No	No
95346_at	Rex2	reduced expression 2	0.9342	0.3665	0.2403	0.443	No	0.9655	0.989	0.0822	0.526	No	No
95347_at	Myt1	myelin transcription factor 1	0.9469	0.3127	0.8802	0.5752	No	0.9686	0.604	0.267	0.162	No	No
95348_at	Cxcl1	chemokine (C-X-C motif) ligand 1	1.2225	0.0025	0.0717	0.0054	No	1.1661	0.135	0.519	0.0485	No	No
95349_g_at	Cxcl1	chemokine (C-X-C motif) ligand 1	1.0476	0.4676	0.3797	0.4028	No	0.9817	0.942	0.438	0.786	No	No
95350_at	Ttr	transthyretin	1.2116	0.0072	0.0007	0.5451	Yes	1.1936	0.0034	0.0003	0.75	Yes	Yes
95351_at	Ncoa6	nuclear receptor coactivator 6	0.8404	0.0083	0.3596	0.0056	No	0.834	0.0004	0.0022	0.0006	No	No
95352_at	D8Ertd28e	DNA segment, Chr 8, ERATO Doi 28, expressed	0.9547	0.2042	0.5059	0.7581	No	1.0719	0.367	0.084	0.0891	No	No
95353_at	Crxos1	Crx opposite strand transcript 1	0.9339	0.0495	0.7269	0.9284	No	0.8522	0.0746	0.134	0.229	No	No
95354_at	D6Ertd439e	DNA segment, Chr 6, ERATO Doi 439, expressed	1.0719	0.3994	0.173	0.5316	No	1.1443	0.172	0.196	0.868	No	No
95355_at	Agtrap	angiotensin II, type I receptor-associated protein	1.0228	0.7645	0.0732	0.8191	No	0.9351	0.154	0.01	0.612	No	No
95356_at	Apoe	apolipoprotein E	0.984	0.4031	0.0013	0.0024	No	0.9502	0.251	0.0004	0.0012	No	No
95357_at	Nvl	nuclear VCP-like	1.0202	0.69	0.0238	0.1058	No	0.8967	0.539	0.152	0.406	No	No
95358_at	Pip5k2a	phosphatidylinositol-4-phosphate 5-kinase, type II, alpha	0.9804	0.663	0.3141	0.8939	No	0.9474	0.042	0.0036	0.0026	No	No
95359_at	Hsp90ab1	heat shock protein 90kDa alpha (cytosolic), class B member 1	1.0047	0.8661	0.5713	0.0963	No	0.9752	0.487	0.0389	0.578	No	No
95360_at	Zcchc6	zinc finger, CCHC domain containing 6	1.0566	0.3249	0.1705	0.3289	No	1.0243	0.722	0.69	0.288	No	No
95363_at	Gzmm	granzyme M (lymphocyte met-ase 1)	1.0514	0.3911	0.0811	0.9812	No	1.0604	0.334	0.477	0.774	No	No
95364_at	Gna14	guanine nucleotide binding protein, alpha 14	1.0026	0.9747	0.3713	0.814	No	1.0546	0.455	0.0018	0.709	No	No
95366_at	---	---	0.9918	0.8224	0.3291	0.2406	No	0.9319	0.0888	0.003	0.852	No	No
95368_at	Plxna2	plexin A2	0.899	0.0781	0.1746	0.4719	No	0.9573	0.623	0.0203	0.229	No	No
95369_at	---	---	0.9512	0.4955	0.955	0.9551	No	0.958	0.375	0.275	0.0432	No	No
95370_at	Cdh4	cadherin 4	0.9531	0.6159	0.3556	0.3098	No	1.0042	0.901	0.2	0.674	No	No
95371_g_at	Cdh4	cadherin 4	1.0552	0.5058	0.0592	0.8723	No	1.0126	0.806	0.0978	0.749	No	No
95372_at	Pskh1	Protein serine kinase H1	0.9822	0.7205	0.0136	0.8147	No	0.9498	0.718	0.146	0.293	No	No
95373_at	Cd2	CD2 antigen	1.0181	0.7172	0.1538	0.1195	No	1.0314	0.571	0.946	0.62	No	No
95374_f_at	Cilp2	cartilage intermediate layer protein 2	1.07	0.0652	0.0751	0.1246	No	1.0478	0.213	0.596	0.574	No	No
95375_r_at	Cilp2	cartilage intermediate layer protein 2	1.0472	0.207	0.046	0.168	No	1.0388	0.165	0.0314	0.734	No	No
95376_at	Clcn1	chloride channel 1	0.9811	0.7162	0.6051	0.5967	No	0.9802	0.826	0.967	0.793	No	No
95377_at	Fgf11	fibroblast growth factor 11	1.02	0.5897	0.0168	0.6699	No	1.036	0.252	0.395	0.192	No	No
95378_at	AI842396	expressed sequence AI842396	1.0015	0.9777	0.0508	0.6285	No	1.1197	0.184	0.017	0.0464	No	No
95379_at	Mab21l2	mab-21-like 2 (C. elegans)	0.9001	0.1663	0.4769	0.6828	No	0.8515	0.0554	0.0508	0.49	No	No
95380_at	Cd244	CD244 natural killer cell receptor 2B4	1.0118	0.7679	0.1785	0.0361	No	1.0985	0.113	0.25	0.0673	No	No
95381_at	Kcnk7	potassium channel, subfamily K, member 7	1.0674	0.2335	0.7161	0.3961	No	1.1837	0.0963	0.298	0.122	No	No
95382_at	AI646383	expressed sequence AI646383	1.0174	0.6692	0.5765	0.4293	No	0.9347	0.448	0.0432	0.769	No	No
95383_at	Tm7sf2	transmembrane 7 superfamily member 2	1.02	0.562	0.0053	0.8741	No	0.9411	0.22	0.247	0.766	No	No
95385_at	---	CDNA, clone:Y1G0143G21, strand:unspecified	1.0399	0.5979	0.7243	0.1653	No	1.0017	0.984	0.885	0.265	No	No
95386_at	Lycat	lysocardiolipin acyltransferase	0.9893	0.652	0.8996	0.3104	No	0.9631	0.125	0.0357	0.847	No	No
95387_f_at	Sema4b	Sema domain, immunoglobulin domain (Ig), transmembrane domain (TM) and short cytoplasmic domain, (semaphorin) 4B	1.0123	0.8096	0.0001	0.0665	No	0.9841	0.847	0.0006	0.336	No	No
95388_at	Hist1h1d	histone 1, H1d	0.9701	0.5999	0.3347	0.6048	No	0.913	0.0292	0.0251	0.0126	No	No
95389_at	Cnga1	cyclic nucleotide gated channel alpha 1	0.9126	0.2785	0.3982	0.9707	No	0.9066	0.246	0.0136	0.297	No	No
95390_at	Npas2	neuronal PAS domain protein 2	1.0027	0.9789	0.6101	0.986	No	1.0426	0.149	0.0026	0.0157	No	No
95391_at	Vax1	ventral anterior homeobox containing gene 1	1.0846	0.0918	0.1041	0.4809	No	1.0103	0.837	0.354	0.564	No	No
95392_at	Gcnt2	glucosaminyl (N-acetyl) transferase 2, I-branching enzyme	1.0106	0.7725	0.0067	0.1717	No	1.0945	0.24	0.0262	0.133	No	No
95393_at	Btbd3	BTB (POZ) domain containing 3	0.8714	0.0203	0.0079	0.3927	No	0.91	0.16	0.0381	0.27	No	No
95395_at	Ythdf3	YTH domain family 3	1.0734	0.5445	0.002	0.2437	No	0.9523	0.892	0.0002	0.123	No	No
95396_at	---	---	1.0169	0.6912	0.0447	0.0977	No	0.7948	0.955	0.0166	0.637	No	No
95397_at	D430019H16Rik	RIKEN cDNA D430019H16 gene	1.0553	0.0958	0.0001	0.0452	No	1.0733	0.0468	0.0001	0.223	No	No
95398_at	Ankrd44	Ankyrin repeat domain 44	1.0578	0.0078	0.0013	0.001	No	0.9929	0.894	0.0055	0.0993	No	No
95399_at	---	PREDICTED: Mus musculus similar to dachsous 2 isoform 2 (LOC627094), mRNA	1.0839	0.2171	0.0763	0.7564	No	1.0932	0.137	0.522	0.176	No	No
95400_i_at	Helb	helicase (DNA) B	1.0053	0.9332	0.7962	0.9551	No	1.0211	0.821	0.844	0.392	No	No
95401_at	BC038286	cDNA sequence BC038286	1.0465	0.3143	0.3959	0.4682	No	1.0172	0.552	0.684	0.22	No	No
95404_at	Pafah1b2	platelet-activating factor acetylhydrolase, isoform 1b, alpha2 subunit	0.9939	0.9406	0.3065	0.1779	No	0.9936	0.929	0.567	0.34	No	No
95405_at	Mesdc2	mesoderm development candiate 2	1.0308	0.2768	0.2986	0.0958	No	1.0531	0.193	0.0877	0.417	No	No
95406_at	1810037I17 Rik	RIKEN cDNA 1810037I17 gene	0.9533	0.2023	0.001	0.1453	No	0.9833	0.0376	0	0.0022	No	No
95407_at	Pah	phenylalanine hydroxylase	1.0189	0.356	0.7716	0.7345	No	1.207	0.0042	0.0015	0.0009	No	No
95408_at	2310003F16 Rik	RIKEN cDNA 2310003F16 gene	1.159	0.0968	0.0279	0.4605	No	1.0276	0.627	0.0058	0.634	No	No
95409_at	1110019J04 Rik	RIKEN cDNA 1110019J04 gene	0.9559	0.2107	0.494	0.2308	No	0.9961	0.901	0.12	0.588	No	No
95410_s_at	Col2a1	procollagen, type II, alpha 1	1.0436	0.4546	0.5035	0.0681	No	1.1263	0.204	0.275	0.0687	No	No

95411_at	1110004F10 Rik	RIKEN cDNA 1110004F10 gene	1.1259	0.002	0.0002	0.7443	Yes	1.0635	0.0646	0.0069	0.357	No	No
95412_at	Pdcd6	programmed cell death 6	1.0609	0.3562	0.26	0.9714	No	0.9875	0.665	0.0981	0.279	No	No
95413_at	Ociad1	OCIA domain containing 1	0.9881	0.8159	0.2003	0.3346	No	1.0019	0.954	0.24	0.853	No	No
95414_i_at	C1r	complement component 1, r subcomponent	0.9413	0.3096	0.9112	0.3147	No	1.0213	0.43	0.591	0.0549	No	No
95415_f_at	C1r	complement component 1, r subcomponent	0.8658	0.0271	0.7572	0.2007	No	1.0348	0.606	0.614	0.99	No	No
95416_at	Usp15	ubiquitin specific peptidase 15	0.9143	0.2292	0.076	0.8643	No	0.9187	0.0288	0.435	0.188	No	No
95417_at	Mgat2	mannoside acetylglucosaminyltransferase 2	1.1157	0.2972	0.3482	0.2707	No	1.0567	0.371	0.0402	0.0841	No	No
95418_at	Rasl11b	RAS-like, family 11, member B	0.999	0.9835	0.0455	0.4155	No	1.0145	0.829	0.0203	0.324	No	No
95419_at	H1f0	H1 histone family, member 0	1.047	0.4616	0.0429	0.8426	No	1.072	0.298	0.0162	0.355	No	No
95420_at	Pgd	phosphogluconate dehydrogenase	0.9983	0.9435	0.0003	0.126	No	0.9557	0.207	0.0041	0.167	No	No
95423_at	Pdia4	protein disulfide isomerase associated 4	1.0437	0.3329	0.0003	0.6004	No	0.9928	0.978	0.001	0.535	No	No
95424_at	Sumo3	SMT3 suppressor of mif two 3 homolog 3 (yeast)	1.0381	0.4448	0.7607	0.3086	No	1.0093	0.713	0.0341	0.222	No	No
95425_at	Acadl	acyl-Coenzyme A dehydrogenase, long-chain	0.9526	0.4818	0.0057	0.9791	No	0.9706	0.491	0.0115	0.0108	No	No
95426_at	Echs1	enoyl Coenzyme A hydratase, short chain, 1, mitochondrial	0.8791	0.0025	0.0059	0.0434	Yes	0.9101	0.0034	0.0001	0.0184	Yes	Yes
95427_at	Rpa1	replication protein A1	1.0714	0.0198	0.0017	0.0722	No	1.0547	0.118	0.0214	0.614	No	No
95428_at	Imp4	IMP4, U3 small nucleolar ribonucleoprotein, homolog (yeast)	0.9094	0.0426	0.0946	0.8019	No	0.9613	0.429	0.0073	0.42	No	No
95430_f_at	Spg21	spastic paraplegia 21 homolog (human)	1.1767	0.2037	0.0472	0.5557	No	1.1319	0.321	0.0207	0.472	No	No
95431_at	Tomm70a	translocase of outer mitochondrial membrane 70 homolog A (yeast)	1.1009	0.3826	0.0201	0.8867	No	1.1036	0.234	0.0053	0.163	No	No
95432_f_at	Tomm70a	translocase of outer mitochondrial membrane 70 homolog A (yeast)	1.0496	0.0627	0	0.1646	No	1.0637	0.227	0.0021	0.253	No	No
95433_at	Ddx54	DEAD (Asp-Glu-Ala-Asp) box polypeptide 54	0.9303	0.0694	0.0002	0.5212	No	0.9737	0.631	0.0006	0.873	No	No
95434_at	Arpc3	actin related protein 2/3 complex, subunit 3	1.0149	0.8328	0.1913	0.5336	No	0.9945	0.914	0.286	0.6	No	No
95435_at	Arl8b	ADP-ribosylation factor-like 8B	1.0125	0.7402	0.0372	0.077	No	0.9276	0.275	0.0015	0.686	No	No
95436_at	Sst	somatostatin	1.1361	0.0026	0.32	0.0157	Yes	1.1449	0.0188	0.0792	0.133	No	Yes
95437_at	Rfwd2	ring finger and WD repeat domain 2	1.0178	0.5542	0.0012	0.0096	No	0.967	0.374	0.0239	0.18	No	No
95438_at	Rhot1	ras homolog gene family, member T1	0.9944	0.8189	0.622	0.252	No	0.8755	0.0312	0.0346	0.629	No	No
95439_at	Aadac	arylacetamide deacetylase (esterase)	1.0848	0.2989	0.5493	0.9101	No	1.1371	0.035	0.143	0.234	No	No
95440_at	2810432L12 Rik	RIKEN cDNA 2810432L12 gene	1.0005	0.9909	0.7891	0.1739	No	0.9969	0.936	0.3	0.667	No	No
95441_at	Timm23	translocase of inner mitochondrial membrane 23 homolog (yeast)	1.1658	0.0431	0.5933	0.9686	No	1.1757	0.0072	0.0077	0.437	Yes	Yes
95442_at	BC010787	cDNA sequence BC010787	0.9784	0.3325	0.0301	0.0692	No	1.0199	0.362	0.0006	0.0548	No	No
95444_at	Tmem49	transmembrane protein 49	0.9759	0.5619	0.1481	0.1941	No	0.9403	0.148	0.241	0.0205	No	No
95445_at	4833439L19 Rik	RIKEN cDNA 4833439L19 gene	0.9767	0.2556	0.0305	0.3545	No	0.9872	0.657	0.389	0.106	No	No
95446_at	6330577E15 Rik	RIKEN cDNA 6330577E15 gene	0.95	0.296	0.2239	0.5314	No	0.9737	0.482	0.433	0.261	No	No
95447_at	1810034K20 Rik	RIKEN cDNA 1810034K20 gene	1.0078	0.769	0.1268	0.0682	No	1.0568	0.402	0.0085	0.996	No	No
95448_at	Psmc2	proteasome (prosome, macropain) 26S subunit, ATPase 2	1.0211	0.7339	0.6472	0.9617	No	1.0605	0.259	0.119	0.868	No	No
95449_at	Mif4gd	MIF4G domain containing	1.0433	0.0054	0.0015	0.0149	Yes	0.9486	0.302	0.225	0.376	No	No
95450_at	Grb10	Growth factor receptor bound protein 10	1.04	0.7404	0.5327	0.5195	No	0.9596	0.625	0.222	0.918	No	No
95451_at	2810405J04 Rik	RIKEN cDNA 2810405J04 gene	0.9662	0.3499	0.1047	0.436	No	1.0039	0.923	0.0044	0.93	No	No
95452_i_at	S100a1	S100 calcium binding protein A1	0.959	0.721	0.4885	0.4307	No	0.9369	0.862	0.284	0.341	No	No
95453_f_at	S100a1	S100 calcium binding protein A1	1.0191	0.7886	0.003	0.4925	No	1.0433	0.518	0.0026	0.706	No	No
95454_at	S100a1	S100 calcium binding protein A1	0.9614	0.176	0.5	0.0863	No	0.8781	0.0633	0.222	0.0172	No	No
95455_at	Dnpep	aspartyl aminopeptidase	0.9923	0.7446	0.2286	0.7702	No	0.9584	0.665	0.0802	0.783	No	No
95456_r_at	Shfm1	split hand/foot malformation (ectrodactyly) type 1	0.9563	0.5839	0.1278	0.9223	No	0.9513	0.493	0.0735	0.781	No	No
95457_at	Impad1	inositol monophosphatase domain containing 1	0.981	0.827	0.0499	0.4401	No	0.9339	0.326	0.0197	0.423	No	No
95458_s_at	Impad1	inositol monophosphatase domain containing 1	0.9008	0.1425	0.0017	0.5967	No	0.9648	0.431	0.0016	0.958	No	No
95460_at	Cops5	COP9 (constitutive photomorphogenic) homolog, subunit 5 (Arabidopsis thaliana)	1.031	0.6517	0.1949	0.1539	No	0.9703	0.762	0.7	0.154	No	No
95462_at	Bzw2	basic leucine zipper and W2 domains 2	0.979	0.7868	0.3778	0.8452	No	0.9705	0.692	0.0415	0.896	No	No
95464_at	Tmem37	transmembrane protein 37	0.94	0.0523	0.0043	0.8964	No	0.9941	0.961	0.579	0.642	No	No
95465_s_at	Tmem37	transmembrane protein 37	0.9773	0.402	0.6313	0.3598	No	0.9637	0.417	0.802	0.229	No	No
95466_at	Cotl1	coactosin-like 1 (Dictyostelium)	0.8981	0.3657	0.2966	0.7153	No	0.9577	0.412	0.0131	0.105	No	No
95467_at	Scoc	short coiled-coil protein	1.1406	0.3201	0.0216	0.9067	No	1.0222	0.876	0.0395	0.64	No	No
95468_at	Egln1	EGL nine homolog 1 (C. elegans)	1.0085	0.8176	0.0122	0.8568	No	1.0437	0.212	0.0065	0.107	No	No
95469_at	Btd	biotinidase	0.9504	0.1906	0.0005	0.0256	No	0.9776	0.858	0.0015	0.104	No	No
95470_at	Pak1ip1	PAK1 interacting protein 1	0.9823	0.7479	0.1248	0.3441	No	1.0381	0.526	0.266	0.315	No	No
95471_at	Cdkn1c	cyclin-dependent kinase inhibitor 1C (P57)	1.0814	0.1106	0.0401	0.3274	No	0.9936	0.981	0.0077	0.276	No	No
95472_f_at	Uqcrb	ubiquinol-cytochrome c reductase binding protein	1.0668	0.2659	0.1007	0.041	No	1.064	0.351	0.0485	0.163	No	No
95473_s_at	Bud31	BUD31 homolog (yeast)	0.941	0.3794	0.0195	0.0957	No	0.9052	0.176	0.0009	0.0097	No	No
95474_at	F2r	coagulation factor II (thrombin) receptor	1.1032	0.103	0.0005	0.3475	No	1.0559	0.0595	0.0002	0.172	No	No
95477_at	Tmem59	transmembrane protein 59	1.1096	0.3893	0.3757	0.8246	No	1.0254	0.753	0.397	0.816	No	No
95478_at	Deb1	differentially expressed in B16F10 1	1.0012	0.9514	0.0262	0.014	No	1.0068	0.741	0.275	0.005	No	No
95479_at	C1d	nuclear DNA binding protein	1.0088	0.9437	0.3889	0.5918	No	1.1551	0.254	0.17	0.624	No	No
95480_at	Vps25	vacuolar protein sorting 25 (yeast)	1.021	0.5177	0.0564	0.1065	No	0.9928	0.938	0.0009	0.0561	No	No
95481_at	Usp7	ubiquitin specific peptidase 7	0.9413	0.3958	0.1132	0.4576	No	0.9817	0.73	0.129	0.139	No	No
95482_at	Usp7	ubiquitin specific peptidase 7	0.9721	0.3845	0.5054	0.0702	No	1.0337	0.535	0.642	0.862	No	No
95483_at	Psmd1	proteasome (prosome, macropain) 26S subunit, non-ATPase, 1	0.9821	0.6216	0.0306	0.5147	No	0.9661	0.484	0.0533	0.328	No	No
95485_at	Hadhsc	L-3-hydroxyacyl-Coenzyme A dehydrogenase, short chain	1.0554	0.2044	0.2319	0.8779	No	0.9713	0.55	0.0139	0.326	No	No
95486_at	Ptdsr	phosphatidylserine receptor	0.932	0.0954	0.7009	0.6158	No	0.982	0.717	0.745	0.829	No	No
95488_at	BC010304	cDNA sequence BC010304	0.92	0.2404	0.0218	0.8188	No	0.9345	0.263	0.0307	0.947	No	No
95489_at	Fliih	flightless I homolog (Drosophila)	0.9154	0.0585	0.0733	0.0197	No	0.9383	0.143	0.0796	0.0598	No	No
95490_at	Kdelr1	KDEL (Lys-Asp-Glu-Leu) endoplasmic reticulum protein retention receptor 1	0.9214	0.0911	0.0046	0.5914	No	0.9303	0.256	0.0051	0.442	No	No
95491_at	Park7	Parkinson disease (autosomal recessive, early onset) 7	1.1153	0.0382	0.1325	0.1564	No	1.0688	0.174	0.0597	0.348	No	No
95493_at	Col6a1	procollagen, type VI, alpha 1	0.917	0.0009	0	0.4917	Yes	0.9335	0.0371	0.0007	0.945	No	Yes
95496_at	Prpsap1	phosphoribosyl pyrophosphate synthetase-associated protein 1	0.965	0.2459	0.0054	0.2571	No	1.0067	0.745	0.0224	0.0896	No	No
95497_at	Tipin	timeless interacting protein	1.0187	0.7896	0.7728	0.8611	No	1.0641	0.364	0.0234	0.345	No	No
95498_at	Mrps15	mitochondrial ribosomal protein S15	0.9779	0.7838	0.0139	0.0396	No	0.9223	0.277	0.384	0.0494	No	No
95501_at	2410001C21 Rik	RIKEN cDNA 2410001C21 gene	0.9677	0.5223	0.6566	0.075	No	0.989	0.892	0.948	0.174	No	No
95502_at	Sirt2	sirtuin 2 (silent mating type information regulation 2, homolog) 2 (S. cerevisiae)	1.0129	0.7293	0.0004	0.4808	No	0.9774	0.354	0.0001	0.0821	No	No
95503_at	Mllt11	myeloid/lymphoid or mixed-lineage leukemia (trithorax homolog, Drosophila); translocated to, 11	0.9526	0.4002	0.0035	0.158	No	1.027	0.724	0.938	0.194	No	No
95505_at	Tor1b	torsin family 1, member B	0.9558	0.344	0.0448	0.4708	No	1.0234	0.622	0.0029	0.445	No	No
95506_at	Myo1c	myosin IC	0.9393	0.3612	0.0031	0.3205	No	1.018	0.662	0.442	0.764	No	No
95507_at	Prps1	phosphoribosyl pyrophosphate synthetase 1	0.9851	0.7931	0.0024	0.3985	No	1.0167	0.718	0.0062	0.9	No	No
95508_at	Nckap1	NCK-associated protein 1	1.0284	0.49	0.0274	0.3961	No	1.0464	0.386	0.158	0.842	No	No
95509_at	Sycn	syncollin	0.8972	0.1107	0.06	0.7594	No	0.9569	0.37	0.327	0.861	No	No
95511_at	Itga6	integrin alpha 6	1.0598	0.5397	0.0072	0.8042	No	0.9493	0.205	0.0014	0.282	No	No
95512_at	Pcmt1	protein-L-isoaspartate (D-aspartate) O-methyltransferase 1	1.0398	0.4706	0.0133	0.9331	No	0.9642	0.516	0.0512	0.789	No	No
95513_at	Statip1	signal transducer and activator of transcription interacting protein 1	0.9788	0.6736	0.8639	0.2856	No	1.0427	0.238	0.0593	0.0225	No	No
95514_at	Rcc2	regulator of chromosome condensation 2	1.0805	0.3649	0.2357	0.8472	No	0.9146	0.198	0.0514	0.255	No	No
95516_at	Rab9	RAB9, member RAS oncogene family	1.0305	0.7482	0.4199	0.2915	No	1.0664	0.541	0.097	0.413	No	No
95517_i_at	BC004004	cDNA sequence BC004004	0.9763	0.347	0	0.2277	No	0.9859	0.907	0.0009	0.397	No	No
95518_at	1810015C04 Rik	RIKEN cDNA 1810015C04 gene	0.8203	0.0226	0.0252	0.7221	No	0.8163	0.0018	0.0016	0.289	Yes	Yes
95520_at	Tmbim1	transmembrane BAX inhibitor motif containing 1	0.8605	0.0154	0.0001	0.0587	No	0.7899	0.009	0.0002	0.0054	No	No
95521_s_at	Zfp68	zinc finger protein 68	0.9766	0.6242	0.0284	0.3916	No	1.0327	0.961	0.002	0.0828	No	No
95522_i_at	Zfp68	zinc finger protein 68	0.8735	0.1829	0.1983	0.8019	No	0.9034	0.281	0.553	0.432	No	No
95523_at	2900062L11 Rik	RIKEN cDNA 2900062L11 gene	1.0068	0.7707	0.0009	0.1853	No	1.0638	0.0672	0.0015	0.792	No	No
95525_at	Ncoa6	nuclear receptor coactivator 6	1.0059	0.9171	0.0019	0.4067	No	0.94	0.261	0.857	0.131	No	No
95526_at	Xpo7	exportin 7	0.9125	0.0349	0.8197	0.4729	No	0.921	0.0223	0.0315	0.494	No	No
95527_at	Chaf1a	chromatin assembly factor 1, subunit A (p150)	1.1602	0.0058	0.0102	0.0085	No	1.0908	0.0899	0.0074	0.273	No	No

95528_at	AI507495	expressed sequence AI507495	1.0114	0.8699	0.0152	0.3986	No	1.0883	0.144	0.205	0.127	No	No
95529_at	Dbnl	drebrin-like	1.0203	0.4545	0.1297	0.4368	No	0.9901	0.723	0.0913	0.0146	No	No
95530_at	6330549H03 Rik	RIKEN cDNA 6330549H03 gene	0.901	0.2736	0.0502	0.4723	No	0.8851	0.115	0.0328	0.517	No	No
95531_at	Amot	angiomotin	0.9121	0.3285	0.0092	0.4891	No	0.9093	0.179	0.0035	0.957	No	No
95532_at	D13Ertd608 e	DNA segment, Chr 13, ERATO Doi 608, expressed	0.9554	0.3455	0.3517	0.4417	No	1.0183	0.742	0.679	0.417	No	No
95533_at	Zfp106	zinc finger protein 106	1.0377	0.6532	0.2099	0.8882	No	1.0347	0.551	0.0298	0.364	No	No
95536_at	Tceb3	transcription elongation factor B (SIII), polypeptide 3	0.9823	0.4297	0.0042	0.7468	No	1.0476	0.334	0.0287	0.587	No	No
95537_at	Ulk2	Unc-51 like kinase 2 (C. elegans)	0.9391	0.1756	0.0254	0.5183	No	1.0415	0.381	0.0514	0.247	No	No
95538_at	Arpc5l	actin related protein 2/3 complex, subunit 5-like	1.0193	0.7136	0.7987	0.51	No	0.9982	0.961	0.872	0.464	No	No
95539_at	Rab3ip	RAB3A interacting protein	0.9826	0.5961	0.0009	0.5766	No	1.0093	0.513	0.0002	0.138	No	No
95540_r_at	Ddx27	DEAD (Asp-Glu-Ala-Asp) box polypeptide 27	0.9653	0.0713	0.0143	0.0502	No	1.0489	0.0526	0.0011	0.0061	No	No
95541_at	D6Wsu176e	DNA segment, Chr 6, Wayne State University 176, expressed	1.0954	0.1165	0.0974	0.595	No	1.0646	0.171	0.0222	0.849	No	No
95542_at	Tpm4	tropomyosin 4	1.1286	0.0665	0.3337	0.4659	No	1.0899	0.0192	0.0046	0.44	No	No
95543_at	Tpm4	tropomyosin 4	1.0522	0.2799	0.4391	0.9301	No	1.0888	0.0663	0.0036	0.0279	No	No
95544_at	Rusc1	RUN and SH3 domain containing 1	1.0466	0.2492	0.8673	0.325	No	1.045	0.39	0.03	0.772	No	No
95545_at	Igf1	insulin-like growth factor 1	1.0194	0.7752	0.2732	0.6365	No	1.0368	0.421	0.27	0.107	No	No
95546_g_at	Igf1	insulin-like growth factor 1	0.925	0.1072	0.9014	0.0401	No	0.9773	0.649	0.403	0.389	No	No
95547_at	Rhod	ras homolog gene family, member D	0.914	0.1343	0.0104	0.7158	No	0.9671	0.478	0.0007	0.009	No	No
95549_at	Prim2	DNA primase, p58 subunit	1.0054	0.9371	0.0201	0.118	No	0.9619	0.234	0.0001	0.202	No	No
95550_at	Rpgr	retinitis pigmentosa GTPase regulator	1.0006	0.9891	0.0008	0.0674	No	1.0498	0.578	0.0005	0.147	No	No
95551_at	R3hcc1	R3H domain and coiled-coil containing 1	1.0449	0.0803	0.0009	0.0434	No	1.0053	0.722	0.008	0.0348	No	No
95552_at	Dio1	deiodinase, iodothyronine, type I	1.0065	0.8762	0.0284	0.7708	No	1.0536	0.622	0.264	0.295	No	No
95553_at	Ppat	phosphoribosyl pyrophosphate amidotransferase	0.9345	0.3207	0.8079	0.9114	No	1.0289	0.696	0.221	0.963	No	No
95554_at	Ppat	phosphoribosyl pyrophosphate amidotransferase	1.0133	0.7467	0.0166	0.0826	No	0.9913	0.933	0.859	0.456	No	No
95555_at	Unc119	unc-119 homolog (C. elegans)	0.9676	0.2811	0.0267	0.6649	No	0.9697	0.587	0.0571	0.106	No	No
95556_at	Mrpl45	mitochondrial ribosomal protein L45	0.9175	0.0142	0.0154	0.0186	No	0.8867	0.005	0.665	0.0265	Yes	Yes
95557_at	Bmp1	bone morphogenetic protein 1	0.9743	0.3998	0.0133	0.9429	No	0.952	0.343	0.0434	0.456	No	No
95559_at	6330403K07 Rik	RIKEN cDNA 6330403K07 gene	1.0487	0.3033	0.0435	0.7841	No	1.039	0.55	0.511	0.799	No	No
95561_at	Syce2	synaptonemal complex central element protein 2	1.0833	0.2545	0.6025	0.8215	No	1.0742	0.117	0.0083	0.264	No	No
95562_at	Ndp52	nuclear domain 10 protein 52	0.9163	0.0891	0.048	0.0793	No	1.0776	0.27	0.105	0.251	No	No
95563_at	Arih1	ariadne ubiquitin-conjugating enzyme E2 binding protein homolog 1 (Drosophila)	1.0056	0.9065	0.0422	0.6902	No	0.9785	0.77	0.132	0.929	No	No
95564_at	BC018601	cDNA sequence BC018601	0.9579	0.526	0.0675	0.2043	No	0.91	0.22	0.0411	0.181	No	No
95565_at	Mad2l1bp	MAD2L1 binding protein	0.9953	0.9231	0.0735	0.2289	No	0.9931	0.883	0.232	0.459	No	No
95566_at	Gpld1	glycosylphosphatidylinositol specific phospholipase D1	0.987	0.6575	0.138	0.0586	No	1.0158	0.528	0.0073	0.0595	No	No
95567_at	Sfrs2ip	splicing factor, arginine/serine-rich 2, interacting protein	0.9937	0.8646	0.0057	0.178	No	1.0918	0.0448	0.0094	0.0083	No	No
95568_at	1500011J06 Rik	RIKEN cDNA 1500011J06 gene	0.9968	0.9514	0.0004	0.2293	No	1.0246	0.84	0.0036	0.218	No	No
95569_at	Guca2b	guanylate cyclase activator 2b (retina)	1.0763	0.1399	0.0639	0.9597	No	1.0305	0.416	0.0017	0.63	No	No
95571_at	Slc30a4	solute carrier family 30 (zinc transporter), member 4	0.9782	0.6397	0.2567	0.3195	No	0.8748	0.0126	0.171	0.0358	No	No
95573_at	Baz2a	bromodomain adjacent to zinc finger domain, 2A	0.9824	0.4777	0.0006	0.8578	No	1.0706	0.19	0.0133	0.886	No	No
95574_f_at	Cggbp1	CGG triplet repeat binding protein 1	0.995	0.9734	0.0095	0.5686	No	0.9966	0.61	0.001	0.0173	No	No
95575_r_at	Cggbp1	CGG triplet repeat binding protein 1	0.9733	0.7787	0.2907	0.3047	No	0.9908	0.327	0.0001	0.0517	No	No
95577_at	AI314180	expressed sequence AI314180	0.9138	0.2184	0.0292	0.5148	No	0.8697	0.0426	0.0251	0.277	No	No
95580_at	---	---	0.983	0.7763	0.2979	0.6797	No	0.9442	0.259	0.39	0.375	No	No
95584_at	Dppa2	developmental pluripotency associated 2	0.9939	0.8377	0.0712	0.9239	No	1.0167	0.835	0.0317	0.996	No	No
95585_at	Proc	protein C	1.0168	0.6389	0.0457	0.9971	No	1.0752	0.0995	0.004	0.254	No	No
95586_at	P2rx4	purinergic receptor P2X, ligand-gated ion channel 4	0.965	0.4124	0.0453	0.0152	No	0.9936	0.943	0.948	0.0053	No	No
95587_at	Coq10a	coenzyme Q10 homolog A (yeast)	0.982	0.5097	0.2384	0.3555	No	1.0018	0.997	0.47	0.606	No	No
95588_at	Amacr	alpha-methylacyl-CoA racemase	0.9002	0.0206	0.0014	0.0405	No	0.9554	0.381	0.0016	0.0511	No	No
95590_at	Alg5	asparagine-linked glycosylation 5 homolog (yeast, dolichyl-phosphate beta-glucosyltransferase)	0.9809	0.6947	0.9933	0.3327	No	0.9839	0.649	0.179	0.469	No	No
95591_at	Extl3	exostoses (multiple)-like 3	0.9681	0.0487	0.0001	0.1604	No	1.1409	0.0049	0.001	0.0031	No	No
95592_at	1110019N10 Rik	RIKEN cDNA 1110019N10 gene	0.9522	0.3596	0.0446	0.2035	No	0.9178	0.035	0.017	0.0142	No	No
95593_at	Golph2	golgi phosphoprotein 2	1.0336	0.3732	0.8001	0.873	No	0.9656	0.55	0.73	0.239	No	No
95594_at	Mfn1	mitofusin 1	0.9286	0.022	0.0006	0.4259	No	0.9474	0.421	0.16	0.185	No	No
95595_at	Tanc1	tetratricopeptide repeat, ankyrin repeat and coiled-coil containing 1	0.954	0.533	0.2846	0.9857	No	0.9988	0.992	0.0024	0.877	No	No
95596_at	Foxk2	forkhead box K2	0.9647	0.3226	0.6876	0.5982	No	1.0422	0.579	0.0378	0.932	No	No
95597_at	Ptgs1	prostaglandin-endoperoxide synthase 1	1.0429	0.5692	0.3805	0.7121	No	1.0144	0.819	0.352	0.663	No	No
95599_at	St3gal4	ST3 beta-galactoside alpha-2,3-sialyltransferase 4	0.9487	0.3334	0.0427	0.8653	No	0.9433	0.0181	0.0026	0.0972	No	No
95600_at	Arih2	ariadne homolog 2 (Drosophila)	0.9438	0.0242	0.0039	0.0425	No	0.982	0.328	0.0016	0.899	No	No
95601_at	Ubqln1	ubiquilin 1	0.9965	0.9568	0.174	0.9916	No	1.0966	0.0967	0.433	0.618	No	No
95602_at	Trpc4ap	transient receptor potential cation channel, subfamily C, member 4 associated protein	0.9869	0.4504	0.0017	0.1338	No	0.9491	0.296	0.066	0.321	No	No
95603_at	Gldc	glycine decarboxylase	1.0568	0.1837	0.0785	0.9239	No	1.0512	0.536	0.361	0.498	No	No
95604_at	C330006A16 Rik	RIKEN cDNA C330006A16 gene	0.9634	0.4077	0.2324	0.7382	No	1.0241	0.342	0.0058	0.0157	No	No
95606_at	Syncrip	synaptotagmin binding, cytoplasmic RNA interacting protein	1.054	0.1792	0.0118	0.8407	No	1.0806	0.169	0.0732	0.218	No	No
95607_at	Stard3	START domain containing 3	1.0248	0.4237	0.013	0.1706	No	1.0917	0.127	0.978	0.0531	No	No
95608_at	Ctsb	cathepsin B	0.9121	0.1003	0.0191	0.4714	No	0.9607	0.0352	0.0439	0.181	No	No
95609_at	---	---	0.9863	0.7576	0.467	0.1241	No	0.9761	0.409	0.0002	0.002	No	No
95610_at	Cdc5l	cell division cycle 5-like (S. pombe)	0.9345	0.241	0.5373	0.8642	No	0.9654	0.324	0.0492	0.0456	No	No
95611_at	Lpl	lipoprotein lipase	0.9017	0.332	0.0113	0.3818	No	0.9737	0.706	0.106	0.871	No	No
95612_at	Rfc5	replication factor C (activator 1) 5	1.0502	0.1421	0.0089	0.0492	No	1.0331	0.614	0.0126	0.886	No	No
95613_at	Tmem30a	transmembrane protein 30A	1.0577	0.5065	0.2336	0.8469	No	1.0198	0.721	0.698	0.824	No	No
95614_at	Cbx5	chromobox homolog 5 (Drosophila HP1a)	1.0645	0.3931	0.1161	0.0755	No	1.0613	0.368	0.0527	0.0438	No	No
95616_at	Crsp3	cofactor required for Sp1 transcriptional activation, subunit 3	1.0868	0.3284	0.0862	0.9281	No	1.0266	0.172	0.0002	0.0011	No	No
95617_at	Rbl2 /// LOC635075	retinoblastoma-like 2 /// similar to retinoblastoma-like 2	0.8552	0.0094	0.025	0.2738	Yes	0.8082	0.0075	0.165	0.733	Yes	Yes
95618_at	Rab11fip5	RAB11 family interacting protein 5 (class I)	1.0284	0.3727	0.0259	0.8246	No	1.0088	0.831	0.216	0.857	No	No
95619_at	1700040I03 Rik	RIKEN cDNA 1700040I03 gene	1.0456	0.4248	0.0099	0.0671	No	1.0467	0.535	0.0055	0.86	No	No
95620_at	Dhrs7	dehydrogenase/reductase (SDR family) member 7	0.8458	0.0072	0.377	0.0334	Yes	0.8905	0.0015	0.255	0.0059	No	No
95621_at	9030623C06 Rik	RIKEN cDNA 9030623C06 gene	0.8987	0.0238	0.0171	0.0891	No	0.8921	0.0842	0.0011	0.696	No	No
95622_at	Klhdc2	kelch domain containing 2	1.0974	0.1311	0.0726	0.2375	No	1.1313	0.252	0.676	0.628	No	No
95625_at	Slc6a8	solute carrier family 6 (neurotransmitter transporter, creatine), member 8	0.9934	0.8769	0.102	0.1886	No	0.9904	0.917	0.0158	0.0329	No	No
95626_at	Xpc	xeroderma pigmentosum, complementation group C	0.9381	0.19	0.0314	0.1784	No	0.9094	0.0101	0.0802	0.0469	No	No
95627_at	Wwtr1	WW domain containing transcription regulator 1	1.1603	0.0016	0	0.0012	No	1.179	0.039	0	0.017	No	No
95628_at	Diap3	diaphanous homolog 3 (Drosophila)	1.0347	0.1389	0.0108	0.0874	No	1.0219	0.436	0.0099	0.209	No	No
95629_at	Trappc1	trafficking protein particle complex 1	1.0999	0.0385	0.4105	0.6624	No	1.0615	0.116	0.842	0.797	No	No
95630_at	Sars2	seryl-aminoacyl-tRNA synthetase 2	1.0209	0.6408	0.2666	0.7963	No	1.0648	0.0797	0.0055	0.0215	No	No
95631_at	Ppp4c	protein phosphatase 4, catalytic subunit	1.0816	0.0428	0.0045	0.2275	No	1.0469	0.0965	0.0042	0.0541	No	No
95632_f_at	Mvk /// LOC637711	mevalonate kinase /// similar to mevalonate kinase	1.0001	0.9982	0.0339	0.0386	No	0.9056	0.081	0.437	0.0207	No	No
95633_r_at	Mvk	mevalonate kinase	1.0004	0.9847	0.0019	0.1381	No	0.8778	0.0484	0.003	0.468	No	No
95634_at	0610010K14 Rik	RIKEN cDNA 0610010K14 gene	1.0318	0.5006	0.1445	0.4884	No	1.1616	0.0317	0.0628	0.409	No	No
95635_g_at	0610010K14 Rik	RIKEN cDNA 0610010K14 gene	1.0039	0.8763	0.3431	0.3043	No	1.0688	0.214	0.426	0.727	No	No
95636_at	0610010K14 Rik	RIKEN cDNA 0610010K14 gene	0.9783	0.7942	0.0526	0.9512	No	0.9677	0.503	0.002	1	No	No
95637_at	Flnb	filamin, beta	0.9903	0.828	0.9422	0.2549	No	1.0628	0.094	0.024	0.0034	No	No
95639_at	Chrng	cholinergic receptor, nicotinic, gamma polypeptide	1.0206	0.7488	0.1998	0.7002	No	0.9977	0.981	0.0157	0.323	No	No
95641_at	Plac9	placenta specific 9	0.888	0.0229	0.0223	0.0248	No	0.9614	0.583	0.404	0.283	No	No
95642_at	Elovl1	elongation of very long chain fatty acids (FEN1/Elo2, SUR4/Elo3, yeast)-like 1	1.0132	0.7166	0.1153	0.2315	No	0.9715	0.587	0.0235	0.383	No	No
95643_at	Wdr6	WD repeat domain 6	1.0633	0.0418	0.0044	0.2401	No	1.1124	0.0752	0.0089	0.0493	No	No
95645_at	---	PREDICTED: Mus musculus similar to nitrogen fixation cluster-like (LOC433217), mRNA	1.0283	0.4628	0.0423	0.2474	No	1.0717	0.129	0.0195	0.139	No	No

95646_at	Cpt2	carnitine palmitoyltransferase 2	0.9752	0.6391	0.8422	0.1742	No	0.9541	0.398	0.86	0.0387	No	No
95647_f_at	Mobk1b	MOB1, Mps One Binder kinase activator-like 1B (yeast)	1.0817	0.2382	0.0051	0.1088	No	1.0628	0.328	0.0332	0.177	No	No
95648_at	---	---	1.0563	0.0165	0.0077	0.5191	No	0.9404	0.277	0.402	0.555	No	No
95649_at	Phf5a	PHD finger protein 5A	1.0297	0.3362	0.004	0.0521	No	1.0711	0.0691	0.0003	0.132	No	No
95650_at	Nhp2l1 /// LOC434401 /// LOC637555 /// LOC667636 /// LOC672658	NHP2 non-histone chromosome protein 2-like 1 (S. cerevisiae) /// similar to Nhp2 non-histone chromosome protein 2-like 1 /// similar to NHP2-like protein 1 (High mobility group-like nuclear protein 2 homolog 1) ([U4/U6.U5] tri-snRNP 15.5 kDa protein) (Sperm specific antigen 1) (Fertilization antigen 1) (FA-1) /// similar to Nhp2 non-histone chromosome protein 2-like 1 /// similar to Nhp2 non-histone chromosome protein 2-like 1	0.943	0.3023	0.5243	0.7776	No	0.9367	0.188	0.127	0.995	No	No
95651_at	Myg1	melanocyte proliferating gene 1	1.0843	0.1132	0.6069	0.8964	No	1.0962	0.473	0.507	0.978	No	No
95652_at	Ndufa7	NADH dehydrogenase (ubiquinone) 1 alpha subcomplex, 7 (B14.5a)	0.9618	0.2498	0.0027	0.0074	No	0.922	0.131	0.0327	0.0366	No	No
95653_at	Mrpl37	mitochondrial ribosomal protein L37	1.0038	0.9256	0.0366	0.2001	No	0.9768	0.769	0.0467	0.096	No	No
95654_at	Clic1	chloride intracellular channel 1	1.1104	0.1202	0.2986	0.8795	No	1.1246	0.0397	0.263	0.382	No	No
95655_at	5830411E10Rik	RIKEN cDNA 5830411E10 gene	0.9914	0.815	0.0078	0.0846	No	1.2441	0.143	0.0417	0.994	No	No
95656_i_at	D13Wsu177e	DNA segment, Chr 13, Wayne State University 177, expressed	0.9789	0.6601	0.0546	0.2551	No	0.9998	0.939	0.0284	0.214	No	No
95657_f_at	D13Wsu177e	DNA segment, Chr 13, Wayne State University 177, expressed	1.0972	0.171	0.0025	0.0847	No	1.1063	0.237	0.0059	0.157	No	No
95658_at	Commd1	COMM domain containing 1	1.1044	0.3528	0.0098	0.1371	No	0.9276	0.421	0.0002	0.295	No	No
95659_at	Actl6a	actin-like 6A	1.1001	0.4022	0.0431	0.8968	No	1.1261	0.263	0.131	0.94	No	No
95660_at	Ethe1	ethylmalonic encephalopathy 1	0.9692	0.4108	0.2414	0.8335	No	0.9945	0.845	0.373	0.282	No	No
95661_at	Cd9	CD9 antigen	1.1249	0.0992	0.0933	0.5811	No	1.0166	0.851	0.753	0.39	No	No
95662_at	X83328	EST X83328	0.9973	0.9552	0.1274	0.0748	No	0.9632	0.475	0.14	0.126	No	No
95663_at	Mmp2	matrix metallopeptidase 2	1.008	0.9219	0.8493	0.7773	No	1.0718	0.599	0.783	0.446	No	No
95664_at	---	---	0.9331	0.1932	0.6207	0.7486	No	0.9333	0.0724	0.633	0.0233	No	No
95665_at	Sec14l1	SEC14-like 1 (S. cerevisiae)	0.9291	0.0327	0.0006	0.1953	No	0.9128	0.0076	0.0001	0.181	Yes	Yes
95666_at	Cops8	COP9 (constitutive photomorphogenic) homolog, subunit 8 (Arabidopsis thaliana)	0.9881	0.7614	0.0202	0.3474	No	1.0212	0.584	0.405	0.164	No	No
95668_at	Stmn2	Stathmin-like 2	1.0326	0.6494	0.0025	0.2082	No	0.9996	0.139	0.0003	0.0036	No	No
95669_g_at	Stmn2	stathmin-like 2	1.0084	0.8823	0.0134	0.7705	No	1.041	0.131	0.0027	0.408	No	No
95670_at	Stmn2	stathmin-like 2	1.1486	0.1327	0.0009	0.141	No	1.0671	0.151	0.0009	0.104	No	No
95671_at	Hey1	hairy/enhancer-of-split related with YRPW motif 1	1.0185	0.528	0.1409	0.8042	No	1.0706	0.0209	0.0095	0.0363	No	No
95672_at	Psmc4	proteasome (prosome, macropain) 26S subunit, ATPase, 4	1.0585	0.4654	0.0461	0.7979	No	1.1258	0.248	0.0229	0.255	No	No
95673_s_at	Basp1	brain abundant, membrane attached signal protein 1	1.056	0.3	0.0362	0.0332	No	1.0664	0.142	0.281	0.0386	No	No
95674_r_at	Basp1	brain abundant, membrane attached signal protein 1	1.029	0.1192	0.0145	0.0096	No	0.984	0.218	0.0129	0.193	No	No
95675_at	Map4k3 /// LOC675560	mitogen-activated protein kinase kinase kinase kinase 3 /// similar to mitogen-activated protein kinase kinase kinase kinase 3	1.0079	0.6693	0.0181	0.6303	No	1.0478	0.227	0.0047	0.801	No	No
95676_at	Idh3b	isocitrate dehydrogenase 3 (NAD+) beta	1.0696	0.4159	0.3718	0.8455	No	1.1075	0.161	0.0152	0.292	No	No
95677_at	Wdr57	WD repeat domain 57 (U5 snRNP specific)	1.0823	0.2395	0.1246	0.8827	No	1.0776	0.338	0.0427	0.235	No	No
95679_at	Cdk2ap2	CDK2-associated protein 2	0.9537	0.404	0.2026	0.2029	No	0.979	0.687	0.0877	0.826	No	No
95680_at	Ppp1r2	protein phosphatase 1, regulatory (inhibitor) subunit 2	0.9791	0.5359	0.0251	0.0229	No	0.9923	0.917	0.819	0.765	No	No
95681_f_at	---	---	1.0505	0.1917	0.0006	0.18	No	1.0659	0.241	0.0035	0.35	No	No
95682_at	Ddb1	damage specific DNA binding protein 1	0.898	0.0024	0.2014	0.0023	No	0.8987	0.0058	0.961	0.0255	Yes	No
95683_g_at	Ddb1	damage specific DNA binding protein 1	0.9613	0.3397	0.0132	0.4986	No	0.976	0.538	0.0037	0.862	No	No
95684_at	Ddb1	damage specific DNA binding protein 1	1.0451	0.3944	0.4888	0.8735	No	1.0952	0.0156	0.0218	0.0078	No	No
95685_at	Wdr74	WD repeat domain 74	1.0103	0.8096	0.0283	0.7393	No	0.9334	0.283	0.202	0.285	No	No
95686_at	Rab14	RAB14, member RAS oncogene family	1.0835	0.0832	0.4362	0.5074	No	1.1325	0.154	0.239	0.302	No	No
95688_at	Degs1	degenerative spermatocyte homolog 1 (Drosophila)	1.012	0.852	0.0245	0.6228	No	1.0621	0.401	0.0729	0.523	No	No
95689_at	Mtch1	mitochondrial carrier homolog 1 (C. elegans)	1.0745	0.029	0.2006	0.1339	No	0.9946	0.453	0	0.0002	No	No
95690_at	Samm50	sorting and assembly machinery component 50 homolog (S. cerevisiae)	0.9784	0.5395	0.0299	0.19	No	0.9951	0.927	0.0429	0.85	No	No
95692_at	Srp68	signal recognition particle 68	0.9555	0.227	0.3646	0.1266	No	0.9658	0.459	0.0297	0.307	No	No
95693_at	Idh2	isocitrate dehydrogenase 2 (NADP+), mitochondrial	0.8902	0.0153	0.0006	0.0256	No	0.9164	0.0448	0.0002	0.0195	No	No
95694_at	Top1	topoisomerase (DNA) I	0.9544	0.3312	0.0192	0.6794	No	0.889	0.0298	0.0191	0.182	No	No
95695_at	Slc25a20	solute carrier family 25 (mitochondrial carnitine/acylcarnitine translocase), member 20	1.0213	0.6601	0.0989	0.1257	No	0.9806	0.744	0.432	0.289	No	No
95696_at	Txnl2 /// LOC620016	thioredoxin-like 2 /// similar to thioredoxin-like 2	1.0535	0.1819	0.6965	0.0088	No	1.1412	0.0343	0.0009	0.895	No	No
95697_at	2610009E16Rik /// Eif5a /// LOC640461 /// LOC665733	RIKEN cDNA 2610009E16 gene /// eukaryotic translation initiation factor 5A /// similar to eukaryotic translation initiation factor 5A /// similar to eukaryotic translation initiation factor 5A	1.0518	0.3421	0.5233	0.4018	No	1.0316	0.446	0.818	0.0753	No	No
95698_at	Ndufb7	NADH dehydrogenase (ubiquinone) 1 beta subcomplex, 7	0.9632	0.4852	0.4573	0.3587	No	1.0342	0.397	0.93	0.192	No	No
95699_f_at	Dnajc8 /// LOC669266	DnaJ (Hsp40) homolog, subfamily C, member 8 /// similar to DnaJ (Hsp40) homolog, subfamily C, member 8 (predicted)	1.0599	0.1764	0.9401	0.1394	No	1.0716	0.0985	0.544	0.102	No	No
95700_r_at	Dnajc8 /// LOC669266	DnaJ (Hsp40) homolog, subfamily C, member 8 /// similar to DnaJ (Hsp40) homolog, subfamily C, member 8 (predicted)	0.9988	0.9392	0.0016	0.1522	No	0.9771	0.275	0.928	0.262	No	No
95701_at	Cxxc5	CXXC finger 5	1.1862	0.0146	0.0791	0.1842	No	1.1319	0.0282	0.0257	0.445	No	No
95702_at	Stt3b	STT3, subunit of the oligosaccharyltransferase complex, homolog B (S. cerevisiae)	1.0215	0.5758	0.8334	0.9555	No	0.9736	0.676	0.808	0.0619	No	No
95703_at	Uble1a	ubiquitin-like 1 (sentrin) activating enzyme E1A	1.1004	0.0512	0.0753	0.621	No	1.0695	0.0868	0.0702	0.532	No	No
95704_at	Ap1b1	adaptor protein complex AP-1, beta 1 subunit	1.0151	0.6389	0.8671	0.1	No	1.0141	0.706	0.0747	0.185	No	No
95705_s_at	Actb	actin, beta, cytoplasmic	0.7898	0.7053	0.7468	0.8477	No	0.6924	0.44	0.85	0.726	No	No
95706_at	Lgals3	lectin, galactose binding, soluble 3	1.0042	0.9452	0.0077	0.2974	No	0.9512	0.968	0.0002	0.0117	No	No
95707_at	2900010M23Rik	RIKEN cDNA 2900010M23 gene	1.0403	0.182	0.0008	0.0021	No	1.0798	0.0357	0.0015	0.0209	No	No
95708_at	D3Ucla1	DNA segment, Chr 3, University of California at Los Angeles 1	1.0521	0.0832	0.0035	0.1041	No	1.0488	0.019	0.0238	0.515	No	No
95709_at	Vkorc1	vitamin K epoxide reductase complex, subunit 1	1.036	0.3393	0.001	0.823	No	1.0125	0.503	0.0011	0.0903	No	No
95712_at	Orc6l	origin recognition complex, subunit 6-like (S. cerevisiae)	1.0416	0.1107	0.3091	0.6965	No	1.0542	0.0951	0.151	0.202	No	No
95713_at	Clptm1	cleft lip and palate associated transmembrane protein 1	1.0055	0.9149	0.0153	0.6094	No	0.9772	0.703	0.216	0.0729	No	No
95714_at	0610009D07Rik	RIKEN cDNA 0610009D07 gene	1.1329	0.1372	0.3367	0.8367	No	1.0974	0.156	0.416	0.786	No	No
95715_at	Eif3s12	eukaryotic translation initiation factor 3, subunit 12	0.9951	0.8504	0.0005	0.044	No	0.9663	0.643	0.0011	0.0565	No	No
95716_at	Ywhag	3-monooxygenase/tryptophan 5-monooxygenase activation protein, gamma polypeptide	1.07	0.3003	0.0112	0.8024	No	0.9994	0.866	0	0.335	No	No
95717_at	Elp3	elongation protein 3 homolog (S. cerevisiae)	0.9099	0.01	0.0007	0.0619	No	0.9121	0.0072	0.0002	0.0322	Yes	Yes
95718_f_at	Usmg5	upregulated during skeletal muscle growth 5	1.078	0.1063	0.0028	0.7836	No	1.0223	0.257	0	0.946	No	No
95719_at	Bccip	BRCA2 and CDKN1A interacting protein	1.1161	0.0221	0.169	0.0122	No	1.0651	0.0786	0.177	0.0204	No	No
95721_at	Mapkapk2	MAP kinase-activated protein kinase 2	1.0384	0.413	0.0012	0.4808	No	1.1194	0.0254	0.0001	0.165	No	No
95722_at	Glrx	glutaredoxin	0.9538	0.0859	0.6812	0.0785	No	1.0022	0.979	0.0014	0.0561	No	No
95723_r_at	2610009E16Rik	RIKEN cDNA 2610009E16 gene	1.0583	0.1459	0.0003	0.9762	No	1.0573	0.0715	0.0001	0.797	No	No
95725_at	Acy3	aspartoacylase (aminoacylase) 3	0.9618	0.2234	0.2076	0.1351	No	0.962	0.044	0.0006	0.0129	No	No
95726_at	Mlf2	myeloid leukemia factor 2	1.0428	0.2192	0.002	0.2053	No	0.9981	0.954	0.0002	0.25	No	No
95727_at	Apoa5	apolipoprotein A-V	0.9561	0.3545	0.3477	0.3217	No	1.0876	0.175	0.0502	0.0309	No	No
95728_g_at	Apoa5	apolipoprotein A-V	0.9681	0.3107	0.0404	0.0774	No	0.9639	0.663	0.0101	0.109	No	No
95729_at	Apoa5	apolipoprotein A-V	1.0576	0.5614	0.285	0.5491	No	1.1324	0.0767	0.026	0.822	No	No
95730_at	Mrps34	mitochondrial ribosomal protein S34	0.9833	0.758	0.9273	0.0934	No	1.0745	0.185	0.975	0.0279	No	No
95731_at	Sesn1	sestrin 1	0.6261	0	0.0148	0.1566	Yes	0.6716	0.0023	0.03	0.471	Yes	Yes
95732_at	Sf3b5	splicing factor 3b, subunit 5	1.0059	0.9324	0.5621	0.0731	No	1.0232	0.698	0.13	0.167	No	No
95733_at	Slc29a1	solute carrier family 29 (nucleoside transporters), member 1	0.9681	0.1674	0.6898	0.9571	No	0.9972	0.877	0.0334	0.304	No	No
95734_at	Mrpl3	mitochondrial ribosomal protein L3	0.9606	0.5142	0.1919	0.7333	No	0.9658	0.374	0.354	0.256	No	No

95735_at	Nolc1	nucleolar and coiled-body phosphoprotein 1	0.9836	0.7608	0.4607	0.6911	No	1.0356	0.13	0.756	0.0026	No	No
95736_at	Mrpl4	mitochondrial ribosomal protein L4	1.0201	0.6644	0.2745	0.3621	No	1.0373	0.461	0.141	0.386	No	No
95737_at	1200015A19 Rik	RIKEN cDNA 1200015A19 gene	1.0179	0.8869	0.3101	0.6871	No	0.8993	0.136	0.0093	0.0271	No	No
95738_at	Aldh18a1	aldehyde dehydrogenase 18 family, member A1	1.1543	0.1212	0.0061	0.425	No	1.1003	0.115	0.0005	0.171	No	No
95739_at	Limd2	LIM domain containing 2	1.0769	0.1211	0.4298	0.3458	No	1.1902	0.271	0.947	0.268	No	No
95740_at	2300003P22 Rik	RIKEN cDNA 2300003P22 gene	0.9644	0.586	0.3938	0.7445	No	1.0655	0.236	0.0514	0.537	No	No
95742_at	Psmd13	proteasome (prosome, macropain) 26S subunit, non-ATPase, 13	0.9984	0.9708	0.0213	0.0388	No	0.96	0.568	0.0004	0.0116	No	No
95743_at	Paip2	polyadenylate-binding protein-interacting protein 2	1.1315	0.1301	0.4943	0.3076	No	1.0062	0.915	0.0284	0.792	No	No
95744_at	Atp6v1a	ATPase, H+ transporting, lysosomal V1 subunit A	0.9927	0.8825	0.0617	0.5763	No	0.9355	0.0701	0.0113	0.11	No	No
95745_g_at	Atp6v1a	ATPase, H+ transporting, lysosomal V1 subunit A	1.0497	0.1622	0.0029	0.2337	No	1.0463	0.217	0.0079	0.429	No	No
95746_at	---	---	1.1063	0.1355	0.6575	0.447	No	1.0736	0.151	0.294	0.286	No	No
95747_at	S100a16	S100 calcium binding protein A16	0.8975	0.2364	0.0553	0.1741	No	0.7859	0.364	0.0371	0.198	No	No
95749_at	Armet	arginine-rich, mutated in early stage tumors	1.1342	0.0442	0.588	0.9815	No	1.1071	0.0605	0.162	0.556	No	No
95750_at	Zc3hc1	zinc finger, C3HC type 1	0.9976	0.9416	0.0381	0.6786	No	1.1078	0.0022	0.0004	0.363	Yes	No
95752_at	Sbds	Shwachman-Bodian-Diamond syndrome homolog (human)	0.9568	0.125	0.0311	0.0243	No	0.9491	0.0308	0.0831	0.0076	No	No
95753_at	Brrn1	barren homolog (Drosophila)	1.0115	0.8044	0.0971	0.0936	No	1.0086	0.947	0.467	0.311	No	No
95754_at	Mbtps1	membrane-bound transcription factor peptidase, site 1	0.9621	0.1184	0.0002	0.0261	No	1.0111	0.823	0.0069	0.929	No	No
95755_at	Csda	cold shock domain protein A	1.0442	0.4902	0.3595	0.8896	No	0.9423	0.212	0.012	0.0407	No	No
95756_at	Ftsj3	FtsJ homolog 3 (E. coli)	0.9023	0.0422	0.0003	0.0227	No	0.9609	0.166	0.0005	0.0304	No	No
95758_at	Scd2	stearoyl-Coenzyme A desaturase 2	1.0409	0.2886	0.0248	0.1957	No	0.9944	0.718	0.0061	0.705	No	No
95759_at	2900092E17 Rik	RIKEN cDNA 2900092E17 gene	0.9993	0.9864	0.6152	0.2164	No	0.9891	0.697	0.358	0.184	No	No
95760_at	Elof1	elongation factor 1 homolog (ELF1, S. cerevisiae)	0.9923	0.8844	0.0055	0.0512	No	0.981	0.844	0.0191	0.153	No	No
95765_at	LOC635419	similar to alcohol dehydrogenase 5 (class III), chi polypeptide	0.9399	0.0046	0.0006	0.0042	No	0.9402	0.153	0.105	0.328	No	No
95766_f_at	Defcr2 /// Defcr3 /// Defcr17 /// Defcr23	defensin related cryptdin 2 /// defensin related cryptdin 3 /// defensin related cryptdin 17 /// defensin-related cryptdin 23	0.9569	0.6291	0.2767	0.6453	No	0.9567	0.812	0.871	0.704	No	No
95770_s_at	Fprl1	formyl peptide receptor-like 1	0.9775	0.7771	0.0665	0.6068	No	0.9707	0.294	0.0006	0.154	No	No
95771_i_at	Fzd4	frizzled homolog 4 (Drosophila)	0.9677	0.5935	0.4174	0.1967	No	1.0427	0.433	0.154	0.297	No	No
95772_r_at	Fzd4	frizzled homolog 4 (Drosophila)	1.0335	0.6524	0.3516	0.3227	No	1.0416	0.557	0.113	0.24	No	No
95773_at	H2-D1 /// H2-L /// H2-Q1 /// H2-Q7 /// H2-Q8 /// H2-Q6 /// LOC547343 /// LOC630509 /// LOC634869 /// LOC674192 /// LOC676689 /// LOC676708 /// LOC677617	histocompatibility 2, D region locus 1 /// histocompatibility 2, D region /// histocompatibility 2, Q region locus 1 /// histocompatibility 2, Q region locus 7 /// histocompatibility 2, Q region locus 8 /// histocompatibility 2, Q region locus 6 /// similar to H-2 class I histocompatibility antigen, L-D alpha chain precursor /// similar to H-2 class I histocompatibility antigen, Q7 alpha chain precursor (QA-2 antigen) /// MHC c6/g2 (Qa-2) protein /// region containing histocompatibility 2, Q region locus 9; histocompatibility 2, Q region locus 7 /// similar to H-2 class I histocompatibility antigen, L-D alpha chain precursor /// similar to H-2 class I histocompatibility antigen, L-D alpha chain precursor /// similar to histocompatibility 2, Q region locus 5	0.9835	0.5967	0.308	0.1333	No	1.0141	0.592	0.283	0.0118	No	No
95775_f_at	Klk1b1	kallikrein 1-related peptidase b1	0.9454	0.3864	0.9317	0.9066	No	0.9914	0.802	0.0099	0.45	No	No
95781_at	Mageb1 /// Mageb2 /// Mageb3	melanoma antigen, family B, 1 /// melanoma antigen, family B, 2 /// melanoma antigen, family B, 3	1.0736	0.4813	0.2945	0.2181	No	1.0251	0.279	0.0025	0.0085	No	No
95782_at	Mageb3	melanoma antigen, family B, 3	0.9291	0.1471	0.0682	0.5492	No	0.9831	0.742	0.138	0.882	No	No
95783_g_at	Mageb1 /// Mageb2 /// Mageb3	melanoma antigen, family B, 1 /// melanoma antigen, family B, 2 /// melanoma antigen, family B, 3	0.9664	0.6938	0.4744	0.9315	No	0.9167	0.311	0.0967	0.59	No	No
95784_at	Pira1	paired-Ig-like receptor A1	0.9847	0.5423	0.4405	0.6437	No	0.9727	0.628	0.262	0.477	No	No
95785_s_at	Rab7	RAB7, member RAS oncogene family	0.9968	0.956	0.1428	0.6415	No	0.9571	0.428	0.0171	0.0988	No	No
95786_at	Reg2	regenerating islet-derived 2	0.9776	0.8154	0.5119	0.8005	No	1.0273	0.629	0.407	0.726	No	No
95787_s_at	Scp2	sterol carrier protein 2, liver	0.9865	0.8055	0.2441	0.2598	No	0.9824	0.638	0.0005	0.0038	No	No
95791_s_at	Sfrs2	splicing factor, arginine/serine-rich 2 (SC-35)	1.1841	0.0077	0.4403	0.1617	Yes	1.1535	0.0023	0.171	0.0774	Yes	Yes
95792_at	Zfp106	zinc finger protein 106	1.0126	0.6563	0.9762	0.5614	No	1.1536	0.0776	0.186	0.293	No	No
95793_at	Sprr2d	small proline-rich protein 2D	0.9409	0.2412	0.2862	0.9415	No	0.9925	0.907	0.785	0.593	No	No
95794_f_at	Sprr2i	small proline-rich protein 2I	1.0909	0.0644	0.0361	0.0858	No	1.0571	0.283	0.0442	0.0464	No	No
95795_at	Supt4h2	suppressor of Ty 4 homolog 2 (S. cerevisiae)	1.0457	0.3706	0.2708	0.4731	No	1.0685	0.26	0.0587	0.219	No	No
95796_g_at	Supt4h1 /// Supt4h2	suppressor of Ty 4 homolog 1 (S. cerevisiae) /// suppressor of Ty 4 homolog 2 (S. cerevisiae)	0.9364	0.0651	0.6978	0.7617	No	0.9259	0.102	0.0175	0.61	No	No
95797_f_at	V2r1	vomeronasal 2, receptor, 1	1.0409	0.5534	0.6919	0.1421	No	1.1076	0.151	0.651	0.473	No	No
95798_f_at	V2r1 /// V2r12 /// V2r2 /// V2r8 /// V2r9	vomeronasal 2, receptor, 1 /// vomeronasal 2, receptor, 12 /// vomeronasal 2, receptor, 2 /// vomeronasal 2, receptor, 8 /// vomeronasal 2, receptor, 9	1.0227	0.836	0.3658	0.8186	No	1.0505	0.601	0.996	0.36	No	No
95799_s_at	V2r4 /// V2r5 /// LOC625605	vomeronasal 2, receptor, 4 /// vomeronasal 2, receptor, 5 /// similar to vomeronasal 2, receptor, 4	0.9039	0.3801	0.1319	0.2931	No	0.9455	0.527	0.876	0.477	No	No
95800_s_at	Zfa /// Zfx	zinc finger protein, autosomal /// zinc finger protein X-linked	0.9655	0.608	0.4095	0.2654	No	0.9933	0.904	0.293	0.754	No	No
95801_s_at	Zfp260 /// LOC635007	zinc finger protein 260 /// similar to Zinc finger protein OZF (Zinc finger protein 260) (Zfp-260)	0.9021	0.1755	0.7186	0.5297	No	0.9172	0.157	0.415	0.245	No	No
95803_at	Sirpa	signal-regulatory protein alpha	0.9647	0.7338	0.2451	0.5906	No	0.9816	0.749	0.9	0.368	No	No
95804_g_at	Sirpa	signal-regulatory protein alpha	1.0009	0.9767	0.3094	0.7745	No	1.0302	0.285	0.0455	0.326	No	No
95805_at	Cdc2l1	cell division cycle 2-like 1	0.9207	0.0179	0.0093	0.1775	No	1.0333	0.111	0.0076	0.0686	No	No
95806_f_at	Cma2	chymase 2, mast cell	1.0939	0.2435	0.3117	0.5298	No	1.0805	0.314	0.247	0.443	No	No
95807_at	Csf3r /// LOC433259	colony stimulating factor 3 receptor (granulocyte) /// similar to colony stimulating factor 3 receptor	1.0147	0.1477	0.0001	0.002	No	1.0387	0.234	0.291	0.12	No	No
95808_g_at	Csf3r /// LOC433259	colony stimulating factor 3 receptor (granulocyte) /// similar to colony stimulating factor 3 receptor	1.0332	0.5647	0.025	0.0593	No	1.0687	0.251	0.0036	0.173	No	No
95848_at	LOC667653 /// LOC672953	hypothetical protein LOC667653 /// hypothetical protein LOC672953	0.9476	0.3897	0.0748	0.1027	No	0.9599	0.579	0.0454	0.0396	No	No
95852_at	---	Transcribed locus	1.0406	0.5715	0.9388	0.6057	No	1.0381	0.6	0.101	0.0799	No	No
95853_at	---	---	0.9441	0.4095	0.0367	0.9718	No	0.8751	0.619	0.0384	0.198	No	No
95854_at	C76332	expressed sequence C76332	0.9842	0.8081	0.0947	0.9462	No	0.9906	0.931	0.0087	0.874	No	No
95855_at	Mipol1	mirror-image polydactyly gene 1 homolog (human)	0.9769	0.7187	0.5199	0.9482	No	1.051	0.604	0.867	0.947	No	No
95856_at	Zfml	Zinc finger, matrin-like	1.0247	0.5736	0.7234	0.1109	No	0.9168	0.429	0.895	0.254	No	No
95857_at	E230006M18Rik	RIKEN cDNA E230006M18 gene	0.86	0.0044	0.6869	0.0655	Yes	0.8933	0.244	0.117	0.414	No	No
95858_at	---	Transcribed locus	1.0053	0.948	0.7367	0.2249	No	0.9565	0.275	0.487	0.0206	No	No
95861_at	D15Ertd55e	DNA segment, Chr 15, ERATO Doi 55, expressed	1.0027	0.9573	0.3093	0.9702	No	0.9346	0.408	0.0334	0.16	No	No
95862_at	D2Ertd63e	DNA segment, Chr 2, ERATO Doi 63, expressed	1.0281	0.6027	0.002	0.0495	No	0.9989	0.682	0.0012	0.0591	No	No
95863_at	---	---	1.0306	0.4432	0.8376	0.377	No	1.0338	0.424	0.0474	0.953	No	No
95864_at	C77815	expressed sequence C77815	0.9545	0.3564	0.2356	0.5512	No	0.8989	0.13	0.001	0.601	No	No
95869_at	---	---	1.002	0.9709	0.0991	0.8511	No	1.086	0.19	0.0129	0.37	No	No
95870_at	D6Ertd160e	DNA segment, Chr 6, ERATO Doi 160, expressed	0.9811	0.5421	0.2272	0.3142	No	0.9072	0.141	0.617	0.0777	No	No
95871_at	D1Ertd164e	DNA segment, Chr 1, ERATO Doi 164, expressed	0.9798	0.2713	0.0452	0.0161	No	1.1178	0.234	0.489	0.628	No	No
95872_at	D7Ertd183e	DNA segment, Chr 7, ERATO Doi 183, expressed	0.9289	0.2594	0.0033	0.2762	No	0.9136	0.105	0.0016	0.176	No	No
95875_at	C78651	expressed sequence C78651	1.0746	0.1766	0.432	0.0347	No	0.9938	0.934	0.21	0.764	No	No
95876_at	Dpf3	D4, zinc and double PHD fingers, family 3	0.9672	0.4238	0.0282	0.1555	No	1.0445	0.0539	0.0002	0.0054	No	No
95877_at	2610020H08 Rik	RIKEN cDNA 2610020H08 gene	1.0072	0.8947	0.4857	0.8982	No	1.0707	0.368	0.586	0.57	No	No
95878_at	LOC493582	hypothetical LOC493582	1.0254	0.3704	0.0828	0.3045	No	1.1268	0.278	0.336	0.735	No	No
95879_at	Asf1a	ASF1 anti-silencing function 1 homolog A (S. cerevisiae)	1.0224	0.7641	0.0015	0.837	No	1.0065	0.668	0.0006	0.425	No	No
95880_s_at	Lmbr1	Limb region 1	1.0547	0.3412	0.4266	0.4033	No	1.1428	0.0955	0.276	0.0469	No	No

95881_f_at	Helb	helicase (DNA) B	1.0676	0.0292	0	0.003	No	1.0594	0.272	0.0015	0.158	No	No
95882_at	---	Transcribed locus	1.0434	0.5203	0.1688	0.8704	No	1.0677	0.468	0.0758	0.58	No	No
95883_at	Phf17	PHD finger protein 17	1.0169	0.8401	0.0071	0.9669	No	0.8834	0.0284	0.0742	0.0159	No	No
95884_at	Tlr7	toll-like receptor 7	1.0121	0.7839	0.2632	0.9558	No	0.9456	0.103	0.573	0.231	No	No
95885_at	1200009F10 Rik	RIKEN cDNA 1200009F10 gene	1.1026	0.1855	0.4489	0.2047	No	0.9742	0.39	0.0012	0.197	No	No
95886_g_at	Crebbp	CREB binding protein	0.992	0.7651	0.0001	0.2026	No	0.9849	0.497	0	0.5	No	No
95887_at	Soat1	sterol O-acyltransferase 1	1.0672	0.0965	0.0017	0.0039	No	1.0829	0.072	0.0035	0.0069	No	No
95888_at	Aff4	AF4/FMR2 family, member 4	0.9238	0.2283	0.0011	0.3722	No	0.9161	0.259	0.0025	0.391	No	No
95889_at	Arf3	ADP-ribosylation factor 3	1.0258	0.6124	0.0431	0.142	No	1.0159	0.586	0.109	0.0589	No	No
95890_r_at	Pax5	Paired box gene 5	1.1069	0.0059	0.012	0.0149	Yes	1.2014	0.158	0.32	0.275	No	No
95891_at	Bex6	brain expressed gene 6	1.0032	0.9476	0.498	0.8687	No	1.0236	0.466	0.449	0.812	No	No
95892_at	---	Transcribed locus	0.9441	0.5834	0.728	0.4243	No	0.8748	0.173	0.0031	0.779	No	No
95893_at	Blk	B lymphoid kinase	0.9517	0.4262	0.0211	0.6079	No	0.9465	0.529	0.246	0.892	No	No
95894_at	Fancm	Fanconi anemia, complementation group M	1.0293	0.6394	0.4157	0.1136	No	1.1803	0.111	0.0198	0.303	No	No
95895_at	Rabepk	Rab9 effector protein with kelch motifs	1.0159	0.8673	0.4811	0.7331	No	1.0435	0.563	0.377	0.863	No	No
95896_at	---	---	1.0764	0.3777	0.0676	0.3	No	1.063	0.491	0.52	0.557	No	No
95897_at	Atp2c1	ATPase, Ca++-sequestering	1.0838	0.3573	0.0034	0.3946	No	1.0598	0.332	0.0009	0.369	No	No
95898_at	Cdh4	cadherin 4	0.9443	0.1894	0.1504	0.7063	No	0.9706	0.175	0.0015	0.0524	No	No
95901_f_at	---	Transcribed locus	1.0255	0.823	0.1679	0.7507	No	1.1019	0.0512	0.0026	0.0225	No	No
95902_at	AI465270	expressed sequence AI465270	1.0649	0.0143	0.0417	0.0031	No	1.0731	0.307	0.172	0.146	No	No
95903_at	Zfp398	zinc finger protein 398	1.0983	0.1112	0.8335	0.1065	No	1.0339	0.736	0.566	0.889	No	No
95904_at	6430543G08 Rik	RIKEN cDNA 6430543G08 gene	1.1392	0.0915	0.0358	0.0497	No	1.1363	0.0346	0.0088	0.114	No	No
95905_at	AI118078	expressed sequence AI118078	1.1026	0.1763	0.6097	0.749	No	0.9645	0.298	0.011	0.0252	No	No
95906_at	4833445I07 Rik	RIKEN cDNA 4833445I07 gene	0.984	0.7285	0.047	0.1329	No	1.0193	0.778	0.236	0.204	No	No
95907_at	---	Transcribed locus	1.0256	0.322	0.147	0.4872	No	1.0106	0.758	0.0078	0.163	No	No
95908_at	Klra1	killer cell lectin-like receptor, subfamily A, member 1	0.9925	0.8999	0.6348	0.6908	No	1.0213	0.774	0.127	0.695	No	No
95909_at	---	Transcribed locus	0.9586	0.3095	0.2344	0.6036	No	0.996	0.943	0.0648	0.944	No	No
95910_f_at	Rbed1	RNA binding motif and ELMO domain 1	0.9006	0.076	0.0325	0.1119	No	0.9812	0.769	0.0349	0.694	No	No
95911_at	Hnrpab	heterogeneous nuclear ribonucleoprotein A/B	1.0408	0.494	0.0843	0.316	No	1.0952	0.212	0.935	0.0855	No	No
95912_at	Rnf2	ring finger protein 2	1.0248	0.5731	0.041	0.2351	No	0.9882	0.786	0.0729	0.826	No	No
95913_at	B230333C21 Rik	RIKEN cDNA B230333C21 gene	0.9538	0.5615	0.0192	0.6612	No	1.0491	0.159	0.0072	0.281	No	No
95914_at	Dido1	death inducer-obliterator 1	0.8751	0.0706	0.003	0.632	No	0.9632	0.653	0.0129	0.981	No	No
95915_at	Gpatc2	G patch domain containing 2	0.9657	0.6033	0.431	0.7593	No	0.9469	0.396	0.441	0.734	No	No
95916_at	Med12l	mediator of RNA polymerase II transcription, subunit 12 homolog (yeast)-like	0.8694	0.026	0.0058	0.0869	No	0.9571	0.379	0.0441	0.435	No	No
95917_at	AA407107	expressed sequence AA407107	0.9603	0.3342	0.0163	0.0395	No	1.0312	0.599	0.225	0.168	No	No
95919_at	---	---	0.9618	0.6327	0.03	0.4653	No	0.9776	0.848	0.0004	0.163	No	No
95920_at	BC016495	cDNA sequence BC016495	0.9728	0.4452	0.7564	0.2342	No	0.9644	0.439	0.653	0.34	No	No
95923_at	Mynn	myoneurin	1.0058	0.9226	0.1143	0.6849	No	1.0332	0.29	0.0019	0.162	No	No
95924_at	1700012B15 Rik	RIKEN cDNA 1700012B15 gene	1.0137	0.7446	0.0027	0.7945	No	1.0049	0.984	0.0035	0.484	No	No
95925_at	AA517631	expressed sequence AA517631	0.9845	0.7634	0.3309	0.2658	No	1.0452	0.69	0.683	0.972	No	No
95926_at	---	---	1.0964	0.307	0.2471	0.3369	No	1.0213	0.541	0.0149	0.083	No	No
95927_f_at	2610201A13 Rik	RIKEN cDNA 2610201A13 gene	0.9147	0.3444	0.0837	0.4597	No	0.9184	0.26	0.129	0.46	No	No
95929_at	Nhlh1	nescient helix loop helix 1	1.0037	0.9363	0.7907	0.128	No	0.9605	0.431	0.161	0.437	No	No
95930_at	---	Transcribed locus	0.9881	0.8443	0.6079	0.6297	No	0.9161	0.115	0.0908	0.0974	No	No
95931_at	Crsp2	Cofactor required for Sp1 transcriptional activation, subunit 2	0.9939	0.8895	0.0433	0.7234	No	1.1568	0.0896	0.0441	0.255	No	No
95932_at	Zp1	zona pellucida glycoprotein 1	1.0122	0.7546	0.1873	0.0711	No	1.0619	0.125	0.431	0.0331	No	No
95933_f_at	Zcchc6	Zinc finger, CCHC domain containing 6	1.014	0.7177	0.0633	0.9411	No	1.0332	0.184	0.0017	0.112	No	No
95934_at	D7Ertd187e	DNA segment, Chr 7, ERATO Doi 187, expressed	0.8679	0.0614	0.0506	0.3842	No	0.8763	0.119	0.0594	0.178	No	No
95935_at	Slc26a2	solute carrier family 26 (sulfate transporter), member 2	1.015	0.5328	0.9253	0.9929	No	0.9447	0.16	0.0124	0.0566	No	No
95936_at	---	Transcribed locus	0.9994	0.9873	0.1417	0.1584	No	0.9918	0.937	0.0074	0.127	No	No
95937_at	0610040J01 Rik	RIKEN cDNA 0610040J01 gene	1.0498	0.4179	0.8616	0.3042	No	1.098	0.089	0.392	0.0385	No	No
95938_at	Huwe1	HECT, UBA and WWE domain containing 1	0.9877	0.8015	0.3942	0.2402	No	1.036	0.687	0.166	0.102	No	No
95939_i_at	BC052328	cDNA sequence BC052328	1.0509	0.1382	0	0.0022	No	1.1305	0.054	0.0001	0.0102	No	No
95940_f_at	BC052328	cDNA sequence BC052328	0.9299	0.3084	0.0018	0.1028	No	1.0266	0.636	0.0009	0.0576	No	No
95941_at	AA675344	expressed sequence AA675344	0.877	0.0053	0.0001	0.0061	No	0.9451	0.17	0.0006	0.336	No	No
95943_at	---	---	0.9724	0.6401	0.0017	0.9599	No	0.9509	0.0412	0	0.366	No	No
95944_at	Dhx36	DEAH (Asp-Glu-Ala-His) box polypeptide 36	0.9337	0.6333	0.0005	0.3728	No	1.0475	0.499	0	0.118	No	No
95945_at	Prdm5	PR domain containing 5	0.9889	0.9041	0.8925	0.7761	No	1.1174	0.105	0.409	0.646	No	No
95946_at	Centd1	Centaurin, delta 1	1.0123	0.8729	0.4562	0.7211	No	1.0375	0.659	0.239	0.74	No	No
95947_at	---	---	0.9849	0.5996	0.8598	0.3954	No	0.8843	0.384	0.913	0.843	No	No
95948_at	Grhl2	Grainyhead-like 2 (Drosophila)	0.9546	0.4517	0.9004	0.3463	No	1.0003	0.853	0.0116	0.161	No	No
95949_at	DXErtd11e	DNA segment, Chr X, ERATO Doi 11, expressed	1.0051	0.9539	0.7313	0.3598	No	1.0199	0.7	0.911	0.0417	No	No
95950_at	---	---	0.9734	0.7428	0.0734	0.6186	No	1.2209	0.0079	0.0009	0.893	Yes	No
95951_at	Clec4d	C-type lectin domain family 4, member d	0.8762	0.1485	0.1205	0.7771	No	0.8286	0.0868	0.0863	0.935	No	No
95952_at	Wnk2	WNK lysine deficient protein kinase 2	0.9275	0.0923	0.5378	0.0753	No	0.9552	0.451	0.977	0.402	No	No
95953_at	D12Ertd123e	DNA segment, Chr 12, ERATO Doi 123, expressed	0.9859	0.7591	0.0968	0.0852	No	0.9576	0.655	0.0217	0.482	No	No
95954_at	D7Ertd143e	DNA segment, Chr 7, ERATO Doi 143, expressed	1.0117	0.8324	0.8407	0.6042	No	1.1043	0.0689	0.0088	0.713	No	No
95955_at	Ppp2r5e	Protein phosphatase 2, regulatory subunit B (B56), epsilon isoform	1.0162	0.787	0.0068	0.1058	No	0.9346	0.241	0.0017	0.31	No	No
95956_at	C77068	expressed sequence C77068	1.0579	0.3935	0.3667	0.1617	No	1.039	0.307	0.0017	0.0957	No	No
95957_at	Setdb1	SET domain, bifurcated 1	1.0019	0.9718	0.7502	0.7121	No	1.0015	0.989	0.967	0.132	No	No
95958_at	C76977	expressed sequence C76977	0.926	0.2694	0.5256	0.7789	No	1.0426	0.587	0.817	0.0928	No	No
95959_at	---	---	0.9362	0.5314	0.0573	0.6138	No	0.9964	0.996	0.0245	0.521	No	No
95960_at	D4Ertd117e	DNA segment, Chr 4, ERATO Doi 117, expressed	0.9023	0.1531	0.2711	0.0981	No	0.9465	0.455	0.959	0.25	No	No
95961_at	Nbeal2	neurobeachin-like 2	0.9831	0.7595	0.0313	0.4373	No	1.0168	0.771	0.0723	0.888	No	No
95962_at	Sh2bp1	SH2 domain binding protein 1 (tetratricopeptide repeat containing)	1.0815	0.3183	0.8516	0.1744	No	1.0238	0.771	0.282	0.832	No	No
95963_at	C77370	expressed sequence C77370	0.9242	0.1972	0.193	0.8085	No	0.9232	0.289	0.0605	0.662	No	No
95964_at	Ubap2l	ubiquitin associated protein 2-like	1.0428	0.5231	0.0285	0.445	No	0.9891	0.755	0.64	0.731	No	No
95965_at	C77545	expressed sequence C77545	1.0526	0.5389	0.2304	0.6249	No	1.0438	0.198	0.0015	0.0966	No	No
95966_at	C77609	expressed sequence C77609	1.0133	0.6363	0.154	0.6138	No	0.9833	0.949	0.0109	0.322	No	No
95967_at	C77681	expressed sequence C77681	1.0425	0.5241	0.4755	0.4473	No	1.0393	0.499	0.042	0.912	No	No
95968_at	C77691	expressed sequence C77691	0.9806	0.665	0.0356	0.2703	No	0.9433	0.0344	0.0001	0.0007	No	No
95970_at	C77874	expressed sequence C77874	1.0388	0.5682	0.0103	0.9503	No	1.0286	0.615	0.198	0.745	No	No
95971_at	C78282	expressed sequence C78282	0.981	0.7656	0.1699	0.6713	No	0.9958	0.88	0.0397	0.549	No	No
95972_at	C78444	expressed sequence C78444	0.9542	0.5871	0.2814	0.7242	No	0.9735	0.507	0.0109	0.0189	No	No
95973_at	C78878	expressed sequence C78878	1.0234	0.6368	0.4308	0.3809	No	0.9855	0.758	0.826	0.663	No	No
95974_at	Gbp1	guanylate nucleotide binding protein 1	1.0944	0.0253	0.0428	0.8414	No	1.0786	0.303	0.596	0.411	No	No
95975_at	A930012N16 Rik	RIKEN cDNA A930012N16 gene	0.9623	0.4212	0.1039	0.0701	No	1.076	0.279	0.803	0.51	No	No
95976_at	Fgf10	fibroblast growth factor 10	0.9525	0.4662	0.6689	0.7695	No	1.0747	0.288	0.0449	0.77	No	No
95977_at	D2Ertd239e	DNA segment, Chr 2, ERATO Doi 239, expressed	1.0146	0.3834	0.0202	0.0029	No	1.0946	0.129	0.246	0.103	No	No
95978_at	Atp13a3	ATPase type 13A3	0.9947	0.8883	0.0098	0.5307	No	1.0583	0.466	0.578	0.762	No	No
95979_at	---	---	1.0994	0.0714	0.0459	0.1977	No	1.1526	0.0006	0.0028	0.0199	Yes	No
95980_at	Snx27	sorting nexin family member 27	1.01	0.7355	0.0092	0.209	No	1.0387	0.402	0.081	0.213	No	No
95981_at	C79329	expressed sequence C79329	0.9399	0.2332	0.0019	0.0771	No	0.8567	0.0977	0.0009	0.47	No	No
95982_at	C920021L13 Rik	RIKEN cDNA C920021L13 gene	0.9647	0.3321	0.1393	0.9689	No	1.0225	0.482	0.661	0.783	No	No
95983_at	---	---	1.0357	0.5308	0.0092	0.7633	No	0.8101	0.112	0.0001	0.623	No	No
95984_at	C79468	expressed sequence C79468	1.0492	0.2128	0.0015	0.4522	No	1.0299	0.319	0.0018	0.0871	No	No
95985_at	Brwd3	bromodomain and WD repeat domain containing 3	1.0133	0.8172	0.3977	0.958	No	1.058	0.0859	0.137	0.331	No	No
95986_at	Sspo	SCO-spondin	1.0012	0.9754	0.0024	0.5831	No	0.9323	0.25	0.0005	0.0174	No	No
95987_at	---	---	0.9784	0.28	0.0064	0.2334	No	1.1197	0.277	0.058	0.0823	No	No
95988_r_at	---	Transcribed locus	1.1197	0.027	0.0557	0.0401	No	1.136	0.263	0.085	0.0535	No	No
95989_at	C79777	expressed sequence C79777	1.0354	0.1473	0.0002	0.0022	No	1.0491	0.352	0.0209	0.309	No	No
95990_at	---	---	1.0431	0.5159	0.1304	0.2335	No	1.0642	0.274	0.0143	0.385	No	No
95991_at	---	---	0.9752	0.611	0.2318	0.6591	No	1.0248	0.366	0.0053	0.0168	No	No
95992_at	C79741	expressed sequence C79741	0.991	0.9152	0.3691	0.9282	No	1.062	0.201	0.0166	0.173	No	No
95994_at	C80012	expressed sequence C80012	1.0678	0.0898	0.0972	0.083	No	1.0103	0.82	0.705	0.446	No	No
95995_at	Fgfr2	Fibroblast growth factor receptor 2	0.9953	0.9387	0.7322	0.4476	No	1.0353	0.353	0.443	0.227	No	No

95996_at	C80256	expressed sequence C80256	1.0435	0.4399	0.0906	0.5106	No	1.1993	0.227	0.0762	0.183	No	No
95997_at	---	---	0.9659	0.5337	0.1053	0.1265	No	0.9222	0.111	0.684	0.194	No	No
95998_at	---	---	0.9772	0.6487	0.4804	0.4132	No	0.9297	0.317	0.815	0.631	No	No
95999_at	---	---	0.9587	0.2002	0.0769	0.0105	No	1.0219	0.659	0.381	0.0263	No	No
96000_at	Atp6v0a1	ATPase, H+ transporting, lysosomal V0 subunit A1	0.9548	0.1568	0.0048	0.9287	No	1.0311	0.605	0.182	0.819	No	No
96001_at	Nudcd3	NudC domain containing 3	1.0115	0.8441	0.2766	0.384	No	1.0342	0.488	0.153	0.0823	No	No
96002_at	Vps24	vacuolar protein sorting 24 (yeast)	0.9201	0.0695	0.0446	0.6109	No	0.9699	0.447	0.043	0.566	No	No
96003_at	Mta2	metastasis-associated gene family, member 2	1.0575	0.3643	0.3468	0.9584	No	1.0399	0.541	0.395	0.539	No	No
96004_at	Sri	sorcin	0.9509	0.0488	0.0007	0.0653	No	0.951	0.0321	0.0003	0.0483	No	No
96007_at	Ssr3	signal sequence receptor, gamma	0.9986	0.9626	0.0049	0.007	No	1.0088	0.934	0.0028	0.0103	No	No
96008_at	Dad1	defender against cell death 1	1.0663	0.0745	0.0003	0.1548	No	1.041	0.1	0.0001	0.227	No	No
96009_s_at	Pap	pancreatitis-associated protein	0.9585	0.3973	0.3742	0.98	No	0.9841	0.459	0.0487	0.741	No	No
96010_at	Kpna3	karyopherin (importin) alpha 3	0.9543	0.3008	0.0032	0.3699	No	0.957	0.186	0.0003	0.196	No	No
96011_at	Matr3	matrin 3	1.0356	0.7205	0.1584	0.1726	No	0.9549	0.705	0.0446	0.234	No	No
96012_f_at	Matr3	matrin 3	1.0243	0.7021	0.0087	0.8111	No	1.0102	0.811	0.113	0.605	No	No
96013_r_at	Matr3	matrin 3	1.3056	0.0382	0.0029	0.307	No	0.9855	0.71	0.0096	0.439	No	No
96014_at	D4Ertd196e	DNA segment, Chr 4, ERATO Doi 196, expressed	0.9796	0.6572	0.1322	0.9923	No	0.9487	0.218	0.0784	0.656	No	No
96016_at	2700094K13 Rik	RIKEN cDNA 2700094K13 gene	1.0777	0.1319	0.0043	0.4352	No	1.0933	0.154	0.0292	0.452	No	No
96017_at	0610006I08 Rik	RIKEN cDNA 0610006I08 gene	1.0831	0.0277	0.0623	0.9245	No	1.0504	0.105	0.0109	0.216	No	No
96018_r_at	Zdhhc5	zinc finger, DHHC domain containing 5	1.1644	0.0546	0.0003	0.1655	No	1.1588	0.0067	0	0.177	Yes	No
96019_at	Sypl	synaptophysin-like protein	0.9539	0.4228	0.0056	0.8443	No	0.9145	0.121	0.0576	0.977	No	No
96020_at	C1qb	complement component 1, q subcomponent, beta polypeptide	0.8057	0.0123	0.2579	0.0826	No	0.9109	0.268	0.0095	0.428	No	No
96021_at	Hbld1	HESB like domain containing 1	0.9208	0.1036	0.0262	0.1768	No	0.8987	0.0087	0.0045	0.0011	No	No
96023_at	Vac14	Vac14 homolog (S. cerevisiae)	0.9849	0.8289	0.179	0.7915	No	1.0152	0.694	0.0572	0.367	No	No
96024_at	Ahcy	S-adenosylhomocysteine hydrolase	1.0034	0.9406	0.0177	0.7484	No	1.0153	0.699	0.0913	0.916	No	No
96025_g_at	Ahcy	S-adenosylhomocysteine hydrolase	1.0654	0.0931	0.0057	0.0475	No	1.0452	0.314	0.0124	0.132	No	No
96026_at	Ahcy	S-adenosylhomocysteine hydrolase	1.0016	0.985	0.4419	0.5507	No	1.0914	0.0695	0.0022	0.135	No	No
96027_at	Sf3a1	splicing factor 3a, subunit 1	0.9175	0.0332	0.3795	0.7097	No	1.0884	0.209	0.0013	0.0921	No	No
96028_at	Brp44l	brain protein 44-like	0.9253	0.3613	0.66	0.9772	No	1.0505	0.535	0.374	0.58	No	No
96029_at	Sf3a3	splicing factor 3a, subunit 3	1.047	0.3618	0.2093	0.3563	No	1.034	0.426	0.0752	0.813	No	No
96030_at	Csna	casein alpha	1.0622	0.6106	0.9468	0.9105	No	1.0684	0.255	0.454	0.909	No	No
96031_r_at	Fbxl5	F-box and leucine-rich repeat protein 5	0.9569	0.1348	0.0072	0.0491	No	0.8912	0.159	0.0102	0.169	No	No
96032_at	Atp5g1	ATP synthase, H+ transporting, mitochondrial F0 complex, subunit c (subunit 9), isoform 1	1.135	0.0894	0.7032	0.0588	No	1.0863	0.0538	0.918	0.018	No	No
96033_at	Sdc1	syndecan 1	1.132	0.0002	0.0058	0.2815	Yes	1.0856	0.0155	0.127	0.648	No	Yes
96035_at	Bckdha	branched chain ketoacid dehydrogenase E1, alpha polypeptide	0.9179	0.0404	0.007	0.1091	No	0.8655	0.0159	0.0034	0.0716	No	No
96036_at	Fis1	Fission 1 (mitochondrial outer membrane) homolog (yeast)	1.0399	0.7284	0.0382	0.7249	No	1.0843	0.399	0.0401	0.744	No	No
96037_at	Bri3	brain protein I3	1.0019	0.9625	0.059	0.9761	No	1.0264	0.448	0.0235	0.501	No	No
96038_at	Rnase4	ribonuclease, RNase A family 4	0.9589	0.6562	0.4025	0.6447	No	0.872	0.184	0.0712	0.0645	No	No
96041_at	LOC671237	similar to Putative RNA-binding protein 3 (RNA-binding motif protein 3)	1.0733	0.2173	0.1456	0.2904	No	1.0152	0.798	0.244	0.608	No	No
96042_at	Sod2	superoxide dismutase 2, mitochondrial	1.0674	0.1686	0.1601	0.0213	No	1.0712	0.0533	0.0205	0.0012	No	No
96043_at	Ppap2a	phosphatidic acid phosphatase 2a	1.0656	0.1732	0.2191	0.4562	No	1.1388	0.149	0.0533	0.176	No	No
96044_at	Bcl2l10	Bcl2-like 10	1.014	0.8777	0.6364	0.5647	No	1.0503	0.621	0.863	0.667	No	No
96045_at	2010321M09 Rik	RIKEN cDNA 2010321M09 gene	1.0296	0.5431	0.0138	0.0958	No	1.0478	0.0638	0.0007	0.012	No	No
96046_at	Hdac1	histone deacetylase 1	0.9951	0.8226	0.0039	0.0257	No	1.029	0.43	0.0114	0.0262	No	No
96047_at	Rbp4	retinol binding protein 4, plasma	0.9475	0.4148	0.8601	0.2238	No	0.9284	0.285	0.702	0.361	No	No
96048_at	Hrsp12	heat-responsive protein 12	1.1135	0.244	0.3763	0.6171	No	0.9295	0.0607	0.172	0.793	No	No
96049_at	Bgn	biglycan	0.9447	0.1718	0.0002	0.6368	No	0.9458	0.375	0.0021	0.782	No	No
96050_at	Smarcb1	SWI/SNF related, matrix associated, actin dependent regulator of chromatin, subfamily b, member 1	1.0495	0.3502	0.5507	0.107	No	1.1558	0.015	0.977	0.012	No	No
96051_at	Guca2a	guanylate cyclase activator 2a (guanylin)	1.0937	0.2628	0.5746	0.1306	No	1.1975	0.0808	0.174	0.108	No	No
96052_at	Acp1 /// LOC631286 /// LOC668486 /// LOC670469 /// LOC676625	acid phosphatase 1, soluble /// similar to acid phosphatase 1, soluble /// similar to acid phosphatase 1 isoform b /// similar to acid phosphatase 1 isoform b /// similar to acid phosphatase 1 isoform b	0.9982	0.9527	0.2887	0.7183	No	1.0577	0.225	0.537	0.478	No	No
96053_i_at	Acp1 /// LOC619997 /// LOC631286 /// LOC665717	acid phosphatase 1, soluble /// similar to acid phosphatase 1, soluble /// similar to acid phosphatase 1, soluble /// similar to acid phosphatase 1, soluble	0.921	0.0565	0.0901	0.0124	No	0.9768	0.687	0.222	0.0764	No	No
96054_f_at	Acp1 /// LOC619997 /// LOC631286 /// LOC665717	acid phosphatase 1, soluble /// similar to acid phosphatase 1, soluble /// similar to acid phosphatase 1, soluble /// similar to acid phosphatase 1, soluble	1.024	0.725	0.1326	0.3409	No	1.0372	0.538	0.153	0.247	No	No
96055_at	Cck	cholecystokinin	1.0064	0.8808	0.0099	0.7743	No	1.0433	0.0846	0.0344	0.0019	No	No
96056_at	Rhoc	ras homolog gene family, member C	0.9985	0.9708	0.0171	0.7089	No	0.9833	0.84	0.0998	0.884	No	No
96057_at	Aldh2	aldehyde dehydrogenase 2, mitochondrial	1.0296	0.5242	0.0039	0.95	No	1.0369	0.0674	0.0002	0.93	No	No
96058_s_at	Aldh2	aldehyde dehydrogenase 2, mitochondrial	0.9654	0.447	0.828	0.9239	No	1.0041	0.861	0.0602	0.305	No	No
96059_at	Mrpl48	mitochondrial ribosomal protein L48	0.9616	0.3068	0.0241	0.0842	No	1.0046	0.87	0.003	0.688	No	No
96060_at	Serpinb6a	serine (or cysteine) peptidase inhibitor, clade B, member 6a	0.9302	0.2623	0.0057	0.2328	No	0.9358	0.252	0.0011	0.296	No	No
96061_at	Usp14	ubiquitin specific peptidase 14	1.0413	0.4958	0.009	0.7906	No	0.9973	0.94	0.41	0.626	No	No
96063_at	Xrcc5	X-ray repair complementing defective repair in Chinese hamster cells 5	0.9065	0.3023	0.1928	0.1306	No	1.1647	0.0853	0.0013	0.0669	No	No
96064_at	Reg3g	regenerating islet-derived 3 gamma	0.941	0.3656	0.0005	0.0549	No	0.9902	0.711	0.0001	0.0252	No	No
96065_at	Lxn	latexin	1.1023	0.36	0.0387	0.5096	No	1.0623	0.0843	0.352	0.0317	No	No
96066_s_at	Pkm2	pyruvate kinase, muscle	0.983	0.6796	0.0134	0.864	No	0.9607	0.247	0.0018	0.832	No	No
96068_at	Immp1l	IMP1 inner mitochondrial membrane peptidase-like (S. cerevisiae)	1.0764	0.4038	0.814	0.6241	No	1.0048	0.985	0.187	0.758	No	No
96069_at	Akr7a5	aldo-keto reductase family 7, member A5 (aflatoxin aldehyde reductase)	0.9495	0.0363	0.0001	0.03	No	0.9337	0.25	0.0063	0.364	No	No
96070_at	Pfn1 /// LOC621501 /// LOC633983	profilin 1 /// similar to profilin 1 /// similar to profilin 1	0.9964	0.9484	0.0057	0.7425	No	0.9998	0.942	0.0005	0.84	No	No
96071_at	Rps25	ribosomal protein S25	0.9732	0.5818	0.0172	0.0678	No	1.1392	0.262	0.875	0.915	No	No
96072_at	Ldha	lactate dehydrogenase A	1.083	0.0139	0.8102	0.1164	No	1.0208	0.389	0.126	0.0546	No	No
96073_at	Dpf2	D4, zinc and double PHD fingers family 2	0.9882	0.6707	0.0699	0.1761	No	1.0135	0.719	0.0853	0.227	No	No
96074_at	Apof	apolipoprotein F	0.9796	0.5558	0.0012	0.8513	No	1.072	0.43	0.0124	0.106	No	No
96075_at	Wdr1	WD repeat domain 1	1.0342	0.2549	0.0015	0.0107	No	1.0797	0.218	0.261	0.0538	No	No
96076_at	Stx5a	syntaxin 5A	1.0091	0.6961	0.0008	0.0807	No	0.9283	0.229	0.0021	0.0073	No	No
96077_at	Slc17a1	solute carrier family 17 (sodium phosphate), member 1	1.2196	0.0495	0.5355	0.1358	No	1.2397	0.0924	0.341	0.0963	No	No
96078_g_at	Slc17a1	solute carrier family 17 (sodium phosphate), member 1	1.0349	0.3479	0.8973	0.2215	No	1.0902	0.063	0.0097	0.0141	No	No
96079_at	0610010K06 Rik	RIKEN cDNA 0610010K06 gene	0.9423	0.168	0.0041	0.2589	No	0.9674	0.139	0.0021	0.0254	No	No
96081_at	Tk1	thymidine kinase 1	1.0475	0.0328	0.0958	0.4082	No	0.963	0.449	0.989	0.608	No	No
96082_at	Mrpl30	mitochondrial ribosomal protein L30	1.016	0.7857	0.4965	0.6726	No	1.0065	0.725	0.0085	0.107	No	No
96083_s_at	Hnrpdl	heterogeneous nuclear ribonucleoprotein D-like	1.2846	0.0013	0.0003	0.0242	Yes	1.2476	0.0117	0.0046	0.11	No	Yes
96084_at	Hnrpdl	heterogeneous nuclear ribonucleoprotein D-like	1.0703	0.1818	0.0036	0.1173	No	1.0423	0.295	0.849	0.592	No	No
96085_at	Gsta4	glutathione S-transferase, alpha 4	1.0766	0.0625	0.0018	0.1805	No	1.0514	0.295	0.211	0.736	No	No
96086_at	1110031B06 Rik	RIKEN cDNA 1110031B06 gene	1.0437	0.3406	0.1151	0.322	No	1.0838	0.0912	0.317	0.53	No	No
96087_at	Mgat1	mannoside acetylglucosaminyltransferase 1	1.0379	0.4523	0.7997	0.5984	No	0.9962	0.871	0.0019	0.543	No	No
96088_at	Ndrg2	N-myc downstream regulated gene 2	0.7426	0.0014	0.0876	0.01	Yes	0.737	0.0011	0.149	0.007	No	No

96089_at	4931406C07 Rik	RIKEN cDNA 4931406C07 gene	0.8969	0.0792	0.0144	0.5633	No	0.8825	0.0142	0.0005	0.0639	No	No
96090_g_at	4931406C07 Rik	RIKEN cDNA 4931406C07 gene	1.0193	0.6064	0.0323	0.9991	No	0.9498	0.489	0.671	0.679	No	No
96091_at	4931406C07 Rik	RIKEN cDNA 4931406C07 gene	0.8967	0.0778	0.0011	0.0582	No	0.9157	0.177	0.0009	0.0317	No	No
96092_at	Hp	haptoglobin	0.9175	0.2079	0.1114	0.4759	No	0.8968	0.118	0.0348	0.254	No	No
96093_at	0610007P14 Rik	RIKEN cDNA 0610007P14 gene	1.0283	0.5619	0.2772	0.2689	No	0.9994	0.998	0.116	0.416	No	No
96094_at	Apoa1	apolipoprotein A-I	0.9952	0.6926	0	0.0009	No	1.0479	0.134	0	0.0811	No	No
96095_i_at	Hsdl2	hydroxysteroid dehydrogenase like 2	1.0243	0.5077	0.1595	0.8152	No	1.1252	0.18	0.481	0.367	No	No
96096_f_at	Hsdl2	hydroxysteroid dehydrogenase like 2	1.1085	0.1149	0.0015	0.3282	No	1.1087	0.177	0.0095	0.288	No	No
96097_at	Chmp6	chromatin modifying protein 6	1.0539	0.1792	0.0167	0.3283	No	1.0554	0.0441	0.0681	0.439	No	No
96098_at	Mrpl36	mitochondrial ribosomal protein L36	1.0006	0.9893	0.6065	0.6173	No	0.9855	0.791	0.1	0.622	No	No
96099_at	Csnk2b	casein kinase II, beta subunit	0.9934	0.7384	0.0061	0.3213	No	0.9994	0.98	0.0894	0.896	No	No
96101_at	Spint2	serine protease inhibitor, Kunitz type 2	1.0844	0.2134	0.0251	0.5098	No	1.1671	0.0083	0.0672	0.0191	Yes	No
96102_i_at	Rad23b	RAD23b homolog (S. cerevisiae)	1.0958	0.0826	0.1298	0.5121	No	1.0516	0.307	0.898	0.316	No	No
96103_f_at	Rad23b	RAD23b homolog (S. cerevisiae)	0.9857	0.6283	0.0066	0.688	No	0.9708	0.374	0.0173	0.552	No	No
96104_at	3732413I11 Rik	RIKEN cDNA 3732413I11 gene	0.9531	0.2805	0.2364	0.2466	No	0.9256	0.589	0.0684	0.942	No	No
96106_at	Rnaseh2a	ribonuclease H2, large subunit	0.9183	0.4091	0.0463	0.0952	No	0.8503	0.155	0.0009	0.394	No	No
96109_at	Klf2	Kruppel-like factor 2 (lung)	0.9522	0.3221	0.1007	0.3189	No	0.8483	0.0083	0.0086	0.0167	Yes	No
96110_at	Cbr1	carbonyl reductase 1	0.9598	0.6432	0.3006	0.4645	No	1.0119	0.846	0.0895	1	No	No
96112_at	Etfa	electron transferring flavoprotein, alpha polypeptide	1.2192	0.1017	0.0058	0.8233	No	1.0486	0.394	0.0218	0.834	No	No
96113_at	D18Wsu98e	DNA segment, Chr 18, Wayne State University 98, expressed	0.9825	0.2499	0.2551	0.9471	No	1.0043	0.911	0.0232	0.154	No	No
96114_at	Ppp1r1a	protein phosphatase 1, regulatory (inhibitor) subunit 1A	0.9724	0.6148	0.0742	0.5182	No	0.9248	0.332	0.0267	0.857	No	No
96115_at	Reep5	receptor accessory protein 5	1.016	0.7075	0.0207	0.8733	No	0.9872	0.693	0.008	0.444	No	No
96116_at	H13	Histocompatibility 13	0.9443	0.1292	0.0202	0.976	No	1.0359	0.503	0.311	0.519	No	No
96117_r_at	H13	histocompatibility 13	0.9378	0.0928	0.8533	0.0355	No	0.9885	0.94	0	0.0053	No	No
96118_at	Angptl4	Angiopoietin-like 4	1.0582	0.2063	0.7151	0.1565	No	1.0434	0.159	0.161	0.0792	No	No
96119_s_at	Angptl4	angiopoietin-like 4	1.0354	0.2837	0.0907	0.0805	No	1.0095	0.834	0.111	0.0931	No	No
96120_at	Dnajb6	DnaJ (Hsp40) homolog, subfamily B, member 6	0.976	0.458	0.1498	0.0548	No	1.0069	0.899	0.138	0.31	No	No
96121_at	Ints5	integrator complex subunit 5	0.9213	0.018	0.0023	0.0871	No	0.9383	0.155	0.0346	0.0354	No	No
96122_at	2310016A09 Rik	RIKEN cDNA 2310016A09 gene	0.9788	0.7225	0.1372	0.6864	No	1.0236	0.468	0.0525	0.345	No	No
96123_at	Lbp	lipopolysaccharide binding protein	0.9534	0.1298	0.1191	0.3947	No	0.9636	0.318	0.0081	0.782	No	No
96124_at	Trak1	trafficking protein, kinesin binding 1	0.959	0.2316	0.0008	0.2732	No	0.9556	0.15	0.0011	0.341	No	No
96125_at	Daxx	Fas death domain-associated protein	0.9453	0.0129	0.5129	0.0033	No	0.9461	0.28	0.258	0.153	No	No
96126_at	Sgpl1	sphingosine phosphate lyase 1	1.079	0.0383	0.0009	0.0217	No	1.0243	0.389	0.0001	0.431	No	No
96127_at	Sgpl1	sphingosine phosphate lyase 1	1.0868	0.1216	0.0628	0.1372	No	1.165	0.301	0.721	0.636	No	No
96128_at	Pctk3	PCTAIRE-motif protein kinase 3	1.0071	0.836	0.4876	0.6242	No	0.9269	0.191	0.241	0.633	No	No
96130_at	Slk	STE20-like kinase (yeast)	1.0358	0.2417	0	0.4758	No	0.9946	0.791	0.0022	0.28	No	No
96131_at	Tbc1d10a	TBC1 domain family, member 10a	0.9812	0.582	0.0026	0.0326	No	0.9285	0.228	0.074	0.87	No	No
96132_at	Apcdd1	adenomatosis polyposis coli down-regulated 1	0.9692	0.155	0.1871	0.0481	No	0.9513	0.183	0.945	0.354	No	No
96134_at	Reep6	receptor accessory protein 6	0.9279	0.1831	0.7911	0.0589	No	0.9833	0.756	0.0629	0.22	No	No
96135_at	3110003A17 Rik	RIKEN cDNA 3110003A17 gene	1.1273	0.1093	0.0218	0.7065	No	1.0516	0.354	0.172	0.867	No	No
96136_at	Exosc10	exosome component 10	0.9898	0.819	0.0014	0.0746	No	0.9831	0.749	0.0017	0.806	No	No
96138_at	Mars	methionine-tRNA synthetase	0.991	0.7429	0.3344	0.1221	No	0.9743	0.433	0.0155	0.157	No	No
96139_at	Gcs1	glucosidase 1	1.0863	0.0345	0.0005	0.4697	No	1.0169	0.472	0.0009	0.72	No	No
96140_at	Pcm1	pericentriolar material 1	1.0358	0.5042	0.0001	0.3887	No	0.9877	0.608	0.0004	0.0562	No	No
96143_at	Epb4.1l4b	erythrocyte protein band 4.1-like 4b	0.9009	0.1215	0.0637	0.1108	No	0.9724	0.534	0.0472	0.592	No	No
96144_at	Id4	inhibitor of DNA binding 4	1.0844	0.1322	0.0028	0.4277	No	1.1224	0.0872	0.0097	0.605	No	No
96145_at	Pigt	phosphatidylinositol glycan, class T	0.8953	0.0796	0.0134	0.7319	No	0.9717	0.431	0.0996	0.16	No	No
96146_at	Btg3 /// LOC640416 /// LOC654432	B-cell translocation gene 3 /// similar to BTG3 protein (Tob5 protein) (Abundant in neuroepithelium area protein) /// B-cell translocation gene 3 pseudogene	1.0717	0.2796	0.082	0.761	No	1.0804	0.258	0.214	0.916	No	No
96147_at	Mafg	v-maf musculoaponeurotic fibrosarcoma oncogene family, protein G (avian)	1.0447	0.4096	0.1184	0.6826	No	1.0659	0.285	0.0411	0.591	No	No
96148_at	Senp6	SUMO/sentrin specific peptidase 6	1.0744	0.2429	0.1574	0.933	No	0.9868	0.833	0.0066	0.506	No	No
96151_at	Mocos	molybdenum cofactor sulfurase	1.0205	0.4246	0.0041	0.1751	No	1.0516	0.424	0.0286	0.79	No	No
96152_at	Narg1	NMDA receptor-regulated gene 1	0.9661	0.6489	0.0129	0.4363	No	1.0033	0.559	0.0065	0.0023	No	No
96153_at	Ngp	neutrophilic granule protein	0.9457	0.0595	0.0005	0.044	No	1.0254	0.828	0.0043	0.0818	No	No
96154_at	Renbp	renin binding protein	0.9807	0.5733	0.3334	0.5648	No	1.0295	0.584	0.717	0.322	No	No
96155_at	Cdk5rap3	CDK5 regulatory subunit associated protein 3	0.9828	0.0814	0.0578	0.001	No	0.9986	0.799	0.0312	0.0016	No	No
96156_at	1110008H02 Rik	RIKEN cDNA 1110008H02 gene	1.0058	0.9086	0.0029	0.0744	No	1.0004	0.917	0.0151	0.419	No	No
96157_at	Zfp91	zinc finger protein 91	0.998	0.9179	0.0013	0.3172	No	1.0307	0.344	0.0007	0.248	No	No
96158_at	BC017133	cDNA sequence BC017133	0.9448	0.2715	0.1578	0.8873	No	0.9403	0.143	0.565	0.289	No	No
96160_at	Slc6a9	solute carrier family 6 (neurotransmitter transporter, glycine), member 9	0.9981	0.9514	0.1541	0.2561	No	0.9697	0.623	0.336	0.267	No	No
96162_at	Fbxo15	F-box protein 15	1.0435	0.4257	0.6091	0.3959	No	1.0929	0.278	0.276	0.0323	No	No
96165_at	LOC677213	similar to U2AF homology motif (UHM) kinase 1	0.9528	0.2731	0.0011	0.6226	No	0.977	0.653	0.0196	0.576	No	No
96166_at	Tex261	testis expressed gene 261	1.0277	0.297	0.003	0.7322	No	1.0293	0.393	0.0467	0.997	No	No
96167_at	Bag3	Bcl2-associated athanogene 3	0.9415	0.1065	0.8775	0.1285	No	0.9724	0.188	0.267	0.0473	No	No
96168_at	Kif23	kinesin family member 23	1.0858	0.3989	0.2535	0.8674	No	1.0734	0.365	0.0933	0.119	No	No
96169_at	---	CDNA clone IMAGE:30031514	1.1137	0.0189	0.3692	0.0279	No	1.0432	0.405	0.466	0.0767	No	No
96171_at	Deaf1	deformed epidermal autoregulatory factor 1 (Drosophila)	1.037	0.2891	0.1055	0.6542	No	1.0863	0.263	0.663	0.664	No	No
96172_at	Gimap4	GTPase, IMAP family member 4	0.9922	0.9239	0.4909	0.7993	No	1.1129	0.086	0.0023	0.512	No	No
96174_at	Pom121	nuclear pore membrane protein 121	0.9666	0.2806	0.5879	0.419	No	1.011	0.834	0.0977	0.535	No	No
96176_at	Arih2	ariadne homolog 2 (Drosophila)	0.9707	0.5304	0.0662	0.0771	No	1.086	0.219	0.655	0.52	No	No
96177_at	Mms19l	MMS19 (MET18 S. cerevisiae)-like	0.9081	0.0449	0.0465	0.3629	No	1.0261	0.381	0.0213	0.036	No	No
96178_at	Myst2	MYST histone acetyltransferase 2	1.0731	0.0837	0.3364	0.1213	No	1.0364	0.112	0.203	0.0115	No	No
96180_at	---	---	0.995	0.9465	0.8883	0.7931	No	0.8718	0.065	0.409	0.442	No	No
96183_at	Foxp1	forkhead box P1	0.941	0.1844	0.0065	0.0633	No	0.9219	0.162	0.34	0.146	No	No
96184_at	Rnpepl1	arginyl aminopeptidase (aminopeptidase B)-like 1	0.973	0.6695	0.0346	0.1538	No	0.9218	0.0604	0.295	0.0129	No	No
96185_at	Ap3d1	adaptor-related protein complex 3, delta 1 subunit	1.0345	0.3127	0.0009	0.0294	No	0.9904	0.541	0.0005	0.102	No	No
96186_at	Lrp10	low-density lipoprotein receptor-related protein 10	1.0069	0.8999	0.0273	0.5384	No	1.0367	0.534	0.0557	0.493	No	No
96187_at	Pkp4	plakophilin 4	1.0756	0.1371	0.0005	0.3532	No	1.0296	0.638	0.0007	0.787	No	No
96188_at	Adar	adenosine deaminase, RNA-specific	1.0905	0.0421	0.429	0.8543	No	0.9898	0.394	0.0009	0.0126	No	No
96189_at	Zcchc10	zinc finger, CCHC domain containing 10	1.0906	0.4746	0.1071	0.4132	No	0.9406	0.313	0.225	0.818	No	No
96191_at	Arfgef1	ADP-ribosylation factor guanine nucleotide-exchange factor 1(brefeldin A-inhibited)	0.8503	0.0009	0.1414	0.0521	Yes	0.8904	0.0655	0.302	0.724	No	No
96192_at	Sp3	trans-acting transcription factor 3	0.8829	0.1851	0.0111	0.8515	No	0.8927	0.17	0.0008	0.618	No	No
96193_at	Dmwd	dystrophia myotonica-containing WD repeat motif	0.9821	0.6934	0.0061	0.2806	No	0.9604	0.52	0.0418	0.6	No	No
96195_at	Efs	embryonal Fyn-associated substrate	1.0279	0.4549	0.893	0.7735	No	1.0557	0.194	0.0037	0.233	No	No
96196_i_at	5730589K01 Rik	RIKEN cDNA 5730589K01 gene	0.9539	0.1706	0.0021	0.0863	No	1.032	0.827	0.0004	0.0093	No	No
96197_f_at	5730589K01 Rik	RIKEN cDNA 5730589K01 gene	1.0077	0.8931	0.0439	0.4301	No	0.9589	0.426	0.027	0.885	No	No
96198_at	Prkcz	protein kinase C, zeta	1.049	0.1176	0.2281	0.113	No	1.0401	0.309	0.0025	0.0963	No	No
96199_at	Mtm1	X-linked myotubular myopathy gene 1	1.0997	0.0896	0.0137	0.0145	No	1.0941	0.0261	0.0967	0.804	No	No
96200_at	Cdca4	cell division cycle associated 4	1.045	0.1271	0.0022	0.3893	No	1.0347	0.512	0.106	0.505	No	No
96201_at	Gpr172b	G protein-coupled receptor 172B	0.9564	0.3251	0.3979	0.1524	No	0.8997	0.0455	0.18	0.0139	No	No
96202_at	Slc1a2	solute carrier family 1 (glial high affinity glutamate transporter), member 2	1.0759	0.3602	0.2796	0.1593	No	1.0807	0.283	0.0229	0.14	No	No
96203_at	Calml4	calmodulin-like 4	1.1066	0.0824	0.0014	0.3477	No	0.9659	0.601	0.011	0.709	No	No
96204_at	Sh3bgr	SH3-binding domain glutamic acid-rich protein	0.8892	0.2619	0.0372	0.2735	No	1.0029	0.837	0.0101	0.314	No	No
96205_at	Sh3bgr	SH3-binding domain glutamic acid-rich protein	0.7966	0.0139	0.5811	0.0137	No	0.8696	0.0151	0.36	0.0798	No	No
96206_at	Phldb1	pleckstrin homology-like domain, family B, member 1	0.9798	0.6207	0.0178	0.1343	No	0.9385	0.277	0.0066	0.18	No	No
96207_at	Rbms1	RNA binding motif, single stranded interacting protein 1	0.8929	0.1443	0.0099	0.6573	No	0.8692	0.154	0.0165	0.937	No	No
96208_at	Grlf1	glucocorticoid receptor DNA binding factor 1	1.0074	0.8629	0.0731	0.5553	No	1.0247	0.593	0.0135	0.7	No	No
96211_at	2310004I03 Rik	RIKEN cDNA 2310004I03 gene	0.8435	0.0019	0.0927	0.4581	Yes	1.0057	0.917	0.222	0.322	No	No

96212_at	2310061I04 Rik	RIKEN cDNA 2310061I04 gene	0.9086	0.08	0.0471	0.049	No	1.0603	0.134	0.0128	0.281	No	No
96214_at	---	---	1.0021	0.9649	0.0143	0.1206	No	1.0433	0.504	0.0515	0.511	No	No
96215_f_at	---	---	1.0556	0.4649	0.0556	0.8222	No	1.0992	0.244	0.0105	0.387	No	No
96216_at	Ormdl1	ORM1-like 1 (S. cerevisiae)	1.0335	0.2596	0.5468	0.2166	No	1.0983	0.0883	0.0024	0.628	No	No
96217_at	Polb	polymerase (DNA directed), beta	0.9956	0.9398	0.0641	0.188	No	0.9451	0.313	0.631	0.0633	No	No
96218_at	Fntb	farnesyltransferase, CAAX box, beta	0.9046	0.004	0.0002	0.2424	Yes	0.9437	0.0978	0.0139	0.213	No	No
96219_at	Smap1l	stromal membrane-associated protein 1-like	0.9887	0.7105	0.002	0.181	No	1.0381	0.167	0.002	0.087	No	No
96220_at	Lig3	ligase III, DNA, ATP-dependent	0.923	0.3332	0.008	0.2848	No	0.9555	0.36	0.0045	0.091	No	No
96221_at	Traf3ip2	Traf3 interacting protein 2	0.9732	0.4527	0.0588	0.0546	No	0.9663	0.278	0.0539	0.765	No	No
96222_at	BC003993	cDNA sequence BC003993	0.8179	0.0379	0.0497	0.1494	No	0.8859	0.0592	0.0312	0.22	No	No
96223_at	Dcp1a	decapping enzyme	0.9822	0.6782	0.5096	0.328	No	0.9416	0.0893	0.223	0.934	No	No
96224_at	Btrc	beta-transducin repeat containing protein	1.0005	0.9892	0.61	0.414	No	1.004	0.929	0.413	0.0703	No	No
96226_at	Nt5c3l	5'-nucleotidase, cytosolic III-like	1.0275	0.4991	0.0003	0.0536	No	0.9709	0.771	0	0.0641	No	No
96227_at	Serpina6	serine (or cysteine) peptidase inhibitor, clade A, member 6	1.0257	0.4005	0.953	0.7584	No	1.0904	0.24	0.876	0.799	No	No
96228_at	Cyp11a1	cytochrome P450, family 11, subfamily a, polypeptide 1	0.9748	0.7132	0.009	0.2659	No	0.9553	0.469	0.0101	0.0766	No	No
96229_at	Crp	C-reactive protein, pentraxin-related	0.9842	0.8599	0.4636	0.4809	No	1.0717	0.48	0.14	0.132	No	No
96230_at	Cntrob	centrobin, centrosomal BRCA2 interacting protein	1.0102	0.7744	0.0374	0.0354	No	1.0407	0.389	0.754	0.151	No	No
96231_at	Bphl	biphenyl hydrolase-like (serine hydrolase, breast epithelial mucin-associated antigen)	0.9222	0.0484	0.0735	0.0199	No	1.0624	0.207	0.0542	0.724	No	No
96232_at	Cul2	cullin 2	1.0076	0.8953	0.0173	0.5304	No	0.9273	0.0254	0.0522	0.0182	No	No
96234_at	Cpsf3	cleavage and polyadenylation specificity factor 3	1.0003	0.996	0.736	0.5873	No	0.9811	0.666	0.013	0.97	No	No
96236_at	Cdc16	CDC16 cell division cycle 16 homolog (S. cerevisiae)	0.9339	0.0447	0.0016	0.0406	No	0.9281	0.128	0.0025	0.0675	No	No
96237_at	BC054059	cDNA sequence BC054059	1.1219	0.003	0.0059	0.0035	No	1.0915	0.0669	0.0058	0.509	No	No
96238_at	---	---	0.9791	0.834	0.1238	0.9477	No	0.9925	0.939	0.824	0.691	No	No
96239_at	Vrk3	vaccinia related kinase 3	0.9581	0.0972	0.001	0.0337	No	0.8957	0.486	0.0726	0.784	No	No
96240_at	Tmem106c	transmembrane protein 106C	0.9968	0.9493	0.0095	0.9513	No	0.9461	0.645	0.474	0.648	No	No
96241_at	D10Bwg1364e	DNA segment, Chr 10, Brigham & Women's Genetics 1364 expressed	1.0559	0.1348	0.0009	0.0098	No	1.0746	0.17	0.0016	0.242	No	No
96242_at	D10Bwg1364e	DNA segment, Chr 10, Brigham & Women's Genetics 1364 expressed	1.0993	0.0512	0.1991	0.3131	No	1.0334	0.575	0.0149	0.494	No	No
96243_f_at	Aldh9a1	aldehyde dehydrogenase 9, subfamily A1	1.1527	0.2463	0.1315	0.6024	No	1.074	0.373	0.0305	0.11	No	No
96244_at	Uchl1	ubiquitin carboxy-terminal hydrolase L1	1.041	0.5508	0.0188	0.9703	No	0.9931	0.609	0.0013	0.0727	No	No
96245_at	Ddx56	DEAD (Asp-Glu-Ala-Asp) box polypeptide 56	1.0466	0.2219	0.0155	0.1768	No	1.0585	0.19	0.0319	0.174	No	No
96249_at	38975	selenoprotein	0.9922	0.8646	0.0789	0.5889	No	0.9924	0.9	0.339	0.534	No	No
96252_at	Pdcd6ip	programmed cell death 6 interacting protein	0.9794	0.7441	0.7657	0.9708	No	1.0023	0.956	0.522	0.96	No	No
96254_at	Dnajb1	DnaJ (Hsp40) homolog, subfamily B, member 1	1.0132	0.7497	0.1275	0.0199	No	1.0575	0.594	0.354	0.257	No	No
96255_at	Bnip3l	BCL2/adenovirus E1B interacting protein 3-like	1.0091	0.877	0.7313	0.527	No	0.9435	0.491	0.127	0.478	No	No
96256_at	Prdx3	peroxiredoxin 3	0.9623	0.2765	0.0239	0.53	No	0.9897	0.484	0.0053	0.8	No	No
96257_at	Hp1bp3	heterochromatin protein 1, binding protein 3	0.9907	0.8126	0.0021	0.126	No	1.0252	0.465	0.0003	0.623	No	No
96258_at	Mgst3	microsomal glutathione S-transferase 3	1.0071	0.9117	0.3141	0.1408	No	1.002	0.955	0.719	0.208	No	No
96259_at	Npepps	aminopeptidase puromycin sensitive	0.9336	0.0683	0.1334	0.2062	No	1.0045	0.922	0.0498	0.0396	No	No
96260_at	AL033314	expressed sequence AL033314	1.0418	0.5206	0.0245	0.4732	No	1.1167	0.0938	0.0014	0.0943	No	No
96261_at	2310028O11 Rik	RIKEN cDNA 2310028O11 gene	1.0547	0.2221	0.1891	0.5699	No	1.0138	0.828	0.218	0.102	No	No
96262_at	Rab5c	RAB5C, member RAS oncogene family	0.9422	0.1232	0.0576	0.063	No	1.0131	0.771	0.0027	0.0304	No	No
96263_at	Rshl2	radial spokehead-like 2	0.93	0.2909	0.1568	0.4045	No	0.9172	0.168	0.805	0.51	No	No
96264_at	Wdr45l	Wdr45 like	0.9809	0.5584	0.0021	0.0583	No	0.9799	0.572	0.0042	0.599	No	No
96266_at	Hnrpm	heterogeneous nuclear ribonucleoprotein M	0.9922	0.9011	0.0455	0.3334	No	1.0638	0.0373	0	0.553	No	No
96267_at	Ndufv1	NADH dehydrogenase (ubiquinone) flavoprotein 1	1.0055	0.7708	0.1546	0.1433	No	0.9962	0.837	0.376	0.0687	No	No
96268_at	Suclg1	succinate-CoA ligase, GDP-forming, alpha subunit	1.0729	0.115	0.7947	0.1134	No	1.0899	0.174	0.0138	0.316	No	No
96269_at	Idi1	isopentenyl-diphosphate delta isomerase	1.1667	0.0553	0.1412	0.9203	No	1.2255	0.0435	0.0229	0.466	No	No
96270_at	D11Bwg0434e	DNA segment, Chr 11, Brigham & Women's Genetics 0434 expressed	0.9961	0.9309	0.0121	0.8713	No	1.0079	0.818	0.0268	0.759	No	No
96271_at	Tmem123	transmembrane protein 123	1.0416	0.5295	0.0066	0.5772	No	1.0412	0.549	0.0038	0.481	No	No
96272_at	Ptprf	protein tyrosine phosphatase, receptor type, F	0.9968	0.942	0.1697	0.2619	No	0.9602	0.412	0.6	0.618	No	No
96273_at	Nrgn	neurogranin	0.945	0.1208	0.0006	0.011	No	1.0623	0.172	0.0617	0.853	No	No
96275_f_at	Tm9sf3	transmembrane 9 superfamily member 3	1.0814	0.4099	0.0385	0.6908	No	1.0377	0.536	0.0051	0.436	No	No
96276_r_at	Tm9sf3	transmembrane 9 superfamily member 3	1.0009	0.9892	0.0074	0.4332	No	1.0342	0.666	0.764	0.0646	No	No
96277_at	Arpc5	Actin related protein 2/3 complex, subunit 5	1.0206	0.6786	0.0547	0.6066	No	0.9305	0.39	0.149	0.506	No	No
96278_at	Prr13	proline rich 13	0.9003	0.174	0.5988	0.6099	No	1.0283	0.562	0.0062	0.684	No	No
96280_at	Ndufa2	NADH dehydrogenase (ubiquinone) 1 alpha subcomplex, 2	1.0607	0.3205	0.7161	0.8667	No	1.0564	0.223	0.0106	0.182	No	No
96281_at	Atp6v1g1	ATPase, H+ transporting, lysosomal V1 subunit G1	1.1029	0.0725	0.0921	0.9937	No	1.0209	0.554	0.117	0.897	No	No
96283_at	Itm2c	integral membrane protein 2C	0.9921	0.8133	0.071	0.1766	No	0.9686	0.467	0.0107	0.0589	No	No
96284_at	Csnk1g2	casein kinase 1, gamma 2	0.9886	0.6774	0.0001	0.4728	No	1.0023	0.811	0.0009	0.525	No	No
96285_at	Myadm	myeloid-associated differentiation marker	0.8456	0.0013	0.7095	0.6768	Yes	0.871	0.007	0.0357	0.992	Yes	Yes
96286_at	Ubap1	ubiquitin-associated protein 1	0.9767	0.1955	0.0093	0.6079	No	0.9379	0.259	0.342	0.367	No	No
96287_at	Dut	deoxyuridine triphosphatase	0.9795	0.5877	0.1424	0.2897	No	1.0514	0.417	0.499	0.505	No	No
96288_at	1190002C06 Rik	RIKEN cDNA 1190002C06 gene	0.9961	0.9341	0.0092	0.5888	No	1.0663	0.108	0.192	0.0423	No	No
96289_at	Stoml2	stomatin (Epb7.2)-like 2	0.9377	0.2049	0.9723	0.0691	No	1.009	0.668	0.0153	0.0872	No	No
96290_f_at	Rpl21	ribosomal protein L21	1.0763	0.0546	0.0838	0.0879	No	1.0205	0.321	0.568	0.0069	No	No
96291_f_at	Ndufs6 /// LOC623286 /// LOC631040 /// LOC675068	NADH dehydrogenase (ubiquinone) Fe-S protein 6 /// similar to NADH dehydrogenase (ubiquinone) Fe-S protein 6 /// similar to NADH dehydrogenase (ubiquinone) Fe-S protein 6 /// similar to NADH dehydrogenase (ubiquinone) Fe-S protein 6	1.0373	0.4832	0.0057	0.0126	No	1.1148	0.0922	0.241	0.0242	No	No
96292_r_at	Ndufs6 /// LOC631040	NADH dehydrogenase (ubiquinone) Fe-S protein 6 /// similar to NADH dehydrogenase (ubiquinone) Fe-S protein 6	1.0676	0.2469	0.0051	0.8815	No	1.126	0.0557	0.0294	0.586	No	No
96293_at	2410015N17 Rik	RIKEN cDNA 2410015N17 gene	1.0034	0.9551	0.2802	0.6143	No	1.0259	0.604	0.181	0.202	No	No
96294_s_at	2410015N17 Rik	RIKEN cDNA 2410015N17 gene	1.0037	0.9235	0.0013	0.17	No	0.9744	0.733	0.0022	0.161	No	No
96295_at	Psat1	phosphoserine aminotransferase 1	1.066	0.4482	0.0168	0.6347	No	1.0951	0.0505	0.692	0.373	No	No
96296_at	Mrpl15	mitochondrial ribosomal protein L15	0.9428	0.0402	0.0012	0.0247	No	0.9575	0.35	0.0061	0.0992	No	No
96297_at	Ebna1bp2	EBNA1 binding protein 2	1.1166	0.1733	0.1042	0.2912	No	1.0462	0.411	0.0179	0.312	No	No
96298_f_at	Dynll1	dynein light chain LC8-type 1	1.0272	0.4188	0.0004	0.221	No	1.0062	0.357	0.0001	0.0267	No	No
96299_at	---	Transcribed locus, strongly similar to XP_580165.1 PREDICTED: hypothetical protein XP_580165 [Rattus norvegicus]	1.0897	0.1106	0.009	0.0135	No	0.9957	0.921	0.0241	0.0942	No	No
96300_f_at	Rps27	ribosomal protein S27	1.01	0.8436	0.1697	0.7418	No	0.9989	0.969	0.006	0.128	No	No
96301_at	Rps27	ribosomal protein S27	0.9907	0.849	0.0615	0.9246	No	1.0457	0.227	0.389	0.0251	No	No
96302_at	Sfrs7	Splicing factor, arginine/serine-rich 7	1.0636	0.4929	0.0017	0.4805	No	1.0365	0.569	0.0008	0.91	No	No
96305_at	Bud31	BUD31 homolog (yeast)	1.0052	0.8599	0.045	0.0524	No	0.9931	0.653	0.0002	0.0012	No	No
96306_at	Polr2i	polymerase (RNA) II (DNA directed) polypeptide I	0.9899	0.7335	0.0081	0.06	No	0.9354	0.322	0.0094	0.0245	No	No
96307_s_at	1100001I22 Rik	RIKEN cDNA 1100001I22 gene	0.9887	0.5022	0.0045	0.8152	No	0.9721	0.119	0.0057	0.12	No	No
96308_r_at	1100001I22 Rik	RIKEN cDNA 1100001I22 gene	1.016	0.7909	0.0334	0.3029	No	1.0832	0.243	0.934	0.228	No	No
96309_r_at	1100001I22 Rik	RIKEN cDNA 1100001I22 gene	0.93	0.4185	0.0002	0.3006	No	0.949	0.267	0.0001	0.47	No	No
96310_at	Mbp	myelin basic protein	0.9426	0.0296	0.0055	0.5303	No	1.1024	0.0972	0.817	0.135	No	No
96311_at	Mbp	myelin basic protein	1.0064	0.7857	0.0026	0.0042	No	1.047	0.768	0.008	0.354	No	No
96313_at	Rasgrf1	RAS protein-specific guanine nucleotide-releasing factor 1	0.9915	0.6346	0.1629	0.4184	No	1.0839	0.68	0.386	0.985	No	No

96316_at	Sumo2 /// LOC236622 /// LOC546828 /// LOC628282 /// LOC665283 /// LOC671851 /// LOC672381 /// LOC675440 /// LOC676940 /// LOC677190	SMT3 suppressor of mif two 3 homolog 2 (yeast) /// similar to SMT3 suppressor of mif two 3 homolog 2 /// similar to small ubiquitin-like modifier 2 isoform b precursor /// similar to SMT3 suppressor of mif two 3 homolog 2 /// similar to SMT3 suppressor of mif two 3 homolog 2 /// similar to SMT3 suppressor of mif two 3 homolog 2 /// similar to SMT3 suppressor of mif two 3 homolog 2 /// similar to SMT3 suppressor of mif two 3 homolog 2 /// similar to SMT3 suppressor of mif two 3 homolog 2	0.9725	0.7373	0.4922	0.6172	No	1.0444	0.341	0.0251	0.343	No	No
96318_at	D17Wsu104e	DNA segment, Chr 17, Wayne State University 104, expressed	1.0676	0.2162	0.0026	0.2685	No	1.0593	0.0592	0.0002	0.386	No	No
96319_at	Cdc20	cell division cycle 20 homolog (S. cerevisiae)	0.9971	0.9334	0.4402	0.5105	No	0.9006	0.0521	0.741	0.0708	No	No
96320_at	2310044H10Rik	RIKEN cDNA 2310044H10 gene	1.0404	0.5074	0.005	0.0917	No	0.9798	0.764	0.0147	0.136	No	No
96321_at	Ndufa9	NADH dehydrogenase (ubiquinone) 1 alpha subcomplex, 9	1.0001	0.9976	0.0328	0.3995	No	1.0129	0.769	0.0301	0.548	No	No
96322_at	Edf1	endothelial differentiation-related factor 1	1.0543	0.4012	0.033	0.3753	No	1.0848	0.0844	0.0578	0.0224	No	No
96324_at	Kars /// LOC631033	lysyl-tRNA synthetase /// similar to lysyl-tRNA synthetase	1.0761	0.394	0.8541	0.198	No	1.0887	0.0134	0.0037	0.0162	No	No
96325_at	2510039O18Rik	RIKEN cDNA 2510039O18 gene	1.0738	0.2499	0.365	0.1655	No	1.0387	0.709	0.0375	0.0283	No	No
96326_at	Tat	tyrosine aminotransferase	1.0289	0.7346	0.7615	0.3355	No	1.0549	0.4	0.909	0.626	No	No
96327_at	Zfp306	zinc finger protein 306	1.0495	0.0366	0.3969	0.0281	No	1.0346	0.343	0.694	0.0451	No	No
96329_at	2410104I19Rik	RIKEN cDNA 2410104I19 gene	0.9442	0.225	0.0368	0.6275	No	0.9683	0.448	0.0042	0.184	No	No
96331_at	Snx2	sorting nexin 2	1.1162	0.0685	0.0031	0.9875	No	1.0093	0.914	0.0187	0.486	No	No
96332_at	Snx2	sorting nexin 2	1.0333	0.3313	0.23	0.8457	No	0.9802	0.848	0.235	0.586	No	No
96333_g_at	Snx2	sorting nexin 2	0.9752	0.5782	0.5717	0.8436	No	0.9574	0.422	0.0901	0.853	No	No
96334_f_at	C730004C24Rik	RIKEN cDNA C730004C24 gene	0.9812	0.6255	0.0721	0.7828	No	0.9442	0.277	0.0779	0.35	No	No
96335_at	Mrpl38	mitochondrial ribosomal protein L38	0.9815	0.5546	0.0168	0.0283	No	0.9796	0.208	0.0691	0.0394	No	No
96336_at	Gatm	glycine amidinotransferase (L-arginine:glycine amidinotransferase)	0.9493	0.4293	0.7219	0.3037	No	0.9463	0.26	0.0048	0.574	No	No
96337_at	Gp1bb /// Sept5	glycoprotein Ib, beta polypeptide /// septin 5	0.8552	0.0702	0.0036	0.4986	No	0.8827	0.108	0.583	0.56	No	No
96338_at	Egln2	EGL nine homolog 2 (C. elegans)	1.0134	0.7238	0.3679	0.2917	No	0.956	0.186	0.0144	0.138	No	No
96339_at	---	Transcribed locus	1.1532	0.0269	0.8099	0.0267	No	1.196	0.0551	0.904	0.165	No	No
96340_at	Tmem50b	transmembrane protein 50B	1.0211	0.605	0.2114	0.4934	No	0.9788	0.437	0.004	0.077	No	No
96341_at	Syf2	SYF2 homolog, RNA splicing factor (S. cerevisiae)	0.8751	0.0426	0.2825	0.0666	No	0.9063	0.0667	0.0844	0.753	No	No
96342_at	Hspc171	HSPC171 protein	1.0734	0.0036	0.0001	0.0028	No	0.992	0.932	0.0007	0.0027	No	No
96343_at	Actn4	actinin alpha 4	1.1458	0.136	0.2021	0.8409	No	1.0262	0.432	0.383	0.0667	No	No
96344_at	Eno3	enolase 3, beta muscle	0.7251	0.0002	0.4067	0.0002	No	0.7598	0.0011	0.335	0.0007	No	No
96345_at	Ergic3	ERGIC and golgi 3	1.0067	0.9097	0.0247	0.2181	No	0.9909	0.922	0.0024	0.235	No	No
96346_at	Cdo1	cysteine dioxygenase 1, cytosolic	0.946	0.2744	0.0116	0.4996	No	0.8864	0.0385	0.0368	0.996	No	No
96347_at	Rcn3	reticulocalbin 3, EF-hand calcium binding domain	0.9771	0.5796	0.6918	0.7594	No	1.1113	0.16	0.0274	0.0468	No	No
96348_at	Pnpla2	patatin-like phospholipase domain containing 2	0.9337	0.0491	0.0073	0.0179	No	0.9267	0.0445	0.0009	0.0113	No	No
96351_at	Btf3l4	basic transcription factor 3-like 4	1.0406	0.4358	0.0305	0.658	No	1.0736	0.463	0.583	0.692	No	No
96352_at	2400001E08Rik	RIKEN cDNA 2400001E08 gene	1.0225	0.7032	0.0798	0.5376	No	0.9879	0.928	0.313	0.0678	No	No
96353_at	Tmem14c	transmembrane protein 14C	1.0411	0.0078	0.0087	0.0031	No	1.039	0.353	0.515	0.143	No	No
96354_at	Mbnl1	muscleblind-like 1 (Drosophila)	1.0516	0.3842	0.083	0.8807	No	1.0178	0.784	0.248	0.878	No	No
96355_at	D10Ertd322e /// LOC623346 /// LOC630452 /// LOC670566 /// LOC675006	DNA segment, Chr 10, ERATO Doi 322, expressed /// similar to mitochondrial ribosomal protein L42 /// similar to mitochondrial ribosomal protein L42 /// hypothetical protein LOC670566 /// hypothetical protein LOC675006	0.9783	0.7071	0.5112	0.4893	No	1.0237	0.825	0.632	0.0571	No	No
96356_at	Arpc1b	Actin related protein 2/3 complex, subunit 1B	0.9482	0.5362	0.0974	0.9768	No	1.0462	0.777	0.243	0.592	No	No
96357_at	Arpc1b /// LOC434782 /// LOC671552	actin related protein 2/3 complex, subunit 1B /// similar to Actin-related protein 2/3 complex subunit 1B (ARP2/3 complex 41 kDa subunit) (p41-ARC) /// similar to Actin-related protein 2/3 complex subunit 1B (ARP2/3 complex 41 kDa subunit) (p41-ARC)	0.9394	0.0895	0.0074	0.1338	No	1.0205	0.612	0.0157	0.766	No	No
96358_at	Rps23	ribosomal protein S23	0.9928	0.7946	0.0039	0.4401	No	0.9852	0.27	0.0002	0.0275	No	No
96359_at	Hdlbp	high density lipoprotein (HDL) binding protein	0.9905	0.8281	0.1032	0.6341	No	1.0232	0.436	0.014	0.57	No	No
96360_at	Arhgdia	Rho GDP dissociation inhibitor (GDI) alpha	0.9912	0.8769	0.0034	0.4264	No	0.9517	0.0967	0.774	0.15	No	No
96365_at	Slco6c1	solute carrier organic anion transporter family, member 6c1	1.0194	0.4149	0.0389	0.1779	No	0.8111	0.197	0.96	0.0465	No	No
96367_at	Ubr2	Ubiquitin protein ligase E3 component n-recognin 2	0.8891	0.1414	0.4041	0.8174	No	1.0534	0.528	0.0622	0.219	No	No
96373_at	Ube2h	Ubiquitin-conjugating enzyme E2H	0.9848	0.8435	0.0556	0.1909	No	0.9775	0.749	0.0746	0.168	No	No
96374_at	C80171	expressed sequence C80171	0.9728	0.6659	0.0538	0.6134	No	1.0323	0.816	0.312	0.443	No	No
96375_at	D9Ertd306e	DNA segment, Chr 9, ERATO Doi 306, expressed	1.1135	0.0647	0.0007	0.1306	No	1.1588	0.166	0.0209	0.0508	No	No
96387_at	1700016D02Rik /// LOC666415 /// LOC666699 /// LOC676410	RIKEN cDNA 1700016D02 gene /// hypothetical protein LOC666415 /// hypothetical protein LOC666699 /// hypothetical protein LOC676410	0.9448	0.0262	0.5008	0.0114	No	1.0145	0.818	0.771	0.466	No	No
96388_at	C80678	expressed sequence C80678	0.9941	0.8143	0.111	0.1622	No	0.9958	0.912	0.584	0.88	No	No
96389_at	C80719	expressed sequence C80719	0.9632	0.2878	0.7243	0.0754	No	0.9654	0.756	0.0074	0.113	No	No
96390_at	Usp53	Ubiquitin specific peptidase 53	1.2203	0.0167	0.5609	0.006	No	1.1799	0.126	0.197	0.151	No	No
96391_at	---	Transcribed locus, moderately similar to XP_574723.1 PREDICTED: similar to LRRGT00097 [Rattus norvegicus]	0.97	0.4494	0.1811	0.6928	No	0.9722	0.682	0.303	0.496	No	No
96392_at	D4Ertd335e	DNA segment, Chr 4, ERATO Doi 335, expressed	1.0413	0.4362	0.4229	0.792	No	0.9921	0.593	0.0091	0.0091	No	No
96393_at	---	Transcribed locus	1.016	0.4282	0.4069	0.0569	No	1.067	0.362	0.412	0.135	No	No
96394_at	D2Ertd357e	DNA segment, Chr 2, ERATO Doi 357, expressed	1.0315	0.6564	0.4446	0.0924	No	1.0697	0.37	0.226	0.403	No	No
96400_at	---	---	0.9562	0.5949	0.1867	0.9898	No	0.914	0.164	0.0078	0.392	No	No
96408_at	---	---	1.085	0.068	0.0056	0.2161	No	0.9719	0.538	0.0007	0.635	No	No
96413_at	Akap7	A kinase (PRKA) anchor protein 7	1.0292	0.3216	0.0748	0.4788	No	1.5708	0.0144	0.185	0.232	No	No
96414_at	Nsccn1	non-selective cation channel 1	1.024	0.6836	0.088	0.1353	No	1.0516	0.364	0.041	0.0488	No	No
96416_f_at	Hist1h3g /// Hist1h3f /// Hist1h3d /// Hist1h3e /// Hist1h3h /// Hist1h3i	histone 1, H3g /// histone 1, H3f /// histone1, H3d /// histone 1, H3e /// histone 1, H3h /// histone 1, H3i	1.0417	0.1286	0.0005	0.16	No	1.1529	0.0091	0.0189	0.0206	Yes	No
96417_s_at	Hoxb8	homeo box B8	1.0584	0.3612	0.0695	0.306	No	1.0038	0.961	0.392	0.613	No	No
96418_r_at	---	---	1.1506	0.0086	0.171	0.0061	No	1.1726	0.0007	0.008	0.0016	No	No
96419_f_at	Ifna1 /// Ifna11 /// Ifna5 /// Ifna6 /// Ifna7 /// Ifna9 /// Ifnab /// LOC384100	interferon alpha family, gene 1 /// interferon alpha family, gene 11 /// interferon alpha family, gene 5 /// interferon alpha family, gene 6 /// interferon alpha family, gene 7 /// interferon alpha family, gene 9 /// interferon alpha family, gene B /// interferon alpha 8/6 precursor; IFNa8/6	1.0066	0.8024	0.3961	0.0937	No	1.0494	0.484	0.0349	0.444	No	No
96420_at	Itpr2	inositol 1,4,5-triphosphate receptor 2	1.0137	0.7691	0.717	0.1432	No	1.1192	0.0203	0.0021	0.0489	No	No

96421_at	Sult1c1	sulfotransferase family, cytosolic, 1C, member 1	0.9924	0.8628	0.2319	0.5347	No	0.9859	0.958	0.0175	0.277	No	No
96422_at	Nkx2-9	NK2 transcription factor related, locus 9 (Drosophila)	0.9786	0.6763	0.8641	0.2705	No	0.9688	0.55	0.298	0.619	No	No
96423_at	Alx3	aristaless 3	1.0136	0.6374	0.1806	0.2757	No	0.9298	0.251	0.922	0.799	No	No
96424_at	---	---	1.0548	0.3059	0.3657	0.4752	No	0.9897	0.896	0.0415	0.634	No	No
96426_at	Tmsb4x	thymosin, beta 4, X chromosome	1.0508	0.1799	0.0417	0.0711	No	1.0112	0.423	0.264	0.047	No	No
96431_at	D1Ertd83e	DNA segment, Chr 1, ERATO Doi 83, expressed	0.9227	0.2201	0.0483	0.6996	No	0.9206	0.177	0.043	0.36	No	No
96433_at	Usp29	ubiquitin specific peptidase 29	0.8919	0.1407	0.8219	0.7807	No	0.8466	0.106	0.0113	0.586	No	No
96435_at	Ttf1	transcription termination factor 1	0.9862	0.8258	0.1765	0.8221	No	1.0776	0.11	0.0021	0.26	No	No
96464_at	Plxnb2	plexin B2	1.0251	0.6991	0.0995	0.8103	No	1.0156	0.58	0.0327	0.457	No	No
96474_at	Zfp161	zinc finger protein 161	0.9662	0.3751	0.63	0.2349	No	0.9848	0.583	0.151	0.451	No	No
96481_at	C80638	expressed sequence C80638	0.945	0.2618	0.055	0.7482	No	0.9047	0.021	0.544	0.598	No	No
96482_at	---	---	0.9936	0.9201	0.2412	0.8408	No	1.0725	0.211	0.468	0.914	No	No
96483_at	C80113	expressed sequence C80113	1.0192	0.7773	0.4177	0.9897	No	0.9964	0.978	0.378	0.943	No	No
96484_at	C80060	expressed sequence C80060	0.9821	0.6702	0.0335	0.2307	No	1.1229	0.134	0.107	0.0142	No	No
96485_at	---	Transcribed locus	1.0304	0.5144	0.0558	0.6557	No	1.0454	0.572	0.107	0.628	No	No
96486_at	Sntb1	Syntrophin, basic 1	1.1189	0.0986	0.2655	0.572	No	1.1524	0.107	0.0284	0.781	No	No
96487_at	C81072	expressed sequence C81072	1.083	0.0934	0.1839	0.3382	No	1.1873	0.0436	0.364	0.947	No	No
96488_at	---	---	1.0541	0.3924	0.0184	0.5349	No	1.2016	0.37	0.152	0.92	No	No
96489_at	Stt3a	STT3, subunit of the oligosaccharyltransferase complex, homolog A (S. cerevisiae)	1.0246	0.7508	0.4104	0.3157	No	1.1173	0.234	0.207	0.121	No	No
96490_at	Rhbdl3	rhomboid, veinlet-like 3 (Drosophila)	1.101	0.144	0.1943	0.0839	No	1.0772	0.0298	0.0008	0.0038	No	No
96491_at	Zfyve16	zinc finger, FYVE domain containing 16	0.9945	0.8679	0.0132	0.2817	No	0.9858	0.677	0.0773	0.0653	No	No
96492_at	Gm1683	gene model 1683, (NCBI)	1.0023	0.9613	0.0367	0.167	No	0.8991	0.0116	0.0033	0.172	No	No
96493_at	Nek9	NIMA (never in mitosis gene a)-related expressed kinase 9	0.9734	0.5954	0.0313	0.7604	No	0.9857	0.581	0.0017	1	No	No
96494_at	Klhl24	kelch-like 24 (Drosophila)	0.8481	0.0395	0.4104	0.9629	No	0.8748	0.0225	0.712	0.336	No	No
96495_at	Myt1l	myelin transcription factor 1-like	1.0373	0.5426	0.0015	0.4568	No	0.9552	0.118	0.0889	0.487	No	No
96496_g_at	Myt1l	myelin transcription factor 1-like	0.9799	0.6924	0.0912	0.0643	No	1.0191	0.894	0.0044	0.0357	No	No
96497_s_at	Myt1l	myelin transcription factor 1-like	1.0739	0.4151	0.0874	0.9808	No	1.0301	0.75	0.53	0.757	No	No
96498_at	Dmc1h	disrupted meiotic cDNA 1 homolog	1.0769	0.0236	0.0004	0.0435	No	1.0757	0.0058	0.0001	0.0003	No	No
96499_at	AI594671	expressed sequence AI594671	1.0275	0.5498	0.9447	0.4925	No	1.0357	0.66	0.0123	0.382	No	No
96500_at	Gata5	GATA binding protein 5	0.9076	0.1596	0.2195	0.3274	No	1.0175	0.387	0.0039	0.0118	No	No
96501_at	AI194738	expressed sequence AI194738	0.9557	0.3685	0.1187	0.4012	No	0.9809	0.641	0.717	0.308	No	No
96502_at	Phex	phosphate regulating gene with homologies to endopeptidases on the X chromosome (hypophosphatemia, vitamin D resistant rickets)	0.9951	0.8531	0.1834	0.0784	No	1.0016	0.943	0.945	0.498	No	No
96503_at	Krt2-16	keratin complex 2, basic, gene 16	0.8917	0.051	0.1022	0.4084	No	0.9785	0.266	0.0007	0.0399	No	No
96504_at	Pax8	paired box gene 8	1.0208	0.7033	0.029	0.5751	No	0.9746	0.743	0.0519	0.36	No	No
96505_at	Mtag2	metastasis associated gene 2	1.0334	0.4359	0.116	0.7591	No	1.0631	0.27	0.0199	0.135	No	No
96506_at	Alk	anaplastic lymphoma kinase	0.9525	0.1249	0.2884	0.2129	No	1.0014	0.921	0.0013	0.0172	No	No
96507_at	Evx2	even skipped homeotic gene 2 homolog	1.0417	0.2288	0.0943	0.1753	No	0.9879	0.711	0.271	0.634	No	No
96508_at	Ncoa2	nuclear receptor coactivator 2	0.9462	0.043	0.0037	0.0143	No	1.122	0.0669	0.0071	0.227	No	No
96509_at	Ms4a2	membrane-spanning 4-domains, subfamily A, member 2	0.9608	0.2717	0.6881	0.8505	No	0.5463	0.106	0.121	0.219	No	No
96510_at	Angpt1	angiopoietin 1	0.9035	0.0145	0.6612	0.0271	No	0.9555	0.423	0.501	0.29	No	No
96511_s_at	Vav1	vav 1 oncogene	0.9737	0.6236	0.1105	0.3337	No	0.9483	0.197	0.0099	0.414	No	No
96512_at	Fmnl3	formin-like 3	1.0059	0.9121	0.1563	0.5874	No	1.0879	0.0667	0.738	0.0083	No	No
96513_at	AI451896	expressed sequence AI451896	1.0194	0.5264	0.0001	0.0319	No	1.0461	0.174	0.0008	0.247	No	No
96514_at	Esr2	estrogen receptor 2 (beta)	0.994	0.8948	0.5217	0.8385	No	1.1701	0.0831	0.409	0.514	No	No
96515_at	Il4i1	interleukin 4 induced 1	0.8939	0.2673	0.1979	0.2235	No	0.9708	0.562	0.534	0.05	No	No
96516_at	Srgap2	SLIT-ROBO Rho GTPase activating protein 2	0.8904	0.0325	0.0214	0.0063	No	0.9229	0.354	0.302	0.0824	No	No
96517_at	Habp2	hyaluronic acid binding protein 2	1.0925	0.2395	0.0174	0.9928	No	1.0246	0.689	0.222	0.521	No	No
96518_at	Wwc1	WW, C2 and coiled-coil domain containing 1	1.0561	0.291	0.2866	0.2869	No	1.207	0.003	0.0005	0.026	Yes	No
96519_at	Pdxk	pyridoxal (pyridoxine, vitamin B6) kinase	0.9978	0.9534	0.1123	0.3114	No	1.1704	0.0924	0.766	0.359	No	No
96520_at	D9Ertd720e	DNA segment, Chr 9, ERATO Doi 720, expressed	0.9905	0.8042	0.1378	0.2181	No	0.9642	0.618	0.0665	0.241	No	No
96521_at	Mos	Moloney sarcoma oncogene	0.956	0.4604	0.3468	0.6096	No	0.9269	0.0986	0.0127	0.0241	No	No
96522_at	Calm1	calmodulin 1	1.0176	0.7365	0.0237	0.2091	No	1.0465	0.535	0.0132	0.23	No	No
96523_at	En1	engrailed 1	1.1047	0.0577	0.1103	0.0421	No	1.1969	0.004	0.827	0.0058	No	No
96524_at	Cdr2l	cerebellar degeneration-related protein 2-like	1.028	0.1997	0.0841	0.2835	No	1.0333	0.281	0.95	0.322	No	No
96525_at	Il10ra	interleukin 10 receptor, alpha	0.9902	0.8626	0.0378	0.032	No	1.136	0.0892	0.0099	0.0328	No	No
96526_at	D030029J20Rik	RIKEN cDNA D030029J20 gene	0.943	0.5316	0.0006	0.5291	No	0.8838	0.189	0.0008	0.651	No	No
96527_at	---	---	0.9539	0.4941	0.1073	0.8745	No	0.9904	0.681	0.004	0.178	No	No
96528_at	Arl4a	ADP-ribosylation factor-like 4A	1.0147	0.8394	0.1485	0.8069	No	1.209	0.0592	0.0349	0.0509	No	No
96529_at	Ap1gbp1	AP1 gamma subunit binding protein 1	0.966	0.4807	0.002	0.1259	No	1.0599	0.0186	0.0002	0.0055	No	No
96530_at	---	Transcribed locus	0.97	0.4369	0.9444	0.1932	No	0.9695	0.576	0.776	0.43	No	No
96531_at	Tbl3	transducin (beta)-like 3	1.0707	0.2246	0.1697	0.316	No	1.1298	0.047	0.426	0.262	No	No
96532_at	Ddx50	DEAD (Asp-Glu-Ala-Asp) box polypeptide 50	0.9708	0.3437	0	0.5557	No	0.9838	0.469	0	0.0163	No	No
96533_at	---	---	0.9549	0.34	0.0123	0.9462	No	1.1332	0.0837	0.668	0.122	No	No
96534_at	Vldlr	very low density lipoprotein receptor	0.9769	0.3998	0.0006	0.639	No	0.9987	0.708	0.008	0.0633	No	No
96535_at	Zfp499	zinc finger protein 499	1.0049	0.8389	0.871	0.591	No	0.9968	0.926	0.21	0.917	No	No
96536_at	---	---	1.0657	0.3172	0.348	0.5775	No	0.941	0.449	0.1	0.438	No	No
96537_at	Slurp1	secreted Ly6/Plaur domain containing 1	0.9896	0.8499	0.0205	0.7049	No	1.1217	0.135	0.178	0.0863	No	No
96538_at	Centb1	centaurin, beta 1	1.0103	0.8629	0.006	0.0437	No	1.0685	0.354	0.0241	0.186	No	No
96539_at	Serbp1	Serpine1 mRNA binding protein 1	0.9802	0.5476	0.0425	0.003	No	1.0194	0.383	0.0265	0.0017	No	No
96540_at	---	---	0.9643	0.5188	0.5658	0.1134	No	0.904	0.0314	0.0614	0.117	No	No
96541_at	AA408420	expressed sequence AA408420	0.9039	0.0289	0.1269	0.0922	No	0.9566	0.444	0.0605	0.33	No	No
96542_at	Surf4	surfeit gene 4	1.0267	0.3299	0.0009	0.18	No	1.0078	0.729	0.0185	0.0919	No	No
96543_at	---	0 day neonate thymus cDNA, RIKEN full-length enriched library, clone:A430079C03 product:unclassifiable, full insert sequence	0.9644	0.4183	0.6935	0.9099	No	1.0333	0.659	0.291	0.367	No	No
96544_at	AA672641	expressed sequence AA672641	1.0196	0.5932	0.0157	0.4413	No	1.1827	0.0422	0.888	0.0878	No	No
96545_s_at	Pcid2	PCI domain containing 2	1.0757	0.4762	0.0233	0.7103	No	1.0592	0.289	0.0062	0.813	No	No
96546_r_at	Pcid2	PCI domain containing 2	1.0331	0.2738	0.4751	0.8961	No	0.982	0.416	0.0047	0.0344	No	No
96547_at	Tmod1	Tropomodulin 1	1.0323	0.3478	0.0112	0.0094	No	1.0776	0.105	0.0249	0.0192	No	No
96548_at	B4galnt2	beta-1,4-N-acetyl-galactosaminyl transferase 2	0.8854	0.0162	0.0109	0.0075	No	0.9055	0.433	0.999	0.301	No	No
96549_at	Chrnd	cholinergic receptor, nicotinic, delta polypeptide	0.9403	0.2151	0.0524	0.6155	No	1.0134	0.864	0.349	0.93	No	No
96550_at	C1galt1c1	C1GALT1-specific chaperone 1	0.972	0.5986	0.4772	0.5948	No	0.9825	0.581	0.315	0.782	No	No
96551_at	Clec4e	C-type lectin domain family 4, member e	0.9851	0.853	0.3163	0.9605	No	0.996	0.933	0.0483	0.946	No	No
96552_at	Wdr75	WD repeat domain 75	0.9582	0.4532	0.1916	0.5418	No	0.9986	0.993	0.0511	0.708	No	No
96553_at	Gpr65	G-protein coupled receptor 65	0.9956	0.9453	0.1469	0.3561	No	0.946	0.241	0.107	0.183	No	No
96554_r_at	Taf15	TAF15 RNA polymerase II, TATA box binding protein (TBP)-associated factor	0.8631	0.1216	0.6217	0.9596	No	0.922	0.0325	0.0003	0.187	No	No
96555_at	---	---	0.9853	0.7155	0.2411	0.4742	No	1.0618	0.483	0.363	0.992	No	No
96556_at	Kalrn	kalirin, RhoGEF kinase	0.8741	0.1292	0.7704	0.2369	No	1.0568	0.304	0.0076	0.0444	No	No
96557_at	Ptch2	patched homolog 2	1.0391	0.6256	0.1807	0.8686	No	1.1305	0.0995	0.0187	0.5	No	No
96558_at	Mutyh	mutY homolog (E. coli)	1.0117	0.4814	0.0096	0.008	No	0.9919	0.821	0.329	0.989	No	No
96559_at	AA536717	Expressed sequence AA536717	1.1455	0.0954	0.6446	0.3215	No	1.0234	0.738	0.168	0.861	No	No
96560_at	Myo6	myosin VI	1.0919	0.0208	0.002	0.007	No	1.0913	0.0328	0.0559	0.0071	No	No
96561_at	Nfatc2ip	nuclear factor of activated T-cells, cytoplasmic, calcineurin-dependent 2 interacting protein	1.0188	0.7125	0.0017	0.9066	No	1.0475	0.542	0.0085	0.764	No	No
96562_at	Slc11a1	solute carrier family 11 (proton-coupled divalent metal ion transporters), member 1	0.9578	0.2977	0.9941	0.1763	No	0.9632	0.467	0.256	0.306	No	No
96563_at	Usp24	ubiquitin specific peptidase 24	0.9387	0.0311	0.9516	0.0671	No	0.9719	0.56	0.0742	0.207	No	No
96564_at	Hspa8 /// LOC621284 /// LOC666904 /// LOC669493	heat shock protein 8 /// similar to heat shock protein 8 /// similar to heat shock protein 8 /// similar to heat shock protein 8	1.0636	0.457	0.134	0.4771	No	1.0375	0.458	0.801	0.915	No	No
96565_at	Nvl	Nuclear VCP-like	0.9404	0.3059	0.0601	0.5558	No	0.9347	0.0272	0.0016	0.0988	No	No
96566_at	Tgm3	transglutaminase 3, E polypeptide	0.9792	0.7406	0.5253	0.6915	No	1.0302	0.722	0.743	0.395	No	No
96567_at	Rho	rhodopsin	0.918	0.0523	0.0737	0.6777	No	0.9713	0.551	0.386	0.422	No	No
96568_at	Cntn3	contactin 3	0.969	0.0882	0.003	0.0645	No	0.918	0.0461	0.0129	0.0836	No	No
96569_at	Fignl1	fidgetin-like 1	1.0254	0.4558	0.0114	0.0218	No	1.0368	0.236	0.012	0.0409	No	No
96570_at	Cdc73	Vcell division cycle 73, Paf1/RNA polymerase II complex component, homolog (S. cerevisiae)	1.0016	0.9815	0.0046	0.0419	No	1.0494	0.384	0.0373	0.0577	No	No
96571_at	Prkcz	protein kinase C, zeta	1.0069	0.6759	0.0021	0.2885	No	1.0252	0.539	0.4	0.274	No	No
96572_at	Azi2	5-azacytidine induced gene 2	0.961	0.2094	0.0039	0.8935	No	0.9717	0.605	0.0987	0.856	No	No
96573_at	Actg1	actin, gamma, cytoplasmic 1	1.0798	0.1585	0.0282	0.0584	No	1.0341	0.441	0.068	0.238	No	No
96574_at	Il9	interleukin 9	1.0343	0.6422	0.4208	0.4195	No	1.0614	0.508	0.0048	0.865	No	No
96575_at	Rpl8	ribosomal protein L8	1.0728	0.0417	0.0093	0.1056	No	1.0316	0.208	0.0155	0.175	No	No

96576_at	Phgdhl1	Phosphoglycerate dehydrogenase like 1	1.0339	0.4079	0.7844	0.7971	No	1.0956	0.086	0.152	0.407	No	No
96577_i_at	Ubap2l	ubiquitin associated protein 2-like	0.9244	0.3116	0.0055	0.361	No	0.9576	0.326	0.127	0.49	No	No
96578_r_at	Ubap2l	ubiquitin associated protein 2-like	1.0769	0.1239	0.0005	0.4242	No	1.0035	0.875	0.0004	0.712	No	No
96579_at	Sass6	spindle assembly 6 homolog (C. elegans)	0.9982	0.9767	0.0166	0.8722	No	0.985	0.841	0.005	0.445	No	No
96580_at	Pbx3	pre B-cell leukemia transcription factor 3	0.9882	0.79	0.9983	0.7046	No	0.9653	0.316	0.0707	0.212	No	No
96581_at	Ncam2	neural cell adhesion molecule 2	1.0326	0.3157	0.0149	0.0966	No	0.98	0.706	0.001	0.279	No	No
96582_at	Ncam2	neural cell adhesion molecule 2	0.955	0.2976	0.9979	0.8384	No	1.0287	0.361	0.009	0.0375	No	No
96583_s_at	Kif5a /// Kif5c	kinesin family member 5A /// kinesin family member 5C	1.0262	0.5989	0.4217	0.7334	No	1.0902	0.0865	0.0083	0.768	No	No
96584_f_at	Tcstv3	2-cell-stage, variable group, member 3	0.9975	0.9523	0.1723	0.1725	No	0.8896	0.0394	0.0228	0.185	No	No
96585_at	Avpr2	arginine vasopressin receptor 2	0.9776	0.6211	0.24	0.7663	No	0.9452	0.252	0.112	0.289	No	No
96586_at	Crygc	crystallin, gamma C	0.9173	0.1184	0.2736	0.3965	No	1.0763	0.175	0.11	0.248	No	No
96587_at	Arf3	ADP-ribosylation factor 3	0.8402	0.1336	0.525	0.678	No	0.931	0.35	0.215	0.939	No	No
96588_at	Ptger3	prostaglandin E receptor 3 (subtype EP3)	1.0214	0.6458	0.5368	0.1312	No	1.0868	0.0568	0.165	0.478	No	No
96589_at	Ptger3	prostaglandin E receptor 3 (subtype EP3)	1.0342	0.5065	0.0088	0.4519	No	1.2003	0.135	0.011	0.791	No	No
96590_f_at	Zq20d1	zinc finger, A20 domain containing 1	0.9241	0.0109	0.01	0.6422	No	1.0753	0.16	0.0299	0.455	No	No
96591_at	Reln	reelin	1.0457	0.5035	0.0034	0.0869	No	1.0804	0.0243	0.0006	0.0029	No	No
96592_at	Pik3r1	phosphatidylinositol 3-kinase, regulatory subunit, polypeptide 1 (p85 alpha)	0.9034	0.1566	0.1772	0.986	No	0.9171	0.122	0.0085	0.144	No	No
96593_at	Elk1	ELK1, member of ETS oncogene family	0.9538	0.3247	0.0782	0.3689	No	1.0146	0.752	0.794	0.518	No	No
96594_at	---	---	1.1026	0.0342	0.0001	0.0403	No	1.042	0.127	0.0004	0.0181	No	No
96595_at	Pax1	paired box gene 1	1.0363	0.4217	0.0106	0.7264	No	1.0472	0.328	0.692	0.171	No	No
96596_at	Ndrg1 /// Ndrl	N-myc downstream regulated gene 1 /// N-myc downstream regulated-like	0.8953	0.15	0.8377	0.8369	No	0.841	0.0063	0.0022	0.362	Yes	No
96597_at	Pet2	plasmacytoma expressed transcript 2	0.9845	0.7546	0.9636	0.4224	No	1.0373	0.675	0.117	0.177	No	No
96598_at	Ccnj	cyclin J	1.0431	0.6247	0.0179	0.7022	No	1.0379	0.324	0.0004	0.432	No	No
96599_at	C330016K18 Rik	RIKEN cDNA C330016K18 gene	1.0144	0.4693	0	0.1058	No	1.0352	0.519	0.0001	0.692	No	No
96600_at	Arhgap29	Rho GTPase activating protein 29	0.9876	0.5388	0.006	0.0263	No	0.9149	0.311	0.119	0.729	No	No
96601_at	Qscn6	quiescin Q6	1.033	0.6162	0.0037	0.8151	No	0.9033	0.226	0.031	0.912	No	No
96602_g_at	Qscn6	quiescin Q6	1.0142	0.7526	0.0493	0.5649	No	1.0062	0.918	0.014	0.39	No	No
96603_at	Qscn6	quiescin Q6	0.9152	0.1882	0.3368	0.0722	No	1.1403	0.134	0.0031	0.0379	No	No
96604_at	Dus1l	dihydrouridine synthase 1-like (S. cerevisiae)	0.9244	0.1408	0.1313	0.1428	No	0.9695	0.147	0.0032	0.0177	No	No
96605_at	0610011I04 Rik	RIKEN cDNA 0610011I04 gene	0.9054	0.0627	0.0048	0.1264	No	0.9109	0.123	0.0043	0.167	No	No
96606_at	1500003O03 Rik	RIKEN cDNA 1500003O03 gene	0.943	0.0533	0.0001	0.8973	No	0.9325	0.109	0.0008	0.831	No	No
96607_at	1500003O03 Rik	RIKEN cDNA 1500003O03 gene	0.9428	0.0475	0.6005	0.1216	No	1.0049	0.517	0.0021	0.0054	No	No
96608_at	Phyh	phytanoyl-CoA hydroxylase	0.867	0.0456	0.0549	0.2072	No	0.8915	0.0011	0.0004	0.0833	Yes	Yes
96609_at	Sfrs11	splicing factor, arginine/serine-rich 11	1.042	0.4293	0.0037	0.2959	No	1.0036	0.932	0.0123	0.832	No	No
96610_at	Atp6v1h	ATPase, H+ transporting, lysosomal V1 subunit H	0.9828	0.1562	0.002	0.002	No	1.0054	0.79	0.0254	0.102	No	No
96611_at	Ndufa11	NADH dehydrogenase (ubiquinone) 1 alpha subcomplex 11	1.002	0.9626	0.128	0.1712	No	1.0131	0.73	0.087	0.135	No	No
96613_at	5730536A07 Rik	RIKEN cDNA 5730536A07 gene	1.0362	0.6009	0.8665	0.8386	No	1.0477	0.554	0.998	0.983	No	No
96614_at	4933426M11 Rik	RIKEN cDNA 4933426M11 gene	0.912	0.1372	0.4149	0.9471	No	1.0532	0.408	0.0437	0.205	No	No
96615_at	Ypel3	yippee-like 3 (Drosophila)	0.948	0.1248	0.0047	0.6651	No	0.9125	0.0295	0.354	0.228	No	No
96616_at	Atad1	ATPase family, AAA domain containing 1	1.0331	0.5877	0.1112	0.626	No	0.9413	0.365	0.0193	0.455	No	No
96617_at	Drap1	Dr1 associated protein 1 (negative cofactor 2 alpha)	0.9911	0.8624	0.0296	0.3867	No	1.0319	0.476	0.003	0.697	No	No
96618_at	Rpp21	ribonuclease P 21 subunit (human)	1.0407	0.2074	0.0054	0.0536	No	1.1004	0.0123	0.0224	0.0005	No	No
96619_at	Abcf3	ATP-binding cassette, sub-family F (GCN20), member 3	1.0463	0.2919	0.0104	0.1778	No	1.0718	0.223	0.0842	0.159	No	No
96620_at	Gsg1	germ cell-specific gene 1	1.0781	0.2848	0.0483	0.4091	No	1.0583	0.397	0.0176	0.144	No	No
96621_at	Nop17	NOP17	0.9728	0.593	0.0296	0.3654	No	1.0005	0.991	0.0177	0.785	No	No
96623_at	Ugcg	UDP-glucose ceramide glucosyltransferase	1.0145	0.8498	0.015	0.6515	No	0.9309	0.437	0.0297	0.306	No	No
96624_r_at	Eef1b2	Eukaryotic translation elongation factor 1 beta 2	0.9782	0.152	0.0001	0.0016	No	1.076	0.268	0.0177	0.0295	No	No
96625_at	Wdhd1	WD repeat and HMG-box DNA binding protein 1	1.1674	0.0096	0.0193	0.0165	Yes	1.1427	0.0119	0.0041	0.0146	No	Yes
96626_at	Tufm /// LOC669832	Tu translation elongation factor, mitochondrial /// similar to Tu translation elongation factor, mitochondrial	0.9484	0.3448	0.9978	0.2177	No	0.9633	0.416	0.0071	0.255	No	No
96627_at	Ebp	phenylalkylamine Ca2+ antagonist (emopamil) binding protein	1.0265	0.707	0.1633	0.4209	No	0.9982	0.999	0.576	0.0912	No	No
96628_at	Eprs /// LOC633677	glutamyl-prolyl-tRNA synthetase /// similar to Bifunctional aminoacyl-tRNA synthetase	1.1578	0.0508	0.0049	0.5076	No	1.0481	0.515	0.161	0.672	No	No
96629_at	Nudt19	nudix (nucleoside diphosphate linked moiety X)-type motif 19	1.092	0.0873	0.0009	0.1009	No	1.0585	0.414	0.0129	0.507	No	No
96630_at	Spink3	serine peptidase inhibitor, Kazal type 3	1.0864	0.0533	0.6068	0.8444	No	1.1026	0.204	0.195	0.26	No	No
96632_at	Morf4l2 /// LOC433261 /// LOC677515	mortality factor 4 like 2 /// similar to Mortality factor 4-like protein 2 (MORF-related gene X protein) (Transcription factor-like protein MRGX) (Sid 393) /// similar to Mortality factor 4-like protein 2 (MORF-related gene X protein) (Transcription factor-like protein MRGX) (Sid 393)	1.0526	0.2187	0.0909	0.2332	No	1.0194	0.555	0.551	0.271	No	No
96633_s_at	Morf4l2	mortality factor 4 like 2	1.1131	0.1017	0.8409	0.4577	No	1.0434	0.446	0.223	0.944	No	No
96634_at	5730469M10 Rik	RIKEN cDNA 5730469M10 gene	0.9672	0.2728	0.0004	0.0984	No	1.0407	0.495	0.0114	0.49	No	No
96635_at	---	---	0.9968	0.841	0.0179	0.1177	No	1.0099	0.341	0.0003	0.0186	No	No
96636_at	Dctn5	dynactin 5	0.9285	0.1899	0.9582	0.0603	No	0.9652	0.149	0.152	0.0229	No	No
96637_at	Tbc1d1	TBC1 domain family, member 1	1.0066	0.8682	0.0348	0.703	No	0.9726	0.533	0.284	0.346	No	No
96638_at	Ap2s1	Adaptor-related protein complex 2, sigma 1 subunit	1.0399	0.1356	0.0138	0.2325	No	1.204	0.0268	0.0045	0.0576	No	No
96639_at	Ap2s1	Adaptor-related protein complex 2, sigma 1 subunit	0.9634	0.5133	0.9204	0.2358	No	0.9534	0.589	0.331	0.213	No	No
96640_at	3110001A13 Rik	RIKEN cDNA 3110001A13 gene	1.0391	0.2595	0.7488	0.0683	No	1.0366	0.192	0.0508	0.0216	No	No
96641_at	Parl	presenilin associated, rhomboid-like	0.9949	0.8804	0.8768	0.038	No	0.9653	0.42	0.641	0.0551	No	No
96643_at	Wfdc2	WAP four-disulfide core domain 2	1.0613	0.0673	0.0003	0.0053	No	1.1079	0.0654	0.0026	0.324	No	No
96644_at	Bysl	bystin-like	1.0166	0.7298	0.1879	0.2793	No	1.0096	0.642	0.0022	0.125	No	No
96646_at	Usp39	ubiquitin specific peptidase 39	0.9874	0.3679	0.0009	0.0872	No	1.0003	0.875	0.0043	0.214	No	No
96647_at	Dcun1d5	DCN1, defective in cullin neddylation 1, domain containing 5 (S. cerevisiae)	1.1757	0.0244	0.8849	0.9227	No	1.0477	0.182	0.0053	0.981	No	No
96648_at	Coro1a	coronin, actin binding protein 1A	0.9381	0.2857	0.2733	0.103	No	0.907	0.0185	0.0459	0.327	No	No
96649_at	Txndc14	thioredoxin domain containing 14	1.0102	0.7316	0.0016	0.4409	No	1.0428	0.328	0.0056	0.365	No	No
96650_at	Auh	AU RNA binding protein/enoyl-coenzyme A hydratase	0.8897	0.005	0.2364	0.0021	No	0.9539	0.418	0.826	0.531	No	No
96651_at	Smarce1	SWI/SNF related, matrix associated, actin dependent regulator of chromatin, subfamily e, member 1	1.0476	0.0617	0.7674	0.0224	No	1.0929	0.0368	0.167	0.581	No	No
96652_at	Mrpl28	mitochondrial ribosomal protein L28	0.9819	0.6408	0.3521	0.034	No	1.0155	0.572	0.107	0.0085	No	No
96653_at	Rnaset2	ribonuclease T2	1.1126	0.2012	0.6224	0.4686	No	1.1075	0.167	0.0321	0.475	No	No
96654_at	Wdr48	WD repeat domain 48	0.9423	0.2195	0.0055	0.4432	No	0.8862	0.108	0.109	0.14	No	No
96655_g_at	Wdr48	WD repeat domain 48	0.9368	0.0974	0.6151	0.1532	No	0.9492	0.365	0.679	0.966	No	No
96656_at	Wdr48	WD repeat domain 48	0.9635	0.6801	0.5622	0.413	No	0.9942	0.951	0.772	0.522	No	No
96657_at	Sat1	spermidine/spermine N1-acetyl transferase 1	0.8634	0.0244	0.2144	0.3418	No	0.7756	0.0024	0.008	0.031	Yes	Yes
96658_at	2900010J23 Rik	RIKEN cDNA 2900010J23 gene	0.9369	0.3902	0.0079	0.0557	No	0.9131	0.465	0.0096	0.194	No	No
96661_at	Nrd1	nardilysin, N-arginine dibasic convertase, NRD convertase 1	0.9675	0.4464	0.1009	0.3225	No	1.0147	0.832	0.444	0.899	No	No
96662_at	Ppap2b	phosphatidic acid phosphatase type 2B	1.1089	0.1414	0.0999	0.3707	No	1.0193	0.664	0.0277	0.454	No	No
96663_at	Surf6	surfeit gene 6	1.0198	0.7422	0.0039	0.0319	No	1.1341	0.0364	0.0007	0.0118	No	No
96664_at	Huwe1	HECT, UBA and WWE domain containing 1	1.0515	0.0447	0.0011	0.0024	No	1.0501	0.222	0.0704	0.155	No	No
96665_at	Vps26a	vacuolar protein sorting 26 homolog A (yeast)	1.0033	0.9157	0.029	0.8944	No	0.9892	0.808	0.0489	0.649	No	No
96666_at	Ntan1	N-terminal Asn amidase	1.2471	0.0507	0.1361	0.3955	No	1.0011	0.986	0.0008	0.905	No	No
96667_at	Vps41	vacuolar protein sorting 41 (yeast)	1.0285	0.7197	0.4435	0.2766	No	0.9648	0.615	0.639	0.218	No	No
96668_at	Timm17b	translocase of inner mitochondrial membrane 17b	1.0393	0.0552	0.0019	0.0221	No	1.041	0.0336	0.0249	0.016	No	No
96669_at	2400003C14 Rik	RIKEN cDNA 2400003C14 gene	0.9658	0.2702	0.1336	0.3882	No	0.9665	0.258	0.0048	0.439	No	No
96670_at	Gstk1	glutathione S-transferase kappa 1	0.9071	0.004	0.0207	0.0047	No	0.9113	0.108	0.163	0.0406	No	No
96672_at	Hod	homeobox only domain	1.0266	0.6779	0.7444	0.4091	No	0.9427	0.177	0.979	0.571	No	No
96674_at	Tnpo3	transportin 3	1.0298	0.4643	0.0074	0.3785	No	1.0875	0.241	0.0254	0.909	No	No
96675_at	Unc50	unc-50 homolog (C. elegans)	1.0446	0.261	0.0059	0.6357	No	1.0103	0.751	0.0077	0.615	No	No
96676_at	Chchd7	coiled-coil-helix-coiled-coil-helix domain containing 7	0.941	0.377	0.0054	0.1133	No	0.9719	0.736	0.0037	0.12	No	No

96677_at	Setd8	SET domain containing (lysine methyltransferase) 8	0.9167	0.1196	0.0108	0.9199	No	1.0451	0.319	0.0904	0.64	No	No
96678_at	Dhrs4	dehydrogenase/reductase (SDR family) member 4	1.0231	0.3028	0.0006	0.1409	No	1.0211	0.334	0.0001	0.854	No	No
96679_at	Dnajb9	DnaJ (Hsp40) homolog, subfamily B, member 9	0.8294	0.0033	0.0007	0.0036	No	0.8176	0.0025	0.0009	0.0112	Yes	No
96680_at	Dnajb9	DnaJ (Hsp40) homolog, subfamily B, member 9	1.0409	0.6778	0.0169	0.6524	No	0.9473	0.538	0.0463	0.195	No	No
96682_at	St6galnac4	ST6 (alpha-N-acetyl-neuraminyl-2,3-beta-galactosyl-1,3)-N-acetylgalactosaminide alpha-2,6-sialyltransferase 4	0.989	0.7264	0.1077	0.0962	No	1.029	0.0157	0.0001	0.848	No	No
96684_at	Grsf1	G-rich RNA sequence binding factor 1	0.9401	0.3548	0.1945	0.4583	No	0.9214	0.197	0.714	0.0426	No	No
96685_at	1110002E23 Rik /// Gm939	RIKEN cDNA 1110002E23 gene /// gene model 939, (NCBI)	0.9682	0.4354	0.2793	0.1968	No	0.9895	0.764	0.59	0.43	No	No
96686_i_at	2010100O12 Rik	RIKEN cDNA 2010100O12 gene	1.0539	0.3311	0.0185	0.0245	No	0.9882	0.764	0.0125	0.012	No	No
96687_f_at	2010100O12 Rik /// LOC624701	RIKEN cDNA 2010100O12 gene /// similar to CG6878-PA	1.0301	0.7007	0.4442	0.04	No	1.1112	0.277	0.514	0.0702	No	No
96688_at	Tmem77	transmembrane protein 77	0.9818	0.6143	0.0001	0.0339	No	1.0657	0.239	0.0006	0.485	No	No
96691_at	Taf11	TAF11 RNA polymerase II, TATA box binding protein (TBP)-associated factor	1.0147	0.7874	0.0606	0.712	No	1.0043	0.819	0.018	0.198	No	No
96692_at	Fgfr1op2	FGFR1 oncogene partner 2	1.0107	0.7737	0.4383	0.8441	No	0.9392	0.0569	0.176	0.287	No	No
96693_at	Rars	arginyl-tRNA synthetase	1.0245	0.5359	0.1062	0.3344	No	0.9839	0.615	0.0022	0.443	No	No
96694_at	Tcl1b1	T-cell leukemia/lymphoma 1B, 1	1.0931	0.3028	0.9045	0.8039	No	1.0898	0.175	0.0384	0.162	No	No
96695_at	Ube2a	ubiquitin-conjugating enzyme E2A, RAD6 homolog (S. cerevisiae)	0.9112	0.0367	0.0573	0.2797	No	0.9434	0.263	0.0299	0.556	No	No
96696_at	Prmt1	protein arginine N-methyltransferase 1	1.2311	0.0686	0.4668	0.7219	No	1.1111	0.362	0.259	0.3	No	No
96698_at	Psmd5	proteasome (prosome, macropain) 26S subunit, non-ATPase, 5	0.9877	0.6156	0.0026	0.044	No	1.0159	0.288	0.0003	0.0351	No	No
96699_at	Hmgn1	high mobility group nucleosomal binding domain 1	1.013	0.7521	0.0106	0.4589	No	0.9915	0.896	0.0302	0.66	No	No
96700_r_at	Rap1b	RAS related protein 1b	1.077	0.275	0.1289	0.1355	No	1.1168	0.171	0.9	0.394	No	No
96701_at	Uble1b	ubiquitin-like 1 (sentrin) activating enzyme E1B	1.1562	0.0896	0.0153	0.5454	No	1.0912	0.188	0.01	0.411	No	No
96703_at	Maged1	melanoma antigen, family D, 1	0.9901	0.7708	0.0358	0.8284	No	0.9956	0.856	0	0.0208	No	No
96704_at	Sfn	stratifin	0.8864	0.0311	0.0008	0.2487	No	0.8792	0.0014	0.0001	0.038	Yes	Yes
96707_at	Zipro1	zinc finger proliferation 1	1.0168	0.5741	0.0065	0.0343	No	1.0092	0.638	0.0041	0.117	No	No
96708_at	Tmed3	transmembrane emp24 domain containing 3	1.1109	0.1507	0.008	0.5608	No	1.0389	0.368	0.0002	0.524	No	No
96709_at	1110008P14 Rik	RIKEN cDNA 1110008P14 gene	1.0891	0.1052	0.1131	0.6498	No	1.0327	0.456	0.72	0.296	No	No
96710_at	H2afv	H2A histone family, member V	1.1691	0.0314	0.1206	0.2972	No	1.1099	0.182	0.967	0.231	No	No
96711_at	Znrd1	zinc ribbon domain containing, 1	1.0243	0.6582	0.9984	0.4424	No	0.987	0.799	0.961	0.13	No	No
96712_at	Smoc1	SPARC related modular calcium binding 1	1.0111	0.6316	0.229	0.3207	No	1	0.994	0.143	0.763	No	No
96713_at	Papss2	3'-phosphoadenosine 5'-phosphosulfate synthase 2	0.8061	0.0112	0.0042	0.2867	No	0.8682	0.0914	0.0025	0.701	No	No
96716_at	1110003E01 Rik	RIKEN cDNA 1110003E01 gene	1.0295	0.0315	0.0001	0.022	No	1.0831	0.147	0.0331	0.698	No	No
96717_at	Ddx47	DEAD (Asp-Glu-Ala-Asp) box polypeptide 47	0.921	0.038	0.0063	0.5613	No	1.008	0.997	0.376	0.0623	No	No
96718_at	Mtvr2	mammary tumor virus receptor 2	0.9966	0.8482	0.0007	0.1166	No	1.0186	0.299	0.0024	0.138	No	No
96719_i_at	Pvalb	parvalbumin	1.0619	0.1847	0.0466	0.0661	No	1.0169	0.479	0.0142	0.0009	No	No
96720_f_at	Pvalb	parvalbumin	1.1238	0.0011	0.0051	0.0074	No	1.1021	0.0794	0.0169	0.037	No	No
96722_at	Phkb	phosphorylase kinase beta	1.0594	0.3566	0.0779	0.8932	No	0.8713	0.0869	0.0122	0.976	No	No
96723_f_at	Ssx2ip	synovial sarcoma, X breakpoint 2 interacting protein	1.1154	0.0814	0.001	0.0737	No	1.0939	0.0037	0	0.0143	Yes	No
96724_r_at	Ssx2ip	synovial sarcoma, X breakpoint 2 interacting protein	1.0292	0.1445	0	0.0118	No	0.9967	0.705	0.0002	0.0973	No	No
96725_at	Cic	capicua homolog (Drosophila)	0.9981	0.9577	0.5105	0.3432	No	0.9519	0.171	0.936	0.0293	No	No
96726_at	Cdk8	cyclin-dependent kinase 8	1.0768	0.4013	0.0119	0.9049	No	1.0366	0.126	0.0001	0.0303	No	No
96728_at	Wdr45	WD repeat domain 45	0.8931	0.0556	0.3356	0.7188	No	0.9973	0.984	0.0614	0.678	No	No
96729_at	Eif4ebp2	eukaryotic translation initiation factor 4E binding protein 2	1.0084	0.8438	0.0494	0.0932	No	1.1042	0.0808	0.0263	0.0061	No	No
96730_at	Tpp2	tripeptidyl peptidase II	0.9095	0.0618	0.0556	0.0816	No	0.9618	0.441	0.031	0.572	No	No
96731_at	1110001P04 Rik	RIKEN cDNA 1110001P04 gene	0.8915	0.2161	0.5971	0.9865	No	0.9274	0.18	0.0907	0.622	No	No
96732_at	Wdr40a	WD repeat domain 40A	0.9283	0.0875	0.0878	0.4965	No	0.961	0.25	0.892	0.721	No	No
96733_at	Rap1gds1	RAP1, GTP-GDP dissociation stimulator 1	0.9693	0.4442	0.564	0.0306	No	0.9809	0.224	0.304	0.0847	No	No
96734_at	Synj2bp	synaptojanin 2 binding protein	0.9932	0.7805	0.0007	0.2409	No	1.0518	0.242	0.0017	0.325	No	No
96735_at	Stard10	START domain containing 10	0.9587	0.2322	0.0129	0.0475	No	0.9809	0.742	0.121	0.0536	No	No
96736_at	Lpp	LIM domain containing preferred translocation partner in lipoma	0.9237	0.1927	0.0005	0.0719	No	0.8871	0.144	0.0004	0.117	No	No
96737_at	Tbc1d22a	TBC1 domain family, member 22a	0.9694	0.3236	0.3061	0.176	No	0.9704	0.263	0.886	0.46	No	No
96738_at	Adam9	a disintegrin and metallopeptidase domain 9 (meltrin gamma)	1.0409	0.1352	0.0001	0.0029	No	1.0054	0.708	0.0162	0.197	No	No
96739_at	Ufm1	Ubiquitin-fold modifier 1	1.1355	0.0782	0.0026	0.8038	No	1.1521	0.0599	0.0002	0.0466	No	No
96741_at	Phf12	PHD finger protein 12	1.1356	0.2319	0.0094	0.1478	No	1.1105	0.139	0.0101	0.38	No	No
96742_at	Dpt	dermatopontin	0.8493	0.0272	0.0533	0.022	No	0.8094	0.0055	0.0115	0.0079	No	No
96743_at	1810035L17 Rik	RIKEN cDNA 1810035L17 gene	1.0222	0.8031	0.5572	0.3864	No	1.0471	0.467	0.0287	0.188	No	No
96744_at	Acp6	acid phosphatase 6, lysophosphatidic	1.0324	0.6522	0.1845	0.7643	No	1.0189	0.697	0.492	0.203	No	No
96745_at	Dlat	dihydrolipoamide S-acetyltransferase (E2 component of pyruvate dehydrogenase complex)	1.0718	0.0934	0.0167	0.0163	No	1.1707	0.03	0.0063	0.0641	No	No
96746_at	Dlat	dihydrolipoamide S-acetyltransferase (E2 component of pyruvate dehydrogenase complex)	0.9131	0.119	0.0278	0.6699	No	0.9483	0.256	0.0004	0.296	No	No
96747_at	Rhou	ras homolog gene family, member U	1.0007	0.9865	0.3208	0.2078	No	0.9246	0.036	0.0325	0.184	No	No
96748_i_at	Tmem30b	transmembrane protein 30B	1.2004	0.0683	0.0881	0.456	No	1.1635	0.0999	0.0395	0.734	No	No
96749_f_at	Tmem30b	transmembrane protein 30B	1.3738	0.0937	0.0068	0.3752	No	1.1552	0.357	0.0289	0.289	No	No
96750_at	Ilkap	integrin-linked kinase-associated serine/threonine phosphatase 2C	0.9413	0.4676	0.1696	0.5531	No	0.9772	0.542	0.576	0.149	No	No
96751_at	Arl5a	ADP-ribosylation factor-like 5A	0.9739	0.6812	0.0045	0.6861	No	1.0135	0.712	0.0014	0.546	No	No
96752_at	Icam1	intercellular adhesion molecule	1.0453	0.2094	0.6027	0.6009	No	1.0847	0.187	0.451	0.675	No	No
96753_at	Bcl7c	B-cell CLL/lymphoma 7C	1.0521	0.269	0.0103	0.2827	No	0.9132	0.0169	0	0.0114	No	No
96754_s_at	---	Transcribed locus	0.9817	0.5431	0.0006	0.9251	No	0.9927	0.988	0.0008	0.186	No	No
96755_at	Etf1	Eukaryotic translation termination factor 1	1.0202	0.3581	0.078	0.1896	No	1.0481	0.248	0.459	0.0626	No	No
96756_at	1110007M04 Rik	RIKEN cDNA 1110007M04 gene	0.9394	0.2271	0.1994	0.7616	No	0.9365	0.0199	0.0069	0.801	No	No
96757_at	D10Jhu81e	DNA segment, Chr 10, Johns Hopkins University 81 expressed	0.9463	0.0335	0.0003	0.0059	No	0.9474	0.227	0.874	0.161	No	No
96758_s_at	Sec14l2	SEC14-like 2 (S. cerevisiae)	0.9583	0.2756	0.2466	0.0579	No	0.9945	0.874	0.15	0.452	No	No
96759_r_at	Sec14l2	SEC14-like 2 (S. cerevisiae)	0.9208	0.1565	0.0001	0.6472	No	1.0026	0.654	0.0001	0.0691	No	No
96760_at	Timm9	translocase of inner mitochondrial membrane 9 homolog (yeast)	0.9182	0.2523	0.1521	0.4775	No	1.048	0.707	0.674	0.997	No	No
96761_at	Slc44a4	solute carrier family 44, member 4	1.1316	0.0797	0.1041	0.1623	No	1.11	0.106	0.107	0.0906	No	No
96762_at	Tnpo1	transportin 1	0.9585	0.4958	0.0115	0.2841	No	0.9579	0.553	0.0409	0.511	No	No
96763_at	Sardh	sarcosine dehydrogenase	1.0462	0.1511	0.0215	0.5016	No	1.0294	0.424	0.0093	0.635	No	No
96764_at	Iigp1	interferon inducible GTPase 1	1.0558	0.2728	0.0202	0.0828	No	1.0236	0.936	0.679	0.551	No	No
96765_at	Peg3	paternally expressed 3	0.9437	0.24	0.0265	0.5235	No	0.91	0.0137	0.0479	0.795	No	No
96766_s_at	Tyro3	TYRO3 protein tyrosine kinase 3	1.1366	0.0029	0.1168	0.2324	Yes	1.1275	0.0447	0.0613	0.505	No	Yes
96767_at	Mbc2	membrane bound C2 domain containing protein	0.9711	0.3425	0.1085	0.1658	No	0.9488	0.0972	0.0271	0.066	No	No
96768_at	Fbxw4	F-box and WD-40 domain protein 4	0.9733	0.2532	0.0011	0.8787	No	0.9732	0.159	0.0001	0.187	No	No
96770_at	Dnase1l2	deoxyribonuclease 1-like 2	0.9949	0.852	0.0048	0.821	No	1.1214	0.225	0.0484	0.938	No	No
96771_at	Erbb3	v-erb-b2 erythroblastic leukemia viral oncogene homolog 3 (avian)	0.9538	0.2957	0.0014	0.728	No	0.9738	0.368	0.0016	0.219	No	No
96772_at	Prim1	DNA primase, p49 subunit	1.0511	0.2526	0.0117	0.963	No	1.0283	0.628	0.0364	0.853	No	No
96773_at	Txndc4	thioredoxin domain containing 4 (endoplasmic reticulum)	0.9555	0.3559	0.0019	0.4218	No	0.9571	0.134	0	0.265	No	No
96774_at	Plekhc1	pleckstrin homology domain containing, family C (with FERM domain) member 1	1.0034	0.9677	0.0513	0.4124	No	0.9272	0.357	0.267	0.455	No	No
96775_at	Cbx1	chromobox homolog 1 (Drosophila HP1 beta)	1.0496	0.697	0.6473	0.4897	No	1.1751	0.0982	0.0231	0.543	No	No
96777_at	Sf3b1	splicing factor 3b, subunit 1	1.1347	0.1653	0.0148	0.1692	No	1.065	0.189	0.0033	0.0544	No	No
96778_at	Rrs1	RRS1 ribosome biogenesis regulator homolog (S. cerevisiae)	0.9285	0.2004	0.0087	0.2131	No	0.9549	0.0344	0	0.0283	No	No
96779_f_at	2410022L05 Rik	RIKEN cDNA 2410022L05 gene	1.2242	0.0398	0.0144	0.4661	No	1.2419	0.0052	0.0018	0.311	Yes	Yes
96780_at	2410022L05 Rik	RIKEN cDNA 2410022L05 gene	1.0326	0.6589	0.0863	0.5149	No	1.1136	0.0398	0.0017	0.029	No	No
96781_at	Rrn3	RRN3 RNA polymerase I transcription factor homolog (yeast)	1.0134	0.8768	0.3924	0.6109	No	0.9669	0.504	0.316	0.283	No	No
96782_at	Krt2-19	keratin complex 2, basic, gene 19	1.018	0.5407	0.0551	0.5208	No	1.0247	0.111	0.188	0.0631	No	No

96783_at	Mucdhl	mucin and cadherin like	1.0783	0.1731	0.0887	0.8923	No	1.0673	0.256	0.267	0.85	No	No
96784_at	Anln	anillin, actin binding protein (scraps homolog, Drosophila)	1.0513	0.1155	0.0072	0.0148	No	0.9558	0.388	0.36	0.407	No	No
96785_at	Ankrd47	ankyrin repeat domain 47	0.8635	0.0483	0.0628	0.0146	No	0.8944	0.024	0.204	0.0166	No	No
96786_s_at	Ankrd47	ankyrin repeat domain 47	0.9384	0.1805	0.0012	0.5165	No	0.9653	0.426	0.0031	0.592	No	No
96787_at	Serpina10	serine (or cysteine) peptidase inhibitor, clade A (alpha-1 antiproteinase, antitrypsin), member 10	0.9896	0.822	0.0539	0.5986	No	0.9887	0.847	0.381	0.192	No	No
96789_i_at	Galm	galactose mutarotase	0.8887	0.0291	0.5501	0.2077	No	0.9323	0.0428	0.0123	0.935	No	No
96790_f_at	Galm	galactose mutarotase	0.9108	0.0867	0.1428	0.3982	No	0.9287	0.137	0.18	0.0063	No	No
96791_at	1500005K14 Rik	RIKEN cDNA 1500005K14 gene	1.0915	0.0188	0.0007	0.1723	No	1.1134	0.0149	0.0003	0.143	No	No
96792_at	Apob	apolipoprotein B	0.9885	0.8193	0.3247	0.6564	No	0.9098	0.156	0.0608	0.736	No	No
96793_at	Dmap1	DNA methyltransferase 1-associated protein 1	0.9253	0.0004	0.0013	0.0001	No	1.0619	0.0315	0.372	0.497	No	No
96794_at	Zfp622	zinc finger protein 622	0.9495	0.229	0.3593	0.5993	No	0.9383	0.0448	0.819	0.175	No	No
96795_at	Faah	fatty acid amide hydrolase	1.0073	0.889	0.3886	0.9654	No	1.0074	0.863	0.574	0.0661	No	No
96796_f_at	Ugt2b5	UDP glucuronosyltransferase 2 family, polypeptide B5	0.9961	0.9339	0.6508	0.2177	No	1.0763	0.206	0.905	0.461	No	No
96797_s_at	Fbxw5	F-box and WD-40 domain protein 5	0.966	0.2278	0.9378	0.0191	No	0.9952	0.627	0.0039	0.0084	No	No
96798_r_at	Fbxw5	F-box and WD-40 domain protein 5	1.0141	0.791	0.2172	0.5795	No	1.2825	0.0603	0.0252	0.064	No	No
96799_at	Fbxw5	F-box and WD-40 domain protein 5	1.0238	0.4964	0.0008	0.477	No	1.031	0.312	0.0005	0.888	No	No
96801_at	Ak1	adenylate kinase 1	0.7761	0.006	0.8965	0.0354	Yes	0.8202	0.0108	0.0268	0.0253	No	Yes
96802_at	Pdcd7	programmed cell death protein 7	1.0698	0.0492	0.0137	0.0049	No	1.1197	0.029	0.03	0.0088	No	No
96803_at	Gbe1	glucan (1,4-alpha-), branching enzyme 1	0.8993	0.3742	0.1268	0.3997	No	1.048	0.375	0.0004	0.434	No	No
96804_at	Nol1	nucleolar protein 1	0.9871	0.4825	0.6302	0.5117	No	1.0596	0.043	0.53	0.0439	No	No
96806_at	Lpin2	lipin 2	0.9789	0.6494	0.1734	0.148	No	1.0339	0.309	0.0057	0.242	No	No
96807_at	Il2rg	interleukin 2 receptor, gamma chain	1.0463	0.386	0.0361	0.094	No	1.1636	0.0231	0.0035	0.0096	No	No
96808_at	---	---	1.0133	0.6466	0.8062	0.0633	No	1.0364	0.0844	0.0167	0.0013	No	No
96810_at	Lmo2	LIM domain only 2	1.0729	0.264	0.1298	0.2287	No	1.0976	0.136	0.0274	0.572	No	No
96811_at	Rab31	RAB31, member RAS oncogene family	1.0455	0.2192	0.6084	0.5158	No	1	0.985	0.0087	0.968	No	No
96812_at	Smo	smoothened homolog (Drosophila)	1.0631	0.3632	0.6351	0.8868	No	0.9798	0.711	0.0608	0.993	No	No
96813_f_at	Otud5	OTU domain containing 5	1.0118	0.81	0.0048	0.055	No	1.0286	0.779	0.201	0.0135	No	No
96814_r_at	Otud5	OTU domain containing 5	1	0.9991	0.8863	0.006	No	0.9455	0.0714	0.315	0.0472	No	No
96817_at	Anks3	Ankyrin repeat and sterile alpha motif domain containing 3	0.962	0.2899	0.0936	0.7509	No	1.0695	0.0807	0.0124	0.0639	No	No
96818_at	Dtx2	deltex 2 homolog (Drosophila)	0.9154	0.1249	0.0022	0.0503	No	0.924	0.0808	0.003	0.0268	No	No
96819_at	2310035K24 Rik	RIKEN cDNA 2310035K24 gene	1.0039	0.917	0.6132	0.0853	No	0.8955	0.0049	0.0001	0.0124	Yes	No
96822_at	Eif2b5	eukaryotic translation initiation factor 2B, subunit 5 epsilon	0.9621	0.3531	0.0228	0.0606	No	0.9113	0.0722	0.0204	0.0186	No	No
96824_at	Sox15	SRY-box containing gene 15	1.0106	0.8223	0.0058	0.8164	No	0.9899	0.949	0.0123	0.195	No	No
96825_at	Tenc1	tensin like C1 domain-containing phosphatase	0.8452	0.0065	0.0002	0.9063	Yes	0.8861	0.0171	0.0001	0.988	No	Yes
96826_at	Mfap4	Microfibrillar-associated protein 4	1.1079	0.1697	0.3394	0.0797	No	1.1677	0.0097	0.0052	0.0058	No	No
96827_at	Cad	carbamoyl-phosphate synthetase 2, aspartate transcarbamylase, and dihydroorotase	0.9915	0.7739	0.0097	0.2792	No	1.0306	0.272	0.208	0.041	No	No
96828_at	Gnmt	glycine N-methyltransferase	0.9408	0.2027	0.0107	0.7002	No	1.0467	0.407	0.0013	0.0249	No	No
96829_at	D19Wsu162 e	DNA segment, Chr 19, Wayne State University 162, expressed	0.9491	0.0323	0.0012	0.0005	No	0.9497	0.0419	0.0021	0.0026	No	No
96831_at	Pdia5	protein disulfide isomerase associated 5	0.9214	0.3017	0.8808	0.1289	No	0.9642	0.178	0.0023	0.0416	No	No
96832_at	Slc39a1	solute carrier family 39 (zinc transporter), member 1	0.8946	0.1501	0.1619	0.0665	No	0.8786	0.0321	0.204	0.026	No	No
96833_at	Nucks1	nuclear casein kinase and cyclin-dependent kinase substrate 1	0.9699	0.2934	0.4467	0.4262	No	1.0402	0.121	0.0018	0.0176	No	No
96834_at	Sfrs9	splicing factor, arginine/serine rich 9	1.1362	0.1297	0.6662	0.881	No	1.1603	0.0333	0.903	0.418	No	No
96835_at	Matn4	matrilin 4	1.0638	0.1365	0.0003	0.5155	No	1.0489	0.167	0.0003	0.241	No	No
96836_r_at	Zfp161	zinc finger protein 161	1.1403	0.1943	0.1267	0.5946	No	1.1876	0.378	0.466	0.943	No	No
96837_at	ORF61	open reading frame 61	0.9864	0.4716	0.0001	0.1829	No	0.9843	0.178	0.0006	0.0018	No	No
96838_at	Rce1	RCE1 homolog, prenyl protein peptidase (S. cerevisiae)	0.9641	0.029	0.0001	0.0083	No	1.0006	0.999	0.0821	0.868	No	No
96839_at	1110014N23 Rik	RIKEN cDNA 1110014N23 gene	1.0345	0.4191	0.35	0.037	No	1.0494	0.213	0.169	0.366	No	No
96840_at	Gabarapl2	gamma-aminobutyric acid (GABA-A) receptor-associated protein-like 2	0.9297	0.1187	0.0669	0.0476	No	0.9571	0.35	0.563	0.121	No	No
96841_at	Pim3	proviral integration site 3	0.8673	0.0218	0.0041	0.3016	No	0.8881	0.0142	0.0008	0.682	No	No
96843_at	Eif4e2	eukaryotic translation initiation factor 4E member 2	1.018	0.5204	0.8555	0.2494	No	0.9652	0.135	0.0023	0.892	No	No
96845_at	Slc30a9	solute carrier family 30 (zinc transporter), member 9	1.0512	0.2565	0.0003	0.1482	No	0.9993	0.853	0.0121	0.0434	No	No
96846_at	Serpinc1	serine (or cysteine) peptidase inhibitor, clade C (antithrombin), member 1	1.0912	0.0554	0.3313	0.0245	No	1.0315	0.543	0.984	0.353	No	No
96847_at	Vps28	vacuolar protein sorting 28 (yeast)	1.0576	0.3612	0.0908	0.2625	No	1.0533	0.22	0.419	0.29	No	No
96848_at	Pmpca	peptidase (mitochondrial processing) alpha	1.0932	0.2314	0.4029	0.2737	No	0.9952	0.908	0.0016	0.832	No	No
96849_at	Timm8a1	translocase of inner mitochondrial membrane 8 homolog a1 (yeast)	1.0748	0.4835	0.029	0.7751	No	1.0458	0.528	0.297	0.874	No	No
96850_at	Eif4g3	eukaryotic translation initiation factor 4 gamma, 3	0.979	0.6865	0.0025	0.1395	No	0.9627	0.51	0.0019	0.906	No	No
96852_at	Prkar1a	protein kinase, cAMP dependent regulatory, type I, alpha	1.0036	0.9603	0.1034	0.2285	No	1.0566	0.29	0.0862	0.292	No	No
96854_at	Copa	coatomer protein complex subunit alpha	1.0976	0.0711	0.7038	0.8426	No	1.0309	0.5	0.11	0.984	No	No
96855_at	Spcs2	signal peptidase complex subunit 2 homolog (S. cerevisiae)	1.004	0.9368	0.1343	0.3461	No	1.0384	0.327	0.104	0.167	No	No
96856_at	C1qbp	complement component 1, q subcomponent binding protein	1.0338	0.7627	0.1386	0.463	No	0.9443	0.405	0.0007	0.458	No	No
96857_at	C1qbp	complement component 1, q subcomponent binding protein	1.1149	0.0331	0.011	0.1367	No	1.0087	0.868	0.052	0.636	No	No
96858_at	Pdcd8	programmed cell death 8	1.0348	0.3811	0.1737	0.2479	No	1.0432	0.376	0.259	0.123	No	No
96859_at	Rnf10	ring finger protein 10	0.9873	0.6757	0.0014	0.4614	No	0.9773	0.313	0.0007	0.748	No	No
96861_at	Mrpl50	mitochondrial ribosomal protein L50	0.9641	0.2198	0.0188	0.2876	No	0.9358	0.133	0.174	0.129	No	No
96862_at	1110002B05 Rik	RIKEN cDNA 1110002B05 gene	1.0108	0.9076	0.4967	0.496	No	0.9771	0.753	0.0381	0.616	No	No
96864_at	Mrps26	mitochondrial ribosomal protein S26	1.0006	0.9909	0.0302	0.1486	No	1.1047	0.0728	0.0834	0.274	No	No
96865_at	---	0 day neonate cerebellum cDNA, RIKEN full-length enriched library, clone:C230070N19 product:myristoylated alanine rich protein kinase C substrate, full insert sequence	0.9635	0.6325	0.0077	0.2566	No	0.9756	0.896	0.0167	0.122	No	No
96866_at	Avpi1	arginine vasopressin-induced 1	0.9735	0.5897	0.0054	0.4442	No	1.0442	0.361	0.0024	0.489	No	No
96867_at	Azgp1	alpha-2-glycoprotein 1, zinc	0.9523	0.2313	0.1842	0.901	No	0.9945	0.919	0.0646	0.757	No	No
96868_at	Fgb	fibrinogen, B beta polypeptide	0.9995	0.996	0.4986	0.8578	No	0.8522	0.136	0.0137	0.516	No	No
96869_at	Gabarap	gamma-aminobutyric acid receptor associated protein	0.9914	0.9048	0.0151	0.3191	No	0.9349	0.145	0.0033	0.255	No	No
96870_at	Aco2	aconitase 2, mitochondrial	1.0359	0.4747	0.0135	0.1089	No	1.0047	0.692	0.0004	0.0306	No	No
96871_at	2310042G06 Rik	RIKEN cDNA 2310042G06 gene	0.9642	0.2785	0.0029	0.0105	No	0.9892	0.753	0.0469	0.0903	No	No
96872_at	Sgta	small glutamine-rich tetratricopeptide repeat (TPR)-containing, alpha	0.992	0.7853	0.0332	0.6158	No	1.008	0.88	0.0533	0.681	No	No
96873_at	Tax1bp1	Tax1 (human T-cell leukemia virus type I) binding protein 1	1.0836	0.126	0.0034	0.0182	No	1.0679	0.122	0.0039	0.0174	No	No
96874_g_at	Tax1bp1	Tax1 (human T-cell leukemia virus type I) binding protein 1	1.0474	0.3219	0.0028	0.4096	No	1.0235	0.701	0.0134	0.29	No	No
96875_r_at	Tax1bp1	Tax1 (human T-cell leukemia virus type I) binding protein 1	1.1644	0.0033	0.0001	0.0336	Yes	1.101	0.199	0.0058	0.0932	No	No
96876_at	Laptm4a	lysosomal-associated protein transmembrane 4A	1.0734	0.0771	0.0595	0.9455	No	1.0217	0.544	0.0048	0.269	No	No
96878_at	Cyb5b	cytochrome b5 type B	1.2027	0.1098	0.8314	0.7481	No	1.0665	0.243	0.0072	0.665	No	No
96879_at	Ogdh	oxoglutarate dehydrogenase (lipoamide)	0.9814	0.5354	0.0017	0.2975	No	0.9738	0.517	0.0008	0.677	No	No
96881_at	Commd6	COMM domain containing 6	1.1791	0.0929	0.3896	0.6857	No	1.1159	0.154	0.0903	0.701	No	No
96882_at	Ga17	dendritic cell protein GA17	1.0462	0.105	0.0006	0.9245	No	1.0202	0.712	0.005	0.994	No	No
96883_at	Eif3s4	eukaryotic translation initiation factor 3, subunit 4 (delta)	0.9919	0.528	0	0.0054	No	0.9731	0.732	0.0002	0.018	No	No
96884_at	Carhsp1	calcium regulated heat stable protein 1	0.9709	0.4696	0.0058	0.7527	No	0.9668	0.291	0.0005	0.775	No	No
96885_at	Nt5dc2	5'-nucleotidase domain containing 2	1.0637	0.0659	0.0009	0.2669	No	1.0508	0.217	0.0008	0.686	No	No
96886_at	Stab1	stabilin 1	1.0147	0.5078	0.0334	0.0356	No	1.0277	0.616	0.354	0.255	No	No
96887_at	Ndufb11	NADH dehydrogenase (ubiquinone) 1 beta subcomplex, 11	0.9883	0.781	0.0261	0.5888	No	0.9865	0.923	0.0041	0.14	No	No
96888_at	Akr1a4	aldo-keto reductase family 1, member A4 (aldehyde reductase)	1.0762	0.0653	0.0022	0.6353	No	1.0328	0.0818	0	0.437	No	No

96890_at	1300002A08 Rik	RIKEN cDNA 1300002A08 gene	0.8813	0.0582	0.0039	0.0838	No	0.9269	0.289	0.0531	0.742	No	No
96891_at	Anp32b /// LOC621961	acidic nuclear phosphoprotein 32 family, member B /// similar to acidic nuclear phosphoprotein 32 family, member B	1.1713	0.0439	0.111	0.7295	No	1.0348	0.374	0.0258	0.212	No	No
96892_at	Psma1	proteasome (prosome, macropain) subunit, alpha type 1	1.1244	0.1049	0.2397	0.5527	No	1.0218	0.354	0.001	0.0613	No	No
96894_at	Tmed4	transmembrane emp24 protein transport domain containing 4	0.968	0.2952	0.8556	0.0518	No	1.0233	0.487	0.624	0.939	No	No
96895_at	Pon1	paraoxonase 1	0.9909	0.8852	0.5337	0.618	No	0.976	0.629	0.0055	0.364	No	No
96896_at	Actr2	ARP2 actin-related protein 2 homolog (yeast)	0.9859	0.8835	0.1789	0.9895	No	0.9927	0.963	1	0.365	No	No
96898_at	Atp5f1 /// LOC667832 /// LOC671112 /// LOC676414	ATP synthase, H+ transporting, mitochondrial F0 complex, subunit b, isoform 1 /// similar to ATP synthase B chain, mitochondrial precursor /// similar to ATP synthase B chain, mitochondrial precursor /// similar to ATP synthase B chain, mitochondrial precursor	1.0338	0.2686	0.0203	0.5283	No	0.9949	0.794	0	0.0572	No	No
96899_at	Ndufs3	NADH dehydrogenase (ubiquinone) Fe-S protein 3	0.9821	0.7156	0.2551	0.3751	No	1.0083	0.862	0.226	0.254	No	No
96900_at	Ndg2	Nur77 downstream gene 2	1.0509	0.1817	0.958	0.0385	No	0.9702	0.367	0.491	0.0219	No	No
96902_at	Ccdc53	coiled-coil domain containing 53	0.8991	0.0391	0.1864	0.0083	No	0.8808	0.0723	0.0959	0.0211	No	No
96904_at	Mrps7	mitchondrial ribosomal protein S7	1.0193	0.6538	0.0494	0.0795	No	0.9747	0.678	0.0807	0.0084	No	No
96905_at	Mir16	membrane interacting protein of RGS16	0.9648	0.6526	0.4555	0.9481	No	0.8842	0.0309	0.0074	0.492	No	No
96906_at	Chchd2 /// LOC433806 /// LOC665367 /// LOC670875	coiled-coil-helix-coiled-coil-helix domain containing 2 /// similar to coiled-coil-helix-coiled-coil-helix domain containing 2 /// similar to coiled-coil-helix-coiled-coil-helix domain containing 2 /// similar to coiled-coil-helix-coiled-coil-helix domain containing 2	0.9486	0.5228	0.8918	0.6457	No	1.0461	0.31	0.46	0.0559	No	No
96907_at	Cherp	calcium homeostasis endoplasmic reticulum protein	0.9625	0.1344	0.0003	0.1474	No	1.0303	0.639	0.0882	0.881	No	No
96908_at	1110014F24 Rik	RIKEN cDNA 1110014F24 gene	1.004	0.9653	0.0285	0.4199	No	1.1347	0.011	0.0002	0.187	No	No
96909_at	Ndufab1	NADH dehydrogenase (ubiquinone) 1, alpha/beta subcomplex, 1	1.1475	0.0179	0.0344	0.1379	No	1.1086	0.0465	0.13	0.153	No	No
96910_at	Acsm2	acyl-CoA synthetase medium-chain family member 2	1.0296	0.4324	0.9661	0.5855	No	0.9496	0.558	0.617	0.416	No	No
96911_at	Gnb2	guanine nucleotide binding protein, beta 2	1.0624	0.3499	0.3009	0.4955	No	0.9992	0.939	0.0598	0.737	No	No
96912_s_at	Ctla2a /// Ctla2b	cytotoxic T lymphocyte-associated protein 2 alpha /// cytotoxic T lymphocyte-associated protein 2 beta	0.9459	0.1258	0.3387	0.4445	No	0.9394	0.402	0.396	0.169	No	No
96913_at	Hadhb /// LOC623031	hydroxyacyl-Coenzyme A dehydrogenase/3-ketoacyl-Coenzyme A thiolase/enoyl-Coenzyme A hydratase (trifunctional protein), beta subunit /// similar to hydroxyacyl-Coenzyme A dehydrogenase/3-ketoacyl-Coenzyme A thiolase/enoyl-Coenzyme A hydratase (trifunctional protein), beta subunit	1.051	0.0945	0.0021	0.1912	No	1.0553	0.213	0.0134	0.887	No	No
96915_f_at	Ndufa3	NADH dehydrogenase (ubiquinone) 1 alpha subcomplex, 3	1.1471	0.024	0.0069	0.4242	No	1.0189	0.581	0.0092	0.203	No	No
96916_at	Mrpl33	mitochondrial ribosomal protein L33	1.0379	0.0067	0	0.0002	No	1.1027	0.216	0.881	0.133	No	No
96917_at	2410166I05 Rik	RIKEN cDNA 2410166I05 gene	0.9409	0.032	0.0055	0.8016	No	1.0097	0.759	0.0448	0.24	No	No
96918_at	Fbp1	fructose bisphosphatase 1	0.9563	0.1591	0.0106	0.059	No	0.9821	0.814	0.0764	0.672	No	No
96919_at	Atp6v0c	ATPase, H+ transporting, lysosomal V0 subunit C	1.0234	0.2782	0.0006	0.0074	No	0.9832	0.209	0.0174	0.0222	No	No
96920_at	Htra1	HtrA serine peptidase 1	1.0739	0.0179	0.3648	0.0652	No	1.066	0.0438	0.0346	0.0158	No	No
96921_at	Ttc1	tetratricopeptide repeat domain 1	0.9653	0.2874	0.0786	0.3505	No	0.9577	0.198	0.815	0.237	No	No
96924_at	Wdr82	WD repeat domain containing 82	1.0202	0.5838	0.0249	0.8958	No	1.0403	0.0293	0.0048	0.0909	No	No
96925_at	Glt25d1	glycosyltransferase 25 domain containing 1	1.0139	0.8361	0.003	0.7806	No	1.0013	0.838	0.0003	0.155	No	No
96926_at	Smoc2	SPARC related modular calcium binding 2	0.9601	0.187	0.0002	0.1967	No	1.0169	0.439	0	0.979	No	No
96929_at	Ehd1	EH-domain containing 1	1.0201	0.5227	0.2453	0.0643	No	1.1954	0.0337	0.195	0.0387	No	No
96930_at	Ehd1	EH-domain containing 1	1.0368	0.5074	0.0172	0.8283	No	1.033	0.138	0.0072	0.271	No	No
96931_at	---	---	1.0075	0.8513	0.3066	0.5257	No	1.0487	0.448	0.821	0.0781	No	No
96932_at	Nudcd2	NudC domain containing 2	1.0016	0.9741	0.0029	0.7915	No	1.0223	0.506	0.0028	0.2	No	No
96934_at	Myeov2	myeloma overexpressed 2	0.9632	0.5247	0.0625	0.2364	No	0.9447	0.305	0.0462	0.0166	No	No
96935_at	Pdzk1ip1	PDZK1 interacting protein 1	1.0272	0.5821	0.3838	0.1259	No	1.0287	0.251	0.0013	0.034	No	No
96936_at	Copg	coatomer protein complex, subunit gamma	0.9876	0.4642	0.1533	0.1574	No	0.9832	0.594	0.271	0.984	No	No
96937_at	Pfdn1	prefoldin 1	1.0918	0.2305	0.72	0.1002	No	1.1824	0.356	0.497	0.1	No	No
96938_at	Keg1	kidney expressed gene 1	1.0087	0.8403	0.4008	0.778	No	1.0023	0.994	0.232	0.0582	No	No
96939_at	Myl9	myosin, light polypeptide 9, regulatory	1.0973	0.0227	0.0041	0.0125	No	0.9842	0.893	0.0842	0.583	No	No
96940_at	Tead2	TEA domain family member 2	0.9932	0.6671	0	0.171	No	1.0607	0.044	0.0001	0.0961	No	No
96941_at	Rabl4	RAB, member of RAS oncogene family-like 4	0.984	0.6137	0.0017	0.4825	No	0.9819	0.641	0.005	0.548	No	No
96942_at	Eif3s6ip	eukaryotic translation initiation factor 3, subunit 6 interacting protein	0.9857	0.6048	0.1032	0.0299	No	0.9818	0.417	0.17	0.0205	No	No
96943_at	Gps1	G protein pathway suppressor 1	1.0698	0.0141	0.4066	0.0068	No	1.1148	0.0541	0.854	0.0424	No	No
96945_at	Snap23	synaptosomal-associated protein 23	0.9283	0.02	0.1666	0.0062	No	0.9493	0.367	0.978	0.102	No	No
96946_at	Lsm1	LSM1 homolog, U6 small nuclear RNA associated (S. cerevisiae)	0.9632	0.3005	0.0165	0.111	No	1.0391	0.551	0.0611	0.828	No	No
96947_at	Etfb	electron transferring flavoprotein, beta polypeptide	1.0443	0.3069	0.0327	0.2401	No	1.0477	0.0974	0.0025	0.0292	No	No
96948_at	Qdpr	quininoid dihydropteridine reductase	1.0862	0.152	0.0343	0.8384	No	1.0953	0.088	0.0231	0.741	No	No
96949_at	Ythdf1	YTH domain family 1	0.997	0.9343	0.001	0.0996	No	0.9421	0.358	0.0057	0.642	No	No
96950_at	Ccl19	chemokine (C-C motif) ligand 19	1.0465	0.3801	0.058	0.1501	No	1.0628	0.241	0.028	0.428	No	No
96951_at	Atp6v1d	ATPase, H+ transporting, lysosomal V1 subunit D	0.9864	0.7839	0.0099	0.6646	No	1.025	0.697	0.343	0.233	No	No
96952_at	Psma6	proteasome (prosome, macropain) subunit, alpha type 6	1.0297	0.4998	0.5386	0.2226	No	1.002	0.844	0.0269	0.231	No	No
96953_at	Cxcl14	chemokine (C-X-C motif) ligand 14	0.9208	0.0764	0.2999	0.2319	No	0.9408	0.189	0.497	0.509	No	No
96954_at	Stk24	serine/threonine kinase 24 (STE20 homolog, yeast)	1.0539	0.3795	0.2593	0.1234	No	1.0102	0.75	0.0294	0.0052	No	No
96955_at	Atp6v0e2	ATPase, H+ transporting, lysosomal V0 subunit E2	1.0089	0.8585	0.1294	0.423	No	1.0105	0.687	0.0163	0.598	No	No
96956_at	Prdx5	peroxiredoxin 5	1.0533	0.2881	0.5511	0.7052	No	1.039	0.317	0.0259	0.415	No	No
96957_at	Cib1	calcium and integrin binding 1 (calmyrin)	1.0515	0.205	0.0248	0.0486	No	1.0268	0.509	0.0026	0.298	No	No
96958_at	Cib1	calcium and integrin binding 1 (calmyrin)	0.9343	0.314	0.1133	0.5366	No	0.8927	0.266	0.332	0.53	No	No
96959_at	Ube2n	ubiquitin-conjugating enzyme E2N	1.0975	0.1757	0.1587	0.4342	No	1.099	0.158	0.245	0.347	No	No
96961_at	Pcgf2	Polycomb group ring finger 2	1.0165	0.7794	0.5174	0.3865	No	1.1168	0.0846	0.329	0.0725	No	No
96962_at	Rpl6	ribosomal protein L6	0.9957	0.8509	0.0428	0.032	No	0.9756	0.0905	0.0267	0.0216	No	No
96963_s_at	---	Anti-preS1 immunoglobulin light chain variable region /// Immunoglobulin kappa chain complex	1.1428	0.2092	0.3596	0.1169	No	1.0929	0.354	0.115	0.327	No	No
96964_at	Igkv6-23 /// Igk-V19-17 /// Igk-V19-14 /// Igk-V19-13 /// LOC669053 /// LOC669070 /// LOC672450 /// LOC673123 /// LOC676136 /// LOC676162 /// LOC676175	immunoglobulin kappa chain variable 6-23 /// immunoglobulin kappa chain variable 19 (V19)-17 /// immunoglobulin kappa chain variable 19 (V19)-14 /// immunoglobulin kappa chain variable 19 (V19)-13 /// similar to Ig kappa chain V-V region MPC11 precursor /// similar to Ig kappa chain V-V region MPC11 precursor /// similar to Ig kappa chain V-V region MPC11 precursor /// similar to Ig kappa chain V-V region MPC11 precursor /// similar to Ig kappa chain V-V region MPC11 precursor /// similar to Ig kappa chain V-V region MPC11 precursor /// similar to Ig kappa chain V-V region MPC11 precursor	1.0491	0.5142	0.0793	0.4793	No	0.9011	0.127	0.0086	0.318	No	No
96965_at	Crygd /// Crygf	crystallin, gamma D /// crystallin, gamma F	0.9773	0.6182	0.7866	0.1023	No	0.9617	0.472	0.533	0.777	No	No
96966_at	Igh-1a	Immunoglobulin heavy chain 1a (serum IgG2a)	1.156	0.0821	0.6901	0.2726	No	0.8188	0.295	0.0015	0.298	No	No
96967_at	---	PREDICTED: Mus musculus similar to T-cell receptor beta chain V region CTL-L17 precursor (LOC386479), mRNA	0.9859	0.6948	0.0083	0.3803	No	1.0344	0.797	0.0875	0.884	No	No
96968_at	Ifnz	interferon zeta	1.0098	0.8974	0.3094	0.7291	No	1.0536	0.194	0.412	0.649	No	No
96969_at	Igkv2-112	immunoglobulin kappa chain variable 2-112	1.0498	0.2293	0.0279	0.0479	No	0.9366	0.455	0.113	0.921	No	No
96970_at	LOC238447	Similar to immunoglobulin heavy chain variable region	1.0066	0.9186	0.5343	0.6593	No	1.0412	0.471	0.0442	0.517	No	No

96971_f_at	Gm189 /// Gm459 /// Gm1418 /// Gm1419 /// Igkv4-61 /// Igkv4-56 /// Igkv4-73 /// Igkv4-69 /// Igkv4-63 /// LOC636646 /// LOC636677 /// LOC636730 /// Igkv4-62 /// LOC636818 /// Igkv4-77 /// LOC672339 /// LOC672342 /// LOC673628 /// LOC673793 ///	gene model 189, (NCBI) /// gene model 459, (NCBI) /// gene model 1418, (NCBI) /// gene model 1419, (NCBI) /// immunoglobulin kappa chain variable 4-61 /// immunoglobulin kappa chain variable 4-56 /// immunoglobulin kappa chain variable 4-73 /// immunoglobulin kappa chain variable 4-69 /// immunoglobulin kappa chain variable 4-63 /// similar to Ig kappa chain V-IV region S107B precursor /// similar to Ig kappa chain V-IV region S107B precursor /// similar to Ig kappa chain V-IV region S107B precursor /// immunoglobulin kappa chain variable 4-62 /// similar to Ig kappa chain V-VI region NQ2-17.4.1 /// immunoglobulin kappa chain variable 4-77 /// similar to Ig kappa chain V-IV region S107B precursor /// similar to Ig kappa chain V-IV region S107B precursor /// similar to Ig kappa chain V-IV region S107B precursor /// similar to Ig kappa chain V-IV region S107B precursor /// similar to Ig kappa chain V-IV region S107B precursor /// similar to Ig kappa chain V-IV region S107B precursor	0.9973	0.9609	0.8201	0.879	No	1.039	0.53	0.59	0.155	No	No
96972_f_at	Gm1077 /// Igkv4-73 /// LOC636646	gene model 1077, (NCBI) /// immunoglobulin kappa chain variable 4-73 /// similar to Ig kappa chain V-IV region S107B precursor	1.0514	0.3462	0.5791	0.1682	No	1.0411	0.309	0.618	0.0876	No	No
96973_f_at	LOC640696	similar to Ig heavy chain V-I region V35 precursor	1.021	0.7149	0.0207	0.9502	No	0.9674	0.731	0.0066	0.84	No	No
96974_at	LOC624610 /// LOC675735	hypothetical protein LOC624610 /// similar to Ig kappa chain V-V region L6 precursor	1.0232	0.2786	0.012	0.1102	No	1.1752	0.0333	0.0492	0.0159	No	No
96975_at	Igk-V1 /// Igkv1-117 /// Igk-V5 /// Cr1 /// Igkv1-110	immunoglobulin kappa chain variable 1 (V1) /// immunoglobulin kappa chain variable 1-117 /// immunoglobulin kappa chain variable 5 (V5 family) /// Ig kappa chain /// immunoglobulin kappa chain variable 1-110	1.1074	0.015	0.004	0.0011	No	1.0137	0.827	0.0606	0.238	No	No
96976_f_at	H2-T18 /// H2-T3 /// H2-Tw3 /// H2-T3-like /// LOC633417 /// LOC674370	histocompatibility 2, T region locus 18 /// histocompatibility 2, T region locus 3 /// similar to histocompatibility 2, T region locus 3 /// MHC class I antigen /// similar to histocompatibility 2, T region locus 3 /// similar to histocompatibility 2, T region locus 3	1.017	0.7905	0.4408	0.461	No	1.103	0.101	0.013	0.254	No	No
96977_at	LOC667782	similar to RT1 class Ib gene, H2-TL-like, grc region (N3)	1.1696	0.0276	0.1194	0.0794	No	1.1319	0.013	0.0134	0.722	No	No
96978_at	Sytl4	synaptotagmin-like 4	1.0594	0.3523	0.0332	0.0819	No	1.0628	0.266	0.0885	0.358	No	No
96979_at	Syt10	synaptotagmin X	0.857	0.0185	0.0029	0.0083	No	1.0146	0.742	0.0826	0.493	No	No
96980_at	Myh10	myosin, heavy polypeptide 10, non-muscle	0.9921	0.9199	0.3377	0.6701	No	1.0686	0.401	0.135	0.566	No	No
96981_at	Olfr1507	olfactory receptor 1507	0.9159	0.4721	0.1306	0.8104	No	0.8417	0.0809	0.0045	0.302	No	No
96982_g_at	Olfr1507 /// Olfr1508	olfactory receptor 1507 /// olfactory receptor 1508	1.0376	0.358	0.6903	0.6867	No	1.0958	0.111	0.009	0.56	No	No
96983_at	Olfr1508	olfactory receptor 1508	1.0946	0.3214	0.4124	0.4378	No	1.0401	0.562	0.534	0.189	No	No
96984_at	Olfr1509	olfactory receptor 1509	1.0197	0.6148	0.258	0.2284	No	1.0567	0.0854	0.104	0.924	No	No
96985_at	Olfr1264	olfactory receptor 1264	0.9979	0.9714	0.3868	0.9371	No	1.0771	0.369	0.172	0.31	No	No
96986_at	Olfr140	olfactory receptor 140	1.0267	0.7154	0.5166	0.3187	No	1.0472	0.178	0.652	0.0819	No	No
96987_at	Mllt7	myeloid/lymphoid or mixed lineage-leukemia translocation to 7 homolog (Drosophila)	1.0708	0.1168	0.0561	0.1867	No	1.0139	0.663	0.546	0.446	No	No
96992_r_at	LOC637260 /// Igk-V19-17 /// LOC673123 /// LOC673777 /// LOC676136	similar to Ig kappa chain V-IV region B17 precursor /// immunoglobulin kappa chain variable 19 (V19)-17 /// similar to Ig kappa chain V-V region MPC11 precursor /// similar to Ig kappa chain V-IV region precursor /// similar to Ig kappa chain V-V region MPC11 precursor	1.1208	0.0068	0.0827	0.0016	No	0.9627	0.688	0.608	0.912	No	No
96993_at	Pax5	paired box gene 5	1.0821	0.1938	0.1072	0.6729	No	1.1036	0.313	0.0054	0.146	No	No
96994_at	Hic1	hypermethylated in cancer 1	0.9449	0.2931	0.2055	0.438	No	1.0583	0.375	0.0071	0.578	No	No
96995_at	LOC622159 /// LOC665000 /// LOC671878 /// LOC673187 /// LOC674839	similar to spermine synthase /// similar to spermine synthase /// similar to Spermine synthase (Spermidine aminopropyltransferase) (SPMSY) /// hypothetical protein LOC673187 /// similar to spermine synthase	1.0054	0.9415	0.0267	0.6289	No	1.0409	0.188	0.0001	0.527	No	No
96996_at	Chrna6	cholinergic receptor, nicotinic, alpha polypeptide 6	0.955	0.2903	0.0204	0.8465	No	1.0022	0.848	0.131	0.0405	No	No
96997_at	Accn5	amiloride-sensitive cation channel 5, intestinal	1.0379	0.59	0.7391	0.3953	No	1.1509	0.295	0.46	0.859	No	No
96998_at	Galnt6	UDP-N-acetyl-alpha-D-galactosamine:polypeptide N-acetylgalactosaminyltransferase 6	0.9721	0.3957	0.0241	0.2395	No	1.0015	0.955	0.761	0.231	No	No
96999_at	Olfr155	olfactory receptor 155	1.0437	0.1603	0.0777	0.0479	No	1.0226	0.82	0.854	0.112	No	No
97000_s_at	Olfr156 /// Olfr157	olfactory receptor 156 /// olfactory receptor 157	1.0327	0.0517	0.0695	0.0012	No	1.0398	0.122	0.025	0.0026	No	No
97001_r_at	Olfr157	olfactory receptor 157	1.0958	0.2339	0.0554	0.1921	No	1.0098	0.904	0.357	0.124	No	No
97002_f_at	Olfr29-ps1	olfactory receptor 29, pseudogene 1	1.0673	0.3544	0.0313	0.4814	No	1.0827	0.13	0.0064	0.238	No	No
97003_at	Olfr159	olfactory receptor 159	0.945	0.4843	0.1529	0.7851	No	1.0011	0.747	0.0018	0.197	No	No
97004_at	Olfr71	olfactory receptor 71	0.9875	0.7625	0.0287	0.7751	No	1.0377	0.217	0.0121	0.0359	No	No
97005_at	Olfr70	olfactory receptor 70	1.0048	0.9337	0.0559	0.7817	No	0.9865	0.789	0.172	0.146	No	No
97006_s_at	Lifr	leukemia inhibitory factor receptor	0.8781	0.02	0.0052	0.0374	No	0.9772	0.622	0.0047	0.827	No	No
97007_at	LOC545814 /// 4931427P12Rik /// LOC622486	sperm motility kinase 2-like /// RIKEN cDNA 4931427P12 gene /// similar to serine/threonine kinase	0.9273	0.2177	0.0527	0.1951	No	0.983	0.753	0.339	0.191	No	No

97008_f_at	Igh /// Igh-1a /// AI324046 /// LOC382692 /// LOC628614 /// LOC629869 /// LOC629884 /// LOC629897 /// LOC634081 /// LOC634100 /// LOC634136 /// LOC634206 /// LOC634275 /// LOC634338 /// LOC634541	immunoglobulin heavy chain complex /// immunoglobulin heavy chain 1a (serum IgG2a) /// expressed sequence AI324046 /// similar to Ig heavy chain V-I region HG3 precursor /// similar to Ig heavy chain V-I region V35 precursor /// similar to Ig heavy chain V region 108A precursor /// similar to Ig heavy chain V region AC38 205.12 /// similar to Ig heavy chain V-I region HG3 precursor /// similar to Ig heavy chain V-I region HG3 precursor /// similar to Ig heavy chain V region 108A precursor /// similar to Ig heavy chain V-I region V35 precursor /// similar to Ig heavy chain V-I region V35 precursor /// similar to Ig heavy chain V region 108A precursor /// similar to Ig heavy chain V-I region V35 precursor /// similar to Ig heavy chain V region 108A precursor /// similar to Ig heavy chain V region 108A precursor /// similar to Ig heavy chain V-I region V35 precursor /// similar to Ig heavy chain V-I region HG3 precursor /// similar to Ig heavy chain V region 108A precursor /// similar to Ig heavy chain V-I region HG3 precursor /// similar to Ig heavy chain V region 108A precursor	1.0002	0.9986	0.0286	0.864	No	1.0382	0.377	0.0098	0.0118	No	No
97009_f_at	LOC629906 /// LOC640492	similar to Ig heavy chain V-I region V35 precursor /// similar to Ig heavy chain V-I region V35 precursor	1.0006	0.9908	0.8025	0.491	No	1.0702	0.301	0.643	0.183	No	No
97010_at	Chrnb2	cholinergic receptor, nicotinic, beta polypeptide 2 (neuronal)	1.0291	0.3762	0.8337	0.3923	No	0.928	0.405	0.336	0.13	No	No
97012_f_at	Aurkaip1	aurora kinase A interacting protein 1	0.971	0.4094	0.0534	0.1183	No	0.994	0.918	0.0007	0.428	No	No
97013_f_at	Cyba	cytochrome b-245, alpha polypeptide	1.007	0.7936	0.01	0.0073	No	1.0169	0.356	0.0022	0.0145	No	No
97017_f_at	---	---	0.9663	0.3879	0.0094	0.775	No	1.1098	0.129	0.0022	0.544	No	No
97048_at	A130038H09 Rik	RIKEN cDNA A130038H09 gene	1.0546	0.4636	0.0381	0.5356	No	1.0288	0.632	0.0755	0.229	No	No
97049_at	---	---	0.887	0.2241	0.1427	0.5434	No	0.8866	0.327	0.0125	0.935	No	No
97052_at	Vwf	Von Willebrand factor homolog	1.0166	0.6938	0.0012	0.3806	No	0.9993	0.881	0.0049	0.416	No	No
97053_at	Xcr1	chemokine (C motif) receptor 1	1.0401	0.4193	0.1117	0.7408	No	1.0394	0.223	0.0029	0.256	No	No
97054_at	Mapkbp1	mitogen activated protein kinase binding protein 1	1.0027	0.9422	0.0663	0.6291	No	0.9844	0.665	0.0012	0.328	No	No
97055_s_at	Prdx1	peroxiredoxin 1	1.0114	0.8328	0.1891	0.955	No	0.9945	0.855	0.102	0.594	No	No
97058_f_at	Rab33b	RAB33B, member of RAS oncogene family	1.0236	0.5721	0.0056	0.7267	No	1.0012	0.896	0.0029	0.332	No	No
97060_at	Ywhaq /// LOC546165 /// LOC636369	tyrosine 3-monooxygenase/tryptophan 5-monooxygenase activation protein, theta polypeptide /// similar to 14-3-3 protein theta (14-3-3 protein tau) /// similar to 14-3-3 protein theta (14-3-3 protein tau)	1.0559	0.1988	0.1086	0.4821	No	1.0593	0.279	0.0039	0.846	No	No
97061_g_at	Ywhaq	tyrosine 3-monooxygenase/tryptophan 5-monooxygenase activation protein, theta polypeptide	1.1249	0.1216	0.3174	0.4438	No	1.0574	0.243	0.37	0.213	No	No
97073_at	---	---	1.0161	0.6996	0.0114	0.2901	No	1.0269	0.724	0.323	0.624	No	No
97077_f_at	Rtn3	reticulon 3	0.976	0.5609	0.2442	0.735	No	1.0243	0.555	0.653	0.507	No	No
97080_at	C79562	expressed sequence C79562	1.1165	0.0659	0.0769	0.122	No	1.0815	0.0714	0.965	0.375	No	No
97082_at	p	pink-eyed dilution	1.0109	0.768	0.4743	0.5171	No	1.0388	0.394	0.0118	0.593	No	No
97083_at	Eif2s2	eukaryotic translation initiation factor 2, subunit 2 (beta)	0.8974	0.0156	0.0003	0.1152	No	0.8778	0.0006	0.0004	0.0017	No	No
97084_at	C80993	expressed sequence C80993	0.9288	0.6142	0.0203	0.4686	No	1.0173	0.898	0.0425	0.496	No	No
97085_at	D8Ertd362e	DNA segment, Chr 8, ERATO Doi 362, expressed	1.0089	0.7891	0.2922	0.3867	No	1.0096	0.873	0.348	0.064	No	No
97086_i_at	Psg-ps1	pregnancy specific glycoprotein pseudogene 1	0.9654	0.6909	0.0087	0.9397	No	0.923	0.172	0.0019	0.198	No	No
97087_f_at	Psg-ps1	pregnancy specific glycoprotein pseudogene 1	0.8663	0.0559	0.1369	0.0665	No	0.9477	0.524	0.437	0.641	No	No
97088_at	Ptpn6	Protein tyrosine phosphatase, non-receptor type 6	0.9711	0.7544	0.0375	0.4377	No	0.986	0.752	0.001	0.967	No	No
97089_at	Folh1	folate hydrolase	0.9071	0.1973	0.2999	0.9404	No	0.8452	0.258	0.26	0.572	No	No
97090_at	Tcf20	transcription factor 20	0.9484	0.348	0.4608	0.2222	No	0.9478	0.418	0.141	0.259	No	No
97091_at	Ripk1	receptor (TNFRSF)-interacting serine-threonine kinase 1	0.9136	0.0085	0.1105	0.0693	Yes	0.9368	0.422	0.164	0.711	No	No
97092_at	Mesp2	mesoderm posterior 2	1.0865	0.3837	0.0453	0.7237	No	1.1355	0.169	0.0654	0.735	No	No
97094_at	Phkg1	phosphorylase kinase gamma 1	0.9123	0.2203	0.7394	0.0676	No	0.789	0.0208	0.244	0.0273	No	No
97095_at	Bub1	Budding uninhibited by benzimidazoles 1 homolog (S. cerevisiae)	0.9164	0.2412	0.2558	0.6798	No	1.105	0.116	0.706	0.276	No	No
97096_at	Prkar2a	protein kinase, cAMP dependent regulatory, type II alpha	1.005	0.9228	0.7589	0.5956	No	1.0783	0.0937	0.241	0.0415	No	No
97097_at	Myo9a	myosin IXa	0.948	0.2574	0.0022	0.9425	No	0.9221	0.204	0.0045	0.523	No	No
97098_at	Tha1	threonine aldolase 1	0.93	0.0138	0.0006	0.0033	No	0.9202	0.157	0.542	0.0132	No	No
97099_at	Slc4a8	solute carrier family 4 (anion exchanger), member 8	0.8983	0.28	0.259	0.4435	No	0.9473	0.415	0.0968	0.312	No	No
97100_at	Kcnn1	potassium intermediate/small conductance calcium-activated channel, subfamily N, member 1	1.0436	0.4567	0.0795	0.8041	No	1.005	0.953	0.0938	0.921	No	No
97101_at	Myl6b	Myosin, light polypeptide 6B	0.9864	0.8223	0.7079	0.297	No	1.0298	0.577	0.0492	0.512	No	No
97102_at	Yme1l1	YME1-like 1 (S. cerevisiae)	1.0086	0.8462	0.005	0.7762	No	1.005	0.953	0.0277	0.662	No	No
97103_at	Rfk	riboflavin kinase	1.0018	0.9672	0.1732	0.3623	No	1.0945	0.0695	0.0294	0.0512	No	No
97104_g_at	Rfk	riboflavin kinase	1.0733	0.1673	0.0147	0.4751	No	1.1169	0.0452	0.157	0.514	No	No
97105_at	Zc3h12c	zinc finger CCCH-type containing 12C	1.0109	0.861	0.0017	0.1159	No	0.98	0.997	0.0004	0.154	No	No
97106_at	Map3k8	mitogen activated protein kinase kinase kinase 8	0.9568	0.4131	0.0939	0.1317	No	1.037	0.127	0.0186	0.0553	No	No
97107_at	Mterfd3	MTERF domain containing 3	1.0171	0.6936	0.0139	0.6155	No	0.9992	0.794	0.0007	0.211	No	No
97108_at	Recql5	RecQ protein-like 5	0.9867	0.8488	0.4063	0.2798	No	1	0.965	0.126	0.33	No	No
97109_at	Tpsab1	tryptase alpha/beta 1	0.8176	0.011	0.2425	0.0066	No	0.9339	0.221	0.591	0.0974	No	No
97110_at	---	Transcribed locus	0.9097	0.0882	0.0001	0.9007	No	1.0275	0.874	0.0051	0.435	No	No
97111_at	Trak2	trafficking protein, kinesin binding 2	0.9971	0.9562	0.0024	0.3622	No	1.0439	0.158	0.002	0.0084	No	No
97112_at	Mrvi1	MRV integration site 1	0.9803	0.7944	0.6579	0.8045	No	0.9326	0.378	0.183	0.516	No	No
97113_at	Fasl	Fas ligand (TNF superfamily, member 6)	1.0536	0.3827	0.1921	0.8532	No	0.9512	0.328	0.0539	0.997	No	No
97114_at	Psap	prosaposin	0.897	0.0125	0.0831	0.5603	No	0.9142	0.0036	0.458	0.098	Yes	Yes
97115_at	LOC626877	Similar to zinc finger protein 709	0.9749	0.6079	0.0145	0.4445	No	0.935	0.253	0.0879	0.869	No	No
97116_at	AW544981	expressed sequence AW544981	0.9268	0.2598	0.3753	0.1998	No	1.0468	0.409	0.61	0.974	No	No
97117_at	Dlx1as	distal-less homeobox 1, antisense	0.9455	0.4	0.2807	0.5546	No	1.0389	0.488	0.0227	0.821	No	No
97118_at	A630007B06 Rik /// LOC671029	RIKEN cDNA A630007B06 gene /// similar to oocyte-testis gene 1	0.9647	0.4241	0.0063	0.1638	No	1.1734	0.0254	0.425	0.13	No	No
97119_at	AI596198	expressed sequence AI596198	0.942	0.3893	0.2371	0.5896	No	0.9837	0.83	0.0655	0.991	No	No
97120_at	D7Ertd59e	DNA segment, Chr 7, ERATO Doi 59, expressed	1.0023	0.9436	0.1486	0.1811	No	1.0725	0.124	0.009	0.181	No	No
97121_at	Fgf6	fibroblast growth factor 6	0.9645	0.5017	0.1205	0.3333	No	1.0027	0.982	0.129	0.401	No	No
97122_at	Six5	sine oculis-related homeobox 5 homolog (Drosophila)	0.9374	0.0251	0.6277	0.6572	No	0.9777	0.551	0.0189	0.975	No	No
97123_at	Nr0b2	nuclear receptor subfamily 0, group B, member 2	1.0466	0.3003	0.0062	0.8685	No	0.984	0.681	0.0035	0.197	No	No
97124_at	Dfna5h	deafness, autosomal dominant 5 homolog (human)	0.9466	0.7159	0.0046	0.7169	No	0.9648	0.963	0.0024	0.372	No	No
97125_f_at	LOC56628	MHC (A.CA/J(H-2K-f) class I antigen	0.9336	0.1151	0.0088	0.3379	No	0.9528	0.316	0.0642	0.596	No	No
97126_at	Ryr3	ryanodine receptor 3	1.0305	0.2396	0.003	0.0502	No	0.9834	0.9	0.0452	0.762	No	No
97127_f_at	V2r15	vomeronasal 2, receptor, 15	1.0043	0.8848	0.4525	0.0747	No	0.9429	0.31	0.713	0.0896	No	No
97128_at	Il5ra	interleukin 5 receptor, alpha	1.0185	0.7344	0.477	0.271	No	1.0901	0.661	0.317	0.836	No	No
97129_at	BC052688	cDNA sequence BC052688	1.0415	0.1688	0.8416	0.0624	No	1.1494	0.14	0.83	0.688	No	No
97130_at	Ate1	arginine-tRNA-protein transferase 1	0.9577	0.682	0.0746	0.4381	No	1.1092	0.252	0.0543	0.195	No	No
97131_at	Stk11ip	serine/threonine kinase 11 interacting protein	0.9296	0.0581	0.0531	0.1507	No	1.0634	0.233	0.0007	0.362	No	No
97132_at	Soat2	sterol O-acyltransferase 2	0.9761	0.6866	0.2347	0.7657	No	1.0558	0.349	0.11	0.341	No	No
97133_at	AA673488	Expressed sequence AA673488	1.0918	0.3583	0.7276	0.8009	No	1.0603	0.395	0.548	0.883	No	No
97134_at	AA517864	expressed sequence AA517864	1.0061	0.8877	0.1429	0.2347	No	0.9162	0.372	0.499	0.649	No	No
97135_at	Rfx4	Regulatory factor X, 4 (influences HLA class II expression)	0.995	0.9472	0.0162	0.2212	No	1.035	0.786	0.928	0.341	No	No
97136_at	Trpm7	Transient receptor potential cation channel, subfamily M, member 7	1.0395	0.1375	0.0001	0.0141	No	1.2773	0.108	0.0573	0.244	No	No
97137_at	C77027	expressed sequence C77027	0.9576	0.0078	0.0001	0.0187	Yes	0.8713	0.0549	0.0065	0.788	No	No
97138_at	BC026657	cDNA sequence BC026657	1.0219	0.5684	0.0017	0.1817	No	1.0542	0.255	0.0014	0.309	No	No
97139_at	C77614	EST C77614	0.9709	0.5777	0.1245	0.8503	No	1.0493	0.555	0.527	0.885	No	No
97140_at	D1Ertd75e	DNA segment, Chr 1, ERATO Doi 75, expressed	0.9897	0.8284	0.239	0.2598	No	1.0932	0.262	0.868	0.358	No	No
97141_s_at	D3Ertd229e	DNA segment, Chr 3, ERATO Doi 229, expressed	1.0699	0.1786	0.0639	0.0453	No	1.0902	0.228	0.508	0.041	No	No

97142_at	DXErtd242e	DNA segment, Chr X, ERATO Doi 242, expressed	1.0153	0.3653	0.0034	0.0062	No	1.0461	0.379	0.151	0.267	No	No
97143_at	Ing4	Inhibitor of growth family, member 4	0.993	0.7369	0.3239	0.0531	No	1.0993	0.0431	0.163	0.0651	No	No
97144_at	C78441	expressed sequence C78441	1.088	0.1959	0.9988	0.3973	No	0.9636	0.163	0.011	0.0143	No	No
97145_at	C80197 /// Spfh1	expressed sequence C80197 /// SPFH domain family, member 1	1.1045	0.0317	0.2022	0.0605	No	1.0344	0.12	0.0059	0.0078	No	No
97146_g_at	C80197	expressed sequence C80197	0.9553	0.2035	0.635	0.0548	No	0.8829	0.106	0.225	0.0658	No	No
97147_at	C530030P08Rik	RIKEN cDNA C530030P08 gene	1.0717	0.3687	0.7674	0.393	No	1.1481	0.328	0.958	0.254	No	No
97148_at	Kifc1	Kinesin family member C1	0.9465	0.4556	0.6654	0.8427	No	1.0568	0.636	0.225	0.816	No	No
97149_at	Rax	retina and anterior neural fold homeobox	0.908	0.0432	0.0082	0.0215	No	0.9336	0.0972	0.037	0.0998	No	No
97150_at	Slc10a2	solute carrier family 10, member 2	0.9965	0.9624	0.2463	0.627	No	1.0469	0.761	0.0161	0.669	No	No
97151_at	Gpr27	G protein-coupled receptor 27	1.0804	0.1668	0.7961	0.8517	No	0.9613	0.142	0.651	0.0217	No	No
97152_at	Krtap8-2	keratin associated protein 8-2	1.0423	0.5682	0.7975	0.8097	No	1.005	0.921	0.0629	0.0781	No	No
97153_at	Inhbe	inhibin beta E	0.9183	0.2634	0.0265	0.6554	No	0.9423	0.278	0.0013	0.464	No	No
97154_f_at	MGC117846	Similar to zinc finger protein 665	1.0318	0.3771	0.0062	0.5734	No	0.8581	0.0252	0.337	0.106	No	No
97155_at	Gdf8	growth differentiation factor 8	0.9287	0.0505	0.0005	0.7156	No	0.9955	0.925	0.0037	0.928	No	No
97156_at	Hes3	hairy and enhancer of split 3 (Drosophila)	1.0183	0.6447	0.8768	0.1606	No	1.0626	0.0475	0.0032	0.991	No	No
97157_at	Nkx3-1	NK-3 transcription factor, locus 1 (Drosophila)	1.1164	0.0332	0.308	0.1374	No	1.1167	0.0061	0.001	0.0018	No	No
97158_at	Zfp651	zinc finger protein 651	0.9861	0.6016	0.0038	0.4193	No	1.0446	0.203	0.0069	0.743	No	No
97159_at	Aire	autoimmune regulator (autoimmune polyendocrinopathy candidiasis ectodermal dystrophy)	1.0128	0.742	0.0092	0.0405	No	1.1147	0.133	0.52	0.0479	No	No
97160_at	Sparc	secreted acidic cysteine rich glycoprotein	1.0526	0.6144	0.0181	0.7425	No	0.9956	0.648	0	0.341	No	No
97161_at	Sfpq	splicing factor proline/glutamine rich (polypyrimidine tract binding protein associated)	0.9131	0.1845	0.01	0.8443	No	0.9411	0.443	0.0066	0.983	No	No
97162_at	Ifi203	Interferon activated gene 203	0.9796	0.6558	0.7848	0.9361	No	0.9828	0.742	0.0061	0.98	No	No
97163_g_at	D030022P06Rik	RIKEN cDNA D030022P06 gene	0.9658	0.648	0.5511	0.2008	No	0.9895	0.93	0.164	0.509	No	No
97164_at	Dleu8	deleted in lymphocytic leukemia 8	1.1068	0.0814	0.4496	0.1051	No	1.0621	0.0109	0.0013	0.18	No	No
97165_r_at	D2Ertd93e	DNA segment, Chr 2, ERATO Doi 93, expressed	1.1201	0.1264	0.004	0.0225	No	1.0919	0.0571	0.0007	0.0132	No	No
97166_at	Elac2	elaC homolog 2 (E. coli)	1.0711	0.0031	0.0052	0.0036	No	1.0257	0.128	0.0019	0.2	No	No
97167_g_at	Elac2	elaC homolog 2 (E. coli)	1.0682	0.2042	0.0278	0.0906	No	1.0878	0.0065	0.0014	0.0048	No	No
97168_at	P2ry1	purinergic receptor P2Y, G-protein coupled 1	1.0915	0.3133	0.3422	0.9949	No	1.0592	0.27	0.0082	0.209	No	No
97169_f_at	LOC639910	hypothetical protein LOC639910	1.0449	0.4484	0.6258	0.5886	No	1.02	0.64	0.0177	0.238	No	No
97170_at	C78344	expressed sequence C78344	0.9232	0.0098	0.0264	0.3115	Yes	0.9674	0.707	0.0279	0.587	No	No
97171_f_at	---	Immunoglobulin kappa chain complex	1.0632	0.1498	0.0083	0.6767	No	0.9285	0.114	0.0072	0.0133	No	No
97172_s_at	Abcc9	ATP-binding cassette, sub-family C (CFTR/MRP), member 9	0.8811	0.2227	0.0589	0.8868	No	0.9291	0.449	0.213	0.658	No	No
97173_f_at	H2-Q2 /// LOC630499 /// LOC633617	histocompatibility 2, Q region locus 2 /// similar to H-2 class I histocompatibility antigen, D-K alpha chain precursor (H-2D(K)) /// similar to H-2 class I histocompatibility antigen, D-K alpha chain precursor (H-2D(K))	0.9485	0.2442	0.0016	0.0143	No	0.9429	0.494	0	0.0037	No	No
97174_r_at	2410017I17Rik	RIKEN cDNA 2410017I17 gene	1.0331	0.0312	0	0.0685	No	1.0244	0.603	0.111	0.933	No	No
97175_at	Mocs1	molybdenum cofactor synthesis 1	0.9346	0.3676	0.2168	0.487	No	0.9508	0.41	0.0951	0.0814	No	No
97176_at	Atp8b1	ATPase, class I, type 8B, member 1	0.9745	0.5545	0.0011	0.1232	No	1.0029	0.656	0.0002	0.117	No	No
97177_at	---	ORFs (putative primordial protein)	1.0324	0.3862	0.0199	0.0023	No	0.9755	0.858	0.0072	0.0278	No	No
97178_at	AI314034	expressed sequence AI314034	1.0496	0.1897	0.0004	0.0298	No	0.9586	0.167	0.0009	0.124	No	No
97179_at	BC048355	cDNA sequence BC048355	1.0086	0.6054	0.0057	0.0569	No	1.0231	0.422	0.778	0.0359	No	No
97180_f_at	Hbb-y	hemoglobin Y, beta-like embryonic chain	0.9656	0.0184	0.0022	0.0017	No	0.9995	0.988	0.248	0.458	No	No
97181_f_at	Ccrn4l /// Sgip1 /// LOC280487 /// Gag /// LOC639390	CCR4 carbon catabolite repression 4-like (S. cerevisiae) /// SH3-domain GRB2-like (endophilin) interacting protein 1 /// pol polyprotein /// Gag /// gag protein	0.9426	0.1347	0.0244	0.3028	No	0.9451	0.358	0.0164	0.622	No	No
97182_at	Ccne2	cyclin E2	1.0138	0.7115	0.8986	0.9143	No	0.9663	0.9	0.928	0.895	No	No
97184_at	Tbc1d22a	TBC1 domain family, member 22a	0.9408	0.2985	0.1897	0.7583	No	0.9493	0.307	0.643	0.0601	No	No
97185_at	Arnt	aryl hydrocarbon receptor nuclear translocator	1.0434	0.3722	0.0675	0.0849	No	1.1289	0.155	0.108	0.273	No	No
97186_s_at	Stk25	Serine/threonine kinase 25 (yeast)	0.9815	0.6899	0.6084	0.4348	No	1.0031	0.925	0.547	0.287	No	No
97187_at	D7Wsu130e	DNA segment, Chr 7, Wayne State University 130, expressed	1.0611	0.2447	0.3219	0.8822	No	1.0702	0.118	0.0545	0.0602	No	No
97188_at	AW121686	expressed sequence AW121686	0.9382	0.2782	0.1914	0.2473	No	0.9111	0.391	0.25	0.143	No	No
97189_at	Ube3c	Ubiquitin protein ligase E3C	1.0766	0.2066	0.1914	0.1112	No	1.156	0.0423	0.0105	0.0704	No	No
97190_f_at	---	---	0.9844	0.8083	0.2857	0.1282	No	0.9255	0.885	0.385	0.475	No	No
97191_at	---	---	1.0093	0.8756	0.0649	0.2514	No	0.9123	0.171	0.0276	0.144	No	No
97192_at	---	---	1.0461	0.3703	0.0944	0.1285	No	1.1273	0.0664	0.0203	0.0445	No	No
97193_at	Tcfcp2l1	transcription factor CP2-like 1	1.0387	0.6099	0.2508	0.2264	No	1.0533	0.135	0.699	0.022	No	No
97194_at	AA408251	expressed sequence AA408251	1.0606	0.4312	0.0181	0.5311	No	1.122	0.104	0.0272	0.324	No	No
97195_at	Gnai1	guanine nucleotide binding protein, alpha inhibiting 1	0.8598	0.0464	0.7789	0.8113	No	0.8896	0.0208	0.101	0.0829	No	No
97196_at	Psg19	pregnancy specific glycoprotein 19	0.9788	0.7496	0.8375	0.4147	No	1.142	0.0824	0.0391	0.143	No	No
97197_r_at	Fv4 /// AI506816 /// LOC622147 /// LOC628577 /// Env /// LOC664798 /// LOC666404 /// LOC668227 /// LOC668269 /// LOC669098 /// LOC669176 /// LOC669658 /// LOC669821 /// LOC670510 /// LOC671760 /// LOC672016	Friend virus susceptibility 4 /// expressed sequence AI506816 /// similar to melanoma antigen /// similar to melanoma antigen /// envelope polyprotein /// similar to melanoma antigen /// similar to melanoma antigen /// similar to melanoma antigen /// similar to melanoma antigen /// similar to melanoma antigen /// similar to melanoma antigen /// similar to melanoma antigen /// hypothetical protein LOC670510 /// similar to melanoma antigen /// similar to melanoma antigen /// similar to melanoma antigen	1.004	0.9301	0.0365	0.0071	No	1.0716	0.988	0.0004	0.0003	No	No
97198_at	Abca1	ATP-binding cassette, sub-family A (ABC1), member 1	0.8201	0.0348	0.0059	0.1274	No	0.8887	0.113	0.0014	0.0431	No	No
97199_at	Rbm12 /// Cpne1	RNA binding motif protein 12 /// copine I	0.9553	0.2548	0.1412	0.4	No	0.8781	0.0759	0.264	0.486	No	No
97200_f_at	Snrpe	small nuclear ribonucleoprotein E	1.1268	0.1345	0.2986	0.6981	No	1.1583	0.03	0.375	0.349	No	No
97201_s_at	Ndufa5	NADH dehydrogenase (ubiquinone) 1 alpha subcomplex, 5	1.0399	0.4745	0.3437	0.1303	No	1.0166	0.23	0.0003	0.0063	No	No
97203_at	Marcksl1	MARCKS-like 1	1.0626	0.3115	0.6625	0.6445	No	1.0484	0.166	0.322	0.503	No	No
97204_s_at	Dnajc15	DnaJ (Hsp40) homolog, subfamily C, member 15	1.0488	0.1603	0.1005	0.3092	No	1.0094	0.739	0.0143	0.237	No	No
97205_at	Eif3s1	eukaryotic translation initiation factor 3, subunit 1 alpha	1.0362	0.6835	0.0394	0.2127	No	1.0444	0.701	0.0705	0.276	No	No
97206_at	Spint1	serine protease inhibitor, Kunitz type 1	1.0365	0.4811	0.3275	0.3642	No	1.1341	0.0019	0.003	0.0603	Yes	No
97207_f_at	Lypla1	lysophospholipase 1	1.0848	0.0499	0.0001	0.3483	No	1.0866	0.134	0.0004	0.597	No	No
97208_at	Ash2l	ash2 (absent, small, or homeotic)-like (Drosophila)	0.9518	0.2482	0.8758	0.2206	No	0.9085	0.0152	0.173	0.104	No	No
97210_at	1700037H04Rik	RIKEN cDNA 1700037H04 gene	1.0055	0.8935	0.1702	0.213	No	0.9919	0.69	0.491	0.0969	No	No
97211_at	Armcx2	armadillo repeat containing, X-linked 2	1.0454	0.0662	0.1655	0.1375	No	1.0285	0.318	0.0041	0.986	No	No
97213_at	Fem1a	feminization 1 homolog a (C. elegans)	0.846	0.0231	0.8583	0.0452	No	0.8908	0.0365	0.213	0.0123	No	No
97216_at	Pzp	pregnancy zone protein	1.0388	0.5118	0.4804	0.44	No	1.1332	0.107	0.0024	0.184	No	No
97217_at	Ahcyl1	S-adenosylhomocysteine hydrolase-like 1	0.982	0.6985	0.0519	0.519	No	0.9575	0.308	0.0255	0.951	No	No
97220_at	Dscr2	Down syndrome critical region homolog 2 (human)	0.9745	0.5416	0.0718	0.0593	No	0.9471	0.166	0.0028	0.0079	No	No

97222_at	Rab6	RAB6, member RAS oncogene family	1.028	0.5618	0.0126	0.5661	No	0.988	0.699	0.0029	0.447	No	No
97224_at	Pnrc1	proline-rich nuclear receptor coactivator 1	0.8978	0.1415	0.8821	0.704	No	0.7937	0.0936	0.446	0.416	No	No
97226_at	Gna12	guanine nucleotide binding protein, alpha 12	0.995	0.9436	0.0195	0.998	No	1.0141	0.817	0.0073	0.638	No	No
97227_at	Gna12	guanine nucleotide binding protein, alpha 12	1.0981	0.1371	0.0119	0.7993	No	1.0428	0.229	0.0017	0.382	No	No
97228_at	Rogdi	rogdi homolog (Drosophila)	1.0223	0.4824	0.1234	0.692	No	1.0005	0.986	0.0671	0.29	No	No
97229_at	5730427N09Rik	RIKEN cDNA 5730427N09 gene	0.9853	0.7946	0.4658	0.4088	No	0.9556	0.488	0.629	0.525	No	No
97232_at	Gfm2	G elongation factor, mitochondrial 2	0.9719	0.5372	0.3013	0.7213	No	1.0607	0.509	0.284	0.161	No	No
97234_at	Apobec2	apolipoprotein B editing complex 2	0.9729	0.6627	0.9969	0.8564	No	0.9113	0.0668	0.0503	0.844	No	No
97235_f_at	Apobec2	apolipoprotein B editing complex 2	0.8366	0.1303	0.1106	0.0105	No	0.9101	0.147	0.0327	0.0059	No	No
97236_r_at	Apobec2	apolipoprotein B editing complex 2	1.029	0.5066	0.0208	0.2809	No	1.0158	0.96	0.0109	0.197	No	No
97237_at	1810003N24Rik	RIKEN cDNA 1810003N24 gene	1.1733	0.1215	0.0094	0.7286	No	1.1579	0.106	0.0037	0.364	No	No
97238_at	Tacc3	transforming, acidic coiled-coil containing protein 3	1.0437	0.4553	0.1226	0.5384	No	1.1598	0.137	0.0246	0.153	No	No
97239_at	D19Ertd721e	DNA segment, Chr 19, ERATO Doi 721, expressed	1.1243	0.0001	0	0.0001	No	1.1094	0.135	0.0026	0.448	No	No
97240_g_at	D19Ertd721e	DNA segment, Chr 19, ERATO Doi 721, expressed	1.031	0.5654	0.0127	0.3448	No	1.0311	0.508	0.0132	0.507	No	No
97241_at	D19Ertd721e	DNA segment, Chr 19, ERATO Doi 721, expressed	0.9943	0.9259	0.4169	0.6147	No	1.0471	0.399	0.591	0.575	No	No
97242_at	0610010O12Rik	RIKEN cDNA 0610010O12 gene	0.9325	0.2444	0.6688	0.4719	No	0.9762	0.254	0.0128	0.0173	No	No
97243_at	Slc9a3r1	solute carrier family 9 (sodium/hydrogen exchanger), isoform 3 regulator 1	0.988	0.7403	0.6577	0.565	No	1.1229	0.0543	0.396	0.0717	No	No
97247_at	Isoc1	Isochorismatase domain containing 1	1.0165	0.7243	0.4935	0.254	No	1.0561	0.148	0.0668	0.008	No	No
97248_at	Dbi	diazepam binding inhibitor	1.1736	0.0043	0.0053	0.527	Yes	1.0929	0.0199	0.0002	0.324	No	Yes
97249_at	Exosc5	exosome component 5	0.979	0.4381	0.0006	0.4234	No	1.0176	0.615	0.0568	0.211	No	No
97250_at	Nola3	nucleolar protein family A, member 3	1.0362	0.2173	0.0223	0.0939	No	1.0374	0.272	0.0037	0.531	No	No
97251_at	Mrps10	mitochondrial ribosomal protein S10	1.0486	0.2024	0.0023	0.9245	No	1.0606	0.11	0.0012	0.793	No	No
97252_at	2610012O22Rik	RIKEN cDNA 2610012O22 gene	0.9913	0.8113	0.0394	0.1	No	1.0589	0.0139	0.0002	0.605	No	No
97253_at	Tpd52l2	tumor protein D52-like 2	1.0988	0.2476	0.5559	0.9832	No	1.0948	0.153	0.0154	0.464	No	No
97254_at	Rbm8a	RNA binding motif protein 8a	1.0928	0.0081	0.1082	0.4124	Yes	1.2031	0.0232	0.263	0.906	No	Yes
97255_at	Cugbp2	CUG triplet repeat, RNA binding protein 2	0.9485	0.6295	0.0054	0.997	No	0.9632	0.68	0.612	0.0863	No	No
97256_at	Ndufa12	NADH dehydrogenase (ubiquinone) 1 alpha subcomplex, 12	1.0822	0.4714	0.0819	0.4706	No	1.233	0.0195	0.039	0.0486	No	No
97257_at	---	Transcribed locus	1.0251	0.6501	0.0189	0.8562	No	0.9431	0.751	0.006	0.421	No	No
97258_at	Lactb2	lactamase, beta 2	0.9804	0.7589	0.2029	0.7036	No	0.9202	0.0347	0.0001	0.0253	No	No
97259_at	Arpp19	cAMP-regulated phosphoprotein 19	1.1306	0.1189	0.1803	0.8609	No	0.8988	0.265	0.0077	0.346	No	No
97260_at	Arpp19	cAMP-regulated phosphoprotein 19	1.0155	0.2974	0.0009	0.0015	No	0.982	0.787	0.0092	0.54	No	No
97261_at	Dnaja1	DnaJ (Hsp40) homolog, subfamily A, member 1	1.1145	0.0312	0.0023	0.7037	No	1.0584	0.111	0.0029	0.404	No	No
97262_at	Csnk1d	casein kinase 1, delta	0.9509	0.1825	0.0183	0.7848	No	0.9477	0.0804	0.494	0.534	No	No
97263_s_at	Csnk1d	casein kinase 1, delta	1.0133	0.6579	0.405	0.0278	No	1.0134	0.701	0.243	0.0535	No	No
97264_r_at	Csnk1d	casein kinase 1, delta	0.9678	0.3765	0.0015	0.1234	No	1.019	0.418	0.0027	0.856	No	No
97265_at	1810013D10Rik	RIKEN cDNA 1810013D10 gene	0.9831	0.7473	0.9411	0.6084	No	0.9331	0.184	0.428	0.57	No	No
97267_at	6330407G11Rik	RIKEN cDNA 6330407G11 gene	1.0098	0.811	0.0917	0.77	No	0.9965	0.88	0.0259	0.468	No	No
97268_i_at	Krtcap2	keratinocyte associated protein 2	1.0173	0.6926	0.0017	0.3145	No	1.0529	0.18	0.0005	0.449	No	No
97269_f_at	Krtcap2	keratinocyte associated protein 2	1.0362	0.4182	0.0004	0.0103	No	0.9839	0.638	0.0005	0.0242	No	No
97270_at	Nxn	nucleoredoxin	0.9848	0.7821	0.5891	0.375	No	0.9867	0.653	0.0233	0.24	No	No
97271_at	Lsmd1	LSM domain containing 1	1.1077	0.1204	0.0032	0.079	No	1.0423	0.236	0.0004	0.106	No	No
97272_at	LOC432864 /// LOC544954 /// LOC672424	hypothetical LOC432864 /// similar to Heterogeneous nuclear ribonucleoprotein A1 (Helix-destabilizing protein) (Single-strand binding protein) (hnRNP core protein A1) (HDP-1) (Topoisomerase-inhibitor suppressed) /// similar to Heterogeneous nuclear ribonucleoprotein A1 (Helix-destabilizing protein) (Single-strand binding protein) (hnRNP core protein A1) (HDP-1) (Topoisomerase-inhibitor suppressed)	0.9805	0.7402	0.0153	0.9289	No	0.9212	0.218	0.0163	0.741	No	No
97273_at	Ars2	arsenate resistance protein 2	1.0477	0.3282	0.0027	0.3854	No	1.025	0.498	0.0028	0.286	No	No
97274_at	Psmd14	proteasome (prosome, macropain) 26S subunit, non-ATPase, 14	1.1178	0.1814	0.1021	0.3705	No	1.0938	0.38	0.161	0.269	No	No
97276_at	Ckap1	cytoskeleton-associated protein 1	1.1057	0.0306	0.0034	0.0794	No	1.1227	0.105	0.0167	0.698	No	No
97277_at	Triap1	TP53 regulated inhibitor of apoptosis 1	0.991	0.8674	0.2479	0.2671	No	1.0592	0.163	0.0033	0.585	No	No
97278_at	Wbscr1	Williams-Beuren syndrome chromosome region 1 homolog (human)	1.1103	0.0468	0.0649	0.0147	No	1.0732	0.5	0.262	0.138	No	No
97279_at	Hibadh	3-hydroxyisobutyrate dehydrogenase	0.9866	0.7956	0.0502	0.7052	No	0.9859	0.715	0.295	0.352	No	No
97281_at	LOC640502	similar to UDP-N-acetylhexosamine pyrophosphorylase	0.9045	0.1188	0.5551	0.2633	No	0.9903	0.648	0.0007	0.422	No	No
97282_at	Mela	melanoma antigen	0.9987	0.9725	0.0918	0.338	No	0.9939	0.949	0.493	0.0717	No	No
97283_at	Dppa3	developmental pluripotency-associated 3	1.0302	0.7146	0.2126	0.647	No	1.1014	0.275	0.509	0.266	No	No
97284_at	Bcl2l13	BCL2-like 13 (apoptosis facilitator)	1.0073	0.8138	0.495	0.1015	No	1.0511	0.223	0.0817	0.0303	No	No
97285_f_at	Ubxd1	UBX domain containing 1	0.9424	0.402	0.0177	0.1776	No	0.8427	0.304	0.0317	0.547	No	No
97287_at	Pdzk1	PDZ domain containing 1	0.9772	0.4126	0.6726	0.1344	No	0.9565	0.421	0.766	0.0659	No	No
97288_at	Pdzk1	PDZ domain containing 1	1.108	0.2041	0.0219	0.031	No	1.04	0.428	0.0282	0.0359	No	No
97292_at	---	---	1.0264	0.5787	0.0058	0.1479	No	0.9828	0.721	0.0125	0.312	No	No
97293_at	Rbm10	RNA binding motif protein 10	0.9088	0.0185	0.0012	0.1634	No	0.9021	0.0109	0.0007	0.0059	No	No
97295_at	Cdca8	cell division cycle associated 8	1.0769	0.0343	0.279	0.0083	No	1.0942	0.032	0.544	0.0041	No	No
97296_at	Mrpl44	mitochondrial ribosomal protein L44	0.8413	0.0775	0.2054	0.4627	No	0.8345	0.0326	0.0582	0.313	No	No
97297_at	Pcp4l1	Purkinje cell protein 4-like 1	1.0256	0.4337	0.7807	0.4013	No	1.0227	0.706	0.0501	0.621	No	No
97300_at	Flna	filamin, alpha	1.0397	0.6368	0.1264	0.173	No	1.2466	0.0792	0.804	0.175	No	No
97301_at	Rab14	RAB14, member RAS oncogene family	1.0345	0.539	0.0141	0.8933	No	0.9494	0.198	0.0331	0.833	No	No
97302_at	Ivns1abp	influenza virus NS1A binding protein	1.0786	0.4568	0.1874	0.927	No	0.971	0.609	0.0023	0.117	No	No
97304_at	Ubp1	upstream binding protein 1	0.989	0.7933	0.0127	0.8913	No	0.995	0.93	0.824	0.833	No	No
97305_at	Nip7	nuclear import 7 homolog (S. cerevisiae)	1.1063	0.0738	0.0197	0.0247	No	1.0829	0.172	0.0016	0.0058	No	No
97307_f_at	Ndufb5	NADH dehydrogenase (ubiquinone) 1 beta subcomplex, 5	1.0331	0.3732	0.0182	0.061	No	1.0321	0.564	0.121	0.0618	No	No
97308_at	Tmem11	transmembrane protein 11	1.0031	0.8873	0.0044	0.0669	No	1.0214	0.44	0.0146	0.0315	No	No
97309_at	St13	suppression of tumorigenicity 13	1.0396	0.5859	0.3606	0.6103	No	0.9101	0.0179	0.059	0.0294	No	No
97310_at	St13	suppression of tumorigenicity 13	0.9733	0.5825	0.0064	0.387	No	0.9892	0.734	0.0006	0.707	No	No
97311_at	6720456B07Rik	RIKEN cDNA 6720456B07 gene	0.9778	0.5929	0.0134	0.0441	No	1.0064	0.993	0.0032	0.16	No	No
97312_at	Cd164	CD164 antigen	1.0959	0.1198	0.006	0.1702	No	1.0346	0.305	0.18	0.107	No	No
97313_at	Gdi1	guanosine diphosphate (GDP) dissociation inhibitor 1	1.0021	0.9392	0.0029	0.7051	No	0.9872	0.721	0.0117	0.578	No	No
97315_at	Polr2d	polymerase (RNA) II (DNA directed) polypeptide D	1.07	0.1596	0.3972	0.0605	No	1.0968	0.0236	0.004	0.166	No	No
97316_at	Ehhadh	enoyl-Coenzyme A, hydratase/3-hydroxyacyl Coenzyme A dehydrogenase	1.0282	0.43	0.0507	0.0668	No	0.9969	0.957	0.0021	0.934	No	No
97317_at	Enpp2	ectonucleotide pyrophosphatase/phosphodiesterase 2	1.0332	0.5811	0.898	0.8864	No	1.0324	0.468	0.38	0.979	No	No
97318_at	Hars2	histidyl tRNA synthetase 2	1.0826	0.1643	0.0325	0.5894	No	1.1102	0.0812	0.0101	0.983	No	No
97319_at	Rrad	Ras-related associated with diabetes	1.0209	0.6472	0.2451	0.4683	No	1.076	0.172	0.425	0.805	No	No
97320_at	Slc39a4	solute carrier family 39 (zinc transporter), member 4	1.0322	0.4848	0.3169	0.6148	No	1.0544	0.275	0.911	0.567	No	No
97322_at	Ms4a6b	membrane-spanning 4-domains, subfamily A, member 6B	0.8035	0.0092	0.0078	0.9509	Yes	0.8492	0.0131	0.0009	0.472	No	Yes
97324_at	5133401N09Rik	RIKEN cDNA 5133401N09 gene	0.9671	0.3828	0.1863	0.2066	No	1.0676	0.0233	0.292	0.218	No	No
97325_at	Pxk	PX domain containing serine/threonine kinase	0.8931	0.0045	0.0498	0.1835	Yes	0.9361	0.0528	0.0049	0.964	No	No
97327_at	Fen1	flap structure specific endonuclease 1	1.1906	0.0459	0.0219	0.2535	No	1.2012	0.0156	0.0502	0.286	No	No
97328_at	Mark4	MAP/microtubule affinity-regulating kinase 4	1.0325	0.4911	0.0459	0.0987	No	1.0952	0.2	0.233	0.19	No	No
97329_at	Ints3	integrator complex subunit 3	0.9374	0.127	0.0035	0.3238	No	0.9317	0.33	0.063	0.958	No	No
97330_at	Abcf1	ATP-binding cassette, sub-family F (GCN20), member 1	0.9351	0.2594	0.6441	0.4355	No	0.9161	0.29	0.0013	0.268	No	No
97331_at	Capn10	calpain 10	0.9546	0.0635	0.0059	0.7266	No	0.9634	0.364	0.983	0.773	No	No
97332_at	Pnkp	polynucleotide kinase 3'- phosphatase	0.9659	0.0055	0.0001	0.0009	No	0.9544	0.246	0.155	0.296	No	No
97333_at	Dync1h1	dynein cytoplasmic 1 heavy chain 1	0.8851	0.0223	0.0008	0.5957	No	0.9147	0.015	0.0002	0.929	No	No
97334_at	Hes6	hairy and enhancer of split 6 (Drosophila)	0.9972	0.9594	0.107	0.2495	No	0.9722	0.67	0.0475	0.118	No	No
97335_at	Timd2	T-cell immunoglobulin and mucin domain containing 2	0.9862	0.7682	0.1092	0.8362	No	1.1364	0.484	0.174	0.125	No	No

97336_at	Ctsf	cathepsin F	0.9236	0.1062	0.0682	0.0887	No	0.9383	0.201	0.0754	0.183	No	No
97338_at	Rprc1	arginine/proline rich coiled-coil 1	1.1281	0.0055	0.0009	0.0029	No	1.2369	0.0073	0.0283	0.0061	No	No
97339_at	Git1	G protein-coupled receptor kinase-interactor 1	0.994	0.916	0.2619	0.5681	No	0.991	0.736	0.0027	0.408	No	No
97340_at	Sart3	squamous cell carcinoma antigen recognized by T-cells 3	1.0201	0.2246	0.0139	0.043	No	1.062	0.415	0.155	0.993	No	No
97341_at	Praf2	PRA1 domain family 2	1.1108	0.0898	0.0079	0.5661	No	1.0889	0.25	0.0484	0.883	No	No
97342_at	Mrps14	mitochondrial ribosomal protein S14	1.0437	0.2688	0.0042	0.5419	No	1.0835	0.0702	0.0013	0.442	No	No
97343_at	Bcdin3	bin3, bicoid-interacting 3, homolog (Drosophila)	0.9977	0.9028	0.3236	0.8439	No	1.052	0.214	0.112	0.881	No	No
97345_at	Anp32e	acidic (leucine-rich) nuclear phosphoprotein 32 family, member E	1.0246	0.5791	0.0347	0.3209	No	1.2632	0.0282	0.0107	0.841	No	No
97346_at	2610001J05 Rik	RIKEN cDNA 2610001J05 gene	1.1113	0.0678	0.0227	0.11	No	1.001	0.92	0.0133	0.836	No	No
97347_at	Ltbp4	latent transforming growth factor beta binding protein 4	0.986	0.714	0.0024	0.2182	No	1.0562	0.479	0.0062	0.191	No	No
97349_at	Frmd6	FERM domain containing 6	1.0153	0.7099	0.001	0.0073	No	0.9432	0.674	0.0021	0.0376	No	No
97351_i_at	Cox6b2	cytochrome c oxidase subunit VIb polypeptide 2	1.0897	0.0991	0.3782	0.5571	No	1.0377	0.73	0.455	0.377	No	No
97352_f_at	Cox6b2	cytochrome c oxidase subunit VIb polypeptide 2	1.0594	0.2399	0.5185	0.2128	No	1.0313	0.421	0.0895	0.0424	No	No
97353_at	Dab2ip	disabled homolog 2 (Drosophila) interacting protein	0.9232	0.0091	0.0002	0.0175	Yes	0.9353	0.332	0.0917	0.287	No	No
97354_at	Gtf3c1	general transcription factor III C 1	1.0227	0.2054	0.0268	0.0855	No	1.1241	0.0878	0.0997	0.0361	No	No
97355_at	Alkbh5	alkB, alkylation repair homolog 5 (E. coli)	0.9803	0.4943	0.012	0.0614	No	1.0342	0.676	0.669	0.112	No	No
97356_at	1810008O21 Rik	RIKEN cDNA 1810008O21 gene	1.0202	0.3194	0.9061	0.1482	No	1.0029	0.9	0.0222	0.428	No	No
97357_at	Mef2c	myocyte enhancer factor 2C	1.0214	0.8815	0.0323	0.354	No	0.9997	0.964	0.0181	0.989	No	No
97358_at	Lphn1	latrophilin 1	1.0693	0.1227	0.0556	0.2277	No	1.0614	0.0731	0.003	0.0338	No	No
97359_at	Tm9sf1	transmembrane 9 superfamily member 1	1.0556	0.0502	0.0008	0.9472	No	1.0092	0.856	0.0375	0.587	No	No
97360_at	Cmtm3	CKLF-like MARVEL transmembrane domain containing 3	1.0243	0.6501	0.3921	0.9474	No	1.0163	0.93	0.663	0.937	No	No
97363_at	Ptk2	PTK2 protein tyrosine kinase 2	0.9709	0.4202	0.1947	0.4902	No	0.9242	0.0567	0.17	0.279	No	No
97364_at	Asf1b	ASF1 anti-silencing function 1 homolog B (S. cerevisiae)	1.0973	0.0937	0.0252	0.4001	No	1.0557	0.269	0.005	0.164	No	No
97365_at	Coro2b	coronin, actin binding protein, 2B	0.9952	0.9219	0.4742	0.0399	No	1.0301	0.65	0.253	0.554	No	No
97366_at	Lemd2	LEM domain containing 2	0.9508	0.1178	0.4772	0.394	No	0.9782	0.499	0.03	0.201	No	No
97367_at	Akap1	A kinase (PRKA) anchor protein 1	1.022	0.7791	0.2324	0.3519	No	1.0326	0.433	0.005	0.0671	No	No
97368_at	Akap1	A kinase (PRKA) anchor protein 1	0.9708	0.2686	0.3455	0.1042	No	1.0049	0.836	0.1	0.177	No	No
97369_g_at	Akap1	A kinase (PRKA) anchor protein 1	0.9737	0.668	0.5031	0.6505	No	0.9697	0.541	0.642	0.851	No	No
97370_at	Spryd4	SPRY domain containing 4	0.9925	0.8328	0.5215	0.2221	No	1.0067	0.862	0.024	0.254	No	No
97371_at	2310022K01 Rik	RIKEN cDNA 2310022K01 gene	0.9954	0.9189	0.2244	0.859	No	0.968	0.379	0.837	0.0958	No	No
97372_at	Dazap1	DAZ associated protein 1	1.1023	0.0527	0.052	0.016	No	1.0622	0.236	0.0099	0.0432	No	No
97373_at	Slc25a19	solute carrier family 25 (mitochondrial deoxynucleotide carrier), member 19	1.0273	0.4107	0.011	0.3279	No	0.987	0.671	0.159	0.0705	No	No
97374_at	2810025M15 Rik	RIKEN cDNA 2810025M15 gene	0.9522	0.2297	0.0181	0.1416	No	0.9414	0.172	0.556	0.445	No	No
97375_at	Pkd1	polycystic kidney disease 1 homolog	0.9549	0.4367	0.1129	0.0687	No	0.9181	0.055	0.0049	0.262	No	No
97377_at	Coil	coilin	1.0096	0.8164	0.5748	0.1051	No	1.0742	0.0677	0.004	0.346	No	No
97379_at	Fbp2	fructose bisphosphatase 2	0.9362	0.3702	0.9348	0.515	No	0.9798	0.705	0.341	0.444	No	No
97380_at	2700069A02 Rik	RIKEN cDNA 2700069A02 gene	1.0216	0.7651	0.7151	0.9259	No	0.9937	0.913	0.312	0.644	No	No
97381_s_at	Tcp11	t-complex protein 11	0.9744	0.5542	0.6764	0.7476	No	1.0153	0.635	0.331	0.082	No	No
97382_at	Tcp11	t-complex protein 11	0.9786	0.4389	0.0995	0.4842	No	0.8934	0.105	0.132	0.971	No	No
97383_at	Slc6a6	solute carrier family 6 (neurotransmitter transporter, taurine), member 6	0.896	0.0561	0.3618	0.9102	No	0.8669	0.0081	0.198	0.454	Yes	No
97384_at	Gmfg	glia maturation factor, gamma	0.9501	0.1658	0.5922	0.0567	No	0.9771	0.44	0.782	0.0371	No	No
97385_at	Nagk	N-acetylglucosamine kinase	1.0353	0.4697	0.3072	0.2856	No	1.0184	0.869	0.187	0.123	No	No
97386_at	Zfp703	zinc finger protein 703	0.9665	0.1969	0.0008	0.8887	No	1.0886	0.176	0.0023	0.282	No	No
97387_at	D1Bwg1363e	DNA segment, Chr 1, Brigham & Women's Genetics 1363 expressed	1.0382	0.0719	0	0.2498	No	1.0061	0.682	0.0001	0.131	No	No
97389_at	Cdc25a	cell division cycle 25 homolog A (S. cerevisiae)	1.0816	0.0903	0.3073	0.0368	No	1.0961	0.0082	0.274	0.0563	Yes	No
97390_at	Cdc25a	cell division cycle 25 homolog A (S. cerevisiae)	1.1031	0.0647	0.0195	0.2884	No	1.1795	0.0503	0.132	0.31	No	No
97391_at	D14Ertd500e	DNA segment, Chr 14, ERATO Doi 500, expressed	0.9494	0.341	0.6463	0.9723	No	1.0064	0.903	0.0319	0.237	No	No
97392_at	Tmem129	transmembrane protein 129	1.0005	0.9822	0.0243	0.0875	No	0.9874	0.735	0.0056	0.928	No	No
97393_at	Vrk1	vaccinia related kinase 1	0.8873	0.2198	0.2005	0.1831	No	0.9384	0.583	0.111	0.101	No	No
97394_at	Smarca5 /// 9230115A19 Rik /// LOC675704	SWI/SNF related, matrix associated, actin dependent regulator of chromatin, subfamily a, member 5 /// RIKEN cDNA 9230115A19 gene /// similar to SWI/SNF related, matrix associated, actin dependent regulator of chromatin, subfamily a, member 5	0.9773	0.8287	0.0247	0.4269	No	0.9962	0.944	0.0315	0.112	No	No
97395_at	Prpf19	PRP19/PSO4 pre-mRNA processing factor 19 homolog (S. cerevisiae)	1.0146	0.813	0.5317	0.2809	No	1.0527	0.263	0.0309	0.419	No	No
97397_at	Coq5	coenzyme Q5 homolog, methyltransferase (yeast)	0.9386	0.1578	0.8355	0.0553	No	1.021	0.173	0.0001	0.566	No	No
97398_at	Pck2	phosphoenolpyruvate carboxykinase 2 (mitochondrial)	0.9361	0.2975	0.0493	0.3295	No	0.9059	0.125	0.613	0.0587	No	No
97400_at	1110039B18 Rik	RIKEN cDNA 1110039B18 gene	0.9533	0.1188	0.0025	0.3681	No	0.9631	0.535	0.014	0.872	No	No
97401_at	Josd1	Josephin domain containing 1	0.919	0.1143	0.2421	0.0283	No	0.8668	0.0012	0.466	0.0043	No	No
97402_at	Inmt	indolethylamine N-methyltransferase	0.9509	0.5402	0.9136	0.456	No	1.0602	0.184	0.662	0.0607	No	No
97403_at	Tsfm	Ts translation elongation factor, mitochondrial	0.9739	0.2521	0.0884	0.4784	No	1.0039	0.96	0.161	0.107	No	No
97404_at	1500034J01 Rik	RIKEN cDNA 1500034J01 gene	1.1192	0.1396	0.1344	0.5737	No	1.1015	0.338	0.432	0.906	No	No
97405_at	Rps6ka1	ribosomal protein S6 kinase polypeptide 1	1.0249	0.7397	0.2692	0.9868	No	0.9125	0.034	0.0031	0.0103	No	No
97406_at	Cbfa2t2h	core-binding factor, runt domain, alpha subunit 2, translocated to, 2 homolog (human)	1.0383	0.159	0.0136	0.1819	No	1.0212	0.572	0.262	0.94	No	No
97407_at	Ubadc1	ubiquitin associated domain containing 1	0.8888	0.0281	0.7255	0.0092	No	0.8951	0.0485	0.742	0.0559	No	No
97409_at	Irgm	immunity-related GTPase family, M	0.9554	0.3957	0.0597	0.1847	No	0.9289	0.408	0.408	0.578	No	No
97410_at	Mcart1	mitochondrial carrier triple repeat 1	1.051	0.3881	0.5134	0.7394	No	0.9921	0.81	0.0224	0.718	No	No
97411_at	Ect2	ect2 oncogene	0.9971	0.9539	0.0016	0.0937	No	1.0415	0.185	0.0004	0.0746	No	No
97412_at	3300001G02 Rik	RIKEN cDNA 3300001G02 gene	1.0433	0.0839	0.0074	0.39	No	0.9593	0.42	0.687	0.24	No	No
97413_at	1600029D21 Rik	RIKEN cDNA 1600029D21 gene	1.0534	0.3492	0.7965	0.1851	No	0.9591	0.513	0.352	0.73	No	No
97414_at	Noc4l	nucleolar complex associated 4 homolog (S. cerevisiae)	1.0167	0.6017	0.5084	0.0702	No	0.9875	0.387	0.588	0.013	No	No
97415_at	Rab3d	RAB3D, member RAS oncogene family	1.0806	0.0148	0.3264	0.102	No	1.1096	0.0006	0.0013	0.0015	No	No
97418_at	Bhmt2	betaine-homocysteine methyltransferase 2	1.0268	0.5123	0.7934	0.0969	No	1.2395	0.0829	0.168	0.06	No	No
97419_at	Commd7 /// LOC631742 /// LOC674161	COMM domain containing 7 /// similar to COMM domain containing protein 7 /// similar to COMM domain-containing protein 7	1.0631	0.1364	0.5022	0.695	No	1.0522	0.171	0.257	0.0364	No	No
97420_at	Lrg1	leucine-rich alpha-2-glycoprotein 1	0.8262	0.0365	0.3011	0.1146	No	0.9792	0.296	0.164	0.333	No	No
97421_at	Smc2l1	SMC2 structural maintenance of chromosomes 2-like 1 (yeast)	1.1067	0.3291	0.0131	0.0896	No	0.967	0.728	0.0031	0.0282	No	No
97422_at	Iars2	isoleucine-tRNA synthetase 2, mitochondrial	0.8705	0.018	0.7524	0.0251	No	1.0081	0.876	0.35	0.426	No	No
97423_at	1500035H01 Rik	RIKEN cDNA 1500035H01 gene	0.9443	0.2477	0.0032	0.3213	No	0.9593	0.553	0.0039	0.454	No	No
97424_at	Arl6ip5	ADP-ribosylation factor-like 6 interacting protein 5	0.9213	0.2962	0.1642	0.1127	No	0.901	0.163	0.0173	0.322	No	No
97425_at	2210409B22 Rik	RIKEN cDNA 2210409B22 gene	0.9886	0.6886	0.01	0.7353	No	0.9613	0.231	0.001	0.617	No	No
97426_at	Emp1	epithelial membrane protein 1	1.0199	0.3717	0.0208	0.1162	No	0.9638	0.104	0.688	0.724	No	No
97427_at	Mbl2	mannose binding lectin (C)	1.03	0.4435	0.0165	0.7113	No	0.8534	0.325	0.005	0.794	No	No
97428_at	Dom3z	DOM-3 homolog Z (C. elegans)	0.9943	0.8523	0.0851	0.3942	No	1.1102	0.111	0.951	0.681	No	No
97429_at	Snrk	SNF related kinase	0.8994	0.0979	0.3994	0.7408	No	0.9127	0.0624	0.0034	0.137	No	No
97430_at	Slc37a4	solute carrier family 37 (glycerol-6-phosphate transporter), member 4	0.9298	0.0163	0.0427	0.0859	No	1.0583	0.153	0.0393	0.338	No	No
97431_at	Slc22a6	solute carrier family 22 (organic anion transporter), member 6	0.9281	0.4205	0.4061	0.314	No	1.0572	0.692	0.0119	0.0104	No	No
97433_at	Mcm3ap	minichromosome maintenance deficient 3 (S. cerevisiae) associated protein	0.9699	0.4495	0.1687	0.6526	No	1.1338	0.0076	0.96	0.106	Yes	No
97434_at	Nsun4	NOL1/NOP2/Sun domain family, member 4	1.1127	0.1572	0.0493	0.4887	No	1.0541	0.492	0.921	0.289	No	No
97435_at	F13b	coagulation factor XIII, beta subunit	0.9849	0.8515	0.329	0.9648	No	1.0062	0.916	0.527	0.329	No	No
97436_at	Man2c1	mannosidase, alpha, class 2C, member 1	0.9487	0.2548	0.0018	0.4533	No	0.9825	0.853	0.0051	0.348	No	No

97437_f_at	Ankib1	ankyrin repeat and IBR domain containing 1	1.0197	0.8042	0.0028	0.9377	No	1.042	0.674	0.0018	0.719	No	No
97438_r_at	Ankib1	ankyrin repeat and IBR domain containing 1	1.0618	0.3651	0.0089	0.9235	No	1.0891	0.0256	0.0001	0.732	No	No
97441_at	Acin1	apoptotic chromatin condensation inducer 1	0.9782	0.7061	0.0324	0.4786	No	0.9038	0.0769	0.0143	0.0346	No	No
97442_at	Slc39a8	solute carrier family 39 (metal ion transporter), member 8	0.8987	0.0314	0.456	0.2984	No	0.9774	0.66	0.0036	0.0123	No	No
97443_at	Mrpl52	mitochondrial ribosomal protein L52	1.0926	0.0843	0.0056	0.6349	No	1.06	0.152	0.0018	0.0803	No	No
97444_at	Ifi30	interferon gamma inducible protein 30	1.0156	0.5956	0.0004	0.3458	No	0.9699	0.476	0.0005	0.133	No	No
97445_at	Ppid	peptidylprolyl isomerase D (cyclophilin D)	1.1698	0.0874	0.0014	0.776	No	1.1322	0.0656	0.0003	0.535	No	No
97446_at	Dhx30	DEAH (Asp-Glu-Ala-His) box polypeptide 30	0.9828	0.3538	0.0012	0.1972	No	0.9962	0.974	0.11	0.26	No	No
97447_at	Map1lc3a	microtubule-associated protein 1 light chain 3 alpha	0.9795	0.6326	0.002	0.2107	No	0.9488	0.212	0.0263	0.0105	No	No
97448_at	4933439C20 Rik /// Pisd	RIKEN cDNA 4933439C20 gene /// phosphatidylserine decarboxylase	1.0122	0.8314	0.033	0.177	No	1.0206	0.638	0.171	0.164	No	No
97449_at	Aldh7a1	aldehyde dehydrogenase family 7, member A1	0.9911	0.6534	0.0004	0.253	No	0.9891	0.431	0.0001	0.965	No	No
97450_s_at	Aldh7a1	aldehyde dehydrogenase family 7, member A1	0.9339	0.3419	0.2322	0.6122	No	0.9686	0.627	0.0298	0.834	No	No
97451_at	Mcfd2	multiple coagulation factor deficiency 2	0.9731	0.024	0.0221	0.004	No	0.9628	0.0261	0.271	0.123	No	No
97452_at	H2afy	H2A histone family, member Y	1.1547	0.0004	0.0001	0.3093	Yes	1.1883	0.0003	0.0001	0.843	Yes	Yes
97456_at	Acsl5	acyl-CoA synthetase long-chain family member 5	1.0599	0.0154	0.0011	0.0027	No	1.0345	0.0915	0.0016	0.0035	No	No
97458_at	Gnb1	guanine nucleotide binding protein, beta 1	0.9939	0.8823	0.0719	0.6232	No	1.0589	0.355	0.159	0.154	No	No
97459_at	Psma4	proteasome (prosome, macropain) subunit, alpha type 4	1.1285	0.0485	0.2106	0.2585	No	1.1115	0.0463	0.443	0.0694	No	No
97460_at	Ube2r2	ubiquitin-conjugating enzyme E2R 2	0.9757	0.3017	0.0054	0.0198	No	0.9513	0.0704	0.0004	0.0315	No	No
97462_at	Zfp706	zinc finger protein 706	1.0106	0.7352	0.1317	0.5752	No	0.9847	0.36	0.0002	0.502	No	No
97463_q_at	Zfp706	zinc finger protein 706	1.0356	0.4572	0.0781	0.1862	No	0.988	0.633	0.329	0.08	No	No
97464_at	Zfp706	zinc finger protein 706	1.0176	0.8457	0.009	0.4834	No	1.0217	0.696	0.032	0.7	No	No
97465_at	Slc6a18	solute carrier family 6 (neurotransmitter transporter), member 18	1.0163	0.5568	0.0925	0.2848	No	0.9338	0.263	0.69	0.222	No	No
97468_at	Cks1b	CDC28 protein kinase 1b	1.086	0.0061	0.0309	0.6251	Yes	1.0812	0.0698	0.0499	0.431	No	No
97469_at	Krtap3-3	keratin associated protein 3-3	1.082	0.0452	0.0009	0.1533	No	0.9985	0.999	0.0358	0.696	No	No
97470_at	Krtap3-1	keratin associated protein 3-1	1.0098	0.8175	0.1666	0.4576	No	1.0323	0.242	0.0097	0.464	No	No
97471_at	Armc1	armadillo repeat containing 1	0.9844	0.389	0.0006	0.163	No	1.0335	0.471	0.143	0.817	No	No
97472_at	Slc25a17	solute carrier family 25 (mitochondrial carrier, peroxisomal membrane protein), member 17	0.9781	0.5754	0.02	0.1376	No	0.9692	0.637	0.0325	0.969	No	No
97473_at	Tspan4	tetraspanin 4	0.9401	0.0755	0.0006	0.0169	No	0.958	0.29	0.0003	0.0099	No	No
97474_r_at	Ptn	pleiotrophin	1.1411	0.1433	0.0035	0.1007	No	1.134	0.282	0.271	0.413	No	No
97475_at	---	---	0.9612	0.4751	0.0051	0.3102	No	1.083	0.143	0.0009	0.23	No	No
97477_at	Timm8b	translocase of inner mitochondrial membrane 8 homolog b (yeast)	0.993	0.9226	0.0097	0.5747	No	0.9924	0.66	0.0001	0.678	No	No
97478_at	Mak10	MAK10 homolog, amino-acid N-acetyltransferase subunit, (S. cerevisiae)	1.0595	0.1463	0.0052	0.5586	No	1.0553	0.562	0.0002	0.0035	No	No
97479_at	Ube2l3	ubiquitin-conjugating enzyme E2L 3	1.0678	0.1196	0.0093	0.2022	No	1.0713	0.0142	0.0724	0.0111	No	No
97480_f_at	Dnajb3	DnaJ (Hsp40) homolog, subfamily B, member 3	1.2133	0.1298	0.1197	0.7613	No	1.0125	0.849	0.426	0.297	No	No
97481_r_at	Dnajb3	DnaJ (Hsp40) homolog, subfamily B, member 3	1.0044	0.9013	0.0003	0.0501	No	1.0559	0.4	0.0217	0.913	No	No
97482_at	Cab39l	calcium binding protein 39-like	0.953	0.1967	0.0012	0.601	No	0.8849	0.0335	0.0028	0.641	No	No
97483_at	Rpl19	ribosomal protein L19	0.9741	0.4142	0.0002	0.5523	No	0.9981	0.966	0.0009	0.699	No	No
97484_at	Zmynd11	zinc finger, MYND domain containing 11	1.0076	0.826	0.0028	0.2248	No	0.9458	0.356	0.182	0.517	No	No
97485_at	---	---	0.9935	0.8708	0.0011	0.3739	No	1.0195	0.554	0.0095	0.115	No	No
97486_at	U2af1	U2 small nuclear ribonucleoprotein auxiliary factor (U2AF) 1	1.0925	0.1339	0.5258	0.4194	No	1.1228	0.0721	0.0326	0.0651	No	No
97487_at	Serpine2	serine (or cysteine) peptidase inhibitor, clade E, member 2	1.045	0.5045	0.3775	0.6278	No	1.0319	0.676	0.0837	0.967	No	No
97488_at	1200011O22 Rik	RIKEN cDNA 1200011O22 gene	0.9609	0.1363	0.0068	0.3879	No	0.9814	0.118	0.0011	0.174	No	No
97489_at	Pygb	brain glycogen phosphorylase	1.0611	0.3603	0.2371	0.4854	No	1.0643	0.307	0.0875	0.885	No	No
97490_at	Bcl7b	B-cell CLL/lymphoma 7B	0.9604	0.3129	0.0419	0.0042	No	0.9691	0.349	0.0526	0.0025	No	No
97491_at	Hnrpr	heterogeneous nuclear ribonucleoprotein R	1.0985	0.3145	0.0105	0.8202	No	1.0807	0.389	0.0608	0.847	No	No
97492_at	Txndc12	thioredoxin domain containing 12 (endoplasmic reticulum)	1.0613	0.2955	0.0258	0.2722	No	1.0821	0.123	0.0432	0.266	No	No
97496_f_at	Prkcdbp	protein kinase C, delta binding protein	1.1064	0.2984	0.0415	0.1219	No	1.0727	0.45	0.0206	0.411	No	No
97497_at	Notch1	Notch gene homolog 1 (Drosophila)	0.9391	0.105	0.0013	0.5224	No	0.962	0.153	0.0004	0.167	No	No
97498_at	Fhl1	four and a half LIM domains 1	0.7852	0.0068	0.0111	0.0038	No	0.7991	0.0018	0.0127	0.0008	No	No
97499_at	Fhl1	four and a half LIM domains 1	0.9744	0.5561	0.0212	0.7236	No	1.0096	0.886	0.283	0.709	No	No
97500_g_at	Fhl1	four and a half LIM domains 1	0.9122	0.152	0.1156	0.0229	No	0.9468	0.274	0.121	0.197	No	No
97502_at	Dld	dihydrolipoamide dehydrogenase	1.0067	0.6621	0	0.0045	No	1.0282	0.533	0.0019	0.278	No	No
97504_at	Ccnd2	cyclin D2	1.0411	0.6361	0.0149	0.82	No	1.0779	0	0	0	No	No
97505_at	Arl1	ADP-ribosylation factor-like 1	1.0386	0.4905	0.0443	0.1613	No	1.0619	0.302	0.102	0.551	No	No
97506_at	Rnf2	ring finger protein 2	0.98	0.7549	0.0813	0.2651	No	0.9762	0.774	0.0823	0.194	No	No
97507_at	Lgals3bp	lectin, galactoside-binding, soluble, 3 binding protein	0.9519	0.0485	0.0004	0.9156	No	0.9467	0.0609	0.0034	0.816	No	No
97508_at	Mdh1	malate dehydrogenase 1, NAD (soluble)	0.9898	0.7239	0.0783	0.1494	No	0.9594	0.481	0.137	0.662	No	No
97509_f_at	Fgfr1	fibroblast growth factor receptor 1	0.9574	0.4512	0.9168	0.866	No	0.9668	0.179	0.0028	0.537	No	No
97510_at	Mgll	monoglyceride lipase	1.1071	0.2418	0.1169	0.1392	No	1.1052	0.36	0.0804	0.195	No	No
97511_at	Mgll	monoglyceride lipase	1.0185	0.4338	0.003	0.2103	No	1.0511	0.211	0.0114	0.53	No	No
97512_at	2010107E04 Rik	RIKEN cDNA 2010107E04 gene	0.9361	0.2831	0.0497	0.7634	No	0.9131	0.164	0.663	0.301	No	No
97514_at	1810063B05 Rik	RIKEN cDNA 1810063B05 gene	1.022	0.6142	0.1409	0.32	No	0.9766	0.468	0.0233	0.0471	No	No
97515_at	Hsd17b4	hydroxysteroid (17-beta) dehydrogenase 4	0.964	0.4685	0.2285	0.8114	No	0.9573	0.448	0.128	0.943	No	No
97516_at	Ganab	alpha glucosidase 2 alpha neutral subunit	1.0265	0.4437	0.8225	0.0475	No	1.0145	0.654	0.748	0.0299	No	No
97517_at	Exosc4	exosome component 4	0.947	0.2853	0.0096	0.0837	No	1.0348	0.507	0.014	0.979	No	No
97518_at	Fdft1	farnesyl diphosphate farnesyl transferase 1	1.1008	0.397	0.4527	0.5279	No	0.9958	0.579	0.0183	0.0004	No	No
97519_at	Spp1	secreted phosphoprotein 1	0.9153	0.0999	0.0016	0.0421	No	0.9148	0.137	0.0101	0.032	No	No
97520_s_at	Nnat	neuronatin	1.182	0.0377	0.0232	0.118	No	1.1273	0.0748	0.439	0.472	No	No
97521_at	Ass1	argininosuccinate synthetase 1	0.9659	0.4568	0.3551	0.1529	No	0.9054	0.328	0.998	0.473	No	No
97523_i_at	Amy2 /// 1810008N23 Rik /// LOC671344	amylase 2, pancreatic /// RIKEN cDNA 1810008N23 gene /// similar to Pancreatic alpha-amylase precursor (PA) (1,4-alpha-D-glucan glucanohydrolase)	0.9508	0.3712	0.0599	0.353	No	1.085	0.58	0.242	0.116	No	No
97524_f_at	Amy2 /// 1810008N23 Rik	amylase 2, pancreatic /// RIKEN cDNA 1810008N23 gene	0.9608	0.3218	0.0424	0.8074	No	0.9203	0.377	0.0272	0.674	No	No
97525_at	Gyk	glycerol kinase	1.0263	0.5585	0.0099	0.9782	No	0.9203	0.291	0.0204	0.355	No	No
97526_at	Ap3m1	adaptor-related protein complex 3, mu 1 subunit	1.0582	0.03	0.0037	0.0389	No	0.9631	0.481	0.125	0.635	No	No
97527_at	Cks2	CDC28 protein kinase regulatory subunit 2	0.9661	0.5169	0.0303	0.9621	No	0.9678	0.451	0.0285	0.845	No	No
97528_at	Cnih	cornichon homolog (Drosophila)	0.9959	0.8645	0.5853	0.2426	No	0.9999	0.737	0	0.032	No	No
97529_at	Anxa8	annexin A8	0.8285	0.0133	0.0078	0.04	No	1.0071	0.962	0.0137	0.168	No	No
97530_at	Ube2i	ubiquitin-conjugating enzyme E2I	1.0016	0.9181	0.0004	0.55	No	1.0183	0.665	0.0163	0.401	No	No
97531_at	G0s2	G0/G1 switch gene 2	0.9626	0.2654	0.2434	0.7636	No	1.0868	0.195	0.0456	0.0285	No	No
97532_at	Ppp5c	protein phosphatase 5, catalytic subunit	0.9902	0.8306	0.0107	0.2947	No	0.9524	0.482	0.0025	0.017	No	No
97533_at	Fcgrt	Fc receptor, IgG, alpha chain transporter	0.9918	0.7914	0.4896	0.2189	No	0.9777	0.444	0.0697	0.0319	No	No
97535_at	Ywhah	tyrosine 3-monooxygenase/tryptophan 5-monooxygenase activation protein, eta polypeptide	1.0511	0.1586	0.0001	0.8479	No	1.0701	0.15	0.0009	0.631	No	No
97536_at	Wdtc1	WD and tetratricopeptide repeats 1	0.9312	0.0608	0.0052	0.6633	No	0.9347	0.0455	0.0023	0.674	No	No
97538_at	Gusb	glucuronidase, beta	0.9627	0.4011	0.1388	0.026	No	0.9618	0.374	0.202	0.335	No	No
97539_at	Prmt3	protein arginine N-methyltransferase 3	0.9578	0.2798	0.0014	0.8389	No	0.9774	0.455	0.0023	0.075	No	No
97540_f_at	H2-D1	histocompatibility 2, D region locus 1	0.9894	0.6261	0.0004	0.043	No	1.0043	0.731	0.0014	0.167	No	No
97541_f_at	H2-D1	histocompatibility 2, D region locus 1	1.0185	0.8764	0.2084	0.4051	No	0.9539	0.255	0.0006	0.226	No	No
97542_at	Myl6 /// LOC433688 /// LOC546101	myosin, light polypeptide 6, alkali, smooth muscle and non-muscle /// similar to Myosin light polypeptide 6 (Myosin light chain alkali 3) (Myosin light chain 3) (MLC-3) (LC17) /// similar to myosin, light polypeptide 6, alkali, smooth muscle and non-muscle	1.0773	0.1506	0.1306	0.5502	No	1.0077	0.758	0.0001	0.226	No	No
97543_at	38962	septin 2	1.0751	0.3248	0.0078	0.183	No	0.9596	0.333	0.0946	0.192	No	No
97544_at	Ywhaz	tyrosine 3-monooxygenase/tryptophan 5-monooxygenase activation protein, zeta polypeptide	0.8707	0.1235	0.0665	0.2111	No	0.9367	0.0624	0.0007	0.0293	No	No
97546_at	Cldn1	claudin 1	0.8862	0.3695	0.322	0.9124	No	0.8653	0.0936	0.0629	0.199	No	No
97548_at	Prpf39	PRP39 pre-mRNA processing factor 39 homolog (yeast)	1.1284	0.4563	0.1185	0.3405	No	1.0318	0.703	0.0532	0.369	No	No
97549_at	Cfl2	cofilin 2, muscle	0.9545	0.5829	0.0728	0.5223	No	0.9232	0.356	0.236	0.999	No	No
97550_at	Hdac7a	histone deacetylase 7A	1.0033	0.9394	0.5717	0.5801	No	0.9763	0.51	0.0257	0.708	No	No
97551_at	Hip1r	huntingtin interacting protein 1 related	1.0276	0.5898	0.201	0.7272	No	1.0098	0.819	0.0977	0.974	No	No

97553_at	Galnt2	UDP-N-acetyl-alpha-D-galactosamine:polypeptide N-acetylgalactosaminyltransferase 2	0.9999	0.9968	0.0258	0.3194	No	0.9786	0.182	0.0036	0.0429	No	No
97554_at	BC005624	cDNA sequence BC005624	0.917	0.2283	0.0659	0.7374	No	0.9589	0.0024	0	0.932	Yes	No
97555_at	Cbx7	chromobox homolog 7	1.0553	0.4407	0.2903	0.9386	No	1.0786	0.38	0.593	0.321	No	No
97556_at	Anp32e	acidic (leucine-rich) nuclear phosphoprotein 32 family, member E	1.0371	0.4891	0.0023	0.1825	No	1.0979	0.0843	0.0125	0.0909	No	No
97557_at	Tor2a	torsin family 2, member A	1.0124	0.6792	0.2169	0.1828	No	0.9862	0.85	0.311	0.203	No	No
97559_at	Eef2	eukaryotic translation elongation factor 2	0.9572	0.3884	0.0011	0.2776	No	0.9369	0.0575	0.0006	0.024	No	No
97560_at	Psap	prosaposin	0.7835	0.0427	0.1208	0.5005	No	0.8649	0.153	0.265	0.544	No	No
97561_at	Gucy2c	guanylate cyclase 2c	1.0261	0.5812	0.3591	0.7411	No	1.0793	0.18	0.851	0.612	No	No
97562_r_at	Vav1	vav 1 oncogene	1.0083	0.9088	0.1214	0.1104	No	1.0233	0.476	0.0074	0.169	No	No
97563_f_at	LOC211331	similar to Ig heavy chain V region 93G7 precursor	0.9726	0.7363	0.2474	0.83	No	1.0081	0.858	0.0067	0.658	No	No
97564_f_at	Igh-V7183 /// Igk-V1 /// Igkv1-117 /// Cr1 /// Igkv1-110	immunoglobulin heavy chain (V7183 family) /// immunoglobulin kappa chain variable 1 (V1) /// immunoglobulin kappa chain variable 1-117 /// Ig kappa chain /// immunoglobulin kappa chain variable 1-110	1.0281	0.5374	0.7335	0.2493	No	1.0869	0.297	0.793	0.667	No	No
97565_r_at	Tfdp1 /// LOC664889 /// LOC672007 /// LOC674691	transcription factor Dp 1 /// similar to Transcription factor Dp-1 (E2F dimerization partner 1) (DRTF1-polypeptide 1) /// similar to Transcription factor Dp-1 (E2F dimerization partner 1) (DRTF1-polypeptide 1) /// similar to Transcription factor Dp-1 (E2F dimerization partner 1) (DRTF1-polypeptide 1)	1.2036	0.0055	0.0045	0.0041	No	1.1457	0.165	0.725	0.138	No	No
97566_f_at	Gm459 /// Gm1419 /// Gm1499 /// Igkv4-61 /// Igkv4-69 /// LOC636677 /// Igkv4-59 /// LOC672342	gene model 459, (NCBI) /// gene model 1419, (NCBI) /// gene model 1499, (NCBI) /// immunoglobulin kappa chain variable 4-61 /// immunoglobulin kappa chain variable 4-69 /// similar to Ig kappa chain V-IV region S107B precursor /// immunoglobulin kappa chain variable 4-59 /// similar to Ig kappa chain V-IV region S107B precursor	1.0359	0.5212	0.2251	0.3631	No	1.0742	0.0584	0.205	0.0086	No	No
97567_f_at	Gm1419 /// Gm1499 /// Gm1502 /// Igkv4-61 /// Igkv4-59 /// LOC672342	gene model 1419, (NCBI) /// gene model 1499, (NCBI) /// gene model 1502, (NCBI) /// immunoglobulin kappa chain variable 4-61 /// immunoglobulin kappa chain variable 4-59 /// similar to Ig kappa chain V-IV region S107B precursor	1.0031	0.8649	0.0439	0.1897	No	1.0607	0.137	0.434	0.153	No	No
97568_at	Igh-V /// LOC382692 /// LOC628614 /// LOC629869 /// LOC629871 /// LOC629884 /// LOC629893 /// LOC629897 /// LOC629906 /// LOC629908 /// LOC629915 /// LOC631595 /// LOC634081 /// LOC634100 ///	immunoglobulin heavy chain variable region /// similar to Ig heavy chain V-I region HG3 precursor /// similar to Ig heavy chain V-I region V35 precursor /// similar to Ig heavy chain V region 108A precursor /// similar to Ig heavy chain V-I region V35 precursor /// similar to Ig heavy chain V region AC38 205.12 /// similar to Ig heavy chain V region 108A precursor /// similar to Ig heavy chain V-I region HG3 precursor /// similar to Ig heavy chain V-I region V35 precursor /// similar to Ig heavy chain V-I region V35 precursor /// similar to Ig heavy chain V region 102 precursor /// similar to Ig heavy chain V region 108A precursor /// similar to Ig heavy chain V-I region HG3 precursor /// similar to Ig heavy chain V region 108A precursor /// similar to Ig heavy chain V region 108A precursor /// similar to Ig heavy chain V-I region V35 precursor /// similar to Ig heavy chain V-I region V35 precursor /// similar to Ig heavy chain V-I region V35 precursor /// similar to Ig heavy chain V region 108A precursor /// similar to Ig heavy chain V-I region V35 precursor /// similar to Ig he	1.084	0.1214	0.9535	0.3268	No	1.0117	0.803	0.894	0.672	No	No
97569_at	LOC637260 /// LOC669091 /// LOC673777	similar to Ig kappa chain V-IV region B17 precursor /// similar to Ig kappa chain V-IV region precursor /// similar to Ig kappa chain V-IV region precursor	1.0184	0.6959	0.1125	0.3184	No	1.0235	0.761	0.938	0.241	No	No
97570_at	---	Ig kappa active V-J region mRNA from PC-binding hybridoma HPCA97	1.0088	0.9241	0.7867	0.9832	No	0.9936	0.921	0.472	0.985	No	No
97571_r_at	Tnfrsf8	tumor necrosis factor receptor superfamily, member 8	1.0256	0.7538	0.0422	0.7719	No	1.0725	0.144	0.569	0.0691	No	No
97572_at	Ibsp	integrin binding sialoprotein	0.9952	0.922	0.1528	0.9702	No	0.9508	0.134	0.0408	0.712	No	No
97573_at	Tyk2	tyrosine kinase 2	0.9208	0.2216	0.0902	0.4072	No	0.9316	0.0045	0.0001	0.0005	No	No
97574_f_at	AI324046 /// LOC634206 /// LOC634222 /// LOC634284 /// LOC634430 /// LOC640522	expressed sequence AI324046 /// similar to Ig heavy chain V-I region V35 precursor /// similar to Ig heavy chain V-I region V35 precursor /// similar to Ig heavy chain V-I region V35 precursor /// similar to Ig heavy chain V region 108A precursor /// similar to Ig heavy chain V region 108A precursor	1.0677	0.1368	0.4427	0.1317	No	1.1352	0.154	0.182	0.0643	No	No
97575_f_at	Igh	Immunoglobulin heavy chain complex	1.053	0.2685	0.0049	0.6878	No	0.8883	0.173	0.0035	0.722	No	No
97576_f_at	LOC628614 /// LOC673196 /// LOC673768	similar to Ig heavy chain V-I region V35 precursor /// similar to Ig heavy chain V-I region V35 precursor /// similar to Ig heavy chain V-I region V35 precursor	0.9973	0.9713	0.0067	0.1716	No	0.9517	0.0834	0.0001	0.215	No	No
97577_f_at	Igh-6	immunoglobulin heavy chain 6 (heavy chain of IgM)	0.9436	0.3602	0.1376	0.2669	No	1.0176	0.734	0.158	0.132	No	No
97578_at	---	---	1.0604	0.0081	0.0102	0.0004	No	1.1085	0.0943	0.172	0.0126	No	No
97579_f_at	Crygf	crystallin, gamma F	1.0462	0.3757	0.0221	0.3613	No	1.0008	0.949	0.0395	0.901	No	No
97580_at	Trpc5	transient receptor potential cation channel, subfamily C, member 5	1.0612	0.036	0.1089	0.0094	No	1.1393	0.0482	0.214	0.168	No	No
97581_at	Odz4	odd Oz/ten-m homolog 4 (Drosophila)	1.1057	0.0087	0.0025	0.0033	No	1.0104	0.68	0.0036	0.251	No	No
97593_f_at	Fliih	flightless I homolog (Drosophila)	0.8559	0.0219	0.1893	0.0087	No	0.8594	0.13	0.0918	0.0712	No	No
97594_r_at	Fliih	flightless I homolog (Drosophila)	1.0212	0.6379	0.0631	0.2551	No	1.102	0.0495	0.0112	0.0584	No	No
97598_at	---	Transcribed locus, weakly similar to NP_570953.1 CAMP [Mus musculus]	1.0108	0.8971	0.0369	0.7539	No	1.0432	0.548	0.016	0.662	No	No
97601_f_at	Pkp1	plakophilin 1	1.0253	0.4374	0.106	0.225	No	1.0703	0.0594	0.0064	0.142	No	No
97610_f_at	AI450241	expressed sequence AI450241	1.0913	0.0206	0.8573	0.8293	No	0.9992	0.985	0.883	0.617	No	No
97645_f_at	Gm428 /// LOC623180 /// LOC623197 /// LOC623210 /// LOC623219	gene model 428, (NCBI) /// hypothetical LOC623180 /// hypothetical LOC623197 /// hypothetical LOC623210 /// hypothetical LOC623219	1.0013	0.9805	0.1987	0.3025	No	1.0198	0.764	0.0688	0.133	No	No
97647_at	Rps16	ribosomal protein S16	0.9839	0.6433	0.0765	0.169	No	0.9644	0.227	0.0103	0.0337	No	No
97651_at	Polk	polymerase (DNA directed), kappa	0.9946	0.8861	0.0098	0.4787	No	0.7935	0.177	0.0011	0.0217	No	No
97652_at	Ghrhr	growth hormone releasing hormone receptor	1.0108	0.893	0.4066	0.6205	No	0.9571	0.659	0.735	0.667	No	No
97653_at	Mem1	maternal embryonic message 1	1.0086	0.635	0.9121	0.1742	No	1.017	0.67	0.33	0.0666	No	No
97654_at	Dlx6as	distal-less homeobox 6, antisense	0.8915	0.0203	0.0002	0.0119	No	0.8489	0.0148	0.0021	0.0037	No	No
97655_at	Dsc3	desmocollin 3	1.053	0.1226	0.0874	0.36	No	0.9338	0.133	0.0035	0.541	No	No
97658_f_at	Ins1	insulin I	0.972	0.7022	0.1407	0.9405	No	1.0081	0.933	0.339	0.262	No	No
97659_r_at	Ins1	insulin I	0.9897	0.7186	0.0171	0.1427	No	0.9419	0.205	0.0065	0.433	No	No

97660_s_at	Tcf7l2	transcription factor 7-like 2, T-cell specific, HMG-box	1.0229	0.5398	0.0118	0.6778	No	1.0061	0.892	0.0199	0.61	No	No
97661_at	Tenr	testis nuclear RNA binding protein	0.9987	0.9707	0.1893	0.9887	No	0.9351	0.445	0.0447	0.397	No	No
97665_i_at	C76213	expressed sequence C76213	1.0616	0.2348	0.2233	0.8893	No	0.9902	0.88	0.37	0.996	No	No
97666_r_at	C76213	expressed sequence C76213	0.9948	0.8627	0.0002	0.2863	No	1.0193	0.624	0.526	0.134	No	No
97667_at	---	---	0.9585	0.4306	0.9347	0.1821	No	1.0278	0.712	0.322	0.114	No	No
97678_r_at	Cts7	cathepsin 7	0.9787	0.548	0.2	0.133	No	0.5543	0.27	0.286	0.187	No	No
97679_at	Tlx2	T-cell leukemia, homeobox 2	0.959	0.4999	0.011	0.8266	No	0.9962	0.966	0.105	0.483	No	No
97680_at	Mug-ps1 /// Mug1 /// Mug2 /// LOC434083 /// LOC669343	murinoglobulin, pseudogene 1 /// murinoglobulin 1 /// murinoglobulin 2 /// similar to murinoglobulin 1 /// similar to murinoglobulin 1	0.9156	0.1195	0.3191	0.9906	No	0.9204	0.164	0.322	0.397	No	No
97681_f_at	Gstm3	glutathione S-transferase, mu 3	0.9843	0.6477	0.8078	0.0824	No	0.9145	0.0059	0.364	0.0002	No	No
97682_r_at	Gstm3	glutathione S-transferase, mu 3	0.9468	0.5781	0.1212	0.5387	No	0.9459	0.433	0.0446	0.687	No	No
97683_at	Tnfrsf9	tumor necrosis factor receptor superfamily, member 9	1.0265	0.2519	0.7797	0.3957	No	0.9714	0.391	0.386	0.55	No	No
97684_at	Pick1	protein interacting with C kinase 1	0.958	0.3183	0.8091	0.0712	No	0.9286	0.211	0.616	0.296	No	No
97685_at	1700023D09 Rik	RIKEN cDNA 1700023D09 gene	0.9537	0.354	0.2583	0.3577	No	1.0231	0.654	0.117	0.552	No	No
97686_r_at	Prcc	papillary renal cell carcinoma (translocation-associated)	0.9381	0.0894	0.0409	0.7841	No	0.9449	0.0902	0.0169	0.03	No	No
97687_at	---	---	1.0972	0.1219	0.231	0.039	No	1.2365	0.0047	0.0028	0.0452	Yes	No
97688_at	---	16 days neonate thymus cDNA, RIKEN full-length enriched library, clone:A130082C12 product:unclassifiable, full insert sequence	1.0296	0.4778	0.4405	0.2085	No	0.9844	0.839	0.0075	0.0162	No	No
97689_at	F3	coagulation factor III	1.0159	0.8349	0.1231	0.7835	No	0.9811	0.862	0.0035	0.195	No	No
97690_at	---	---	1.0809	0.252	0.0742	0.7973	No	1.0351	0.557	0.0105	0.717	No	No
97691_at	Rpl27a	Ribosomal protein L27a	0.9948	0.9432	0.1549	0.2682	No	1.1032	0.158	0.08	0.708	No	No
97692_at	Htr2c	5-hydroxytryptamine (serotonin) receptor 2C	1.0282	0.4823	0.8285	0.4759	No	0.8274	0.632	0	0.0198	No	No
97693_at	C78513	EST C78513	0.968	0.2544	0.3066	0.798	No	1.0776	0.138	0.0716	0.304	No	No
97694_at	2310022L06 Rik	RIKEN cDNA 2310022L06 gene	0.9808	0.7738	0.1785	0.9694	No	1.0375	0.205	0	0.0047	No	No
97695_s_at	Rpl7	ribosomal protein L7	1.0947	0.0521	0.0527	0.1736	No	1.0394	0.319	0.0608	0.392	No	No
97696_r_at	Rpl7	ribosomal protein L7	1.0477	0.4616	0.0143	0.2943	No	1.0288	0.283	0.0023	0.182	No	No
97697_at	Rpl7	ribosomal protein L7	0.9732	0.6779	0.033	0.9304	No	0.9564	0.534	0.0098	0.734	No	No
97698_at	Svs6	seminal vesicle secretion 6	0.9237	0.08	0.2454	0.4082	No	0.757	0.0691	0.0206	0.0429	No	No
97699_at	Plxna1	plexin A1	0.9895	0.8582	0.2383	0.3048	No	0.9442	0.368	0.284	0.401	No	No
97700_at	H2-K1	Histocompatibility 2, K1, K region	1.046	0.2735	0.1349	0.0919	No	1.0504	0.0334	0.0011	0.872	No	No
97701_at	Bcas2	breast carcinoma amplified sequence 2	1.023	0.7415	0.028	0.865	No	0.9912	0.712	0.0514	0.0248	No	No
97702_at	---	Transcribed locus	1.0004	0.9925	0.0713	0.2155	No	0.9471	0.407	0.54	0.77	No	No
97703_at	Bxdc1	brix domain containing 1	1.0433	0.3802	0.1045	0.6431	No	0.9624	0.439	0.0533	0.982	No	No
97704_at	Ell2	elongation factor RNA polymerase II 2	0.9487	0.1373	0.6479	0.8388	No	0.889	0.139	0.803	0.762	No	No
97705_at	Snapc3	small nuclear RNA activating complex, polypeptide 3	0.9691	0.3444	0.0516	0.0182	No	0.992	0.64	0.0195	0.0232	No	No
97706_at	Marcksl1	MARCKS-like 1	1.0383	0.4968	0.1995	0.5866	No	1.0142	0.715	0.211	0.0638	No	No
97707_at	LOC541456	hypothetical LOC541456	0.904	0.2455	0.3444	0.6657	No	0.8261	0.285	0.552	0.287	No	No
97708_at	---	---	1.0637	0.5754	0.8585	0.2981	No	0.9661	0.642	0	0.952	No	No
97709_at	Il7	interleukin 7	0.9893	0.3518	0.0012	0.0364	No	1.0625	0.458	0.935	0.0717	No	No
97710_f_at	Tbc1d24	TBC1 domain family, member 24	1.0919	0.1063	0.0057	0.9206	No	0.9332	0.321	0.12	0.159	No	No
97711_at	Npr3	natriuretic peptide receptor 3	0.8437	0.0206	0.0068	0.4888	No	1.0416	0.284	0.271	0.174	No	No
97712_at	Pip5k3	phosphatidylinositol-3-phosphate/phosphatidylinositol 5-kinase, type III	1.0264	0.674	0.1014	0.1093	No	0.9594	0.282	0.325	0.277	No	No
97713_at	2210010A19 Rik	RIKEN cDNA 2210010A19 gene	1.0028	0.9297	0.1444	0.3349	No	1.0048	0.841	0.0228	0.667	No	No
97714_r_at	---	---	1.0149	0.7514	0.6204	0.532	No	1.0884	0.459	0.668	0.196	No	No
97717_at	Tcf15	transcription factor 15	0.9476	0.3416	0.5706	0.425	No	1.0727	0.241	0.0353	0.355	No	No
97718_at	Ctla4	cytotoxic T-lymphocyte-associated protein 4	0.9843	0.6918	0.2503	0.7638	No	0.9094	0.108	0.291	0.728	No	No
97719_at	Mst1r	macrophage stimulating 1 receptor (c-met-related tyrosine kinase)	1.0032	0.9313	0.0336	0.8843	No	0.9378	0.0942	0.011	0.227	No	No
97720_at	Igh-VS107 /// LOC674066 /// LOC674141	immunoglobulin heavy chain (S107 family) /// similar to Ig heavy chain V region M167 precursor /// similar to Ig heavy chain V region M167 precursor	0.9063	0.0286	0.0077	0.4421	No	0.9759	0.77	0.119	0.268	No	No
97721_at	Fgf15	fibroblast growth factor 15	0.9913	0.8508	0.46	0.9018	No	1.0372	0.393	0.203	0.144	No	No
97722_at	Ssr1	signal sequence receptor, alpha	1.0328	0.5423	0.2892	0.7548	No	1.0313	0.459	0.456	0.246	No	No
97723_at	Cbx2	chromobox homolog 2 (Drosophila Pc class)	1.0085	0.8676	0.1513	0.712	No	1.0034	0.958	0.517	0.587	No	No
97724_at	Cry2	cryptochrome 2 (photolyase-like)	1.0021	0.9653	0.4949	0.9981	No	1.02	0.749	0.169	0.945	No	No
97725_at	Bmpr1b	bone morphogenetic protein receptor, type 1B	0.9188	0.153	0.3904	0.945	No	0.7886	0.0683	0.0928	0.22	No	No
97726_at	Grin2c	glutamate receptor, ionotropic, NMDA2C (epsilon 3)	1.0085	0.8508	0.104	0.0758	No	0.9312	0.0353	0.0056	0.0102	No	No
97727_at	Nog	noggin	1.0803	0.2405	0.0048	0.9921	No	1.0478	0.178	0.0003	0.0654	No	No
97728_at	Ntn1	netrin 1	0.9885	0.819	0.011	0.0283	No	1.0565	0.209	0.0061	0.378	No	No
97729_at	Enc1	ectodermal-neural cortex 1	0.9764	0.7781	0.1055	0.7349	No	1.015	0.937	0.0598	0.365	No	No
97730_at	Abca4	ATP-binding cassette, sub-family A (ABC1), member 4	0.9722	0.5306	0.671	0.4658	No	0.9793	0.846	0.2	0.243	No	No
97731_at	Indo	indoleamine-pyrrole 2,3 dioxygenase	0.9787	0.5087	0.8233	0.8034	No	0.9699	0.186	0.0334	0.293	No	No
97732_at	Pde6g	phosphodiesterase 6G, cGMP-specific, rod, gamma	1.0497	0.5614	0.0635	0.6948	No	1.0848	0.0937	0.0003	0.0862	No	No
97733_at	Adora2b	adenosine A2b receptor	1.016	0.6397	0.7333	0.3159	No	0.9396	0.0324	0.253	0.0117	No	No
97734_at	Thpo	thrombopoietin	1.0064	0.7919	0.8094	0.914	No	1.0628	0.262	0.246	0.871	No	No
97735_at	Trhr	thyrotropin releasing hormone receptor	0.9705	0.133	0.0215	0.0103	No	0.902	0.0638	0.378	0.233	No	No
97736_at	Gfm2	G elongation factor, mitochondrial 2	1.087	0.2625	0.4792	0.325	No	1.1187	0.342	0.11	0.569	No	No
97737_f_at	BC034204	cDNA sequence BC034204	1.001	0.9775	0.1914	0.984	No	1.0183	0.7	0.05	0.88	No	No
97738_r_at	BC034204	cDNA sequence BC034204	0.9801	0.6224	0.0567	0.5082	No	1.0437	0.802	0.977	0.932	No	No
97739_at	Ube2o	ubiquitin-conjugating enzyme E2O	0.9548	0.4534	0.0328	0.9096	No	0.9724	0.567	0.0047	0.457	No	No
97740_at	Dusp16	dual specificity phosphatase 16	0.9601	0.5381	0.0849	0.7548	No	0.9983	0.969	0.561	0.609	No	No
97741_at	Tshb	thyroid stimulating hormone, beta subunit	1.1191	0.0112	0.0079	0.0017	No	1.08	0.331	0.441	0.122	No	No
97742_s_at	Fgf8	fibroblast growth factor 8	0.9023	0.1167	0.0315	0.0935	No	0.9107	0.117	0.0126	0.462	No	No
97743_at	Zc3h3	zinc finger CCCH type containing 3	0.925	0.1328	0.9483	0.4877	No	0.9639	0.178	0.0023	0.0241	No	No
97744_at	Hpcal4	Hippocalcin-like 4	1.076	0.0462	0.0011	0.3719	No	1.1334	0.0335	0.0011	0.134	No	No
97745_at	Hoxa4	homeo box A4	1.0937	0.3219	0.6493	0.1261	No	1.0362	0.274	0.109	0.0517	No	No
97746_f_at	Hoxa4	homeo box A4	1.1078	0.0955	0.7811	0.1099	No	1.3176	0.0289	0.143	0.0204	No	No
97747_r_at	Hoxa4	homeo box A4	0.974	0.4612	0.0098	0.3565	No	1.1266	0.0185	0.0224	0.04	No	No
97748_at	Slc6a8	Solute carrier family 6 (neurotransmitter transporter, creatine), member 8	0.9607	0.4353	0.0507	0.7222	No	0.962	0.443	0.864	0.755	No	No
97749_at	Cd47	CD47 antigen (Rh-related antigen, integrin-associated signal transducer)	1.0777	0.4347	0.8714	0.6955	No	1.089	0.221	0.0011	0.178	No	No
97750_at	Rpsa	ribosomal protein SA	1.0098	0.8276	0.0271	0.1082	No	1.031	0.266	0.0022	0.0935	No	No
97751_f_at	LOC14433 /// AK190093	similar to glyceraldehyde-3-phosphate dehydrogenase /// cDNA sequence AK190093	1.034	0.0322	0.0422	0.8666	No	0.9777	0.742	0.204	0.499	No	No
97752_at	E130013N09 Rik	RIKEN cDNA E130013N09 gene	0.9556	0.5306	0.0827	0.4615	No	1.0097	0.761	0.0141	0.417	No	No
97753_at	Pclo	piccolo (presynaptic cytomatrix protein)	0.9869	0.7211	0.0028	0.889	No	1.0546	0.689	0.287	0.052	No	No
97754_at	G630024C0 7Rik	RIKEN cDNA G630024C07 gene	0.9942	0.9031	0.276	0.1019	No	1.0028	0.951	0.749	0.341	No	No
97755_at	Drd4	dopamine receptor 4	1.0875	0.0322	0.5163	0.0197	No	1.079	0.134	0.677	0.251	No	No
97756_s_at	Pcdha4 /// Pcdha6 /// Pcdha7 /// Pcdha5 /// Pcdha11 /// Pcdha10 /// Pcdha1 /// Pcdha9 /// Pcdha@ /// Pcdha3 /// Pcdha12 /// Pcdha2 /// Pcdha8 /// Pcdhac1 /// Pcdhac2	protocadherin alpha 4 /// protocadherin alpha 6 /// protocadherin alpha 7 /// protocadherin alpha 5 /// protocadherin alpha 11 /// protocadherin alpha 10 /// protocadherin alpha 1 /// protocadherin alpha 9 /// protocadherin alpha cluster /// protocadherin alpha 3 /// protocadherin alpha 12 /// protocadherin alpha 2 /// protocadherin alpha 8 /// protocadherin alpha subfamily C, 1 /// protocadherin alpha subfamily C, 2	1.0421	0.1561	0.0005	0.0203	No	1.0282	0.396	0.0115	0.0993	No	No
97757_at	Sva	seminal vesicle antigen	0.878	0.1484	0.0712	0.463	No	0.9221	0.432	0.015	0.264	No	No

97758_at	Prdx1	peroxiredoxin 1	1.123	0.0235	0.0198	0.0959	No	1.0789	0.0468	0.0011	0.0758	No	No
97759_at	Kcnma1	potassium large conductance calcium-activated channel, subfamily M, alpha member 1	1.0024	0.9256	0	0.0359	No	0.907	0.0618	0.0002	0.502	No	No
97760_at	Mtap2 /// A730034C02	microtubule-associated protein 2 /// hypothetical protein A730034C02	0.9778	0.7441	0.522	0.4928	No	0.9805	0.688	0.559	0.507	No	No
97761_f_at	Klra13 /// Klra7 /// Klra23	killer cell lectin-like receptor subfamily A, member 13 /// killer cell lectin-like receptor, subfamily A, member 7 /// killer cell lectin-like receptor subfamily A, member 23	0.9711	0.2025	0.0043	0.1232	No	1.0029	0.986	0.18	0.257	No	No
97762_f_at	Klra13 /// Klra7 /// Klra23	killer cell lectin-like receptor subfamily A, member 13 /// killer cell lectin-like receptor, subfamily A, member 7 /// killer cell lectin-like receptor subfamily A, member 23	0.9506	0.4373	0.0804	0.7723	No	1.0397	0.721	0.332	0.689	No	No
97763_at	Ncf1	neutrophil cytosolic factor 1	0.9262	0.1054	0.0263	0.4036	No	1.0091	0.878	0.107	0.797	No	No
97764_at	Ros1	Ros1 proto-oncogene	1.0166	0.8618	0.3029	0.6033	No	1.3429	0.0112	0.019	0.105	No	No
97765_g_at	Ros1	Ros1 proto-oncogene	0.9222	0.1195	0.0084	0.1313	No	1.0201	0.759	0.0022	0.796	No	No
97766_at	Ros1	Ros1 proto-oncogene	0.9446	0.348	0.2249	0.9506	No	1.0233	0.553	0.0134	0.13	No	No
97767_at	Krt1-2	keratin complex 1, acidic, gene 2	0.9614	0.3558	0.4845	0.9156	No	1.0047	0.828	0.032	0.434	No	No
97768_at	Dsc2	desmocollin 2	0.9834	0.614	0.0447	0.5888	No	0.9606	0.654	0.0933	0.734	No	No
97769_at	Ptgfr	prostaglandin F receptor	0.9318	0.4351	0.383	0.9141	No	1.0271	0.532	0.0157	0.153	No	No
97770_s_at	D6Wsu176e	DNA segment, Chr 6, Wayne State University 176, expressed	1.0248	0.7189	0.0263	0.0883	No	0.9958	0.687	0.0355	0.0392	No	No
97771_r_at	D6Wsu176e	DNA segment, Chr 6, Wayne State University 176, expressed	1.0371	0.5952	0.2873	0.0685	No	1.1367	0.143	0.0061	0.0409	No	No
97772_at	Plau	plasminogen activator, urokinase	0.9937	0.896	0.6545	0.3682	No	0.9391	0.0491	0.174	0.028	No	No
97773_at	Cd34	CD34 antigen	0.9639	0.4694	0.0162	0.0727	No	1.0491	0.116	0.0005	0.0431	No	No
97774_at	Oprm1	opioid receptor, mu 1	0.974	0.5138	0.0101	0.298	No	0.9411	0.194	0.041	0.959	No	No
97775_at	AA511254	expressed sequence AA511254	1.1098	0.3119	0.5101	0.3738	No	1.111	0.103	0.0232	0.163	No	No
97776_at	Drd2	dopamine receptor 2	1.0212	0.7432	0.293	0.9145	No	1.0666	0.0407	0.0146	0.0326	No	No
97777_at	Nkx2-5	NK2 transcription factor related, locus 5 (Drosophila)	0.9987	0.9819	0.0213	0.8883	No	1.0589	0.375	0.087	0.764	No	No
97778_at	St3gal3	ST3 beta-galactoside alpha-2,3-sialyltransferase 3	0.9953	0.8533	0.0013	0.6021	No	0.9103	0.168	0.0203	0.325	No	No
97779_at	Mcpt8	mast cell protease 8	0.8873	0.143	0.5888	0.3267	No	0.9418	0.122	0.718	0.0249	No	No
97780_at	Usp42	ubiquitin specific peptidase 42	1.1045	0.0262	0.0616	0.1556	No	1.2254	0.0565	0.0336	0.285	No	No
97781_at	Ncr1	natural cytotoxicity triggering receptor 1	1.0529	0.2638	0.0104	0.2219	No	0.9964	0.757	0.0034	0.159	No	No
97782_at	Defb2	defensin beta 2	0.9698	0.2752	0.0214	0.0852	No	0.9436	0.171	0.001	0.0221	No	No
97783_at	Ccl17	chemokine (C-C motif) ligand 17	0.9686	0.4701	0.0457	0.2027	No	1.023	0.682	0.0488	0.378	No	No
97784_at	Aloxe3	arachidonate lipoxygenase 3	1.0016	0.9692	0.6637	0.8139	No	1.0751	0.0348	0.0047	0.0111	No	No
97785_at	D0H6S2654E	DNA segment, human D6S2654E	1.0037	0.9504	0.0033	0.2776	No	1.0319	0.644	0.0059	0.254	No	No
97786_at	Ankrd2	ankyrin repeat domain 2 (stretch responsive muscle)	0.7533	0.0002	0.0549	0.0001	No	0.8748	0.0414	0.011	0.018	No	No
97787_at	Slc6a3	solute carrier family 6 (neurotransmitter transporter, dopamine), member 3	0.99	0.7354	0.1539	0.3489	No	1.0793	0.222	0.251	0.256	No	No
97788_at	Krt1-12	keratin complex 1, acidic, gene 12	1.0294	0.6859	0.4429	0.2294	No	1.1194	0.261	0.0161	0.742	No	No
97789_at	Foxh1	forkhead box H1	0.9907	0.769	0.0208	0.1183	No	0.9937	0.878	0.847	0.122	No	No
97790_s_at	Lama3 /// LOC669814	laminin, alpha 3 /// similar to Laminin alpha-3 chain precursor (Nicein alpha subunit)	0.9888	0.7588	0.0039	0.8236	No	1.0609	0.237	0.0775	0.434	No	No
97791_at	Sema5b	sema domain, seven thrombospondin repeats (type 1 and type 1-like), transmembrane domain (TM) and short cytoplasmic domain, (semaphorin) 5B	0.9424	0.498	0.0837	0.4775	No	0.9409	0.247	0.406	0.427	No	No
97792_at	Neurog2	neurogenin 2	1.0309	0.3089	0.8615	0.4627	No	1.0588	0.316	0.0336	0.183	No	No
97793_at	Gria3	glutamate receptor, ionotropic, AMPA3 (alpha 3)	0.9746	0.5342	0.8333	0.3004	No	0.9578	0.453	0.879	0.177	No	No
97794_at	Sema7a	sema domain, immunoglobulin domain (Ig), and GPI membrane anchor, (semaphorin) 7A	0.9876	0.7873	0.038	0.6369	No	0.9828	0.459	0.831	0.0081	No	No
97795_at	Calcrl	calcitonin receptor-like	0.9976	0.9702	0.9844	0.5192	No	0.9002	0.709	0.0749	0.155	No	No
97796_at	Crsp2	cofactor required for Sp1 transcriptional activation, subunit 2	0.9914	0.7918	0.011	0.9609	No	1.0094	0.422	0.0007	0.0106	No	No
97797_at	Asnsd1	asparagine synthetase domain containing 1	0.957	0.2224	0.003	0.618	No	0.9578	0.0346	0	0.253	No	No
97798_at	4930504E06 Rik	RIKEN cDNA 4930504E06 gene	0.9322	0.1813	0.0441	0.2622	No	0.9852	0.802	0.0896	0.57	No	No
97800_at	Fastk	Fas-activated serine/threonine kinase	0.9943	0.9369	0.0887	0.5466	No	1.035	0.477	0.0123	0.226	No	No
97802_at	Bxdc5	brix domain containing 5	1.0909	0.0984	0.9536	0.283	No	0.9972	0.942	0.668	0.234	No	No
97803_at	Mpp1	membrane protein, palmitoylated	0.9913	0.7717	0.7742	0.7477	No	0.9931	0.848	0.0973	0.794	No	No
97807_at	Ufc1	ubiquitin-fold modifier conjugating enzyme 1	0.9978	0.9752	0.2149	0.1448	No	0.9645	0.598	0.0005	0.0329	No	No
97808_at	Sf3b1	splicing factor 3b, subunit 1	1.0959	0.1078	0	0.8925	No	1.0491	0.302	0	0.0056	No	No
97809_at	Ehmt2	euchromatic histone lysine N-methyltransferase 2	1.0444	0.1952	0.0011	0.3079	No	1.0065	0.546	0.0001	0.105	No	No
97811_at	Arfgap3	ADP-ribosylation factor GTPase activating protein 3	1.0243	0.4889	0.0047	0.4998	No	1.0507	0.0018	0	0.0005	No	No
97812_at	Ranbp9	RAN binding protein 9	0.9756	0.792	0.0717	0.5539	No	0.8784	0.0386	0.741	0.725	No	No
97813_at	Rela	v-rel reticuloendotheliosis viral oncogene homolog A (avian)	0.9465	0.1403	0.0027	0.668	No	0.961	0.167	0.0066	0.592	No	No
97814_at	Krt1-3	keratin complex 1, acidic, gene 3	0.9912	0.7652	0.0691	0.2002	No	0.9947	0.896	0.0296	0.538	No	No
97816_at	2600011C06 Rik	RIKEN cDNA 2600011C06 gene	0.9539	0.497	0.0033	0.2345	No	0.9678	0.836	0.0854	0.653	No	No
97817_at	Cdc42se1	CDC42 small effector 1	0.9576	0.4811	0.0639	0.5214	No	0.9656	0.526	0.0423	0.112	No	No
97818_at	Snx4	sorting nexin 4	0.9763	0.715	0.2734	0.5678	No	1.0574	0.0708	0.0022	0.212	No	No
97819_at	Gsto1	glutathione S-transferase omega 1	0.9775	0.6235	0.015	0.4468	No	0.9785	0.731	0.025	0.125	No	No
97820_at	Galk1	galactokinase 1	1.0883	0.2013	0.0084	0.7143	No	1.1676	0.0969	0.0102	0.453	No	No
97821_at	Pak2	p21 (CDKN1A)-activated kinase 2	0.9872	0.7647	0.0896	0.9593	No	1.0264	0.365	0.0078	0.811	No	No
97822_at	Pak2	p21 (CDKN1A)-activated kinase 2	0.9863	0.7773	0.4943	0.2343	No	1.0159	0.689	0.0079	0.996	No	No
97823_g_at	Pak2	p21 (CDKN1A)-activated kinase 2	1.0044	0.8128	0.0012	0.0044	No	0.9895	0.771	0.0001	0.0186	No	No
97824_at	Nola2	nucleolar protein family A, member 2	1.0293	0.4413	0.0317	0.1186	No	1.0892	0.0104	0.0015	0.0114	No	No
97825_at	Perp	PERP, TP53 apoptosis effector	0.9101	0.1531	0.0345	0.0168	No	0.8787	0.0889	0.0039	0.0272	No	No
97826_at	Fcgbp	Fc fragment of IgG binding protein	0.9673	0.4906	0.387	0.8731	No	0.9808	0.623	0.91	0.188	No	No
97828_at	Siva	Cd27 binding protein (Hindu God of destruction)	1.0359	0.3548	0.0079	0.4832	No	1.023	0.62	0.0983	0.966	No	No
97829_at	Cdipt	CDP-diacylglycerol--inositol 3-phosphatidyltransferase (phosphatidylinositol synthase)	1.0238	0.3839	0.1318	0.4865	No	1.0078	0.831	0.729	0.971	No	No
97830_at	Pecam1	platelet/endothelial cell adhesion molecule 1	1.0665	0.0646	0.4879	0.007	No	1.0568	0.183	0.528	0.0231	No	No
97832_at	Cd97	CD97 antigen	1.0154	0.7062	0.1612	0.4394	No	0.9956	0.931	0.202	0.817	No	No
97833_at	Pfkp	phosphofructokinase, platelet	1.038	0.5049	0.0017	0.6247	No	0.9827	0.845	0.0185	0.642	No	No
97834_g_at	Pfkp	phosphofructokinase, platelet	0.9473	0.1368	0.0002	0.6723	No	0.9644	0.0371	0	0.141	No	No
97835_at	Rarres2	retinoic acid receptor responder (tazarotene induced) 2	0.8794	0.0178	0.0096	0.0105	No	0.8896	0.0334	0.0017	0.0207	No	No
97836_at	Rnf7	ring finger protein 7	1.3494	0.0211	0.0036	0.1531	No	1.0806	0.106	0.131	0.23	No	No
97838_at	Snord22	small nucleolar RNA, C/D box 22	0.916	0.0363	0.1552	0.2576	No	0.9147	0.235	0.242	0.232	No	No
97839_at	Snx6	sorting nexin 6	1.0705	0.19	0.0148	0.9522	No	1.0485	0.392	0.0437	0.621	No	No
97841_at	Chmp2a	chromatin modifying protein 2A	0.9878	0.8179	0.0304	0.1828	No	0.9739	0.788	0.0115	0.156	No	No
97843_at	Ncoa4 /// 1110034E15 Rik /// LOC627557	nuclear receptor coactivator 4 /// RIKEN cDNA 1110034E15 gene /// similar to nuclear receptor coactivator 4	0.9892	0.8664	0.0018	0.2107	No	0.937	0.405	0.0347	0.17	No	No
97844_at	Rgs2	regulator of G-protein signaling 2	1.0361	0.3249	0.0001	0.0812	No	1.0605	0.463	0.0006	0.136	No	No
97845_at	Wdr5	WD repeat domain 5	0.9508	0.2532	0.0839	0.9111	No	0.9798	0.659	0.882	0.812	No	No
97846_at	Cdc5l	cell division cycle 5-like (S. pombe)	0.9307	0.2421	0.0406	0.2737	No	1.0053	0.893	0.0002	0.11	No	No
97847_at	Rbmx	RNA binding motif protein, X chromosome	1.0387	0.4053	0.7757	0.9687	No	1.0778	0.25	0.136	0.102	No	No
97848_at	Rbmx	RNA binding motif protein, X chromosome	0.9934	0.9219	0.0427	0.2466	No	0.9461	0.432	0.0097	0.0634	No	No
97849_at	Cav1	caveolin, caveolae protein 1	0.9776	0.4631	0.007	0.0089	No	0.9612	0.261	0.0222	0.0186	No	No
97853_at	Psip1	PC4 and SFRS1 interacting protein 1	1.334	0.0111	0.0011	0.0111	No	1.146	0.133	0.0084	0.168	No	No
97857_at	Zdhhc3	zinc finger, DHHC domain containing 3	1.0883	0.1792	0.2184	0.3908	No	1.0268	0.475	0.452	0.267	No	No
97858_at	Inpp5a	inositol polyphosphate-5-phosphatase A	1.0091	0.6572	0.4638	0.3807	No	0.9659	0.371	0.992	0.0374	No	No
97859_at	Inpp5a	inositol polyphosphate-5-phosphatase A	0.9074	0.1757	0.3866	0.2818	No	0.9182	0.142	0.0617	0.0989	No	No
97860_at	Nap1l4	nucleosome assembly protein 1-like 4	0.984	0.7335	0.605	0.5239	No	0.9788	0.676	0.0016	0.307	No	No
97861_at	Tmem111	transmembrane protein 111	1.0484	0.3363	0.2347	0.2389	No	1.1935	0.13	0.621	0.306	No	No
97862_s_at	Tmem111	transmembrane protein 111	1.0207	0.6122	0.003	0.4978	No	0.995	0.978	0.0142	0.35	No	No
97863_at	D8Ertd354e	DNA segment, Chr 8, ERATO Doi 354, expressed	1.0236	0.3984	0.0038	0.1822	No	1.0241	0.478	0.0233	0.809	No	No
97864_at	2510049I19 Rik	RIKEN cDNA 2510049I19 gene	0.9937	0.9015	0.0079	0.1592	No	1.0247	0.57	0.019	0.182	No	No
97865_g_at	2510049I19 Rik	RIKEN cDNA 2510049I19 gene	0.91	0.0474	0.0293	0.0847	No	0.9448	0.253	0.992	0.42	No	No
97866_at	2510049I19 Rik	RIKEN cDNA 2510049I19 gene	1.068	0.4505	0.0902	0.8411	No	0.959	0.246	0.0035	0.62	No	No

97867_at	Hsd11b1	hydroxysteroid 11-beta dehydrogenase 1	0.9293	0.155	0.431	0.4482	No	0.9109	0.108	0.193	0.259	No	No
97868_at	Dnaja3	DnaJ (Hsp40) homolog, subfamily A, member 3	0.9382	0.0411	0.0217	0.0209	No	1.0539	0.164	0.046	0.243	No	No
97869_at	Etfdh	electron transferring flavoprotein, dehydrogenase	0.9757	0.6323	0.0066	0.0953	No	1.022	0.656	0.0005	0.268	No	No
97870_s_at	Ero1l	ERO1-like (S. cerevisiae)	0.9738	0.0896	0.003	0.0006	No	1.0247	0.241	0.0065	0.0046	No	No
97871_at	---	---	0.9942	0.8607	0.004	0.408	No	1.018	0.656	0.017	0.878	No	No
97873_at	4432404C02Rik	RIKEN cDNA 4432404C02 gene	0.9544	0.2283	0.1602	0.4337	No	0.9726	0.244	0.037	0.471	No	No
97874_at	1500032D16Rik	RIKEN cDNA 1500032D16 gene	0.9975	0.9726	0.0194	0.0652	No	0.963	0.363	0.0026	0.0053	No	No
97875_at	Adrm1	adhesion regulating molecule 1	1.0129	0.7222	0.0042	0.4696	No	0.9855	0.668	0.0018	0.036	No	No
97876_at	Vps29	vacuolar protein sorting 29 (S. pombe)	1.0849	0.0693	0.0085	0.3964	No	0.9943	0.846	0.747	0.0206	No	No
97880_at	Dlst	dihydrolipoamide S-succinyltransferase (E2 component of 2-oxo-glutarate complex)	1.007	0.7787	0.1636	0.2056	No	1.0635	0.0939	0.216	0.215	No	No
97882_at	Sec61a1	Sec61 alpha 1 subunit (S. cerevisiae)	1.0427	0.3069	0.0002	0.3644	No	1.0646	0.0282	0	0.04	No	No
97884_at	Mrps11	mitochondrial ribosomal protein S11	1.069	0.0374	0.0119	0.0165	No	1.0138	0.487	0.003	0.102	No	No
97885_at	1810009M01Rik	RIKEN cDNA 1810009M01 gene	0.9272	0.0075	0.0003	0.0039	No	0.9393	0.0501	0.0043	0.15	No	No
97886_at	Spr	sepiapterin reductase	1.0604	0.2738	0.0041	0.9909	No	1.0667	0.173	0.0015	0.713	No	No
97887_at	Apoc2	apolipoprotein C-II	1.0204	0.8019	0.0669	0.8616	No	1.0541	0.102	0.108	0.368	No	No
97888_at	Frag1	FGF receptor activating protein 1	1.0084	0.9069	0.1378	0.7733	No	0.9788	0.281	0.0031	0.04	No	No
97889_at	Fabp2	fatty acid binding protein 2, intestinal	0.9911	0.9214	0.2652	0.7235	No	1.0818	0.259	0.749	0.0793	No	No
97890_at	Sgk	serum/glucocorticoid regulated kinase	0.7859	0.0147	0.0439	0.744	No	0.7532	0.0221	0.333	0.791	No	No
97891_at	Sac3d1	SAC3 domain containing 1	0.9622	0.3891	0.0371	0.3354	No	1.0764	0.153	0.277	0.212	No	No
97892_at	Brd8	bromodomain containing 8	1.0416	0.4204	0.0554	0.3726	No	1.0512	0.448	0.873	0.655	No	No
97893_at	Tbpl1	TATA box binding protein-like 1	0.9739	0.6142	0.0955	0.3919	No	1.0122	0.744	0.49	0.448	No	No
97894_at	Vars2	valyl-tRNA synthetase 2	1.0183	0.3834	0.001	0.2227	No	1.1062	0.0287	0.0123	0.0231	No	No
97895_f_at	Hat1	histone aminotransferase 1	0.9627	0.0736	0.0258	0.0095	No	0.9879	0.772	0.592	0.419	No	No
97896_r_at	Hat1	histone aminotransferase 1	1.0695	0.4119	0.0187	0.5947	No	0.9136	0.0682	0.006	0.969	No	No
97897_at	C78339	expressed sequence C78339	0.9068	0.0342	0.0216	0.2401	No	0.8855	0.0288	0.0049	0.889	No	No
97900_at	Txndc9	thioredoxin domain containing 9	1.017	0.7756	0.0249	0.0806	No	1.0882	0.307	0.0029	0.0578	No	No
97901_at	Ubtf	upstream binding transcription factor, RNA polymerase I	1.0236	0.5653	0.028	0.6376	No	1.1163	0.0096	0.0133	0.446	Yes	No
97903_at	Tsen34	tRNA splicing endonuclease 34 homolog (SEN34, S. cerevisiae)	1.0939	0.0099	0.0015	0.3052	Yes	1.1416	0.0039	0.0005	0.539	Yes	Yes
97904_at	Actr3	ARP3 actin-related protein 3 homolog (yeast)	1.053	0.4407	0.17	0.8864	No	0.9771	0.354	0.002	0.988	No	No
97906_at	Siah2	seven in absentia 2	0.9997	0.9948	0.3124	0.7356	No	1.0523	0.27	0.263	0.351	No	No
97907_at	Lsm7	LSM7 homolog, U6 small nuclear RNA associated (S. cerevisiae)	1.0084	0.8917	0.0259	0.4175	No	0.9981	0.96	0.0708	0.349	No	No
97908_at	Rmnd5a	required for meiotic nuclear division 5 homolog A (S. cerevisiae)	0.9774	0.5837	0.0038	0.0775	No	0.9654	0.339	0.002	0.102	No	No
97909_at	Stmn1	stathmin 1	1.0248	0.5201	0.008	0.6202	No	1.0828	0.0686	0.0335	0.167	No	No
97910_at	Stmn1 /// LOC639458	stathmin 1 /// similar to Stathmin (Phosphoprotein p19) (pp19) (Oncoprotein 18) (Op18) (Leukemia-associated phosphoprotein p18) (pp17) (Prosolin) (Metablastin) (Pr22 protein) (Leukemia-associated gene protein)	1.0159	0.8018	0.448	0.452	No	1.0844	0.16	0.717	0.564	No	No
97911_at	0910001L09Rik	RIKEN cDNA 0910001L09 gene	1.0433	0.3828	0.0632	0.9692	No	1.0683	0.212	0.0047	0.07	No	No
97912_at	Tspan3	tetraspanin 3	0.9763	0.8244	0.0073	0.7291	No	1.1495	0.0704	0.0369	0.572	No	No
97914_at	Hspa9a	heat shock protein 9A	1.0292	0.5875	0.1641	0.4513	No	1.0347	0.79	0.13	0.389	No	No
97915_at	Plekhb2	pleckstrin homology domain containing, family B (evectins) member 2	1.0196	0.6762	0.531	0.7447	No	1.0122	0.685	0.683	0.781	No	No
97916_at	5730494N06Rik	RIKEN cDNA 5730494N06 gene	1.0668	0.2429	0.6225	0.2186	No	1.0865	0.126	0.0874	0.918	No	No
97917_at	Bloc1s1	biogenesis of lysosome-related organelles complex-1, subunit 1	1.0018	0.9628	0.0838	0.0375	No	1.0016	0.987	0.951	0.381	No	No
97918_at	AA536743	expressed sequence AA536743	1.0133	0.6837	0.0004	0.041	No	1.0171	0.343	0.0007	0.0416	No	No
97919_at	Prpf38b	PRP38 pre-mRNA processing factor 38 (yeast) domain containing B	0.98	0.6993	0.0005	0.1229	No	1.002	0.668	0.0004	0.182	No	No
97920_at	Krt2-7	keratin complex 2, basic, gene 7	0.9679	0.4387	0.2318	0.0149	No	0.9617	0.262	0.105	0.0409	No	No
97921_at	Agrn	agrin	1.0427	0.3984	0.2255	0.2269	No	1.0024	0.951	0.15	0.858	No	No
97922_at	Cyb5r4	cytochrome b5 reductase 4	0.9303	0.2198	0.0846	0.7724	No	0.983	0.762	0.0485	0.809	No	No
97923_at	Herc1	hect (homologous to the E6-AP (UBE3A) carboxyl terminus) domain and RCC1 (CHC1)-like domain (RLD) 1	1.0904	0.0104	0	0.5939	No	0.9718	0.67	0	0.166	No	No
97924_at	Gne	glucosamine	1.0087	0.839	0.1957	0.2522	No	1.0237	0.68	0.314	0.802	No	No
97925_at	Csnk1e	casein kinase 1, epsilon	1.047	0.2114	0.0005	0.9908	No	1.1201	0.0482	0.0012	0.15	No	No
97926_s_at	Pparg	peroxisome proliferator activated receptor gamma	1.0696	0.3947	0.0731	0.9981	No	1.0834	0.27	0.0454	0.979	No	No
97927_at	Rhog	ras homolog gene family, member G	0.9761	0.1732	0.0001	0.297	No	0.973	0.223	0.183	0.487	No	No
97928_at	Cln6	ceroid-lipofuscinosis, neuronal 6	0.9218	0.1097	0.042	0.3457	No	0.9319	0.0122	0.0981	0.0628	No	No
97929_r_at	Psmd9	proteasome (prosome, macropain) 26S subunit, non-ATPase, 9	1.0109	0.7682	0.154	0.6986	No	1.0102	0.584	0.0008	0.21	No	No
97930_f_at	Cd151	CD151 antigen	0.981	0.371	0.2921	0.025	No	0.9914	0.859	0.313	0.382	No	No
97931_i_at	Cd151	CD151 antigen	1.0349	0.4683	0.0062	0.5442	No	0.953	0.568	0.0475	0.832	No	No
97932_f_at	Cd151	CD151 antigen	1.0268	0.5211	0.9054	0.2373	No	1.0096	0.945	0.17	0.103	No	No
97933_at	2010111I01Rik	RIKEN cDNA 2010111I01 gene	0.979	0.3514	0.0124	0.0094	No	0.9608	0.133	0.0031	0.0348	No	No
97934_at	Galnt1	UDP-N-acetyl-alpha-D-galactosamine:polypeptide N-acetylgalactosaminyltransferase 1	0.9949	0.9006	0.5077	0.7331	No	1.0118	0.492	0.0014	0.068	No	No
97935_at	4121402D02Rik	RIKEN cDNA 4121402D02 gene	0.9311	0.0917	0.7979	0.0503	No	0.9583	0.0532	0.0012	0.0054	No	No
97936_at	Zfp746	zinc finger protein 746	1.056	0.5638	0.1357	0.6039	No	0.9642	0.387	0.409	0.666	No	No
97937_at	Klf5	Kruppel-like factor 5	0.9753	0.1399	0.0002	0.0002	No	0.9687	0.217	0.0078	0.0009	No	No
97939_at	Txndc1	thioredoxin domain containing 1	1.0203	0.6942	0.4164	0.77	No	1.029	0.536	0.0105	0.311	No	No
97940_at	Csh1	chorionic somatomammotropin hormone 1	1.0624	0.1384	0.0062	0.7723	No	1.099	0.0449	0.001	0.605	No	No
97941_at	Capn6	calpain 6	0.9517	0.261	0.0015	0.8584	No	0.9342	0.256	0.0208	0.567	No	No
97942_g_at	Capn6	calpain 6	0.9983	0.9631	0.1194	0.0824	No	0.9752	0.29	0.14	0.0242	No	No
97943_at	Capn6	calpain 6	1.0028	0.9081	0.0003	0.0667	No	0.9511	0.0837	0.0003	0.154	No	No
97944_f_at	Tcra /// A430107P09Rik	T-cell receptor alpha chain /// RIKEN cDNA A430107P09 gene	0.9569	0.4702	0.067	0.4043	No	0.912	0.241	0.116	0.468	No	No
97945_at	Tcra /// A130082M07Rik /// A430107P09Rik	T-cell receptor alpha chain /// RIKEN cDNA A130082M07 gene /// RIKEN cDNA A430107P09 gene	1.0255	0.415	0.1456	0.5753	No	0.9903	0.808	0.0453	0.373	No	No
97946_at	Prlpf	prolactin-like protein F	0.9872	0.7581	0.5389	0.7982	No	0.8873	0.443	0.93	0.42	No	No
97947_at	---	---	1.045	0.5614	0.0039	0.1096	No	1.0126	0.858	0.212	0.799	No	No
97948_at	Rb1	retinoblastoma 1	1.0193	0.8587	0.0378	0.9804	No	0.945	0.435	0.456	0.943	No	No
97949_at	Fgl2	fibrinogen-like protein 2	0.9015	0.0648	0.0021	0.2335	No	0.8633	0.003	0.0014	0.199	Yes	No
97950_at	Xdh	xanthine dehydrogenase	0.9572	0.4088	0.1371	0.7222	No	0.9624	0.594	0.664	0.814	No	No
97951_s_at	Tsc2	tuberous sclerosis 2	0.9566	0.1536	0.0027	0.1487	No	0.9541	0.0039	0	0.0005	No	No
97952_at	Tsc2	tuberous sclerosis 2	1.0667	0.2775	0.0081	0.3701	No	1.087	0.403	0.253	0.0956	No	No
97953_g_at	Tsc2	tuberous sclerosis 2	0.9928	0.8071	0.0022	0.9986	No	0.9272	0.0168	0.0373	0.004	No	No
97954_at	Tyrp1	tyrosinase-related protein 1	1.0069	0.8968	0.6948	0.4621	No	1.0262	0.549	0.989	0.0916	No	No
97955_at	Tyrp1	tyrosinase-related protein 1	0.8732	0.0315	0.8555	0.0725	No	0.8766	0.182	0.465	0.393	No	No
97956_g_at	Tyrp1	tyrosinase-related protein 1	1.0072	0.8757	0.7449	0.1508	No	1.1233	0.113	0.378	0.0699	No	No
97957_at	Slc27a4	solute carrier family 27 (fatty acid transporter), member 4	0.9966	0.8624	0.0054	0.0012	No	0.9138	0.0075	0.0626	0.0009	No	No
97958_at	Prkcbp1	protein kinase C binding protein 1	0.873	0.0865	0.6734	0.8972	No	0.7912	0.0335	0.729	0.174	No	No
97960_at	Usp22	ubiquitin specific peptidase 22	1.0848	0.2516	0.0415	0.1808	No	1.0796	0.232	0.0032	0.133	No	No
97962_at	Synj2	synaptojanin 2	1.0299	0.6516	0.2089	0.2084	No	0.9408	0.126	0.135	0.0298	No	No
97963_at	Sipa1	signal-induced proliferation associated gene 1	1.0114	0.516	0.0059	0.7401	No	1.0331	0.264	0.0164	0.607	No	No
97964_at	Fkbp11	FK506 binding protein 11	1.0496	0.4765	0.0106	0.9598	No	1.0364	0.0981	0.0001	0.0437	No	No
97965_at	Pla2g6	phospholipase A2, group VI	0.9341	0.2012	0.3825	0.1579	No	0.8522	0.0245	0.78	0.0397	No	No
97966_at	Ncoa5	nuclear receptor coactivator 5	1.0345	0.6606	0.096	0.9538	No	1.0264	0.496	0.0069	0.776	No	No
97967_at	Plxnd1	Plexin D1	1.029	0.6585	0.2144	0.5907	No	1.0675	0.185	0.471	0.319	No	No
97969_at	Nr1h4	nuclear receptor subfamily 1, group H, member 4	0.9796	0.5387	0.2627	0.673	No	0.9557	0.331	0.0083	0.579	No	No
97970_at	Mfi2	antigen p97 (melanoma associated) identified by monoclonal antibodies 133.2 and 96.5	0.9392	0.0403	0	0.8488	No	0.9453	0.348	0.058	0.859	No	No
97971_at	Phkg2	phosphorylase kinase, gamma 2 (testis)	0.9407	0.1996	0.6199	0.0327	No	0.9536	0.384	0.168	0.607	No	No

97972_at	Rnf103	ring finger protein 103	1.041	0.691	0.0261	0.8372	No	0.9882	0.812	0.0258	0.68	No	No
97973_at	Tal1	T-cell acute lymphocytic leukemia 1	1.0576	0.3195	0.6133	0.3888	No	1.1058	0.171	0.395	0.179	No	No
97974_at	Zfpm1	zinc finger protein, multitype 1	1.03	0.5564	0.2541	0.5946	No	1.0465	0.358	0.763	0.174	No	No
97975_at	Nfatc3	nuclear factor of activated T-cells, cytoplasmic, calcineurin-dependent 3	1.0491	0.0798	0.0005	0.099	No	1.0223	0.737	0.0104	0.0177	No	No
97976_at	Ktn1	kinectin 1	0.9792	0.3453	0.0044	0.1406	No	1.021	0.74	0.412	0.183	No	No
97977_at	Ntn1	netrin 1	0.992	0.8435	0.0179	0.3049	No	1.0762	0.0492	0.0409	0.308	No	No
97979_at	Ppp1r7	protein phosphatase 1, regulatory (inhibitor) subunit 7	0.9604	0.1299	0.0486	0.1555	No	1.0053	0.899	0.918	0.198	No	No
97980_at	Ltbr	lymphotoxin B receptor	0.9501	0.2663	0.0339	0.3177	No	0.9835	0.791	0.0122	0.306	No	No
97982_at	Emid2	EMI domain containing 2	1.053	0.2085	0.0232	0.3418	No	1.0373	0.0583	0.0001	0.147	No	No
97983_s_at	Stxbp1	syntaxin binding protein 1	0.9061	0.1446	0.006	0.2637	No	0.9596	0.292	0.0005	0.589	No	No
97984_i_at	Kns2	kinesin 2	1.0095	0.8426	0.0247	0.6268	No	1.0289	0.583	0.425	0.559	No	No
97985_f_at	Kns2	kinesin 2	0.9821	0.6476	0.3127	0.363	No	1.0232	0.297	0.0059	0.231	No	No
97986_at	Entpd2	ectonucleoside triphosphate diphosphohydrolase 2	0.9993	0.9816	0.0114	0.0584	No	0.9697	0.562	0.0363	0.93	No	No
97987_at	Igfals	insulin-like growth factor binding protein, acid labile subunit	0.9563	0.1104	0.0032	0.6816	No	0.9755	0.391	0.92	0.634	No	No
97988_at	Mrg1	myeloid ecotropic viral integration site-related gene 1	1.08	0.1531	0.0282	0.2985	No	0.9635	0.587	0.0466	0.924	No	No
97989_at	Ppp3cb	protein phosphatase 3, catalytic subunit, beta isoform	1.0218	0.7317	0.0144	0.8413	No	0.9556	0.0266	0	0.0001	No	No
97990_at	Myh11	myosin, heavy polypeptide 11, smooth muscle	0.9201	0.184	0.0342	0.3169	No	0.9472	0.358	0.0034	0.019	No	No
97991_at	Kras	v-Ki-ras2 Kirsten rat sarcoma viral oncogene homolog	1.1125	0	0.0003	0.0001	No	1.0512	0.392	0.0149	0.529	No	No
97992_at	Smg5	Smg-5 homolog, nonsense mediated mRNA decay factor (C. elegans)	0.9563	0.4473	0.0239	0.4532	No	0.9272	0.112	0.022	0.596	No	No
97993_at	Uros	uroporphyrinogen III synthase	1.0032	0.8959	0.0382	0.5211	No	1.095	0.109	0.0055	0.0902	No	No
97994_at	Tcf7	transcription factor 7, T-cell specific	0.9948	0.9321	0.6773	0.8402	No	1.0209	0.688	0.01	0.334	No	No
97995_at	Tcf7	transcription factor 7, T-cell specific	0.9784	0.5526	0.122	0.6033	No	0.9496	0.0744	0.0104	0.899	No	No
97996_at	Papola	poly (A) polymerase alpha	0.9996	0.9935	0.008	0.4812	No	1.1477	0.0776	0.0133	0.203	No	No
97997_at	Sfrp1	secreted frizzled-related sequence protein 1	1.0693	0.3932	0.0077	0.5098	No	1.0038	0.946	0.0274	0.81	No	No
97998_at	Atn1	atrophin 1	0.8588	0.0807	0.1315	0.7455	No	0.9622	0.689	0.174	0.934	No	No
98000_at	Muc13	mucin 13, epithelial transmembrane	0.9894	0.9219	0.7846	0.4959	No	0.9817	0.85	0.558	0.73	No	No
98001_at	Arhgef1	Rho guanine nucleotide exchange factor (GEF) 1	0.8746	0.0572	0.0517	0.2702	No	0.9316	0.0583	0.002	0.0372	No	No
98002_at	Irf8	interferon regulatory factor 8	0.9621	0.5193	0.3078	0.2624	No	1.0698	0.0628	0.0137	0.0572	No	No
98003_at	Lilrb3 /// LOC619608	leukocyte immunoglobulin-like receptor, subfamily B (with TM and ITIM domains), member 3 /// hypothetical LOC619608	0.9499	0.6221	0.2076	0.8825	No	1.065	0.499	0.459	0.382	No	No
98004_at	Pkia	protein kinase inhibitor, alpha	1.0133	0.8312	0.0223	0.8154	No	0.9382	0.406	0.0275	0.717	No	No
98005_at	Pkia	protein kinase inhibitor, alpha	1.126	0.0382	0.001	0.4412	No	1.058	0.428	0.0005	0.439	No	No
98006_at	Pola2	polymerase (DNA directed), alpha 2	1.0201	0.6261	0.9834	0.341	No	1.0058	0.914	0.0953	0.0676	No	No
98007_at	Rps6ka2	ribosomal protein S6 kinase, polypeptide 2	1.0367	0.2455	0.1495	0.2411	No	1.0398	0.0721	0.0068	0.131	No	No
98008_at	Cx3cl1	chemokine (C-X3-C motif) ligand 1	0.8976	0.0308	0.0043	0.3601	No	0.9207	0.0814	0.0031	0.706	No	No
98010_at	Nfe2	nuclear factor, erythroid derived 2	0.958	0.3742	0.2046	0.6159	No	1.0656	0.0915	0.362	0.0032	No	No
98011_at	Gabbr1	gamma-aminobutyric acid (GABA-B) receptor, 1	0.9693	0.4914	0.0988	0.6298	No	0.9442	0.0945	0.0085	0.0466	No	No
98013_at	D15Ertd621e	DNA segment, Chr 15, ERATO Doi 621, expressed	0.9838	0.9042	0.0133	0.9873	No	0.9864	0.788	0.006	0.596	No	No
98014_at	Itih1	inter-alpha trypsin inhibitor, heavy chain 1	1.1103	0.3706	0.2421	0.76	No	1.0673	0.107	0.0436	0.0031	No	No
98015_at	Scamp3	secretory carrier membrane protein 3	0.9948	0.9103	0.5196	0.1809	No	1.0565	0.389	0.994	0.438	No	No
98016_at	D3Wsu161e	DNA segment, Chr 3, Wayne State University 161, expressed	1.0094	0.8337	0.0053	0.1497	No	0.987	0.603	0.0001	0.976	No	No
98017_at	Pelo	pelota homolog (Drosophila)	1.0513	0.2496	0.0019	0.0289	No	1.0383	0.336	0.0066	0.111	No	No
98018_at	Procr	protein C receptor, endothelial	0.9231	0.0857	0.1298	0.0788	No	0.9388	0.302	0.068	0.437	No	No
98019_at	Tgfb1i1	transforming growth factor beta 1 induced transcript 1	0.9994	0.9834	0.0339	0.1991	No	1.0455	0.0135	0.0614	0.0111	No	No
98020_at	Drg1	developmentally regulated GTP binding protein 1	0.968	0.5234	0.3955	0.3143	No	0.8989	0.0259	0.806	0.025	No	No
98021_at	Polr1e	polymerase (RNA) I polypeptide E	1.0204	0.6826	0.0068	0.5324	No	1.1346	0.108	0.028	0.937	No	No
98022_at	Pcsk7	proprotein convertase subtilisin/kexin type 7	0.9931	0.8113	0.8342	0.7149	No	1.0902	0.0697	0.192	0.894	No	No
98023_r_at	Plfr	proliferin related protein	1.0408	0.3511	0.8553	0.9531	No	1.3695	0.0476	0.0608	0.126	No	No
98024_at	Nfyb	nuclear transcription factor-Y beta	0.985	0.8131	0.0087	0.3959	No	0.987	0.719	0.0045	0.99	No	No
98025_at	Evi2a	ecotropic viral integration site 2a	1.1857	0.126	0.4052	0.9771	No	1.174	0.172	0.432	0.841	No	No
98026_g_at	Evi2a	ecotropic viral integration site 2a	1.1701	0.0024	0.0053	0.0128	Yes	1.0911	0.198	0.0469	0.691	No	No
98027_at	Col9a2	procollagen, type IX, alpha 2	0.9341	0.3272	0.0003	0.968	No	0.9084	0.0524	0	0.229	No	No
98028_at	Twist1	twist gene homolog 1 (Drosophila)	1.0164	0.7291	0.4846	0.7279	No	0.9509	0.411	0.388	0.765	No	No
98029_at	3110056O03Rik	RIKEN cDNA 3110056O03 gene	0.9966	0.9294	0.0195	0.0521	No	1.064	0.0491	0.0055	0.65	No	No
98030_at	Trim30	tripartite motif protein 30	0.9586	0.5149	0.2451	0.3079	No	0.9939	0.943	0	0.823	No	No
98031_at	Bok	Bcl-2-related ovarian killer protein	1.0394	0.1227	0.0026	0.6962	No	1.1034	0.0939	0.0944	0.0559	No	No
98032_at	Zfp35	zinc finger protein 35	0.9393	0.1422	0.5539	0.3813	No	1.0158	0.538	0.0627	0.162	No	No
98033_at	1100001H23Rik	RIKEN cDNA 1100001H23 gene	1.0267	0.4119	0.1592	0.6411	No	1.0416	0.333	0.431	0.17	No	No
98034_at	H2-DMb1	histocompatibility 2, class II, locus Mb1	0.8378	0.121	0.0237	0.1169	No	0.9606	0.68	0.0246	0.407	No	No
98035_g_at	H2-DMb1	histocompatibility 2, class II, locus Mb1	0.9772	0.5863	0.101	0.9423	No	1.009	0.774	0.119	0.769	No	No
98036_at	Marveld1	MARVEL (membrane-associating) domain containing 1	0.9426	0.0053	0	0.4297	Yes	0.949	0.0588	0.0184	0.187	No	No
98037_at	Hbxip	hepatitis B virus x interacting protein	1.0313	0.4727	0.0429	0.0575	No	0.9827	0.887	0.0051	0.032	No	No
98038_at	Hmgb3	high mobility group box 3	1.1617	0.0305	0.0178	0.1743	No	1.1694	0.0232	0.0549	0.365	No	No
98039_at	2410015M20Rik	RIKEN cDNA 2410015M20 gene	1.0283	0.0598	0.0001	0.0003	No	0.9831	0.761	0.217	0.0163	No	No
98040_at	Tcfe2a	transcription factor E2a	1.0797	0.1395	0.0124	0.0464	No	1.1188	0.135	0.919	0.183	No	No
98041_at	Rnase1	ribonuclease, RNase A family, 1 (pancreatic)	1.04	0.7062	0.0201	0.0831	No	1.3269	0.0041	0.0009	0.933	Yes	No
98042_at	Scpep1	serine carboxypeptidase 1	1.0753	0.0827	0.131	0.5897	No	1.0548	0.368	0.439	0.579	No	No
98044_at	Dab2	disabled homolog 2 (Drosophila)	0.9863	0.8214	0.7787	0.5137	No	0.8744	0.0459	0.151	0.14	No	No
98045_s_at	Dab2	disabled homolog 2 (Drosophila)	1.0359	0.5888	0.5929	0.9182	No	0.9933	0.757	0.0005	0.725	No	No
98047_at	5730410I19Rik	RIKEN cDNA 5730410I19 gene	1.0177	0.6589	0.4601	0.7629	No	0.9939	0.888	0.325	0.672	No	No
98048_at	Fusip1 /// LOC432730	FUS interacting protein (serine-arginine rich) 1 /// hypothetical LOC432730	0.9912	0.9358	0.6575	0.4621	No	1.0418	0.733	0.715	0.839	No	No
98049_at	1300018I05Rik	RIKEN cDNA 1300018I05 gene	0.9748	0.5917	0.0583	0.0974	No	1.0918	0.392	0.24	0.621	No	No
98051_at	Ric8	resistance to inhibitors of cholinesterase 8 homolog (C. elegans)	1.0452	0.1508	0.6219	0.0529	No	1.0285	0.381	0.03	0.494	No	No
98052_at	Bet1l	blocked early in transport 1 homolog (S. cerevisiae)-like	1.0017	0.9698	0.0778	0.2391	No	1.0861	0.0242	0.794	0.073	No	No
98053_at	Ywhab	tyrosine 3-monooxygenase/tryptophan 5-monooxygenase activation protein, beta polypeptide	1.1672	0.042	0.2617	0.796	No	1.1533	0.0026	0.0131	0.585	Yes	Yes
98054_at	Ecm1	extracellular matrix protein 1	0.8942	0.0419	0.0016	0.2809	No	0.9935	0.936	0.0001	0.635	No	No
98055_at	Blcap	bladder cancer associated protein homolog (human)	0.856	0.0388	0.0069	0.8348	No	0.981	0.604	0.0447	0.373	No	No
98056_at	Phlda3	pleckstrin homology-like domain, family A, member 3	0.9972	0.8074	0.0217	0.006	No	0.9718	0.568	0.273	0.269	No	No
98057_at	Rnf166	ring finger protein 166	0.9293	0.1207	0.4954	0.088	No	0.9567	0.105	0.495	0.0419	No	No
98059_s_at	Lmna	lamin A	0.9398	0.3222	0.0068	0.2808	No	0.9856	0.847	0.0045	0.528	No	No
98060_at	Lmna	lamin A	0.8875	0.0022	0	0.6734	Yes	0.9636	0.402	0.166	0.977	No	No
98061_at	Nudt16l1	nudix (nucleoside diphosphate linked moiety X)-type motif 16-like 1	1.0542	0.1288	0.8705	0.8035	No	1.0227	0.704	0.805	0.615	No	No
98063_at	Glycam1	glycosylation dependent cell adhesion molecule 1	0.9702	0.6072	0.2877	0.8547	No	1.1277	0.118	0.0362	0.0704	No	No
98064_at	Aamp	angio-associated migratory protein	1.0121	0.7976	0.6143	0.3729	No	1.0081	0.58	0.0006	0.14	No	No
98065_at	Ormdl3	ORM1-like 3 (S. cerevisiae)	1.0988	0.0519	0.1966	0.5358	No	1.0929	0.0234	0.187	0.0727	No	No
98066_r_at	Brd2	bromodomain containing 2	1.0261	0.4579	0.0007	0.0423	No	0.9904	0.751	0.0172	0.23	No	No
98067_at	Cdkn1a	cyclin-dependent kinase inhibitor 1A (P21)	0.9688	0.0889	0	0.0061	No	0.9519	0.0852	0.0003	0.918	No	No
98069_s_at	2400003N08Rik	RIKEN cDNA 2400003N08 gene	1.0657	0.1108	0.0036	0.0491	No	1.1218	0.0424	0.0017	0.315	No	No
98070_at	2400003N08Rik	RIKEN cDNA 2400003N08 gene	0.9932	0.8567	0.4199	0.0237	No	0.8945	0.0924	0.031	0.0867	No	No
98071_f_at	Dck	deoxycytidine kinase	1.1251	0.1651	0.0135	0.1857	No	1.0804	0.0784	0.0048	0.0336	No	No
98072_r_at	Dck	deoxycytidine kinase	1.1755	0.0993	0.0035	0.1451	No	1.4288	0.0228	0.0047	0.0432	No	No
98073_at	Cutl1	cut-like 1 (Drosophila)	0.9713	0.4643	0.0376	0.8615	No	0.965	0.0367	0.915	0.0275	No	No
98075_at	G431001I09Rik	RIKEN cDNA G431001I09 gene	1.0698	0.0582	0.3295	0.8851	No	0.9655	0.489	0.524	0.853	No	No
98076_at	Erp29	endoplasmic reticulum protein 29	1.018	0.4751	0.0001	0.085	No	1.0938	0.0006	0	0.0051	No	No
98077_at	Snrpd3	small nuclear ribonucleoprotein D3	1.0608	0.5801	0.0336	0.1937	No	1.0756	0.332	0.0362	0.386	No	No
98078_at	Aph1a	anterior pharynx defective 1a homolog (C. elegans)	1.0371	0.5477	0.1085	0.1911	No	1.0761	0.0059	0.602	0.001	No	No

98079_at	Car14	carbonic anhydrase 14	0.8204	0.0145	0.0242	0.5721	No	0.9007	0.005	0.0008	0.15	Yes	Yes
98081_at	Rpo1-3	RNA polymerase 1-3	0.9738	0.6679	0.0157	0.0773	No	0.9664	0.784	0.0088	0.298	No	No
98082_at	Rpo1-3	RNA polymerase 1-3	0.9583	0.3758	0.3963	0.2593	No	0.9736	0.479	0.0773	0.115	No	No
98083_at	Klf6	Kruppel-like factor 6	0.8108	0.014	0.9357	0.1163	No	0.9107	0.126	0.427	0.349	No	No
98084_at	Arl2bp	ADP-ribosylation factor-like 2 binding protein	1.0358	0.0046	0	0.0291	Yes	1.0809	0.0188	0.0014	0.24	No	Yes
98085_f_at	Tia1	Cytotoxic granule-associated RNA binding protein 1	1.0545	0.0488	0.2945	0.0271	No	1.0286	0.0328	0.0205	0.0008	No	No
98086_r_at	Rps28	ribosomal protein S28	0.9555	0.6651	0.0005	0.9464	No	1.0102	0.959	0.0005	0.677	No	No
98087_at	Tbk1	TANK-binding kinase 1	1.0147	0.7582	0.0014	0.2488	No	1.0447	0.288	0.0981	0.167	No	No
98088_at	Cd14	CD14 antigen	0.9781	0.5382	0.0192	0.6681	No	0.9562	0.386	0.0041	0.504	No	No
98089_at	1190005P17 Rik	RIKEN cDNA 1190005P17 gene	1.0527	0.4074	0.4607	0.1118	No	0.9925	0.876	0.934	0.118	No	No
98090_at	Krr1	KRR1, small subunit (SSU) processome component, homolog (yeast)	0.9238	0.0226	0.6064	0.0144	No	0.955	0.475	0.158	0.609	No	No
98092_at	Plac8	placenta-specific 8	0.9668	0.4766	0.6446	0.2195	No	0.9338	0.0128	0.038	0.0019	No	No
98094_f_at	Amfr	autocrine motility factor receptor	1.0304	0.1063	0.0001	0.5983	No	1.0292	0.534	0.165	0.0971	No	No
98096_f_at	Cog8	component of oligomeric golgi complex 8	0.9461	0.376	0.0103	0.2746	No	0.9485	0.614	0.0839	0.583	No	No
98097_r_at	Cog8	component of oligomeric golgi complex 8	1.0258	0.6679	0.0067	0.1607	No	1.0318	0.2	0.0006	0.0945	No	No
98098_at	Car1	carbonic anhydrase 1	1.0327	0.5781	0.8188	0.7567	No	1.0689	0.344	0.0437	0.402	No	No
98099_at	Nudt9	nudix (nucleoside diphosphate linked moiety X)-type motif 9	1.0078	0.8961	0.0267	0.3835	No	1.0182	0.695	0.326	0.444	No	No
98101_at	Gtf2a2	general transcription factor II A, 2	1.1185	0.0905	0.3304	0.826	No	1.0165	0.505	0.0332	0.0216	No	No
98102_at	Pdha1	pyruvate dehydrogenase E1 alpha 1	0.8985	0.0326	0.065	0.3594	No	0.9124	0.0587	0.0048	0.382	No	No
98104_at	Atp6v0b	ATPase, H+ transporting, lysosomal V0 subunit B	1.0737	0.1623	0.1976	0.4787	No	1.009	0.86	0.0133	0.884	No	No
98106_at	Timm44	translocase of inner mitochondrial membrane 44	0.9208	0.0054	0.1535	0.029	Yes	0.9587	0.361	0.0212	0.486	No	No
98107_at	Coro1c	coronin, actin binding protein 1C	0.9901	0.8043	0.0157	0.595	No	1.0299	0.0195	0.0025	0.488	No	No
98108_at	Crabp1	cellular retinoic acid binding protein I	1.2095	0.0015	0.2782	0.0728	Yes	1.2167	0.0157	0.667	0.225	No	Yes
98109_at	Mrpl55	mitochondrial ribosomal protein L55	0.96	0.4376	0.2281	0.2421	No	1.0144	0.752	0.0008	0.958	No	No
98110_at	Mdm2	transformed mouse 3T3 cell double minute 2	1.1059	0.0898	0.0053	0.5896	No	0.9864	0.831	0.34	0.217	No	No
98111_at	Hsp110	heat shock protein 110	0.9627	0.4043	0.0041	0.0809	No	0.9602	0.414	0.0059	0.277	No	No
98112_r_at	Lap3	leucine aminopeptidase 3	1.1101	0.3102	0.0244	0.2759	No	1.2257	0.174	0.016	0.927	No	No
98113_at	Psmb1	proteasome (prosome, macropain) subunit, beta type 1	1.1248	0.0072	0.0027	0.0954	Yes	0.9905	0.97	0.0003	0.0531	No	No
98114_at	Npc1	Niemann Pick type C1	1.0537	0.0768	0.0101	0.0682	No	1.1147	0.0421	0.0257	0.0211	No	No
98116_at	Hpxn	hemopexin	0.9775	0.7097	0.0511	0.061	No	1.1237	0.105	0.314	0.503	No	No
98117_at	Ndufa1	NADH dehydrogenase (ubiquinone) 1 alpha subcomplex, 1	0.9862	0.8027	0.1404	0.3543	No	1.0054	0.814	0.0891	0.0564	No	No
98118_at	Ndufa1	NADH dehydrogenase (ubiquinone) 1 alpha subcomplex, 1	0.877	0.3749	0.0013	0.259	No	1.3198	0.0986	0	0.12	No	No
98119_at	Rpl30 /// LOC625281 /// LOC664969 /// LOC666899 /// LOC667779 /// LOC669479 /// LOC670014 /// LOC671807 /// LOC675317 /// LOC675333 /// LOC677010	ribosomal protein L30 /// similar to ribosomal protein L30 /// similar to ribosomal protein L30 /// similar to ribosomal protein L30 /// similar to ribosomal protein L30 /// similar to ribosomal protein L30 /// similar to ribosomal protein L30 /// similar to ribosomal protein L30 /// similar to ribosomal protein L30	1.044	0.2174	0.004	0.021	No	1.0176	0.356	0.0013	0.0231	No	No
98120_at	Mrpl27	mitochondrial ribosomal protein L27	1.0038	0.921	0.0289	0.0796	No	0.9989	0.609	0.0008	0.0082	No	No
98121_at	Fnta	farnesyltransferase, CAAX box, alpha	0.9967	0.9511	0.5305	0.9275	No	1.0287	0.683	0.475	0.576	No	No
98122_at	Lmo4	LIM domain only 4	1.0403	0.6422	0.0101	0.937	No	1.0188	0.311	0	0.201	No	No
98123_at	Aadat	aminoadipate aminotransferase	1.0309	0.6582	0.4569	0.5572	No	1.0778	0.483	0.105	0.837	No	No
98124_at	0610011F06 Rik	RIKEN cDNA 0610011F06 gene	0.9485	0.1642	0.0083	0.0231	No	1.0014	0.929	0.246	0.0374	No	No
98125_at	Tm2d3	TM2 domain containing 3	0.99	0.8134	0.225	0.8428	No	0.9347	0.335	0.0115	0.389	No	No
98126_s_at	Atp2a1	ATPase, Ca++ transporting, cardiac muscle, fast twitch 1	0.6074	0.0008	0.0813	0.0034	No	0.7637	0.0006	0.95	0.0023	No	No
98127_at	Capza2	capping protein (actin filament) muscle Z-line, alpha 2	0.9917	0.9	0.1562	0.6597	No	0.9559	0.51	0.391	0.737	No	No
98128_at	Atp5j	ATP synthase, H+ transporting, mitochondrial F0 complex, subunit F	1.099	0.0786	0.0068	0.1238	No	1.0374	0.432	0.015	0.213	No	No
98129_at	Tmsb10	thymosin, beta 10	1.0602	0.2506	0.7867	0.4546	No	1.0228	0.524	0.0727	0.215	No	No
98130_at	Txn2	thioredoxin 2	1.021	0.5734	0.2863	0.3739	No	1.0445	0.132	0.108	0.561	No	No
98131_at	Cryz	crystallin, zeta	1.0079	0.7834	0.0959	0.1738	No	1.0105	0.841	0.0622	0.065	No	No
98132_at	Cycs /// LOC670717 /// LOC672195	cytochrome c, somatic /// similar to Cytochrome c, somatic /// similar to Cytochrome c, somatic	1.023	0.6556	0.0028	0.1364	No	1.0748	0.42	0.0297	0.166	No	No
98133_at	Calb1	calbindin-28K	1.2266	0.0156	0.0081	0.902	No	1.1008	0.212	0.0669	0.63	No	No
98134_at	2610101N10 Rik	RIKEN cDNA 2610101N10 gene	0.9978	0.9448	0.2314	0.6292	No	1.0077	0.876	0.929	0.39	No	No
98135_r_at	Otud6b	OTU domain containing 6B	0.9834	0.5171	0.0115	0.1612	No	1.2419	0.167	0.0431	0.142	No	No
98136_at	Sms /// LOC671878	spermine synthase /// similar to Spermine synthase (Spermidine aminopropyltransferase) (SPMSY)	0.979	0.4884	0.0104	0.6603	No	0.9949	0.914	0.831	0.795	No	No
98137_at	Car5a	carbonic anhydrase 5a, mitochondrial	1.0684	0.2727	0.0395	0.2327	No	0.9737	0.515	0.01	0.612	No	No
98138_at	Rpl7l1	ribosomal protein L7-like 1	0.9263	0.2784	0.7067	0.1823	No	1.0278	0.424	0.0136	0.133	No	No
98139_at	Vdac1	voltage-dependent anion channel 1	1.0258	0.8066	0.0605	0.432	No	1.0356	0.562	0.614	0.122	No	No
98140_at	Cdh1	cadherin 1	1.0228	0.6469	0.0374	0.0614	No	0.9818	0.896	0.0564	0.684	No	No
98141_at	Eif5b	eukaryotic translation initiation factor 5B	0.8607	0.0863	0.047	0.8687	No	0.9459	0.26	0.293	0.83	No	No
98142_at	Eif5b	eukaryotic translation initiation factor 5B	0.9364	0.5164	0.0874	0.5489	No	1	0.78	0.0009	0.336	No	No
98143_at	Fut8	fucosyltransferase 8	0.9598	0.4916	0.7196	0.937	No	0.8933	0.0115	0.161	0.0946	No	No
98144_f_at	Tspan31	tetraspanin 31	0.9422	0.2025	0.0107	0.0822	No	0.957	0.298	0.161	0.232	No	No
98146_at	Nup88	nucleoporin 88	1.01	0.8379	0.3019	0.7172	No	1.0102	0.677	0.0004	0.747	No	No
98147_at	Usp5	ubiquitin specific peptidase 5 (isopeptidase T)	1.0311	0.0155	0.0002	0.0014	No	1.093	0.0345	0.351	0.117	No	No
98148_at	1110033J19 Rik	RIKEN cDNA 1110033J19 gene	1.1029	0.1542	0.6336	0.678	No	1.1005	0.108	0.0375	0.524	No	No
98149_s_at	1110033J19 Rik	RIKEN cDNA 1110033J19 gene	1.0148	0.7635	0.5232	0.6483	No	1.1059	0.108	0.764	0.355	No	No
98150_at	Rab11b	RAB11B, member RAS oncogene family	1.0556	0.2472	0.7614	0.7958	No	1.074	0.277	0.584	0.938	No	No
98151_s_at	Ctnnd1	catenin (cadherin associated protein), delta 1	0.9555	0.5302	0.0312	0.1326	No	0.8751	0.185	0.986	0.668	No	No
98152_at	Ctnnd1	catenin (cadherin associated protein), delta 1	0.9631	0.0986	0.0002	0.0071	No	0.9829	0.739	0.0026	0.0582	No	No
98153_at	Cct3	chaperonin subunit 3 (gamma)	1.0793	0.1019	0.0358	0.9815	No	1.0433	0.28	0.571	0.586	No	No
98154_at	Gtf3c2	general transcription factor IIIC, polypeptide 2, beta	1.0363	0.2332	0.0004	0.0729	No	1.0259	0.501	0.0047	0.164	No	No
98155_r_at	Pold2	polymerase (DNA directed), delta 2, regulatory subunit	1.0281	0.6012	0.0419	0.2046	No	0.9785	0.57	0.0489	0.0765	No	No
98168_at	Rpl7a	ribosomal protein L7a	1.0571	0.2643	0.1002	0.1059	No	1.0043	0.947	0.638	0.249	No	No
98169_s_at	Fzd3	frizzled homolog 3 (Drosophila)	1.1496	0.0417	0.0104	0.0659	No	1.1159	0.0549	0.0223	0.0718	No	No
98240_at	Il12rb1	interleukin 12 receptor, beta 1	0.9985	0.9803	0.2311	0.9053	No	1.0228	0.831	0.0399	0.215	No	No
98245_at	Csnk2a2	casein kinase II, alpha 2, polypeptide	1.0627	0.1269	0.0499	0.0997	No	0.9866	0.442	0.117	0.0204	No	No
98247_at	LOC637741	hypothetical protein LOC637741	0.9637	0.7203	0.2739	0.9881	No	1.0464	0.479	0.329	0.709	No	No
98254_f_at	Ccrn4l /// Sgip1 /// Gag /// LOC639390	CCR4 carbon catabolite repression 4-like (S. cerevisiae) /// SH3-domain GRB2-like (endophilin) interacting protein 1 /// Gag /// gag protein	0.9182	0.0873	0.0127	0.7089	No	0.9145	0.0163	0.0003	0.627	No	No
98276_at	Gab2	growth factor receptor bound protein 2-associated protein 2	0.9704	0.4991	0.0249	0.8951	No	0.9461	0.243	0.0117	0.266	No	No
98277_at	Zfpn1a4	zinc finger protein, subfamily 1A, 4 (Eos)	1.008	0.9024	0.1539	0.7228	No	1.0543	0.185	0.0372	0.629	No	No
98278_at	Spry4	sprouty homolog 4 (Drosophila)	1.0061	0.887	0.2133	0.0752	No	1.1394	0.0189	0.333	0.0096	No	No
98279_at	Insrr	insulin receptor-related receptor	0.9655	0.4408	0.0027	0.1936	No	1.047	0.603	0.91	0.178	No	No
98280_at	Mmp16	matrix metallopeptidase 16	1.0029	0.9738	0.3664	0.404	No	0.9771	0.722	0.346	0.279	No	No
98281_at	AJ242955	hypothetical protein, P4(21)n gene	0.9812	0.6993	0.1673	0.4284	No	1.0075	0.988	0.384	0.602	No	No

98282_at	Icos	inducible T-cell co-stimulator	1.0793	0.1005	0.9797	0.0209	No	1.0725	0.0717	0.982	0.0086	No	No
98283_at	Xrn1	5'-3' exoribonuclease 1	1.0668	0.2574	0.0191	0.373	No	0.9953	0.942	0.518	0.371	No	No
98284_f_at	H2-T18	histocompatibility 2, T region locus 18	0.9896	0.6568	0.3365	0.1991	No	0.996	0.951	0.0054	0.244	No	No
98285_at	LOC677248	similar to zinc finger protein 146	0.942	0.493	0.4774	0.5157	No	0.9655	0.661	0.407	0.404	No	No
98286_at	Ly6g6d	lymphocyte antigen 6 complex, locus G6D	0.9392	0.3622	0.0796	0.1799	No	0.9718	0.133	0.0001	0.148	No	No
98287_at	Dpp6	dipeptidylpeptidase 6	0.9683	0.5667	0.0134	0.6923	No	0.9557	0.296	0.0084	0.43	No	No
98288_at	Klk4	kallikrein 4 (prostase, enamel matrix, prostate)	1.0282	0.6539	0.0454	0.5074	No	0.9957	0.952	0.183	0.27	No	No
98289_at	Elf4	E74-like factor 4 (ets domain transcription factor)	1.0297	0.5952	0.0389	0.3571	No	0.9247	0.0571	0.0014	0.845	No	No
98290_at	Plcg1	phospholipase C, gamma 1	0.961	0.6133	0.0872	0.9899	No	0.9224	0.165	0.644	0.988	No	No
98291_at	B3galt6	UDP-Gal:betaGal beta 1,3-galactosyltransferase, polypeptide 6	1.1061	0.0153	0.0747	0.4382	No	1.0792	0.0962	0.0063	0.871	No	No
98292_at	Gsbs	G substrate	0.9362	0.4507	0.9227	0.8627	No	0.9131	0.15	0.632	0.832	No	No
98293_g_at	Gsbs	G substrate	0.9818	0.4791	0.2226	0.2241	No	1.0006	0.992	0.254	0.565	No	No
98294_at	---	---	1.0859	0.3515	0.0308	0.481	No	0.9006	0.27	0.421	0.443	No	No
98295_at	Cyp2c39	cytochrome P450, family 2, subfamily c, polypeptide 39	1.0351	0.2837	0.0052	0.6209	No	0.9939	0.52	0.0006	0.013	No	No
98296_at	Tulp1	tubby like protein 1	1.0225	0.7534	0.0679	0.9808	No	0.9382	0.184	0.681	0.0516	No	No
98297_at	Lgr5	leucine rich repeat containing G protein coupled receptor 5	0.9869	0.6893	0.6017	0.0486	No	0.9329	0.201	0.0407	0.0736	No	No
98298_at	Dpysl2	dihydropyrimidinase-like 2	1.0356	0.4106	0.0175	0.4044	No	1.0273	0.398	0.0064	0.072	No	No
98299_s_at	Slfn3 /// Slfn4	schlafen 3 /// schlafen 4	0.8987	0.2234	0.0778	0.9176	No	1.0187	0.683	0.0491	0.124	No	No
98300_at	Cacna2d3	calcium channel, voltage-dependent, alpha2/delta subunit 3	1.0172	0.3898	0.0243	0.0396	No	0.9604	0.328	0.119	0.19	No	No
98301_at	Tectb	tectorin beta	1.3983	0.0007	0.0006	0.2202	Yes	1.4073	0.0004	0.0002	0.298	Yes	Yes
98302_at	Scn10a	sodium channel, voltage-gated, type X, alpha	0.9625	0.5268	0.0029	0.4218	No	1.0723	0.176	0.0016	0.0998	No	No
98303_at	Cep110	centrosomal protein 110	0.9711	0.5221	0.9717	0.4143	No	1.0484	0.0557	0.0048	0.814	No	No
98304_at	Tlr6	toll-like receptor 6	1.0999	0.0396	0.0687	0.3928	No	1.1557	0.0295	0.0619	0.0516	No	No
98305_at	Foxm1	forkhead box M1	0.9413	0.0994	0.0274	0.8442	No	0.9624	0.0485	0.114	0.0043	No	No
98306_g_at	Foxm1	forkhead box M1	1.0373	0.1328	0.7026	0.0814	No	1.0092	0.398	0.568	0.317	No	No
98307_at	Rasgrp1	RAS guanyl releasing protein 1	0.9938	0.9511	0.1008	0.9077	No	0.9557	0.489	0.73	0.95	No	No
98308_at	Myh1	myosin, heavy polypeptide 1, skeletal muscle, adult	0.5361	0.0042	0.3761	0.0051	No	0.4855	0.0018	0.22	0.0031	No	No
98309_at	Ccbp2	chemokine binding protein 2	0.9357	0.158	0.6209	0.7565	No	0.968	0.627	0.165	0.558	No	No
98310_at	Znrf4	zinc and ring finger 4	0.9438	0.4324	0.0363	0.6395	No	1.01	0.839	0.0067	0.625	No	No
98311_at	Pmfbp1	polyamine modulated factor 1 binding protein 1	1.0254	0.2216	0.0007	0.1187	No	0.999	0.916	0.0953	0.0531	No	No
98312_at	Rspo1	R-spondin homolog (Xenopus laevis)	0.987	0.833	0.3451	0.3709	No	1.0372	0.478	0.0973	0.187	No	No
98313_at	Odz4	odd Oz/ten-m homolog 4 (Drosophila)	0.9434	0.2449	0.0422	0.1519	No	0.9756	0.614	0.0506	0.876	No	No
98314_g_at	Odz4	odd Oz/ten-m homolog 4 (Drosophila)	0.9913	0.8036	0.0054	0.4756	No	1.0067	0.98	0.0022	0.247	No	No
98315_at	Tecta	tectorin alpha	1.0979	0.0261	0.0004	0.659	No	1.1299	0.0137	0.0003	0.683	No	No
98316_at	Chic1	cysteine-rich hydrophobic domain 1	1.0047	0.9403	0.172	0.2261	No	0.977	0.418	0.0033	0.0665	No	No
98317_at	Phox2b	paired-like homeobox 2b	0.9382	0.1729	0.0875	0.7429	No	0.9542	0.257	0.032	0.0271	No	No
98318_at	Tnfsf7	tumor necrosis factor (ligand) superfamily, member 7	1.0245	0.3802	0.1416	0.1776	No	1.0181	0.737	0.0265	0.241	No	No
98319_at	Dsg2	desmoglein 2	0.9407	0.3994	0.1668	0.6273	No	0.9333	0.368	0.0381	0.552	No	No
98320_at	Cyp26a1	cytochrome P450, family 26, subfamily a, polypeptide 1	1.0052	0.9136	0.2467	0.7098	No	1.0333	0.432	0.298	0.568	No	No
98321_at	Olfr90	olfactory receptor 90	1.0778	0.0594	0.038	0.0503	No	0.9456	0.307	0.0064	0.943	No	No
98322_at	Slc22a5	solute carrier family 22 (organic cation transporter), member 5	1.0061	0.7823	0.0266	0.9867	No	1.0712	0.0767	0.174	0.0143	No	No
98323_at	Ccr9	chemokine (C-C motif) receptor 9	0.9929	0.8607	0.67	0.3688	No	1.1504	0.0769	0.143	0.0377	No	No
98324_at	Foxa3	forkhead box A3	1.1254	0.0683	0.0746	0.2332	No	0.9976	0.86	0.0767	0.031	No	No
98325_at	AA517545	expressed sequence AA517545	0.9757	0.3238	0.0135	0.0367	No	1.0333	0.621	0.0495	0.581	No	No
98326_f_at	Ccdc96	coiled-coil domain containing 96	1.0621	0.4859	0.3376	0.2736	No	1.0341	0.553	0.0189	0.142	No	No
98327_at	Usp45	Ubiquitin specific petidase 45	0.9601	0.5243	0.1508	0.2619	No	1.0179	0.901	0.642	0.369	No	No
98328_at	Tshr	thyroid stimulating hormone receptor	0.8927	0.1711	0.1567	0.6184	No	0.9272	0.271	0.0329	0.71	No	No
98329_at	Pfkfb2	6-phosphofructo-2-kinase/fructose-2,6-biphosphatase 2	0.9035	0.1344	0.247	0.4885	No	1.0726	0.281	0.591	0.0368	No	No
98330_at	Zic3	zinc finger protein of the cerebellum 3	0.9263	0.1804	0.027	0.6214	No	1.0249	0.257	0.0093	0.207	No	No
98331_at	Col3a1	procollagen, type III, alpha 1	1.0396	0.5337	0.5554	0.0659	No	0.945	0.0777	0.299	0.325	No	No
98332_at	Kcnj9	potassium inwardly-rectifying channel, subfamily J, member 9	0.9693	0.6132	0.1224	0.521	No	0.97	0.757	0.533	0.231	No	No
98333_at	Rps18	ribosomal protein S18	1.0343	0.2013	0.0006	0.1056	No	1.0204	0.173	0.0002	0.0281	No	No
98334_at	Crtam	cytotoxic and regulatory T cell molecule	0.9992	0.9883	0.0301	0.8861	No	0.9854	0.781	0.014	0.798	No	No
98335_at	Recc1	replication factor C 1	1.0249	0.7545	0.6542	0.8207	No	1.0243	0.686	0.0777	0.742	No	No
98336_s_at	Recc1	replication factor C 1	0.9288	0.2165	0.5832	0.8727	No	0.9272	0.279	0.598	0.985	No	No
98337_at	Nkx1-2	NK1 transcription factor related, locus 2 (Drosophila)	1.0392	0.4749	0.2029	0.8955	No	1.0219	0.731	0.232	0.753	No	No
98338_at	En2	engrailed 2	1.0319	0.7303	0.4782	0.1783	No	1.0826	0.18	0.27	0.0494	No	No
98339_at	Syt11	synaptotagmin XI	0.9923	0.8694	0.1084	0.5969	No	0.9957	0.9	0.802	0.38	No	No
98340_at	AA407331	expressed sequence AA407331	1.1199	0.2223	0.0338	0.2364	No	1.0842	0.231	0.0062	0.0696	No	No
98341_f_at	Mybbp1a	MYB binding protein (P160) 1a	1.0157	0.8409	0.0259	0.5653	No	0.9731	0.605	0	0.0056	No	No
98342_at	Rpl10	ribosomal protein 10	1.0179	0.6007	0.2131	0.4502	No	0.9859	0.57	0.0991	0.102	No	No
98343_s_at	Zfy2	zinc finger protein 2, Y linked	1.0378	0.2053	0.4956	0.5398	No	0.8945	0.925	0.785	0.176	No	No
98344_f_at	LOC245297 /// LOC668662	similar to RIKEN cDNA 6030426L16 /// similar to gonadotropin inducible ovarian transcription factor 1	0.9882	0.7205	0.006	0.411	No	0.923	0.0182	0.0874	0.0274	No	No
98345_at	AA536748	expressed sequence AA536748	1.0076	0.7416	0.0094	0.2973	No	1.073	0.303	0.751	0.0241	No	No
98346_at	Lrrc54	leucine rich repeat containing 54	0.8534	0.0626	0.0243	0.5636	No	0.8797	0.154	0.0142	0.293	No	No
98347_at	Cdx4	caudal type homeo box 4	1.0125	0.8334	0.938	0.1809	No	1.0655	0.278	0.005	0.354	No	No
98348_at	Fzd3	frizzled homolog 3 (Drosophila)	0.9884	0.816	0.0403	0.8318	No	0.9819	0.357	0.0053	0.465	No	No
98349_at	Il6st	interleukin 6 signal transducer	0.8432	0.0242	0.0064	0.284	No	0.9828	0.775	0.0173	0.511	No	No
98350_at	Sstr2	somatostatin receptor 2	1.0025	0.8523	0.0649	0.7972	No	0.9905	0.977	0.0043	0.298	No	No
98351_g_at	Sstr2	somatostatin receptor 2	1.1139	0.0614	0.0685	0.0136	No	1.1323	0.0415	0.0995	0.0071	No	No
98352_r_at	Sstr2	somatostatin receptor 2	0.9113	0.2166	0.0086	0.4166	No	0.9006	0.124	0.0145	0.192	No	No
98353_at	Cyp4a10 /// BC013476	cytochrome P450, family 4, subfamily a, polypeptide 10 /// cDNA sequence BC013476	1.052	0.1926	0.0018	0.0107	No	0.9485	0.407	0.361	0.524	No	No
98354_at	Ptcra	pre T-cell antigen receptor alpha	1.0486	0.0061	0.0162	0.0117	Yes	1.029	0.473	0.95	0.547	No	No
98355_at	Gpr133 /// LOC669205	G protein-coupled receptor 133 /// similar to G protein-coupled receptor 133	0.9371	0.0508	0.0008	0.0073	No	1.0262	0.593	0.983	0.439	No	No
98356_at	D16Wsu65e	DNA segment, Chr 16, Wayne State University 65, expressed	0.9783	0.6227	0.0242	0.6547	No	1.0224	0.72	0.0015	0.0737	No	No
98357_at	AA545190	EST AA545190	0.978	0.397	0.82	0.2769	No	1.003	0.889	0.138	0.13	No	No
98358_at	4933416E03 Rik	RIKEN cDNA 4933416E03 gene	1.0583	0.5852	0.069	0.6897	No	1.1525	0.0567	0.0021	0.484	No	No
98360_at	Cacna1b	calcium channel, voltage-dependent, N type, alpha 1B subunit	0.9961	0.9297	0.4517	0.9939	No	1.0085	0.482	0.002	0.0311	No	No
98361_at	Adcyap1r1	adenylate cyclase activating polypeptide 1 receptor 1	1.1199	0.0382	0.055	0.1672	No	1.0646	0.459	0.243	0.301	No	No
98362_s_at	Adcyap1r1	adenylate cyclase activating polypeptide 1 receptor 1	0.9173	0.2017	0.0851	0.9476	No	0.9285	0.13	0.0034	0.719	No	No
98363_at	Nxph2	neurexophilin 2	0.9809	0.767	0.6692	0.2175	No	0.9749	0.957	0.0538	0.192	No	No
98364_at	Kcnd2	potassium voltage-gated channel, Shal-related family, member 2	0.9775	0.7667	0.1933	0.5342	No	0.8356	0.195	0.0281	0.581	No	No
98365_at	Nos1	nitric oxide synthase 1, neuronal	1.0151	0.7722	0.0279	0.7367	No	0.9677	0.403	0.14	0.299	No	No
98366_at	Itgav	integrin alpha V	0.8876	0.0106	0.3694	0.385	No	0.9125	0.0423	0.0779	0.874	No	No
98367_at	Hoxd3	homeo box D3	0.999	0.9834	0.4658	0.588	No	1.0381	0.7	0.199	0.901	No	No
98368_at	Prkg2	protein kinase, cGMP-dependent, type II	1.0022	0.9572	0.0075	0.4595	No	0.8511	0.0195	0.12	0.177	No	No
98369_f_at	2610028J07 Rik	RIKEN cDNA 2610028J07 gene	0.9183	0.0119	0.0078	0.1972	No	0.9275	0.0867	0.0013	0.987	No	No
98370_at	Upk2	uroplakin 2	0.9672	0.6972	0.3308	0.7722	No	0.9781	0.715	0.22	0.5	No	No
98371_at	Cbfa2t2h	core-binding factor, runt domain, alpha subunit 2, translocated to, 2 homolog (human)	1.0509	0.4847	0.4781	0.7379	No	0.9307	0.448	0.984	0.773	No	No
98372_at	Aldh1a3	aldehyde dehydrogenase family 1, subfamily A3	1.0258	0.2725	0.0132	0.0328	No	1.0583	0.651	0.937	0.629	No	No
98373_at	Ms4a4c	membrane-spanning 4-domains, subfamily A, member 4C	1.0742	0.3153	0.2421	0.0754	No	1.2325	0.2	0.334	0.0852	No	No
98374_at	Il5	interleukin 5	0.9694	0.6513	0.1481	0.6129	No	0.882	0.184	0.14	0.0764	No	No
98375_at	Vipr2	vasoactive intestinal peptide receptor 2	0.9501	0.4365	0.621	0.6369	No	1.0565	0.259	0.0029	0.299	No	No
98376_at	Epha5	Eph receptor A5	1.0777	0.3061	0.6795	0.5428	No	0.9878	0.927	0.811	0.21	No	No
98379_r_at	Rfwd2 /// LOC621205	ring finger and WD repeat domain 2 /// similar to constitutive photomorphogenic protein 1	0.955	0.1835	0.0367	0.9414	No	0.9821	0.615	0.0397	0.875	No	No
98380_at	Kcnh1	potassium voltage-gated channel, subfamily H (eag-related), member 1	0.9522	0.4624	0.1313	0.7175	No	0.9652	0.658	0.662	0.559	No	No
98381_at	Nat12	N-acetyltransferase 12	0.9865	0.8569	0.2008	0.3819	No	1.0852	0.206	0.0176	0.343	No	No

98382_f_at	Prpmp5 /// MP4 /// MGC78233 /// BC061494 /// LOC674323	proline-rich protein MP5 /// proline rich protein MP4 /// similar to proline rich protein MP4 /// cDNA sequence BC061494 /// similar to proline rich protein MP4	0.9213	0.2565	0.1289	0.4859	No	0.9605	0.411	0.0438	0.407	No	No
98383_r_at	Prpmp5	proline-rich protein MP5	1.0052	0.731	0.0004	0.001	No	0.9718	0.436	0.0014	0.723	No	No
98384_at	Ptk6	PTK6 protein tyrosine kinase 6	0.9885	0.8347	0.3376	0.0677	No	0.9917	0.995	0.828	0.56	No	No
98385_at	Ptpn14	protein tyrosine phosphatase, non-receptor type 14	1.0079	0.8889	0.5655	0.3536	No	1.0063	0.967	0.0071	0.0704	No	No
98386_s_at	Cacna1c	calcium channel, voltage-dependent, L type, alpha 1C subunit	1.0699	0.191	0.4054	0.0772	No	1.0467	0.231	0.175	0.0964	No	No
98387_at	Bdkrb2	bradykinin receptor, beta 2	1.0441	0.3188	0.0058	0.7466	No	1.0585	0.31	0.134	0.801	No	No
98388_at	Mtrf1l	Mitochondrial translational release factor 1-like	1.1019	0.2355	0.5315	0.0575	No	1.0454	0.509	0.0635	0.289	No	No
98389_at	Crlf1	Cytokine receptor-like factor 1	1.0542	0.2913	0.0156	0.7517	No	1.0324	0.445	0.0366	0.156	No	No
98390_at	Impdh1	inosine 5'-phosphate dehydrogenase 1	0.9959	0.8622	0.0006	0.3358	No	0.9881	0.796	0.0318	0.356	No	No
98391_at	Klk1b11	kallikrein 1-related peptidase b11	0.8457	0.1468	0.1095	0.3415	No	0.8511	0.25	0.235	0.813	No	No
98392_at	Lag3	lymphocyte-activation gene 3	0.9358	0.0713	0.4574	0.1556	No	1.0549	0.307	0.535	0.461	No	No
98394_at	Dlx1	distal-less homeobox 1	0.8723	0.0676	0.7999	0.7088	No	0.9471	0.357	0.0049	0.574	No	No
98395_at	Crhr2	corticotropin releasing hormone receptor 2	0.9589	0.4726	0.1843	0.1876	No	0.8875	0.0165	0.0051	0.0679	No	No
98397_at	Blzf1	basic leucine zipper nuclear factor 1	1.0004	0.9942	0.0083	0.1977	No	0.8866	0.0956	0.0001	0.0071	No	No
98398_s_at	Apobec1	apolipoprotein B editing complex 1	1.0797	0.032	0.0325	0.2079	No	1.0865	0.0736	0.0215	0.178	No	No
98400_at	Solt	SoxLZ/Sox6 leucine zipper binding protein in testis	1.0894	0.2195	0.5877	0.1911	No	1.0496	0.4	0.0161	0.9	No	No
98401_at	Hsd3b3	hydroxysteroid dehydrogenase-3, delta<5>-3-beta	0.9667	0.2601	0.0362	0.856	No	0.9528	0.555	0.851	0.756	No	No
98402_at	Macf1	microtubule-actin crosslinking factor 1	1.0018	0.9425	0.002	0.1775	No	0.9404	0.233	0.0002	0.894	No	No
98403_at	Gna-rs1	guanine nucleotide binding protein, related sequence 1	1.0171	0.4542	0.0035	0.0038	No	0.9843	0.792	0.0549	0.853	No	No
98404_at	U2af2	U2 small nuclear ribonucleoprotein auxiliary factor (U2AF) 2	1.0089	0.6973	0.0088	0.0974	No	1.0165	0.0309	0.0001	0.0046	No	No
98405_at	Serpinb9	serine (or cysteine) peptidase inhibitor, clade B, member 9	0.9424	0.0108	0.0022	0.9881	No	0.9754	0.401	0.166	0.999	No	No
98406_at	Ccl5	chemokine (C-C motif) ligand 5	0.9828	0.7819	0.0524	0.9519	No	0.9102	0.0517	0.0086	0.154	No	No
98407_at	Efnb1	ephrin B1	1.1281	0.1243	0.2406	0.7509	No	1.1369	0.0351	0.0112	0.475	No	No
98408_at	Hhex	hematopoietically expressed homeobox	1.0079	0.9309	0.0357	0.5196	No	0.9503	0.151	0.43	0.0265	No	No
98409_at	Myo1b	myosin IB	1.0356	0.5827	0.0163	0.915	No	0.9993	0.793	0.0003	0.0124	No	No
98410_at	Iigp2	interferon inducible GTPase 2	0.9931	0.839	0.0146	0.9407	No	0.9292	0.123	0.0918	0.724	No	No
98413_at	Pdlim7	PDZ and LIM domain 7	0.899	0.1714	0.0671	0.182	No	1.0827	0.0983	0.002	0.793	No	No
98414_at	Zfp42	zinc finger protein 42	0.977	0.6757	0.0043	0.2234	No	0.9261	0.103	0.0001	0.306	No	No
98415_at	Crlf3	cytokine receptor-like factor 3	0.9227	0.083	0.0025	0.1605	No	0.9667	0.419	0.0411	0.217	No	No
98416_at	Traf2	Tnf receptor-associated factor 2	1.0368	0.3527	0.0404	0.3033	No	0.9721	0.0742	0.39	0.0004	No	No
98417_at	Mx1	myxovirus (influenza virus) resistance 1	1.0186	0.2062	0.0016	0.0261	No	1.0563	0.453	0.827	0.233	No	No
98418_at	Dvl1	dishevelled, dsh homolog 1 (Drosophila)	0.9773	0.5922	0.0323	0.2212	No	1.0045	0.908	0.0033	0.994	No	No
98419_at	Meox1	mesenchyme homeobox 1	0.9322	0.335	0.4663	0.6724	No	0.9182	0.339	0.143	0.663	No	No
98420_at	Impa2	inositol (myo)-1(or 4)-monophosphatase 2	0.9571	0.676	0.4405	0.4396	No	1.0164	0.633	0.0021	0.267	No	No
98421_at	Il17d	Interleukin 17D	1.0124	0.7112	0.0063	0.1201	No	1.0313	0.49	0.0033	0.154	No	No
98423_at	Gjb2	gap junction membrane channel protein beta 2	1.2198	0.0034	0.0003	0.0148	Yes	1.1924	0.0056	0.0011	0.0155	Yes	Yes
98424_at	Ptpn13	protein tyrosine phosphatase, non-receptor type 13	1.029	0.5359	0.0024	0.9401	No	1.0709	0.307	0.0012	0.836	No	No
98426_at	Ppt2	palmitoyl-protein thioesterase 2	1.0227	0.4771	0.0359	0.595	No	0.9563	0.326	0.0091	0.17	No	No
98427_s_at	Nfkb1	nuclear factor of kappa light chain gene enhancer in B-cells 1, p105	0.9886	0.7286	0.003	0.1355	No	0.9976	0.874	0.006	0.406	No	No
98428_at	Spast	spastin	0.9594	0.4573	0.1942	0.1622	No	0.9711	0.559	0.139	0.987	No	No
98429_at	Lypla2	lysophospholipase 2	1.0237	0.5932	0.1938	0.6824	No	1.0715	0.0086	0.0041	0.667	Yes	No
98430_at	Surf5	surfeit gene 5	0.9833	0.5449	0.0015	0.0937	No	1.0402	0.233	0.0227	0.514	No	No
98431_at	Dusp12	dual specificity phosphatase 12	1.1028	0.0343	0.774	0.7259	No	1.0691	0.0933	0.793	0.269	No	No
98432_at	X99384	cDNA sequence X99384	1.0294	0.1692	0.0702	0.007	No	1.0772	0.19	0.295	0.0483	No	No
98433_at	Bid	BH3 interacting domain death agonist	0.9541	0.1004	0.1508	0.3908	No	1.1546	0.0154	0.0527	0.0124	No	No
98434_at	Arhgef7	Rho guanine nucleotide exchange factor (GEF7)	1.0537	0.1666	0.2497	0.4417	No	1.0578	0.311	0.0089	0.353	No	No
98435_at	Adssl1	adenylosuccinate synthetase like 1	0.8089	0.0024	0.0372	0.0014	No	0.7712	0.0006	0.0064	0.0008	No	No
98436_s_at	Casp3	caspase 3	1.0475	0.6183	0.0655	0.3422	No	0.9452	0.0491	0.43	0.344	No	No
98437_at	Casp3	caspase 3	1.0135	0.8149	0.8423	0.5221	No	0.9931	0.418	0.0008	0.0031	No	No
98438_f_at	H2-Q7 /// H2-Q8 /// LOC630509 /// LOC674192	histocompatibility 2, Q region locus 7 /// histocompatibility 2, Q region locus 8 /// similar to H-2 class I histocompatibility antigen, Q7 alpha chain precursor (QA-2 antigen) /// region containing histocompatibility 2, Q region locus 9; histocompatibility 2, Q region locus 7	0.9701	0.5105	0.2977	0.2356	No	0.9863	0.643	0.0041	0.0403	No	No
98439_at	Rps6kb1	ribosomal protein S6 kinase, polypeptide 1	1.0477	0.3852	0.189	0.8634	No	1.0186	0.792	0.0025	0.488	No	No
98440_at	Ltb4dh	leukotriene B4 12-hydroxydehydrogenase	0.9882	0.8728	0.9104	0.3976	No	1.0184	0.665	0.0286	0.518	No	No
98441_at	Fmr1	fragile X mental retardation syndrome 1 homolog	0.9693	0.6828	0.0012	0.3862	No	0.9766	0.855	0.0008	0.501	No	No
98443_at	Lep	leptin	0.9792	0.7771	0.5409	0.2388	No	1.0083	0.921	0.239	0.0374	No	No
98444_g_at	Lep	leptin	0.9575	0.1584	0.0256	0.3021	No	0.967	0.334	0.0598	0.617	No	No
98445_at	Gga1	golgi associated, gamma adaptin ear containing, ARF binding protein 1	0.9282	0.0036	0.0012	0.0328	Yes	0.9459	0.117	0.103	0.365	No	No
98446_s_at	Cct3	chaperonin subunit 3 (gamma)	1.0413	0.4083	0.2942	0.1562	No	1.104	0.0745	0.0111	0.523	No	No
98447_at	Cebpa	CCAAT/enhancer binding protein (C/EBP), alpha	1.1078	0.0948	0.6619	0.2609	No	1.0395	0.389	0.388	0.193	No	No
98448_at	Casc3	cancer susceptibility candidate 3	0.9628	0.4673	0.0416	0.2372	No	1.0514	0.339	0.0184	0.869	No	No
98449_at	Sart1	squamous cell carcinoma antigen recognized by T-cells 1	1.0438	0.5447	0.1618	0.6962	No	0.947	0.274	0.002	0.29	No	No
98451_at	Dnajb10	DnaJ (Hsp40) homolog, subfamily B, member 10	0.9944	0.9146	0.0452	0.0525	No	1.0101	0.998	0.0774	0.106	No	No
98452_at	Flt1	FMS-like tyrosine kinase 1	1.0715	0.0127	0.0004	0.0072	No	1.0791	0.179	0.0079	0.507	No	No
98453_at	Flt1	FMS-like tyrosine kinase 1	0.9901	0.8471	0.4316	0.5085	No	0.9751	0.675	0.99	0.744	No	No
98454_at	Palm	paralemmin	0.9618	0.0653	0.0008	0.0777	No	1.0106	0.66	0.0229	0.358	No	No
98455_at	Atf2	activating transcription factor 2	1.0251	0.6603	0.0012	0.1344	No	0.9829	0.354	0.0001	0.126	No	No
98456_at	Stk19	serine/threonine kinase 19	0.9301	0.0698	0.2703	0.2906	No	0.9454	0.179	0.298	0.479	No	No
98457_at	Slc4a4	solute carrier family 4 (anion exchanger), member 4	0.8733	0.0491	0.0056	0.0851	No	0.9425	0.184	0.0019	0.121	No	No
98458_at	1190007F08Rik	RIKEN cDNA 1190007F08 gene	1.0178	0.7302	0.024	0.6332	No	1.0892	0.15	0.532	0.527	No	No
98459_at	Shmt1	serine hydroxymethyl transferase 1 (soluble)	0.9769	0.5156	0.0523	0.4029	No	1.0124	0.825	0.383	0.866	No	No
98460_at	Fto	fatso	0.9628	0.3658	0.0097	0.7957	No	0.9797	0.434	0.308	0.384	No	No
98461_at	Klc4	kinesin light chain 4	1.0167	0.4406	0.0016	0.3431	No	1.0297	0.817	0.0321	0.545	No	No
98462_s_at	Klc4	kinesin light chain 4	1.0027	0.951	0.0012	0.1571	No	0.9988	0.963	0.0035	0.401	No	No
98463_at	Smarca4	SWI/SNF related, matrix associated, actin dependent regulator of chromatin, subfamily a, member 4	1.0243	0.611	0.2376	0.4507	No	1.0417	0.53	0.906	0.988	No	No
98464_at	1110011C06Rik	RIKEN cDNA 1110011C06 gene	0.9216	0.3429	0.003	0.2739	No	0.9624	0.204	0.0009	0.0637	No	No
98465_f_at	Ifi204 /// LOC672547	interferon activated gene 204 /// similar to Interferon-activatable protein 204 (Ifi-204) (Interferon-inducible protein p204)	0.7907	0.0175	0.9357	0.1373	No	0.9362	0.269	0.0001	0.538	No	No
98466_r_at	LOC672547	similar to Interferon-activatable protein 204 (Ifi-204) (Interferon-inducible protein p204)	1.0263	0.4071	0.0803	0.7218	No	0.534	0.0055	0.0005	0.0319	Yes	No
98467_at	Itih4	inter alpha-trypsin inhibitor, heavy chain 4	0.9892	0.8501	0.0449	0.4251	No	1.0785	0.397	0.251	0.195	No	No
98468_r_at	Brd1	bromodomain containing 1	1.0157	0.876	0.0121	0.6048	No	1.0099	0.926	0.0044	0.897	No	No
98469_at	Aurkb	aurora kinase B	1.1226	0.0065	0.8934	0.0804	Yes	1.056	0.312	0.0053	0.116	No	No
98470_at	Slc25a14	solute carrier family 25 (mitochondrial carrier, brain), member 14	1.0514	0.4536	0.7927	0.2641	No	0.9388	0.658	0.12	0.0493	No	No
98471_f_at	Kifc1	kinesin family member C1	0.986	0.6407	0.0784	0.1961	No	1.0229	0.326	0.0075	0.129	No	No
98472_at	H2-T23 /// C920025E04Rik	histocompatibility 2, T region locus 23 /// RIKEN cDNA C920025E04 gene	0.8986	0.02	0.0147	0.004	No	0.9129	0.0925	0.0338	0.057	No	No
98473_at	Arg2	arginase type II	1.0281	0.5299	0.9537	0.3894	No	1.1119	0.585	0.351	0.328	No	No
98474_r_at	Tnfaip6	tumor necrosis factor alpha induced protein 6	1.0584	0.5084	0.0116	0.6721	No	1.1084	0.401	0.0704	0.564	No	No
98475_at	Matn2	matrilin 2	0.8584	0.147	0.1298	0.7519	No	0.8935	0.0438	0.0668	0.12	No	No
98476_at	2900054D09Rik	RIKEN cDNA 2900054D09 gene	0.9077	0.1487	0.0446	0.3276	No	0.932	0.0355	0.0006	0.596	No	No
98477_s_at	Ank3	ankyrin 3, epithelial	0.8488	0.0463	0.4232	0.1222	No	0.8738	0.003	0.0143	0.005	No	No
98478_at	Ccng2	cyclin G2	0.9838	0.5311	0.0009	0.3223	No	0.9735	0.49	0.0011	0.209	No	No
98479_f_at	Nr2c2	nuclear receptor subfamily 2, group C, member 2	1.0232	0.8152	0.4793	0.7711	No	1.0234	0.786	0.169	0.661	No	No
98480_s_at	Ren1 /// Ren2	renin 1 structural /// renin 2 tandem duplication of Ren1	0.982	0.8815	0.2406	0.4392	No	0.874	0.274	0.0312	0.439	No	No
98481_at	Masp2	mannan-binding lectin serine peptidase 2	1.0247	0.6157	0.0067	0.0911	No	0.991	0.698	0.0245	0.042	No	No
98482_at	Pthr1	parathyroid hormone receptor 1	1.0539	0.396	0.0002	0.3982	No	1.0399	0.3	0.0001	0.575	No	No

98483_at	Cacnb3	calcium channel, voltage-dependent, beta 3 subunit	1.0453	0.5396	0.0267	0.4467	No	1.1229	0.0075	0.0046	0.12	Yes	No
98484_at	Ica1	islet cell autoantigen 1	1.0148	0.6282	0.0124	0.6178	No	1.059	0.389	0.0514	0.499	No	No
98485_at	Rhpn2	rhophilin, Rho GTPase binding protein 2	1.0473	0.2547	0.0048	0.0196	No	1.0718	0.249	0.0015	0.594	No	No
98486_at	Stmn4	stathmin-like 4	1.0079	0.8544	0.7347	0.8738	No	0.95	0.272	0.426	0.921	No	No
98488_at	Myh4	myosin, heavy polypeptide 4, skeletal muscle	0.9143	0.1594	0.6029	0.4282	No	0.8727	0.229	0.305	0.75	No	No
98489_at	Dlg7	discs, large homolog 7 (Drosophila)	1.037	0.1162	0.1823	0.0724	No	1.0859	0.318	0.286	0.529	No	No
98490_at	Arl8b	ADP-ribosylation factor-like 8B	0.844	0.1733	0.0105	0.7269	No	0.8453	0.117	0.006	0.774	No	No
98491_at	Arl8b	ADP-ribosylation factor-like 8B	1.0579	0.2386	0.693	0.2347	No	1.0102	0.433	0.0014	0.0176	No	No
98492_at	Cmtm7	CKLF-like MARVEL transmembrane domain containing 7	1.029	0.1497	0.0002	0.0134	No	1.0113	0.904	0.28	0.239	No	No
98493_at	3200002M19 Rik	RIKEN cDNA 3200002M19 gene	0.979	0.7805	0.5231	0.6225	No	0.9759	0.663	0.228	0.639	No	No
98495_at	5033414D02 Rik	RIKEN cDNA 5033414D02 gene	1.039	0.0994	0.8879	0.0548	No	1.0321	0.447	0.382	0.743	No	No
98496_at	Gys1	glycogen synthase 1, muscle	0.8827	0.0142	0.0041	0.1073	No	0.8551	0.0013	0.0027	0.0028	No	No
98497_at	Eps15l1	epidermal growth factor receptor pathway substrate 15-like 1	0.922	0.0156	0.0025	0.3465	No	0.9676	0.156	0.0012	0.416	No	No
98498_at	Casp7	caspase 7	1.0052	0.7847	0.0409	0.5111	No	1.0129	0.287	0.0057	0.002	No	No
98499_s_at	Casp7	caspase 7	0.9957	0.9	0.1985	0.574	No	1.0444	0.136	0.0037	0.0306	No	No
98500_at	Il1rl1	interleukin 1 receptor-like 1	0.9146	0.3113	0.3658	0.5052	No	0.9621	0.794	0.0354	0.685	No	No
98501_at	Il1rl1	interleukin 1 receptor-like 1	1.0435	0.2388	0.8643	0.0629	No	1.0768	0.36	0.627	0.763	No	No
98502_at	Dusp19	dual specificity phosphatase 19	0.8937	0.0157	0.0042	0.6553	No	0.9394	0.0871	0.331	0.743	No	No
98503_at	Evi5	ecotropic viral integration site 5	1.0174	0.8424	0.002	0.6319	No	1.0788	0.0219	0	0.787	No	No
98504_at	Rock2	Rho-associated coiled-coil forming kinase 2	0.8572	0.1237	0.1861	0.9873	No	0.8566	0.263	0.0891	0.682	No	No
98505_i_at	Cpox	coproporphyrinogen oxidase	0.9341	0.1749	0.2207	0.6444	No	0.9368	0.227	0.0052	0.982	No	No
98506_r_at	Cpox	coproporphyrinogen oxidase	0.976	0.6981	0.0183	0.8471	No	0.9344	0.334	0.0448	0.488	No	No
98507_at	Nr1d1	nuclear receptor subfamily 1, group D, member 1	0.9898	0.6372	0.0052	0.8012	No	1.061	0.334	0.226	0.209	No	No
98508_s_at	Ppap2a	phosphatidic acid phosphatase 2a	0.897	0.0969	0.8181	0.2359	No	0.9134	0.0797	0.278	0.244	No	No
98509_at	BC002199	cDNA sequence BC002199	1.0844	0.1637	0.0058	0.3139	No	1.0559	0.115	0.0036	0.199	No	No
98511_at	Raly	hnRNP-associated with lethal yellow	0.9828	0.7378	0.0059	0.4201	No	1.0026	0.75	0.0053	0.0827	No	No
98512_at	Banp	Btg3 associated nuclear protein	0.985	0.7308	0.4334	0.2992	No	0.9639	0.236	0.002	0.97	No	No
98513_at	Gbl	G protein beta subunit-like	1.0557	0.3349	0.4088	0.547	No	0.9994	0.911	0.168	0.128	No	No
98514_at	Tfpi	tissue factor pathway inhibitor	1.04	0.5892	0.0735	0.5355	No	1.0631	0.292	0.201	0.263	No	No
98515_at	Gtf2e2	general transcription factor II E, polypeptide 2 (beta subunit)	0.9963	0.9645	0.4432	0.6973	No	1.0884	0.0724	0.117	0.012	No	No
98516_at	BC056474	cDNA sequence BC056474	1.1222	0.0056	0.0001	0.0039	No	1.0919	0.0511	0.0004	0.0304	No	No
98518_f_at	2300002G24 Rik	RIKEN cDNA 2300002G24 gene	1.0886	0.0962	0.0058	0.7528	No	1.1302	0.153	0.0353	0.755	No	No
98519_r_at	2300002G24 Rik	RIKEN cDNA 2300002G24 gene	1.0793	0.107	0.7505	0.1252	No	1.0928	0.0393	0.0029	0.0008	No	No
98521_at	Vamp3	vesicle-associated membrane protein 3	1.1028	0.3974	0.3521	0.4295	No	1.1983	0.0892	0.544	0.448	No	No
98522_at	Psmd8	proteasome (prosome, macropain) 26S subunit, non-ATPase, 8	1.0322	0.6089	0.9683	0.2865	No	0.9923	0.924	0.0142	0.026	No	No
98523_at	Rps29	ribosomal protein S29	1.0458	0.3702	0.028	0.0433	No	0.9841	0.723	0.567	0.296	No	No
98524_f_at	Mtm1	X-linked myotubular myopathy gene 1	0.9348	0.3894	0.0007	0.031	No	0.9063	0.534	0.0009	0.0404	No	No
98525_f_at	Erdr1	erythroid differentiation regulator 1	1.0695	0.0732	0.0004	0.0561	No	1.1116	0.0427	0.0003	0.0439	No	No
98526_r_at	Erdr1	Erythroid differentiation regulator 1	1.0033	0.9206	0.6223	0.1355	No	0.9205	0.147	0.0921	0.0436	No	No
98527_at	Dci	dodecenoyl-Coenzyme A delta isomerase (3,2 trans-enoyl-Coenyme A isomerase)	1.0291	0.3575	0.0007	0.5165	No	1.0741	0.0995	0.0126	0.625	No	No
98528_at	---	---	1.0052	0.8209	0.0021	0.8946	No	1.0042	0.975	0.0487	0.572	No	No
98529_at	Dtprp	decidual/trophoblast prolactin-related protein	0.9752	0.75	0.0347	0.655	No	0.9587	0.819	0.0938	0.616	No	No
98530_at	Gas5	growth arrest specific 5	0.9311	0.3953	0.0196	0.6963	No	0.9508	0.713	0.118	0.221	No	No
98531_g_at	Gas5	growth arrest specific 5	0.9496	0.3427	0.0214	0.0693	No	0.9584	0.581	0.0341	0.114	No	No
98532_at	Cdk2ap1	CDK2 (cyclin-dependent kinase 2)-associated protein 1	1.2244	0.0035	0.0073	0.5041	Yes	1.2352	0.0032	0.0011	0.187	Yes	Yes
98533_at	Cyb5	cytochrome b-5	1.0095	0.8858	0.0055	0.3079	No	0.973	0.614	0.0152	0.212	No	No
98534_at	Sap18	Sin3-associated polypeptide 18	1.0384	0.5463	0.0731	0.0613	No	1.0273	0.457	0.0067	0.237	No	No
98535_at	Comt	catechol-O-methyltransferase	0.8848	0.1016	0.7208	0.9311	No	0.902	0.128	0.18	0.253	No	No
98538_at	2610507B11 Rik	RIKEN cDNA 2610507B11 gene	1.0014	0.9787	0.3497	0.3518	No	0.9819	0.628	0.0941	0.665	No	No
98539_at	Cops2	COP9 (constitutive photomorphogenic) homolog, subunit 2 (Arabidopsis thaliana)	1.0773	0.1861	0.0049	0.715	No	1.0498	0.464	0.0343	0.736	No	No
98540_g_at	Cops2	COP9 (constitutive photomorphogenic) homolog, subunit 2 (Arabidopsis thaliana)	1.0193	0.7596	0.0465	0.1015	No	0.9395	0.433	0.0259	0.161	No	No
98543_at	Ctss	cathepsin S	0.9088	0.0618	0.0007	0.4752	No	0.9476	0.0442	0.0001	0.139	No	No
98544_at	Guk1	guanylate kinase 1	1.0481	0.4568	0.116	0.6725	No	1.034	0.458	0.566	0.587	No	No
98545_at	Phb2	prohibitin 2	1.1086	0.0247	0.0023	0.0552	No	1.1009	0.0366	0.0023	0.152	No	No
98547_at	Mrps12	mitochondrial ribosomal protein S12	0.9993	0.9857	0.0189	0.5265	No	1.0152	0.647	0.0021	0.918	No	No
98549_at	Vtn	vitronectin	1.0142	0.5834	0.0042	0.257	No	1.0719	0.02	0.105	0.272	No	No
98550_at	Set	SET translocation	0.9267	0.0885	0.0018	0.0198	No	0.981	0.332	0.0009	0.0258	No	No
98552_at	Pmf1	polyamine-modulated factor 1	0.9585	0.1957	0.0003	0.4621	No	0.8778	0.0926	0.002	0.0186	No	No
98553_at	Slmap	sarcolemma associated protein	0.9321	0.4484	0.0114	0.6501	No	0.8973	0.102	0.0057	0.471	No	No
98554_at	Ndrg3	N-myc downstream regulated gene 3	1.023	0.6611	0.567	0.7722	No	1.0243	0.802	0.362	0.225	No	No
98555_at	Ttc3	tetratricopeptide repeat domain 3	1.0643	0.1492	0.0027	0.0549	No	1.0291	0.385	0.0095	0.0797	No	No
98556_at	Psmb4	proteasome (prosome, macropain) subunit, beta type 4	0.9675	0.4538	0.1552	0.5453	No	1.0608	0.415	0.168	0.269	No	No
98557_f_at	Psmb4	proteasome (prosome, macropain) subunit, beta type 4	1.0914	0.1889	0.4508	0.6615	No	1.0284	0.474	0.0098	0.13	No	No
98558_r_at	Psmb4	proteasome (prosome, macropain) subunit, beta type 4	1.0432	0.0183	0.019	0.2727	No	1.346	0.0026	0.0041	0.0038	No	No
98559_at	Smtn	smoothelin	0.9527	0.3567	0.0093	0.7258	No	0.9942	0.58	0.0004	0.0537	No	No
98560_at	Cltb	clathrin, light polypeptide (Lcb)	1.003	0.8865	0.019	0.0075	No	0.9559	0.251	0.0055	0.161	No	No
98561_at	Tnni1	troponin I, skeletal, slow 1	0.8782	0.0019	0.3309	0.0069	No	0.9231	0.193	0.785	0.353	No	No
98562_at	C1qa	complement component 1, q subcomponent, alpha polypeptide	0.8372	0.0093	0.0595	0.0196	Yes	0.826	0.0302	0.653	0.0269	No	Yes
98563_f_at	Mcrs1 /// LOC433780 /// LOC666215	microspherule protein 1 /// similar to Microspherule protein 1 (58 kDa microspherule protein) /// similar to Microspherule protein 1 (58 kDa microspherule protein)	0.9919	0.6994	0.0002	0.4461	No	1.0151	0.59	0.0004	0.654	No	No
98564_f_at	Rps26	ribosomal protein S26	1.0197	0.5913	0.2679	0.043	No	0.9901	0.551	0.044	0.0156	No	No
98565_at	Rps26 /// LOC626175 /// LOC664891 /// LOC676684	ribosomal protein S26 /// similar to 40S ribosomal protein S26 /// similar to 40S ribosomal protein S26 /// similar to 40S ribosomal protein S26	1.0721	0.1602	0.0121	0.0869	No	1.1891	0.0271	0.0075	0.215	No	No
98568_at	Csh2	chorionic somatomammotropin hormone 2	0.9947	0.9289	0.6321	0.6256	No	1.0404	0.648	0.23	0.721	No	No
98569_at	Slc25a25	solute carrier family 25 (mitochondrial carrier, phosphate carrier), member 25	1.0601	0.0919	0.2129	0.1518	No	1.1014	0.0462	0.804	0.0932	No	No
98570_at	Naca	nascent polypeptide-associated complex alpha polypeptide	1.073	0.284	0.1166	0.2795	No	1.2161	0.208	0.0529	0.745	No	No
98571_s_at	Naca /// LOC628277 /// LOC633817 /// LOC666761 /// LOC669373 /// LOC674918	nascent polypeptide-associated complex alpha polypeptide /// similar to nascent polypeptide-associated complex alpha polypeptide /// similar to nascent polypeptide-associated complex alpha polypeptide /// similar to nascent polypeptide-associated complex alpha polypeptide /// similar to nascent polypeptide-associated complex alpha polypeptide /// similar to nascent polypeptide-associated complex alpha polypeptide	1.0541	0.4438	0.0136	0.7062	No	0.9975	0.904	0.0181	0.0905	No	No
98572_at	Dnajb11	DnaJ (Hsp40) homolog, subfamily B, member 11	1.0251	0.6092	0.0296	0.3603	No	1.0481	0.197	0.032	0.718	No	No
98573_r_at	Ranbp1	RAN binding protein 1	1.1613	0.0731	0.2648	0.3195	No	1.1485	0.201	0.851	0.567	No	No
98574_at	Prpf8	pre-mRNA processing factor 8	1.0468	0.5114	0.3435	0.4114	No	1.0887	0.179	0.0701	0.908	No	No
98575_at	Fasn	fatty acid synthase	1.0906	0.1178	0.0171	0.5384	No	1.0484	0.425	0.0108	0.865	No	No
98577_f_at	---	---	1.0798	0.3917	0.0027	0.1206	No	0.9857	0.628	0.0021	0.0331	No	No
98578_at	Omt2b	oocyte maturation, beta	0.9752	0.6121	0.4239	0.9502	No	0.943	0.209	0.068	0.992	No	No
98579_at	Egr1	early growth response 1	0.9932	0.8623	0.0374	0.5751	No	1.0082	0.729	0.0509	0.142	No	No
98580_at	Ppm1a	protein phosphatase 1A, magnesium dependent, alpha isoform	1.0692	0.4157	0.0972	0.2186	No	1.1611	0.26	0.0725	0.641	No	No
98582_at	Hgd	homogentisate 1, 2-dioxygenase	1.0949	0.1334	0.0035	0.5824	No	0.9	0.0957	0.0129	0.0861	No	No

98583_at	Btg4	B-cell translocation gene 4	1.0352	0.5717	0.7892	0.1535	No	1.0212	0.789	0.189	0.301	No	No
98586_at	Nap1l1	nucleosome assembly protein 1-like 1	1.0095	0.9241	0.5453	0.8754	No	0.9975	0.937	0.0563	0.528	No	No
98587_at	Nap1l1	nucleosome assembly protein 1-like 1	0.979	0.7909	0.0305	0.5879	No	0.9508	0.31	0.0012	0.593	No	No
98588_at	Fah	fumarylacetoacetate hydrolase	0.9256	0.0823	0.0473	0.6595	No	0.8977	0.0301	0.0305	0.0129	No	No
98589_at	Adfp	adipose differentiation related protein	1.0533	0.289	0.0181	0.0479	No	1.0249	0.507	0.294	0.0462	No	No
98590_at	Sdc4	syndecan 4	0.9297	0.4954	0.0086	0.6432	No	1.0801	0.265	0.829	0.354	No	No
98593_at	Cmas	cytidine monophospho-N-acetylneuraminic acid synthetase	1.0628	0.1235	0.6156	0.0366	No	1.0596	0.04	0.549	0.08	No	No
98594_at	1190002N15 Rik	RIKEN cDNA 1190002N15 gene	0.9489	0.4687	0.382	0.3111	No	0.9557	0.536	0.0205	0.853	No	No
98595_at	Irak1	interleukin-1 receptor-associated kinase 1	1.0252	0.6414	0.0035	0.8709	No	1.0407	0.541	0.0082	0.362	No	No
98596_s_at	St3gal5	ST3 beta-galactoside alpha-2,3-sialyltransferase 5	0.9292	0.1384	0.0549	0.3739	No	1.0127	0.336	0	0.0804	No	No
98597_at	Ube2l6	ubiquitin-conjugating enzyme E2L 6	1.0575	0.1128	0.0147	0.8333	No	1.0107	0.781	0.0065	0.681	No	No
98598_at	C78891	expressed sequence C78891	1.0085	0.8794	0.0611	0.0911	No	0.9641	0.762	0.81	0.996	No	No
98599_at	Hdgfrp2	hepatoma-derived growth factor, related protein 2	0.9428	0.0133	0.0004	0.0026	No	1.0212	0.291	0.0166	0.08	No	No
98600_at	S100a11	S100 calcium binding protein A11 (calizzarin)	1.0628	0.2958	0.0143	0.1497	No	1.023	0.562	0.0064	0.378	No	No
98602_at	Rangap1	RAN GTPase activating protein 1	1.0594	0.2703	0.3379	0.3498	No	1.0288	0.345	0.0074	0.645	No	No
98603_s_at	Rangap1	RAN GTPase activating protein 1	1.0657	0.2754	0.0234	0.8097	No	1.1156	0.0437	0.0105	0.607	No	No
98604_at	Nnp1	novel nuclear protein 1	0.9719	0.672	0.6712	0.4834	No	1.0502	0.0439	0.0052	0.554	No	No
98605_at	Wars	tryptophanyl-tRNA synthetase	1.0046	0.8925	0.0371	0.4481	No	1.0295	0.552	0.359	0.231	No	No
98606_s_at	Wars	tryptophanyl-tRNA synthetase	1.0596	0.2892	0.1379	0.8545	No	1.1254	0.0673	0.143	0.0518	No	No
98608_at	Etf1	eukaryotic translation termination factor 1	1.0161	0.6403	0.7493	0.8867	No	1.0536	0.403	0.816	0.584	No	No
98609_at	38969	septin 9	1.0201	0.6574	0.036	0.6526	No	1.0335	0.488	0.0108	0.778	No	No
98610_at	Mrps28	mitochondrial ribosomal protein S28	1.0184	0.7664	0.0337	0.268	No	1.0552	0.417	0.115	0.517	No	No
98612_at	Cyp2d10	cytochrome P450, family 2, subfamily d, polypeptide 10	0.9382	0.3561	0.014	0.1806	No	0.9736	0.846	0.0228	0.209	No	No
98613_at	2700085E05 Rik	RIKEN cDNA 2700085E05 gene	1.0685	0.2656	0.0655	0.6194	No	1.0057	0.908	0.065	0.86	No	No
98614_at	Nphp1	nephronophthisis 1 (juvenile) homolog (human)	1.0521	0.0738	0.0354	0.2323	No	1.119	0.0005	0	0.0155	Yes	No
98615_at	0610041E09 Rik	RIKEN cDNA 0610041E09 gene	1.0787	0.3419	0.3306	0.8335	No	1.1883	0.0353	0.0777	0.857	No	No
98616_f_at	LOC671894	similar to myosin, heavy polypeptide 4, skeletal muscle	0.7538	0.0001	0.0071	0.0006	No	0.7258	0.004	0.265	0.0237	Yes	No
98617_at	Wbp11	WW domain binding protein 11	0.9882	0.7349	0.5568	0.0384	No	0.9971	0.968	0.133	0.184	No	No
98618_at	Dtymk	deoxythymidylate kinase	1.1264	0.1657	0.2056	0.7709	No	0.9686	0.342	0.0612	0.0205	No	No
98619_at	Dtymk	deoxythymidylate kinase	1.0669	0.3099	0.3156	0.4258	No	1.0327	0.675	0.778	0.178	No	No
98621_at	Gnptg	N-acetylglucosamine-1-phosphotransferase, gamma subunit	0.9391	0.4014	0.2513	0.4353	No	0.9449	0.33	0.462	0.445	No	No
98622_at	Igf2	insulin-like growth factor 2	1.0236	0.6724	0.0563	0.2303	No	1.0822	0.164	0.0232	0.787	No	No
98623_g_at	Igf2	insulin-like growth factor 2	0.9663	0.0583	0.3191	0.072	No	0.9761	0.0371	0.39	0.0808	No	No
98624_at	Rnpc1	RNA-binding region (RNP1, RRM) containing 1	0.9218	0.3887	0.3534	0.8049	No	0.9245	0.152	0.417	0.97	No	No
98625_s_at	Adh5 /// LOC635419	alcohol dehydrogenase 5 (class III), chi polypeptide /// similar to alcohol dehydrogenase 5 (class III), chi polypeptide	1.0396	0.5927	0.2569	0.873	No	1.0048	0.919	0.17	0.773	No	No
98626_at	1810017G16 Rik	RIKEN cDNA 1810017G16 gene	1.0015	0.9751	0.016	0.1263	No	0.9905	0.897	0.0232	0.64	No	No
98627_at	Igfbp2	insulin-like growth factor binding protein 2	1.079	0.0583	0.0509	0.9625	No	1.0448	0.165	0.428	0.898	No	No
98628_f_at	Hif1a	hypoxia inducible factor 1, alpha subunit	1.0015	0.976	0.0011	0.5376	No	0.9802	0.926	0.0026	0.351	No	No
98629_f_at	Hif1a	hypoxia inducible factor 1, alpha subunit	0.9606	0.3657	0.0004	0.8598	No	0.9651	0.69	0.0029	0.556	No	No
98630_at	Nsdhl	NAD(P) dependent steroid dehydrogenase-like	1.0447	0.4848	0.1067	0.4035	No	1.0284	0.558	0.0362	0.765	No	No
98631_g_at	Nsdhl	NAD(P) dependent steroid dehydrogenase-like	0.9785	0.6754	0.0111	0.442	No	1.0172	0.601	0.0051	0.498	No	No
98632_at	Ada	adenosine deaminase	0.9559	0.5723	0.1008	0.8411	No	0.9988	0.855	0.0138	0.172	No	No
98633_at	Tmem43	transmembrane protein 43	0.9245	0.0076	0.0003	0.0106	Yes	0.9654	0.296	0.005	0.365	No	No
98635_at	Ube2z	ubiquitin-conjugating enzyme E2Z (putative)	0.9469	0.1505	0.621	0.9756	No	0.9997	0.969	0.0079	0.42	No	No
98726_at	Pgr	progesterone receptor	0.9258	0.0405	0.6907	0.5133	No	0.9441	0.621	0.0436	0.518	No	No
98727_at	Gucy2e	guanylate cyclase 2e	1.0599	0.3487	0.0157	0.7922	No	1.0003	0.878	0.0033	0.42	No	No
98728_at	Tnfrsf8	tumor necrosis factor receptor superfamily, member 8	0.9605	0.3195	0.2576	0.0318	No	1.0663	0.0155	0.0009	0.0072	No	No
98729_at	Fnbp1	formin binding protein 1	1.0083	0.8636	0.1162	0.773	No	1.0164	0.436	0.0002	0.391	No	No
98730_at	Fgf17	fibroblast growth factor 17	1.0435	0.6117	0.0754	0.7662	No	1.0971	0.098	0.0121	0.472	No	No
98731_at	Rab5b	RAB5B, member RAS oncogene family	0.9799	0.1458	0.3426	0.1477	No	0.9992	0.999	0.162	0.31	No	No
98732_at	Elk4	ELK4, member of ETS oncogene family	0.9691	0.635	0.4365	0.9074	No	1.0355	0.236	0.193	0.102	No	No
98756_at	2810043O03 Rik	RIKEN cDNA 2810043O03 gene	1.1477	0.1981	0.2228	0.0813	No	1.1735	0.0007	0.0024	0.0004	No	No
98758_at	Alox15	arachidonate 15-lipoxygenase	1.0329	0.492	0.08	0.4908	No	1.0452	0.437	0.0462	0.767	No	No
98759_f_at	Tuba1 /// Tuba2 /// Tuba6	tubulin, alpha 1 /// tubulin, alpha 2 /// tubulin, alpha 6	1.0732	0.0278	0.7648	0.11	No	1.049	0.0861	0.779	0.283	No	No
98760_at	Zfhx4	zinc finger homeodomain 4	0.9751	0.6995	0.7416	0.2154	No	1.0724	0.367	0.418	0.7	No	No
98761_i_at	Zfp97 /// BC018101	zinc finger protein 97 /// cDNA sequence BC018101	1.1155	0.0861	0.0007	0.7549	No	1.043	0.373	0.0031	0.917	No	No
98762_f_at	Zfp97	zinc finger protein 97	1.0703	0.2136	0.1367	0.5832	No	1.1069	0.0576	0.163	0.459	No	No
98763_at	D0Kist4	DNA segment, KIST 4	0.9675	0.4739	0.0176	0.1731	No	0.9214	0.0455	0.0014	0.108	No	No
98764_at	D0Kist2	DNA segment, KIST 2	1.0159	0.5689	0.0744	0.1804	No	1.9603	0.198	0.494	0.0356	No	No
98765_f_at	LOC382653 /// LOC544903	immunoglobulin heavy chain variable region /// similar to immunoglobulin mu-chain	0.9927	0.8974	0.1037	0.6623	No	1.0406	0.285	0.0036	0.828	No	No
98766_at	Sh3bp5	SH3-domain binding protein 5 (BTK-associated)	0.9881	0.5558	0.168	0.8549	No	0.9224	0.0521	0.152	0.452	No	No
98767_at	Yy1	YY1 transcription factor	0.9714	0.5387	0.002	0.8751	No	0.9802	0.679	0.0093	0.78	No	No
98768_at	Ptger2	prostaglandin E receptor 2 (subtype EP2)	1.0422	0.2361	0.3373	0.1539	No	1.0727	0.347	0.16	0.522	No	No
98770_at	Cenpc1	centromere autoantigen C1	0.9454	0.1498	0.063	0.0322	No	0.9894	0.713	0.118	0.263	No	No
98771_at	Ephb2	Eph receptor B2	1.0056	0.8796	0.0049	0.3921	No	0.9962	0.989	0.0077	0.584	No	No
98772_at	Cxcl5	chemokine (C-X-C motif) ligand 5	1.1155	0.0074	0.3565	0.2747	Yes	1.35	0.0275	0.339	0.171	No	Yes
98773_s_at	Irg1	immunoresponsive gene 1	1.0545	0.3223	0.9288	0.1813	No	1.2798	0.0631	0.784	0.115	No	No
98774_at	Irg1	immunoresponsive gene 1	1.0104	0.7553	0.1262	0.1303	No	0.9673	0.201	0.0019	0.174	No	No
98775_at	Stk3	serine/threonine kinase 3 (Ste20, yeast homolog)	0.945	0.38	0.9516	0.3079	No	1.0194	0.851	0.61	0.538	No	No
98776_at	Igh-6	immunoglobulin heavy chain 6 (heavy chain of IgM)	0.8874	0.2529	0.2072	0.642	No	0.9919	0.921	0.814	0.908	No	No
98777_at	Vipr1	vasoactive intestinal peptide receptor 1	0.9938	0.9123	0.2642	0.0777	No	0.9271	0.0537	0.0592	0.5	No	No
98778_at	38971	septin 11	0.9687	0.2953	0.0265	0.4659	No	1.0059	0.91	0.0178	0.785	No	No
98779_at	Olfr91	olfactory receptor 91	1.0199	0.7656	0.198	0.222	No	1.0591	0.284	0.0996	0.62	No	No
98780_at	Hoxb3	homeo box B3	1.0329	0.6263	0.0241	0.952	No	1.045	0.349	0.109	0.0074	No	No
98781_at	Grpr	gastrin releasing peptide receptor	1.061	0.1899	0.3268	0.8101	No	1.0141	0.856	0.955	0.621	No	No
98782_at	Cplx2	complexin 2	1.1268	0.1696	0.0064	0.4092	No	1.1947	0.0077	0.0013	0.112	Yes	No
98783_at	Rbmy1a1	RNA binding motif protein, Y chromosome, family 1, member A1	0.9299	0.2492	0.0302	0.7702	No	1.02	0.804	0.0007	0.478	No	No
98784_at	Gpr12	G-protein coupled receptor 12	0.9988	0.9816	0.7961	0.6949	No	1.0002	0.923	0.0229	0.573	No	No
98785_at	---	Transcribed locus	0.9677	0.602	0.0541	0.9233	No	1.1377	0.0616	0.0826	0.0931	No	No
98786_at	Gpr3	G-protein coupled receptor 3	0.982	0.7098	0.3474	0.9043	No	1.046	0.432	0.0227	0.126	No	No
98787_at	Kcnj11	potassium inwardly rectifying channel, subfamily J, member 11	1.0126	0.7876	0.6328	0.6744	No	1.0279	0.363	0.246	0.887	No	No
98788_at	Pit1	pituitary specific transcription factor 1	0.8856	0.266	0.089	0.3513	No	0.8678	0.006	0.095	0.004	No	No
98789_at	Cdkn2a	cyclin-dependent kinase inhibitor 2A	1.0007	0.9897	0.0419	0.1769	No	0.8937	0.0712	0.91	0.168	No	No
98790_s_at	Meis1	myeloid ecotropic viral integration site 1	0.9915	0.9152	0.8737	0.4778	No	0.8727	0.109	0.652	0.446	No	No
98791_at	Rai1	retinoic acid induced 1	1.035	0.3519	0.4621	0.7593	No	1.0239	0.236	0.0262	0.144	No	No
98792_at	AW011956	expressed sequence AW011956	0.9999	0.9994	0.1051	0.9008	No	0.9768	0.788	0.227	0.461	No	No
98793_at	Gdf5	growth differentiation factor 5	1.0203	0.3604	0.0176	0.0657	No	0.9176	0.0534	0.846	0.765	No	No
98794_at	Srms	src-related kinase lacking C-terminal regulatory tyrosine and N-terminal myristylation sites	0.9208	0.1679	0.1475	0.3137	No	0.7316	0.0487	0.31	0.0674	No	No
98795_at	Thrb	thyroid hormone receptor beta	1.0108	0.9058	0.6042	0.391	No	0.9844	0.584	0.0554	0.167	No	No
98796_at	Mip	major intrinsic protein of eye lens fiber	1.0109	0.8799	0.158	0.1618	No	0.9227	0.546	0.0362	0.0688	No	No
98797_at	Gast	gastrin	0.9299	0.2638	0.2146	0.566	No	0.9385	0.0845	0.0193	0.0502	No	No
98798_at	Cspg3	chondroitin sulfate proteoglycan 3	1.0605	0.1746	0.0034	0.0758	No	1.0294	0.473	0.04	0.841	No	No
98799_g_at	Cspg3	chondroitin sulfate proteoglycan 3	1.0315	0.5377	0.5653	0.7542	No	1.0082	0.906	0.0405	0.99	No	No
98800_at	Slc23a3	solute carrier family 23 (nucleobase transporters), member 3	1.0243	0.6674	0.1858	0.4156	No	1.0361	0.618	0.0077	0.137	No	No
98801_at	Klra2	killer cell lectin-like receptor, subfamily A, member 2	0.9657	0.2042	0.2811	0.0791	No	1.1187	0.3	0.958	0.0144	No	No
98802_at	Ereg	epiregulin	1.0113	0.6135	0.2843	0.3612	No	0.9882	0.782	0.0532	0.01	No	No
98803_at	Zfp354a	zinc finger protein 354A	0.9697	0.5647	0.0061	0.4114	No	0.8301	0.0933	0.0601	0.0619	No	No
98804_at	Hesx1	homeo box gene expressed in ES cells	1.0174	0.7446	0.4307	0.6067	No	1.0961	0.261	0.25	0.461	No	No
98805_at	Tub	tubby candidate gene	0.9969	0.9718	0.128	0.4298	No	0.9603	0.712	0.284	0.867	No	No
98806_s_at	Tub	tubby candidate gene	1.092	0.018	0.7201	0.0198	No	1.1066	0.0963	0.161	0.0135	No	No

98807_at	Gnat2	guanine nucleotide binding protein, alpha transducing 2	0.9743	0.6167	0.8717	0.8949	No	1.1557	0.0826	0.214	0.0984	No	No
98808_at	Neurod2	neurogenic differentiation 2	1.0603	0.2401	0.018	0.3384	No	1.1324	0.224	0.109	0.25	No	No
98809_s_at	Nf1	neurofibromatosis 1	0.9784	0.7184	0.861	0.8643	No	1.0212	0.807	0.384	0.717	No	No
98810_at	Htr5b	5-hydroxytryptamine (serotonin) receptor 5B	1.0062	0.8381	0.7098	0.0219	No	0.9919	0.867	0.0318	0.298	No	No
98811_at	Ptgir	prostaglandin I receptor (IP)	0.9689	0.3482	0.0351	0.6946	No	1.0675	0.443	0.819	0.245	No	No
98812_at	Cd40lg	CD40 ligand	1.1187	0.0387	0.6249	0.0292	No	1.1126	0.174	0.19	0.0275	No	No
98813_at	Rel	reticuloendotheliosis oncogene	1.0801	0.1644	0.1012	0.2794	No	1.0694	0.429	0.452	0.679	No	No
98814_at	Csnd	casein delta	0.9686	0.4527	0.0259	0.8923	No	0.9183	0.489	0.133	0.526	No	No
98815_at	Evx1	even skipped homeotic gene 1 homolog	1.006	0.9058	0.122	0.8096	No	1.0472	0.219	0.0098	0.287	No	No
98816_s_at	Evx1	even skipped homeotic gene 1 homolog	0.9995	0.995	0.1793	0.5974	No	1.0479	0.436	0.167	0.215	No	No
98817_at	Fst	follistatin	0.9768	0.3308	0.0002	0.7795	No	0.8812	0.0229	0.0105	0.806	No	No
98818_at	Nr3c1	nuclear receptor subfamily 3, group C, member 1	0.9436	0.494	0.4436	0.2334	No	1.0239	0.72	0.0459	0.654	No	No
98819_at	Hoxd1	homeo box D1	1.1232	0.1441	0.0653	0.2018	No	1.1325	0.481	0.962	0.711	No	No
98820_g_at	Hoxd1	homeo box D1	0.884	0.0367	0.0483	0.2896	No	0.8813	0.211	0.0024	0.445	No	No
98821_at	Ipf1	insulin promoter factor 1, homeodomain transcription factor	0.9293	0.1405	0.7665	0.2432	No	1.1309	0.239	0.0633	0.185	No	No
98822_at	Isg15	ISG15 ubiquitin-like modifier	0.9564	0.1995	0.7254	0.6755	No	1.0056	0.834	0.688	0.0307	No	No
98823_at	Kcnj2	potassium inwardly-rectifying channel, subfamily J, member 2	0.9728	0.5224	0.3688	0.9275	No	1.0061	0.935	0.22	0.655	No	No
98824_at	Irs1	insulin receptor substrate 1	0.9822	0.8174	0.7092	0.6978	No	0.9827	0.784	0.12	0.749	No	No
98825_at	Kcne1	potassium voltage-gated channel, Isk-related subfamily, member 1	1.0644	0.2129	0.0252	0.3566	No	1.0733	0.0444	0.119	0.0103	No	No
98826_at	Fgf4	fibroblast growth factor 4	0.9777	0.6576	0.1404	0.7287	No	0.9283	0.334	0.0878	0.201	No	No
98827_i_at	Kif5c	kinesin family member 5C	1.0016	0.9763	0.0549	0.1949	No	0.944	0.857	0.0548	0.374	No	No
98828_at	Itgam	integrin alpha M	0.9823	0.6914	0.0734	0.9024	No	0.9851	0.72	0.044	0.0982	No	No
98829_at	Kcnj12	potassium inwardly-rectifying channel, subfamily J, member 12	0.9285	0.1852	0.2075	0.7381	No	0.8695	0.142	0.427	0.18	No	No
98830_at	Sbp	spermine binding protein	0.9414	0.4883	0.1703	0.9981	No	1.0654	0.597	0.378	0.606	No	No
98831_at	Foxj1	forkhead box J1	0.9162	0.1947	0.0204	0.2688	No	0.9948	0.954	0.0076	0.589	No	No
98832_at	Tpo	thyroid peroxidase	0.9113	0.3806	0.3353	0.7667	No	0.9753	0.801	0.484	0.78	No	No
98833_at	Mmp3	matrix metallopeptidase 3	0.9563	0.5047	0.3319	0.4705	No	0.9772	0.369	0.0324	0.0113	No	No
98834_at	Itga2	integrin alpha 2	1.0168	0.8002	0.2042	0.9176	No	1.0003	0.846	0.0021	0.116	No	No
98835_at	Sema3a	sema domain, immunoglobulin domain (Ig), short basic domain, secreted, (semaphorin) 3A	0.9999	0.9966	0.7358	0.515	No	0.9387	0.0664	0.0028	0.0463	No	No
98836_at	Pdcd1	programmed cell death 1	0.9822	0.764	0.0211	0.9942	No	1.1147	0.0212	0.0088	0.0197	No	No
98837_at	Rln1	relaxin 1	1.0165	0.7567	0.8165	0.2574	No	0.9691	0.687	0.858	0.968	No	No
98838_at	Pax9	paired box gene 9	1.0609	0.2571	0.4519	0.384	No	1.162	0.349	0.797	0.293	No	No
98839_at	Six2	sine oculis-related homeobox 2 homolog (Drosophila)	1.0298	0.3656	0.0004	0.6231	No	0.9758	0.681	0.001	0.196	No	No
98840_at	Bmx	BMX non-receptor tyrosine kinase	1.0809	0.2242	0.8783	0.1654	No	1.0971	0.249	0.159	0.0532	No	No
98841_at	Acvr2a	activin receptor IIA	1.0927	0.1529	0.0089	0.3436	No	1.0985	0.0642	0.0013	0.109	No	No
98842_at	Svs5	seminal vesicle secretion 5	1.0258	0.2204	0.0003	0.2061	No	1.0327	0.711	0.226	0.873	No	No
98843_at	Zic2	Zic finger protein of the cerebellum 2	0.9424	0.3222	0.0058	0.0394	No	0.9658	0.632	0.137	0.264	No	No
98844_at	Zic2	Zic finger protein of the cerebellum 2	0.9904	0.863	0.467	0.8378	No	0.9466	0.175	0.318	0.172	No	No
98845_at	Fgf5	fibroblast growth factor 5	0.9603	0.5592	0.0266	0.9596	No	0.9133	0.301	0.642	0.539	No	No
98846_f_at	Psg17	pregnancy specific glycoprotein 17	1.0054	0.8842	0.0228	0.3497	No	0.9529	0.354	0.0547	0.153	No	No
98847_at	Sdc3	syndecan 3	0.9489	0.1357	0.0019	0.0214	No	0.9935	0.93	0.062	0.379	No	No
98848_at	Sorbs3	sorbin and SH3 domain containing 3	0.8599	0.0199	0.011	0.0771	No	0.9212	0.0614	0.0622	0.0759	No	No
98849_at	D8Bwg1414e	DNA segment, Chr 8, Brigham & Women's Genetics 1414 expressed	0.9729	0.6435	0.0023	0.1985	No	1.0286	0.715	0.0062	0.9	No	No
98850_at	Tll1	tolloid-like	0.9922	0.9141	0.2	0.4056	No	0.972	0.847	0.0968	0.261	No	No
98851_at	Msi1	Musashi homolog 1(Drosophila)	0.9971	0.964	0.0066	0.9875	No	0.9808	0.597	0.0026	0.461	No	No
98852_at	Sall3	sal-like 3 (Drosophila)	0.9639	0.3506	0.1027	0.5855	No	1.0485	0.464	0.0586	0.758	No	No
98853_at	Pla2r1	phospholipase A2 receptor 1	1.0733	0.102	0.1001	0.0364	No	1.1254	0.244	0.0295	0.312	No	No
98854_at	Atxn1	ataxin 1	1.1235	0.1003	0.1412	0.7019	No	1.2234	0.0981	0.872	0.586	No	No
98855_r_at	A930001N09 Rik	RIKEN cDNA A930001N09 gene	1.0466	0.5599	0.0069	0.6543	No	1.012	0.734	0.0484	0.562	No	No
98856_at	Ptgdr	prostaglandin D receptor	1.0276	0.656	0.2544	0.442	No	1.1362	0.21	0.784	0.212	No	No
98857_at	Neurod6	neurogenic differentiation 6	1.1103	0.2763	0.5181	0.2375	No	1.2952	0.151	0.058	0.967	No	No
98858_at	Gip	gastric inhibitory polypeptide	0.958	0.5823	0.1549	0.8347	No	1.111	0.0127	0.49	0.0205	No	No
98859_at	Acp5	acid phosphatase 5, tartrate resistant	0.8841	0.0204	0.0002	0.0024	No	0.8695	0.001	0	0.0029	No	No
98860_at	Sts	steroid sulfatase	0.8539	0.0509	0.0397	0.1538	No	0.9219	0.228	0.0898	0.0612	No	No
98861_at	---	---	1.0922	0.2454	0.3613	0.5199	No	1.0297	0.234	0.0297	0.0942	No	No
98862_at	Wnt10a	wingless related MMTV integration site 10a	0.9889	0.7984	0.0551	0.9882	No	1.0663	0.0876	0.173	0.272	No	No
98863_at	Grik2	glutamate receptor, ionotropic, kainate 2 (beta 2)	0.9876	0.8077	0.1534	0.2985	No	0.9302	0.82	0.0484	0.187	No	No
98864_s_at	Grik2	glutamate receptor, ionotropic, kainate 2 (beta 2)	0.9455	0.2641	0.9381	0.6064	No	0.9911	0.867	0.157	0.507	No	No
98865_at	Has2	hyaluronan synthase 2	0.9888	0.7241	0.0982	0.5051	No	1.0601	0.161	0.239	0.0174	No	No
98866_at	Dlx6	distal-less homeobox 6	0.9086	0.2496	0.0593	0.679	No	0.9191	0.366	0.0339	0.513	No	No
98867_at	Dlx6as	distal-less homeobox 6, antisense	1.0073	0.8388	0.0114	0.1372	No	1.07	0.334	0.909	0.542	No	No
98868_at	Bcl2	B-cell leukemia/lymphoma 2	1.0162	0.7807	0.0894	0.5143	No	0.9569	0.397	0.0035	0.907	No	No
98869_g_at	Bcl2	B-cell leukemia/lymphoma 2	0.9417	0.125	0.0004	0.0828	No	0.9602	0.305	0.0003	0.226	No	No
98870_at	Bcl2	B-cell leukemia/lymphoma 2	0.9783	0.2404	0.0021	0.4768	No	1.0347	0.247	0.003	0.345	No	No
98871_at	Gpr143	G protein-coupled receptor 143	1.0113	0.5515	0.0005	0.0146	No	1.0798	0.359	0.289	0.0292	No	No
98872_at	Ugt8a	UDP galactosyltransferase 8A	0.9958	0.8912	0.0255	0.8949	No	0.9968	0.958	0.0891	0.991	No	No
98873_at	Dlx4	distal-less homeobox 4	0.9806	0.3642	0.0577	0.9023	No	1.008	0.47	0.0025	0.0048	No	No
98874_at	Traf6	Tnf receptor-associated factor 6	0.9549	0.0985	0.0649	0.9194	No	1.0089	0.635	0.01	0.218	No	No
98875_at	D2Ertd391e	DNA segment, Chr 2, ERATO Doi 391, expressed	0.8715	0.0664	0.1116	0.5111	No	0.9146	0.0984	0.0012	0.304	No	No
98876_at	Mrpl11	mitochondrial ribosomal protein L11	0.9535	0.41	0.0868	0.3959	No	0.9464	0.338	0.846	0.0367	No	No
98878_r_at	Aaas	achalasia, adrenocortical insufficiency, alacrimia	0.8884	0.0047	0.0001	0.0172	Yes	0.9245	0.0138	0	0.153	No	Yes
98880_at	D6Wsu116e	DNA segment, Chr 6, Wayne State University 116, expressed	0.9591	0.3006	0.0594	0.7971	No	0.9837	0.428	0.0498	0.584	No	No
98881_at	Hdhd2	haloacid dehalogenase-like hydrolase domain containing 2	1.0347	0.6396	0.0084	0.3021	No	1.0731	0.0481	0.0003	0.124	No	No
98882_s_at	Ndel1	nuclear distribution gene E-like homolog 1 (A. nidulans)	0.994	0.7731	0.0058	0.1121	No	0.9852	0.704	0.0181	0.0613	No	No
98883_r_at	Ndel1	nuclear distribution gene E-like homolog 1 (A. nidulans)	1.0308	0.2662	0.0002	0.02	No	0.8923	0.22	0.0002	0.953	No	No
98884_r_at	Ndel1	nuclear distribution gene E-like homolog 1 (A. nidulans)	1.1474	0.0998	0.0016	0.7084	No	1.1833	0.161	0.0609	0.809	No	No
98886_at	2810410M20 Rik /// LOC675211	RIKEN cDNA 2810410M20 gene /// similar to AIP1	1.0531	0.0439	0.0051	0.087	No	1.0819	0.0042	0.0016	0.13	Yes	Yes
98887_at	Napa	N-ethylmaleimide sensitive fusion protein attachment protein alpha	1.0235	0.6126	0.1147	0.0361	No	1.0058	0.535	0.0032	0.0039	No	No
98889_at	Aof2	amine oxidase (flavin containing) domain 2	1.074	0.1745	0.5761	0.1374	No	1.0833	0.208	0.16	0.778	No	No
98890_at	1700012G19 Rik	RIKEN cDNA 1700012G19 gene	1.0405	0.2763	0.5535	0.2575	No	0.9752	0.298	0.0646	0.0165	No	No
98891_at	1600012H06 Rik	RIKEN cDNA 1600012H06 gene	0.9274	0.0092	0.001	0.0136	Yes	1.0566	0.256	0.0208	0.259	No	No
98892_at	Lpin1	lipin 1	0.7129	0.0204	0.0828	0.2586	No	0.909	0.0062	0.211	0.0074	No	No
98893_at	Pb1	polybromo 1	1.0783	0.273	0.0004	0.3286	No	1.0643	0.573	0.0014	0.889	No	No
98894_at	Pb1	polybromo 1	0.961	0.4762	0.2935	0.4125	No	0.9335	0.331	0.0192	0.823	No	No
98896_at	5830457O10 Rik	RIKEN cDNA 5830457O10 gene	0.9769	0.5934	0.0002	0.1361	No	0.9667	0.0093	0	0.001	No	No
98901_at	1110008B24 Rik /// LOC633406	RIKEN cDNA 1110008B24 gene /// similar to Protein C14orf111 homolog	0.9475	0.1147	0.8305	0.006	No	0.9805	0.642	0.0009	0.0234	No	No
98902_at	Mrpl14	Mitochondrial ribosomal protein L14	1.0174	0.6968	0.1016	0.1858	No	1.0572	0.252	0.825	0.437	No	No
98903_at	Mrpl14	Mitochondrial ribosomal protein L14	1.0389	0.4489	0.1783	0.1588	No	1.1067	0.112	0.569	0.0146	No	No
98904_at	Mrpl35	mitochondrial ribosomal protein L35	1.002	0.9581	0.0427	0.1221	No	0.9444	0.238	0.683	0.134	No	No
98905_at	38967	septin 7	0.966	0.5425	0.9339	0.1235	No	0.9824	0.739	0.523	0.256	No	No
98906_at	Fbxo9	f-box only protein 9	0.9312	0.4286	0.0463	0.6076	No	0.9571	0.354	0.247	0.286	No	No
98908_at	Sppl3	signal peptide peptidase 3	1.0619	0.1226	0.0003	0.4	No	1.0902	0.0202	0.0004	0.635	No	No
98909_at	Lias	lipoic acid synthetase	1.1022	0.1305	0.0051	0.133	No	1.0813	0.0271	0.0001	0.0438	No	No

98910_at	1110014K08 Rik /// LOC664786 /// LOC664849 /// LOC669054 /// LOC672175	RIKEN cDNA 1110014K08 gene /// hypothetical protein LOC664786 /// hypothetical protein LOC664849 /// hypothetical protein LOC669054 /// hypothetical protein LOC672175	0.9141	0.0049	0.0007	0.099	Yes	0.9567	0.0032	0.0001	0.546	Yes	Yes
98911_at	Jak1	Janus kinase 1	0.9296	0.1088	0.0249	0.0649	No	0.8981	0.0013	0.0381	0.325	Yes	No
98912_at	Larp5	La ribonucleoprotein domain family, member 5	0.9087	0.203	0.0962	0.8985	No	0.9112	0.0394	0.0228	0.0657	No	No
98914_at	Asf1a	ASF1 anti-silencing function 1 homolog A (S. cerevisiae)	1.0577	0.3201	0.8842	0.2992	No	1.0749	0.0944	0.0401	0.754	No	No
98915_at	Rnf149	ring finger protein 149	0.9224	0.3	0.3276	0.7598	No	0.8852	0.0943	0.181	0.988	No	No
98916_at	Ppp2r2a	protein phosphatase 2 (formerly 2A), regulatory subunit B (PR 52), alpha isoform	1.0578	0.2619	0.3362	0.0309	No	1.0889	0.0999	0.0607	0.0229	No	No
98917_at	Hiatl1	hippocampus abundant transcript-like 1	1.0171	0.8332	0.0058	0.5298	No	1.022	0.852	0.0099	0.68	No	No
98918_at	Txndc5	thioredoxin domain containing 5	1.0123	0.7663	0.0716	0.6175	No	1.0474	0.259	0.0025	0.276	No	No
98919_at	Tm2d2	TM2 domain containing 2	1.0409	0.5441	0.0203	0.1806	No	1.0752	0.268	0.0623	0.403	No	No
98920_g_at	Tm2d2	TM2 domain containing 2	0.9386	0.3895	0.5169	0.9876	No	0.9802	0.781	0.0461	0.829	No	No
98921_at	Tm2d2	TM2 domain containing 2	1.0004	0.9923	0.2034	0.7003	No	1.0341	0.569	0.923	0.609	No	No
98922_at	Stt3a	STT3, subunit of the oligosaccharyltransferase complex, homolog A (S. cerevisiae)	1.0887	0.2717	0.0573	0.2278	No	1.0171	0.677	0.0097	0.328	No	No
98923_at	Rcl1	RNA terminal phosphate cyclase-like 1	0.9933	0.7876	0.8605	0.356	No	0.9658	0.299	0.232	0.286	No	No
98924_at	Art3	ADP-ribosyltransferase 3	0.849	0.1378	0.2105	0.6366	No	0.7428	0.0008	0.0955	0.0024	No	No
98925_at	Vamp2	vesicle-associated membrane protein 2	0.9649	0.6507	0.5952	0.5472	No	0.9145	0.235	0.102	0.587	No	No
98926_at	Vamp2	vesicle-associated membrane protein 2	0.9664	0.6261	0.1747	0.3002	No	1.0132	0.785	0.0917	0.96	No	No
98927_at	Rab6	RAB6, member RAS oncogene family	1.091	0.0138	0.0001	0.0063	No	1.0804	0.13	0.0122	0.0411	No	No
98928_at	Coro1b	coronin, actin binding protein 1B	0.9478	0.0245	0.0001	0.2694	No	0.9411	0.17	0.0011	0.025	No	No
98929_at	Mrps36	mitochondrial ribosomal protein S36	0.9266	0.301	0.0813	0.2794	No	0.9717	0.64	0.275	0.642	No	No
98930_at	Cope	coatomer protein complex, subunit epsilon	1.0434	0.0811	0.0003	0.0097	No	1.0195	0.139	0.0002	0.0036	No	No
98931_at	Gns	glucosamine (N-acetyl)-6-sulfatase	1.034	0.5207	0.5127	0.8854	No	0.9323	0.355	0.842	0.683	No	No
98932_at	4833427B12 Rik	RIKEN cDNA 4833427B12 gene	1.0248	0.7731	0.0936	0.6517	No	0.9583	0.62	0.929	0.936	No	No
98933_at	Mark3	MAP/microtubule affinity-regulating kinase 3	0.969	0.3451	0.0026	0.1435	No	0.9758	0.447	0.0006	0.164	No	No
98934_at	0610007P06 Rik	RIKEN cDNA 0610007P06 gene	0.9975	0.9249	0.7926	0.0344	No	0.9734	0.345	0.32	0.157	No	No
98936_at	Sars	seryl-aminoacyl-tRNA synthetase	0.9615	0.3485	0.0077	0.0604	No	0.9102	0.2	0.714	0.917	No	No
98937_at	Tbrg1	transforming growth factor beta regulated gene 1	0.9891	0.8724	0.0256	0.7361	No	0.9754	0.589	0.0053	0.802	No	No
98938_at	1500026D16 Rik	RIKEN cDNA 1500026D16 gene	1.1189	0.1377	0.0019	0.5685	No	1.0964	0.057	0.0002	0.392	No	No
98940_at	Dars	aspartyl-tRNA synthetase	1.0211	0.7866	0.4528	0.8982	No	0.8643	0.124	0.0007	0.716	No	No
98941_r_at	Prei4	preimplantation protein 4	0.8755	0.2051	0.0009	0.7906	No	0.9469	0.245	0.0005	0.199	No	No
98942_r_at	Prei4	preimplantation protein 4	1.0394	0.6776	0.0115	0.5128	No	1.0534	0.686	0.0219	0.817	No	No
98943_at	Rpa2	replication protein A2	1.0108	0.8847	0.0261	0.2037	No	1.0025	0.965	0.0047	0.761	No	No
98944_at	Sec23b	SEC23B (S. cerevisiae)	1.0425	0.3384	0.2274	0.7616	No	1.0619	0.194	0.0993	0.863	No	No
98945_at	Sh3glb1	SH3-domain GRB2-like B1 (endophilin)	0.9778	0.7452	0.0302	0.6507	No	0.9952	0.897	0.25	0.0701	No	No
98946_at	Wsb1	WD repeat and SOCS box-containing 1	1.0456	0.7169	0.6235	0.4911	No	0.9969	0.876	0.049	0.193	No	No
98947_at	Brd3	bromodomain containing 3	1.0585	0.4616	0.0719	0.438	No	1.2168	0.052	0.314	0.269	No	No
98948_at	Gnl3	guanine nucleotide binding protein-like 3 (nucleolar)	1.076	0.1278	0.1407	0.2938	No	1.0222	0.685	0.451	0.493	No	No
98950_at	Rragc	Ras-related GTP binding C	0.963	0.0865	0.0049	0.1119	No	0.9978	0.956	0.724	0.693	No	No
98951_at	Tcf25	transcription factor 25 (basic helix-loop-helix)	1.0633	0.2642	0.1858	0.2217	No	1.0171	0.371	0.0001	0.322	No	No
98952_at	Tcf25	transcription factor 25 (basic helix-loop-helix)	1.0171	0.4399	0.0003	0.0593	No	1.0218	0.529	0.0017	0.982	No	No
98953_at	Eif1b	eukaryotic translation initiation factor 1B	1.0812	0.0961	0.1649	0.3156	No	1.1559	0.0746	0.201	0.659	No	No
98954_f_at	Oog1	oogenesin 1	0.9905	0.8314	0.4179	0.5753	No	1.0118	0.829	0.0869	0.0354	No	No
98955_at	Stk25	serine/threonine kinase 25 (yeast)	1.1927	0.0538	0.8162	0.8507	No	0.9906	0.785	0.0002	0.265	No	No
98956_at	Tram1	translocating chain-associating membrane protein 1	1.01	0.8276	0.8517	0.1444	No	1.0323	0.503	0.0109	0.787	No	No
98957_at	Jam3	junction adhesion molecule 3	1.01	0.7727	0.0023	0.0971	No	1.0482	0.355	0.0129	0.277	No	No
98958_at	Eny2	enhancer of yellow 2 homolog (Drosophila)	1.0158	0.8519	0.0006	0.2035	No	0.9715	0.881	0.0005	0.295	No	No
98959_at	0610016J10 Rik /// LOC619602	RIKEN cDNA 0610016J10 gene /// hypothetical LOC619602	0.9379	0.3046	0.0293	0.587	No	0.9408	0.0099	0.0004	0.556	Yes	No
98960_s_at	B3galnt1	UDP-GalNAc:betaGlcNAc beta 1,3-galactosaminyltransferase, polypeptide 1	1.0138	0.8244	0.8022	0.3526	No	1.0173	0.569	0.0126	0.156	No	No
98961_at	B3galnt1	UDP-GalNAc:betaGlcNAc beta 1,3-galactosaminyltransferase, polypeptide 1	0.9785	0.6714	0.0552	0.5373	No	1.0442	0.323	0.0016	0.248	No	No
98962_at	Lipc	lipase, hepatic	0.9023	0.1258	0.0245	0.5155	No	0.9906	0.821	0.469	0.0452	No	No
98963_at	Trpv2	transient receptor potential cation channel, subfamily V, member 2	0.8919	0.1471	0.4566	0.3612	No	0.981	0.742	0.88	0.581	No	No
98965_at	Oact5	O-acyltransferase (membrane bound) domain containing 5	0.9206	0.1332	0.4768	0.0378	No	1.0157	0.796	0.0206	0.183	No	No
98966_at	Dbt	dihydrolipoamide branched chain transacylase E2	0.8418	0.0396	0.0006	0.0743	No	0.892	0.0646	0.0011	0.0375	No	No
98967_at	Fabp7	fatty acid binding protein 7, brain	1.4964	0.0021	0.01	0.1445	Yes	1.3255	0.0046	0.0394	0.135	Yes	Yes
98968_at	Myo5a	myosin Va	1.0226	0.7138	0.0005	0.7584	No	1.0443	0.283	0	0.444	No	No
98969_at	Abcd1	ATP-binding cassette, sub-family D (ALD), member 1	0.9311	0.0687	0.0002	0.1646	No	0.8888	0.0067	0.004	0.0045	No	No
98970_at	Ggps1	geranylgeranyl diphosphate synthase 1	1.0515	0.0251	0.0262	0.8306	No	1.0737	0.0688	0.0101	0.859	No	No
98971_at	Rad54l	RAD54 like (S. cerevisiae)	0.9787	0.5915	0.9886	0.2943	No	0.9973	0.567	0.0002	0.0073	No	No
98972_at	Usp8	ubiquitin specific peptidase 8	0.8927	0.0283	0.0351	0.0781	No	0.8742	0.0552	0.522	0.0331	No	No
98973_at	Wdr43	WD repeat domain 43	0.9169	0.0451	0.1262	0.4435	No	0.9592	0.0746	0.554	0.371	No	No
98974_at	Igh-1a	Immunoglobulin heavy chain 1a (serum IgG2a)	0.9409	0.2465	0.1609	0.444	No	0.8514	0.196	0.0518	0.608	No	No
98975_at	Nup93	nucleoporin 93	1.1502	0.0102	0.0026	0.0115	No	1.0712	0.199	0.01	0.21	No	No
98976_at	Adamdec1	ADAM-like, decysin 1	1.026	0.6198	0.0556	0.7033	No	1.0166	0.828	0.107	0.466	No	No
98977_at	Tinf2	Terf1 (TRF1)-interacting nuclear factor 2	1.0373	0.5109	0.0071	0.6713	No	1.0049	0.892	0.0036	0.862	No	No
98979_at	Mnat1	menage a trois 1	0.9462	0.4418	0.3657	0.6171	No	1.0107	0.868	0.624	0.424	No	No
98980_at	Cd37	CD37 antigen	0.9651	0.6131	0.3285	0.6073	No	1.0062	0.871	0.0019	0.855	No	No
98981_s_at	Tcf12	transcription factor 12	0.9827	0.8225	0.0409	0.6761	No	0.9842	0.695	0.0212	0.271	No	No
98982_at	Tmpo	thymopoietin	0.9856	0.5549	0.0001	0.0219	No	1.0364	0.0122	0.0001	0.0003	No	No
98983_at	P4ha2	procollagen-proline, 2-oxoglutarate 4-dioxygenase (proline 4-hydroxylase), alpha II polypeptide	0.9872	0.6387	0.0315	0.5002	No	1.0528	0.163	0.0188	0.307	No	No
98984_f_at	Gpd2	glycerol phosphate dehydrogenase 2, mitochondrial	0.9475	0.351	0.0005	0.0357	No	1.0612	0.135	0.0017	0.103	No	No
98987_at	Faf1	Fas-associated factor 1	1.1204	0.0344	0.0027	0.0243	No	0.9923	0.626	0.0001	0.348	No	No
98988_at	Nfkbiz	nuclear factor of kappa light polypeptide gene enhancer in B-cells inhibitor, zeta	1.0016	0.983	0.0032	0.3633	No	1.0261	0.51	0.0462	0.0373	No	No
98989_at	Dhcr7	7-dehydrocholesterol reductase	0.9879	0.8302	0.0347	0.1475	No	1.023	0.807	0.0015	0.0316	No	No
98990_at	Bcar1	breast cancer anti-estrogen resistance 1	0.9822	0.7099	0.0169	0.5256	No	0.9	0.112	0.042	0.218	No	No
98991_at	Smarcad1	SWI/SNF-related, matrix-associated actin-dependent regulator of chromatin, subfamily a, containing DEAD/H box 1`	0.9643	0.5295	0.0075	0.6115	No	1.0128	0.812	0.0051	0.959	No	No
98992_at	Eppb9	endothelial precursor protein B9	1.0063	0.9049	0.0828	0.1272	No	1.0696	0.0647	0.0043	0.395	No	No
98993_at	Ppp2r5c	protein phosphatase 2, regulatory subunit B (B56), gamma isoform	1.1124	0.1373	0.0727	0.9834	No	1.0917	0.233	0.383	0.132	No	No
98994_at	Slc34a2	solute carrier family 34 (sodium phosphate), member 2	0.9647	0.2247	0.0029	0.444	No	1.0119	0.809	0.0115	0.932	No	No
98995_at	E2f5	E2F transcription factor 5	0.9804	0.2834	0.011	0.916	No	0.9427	0.0188	0.532	0.0419	No	No
98996_at	Plk4	polo-like kinase 4 (Drosophila)	1.0767	0.2292	0.0007	0.6437	No	1.0878	0.283	0.0011	0.495	No	No
98997_f_at	Krt1-24	keratin complex 1, acidic, gene 24	1.0056	0.9357	0.523	0.0507	No	0.9979	0.795	0.0028	0.217	No	No
98998_r_at	Krt1-24	keratin complex 1, acidic, gene 24	0.9679	0.3473	0.8503	0.9872	No	0.9537	0.327	0.822	0.687	No	No
98999_at	Adsl	adenylosuccinate lyase	1.0442	0.2832	0.5399	0.0104	No	1.0039	0.682	0.0004	0.113	No	No
99000_at	Mapk7	mitogen activated protein kinase 7	1.0252	0.5235	0.0503	0.1209	No	1.1103	0.03	0.0039	0.572	No	No
99001_at	Zfp292	zinc finger protein 292	0.9063	0.0166	0.0001	0.7915	No	0.951	0.0613	0.0001	0.657	No	No
99004_r_at	LOC236469 /// LOC547109 /// LOC666747	similar to bloodthirsty /// similar to tripartite motif-containing 43 /// similar to tripartite motif protein 17	1.0327	0.7323	0.8311	0.249	No	1.1594	0.0933	0.504	0.0287	No	No
99005_at	Rad1	RAD1 homolog (S. pombe)	1.0131	0.7434	0.0333	0.9705	No	1.0442	0.1	0.0002	0.733	No	No
99006_at	2810040C05 Rik	RIKEN cDNA 2810040C05 gene	0.9719	0.7349	0.5234	0.9072	No	1.0085	0.869	0.535	0.994	No	No
99007_at	Flot2	flotillin 2	0.9754	0.4839	0.1013	0.4838	No	1.0428	0.224	0.185	0.0089	No	No
99009_at	Nnt	nicotinamide nucleotide transhydrogenase	1.0309	0.6496	0.0328	0.3732	No	0.919	0.0473	0.0946	0.427	No	No

99010_at	Islr	immunoglobulin superfamily containing leucine-rich repeat	1.022	0.5778	0.0013	0.1488	No	1.0052	0.911	0.0002	0.715	No	No
99011_at	Galnt3	UDP-N-acetyl-alpha-D-galactosamine:polypeptide N-acetylgalactosaminyltransferase 3	0.9885	0.8398	0.9216	0.8048	No	0.9855	0.778	0.154	0.228	No	No
99012_at	Spef1	sperm flagellar 1	1.0383	0.0513	0.0016	0.9454	No	1.0025	0.958	0.0266	0.363	No	No
99013_f_at	Tmod3	tropomodulin 3	0.9652	0.4603	0.0009	0.2074	No	0.9732	0.466	0.0012	0.332	No	No
99014_at	Apbb1	amyloid beta (A4) precursor protein-binding, family B, member 1	0.9427	0.1271	0.9225	0.4713	No	0.9618	0.186	0.844	0.136	No	No
99015_at	Pml	promyelocytic leukemia	0.9882	0.8388	0.7203	0.1326	No	0.9853	0.794	0.724	0.231	No	No
99016_at	---	---	1.0901	0.2095	0.1861	0.1127	No	1.1701	0.007	0.0475	0.0035	No	No
99018_at	Bclaf1	BCL2-associated transcription factor 1	0.8605	0.1299	0.0374	0.9207	No	0.8816	0.129	0.311	0.817	No	No
99019_at	Por	P450 (cytochrome) oxidoreductase	0.9546	0.1593	0.0055	0.8931	No	1.0098	0.801	0.0043	0.465	No	No
99020_at	Yy1 /// LOC629517 /// LOC675872	YY1 transcription factor /// hypothetical LOC629517 /// hypothetical protein LOC675872	0.9424	0.3817	0.1055	0.3782	No	1.0521	0.117	0.0049	0.198	No	No
99021_at	Prrx1	paired related homeobox 1	0.9631	0.7032	0.0484	0.166	No	0.8948	0.228	0.0024	0.684	No	No
99023_at	Pafah1b2	platelet-activating factor acetylhydrolase, isoform 1b, alpha2 subunit	1.0164	0.3046	0.0005	0.0023	No	0.9936	0.842	0.122	0.765	No	No
99024_at	Mxd4	Max dimerization protein 4	0.9182	0.1598	0.8919	0.2487	No	0.9409	0.293	0.839	0.47	No	No
99025_at	Ddx19a	DEAD (Asp-Glu-Ala-Asp) box polypeptide 19a	1.0073	0.9199	0.1544	0.3239	No	1.0622	0.45	0.0784	0.745	No	No
99026_at	Bcl2l1	Bcl2-like 1	0.8922	0.2674	0.0958	0.3533	No	0.8396	0.16	0.0217	0.0466	No	No
99027_at	Bcl2l1	Bcl2-like 1	0.9769	0.4276	0.0091	0.2038	No	0.9837	0.759	0.674	0.948	No	No
99028_at	Rnf8	ring finger protein 8	0.998	0.9689	0.0455	0.1892	No	1.0435	0.664	0.0568	0.343	No	No
99029_at	Pias1	protein inhibitor of activated STAT 1	0.925	0.2002	0.8177	0.2673	No	1.0726	0.21	0.0004	0.147	No	No
99030_at	Il7r	interleukin 7 receptor	1.0297	0.5566	0.3728	0.1415	No	1.1411	0.117	0.759	0.0334	No	No
99031_at	Ankrd17	ankyrin repeat domain 17	0.9931	0.8433	0.0001	0.1951	No	0.9606	0.65	0.0103	0.57	No	No
99032_at	Rasd1	RAS, dexamethasone-induced 1	0.9228	0.4956	0.0552	0.8456	No	0.9936	0.912	0.0285	0.482	No	No
99033_at	Itpk1	inositol 1,3,4-triphosphate 5/6 kinase	0.9603	0.0489	0.0954	0.2067	No	1.0117	0.711	0.0076	0.608	No	No
99034_at	Irx3	Iroquois related homeobox 3 (Drosophila)	1.189	0.0269	0.0745	0.4047	No	1.1601	0.0245	0.0615	0.0642	No	No
99035_at	Pcyt1a	phosphate cytidylyltransferase 1, choline, alpha isoform	1.0015	0.9737	0.9011	0.7073	No	1.0168	0.649	0.23	0.4	No	No
99036_s_at	Map2k7	mitogen activated protein kinase kinase 7	1.0174	0.7431	0.3266	0.4015	No	1.0727	0.44	0.263	0.701	No	No
99037_at	Map2k7	mitogen activated protein kinase kinase 7	1.0678	0.2344	0.5155	0.4689	No	1.0511	0.2	0.943	0.071	No	No
99038_at	Adss	adenylosuccinate synthetase, non muscle	1.0297	0.5474	0.9657	0.2898	No	1.0064	0.848	0.119	0.236	No	No
99039_g_at	Adss	adenylosuccinate synthetase, non muscle	1.0326	0.5186	0.7592	0.4429	No	1.0079	0.82	0.374	0.398	No	No
99040_at	Slc6a4	solute carrier family 6 (neurotransmitter transporter, serotonin), member 4	0.9425	0.3156	0.2035	0.1361	No	0.9556	0.286	0.0064	0.916	No	No
99041_at	Taf1b	TATA box binding protein (Tbp)-associated factor, RNA polymerase I, B	0.973	0.1504	0.3843	0.8072	No	1.0159	0.514	0.0943	0.212	No	No
99042_s_at	Shox2	short stature homeobox 2	1.0555	0.2324	0.0028	0.947	No	1.0584	0.301	0.01	0.557	No	No
99043_s_at	Shox2	short stature homeobox 2	0.9565	0.2246	0.4506	0.4441	No	0.9694	0.977	0.012	0.0984	No	No
99044_at	Zfp358	zinc finger protein 358	0.9898	0.7221	0.0038	0.9616	No	1.0653	0.0672	0.0504	0.381	No	No
99045_at	Eno2	enolase 2, gamma neuronal	1.0041	0.9032	0.0023	0.3701	No	0.9807	0.0984	0.003	0.0183	No	No
99046_at	Mobp	myelin-associated oligodendrocytic basic protein	1.0104	0.8302	0.0239	0.1079	No	1.2579	0.0228	0.0019	0.505	No	No
99047_at	Mobp	myelin-associated oligodendrocytic basic protein	0.9554	0.4241	0.1556	0.3974	No	0.8621	0.0113	0.0116	0.167	No	No
99048_g_at	Mobp	myelin-associated oligodendrocytic basic protein	1.0596	0.1997	0.15	0.8242	No	1.1452	0.0762	0.414	0.0801	No	No
99049_at	Casp2	caspase 2	0.9907	0.8236	0.0553	0.7973	No	0.9727	0.495	0.806	0.159	No	No
99050_at	Cav3	caveolin 3	0.8108	0.001	0.1189	0.0012	No	0.9213	0.0643	0.36	0.717	No	No
99051_at	S100a4	S100 calcium binding protein A4	0.96	0.3545	0.0149	0.4552	No	0.9488	0.162	0.0116	0.0337	No	No
99052_at	Zfhx1a	zinc finger homeobox 1a	0.9849	0.6506	0.0052	0.1211	No	0.9442	0.33	0.0373	0.73	No	No
99053_at	Icam2	intercellular adhesion molecule 2	0.8801	0.1601	0.5016	0.5611	No	0.8869	0.135	0.134	0.478	No	No
99054_at	Llgl1	lethal giant larvae homolog 1 (Drosophila)	1.0316	0.088	0.253	0.0127	No	0.9868	0.704	0.16	0.67	No	No
99055_at	St6galnac2	ST6 (alpha-N-acetyl-neuraminyl-2,3-beta-galactosyl-1,3)-N-acetylgalactosaminide alpha-2,6-sialyltransferase 2	0.9081	0.103	0.864	0.9601	No	0.9679	0.548	0.0325	0.0695	No	No
99056_at	Pcbd1	pterin 4 alpha carbinolamine dehydratase/dimerization cofactor of hepatocyte nuclear factor 1 alpha (TCF1) 1	1.0466	0.5091	0.5181	0.9752	No	1.2024	0.0668	0.0395	0.389	No	No
99057_at	Thy1	thymus cell antigen 1, theta	0.9123	0.1499	0.0289	0.0172	No	0.9957	0.964	0.0079	0.515	No	No
99058_at	Hmga2	high mobility group AT-hook 2	1.0718	0.0788	0.0009	0.0085	No	0.965	0.81	0.0085	0.0208	No	No
99059_at	Elf3	E74-like factor 3	0.9819	0.7588	0.2192	0.3766	No	1.0761	0.116	0.236	0.526	No	No
99062_at	Pvrl3	poliovirus receptor-related 3	0.9583	0.4933	0.01	0.5994	No	0.9537	0.136	0.0001	0.486	No	No
99063_at	Stx8	syntaxin 8	0.9798	0.4851	0.9434	0.1386	No	1.01	0.791	0.683	0.29	No	No
99064_at	Usp4	ubiquitin specific peptidase 4 (proto-oncogene)	1.032	0.306	0.0562	0.308	No	1.0168	0.547	0.0024	0.133	No	No
99065_at	Csnk	casein kappa	0.9867	0.8429	0.8618	0.6465	No	1.0626	0.102	0.308	0.421	No	No
99067_at	Gas6	growth arrest specific 6	0.8774	0.0365	0.0188	0.0952	No	0.9259	0.114	0.0075	0.249	No	No
99068_at	Anapc1	anaphase promoting complex subunit 1	0.9157	0.028	0.437	0.0616	No	1.1348	0.0093	0.62	0.0034	No	No
99069_at	Ntrk2	neurotrophic tyrosine kinase, receptor, type 2	0.9214	0.4551	0.1501	0.639	No	1.0391	0.641	0.197	0.496	No	No
99070_at	Chuk	conserved helix-loop-helix ubiquitous kinase	1.0598	0.4468	0.0158	0.5317	No	1.0131	0.836	0.0361	0.536	No	No
99071_at	Mpeg1 /// LOC671359	macrophage expressed gene 1 /// similar to macrophage expressed gene 1	1.0444	0.1214	0.001	0.0075	No	1.0447	0.465	0.306	0.937	No	No
99073_at	Ccnf	cyclin F	0.9486	0.1234	0.8128	0.0417	No	0.9836	0.289	0.974	0.446	No	No
99074_at	Vil1	villin 1	0.9879	0.837	0.1529	0.1846	No	0.9594	0.363	0.257	0.0187	No	No
99076_at	Nr1d2	nuclear receptor subfamily 1, group D, member 2	1.0211	0.7437	0.0901	0.3997	No	1.074	0.0155	0.017	0.0033	No	No
99077_at	Thra	thyroid hormone receptor alpha	1.0873	0.1406	0.0188	0.0889	No	0.9693	0.769	0.009	0.481	No	No
99078_at	Dohh	deoxyhypusine hydroxylase/monooxygenase	0.9555	0.34	0.8944	0.1319	No	0.9543	0.447	0.304	0.156	No	No
99080_at	Ccdc6	coiled-coil domain containing 6	0.8387	0.0098	0.1596	0.2205	Yes	0.9078	0.117	0.149	0.257	No	No
99081_at	Serping1	serine (or cysteine) peptidase inhibitor, clade G, member 1	0.9816	0.384	0.5631	0.2229	No	1.115	0.304	0.417	0.439	No	No
99082_at	Fkbp10	FK506 binding protein 10	1.0138	0.786	0.0009	0.3334	No	1.0095	0.694	0.0005	0.451	No	No
99083_at	Cyp2c37	cytochrome P450, family 2. subfamily c, polypeptide 37	0.9959	0.9282	0.0808	0.6467	No	1.0229	0.574	0.365	0.924	No	No
99084_s_at	Usp3	ubiquitin specific peptidase 3	1.002	0.9531	0.0057	0.4	No	0.9152	0.0376	0.063	0.0081	No	No
99085_at	Usp3	ubiquitin specific peptidase 3	0.9768	0.6541	0.2375	0.8823	No	1.0398	0.0447	0.0132	0.0024	No	No
99086_g_at	Usp3	ubiquitin specific peptidase 3	1.0417	0.3627	0.0116	0.22	No	1.0549	0.176	0.0052	0.501	No	No
99087_at	Zwint	ZW10 interactor	1.0112	0.3302	0	0.0195	No	1.0115	0.805	0.0047	0.0472	No	No
99089_at	Mal	myelin and lymphocyte protein, T-cell differentiation protein	0.9918	0.8133	0.0564	0.6541	No	0.9545	0.057	0.0614	0.0037	No	No
99093_at	Rps10 /// LOC621772 /// LOC636539 /// LOC666274 /// LOC668457	ribosomal protein S10 /// similar to ribosomal protein S10 /// similar to ribosomal protein S10 /// similar to ribosomal protein S10 /// similar to ribosomal protein S10	1.0611	0.3837	0.0114	0.5741	No	1.0436	0.281	0.0071	0.159	No	No
99094_at	Slc12a1	solute carrier family 12, member 1	1.0973	0.2591	0.7535	0.237	No	1.1373	0.187	0.091	0.0533	No	No
99095_at	Max	Max protein	0.929	0.4942	0.9768	0.5164	No	0.8861	0.157	0.146	0.485	No	No
99096_at	Ddx24	DEAD (Asp-Glu-Ala-Asp) box polypeptide 24	1.026	0.5503	0.0242	0.2578	No	0.9677	0.497	0	0.0025	No	No
99097_at	Eif4e	eukaryotic translation initiation factor 4E	1.0018	0.936	0.0359	0.534	No	1.0779	0.036	0.0308	0.0109	No	No
99098_at	Fdps	farnesyl diphosphate synthetase	1.0904	0.1861	0.0025	0.5731	No	1.0645	0.333	0.0041	0.604	No	No
99099_at	Stat3	signal transducer and activator of transcription 3	0.9568	0.3169	0.022	0.6951	No	0.9368	0.165	0.222	0.16	No	No
99100_at	Stat3	signal transducer and activator of transcription 3	0.885	0.0798	0.0066	0.3599	No	0.936	0.239	0.265	0.551	No	No
99101_at	Eif3s7	eukaryotic translation initiation factor 3, subunit 7 (zeta)	1	0.9996	0.0284	0.0827	No	0.9875	0.811	0.0118	0.556	No	No
99102_at	Usp9x	ubiquitin specific peptidase 9, X chromosome	1.1027	0.2098	0.0455	0.1972	No	1.0059	0.805	0.0422	0.132	No	No
99103_at	Irf3	interferon regulatory factor 3	0.9686	0.286	0.0893	0.7514	No	0.9518	0.305	0.0008	0.581	No	No
99104_at	Adipoq	adiponectin, C1Q and collagen domain containing	1.2268	0.0218	0.0033	0.7225	No	1.0402	0.343	0.019	0.0775	No	No
99106_at	Cops6	COP9 (constitutive photomorphogenic) homolog, subunit 6 (Arabidopsis thaliana)	1.0581	0.1387	0.0004	0.02	No	1.0282	0.326	0.0028	0.0267	No	No
99107_at	Ghr	growth hormone receptor	0.8976	0.0523	0.0078	0.1538	No	0.8522	0.0131	0.746	0.0281	No	No
99108_s_at	Ghr	growth hormone receptor	0.8503	0.0008	0.0161	0.0017	No	0.8301	0.0009	0.108	0.0103	Yes	No
99109_at	Ier2	immediate early response 2	1.0888	0.1396	0.7119	0.2394	No	1.0116	0.999	0.0971	0.0951	No	No
99111_at	Clpb	ClpB caseinolytic peptidase B homolog (E. coli)	0.9418	0.418	0.1475	0.1954	No	0.9419	0.0285	0.0001	0.0084	No	No
99112_at	Slc25a10	solute carrier family 25 (mitochondrial carrier, dicarboxylate transporter), member 10	1.0546	0.1655	0.1168	0.1648	No	1.1364	0.0185	0.003	0.348	No	No
99113_at	Cops3	COP9 (constitutive photomorphogenic) homolog, subunit 3 (Arabidopsis thaliana)	0.9825	0.8016	0.4328	0.8726	No	0.9913	0.932	0.0011	0.239	No	No
99114_r_at	Etv2 /// Cox6b1	ets variant gene 2 /// cytochrome c oxidase, subunit VIb polypeptide 1	1.0869	0.2998	0.355	0.1924	No	0.9413	0.0103	0.0045	0.157	No	No

99115_at	Uqcrh	ubiquinol-cytochrome c reductase hinge protein	1.0207	0.7485	0.1849	0.5521	No	0.9161	0.204	0.0313	0.272	No	No
99118_at	Prap1	proline-rich acidic protein 1	0.9439	0.3766	0.7284	0.977	No	1.0085	0.916	0.728	0.705	No	No
99119_at	Cfl1	cofilin 1, non-muscle	0.9525	0.6202	0.0098	0.1822	No	0.9834	0.523	0.0024	0.132	No	No
99120_f_at	Chd4	chromodomain helicase DNA binding protein 4	0.9877	0.6131	0.0167	0.2641	No	1.0254	0.477	0.447	0.248	No	No
99121_at	Fxr1h	fragile X mental retardation gene 1, autosomal homolog	0.9509	0.6393	0.7983	0.4798	No	0.9129	0.597	0.279	0.639	No	No
99123_s_at	Fxc1	fractured callus expressed transcript 1	1.0113	0.8215	0.0039	0.412	No	1.0694	0.0199	0.0001	0.0717	No	No
99124_at	Fxc1	fractured callus expressed transcript 1	0.988	0.7546	0.2496	0.8317	No	0.9844	0.734	0.317	0.148	No	No
99126_at	Xist	inactive X specific transcripts	0.6987	0.0127	0.0002	0.0984	No	0.7713	0.0272	0.0001	0.445	No	No
99127_at	Atxn10	ataxin 10	1.0698	0.0777	0.5181	0.94	No	1.0237	0.551	0.0747	0.919	No	No
99128_at	Atp5o	ATP synthase, H+ transporting, mitochondrial F1 complex, O subunit	1.0319	0.3034	0.6579	0.0035	No	0.9766	0.317	0.0638	0.025	No	No
99129_at	Tnfsf5ip1	tumor necrosis factor superfamily, member 5-induced protein 1	1.0755	0.3071	0.0784	0.2176	No	0.9962	0.979	0.31	0.0563	No	No
99130_at	Csnb	casein beta	1.0185	0.6905	0.0498	0.2953	No	0.9812	0.637	0.225	0.0099	No	No
99133_at	Slc3a2	solute carrier family 3 (activators of dibasic and neutral amino acid transport), member 2	0.9601	0.0401	0.0986	0.0096	No	0.9731	0.395	0.896	0.727	No	No
99134_at	Tcte3	t-complex-associated testis expressed 3	0.919	0.0705	0.005	0.6383	No	0.9493	0.285	0.0029	0.662	No	No
99135_at	Cdc37	cell division cycle 37 homolog (S. cerevisiae)	1.0082	0.8536	0.0022	0.0437	No	0.9977	0.935	0.0042	0.175	No	No
99136_at	Axl	AXL receptor tyrosine kinase	0.9158	0.0914	0.7189	0.0861	No	1.0644	0.169	0.0036	0.13	No	No
99138_at	Rcc1	regulator of chromosome condensation 1	0.9902	0.8564	0.2921	0.4954	No	0.9939	0.625	0.0005	0.0939	No	No
99139_at	Vps26b	vacuolar protein sorting 26 homolog B (yeast)	0.9802	0.7902	0.16	0.3404	No	0.9577	0.362	0.019	0.228	No	No
99140_at	Mrpl16	mitochondrial ribosomal protein L16	1.0304	0.5241	0.0118	0.3519	No	1.019	0.536	0.182	0.0057	No	No
99141_at	Gm2a	GM2 ganglioside activator protein	0.9213	0.0572	0.0869	0.1359	No	0.9208	0.025	0.039	0.0145	No	No
99142_at	Paqr7	progestin and adipoQ receptor family member VII	1.0412	0.0477	0.0225	0.0066	No	1.0056	0.833	0.329	0.347	No	No
99143_at	Tgoln1	trans-golgi network protein	1.0744	0.1162	0.0005	0.2823	No	0.9779	0.415	0.0001	0.482	No	No
99144_s_at	Tgoln1	trans-golgi network protein	1.0364	0.5713	0.7867	0.2039	No	0.9265	0.411	0.604	0.694	No	No
99146_at	Stx6	syntaxin 6	1.102	0.1442	0.1815	0.4738	No	1.1648	0.114	0.926	0.482	No	No
99147_at	Znhit1	zinc finger, HIT domain containing 1	0.9934	0.8651	0.0169	0.579	No	0.9788	0.674	0.0002	0.0574	No	No
99148_at	Fh1	fumarate hydratase 1	0.9533	0.2568	0.039	0.1439	No	0.9836	0.687	0.299	0.134	No	No
99149_at	LOC630539	similar to mouse RING finger 1	0.9454	0.4097	0.1865	0.035	No	0.9254	0.373	0.0429	0.0469	No	No
99150_at	Ict1	immature colon carcinoma transcript 1	0.9651	0.4479	0.0681	0.0712	No	1.0038	0.838	0.339	0.0038	No	No
99151_at	Exosc7	exosome component 7	0.9986	0.9107	0.1158	0.921	No	0.9884	0.49	0.0015	0.369	No	No
99152_at	Fmod	fibromodulin	0.9124	0.1064	0.0012	0.9506	No	0.9028	0.0422	0.0002	0.497	No	No
99153_at	Mrpl53	mitochondrial ribosomal protein L53	1.0022	0.9759	0.6092	0.1617	No	1.038	0.422	0.105	0.0208	No	No
99154_s_at	1810020D17 Rik	RIKEN cDNA 1810020D17 gene	1.0203	0.4092	0.4353	0.126	No	1.0639	0.115	0.918	0.312	No	No
99156_at	Pdzd11	PDZ domain containing 11	0.9253	0.0564	0.0004	0.0991	No	0.9579	0.644	0.0897	0.693	No	No
99157_at	2310004N24 Rik	RIKEN cDNA 2310004N24 gene	1.0216	0.597	0.0182	0.1096	No	1.024	0.493	0.025	0.385	No	No
99158_at	Ostf1	osteoclast stimulating factor 1	0.9792	0.638	0.531	0.8976	No	0.9867	0.804	0.367	0.751	No	No
99159_at	Ppif	peptidylprolyl isomerase F (cyclophilin F)	0.9957	0.8099	0.7687	0.0162	No	1.0581	0.142	0.0081	0.366	No	No
99160_s_at	Grina	glutamate receptor, ionotropic, N-methyl D-asparate-associated protein 1 (glutamate binding)	0.9741	0.7	0.98	0.4777	No	0.9887	0.916	0.0564	0.228	No	No
99161_at	Grina	glutamate receptor, ionotropic, N-methyl D-asparate-associated protein 1 (glutamate binding)	0.9654	0.4262	0.0483	0.7806	No	0.9763	0.543	0.942	0.885	No	No
99162_at	Smndc1	survival motor neuron domain containing 1	0.9778	0.4771	0.0001	0.109	No	0.9952	0.856	0.0005	0.925	No	No
99163_at	4921506J03 Rik	RIKEN cDNA 4921506J03 gene	0.9928	0.8491	0.0032	0.5124	No	0.9408	0.267	0.0109	0.0868	No	No
99164_at	Mapbpip	mitogen activated protein binding protein interacting protein	1.0552	0.4143	0.3893	0.2943	No	1.0456	0.516	0.361	0.29	No	No
99166_at	0610012G03 Rik	RIKEN cDNA 0610012G03 gene	1.0266	0.5884	0.0015	0.0596	No	1.0029	0.54	0.0005	0.0436	No	No
99167_at	Commd4	COMM domain containing 4	0.9785	0.7303	0.8429	0.9213	No	0.9794	0.766	0.273	0.218	No	No
99168_at	Pigs	phosphatidylinositol glycan, class S	0.9983	0.9697	0.008	0.402	No	1.0564	0.303	0.0305	0.571	No	No
99169_at	Prmt4	protein arginine N-methyltransferase 4	1.0319	0.2662	0.0005	0.0067	No	1.0772	0.0747	0.0034	0.127	No	No
99171_at	Ints6	integrator complex subunit 6	1.1707	0.0771	0.1234	0.0072	No	1.1337	0.165	0.132	0.0172	No	No
99172_at	Tfam	transcription factor A, mitochondrial	1.0585	0.3986	0.2702	0.7226	No	1.1178	0.0759	0.326	0.0271	No	No
99175_at	Gng10	guanine nucleotide binding protein (G protein), gamma 10	1.0722	0.5246	0.9524	0.5199	No	0.9712	0.277	0.971	0.16	No	No
99176_at	Fibp	fibroblast growth factor (acidic) intracellular binding protein	1.0555	0.215	0.0013	0.2709	No	1.0559	0.28	0.0026	0.599	No	No
99178_at	Gpm6b	glycoprotein m6b	1.0327	0.787	0.295	0.9197	No	1.0412	0.564	0.111	0.249	No	No
99179_at	Cuedc2	CUE domain containing 2	1.0658	0.3376	0.0216	0.3461	No	1.0415	0.093	0.0001	0.0272	No	No
99180_at	Gtpbp4	GTP binding protein 4	0.9213	0.214	0.4353	0.2409	No	0.9229	0.225	0.0056	0.807	No	No
99182_at	Rpp14	ribonuclease P 14 subunit (human)	1.0402	0.044	0.0007	0.1395	No	1.0464	0.105	0.0002	0.266	No	No
99183_at	Ppp3r1	protein phosphatase 3, regulatory subunit B, alpha isoform (calcineurin B, type I)	0.9424	0.5043	0.3706	0.4392	No	0.9552	0.521	0.905	0.373	No	No
99184_at	Csad	cysteine sulfinic acid decarboxylase	1.004	0.8996	0.0229	0.3327	No	1.1099	0.0298	0.0192	0.0189	No	No
99185_at	Mrpl41	mitochondrial ribosomal protein L41	1.0726	0.2096	0.0168	0.8275	No	1.0479	0.406	0.743	0.841	No	No
99186_at	Ccna2	cyclin A2	1.0428	0.447	0.5149	0.872	No	1.0338	0.207	0.0042	0.149	No	No
99187_f_at	2010315L10 Rik	RIKEN cDNA 2010315L10 gene	0.883	0.0223	0.0031	0.0053	No	0.8719	0.0001	0	0.0001	No	No
99188_at	2010315L10 Rik	RIKEN cDNA 2010315L10 gene	0.9519	0.2911	0.0041	0.3051	No	0.928	0.166	0.0006	0.0705	No	No
99190_at	Pstk	phosphoseryl-tRNA kinase	1.0025	0.9635	0.1242	0.0555	No	0.9497	0.312	0.655	0.0295	No	No
99191_at	Cri1	CREBBP/EP300 inhibitory protein 1	1.04	0.5559	0.1041	0.7765	No	1.0191	0.68	0.0553	0.557	No	No
99194_at	Pnpt1 /// LOC641190	polyribonucleotide nucleotidyltransferase 1 /// similar to polyribonucleotide nucleotidyltransferase 1	1.0664	0.1425	0.0855	0.1179	No	0.9742	0.692	0.798	0.161	No	No
99195_at	Pcnp /// LOC666005 /// LOC670412 /// LOC674299	PEST proteolytic signal containing nuclear protein /// similar to PEST-containing nuclear protein (PCNP) /// similar to PEST-containing nuclear protein (PCNP) /// similar to PEST-containing nuclear protein (PCNP)	1.0739	0.2184	0.0777	0.1212	No	1.0235	0.976	0.785	0.36	No	No
99196_at	Pcnp	PEST proteolytic signal containing nuclear protein	1.0252	0.7789	0.8443	0.944	No	0.9946	0.951	0.465	0.893	No	No
99197_at	Gc	group specific component	0.9149	0.4529	0.9885	0.3279	No	1.0652	0.128	0.0046	0.69	No	No
99198_at	LOC245405	similar to 60S ribosomal protein L32	0.951	0.2792	0.2453	0.3497	No	1.0588	0.139	0.0158	0.0341	No	No
99236_at	Lcn5	lipocalin 5	0.9976	0.981	0.0962	0.9185	No	1.0349	0.711	0.0135	0.944	No	No
99237_at	---	---	0.9527	0.072	0.4203	0.2092	No	0.6825	0.163	0.867	0.405	No	No
99238_at	Gnb3	guanine nucleotide binding protein, beta 3	0.9952	0.9025	0.9849	0.8034	No	1.0284	0.433	0.442	0.313	No	No
99239_at	Axin2	axin2	1.0164	0.6768	0.2873	0.8401	No	0.9995	0.996	0.782	0.872	No	No
99269_g_at	Tdo2	tryptophan 2,3-dioxygenase	0.9394	0.3062	0.6578	0.9544	No	1.0875	0.248	0.354	0.991	No	No
99320_at	St8sia5	ST8 alpha-N-acetyl-neuraminide alpha-2,8-sialyltransferase 5	1.009	0.8223	0.3332	0.7149	No	1.0953	0.0177	0.0352	0.264	No	No
99321_at	Fert2	fer (fms/fps related) protein kinase, testis specific 2	0.8587	0.0402	0.5193	0.1449	No	0.8566	0.0415	0.0012	0.0385	No	No
99322_at	Kcnq1	potassium voltage-gated channel, subfamily Q, member 1	0.984	0.7521	0.6233	0.3536	No	1.0359	0.541	0.0059	0.219	No	No
99323_at	Il12rb2	interleukin 12 receptor, beta 2	0.9869	0.894	0.3752	0.506	No	0.8984	0.482	0.146	0.677	No	No
99324_at	Prss7	protease, serine, 7 (enterokinase)	1.0534	0.2775	0.3059	0.1531	No	1.2167	0.152	0.26	0.279	No	No
99325_at	Wnt3	wingless-related MMTV integration site 3	1.0611	0.05	0.0182	0.183	No	1.0726	0.122	0.461	0.0187	No	No
99326_at	Pla2g2c	phospholipase A2, group IIC	0.9901	0.7653	0.4854	0.0832	No	0.9869	0.669	0.887	0.0077	No	No
99327_at	Klk8	kallikrein 8	0.9337	0.1071	0.0012	0.0586	No	0.9401	0.104	0.0004	0.0417	No	No
99328_at	Dlx3	distal-less homeobox 3	0.9683	0.2457	0.0027	0.7997	No	0.927	0.0548	0.0007	0.078	No	No
99329_at	Abcc1	ATP-binding cassette, sub-family C (CFTR/MRP), member 1	0.9923	0.9146	0.1113	0.4864	No	0.9869	0.901	0.345	0.126	No	No
99330_at	Csf2ra	colony stimulating factor 2 receptor, alpha, low-affinity (granulocyte-macrophage)	0.9704	0.0108	0.0075	0.0025	No	0.9704	0.536	0.945	0.661	No	No
99331_at	Speg	SPEG complex locus	0.9003	0.1811	0.6147	0.7539	No	0.9016	0.0418	0.0175	0.255	No	No
99332_at	Oprd1	opioid receptor, delta 1	0.9904	0.8841	0.0351	0.9287	No	0.8999	0.148	0.089	0.852	No	No
99333_at	Sele	selectin, endothelial cell	0.9552	0.5891	0.9948	0.8699	No	1.0692	0.357	0.666	0.685	No	No
99334_at	Ifng	interferon gamma	0.9255	0.2119	0.0152	0.2884	No	0.6988	0.216	0.0157	0.424	No	No
99335_at	Hk1	hexokinase 1	1.0116	0.8171	0.3947	0.0826	No	0.9489	0.104	0.395	0.0119	No	No
99336_at	Rps5	ribosomal protein S5	1.0446	0.052	0.0021	0.014	No	1.0111	0.332	0.0004	0.0108	No	No
99337_at	Baiap2	brain-specific angiogenesis inhibitor 1-associated protein 2	0.9622	0.4507	0.4535	0.8851	No	0.9565	0.447	0.22	0.0935	No	No
99338_at	Cand1	cullin associated and neddylation disassociated 1	0.9547	0.2651	0.2307	0.6134	No	1.0515	0.498	0.0319	0.918	No	No
99339_r_at	Kcnd2	potassium voltage-gated channel, Shal-related family, member 2	0.9936	0.772	0.0012	0.712	No	0.9263	0.0736	0.0008	0.0208	No	No
99340_at	Gnb2l1	guanine nucleotide binding protein (G protein), beta polypeptide 2 like 1	1.0149	0.5897	0.0004	0.5156	No	1.0191	0.442	0.0004	0.902	No	No

99341_r_at	9130016M20 Rik	RIKEN cDNA 9130016M20 gene	1.0463	0.0259	0.0147	0.0025	No	1.2532	0.286	0.618	0.0077	No	No
99342_at	Gabrd	gamma-aminobutyric acid (GABA-A) receptor, subunit delta	1.0185	0.7619	0.219	0.0944	No	0.9604	0.307	0.0032	0.0941	No	No
99343_at	4932441K18 Rik	RIKEN cDNA 4932441K18 gene	1.0705	0.379	0.3865	0.2292	No	0.9382	0.551	0.0566	0.377	No	No
99344_at	Ap1s2	Adaptor-related protein complex 1, sigma 2 subunit	0.9734	0.3615	0.1451	0.0834	No	0.9376	0.496	0.806	0.74	No	No
99345_at	Mphosph9	M-phase phosphoprotein 9	1.0143	0.7196	0.0019	0.0484	No	1.0316	0.291	0.0031	0.0799	No	No
99346_at	B4galt3	UDP-Gal:betaGlcNAc beta 1,4-galactosyltransferase, polypeptide 3	0.916	0.1296	0.0021	0.3362	No	0.96	0.716	0.0043	0.677	No	No
99347_f_at	Eml5	echinoderm microtubule associated protein like 5	1.0188	0.7975	0.0238	0.1675	No	1.0357	0.356	0.0064	0.0732	No	No
99348_r_at	Pfkfb3	6-phosphofructo-2-kinase/fructose-2,6-biphosphatase 3	1.0823	0.0753	0.0014	0.6534	No	1.0546	0.152	0.737	0.369	No	No
99349_at	Il17a	interleukin 17A	0.978	0.5099	0.0847	0.9985	No	0.9393	0.391	0.851	0.528	No	No
99350_at	Sec63	SEC63-like (S. cerevisiae)	1.002	0.9615	0.9267	0.8038	No	0.9578	0.408	0.353	0.293	No	No
99351_at	C76132	expressed sequence C76132	1.0307	0.3812	0.0187	0.0319	No	1.0833	0.0566	0.003	0.0249	No	No
99354_s_at	---	---	0.9572	0.3981	0.0282	0.7722	No	0.9489	0.59	0.129	0.773	No	No
99355_r_at	C76554	expressed sequence C76554	0.9974	0.832	0.9253	0.0018	No	0.9406	0.725	0.342	0.614	No	No
99356_r_at	Tnpo1 /// LOC634263	transportin 1 /// similar to transportin 1	1.0836	0.2489	0.0187	0.3977	No	1.1265	0.0173	0.0046	0.0296	No	No
99357_at	C76614	expressed sequence C76614	1.0378	0.4875	0.7963	0.0962	No	1.1747	0.143	0.914	0.076	No	No
99358_at	D15Ertd30e	DNA segment, Chr 15, ERATO Doi 30, expressed	1.0065	0.9069	0.1966	0.1489	No	0.9884	0.796	0.0338	0.365	No	No
99359_at	D9Ertd26e	DNA segment, Chr 9, ERATO Doi 26, expressed	0.877	0.0059	0.0068	0.011	Yes	0.9654	0.288	0.0051	0.361	No	No
99360_at	Serac1	serine active site containing 1	1.0755	0.2518	0.0162	0.4637	No	1.1431	0.131	0.24	0.0608	No	No
99361_at	Wnt8a	wingless-related MMTV integration site 8A	0.9778	0.7641	0.0623	0.6559	No	0.9812	0.66	0.0416	0.147	No	No
99362_at	C77144	expressed sequence C77144	1.0037	0.9357	0.9261	0.8948	No	1.0451	0.257	0.0313	0.154	No	No
99363_at	D6Ertd47e	DNA segment, Chr 6, ERATO Doi 47, expressed	1.0356	0.2125	0.3636	0.1514	No	0.9427	0.515	0.0716	0.28	No	No
99364_at	Rbbp6	retinoblastoma binding protein 6	0.9699	0.8458	0.0816	0.9797	No	1.1271	0.178	0.769	0.394	No	No
99365_at	Coq3	coenzyme Q3 homolog, methyltransferase (yeast)	0.962	0.3311	0.9767	0.0624	No	0.9446	0.227	0.054	0.972	No	No
99366_at	Pqlc3	PQ loop repeat containing	0.9423	0.3489	0.2136	0.0877	No	0.9729	0.533	0.123	0.157	No	No
99367_at	Mapre1	microtubule-associated protein, RP/EB family, member 1	0.9743	0.6097	0.0922	0.0441	No	0.9283	0.521	0.242	0.0865	No	No
99368_at	Agtrl1	angiotensin receptor-like 1	1.064	0.0372	0.1035	0.8261	No	1.0458	0.364	0.222	0.134	No	No
99369_f_at	Igkv10-95	immunoglobulin kappa chain variable 10-95	1.0335	0.5839	0.073	0.6866	No	1.0976	0.381	0.0385	0.613	No	No
99370_at	Klrc1	killer cell lectin-like receptor subfamily C, member 1	0.9971	0.9733	0.7039	0.3266	No	1.1417	0.0467	0.423	0.0617	No	No
99371_at	Kcnd3	potassium voltage-gated channel, Shal-related family, member 3	0.9851	0.7494	0.1272	0.7221	No	0.9525	0.0485	0.63	0.57	No	No
99372_at	Edg5	endothelial differentiation, sphingolipid G-protein-coupled receptor, 5	1.0024	0.9111	0.1037	0.5713	No	1.0601	0	0.0002	0.0006	No	No
99373_at	Gjb4	gap junction membrane channel protein beta 4	0.9699	0.7209	0.1935	0.3621	No	0.9637	0.453	0.0132	0.478	No	No
99374_at	Epo	erythropoietin	0.9333	0.2288	0.2245	0.7717	No	0.9022	0.107	0.0428	0.238	No	No
99375_at	Sec1	secretory blood group 1	0.9855	0.7378	0.532	0.8241	No	1.0127	0.712	0.017	0.234	No	No
99376_at	Kcnj5	potassium inwardly-rectifying channel, subfamily J, member 5	0.9043	0.1316	0.0642	0.887	No	0.9048	0.0099	0.167	0.0839	Yes	No
99377_at	Obp1a	odorant binding protein Ia	1.0164	0.8585	0.9245	0.6646	No	1.0952	0.24	0.0895	0.0514	No	No
99378_f_at	H2-Q1	histocompatibility 2, Q region locus 1	0.9452	0.2618	0.0345	0.0109	No	0.966	0.519	0.0178	0.176	No	No
99379_f_at	LOC676689	similar to H-2 class I histocompatibility antigen, L-D alpha chain precursor	0.991	0.7809	0.0028	0.5812	No	1.0247	0.68	0.0266	0.158	No	No
99380_at	Kcna2	potassium voltage-gated channel, shaker-related subfamily, member 2	0.9714	0.327	0.5178	0.4731	No	0.95	0.0467	0.0074	0.0068	No	No
99381_at	Fut1	fucosyltransferase 1	0.9787	0.5551	0.0061	0.1552	No	0.9877	0.485	0.0009	0.101	No	No
99382_at	Grin2d	glutamate receptor, ionotropic, NMDA2D (epsilon 4)	0.9951	0.903	0.0329	0.5365	No	0.9542	0.351	0.218	0.08	No	No
99383_at	---	---	1.0011	0.9862	0.0663	0.8188	No	0.923	0.246	0.636	0.644	No	No
99384_at	Pim1	proviral integration site 1	1.0662	0.0032	0.0005	0.0018	No	1.0161	0.67	0.569	0.317	No	No
99385_at	Pou3f2	POU domain, class 3, transcription factor 2	1.0603	0.1776	0.0819	0.1696	No	1.0269	0.372	0.0448	0.0603	No	No
99386_at	Pou3f4	POU domain, class 3, transcription factor 4	1.134	0.0116	0.0001	0.1712	No	1.0857	0.058	0.0001	0.644	No	No
99387_at	Fpr1	formyl peptide receptor 1	1.0404	0.5849	0.6534	0.8783	No	1.0132	0.815	0.707	0.576	No	No
99388_at	Src	Rous sarcoma oncogene	0.986	0.6412	0.0096	0.0717	No	0.934	0.0708	0.0002	0.0046	No	No
99389_at	Tcrb-V13 /// LOC545835	T-cell receptor beta, variable 13 /// similar to T-cell receptor beta chain V region CTL-L17 precursor	1.0191	0.7949	0.0441	0.0912	No	1.0183	0.613	0.0011	0.509	No	No
99390_at	Wnt5a	wingless-related MMTV integration site 5A	1.1673	0.1106	0.0002	0.0849	No	1.0828	0.362	0.0001	0.0253	No	No
99391_at	Evi1	ecotropic viral integration site 1	0.9257	0.1423	0.006	0.4406	No	0.9406	0.22	0.009	0.377	No	No
99392_at	Tnfaip3	tumor necrosis factor, alpha-induced protein 3	1.0127	0.8011	0.0726	0.2171	No	1.0843	0.016	0.555	0.152	No	No
99393_at	Bmp5	bone morphogenetic protein 5	1.0418	0.2626	0.1705	0.2537	No	1.0265	0.303	0.109	0.886	No	No
99394_at	Has3	hyaluronan synthase 3	0.9729	0.6292	0.0826	0.9762	No	1.074	0.192	0.0048	0.0786	No	No
99395_at	Opn1sw	opsin 1 (cone pigments), short-wave-sensitive (color blindness, tritan)	1.0082	0.8826	0.3971	0.1057	No	1.0713	0.369	0.314	0.137	No	No
99396_at	Igkv9-120	Immunoglobulin kappa light chain	0.9815	0.5941	0.194	0.0875	No	1.0622	0.4	0.808	0.0386	No	No
99397_at	Ercc2	excision repair cross-complementing rodent repair deficiency, complementation group 2	1.0316	0.4027	0.0316	0.9318	No	1.122	0.0674	0.0091	0.241	No	No
99398_at	Gzmk	granzyme K	1.0229	0.7991	0.1031	0.4488	No	1.0161	0.893	0.337	0.15	No	No
99399_at	Agrp	agouti related protein	0.958	0.1987	0.2519	0.9152	No	1.0524	0.0402	0.0049	0.0015	No	No
99400_at	BC048599	cDNA sequence BC048599	0.9705	0.7105	0.1071	0.8809	No	1.0431	0.836	0.336	0.305	No	No
99401_at	Uhmk1 /// LOC677213	U2AF homology motif (UHM) kinase 1 /// similar to U2AF homology motif (UHM) kinase 1	1.0457	0.4579	0.1696	0.7953	No	1.1727	0.0433	0.172	0.993	No	No
99402_at	Art2b	ADP-ribosyltransferase 2b	1.0233	0.7577	0.5864	0.3868	No	0.9959	0.925	0.956	0.623	No	No
99403_at	Art2a	ADP-ribosyltransferase 2a	0.9415	0.2565	0.1426	0.5538	No	0.9246	0.213	0.0113	0.377	No	No
99404_at	Cyp7a1	cytochrome P450, family 7, subfamily a, polypeptide 1	0.9921	0.8772	0.2431	0.1438	No	1.0171	0.785	0.0542	0.695	No	No
99405_at	Igkv1-135 /// Igkv1-133 /// LOC635601	immunoglobulin kappa chain variable 1-135 /// immunoglobulin kappa chain variable 1-133 /// similar to Ig kappa chain V-II region RPMI 6410 precursor	1.0011	0.9871	0.0768	0.9899	No	0.9169	0.0251	0.0001	0.0717	No	No
99406_at	Fut4	fucosyltransferase 4	1.0262	0.6564	0.0135	0.3152	No	0.9955	0.759	0.0114	0.0713	No	No
99407_at	Omp	olfactory marker protein	0.9319	0.2025	0.0539	0.3976	No	0.982	0.698	0.564	0.562	No	No
99408_at	Slc6a2	solute carrier family 6 (neurotransmitter transporter, noradrenalin), member 2	1.0261	0.2509	0.0058	0.5326	No	0.7881	0.0146	0.0038	0.796	No	No
99409_at	Slc6a2	solute carrier family 6 (neurotransmitter transporter, noradrenalin), member 2	0.9856	0.6525	0.7767	0.4255	No	0.9611	0.342	0.0175	0.902	No	No
99410_at	Kcnu1	potassium channel, subfamily U, member 1	0.9866	0.6073	0.0017	0.6542	No	0.9993	0.98	0.0207	0.81	No	No
99411_at	Htr1b	5-hydroxytryptamine (serotonin) receptor 1B	1.0283	0.5618	0.1679	0.0699	No	1.1173	0.0717	0.323	0.0511	No	No
99412_at	Ccr3	chemokine (C-C motif) receptor 3	0.9509	0.416	0.6253	0.649	No	1.0929	0.0321	0.0002	0.89	No	No
99413_at	Ccr1	chemokine (C-C motif) receptor 1	1.0036	0.9582	0.3681	0.6956	No	1.0101	0.963	0.813	0.379	No	No
99414_at	Rhpn1	rhophilin, Rho GTPase binding protein 1	0.9254	0.2965	0.0682	0.987	No	0.9679	0.631	0.0214	0.845	No	No
99415_at	Fzd8	frizzled homolog 8 (Drosophila)	1.0202	0.6296	0.0133	0.7149	No	1.0146	0.596	0.0087	0.17	No	No
99416_at	Osbpl9	Oxysterol binding protein-like 9	0.8912	0.2799	0.4135	0.6453	No	0.919	0.327	0.461	0.991	No	No
99417_at	Nmur1	neuromedin U receptor 1	0.9613	0.3723	0.0381	0.798	No	1.0209	0.678	0.0078	0.349	No	No
99418_at	Bcl2l11	BCL2-like 11 (apoptosis facilitator)	0.9629	0.4819	0.5651	0.6357	No	1.1081	0.129	0.0239	0.0277	No	No
99419_g_at	Bcl2l11	BCL2-like 11 (apoptosis facilitator)	0.9882	0.8624	0.0618	0.432	No	1.052	0.341	0.0009	0.967	No	No
99420_at	Igh-1a	Immunoglobulin heavy chain 1a (serum IgG2a)	0.9823	0.6316	0.1026	0.8069	No	1.0055	0.895	0.495	0.672	No	No

99421_at	Igh-1a /// LOC544900 /// LOC630195 /// LOC630242 /// LOC630256 /// LOC630282 /// LOC630317 /// LOC638605 /// LOC640046 /// LOC641221 /// LOC668389 /// LOC673364 /// LOC674075 /// LOC674094 ///	immunoglobulin heavy chain 1a (serum IgG2a) /// similar to Ig heavy chain V region PJ14 precursor /// similar to Ig heavy chain V region MC101 precursor /// similar to Ig heavy chain V region MC101 precursor /// similar to Ig heavy chain V region PJ14 precursor /// similar to Ig heavy chain V region MC101 precursor /// similar to Ig heavy chain V region MC101 precursor /// similar to Ig heavy chain V region PJ14 precursor /// similar to Ig heavy chain V region PJ14 precursor /// similar to Ig heavy chain V region MC101 precursor /// similar to Ig heavy chain V region MC101 precursor /// similar to Ig heavy chain V region PJ14 precursor /// similar to Ig heavy chain V region MC101 precursor /// similar to Ig heavy chain V region MC101 precursor /// similar to Ig heavy chain V region MC101 precursor /// similar to Ig heavy chain V region MC101 precursor /// similar to Ig heavy chain V region PJ14 precursor	0.9808	0.7215	0.6192	0.9747	No	1.0713	0.0708	0.0052	0.019	No	No
99422_at	1600014C10 Rik	RIKEN cDNA 1600014C10 gene	1.0352	0.5046	0.107	0.3795	No	0.9305	0.446	0.0098	0.521	No	No
99423_at	Mgat3	mannoside acetylglucosaminyltransferase 3	0.9859	0.8375	0.049	0.9711	No	0.9903	0.799	0.0038	0.861	No	No
99424_at	Fgf14	fibroblast growth factor 14	1.0296	0.2363	0.0006	0.1135	No	1.0319	0.476	0.0009	0.205	No	No
99425_at	Hmgcr	3-hydroxy-3-methylglutaryl-Coenzyme A reductase	0.9572	0.4735	0.3177	0.9305	No	0.8625	0.175	0.814	0.0819	No	No
99426_at	Hoxd9	homeo box D9	0.9264	0.2206	0.1304	0.952	No	1.0093	0.848	0.0795	0.298	No	No
99427_at	Hoxd12	homeo box D12	0.9148	0.1104	0.469	0.9966	No	0.9307	0.498	0.113	0.915	No	No
99428_at	Igll1	immunoglobulin lambda-like polypeptide 1	1.0285	0.6691	0.0728	0.1055	No	1.0689	0.0679	0.0148	0.107	No	No
99429_at	Igll1	immunoglobulin lambda-like polypeptide 1	0.9163	0.3558	0.2843	0.4213	No	1.0056	0.911	0.0024	0.213	No	No
99430_at	Galr2	galanin receptor 2	0.9297	0.1257	0.054	0.692	No	1.0507	0.241	0.0591	0.0856	No	No
99431_at	Soat2	sterol O-acyltransferase 2	1.0598	0.3684	0.4244	0.6101	No	1.1098	0.0023	0.455	0.028	Yes	No
99432_at	Cyln2	cytoplasmic linker 2	0.9598	0.2364	0.0025	0.4673	No	1.0259	0.643	0.0034	0.0566	No	No
99433_at	Pstpip2	proline-serine-threonine phosphatase-interacting protein 2	0.806	0.0378	0.0747	0.1673	No	0.9051	0.0498	0.336	0.46	No	No
99434_at	Cd83	CD83 antigen	1.0525	0.1064	0.6904	0.6673	No	1.0794	0.129	0.171	0.867	No	No
99435_at	Fgf7	fibroblast growth factor 7	0.8972	0.0414	0.0028	0.0544	No	0.9661	0.594	0.005	0.352	No	No
99436_at	Spn	sialophorin	0.9817	0.775	0.0468	0.35	No	1.0763	0.242	0.219	0.679	No	No
99437_at	Ly6f	lymphocyte antigen 6 complex, locus F	0.9848	0.4288	0.1838	0.6305	No	1.0563	0.354	0.0193	0.0704	No	No
99438_at	C78549	expressed sequence C78549	1.0369	0.4212	0.4615	0.3937	No	1.064	0.34	0.412	0.0672	No	No
99439_at	Mas1	MAS1 oncogene	0.9773	0.7009	0.4822	0.6624	No	0.9854	0.812	0.96	0.593	No	No
99440_at	Nfib	nuclear factor I/B	0.8685	0.0184	0.0009	0.1863	No	0.8814	0.0297	0.0007	0.0407	No	No
99441_at	Gphn	gephyrin	1.0329	0.6129	0.0205	0.0765	No	1.0112	0.57	0.0051	0.0514	No	No
99442_at	Resp18	regulated endocrine-specific protein 18	0.9259	0.232	0.0262	0.2498	No	0.9391	0.0296	0.0922	0.0116	No	No
99444_at	Ramp2	receptor (calcitonin) activity modifying protein 2	0.9025	0.0216	0.0004	0.2434	No	0.9103	0.0094	0.0003	0.435	Yes	Yes
99445_at	Slc44a2	solute carrier family 44, member 2	0.9856	0.7607	0.0417	0.6373	No	0.9367	0.427	0.0047	0.681	No	No
99446_at	Ms4a1	membrane-spanning 4-domains, subfamily A, member 1	1.0406	0.3815	0.0759	0.0218	No	1.0808	0.315	0.0231	0.139	No	No
99447_at	1110007L15 Rik	RIKEN cDNA 1110007L15 gene	1.0004	0.9936	0.1727	0.465	No	0.9997	0.955	0.803	0.898	No	No
99448_at	Tln1	talin 1	0.9476	0.3326	0.1418	0.3468	No	0.9619	0.236	0.198	0.75	No	No
99449_at	Kcnq2	potassium voltage-gated channel, subfamily Q, member 2	0.971	0.4718	0.1283	0.9492	No	0.9555	0.306	0.0567	0.984	No	No
99450_at	Kcnq2	potassium voltage-gated channel, subfamily Q, member 2	1.0244	0.73	0.7955	0.6405	No	1.0194	0.802	0.577	0.826	No	No
99451_at	C230093N12Rik	RIKEN cDNA C230093N12 gene	0.9318	0.1633	0.016	0.357	No	1.0165	0.702	0.916	0.218	No	No
99452_at	Lsr	lipolysis stimulated lipoprotein receptor	0.9752	0.5537	0.0083	0.0938	No	1.0393	0.0462	0.0062	0.286	No	No
99453_at	Pde1a	phosphodiesterase 1A, calmodulin-dependent	1.0048	0.8894	0.427	0.1659	No	0.9381	0.12	0.0124	0.0099	No	No
99455_s_at	Acrbp	proacrosin binding protein	0.926	0.0906	0.4016	0.6158	No	1.1833	0.0093	0.0015	0.0051	No	No
99456_at	Acrbp	proacrosin binding protein	0.9446	0.4153	0.9183	0.3101	No	0.9296	0.522	0.587	0.864	No	No
99457_at	Mki67 /// LOC638774	antigen identified by monoclonal antibody Ki 67 /// similar to Antigen KI-67	1.0436	0.2585	0.0001	0.1154	No	1.0505	0.336	0.0041	0.156	No	No
99458_i_at	Mark2	MAP/microtubule affinity-regulating kinase 2	0.957	0.6256	0.3009	0.1257	No	1.013	0.874	0.852	0.348	No	No
99459_f_at	Mark2	MAP/microtubule affinity-regulating kinase 2	0.9509	0.5872	0.0013	0.1667	No	0.9123	0.325	0.0012	0.256	No	No
99460_at	Sema3b	sema domain, immunoglobulin domain (Ig), short basic domain, secreted, (semaphorin) 3B	0.8891	0.0282	0.2499	0.0224	No	1.0197	0.774	0.685	0.402	No	No
99461_at	Hcls1	hematopoietic cell specific Lyn substrate 1	1.0245	0.6709	0.8868	0.7128	No	0.9763	0.642	0.0249	0.913	No	No
99462_at	Top2b	topoisomerase (DNA) II beta	1.0805	0.5966	0.0276	0.6035	No	1.0895	0.52	0.001	0.832	No	No
99463_at	Cyp3a13	cytochrome P450, family 3, subfamily a, polypeptide 13	1.0688	0.2751	0.7704	0.742	No	1.1503	0.0151	0.363	0.752	No	No
99464_at	Rxrip110	retinoid X receptor interacting protein 110	1.0047	0.8379	0.8428	0.1238	No	0.9961	0.998	0.174	0.148	No	No
99465_at	Mecp2	methyl CpG binding protein 2	0.9763	0.4654	0.0017	0.8766	No	0.9343	0.136	0.0016	0.872	No	No
99466_at	Zfp444	zinc finger protein 444	1.0173	0.33	0	0.0101	No	1.0348	0.181	0.0011	0.75	No	No
99467_at	Rasa1	RAS p21 protein activator 1	1.0184	0.7814	0.0012	0.2346	No	1.0135	0.797	0.0044	0.893	No	No
99469_at	Pex6	peroxisomal biogenesis factor 6	0.9062	0.0069	0.0002	0.0053	No	0.9128	0.0485	0.145	0.0634	No	No
99471_at	Stard7	START domain containing 7	1.0046	0.8947	0.5932	0.9768	No	0.9954	0.931	0.137	0.68	No	No
99472_at	Chmp4b	Chromatin modifying protein 4B	0.9737	0.4373	0.0071	0.2252	No	1.0798	0.0363	0.488	0.0195	No	No
99473_at	Chmp4b	Chromatin modifying protein 4B	1.0507	0.4058	0.0105	0.2743	No	1.0012	0.949	0.0077	0.282	No	No
99474_at	Adam5	a disintegrin and metallopeptidase domain 5	0.9686	0.4535	0.8468	0.1779	No	1.2107	0.078	0.292	0.0156	No	No
99475_at	Socs2	suppressor of cytokine signaling 2	0.9818	0.6462	0.7968	0.0985	No	1.0276	0.489	0.236	0.24	No	No
99476_at	Col14a1	procollagen, type XIV, alpha 1	1.0273	0.6945	0.0055	0.5363	No	1.0077	0.888	0.004	0.44	No	No
99477_at	Gng12	guanine nucleotide binding protein (G protein), gamma 12	1.0853	0.1441	0.0572	0.0847	No	1.0333	0.46	0.0499	0.503	No	No
99478_at	Ptprcap	protein tyrosine phosphatase, receptor type, C polypeptide-associated protein	1.0658	0.3716	0.269	0.2888	No	1.0268	0.631	0.114	0.314	No	No
99479_at	Dmbt1	deleted in malignant brain tumors 1	0.9955	0.8644	0.1915	0.4431	No	1.1904	0.0326	0.0702	0.859	No	No
99481_at	Atp1a2	ATPase, Na+/K+ transporting, alpha 2 polypeptide	0.8191	0.0049	0.2063	0.0732	Yes	0.8868	0.113	0.826	0.601	No	No
99485_at	Dffa	DNA fragmentation factor, alpha subunit	0.9361	0.3559	0.136	0.8172	No	0.9759	0.749	0.0346	0.913	No	No
99486_at	Cenpb /// Spef1	centromere autoantigen B /// sperm flagellar 1	0.9658	0.3983	0.016	0.0981	No	0.9898	0.927	0.0516	0.218	No	No
99488_at	Iapp	islet amyloid polypeptide	0.963	0.5738	0.0331	0.3146	No	1.0581	0.499	0.09	0.875	No	No
99489_at	Hspa4l	heat shock protein 4 like	0.9836	0.2236	0.0118	0.8522	No	0.9943	0.855	0.848	0.748	No	No
99490_at	Thoc3	THO complex 3	1.1523	0.018	0.0016	0.7453	No	1.0802	0.0486	0.027	0.141	No	No
99491_at	Il10rb	interleukin 10 receptor, beta	0.9728	0.4273	0.0003	0.0198	No	0.994	0.202	0	0.0027	No	No
99492_at	Ctnnbip1	catenin beta interacting protein 1	1.0318	0.1942	0.0072	0.0141	No	1.0536	0.0625	0.0018	0.0111	No	No
99493_at	Rab28	RAB28, member RAS oncogene family	1.0351	0.6132	0.9789	0.9544	No	0.9425	0.354	0.708	0.678	No	No
99494_at	Serpini1	serine (or cysteine) peptidase inhibitor, clade I, member 1	0.9554	0.3668	0.0011	0.9673	No	0.9076	0.46	0.0039	0.731	No	No
99497_at	Arfip2	ADP-ribosylation factor interacting protein 2	1.0963	0.1132	0.0122	0.985	No	1.1309	0.0583	0.0037	0.79	No	No
99498_at	Glul /// LOC671629	glutamate-ammonia ligase (glutamine synthetase) /// hypothetical protein LOC671629	0.8037	0.0088	0.069	0.3849	Yes	0.6345	0.0031	0.027	0.0107	Yes	Yes
99499_at	Mettl1	methyltransferase-like 1	1.0094	0.8247	0.8614	0.657	No	1.0006	0.996	0.138	0.839	No	No
99500_at	Slc12a2	solute carrier family 12, member 2	1.0602	0.2738	0.0002	0.1214	No	1.0171	0.668	0.0005	0.611	No	No
99501_at	Tloc1	translocation protein 1	0.9584	0.4931	0.0792	0.5872	No	0.9683	0.692	0.405	0.811	No	No
99502_at	Zfp148	zinc finger protein 148	1.01	0.8873	0.0038	0.0825	No	0.9791	0.681	0.09	0.224	No	No
99503_at	Pptc7	PTC7 protein phosphatase homolog (S. cerevisiae)	0.9804	0.2087	0	0.0721	No	0.9837	0.671	0.0026	0.445	No	No
99504_at	St8sia3	ST8 alpha-N-acetyl-neuraminide alpha-2,8-sialyltransferase 3	0.913	0.1022	0.8541	0.2971	No	1.0621	0.164	0.86	0.111	No	No
99505_at	Rab4b	RAB4B, member RAS oncogene family	0.9861	0.6258	0.0171	0.1337	No	1.0765	0.0163	0.001	0.637	No	No
99506_at	Dapk2	death-associated kinase 2	1.0244	0.3478	0.1226	0.8131	No	0.9572	0.065	0.0005	0.288	No	No
99507_at	Ucp1	uncoupling protein 1 (mitochondrial, proton carrier)	2.3906	0.0002	0.0007	0.0189	Yes	1.904	0	0.0001	0.0154	Yes	Yes
99508_at	Fxyd7	FXYD domain-containing ion transport regulator 7	0.9854	0.7273	0.2837	0.9488	No	1.0303	0.528	0.608	0.613	No	No
99509_s_at	Jak3	Janus kinase 3	1.0113	0.5446	0.0095	0.3055	No	0.9548	0.21	0.0086	0.987	No	No
99510_at	Prkcb1	protein kinase C, beta 1	1.0405	0.253	0.0381	0.5405	No	1.0601	0.185	0.0038	0.877	No	No

99511_at	Prkcb1	protein kinase C, beta 1	1.0497	0.3557	0.0489	0.9636	No	1.1286	0.272	0.0942	0.247	No	No
99512_at	Cnot1 /// LOC620499 /// LOC665105	CCR4-NOT transcription complex, subunit 1 /// similar to CCR4-NOT transcription complex, subunit 1 isoform a /// similar to CCR4-NOT transcription complex, subunit 1 isoform a	0.9607	0.0508	0.0009	0.6687	No	0.9874	0.733	0.109	0.723	No	No
99513_at	Pla2g4a	phospholipase A2, group IVA (cytosolic, calcium-dependent)	1.1167	0.0571	0.8947	0.494	No	1.1103	0.147	0.379	0.506	No	No
99514_at	Bmyc	brain expressed myelocytomatosis oncogene	0.9845	0.4899	0.0004	0.0893	No	0.9058	0.146	0.508	0.0601	No	No
99515_at	Ap2b1	adaptor-related protein complex 2, beta 1 subunit	1.0584	0.3887	0.2226	0.6403	No	1.0198	0.747	0.209	0.136	No	No
99516_at	Fxr2h	fragile X mental retardation gene 2, autosomal homolog	0.9708	0.278	0.0001	0.8453	No	1.1154	0.0253	0.0002	0.27	No	No
99517_at	Mfap5	microfibrillar associated protein 5	1.0283	0.5838	0.1671	0.1183	No	0.9036	0.0782	0.188	0.656	No	No
99518_at	Mfap5	microfibrillar associated protein 5	0.8216	0.0386	0.0439	0.0425	No	0.8387	0.0215	0.0484	0.0121	No	No
99521_at	Ak3l1	adenylate kinase 3 alpha-like 1	1.022	0.489	0.0017	0.1189	No	1.0256	0.569	0.0018	0.875	No	No
99522_at	Gsg2	germ cell-specific gene 2	0.9771	0.6653	0.5086	0.1044	No	1.0357	0.51	0.987	0.595	No	No
99523_at	Ranbp10	RAN binding protein 10	0.9858	0.842	0.7368	0.9239	No	0.9777	0.708	0.906	0.872	No	No
99524_at	Slc8a1	solute carrier family 8 (sodium/calcium exchanger), member 1	1.0933	0.2499	0.0069	0.2355	No	1.0866	0.223	0.0221	0.243	No	No
99525_at	Slc8a1	solute carrier family 8 (sodium/calcium exchanger), member 1	1.0225	0.6839	0.8813	0.5177	No	1.0692	0.414	0.952	0.298	No	No
99527_at	Nfe2l3	nuclear factor, erythroid derived 2, like 3	0.9531	0.3556	0.1073	0.8062	No	0.8569	0.246	0.318	0.879	No	No
99528_at	Spin	spindlin	1.1164	0.1087	0.0216	0.1064	No	1.0906	0.145	0.772	0.657	No	No
99529_f_at	Rnf138	ring finger protein 138	1.1785	0.1681	0.8911	0.678	No	1.1771	0.0755	0.862	0.942	No	No
99530_at	Prlpc1	prolactin-like protein C 1	0.9362	0.1056	0.2479	0.2207	No	0.9164	0.0532	0.007	0.459	No	No
99531_at	Syngr4	synaptogyrin 4	0.9893	0.6651	0.6432	0.8712	No	1.0176	0.625	0.632	0.196	No	No
99532_at	Tob1	transducer of ErbB-2.1	0.9336	0.0926	0.0001	0.1986	No	0.9648	0.635	0.0011	0.98	No	No
99534_at	Ghrl	ghrelin	1.0012	0.9676	0.5488	0.9864	No	1.0208	0.722	0.606	0.744	No	No
99535_at	Ccrn4l	CCR4 carbon catabolite repression 4-like (S. cerevisiae)	1.0717	0.1189	0.001	0.4511	No	1.0437	0.523	0.0053	0.904	No	No
99536_at	Cib2	calcium and integrin binding family member 2	1.0476	0.2599	0.7124	0.8022	No	0.8954	0.134	0.0679	0.0124	No	No
99537_at	Ruvbl1	RuvB-like protein 1	1.0396	0.0617	0	0.0129	No	1.0508	0.0344	0.0002	0.0711	No	No
99541_at	Kif11	kinesin family member 11	0.9	0.0586	0.0343	0.7714	No	0.9258	0.162	0.0254	0.675	No	No
99542_at	Pdha2	pyruvate dehydrogenase E1 alpha 2	0.9537	0.6384	0.8012	0.6123	No	1.0171	0.889	0.259	0.722	No	No
99543_s_at	Dguok	deoxyguanosine kinase	0.9694	0.3867	0.0335	0.0737	No	0.9757	0.467	0.0729	0.526	No	No
99544_at	Dguok	deoxyguanosine kinase	0.9204	0.0685	0.0072	0.2376	No	1.0363	0.0037	0	0.001	No	No
99545_at	Tekt1	tektin 1	0.8743	0.2563	0.4741	0.5774	No	1.0441	0.57	0.822	0.475	No	No
99546_at	Fkbp2	FK506 binding protein 2	1.0992	0.0434	0.0054	0.6385	No	1.1295	0.052	0.0112	0.801	No	No
99548_at	Aldh3a1	aldehyde dehydrogenase family 3, subfamily A1	0.9452	0.2631	0.6931	0.6336	No	0.9875	0.776	0.678	0.117	No	No
99549_at	Ogn	osteoglycin	1.0177	0.8171	0.1203	0.8597	No	0.975	0.698	0.778	0.962	No	No
99551_f_at	Defcr5	defensin related cryptdin 5	0.9854	0.6827	0.3848	0.8828	No	1.0451	0.0584	0.94	0.143	No	No
99552_at	Snai2	snail homolog 2 (Drosophila)	0.886	0.0497	0.1561	0.518	No	0.8976	0.0065	0.109	0.0628	Yes	Yes
99553_f_at	Rgs8	regulator of G-protein signaling 8	0.9131	0.3695	0.8623	0.3834	No	0.9637	0.598	0.127	0.935	No	No
99555_at	AA522020	expressed sequence AA522020	1.0044	0.8915	0.0675	0.9499	No	1.011	0.82	0.172	0.312	No	No
99556_s_at	Sry	sex determining region of Chr Y	1.0508	0.6354	0.2388	0.944	No	0.9963	0.946	0.407	0.635	No	No
99557_at	Nelf	nasal embryonic LHRH factor	0.97	0.6578	0.0317	0.0947	No	0.9344	0.0289	0	0.0673	No	No
99558_at	Ccnc	cyclin C	0.9834	0.7395	0.0272	0.496	No	0.9961	0.992	0.0292	0.836	No	No
99559_at	Aldh3a2	aldehyde dehydrogenase family 3, subfamily A2	0.9467	0.1649	0.6267	0.5668	No	1.0813	0.129	0.0082	0.155	No	No
99561_f_at	Cldn7	claudin 7	1.0238	0.5522	0.2813	0.2487	No	1.0372	0.343	0.0189	0.608	No	No
99562_at	Man2b1	mannosidase 2, alpha B1	0.9183	0.0671	0.2247	0.3463	No	0.9381	0.161	0.0808	0.449	No	No
99563_at	Spin	spindlin	0.986	0.7227	0.3936	0.0842	No	0.9953	0.774	0.0169	0.0612	No	No
99564_at	Uhrf1	ubiquitin-like, containing PHD and RING finger domains, 1	1.0738	0.0313	0.0089	0.2839	No	1.0657	0.323	0.232	0.309	No	No
99566_at	Tpi1	triosephosphate isomerase 1	1.0388	0.3068	0.0027	0.1374	No	1.0573	0.234	0.0039	0.261	No	No
99567_at	Sf1	splicing factor 1	0.9475	0.4478	0.7899	0.6686	No	1.0205	0.665	0.0182	0.347	No	No
99569_at	Krt2-18	keratin complex 2, basic, gene 18	1.1126	0.1506	0.8165	0.4808	No	1.0194	0.732	0.0692	0.376	No	No
99570_s_at	Atp2a2	ATPase, Ca++ transporting, cardiac muscle, slow twitch 2	0.8777	0.1466	0.0115	0.3166	No	0.8948	0.0034	0.0001	0.13	Yes	No
99571_at	Acaa1b	acetyl-Coenzyme A acyltransferase 1B	1.0156	0.6882	0.0016	0.1772	No	1.0295	0.278	0.0004	0.0755	No	No
99574_at	Znrf2	zinc and ring finger 2	1.047	0.2963	0.0013	0.6694	No	1.0461	0.35	0.016	0.133	No	No
99575_at	Ubqln1	ubiquilin 1	1.0941	0.2113	0.0211	0.7219	No	0.9942	0.95	0.0191	0.823	No	No
99576_at	Mgst3	microsomal glutathione S-transferase 3	0.9552	0.0762	0.0273	0.2772	No	0.8577	0.182	0.967	0.164	No	No
99577_at	Kitl	kit ligand	1.1265	0.1879	0.0147	0.1585	No	1.0814	0.197	0.0738	0.116	No	No
99578_at	Top2a	topoisomerase (DNA) II alpha	1.0087	0.7495	0.5662	0.0515	No	1.0392	0.231	0.213	0.0208	No	No
99579_at	Atp1b3	ATPase, Na+/K+ transporting, beta 3 polypeptide	1.0724	0.0799	0.363	0.0619	No	1.0891	0.084	0.293	0.989	No	No
99580_s_at	Ugt1a2 /// Ugt1a6a /// Ugt1a10 /// Ugt1a7c /// Ugt1a5 /// Ugt1a9 /// Ugt1a6b /// Ugt1a1	UDP glucuronosyltransferase 1 family, polypeptide A2 /// UDP glucuronosyltransferase 1 family, polypeptide A6A /// UDP glycosyltransferase 1 family, polypeptide A10 /// UDP glucuronosyltransferase 1 family, polypeptide A7C /// UDP glucuronosyltransferase 1 family, polypeptide A5 /// UDP glucuronosyltransferase 1 family, polypeptide A9 /// UDP glucuronosyltransferase 1 family, polypeptide A6B /// UDP glucuronosyltransferase 1 family, polypeptide A1	0.9623	0.1502	0.0008	0.2325	No	1.0663	0.136	0.0255	0.524	No	No
99581_at	Hint1	histidine triad nucleotide binding protein 1	1.1186	0.1327	0.0305	0.7703	No	1.0433	0.324	0.187	0.439	No	No
99582_at	Tacstd1	tumor-associated calcium signal transducer 1	1.1491	0.1133	0.0336	0.1293	No	1.1285	0.0626	0.0166	0.646	No	No
99583_at	Gstp1	glutathione S-transferase, pi 1	0.9526	0.2516	0.0006	0.2736	No	0.9684	0.578	0.0002	0.114	No	No
99584_at	Cd82	CD82 antigen	1.0119	0.2685	0.6119	0.0163	No	1.0588	0.285	0.0439	0.291	No	No
99585_at	Cd82	CD82 antigen	1.0517	0.0948	0.0456	0.0046	No	1.0486	0.401	0.472	0.039	No	No
99586_at	Cst3	cystatin C	0.9031	0.0454	0.017	0.0364	No	0.8817	0.0165	0.0016	0.0195	No	No
99587_at	Rab7	RAB7, member RAS oncogene family	0.9632	0.3773	0.0446	0.1739	No	0.9434	0.138	0.0661	0.0294	No	No
99589_f_at	Prm1	protamine 1	0.9586	0.1638	0.1824	0.0757	No	0.9921	0.888	0.295	0.694	No	No
99590_at	Rps17	ribosomal protein S17	1.0097	0.7398	0.8164	0.3176	No	1.0357	0.193	0.219	0.141	No	No
99591_i_at	Rdh11	retinol dehydrogenase 11	0.912	0.1803	0.0099	0.9437	No	0.9581	0.392	0.0034	0.824	No	No
99592_f_at	Rdh11	retinol dehydrogenase 11	1.0006	0.9843	0.0087	0.7284	No	1.0211	0.622	0.011	0.808	No	No
99593_at	Ndufs5	NADH dehydrogenase (ubiquinone) Fe-S protein 5	1.0267	0.3598	0.0066	0.126	No	0.989	0.937	0.0008	0.0334	No	No
99594_at	Mrpl51	mitochondrial ribosomal protein L51	1.0494	0.2719	0.463	0.5339	No	1.0899	0.0966	0.946	0.182	No	No
99595_at	Sepw1	selenoprotein W, muscle 1	0.9691	0.6627	0.0156	0.9843	No	0.9674	0.839	0.0414	0.471	No	No
99596_f_at	Gnai2	guanine nucleotide binding protein, alpha inhibiting 2	0.9467	0.2423	0.0064	0.0662	No	0.9392	0.297	0.0028	0.0277	No	No
99597_at	Gnai2	guanine nucleotide binding protein, alpha inhibiting 2	1.0464	0.1792	0.0007	0.9692	No	0.9525	0.0814	0.0004	0.052	No	No
99598_g_at	Gnai2	guanine nucleotide binding protein, alpha inhibiting 2	1.0251	0.0415	0	0.2946	No	1.0506	0.003	0	0.189	Yes	Yes
99599_s_at	Ptov1	prostate tumor over expressed gene 1	1.0252	0.3687	0.0001	0.2142	No	1.0269	0.464	0.0003	0.553	No	No
99600_at	Ptov1	prostate tumor over expressed gene 1	1.081	0.2072	0.0004	0.5677	No	1.0279	0.594	0.0011	0.682	No	No
99602_at	Klf10	Kruppel-like factor 10	1.1394	0.0104	0.0053	0.5139	No	1.1334	0.0324	0.0196	0.437	No	No
99603_g_at	Klf10	Kruppel-like factor 10	1.1154	0.0854	0.3439	0.7914	No	1.0785	0.15	0.623	0.688	No	No
99604_at	38781	membrane-associated ring finger (C3HC4) 5	1.049	0.4146	0.0268	0.1997	No	1.1126	0.0343	0.375	0.0627	No	No
99605_at	---	---	1.0066	0.8998	0.045	0.3134	No	0.9707	0.723	0.0412	0.672	No	No
99606_at	Limk2	LIM motif-containing protein kinase 2	0.9952	0.9158	0.4451	0.1741	No	1.0365	0.0738	0.0029	0.0367	No	No
99607_at	Skp1a	S-phase kinase-associated protein 1A	1.1063	0.2308	0.5736	0.5585	No	0.9953	0.992	0.506	0.149	No	No
99608_at	Prdx2	peroxiredoxin 2	1.0402	0.3278	0.1363	0.0862	No	1.0667	0.137	0.0695	0.822	No	No
99609_at	Cxx1a	CAAX box 1 homolog A (human)	1.0026	0.9594	0.0661	0.0437	No	1.0498	0.513	0.111	0.728	No	No
99610_at	Ss18	synovial sarcoma translocation, Chromosome 18	1.0281	0.2238	0.0133	0.0045	No	1.0202	0.92	0.0009	0.157	No	No
99613_at	Mut	methylmalonyl-Coenzyme A mutase	0.9197	0.2097	0.0358	0.9937	No	0.8704	0.0297	0.0412	0.78	No	No
99615_at	0610040D20Rik	RIKEN cDNA 0610040D20 gene	1.1035	0.0152	0.0896	0.0497	No	1.034	0.553	0.986	0.408	No	No
99616_s_at	0610040D20Rik	RIKEN cDNA 0610040D20 gene	1.0072	0.8964	0.2905	0.1291	No	1.0366	0.294	0.0128	0.0321	No	No
99617_at	Sec24c	SEC24 related gene family, member C (S. cerevisiae)	0.9863	0.7074	0.1881	0.7693	No	0.9686	0.404	0.0153	0.445	No	No
99618_at	Uqcr	ubiquinol-cytochrome c reductase (6.4kD) subunit	0.93	0.3137	0.0303	0.0777	No	0.924	0.423	0.0838	0.116	No	No
99619_at	Tmem41b	transmembrane protein 41B	1.0178	0.4052	0.0208	0.3576	No	1.0177	0.373	0.0137	0.148	No	No
99620_at	Sfpq	splicing factor proline/glutamine rich (polypyrimidine tract binding protein associated)	1.2573	0.1855	0.0158	0.7649	No	1.1398	0.225	0.0052	0.379	No	No
99621_s_at	Sfpq	splicing factor proline/glutamine rich (polypyrimidine tract binding protein associated)	0.9965	0.9708	0.156	0.736	No	0.9931	0.982	0.15	0.502	No	No
99622_at	Klf4	Kruppel-like factor 4 (gut)	1.0199	0.6706	0.4831	0.7447	No	0.9968	0.923	0.353	0.167	No	No
99623_s_at	Olfm1	olfactomedin 1	1.0573	0.3701	0.244	0.0919	No	1.1922	0.0064	0.285	0.336	Yes	No

99624_at	9530068E07 Rik	RIKEN cDNA 9530068E07 gene	1.029	0.373	0.3069	0.4885	No	1.03	0.581	0.6	0.936	No	No
99626_i_at	Rpl38	ribosomal protein L38	0.8766	0.0271	0.4322	0.1295	No	0.9246	0.395	0.61	0.163	No	No
99627_r_at	Rpl38	ribosomal protein L38	1.0304	0.7498	0.0804	0.2605	No	1.0586	0.67	0.48	0.111	No	No
99628_at	LOC622469 /// LOC675606	similar to Splicing factor, arginine/serine-rich 5 (Pre-mRNA splicing factor SRP40) (Insulin-induced growth response protein CL-4) (Delayed-early protein HRS) /// similar to Splicing factor, arginine/serine-rich 5 (Pre-mRNA splicing factor SRP40) (Insulin-induced growth response protein CL-4) (Delayed-early protein HRS)	1.0006	0.9919	0.033	0.8231	No	0.9639	0.546	0.227	0.382	No	No
99629_at	Ei24	etoposide induced 2.4 mRNA	1.0601	0.5367	0.0411	0.4967	No	1.1166	0.306	0.0194	0.489	No	No
99630_at	Mrpl54	mitochondrial ribosomal protein L54	0.9379	0.2107	0.0063	0.0186	No	0.9237	0.358	0.821	0.364	No	No
99631_f_at	Cox6a1	cytochrome c oxidase, subunit VI a, polypeptide 1	1.0127	0.7143	0.0743	0.0069	No	1.0317	0.175	0.0021	0.0095	No	No
99632_at	Mad2l1	MAD2 (mitotic arrest deficient, homolog)-like 1 (yeast)	1.0919	0.4986	0.0446	0.5759	No	1.0363	0.5	0.0264	0.138	No	No
99633_at	Ncdn	neurochondrin	1.0013	0.9743	0.0047	0.0504	No	1.0451	0.704	0.0016	0.0313	No	No
99635_at	Ing4	inhibitor of growth family, member 4	1.0047	0.6478	0	0.0218	No	0.9888	0.539	0.0004	0.925	No	No
99636_at	Poldip2	polymerase (DNA-directed), delta interacting protein 2	0.9603	0.0935	0.0005	0.009	No	0.9206	0.0439	0.0014	0.075	No	No
99637_at	Col15a1	procollagen, type XV	1.042	0.0157	0.0002	0.0013	No	1.0192	0.975	0.011	0.178	No	No
99638_at	Col18a1	procollagen, type XVIII, alpha 1	1.0167	0.2258	0.0045	0.8111	No	1.103	0.185	0.0029	0.938	No	No
99639_at	Usp10	ubiquitin specific peptidase 10	1.0248	0.6871	0.0214	0.748	No	1.0066	0.862	0.0019	0.659	No	No
99640_at	Minpp1	multiple inositol polyphosphate histidine phosphatase 1	1.038	0.319	0.2568	0.3536	No	1.0285	0.675	0.0358	0.587	No	No
99641_at	Dbndd2	dysbindin (dystrobrevin binding protein 1) domain containing 2	0.9844	0.6126	0.0016	0.1794	No	0.9765	0.783	0.592	0.947	No	No
99642_i_at	Cpe /// LOC677374	carboxypeptidase E /// similar to carboxypeptidase E	1.043	0.6181	0.8782	0.9732	No	0.9728	0.762	0.294	0.601	No	No
99643_f_at	Cpe /// LOC677374	carboxypeptidase E /// similar to carboxypeptidase E	1.057	0.4483	0.4286	0.2665	No	0.9936	0.973	0.0557	0.228	No	No
99644_at	Zfp289	zinc finger protein 289	1.0252	0.4577	0.15	0.0787	No	0.8928	0.135	0.334	0.527	No	No
99645_at	4921506J03 Rik	RIKEN cDNA 4921506J03 gene	0.9637	0.5781	0.2965	0.3209	No	0.9477	0.363	0.119	0.26	No	No
99646_at	Ppt1	palmitoyl-protein thioesterase 1	0.9939	0.7835	0.8299	0.728	No	0.9852	0.876	0.605	0.471	No	No
99647_at	Rpe	ribulose-5-phosphate-3-epimerase	1.018	0.6674	0.0002	0.746	No	1.0553	0.473	0.0125	0.746	No	No
99648_at	Apoa2	apolipoprotein A-II	1.1796	0.17	0.1247	0.4039	No	1.0768	0.0743	0.108	0.015	No	No
99649_at	---	---	0.9769	0.3106	0	0.1007	No	0.9927	0.977	0.0021	0.166	No	No
99650_at	Csnk1a1	casein kinase 1, alpha 1	1.0427	0.5193	0.0094	0.9279	No	0.9511	0.0423	0.0001	0.968	No	No
99651_at	2610209M04 Rik	RIKEN cDNA 2610209M04 gene	1.0166	0.7469	0.0101	0.2287	No	0.9769	0.748	0.0009	0.252	No	No
99652_at	Bat5	HLA-B associated transcript 5	1.0229	0.6323	0.0059	0.7594	No	1.0375	0.353	0.0428	0.808	No	No
99653_at	Rpl14	ribosomal protein L14	1.1395	0.0542	0.9973	0.1724	No	1.0921	0.469	0.0576	0.867	No	No
99654_s_at	Krtdap	keratinocyte differentiation associated protein	0.9754	0.6649	0.0122	0.0467	No	0.9673	0.383	0.0047	0.0211	No	No
99655_at	Sec11l1	Sec11-like 1 (S. cerevisiae)	1.03	0.4985	0.9058	0.9993	No	0.9983	0.99	0.406	0.719	No	No
99656_at	Trmt1	TRM1 tRNA methyltransferase 1 homolog (S. cerevisiae)	1.0519	0.3084	0.0436	0.2903	No	1.0306	0.748	0.0062	0.279	No	No
99657_at	Rps14 /// LOC545121 /// LOC631751 /// LOC672818 /// LOC672819	ribosomal protein S14 /// similar to ribosomal protein S14 /// similar to ribosomal protein S14 /// similar to ribosomal protein S14 /// similar to ribosomal protein S14	1.0542	0.4612	0.3061	0.9743	No	0.9935	0.89	0.162	0.995	No	No
99658_f_at	Fahd1	fumarylacetoacetate hydrolase domain containing 1	0.9127	0.0712	0.0027	0.0919	No	0.9059	0.0002	0	0.0002	No	No
99659_r_at	Fahd1	fumarylacetoacetate hydrolase domain containing 1	0.8689	0.1973	0.1711	0.7932	No	0.8561	0.178	0.002	0.903	No	No
99660_f_at	Cox7c	cytochrome c oxidase, subunit VIIc	0.9869	0.7719	0.0131	0.321	No	0.9363	0.0951	0.0296	0.0641	No	No
99661_r_at	Cox7c	cytochrome c oxidase, subunit VIIc	0.9818	0.6562	0.0176	0.3042	No	0.9491	0.18	0.0082	0.0464	No	No
99662_at	Nup85	nucleoporin 85	1.0004	0.988	0.0009	0.2054	No	1.0028	0.827	0.0004	0.463	No	No
99663_g_at	Nup85	nucleoporin 85	0.9613	0.1422	0.0117	0.1456	No	1.0119	0.747	0.011	0.134	No	No
99664_at	Pcnt	pericentrin (kendrin)	0.9727	0.5691	0.0101	0.3363	No	0.9894	0.642	0.0091	0.0879	No	No
99665_at	Satb1	special AT-rich sequence binding protein 1	1.0351	0.4905	0.0037	0.4877	No	1.0194	0.711	0.0245	0.619	No	No
99666_at	Cs	citrate synthase	1.0328	0.4584	0.016	0.7037	No	0.9859	0.543	0.58	0.663	No	No
99667_at	Cox6a2	cytochrome c oxidase, subunit VI a, polypeptide 2	0.7385	0.0032	0.3059	0.0019	No	0.8686	0.0002	0.0545	0	No	No
99668_at	Bin1	bridging integrator 1	0.932	0.0667	0.0183	0.0077	No	0.9266	0.0061	0.0089	0.0003	No	No
99669_at	Lgals1	lectin, galactose binding, soluble 1	0.9816	0.5423	0.0051	0.0135	No	0.9698	0.455	0.0006	0.0102	No	No
99670_at	Bad	Bcl-associated death promoter	1.021	0.7234	0.0036	0.2786	No	0.9931	0.883	0.002	0.325	No	No
99671_at	Cfd	complement factor D (adipsin)	0.9891	0.877	0.2852	0.1716	No	0.9477	0.565	0.114	0.254	No	No
99672_at	Clcn4-2	chloride channel 4-2	1.105	0.2803	0.3196	0.4917	No	1.0324	0.666	0.314	0.937	No	No
99674_at	Mrpl43	mitochondrial ribosomal protein L43	1.0298	0.7095	0.0356	0.2378	No	1.0215	0.618	0.0062	0.112	No	No
99675_at	Yif1a	Yip1 interacting factor homolog A (S. cerevisiae)	1.0544	0.1059	0.0009	0.6554	No	1.0629	0.171	0.0056	0.682	No	No
99677_at	2610042O14 Rik	RIKEN cDNA 2610042O14 gene	1.0514	0.1202	0.7367	0.5787	No	1.0378	0.459	0.285	0.805	No	No
99678_f_at	Atp5l /// LOC227112 /// LOC627998 /// LOC632911	ATP synthase, H+ transporting, mitochondrial F0 complex, subunit g /// similar to ATP synthase, H+ transporting, mitochondrial F0 complex, subunit G /// similar to ATP synthase, H+ transporting, mitochondrial F0 complex, subunit G /// similar to ATP synthase, H+ transporting, mitochondrial F0 complex, subunit g	1.0748	0.0931	0.0008	0.5207	No	1.1734	0.13	0.0026	0.373	No	No
99681_at	---	---	0.9663	0.6822	0.2293	0.602	No	0.9984	0.942	0.116	0.715	No	No
99699_at	---	Murine retrovirus readthrough RNA sequence	0.9999	0.998	0.9479	0.8605	No	1.1099	0.26	0.662	0.039	No	No
99700_at	V2r1b	vomeronasal 2, receptor, 1b	1.0165	0.4051	0.7947	0.0759	No	1.0892	0.0839	0.373	0.0739	No	No
99701_f_at	Sprr2b	small proline-rich protein 2B	1.0389	0.4124	0.4623	0.3739	No	1.1158	0.0699	0.203	0.146	No	No
99702_at	---	---	0.9888	0.8029	0.0894	0.2383	No	0.9944	0.853	0.0503	0.365	No	No
99708_at	Prss29	Protease, serine, 29	0.9505	0.0903	0.3672	0.0488	No	0.894	0.0545	0.0474	0.736	No	No
99756_at	---	Transcribed locus	1.0173	0.4354	0.0102	0.0287	No	0.9171	0.378	0.214	0.565	No	No
99776_at	Myo1b	Myosin IB	1.0484	0.3982	0.0284	0.5747	No	0.95	0.286	0.118	0.0228	No	No
99777_s_at	6230416J20 Rik	RIKEN cDNA 6230416J20 gene	0.9681	0.5817	0.3007	0.3274	No	0.9775	0.812	0.101	0.14	No	No
99778_at	Snora65 /// Rpl12 /// LOC546695 /// LOC622589 /// LOC630855 /// LOC633363 /// LOC633570 /// LOC639477 /// LOC668706 /// LOC668782 /// LOC668844 /// LOC669503 /// LOC671640 /// LOC672158 /// LOC672173	small nucleolar RNA, H/ACA box 65 /// ribosomal protein L12 /// similar to 60S ribosomal protein L12 /// similar to 60S ribosomal protein L12 /// similar to 60S ribosomal protein L12 /// similar to 60S ribosomal protein L12 /// similar to 60S ribosomal protein L12 /// similar to 60S ribosomal protein L12 /// similar to 60S ribosomal protein L12 /// similar to 60S ribosomal protein L12 /// similar to 60S ribosomal protein L12 /// similar to 60S ribosomal protein L12 /// similar to 60S ribosomal protein L12 /// similar to 60S ribosomal protein L12 /// similar to 60S ribosomal protein L12	0.9559	0.0838	0.2128	0.0447	No	0.9848	0.317	0.0029	0.001	No	No
99779_at	2810482G21 Rik	RIKEN cDNA 2810482G21 gene	1.0122	0.8515	0.4911	0.4877	No	1.032	0.441	0.0076	0.475	No	No

99798_at	Tcrb-V13	T-cell receptor beta, variable 13	1.1024	0.052	0.4513	0.3166	No	0.9638	0.69	0.668	0.413	No	No
99799_at	Vav1	vav 1 oncogene	0.9807	0.6802	0.0674	0.6445	No	1.1418	0.167	0.226	0.268	No	No
99800_at	L1cam	L1 cell adhesion molecule	0.9863	0.8365	0.0627	0.193	No	0.9614	0.43	0.335	0.199	No	No
99801_at	---	---	1.0044	0.9097	0.284	0.8342	No	0.9	0.114	0.006	0.492	No	No
99802_at	Adra2b	adrenergic receptor, alpha 2b	0.9957	0.9616	0.1108	0.5519	No	0.9385	0.478	0.0584	0.124	No	No
99803_at	Hist1h1b	histone 1, H1b	0.995	0.9112	0.0655	0.1673	No	1.0796	0.121	0.0594	0.373	No	No
99804_at	Adra2c	adrenergic receptor, alpha 2c	1.0838	0.2155	0.8469	0.8321	No	1.1256	0.0033	0.261	0.0012	No	No
99805_at	---	---	0.9569	0.1748	0.0542	0.7326	No	0.9701	0.768	0.165	0.989	No	No
99806_at	Npr3	natriuretic peptide receptor 3	0.9493	0.4213	0.0372	0.6291	No	0.9109	0.23	0.47	0.838	No	No
99807_r_at	Fv1	Friend virus susceptibility 1	0.9057	0.4107	0.7064	0.4564	No	0.8196	0.112	0.94	0.105	No	No
99808_at	Hoxb13	homeo box B13	0.9825	0.8715	0.0527	0.5366	No	0.9983	0.923	0.0524	0.663	No	No
99809_at	Pax2	paired box gene 2	1.0298	0.4825	0.0211	0.3522	No	1.0195	0.423	0.0094	0.1	No	No
99810_at	Gpx2	glutathione peroxidase 2	1.017	0.7696	0.7288	0.585	No	0.9154	0.0016	0.0811	0.0219	Yes	No
99811_at	Npy6r	neuropeptide Y receptor Y6	0.9778	0.5546	0.2852	0.0547	No	1.0524	0.288	0.552	0.0255	No	No
99812_at	Capn3	calpain 3	0.9412	0.3129	0.355	0.0591	No	0.9589	0.617	0.876	0.604	No	No
99813_g_at	Capn3	calpain 3	0.9996	0.9918	0.1374	0.4067	No	0.9299	0.345	0.0067	0.118	No	No
99814_at	Neurog3	neurogenin 3	0.9254	0.2781	0.2303	0.4351	No	0.9464	0.42	0.176	0.77	No	No
99815_at	Hrh2	histamine receptor H 2	0.9805	0.7357	0.018	0.8456	No	1.0667	0.043	0.202	0.225	No	No
99816_at	Hspa2	heat shock protein 2	0.9888	0.7894	0.0087	0.0555	No	0.9928	0.933	0.0709	0.514	No	No
99817_at	Vax2	ventral anterior homeobox containing gene 2	0.961	0.6188	0.4641	0.4426	No	0.9897	0.886	0.624	0.667	No	No
99818_at	C79122	expressed sequence C79122	1.0747	0.1917	0.8737	0.0472	No	1.161	0.0661	0.0219	0.0965	No	No
99819_at	C79709	expressed sequence C79709	0.9864	0.7223	0.3028	0.8	No	0.9893	0.682	0.193	0.232	No	No
99820_f_at	Cecr5	cat eye syndrome chromosome region, candidate 5 homolog (human)	1.1554	0.0661	0.0007	0.068	No	1.1311	0.139	0.0003	0.0622	No	No
99821_at	Chd8	Chromodomain helicase DNA binding protein 8	1.0924	0.1259	0.0516	0.3093	No	1.0677	0.601	0.144	0.436	No	No
99823_r_at	D18Ertd232e	DNA segment, Chr 18, ERATO Doi 232, expressed	1.1767	0.0232	0.0111	0.003	No	1.1323	0.0947	0.0455	0.01	No	No
99824_at	---	---	1.0821	0.3231	0.1329	0.4292	No	1.0347	0.504	0.108	0.491	No	No
99825_at	D030022P06Rik	RIKEN cDNA D030022P06 gene	0.9987	0.9772	0.123	0.1566	No	0.9524	0.225	0.0035	0.354	No	No
99826_at	---	---	0.931	0.2128	0.4371	0.465	No	0.6788	0.134	0.427	0.134	No	No
99827_at	4933403M19Rik	RIKEN cDNA 4933403M19 gene	1.0999	0.0689	0.0503	0.1211	No	1.0609	0.309	0.446	0.471	No	No
99828_at	Ccdc39	coiled-coil domain containing 39	1.013	0.8425	0.0012	0.055	No	1.1106	0.122	0.0317	0.012	No	No
99829_at	Igh-VJ558	immunoglobulin heavy chain (J558 family)	0.9925	0.8994	0.6146	0.5961	No	0.8717	0.18	0.596	0.326	No	No
99830_at	Kalrn	kalirin, RhoGEF kinase	1.084	0.5084	0.4768	0.8556	No	1.0509	0.471	0.262	0.882	No	No
99831_at	Cort	cortistatin	0.9315	0.3704	0.2343	0.258	No	0.9181	0.241	0.088	0.92	No	No
99832_at	Kcnmb1	potassium large conductance calcium-activated channel, subfamily M, beta member 1	0.9921	0.8996	0.1215	0.5828	No	0.9759	0.785	0.0466	0.984	No	No
99833_at	Capn9 /// LOC639715	calpain 9 (nCL-4) /// similar to calpain 9 (nCL-4)	0.9958	0.9401	0.1914	0.1722	No	1.1082	0.12	0.551	0.0344	No	No
99834_at	Nrg3	neuregulin 3	0.9673	0.6203	0.331	0.6459	No	1.0179	0.649	0.0082	0.239	No	No
99835_at	Fosl1	fos-like antigen 1	1.0396	0.4932	0.041	0.1768	No	0.9039	0.365	0.798	0.944	No	No
99836_at	Cyp27b1	cytochrome P450, family 27, subfamily b, polypeptide 1	0.9714	0.6048	0.482	0.2879	No	0.9864	0.733	0.0417	0.121	No	No
99837_at	Galr1	galanin receptor 1	1.0431	0.2389	0.3885	0.3742	No	1.0375	0.671	0.677	0.145	No	No
99838_at	Aoah	acyloxyacyl hydrolase	0.9662	0.4538	0.7648	0.5849	No	0.9083	0.0536	0.0396	0.0875	No	No
99839_at	Gabrr2	gamma-aminobutyric acid (GABA-C) receptor, subunit rho 2	0.9061	0.0721	0.0237	0.1855	No	0.9446	0.245	0.0582	0.0706	No	No
99840_at	Pdyn	prodynorphin	1.0714	0.0307	0.0088	0.6718	No	1.0902	0.159	0.0428	0.321	No	No
99841_at	Blr1	Burkitt lymphoma receptor 1	1.0755	0.2082	0.0003	0.2932	No	1.0756	0.299	0.0001	0.718	No	No
99842_at	Col19a1	procollagen, type XIX, alpha 1	1.1248	0.0385	0.0571	0.0217	No	1.1475	0.0265	0.0125	0.0105	No	No
99843_at	Pitx3	paired-like homeodomain transcription factor 3	0.9992	0.9647	0.0024	0.0749	No	0.9675	0.569	0.697	0.16	No	No
99844_at	Fzd9	frizzled homolog 9 (Drosophila)	0.9576	0.4257	0.0019	0.0298	No	0.8761	0.0713	0.0006	0.0368	No	No
99845_at	Slc1a6	solute carrier family 1 (high affinity aspartate/glutamate transporter), member 6	1.0163	0.4996	0.0022	0.6856	No	1.0271	0.564	0.0023	0.0398	No	No
99846_at	Foxf2	forkhead box F2	1.0983	0.1334	0.1051	0.168	No	1.1102	0.261	0.105	0.423	No	No
99847_at	St3gal1	ST3 beta-galactoside alpha-2,3-sialyltransferase 1	0.9733	0.2818	0.002	0.1459	No	1.0043	0.999	0.782	0.647	No	No
99848_at	Alox8	arachidonate 8-lipoxygenase	0.9644	0.4839	0.8648	0.4329	No	1.0678	0.169	0.1	0.0426	No	No
99849_at	1200016E24Rik	RIKEN cDNA 1200016E24 gene	0.8231	0.0035	0.0034	0.1464	Yes	0.8346	0.0104	0.0211	0.177	No	Yes
99850_at	Igh-1a	Immunoglobulin heavy chain 1a (serum IgG2a)	0.931	0.1955	0.0333	0.1928	No	0.9422	0.351	0.211	0.133	No	No
99851_at	Zfp292	zinc finger protein 292	1.0115	0.4684	0	0.0063	No	0.9955	0.929	0.0008	0.278	No	No
99854_at	Sult3a1	sulfotransferase family 3A, member 1	0.9694	0.2954	0.078	0.0342	No	1.1035	0.122	0.0022	0.07	No	No
99855_at	LOC675366	similar to mitogen activated protein kinase kinase kinase 5	0.9984	0.9635	0.4396	0.8455	No	0.9528	0.177	0.135	0.101	No	No
99856_r_at	Ctnnd2	catenin (cadherin associated protein), delta 2	1.1151	0.0164	0.0014	0.0103	No	1.0567	0.041	0.0002	0.0378	No	No
99860_at	Gnat1	guanine nucleotide binding protein, alpha transducing 1	1.0098	0.896	0.1799	0.5148	No	0.9362	0.14	0.0352	0.17	No	No
99861_at	Dhh	desert hedgehog	1.004	0.7616	0.561	0.0078	No	1.0014	0.954	0.206	0.279	No	No
99862_at	Ahsg	alpha-2-HS-glycoprotein	0.8618	0.078	0.8646	0.2257	No	0.9223	0.195	0.0152	0.638	No	No
99863_at	Slc7a6	solute carrier family 7 (cationic amino acid transporter, y+ system), member 6	1.0172	0.7499	0.4282	0.386	No	0.9749	0.674	0.144	0.588	No	No
99864_at	Adora2b	adenosine A2b receptor	1.0358	0.3036	0.1446	0.0222	No	1.0378	0.41	0.0279	0.0969	No	No
99865_at	Bmpr2	bone morphogenic protein receptor, type II (serine/threonine kinase)	0.9512	0.2684	0.9455	0.8797	No	0.9005	0.0989	0.147	0.0435	No	No
99866_at	Rbms1	RNA binding motif, single stranded interacting protein 1	1.0559	0.382	0.0239	0.2364	No	1.0091	0.548	0.0085	0.2	No	No
99867_at	---	---	0.9263	0.2635	0.0179	0.3638	No	0.9653	0.552	0.0022	0.0905	No	No
99868_at	---	Transcribed locus	1.0514	0.4279	0.678	0.5288	No	1.0327	0.815	0.881	0.139	No	No
99869_at	Hdgfl1	hepatoma derived growth factor-like 1	1.0117	0.6713	0.0018	0.7085	No	1.0045	0.821	0.0061	0.278	No	No
99870_at	Mark1	MAP/microtubule affinity-regulating kinase 1	1.0058	0.9432	0.044	0.7287	No	1.0327	0.573	0.0062	0.897	No	No
99871_f_at	Psg19	Pregnancy specific glycoprotein 19	0.9745	0.4813	0.5851	0.4012	No	0.9886	0.878	0.307	0.491	No	No
99872_s_at	Ftl1 /// Ftl2 /// LOC434624 /// LOC544951 /// LOC634386 /// LOC665937 /// LOC666673 /// LOC669088 /// LOC675170	ferritin light chain 1 /// ferritin light chain 2 /// similar to Ferritin light chain 1 (Ferritin L subunit 1) /// similar to Ferritin light chain 1 (Ferritin L subunit 1) /// similar to Ferritin light chain 1 (Ferritin L subunit 1) /// similar to Ferritin light chain 1 (Ferritin L subunit 1) /// similar to Ferritin light chain 1 (Ferritin L subunit 1) /// similar to Ferritin light chain 1 (Ferritin L subunit 1) /// similar to Ferritin light chain 1 (Ferritin L subunit 1)	1.0472	0.1986	0.0548	0.5187	No	1.0388	0.201	0.0058	0.187	No	No
99873_at	Nkx2-6	NK2 transcription factor related, locus 6 (Drosophila)	0.9587	0.2906	0.0094	0.3105	No	1.0016	0.973	0.0153	0.795	No	No
99874_at	Rap2b	RAP2B, member of RAS oncogene family	0.9915	0.8616	0.1067	0.6498	No	1.1049	0.198	0.565	0.747	No	No
99875_at	Hr	hairless	0.9461	0.1637	0.0234	0.9603	No	0.9641	0.187	0.0127	0.0037	No	No
99876_at	Sla	src-like adaptor	0.9509	0.114	0.1663	0.8751	No	1.0482	0.439	0.631	0.258	No	No
99878_at	Ddx10	DEAD (Asp-Glu-Ala-Asp) box polypeptide 10	0.9507	0.0895	0.2592	0.0125	No	1.0196	0.822	0.605	0.0297	No	No
99880_at	Rfx1	regulatory factor X, 1 (influences HLA class II expression)	0.9881	0.6675	0.0006	0.2265	No	0.9894	0.803	0.185	0.644	No	No
99881_at	Dkk1	dickkopf homolog 1 (Xenopus laevis)	1.0231	0.7326	0.8808	0.3637	No	0.9875	0.737	0.0045	0.878	No	No
99882_at	Ids	iduronate 2-sulfatase	0.9735	0.4059	0.0103	0.5779	No	0.9793	0.818	0.959	0.569	No	No
99883_g_at	Ids	iduronate 2-sulfatase	0.9711	0.7101	0.6363	0.5076	No	0.9697	0.646	0.377	0.806	No	No
99884_at	Ids	iduronate 2-sulfatase	0.9656	0.5158	0.1669	0.448	No	1.0047	0.921	0.933	0.209	No	No

99885_at	LOC380823 /// LOC382696 /// LOC434638 /// LOC619833 /// LOC619994 /// LOC622728 /// LOC629893 /// LOC629908 /// LOC629941 /// LOC631531 /// LOC634100 /// LOC634338 /// LOC634652 /// LOC634791	similar to Ig heavy chain V region B1-8/186-2 precursor /// similar to Ig heavy chain V region 3 precursor /// similar to Ig heavy chain V region VH558 A1/A4 precursor /// similar to Ig heavy chain V region 102 precursor /// similar to Ig heavy chain V region VH558 A1/A4 precursor /// similar to Ig heavy chain V region VH558 A1/A4 precursor /// similar to Ig heavy chain V region 108A precursor /// similar to Ig heavy chain V-I region V35 precursor /// similar to Ig heavy chain V region 3 precursor /// similar to Ig heavy chain V region VH558 B4 precursor /// similar to Ig heavy chain V region 108A precursor /// similar to Ig heavy chain V-I region V35 precursor /// similar to Ig heavy chain V region VH558 A1/A4 precursor /// similar to Ig heavy chain V-I region V35 precursor /// similar to Ig heavy chain V region 23 precursor /// similar to Ig heavy chain V region 3 precursor /// similar to Ig heavy chain V region 102 precursor /// similar to Ig heavy chain V region 3 precursor /// similar to Ig heavy chain V region 3 precursor /// similar to Ig heavy chain V region 108A precursor /// similar to Ig he	0.992	0.905	0.7965	0.7978	No	1.0112	0.914	0.17	0.656	No	No
99886_at	Oprk1	opioid receptor, kappa 1	1.0995	0.1073	0.3186	0.0563	No	1.1868	0.047	0.282	0.0392	No	No
99887_at	Efna5	ephrin A5	0.959	0.4221	0.2105	0.1425	No	0.9775	0.549	0.0456	0.0309	No	No
99888_at	Zan	zonadhesin	1.0184	0.7571	0.0636	0.6472	No	0.9396	0.361	0.742	0.542	No	No
99889_at	Sstr5	somatostatin receptor 5	1.0561	0.3443	0.0562	0.7515	No	1.0431	0.0732	0.0003	0.331	No	No
99890_at	Pdgfb	platelet derived growth factor, B polypeptide	0.9532	0.223	0.0049	0.2728	No	0.9869	0.701	0.0034	0.128	No	No
99891_at	Pou6f1	POU domain, class 6, transcription factor 1	1.0142	0.8306	0.0669	0.3807	No	0.9945	0.938	0.0239	0.179	No	No
99892_at	Cnr1	cannabinoid receptor 1 (brain)	1.0445	0.3611	0.9942	0.0135	No	1.0859	0.0957	0.525	0.013	No	No
99893_at	Fgf13	fibroblast growth factor 13	0.9087	0.0997	0.1796	0.1239	No	1.1333	0.365	0.389	0.978	No	No
99894_at	Ptgfrn	prostaglandin F2 receptor negative regulator	1.0121	0.8606	0.2492	0.6819	No	0.9418	0.397	0.293	0.873	No	No
99895_at	Ccr8	chemokine (C-C motif) receptor 8	1.0007	0.9864	0.1983	0.2724	No	1.0879	0.204	0.861	0.657	No	No
99896_at	Mc5r	melanocortin 5 receptor	1.0245	0.4058	0.3739	0.0634	No	1.0397	0.406	0.59	0.101	No	No
99897_at	Gabrb3	gamma-aminobutyric acid (GABA-A) receptor, subunit beta 3	1.028	0.4249	0.9598	0.2074	No	1.1589	0.008	0.0031	0.0042	No	No
99898_at	Gng4	guanine nucleotide binding protein (G protein), gamma 4 subunit	0.8957	0.0358	0.0384	0.0525	No	0.9222	0.017	0.147	0.0064	No	No
99899_at	Ccr6	chemokine (C-C motif) receptor 6	0.9727	0.4947	0.5221	0.7116	No	1.0366	0.716	0.607	0.699	No	No
99900_at	Crx	cone-rod homeobox containing gene	0.994	0.903	0.1983	0.4673	No	1.037	0.235	0.0036	0.263	No	No
99901_at	Ptrf	polymerase I and transcript release factor	1.0002	0.997	0.0043	0.3427	No	0.9254	0.101	0.551	0.142	No	No
99902_at	Hcn1	hyperpolarization-activated, cyclic nucleotide-gated K+ 1	0.9545	0.5805	0.2843	0.4048	No	1.0692	0.425	0.889	0.836	No	No
99903_at	Dmp1	dentin matrix protein 1	0.9128	0.1295	0.0092	0.7431	No	0.901	0.298	0.351	0.591	No	No
99904_at	Itgb3	integrin beta 3	0.9739	0.5822	0.0114	0.062	No	0.9708	0.592	0.334	0.236	No	No
99905_at	Dub2	deubiquitinating enzyme 2	0.9117	0.1526	0.5708	0.0251	No	0.9444	0.122	0.0413	0.0065	No	No
99906_at	Esx1	extraembryonic, spermatogenesis, homeobox 1	0.9181	0.2749	0.0598	0.5391	No	0.9846	0.389	0.0774	0.0018	No	No
99907_at	Rgs9	regulator of G-protein signaling 9	1.0231	0.6619	0.4587	0.5749	No	1.0791	0.203	0.213	0.0117	No	No
99908_at	Pax4	paired box gene 4	1.0148	0.561	0.0556	0.2155	No	1.1117	0.0844	0.0023	0.014	No	No
99909_at	Trpc6	transient receptor potential cation channel, subfamily C, member 6	0.875	0.0061	0.024	0.3873	Yes	0.9332	0.944	0.0015	0.733	No	No
99910_at	Accn1	amiloride-sensitive cation channel 1, neuronal (degenerin)	0.9715	0.3032	0.0982	0.8474	No	1.0288	0.547	0.259	0.235	No	No
99911_at	Sema6b	sema domain, transmembrane domain (TM), and cytoplasmic domain, (semaphorin) 6B	0.9874	0.7447	0.4645	0.5322	No	1.0166	0.664	0.527	0.855	No	No
99912_at	Aff2	AF4/FMR2 family, member 2	1.0159	0.7299	0.0577	0.9655	No	1.1508	0.0165	0.043	0.587	No	No
99913_at	Chad	chondroadherin	0.8354	0.0005	0	0.0007	No	0.8589	0.0438	0	0.0676	No	No
99914_at	Hand2	heart and neural crest derivatives expressed transcript 2	1.0532	0.6172	0.0753	0.9799	No	1.0444	0.0933	0.0001	0.308	No	No
99915_at	Areg	amphiregulin	0.966	0.608	0.1042	0.6479	No	0.9918	0.959	0.774	0.696	No	No
99916_at	Prkch	protein kinase C, eta	0.9569	0.1089	0.0011	0.0495	No	0.9035	0.0002	0	0.0023	No	No
99917_at	Ezh2	enhancer of zeste homolog 2 (Drosophila)	0.9488	0.2754	0.0181	0.8584	No	0.9261	0.358	0.017	0.653	No	No
99920_at	Terf1	telomeric repeat binding factor 1	1.0015	0.9867	0.1476	0.7797	No	1.1972	0.302	0.0794	0.541	No	No
99922_at	Slc9a1	solute carrier family 9 (sodium/hydrogen exchanger), member 1	0.9786	0.5867	0.0039	0.5896	No	0.9485	0.0685	0.0019	0.342	No	No
99923_at	Pcf11	cleavage and polyadenylation factor subunit homolog (S. cerevisiae)	0.955	0.391	0.0014	0.5065	No	0.8939	0.0234	0.0003	0.783	No	No
99924_at	Tubg1	tubulin, gamma 1	0.9886	0.8107	0.0247	0.2407	No	1.0416	0.559	0.157	0.54	No	No
99925_f_at	Tubg2	tubulin, gamma 2	1.0283	0.6652	0.3544	0.3998	No	1.0003	0.924	0.0112	0.403	No	No
99926_at	Ubtf	upstream binding transcription factor, RNA polymerase I	1.146	0.0317	0.0039	0.0339	No	1.0783	0.0581	0.0022	0.0381	No	No
99927_at	Cfi	complement component factor i	0.9122	0.0376	0.0028	0.0227	No	0.9163	0.328	0.557	0.263	No	No
99928_at	Mtap1b	microtubule-associated protein 1 B	1.0222	0.6101	0.0113	0.5562	No	1.1085	0.309	0.0084	0.714	No	No
99929_at	Ext2	exostoses (multiple) 2	0.9591	0.1375	0.6401	0.0717	No	0.9818	0.639	0.965	0.17	No	No
99930_s_at	Psen2	presenilin 2	0.8692	0.0369	0.0969	0.1764	No	0.9064	0.132	0.0842	0.791	No	No
99931_at	Lama5 /// LOC671909	laminin, alpha 5 /// similar to Laminin alpha-5 chain precursor	0.9655	0.4171	0.6141	0.5855	No	1.0149	0.635	0.374	0.613	No	No
99932_at	Zbtb17	zinc finger and BTB domain containing 17	0.9067	0.161	0.0227	0.2257	No	1.0226	0.925	0.519	0.382	No	No
99933_at	Pvrl2	poliovirus receptor-related 2	0.9849	0.829	0.0632	0.9239	No	1.0365	0.497	0.107	0.69	No	No
99934_at	Pvrl2	poliovirus receptor-related 2	0.9458	0.1094	0.0264	0.9071	No	0.948	0.29	0.071	0.633	No	No
99935_at	Tjp1	tight junction protein 1	0.92	0.1888	0.0015	0.9831	No	0.8923	0.0707	0.0066	0.268	No	No
99936_at	Tie1	tyrosine kinase receptor 1	0.9369	0.3258	0.0634	0.7174	No	0.8819	0.117	0.0067	0.358	No	No
99937_at	Meox2	mesenchyme homeobox 2	0.9204	0.5043	0.4645	0.1655	No	0.908	0.392	0.179	0.0437	No	No
99938_at	Xrcc1	X-ray repair complementing defective repair in Chinese hamster cells 1	0.8636	0.0119	0.0002	0.0782	No	0.8855	0.0217	0.0005	0.585	No	No
99939_at	Cel	carboxyl ester lipase	0.9877	0.8461	0.6824	0.5215	No	1.0496	0.154	0.618	0.0813	No	No
99940_at	2410004N11Rik	RIKEN cDNA 2410004N11 gene	0.9568	0.4224	0.0552	0.7676	No	0.9819	0.697	0.0216	0.513	No	No
99941_at	Es31	esterase 31	1.029	0.3508	0.1202	0.026	No	1.0622	0.26	0.365	0.0327	No	No
99942_s_at	Cnn1	calponin 1	0.9769	0.3439	0.0028	0.0055	No	0.9774	0.53	0.101	0.0122	No	No
99944_at	Hpca	hippocalcin	1.0108	0.7218	0.1829	0.1397	No	1.1504	0.0024	0.0115	0.345	Yes	No
99945_at	Cd19	CD19 antigen	0.9711	0.5281	0.2339	0.7071	No	1.0367	0.618	0.967	0.368	No	No
99947_at	Tial1	Tial1 cytotoxic granule-associated RNA binding protein-like 1	0.9336	0.2101	0.8964	0.091	No	0.9837	0.404	0.188	0.0084	No	No
99948_at	Ccdc95	coiled-coil domain containing 95	0.9805	0.5558	0.015	0.1745	No	1.0433	0.224	0.359	0.0149	No	No
99949_at	Ccdc95	coiled-coil domain containing 95	0.9792	0.7874	0.1069	0.4626	No	0.8859	0.0932	0.333	0.694	No	No
99950_at	Tbp	TATA box binding protein	1.0419	0.3244	0.8909	0.206	No	1.0077	0.713	0.0206	0.695	No	No
99951_at	Rorc	RAR-related orphan receptor gamma	0.9833	0.6992	0.0133	0.169	No	1.0001	0.815	0.0031	0.125	No	No
99952_at	Chi3l1	chitinase 3-like 1	0.8816	0.0939	0.2883	0.0655	No	0.7833	0.0311	0.0382	0.0098	No	No
99953_at	Rgl2	ral guanine nucleotide dissociation stimulator-like 2	1.0539	0.2263	0.3848	0.2773	No	1.0481	0.13	0.0039	0.0739	No	No
99954_at	Rnu3ip2	RNA, U3 small nucleolar interacting protein 2	1.0223	0.6868	0.7075	0.3999	No	1.0669	0.359	0.488	0.585	No	No
99955_at	Numb	numb gene homolog (Drosophila)	0.9591	0.6374	0.1523	0.7127	No	0.9179	0.269	0.551	0.698	No	No
99956_at	Kit	kit oncogene	1.0998	0.1325	0.0034	0.4914	No	0.9353	0.257	0.0068	0.754	No	No
99957_at	Mmp9	matrix metallopeptidase 9	0.979	0.4759	0.1637	0.1108	No	1.0016	0.926	0.0441	0.505	No	No
99958_at	Mcpt2	mast cell protease 2	1.0624	0.2067	0.2327	0.4814	No	1.1054	0.195	0.323	0.764	No	No
99959_at	Ak3l1	adenylate kinase 3 alpha-like 1	1.0482	0.2451	0.0311	0.1987	No	1.0313	0.493	0.0092	0.66	No	No
99960_at	Map2k4	mitogen activated protein kinase kinase 4	0.9193	0.1499	0.7625	0.7135	No	0.9498	0.248	0.869	0.943	No	No
99961_s_at	Cdc2l1	cell division cycle 2-like 1	0.9315	0.1211	0.0238	0.8128	No	0.9759	0.525	0.0548	0.535	No	No
99962_at	Kif2a	Kinesin family member 2A	0.9708	0.6141	0.0126	0.3731	No	0.9326	0.298	0.0089	0.759	No	No
99963_at	---	---	0.9646	0.6293	0.0139	0.4984	No	0.9709	0.827	0.0092	0.342	No	No
99964_at	Vdr	vitamin D receptor	0.8829	0.1229	0.0727	0.1227	No	0.863	0.0069	0.024	0.0994	Yes	No
99965_at	Vdr	vitamin D receptor	0.9645	0.5296	0.0849	0.7318	No	1.0047	0.777	0.0001	0.0775	No	No
99966_at	D2hgdh /// LOC666009 /// LOC673662	D-2-hydroxyglutarate dehydrogenase /// similar to CG3835-PA, isoform A /// similar to CG3835-PA, isoform A	1.085	0.2201	0.1154	0.8095	No	1.0449	0.462	0.51	0.162	No	No
99970_at	Ptpn21	protein tyrosine phosphatase, non-receptor type 21	0.933	0.3151	0.6959	0.2704	No	0.9413	0.191	0.0195	0.305	No	No

99972_at	Tph1	tryptophan hydroxylase 1	0.9189	0.1662	0.6611	0.9111	No	0.9959	0.967	0.0072	0.975	No	No
99973_s_at	Kcnj15	potassium inwardly-rectifying channel, subfamily J, member 15	0.957	0.2257	0.2073	0.8624	No	0.9938	0.873	0.305	0.824	No	No
99974_at	Kcnj15	potassium inwardly-rectifying channel, subfamily J, member 15	1.0114	0.8936	0.8958	0.1306	No	1.0332	0.527	0.0204	0.989	No	No
99975_at	Prkrir	protein-kinase, interferon-inducible double stranded RNA dependent inhibitor, repressor of (P58 repressor)	1.0257	0.6292	0.079	0.0729	No	1.0655	0.567	0.0697	0.466	No	No
99977_at	Fabp6	fatty acid binding protein 6, ileal (gastrotropin)	1.0065	0.9037	0.2236	0.8046	No	1.0346	0.601	0.567	0.446	No	No
99978_s_at	Mapk14	mitogen activated protein kinase 14	1.0185	0.6725	0.0124	0.6846	No	0.9946	0.843	0.0083	0.506	No	No
99979_at	Cyp1b1	cytochrome P450, family 1, subfamily b, polypeptide 1	0.8592	0.0149	0.0012	0.5425	No	0.8451	0.0106	0.0028	0.473	No	No
99980_at	Hoxc6	homeo box C6	1.0342	0.7702	0.2354	0.3204	No	1.0403	0.555	0.0234	0.331	No	No
99981_at	Gnaq	guanine nucleotide binding protein, alpha q polypeptide	0.9332	0.3811	0.0953	0.8254	No	1.0458	0.152	0.126	0.0434	No	No
99982_at	Nfkbib	nuclear factor of kappa light chain gene enhancer in B-cells inhibitor, beta	0.9914	0.8664	0.0194	0.3982	No	0.8941	0.0558	0.129	0.72	No	No
99984_at	Elk3	ELK3, member of ETS oncogene family	0.9624	0.1584	0.0191	0.0276	No	0.9568	0.419	0.0248	0.215	No	No
99985_at	Txnrd1	thioredoxin reductase 1	1.0917	0.0275	0.002	0.3257	No	1.1168	0.0082	0.0043	0.157	Yes	Yes
99986_at	Gosr2	golgi SNAP receptor complex member 2	1.0332	0.091	0.0016	0.0357	No	1.0888	0.0013	0	0.297	Yes	No
99987_at	Zfp574	zinc finger protein 574	1.0338	0.6915	0.1143	0.329	No	0.9375	0.0871	0.0199	0.0082	No	No
99988_at	Dym	dymeclin	0.9586	0.3936	0.393	0.8865	No	0.9239	0.144	0.384	0.274	No	No
99990_at	Rbbp6	retinoblastoma binding protein 6	0.9735	0.6338	0.0008	0.8571	No	0.9564	0.223	0.0005	0.0802	No	No
99991_at	Il17ra	interleukin 17 receptor A	0.9516	0.0633	0.0383	0.0948	No	0.9178	0.0671	0.0062	0.436	No	No
99992_at	Il17ra	interleukin 17 receptor A	0.9789	0.5206	0.1494	0.1526	No	1.0293	0.347	0.0071	0.0666	No	No
99993_at	Anpep	alanyl (membrane) aminopeptidase	1.0411	0.3955	0.0042	0.5887	No	1.0101	0.889	0.0161	0.689	No	No
99994_at	Cidea	cell death-inducing DNA fragmentation factor, alpha subunit-like effector A	1.2461	0.0006	0.0006	0.0286	Yes	1.1969	0.0051	0.0017	0.077	Yes	Yes
99995_at	Cetn1	centrin 1	1.0053	0.9559	0.2168	0.6191	No	1.0354	0.557	0.202	0.809	No	No
99996_at	Pkp1	plakophilin 1	0.9625	0.3195	0.0005	0.2419	No	0.976	0.603	0.885	0.622	No	No
99997_at	Ptger1 /// Pkn1	prostaglandin E receptor 1 (subtype EP1) /// protein kinase N1	0.9758	0.395	0.0132	0.3769	No	0.9875	0.859	0.0892	0.189	No	No
99998_at	Ptger1	prostaglandin E receptor 1 (subtype EP1)	0.945	0.3213	0.0064	0.4951	No	1.0967	0.0873	0.015	0.187	No	No
99999_at	Zfml	zinc finger, matrin-like	0.9675	0.4818	0.0045	0.3866	No	0.949	0.0375	0.0004	0.901	No	No
AFFX-18SRNAMur/X00686_3_at	---	---	0.9443	0.5296	0.3579	0.2554	No	1.0524	0.444	0.157	0.0217	No	No
AFFX-18SRNAMur/X00686_5_at	---	---	0.8927	0.1705	0.5124	0.0724	No	0.9646	0.675	0.178	0.543	No	No
AFFX-18SRNAMur/X00686_M_at	---	---	0.8979	0.1097	0.2278	0.9412	No	0.9298	0.585	0.52	0.947	No	No
AFFX-b-ActinMur/M12481_3_at	Actb	actin, beta, cytoplasmic	1.0375	0.1334	0	0.2086	No	1.0081	0.846	0	0.0661	No	No
AFFX-b-ActinMur/M12481_3_st	Actb	actin, beta, cytoplasmic	0.9606	0.6338	0.0072	0.1781	No	1.0485	0.547	0.0014	0.646	No	No
AFFX-b-ActinMur/M12481_5_at	Actb	actin, beta, cytoplasmic	1.0213	0.3991	0	0.0492	No	0.9871	0.223	0.0002	0.0038	No	No
AFFX-b-ActinMur/M12481_5_st	Actb	actin, beta, cytoplasmic	0.9744	0.7493	0.6188	0.4236	No	1.0092	0.9	0.0166	0.493	No	No
AFFX-b-ActinMur/M12481_M_at	Actb	actin, beta, cytoplasmic	1.1116	0.0543	0	0.5177	No	1.0774	0.0756	0	0.702	No	No
AFFX-b-ActinMur/M12481_M_st	Actb	actin, beta, cytoplasmic	0.9768	0.3259	0.0002	0.1185	No	0.9654	0.482	0.0059	0.479	No	No
AFFX-BioB-3_at	---	---	0.8611	0.2341	0.732	0.7989	No	0.89	0.343	0.581	0.635	No	No
AFFX-BioB-3_st	---	---	0.9616	0.7495	0.9819	0.483	No	0.9411	0.551	0.886	0.686	No	No
AFFX-BioB-5_at	---	---	0.9293	0.4009	0.3281	0.7902	No	0.9242	0.388	0.244	0.533	No	No
AFFX-BioB-5_st	---	---	0.8777	0.4255	0.1847	0.8834	No	0.9337	0.552	0.421	0.58	No	No
AFFX-BioB-M_at	---	---	0.8752	0.2613	0.3604	0.9078	No	0.8889	0.387	0.263	0.971	No	No
AFFX-BioB-M_st	---	---	0.8343	0.3036	0.7095	0.8748	No	0.8623	0.311	0.365	0.873	No	No
AFFX-BioC-3_at	---	---	0.913	0.4028	0.8161	0.8579	No	0.9076	0.332	0.382	0.94	No	No
AFFX-BioC-3_st	---	---	0.925	0.3911	0.1357	0.681	No	1.0609	0.44	0.371	0.521	No	No
AFFX-BioC-5_at	---	---	0.9029	0.4314	0.5132	0.9147	No	0.9093	0.329	0.865	0.733	No	No
AFFX-BioC-5_st	---	---	0.9709	0.7023	0.2391	0.9673	No	0.9283	0.258	0.62	0.312	No	No
AFFX-BioDn-3_at	---	---	0.9542	0.6619	0.509	0.9354	No	0.9992	0.954	0.865	0.857	No	No
AFFX-BioDn-3_st	---	---	1.001	0.9914	0.4098	0.8527	No	1.0384	0.703	0.268	0.898	No	No
AFFX-BioDn-5_at	---	---	0.9088	0.5135	0.1172	0.8919	No	0.9687	0.786	0.57	0.808	No	No
AFFX-BioDn-5_st	---	---	1.0282	0.8354	0.058	0.3644	No	0.9678	0.998	0.0174	0.131	No	No
AFFX-CreX-3_at	---	---	1.0483	0.6797	0.5819	0.5815	No	1.0414	0.643	0.894	0.548	No	No
AFFX-CreX-3_st	---	---	1.0473	0.6548	0.857	0.4345	No	1.0193	0.858	0.623	0.755	No	No
AFFX-CreX-5_at	---	---	1.0262	0.8111	0.1412	0.8509	No	0.9948	0.926	0.148	0.942	No	No
AFFX-CreX-5_st	---	---	1.1714	0.4884	0.9439	0.6972	No	1.0566	0.753	0.763	0.943	No	No
AFFX-DapX-3_at	---	---	0.9631	0.6004	0.0669	0.9819	No	0.9991	0.923	0.0242	0.293	No	No
AFFX-DapX-5_at	---	---	1.0331	0.6065	0.3891	0.8373	No	1.0777	0.472	0.568	0.877	No	No
AFFX-DapX-M_at	---	---	0.9736	0.5174	0.9188	0.3721	No	0.998	0.979	0.291	0.318	No	No
AFFX-GapdhMur/M32599_3_at	LOC14433	similar to glyceraldehyde-3-phosphate dehydrogenase	0.96	0.116	0.0224	0.6811	No	0.9711	0.227	0.0034	0.264	No	No
AFFX-GapdhMur/M32599_3_st	LOC14433	similar to glyceraldehyde-3-phosphate dehydrogenase	0.9615	0.4657	0.0922	0.8058	No	1.0087	0.795	0.0484	0.967	No	No
AFFX-GapdhMur/M32599_5_at	LOC14433	similar to glyceraldehyde-3-phosphate dehydrogenase	1.0116	0.7821	0.048	0.5221	No	0.9934	0.961	0.0032	0.0814	No	No

AFFX-GapdhMur/M32599_5_st	LOC14433	similar to glyceraldehyde-3-phosphate dehydrogenase	0.9273	0.2777	0.1052	0.6475	No	0.9441	0.279	0.0106	0.587	No	No
AFFX-GapdhMur/M32599_M_at	LOC14433	similar to glyceraldehyde-3-phosphate dehydrogenase	0.9964	0.9046	0.0007	0.6755	No	0.9633	0.397	0.0021	0.36	No	No
AFFX-GapdhMur/M32599_M_st	LOC14433	similar to glyceraldehyde-3-phosphate dehydrogenase	0.9766	0.6021	0.1699	0.9595	No	0.9795	0.595	0.135	0.703	No	No
AFFX-LysX-3 at	---	---	1.0209	0.5649	0.8775	0.171	No	0.9531	0.479	0.709	0.673	No	No
AFFX-LysX-5 at	---	---	0.9369	0.0217	0.0066	0.6805	No	0.951	0.498	0.0362	0.964	No	No
AFFX-LysX-M at	---	---	1.0418	0.3561	0.2753	0.2758	No	1.067	0.205	0.173	0.995	No	No
AFFX-MUR b2 at	---	---	1.0608	0.4151	0.0036	0.6032	No	1.0557	0.304	0.0004	0.484	No	No
AFFX-MurFAS at	Fas	Fas (TNF receptor superfamily member)	0.9475	0.2903	0.7235	0.5597	No	1.0064	0.975	0.332	0.252	No	No
AFFX-MurIL10 at	Il10	interleukin 10	0.9871	0.8652	0.3758	0.9638	No	0.9581	0.431	0.531	0.589	No	No
AFFX-MurIL2 at	---	---	1.044	0.5449	0.7701	0.8181	No	1.1351	0.107	0.224	0.109	No	No
AFFX-MurIL4 at	Il4	interleukin 4	0.954	0.3259	0.3079	0.6056	No	0.9146	0.0892	0.0097	0.43	No	No
AFFX-MURINE_b1 at	---	---	1.0772	0.1599	0.0348	0.7865	No	1.0475	0.267	0.0018	0.666	No	No
AFFX-MURINE_B2 at	---	---	1.0725	0.2292	0.0493	0.7663	No	1.0592	0.181	0.0671	0.832	No	No
AFFX-PheX-3 at	---	---	0.9947	0.8981	0.003	0.1696	No	0.93	0.123	0.032	0.477	No	No
AFFX-PheX-5 at	---	---	1.0252	0.475	0.0255	0.9066	No	0.9569	0.362	0.136	0.453	No	No
AFFX-PheX-M at	---	---	1.0248	0.5344	0.0282	0.8075	No	0.9284	0.475	0.575	0.156	No	No
AFFX-PyruCarbMur/L09192_3_at	Pcx	pyruvate carboxylase	0.9351	0.0916	0.2296	0.2537	No	0.888	0.0371	0.676	0.308	No	No
AFFX-PyruCarbMur/L09192_5_at	Pcx	pyruvate carboxylase	0.949	0.0887	0.116	0.7207	No	0.9218	0.0195	0.0039	0.852	No	No
AFFX-PyruCarbMur/L09192_MA at	Pcx	pyruvate carboxylase	1.0604	0.4169	0.2951	0.575	No	1.0542	0.186	0.0157	0.326	No	No
AFFX-PyruCarbMur/L09192_MB at	Pcx	pyruvate carboxylase	0.9525	0.1852	0.0043	0.9201	No	0.9896	0.799	0.0018	0.381	No	No
AFFX-ThrX-3 at	---	---	1.0351	0.4539	0.3568	0.4355	No	1.0158	0.626	0.361	0.233	No	No
AFFX-ThrX-5 at	---	---	1.0231	0.7403	0.3177	0.6049	No	1.0447	0.463	0.103	0.887	No	No
AFFX-ThrX-M at	---	---	1.0366	0.3753	0.0669	0.2673	No	1.0203	0.815	0.887	0.448	No	No
AFFX-TransRecMur/X57349_3_at	Tfrc	transferrin receptor	0.9435	0.2602	0.3863	0.6737	No	0.9392	0.471	0.789	0.891	No	No
AFFX-TransRecMur/X57349_5_at	Tfrc	transferrin receptor	1.0062	0.8512	0.5213	0.8119	No	0.9782	0.461	0.439	0.258	No	No
AFFX-TransRecMur/X57349_M at	Tfrc	transferrin receptor	1.0366	0.0824	0.0002	0.8899	No	0.9895	0.826	0.362	0.615	No	No
AFFX-TrpnX-3 at	---	---	1.0287	0.5413	0.1574	0.9776	No	1.0187	0.983	0.0519	0.442	No	No
AFFX-TrpnX-5 at	---	---	0.9876	0.6346	0.1306	0.3848	No	1.1131	0.075	0.0188	0.863	No	No
AFFX-TrpnX-M at	---	---	0.9632	0.4685	0.6002	0.5188	No	1.0393	0.506	0.0222	0.672	No	No
AFFX-YEL002c/WBP1 at	---	---	0.9876	0.8155	0.0713	0.8711	No	0.8398	0.515	0.121	0.949	No	No
AFFX-YEL018w/_at	---	---	0.9013	0.0888	0.0426	0.3202	No	0.9567	0.622	0.535	0.48	No	No
AFFX-YEL021w/URA3 at	---	---	0.9849	0.7623	0.1493	0.176	No	0.9848	0.646	0.205	0.586	No	No
AFFX-YEL024w/RIP1 at	---	---	1.0294	0.6246	0.1993	0.64	No	0.9891	0.743	0.0368	0.227	No	No
